# Translational contributions to tissue specificity in rhythmic and constitutive gene expression

**DOI:** 10.1186/s13059-017-1222-2

**Published:** 2017-06-16

**Authors:** Violeta Castelo-Szekely, Alaaddin Bulak Arpat, Peggy Janich, David Gatfield

**Affiliations:** 1 Center for Integrative Genomics, University of Lausanne, Génopode, 1015 Lausanne, Switzerland; 2 Vital-IT, Swiss Institute of Bioinformatics, Génopode, 1015 Lausanne, Switzerland

**Keywords:** Circadian clocks, Translation, Ribosome profiling, Kidney, Liver

## Abstract

**Background:**

The daily gene expression oscillations that underlie mammalian circadian rhythms show striking differences between tissues and involve post-transcriptional regulation. Both aspects remain poorly understood. We have used ribosome profiling to explore the contribution of translation efficiency to temporal gene expression in kidney and contrasted our findings with liver data available from the same mice.

**Results:**

Rhythmic translation of constantly abundant messenger RNAs (mRNAs) affects largely non-overlapping transcript sets with distinct phase clustering in the two organs. Moreover, tissue differences in translation efficiency modulate the timing and amount of protein biosynthesis from rhythmic mRNAs, consistent with organ specificity in clock output gene repertoires and rhythmicity parameters. Our comprehensive datasets provided insights into translational control beyond temporal regulation. Between tissues, many transcripts show differences in translation efficiency, which are, however, of markedly smaller scale than mRNA abundance differences. Tissue-specific changes in translation efficiency are associated with specific transcript features and, intriguingly, globally counteracted and compensated transcript abundance variations, leading to higher similarity at the level of protein biosynthesis between both tissues.

**Conclusions:**

We show that tissue specificity in rhythmic gene expression extends to the translatome and contributes to define the identities, the phases and the expression levels of rhythmic protein biosynthesis. Moreover, translational compensation of transcript abundance divergence leads to overall higher similarity at the level of protein production across organs. The unique resources provided through our study will serve to address fundamental questions of post-transcriptional control and differential gene expression in vivo.

**Electronic supplementary material:**

The online version of this article (doi:10.1186/s13059-017-1222-2) contains supplementary material, which is available to authorized users.

## Background

Circadian clocks serve organisms to synchronise behaviour, physiology and gene expression according to time of day. The mammalian circadian system consists of a master clock in the brain’s suprachiasmatic nuclei (SCN) that receives photic inputs from the retina and synchronises peripheral clocks present in most cells throughout the body. The molecular timekeeping mechanism—the core clock—consists of a network of transcriptional activators and repressors interacting in negative feedback loops (reviewed in [1, 2]). In the core loop, the heterodimeric transcription factor ARNTL:CLOCK (also known as BMAL1:CLOCK) drives the expression of its own repressors, encoded by the *Period* (*Per1*, *Per2*, *Per3*) and *Cryptochrome* (*Cry1*, *Cry2*) genes—a configuration also known as the positive and negative limbs of the oscillator. Additional feedback—in particular, an interconnecting limb involving nuclear receptors of the REV-ERB (encoded by genes *Nr1d1*, *Nr1d2*) and ROR (*Rora*, *Rorb*, *Rorc*) family—intersects with the core loop and numerous post-translational modifications of clock proteins further add to the complexity of the circuitry. The final outcome is a set of robustly cycling transcriptional activities peaking at different phases around the day that drive the rhythmic expression of hundreds to thousands of other genes, termed the clock output or clock-controlled genes (CCGs). It is noteworthy that, despite the probably (near-)identical molecular makeup of the core clock across cell types, CCGs show considerable tissue specificity [3]. The co-regulation by core clock and tissue-specific (non-rhythmic) transcription factors may engender such cell type-specific rhythmic expression patterns, as shown to occur in *Drosophila* [4]. Overall, however, the origins of tissue specificity in rhythmic gene output (and even in certain core clock parameters [5]) are poorly understood. Mechanisms that act at the post-transcriptional level and that impact daily messenger RNA (mRNA) and protein accumulation kinetics are plausible players in the generation of cell-type differences as well.

Rhythmic gene expression has been mainly investigated at the transcriptome level, i.e. using mRNA abundances as a primary readout. However, comparison of mRNA levels with datasets of genome-wide transcriptional activity and of protein abundances that have become available recently, has suggested that a surprisingly large fraction of gene expression oscillations may have post-transcriptional origins (reviewed in [6]). The many cases of protein rhythms that are independent of an underlying oscillating transcript (initially reported in a low-throughput mass-spectrometric study from mouse liver ten years ago [7] and recently confirmed at a comprehensive scale [8, 9]) point to important roles for translation, protein degradation and protein secretion in shaping time of day-dependent proteomes. We [10] and others [11] have recently used ribosome profiling, a genome-wide method that assesses translation efficiency through the deep sequencing of ribosome-protected mRNA fragments, to chart the contribution of translational control to daily protein biosynthesis in mouse liver. One conclusion that emerged from the identified cases of translationally generated oscillations was that circadian clock activity and feeding rhythms both contribute to regulating rhythmic gene expression outputs [10, 11]. Notably, the most abundant group of transcripts subject to rhythmic translation, i.e. mRNAs encoding ribosomal proteins and other components of the translation machinery that all contain 5′-terminal oligopyrimidine tract (5′-TOP) sequences regulated by the mammalian target of rapamycin (mTOR) [12], appear to be under the dominant control of feeding [11].

We have now performed ribosome profiling using a second organ from the same cohort of animals, the kidney, which is an emerging circadian model organ with distinct rhythmic functions [13]. By contrasting kidney and liver datasets, we comprehensively assessed commonalities and differences in their translatomes and we evaluated how far the regulation of translation efficiency contributed to tissue specificity in rhythmic and constitutive protein biosynthesis.

## Results

### Around-the-clock ribosome profiling datasets from two organs

**Fig. 1:**
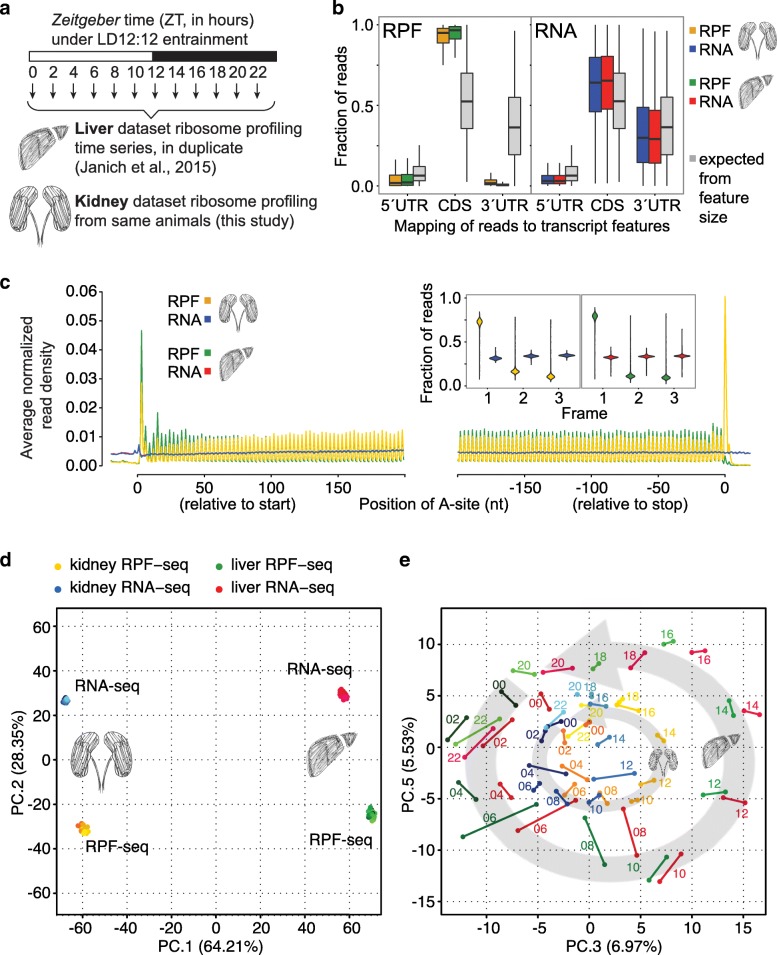
Ribosome profiling around-the-clock in mouse liver and kidney. **a** Overview of the experimental design. Livers and kidneys for ribosome profiling were collected every 2 h for two daily cycles. Each timepoint sample was a pool of organs from two animals. Mice were kept under 12 h:12 h light-dark conditions, with *Zeitgeber* times ZT00 corresponding to lights-on and ZT12 to lights-off. **b** Read distribution to transcript features. RPF-seq (*left*; kidney in *orange*, liver in *green*) and RNA sequencing (RNA-seq) (*right*; *blue* and *red* for kidney and liver, respectively) compared with a distribution expected from the relative feature sizes (*grey*; the distributions based on feature sizes were highly similar for both organs, thus only that for kidney is shown). Note that RPF-seq footprints were enriched on the CDS and depleted from UTRs, whereas RNA-seq reads distributed more homogeneously along transcripts, according to feature size. Of note, the higher level of 3′ UTR footprints in kidney resulted mainly from differences in the efficiency with which stop codon footprints were captured, as described in (**c**). **c** Predicted position of the ribosome’s aminoacyl tRNA-site (A-site) of reads relative to the CDS start and stop codons. Read density at each position was averaged across single protein coding isoform genes (i.e., genes with one main expressed transcript isoform) that had an average RPF RPKM > 5, a CDS > 400 nt in length and were expressed in both organs (*n* = 3037 genes). This analysis revealed the trinucleotide periodicity of RPF-seq (but not RNA-seq) reads in both organs. *Inset*: frame analysis of CDS reads showed preference for the annotated reading frame (frame 1, the same frame as the start codon) in RPF but not in RNA reads. *Violin plots* extend to the range of the data (*n* = 3694 genes for liver, *n* = 4602 genes for kidney). A separate analysis of the higher level of stop codon footprints in kidney, that also led to the differences in 3′ UTR reads in B, can be found in Additional file 1: Figure S2A, B. **d** Principal component analysis (PCA) of kidney and liver RPF-seq and RNA-seq datasets, using the 4000 most variable genes. The first two components reflected the variability coming from organ (PC1, 64.21%) and from RPF/RNA origin of datasets (PC2, 28.35%). **e** PC3 vs. PC5 (together 12.5% of variation) resolved the factor time within each dataset, leading to a representation that resembled the face of a clock. Each *dot* represents one sample, timepoint replicates are joined by a *line* and timepoints within each dataset are sequentially *coloured*. The circular arrangement was larger for liver than kidney, suggesting a higher contribution of hepatic rhythmic genes to overall variability. Additional file 1: Figure S4 shows the scree plot for the ten first components

For our recent study of the liver translatome around-the-clock [10], we had used ribosome profiling [14] (RPF-seq) on a time series of organs collected from mice sacrificed every 2 h over the 24-h day (12 timepoints in duplicate; Fig. 1a). To generate a complementary dataset from a second organ, we chose the kidneys from the same cohort of animals. Liver and kidney express thousands of genes in common [3, 15], thus providing a particularly suitable setting for a cross-organ comparison of gene expression.

Applying the same experimental and computational methods as for liver RPF-seq [10, 16], we obtained comparable high-quality data for kidney (see Additional file 1: Figure S1A–C and Additional file 2 for details on sequencing and mapping outcomes). Briefly, ribosome footprints from both organs showed similar enrichment for protein coding sequences (CDS) of mRNAs and depletion of untranslated regions (UTRs) (Fig. 1b). Like the footprints from liver, those from kidney also exhibited excellent reading frame preference, which allowed resolving the 3-nt periodicity of coding sequences transcriptome-wide (Fig. 1c and Additional file 1: Figure S2A, B). Moreover, the high correlation coefficients seen across replicates of the kidney time series for both RNA-seq and RPF-seq data indicated excellent biological and technical reproducibility (Additional file 1: Figure S3A, B). We also used a recently developed tool, termed Ribo-seq Unit Step Transformation (RUST) [17], to confirm high technical similarity of datasets between organs (Additional file 1: Figure S2C, D). Finally, principal component analysis (PCA) on all available datasets (96 libraries, i.e. RPF-seq and RNA-seq from two organs, 12 timepoints, in duplicate) segregated the data according to the main experimental and biological covariates. PC1 (explaining 64.2% of variation) thus separated libraries according to organ, indicating that tissue origin represented the major source of divergence, followed by PC2 (28.4%) that separated RNA-seq (mRNA abundance) and RPF-seq (footprints/translation) (Fig. 1d). The cyclic nature of the data was resolved in the representation PC3 versus PC5 (together 12.5%), in which timepoints assembled to a near-perfect clock (Fig. 1e). The larger circular arrangement of the liver versus kidney time series suggested that rhythmic gene expression from liver contributed more strongly to overall variation than did kidney rhythms. This observation is in line with the notion that there are more and higher amplitude rhythms in liver than in kidney [3]. Taken together, we concluded that the kidney data were of similarly high quality as our previous liver datasets [10]. Together, they would be suitable for comparative analyses of time of day-dependent and constitutive translation across two tissues.

### Cross-organ differences in translation efficiency are widespread, of moderate scale and partially compensate RNA abundance differences

**Fig. 2:**
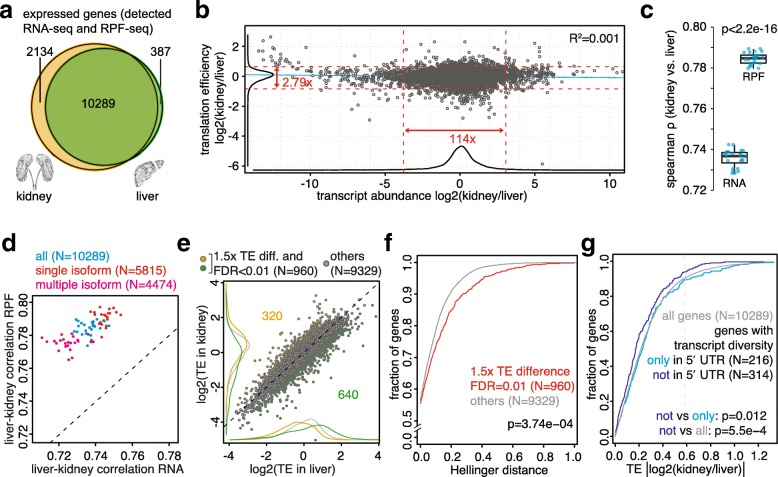
Cross-organ differences in translation efficiency partially compensate RNA abundance differences and show association with transcript features. **a** *Venn diagram* showing the gene expression overlap (i.e. genes detected at both RPF and RNA level) between kidney (*yellow*, *n* = 12,423 genes) and liver (*green*, *n* = 10,676 genes). Same cutoffs on RPKM (reads per kilobase of transcript per million mapped reads) were used for both organs. **b** *Scatterplot* of kidney-to-liver ratio of mRNA abundance versus translation efficiency (TE) for all expressed genes (*n* = 10,289), averaged over all timepoints. Corresponding density curves are plotted on the margins. *Dashed red lines* represent the 2.5 and 97.5 percentiles of each variable and the corresponding fold-change is indicated. Linear regression line is depicted in blue (R^2^ = 0.0009, *p* = 0.0009). While 95% of genes spanned a 114-fold range in mRNA abundance differences across organs, the same number of genes changed less than threefold in TE, underlining that transcript abundance was the main contributor to divergent gene expression. **c** Inter-organ Spearman correlation for RNA-seq and RPF-seq samples. Each *dot* represents the correlation coefficient between kidney and liver for a timepoint and replicate sample. Note that RPF-seq samples consistently correlated significantly better than RNA-seq samples (*p* < 2.2e-16, *n* = 24, paired t-test of Fisher-transformed correlation coefficients). **d** *Scatterplot* of inter-organ RNA vs. RPF correlation coefficients for each sample separately calculated from all (*blue*, *n* = 10,289), from single isoform (*red*, *n* = 5815), and from multiple isoform (*pink*, n = 4474) genes. Consistently better RPF correlation was evident in all cases. **e** Relative TE in liver vs. kidney (data centred and averaged over all timepoints for all expressed genes, *n* = 10,289) showed an overall strong inter-organ correlation. Differential TE—defined as having false discovery rate (FDR)-corrected *p* < 0.01 (Wilcoxon signed rank test on TE) and > 1.5 difference in TE across organs—was apparent for ca. 9% of genes (*yellow* and *green* show cases where TE is higher in kidney and liver, respectively, *n* = 960). **f** Cumulative distribution of Hellinger distances for genes showing differential TE (*red*, *n* = 960), or not (*grey*, *n* = 9329), as detected in (**e**). Hellinger distance was used as a quantitative measure for relative transcript isoform diversity across organs, as described in ‘Results’ and ‘Methods’. The analysis shows that divergent TE correlated with larger diversity in transcript isoform expression (D = 0.0702, *p* = 3.74e-04, two-sample Kolmogorov–Smirnov [KS] test). **g** Cumulative distribution of the kidney-to-liver TE ratio for genes whose transcript diversity originated exclusively from the 5′ UTR (identical CDS and 3' UTR, *light blue*, *n* = 216; these genes show more TE differences across organs) and genes whose transcripts had identical 5′ UTR (and divergent CDS and/or 3′ UTR, *purple*, *n* = 314; these genes show less TE differences across organs). The *vertical dashed grey line* marks the 1.5-fold difference used to define differential TE (as in (**e**)). These results suggested that tissue specificity in TE was partially achieved by expressing transcript isoforms that differed in their 5′ UTRs (note the significant shift towards smaller TE differences for genes with identical 5' UTRs). See also Additional file 1: Figure S9

To what extent do differences in translation efficiency contribute to different gene expression outputs across organs? We addressed this question using the set of 10,289 genes whose expression was detectable in kidney and in liver at both RPF and RNA level (Fig. 2a). From the ratio of CDS-mapping normalised read counts for RPF-seq relative to RNA-seq, we first calculated relative translation efficiencies (TEs) per transcript and for each organ. TEs were overall rather similar between tissues, with 95% of genes falling into a less than threefold range for the kidney/liver TE ratio, as compared with a greater than 100-fold range for the transcript abundance ratio (Fig. 2b). This observation was coherent with the considerably broader spread of mRNA abundances versus TEs across genes within each organ (greater than 500-fold versus just over tenfold, respectively; Additional file 1: Figure S5A, B) and is in line with a dominant role for the regulation of mRNA levels (i.e., transcription and mRNA decay) in controlling quantitative differences in gene output.

Intuitively, we had expected that RNA levels that were widely dissimilar between kidney and liver and subsequently further modulated by organ-specific TEs, would probably give rise to even greater cross-organ divergence at the RPF level. Intriguingly, however, the global correlation between kidney and liver was better for footprint abundances than for transcript abundances (Spearman ρ [RPF]: mean 0.784 vs. ρ [RNA]: mean 0.736; *p* < 2.2e-16; paired t-test of Fisher-transformed correlation coefficients, n = 24) (Fig. 2c; Additional file 1: Figure S3C, D). This phenomenon was observed irrespective of whether the genes expressed only one dominant protein-coding transcript isoform (‘single isoform genes’ in the following) that was common to both organs, or whether they gave rise to different (including tissue-specific) mRNA variants (‘multiple isoform genes’) (Fig. 2d). The observed higher cross-organ concordance of RPFs could have simply had technical reasons, e.g. if the RPF-seq protocol gave more reproducible results than the RNA-seq protocol. We addressed this caveat by comparing measurement errors (MEs) for RNA and RPF data using a similar approach as in a recent publication [18]. We found that MEs scaled inversely with expression levels, as expected, and showed some variation due to organ (Additional file 1: Figure S6A, B, F, G). Especially in liver and among low expressed transcripts, a tendency towards smaller MEs for RPF than for RNA was indeed visible (differences statistically non-significant). In most other cases, however, measurement errors were (in part significantly) higher for the transcripts’ RPF counts than for their RNA counts. Of note, the better cross-organ correlation of RPF vs. RNA levels seen in the full transcript set (Fig. 2c) was also evident within various transcript subsets (Additional file 1: Figure S6C–E, H–L), including such subsets for which RPF MEs were higher than RNA MEs (Additional file 1: Figure S6E, L). It is thus unlikely that technical bias was the reason for the higher RPF correlation. Finally, an analysis that we performed on independent ribosome profiling datasets from rat liver and heart [19] allowed us to confirm the phenomenon of higher concordance of RPF versus RNA abundance also between these organs (Additional file 1: Figure S7A–C). Taken together, these findings are suggestive of a potentially broader biological phenomenon that consists in the partial compensation of differences in a gene’s mRNA expression through counteracting effects exerted through its TE, resulting in the convergence at the level of protein biosynthetic output (footprints, RPF) across tissues.

### Transcript features associated with cross-organ differences in translation efficiency

Do particular transcript features have predictive value for organ-specific differences in translation efficiency? To investigate this question, we selected the genes with significantly different TEs between tissues (*n* = 5013; Wilcoxon signed rank test; FDR < 0.01) and implemented a 1.5-fold cutoff on TE ratio between the organs to retrieve the most pronounced cases (n = 960) (Fig. 2e; Additional file 3). Of these, 533 represented ‘single isoform genes’ with no (or negligible amounts of) expression of tissue-specific mRNA variants. For these genes, we examined whether a higher TE in kidney (*n* = 193) or in liver (*n* = 340) was associated with specific transcript characteristics. Of several features tested, we found that CDS and transcript lengths showed the most significant association with differential TE (Additional file 1: Figure S8A, B). Of note, we had previously seen in liver that shorter coding sequences, i.e., transcripts encoding smaller proteins, are more efficiently translated [10]. Our present analyses suggest that such transcripts are also more prone to tissue-specific regulation at the translational level. Other sequence features showed some bias within the differential TE gene sets as well, although the effects were overall weaker and less consistent. Briefly, the 5′ UTRs of genes with higher TEs in liver were longer and predicted to fold more strongly. By contrast, transcripts with higher kidney TEs were associated with lower 5′ UTR GC content and slightly shorter 3′ UTRs. No association with differential TE was found for the Kozak sequence context score.

We also investigated two functional classes of sequence features, micro RNA (miRNA) binding sites and upstream open reading frames (uORFs), for association with differential TE. Of note, the 960 ‘TE different’ transcripts were not enriched for any predicted miRNA binding sites, making it unlikely that this class of post-transcriptional regulators is a major player in establishing tissue-specific TEs (data not shown). We had previously observed that in the liver the presence of a translated uORF in the 5′ UTR was strongly predictive of low TE at the main ORF [10]. An analogous relationship was also evident in kidney (Additional file 1: Figure S5C). To assess whether uORF translation was associated with TE differences across organs, we compared how the identified uORF-containing transcripts (i.e. single isoform genes showing translated uORFs in at least one organ; *n* = 1377) distributed to the differential versus non-differential TE gene sets. The group of genes with higher TE in liver was significantly enriched for transcripts with translated uORFs (*p* = 6.08e-04; Fisher’s exact test) and there was slight depletion among genes with higher TE in kidney (not significant) (Additional file 1: Figure S8C). Only few differential TE genes exhibited uORF translation that was exclusive to one organ, but there was a tendency for kidney-specific translation of uORFs to be associated with higher TE on the CDS in liver and vice versa (Additional file 1: Figure S8D). For the genes with uORFs translated in both tissues, we expected that cross-organ differences in the strength of uORF usage would negatively correlate with TE differences at the CDS. However, such a trend was only visible for liver differential TE genes (Additional file 1: Figure S8E); and globally, uORF and CDS TEs even showed slightly positive correlation. In summary, these analyses suggested that uORF translation contributed to some extent (and especially for genes that were more efficiently translated in the liver) to cross-organ differences in TE; however, the overall impact appeared limited (see ‘Discussion’).

We next included the ‘multiple isoform genes’ in the analyses and asked whether transcript isoform diversity between the two organs—i.e. the occurrence of tissue-specific mRNA variants generated by alternative transcriptional start sites, splicing and 3′ processing—had any relationship to differential TE. Briefly, using our RNA-seq data we first compiled an inventory of all annotated, protein-coding transcript isoforms and their estimated relative expression levels per gene and tissue. We then used the Hellinger distance [20] as a measure of dissimilarity in isoform expression levels between kidney and liver. A value of 0 for this metric indicates that a gene has identical isoform distribution in both tissues (i.e. these are essentially the ‘single isoform genes’ described above), while a value of 1 denotes a lack of overlap in expressed isoforms. Globally, the 960 genes with differential TE showed significantly higher Hellinger distances than the remainder of the expressed genes (*p* = 3.74e-04; Kolmogorov–Smirnov test) (Fig. 2f). Molecularly, the term ‘transcript isoform’ comprises variations affecting 5′ UTR, CDS and 3′ UTR. By comparing the genes for which all expressed variants affected exclusively one single feature or for which this particular feature was not affected at all, it became apparent that transcript diversity in the 5′ UTR was particularly strongly associated with differential TE (Fig. 2g). By contrast, variation in the CDS showed significantly less association with cross-organ differences in translation efficiency (Additional file 1: Figure S9A, B). Although the low number of available transcripts bearing exclusively 3′ UTR differences precluded a rigorous interpretation, 3′ UTR variation did not appear to be associated with differential TE either (Additional file 1: Figure S9C). Altogether, we thus concluded that TE differences between tissues may, at least in part, have their origin in tissue-specific transcript variants, especially through alternative 5′ UTRs.

Finally, we were interested in whether cross-organ differences in translation efficiency affected specific pathways. For the 640 ‘TE different’ genes that showed increased TE in liver (Fig. 2d), gene ontology (GO) analyses revealed significant enrichment for categories related to transcription (Additional file 3). Conceivably, tissue-specific translational control of transcriptional regulators may thus impact also on the organs’ transcriptomes. The 320 ‘TE different’ genes that were translated better in kidney did not show any significant enrichment.

### Translational modulation of phase of oscillation in kidney

**Fig. 3:**
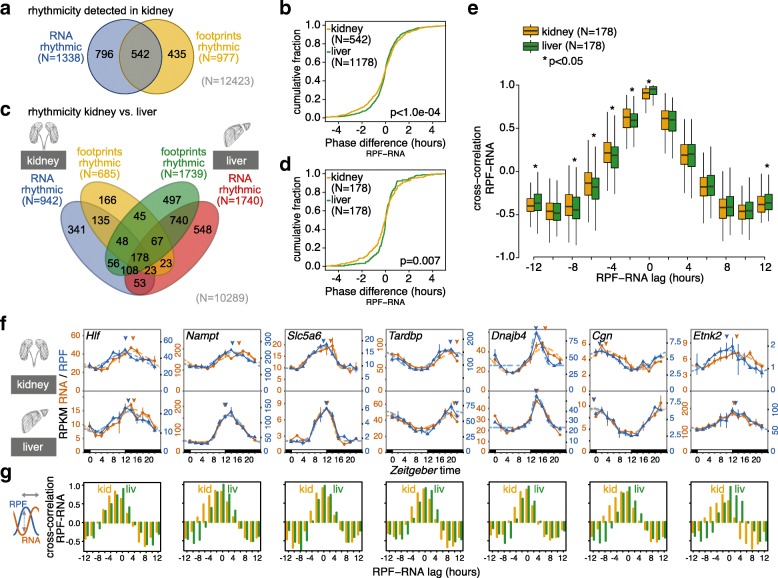
Rhythmicity analyses across organs reveals phase modulation by translation in kidney. **a** *Venn diagram* showing rhythmic genes in kidney. Of the 12,423 expressed genes, 1338 showed 24-h oscillations of > 1.5-fold amplitude in mRNA abundance (RNA-seq, 10.7%) and 977 in footprint abundance (RPF-seq, 7.9%). A total of 542 genes (4.3%) were identified as rhythmic at both levels. **b** Cumulative distribution of phase differences (RPF peak – RNA peak, in hours) for genes rhythmic at both RNA-seq and RPF-seq in liver (*green*, *n* = 1178) and kidney (*yellow*, *n* = 542). The two distributions were significantly different (*p* < 1e-04, permutation test) and reflected that maximal footprint abundance frequently preceded mRNA abundance peaks in kidney (note that the two distributions differed mostly in their negative tail). **c** Four-way Venn diagram of rhythmicity sets for genes expressed in both tissues (*n* = 10,289). Of all genes, 364 and 238 were detected as rhythmic in both organs at the RNA-seq and RPF-seq levels, respectively, and 178 genes were detected as rhythmic throughout (i.e. RNA-seq and RPF-seq, in kidney and liver). **d** Cumulative phase difference distribution in liver (*green*) and kidney (*yellow*) for the 178 common rhythmic genes. As in (**b**), the distributions were significantly different (*p* = 0.007, permutation test) and corroborated that even when comparing the same set of genes, footprint peaks frequently preceded mRNA abundance maxima in kidney. **e** Cross-correlation in kidney (*yellow*) and liver (*green*) of time-resolved RPF-seq profiles relative to the RNA-seq profiles of the *n* = 178 common rhythmic genes. The analysis showed that profile correlations for negative lags (i.e. RPF peaking before RNA) were significantly higher in kidney than liver (* indicate *p* < 0.05, Wilcoxon signed rank test). *Boxplots* represent the interquartile range and whiskers extend to the minimum and maximum expression within 1.5 times the interquartile range. **f** Examples for genes with maxima in RPF (*blue*) preceding those in RNA (*orange*) by several hours in kidney (*top*) but not, or less so, in liver (*bottom*). Arrowheads indicate the peaks in footprint and mRNA abundance as estimated from the rhythmic fits. **g** Cross-correlation analyses of RPF-seq relative to RNA-seq profiles (kidney in *yellow*, liver in *green*) for the genes in (**f**). Maximal correlations of the profiles in kidney were found to be shifted to the left (more negative RPF-to-RNA lags) as compared with liver. For liver, there was no case with a maximal correlation value in negative RPF-to-RNA lags

We next turned to the analysis of factor time across the datasets. We annotated rhythmic events in kidney with the same methodology as previously for liver, including a 1.5-fold cutoff on peak-to-trough amplitudes [10]. A list of the detected RNA and RPF rhythms and genome-wide gene expression plots are provided in Additional file 4 and Additional file 5 under (https://doi.org/10.6084/m9.figshare.4903193), respectively. Our analyses yielded 1338 and 977 genes that cycled at the RNA abundance and footprint level, respectively, with an overlap of 542 genes (Fig. 3a). As discussed later, this relatively modest overlap (542 genes corresponds to 41% and 55% of all ‘RNA rhythmic’ and ‘footprint rhythmic’ cases, respectively) likely underestimates the full extent of shared rhythmicity and only contains the most robustly oscillating gene expression events, which we further explored in the following.

Interestingly, the analysis of rhythmicity parameters across the 542 genes revealed that the timing of their RPF peaks relative to their RNA peaks had a significantly different and broader distribution than the corresponding set from liver (*p* < 1.0e-04; permutation test) (Fig. 3b). This observation suggested that the phase of protein biosynthesis rhythms was subject to marked translational modulation in kidney. In liver, by contrast, RPF peaks were more tightly gated by RNA abundance peaks. Surprisingly, maximal translation tended to precede maximal RNA abundance in kidney (Additional file 1: Figure S10A), as globally the mean RPF peak phase was advanced (–0.123 h) and also RPF rhythms were enriched for phase advances (282) versus delays (260), albeit neither reaching statistical significance (*p* = 0.16, Wilcoxon rank sum test).

The above analyses used different rhythmic gene sets for kidney than for liver, potentially compromising comparability. The observed differences in the RPF-RNA phase relationships could thus have simply arisen from transcript-specific rather than from tissue-specific differences in the timing of translation. We thus analysed the group of 178 genes whose RNA and RPF profiles were rhythmic in both organs (Fig. 3c; Additional file 6 and Additional file 7 (https://doi.org/10.6084/m9.figshare.4903193)). Again, the distribution of RPF-RNA offsets was significantly broader in kidney than in liver (Fig. 3d; *p* = 0.007, permutation test) with an RPF peak phase advance in kidney (mean –0.143 h) and a phase delay in liver (mean 0.036 h) (Additional file 1: Figure S10B, C). We next calculated the gene-wise RPF-RNA peak phase difference in kidney relative to that in liver. More genes showed their RPF maxima earlier (96) than later (82) in kidney versus liver, with a mean advance of –0.178 h (Additional file 1: Figure S10D), but again without passing statistical significance (*p* = 0.152, Wilcoxon rank sum test).

Conceivably, we lost statistical power and introduced error in the above analyses by restricting the phase comparisons merely to the peaks of the rhythmic curve fits. We thus sought a method that would take into account phase differences between RPF and RNA profiles over all data points. To this end, we used cross-correlation to quantify the similarity between the RPF and RNA time series as a function of sliding one series on the time axis relative to the other. When the time series were not shifted against each other at all (RPF-RNA lag = 0 h), the RPF-RNA cross-correlation values were overall highest, as expected, and they were significantly higher in liver, in line with stronger gating of RPF rhythms relative to RNA oscillations in this organ (Fig. 3e). Importantly, when cross-correlation of RNA was calculated with earlier RPF time points (negative RPF-RNA lags; see in particular lags of –2 h to –8 h in Fig. 3e), kidneys scored significantly higher than livers. Sliding the series in the other direction, however, rather led to overall better correlations in the liver (see lags of +4 to +8 in Fig. 3e; liver–kidney difference was non-significant). Taken together, these analyses underscored that there was asymmetry in the data with RPF rhythms preceding RNA rhythms specifically in the kidney.

We confirmed kidney-specific translational phase advances by visual inspection of individual gene expression profiles. Figure 3f shows the profiles for the genes *Hlf*, *Nampt*, *Slc5a6*, *Tardbp*, *Dnajb4*, *Cgn* and *Etnk2*, which all show an RPF phase advance of up to several hours relative to RNA. Cross-correlation analysis for the individual genes also confirmed kidney-specific, phase-advanced translation (Fig. 3g).

At first sight, translation that is phase-advanced to mRNA abundance is counterintuitive. Conceivably, it may occur when translation efficiency is not constant, but decreases over the lifetime of an mRNA. TEs may be higher on freshly synthesised messages that have long poly(A) tails and decrease as a result of gradual deadenylation even before transcript stability and abundance are affected as well [21]. In keeping with the hypothesis of cross-organ differences in poly(A) kinetics, we have observed that most subunits of the major cytoplasmic deadenylase complex, CCR4-NOT, are significantly more highly expressed in kidney than in liver (Additional file 1: Figure S11A–C). Higher deadenylase activity in kidney could provide an attractive molecular explanation for the observed tissue-specific differences in RPF-RNA phasing and for RPF rhythms that are phase-advanced to RNA oscillations.

### High tissue divergence in translationally driven rhythms

Rhythmicity detection algorithms are sensitive to false-negatives, i.e. to classify gene expression profiles as ‘non-rhythmic’ (for example, because they fail imposed thresholds on amplitude or FDR) although the underlying temporal patterns may still be more similar to, and more likely to be, rhythmic than invariable. Of note, the lack of canonical methods to reliably determine true absence of rhythms is a common problem in the field (see recent review by [6] for discussion). Venn diagrams that simply overlap rhythmic gene sets hence need to be interpreted with caution. For these reasons, the extent of ‘RNA only’ and of ‘footprints only’ oscillations in Fig. 3a is likely not reported reliably and subject to overestimation. The heatmaps of the corresponding RNA and RPF profiles support this notion as well (Additional file 1: Figure S12B, D).

**Fig. 4:**
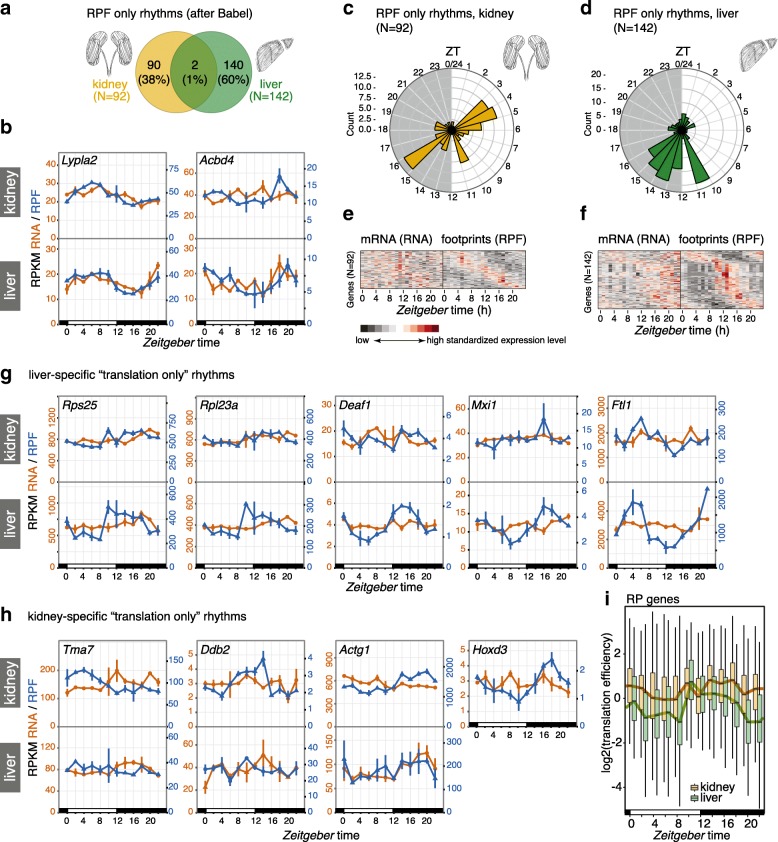
High tissue divergence in translationally driven rhythms. **a** *Venn diagram* of rhythmic RPF-seq sets in kidney (*yellow*, *n* = 92) and liver (*green*, *n* = 142) after the Babel analysis indicated strong tissue specificity of translational control. **b** Daily profiles of RPF-seq RPKM (*blue*) and RNA-seq RPKM (*orange*) for the two genes detected as translationally regulated in both tissues in (**a**). **c**, **d** Circular phase histogram for the 92 (**c**, kidney) and 142 (**d**, liver) genes showing footprint rhythmicity in the organs. Note that the translational upregulation of transcripts observed at the day-to-night transition in liver was absent in kidney. **e**, **f** *Heatmaps* of RNA (*left panels*) and RPF (*right panels*) rhythms for the 92 and 142 genes translationally regulated in kidney (**e**) and in liver (**f**), respectively. Genes are sorted by footprint phase and expression levels are standardized by row (gene). These sets of genes showed rhythmicity in footprint abundance but no oscillation in mRNA. **g**, **h** Daily profiles of RPF-seq RPKM (*blue*) and RNA-seq RPKM (*orange*) for representative examples of translationally generated rhythms specific for liver (**g**) and kidney (**h**). For each gene, the *upper panel* shows the kidney data and the *lower panel* the liver data. *Hoxd3* was not expressed in liver. **i** Translation efficiency (TE) around-the-clock for ribosomal protein (RP) genes expressed in liver (*green*, *n* = 86) and in kidney (*yellow*, *n* = 89). For each timepoint (ZT) *boxplots* represent the interquartile range and *whiskers* extend to the minimum and maximum TE within 1.5 times the interquantile range. *Lines* connect the median of each *boxplot* to ease visualization. Note the global TE upregulation at ZT10 in liver, whereas TEs in kidney remain high over the day

In order to identify the true-positive ‘translation only’ cycling transcripts with higher reliability, we implemented the same methodology as in our previous study [10]. Briefly, we used the analytical framework *Babel* [22] to preselect all transcripts whose translation efficiency changed significantly over the day (and/or whose TEs deviated significantly from the global transcript population). Rhythmicity analyses were then performed on this gene subset and yielded 92 cases with the sought-after temporal profiles of rhythmic translation on non-rhythmic mRNAs (Fig. 4a). Comparison with the 142 genes of the analogous set from liver revealed near-perfect tissue specificity of translationally driven oscillations. Only two genes, *Abcd4* and *Lypla2*, were shared between the organs; they were both among the least compelling cases of ‘translation only rhythms’ that our method had identified, as judged by visual inspection (Fig. 4b).

Interestingly, not only the identity of rhythmically translated genes, but also the time-of-day at which the majority of rhythmic translation events occurred, was highly tissue-specific. The phase histograms thus showed striking differences in the peak time distributions between the organs (Fig. 4c, d; difference in distributions: *p* = 1.66e-04; W = 17.403, df = 2; Watson–Wheeler test for homogeneity of angles). Of note, the enrichment for translational maxima at the light–dark transition (*Zeitgeber* time, ZT10-16; ZT00 corresponds to lights-on and ZT12 to lights-off) that dominated the distribution in liver (Fig. 4d, f) was virtually absent from kidney (Fig. 4c, e). Instead, kidney showed enrichment for transcripts with maximal translation occurring around ZT4 and ZT16. Visual inspection of individual examples confirmed the organ specificity of RPF rhythms. The cases of robust translational oscillations that we [10] and others [11, 12] had previously identified in liver were thus absent or severely blunted in kidney. This included mRNAs encoding ribosomal proteins (RPs), which make up the bulk of genes showing a translational surge at the light–dark transition (e.g. *Rps25*, *Rpl23a*), as well as transcripts encoding the transcription factors *Deaf1* (deformed epidermal autoregulatory factor 1) and *Mxi1* (MAX interactor 1), and mRNAs containing iron-responsive elements in their 5′ UTRs (e.g. Ferritin light chain 1, *Ftl1*) (Fig. 4g), all of which we had previously reported as translationally rhythmic in liver [10]. Rhythmic translation exclusive to kidney was not significantly enriched for particular pathways (data not shown) and the temporal profiles were overall of lower amplitude than those seen for liver; *Tma7* (translational machinery associated 7 homolog), *Ddb2* (damage-specific DNA binding protein 2), *Actg1* (actin, gamma, cytoplasmic 1) and *Hoxd3* (homeobox D3; not expressed in liver) were among the most distinct examples (Fig. 4h).

In summary, we concluded that temporal changes in TE were strikingly tissue-specific and overall relatively rare in kidney. Specifically for transcripts encoding RPs and other components of the translation machinery, which are the most prominent group of TE rhythmic genes in liver, it has been suggested that feeding-dependent mTOR-signalling underlies the translational upsurge at the light–dark transition via a mechanism involving the 5′-terminal oligopyrimidine (5′-TOP) motifs that these transcripts carry [11, 12]. Interestingly, the TE comparison between both tissues revealed that kidney RP translation occurred at a relatively high level throughout the day (Fig. 4i). The lack in rhythmicity for RP genes in this organ may thus result from an absence of translational repression during the light phase rather than a lack in activation in the dark phase. It may indicate that the kidney is less sensitive to systemic cues engendered by feeding and fasting (see ‘Discussion’).

### Different degrees of tissue specificity in core clock gene expression at the level of RNA abundance and protein biosynthesis

Clocks exhibit functional differences across cell types and organs, for example at the level of rhythmicity parameters (e.g. free-running period and phase [5]), of clock output gene repertoires [3], of oscillator strength and robustness [23, 24] or with regard to clock gene loss-of-function phenotypes [25]. Conceivably, the precise timing and level at which the various clock proteins are produced may modulate properties of the clock circuitry and underlie some of the abovementioned functional variations. In order to investigate these possibilities, we compared the expression of core clock components in both organs.

**Fig. 5:**
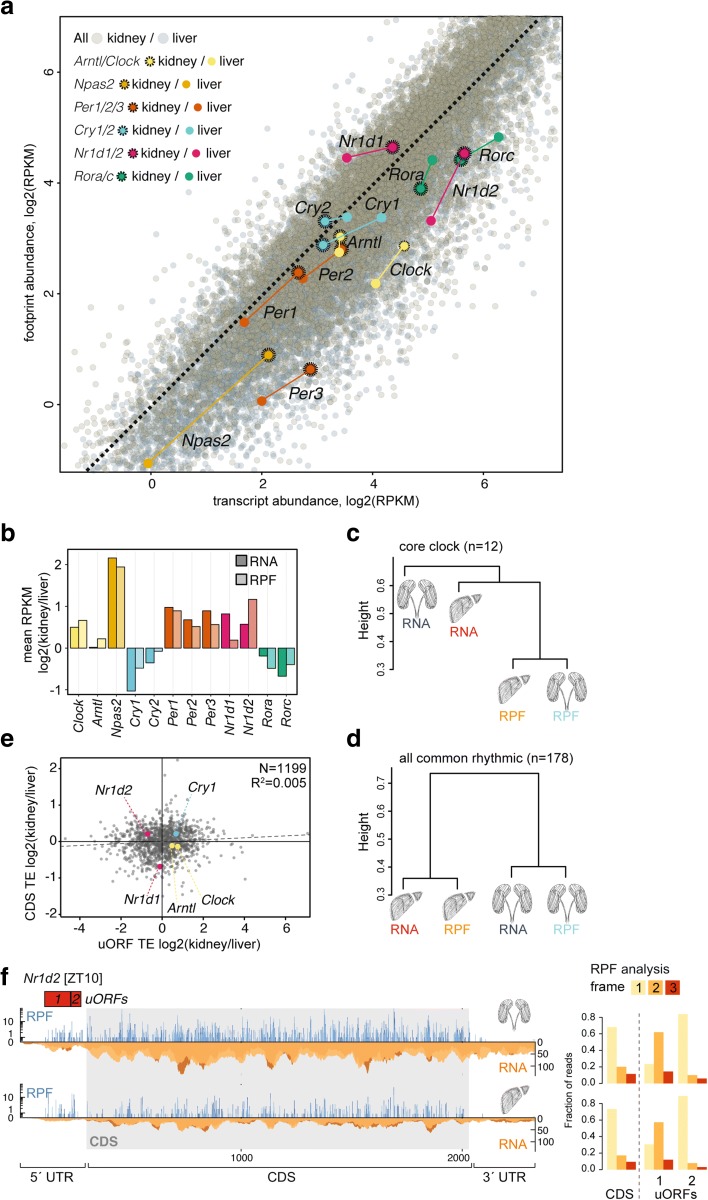
Tissue specificity in core clock gene expression at the level of RNA abundance and translation. **a** *Scatterplot* of transcript abundance (RNA-seq) vs. footprint abundance (RPF-seq) for liver (*grey*) and kidney (*sepia*) (*n* = 10,289), where core clock components are highlighted (kidney, *dots with dashed circles*). *Coloured dashed lines* join the relative locations of each core clock gene between organs. **b** *Bar graph* of the average RPKM ratio between kidney and liver for the main circadian core clock genes, at the level of mRNA abundance (*dark shades*) and ribosome footprints (*light shades*) suggested that translational compensation led to higher similarity at the level of protein biosynthesis (RPF) for several core clock genes. **c** Hierarchical clustering of the organs’ RNA and RPF profiles based on the similarities of the core clock genes expression patterns (*n* = 12, genes shown in B). The height of the branches represents weighted average distances over the considered genes (see ‘Methods’). Note that RPF rhythms in two organs were more similar than RNA and RPF rhythms within an organ. **d** Hierarchical clustering as in (**c**) based on the genes detected as rhythmic throughout (*n* = 178, see Fig. 3c). When compared to the clustering based on core clock gene expression patterns in (**c**), this rhythmic gene set showed an organ-based clustering. **e** *Scatterplot* of kidney/liver ratios of uORF vs. CDS translation efficiencies for genes containing AUG-initiated translated uORFs in both organs (*n* = 1199). uORF-containing core clock genes are highlighted. As also shown in Additional file 1: Figure S8E, differential uORF usage could not globally explain differences in CDS TE across organs (note the lack of negative correlation between the two variables, R^2^ = 0.005, *p* = 0.008). As an exception, the lower uORF TE of *Nr1d2* might have a role in setting relatively higher CDS TE in kidney. **f** RPF (*blue*) and RNA (*orange*) reads mapping along the *Nr1d2* transcript in kidney (*top*) and liver (*bottom*) for the timepoint of maximal CDS translation (ZT10). 5′ UTR and CDS are shown in full, but for better visualization only a portion of the 3′ UTR (the same length as the 5′ UTR) is shown. *Red boxes* indicate the predicted AUG-initiated translated uORFs. *Right panels* show that, similar to the CDS, the uORFs showed clear frame preference, indicative of active translation

We first investigated transcript and footprint RPKMs as averages over timepoints to assess the cumulative daily production of clock RNAs and proteins. Most core clock genes showed a considerable degree of organ specificity in their expression levels that was readily appreciable in the footprint versus transcript abundance representation with both organs overlaid in a single graph (Fig. 5a). Two tissue differences caught our particular attention. First, the balance between the transcriptional activators *Rora/Rorc* and repressors *Nr1d1/Nr1d2* differed markedly between organs and was skewed towards repression in kidney (i.e. higher *Nr1d1/2* and lower *Rora/c* RPKMs in kidney, Fig. 5a, b). These transcriptional regulators bind to shared sequence elements on DNA and form the ‘interconnecting limb’ within the rhythm-generating clock circuitry. In addition, they also control an output branch of the oscillator [1, 2]. It is hence conceivable that adjusting the relative levels of NR1D1/2 versus RORs tailors clock-controlled gene expression in a tissue-specific fashion. Our observation of an active state of this output branch in liver and a more repressed state in kidney is fully consistent with the current knowledge of its target genes and knockout phenotypes, which point to a prominent role in the regulation of hepatic pathways such as lipid, cholesterol and bile acid metabolism [26].

A second tissue difference concerned the main constituents of the negative limb, the Period (*Per*) and Cryptochrome (*Cry*) genes. Heterotypic PER:CRY protein complexes inhibit ARNTL:CLOCK-driven transcriptional activity and thus lie at the core of the oscillator’s principal negative feedback loop. We observed a shift to more PER (and slightly less CRY) biosynthesis in kidney (Fig. 5b). PERs (and in particular PER2) are considered stoichiometrically rate-limiting components of the PER:CRY complex and increased PER2 dosage engenders long periods [27, 28]. Interestingly, tissue explant experiments have shown that kidney clocks free-run with almost 1.5-h longer periods than liver clocks [5], as would be predicted from the increased PER biosynthesis that our analyses revealed. As a more general concept, we deem it conceivable that the modulation of biosynthesis levels for individual clock proteins may be a more general mechanism to engender distinct differences in clock parameters across cell types.

We noted that for the majority of clock genes (*Npas2*, *Cry1*, *Cry2*, *Per1*, *Per2*, *Per3*, *Nr1d1*, *Rorc*) the tissue differences were less pronounced at the RPF than at the RNA level (Fig. 5b), indicating that translation efficiencies partially counteracted RNA expression differences. Only in four cases (*Clock*, *Arntl*, *Nr1d2*, *Rora*) TEs exacerbated transcript abundance differences and led to higher tissue differences at the RPF level. Interestingly, this observation could also be made in the time-resolved data. As a measure of similarity between expression profiles that takes into account profile shape and expression level, we used the Euclidean distances calculated between the four rhythmic traces of each individual gene (i.e. RNA and RPF in kidney and liver; Additional file 1: Figure S13). Hierarchical clustering of the similarities for the ensemble of the 12 main core clock genes showed that RPF profiles from the two organs grouped together (Fig. 5c). The temporal profiles of clock protein biosynthesis between organs were thus more similar than RNA and RPF expression profiles within organs. By contrast, the 178 common rhythmic genes identified in Fig. 3c—serving as a control set for this analysis—revealed within-organ clustering (Fig. 5d). These findings underscored that translational compensation was occurring within the core clock, where it led to more similar expression profiles in clock protein biosynthesis than would have been predicted from the rhythmic RNA abundance. This phenomenon was, however, not a general feature of all rhythmic gene expression.

The transcriptome-wide analyses described further above had shown only weak signs of association between cross-organ differences in TE and in uORF usage (Additional file 1: Figure S8C–E). However, we knew from our previous work in liver that at least five core clock transcripts (*Nr1d1*, *Nr1d2*, *Cry1*, *Clock*, *Arntl*) contained translated, potentially regulatory, AUG-initiated uORFs [10]. We therefore examined whether for any of these concrete cases there was evidence for a connection between uORF translation and cross-organ TE differences. The read distribution along the transcripts (Additional file 1: Figure S14A) and the marked frame preference of RPF reads (Additional file 1: Figure S14B) confirmed that the footprints mapping to our annotated uORFs likely reflected active translation. However, only in one case, *Nr1d2*, there was a distinct anticorrelation between uORF usage and TE differences on the CDS (Fig. 5e). *Nr1d2* contains two translated uORFs in the 5′ UTR (Fig. 5f), whose decreased usage in kidney was accompanied with higher TE on the CDS in this organ (Fig. 5e). For *Nr1d2*, differential uORF usage could thus represent a plausible mechanism that contributes to regulating organ-specific gene expression output at the translational level, keeping NR1D2 biosynthesis low in liver and high in kidney.

## Discussion

Given that the functionally relevant output of most gene expression is the protein, quantitative and genome-wide analyses of protein biosynthesis are of high interest to complement the wealth of transcriptomics data that are already available. Of note, only the fairly recent development of the ribosome profiling technique [29] has made it possible to analyse translational events in a quantitative, high-throughput fashion. Our study of two paradigms of differential gene expression, i.e. its tissue-dependence and its time of day-dependence, is among the first of its kind and, together with the associated datasets and resources, will likely be of wide interest and utility to researchers working in the chronobiology and gene expression fields.

We have addressed several, rather fundamental questions that go beyond the chronobiological focus of the study: How does the dynamic range of translation efficiency compare to that of transcript abundances across two distinct organs of an animal? Is translation efficiency a default transcript property and comparable across two tissues or do TEs become reinterpreted depending on cell type or organ? Does cross-tissue variability of TEs come with any direction, i.e. is there a global tendency to either reinforce or to counteract transcriptomal differences?

To our knowledge, only one previous study has reported on ribosome profiling datasets from two complementary mammalian tissues: rat liver and heart [19]. This study also included animals with different genetic backgrounds as covariates in the experimental design and its main focus was on strain differences in translation rather than on tissue differences. Our analyses based on more than 10,000 genes commonly expressed in liver and kidney show that cross-organ TE differences are widespread, but of limited magnitude. Across genes in a tissue and for individual genes between tissues, the dynamic range of translation efficiencies is thus about 30–50-fold narrower than that of transcript abundances. These findings are coherent with the view that major differences in gene expression are set up at the level of transcription (possibly with some influence coming from RNA stability as well), whereas differences in translation rate have more of a modulatory role. It is intriguing that this modulation is overall characterised by directionality, with TE differences between tissues globally counteracting some of the mRNA abundance differences. Such translational compensation has previously been observed for divergent transcript expression levels across yeast species [30] and across different rat strains [19]; our study now extends this observation to gene expression across organs. Moreover, the idea of translational compensation is conceptually similar to findings that proteomes are evolutionarily more highly conserved than transcriptomes [31, 32]. As an underlying common principle, these cases may indicate that selective pressure on precise gene expression levels likely acts on protein abundances, whereas a certain degree of variability (even noise) in RNA levels may be tolerated without further consequences. It will be exciting to study the underpinnings of translational compensation further, across tissues and across species.

Maybe not unexpectedly, there was no dominant, distinct sequence feature that could serve as a predictor for cross-organ TE differences. Rather, we found several associations with a number of transcript characteristics. Conceivably, these contribute collectively to modulating TEs in concert with the specific cellular and tissue environment and possible cell-type differences in the translation machinery including its regulators and trans-acting factors. While our ribosome profiling studies have allowed us to record the outcome of such regulation at high resolution, understanding its causes represents an exciting challenge for the future. For now, we can only infer that an overarching theme of the identified associations is a connection to 5′ UTRs, which is in also in line with the notion that initiation is rate-limiting for most translation events. We thus observed associations of cross-organ TE differences with 5′ UTR length, with uORF usage, with GC content and folding potential, as well as with transcript isoform diversity that affected the 5′ UTR. We would like to point out that comprehensive uORF annotations remain a bioinformatics challenge that is far from resolved. We have therefore restricted our analyses to AUG-initiated ORFs, inevitably leading to a bias towards false-negatives in uORF annotation. As we will learn how to annotate uORFs more comprehensively and more precisely in the future, it may be worth revisiting the relationship between differential TE and uORF translation in our datasets in order to evaluate whether a clearer role for these regulatory sequence elements will emerge.

Our study has led to novel insights into rhythmic gene expression. The extent to which rhythmicity is generated by the temporal regulation of translation has been the subject of speculation ever since the first report of rhythmic proteins encoded by non-rhythmic mRNAs [7]. Our kidney datasets complement recent time-resolved ribosome profiling data from liver [10, 11] and from a cell line [33]. As compared to liver, the number of transcripts subject to translational rhythms in kidney is slightly lower, but overall in a similar order of magnitude with around 1% of the transcriptome affected. It came as a surprise that translational rhythms were essentially tissue-specific in terms of the affected genes and the phase distributions. A possible explanation could be that these rhythms are driven by rhythmic systemic cues to which tissues do not respond equally. The effects of feeding and mTOR signalling, for example, may be more pronounced in liver due to the dedicated role that this organ plays in energy homeostasis and fasting responses, thus explaining the differences in translational oscillations for RP genes. Beyond the role that translation has in generating rhythms, our analyses have pointed to an additional, rhythmicity-modulating role that appears to affect gene expression quite broadly, i.e. the timing of the phase of protein biosynthesis oscillations relative to that of mRNA abundance rhythms. Consistent with work by the Green lab that showed interactions between polyadenylation status of mRNAs and rhythmic protein expression in the liver [21], it is tempting to speculate that related mechanisms are operative across organs, with tissue-specific deadenylation kinetics tuning the timing of rhythmic protein biosynthesis. Finally, our study is a first attempt to resolve tissue differences in core clock gene expression as a factor contributing to functional differences of the oscillator. It is interesting that the core clock mechanism has a long-standing history of being referred to as a ‘transcription-translation feedback loop’ [34], although the actual feedback occurs at the transcriptional level and possible mechanistic functions of translational regulation have not been much investigated. Our cross-organ comparison of core clock protein biosynthesis suggests that translational control—including through the activity of uORFs [10, 33]—is of regulatory interest and represents a way by which the identical set of core clock genes could form circuitries with different stoichiometry of its main components. As a result, both clock parameters and output gene repertoires may be organ-specifically tuned.

## Conclusions

How translational differences contribute to overall gene expression diversity is still poorly understood. Our study uncovered translational changes that occur across two paradigms of regulated gene expression, i.e. around-the-clock and between tissues. Daily gene expression rhythms generated at the translational level were strongly organ-specific with regard to the identities and phase distributions of affected genes. Moreover, our data indicate that translation efficiency differences between organs can adjust the timing of protein production from rhythmic mRNAs and the levels of core clock protein production, in agreement with the tissue specificities observed in clock output gene sets and clock parameters. Together, these results are consistent with an important role of post-transcriptional mechanisms in mammalian circadian gene expression regulation. Beyond the temporal dimension, we have explored constitutive protein biosynthesis across organs. Our quantitative analyses underscore that gene expression divergence is largely programmed at the transcript abundance level. Interestingly, the widespread differences in translational efficiency that we detected between organs even serve to achieve higher concordance in protein production between tissues. Conceivably, such translational compensation reflects a selective pressure to maintain precise protein levels rather than mRNA levels. The high-resolution genome-wide translatome datasets generated in this study will allow further explorations into the mechanisms of post-transcriptional control and differential gene expression in vivo.

## Methods

### Animals

Twelve-week-old male mice (C57BL/6 J; Janvier Labs) were entrained for two weeks to light:dark 12:12 cycles with ad libitum access to food and water and were anesthetized (isoflurane) and sacrificed every 2 h (ZT0–ZT22, with ZT0 corresponding to ‘lights-on’) for two daily cycles. Livers and kidneys were removed and processed either directly or flash-frozen in liquid N_2_.

### Ribosome profiling

Generation of liver RPF-seq and RNA-seq libraries using the ARTseq ribosome profiling kit (Epicentre) was described recently [10, 16]. Kidney libraries were prepared in the same manner, with a single modification to the order of steps in RPF library preparation. After RNase treatment and recovery of ribosome-protected fragments, 5 μg of material was first ribosomal RNA (rRNA)-depleted (Ribo-Zero magnetic kit, Epicentre) and then purified by 15% PAGE. In the formerly prepared liver libraries, Ribo-Zero treatment and PAGE purification had been inverted because at the time we had found that changing the order had a beneficial effect on obtaining highly concentrated libraries. For the kidney samples, however, we noted that this modified order led to higher contamination with reverse-strand rRNA probes bleeding from the Ribo-Zero kit and we thus reverted to ARTseq’s original order. All other steps and materials were identical between liver and kidney samples and followed the ARTseq ribosome profiling kit instructions. RPF and RNA libraries were sequenced on an Illumina HiSeq 2500.

### Sequencing data processing, alignment and quantification

Processing, quality assessment, alignment and quantification of sequencing data were performed as described previously [10, 16]. Briefly, after adapter trimming using Cutadapt [35], the length distribution of trimmed reads was used to assess the quality of nuclease digestion and size-selection, which is particularly important for RPF libraries (Additional file 1: Figure S1B). Trimmed reads were filtered by size (26–35 nt for RPF; 21–60 nt for RNA) using an in-house Python script and sequentially mapped to mouse rRNA, human rRNA, mitochondrial tRNA, mouse tRNA, mouse cDNA (Ensemble mouse database release 75) using Bowtie v2.2.1 [36] and mouse genome (GRCm38.p2) using Tophat v2.0.11 [37]. Trimmed and filtered sequences were also directly mapped against the mouse genome (Tophat v2.0.11) in order to estimate expressed transcript models in each organ (using Cufflinks v2.2.1 [38]). Transcriptome-mapping reads in the sequential alignment were counted towards their location into the 5′ UTR, CDS or 3′ UTR of the transcript, based on feature annotation (Ensemble mouse release 75). Mappable and countable feature lengths were not calculated for this study (see ‘faux reads analysis’ in the ‘Quantification of mRNA and ribosome footprint abundance’ section of Supplemental Experimental Procedures of previous study [10]) as its contribution was negligible for further analyses. Therefore, RPKM calculations in this study were not corrected with such factor. Read counts in RNA-seq and RPF-seq datasets were normalised with upper quantile method of edgeR [39] and RPKM values were calculated as the number of reads per 1000 bases per geometric mean of normalised read counts per million. Relative translation efficiencies (TE) were calculated as the ratio of RPF-RPKM to RNA-RPKM per gene per sample. Reading frame and nucleotide periodicity analyses were performed as in [10]. PCA relied on a combined matrix of CDS counts for RPF and RNA from both liver and kidney and following the same approach as before [10]. Ribo-seq Unit Step Transformation (RUST) analysis was used to assess whether the sequencing libraries were globally of similar quality in terms of their local footprint densities [17]. RUST is a simple normalisation method that reduces the heterogeneous noise in the data and allows identification of mRNA sequence features that affect footprint densities globally. We used the version 1.2 of the published rust_codon.py standalone python script with minor modifications to reflect the experimental settings as closely as possible (i.e. A-site offsetting). RUST codon profile and corresponding Kullback–Leibler (K–L) divergence for each library (RPF and RNA) was generated against a database of 8012 single protein isoform transcript sequences using all mapped reads with a length of 28–32 nt. The K-L divergences from all samples for each combination of tissue (kidney or liver) and read type (RPF or RNA) were used to generate K-L profiles at the 0, 10, 25, 50, 75, 90 and 100th quantiles.

### Correlation analyses and assessment of translational compensation across organs

Correlation of RNA-seq and RPF-seq across organs: kidney versus liver correlations at the levels of RNA-seq and RPF-seq (i.e. Fig. 2b; Additional file 1: Figure S6C–E, H–L) were calculated in a pairwise fashion for each of the 24 samples (12 timepoints, two replicates/timepoint), as livers and kidneys of each replicate originated from the same animals. Significance of the difference in the Spearman coefficients between both distributions was assessed by paired t-test on Fisher z-transformed coefficients. Heart versus liver correlation at the levels of RNA and RPF-seq (Additional file 1: Figure S7A) was calculated from the study [19], using the BN-Lx reference rat strain data. Since the five heart and liver replicates in this study did not come from the same animals, we calculated all possible pairwise correlation coefficients between heart and liver (i.e. 25) and compared all possible combinations of five coefficients between RNA-seq and RPF-seq (paired t-test on Fisher z-transformed coefficients).

Measurement error: measurement errors (Additional file 1: Figure S6A, B, F, G) were calculated similarly to [18] using the meas.est() function from smatr R package [40]. Genes were first binned according to average expression level (calculated as the fourth root of the product of liver RNA-seq, liver RPF-seq, kidney RNA-seq and kidney RPF-seq) into ten groups, each containing 10% of all genes. Within each bin, the measurement error was calculated separately for RNA-seq and RPF-seq and for liver and kidney, using the two replicates (log of normalised CDS counts) to estimate the error and the 12 timepoint samples to estimate its variability. For the analyses using a filtered gene set (Additional file 1: Figure S6F-G), genes that showed a mean expression ratio (either between organs or between RNA-seq and RPF-seq) greater than 2 for all timepoints were excluded (9236 genes used in analysis).

### Analyses of differential translation efficiency

To test for differential translation efficiency (TE) between liver and kidney we used the Wilcoxon-signed rank paired test, using all 24 samples (12 timepoints; two replicates/timepoint) as replicates; resulting *p* values were FDR-corrected. A gene was defined as having differential TE when FDR < 0.01 and the inter-organ difference in TE was at least 1.5-fold (Fig. 2e).

Analysis of transcript usage diversity across organs: for each gene g, P(g) = (p 1,…,p n) is the vector of the relative expression proportions of its n protein-coding transcripts, as estimated from our RNA-seq analysis (see ‘Sequencing data processing, alignment and quantification’). To quantify the dissimilarity in relative transcript isoform expression between liver L and kidney K, the Hellinger distance H is defined as:

1 $$ H\left({P}_L(g),{P}_K(g)\right)=1/\sqrt{2}\sqrt{{\displaystyle \sum_{i=1}^n}{\left(\sqrt{p_L^i}-\sqrt{p_K^i}\right)}^2} $$

In order to detect the transcript features that were associated with tissue specificity in TE, we selected genes whose transcript diversity between both organs originated from or was excluded from 5′ UTR, CDS, or 3′ UTR, based on feature annotation information for the detected protein-coding transcripts (Fig. 2g and Additional file 1: Figure S9).

Study of transcript characteristics: for single-isoform genes, we investigated whether a particular transcript characteristic (length, GC content, Kozak context, structure) could be predictive of differential TE. Length and GC content were determined directly on the whole transcript and/or on the region of interest (5′ UTR, CDS, 3′ UTR). Kozak context was scored according to the consensus sequence GccA/Gcc**AUG**G, where upper-case letters denote highly conserved bases (scored +3), lower-case letters indicate the most common nucleotides (scored +1) and bold is the start codon (not scored), giving a maximum score of 13. Minimum free energy secondary structures on the 5′ UTR were predicted with RNAfold from ViennaRNA package with default parameters [41].

### Detection and translation efficiency calculation for uORFs

To assess the impact of differential uORF usage on TE differences across organs, uORFs were identified as in our previous study [10]. Briefly, genes expressing a single protein-coding isoform in both organs were used for this analysis (*n* = 5815). We selected uORFs with an AUG start codon and a length of at least 18 nt to the first in-frame stop codon and considered them as translated if the reads showed significant frame bias towards the reading frame of the uORF start codon and if coverage was > 10%. uORF translation efficiency was calculated from the ratio of RPF-seq to RNA-seq reads whose predicted A-sites mapped to the annotated uORF regions. If several uORFs partially of completely overlapped on a given 5′ UTR, a composite uORF was considered for read counting. uORFs overlapping with the CDS in the same frame were not considered. When they overlapped in different frames, only reads mapping to the 5′ UTR-specific uORF sequence (but not the overlapping sequence) was considered for quantifications.

### Rhythmicity analyses

Rhythmicity detection and rhythmic parameter estimations in each dataset (RNA-seq and RPF-seq, liver and kidney) were done based on Akaike information criterion (AIC) model selection as in our previous study [10]. The Babel computational framework [22] was used to detect rhythmically translated genes from constantly expressed mRNAs within each organ. For cross-correlation of time series to compare the daily profiles of rhythmic genes beyond their peak differences, we used the ccf function in R. As we computed the correlations of the RPF-seq with respect to the RNA-seq profiles, negative lag values correspond to RPF leading RNA.

### Hierarchical clustering of rhythmic genes

To evaluate the similarity of the expression profiles for rhythmic genes, a dissimilarity matrix was computed for each gene of interest, based on the Euclidean distance between the RNA-seq and RPF-seq expression profiles within and across organs. A hierarchical clustering tree was constructed on the weighted average of the dissimilarity matrices of genes under consideration (core clock genes in Fig. 5c or all rhythmic genes in Fig. 5d), using the ‘average’ clustering method. The R functions {packages} dist {stats}, fuse {analogue} and hclust {stats} were used for computing the individual dissimilarity matrices, the weighted mean dissimilarity matrix and the hierarchical clustering, respectively.

## Additional files

Additional file 1: Supplementary Figures. This file contains the Supplementary **Figures S1–S14** and Supplementary Figure legends. (PDF 32661 kb)

Additional file 2: Mapping outcome summary. This file contains information on the deep-sequencing data from kidney (raw read counts, mapping summary etc.) (XLSX 23 kb)

Additional file 3: Differential TE analysis. This file contains details of the GO-term analysis on the differential TE gene set of Fig. 2. (XLSX 228 kb)

Additional file 4: Rhythmicity parameters in kidney datasets. This file contains the outcome of the transcriptome-wide rhythmicity analyses on the kidney datasets (related to Fig. 3a). (XLSX 163 kb)

Additional file 5: Rhythmicity parameters of 178 common rhythmic genes. This file contains the outcome of the rhythmicity analyses in kidney and liver for the 178 commonly rhythmic genes (RNA and RPF in kidney and liver; related to Fig. 3c). (XLSX 35 kb)

Additional file 6: Transcriptome-wide kidney RPF (blue) and RNA (orange) levels in the left panels (with “error bars” connecting the two replicates of each timepoint) and TE in the right panels. (ZIP 116896 kb)

Additional file 7: Expression plots for kidney and liver for the 178 common rhythmic genes of Fig. 3c. (ZIP 3338.28 kb)

## References

[1] Dibner C, Schibler U, Albrecht U (2010). The mammalian circadian timing system: organization and coordination of central and peripheral clocks. Annu Rev Physiol.

[2] Partch CL, Green CB, Takahashi JS (2014). Molecular architecture of the mammalian circadian clock. Trends Cell Biol.

[3] Zhang R, Lahens NF, Ballance HI, Hughes ME, Hogenesch JB (2014). A circadian gene expression atlas in mammals: Implications for biology and medicine. Proc Natl Acad Sci U S A.

[4] Meireles-Filho AC, Bardet AF, Yanez-Cuna JO, Stampfel G, Stark A (2014). cis-regulatory requirements for tissue-specific programs of the circadian clock. Curr Biol.

[5] Yoo SH, Yamazaki S, Lowrey PL, Shimomura K, Ko CH, Buhr ED (2004). PERIOD2::LUCIFERASE real-time reporting of circadian dynamics reveals persistent circadian oscillations in mouse peripheral tissues. Proc Natl Acad Sci U S A.

[6] Luck S, Westermark PO (2016). Circadian mRNA expression: insights from modeling and transcriptomics. Cell Mol Life Sci.

[7] Reddy AB, Karp NA, Maywood ES, Sage EA, Deery M, O’Neill JS (2006). Circadian orchestration of the hepatic proteome. Curr Biol.

[8] Mauvoisin D, Wang J, Jouffe C, Martin E, Atger F, Waridel P (2014). Circadian clock-dependent and -independent rhythmic proteomes implement distinct diurnal functions in mouse liver. Proc Natl Acad Sci U S A.

[9] Robles MS, Cox J, Mann M (2014). In-vivo quantitative proteomics reveals a key contribution of post-transcriptional mechanisms to the circadian regulation of liver metabolism. PLoS Genet.

[10] Janich P, Arpat AB, Castelo-Szekely V, Lopes M, Gatfield D (2015). Ribosome profiling reveals the rhythmic liver translatome and circadian clock regulation by upstream open reading frames. Genome Res.

[11] Atger F, Gobet C, Marquis J, Martin E, Wang J, Weger B (2015). Circadian and feeding rhythms differentially affect rhythmic mRNA transcription and translation in mouse liver. Proc Natl Acad Sci U S A.

[12] Jouffe C, Cretenet G, Symul L, Martin E, Atger F, Naef F (2013). The circadian clock coordinates ribosome biogenesis. PLoS Biol.

[13] Bonny O, Vinciguerra M, Gumz ML, Mazzoccoli G (2013). Molecular bases of circadian rhythmicity in renal physiology and pathology. Nephrol Dial Transplant.

[14] Ingolia NT (2014). Ribosome profiling: new views of translation, from single codons to genome scale. Nat Rev Genet.

[15] Brawand D, Soumillon M, Necsulea A, Julien P, Csardi G, Harrigan P (2011). The evolution of gene expression levels in mammalian organs. Nature.

[16] Janich P, Arpat AB, Castelo-Szekely V, Gatfield D (2016). Analyzing the temporal regulation of translation efficiency in mouse liver. Genom Data.

[17] O’Connor PB, Andreev DE, Baranov PV (2016). Comparative survey of the relative impact of mRNA features on local ribosome profiling read density. Nat Commun.

[18] Albert FW, Muzzey D, Weissman JS, Kruglyak L (2014). Genetic influences on translation in yeast. PLoS Genet.

[19] Schafer S, Adami E, Heinig M, Rodrigues KE, Kreuchwig F, Silhavy J (2015). Translational regulation shapes the molecular landscape of complex disease phenotypes. Nat Commun.

[20] Gonzalez-Porta M, Calvo M, Sammeth M, Guigo R (2012). Estimation of alternative splicing variability in human populations. Genome Res.

[21] Kojima S, Sher-Chen EL, Green CB (2012). Circadian control of mRNA polyadenylation dynamics regulates rhythmic protein expression. Genes Dev.

[22] Olshen AB, Hsieh AC, Stumpf CR, Olshen RA, Ruggero D, Taylor BS (2013). Assessing gene-level translational control from ribosome profiling. Bioinformatics.

[23] Lee Y, Chen R, Lee HM, Lee C (2011). Stoichiometric relationship among clock proteins determines robustness of circadian rhythms. J Biol Chem.

[24] Yagita K, Horie K, Koinuma S, Nakamura W, Yamanaka I, Urasaki A (2010). Development of the circadian oscillator during differentiation of mouse embryonic stem cells in vitro. Proc Natl Acad Sci U S A.

[25] Landgraf D, Wang LL, Diemer T, Welsh DK (2016). NPAS2 compensates for loss of CLOCK in peripheral circadian oscillators. PLoS Genet.

[26] Fang B, Lazar MA (2015). Dissecting the Rev-erbalpha cistrome and the mechanisms controlling circadian transcription in liver. Cold Spring Harb Symp Quant Biol.

[27] D’Alessandro M, Beesley S, Kim JK, Chen R, Abich E, Cheng W (2015). A tunable artificial circadian clock in clock-defective mice. Nat Commun.

[28] Gu X, Xing L, Shi G, Liu Z, Wang X, Qu Z (2012). The circadian mutation PER2(S662G) is linked to cell cycle progression and tumorigenesis. Cell Death Differ.

[29] Ingolia NT, Ghaemmaghami S, Newman JR, Weissman JS (2009). Genome-wide analysis in vivo of translation with nucleotide resolution using ribosome profiling. Science.

[30] McManus CJ, May GE, Spealman P, Shteyman A (2014). Ribosome profiling reveals post-transcriptional buffering of divergent gene expression in yeast. Genome Res.

[31] Khan Z, Ford MJ, Cusanovich DA, Mitrano A, Pritchard JK, Gilad Y (2013). Primate transcript and protein expression levels evolve under compensatory selection pressures. Science.

[32] Schrimpf SP, Weiss M, Reiter L, Ahrens CH, Jovanovic M, Malmstrom J (2009). Comparative functional analysis of the Caenorhabditis elegans and Drosophila melanogaster proteomes. PLoS Biol.

[33] Jang C, Lahens NF, Hogenesch JB, Sehgal A (2015). Ribosome profiling reveals an important role for translational control in circadian gene expression. Genome Res.

[34] Dunlap JC (1996). Genetics and molecular analysis of circadian rhythms. Annu Rev Genet.

[35] Martin M (2011). Cutadapt removes adapter sequences from high-throughput sequencing reads. EMBnetjournal.

[36] Langmead B, Salzberg SL (2012). Fast gapped-read alignment with Bowtie 2. Nat Methods.

[37] Trapnell C, Pachter L, Salzberg SL (2009). TopHat: discovering splice junctions with RNA-Seq. Bioinformatics.

[38] Trapnell C, Williams BA, Pertea G, Mortazavi A, Kwan G, van Baren MJ (2010). Transcript assembly and quantification by RNA-Seq reveals unannotated transcripts and isoform switching during cell differentiation. Nat Biotechnol.

[39] Robinson MD, Oshlack A (2010). A scaling normalization method for differential expression analysis of RNA-seq data. Genome Biol.

[40] Warton DI, Wright IJ, Falster DS, Westoby M (2006). Bivariate line-fitting methods for allometry. Biol Rev Camb Philos Soc.

[41] Lorenz R, Bernhart SH, Honer Zu Siederdissen C, Tafer H, Flamm C (2011). ViennaRNA Package 2.0.. Algorithms Mol Biol.

[42] Castelo-Szekely V. gatfieldlab/cross-organ_riboprof cross-organ_riboprof_v1. Zenodo 2017.

