# Supplementary material for: Translational contributions to tissue specificity in rhythmic and constitutive gene expression
Source: Genome Biol. 2017 Jun 16;18:116. doi: 10.1186/s13059-017-1222-2 (PMC5473967; doi:10.1186/s13059-017-1222-2)
Supplement: Supplementary file 1 — Supplementary Figures. This file contains the Supplementary Figures S1–S14 and Supplementary Figure legends. (PDF 32661 kb) [file 13059_2017_1222_MOESM1_ESM.pdf]

SUPPLEMENTARY FIGURE LEGENDS

Translational contributions to tissue-specificity in rhythmic and constitutive gene expression

Violeta Castelo-Szekely, Alaaddin Bulak Arpat, Peggy Janich, David Gatfield

Figure S1. Overview of sequencing outcome and read length distribution for RPF- and RNA-seq data used in the study.

A Summary of outcome of the sequential mapping pipeline, indicating the number (y-axis) and percentage (within bars) of reads mapping to each database, averaged over all timepoints. For each sample of the four datasets an average of more than 20 million reads mapped to the protein-coding transcriptome (cDNA) and was used for the study.

B and C RPF-seq (B) and RNA-seq (C) read length after trimming of adaptors showed that most RPF-seq reads had a length of 29-30 nucleotides in both organs, whereas RNA-seq fragments showed a broader distribution as expected from chemical RNA fragmentation. Boxplots represent the interquartile range and whiskers extend to the minimum and maximum values within 1.5 times the interquartile range.

Figure S2. RPF reads at the stop codon and quality control.

A A-site position of RPF-seq reads in the last 200 nt of the CDS, excluding stop codon reads. Read density along the CDS was similar in liver and kidney; thus the higher read density observed in Fig. 1C at the stop codon does not affect CDS-based calculations.

B Zooming into the footprint read density at the end of the CDS, stop codon, and beginning of the 3′ UTR indicated read differences between organs for the stop codon itself and up to 4 nt downstream, which were increased in kidney. Stop codon reads are counted towards the 3′ UTR and their higher level in kidney thus also explains why our analysis in Fig. 1B shows more 3′ UTR reads for this organ. The remainder of the 3′ UTR shows a similar depletion of reads in both organs.

C and D Ribo-seq Unit Step Transformation (RUST) metafootprint analyses to evaluate the contribution of local mRNA positions to the density of footprints. Light and dark coloured polygonal areas denote the 10%-90% and 25%-75% percentiles,

respectively, and the dash-line denotes the median of the Kullback–Leibler divergence (K–L) profiles of all samples within kidney (C) and liver (D) separately for RPF and RNA reads (colour code in inset). The K-L profile for each sample was calculated from the RUST ratio values of 61 sense codons across a moving window of 40 triplet codons upstream to 20 triplet codons downstream of predicted A-sites. High K-L divergence maxima (lowest relative entropy or highest information gain) are thus found in the vicinity of the A-site in RPF libraries, and the 5′ and 3′ termini of the reads in RNA libraries. Importantly, the profiles are similar for kidney and liver, indicating overall similar footprint quality in the two independent datasets.

Figure S3. High technical and biological reproducibility of datasets.

A and B Spearman correlation of normalised CDS read counts between timepoints and between replicates for kidney RPF-seq (A) and kidney RNA-seq (B) datasets. The correlation coefficient is indicated by the size and shading of the disks. Biological replicates thus show excellent correlation; moreover, the correlation coefficients of different timepoints reflect the rhythmic nature of the data.

C and D Normalised CDS read counts (RPKM) in liver vs. kidney at the RNA (C) and RPF level (D). In these graphs, the averages over all timepoints were compared for the set of commonly expressed genes (N=10289; see Fig. 2A). Note the overall higher Spearman correlation at the footprint level than transcript level. The difference between correlations is highly significant with p=8.7e-110, Z value=-22.23; Steiger test for difference between 2 dependent correlations (Reference: Steiger JH, 1980. Psychological Bulletin, 87, 245-251).

Figure S4. Additional information for Principal Component Analysis.

Scree plot showing the first 10 components of the PCA in Fig. 1D-E. Components 1 and 2 explained most variance, followed by PC3 to PC5, which explained a closely similar proportion of variance in the data; the plateau was apparent from the sixth component.

Figure S5. Contribution of translation efficiency to overall gene expression variation within organs.

A and B Scatterplot of mRNA abundance vs. translation efficiency (TE) in kidney (A; N=12423 genes) and liver (B; N=10676 genes), averaged over all timepoints. Corresponding density lines are plotted on the margins. Dotted red lines represent the 2.5 and 97.5 percentiles, and the corresponding fold change is indicated. The transcript abundance range for 95% of genes thus spanned two orders of magnitude (>500-fold range in either organ), whereas TE dynamic range was less than 12-fold in either organ. Transcript abundance differences can thus be considered the main source of gene expression variability in the tissues. Moreover, Pearson's r values of 0.145 (kidney) and 0.196 (liver) indicate weak positive correlation of transcript abundance and TE.

C Scatterplot of transcript abundance (TA) vs. translation efficiency (TE) in main CDS for kidney and as averages over all timepoints. Highlighted are single protein-coding genes that contain (red) or do not contain (blue) translated uORFs. Corresponding density lines are plotted on the margins. uORF translation is thus clearly associated with significantly reduced translation efficiency. Numbers on the density curves indicate the location shift relative to all transcripts. Genes with translated uORFs: TA, $p=0.16$; TE, $p<2.2e-16$ (Wilcoxon rank sum test). Genes without translated uORFs: TA, $p=8.7e-5$; TE, $p<2.2e-16$ (Wilcoxon rank sum test).

Figure S6. Translational compensation is independent of technical biases in the datasets.

A and B Measurement error (ME) of all genes in the dataset (n=10289) was calculated separately for RNA-seq (red) and RPF-seq (turquoise) for kidney (A) and liver (B), and plotted as a function of increasing average expression levels. Briefly, measurement errors within each bin were calculated as in Albert et al., 2015 (Albert FW, Muzzey D, Weissman JS, Kruglyak L, 2014. PLoS Genetics, 10, e1004692)

using the 12 timepoints as replicates for the measurement estimates (see Methods).

C to E Spearman correlation between liver and kidney for RNA and RPF data for (C) genes showing the highest measurement errors (bin 1 in A, B), (D) bins 2-10, and (E) genes that have higher measurement error in RPF-seq than in RNA-seq samples in both organs (bins 7-10). Each boxplot contains the correlation coefficients between organs for each timepoint and replicate sample. Together these analyses showed that RPF-seq samples have a higher degree of similarity across organs (indicated p-values are from paired t-tests on Fisher-transformed correlation coefficients), even when considering lowly expressed genes with higher associated experimental error (C), or when considering genes with higher RPF-seq than RNA-seq measurement errors (E). These results thus ruled out that a systematic lower measurement error in RPF-seq experiments could have been the underlying cause of the higher correlation in RPF-seq than RNA-seq observed in Fig. 2C.

F and G Same as A and B, but with a filtered gene set in which specifically those genes that showed very different expression levels/high variability between organs or between datasets (RPF-seq, RNA-seq) were removed (see Methods). The reason to also analyse such a filtered set was that we wished to be sure that genes that were widely different in their gene expression level were not distorting the analyses (e.g. specifically causing extreme measurement errors under a condition where expression was very low). Moreover, because the binning into the groups was based on expression level across all sets (calculated as the fourth root of the product of liver RNA-seq, liver RPF-seq, kidney RNA-seq and kidney RPF-seq), the highly variable genes made binning inaccurate. This filtered set thus contained genes with overall better comparability across datasets; of note, the distribution of ME differences using the filtered set was very similar to the full set in A-B.

H to L Inter-organ Spearman correlation in RNA-seq and RPF-seq samples for various gene bins as indicated, using the filtered set. As in C-E, even when considering for example the genes with the highest overall measurement error (H), or

the genes with higher RPF-seq than RNA-seq measurement errors in both organs (L), a significantly higher correlation is observed in RPF-seq samples (paired t-test on Fisher-transformed correlation coefficients).

Figure S7. Translational compensation detected in rat liver and heart.

A) Spearman correlation coefficient between rat heart and liver samples calculated from the data of Schafer et al., 2015 (Schafer S, Adami E, Heinig M, Rodrigues KE, Kreuchwig F, Silhavy J, van Heesch S, Simaite D, Rajewsky N, Cuppen E, Pravenec M, Vingron M, Cook SA, Hubner N, 2015. Nature Communications 8, 7200). Each boxplot contains the correlation coefficients of all possible pairwise comparisons between heart and liver replicates (remark: in this study, organs in each of the five replicates did not necessarily come from the same animals, thus precluding defined pairwise comparisons between same animals). The indicated p-value is the 95th percentile of the ensemble of p-values resulting from all possible comparisons between RPF-seq and RNA-seq correlation coefficients (paired t-test of Fisher-transformed coefficients). This analysis extended our observation of a globally higher conservation between organs at the level of translational output (protein production) than at the level of transcript abundance.

B and C Normalised CDS read counts (RPKM) in rat liver vs. heart at the RPF-seq (B) and RNA-seq level (C), averaged over the five replicates used in the study of Schafer et al., 2015. Note the overall higher Spearman correlation at the footprint level as compared to the mRNA level. The difference between correlations is highly significant with p=2.3e-201, Z value=-30.25; Steiger test for difference between 2 dependent correlations (Reference: Steiger JH, 1980. Psychological Bulletin, 87, 245-251).

Figure S8. Analysis of transcript features with predictive value for differential TE.

A Cumulative distribution of the indicated transcript features for single isoform genes

that do not show differential TE (black, n= 5278), or that show differential TE and either higher TE in kidney (yellow, N=193) or in liver (green, N=340). The indicated p-values are Kolmogorov-Smirnov test results of each group vs. 'all'. Statistically significant comparisons marked in red.

B Same as (A), but in the form of boxplots and using Wilcoxon rank-sum test for the differences between group means (again, marked in red, significant results).

C Fraction of single isoform genes with (blue) or without (red) translated uORFs in either organ. The group with differential and higher TE in liver contained significantly more translated uORF-containing transcripts than the genes not showing differential TE in either organ (p=6.08e-04; Fisher's exact test); for kidney, there was a slight depletion of uORF-containing transcripts (non-significant). This analysis indicates that uORF usage may play a role in setting TE differences across tissues. Note that in this analysis, we did not yet distinguish whether the uORF was translated in liver and/or kidney, but we treated the 1377 genes with a translated uORF in at least one organ as a single group.

D Organ-specific uORF usage and its association with differential TE. The group of genes with uORFs specifically translated in liver was enriched for transcripts better translated in in kidney, and vice versa, consistent with a role of tissue-specific uORF usage in setting TE differences. However, due to the low number of differential TE genes exhibiting uORF translation that was exclusive to one organ for this analysis, the enrichments and depletions did not reach statistical significance.

E Scatterplot of upstream ORF vs. CDS TE differences across organs for genes containing translated uORFs in both organs and detected as differential TE with higher TE in kidney (yellow) or liver (green), or not showing differential TE (grey). An anticorrelation between uORF usage and CDS TE was only observed for genes with differential and higher TE in liver.

Figure S9. Relationship between transcript diversity and differential TE.

A Cumulative distribution of the absolute kidney-to-liver TE ratio for genes whose transcript diversity is present or absent only in the indicated feature. The vertical dotted grey line marks the 1.5-fold difference used to define differential TE. In this Figure, all 7 groups are plotted (transcript diversity only/not in 5′ UTR, CDS, 3′ UTR; all genes); for better visibility, the analyses of individual features are also shown in separate panels, i.e. in Fig. 2G (5′ UTR), Fig. S9B (CDS) and Fig. S9C (3′ UTR). Collectively, these results showed that transcript diversity that originated only within the CDS (red), or that was excluded from the 5′ UTR (purple), or that was present only within the 3′ UTR (dark green), all showed smaller TE differences across organs, thus pointing towards variability within the 5′ UTR as a contributor to tissue-specific TE.

B As in (A) showing the genes with transcript diversity present (red) or absent (orange) only in the CDS. Note that when transcript diversity is only in CDS (i.e. UTRs are identical), there is a significant shift to more similar TEs in both organs. This is consistent with the specific association of 5′ UTR diversity with differential TE that is shown in Fig. 2G.

C As in (A) showing genes with transcript diversity present (dark green) or absent (light green) only in the 3′ UTR.

Figure S10. Analysis of phase differences in RNA and RPF rhythms in kidney and across organs.

A Histogram of phase differences (RPF – RNA, in hours) for all genes that were detected as rhythmic in the kidney RPF and RNA data (N=542; see Fig. 3A). Although the distribution mean was not significantly different from 0, more genes had their footprint abundance peak advanced (N=282) than delayed (N=260) with respect to their mRNA abundance peak.

B and C Histogram of the phase differences (footprints to mRNA abundance, in hours) in kidney (B) and liver (C) for the 178 genes rhythmic in both organs (gene set

shown in Fig. 3C). We observed a broader distribution of phase differences in kidney and globally a phase advance of RPF with respect to RNA (-0.143 hours), as compared to overall stronger phase coherence of RPF and RNA in liver. See also Figures 3D-E.

D Histogram of the differential (kidney – liver) phase difference (RPF – RNA) for the 178 genes that were rhythmic throughout (Fig. 3C). Although statistically not reaching significance, the mean of -0.178 hours and the overall more genes for which the phase difference had negative values (96 vs. 82 genes) were consistent with the finding that RPF rhythms peak earlier than RNA rhythms specifically in kidney.

Figure S11. Higher expression of deadenylase complex subunits in kidney.

A and B Daily expression profiles of the CCR4-NOT complex components in kidney (A) and liver (B) at the RNA (orange) and RPF (blue) level.

C RPF expression of the CCR4-NOT subunits (averages over the day). Boxplots represent the interquartile range and whiskers extend to the minimum and maximum expression within 1.5 times the interquartile range. Note that differences in protein biosynthesis are statistically significant for all subunits ($p<0.05$, two-sample t-test) apart from *Cnot8*.

Figure S12. Heatmaps of all detected RPF and RNA rhythms indicate false-negatives of the rhythmicity detection method.

A Same as Fig. 3A, but re-plotted here for ease of comparison with (B-D).

B-D Heatmap of RNA-seq (left) and RPF-seq (right) expression for genes detected as rhythmic only at the mRNA level (B, N=796), at both levels (C, N=542), and at the ribosome footprints only (D, N=435) in kidney. Gene expression levels are standardised by row (gene). Please note that even the panels that should represent “non-rhythmicity” (i.e. right panel in B and left panel in D) clearly showed underlying rhythmicity, albeit with more noise and/or lower amplitude. Many of these cases were

therefore probably not truly “non-rhythmic” but rather false-negatives of the detection method (see Results section).

Figure S13. Core clock gene expression at RNA and RPF levels in both organs. **Left panels:** Daily expression profiles of the 12 main core clock genes shown in Fig. 5A-C. **Right panels:** Hierarchical clustering of the organs' RNA and RPF profiles for each clock gene. Branch height represent the average Euclidean distance. Note that for 7 out of the 12 core clock genes, protein synthesis profiles were more conserved across organs than mRNA abundance and than RPF-RNA within organs.

Figure S14. Read distribution for uORF-containing core clock genes.

A Normalised read distribution for RPF (in blue) and RNA (in orange) along core clock transcripts containing uORFs in kidney (top) and liver (bottom) for the timepoint of maximal CDS translation. Red boxes indicate AUG-initiated uORFs as predicted in our analyses. For scaling issues and better visualisation, only a portion of the 3′ UTRs, corresponding to the same length as the full 5′ UTR, is depicted (exception *Nr1d1*, for which the 3′ UTR is so short that it is shown full length).

B Read distribution to the three translation frames showed a frame bias of footprint reads for most predicted uORFs that was in a similar range as the frame bias on the CDS. This frame preference is indicative of active translation on the uORFs.

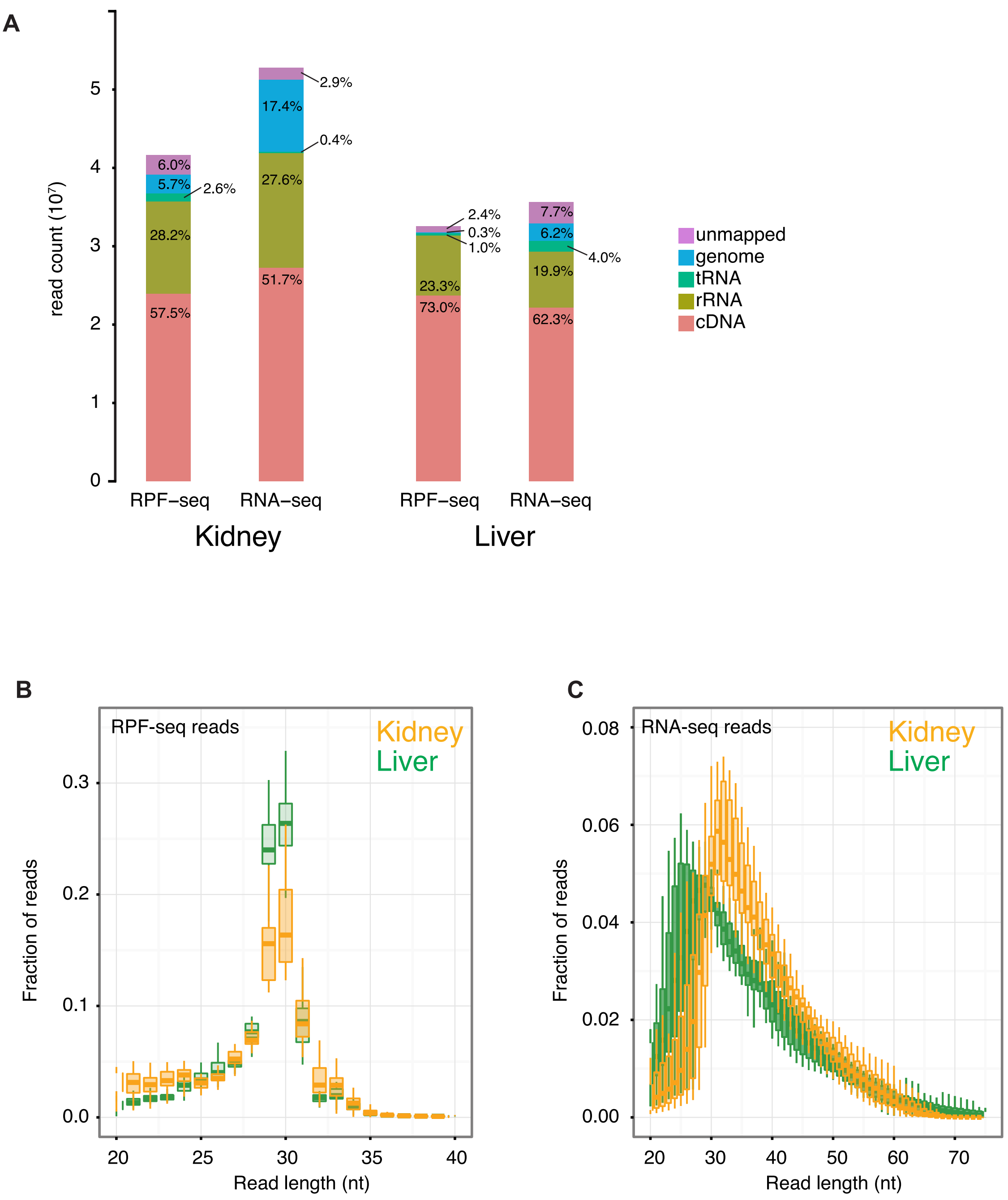
A
read count (10^7)
0
1
2
3
4
5
6.0%
5.7%
2.6%
28.2%
57.5%
2.9%
17.4%
0.4%
27.6%
51.7%
2.4%
0.3%
1.0%
23.3%
73.0%
7.7%
6.2%
4.0%
19.9%
62.3%
unmapped
genome
tRNA
rRNA
cDNA
RPF−seq
RNA−seq
RPF−seq
RNA−seq
Kidney
Liver
B
RPF-seq reads
Kidney
Liver
Fraction of reads
0.0
0.1
0.2
0.3
20
25
30
35
40
Read length (nt)
C
RNA-seq reads
Kidney
Liver
Fraction of reads
0.00
0.02
0.04
0.06
0.08
20
30
40
50
60
70
Read length (nt)

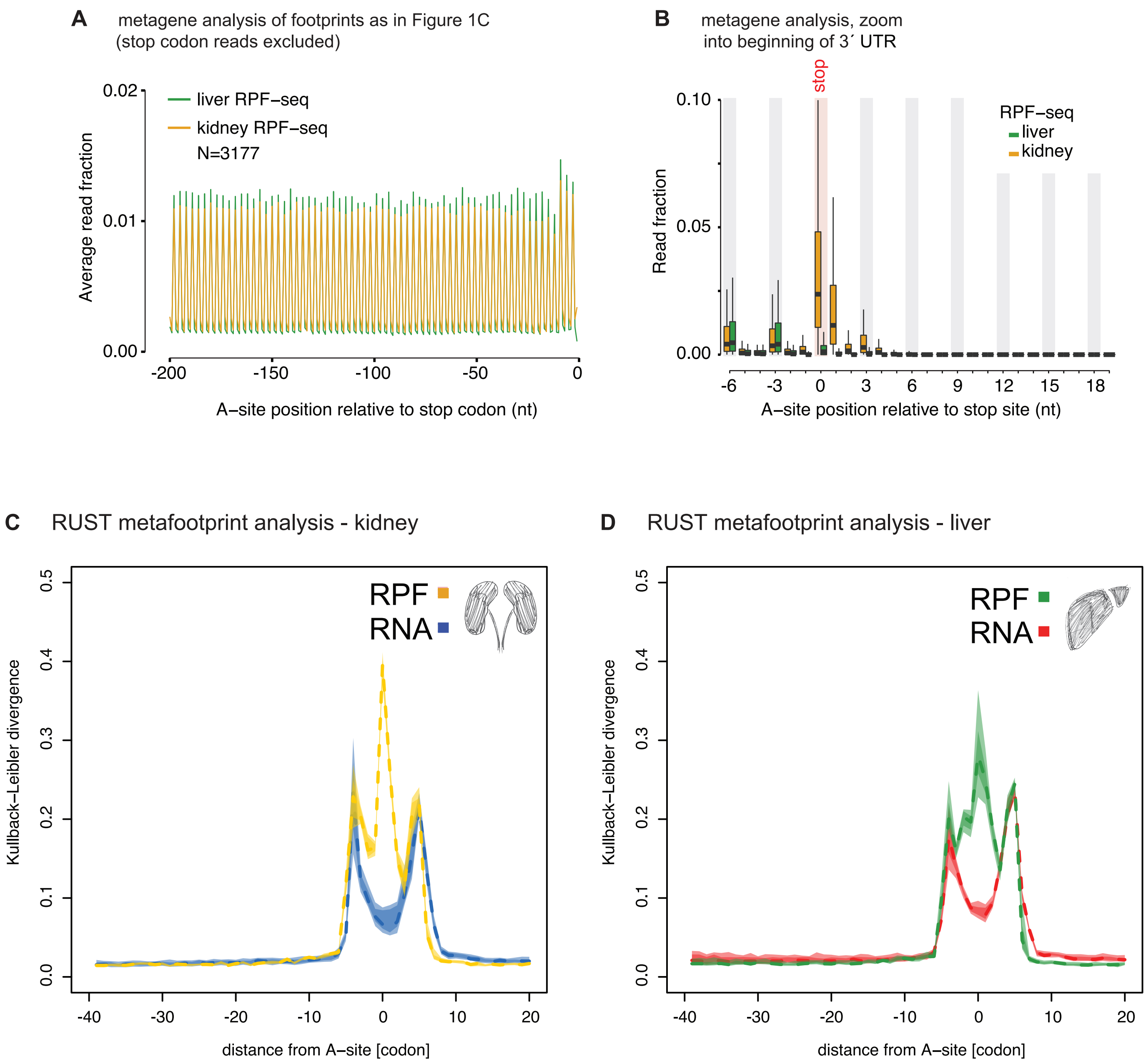
A
metagene analysis of footprints as in Figure 1C
(stop codon reads excluded)
liver RPF–seq
kidney RPF–seq
N=3177
Average read fraction
0.00
0.01
0.02
-200
-150
-100
-50
0
A–site position relative to stop codon (nt)
B
metagene analysis, zoom
into beginning of 3´ UTR
stop
RPF–seq
liver
kidney
Read fraction
0.00
0.05
0.10
-6
-3
0
3
6
9
12
15
18
A–site position relative to stop site (nt)
C
RUST metafootprint analysis - kidney
RPF
RNA
Kullback–Leibler divergence
0.0
0.1
0.2
0.3
0.4
0.5
-40
-30
-20
-10
0
10
20
distance from A–site [codon]
D
RUST metafootprint analysis - liver
RPF
RNA
Kullback–Leibler divergence
0.0
0.1
0.2
0.3
0.4
0.5
-40
-30
-20
-10
0
10
20
distance from A–site [codon]

Castelo-Szekely_Supplementary FigS3

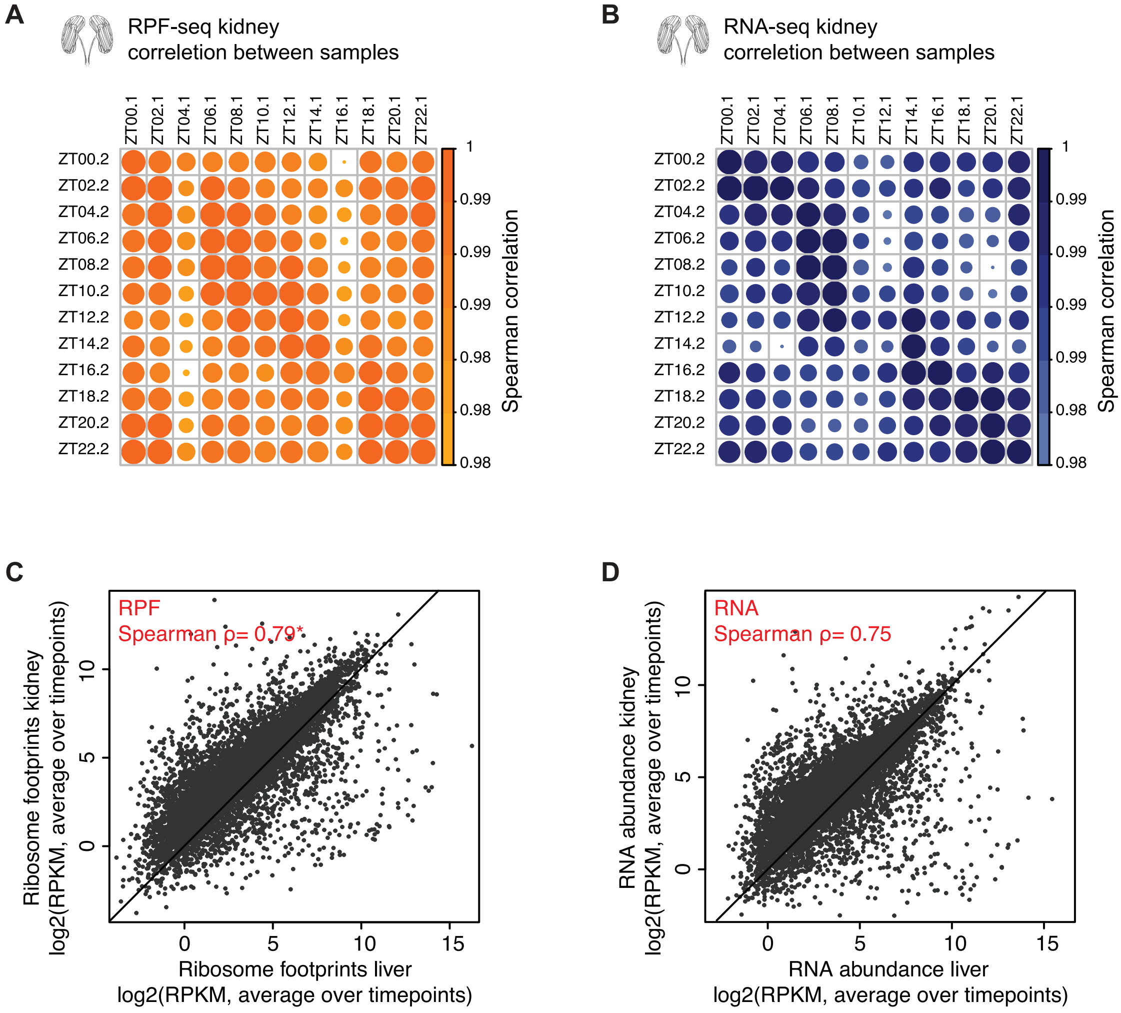

* correlations are significantly different
Steiger test for difference between 2 dependent correlations, Z value=-22.23, p=8.7e-110 (n=10289)

Steiger, J. H. (1980). Tests for comparing elements of a correlation matrix. Psychological Bulletin, 87, 245-251.

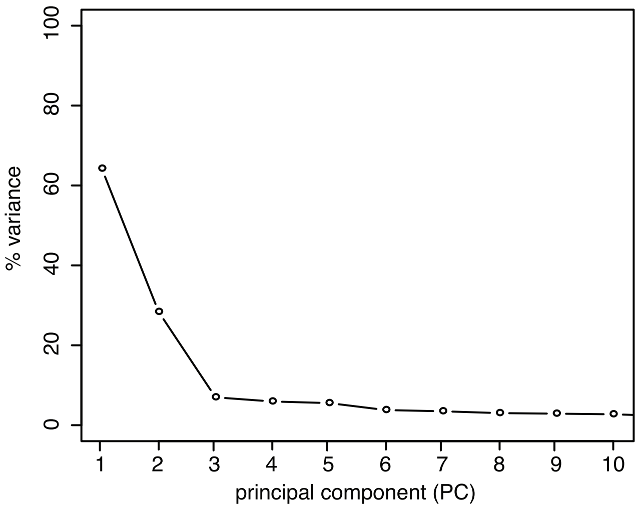
% variance
0
20
40
60
80
100
1
2
3
4
5
6
7
8
9
10
principal component (PC)

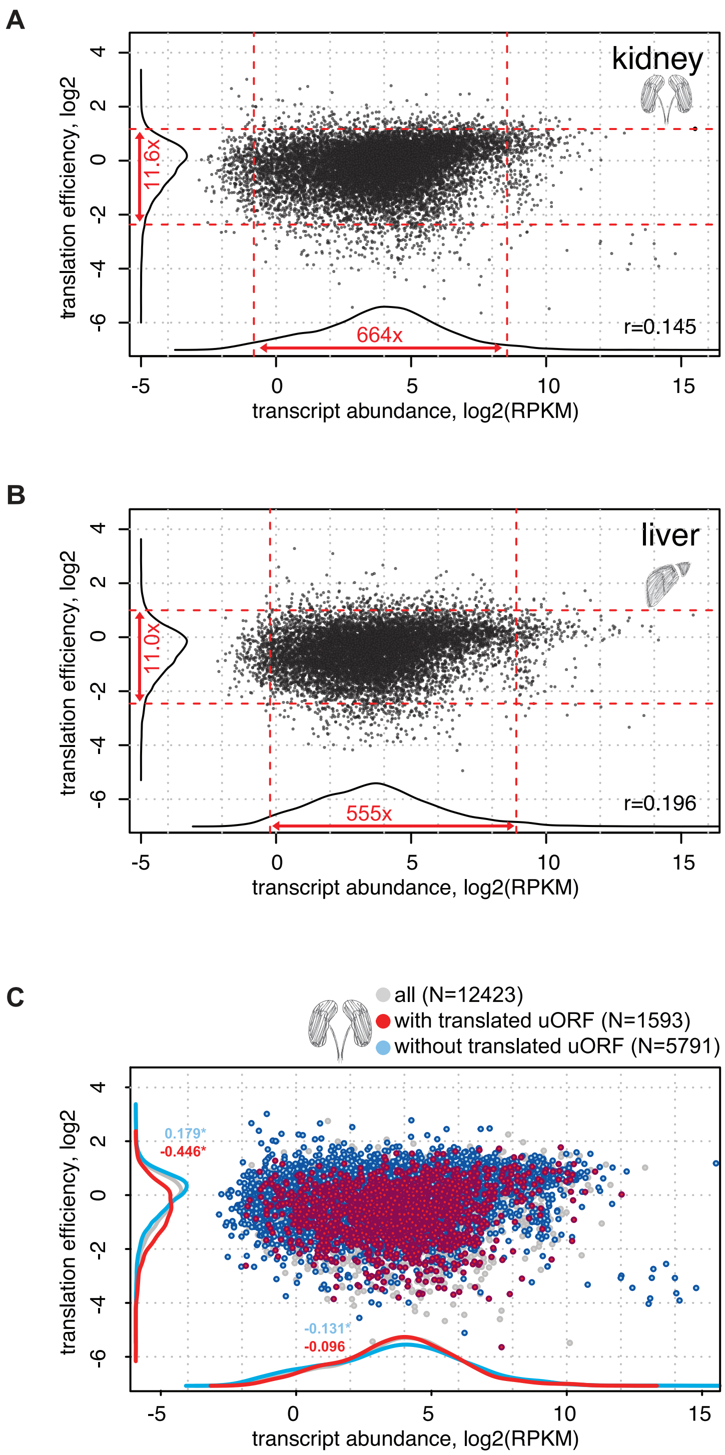
A
kidney
translation efficiency, log2
transcript abundance, log2(RPKM)
11.6x
664x
r=0.145
B
liver
translation efficiency, log2
transcript abundance, log2(RPKM)
11.0x
555x
r=0.196
C
all (N=12423)
with translated uORF (N=1593)
without translated uORF (N=5791)
0.179*
-0.446*
-0.131*
-0.096
translation efficiency, log2
transcript abundance, log2(RPKM)

Measurement errors (MEs), genes binned by expression level

based on all genes (full set, N=10289):

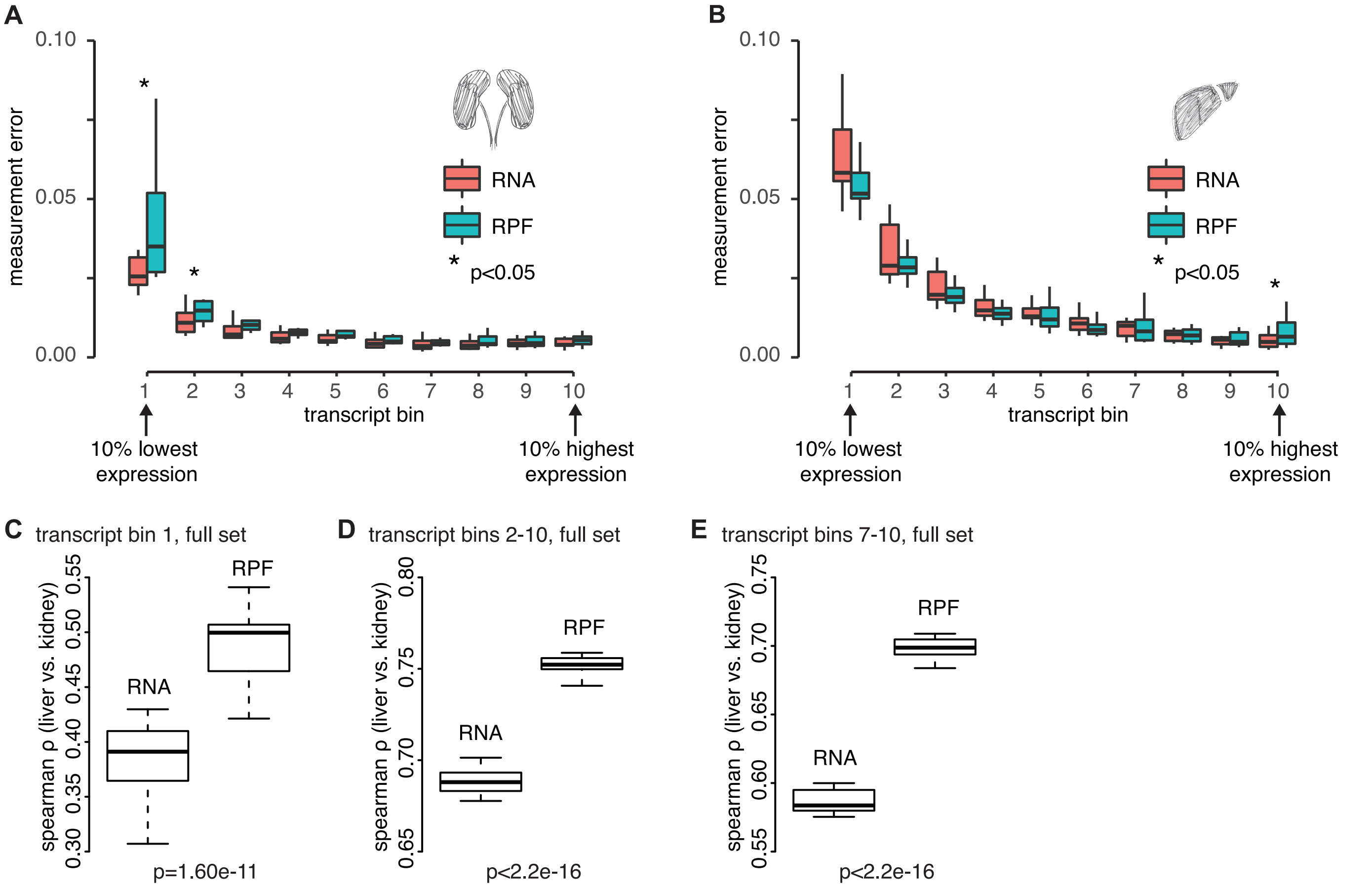

after removal of genes that were most variable in their expression (filtered set, N=9236):

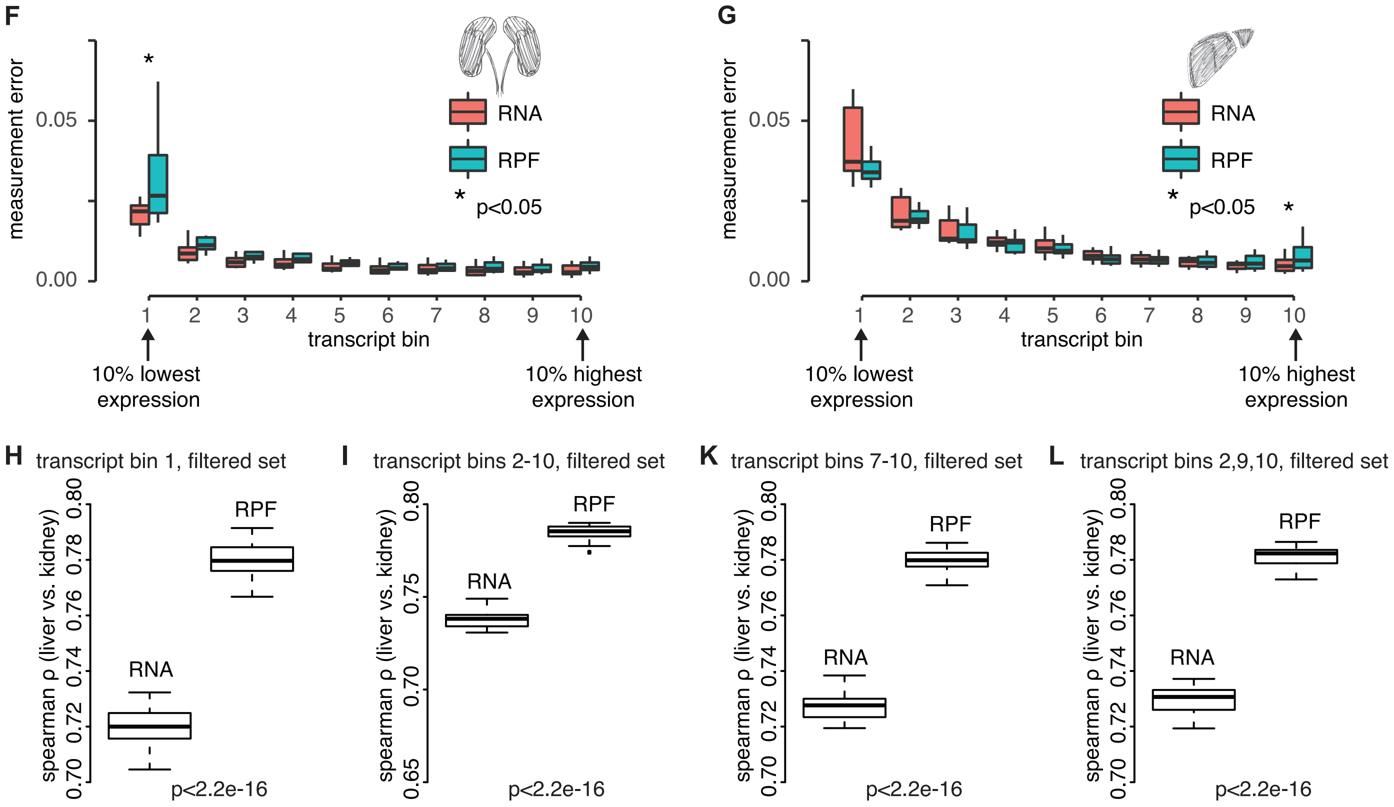

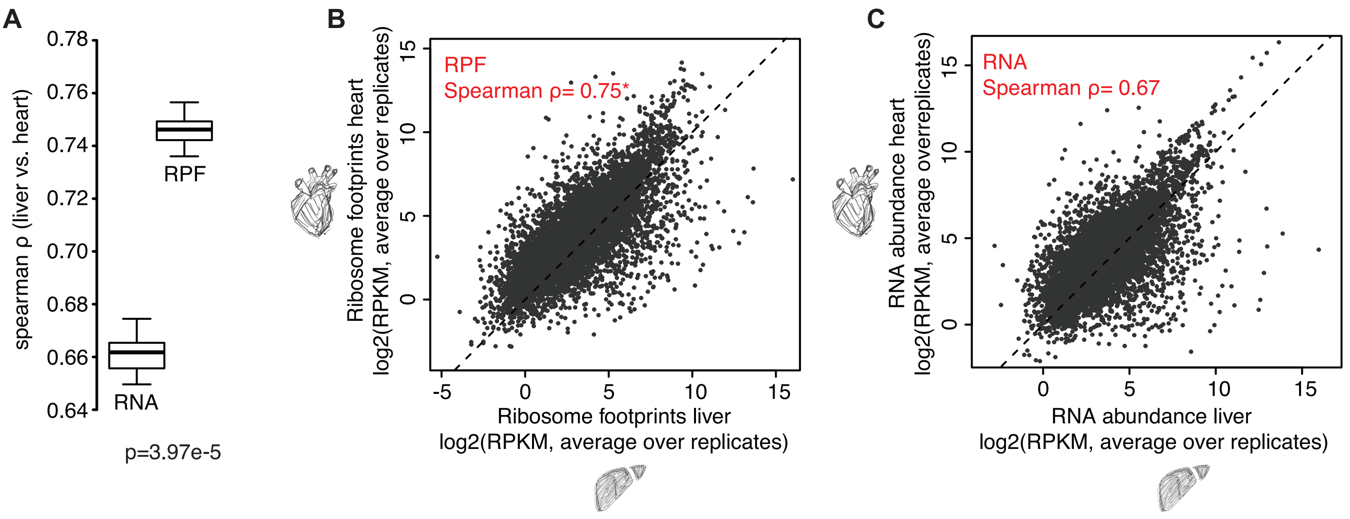

* correlations are significantly different
Steiger test for difference between 2 dependent correlations, Z value=-30.25, p=2.3e-201 (n=9325)

Steiger, J. H. (1980). Tests for comparing elements of a correlation matrix. Psychological Bulletin, 87, 245-251.

Castelo-Szekely_Supplementary FigS8

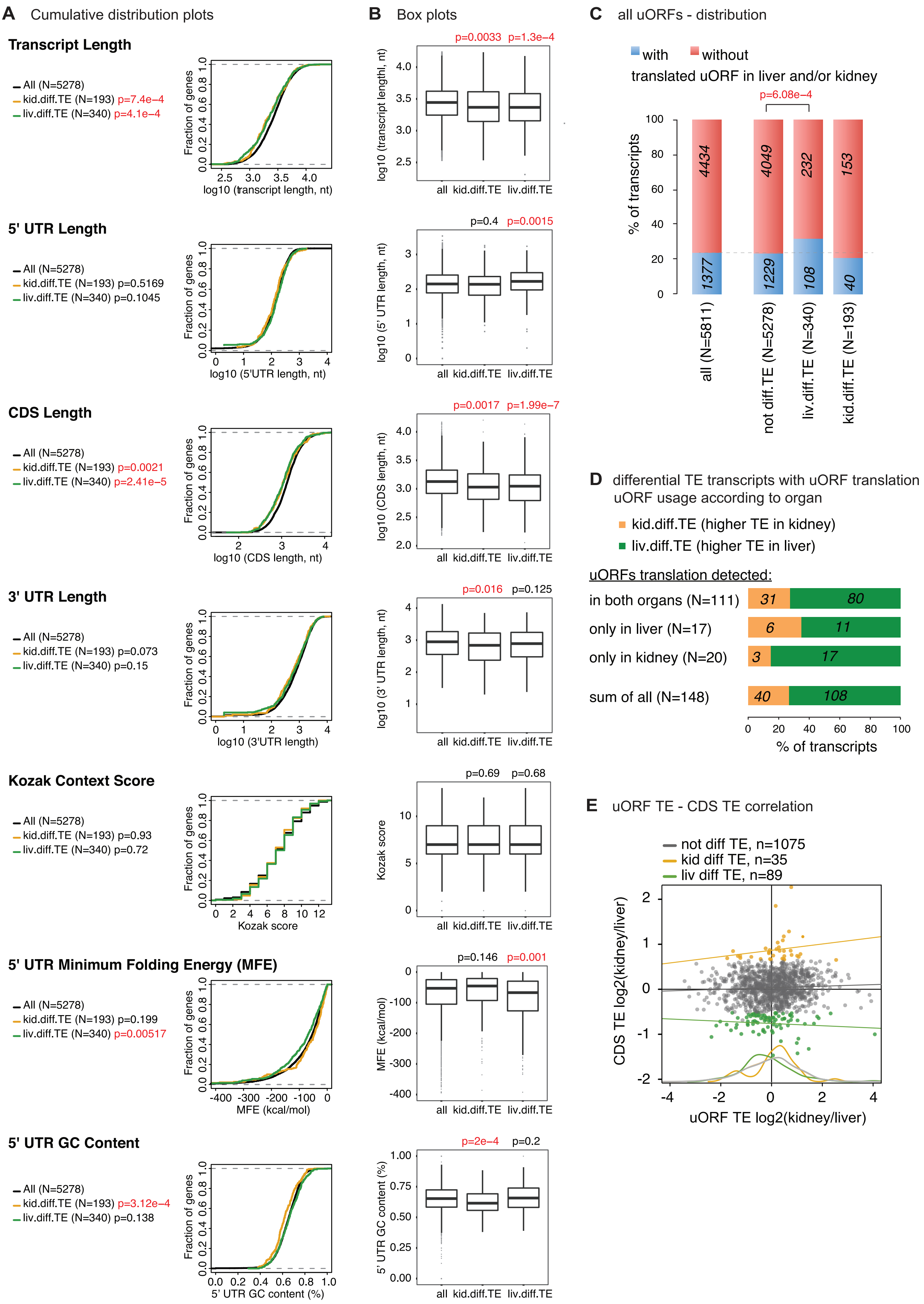

A

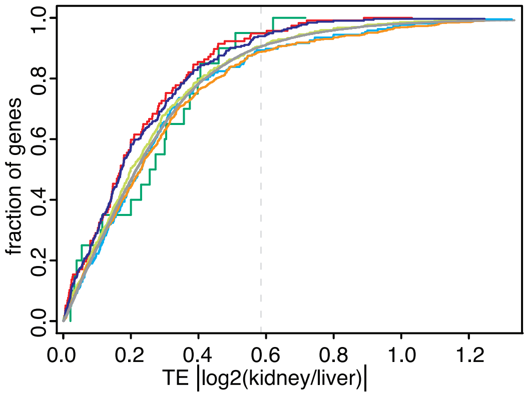

genes with transcript diversity
only in 5′ UTR (N=216), not in 5′ UTR (N=314)
only in CDS (N=117), not in CDS (N=774)
only in 3′ UTR (N=20), not in 3′ UTR (N=497)

all genes (N=10289)

Kolmogorov-Smirnov test
not in 5' UTR vs all: p=5.5e−4
only in CDS vs all: p=0.036
not in 5' UTR vs only in 5' UTR: p=0.012
only in CDS vs not in CDS: p=0.015

B

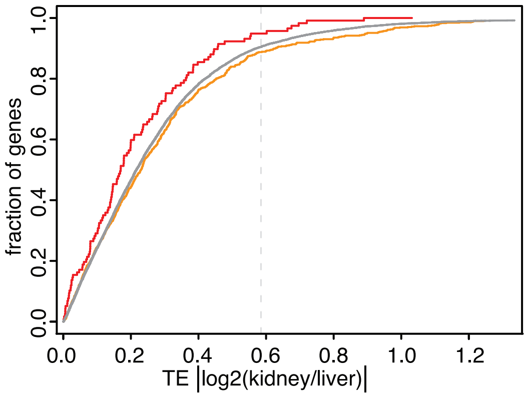

genes with transcript diversity
only in CDS (N=117), not in CDS (N=774)

all genes (N=10289)

Kolmogorov-Smirnov test
only in CDS vs not in CDS: p=0.015
only in CDS vs all: p=0.036

C

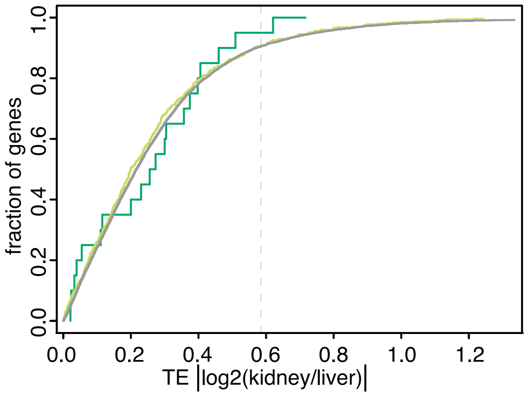

genes with transcript diversity
only in 3′ UTR (N=20), not in 3′ UTR (N=497)

all genes (N=10289)

A Peak phase difference RPF-RNA of rhythmic genes in kidney (N=542)

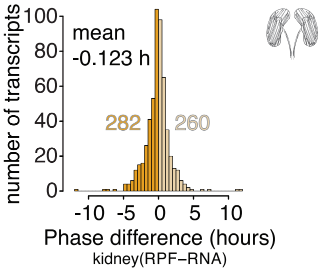

B Peak phase difference RPF-RNA of common rhythmic genes (N=178)

kidney

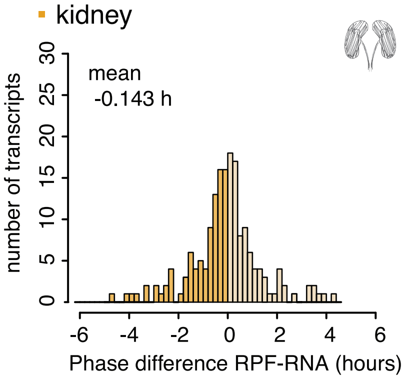

C Peak phase difference RPF-RNA of common rhythmic genes (N=178)

liver

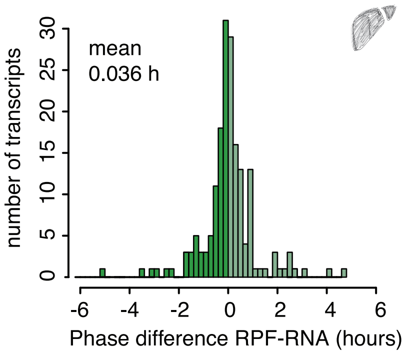

D Peak phase difference (RPF-RNA) in kidney relative to that in liver for the common rhythmic gene set (N=178)

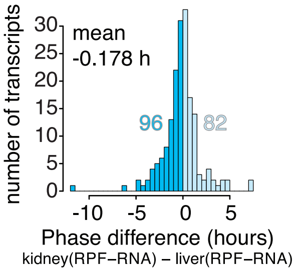

Castelo-Szekely_Supplementary FigS11

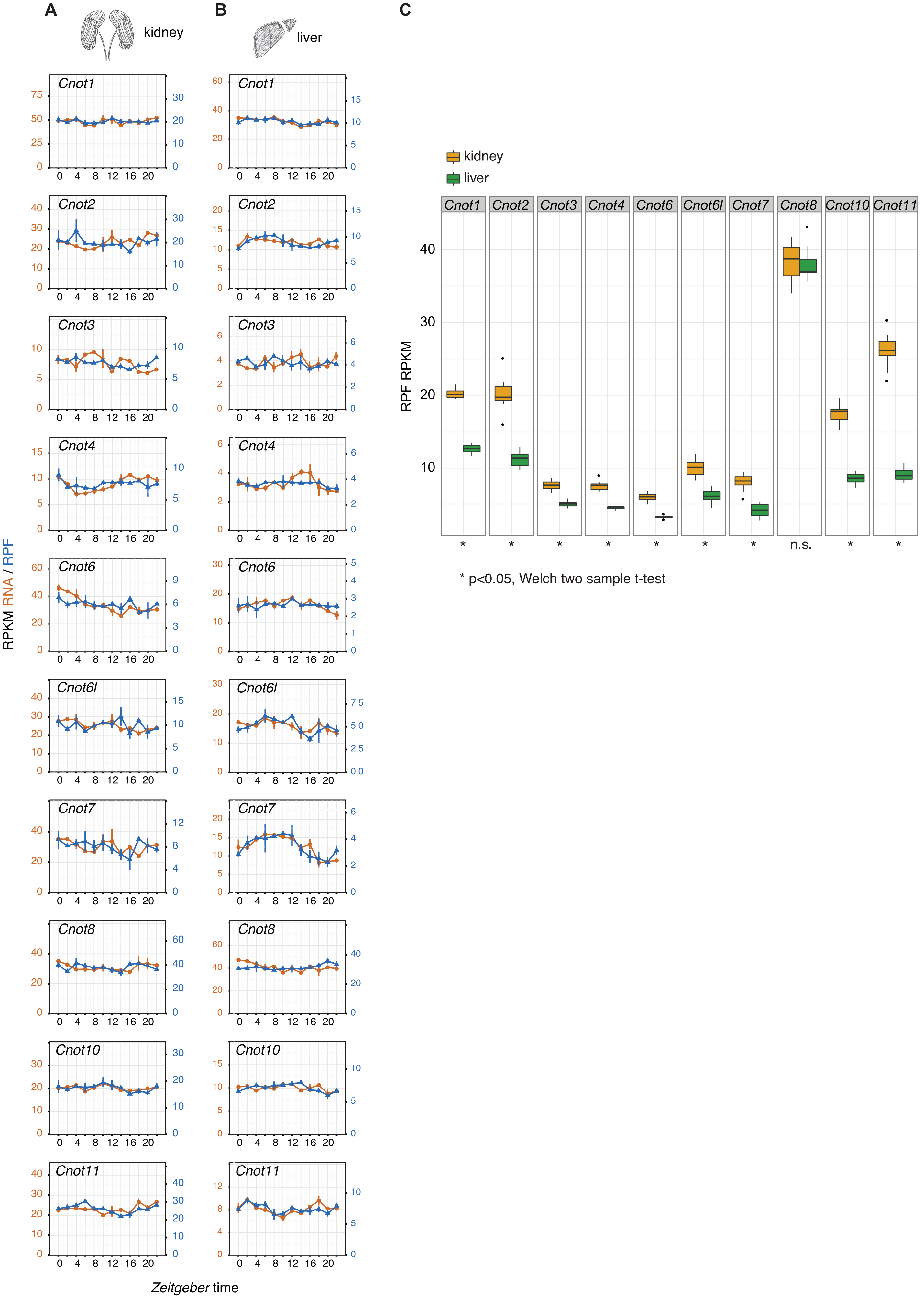

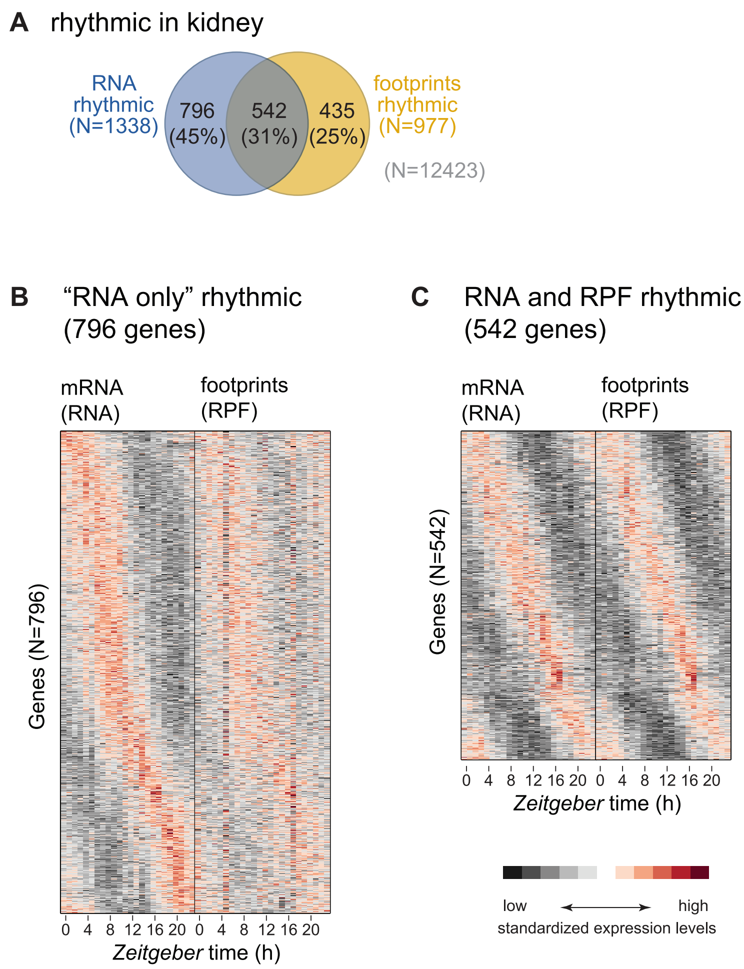
A rhythmic in kidney
RNA rhythmic (N=1338)
796 (45%)
542 (31%)
435 (25%)
footprints rhythmic (N=977)
(N=12423)
B "RNA only" rhythmic (796 genes)
mRNA (RNA)
footprints (RPF)
Genes (N=796)
0 4 8 12 16 20 0 4 8 12 16 20
Zeitgeber time (h)
C RNA and RPF rhythmic (542 genes)
mRNA (RNA)
footprints (RPF)
Genes (N=542)
0 4 8 12 16 20 0 4 8 12 16 20
Zeitgeber time (h)
low
high
standardized expression levels
D "footprints only" rhythmic (435 genes)
mRNA (RNA)
footprints (RPF)
Genes (N=435)
0 4 8 12 16 20 0 4 8 12 16 20
Zeitgeber time (h)

Castelo-Szekely_Supplementary FigS13

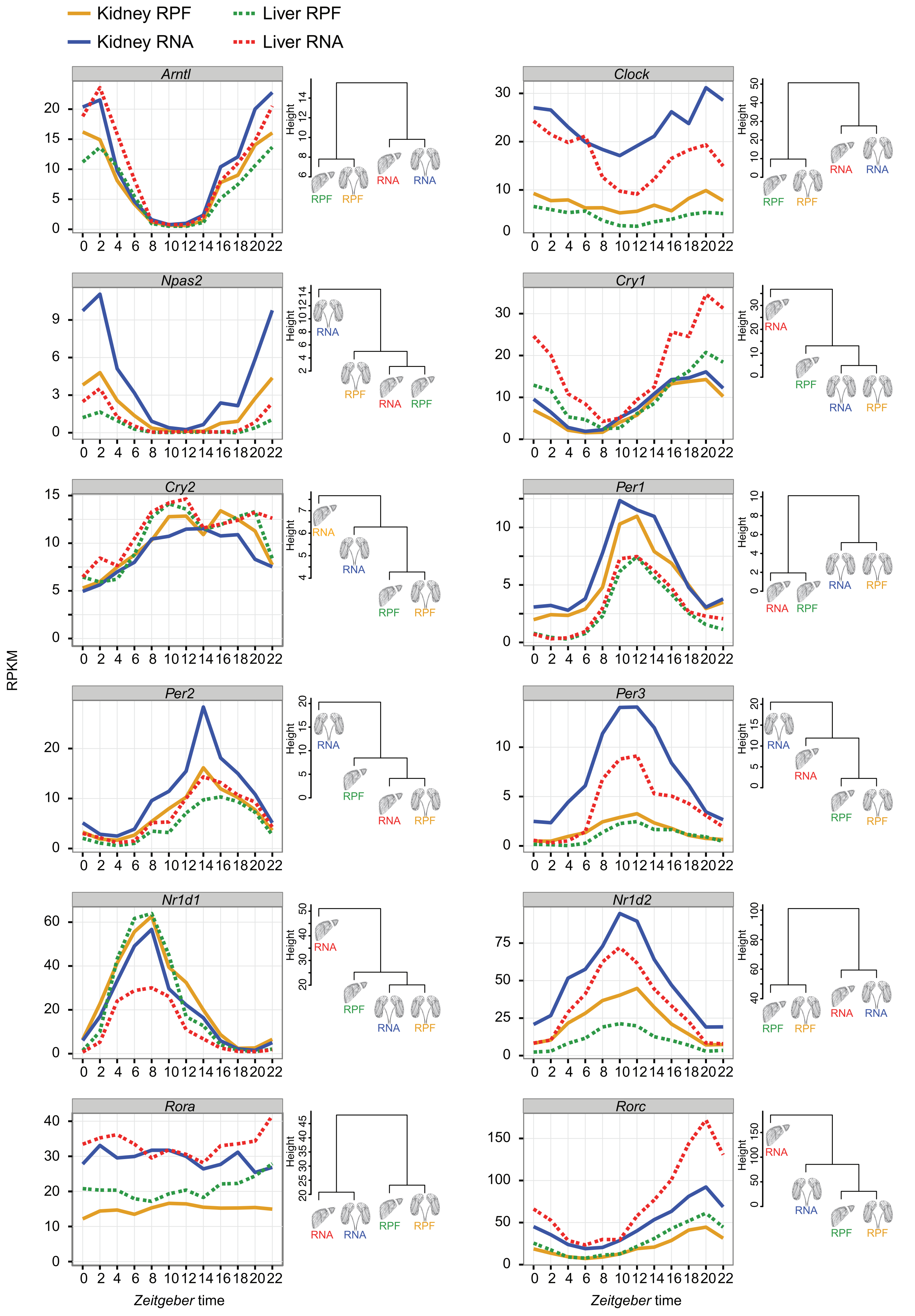

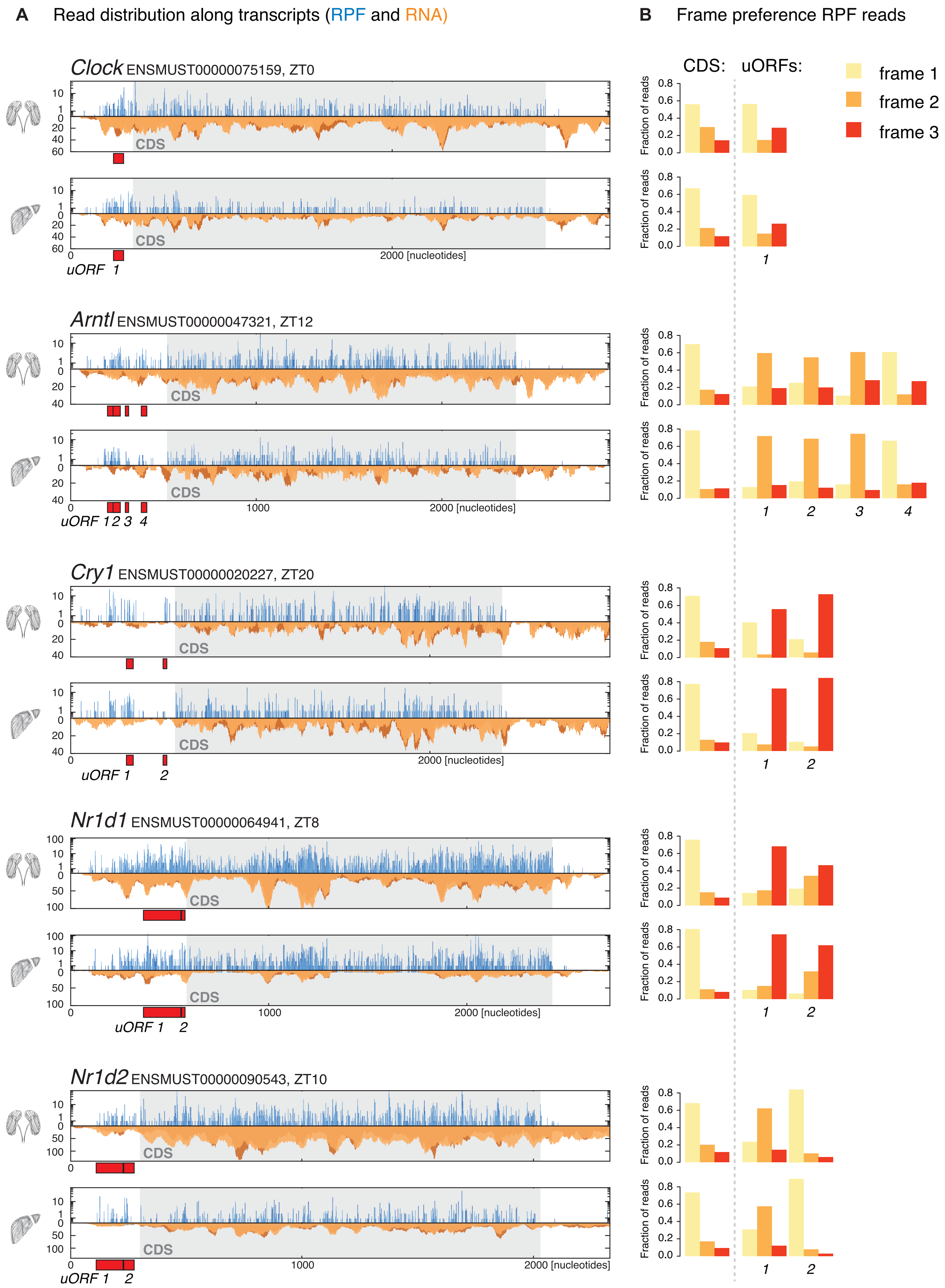
A Read distribution along transcripts (RPF and RNA)
B Frame preference RPF reads
Clock ENSMUST00000075159, ZT0
CDS:
uORFs:
frame 1
frame 2
frame 3
Fraction of reads
CDS
uORF 1
2000 [nucleotides]
Arntl ENSMUST00000047321, ZT12
uORF 1 2 3 4
1000
Cry1 ENSMUST00000020227, ZT20
uORF 1 2
Nr1d1 ENSMUST00000064941, ZT8
Nr1d2 ENSMUST00000090543, ZT10
