# Supplementary figures and images for: Translational contributions to tissue specificity in rhythmic and constitutive gene expression (part 2 of 4)
Source: Genome Biol. 2017 Jun 16;18:116. doi: 10.1186/s13059-017-1222-2 (PMC5473967; doi:10.1186/s13059-017-1222-2)

## 2510002D24Rik

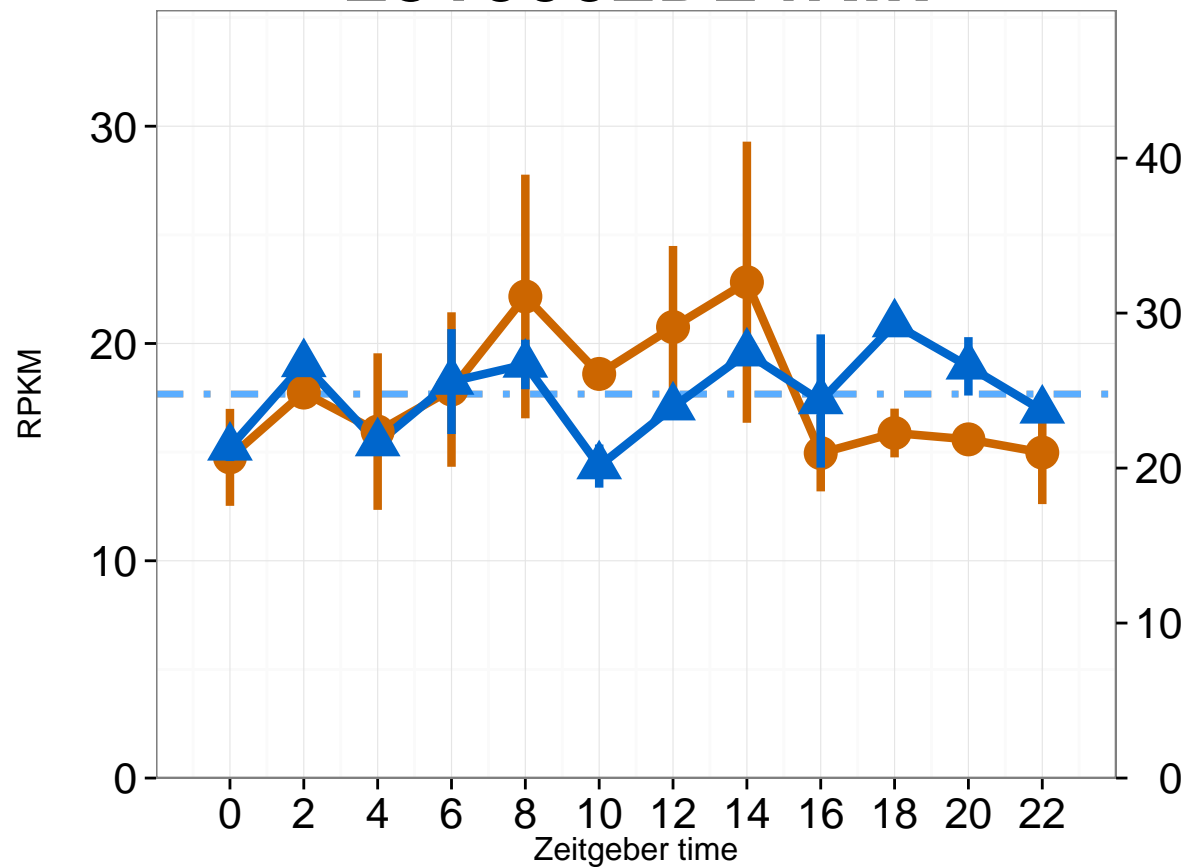

## 2510002D24Rik

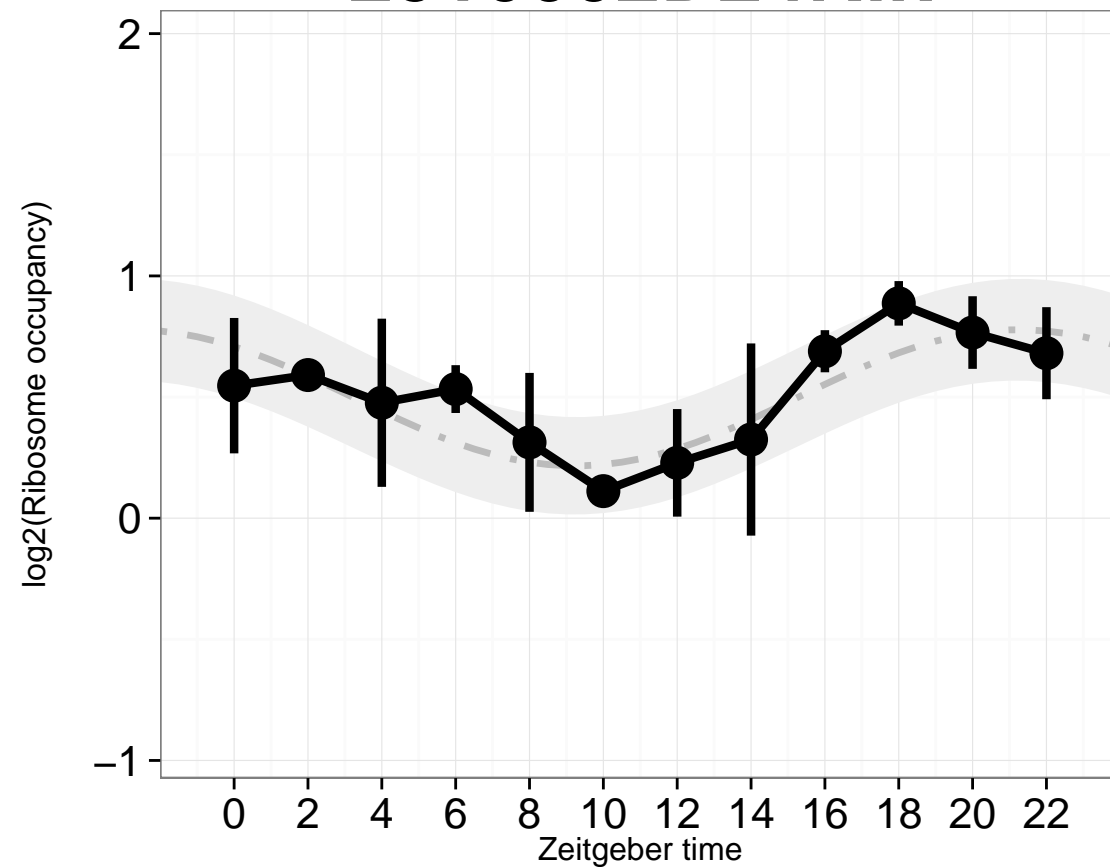

Supplement: Supplementary file 6 — Transcriptome-wide kidney RPF (blue) and RNA (orange) levels in the left panels (with “error bars” connecting the two replicates of each timepoint) and TE in the right panels. (ZIP 116896 kb) [file 13059_2017_1222_MOESM6_ESM.zip › Supp_Dataset_S1/A_RNA_non_rhythmic_RPF_non_rhythmic/2510002D24Rik_kidney_set_A.pdf]

## 2510003E04Rik

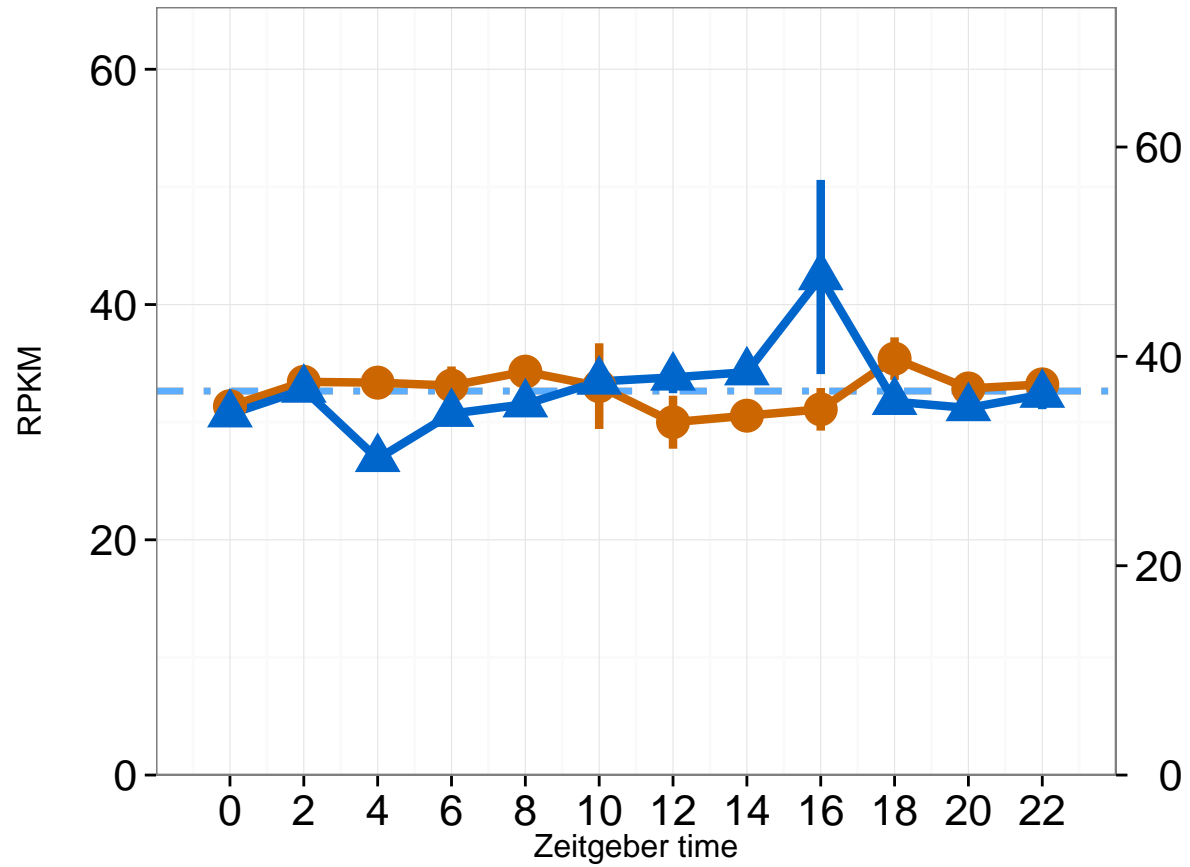

## 2510003E04Rik

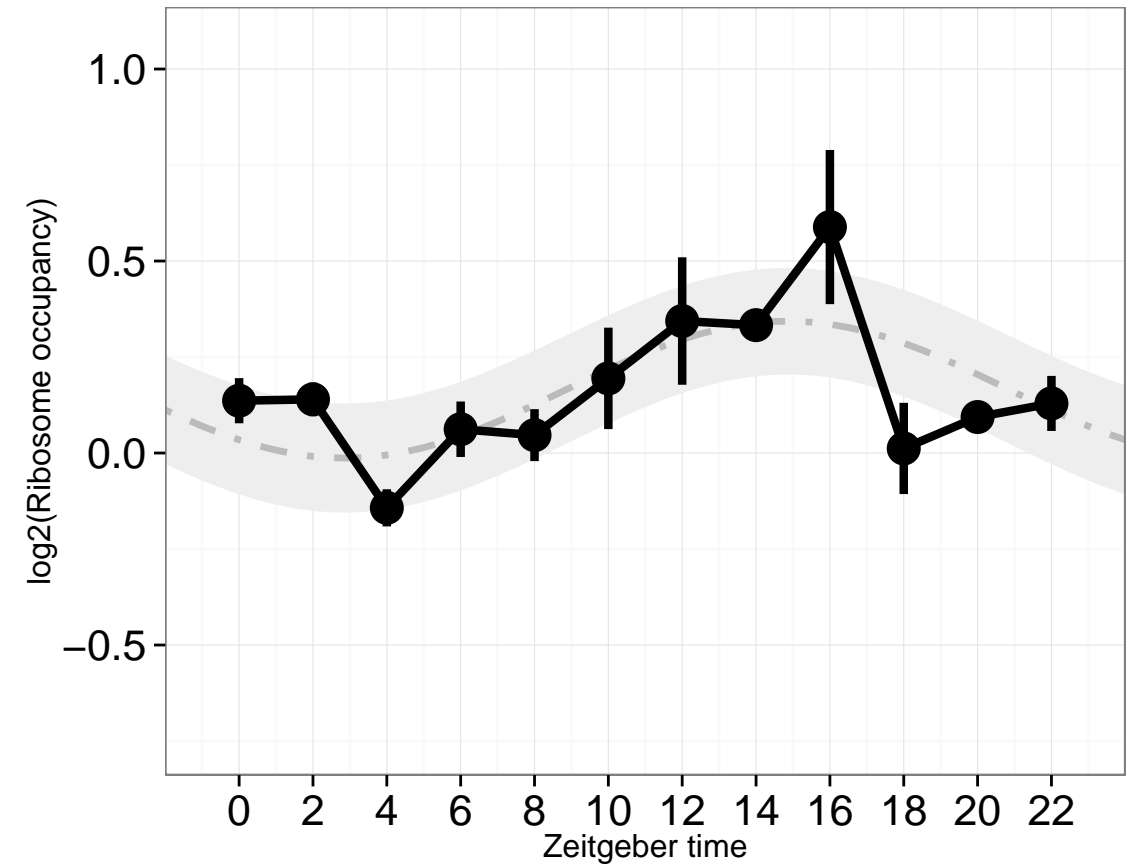

Supplement: Supplementary file 6 — Transcriptome-wide kidney RPF (blue) and RNA (orange) levels in the left panels (with “error bars” connecting the two replicates of each timepoint) and TE in the right panels. (ZIP 116896 kb) [file 13059_2017_1222_MOESM6_ESM.zip › Supp_Dataset_S1/A_RNA_non_rhythmic_RPF_non_rhythmic/2510003E04Rik_kidney_set_A.pdf]

## 2510009E07Rik

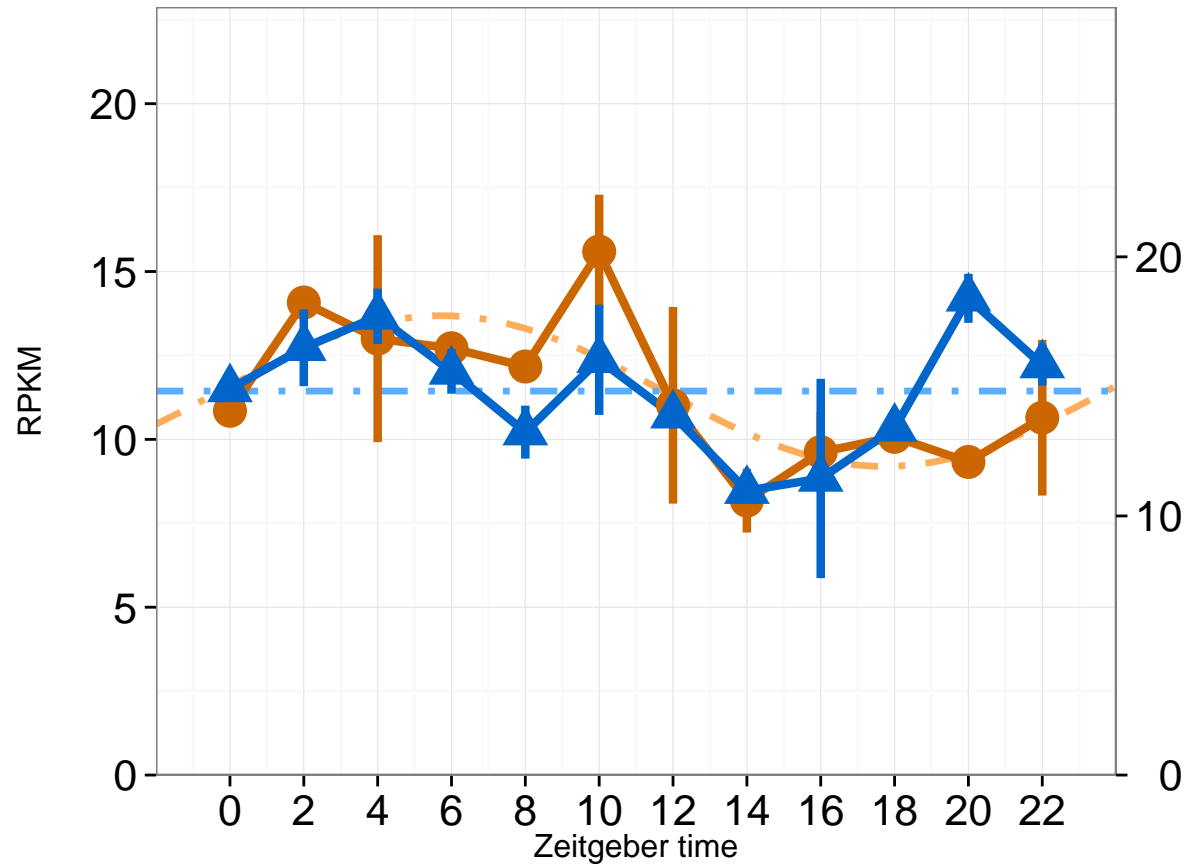

## 2510009E07Rik

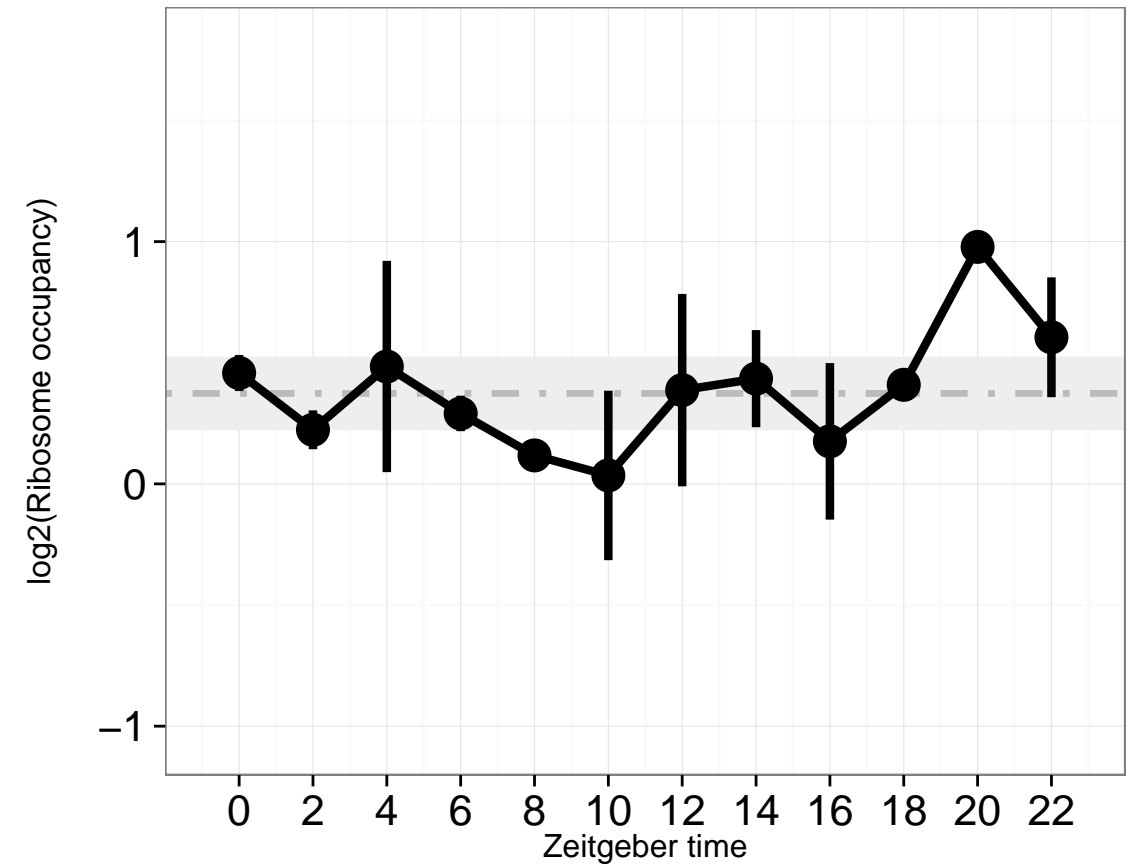

Supplement: Supplementary file 6 — Transcriptome-wide kidney RPF (blue) and RNA (orange) levels in the left panels (with “error bars” connecting the two replicates of each timepoint) and TE in the right panels. (ZIP 116896 kb) [file 13059_2017_1222_MOESM6_ESM.zip › Supp_Dataset_S1/A_RNA_non_rhythmic_RPF_non_rhythmic/2510009E07Rik_kidney_set_A.pdf]

## 2510039O18Rik

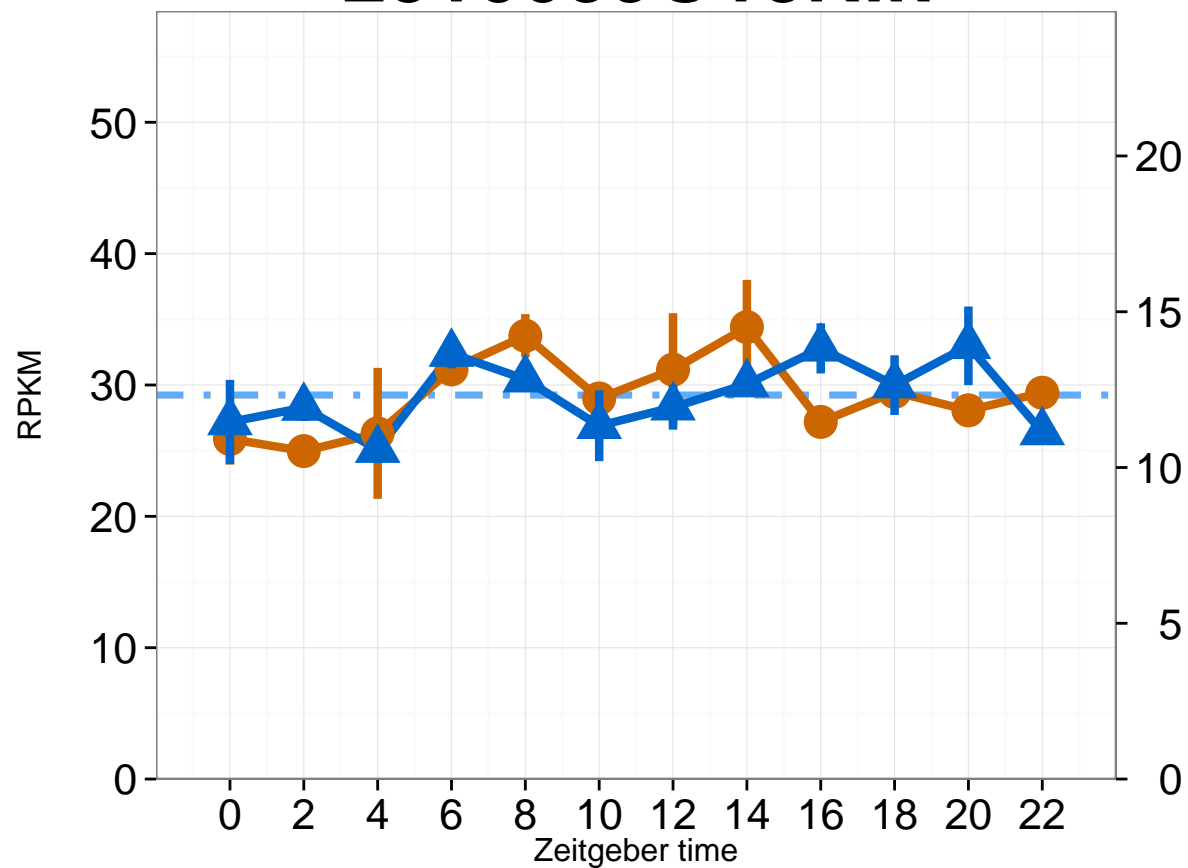

## 2510039O18Rik

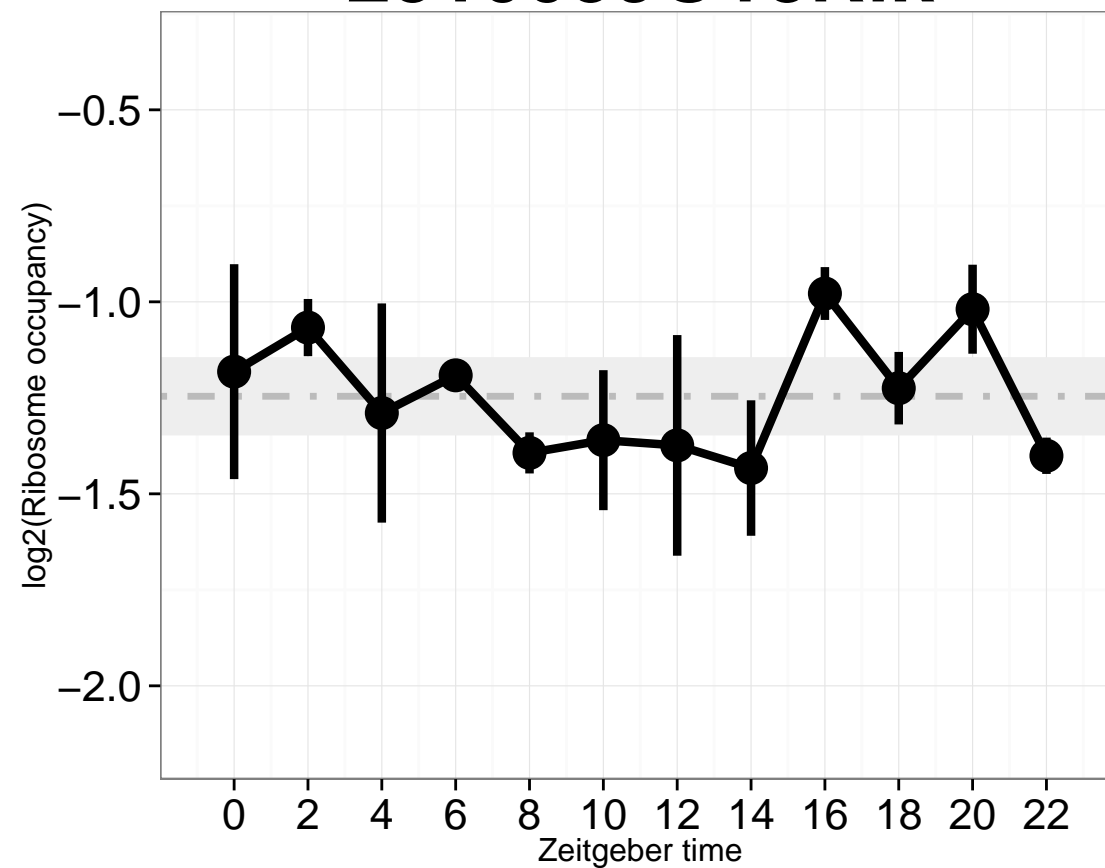

Supplement: Supplementary file 6 — Transcriptome-wide kidney RPF (blue) and RNA (orange) levels in the left panels (with “error bars” connecting the two replicates of each timepoint) and TE in the right panels. (ZIP 116896 kb) [file 13059_2017_1222_MOESM6_ESM.zip › Supp_Dataset_S1/A_RNA_non_rhythmic_RPF_non_rhythmic/2510039O18Rik_kidney_set_A.pdf]

## 2510046G10Rik

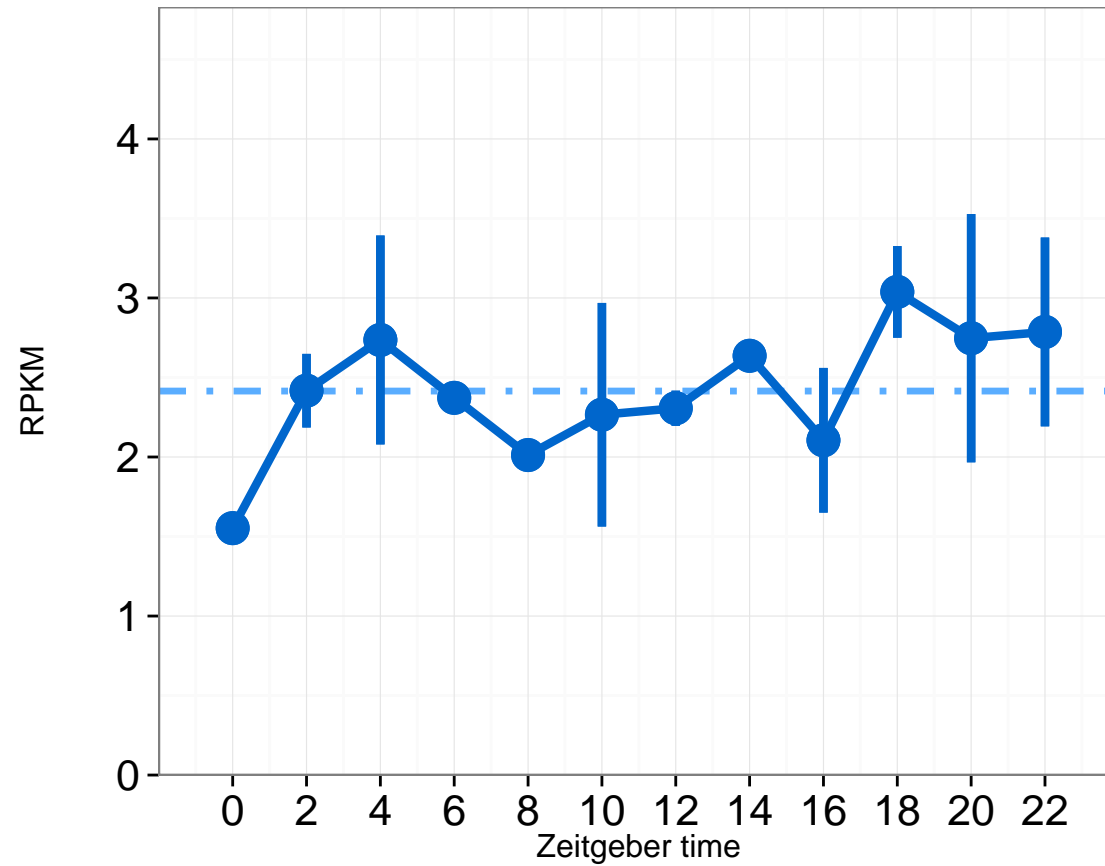

## 2510046G10Rik log2(Ribosome occup

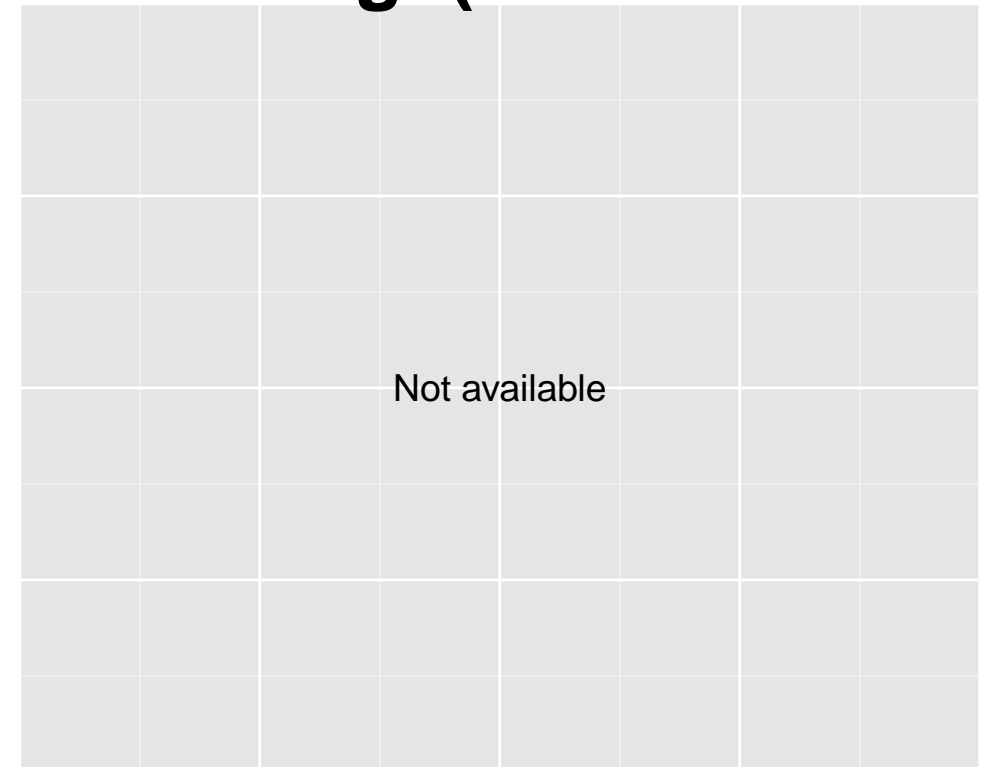

Supplement: Supplementary file 6 — Transcriptome-wide kidney RPF (blue) and RNA (orange) levels in the left panels (with “error bars” connecting the two replicates of each timepoint) and TE in the right panels. (ZIP 116896 kb) [file 13059_2017_1222_MOESM6_ESM.zip › Supp_Dataset_S1/A_RNA_non_rhythmic_RPF_non_rhythmic/2510046G10Rik_kidney_set_A.pdf]

# 2510049J12Rik

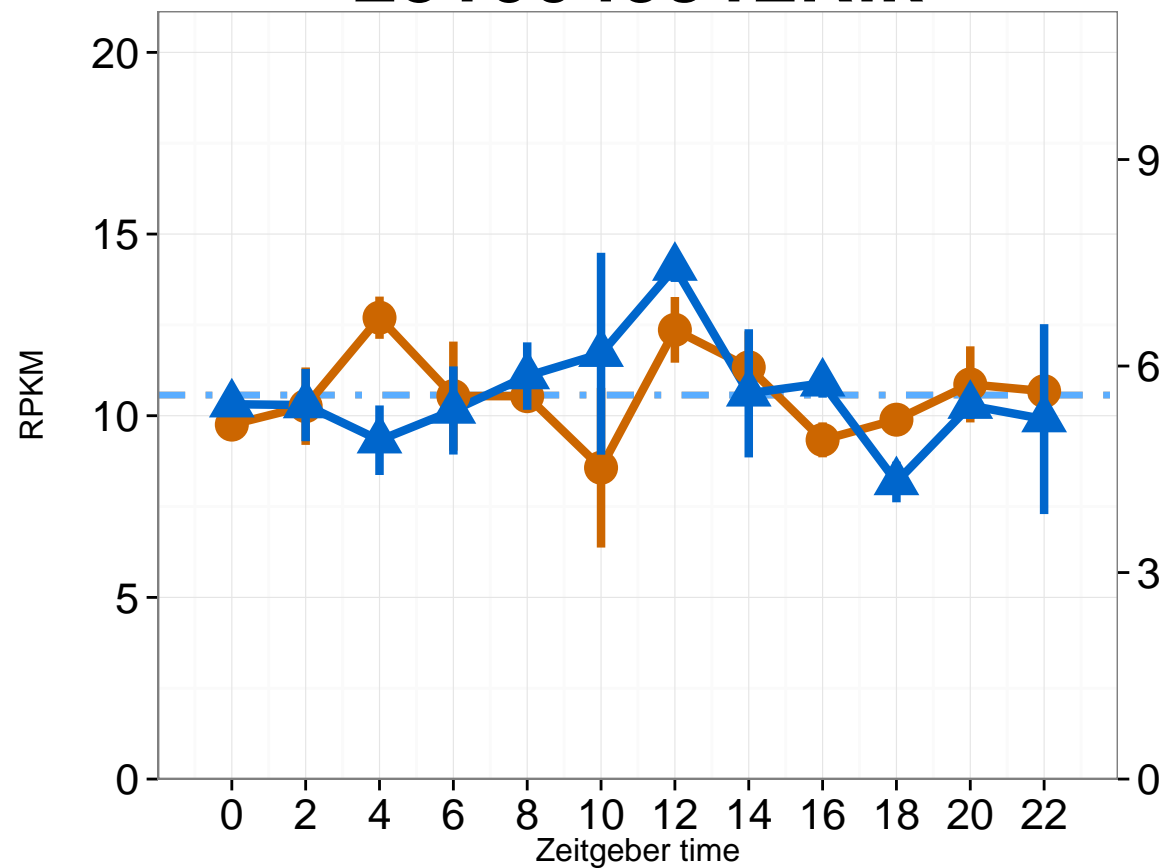

# 2510049J12Rik

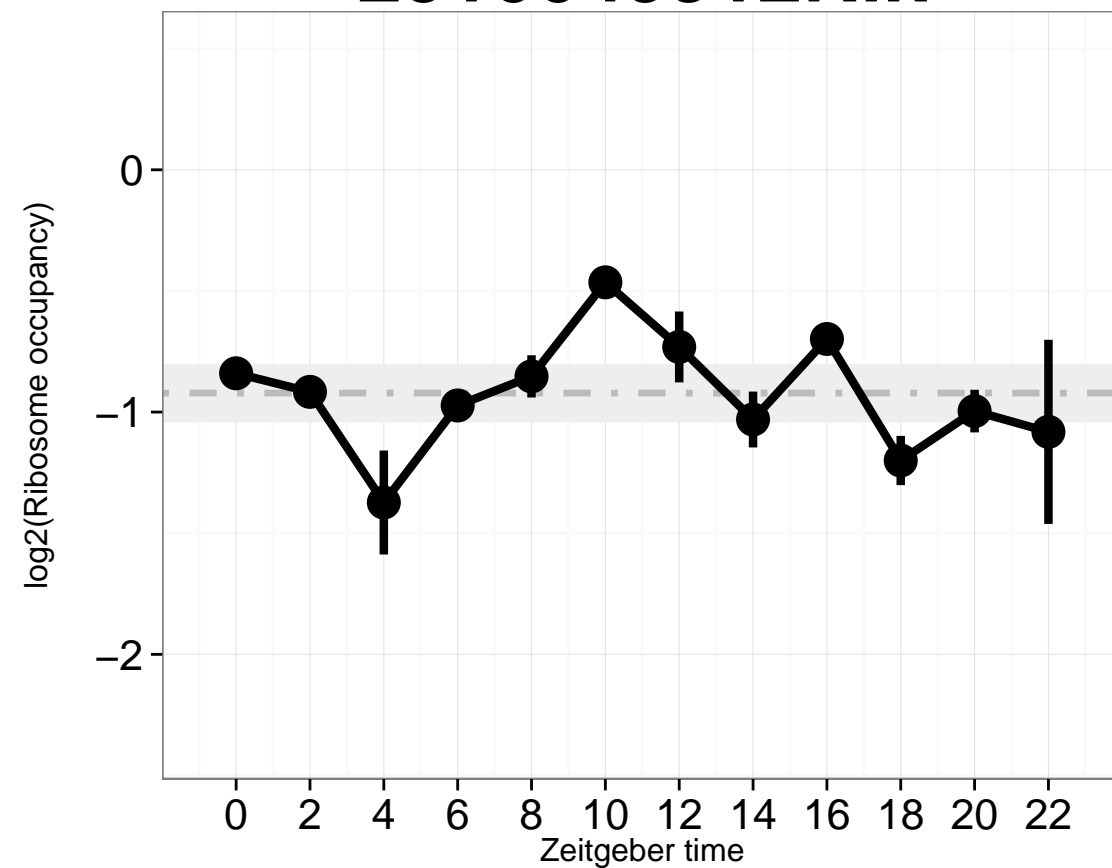

Supplement: Supplementary file 6 — Transcriptome-wide kidney RPF (blue) and RNA (orange) levels in the left panels (with “error bars” connecting the two replicates of each timepoint) and TE in the right panels. (ZIP 116896 kb) [file 13059_2017_1222_MOESM6_ESM.zip › Supp_Dataset_S1/A_RNA_non_rhythmic_RPF_non_rhythmic/2510049J12Rik_kidney_set_A.pdf]

## 2610002M06Rik

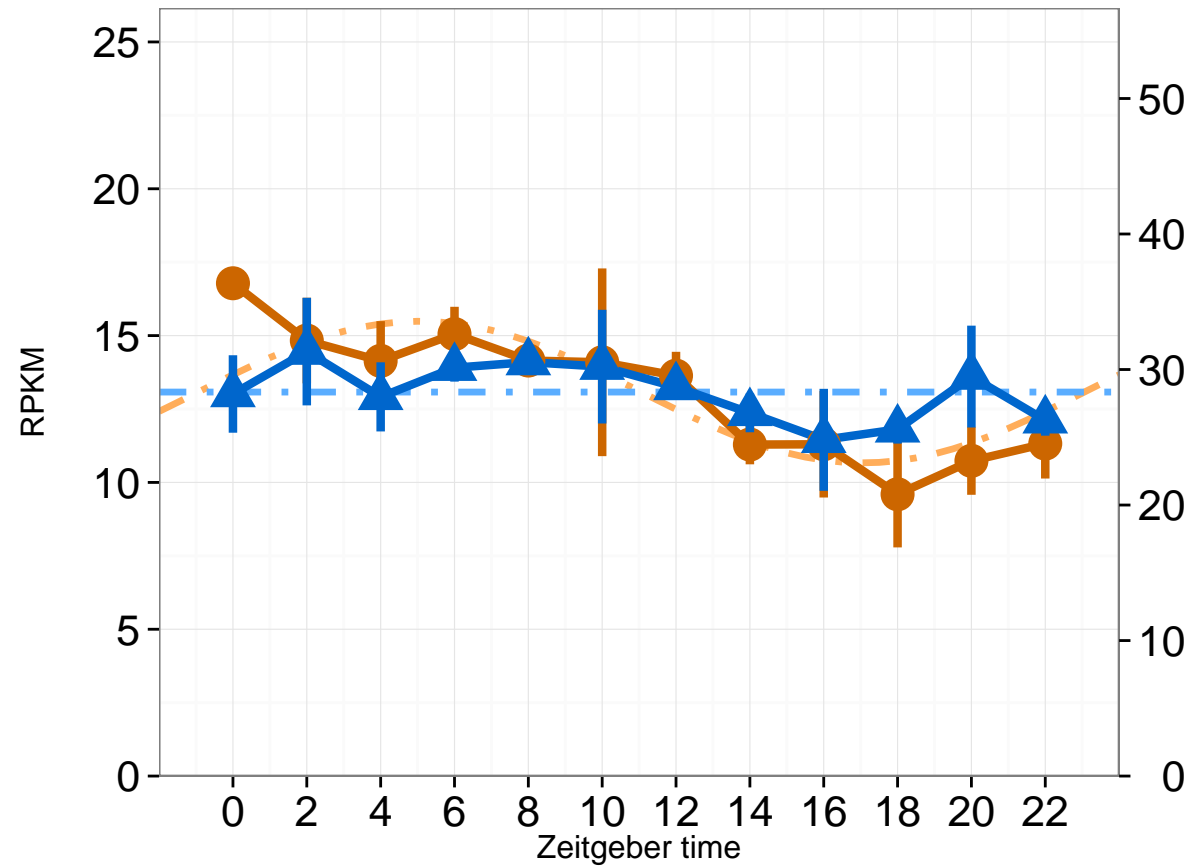

## 2610002M06Rik

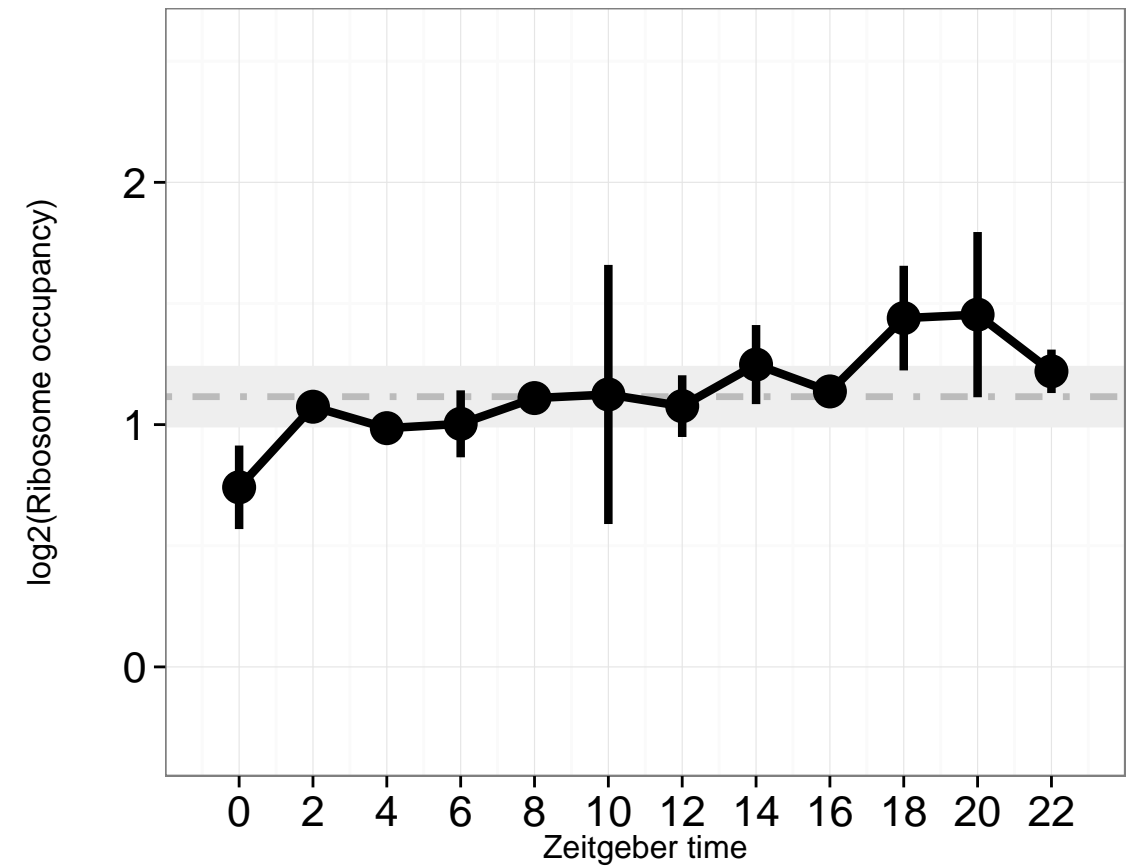

Supplement: Supplementary file 6 — Transcriptome-wide kidney RPF (blue) and RNA (orange) levels in the left panels (with “error bars” connecting the two replicates of each timepoint) and TE in the right panels. (ZIP 116896 kb) [file 13059_2017_1222_MOESM6_ESM.zip › Supp_Dataset_S1/A_RNA_non_rhythmic_RPF_non_rhythmic/2610002M06Rik_kidney_set_A.pdf]

## 2610008E11Rik

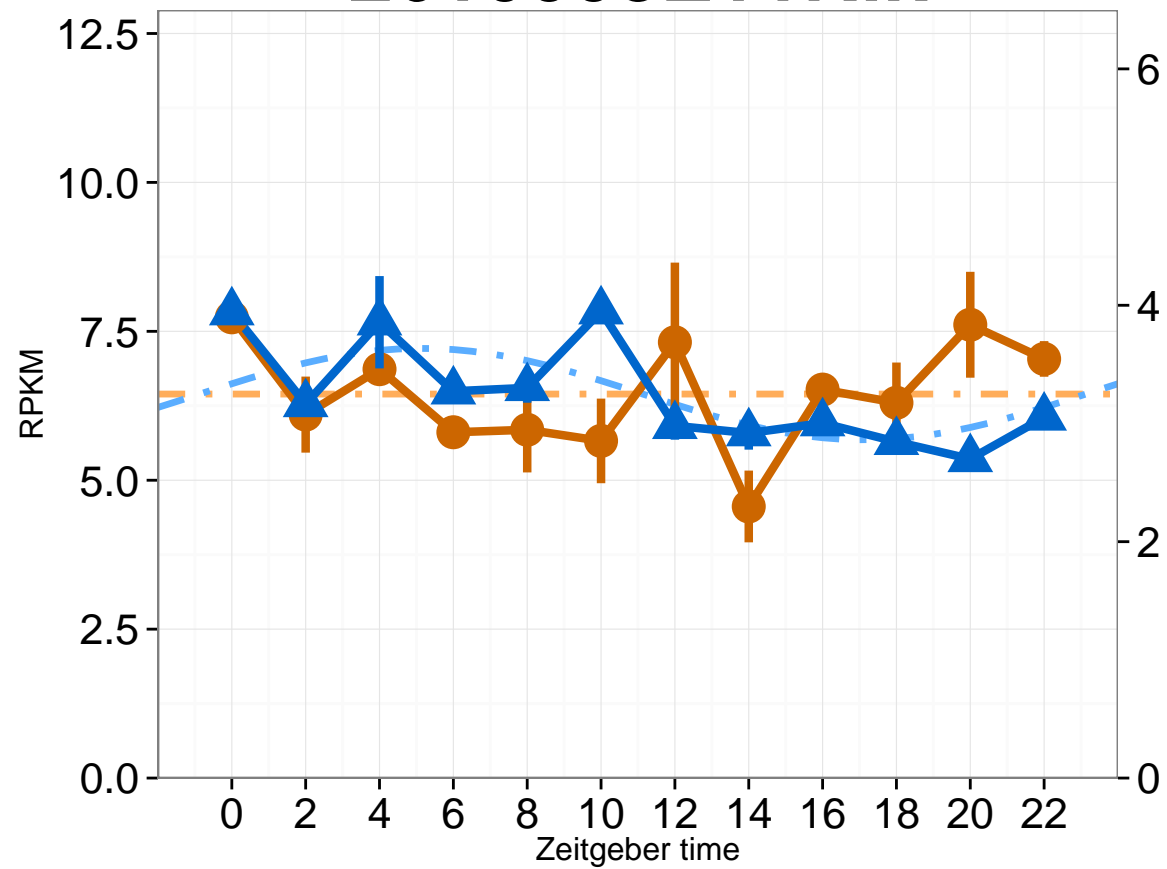

## 2610008E11Rik

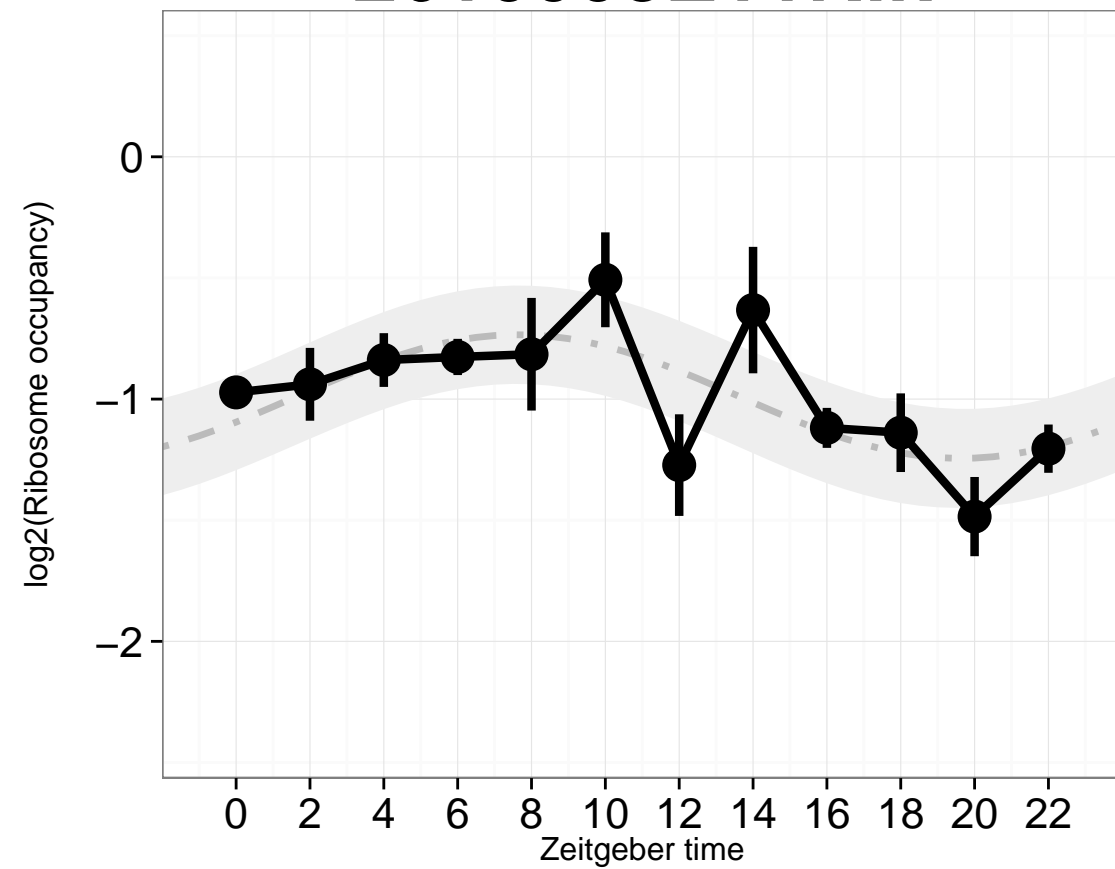

Supplement: Supplementary file 6 — Transcriptome-wide kidney RPF (blue) and RNA (orange) levels in the left panels (with “error bars” connecting the two replicates of each timepoint) and TE in the right panels. (ZIP 116896 kb) [file 13059_2017_1222_MOESM6_ESM.zip › Supp_Dataset_S1/A_RNA_non_rhythmic_RPF_non_rhythmic/2610008E11Rik_kidney_set_A.pdf]

# 2610015P09Rik

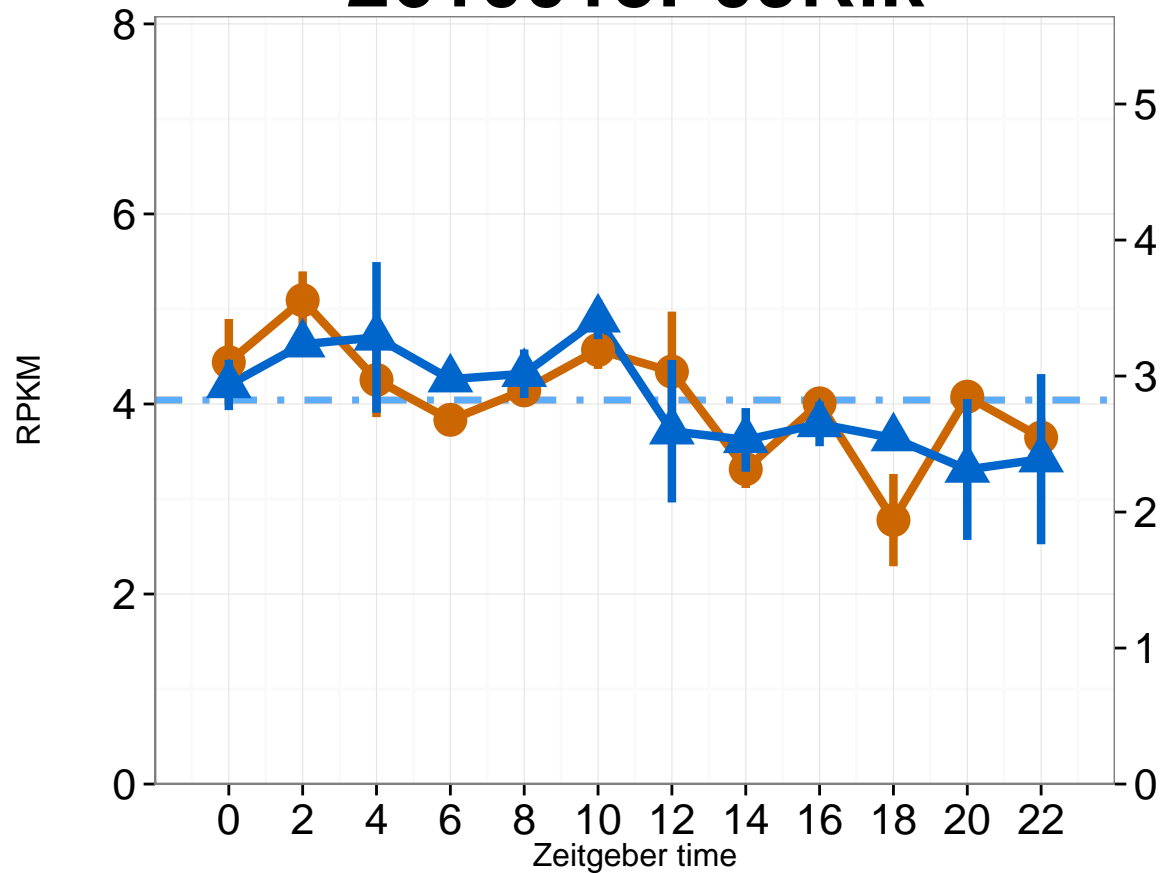

# 2610015P09Rik

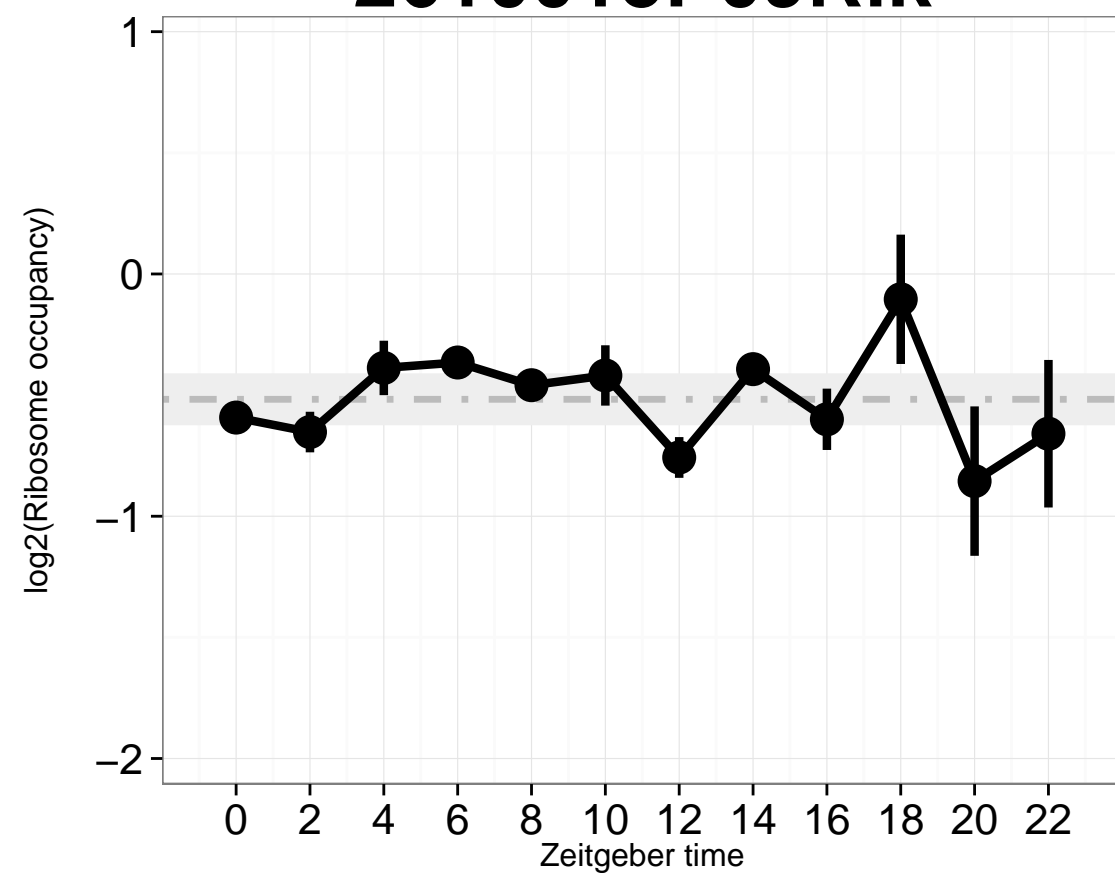

Supplement: Supplementary file 6 — Transcriptome-wide kidney RPF (blue) and RNA (orange) levels in the left panels (with “error bars” connecting the two replicates of each timepoint) and TE in the right panels. (ZIP 116896 kb) [file 13059_2017_1222_MOESM6_ESM.zip › Supp_Dataset_S1/A_RNA_non_rhythmic_RPF_non_rhythmic/2610015P09Rik_kidney_set_A.pdf]

## 2610018G03Rik

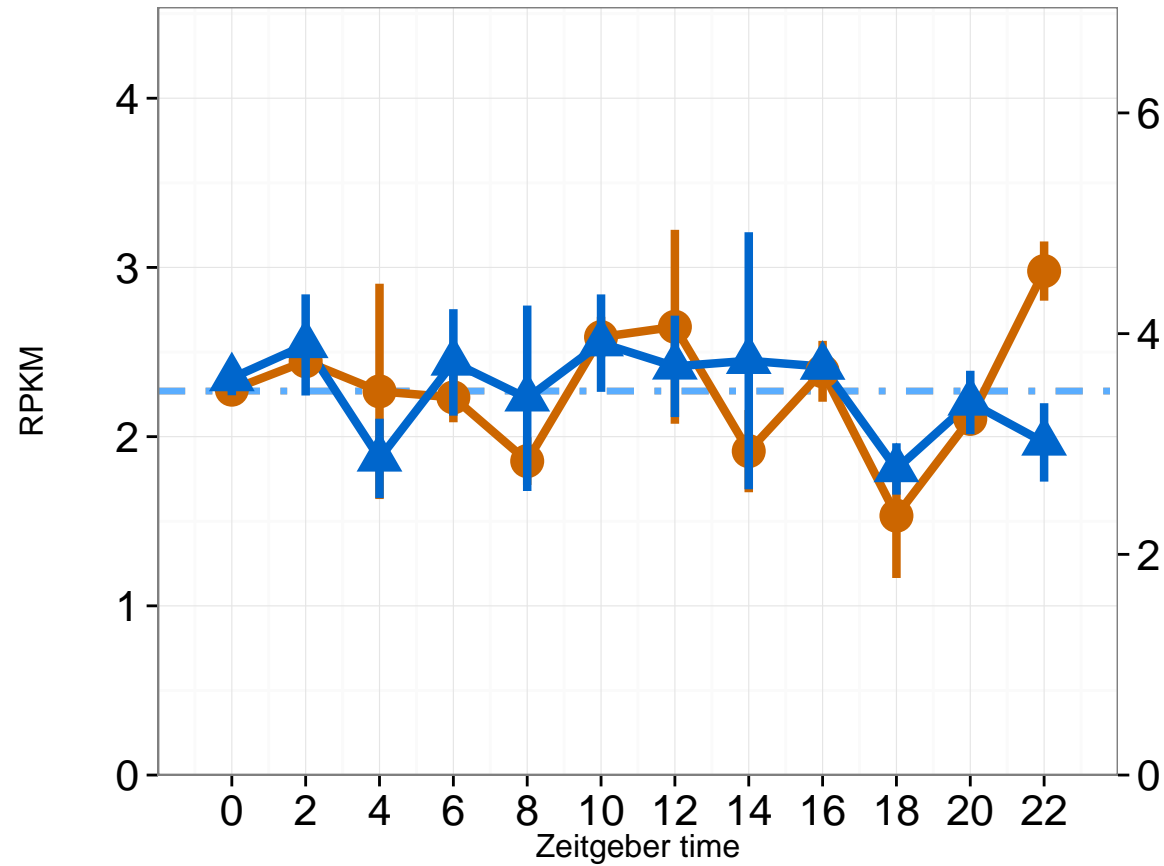

## 2610018G03Rik

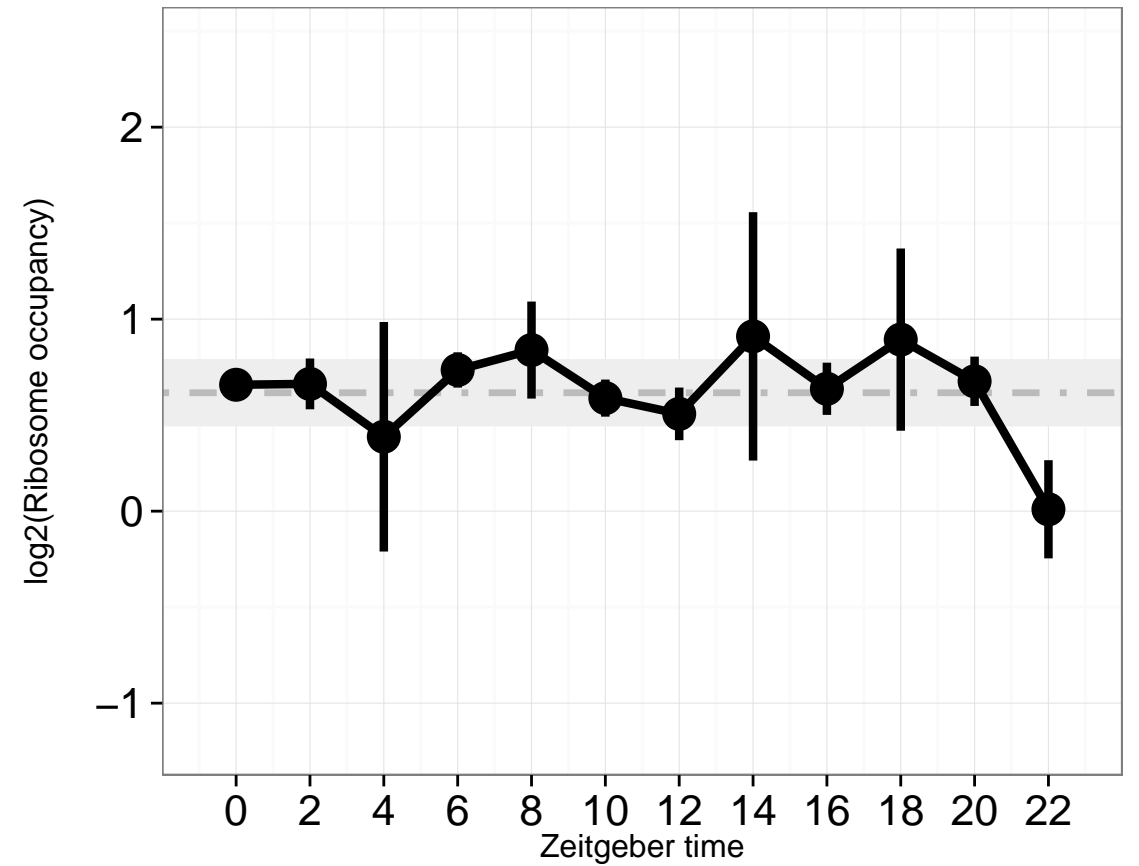

Supplement: Supplementary file 6 — Transcriptome-wide kidney RPF (blue) and RNA (orange) levels in the left panels (with “error bars” connecting the two replicates of each timepoint) and TE in the right panels. (ZIP 116896 kb) [file 13059_2017_1222_MOESM6_ESM.zip › Supp_Dataset_S1/A_RNA_non_rhythmic_RPF_non_rhythmic/2610018G03Rik_kidney_set_A.pdf]

# 2610020H08Rik

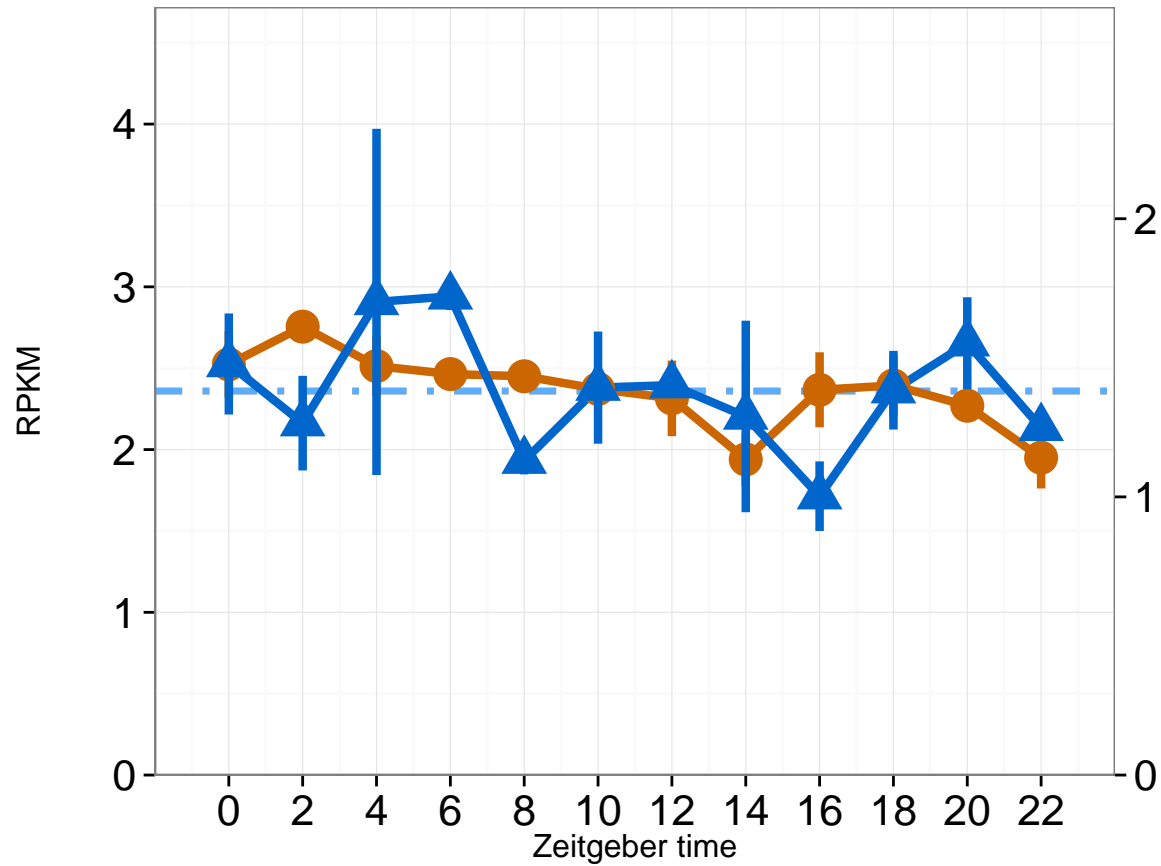

# 2610020H08Rik

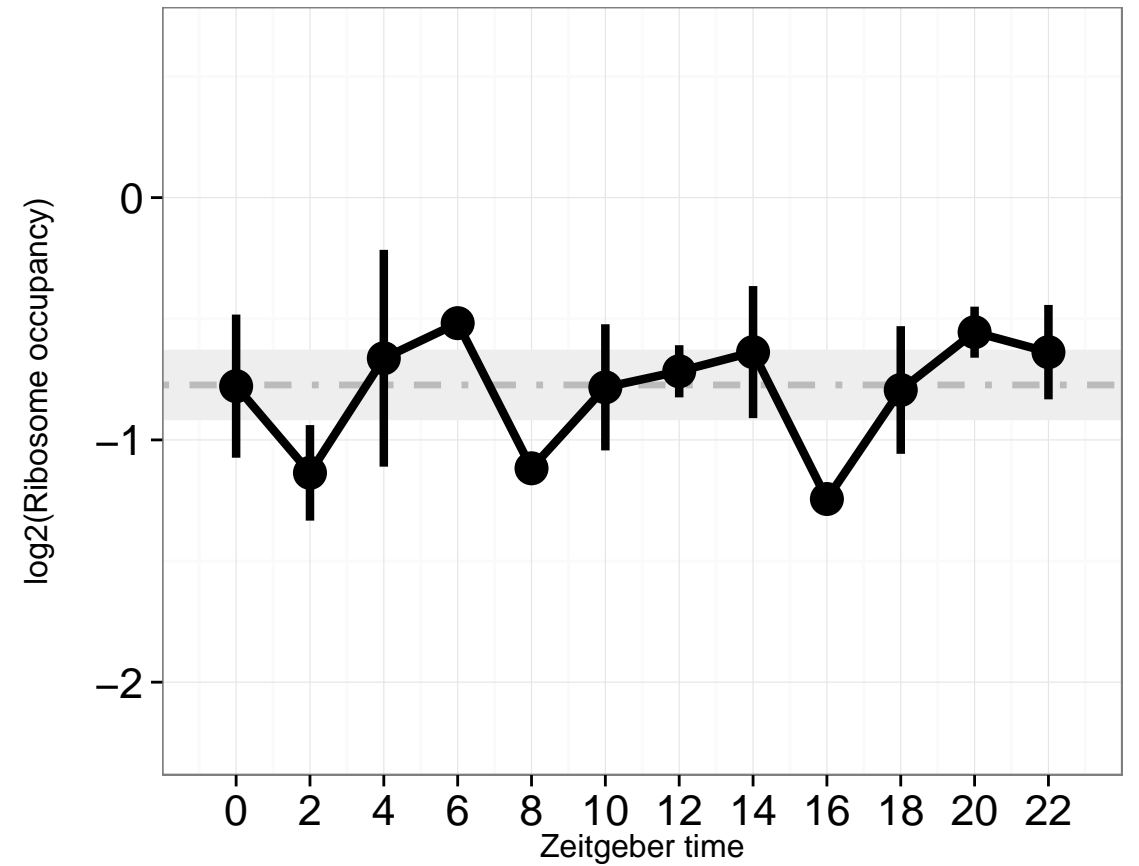

Supplement: Supplementary file 6 — Transcriptome-wide kidney RPF (blue) and RNA (orange) levels in the left panels (with “error bars” connecting the two replicates of each timepoint) and TE in the right panels. (ZIP 116896 kb) [file 13059_2017_1222_MOESM6_ESM.zip › Supp_Dataset_S1/A_RNA_non_rhythmic_RPF_non_rhythmic/2610020H08Rik_kidney_set_A.pdf]

# 2610034B18Rik

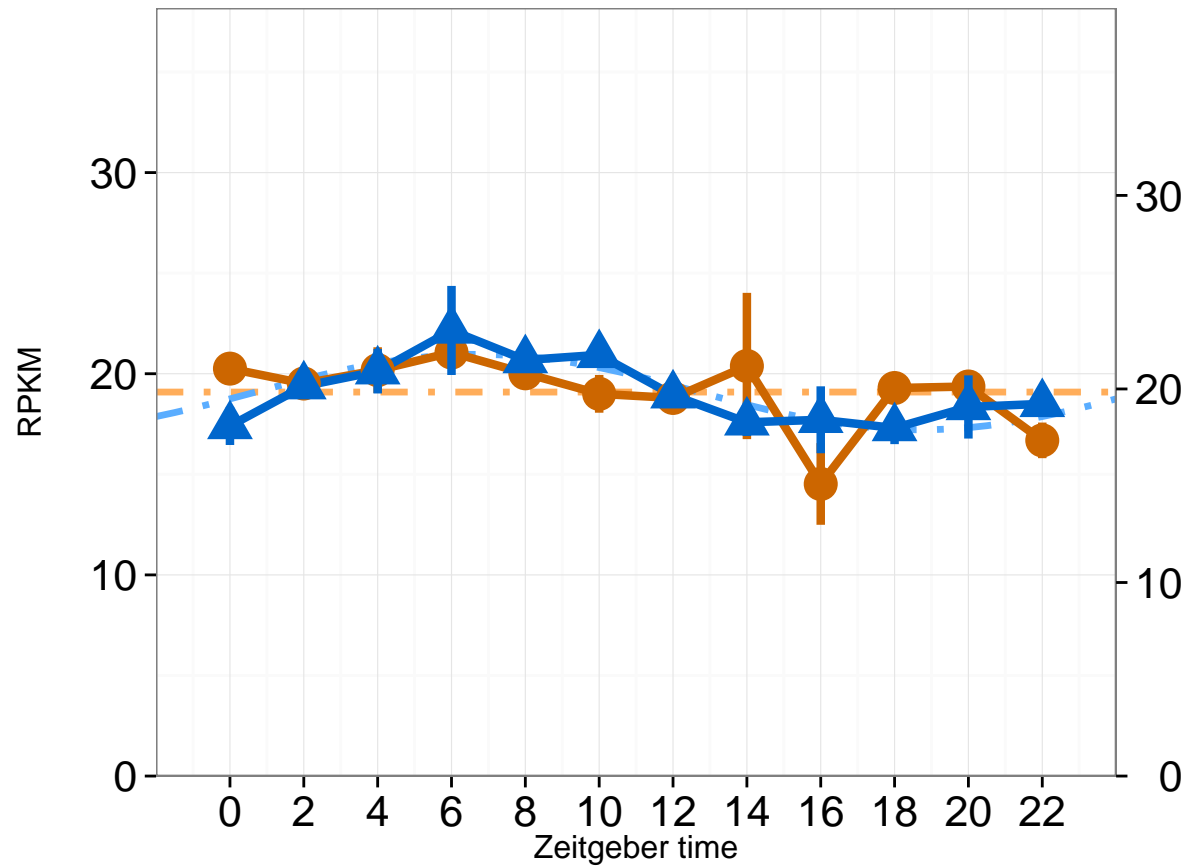

# 2610034B18Rik

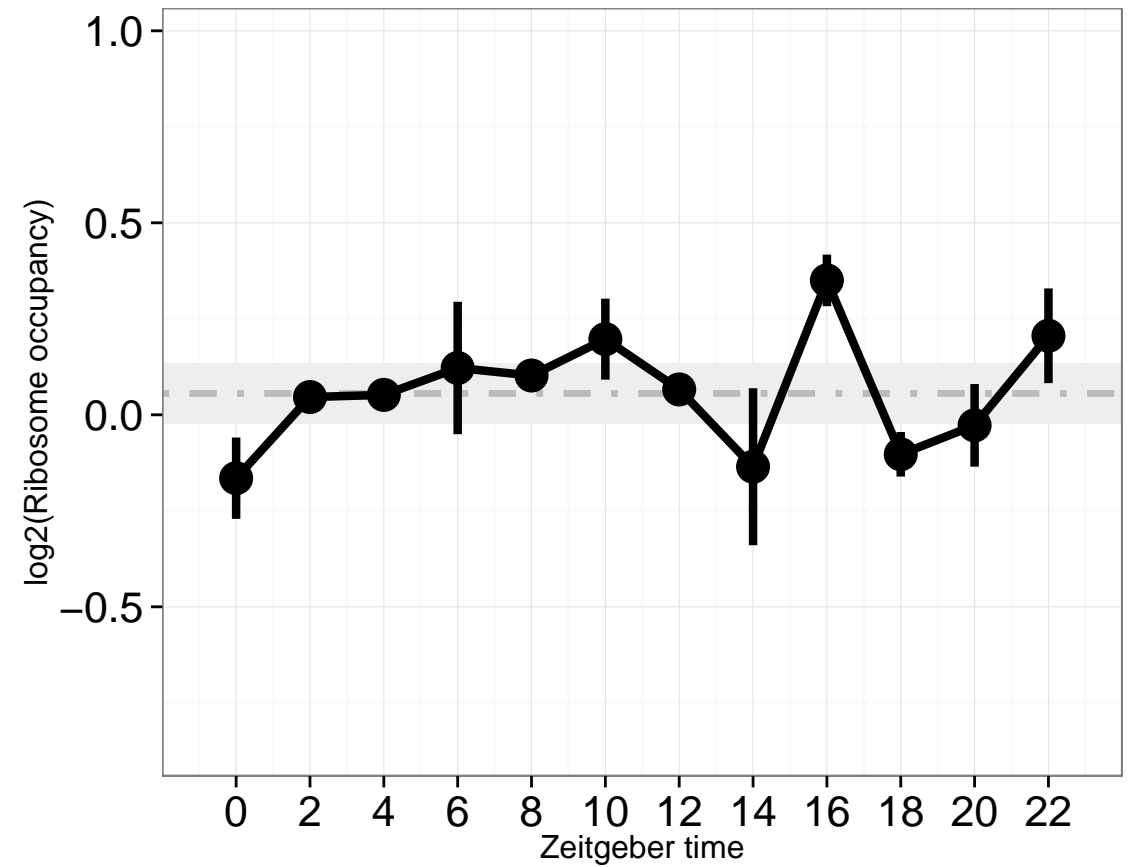

Supplement: Supplementary file 6 — Transcriptome-wide kidney RPF (blue) and RNA (orange) levels in the left panels (with “error bars” connecting the two replicates of each timepoint) and TE in the right panels. (ZIP 116896 kb) [file 13059_2017_1222_MOESM6_ESM.zip › Supp_Dataset_S1/A_RNA_non_rhythmic_RPF_non_rhythmic/2610034B18Rik_kidney_set_A.pdf]

## 2610044O15Rik8

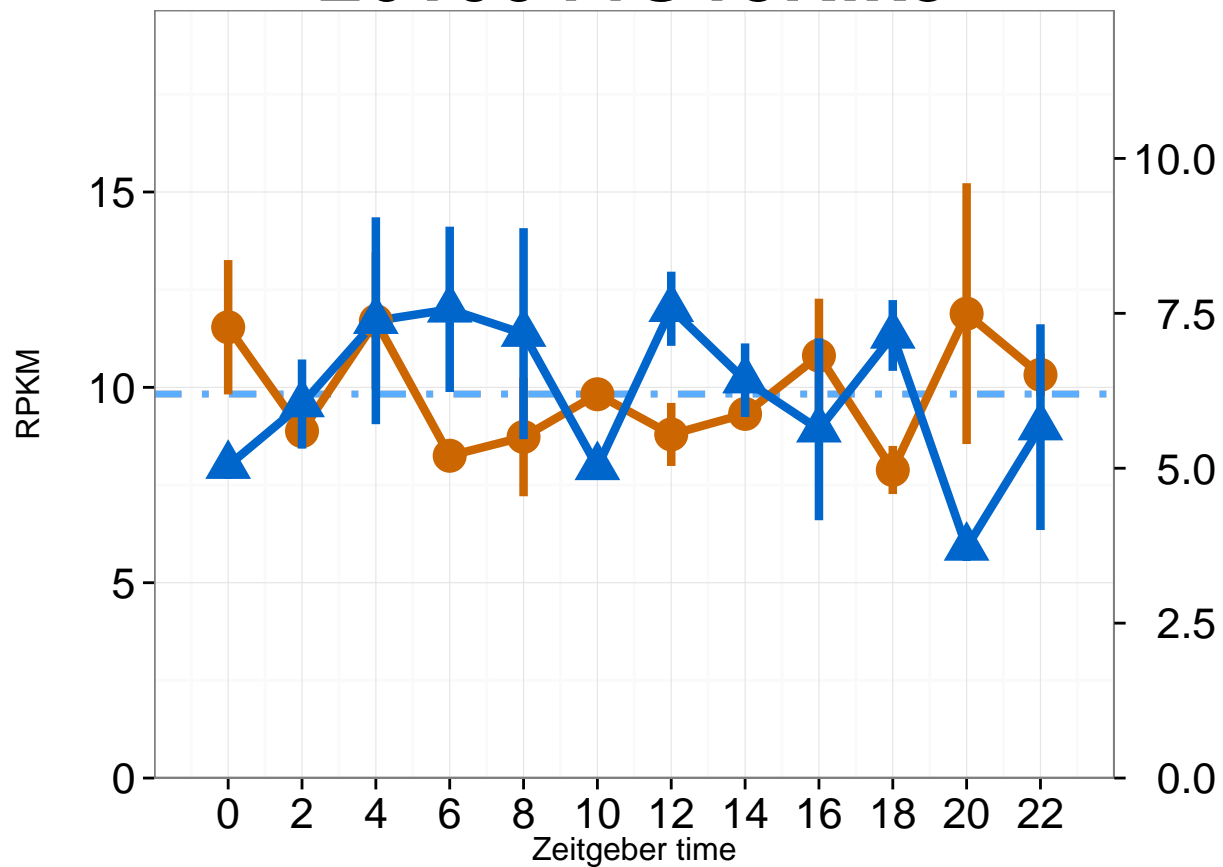

## 2610044O15Rik8

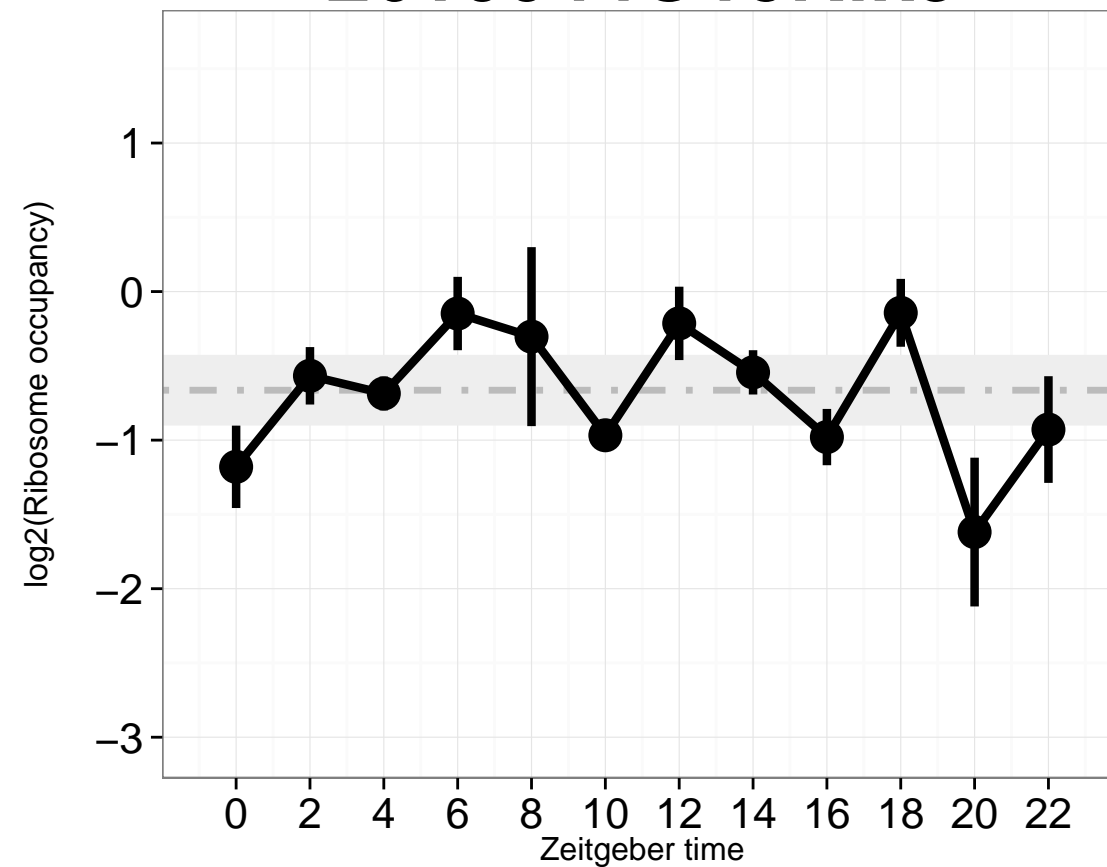

Supplement: Supplementary file 6 — Transcriptome-wide kidney RPF (blue) and RNA (orange) levels in the left panels (with “error bars” connecting the two replicates of each timepoint) and TE in the right panels. (ZIP 116896 kb) [file 13059_2017_1222_MOESM6_ESM.zip › Supp_Dataset_S1/A_RNA_non_rhythmic_RPF_non_rhythmic/2610044O15Rik8_kidney_set_A.pdf]

## 2610301B20Rik

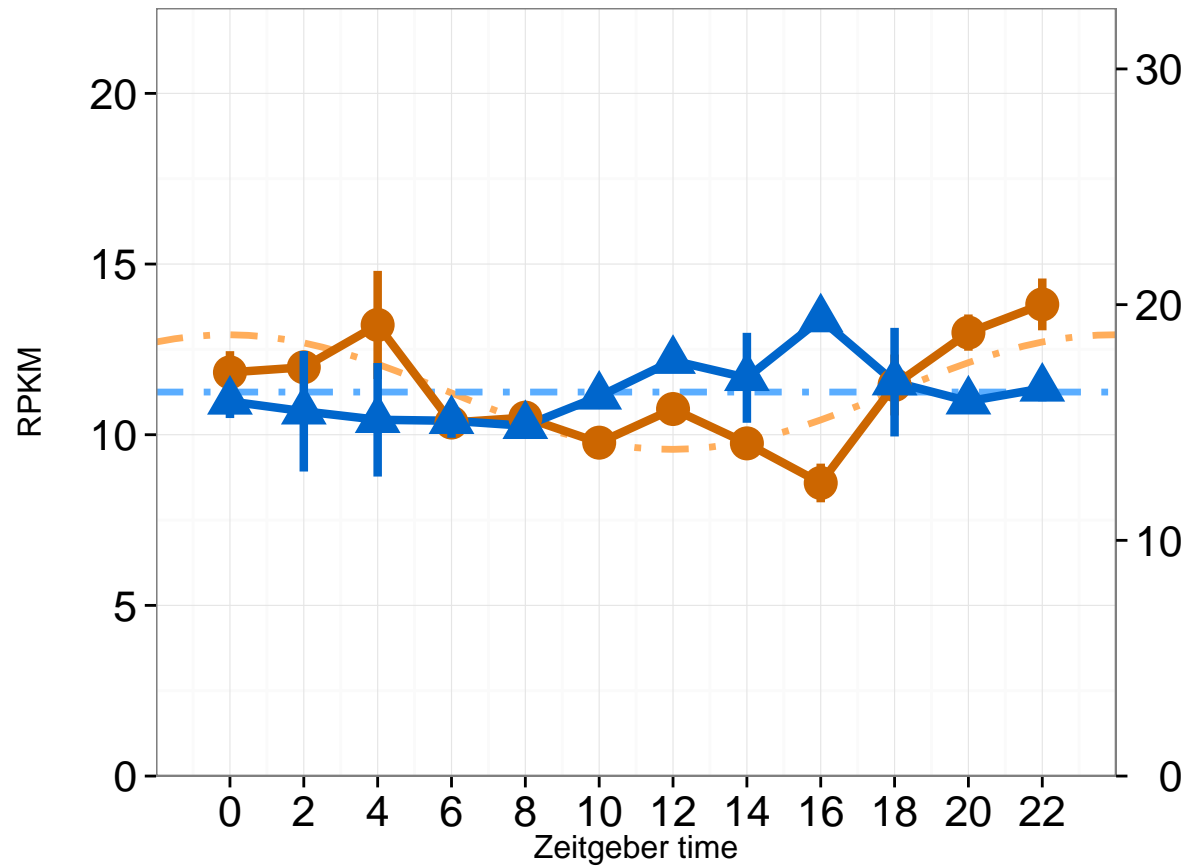

## 2610301B20Rik

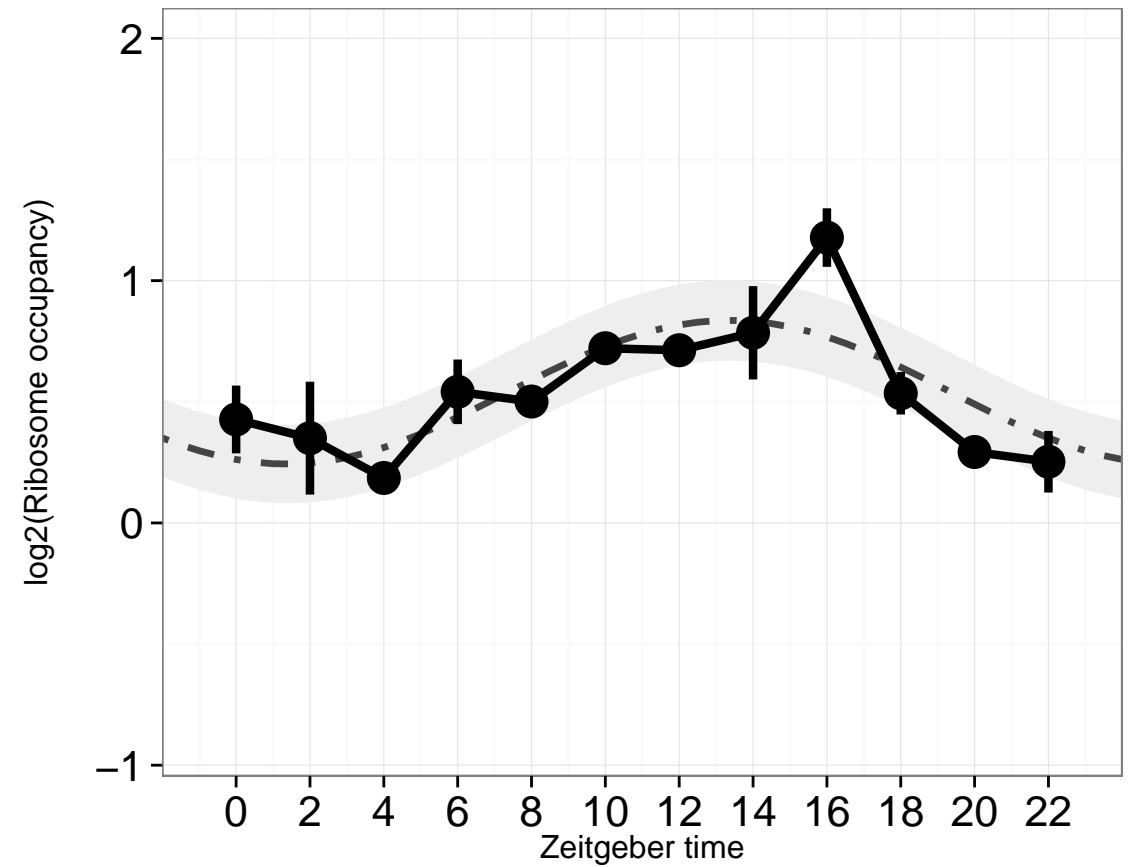

Supplement: Supplementary file 6 — Transcriptome-wide kidney RPF (blue) and RNA (orange) levels in the left panels (with “error bars” connecting the two replicates of each timepoint) and TE in the right panels. (ZIP 116896 kb) [file 13059_2017_1222_MOESM6_ESM.zip › Supp_Dataset_S1/A_RNA_non_rhythmic_RPF_non_rhythmic/2610301B20Rik_kidney_set_A.pdf]

# 2610305D13Rik

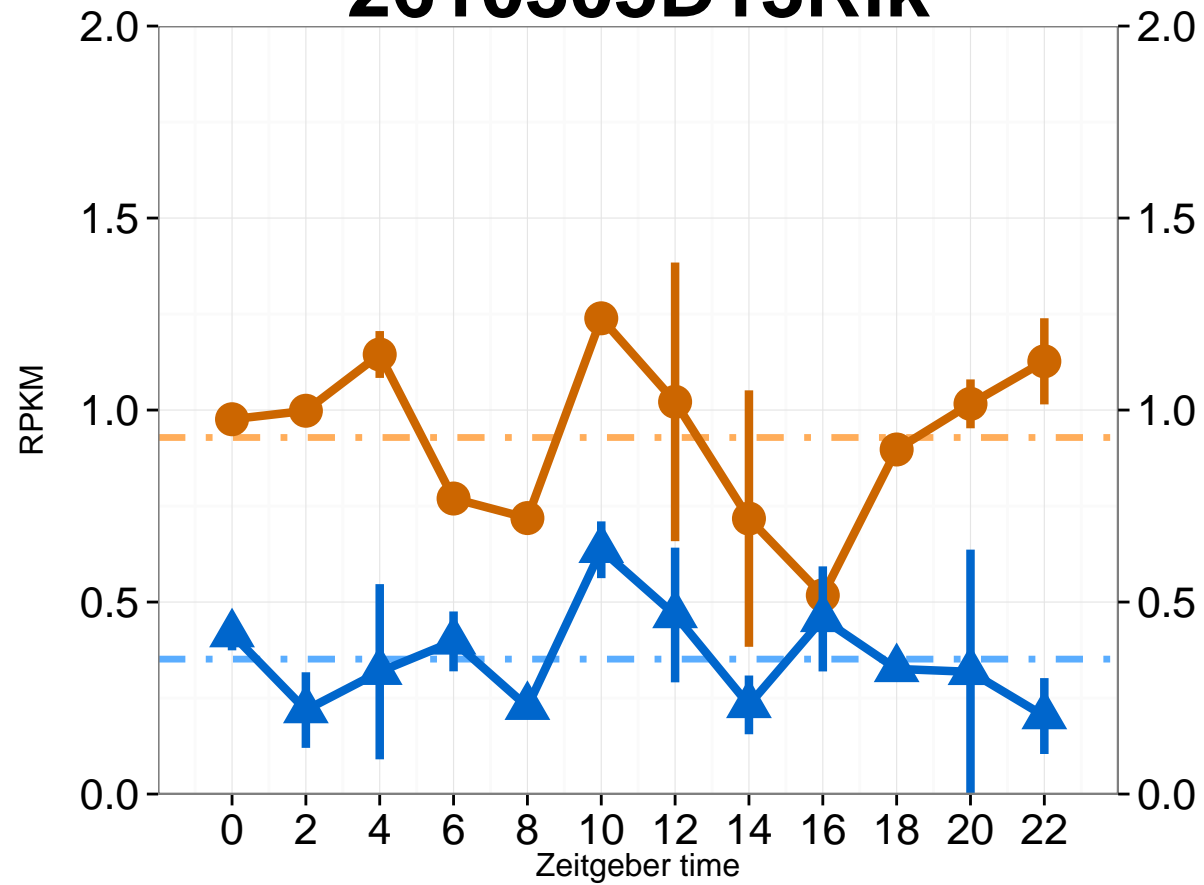

# 2610305D13Rik

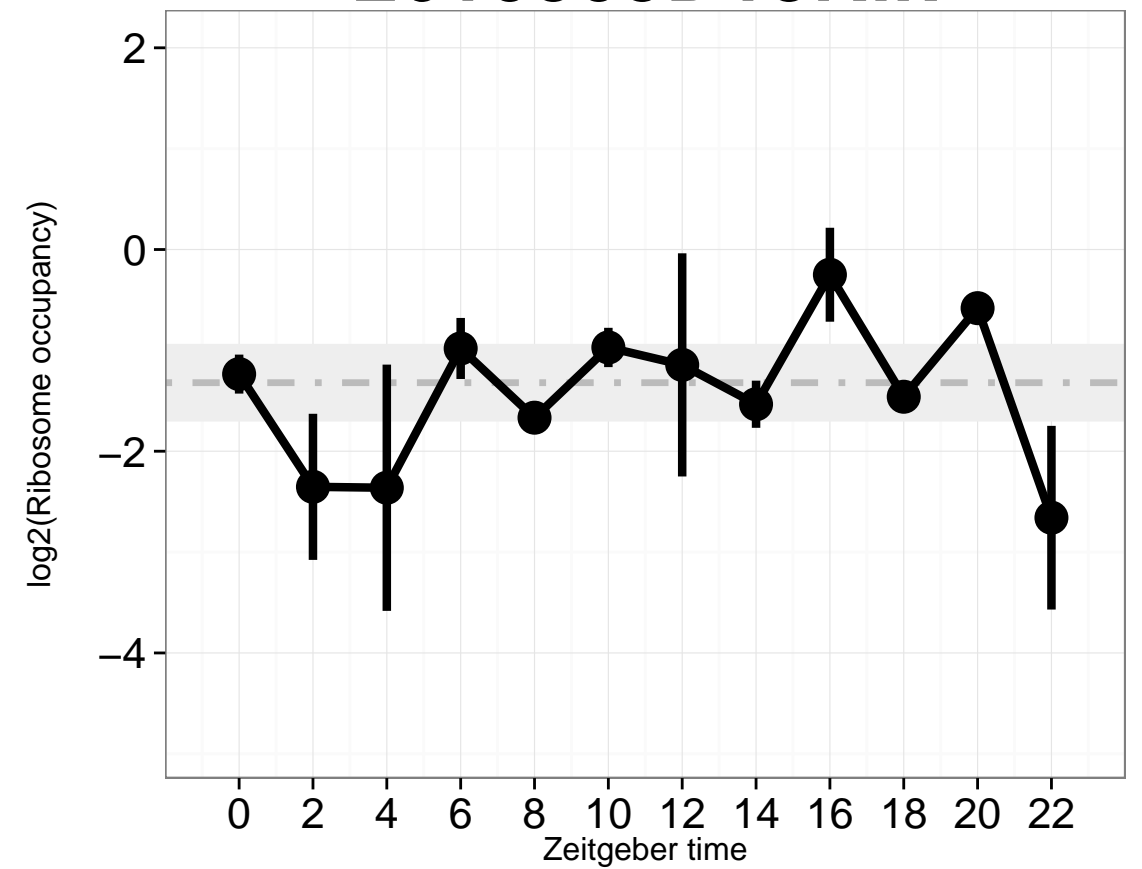

Supplement: Supplementary file 6 — Transcriptome-wide kidney RPF (blue) and RNA (orange) levels in the left panels (with “error bars” connecting the two replicates of each timepoint) and TE in the right panels. (ZIP 116896 kb) [file 13059_2017_1222_MOESM6_ESM.zip › Supp_Dataset_S1/A_RNA_non_rhythmic_RPF_non_rhythmic/2610305D13Rik_kidney_set_A.pdf]

## 2610507B11Rik

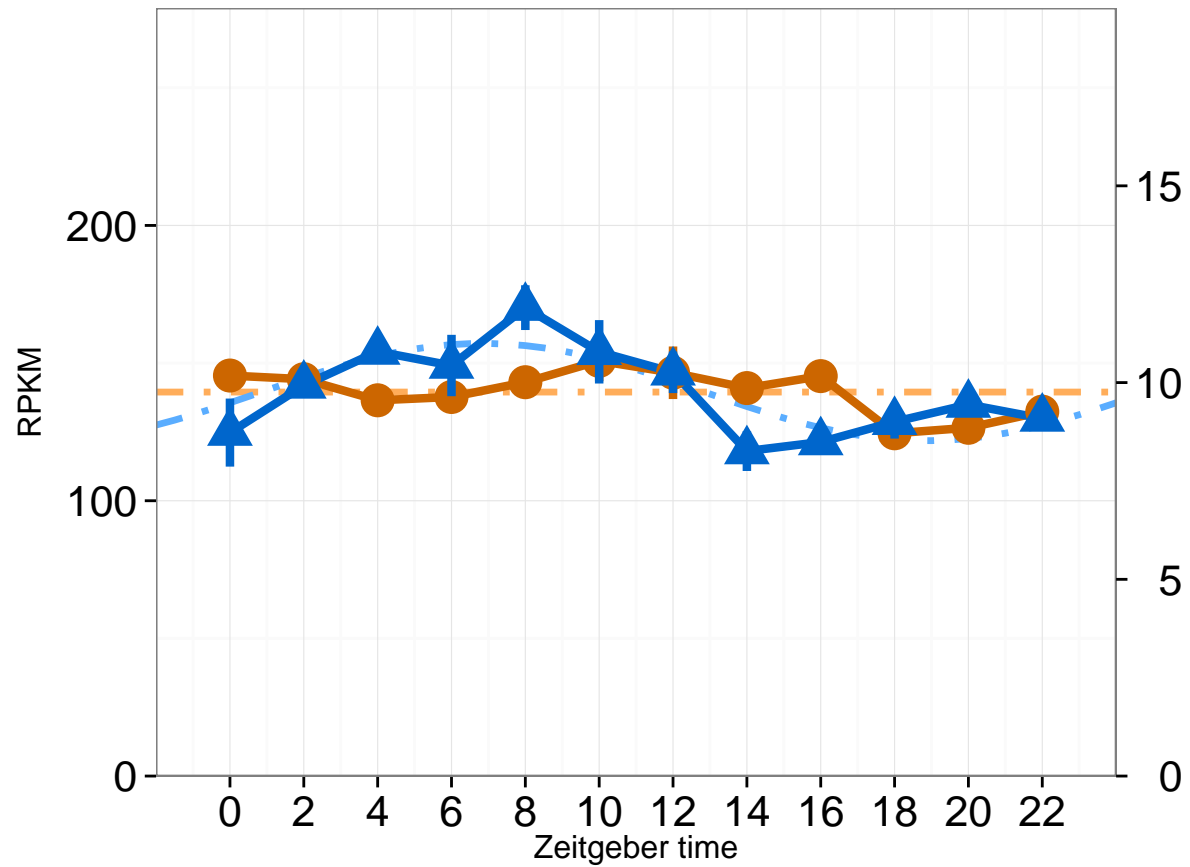

## 2610507B11Rik

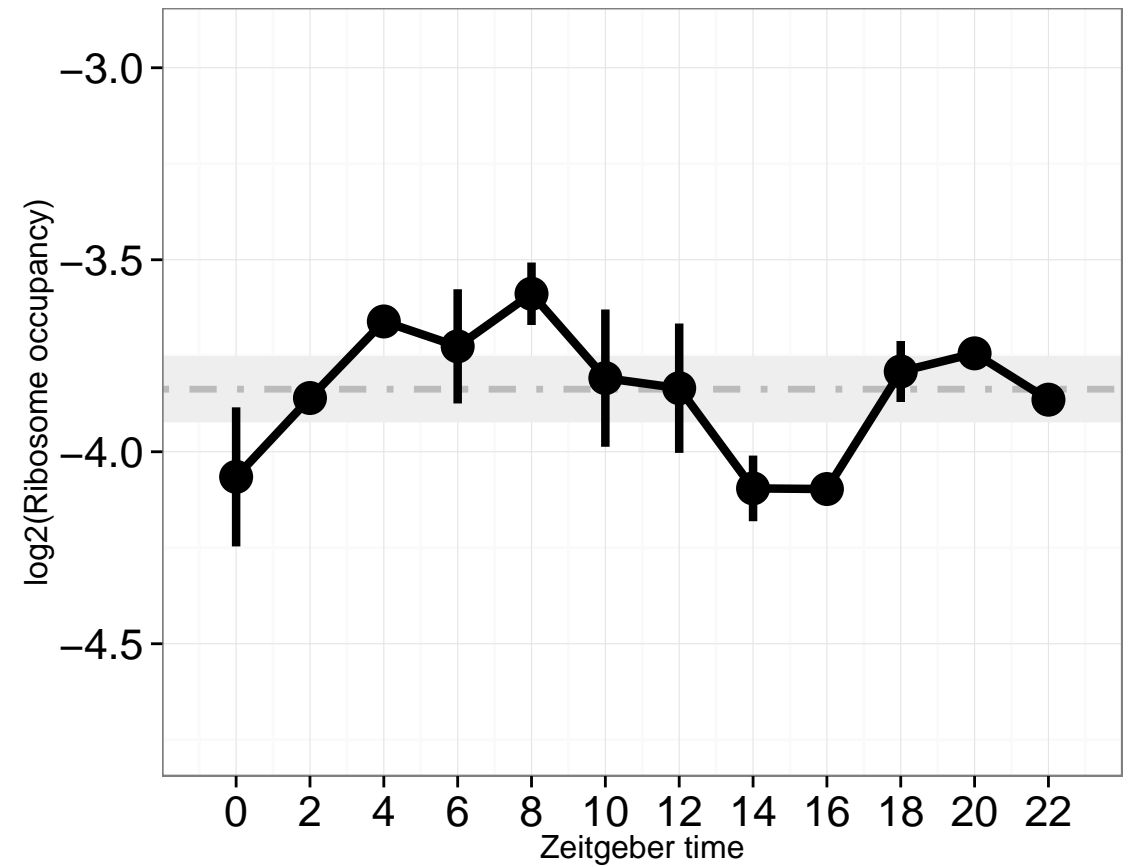

Supplement: Supplementary file 6 — Transcriptome-wide kidney RPF (blue) and RNA (orange) levels in the left panels (with “error bars” connecting the two replicates of each timepoint) and TE in the right panels. (ZIP 116896 kb) [file 13059_2017_1222_MOESM6_ESM.zip › Supp_Dataset_S1/A_RNA_non_rhythmic_RPF_non_rhythmic/2610507B11Rik_kidney_set_A.pdf]

# 2610524H06Rik

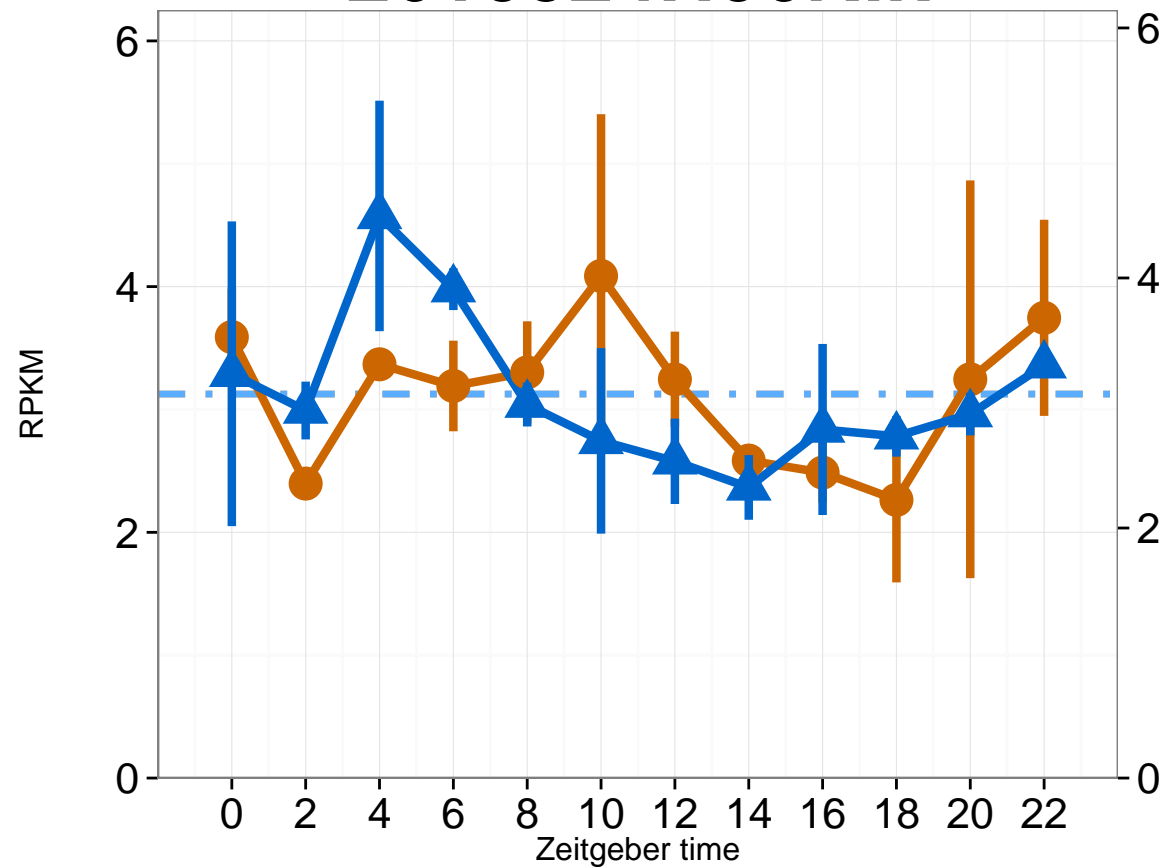

# 2610524H06Rik

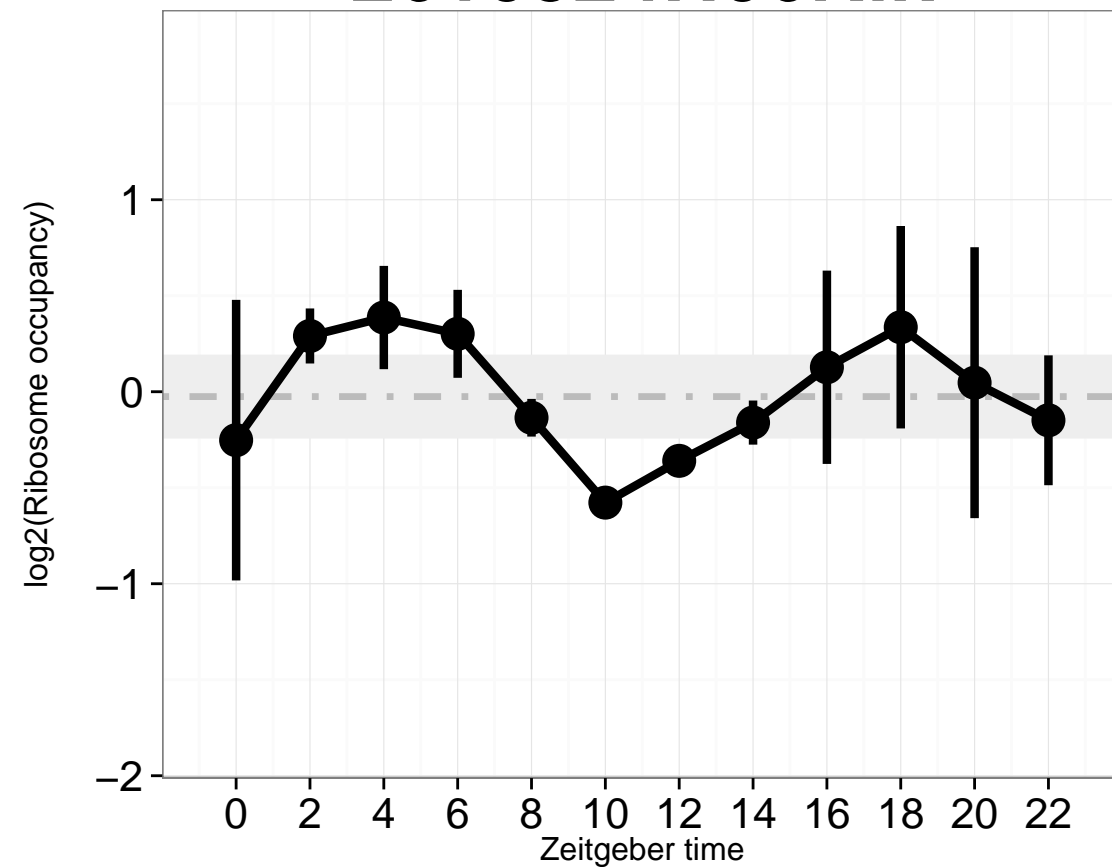

Supplement: Supplementary file 6 — Transcriptome-wide kidney RPF (blue) and RNA (orange) levels in the left panels (with “error bars” connecting the two replicates of each timepoint) and TE in the right panels. (ZIP 116896 kb) [file 13059_2017_1222_MOESM6_ESM.zip › Supp_Dataset_S1/A_RNA_non_rhythmic_RPF_non_rhythmic/2610524H06Rik_kidney_set_A.pdf]

# 2610528J11Rik

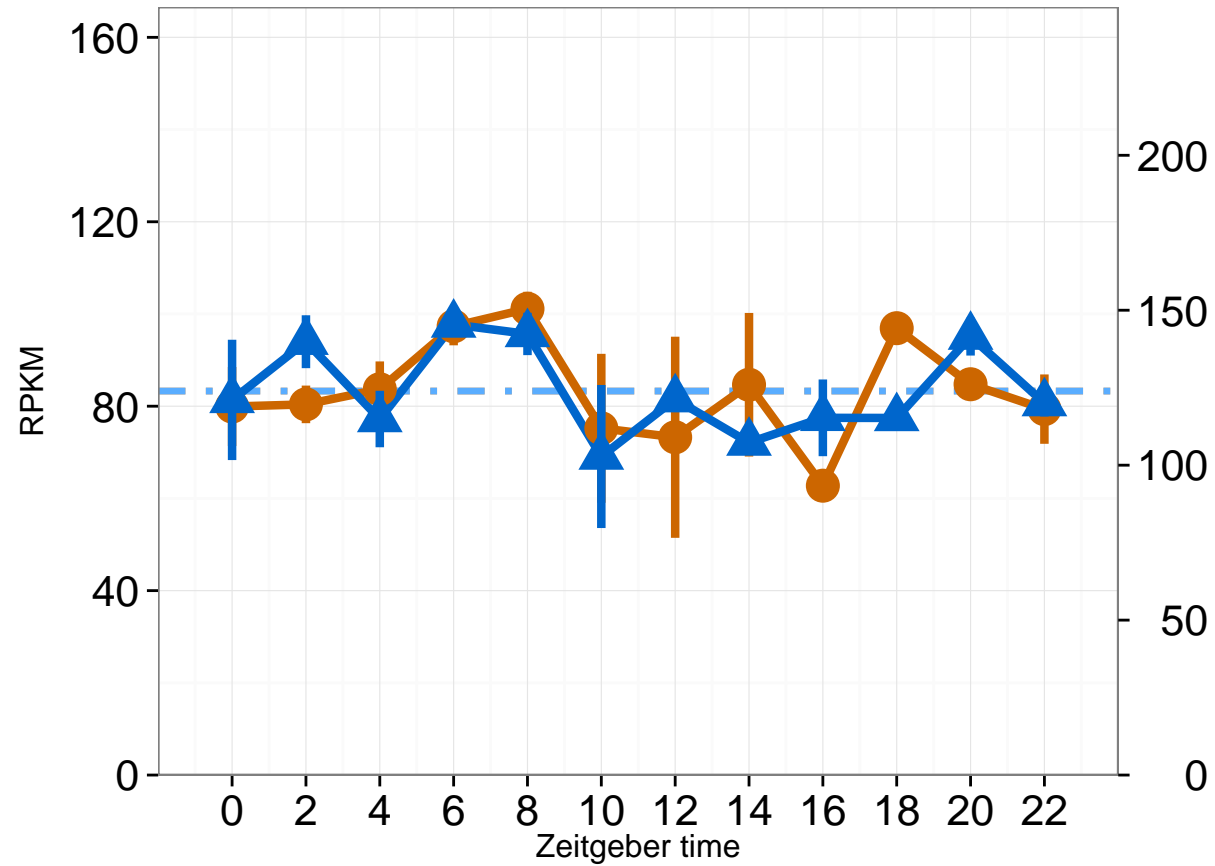

# 2610528J11Rik

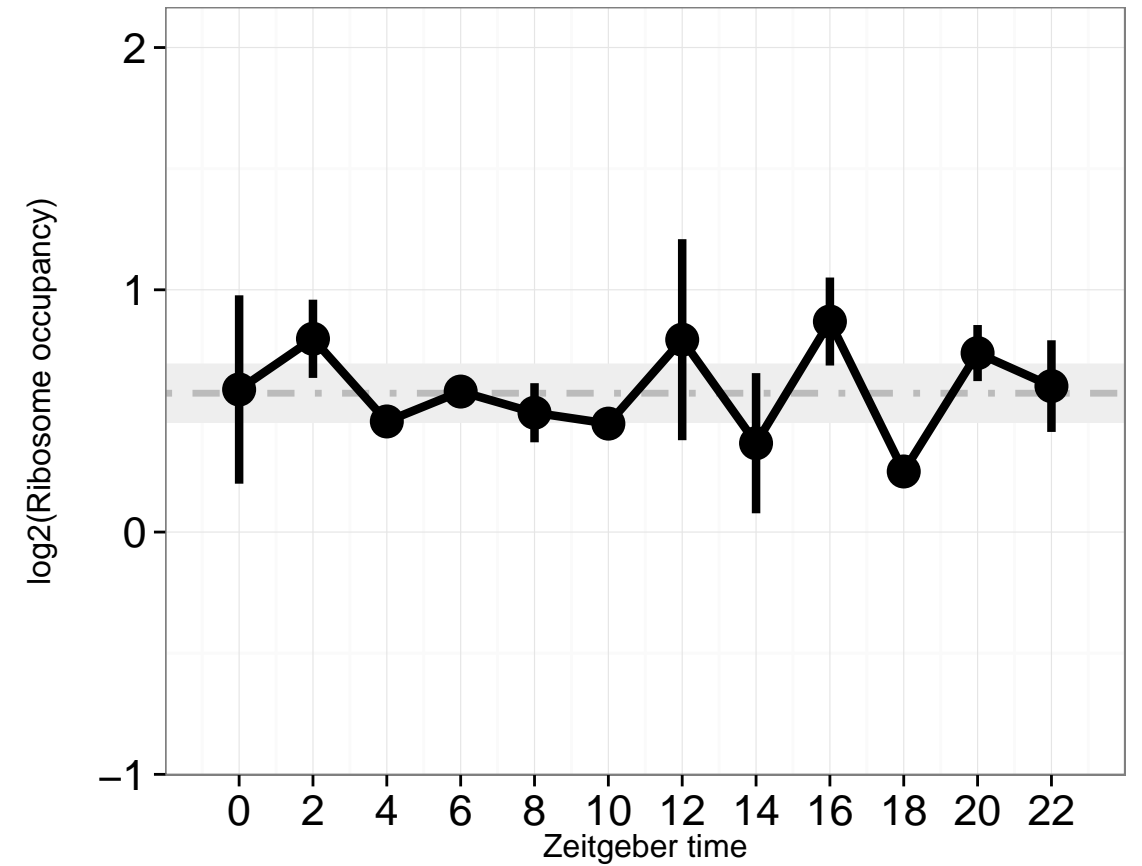

Supplement: Supplementary file 6 — Transcriptome-wide kidney RPF (blue) and RNA (orange) levels in the left panels (with “error bars” connecting the two replicates of each timepoint) and TE in the right panels. (ZIP 116896 kb) [file 13059_2017_1222_MOESM6_ESM.zip › Supp_Dataset_S1/A_RNA_non_rhythmic_RPF_non_rhythmic/2610528J11Rik_kidney_set_A.pdf]

## 2700029M09Rik

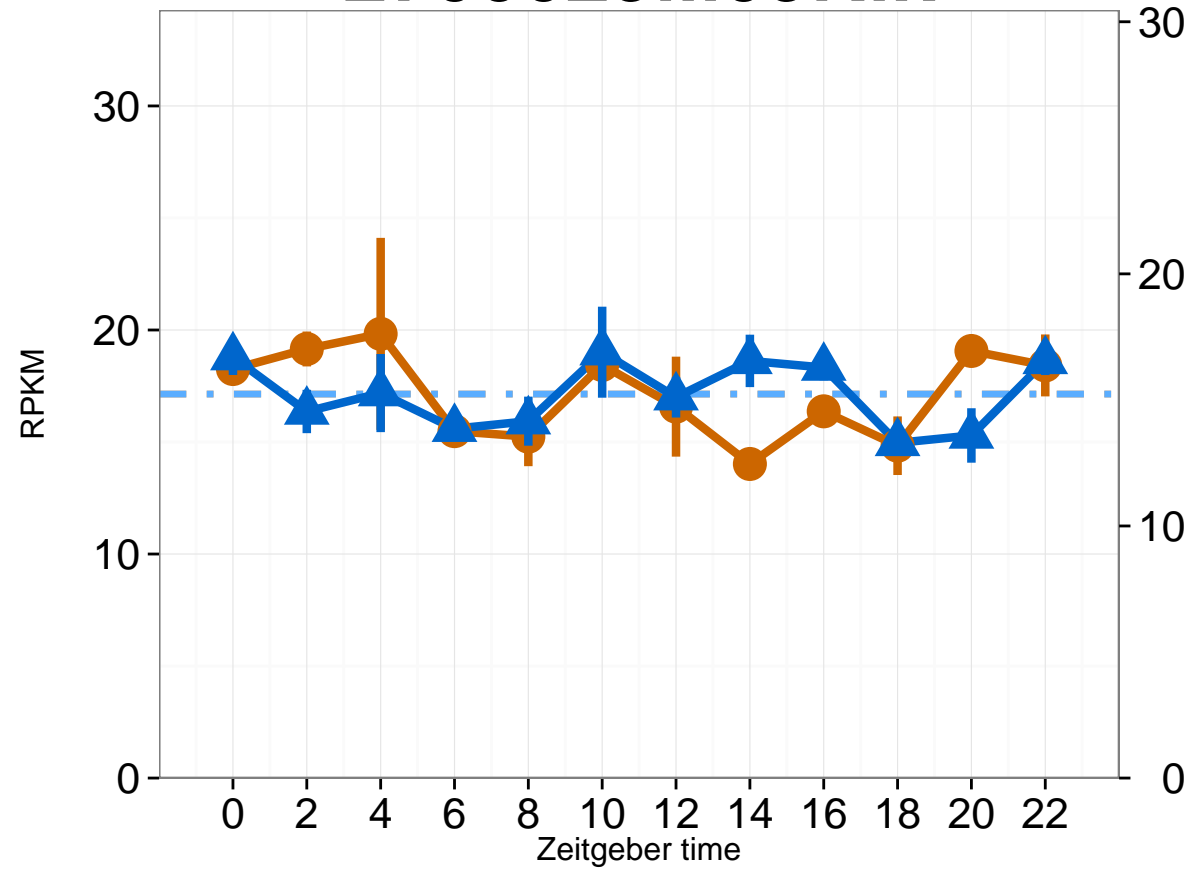

## 2700029M09Rik

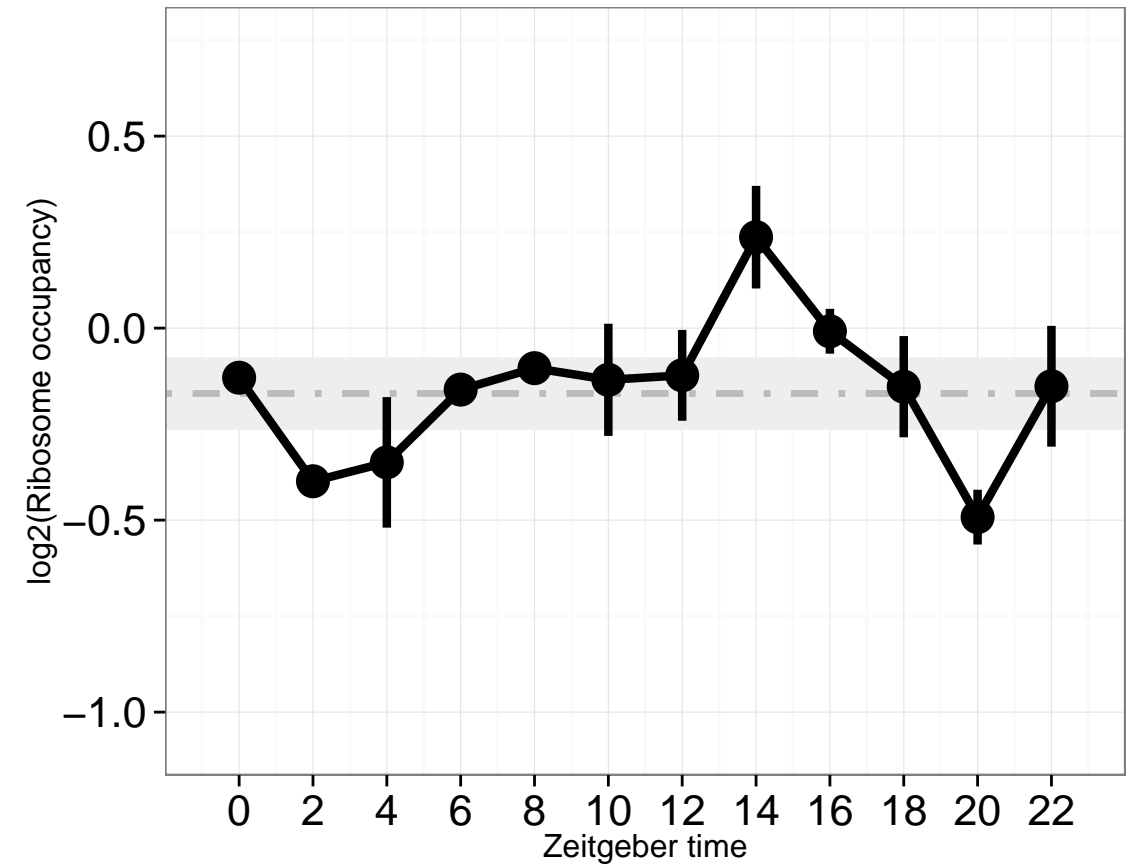

Supplement: Supplementary file 6 — Transcriptome-wide kidney RPF (blue) and RNA (orange) levels in the left panels (with “error bars” connecting the two replicates of each timepoint) and TE in the right panels. (ZIP 116896 kb) [file 13059_2017_1222_MOESM6_ESM.zip › Supp_Dataset_S1/A_RNA_non_rhythmic_RPF_non_rhythmic/2700029M09Rik_kidney_set_A.pdf]

## 2700049A03Rik

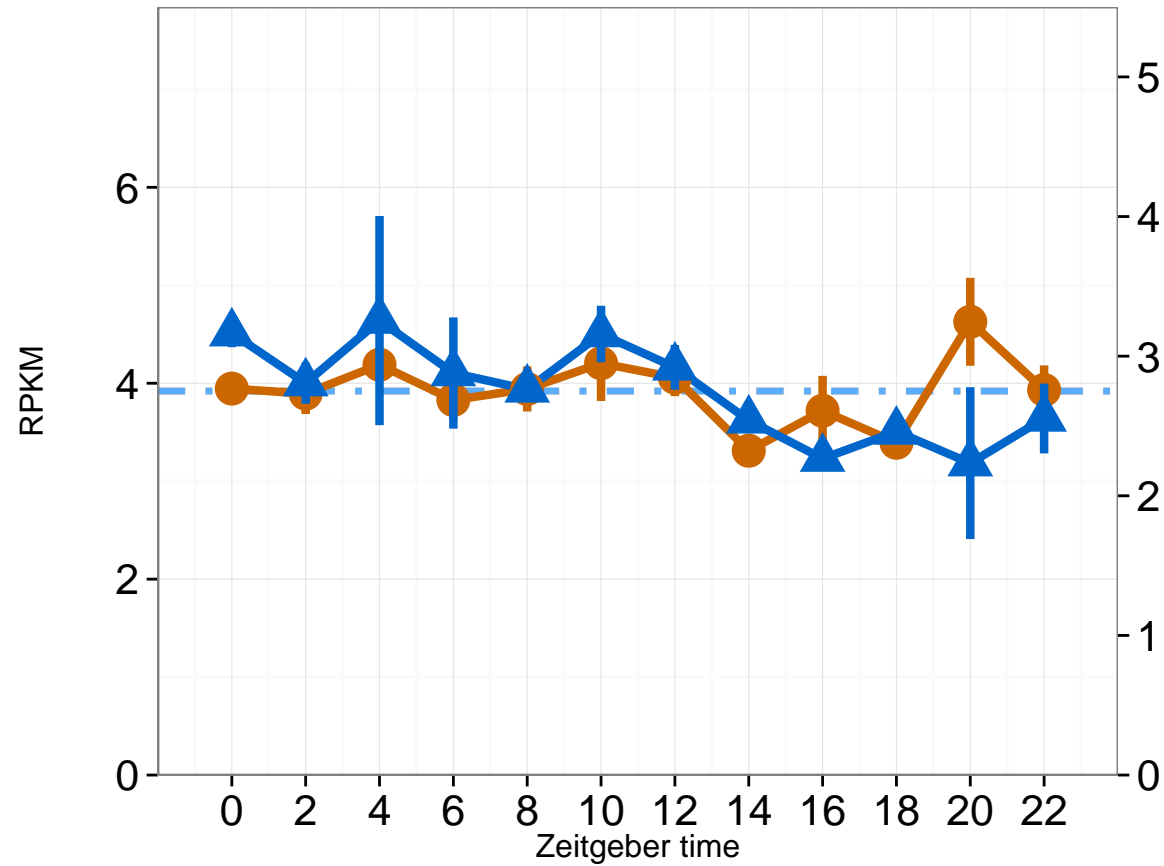

## 2700049A03Rik

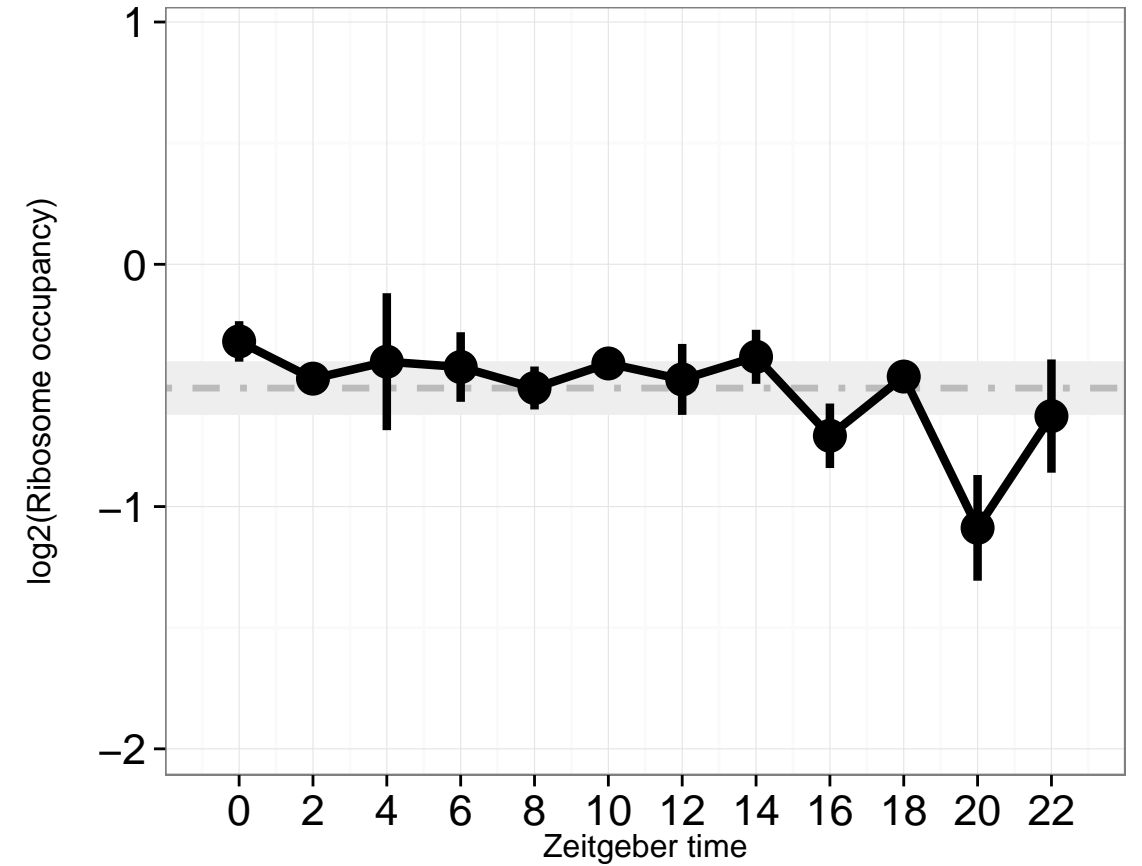

Supplement: Supplementary file 6 — Transcriptome-wide kidney RPF (blue) and RNA (orange) levels in the left panels (with “error bars” connecting the two replicates of each timepoint) and TE in the right panels. (ZIP 116896 kb) [file 13059_2017_1222_MOESM6_ESM.zip › Supp_Dataset_S1/A_RNA_non_rhythmic_RPF_non_rhythmic/2700049A03Rik_kidney_set_A.pdf]

## 2700050L05Rik

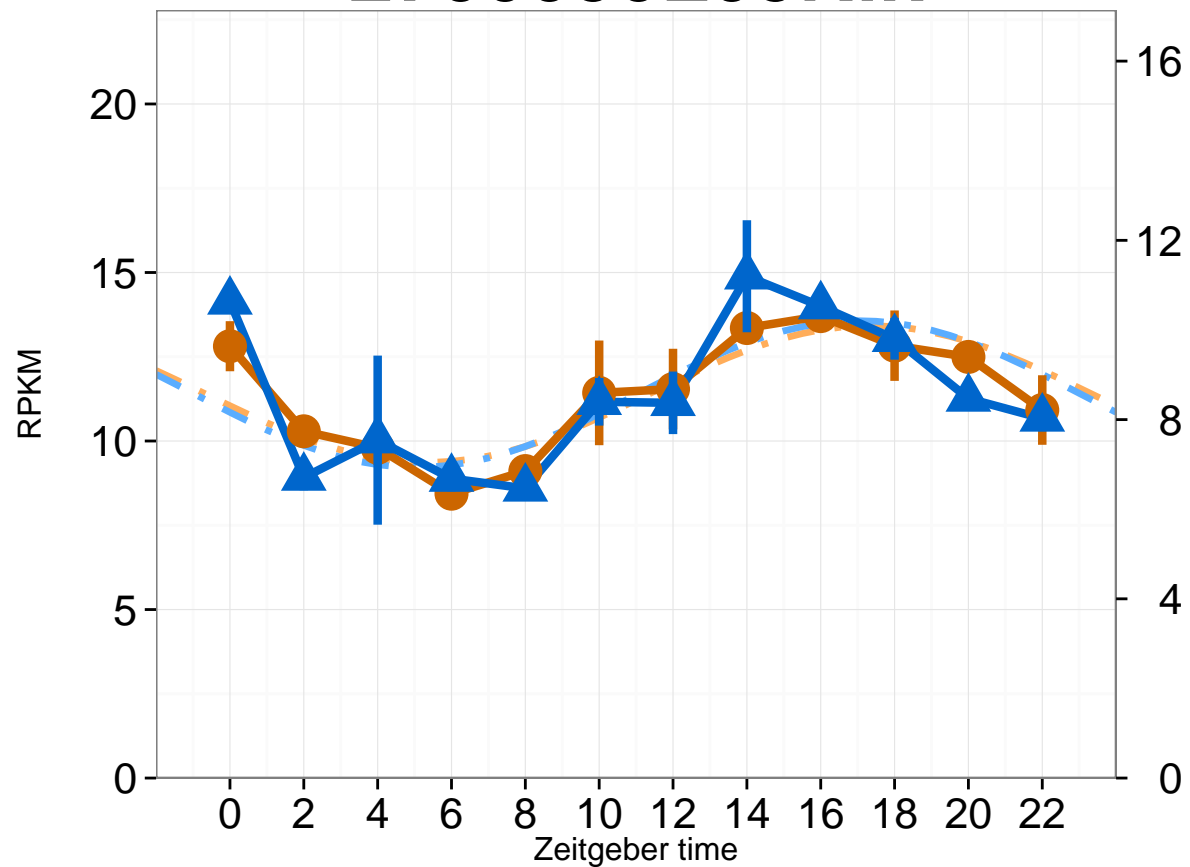

## 2700050L05Rik

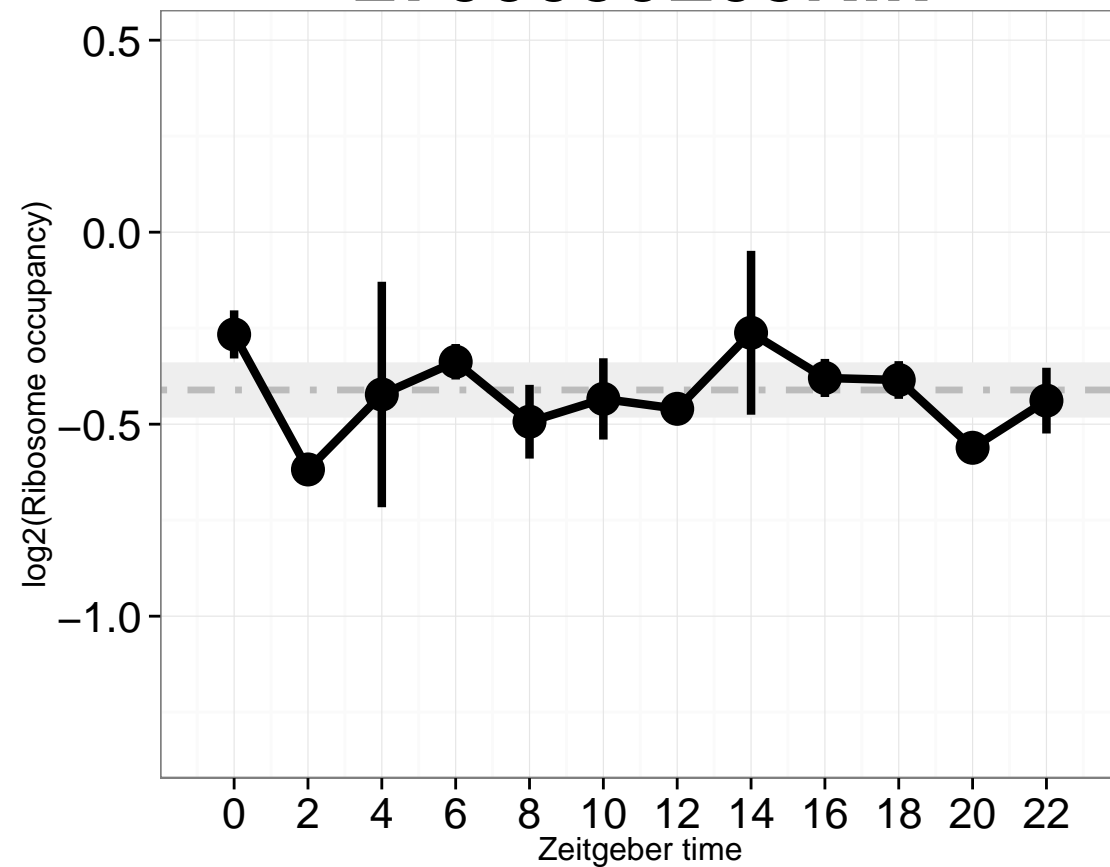

Supplement: Supplementary file 6 — Transcriptome-wide kidney RPF (blue) and RNA (orange) levels in the left panels (with “error bars” connecting the two replicates of each timepoint) and TE in the right panels. (ZIP 116896 kb) [file 13059_2017_1222_MOESM6_ESM.zip › Supp_Dataset_S1/A_RNA_non_rhythmic_RPF_non_rhythmic/2700050L05Rik_kidney_set_A.pdf]

## 2700060E02Rik

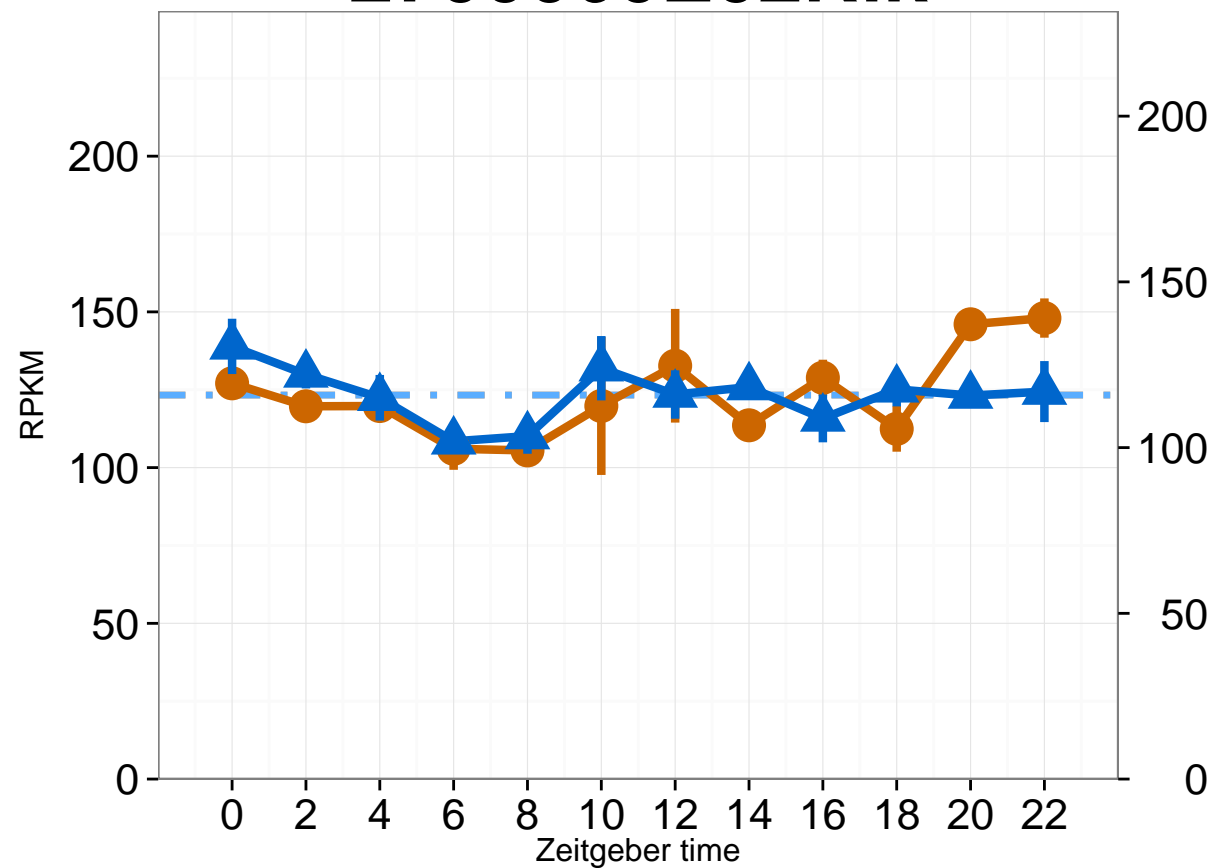

## 2700060E02Rik

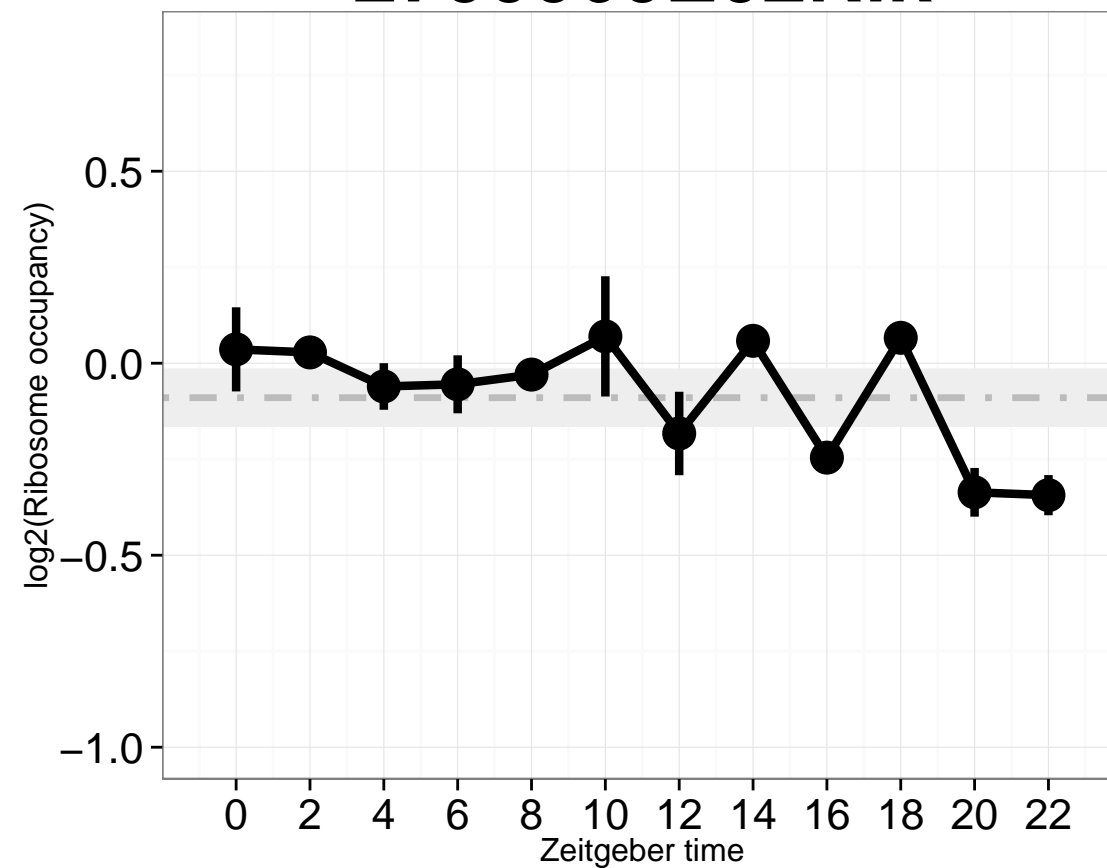

Supplement: Supplementary file 6 — Transcriptome-wide kidney RPF (blue) and RNA (orange) levels in the left panels (with “error bars” connecting the two replicates of each timepoint) and TE in the right panels. (ZIP 116896 kb) [file 13059_2017_1222_MOESM6_ESM.zip › Supp_Dataset_S1/A_RNA_non_rhythmic_RPF_non_rhythmic/2700060E02Rik_kidney_set_A.pdf]

## 2700062C07Rik

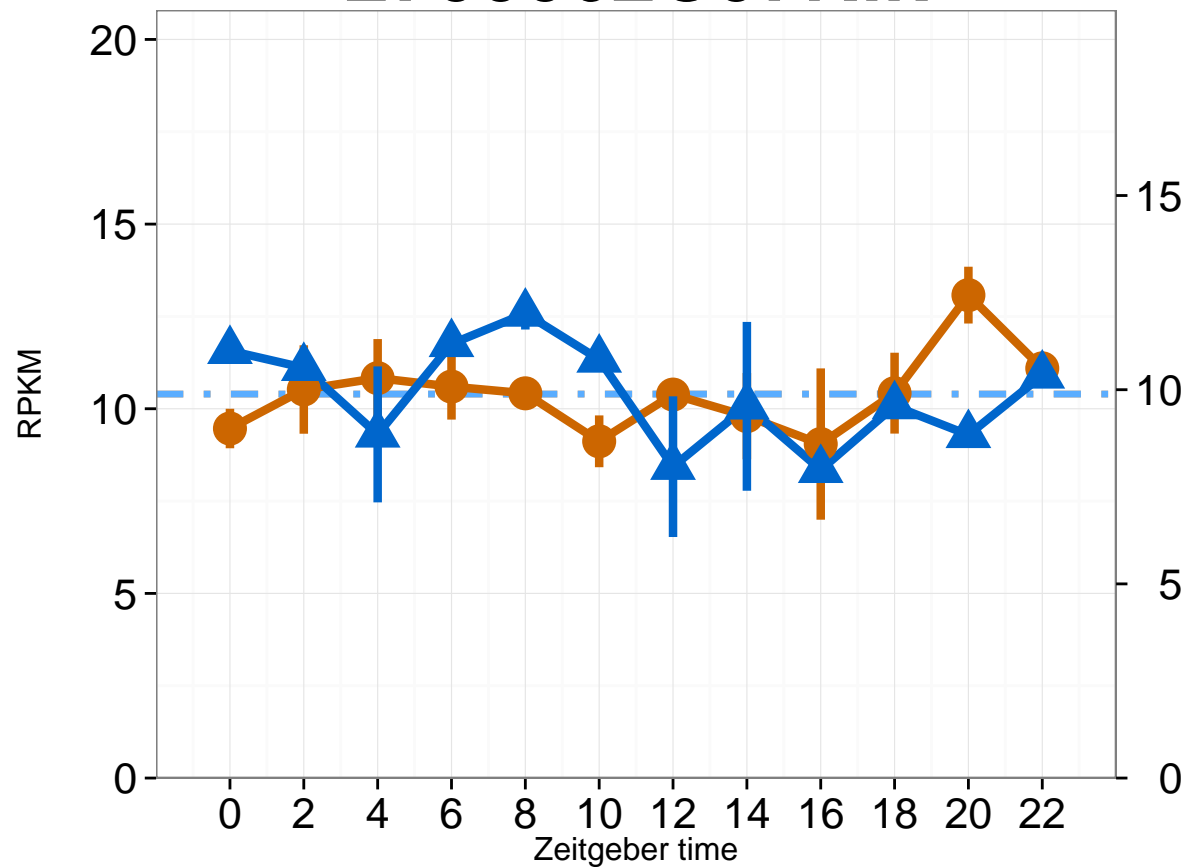

## 2700062C07Rik

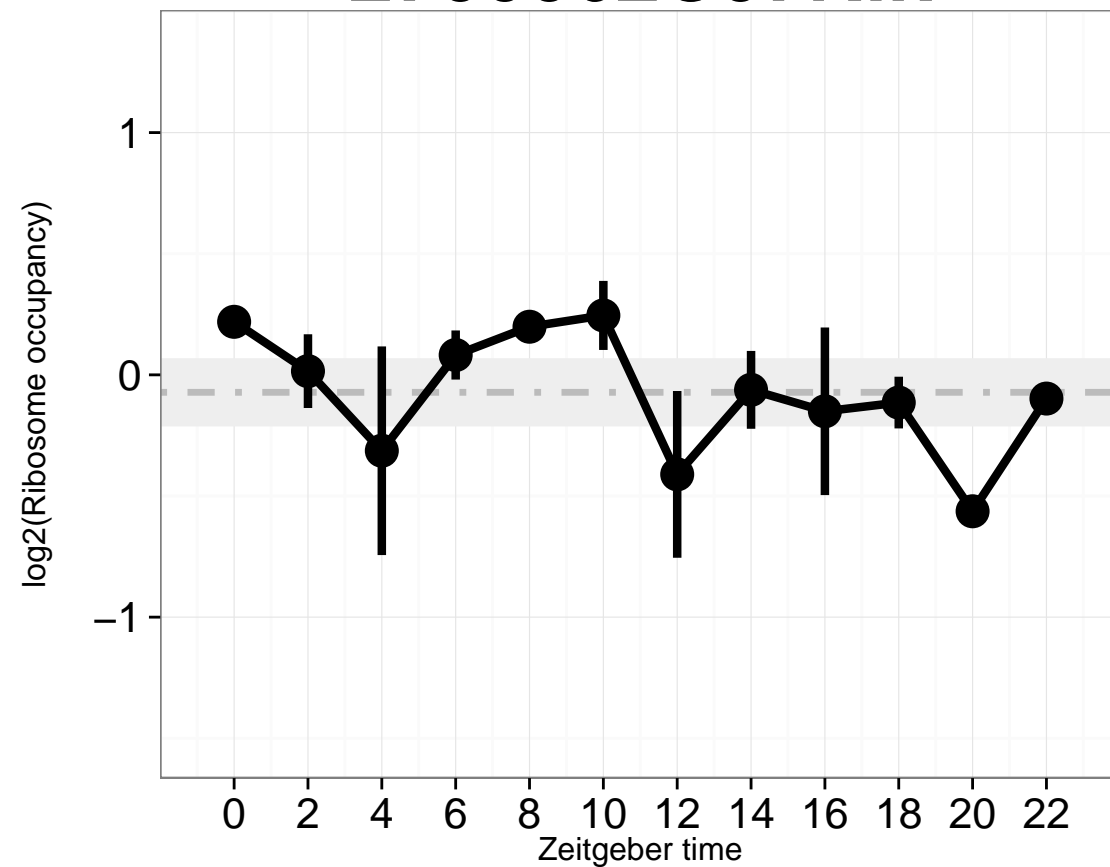

Supplement: Supplementary file 6 — Transcriptome-wide kidney RPF (blue) and RNA (orange) levels in the left panels (with “error bars” connecting the two replicates of each timepoint) and TE in the right panels. (ZIP 116896 kb) [file 13059_2017_1222_MOESM6_ESM.zip › Supp_Dataset_S1/A_RNA_non_rhythmic_RPF_non_rhythmic/2700062C07Rik_kidney_set_A.pdf]

## 2700094K13Rik

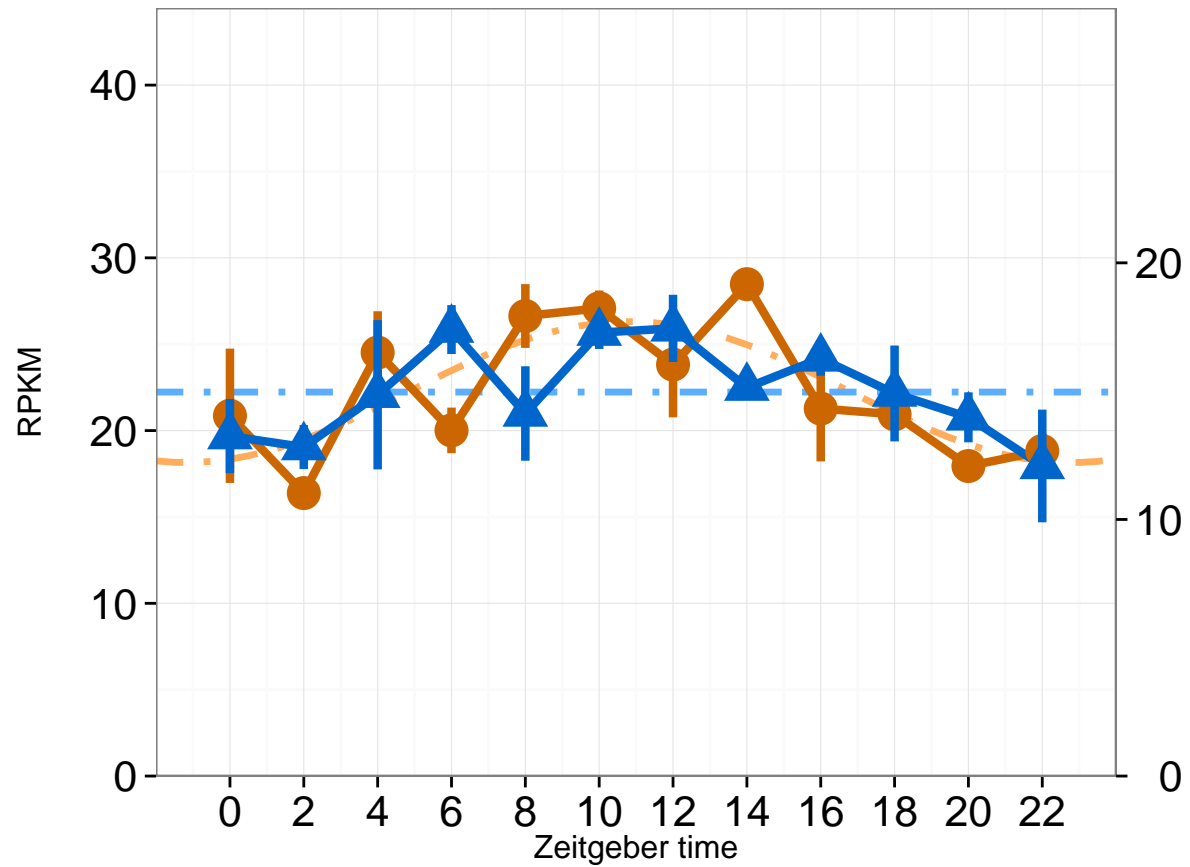

## 2700094K13Rik

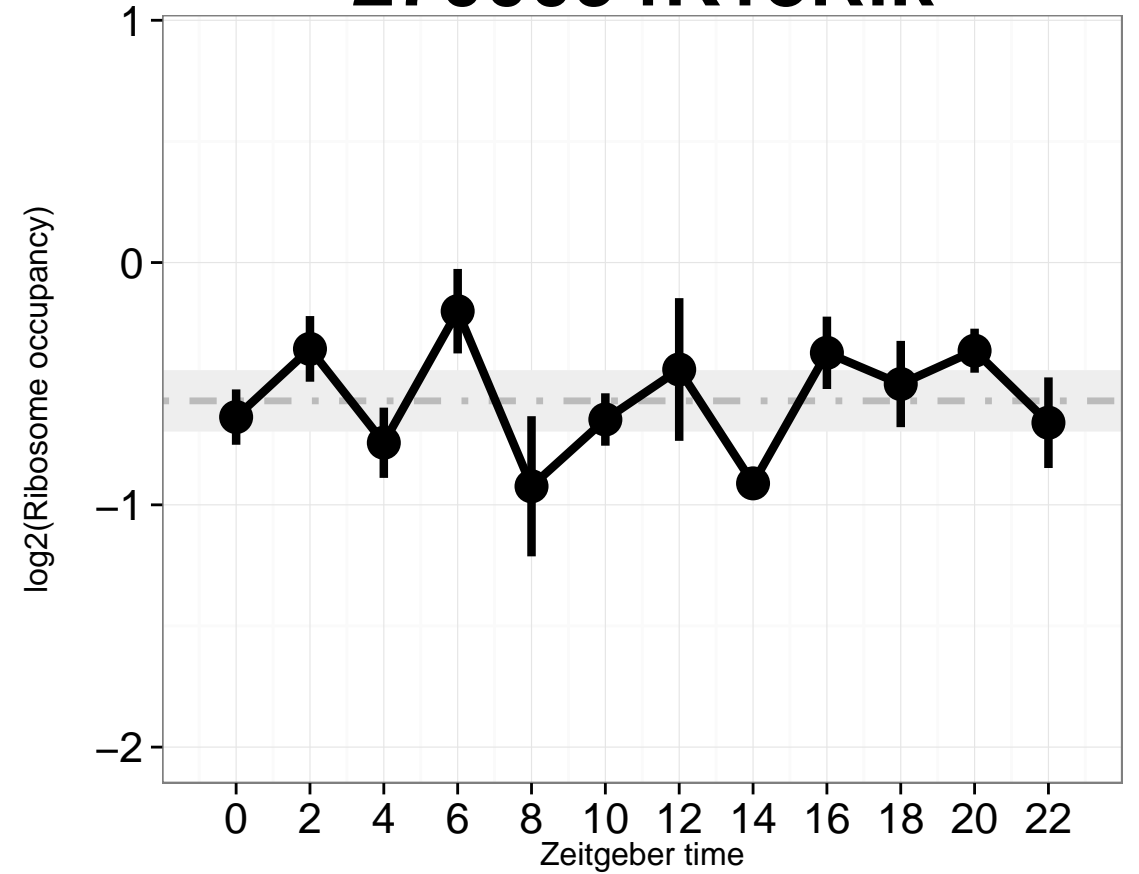

Supplement: Supplementary file 6 — Transcriptome-wide kidney RPF (blue) and RNA (orange) levels in the left panels (with “error bars” connecting the two replicates of each timepoint) and TE in the right panels. (ZIP 116896 kb) [file 13059_2017_1222_MOESM6_ESM.zip › Supp_Dataset_S1/A_RNA_non_rhythmic_RPF_non_rhythmic/2700094K13Rik_kidney_set_A.pdf]

## 2700097O09Rik

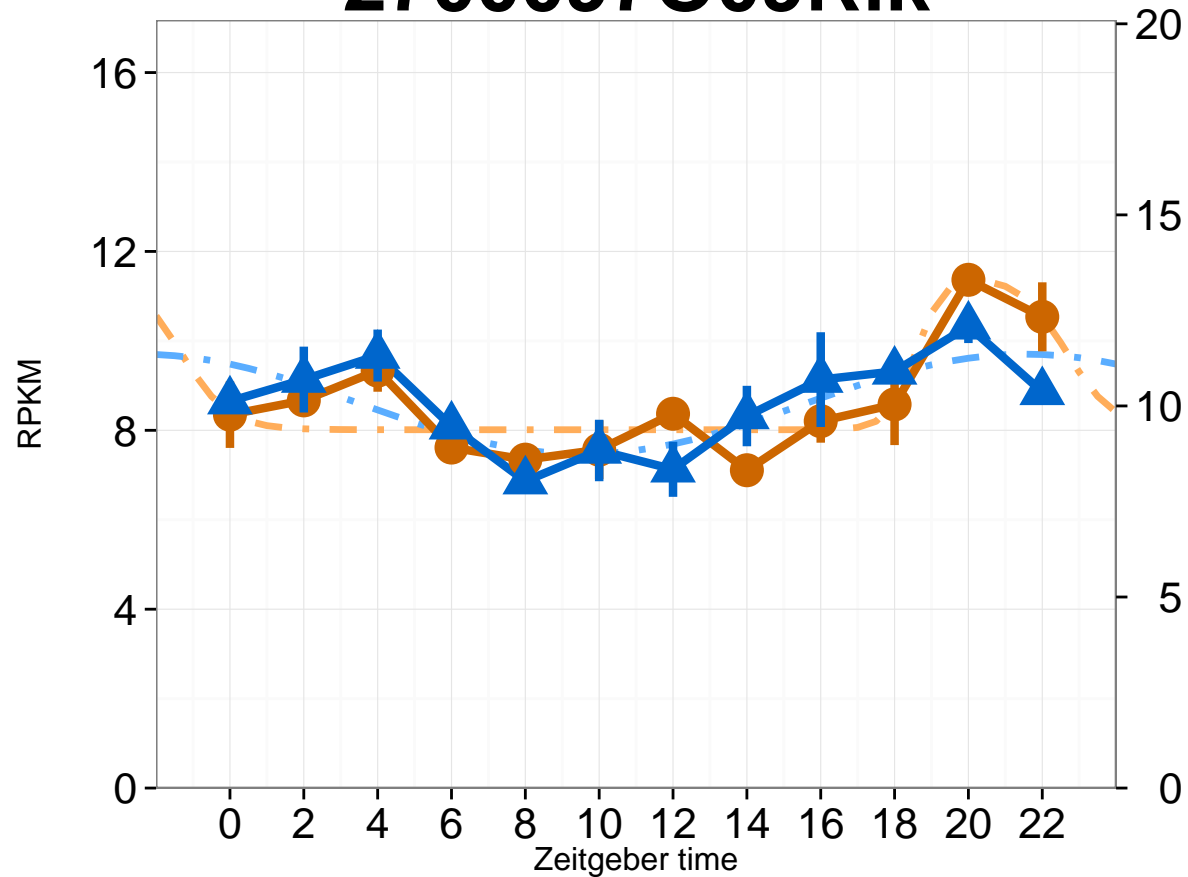

## 2700097O09Rik

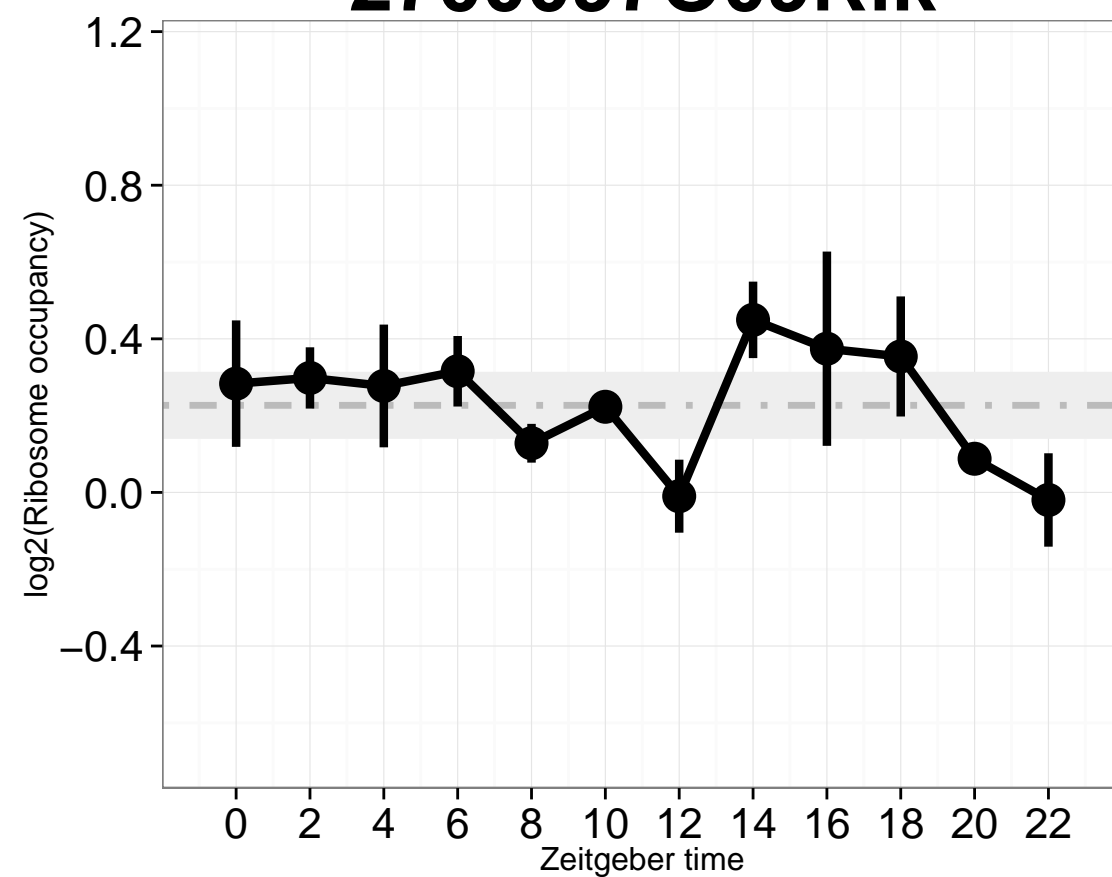

Supplement: Supplementary file 6 — Transcriptome-wide kidney RPF (blue) and RNA (orange) levels in the left panels (with “error bars” connecting the two replicates of each timepoint) and TE in the right panels. (ZIP 116896 kb) [file 13059_2017_1222_MOESM6_ESM.zip › Supp_Dataset_S1/A_RNA_non_rhythmic_RPF_non_rhythmic/2700097O09Rik_kidney_set_A.pdf]

# 2810004N23Rik

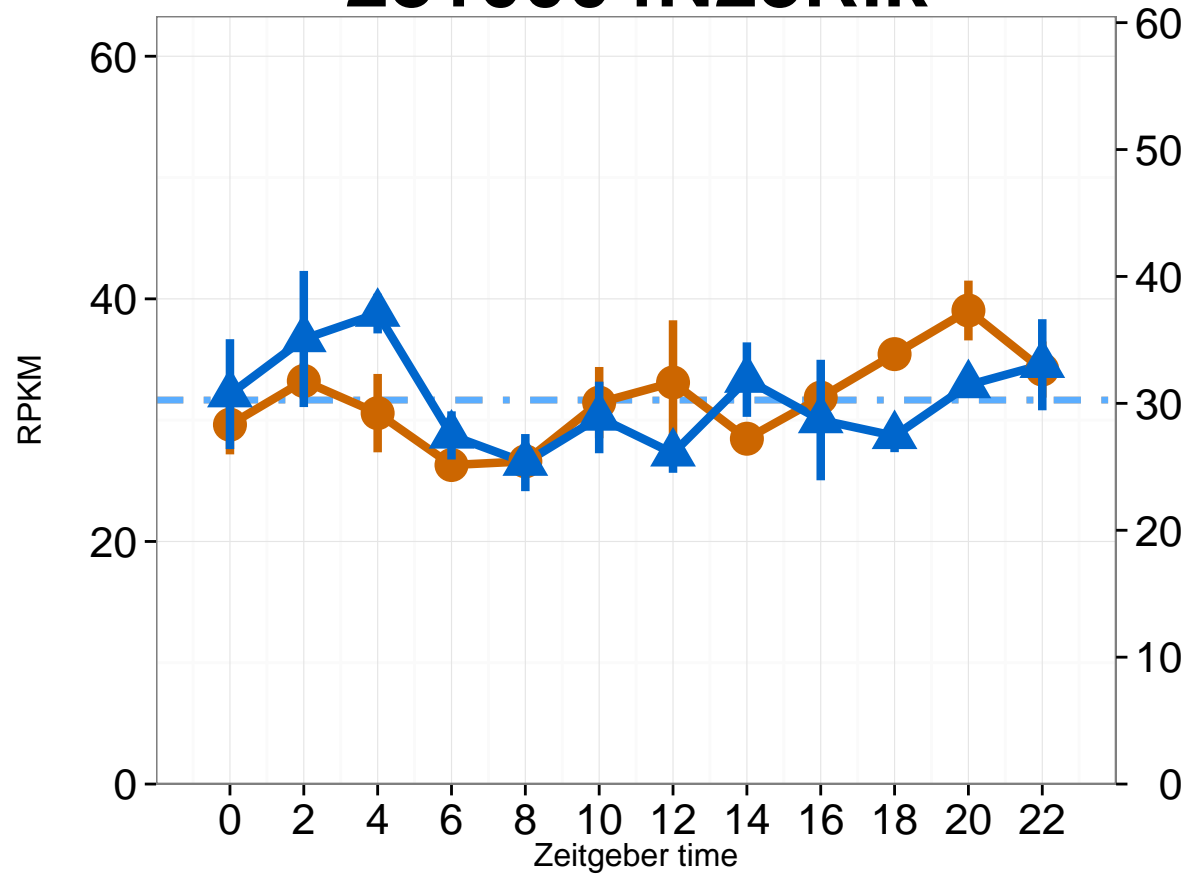

# 2810004N23Rik

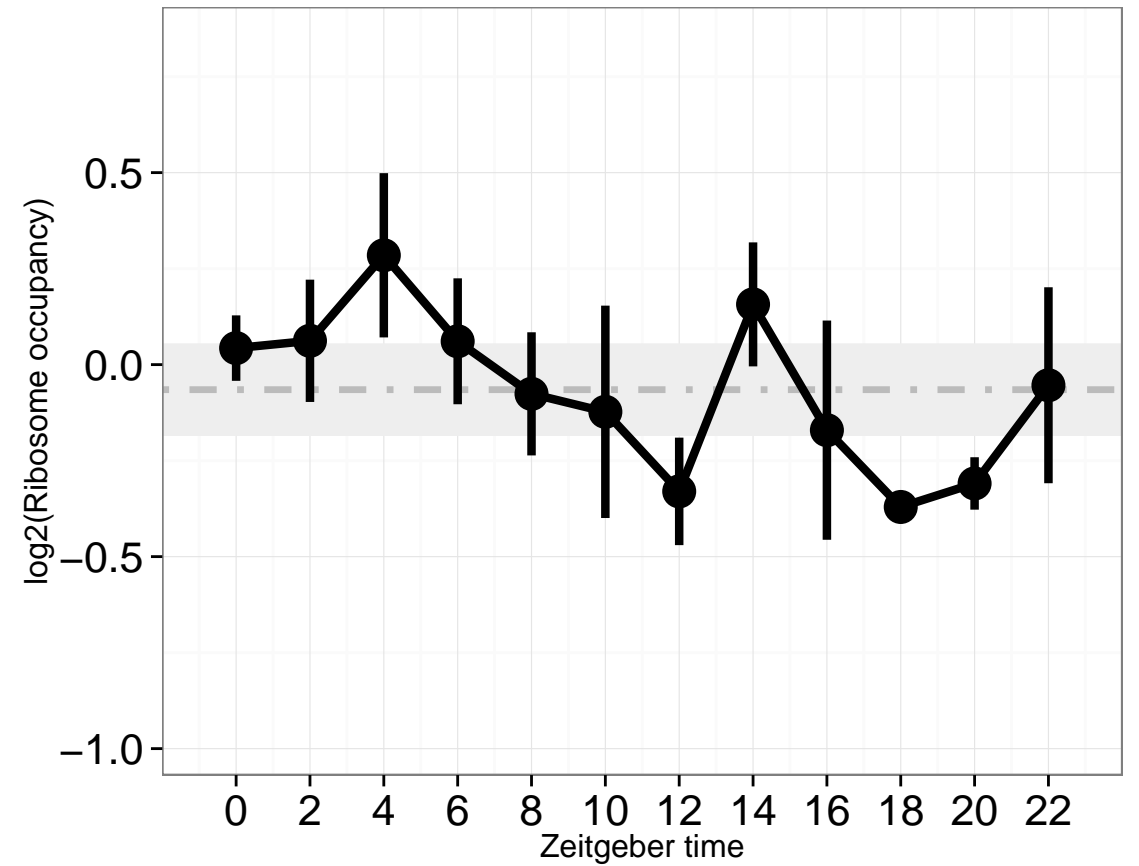

Supplement: Supplementary file 6 — Transcriptome-wide kidney RPF (blue) and RNA (orange) levels in the left panels (with “error bars” connecting the two replicates of each timepoint) and TE in the right panels. (ZIP 116896 kb) [file 13059_2017_1222_MOESM6_ESM.zip › Supp_Dataset_S1/A_RNA_non_rhythmic_RPF_non_rhythmic/2810004N23Rik_kidney_set_A.pdf]

# 2810021J22Rik

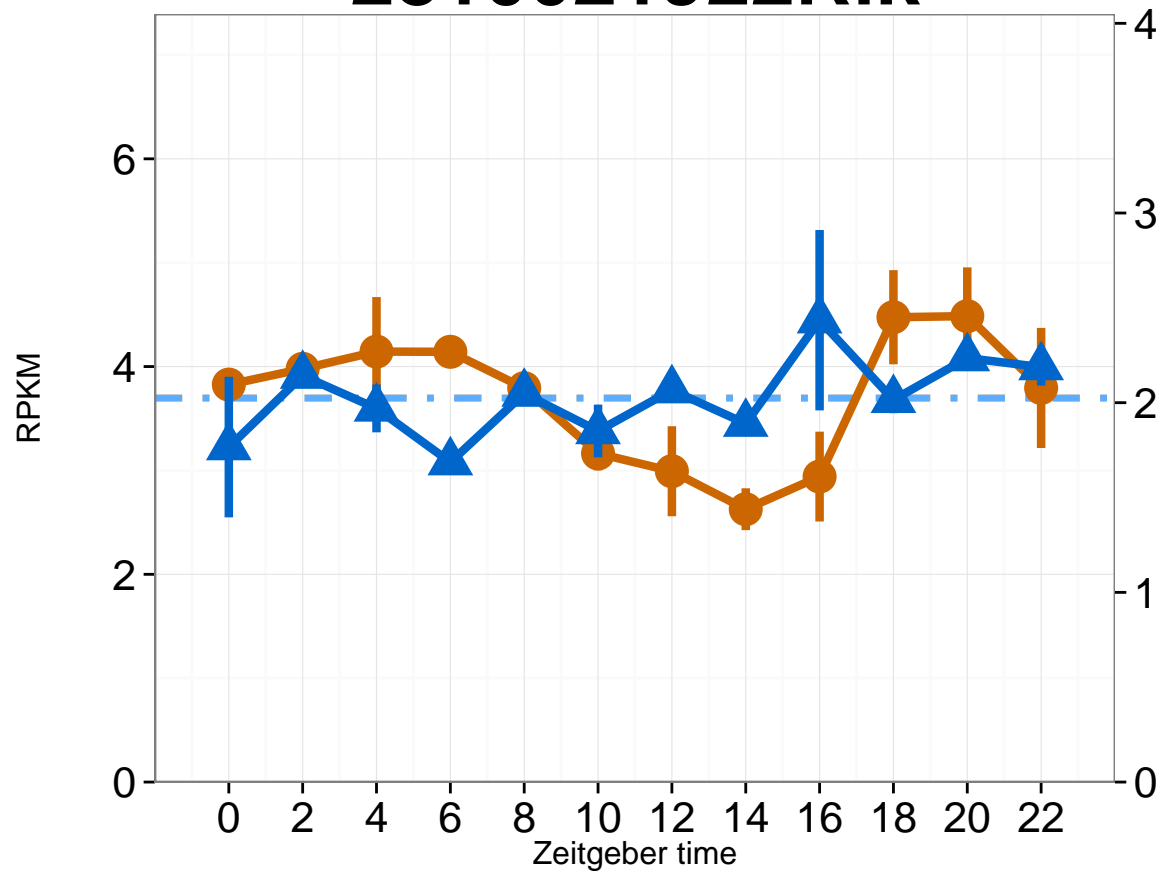

# 2810021J22Rik

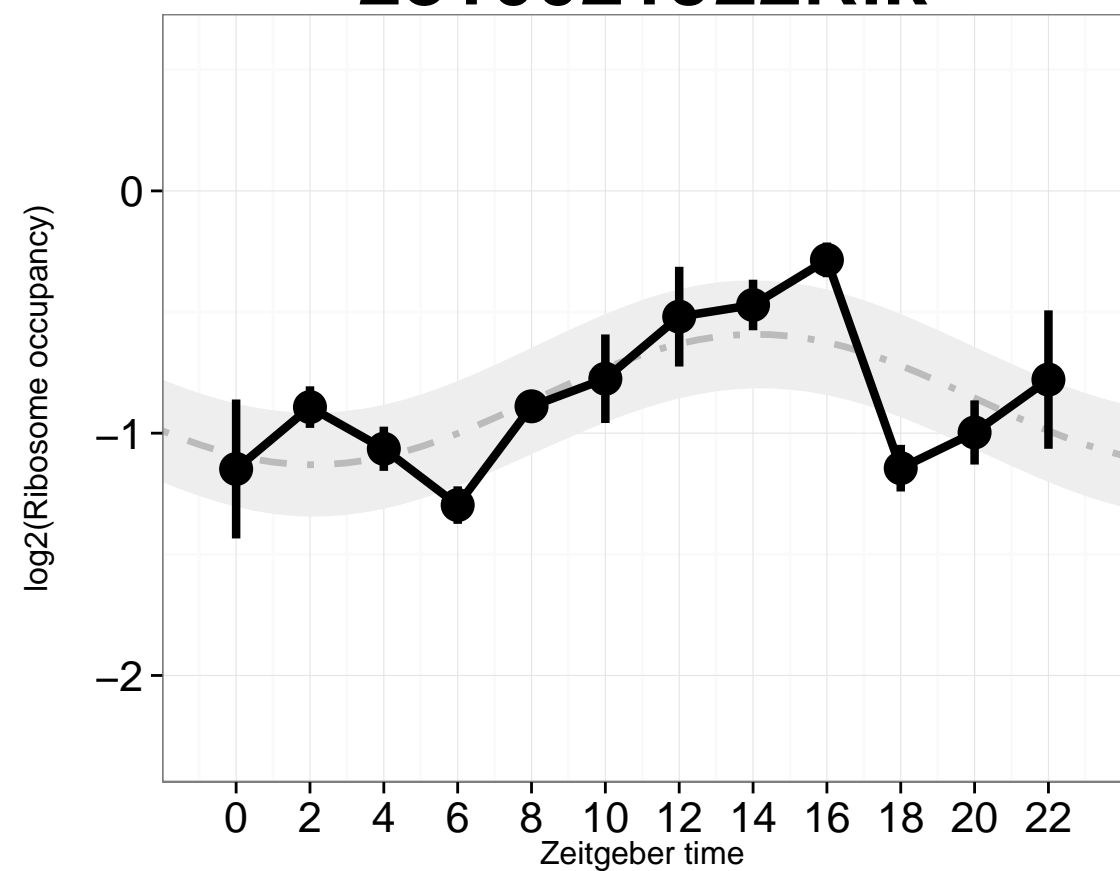

Supplement: Supplementary file 6 — Transcriptome-wide kidney RPF (blue) and RNA (orange) levels in the left panels (with “error bars” connecting the two replicates of each timepoint) and TE in the right panels. (ZIP 116896 kb) [file 13059_2017_1222_MOESM6_ESM.zip › Supp_Dataset_S1/A_RNA_non_rhythmic_RPF_non_rhythmic/2810021J22Rik_kidney_set_A.pdf]

# 2810025M15Rik

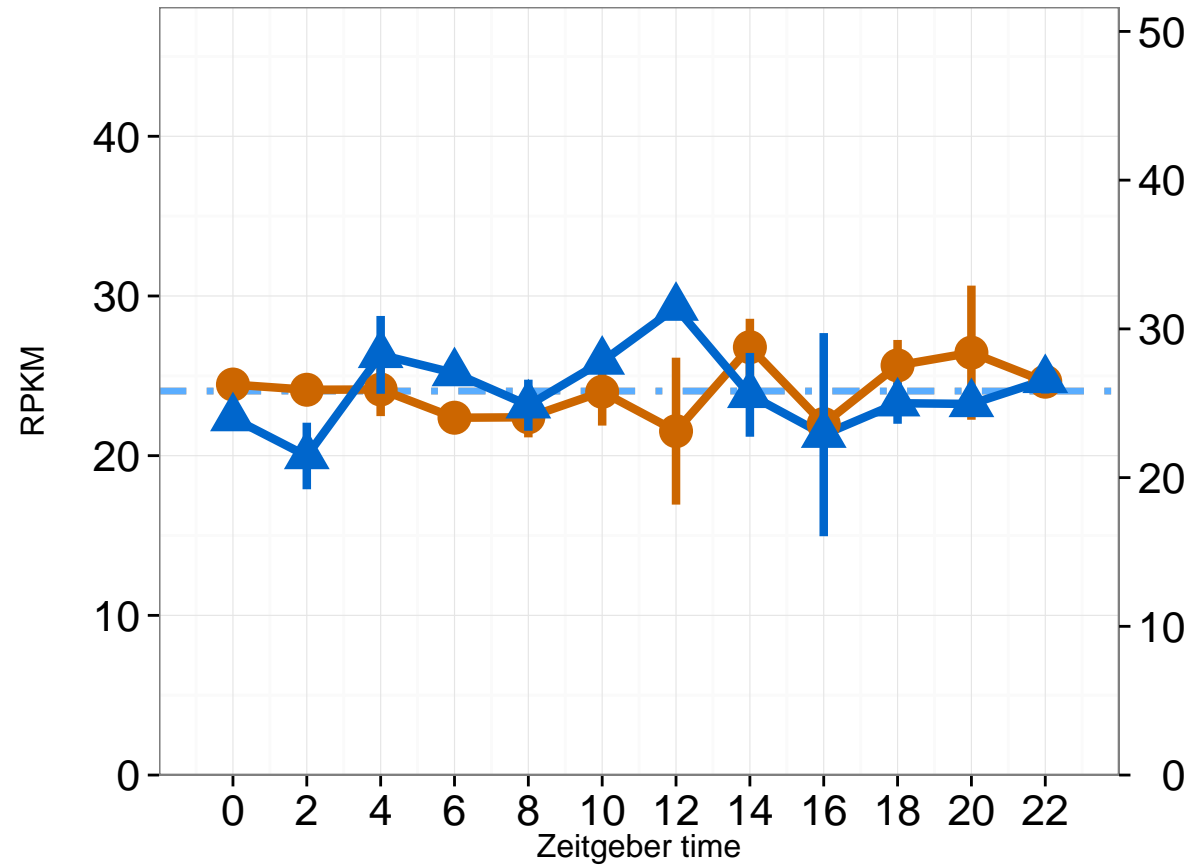

# 2810025M15Rik

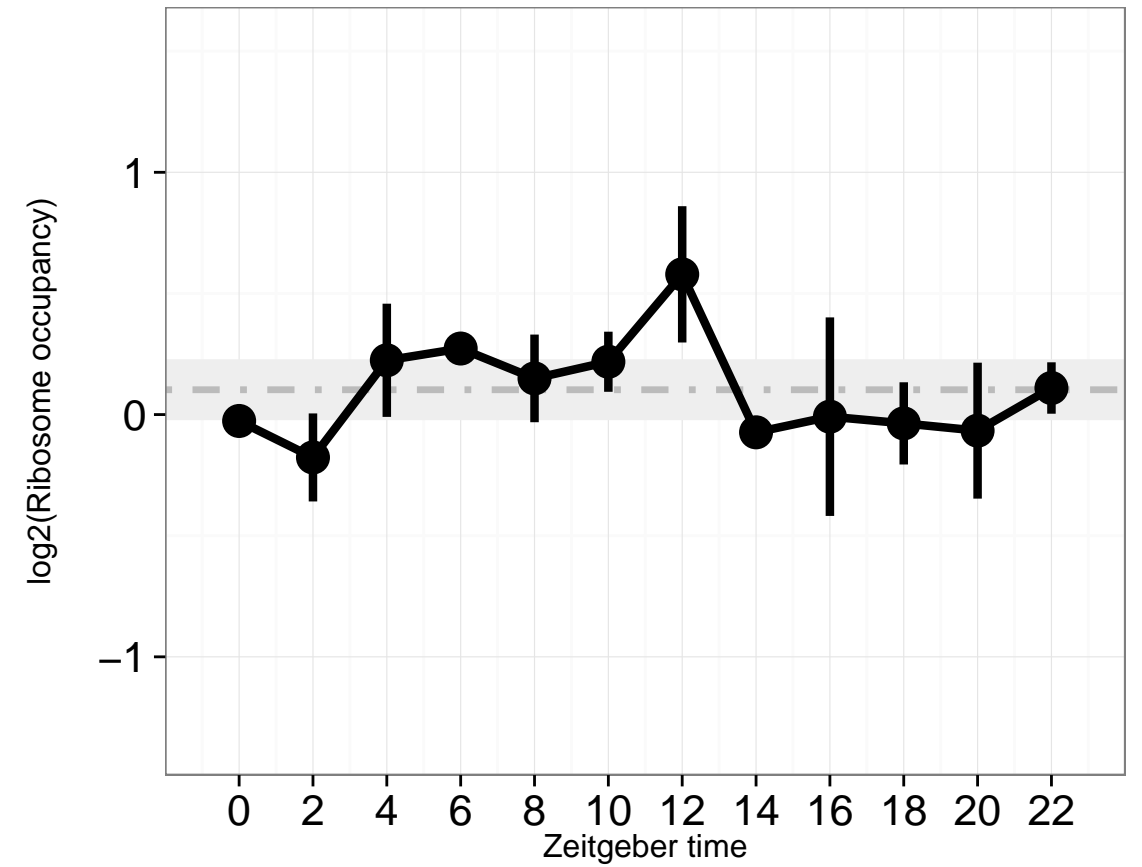

Supplement: Supplementary file 6 — Transcriptome-wide kidney RPF (blue) and RNA (orange) levels in the left panels (with “error bars” connecting the two replicates of each timepoint) and TE in the right panels. (ZIP 116896 kb) [file 13059_2017_1222_MOESM6_ESM.zip › Supp_Dataset_S1/A_RNA_non_rhythmic_RPF_non_rhythmic/2810025M15Rik_kidney_set_A.pdf]

# 2810403A07Rik

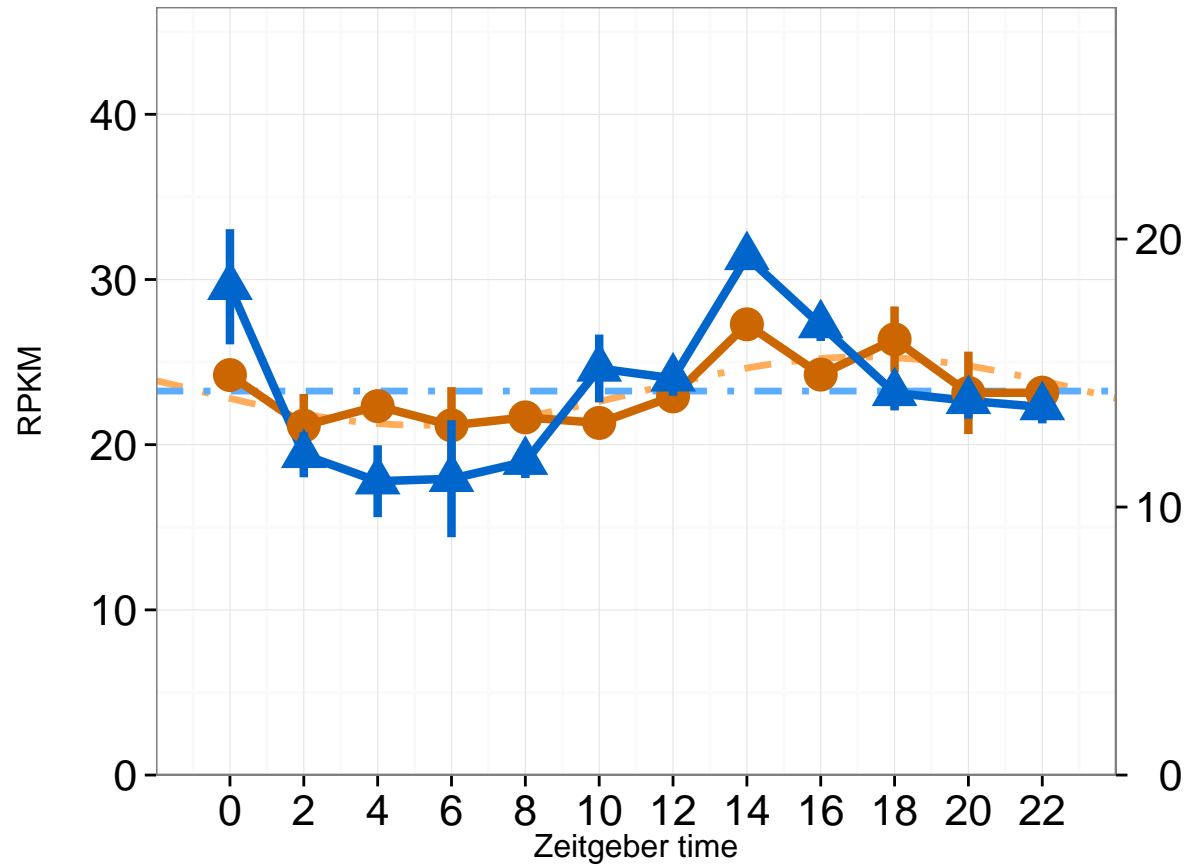

# 2810403A07Rik

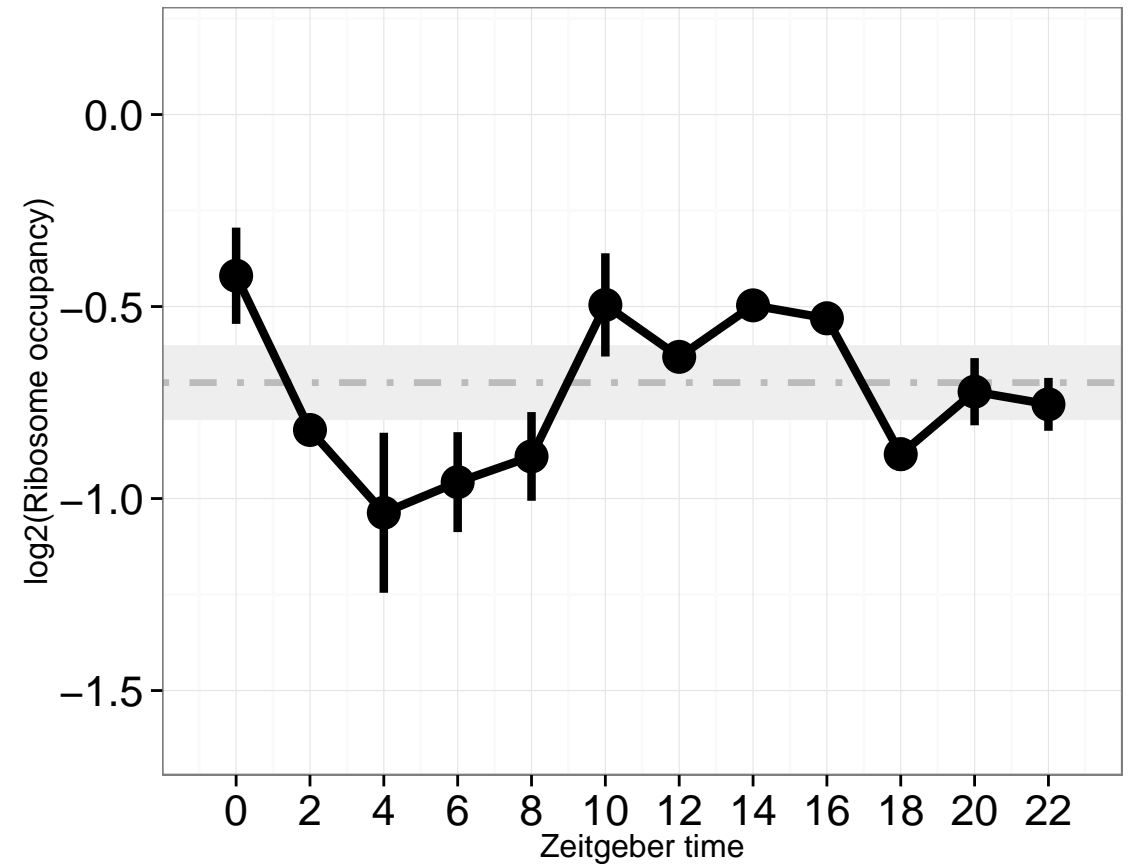

Supplement: Supplementary file 6 — Transcriptome-wide kidney RPF (blue) and RNA (orange) levels in the left panels (with “error bars” connecting the two replicates of each timepoint) and TE in the right panels. (ZIP 116896 kb) [file 13059_2017_1222_MOESM6_ESM.zip › Supp_Dataset_S1/A_RNA_non_rhythmic_RPF_non_rhythmic/2810403A07Rik_kidney_set_A.pdf]

## 2810408A11Rik

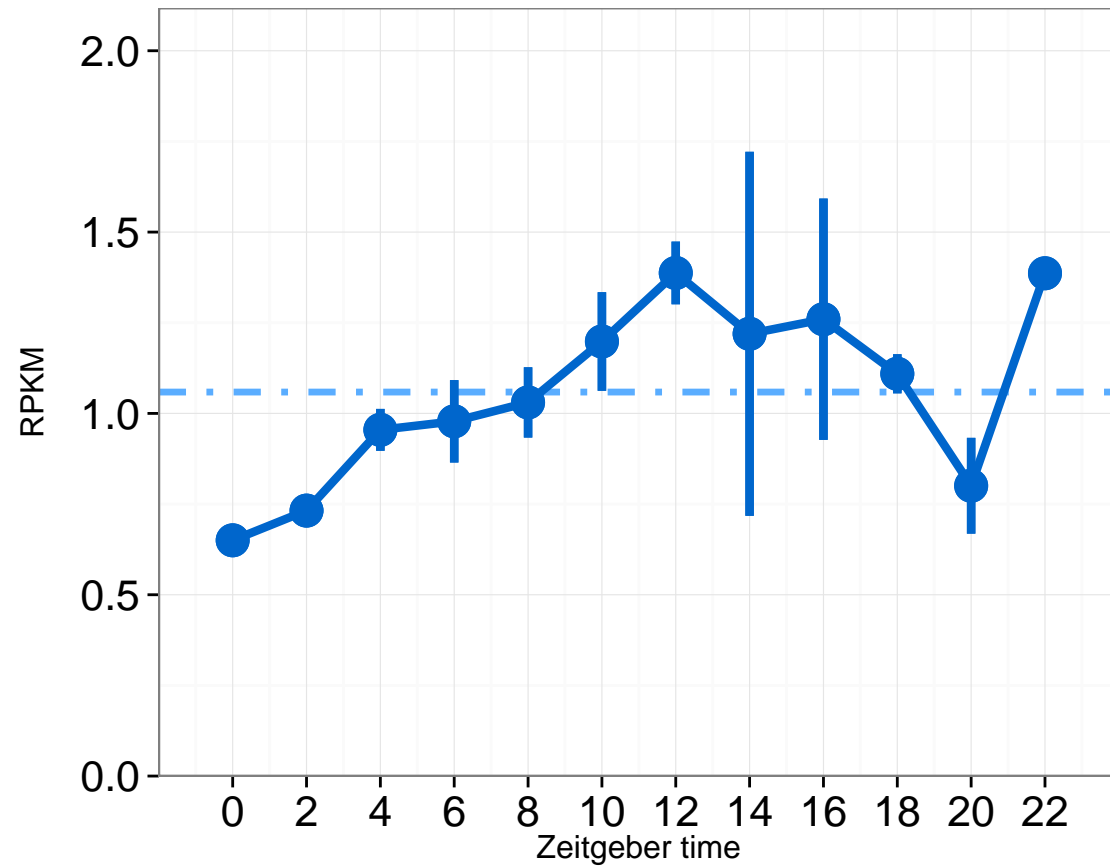

## 2810408A11Rik log2(Ribosome occup

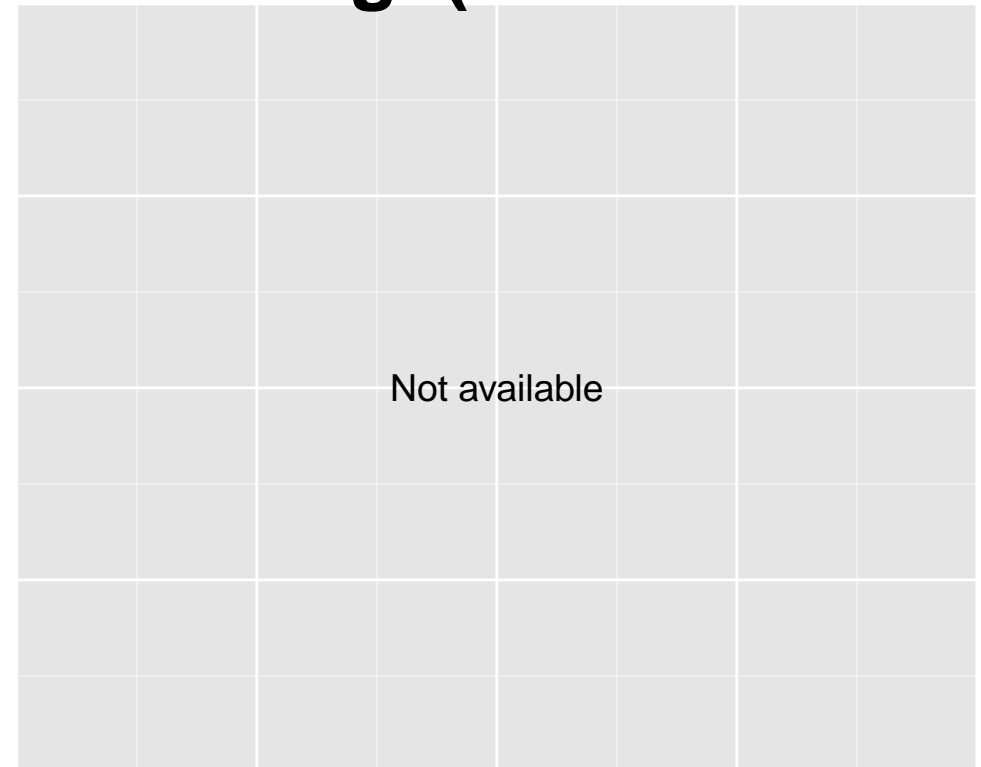

Supplement: Supplementary file 6 — Transcriptome-wide kidney RPF (blue) and RNA (orange) levels in the left panels (with “error bars” connecting the two replicates of each timepoint) and TE in the right panels. (ZIP 116896 kb) [file 13059_2017_1222_MOESM6_ESM.zip › Supp_Dataset_S1/A_RNA_non_rhythmic_RPF_non_rhythmic/2810408A11Rik_kidney_set_A.pdf]

# 2810408M09Rik

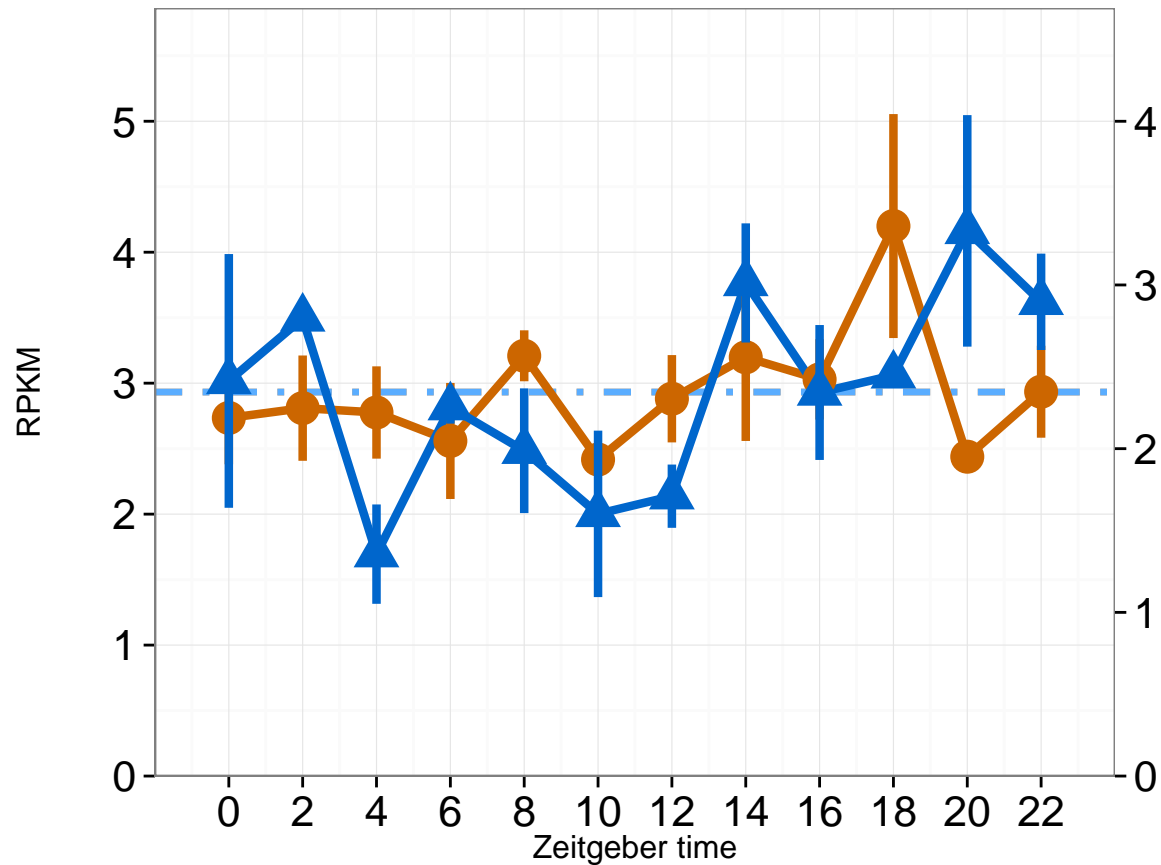

# 2810408M09Rik

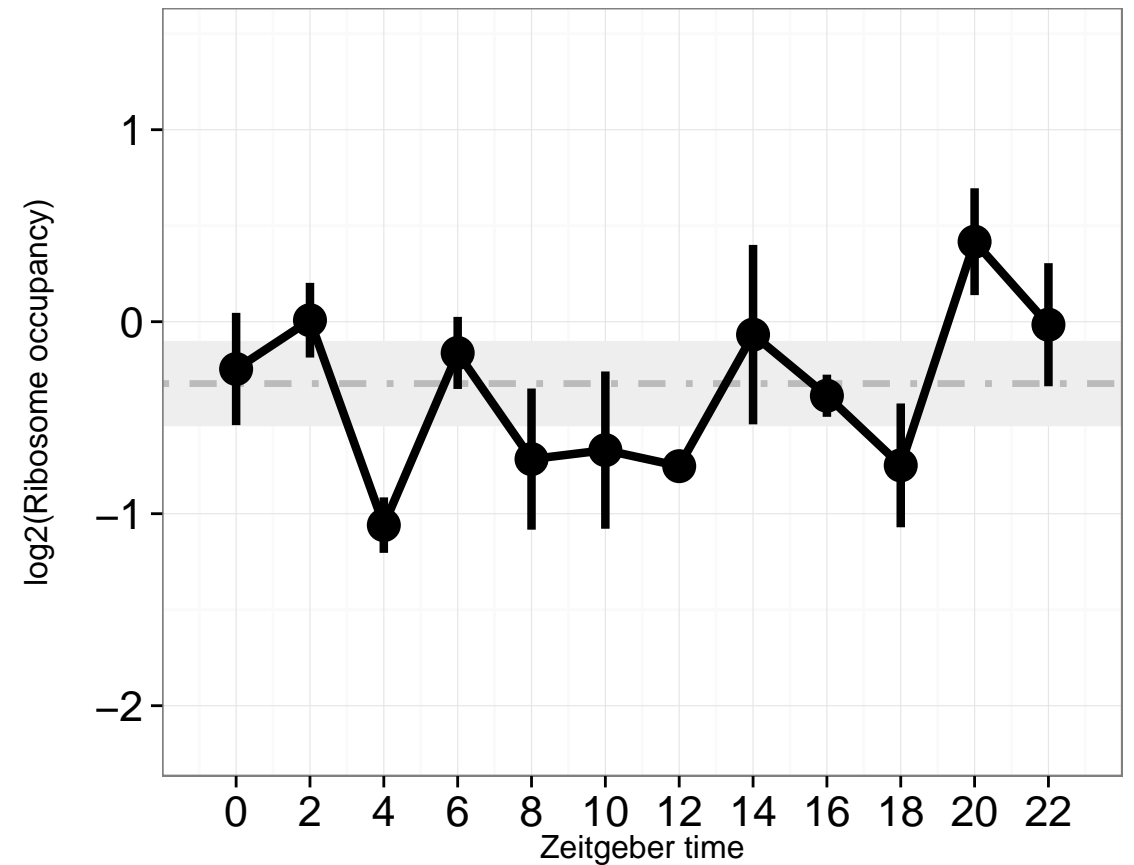

Supplement: Supplementary file 6 — Transcriptome-wide kidney RPF (blue) and RNA (orange) levels in the left panels (with “error bars” connecting the two replicates of each timepoint) and TE in the right panels. (ZIP 116896 kb) [file 13059_2017_1222_MOESM6_ESM.zip › Supp_Dataset_S1/A_RNA_non_rhythmic_RPF_non_rhythmic/2810408M09Rik_kidney_set_A.pdf]

2810417H13Rik TR

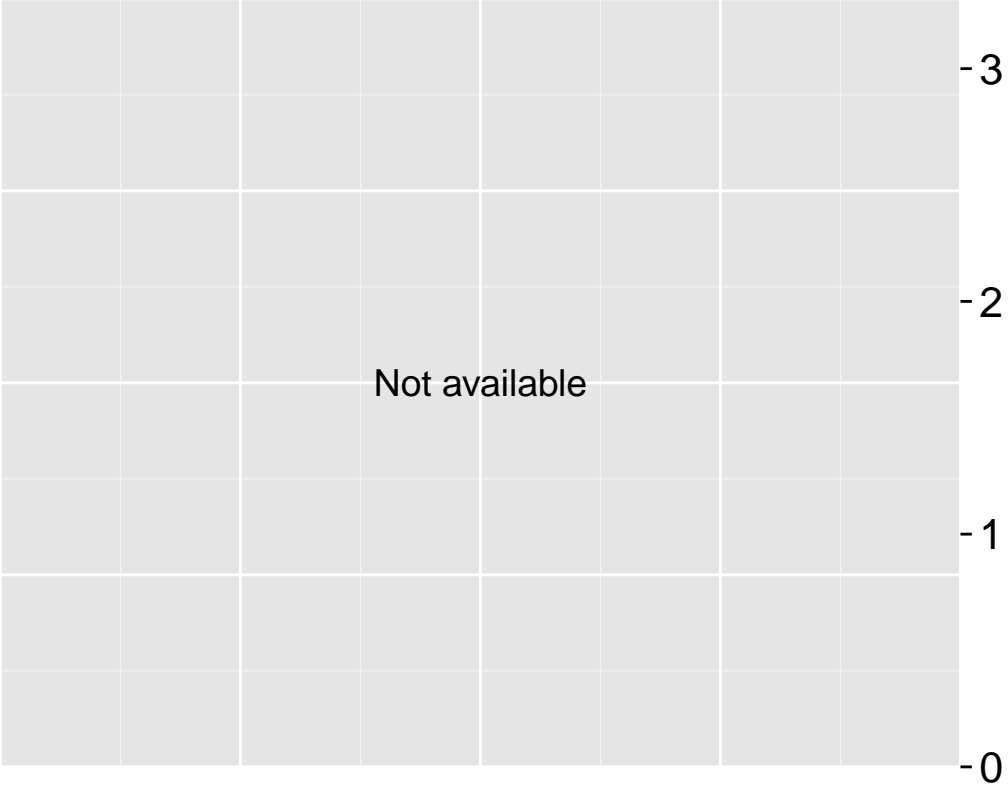

2810417H13Rik log2(Ribosome occup

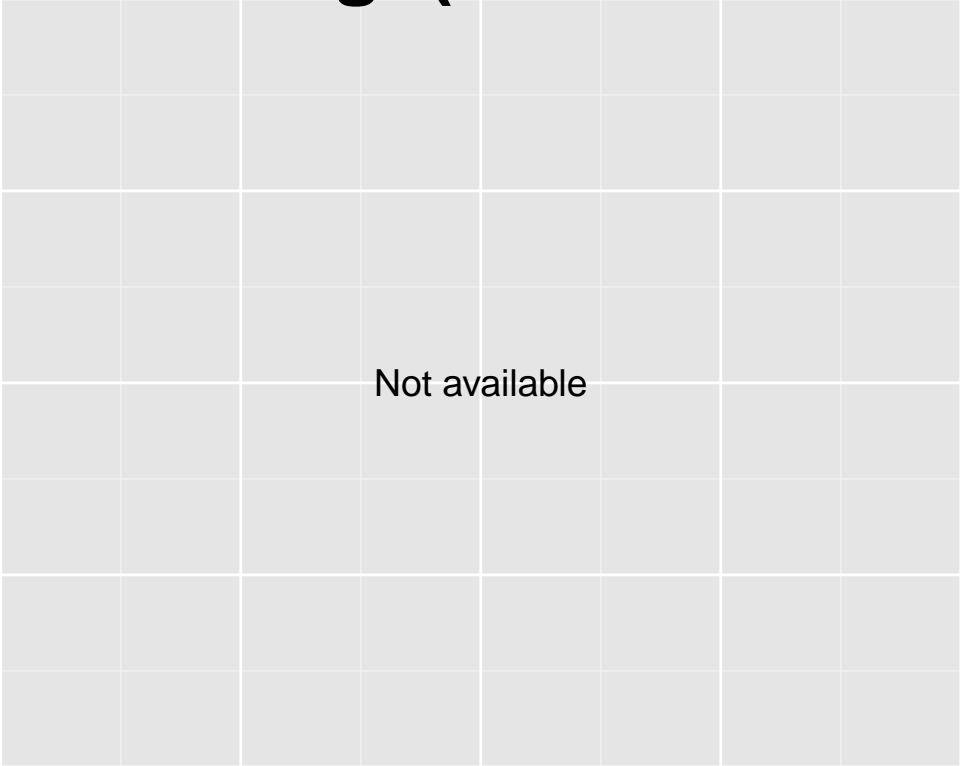

Supplement: Supplementary file 6 — Transcriptome-wide kidney RPF (blue) and RNA (orange) levels in the left panels (with “error bars” connecting the two replicates of each timepoint) and TE in the right panels. (ZIP 116896 kb) [file 13059_2017_1222_MOESM6_ESM.zip › Supp_Dataset_S1/A_RNA_non_rhythmic_RPF_non_rhythmic/2810417H13Rik_kidney_set_A.pdf]

## 2810428I15Rik

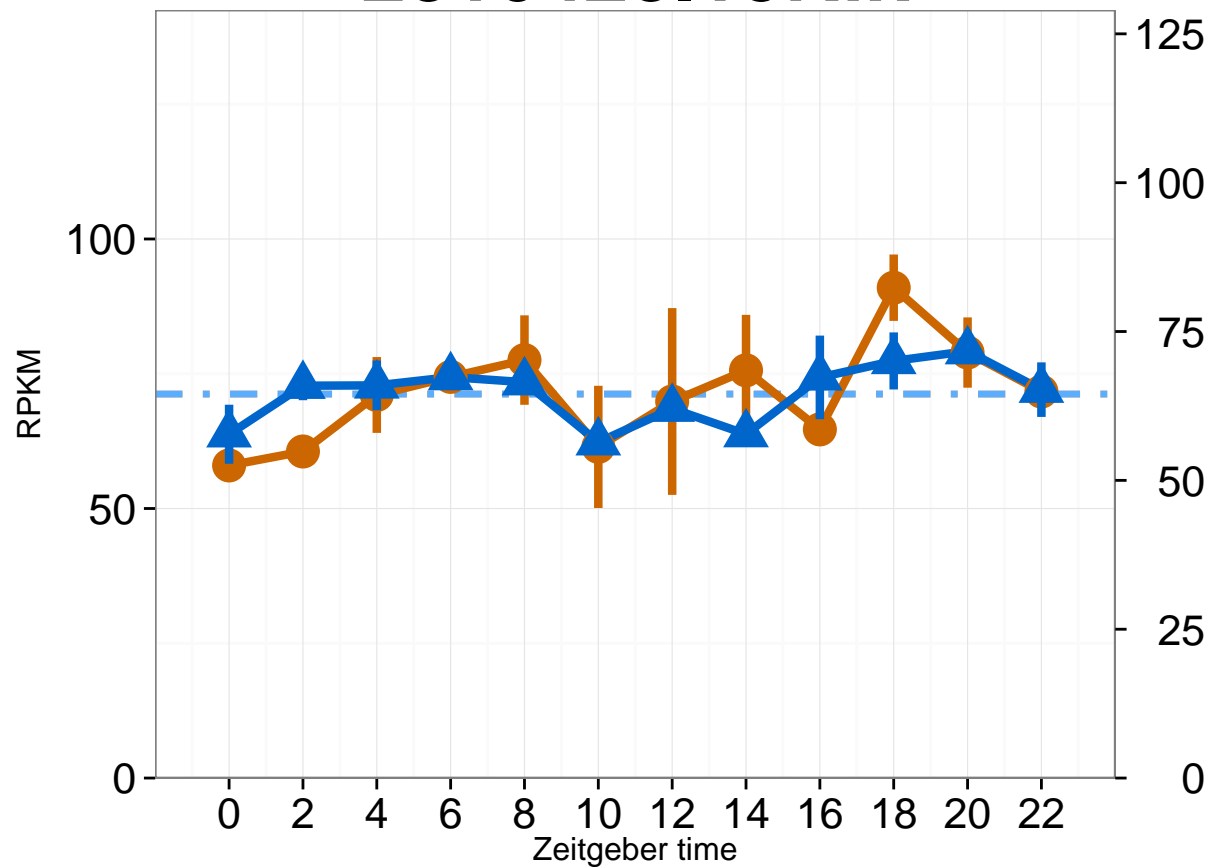

## 2810428I15Rik

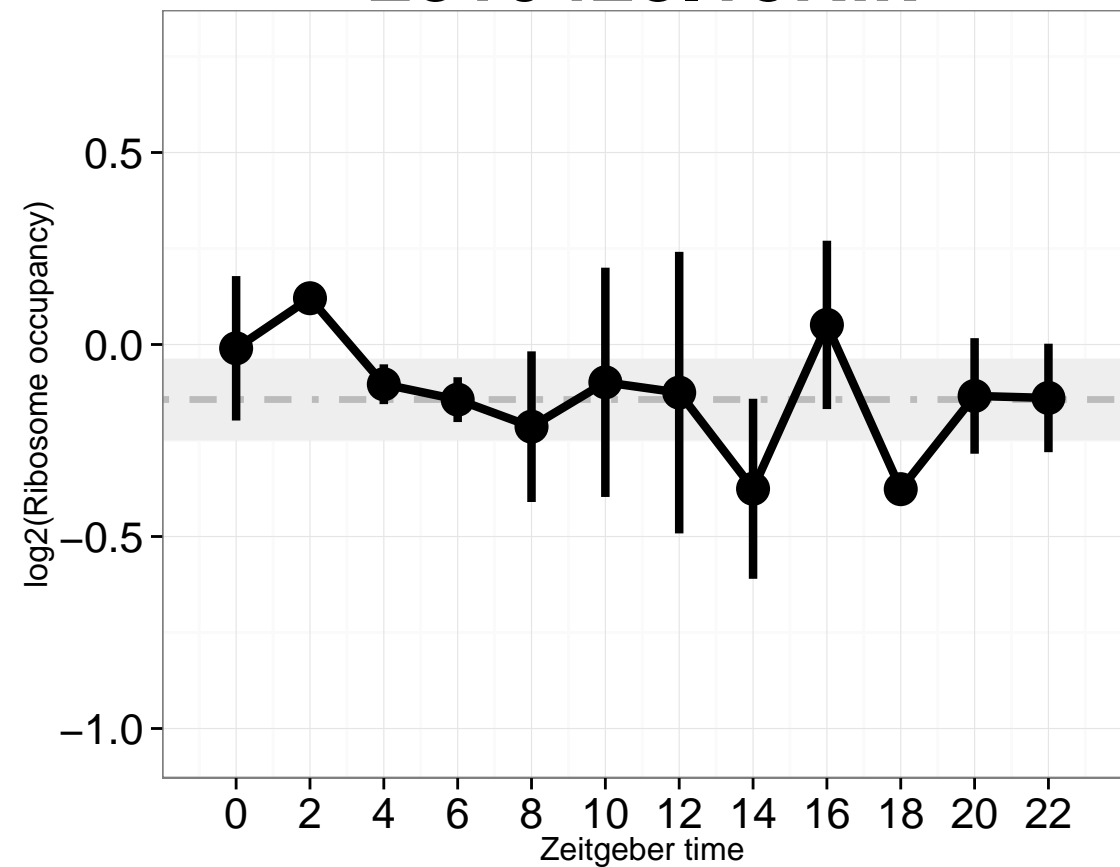

Supplement: Supplementary file 6 — Transcriptome-wide kidney RPF (blue) and RNA (orange) levels in the left panels (with “error bars” connecting the two replicates of each timepoint) and TE in the right panels. (ZIP 116896 kb) [file 13059_2017_1222_MOESM6_ESM.zip › Supp_Dataset_S1/A_RNA_non_rhythmic_RPF_non_rhythmic/2810428I15Rik_kidney_set_A.pdf]

# 2810459M11Rik

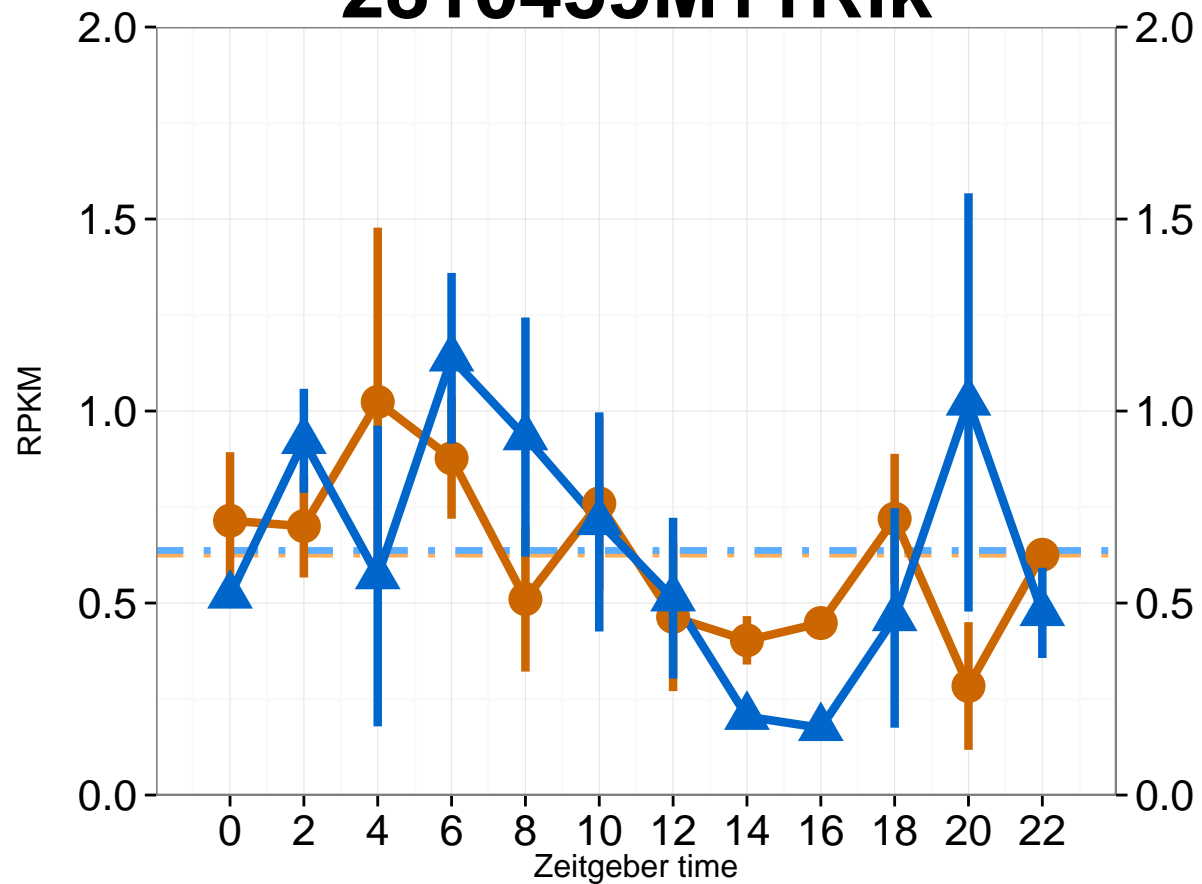

# 2810459M11Rik

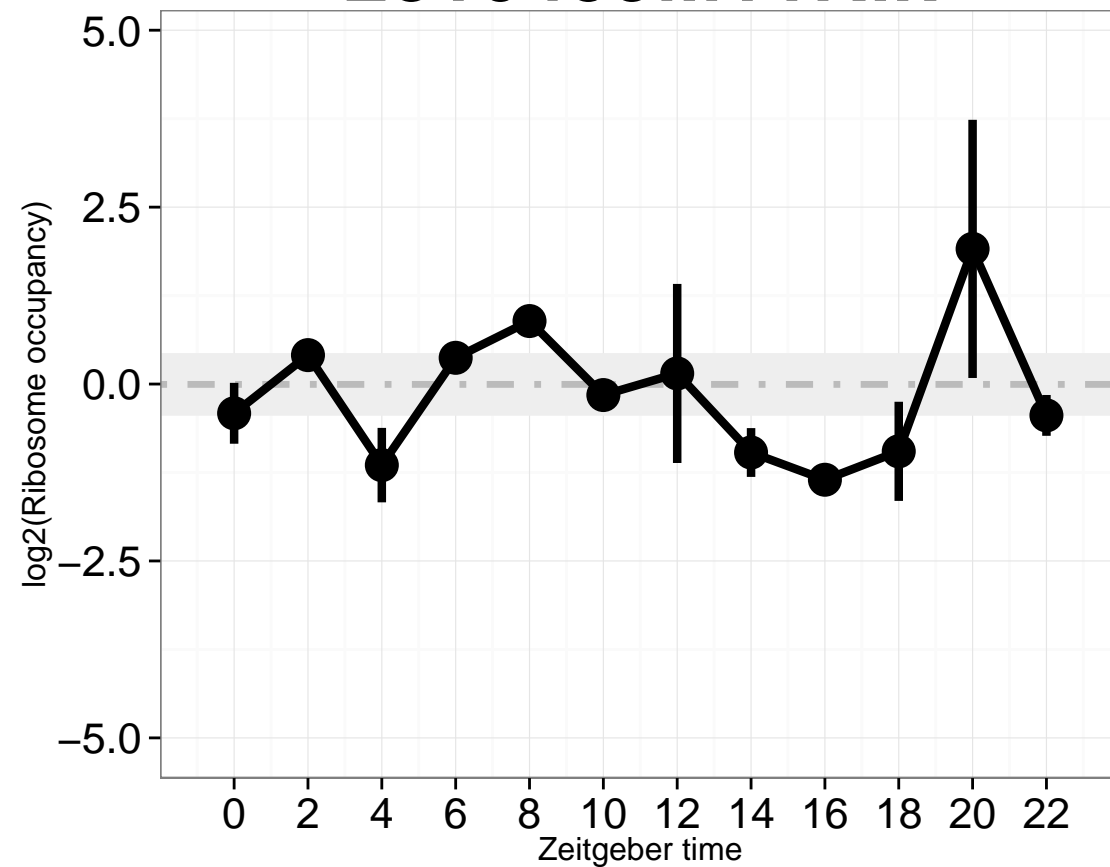

Supplement: Supplementary file 6 — Transcriptome-wide kidney RPF (blue) and RNA (orange) levels in the left panels (with “error bars” connecting the two replicates of each timepoint) and TE in the right panels. (ZIP 116896 kb) [file 13059_2017_1222_MOESM6_ESM.zip › Supp_Dataset_S1/A_RNA_non_rhythmic_RPF_non_rhythmic/2810459M11Rik_kidney_set_A.pdf]

**2810474O19Rik**

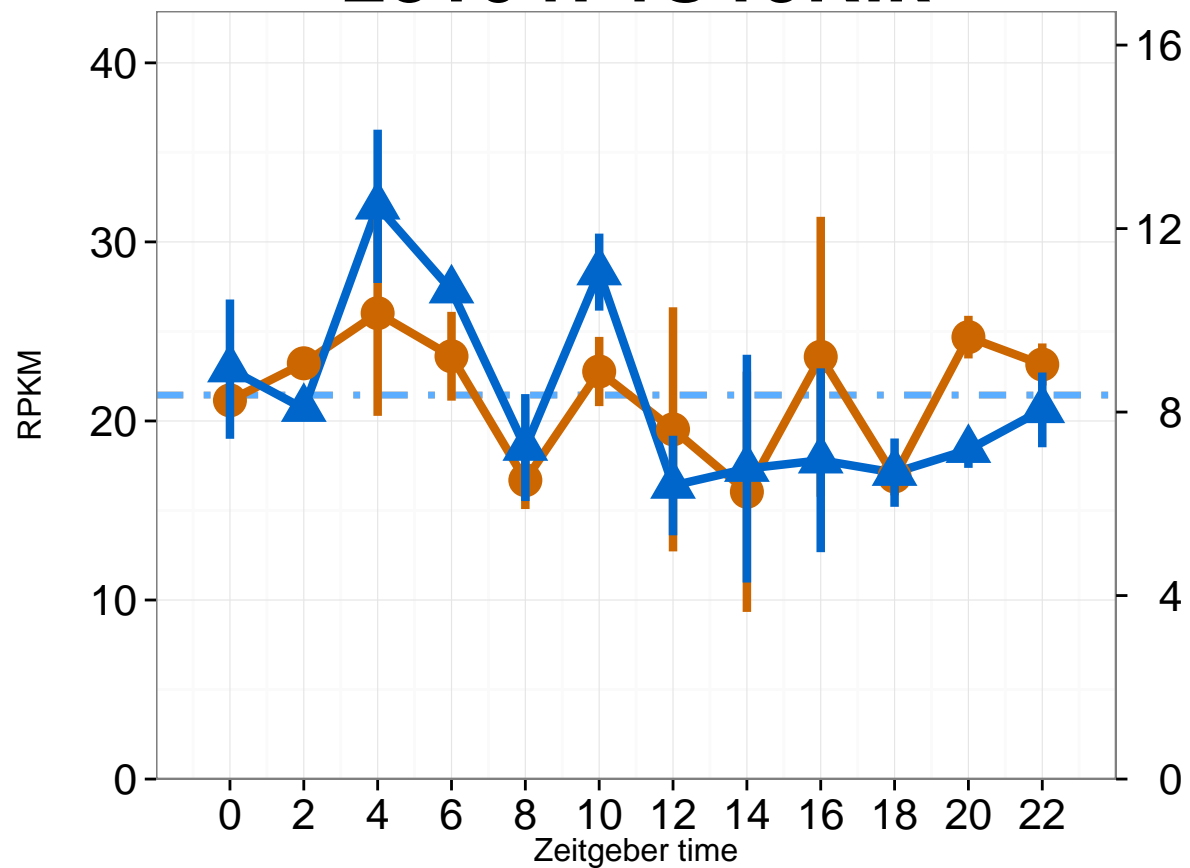

**2810474O19Rik**

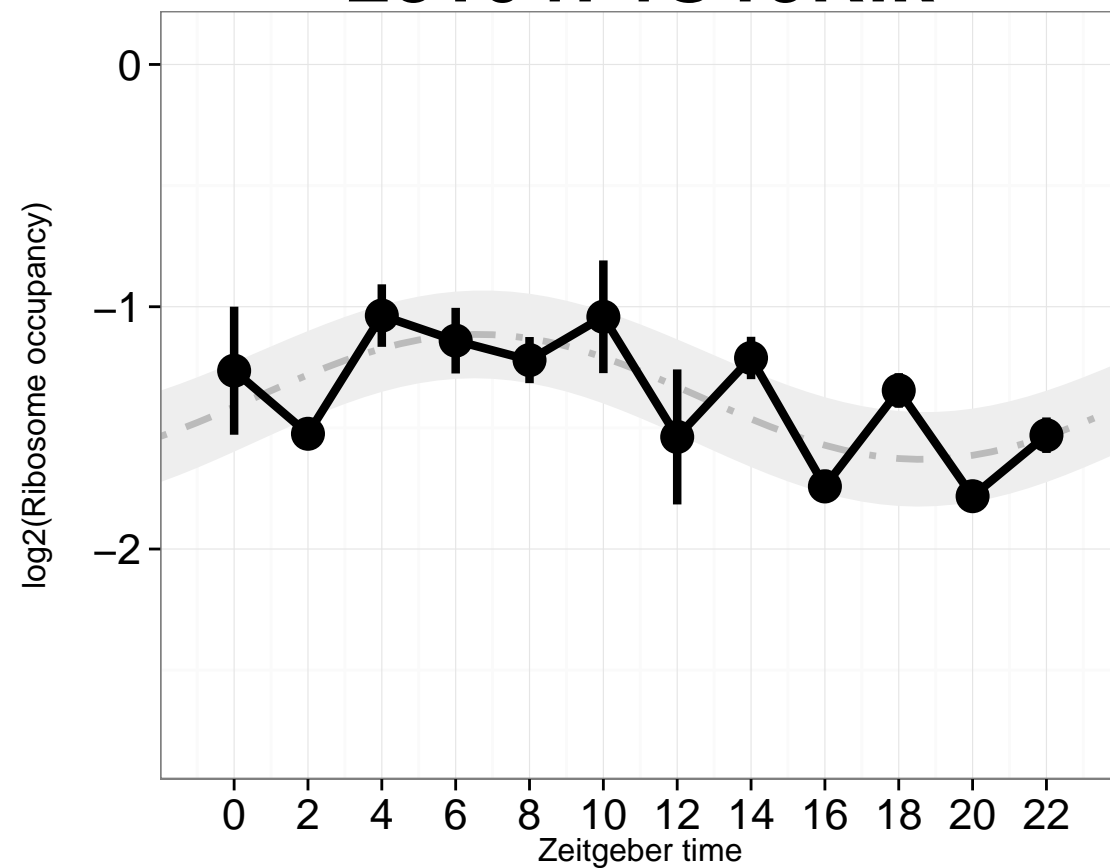

Supplement: Supplementary file 6 — Transcriptome-wide kidney RPF (blue) and RNA (orange) levels in the left panels (with “error bars” connecting the two replicates of each timepoint) and TE in the right panels. (ZIP 116896 kb) [file 13059_2017_1222_MOESM6_ESM.zip › Supp_Dataset_S1/A_RNA_non_rhythmic_RPF_non_rhythmic/2810474O19Rik_kidney_set_A.pdf]

# 2900026A02Rik

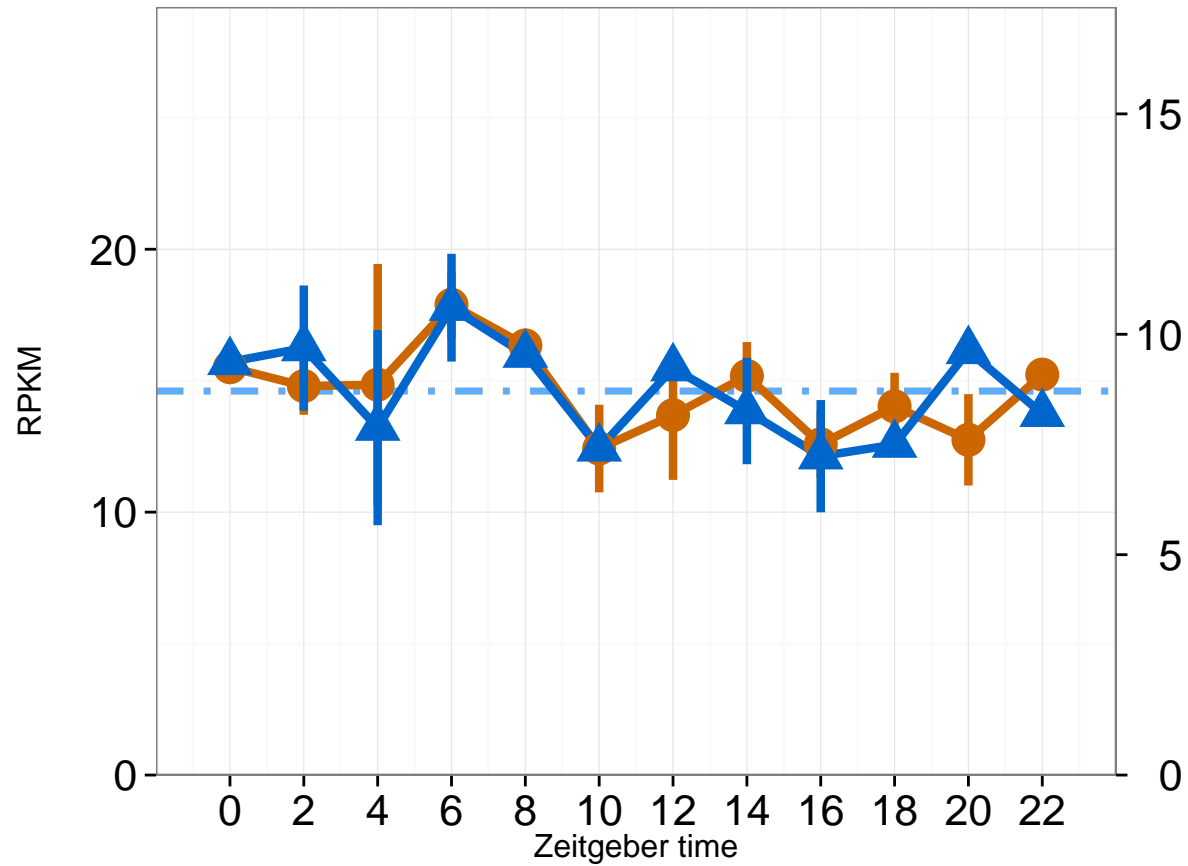

# 2900026A02Rik

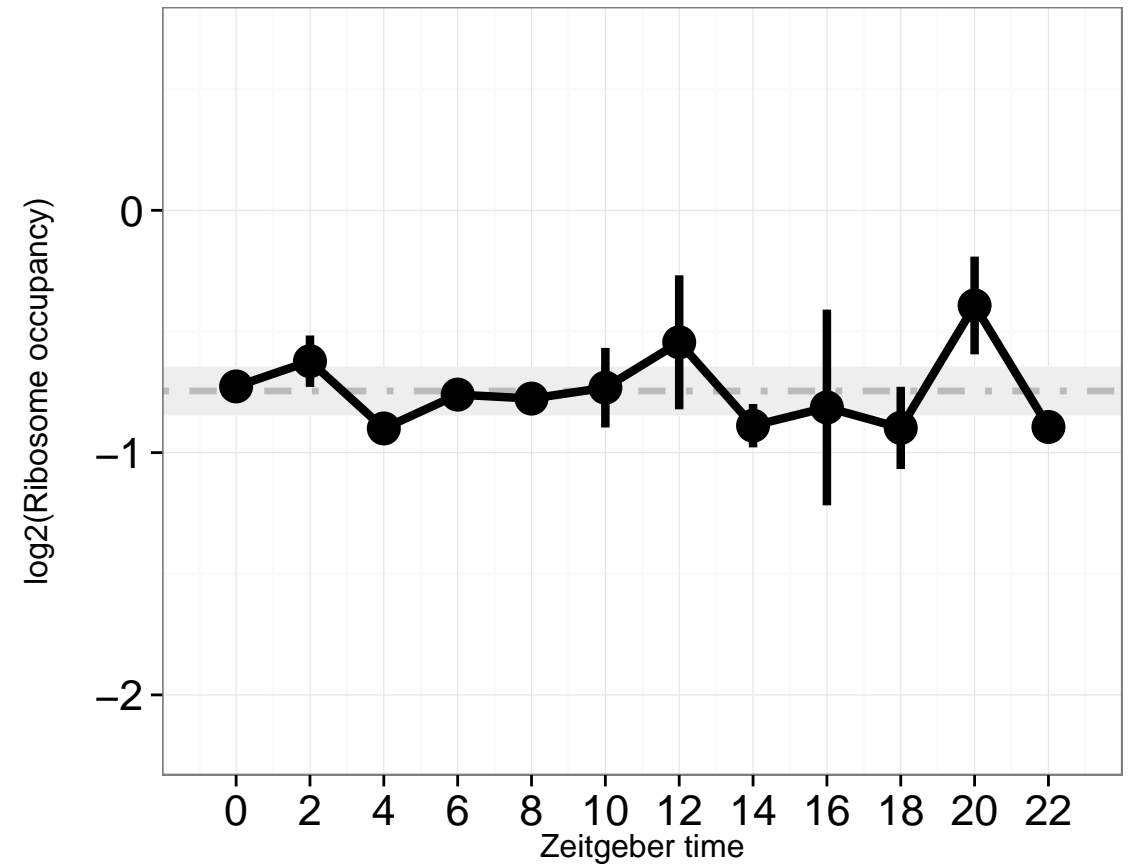

Supplement: Supplementary file 6 — Transcriptome-wide kidney RPF (blue) and RNA (orange) levels in the left panels (with “error bars” connecting the two replicates of each timepoint) and TE in the right panels. (ZIP 116896 kb) [file 13059_2017_1222_MOESM6_ESM.zip › Supp_Dataset_S1/A_RNA_non_rhythmic_RPF_non_rhythmic/2900026A02Rik_kidney_set_A.pdf]

# 3110001I22Rik

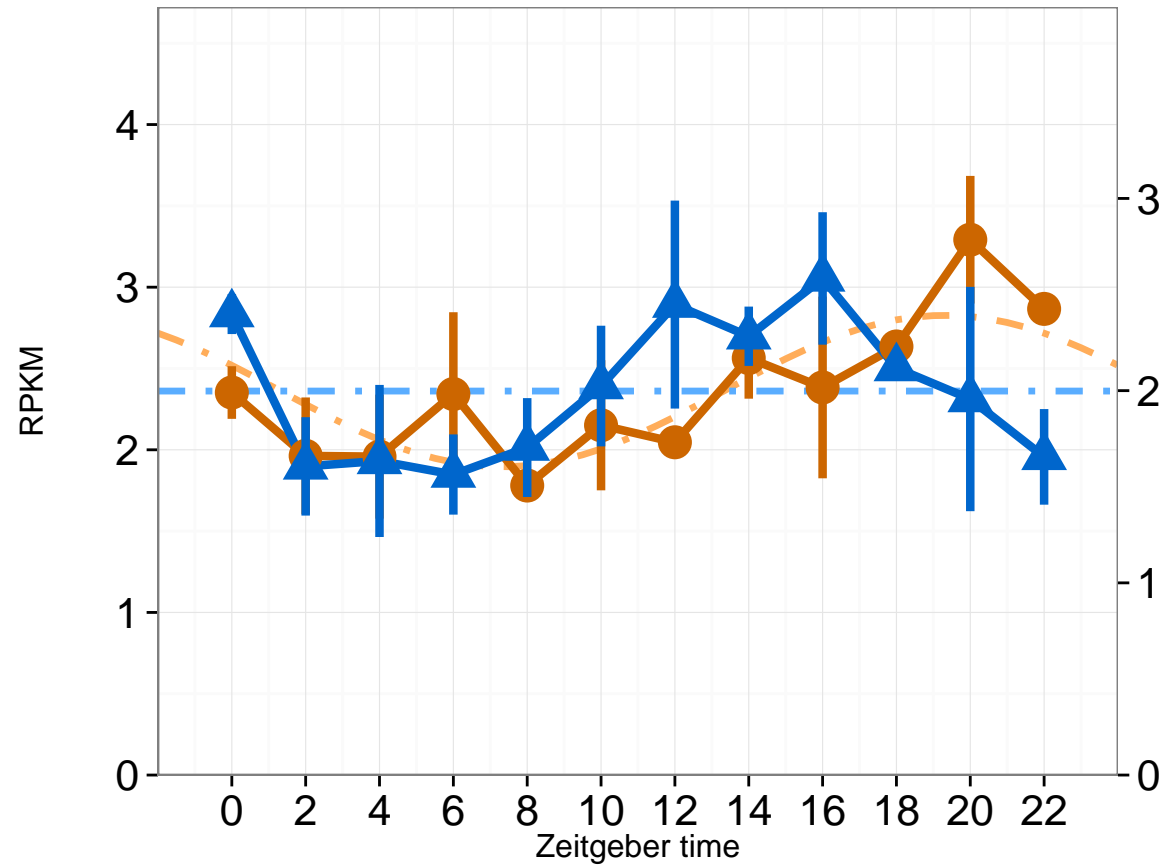

# 3110001I22Rik

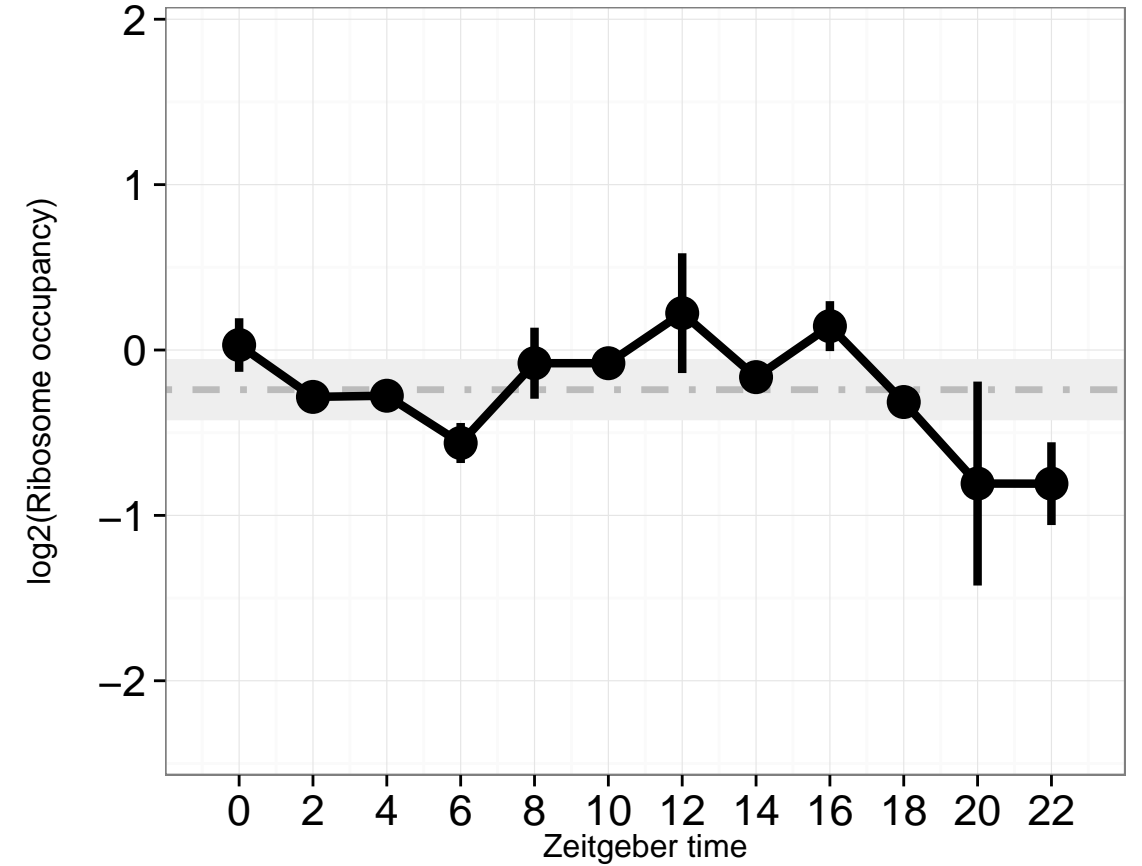

Supplement: Supplementary file 6 — Transcriptome-wide kidney RPF (blue) and RNA (orange) levels in the left panels (with “error bars” connecting the two replicates of each timepoint) and TE in the right panels. (ZIP 116896 kb) [file 13059_2017_1222_MOESM6_ESM.zip › Supp_Dataset_S1/A_RNA_non_rhythmic_RPF_non_rhythmic/3110001I22Rik_kidney_set_A.pdf]

# 3110002H16Rik

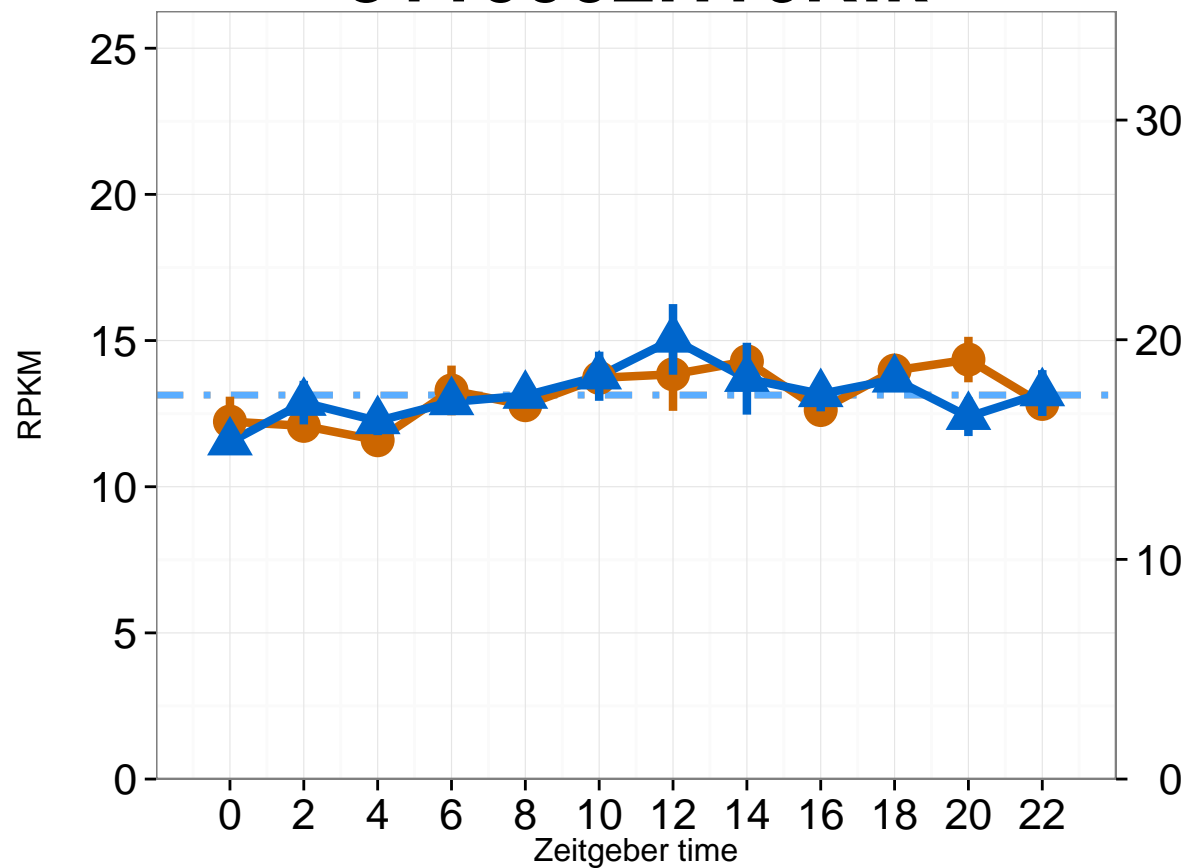

# 3110002H16Rik

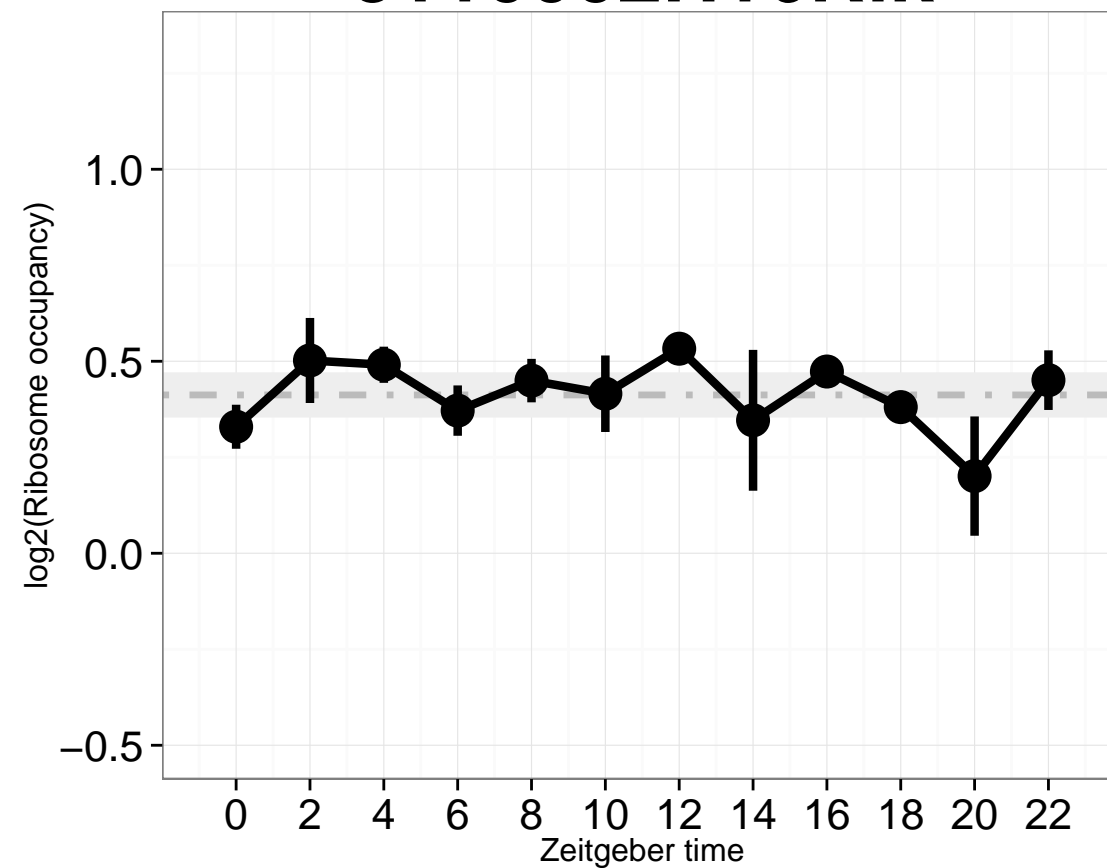

Supplement: Supplementary file 6 — Transcriptome-wide kidney RPF (blue) and RNA (orange) levels in the left panels (with “error bars” connecting the two replicates of each timepoint) and TE in the right panels. (ZIP 116896 kb) [file 13059_2017_1222_MOESM6_ESM.zip › Supp_Dataset_S1/A_RNA_non_rhythmic_RPF_non_rhythmic/3110002H16Rik_kidney_set_A.pdf]

# 3110009E18Rik

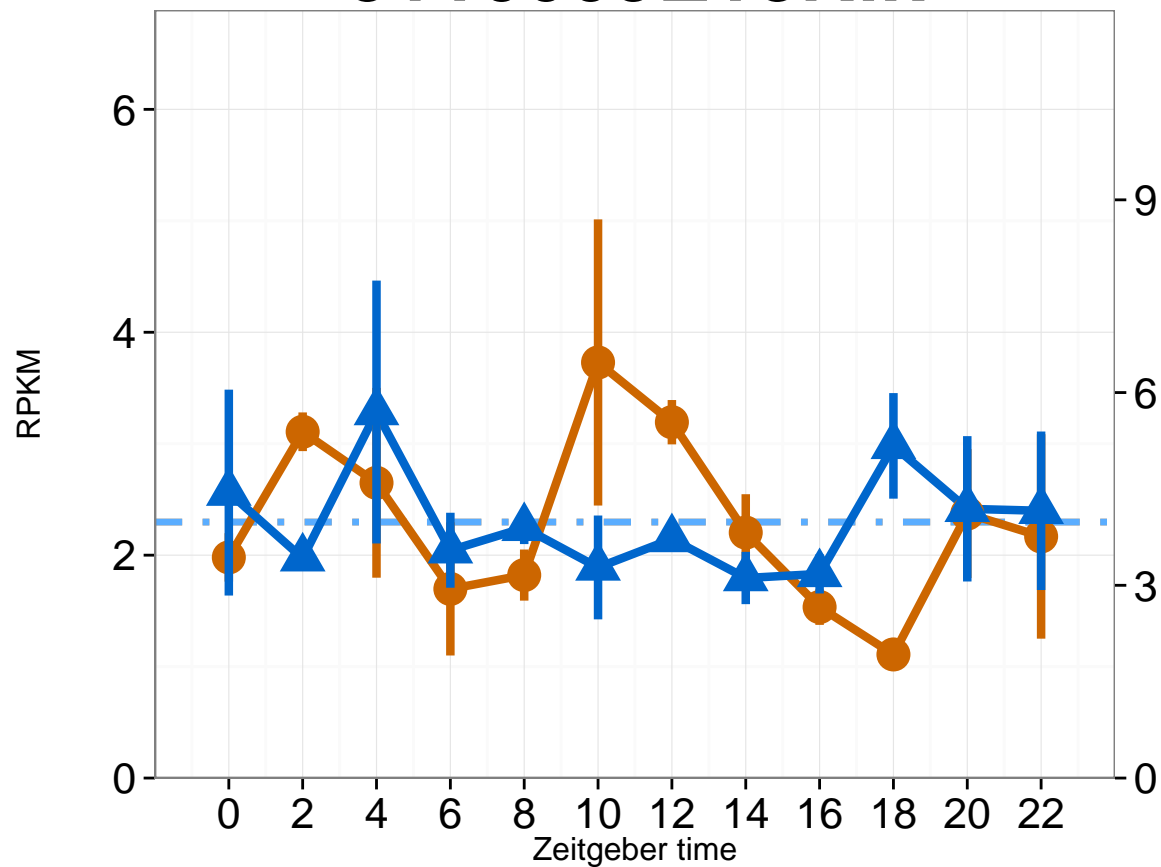

# 3110009E18Rik

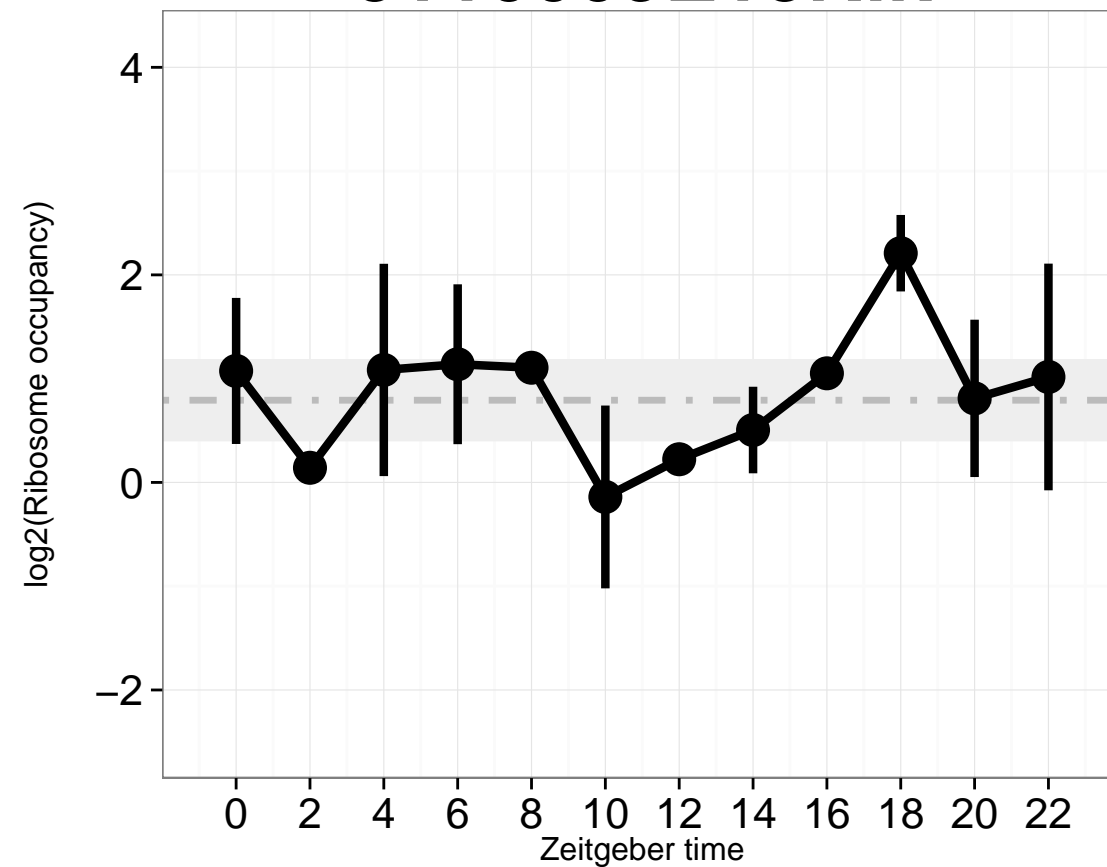

Supplement: Supplementary file 6 — Transcriptome-wide kidney RPF (blue) and RNA (orange) levels in the left panels (with “error bars” connecting the two replicates of each timepoint) and TE in the right panels. (ZIP 116896 kb) [file 13059_2017_1222_MOESM6_ESM.zip › Supp_Dataset_S1/A_RNA_non_rhythmic_RPF_non_rhythmic/3110009E18Rik_kidney_set_A.pdf]

## 3110040N11Rik

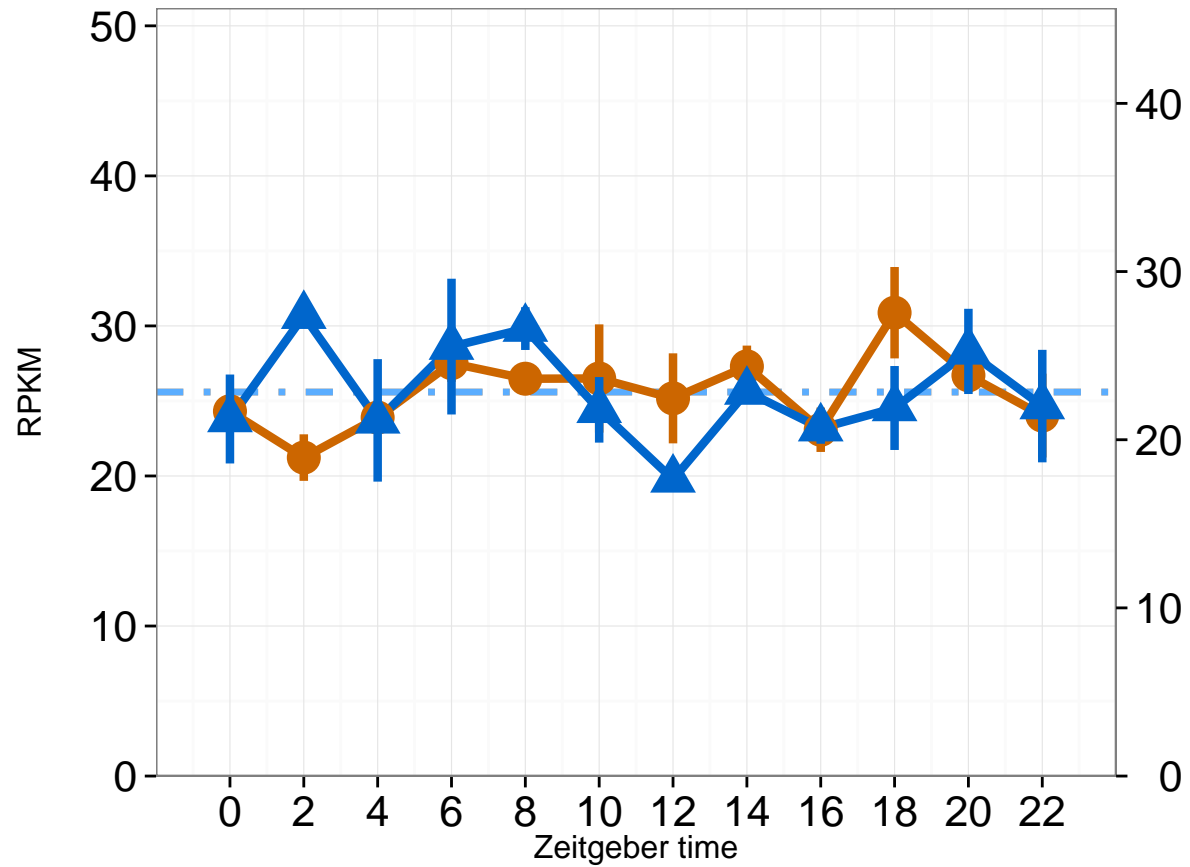

## 3110040N11Rik

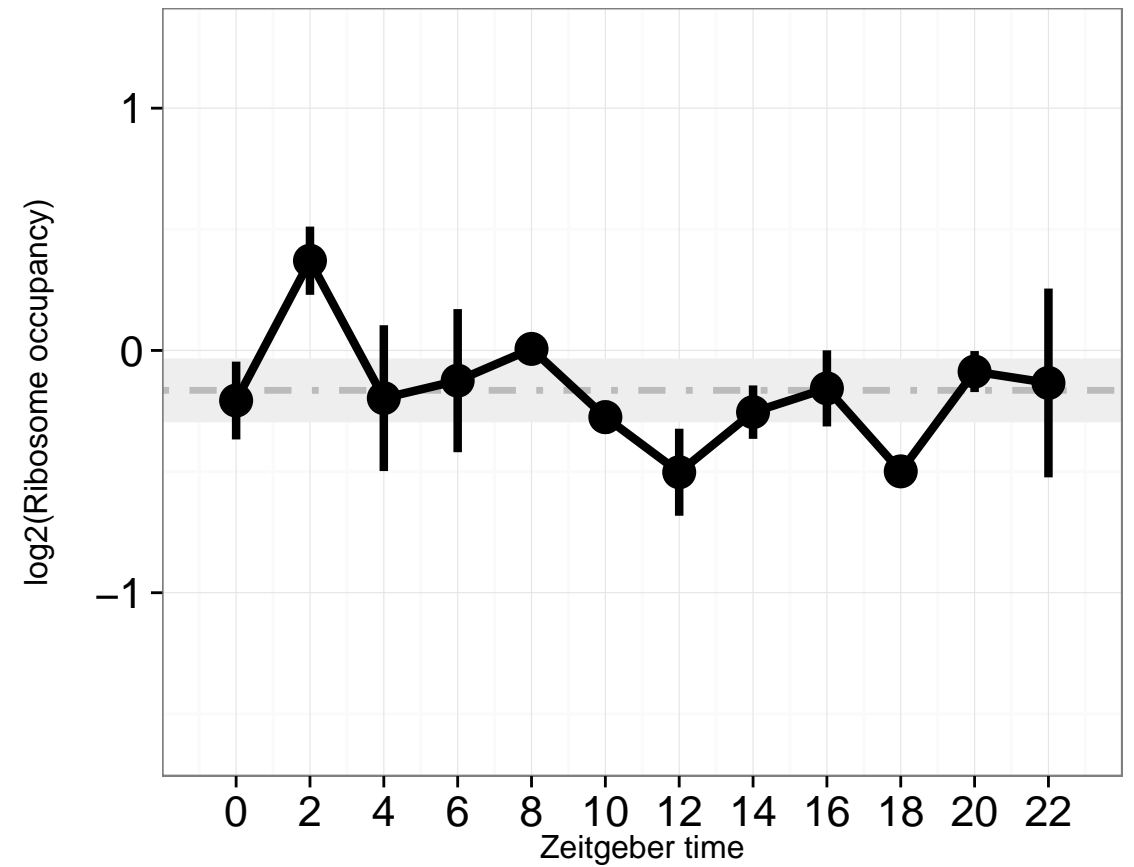

Supplement: Supplementary file 6 — Transcriptome-wide kidney RPF (blue) and RNA (orange) levels in the left panels (with “error bars” connecting the two replicates of each timepoint) and TE in the right panels. (ZIP 116896 kb) [file 13059_2017_1222_MOESM6_ESM.zip › Supp_Dataset_S1/A_RNA_non_rhythmic_RPF_non_rhythmic/3110040N11Rik_kidney_set_A.pdf]

# 3110043O21Rik

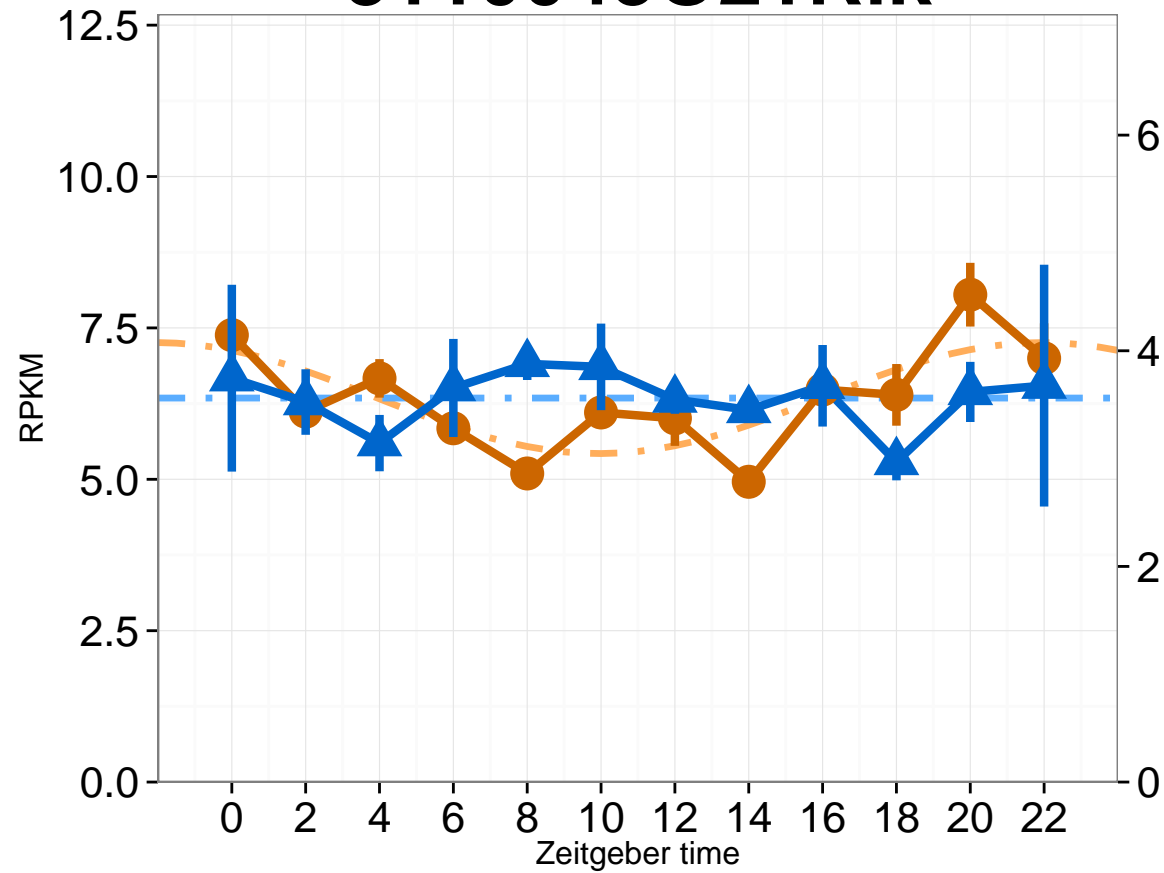

# 3110043O21Rik

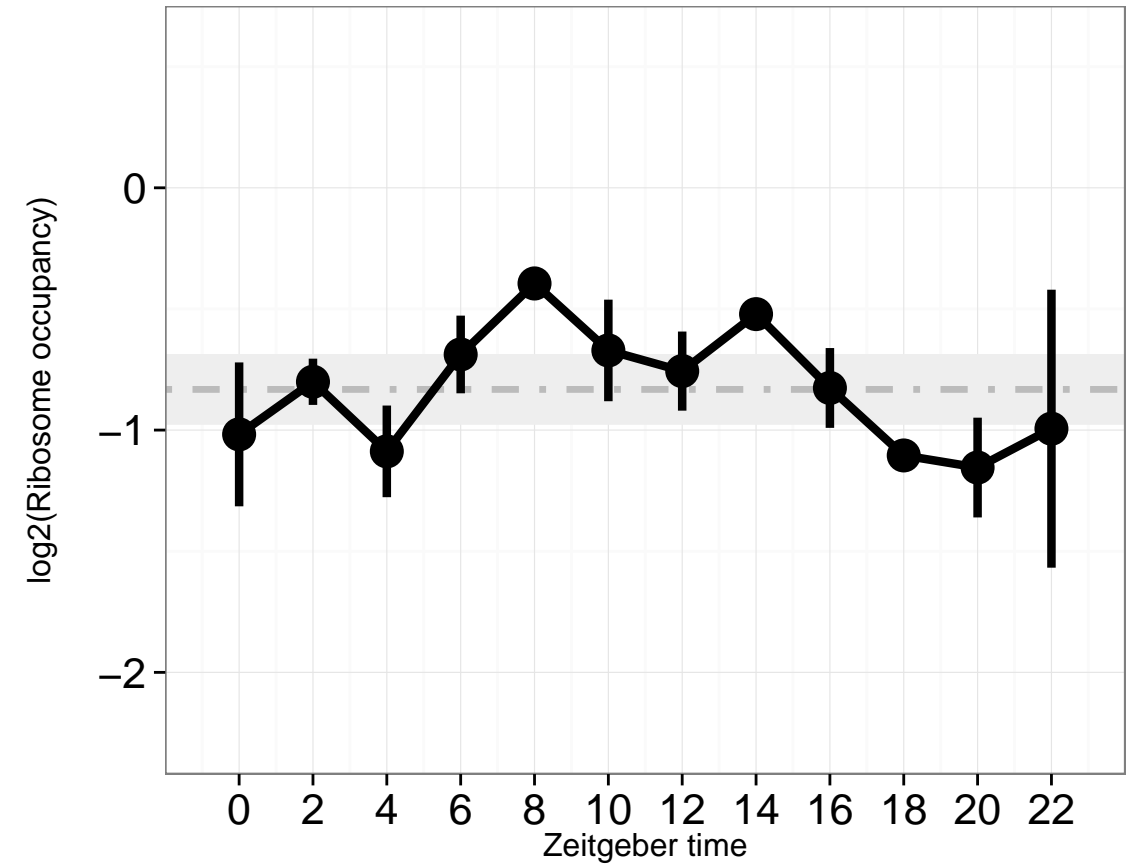

Supplement: Supplementary file 6 — Transcriptome-wide kidney RPF (blue) and RNA (orange) levels in the left panels (with “error bars” connecting the two replicates of each timepoint) and TE in the right panels. (ZIP 116896 kb) [file 13059_2017_1222_MOESM6_ESM.zip › Supp_Dataset_S1/A_RNA_non_rhythmic_RPF_non_rhythmic/3110043O21Rik_kidney_set_A.pdf]

# 3110057O12Rik

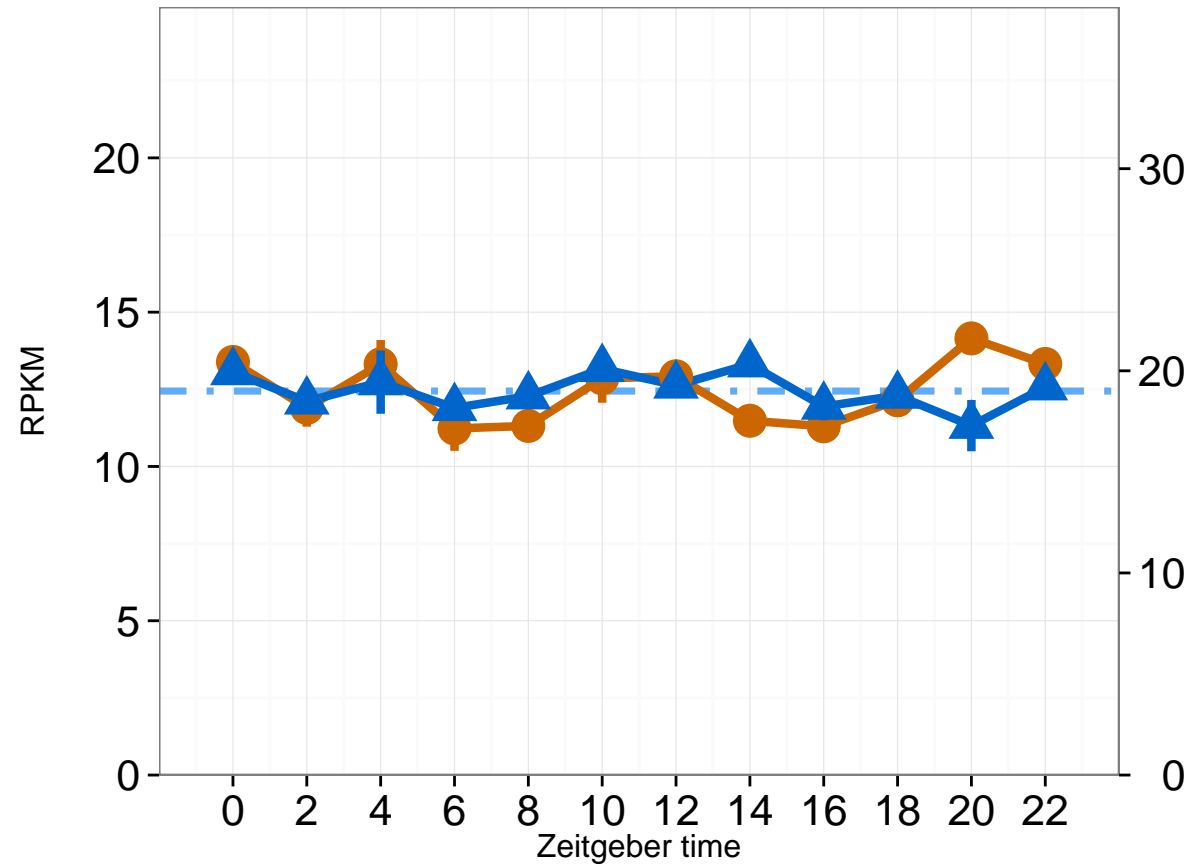

# 3110057O12Rik

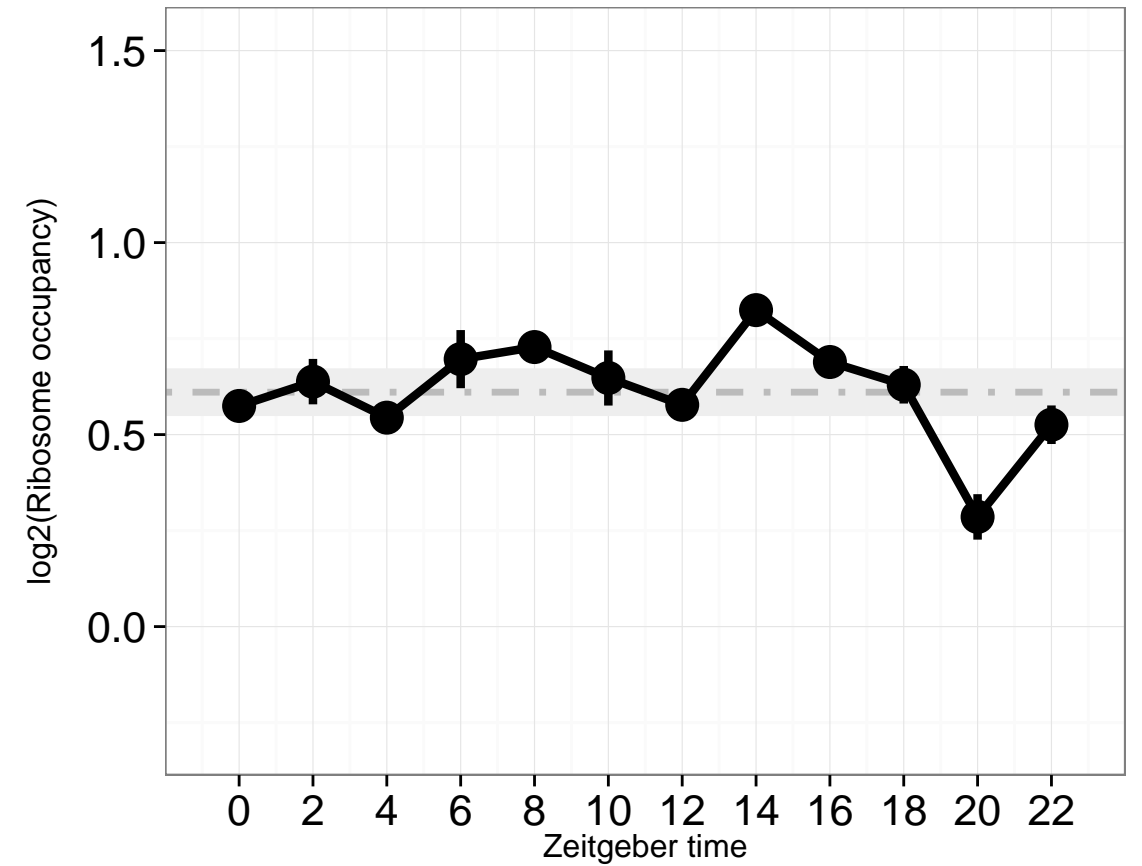

Supplement: Supplementary file 6 — Transcriptome-wide kidney RPF (blue) and RNA (orange) levels in the left panels (with “error bars” connecting the two replicates of each timepoint) and TE in the right panels. (ZIP 116896 kb) [file 13059_2017_1222_MOESM6_ESM.zip › Supp_Dataset_S1/A_RNA_non_rhythmic_RPF_non_rhythmic/3110057O12Rik_kidney_set_A.pdf]

# 3110062M04Rik

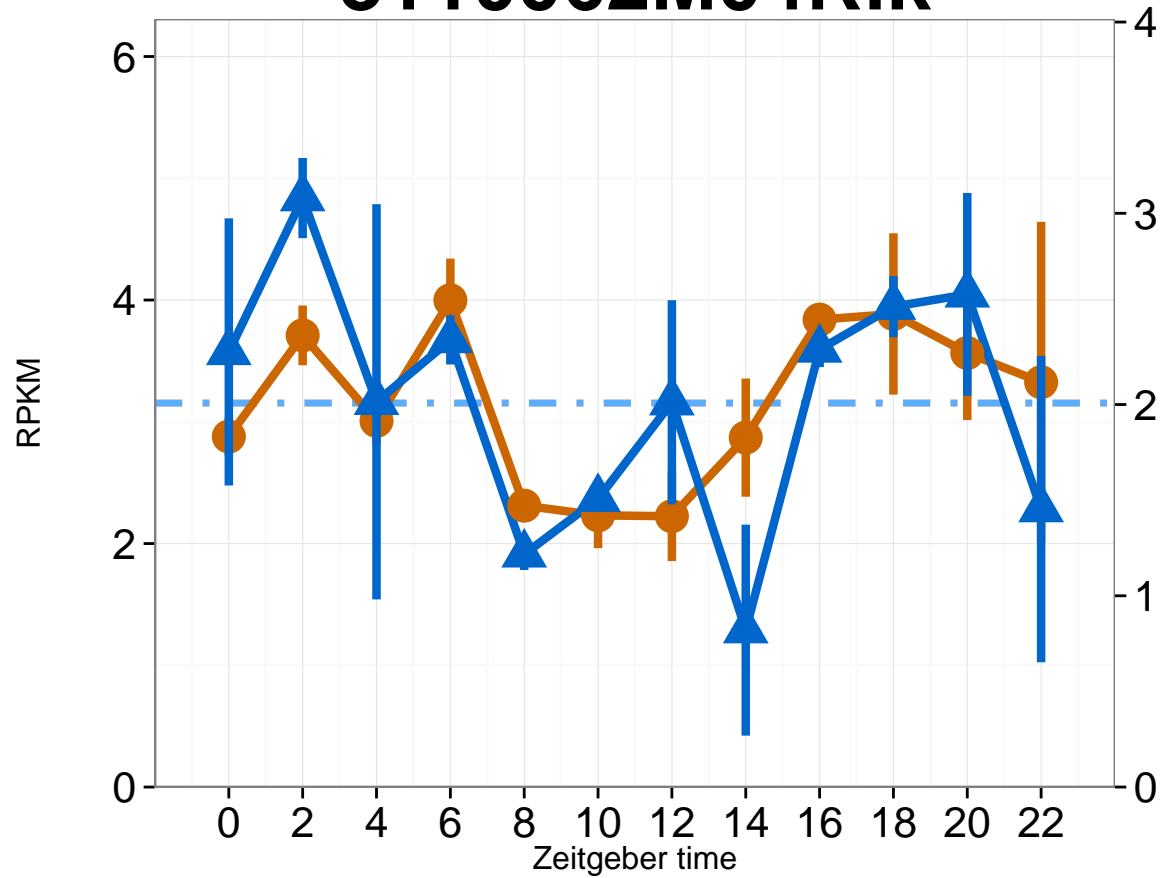

# 3110062M04Rik

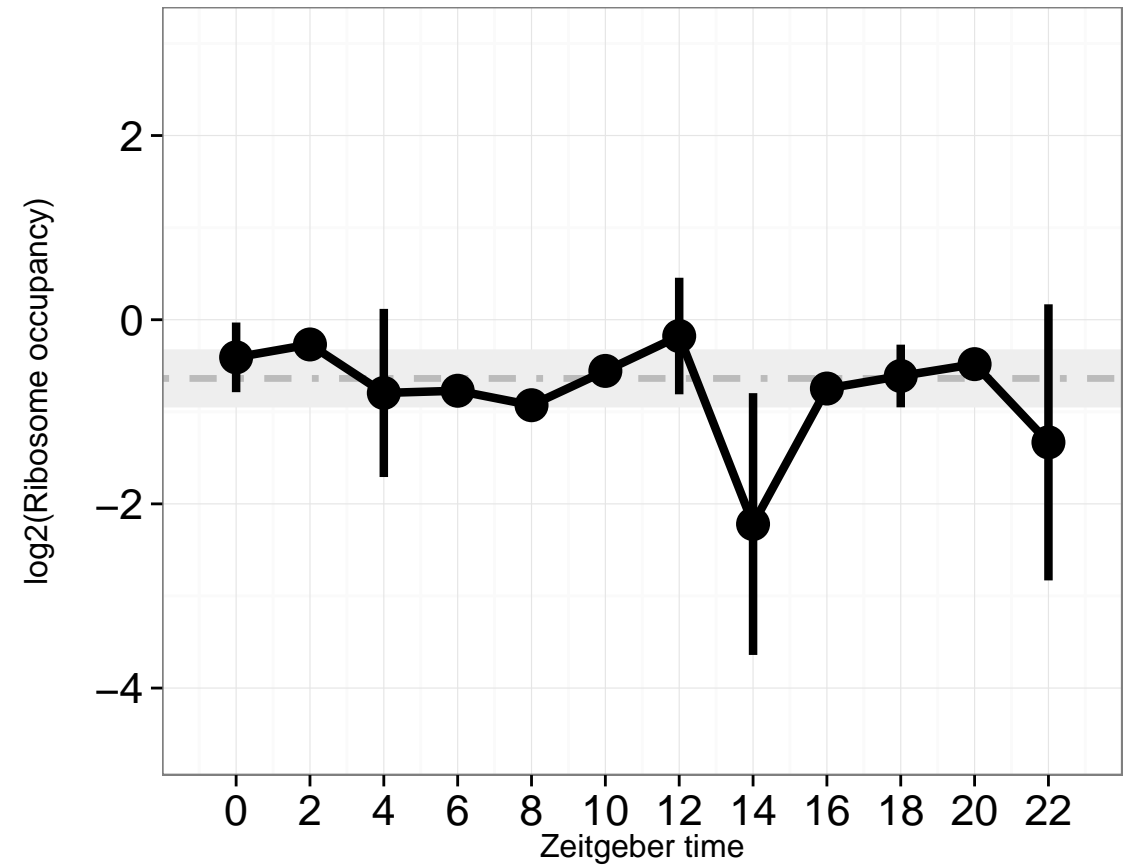

Supplement: Supplementary file 6 — Transcriptome-wide kidney RPF (blue) and RNA (orange) levels in the left panels (with “error bars” connecting the two replicates of each timepoint) and TE in the right panels. (ZIP 116896 kb) [file 13059_2017_1222_MOESM6_ESM.zip › Supp_Dataset_S1/A_RNA_non_rhythmic_RPF_non_rhythmic/3110062M04Rik_kidney_set_A.pdf]

# 3300002I08Rik

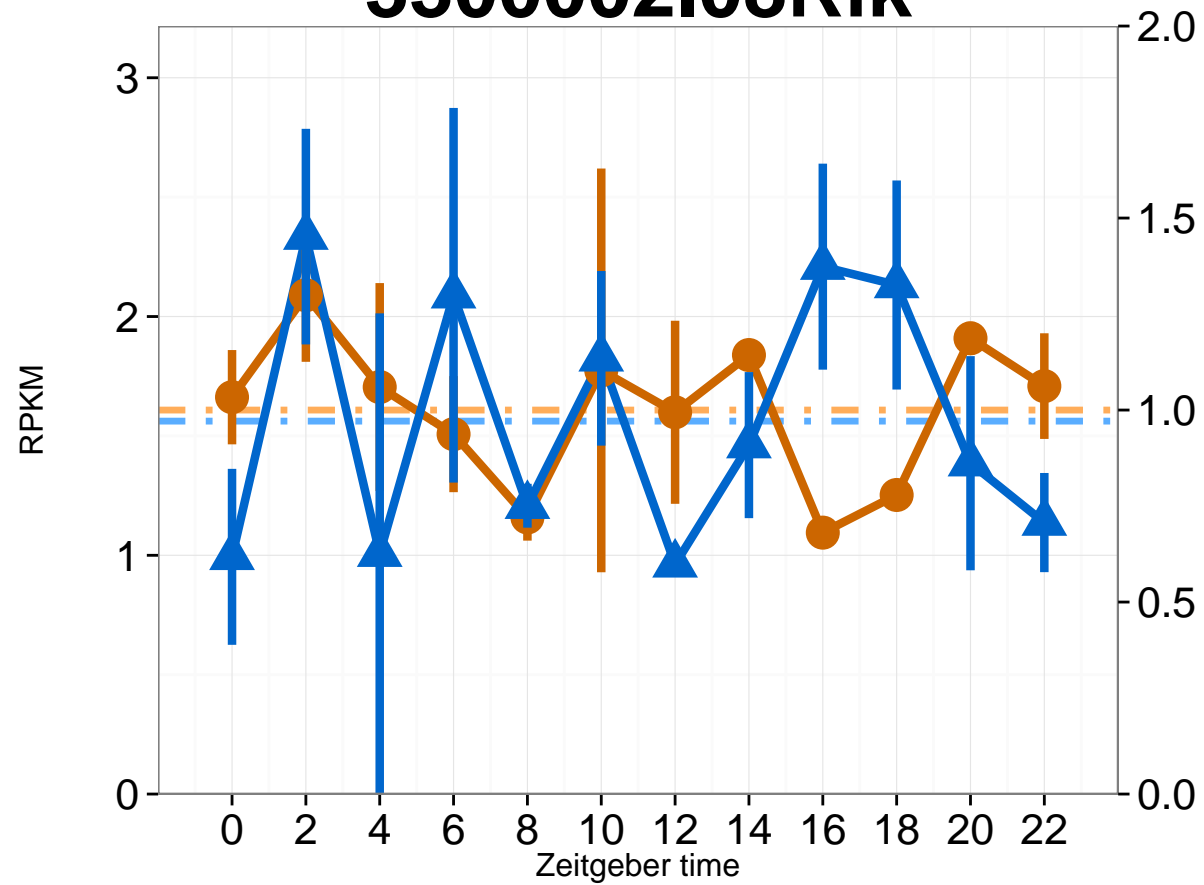

# 3300002I08Rik

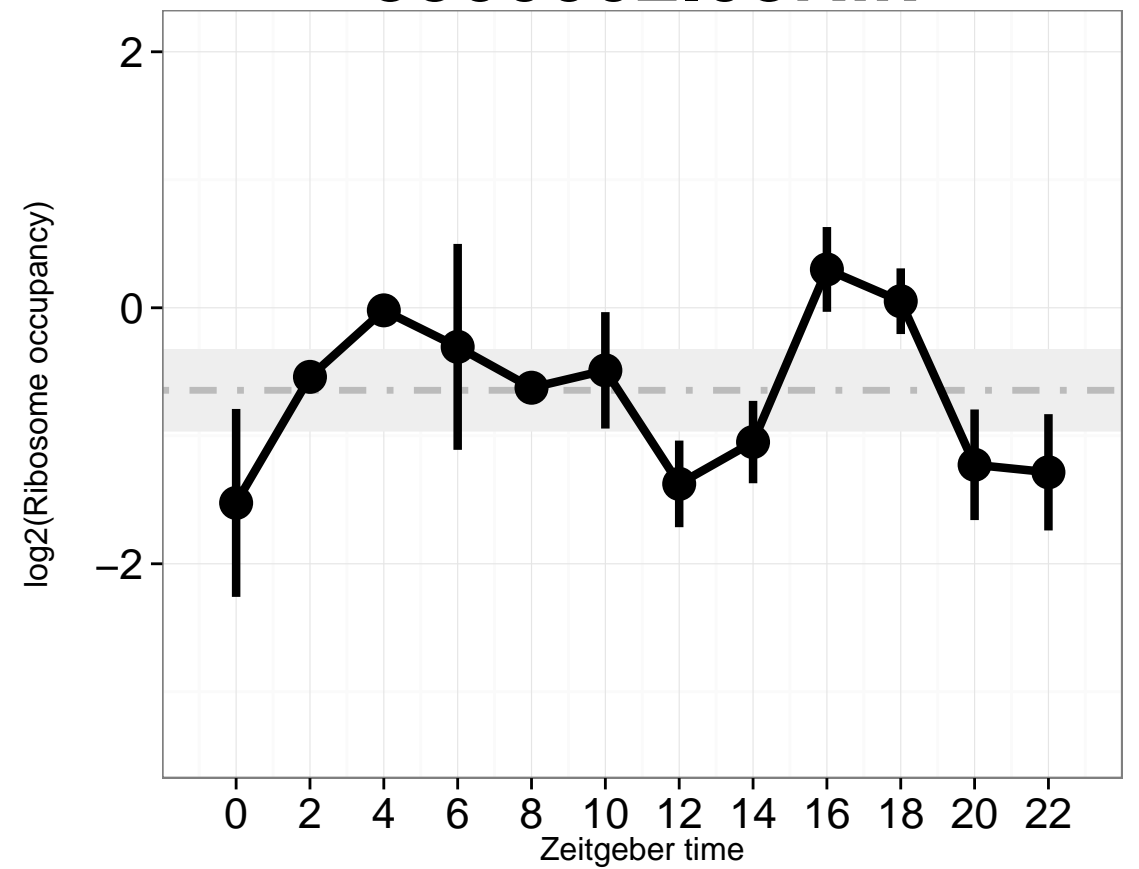

Supplement: Supplementary file 6 — Transcriptome-wide kidney RPF (blue) and RNA (orange) levels in the left panels (with “error bars” connecting the two replicates of each timepoint) and TE in the right panels. (ZIP 116896 kb) [file 13059_2017_1222_MOESM6_ESM.zip › Supp_Dataset_S1/A_RNA_non_rhythmic_RPF_non_rhythmic/3300002I08Rik_kidney_set_A.pdf]

# 3632451O06Rik

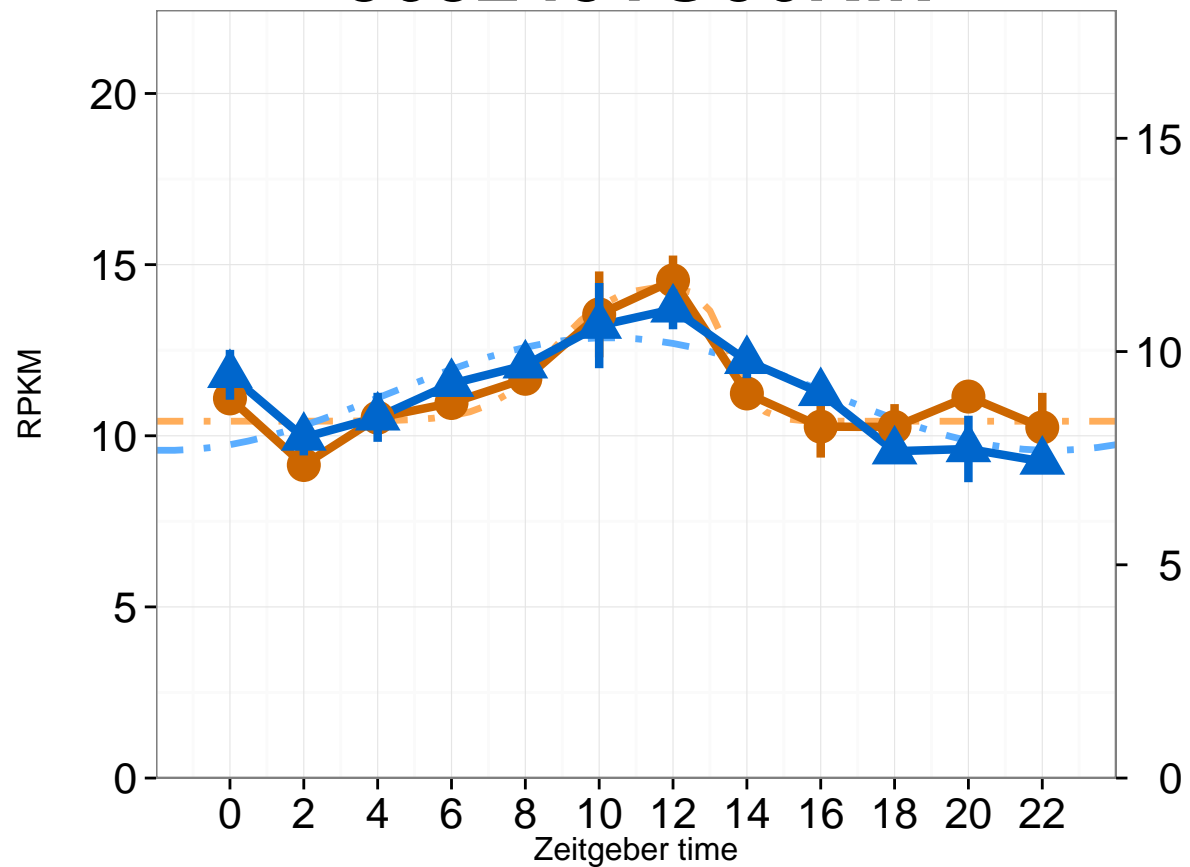

# 3632451O06Rik

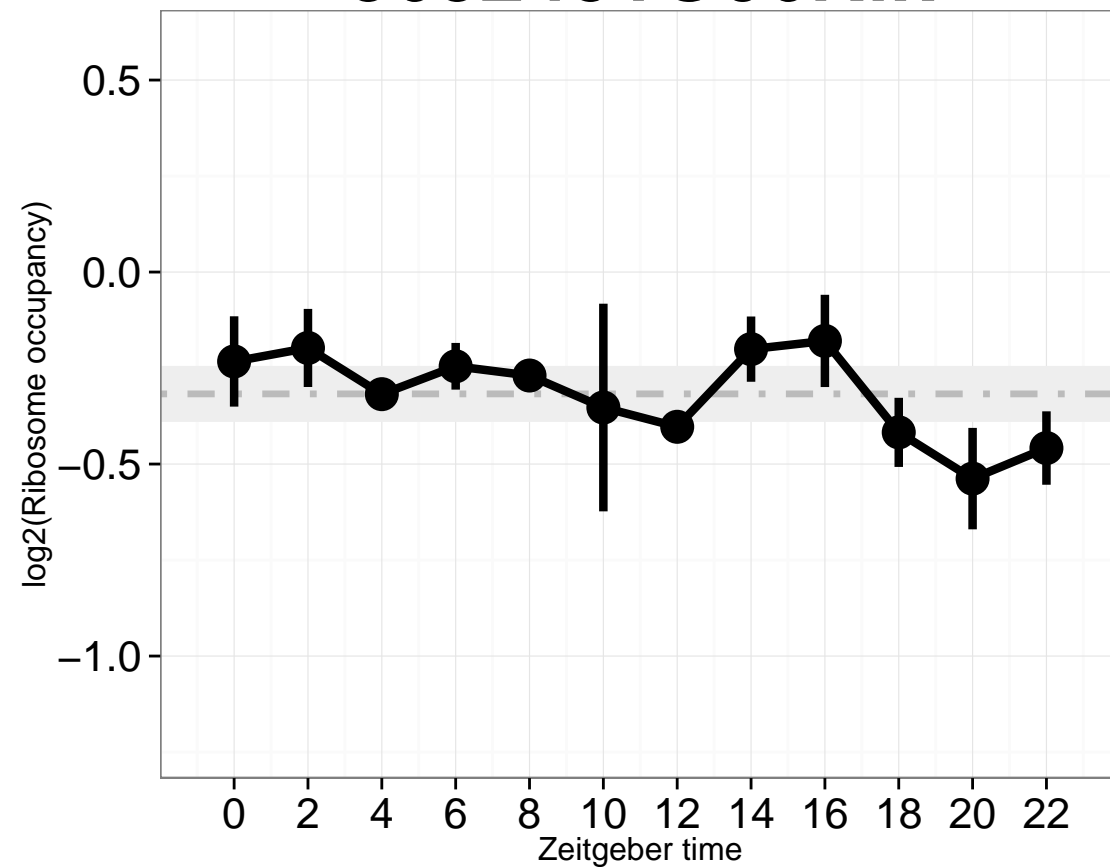

Supplement: Supplementary file 6 — Transcriptome-wide kidney RPF (blue) and RNA (orange) levels in the left panels (with “error bars” connecting the two replicates of each timepoint) and TE in the right panels. (ZIP 116896 kb) [file 13059_2017_1222_MOESM6_ESM.zip › Supp_Dataset_S1/A_RNA_non_rhythmic_RPF_non_rhythmic/3632451O06Rik_kidney_set_A.pdf]

# 3830406C13Rik

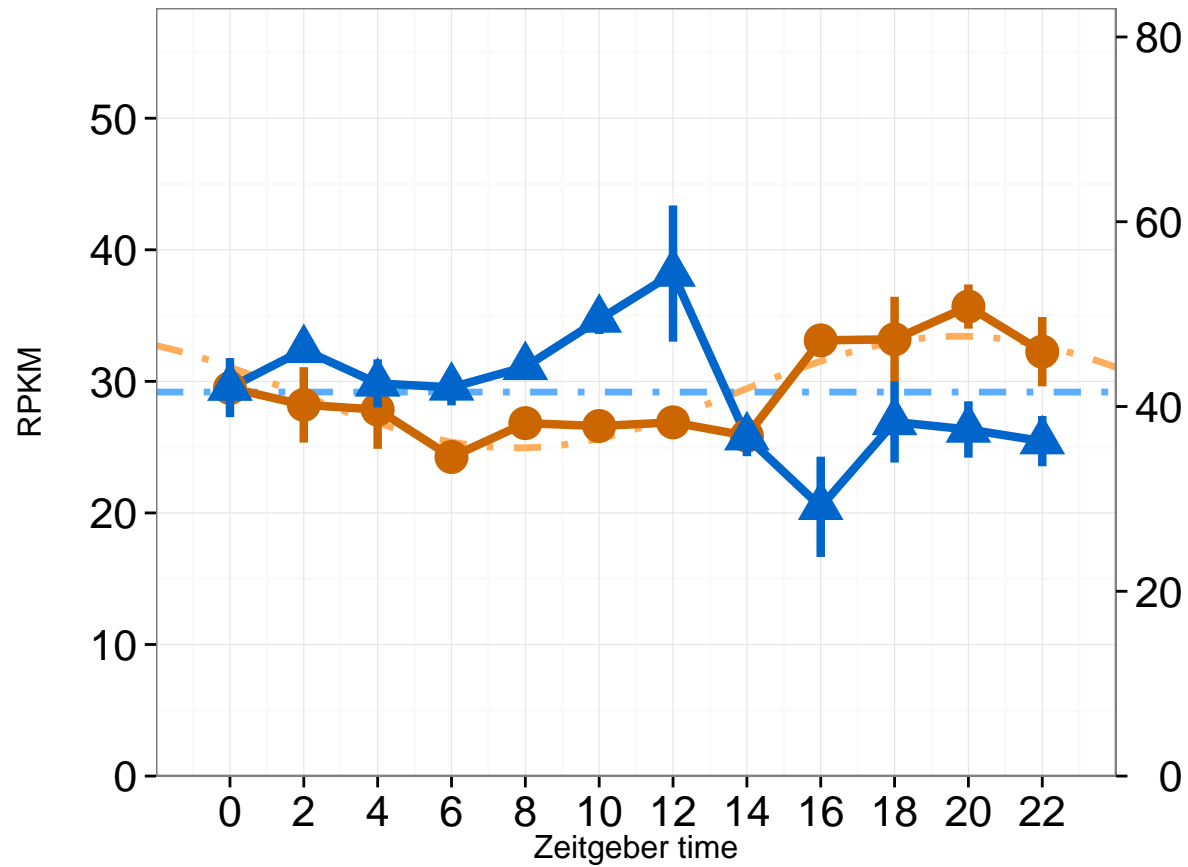

# 3830406C13Rik

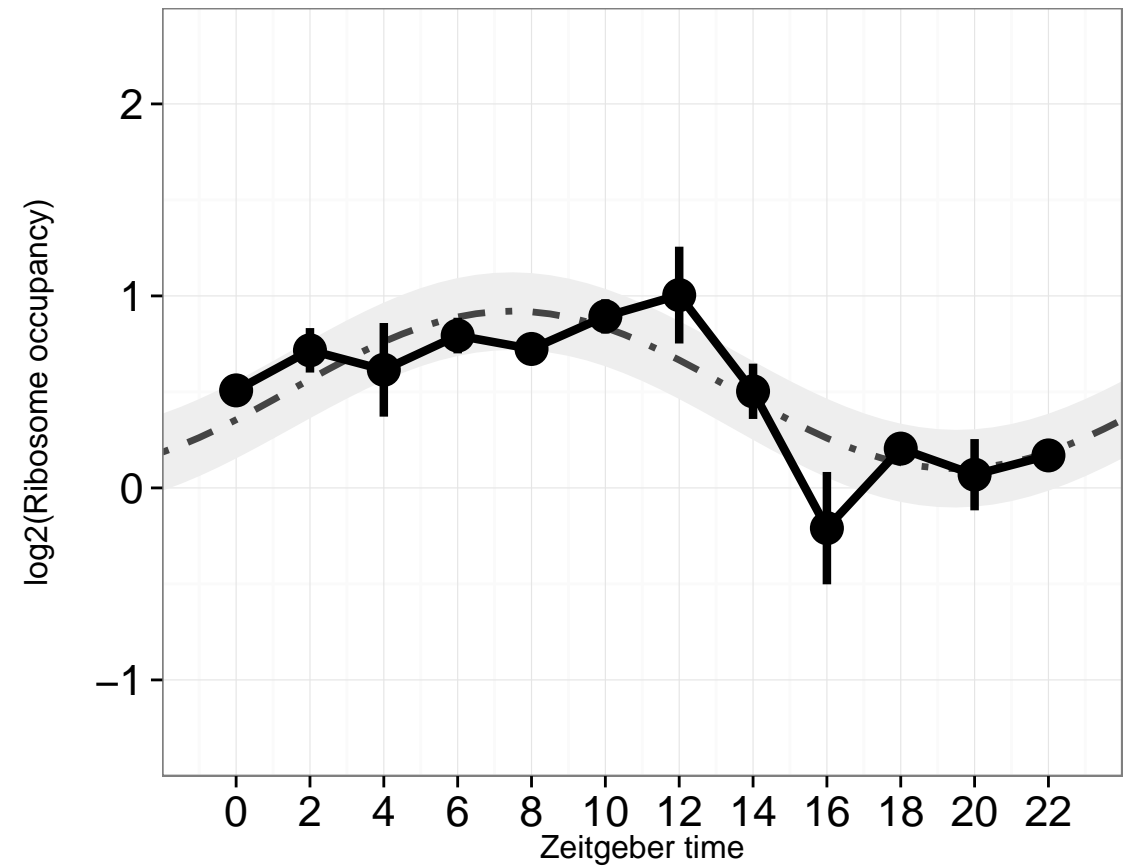

Supplement: Supplementary file 6 — Transcriptome-wide kidney RPF (blue) and RNA (orange) levels in the left panels (with “error bars” connecting the two replicates of each timepoint) and TE in the right panels. (ZIP 116896 kb) [file 13059_2017_1222_MOESM6_ESM.zip › Supp_Dataset_S1/A_RNA_non_rhythmic_RPF_non_rhythmic/3830406C13Rik_kidney_set_A.pdf]

**4430402I18Rik**

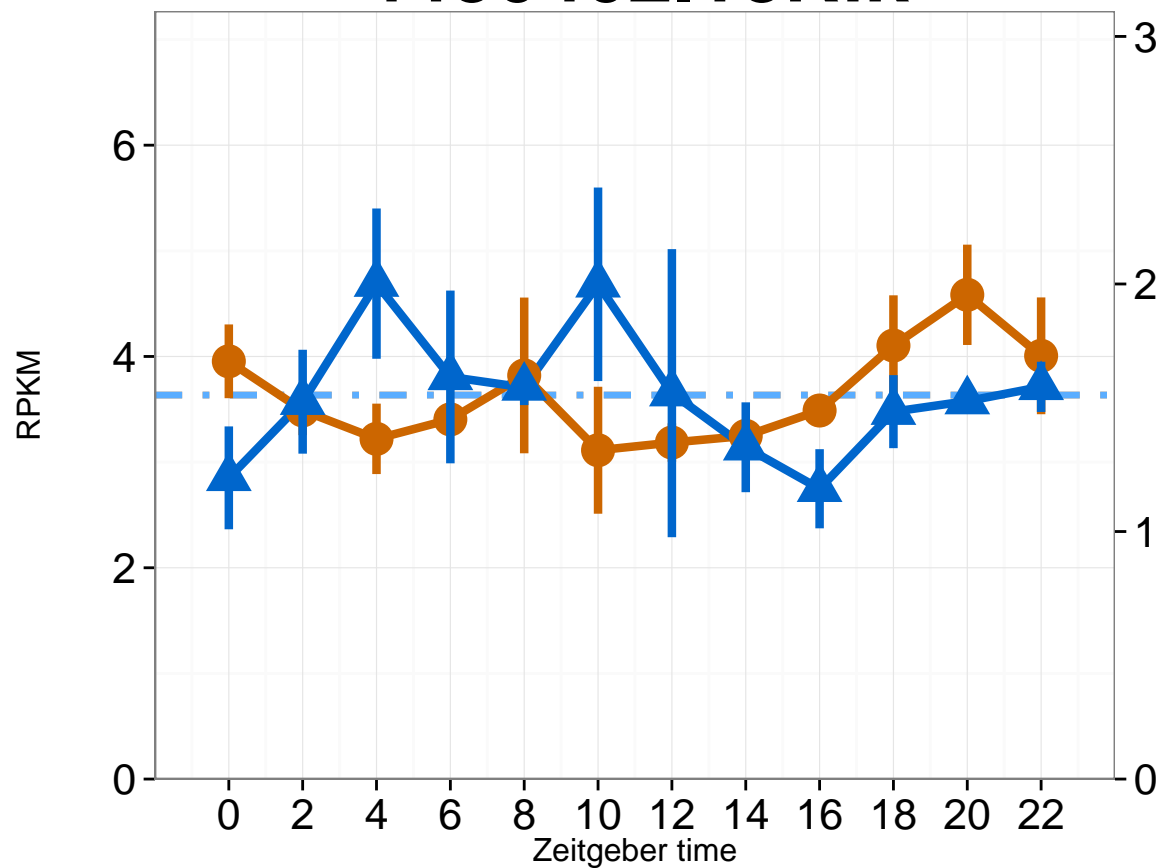

**4430402I18Rik**

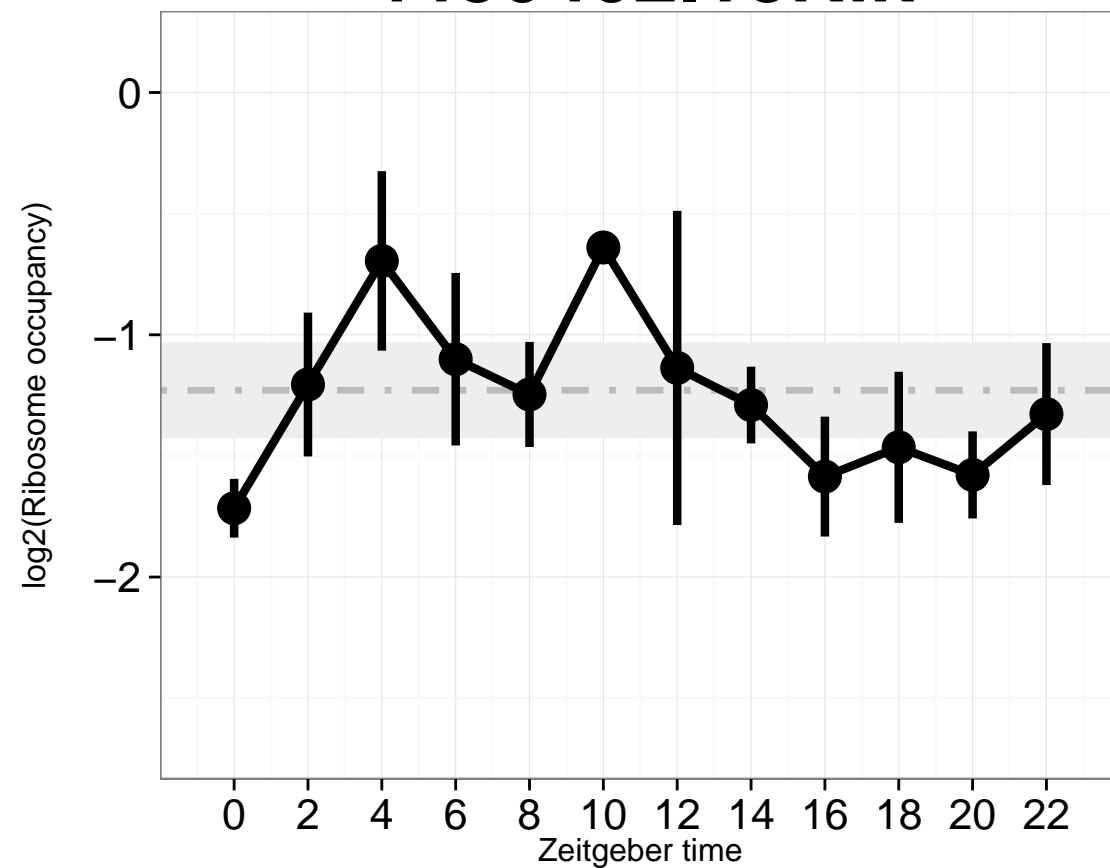

Supplement: Supplementary file 6 — Transcriptome-wide kidney RPF (blue) and RNA (orange) levels in the left panels (with “error bars” connecting the two replicates of each timepoint) and TE in the right panels. (ZIP 116896 kb) [file 13059_2017_1222_MOESM6_ESM.zip › Supp_Dataset_S1/A_RNA_non_rhythmic_RPF_non_rhythmic/4430402I18Rik_kidney_set_A.pdf]

# 4632428N05Rik

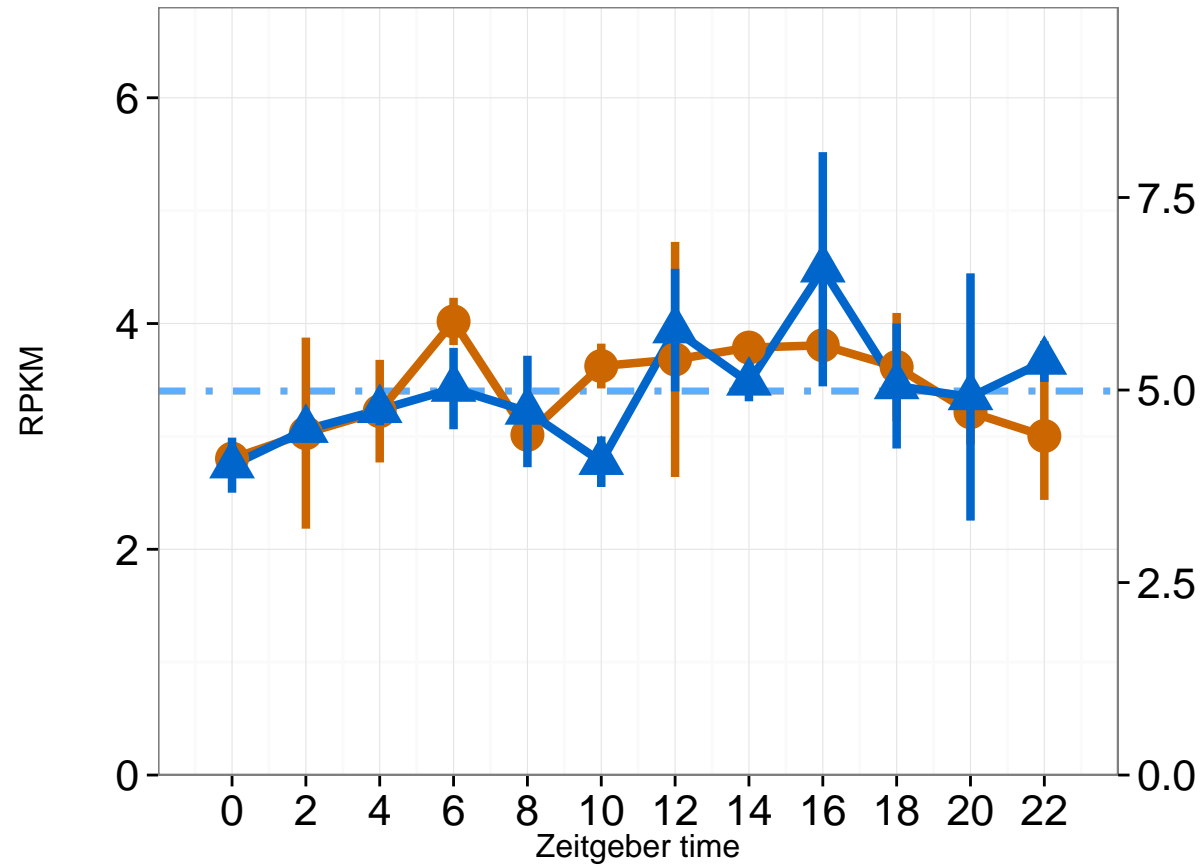

# 4632428N05Rik

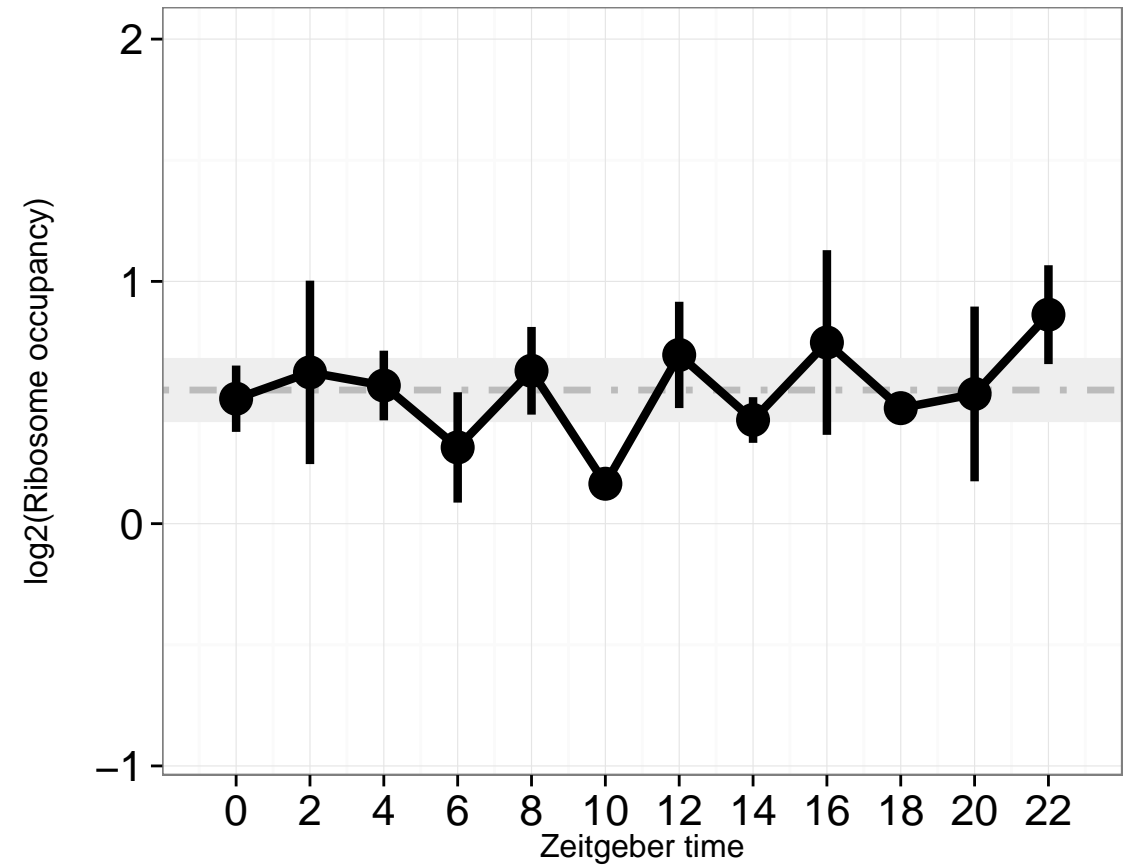

Supplement: Supplementary file 6 — Transcriptome-wide kidney RPF (blue) and RNA (orange) levels in the left panels (with “error bars” connecting the two replicates of each timepoint) and TE in the right panels. (ZIP 116896 kb) [file 13059_2017_1222_MOESM6_ESM.zip › Supp_Dataset_S1/A_RNA_non_rhythmic_RPF_non_rhythmic/4632428N05Rik_kidney_set_A.pdf]

# 4732465J04Rik

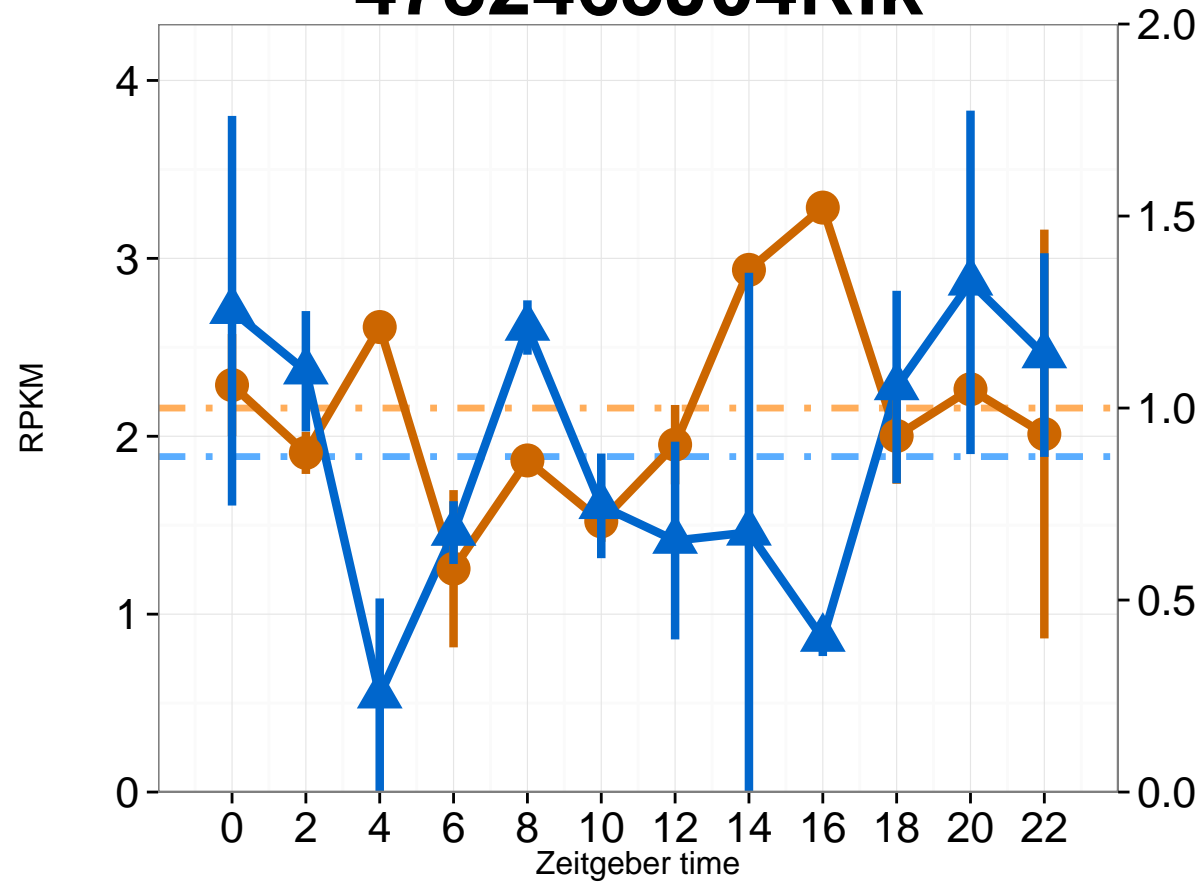

# 4732465J04Rik

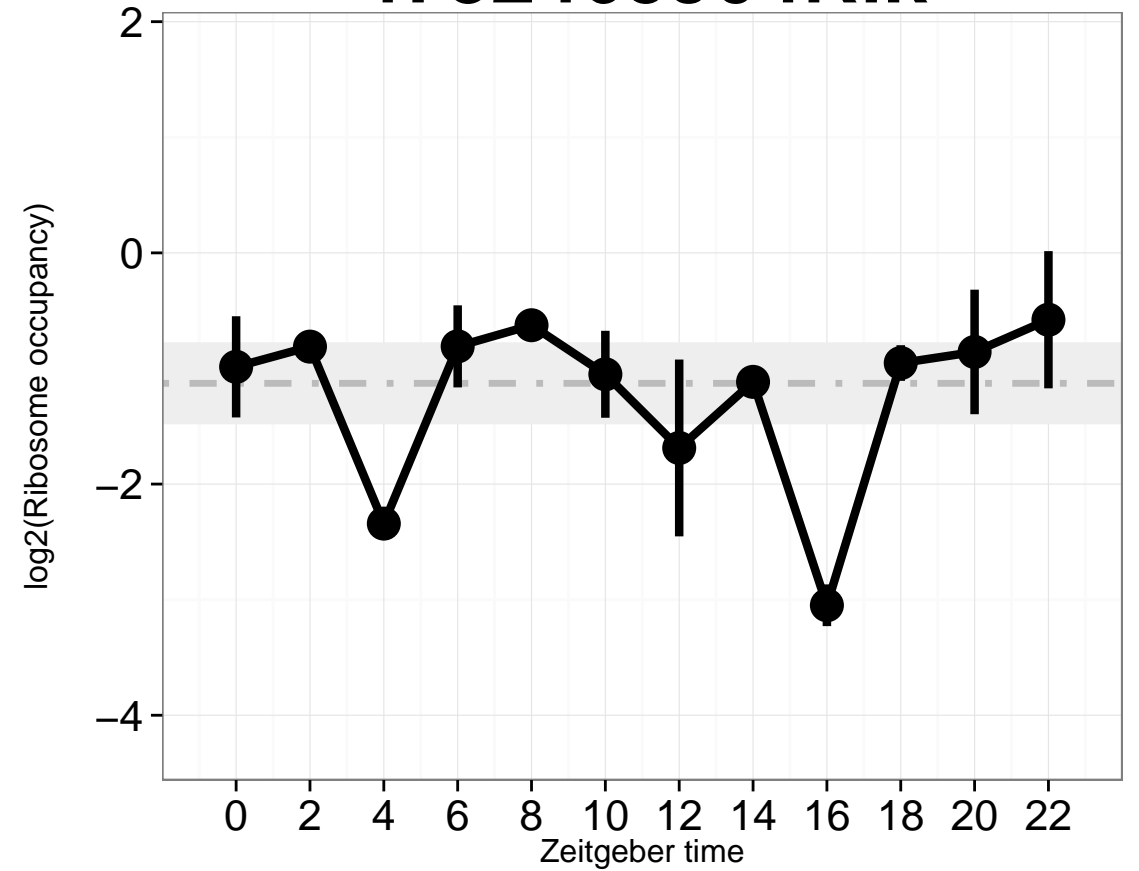

Supplement: Supplementary file 6 — Transcriptome-wide kidney RPF (blue) and RNA (orange) levels in the left panels (with “error bars” connecting the two replicates of each timepoint) and TE in the right panels. (ZIP 116896 kb) [file 13059_2017_1222_MOESM6_ESM.zip › Supp_Dataset_S1/A_RNA_non_rhythmic_RPF_non_rhythmic/4732465J04Rik_kidney_set_A.pdf]

# 4833420G17Rik

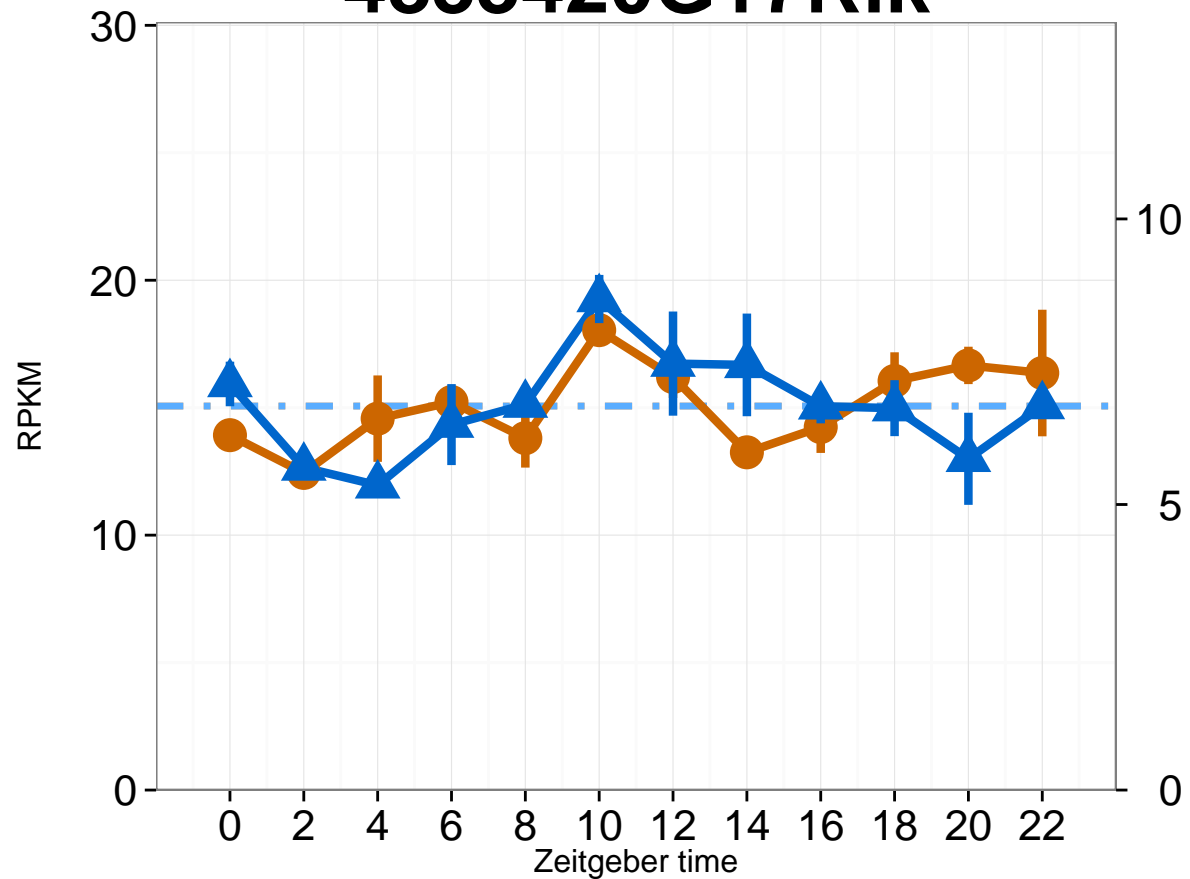

# 4833420G17Rik

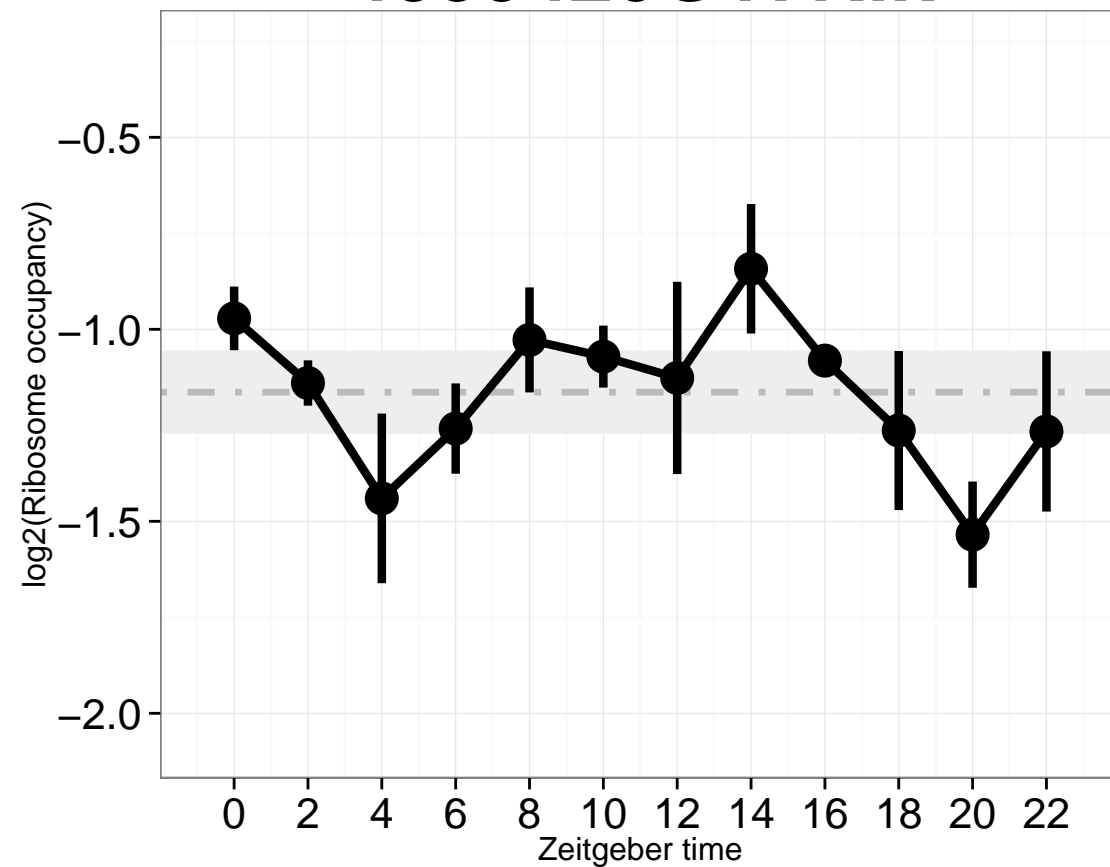

Supplement: Supplementary file 6 — Transcriptome-wide kidney RPF (blue) and RNA (orange) levels in the left panels (with “error bars” connecting the two replicates of each timepoint) and TE in the right panels. (ZIP 116896 kb) [file 13059_2017_1222_MOESM6_ESM.zip › Supp_Dataset_S1/A_RNA_non_rhythmic_RPF_non_rhythmic/4833420G17Rik_kidney_set_A.pdf]

## 4833439L19Rik

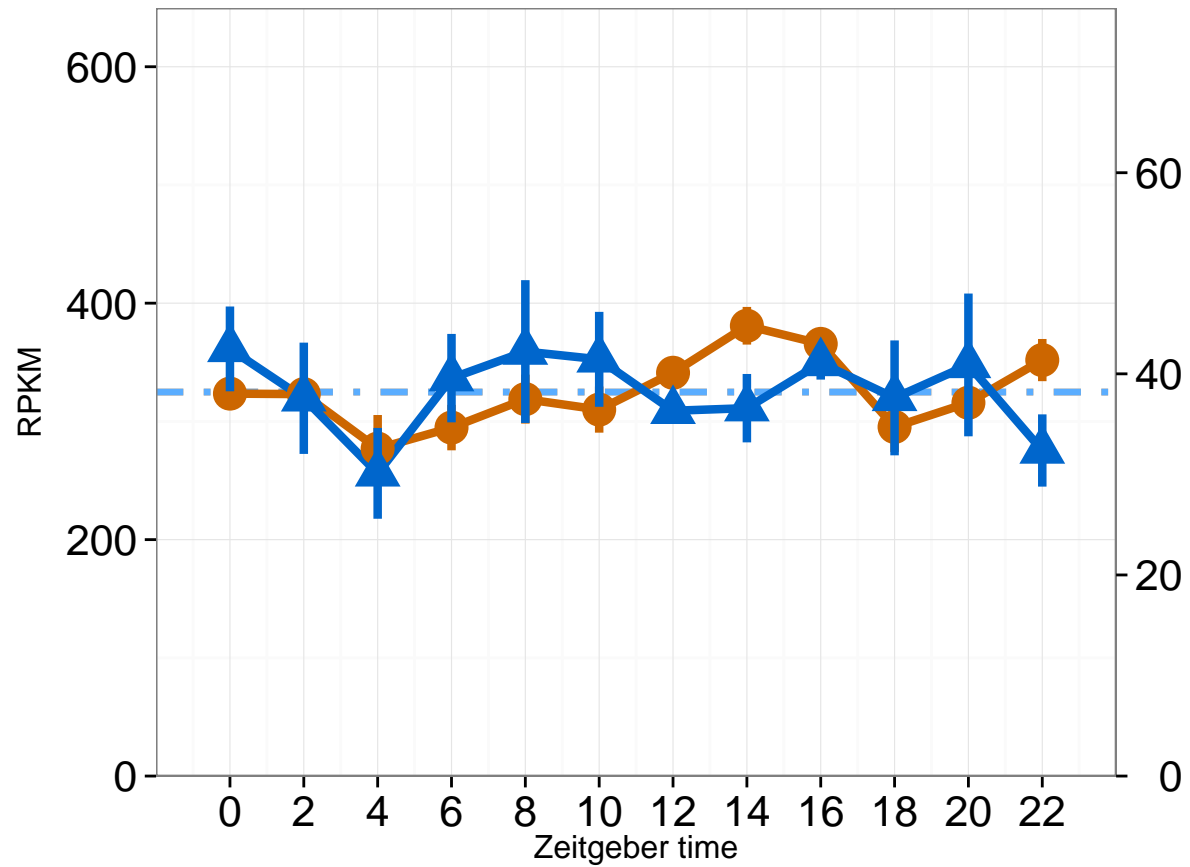

## 4833439L19Rik

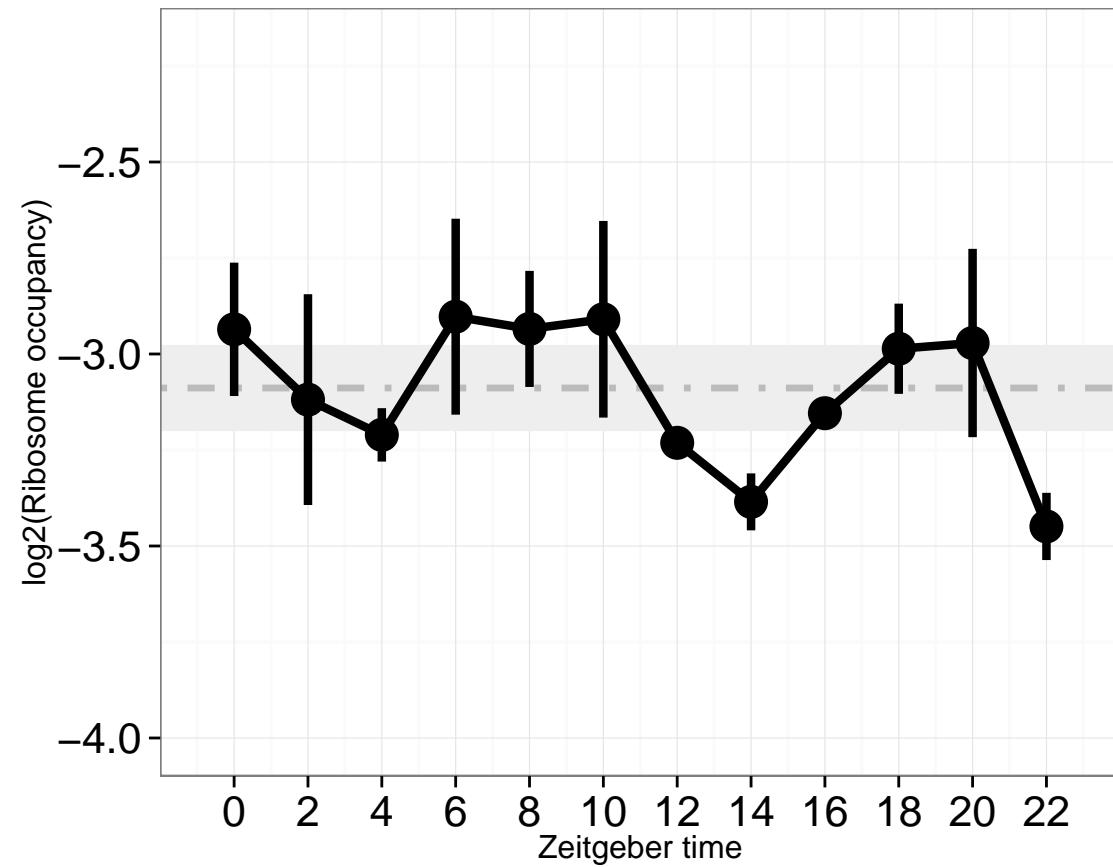

Supplement: Supplementary file 6 — Transcriptome-wide kidney RPF (blue) and RNA (orange) levels in the left panels (with “error bars” connecting the two replicates of each timepoint) and TE in the right panels. (ZIP 116896 kb) [file 13059_2017_1222_MOESM6_ESM.zip › Supp_Dataset_S1/A_RNA_non_rhythmic_RPF_non_rhythmic/4833439L19Rik_kidney_set_A.pdf]

## 4921524J17Rik

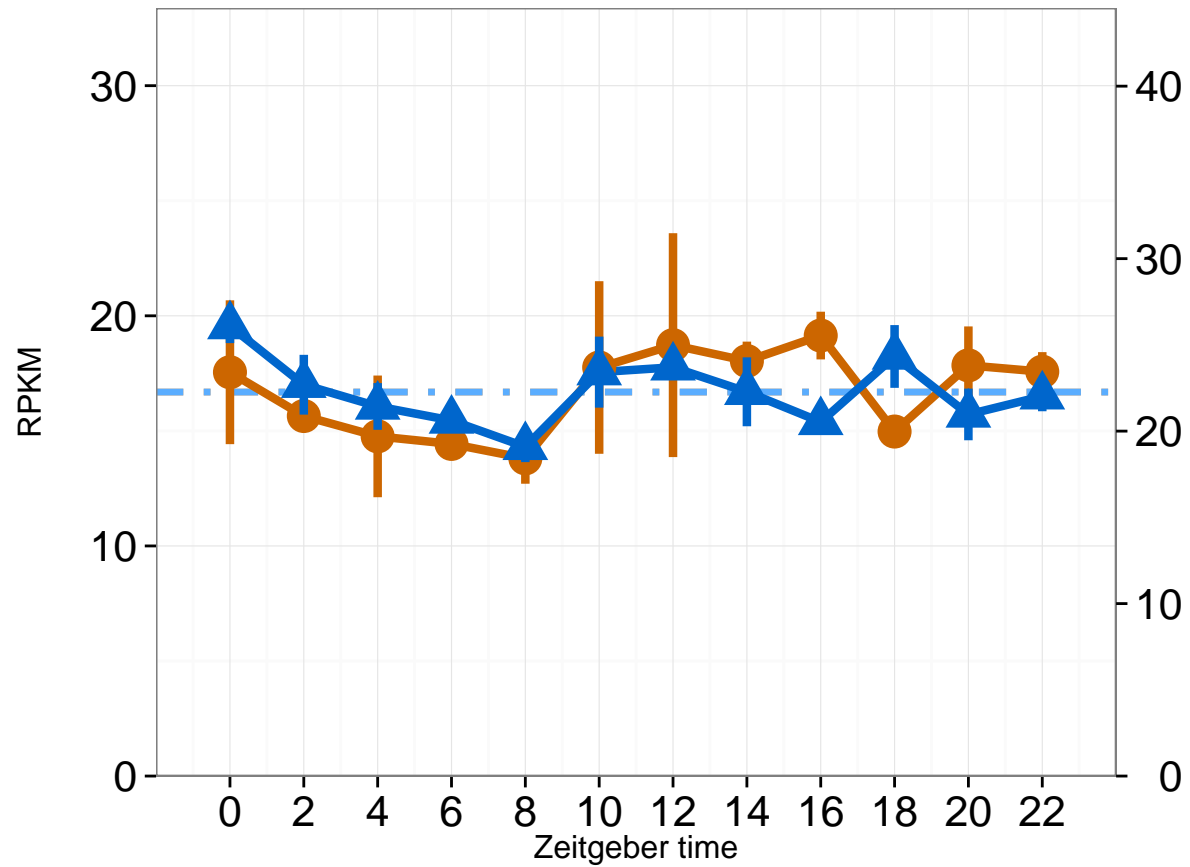

## 4921524J17Rik

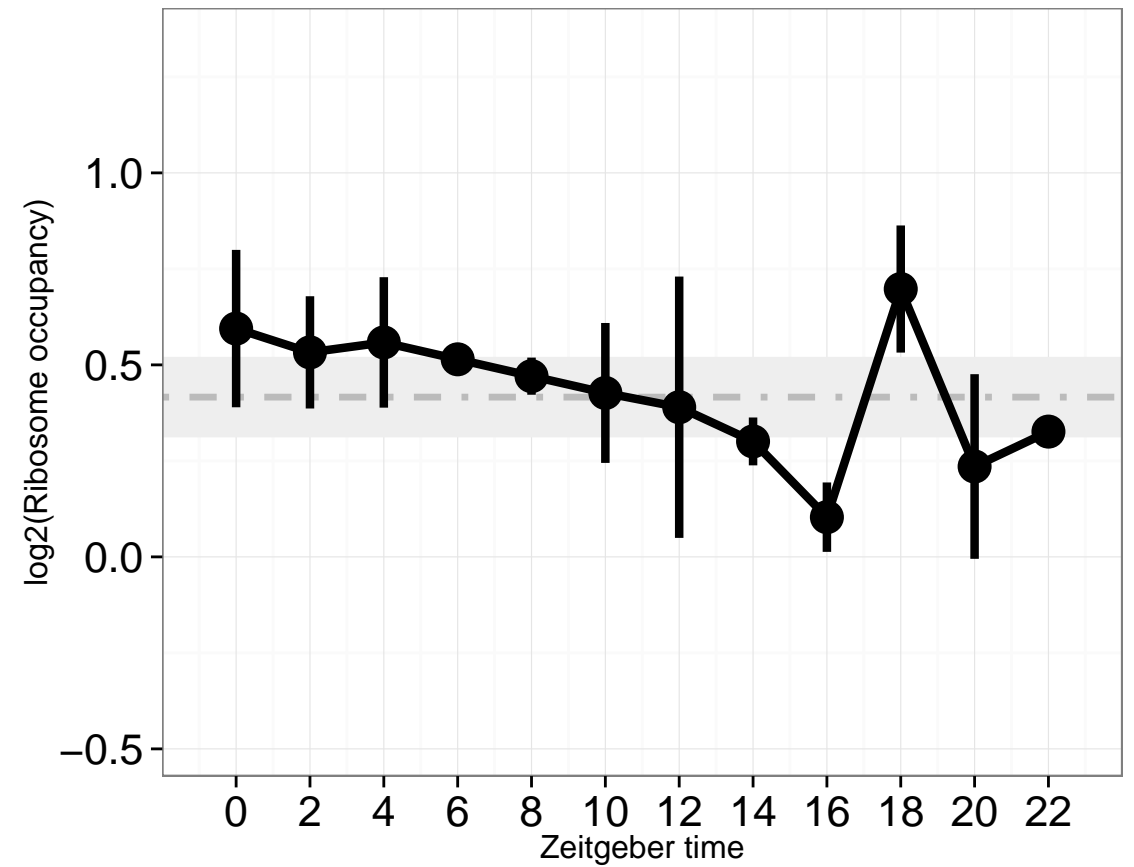

Supplement: Supplementary file 6 — Transcriptome-wide kidney RPF (blue) and RNA (orange) levels in the left panels (with “error bars” connecting the two replicates of each timepoint) and TE in the right panels. (ZIP 116896 kb) [file 13059_2017_1222_MOESM6_ESM.zip › Supp_Dataset_S1/A_RNA_non_rhythmic_RPF_non_rhythmic/4921524J17Rik_kidney_set_A.pdf]

**4921536K21Rik**

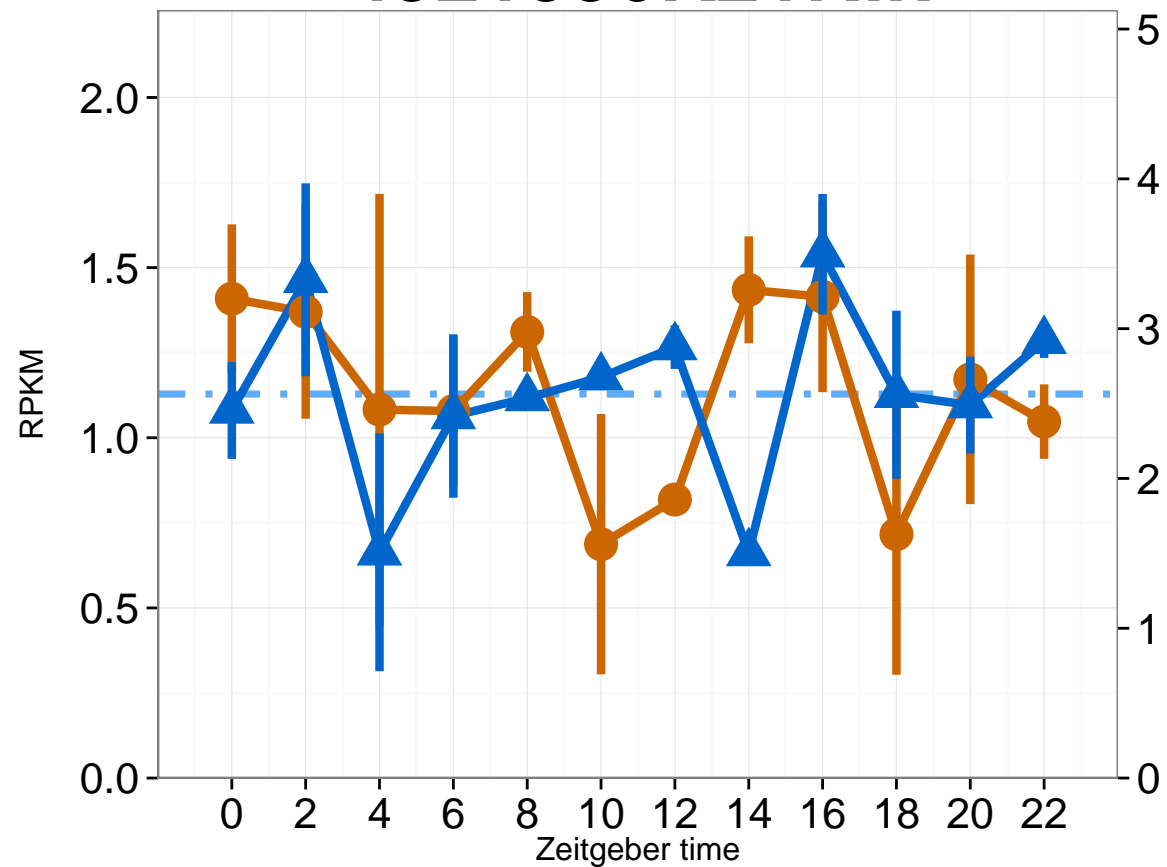

**4921536K21Rik**

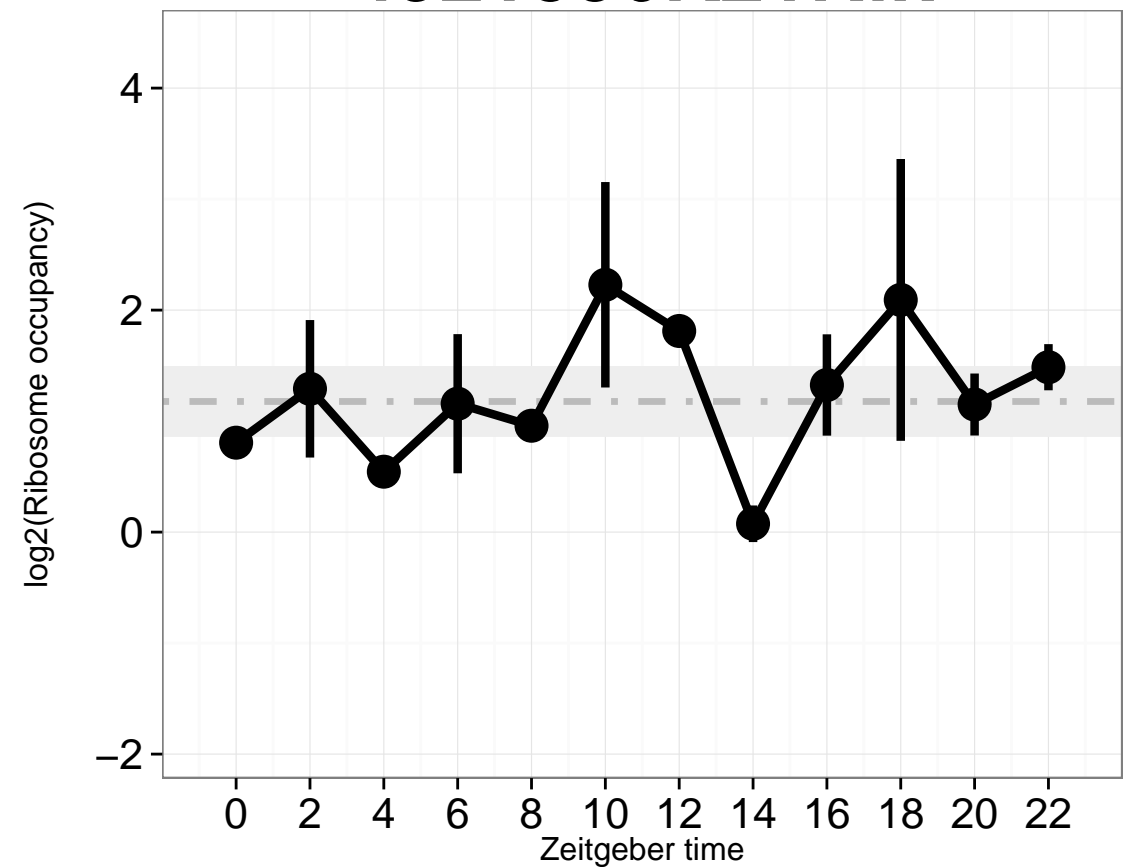

Supplement: Supplementary file 6 — Transcriptome-wide kidney RPF (blue) and RNA (orange) levels in the left panels (with “error bars” connecting the two replicates of each timepoint) and TE in the right panels. (ZIP 116896 kb) [file 13059_2017_1222_MOESM6_ESM.zip › Supp_Dataset_S1/A_RNA_non_rhythmic_RPF_non_rhythmic/4921536K21Rik_kidney_set_A.pdf]

## 4922501C03Rik

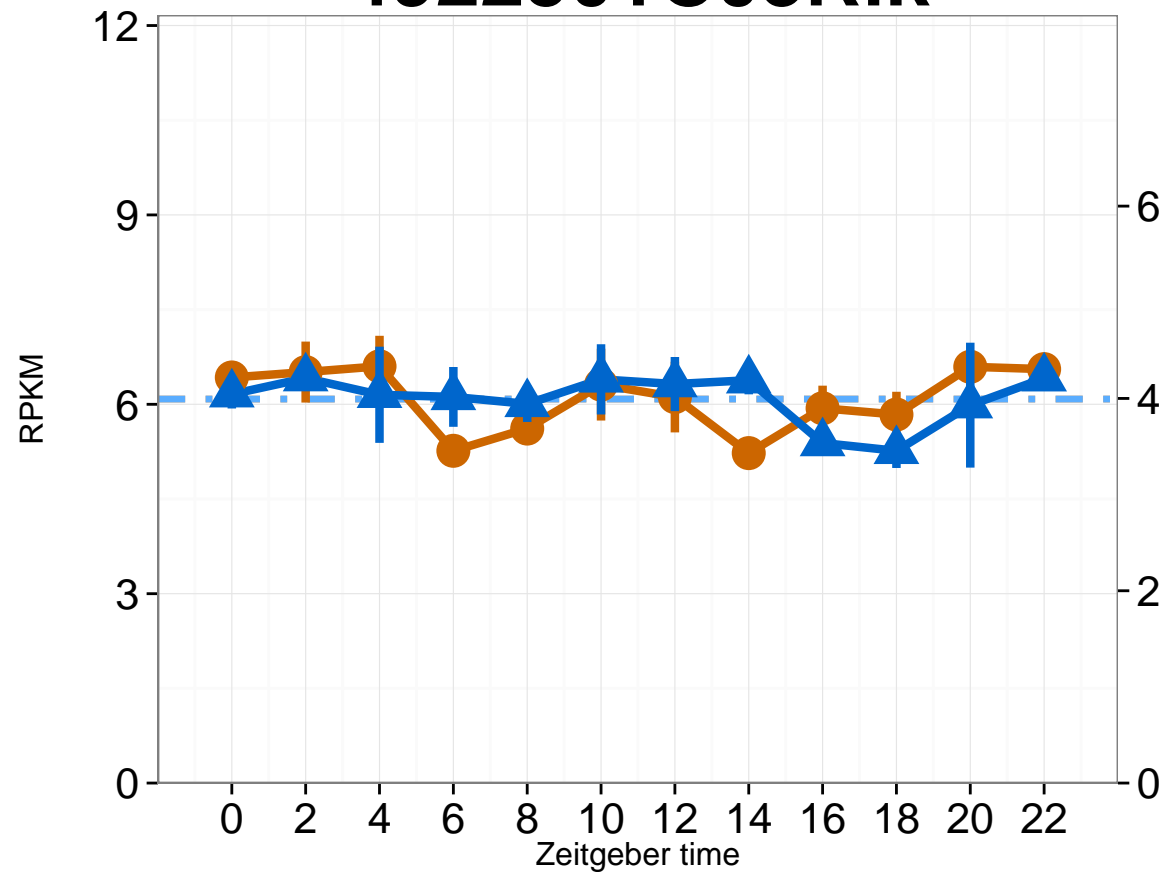

## 4922501C03Rik

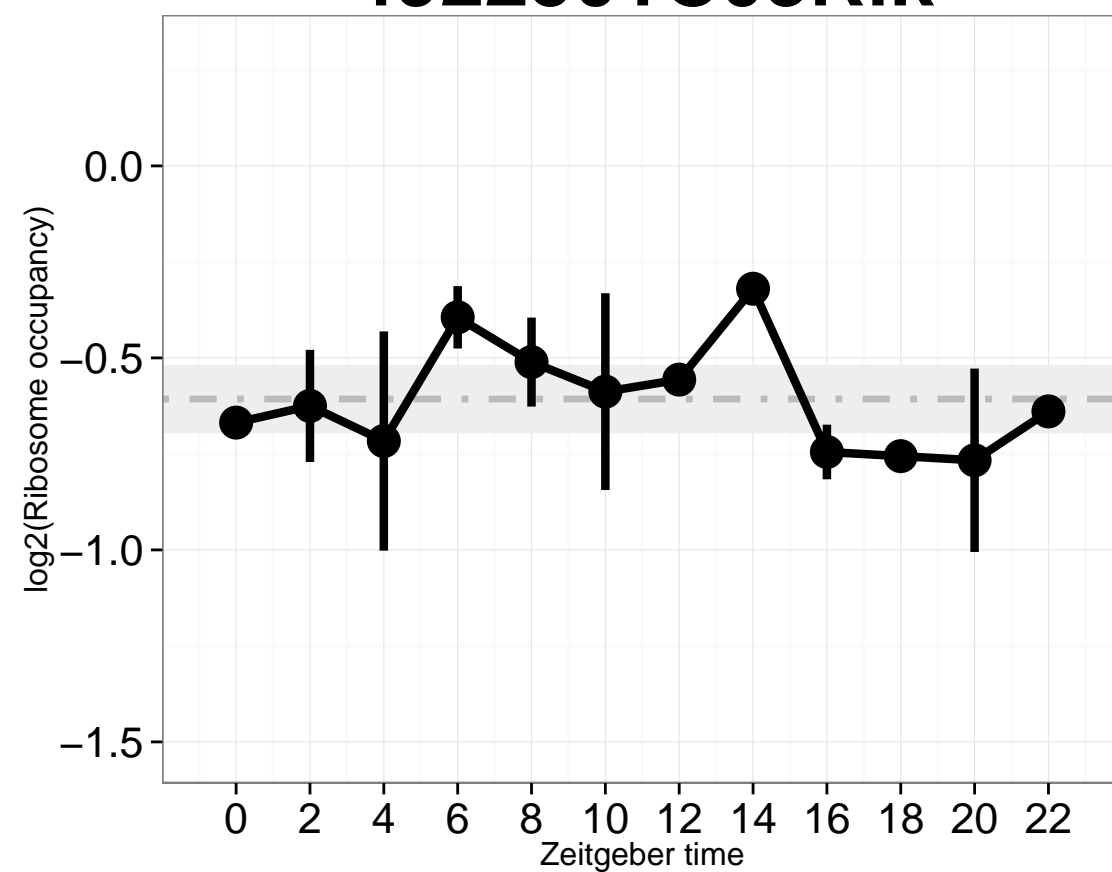

Supplement: Supplementary file 6 — Transcriptome-wide kidney RPF (blue) and RNA (orange) levels in the left panels (with “error bars” connecting the two replicates of each timepoint) and TE in the right panels. (ZIP 116896 kb) [file 13059_2017_1222_MOESM6_ESM.zip › Supp_Dataset_S1/A_RNA_non_rhythmic_RPF_non_rhythmic/4922501C03Rik_kidney_set_A.pdf]

## 4930402H24Rik

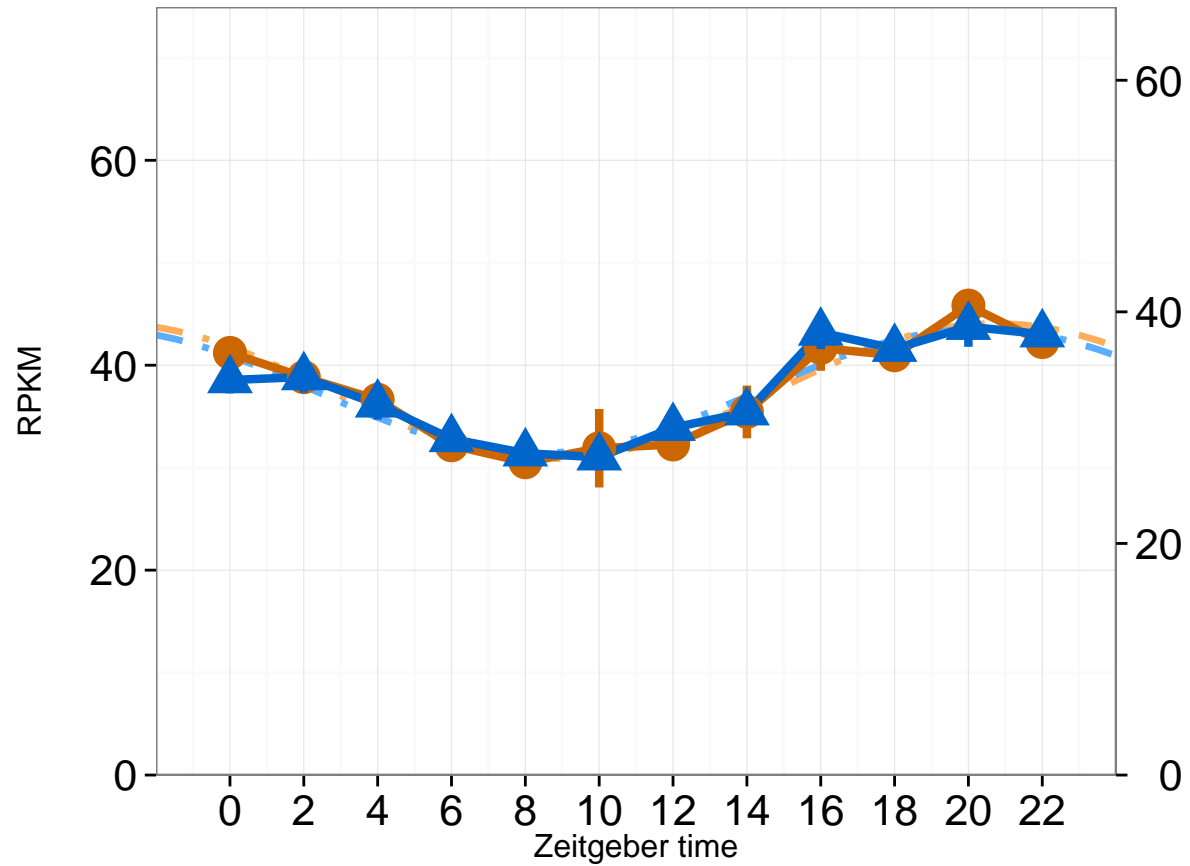

## 4930402H24Rik

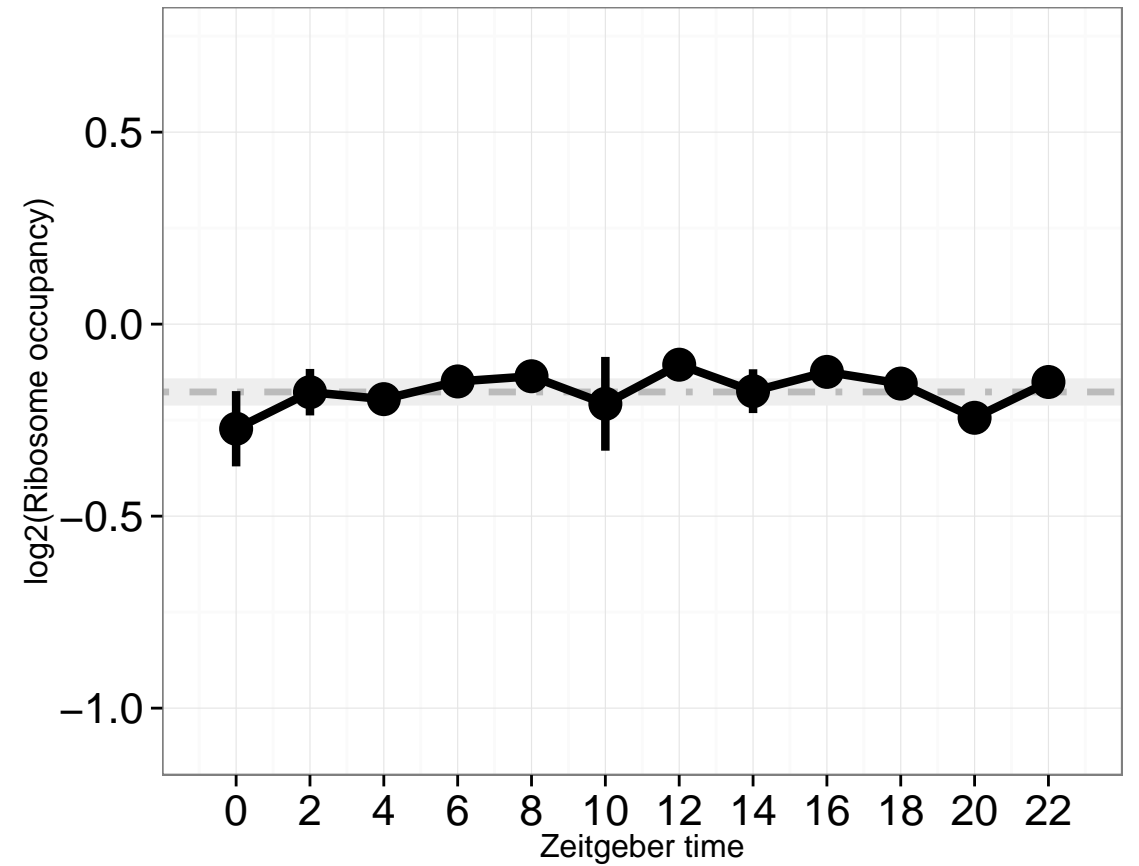

Supplement: Supplementary file 6 — Transcriptome-wide kidney RPF (blue) and RNA (orange) levels in the left panels (with “error bars” connecting the two replicates of each timepoint) and TE in the right panels. (ZIP 116896 kb) [file 13059_2017_1222_MOESM6_ESM.zip › Supp_Dataset_S1/A_RNA_non_rhythmic_RPF_non_rhythmic/4930402H24Rik_kidney_set_A.pdf]

# 4930422G04Rik

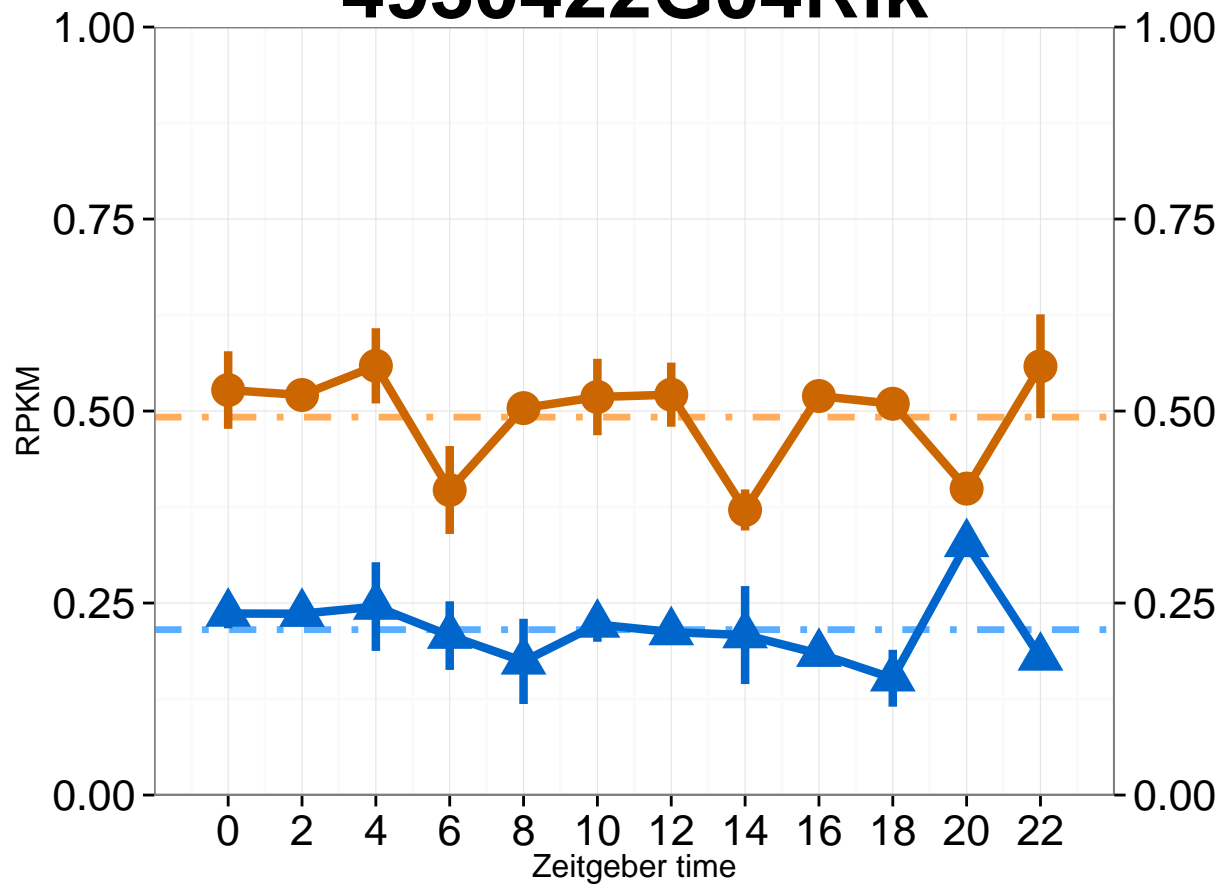

# 4930422G04Rik

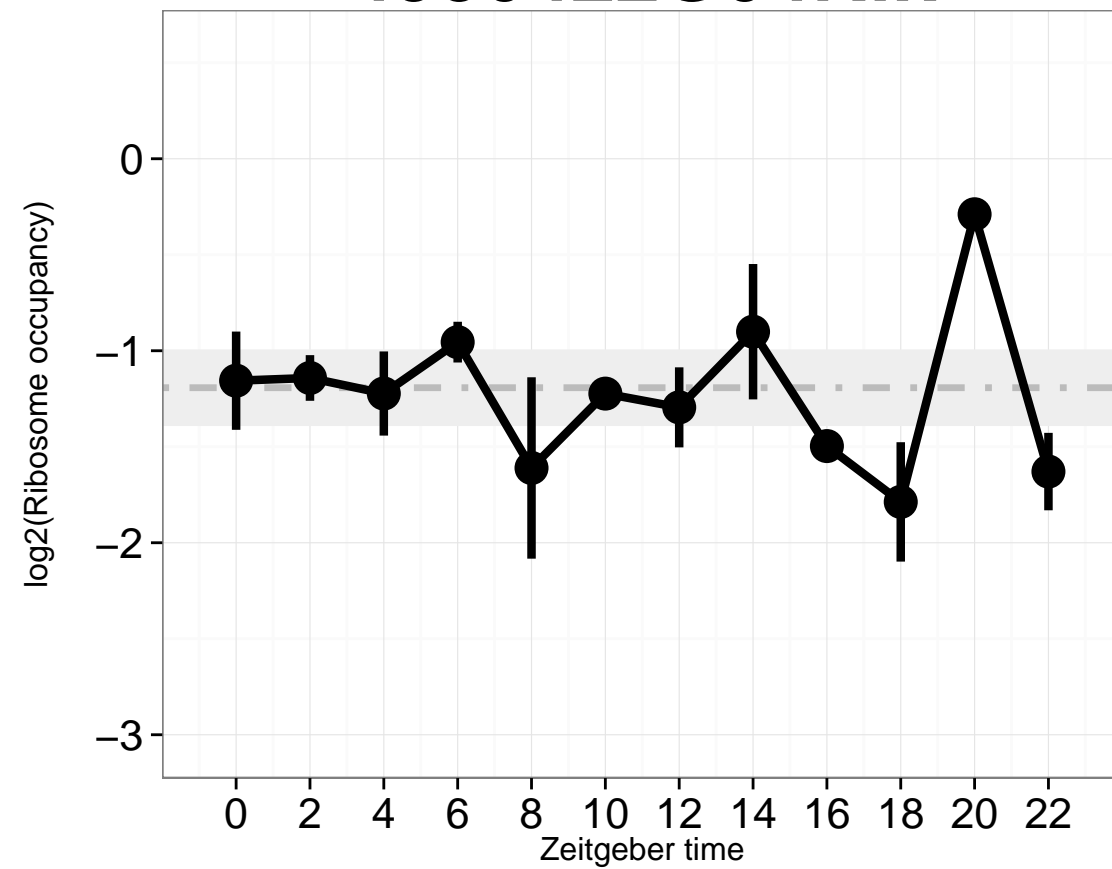

Supplement: Supplementary file 6 — Transcriptome-wide kidney RPF (blue) and RNA (orange) levels in the left panels (with “error bars” connecting the two replicates of each timepoint) and TE in the right panels. (ZIP 116896 kb) [file 13059_2017_1222_MOESM6_ESM.zip › Supp_Dataset_S1/A_RNA_non_rhythmic_RPF_non_rhythmic/4930422G04Rik_kidney_set_A.pdf]

4930423O20Rik

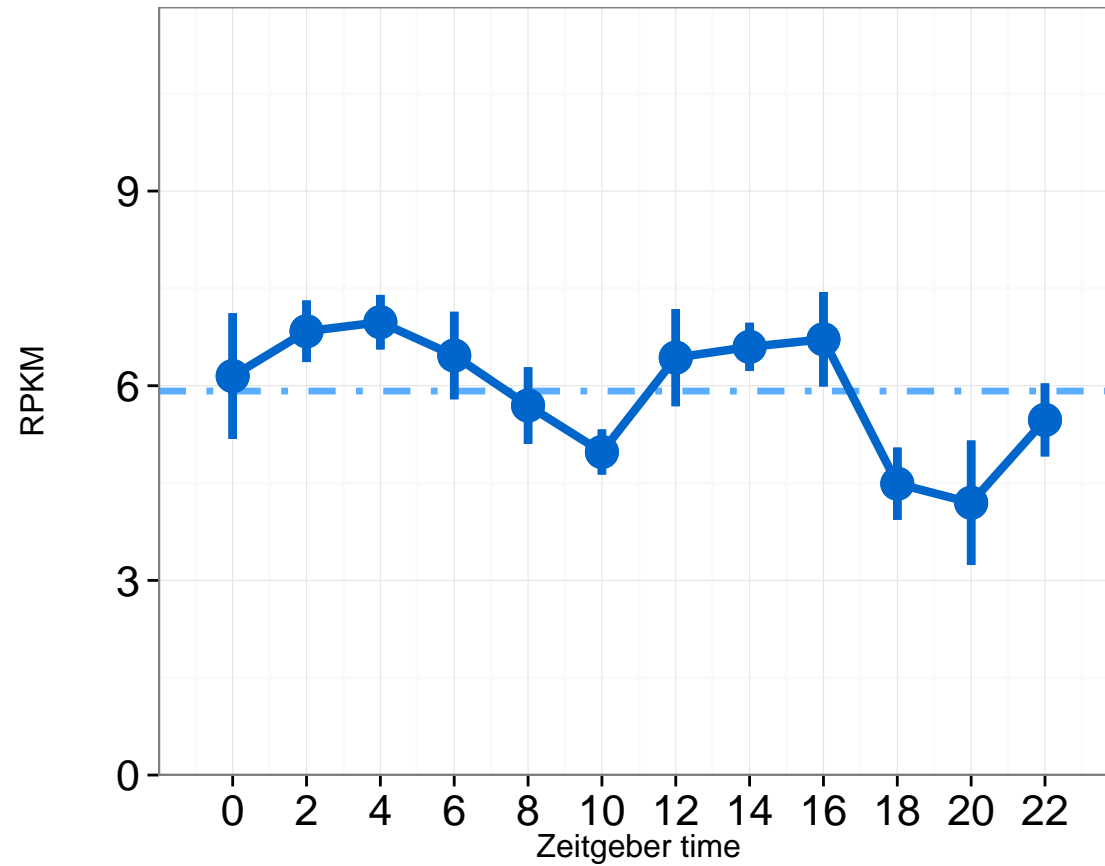

4930423O20Rik log<sub>2</sub>(Ribosome occup

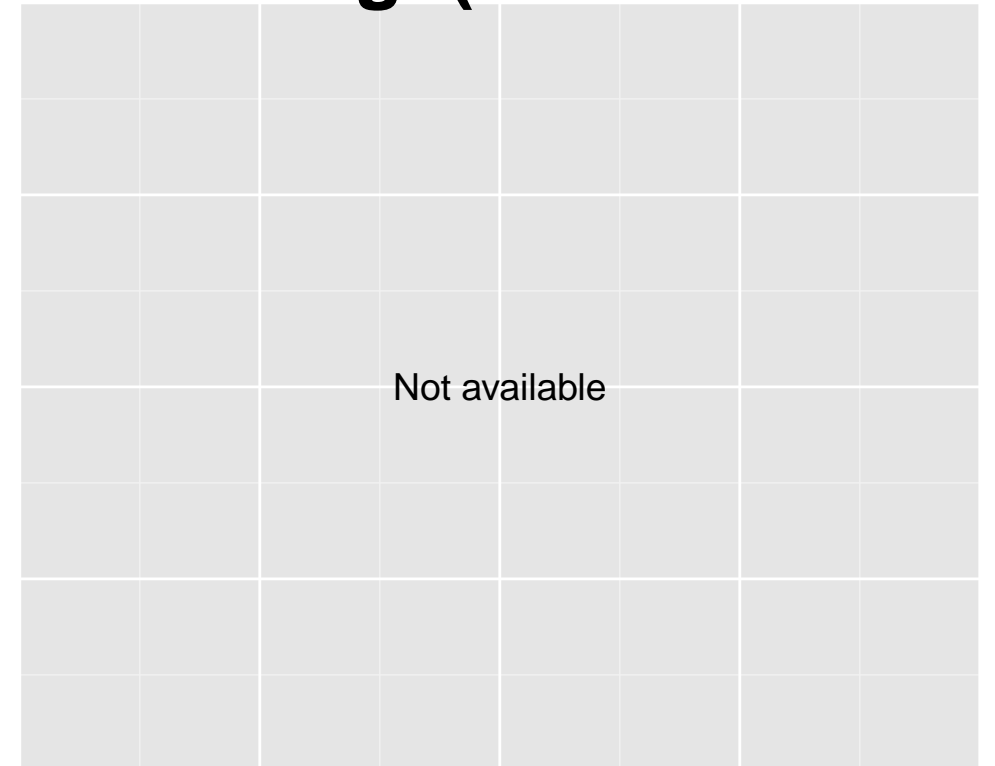

Supplement: Supplementary file 6 — Transcriptome-wide kidney RPF (blue) and RNA (orange) levels in the left panels (with “error bars” connecting the two replicates of each timepoint) and TE in the right panels. (ZIP 116896 kb) [file 13059_2017_1222_MOESM6_ESM.zip › Supp_Dataset_S1/A_RNA_non_rhythmic_RPF_non_rhythmic/4930423O20Rik_kidney_set_A.pdf]

4930425F17Rik

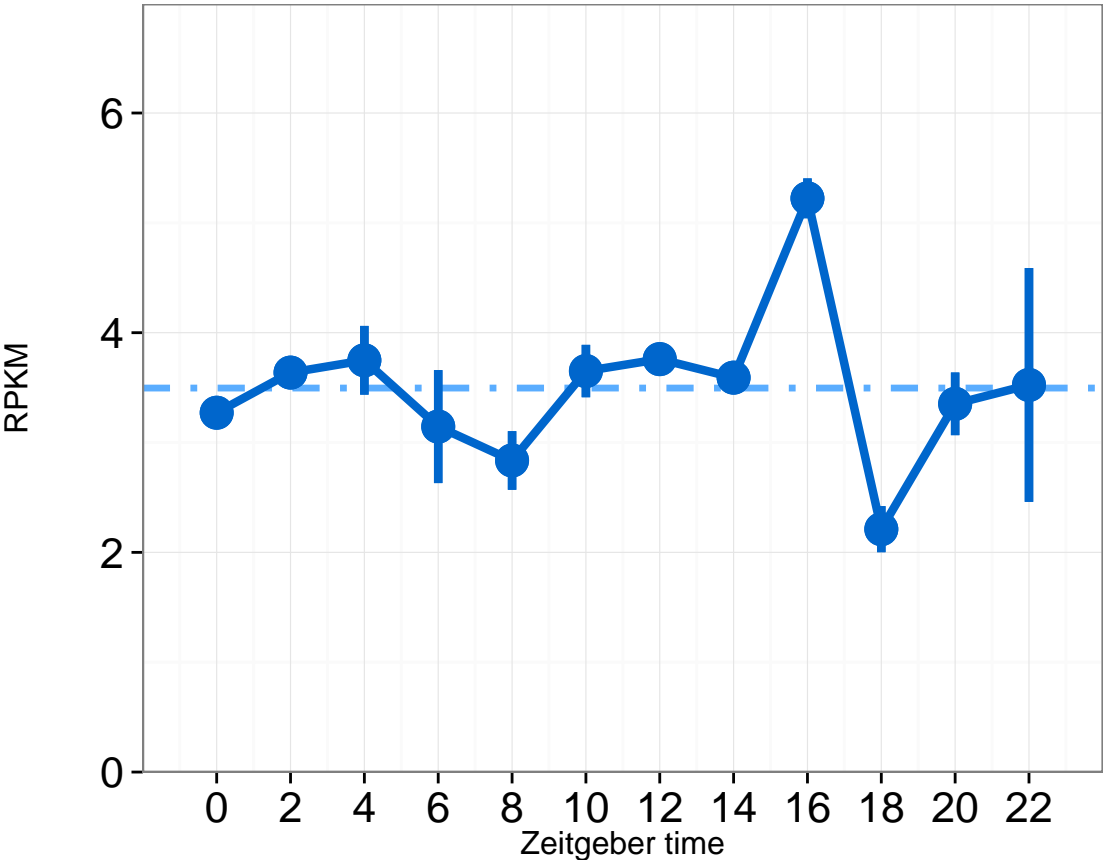

4930425F17Rik log2(Ribosome occupancy)

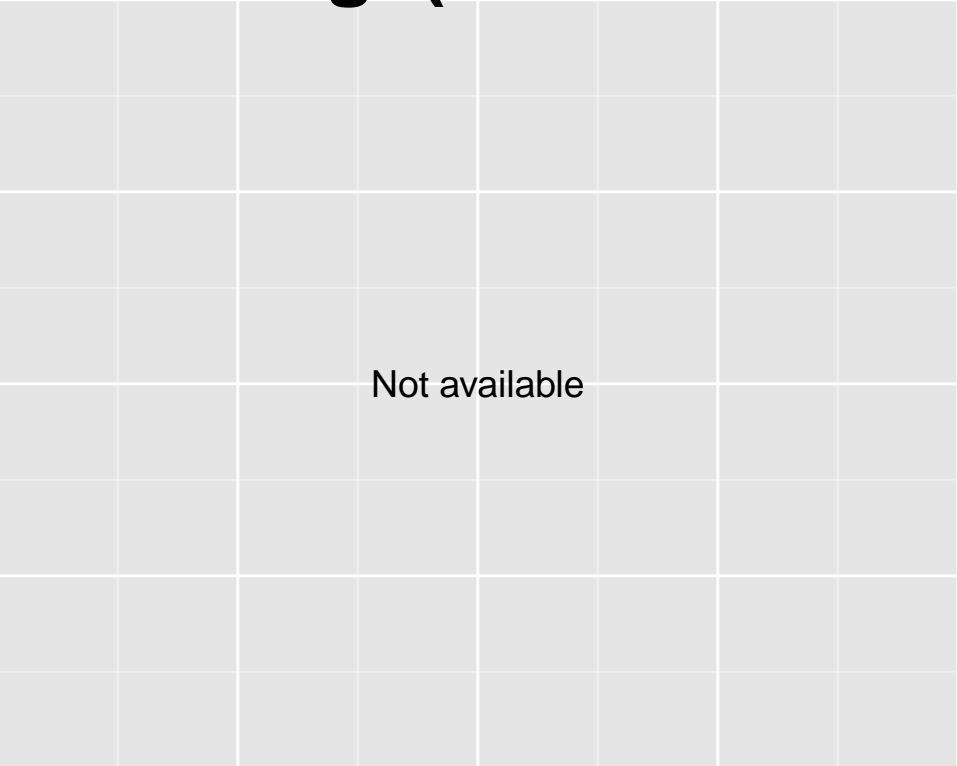

Supplement: Supplementary file 6 — Transcriptome-wide kidney RPF (blue) and RNA (orange) levels in the left panels (with “error bars” connecting the two replicates of each timepoint) and TE in the right panels. (ZIP 116896 kb) [file 13059_2017_1222_MOESM6_ESM.zip › Supp_Dataset_S1/A_RNA_non_rhythmic_RPF_non_rhythmic/4930425F17Rik_kidney_set_A.pdf]

# 4930427A07Rik

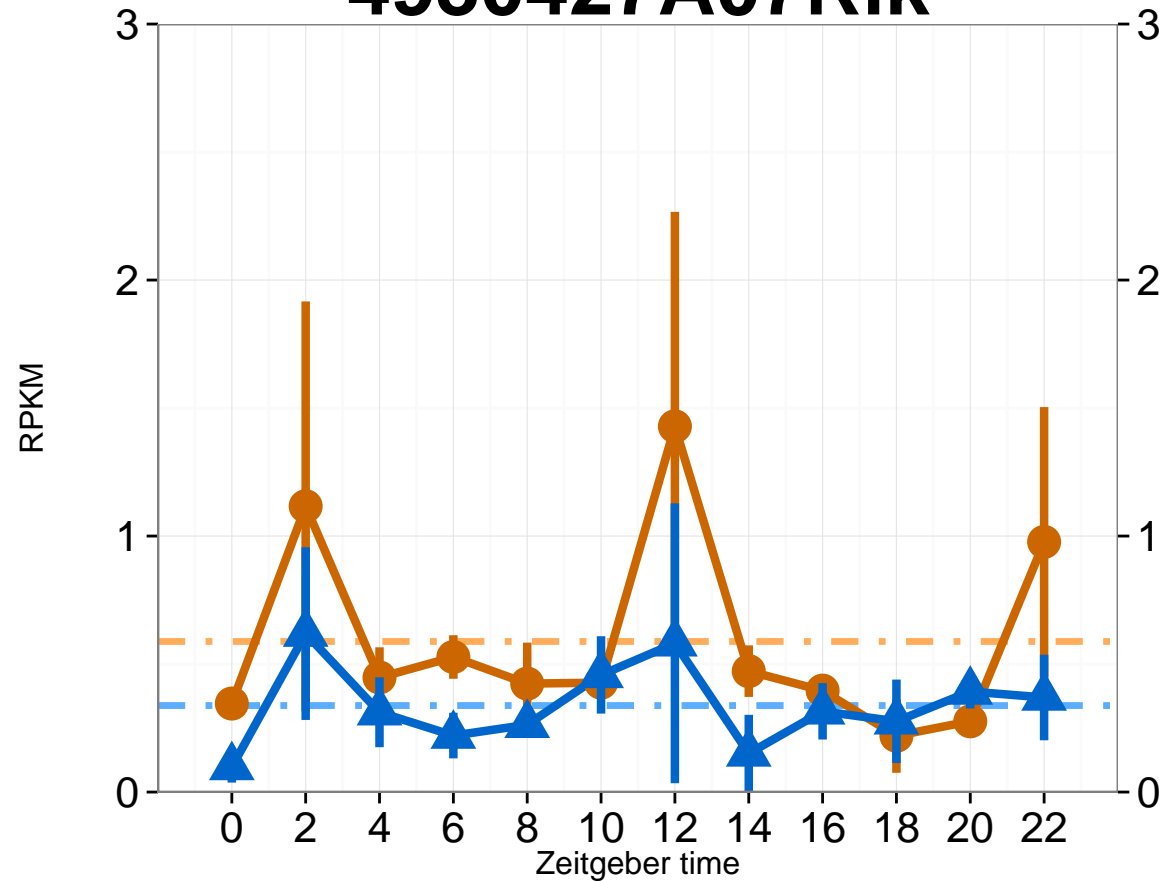

# 4930427A07Rik

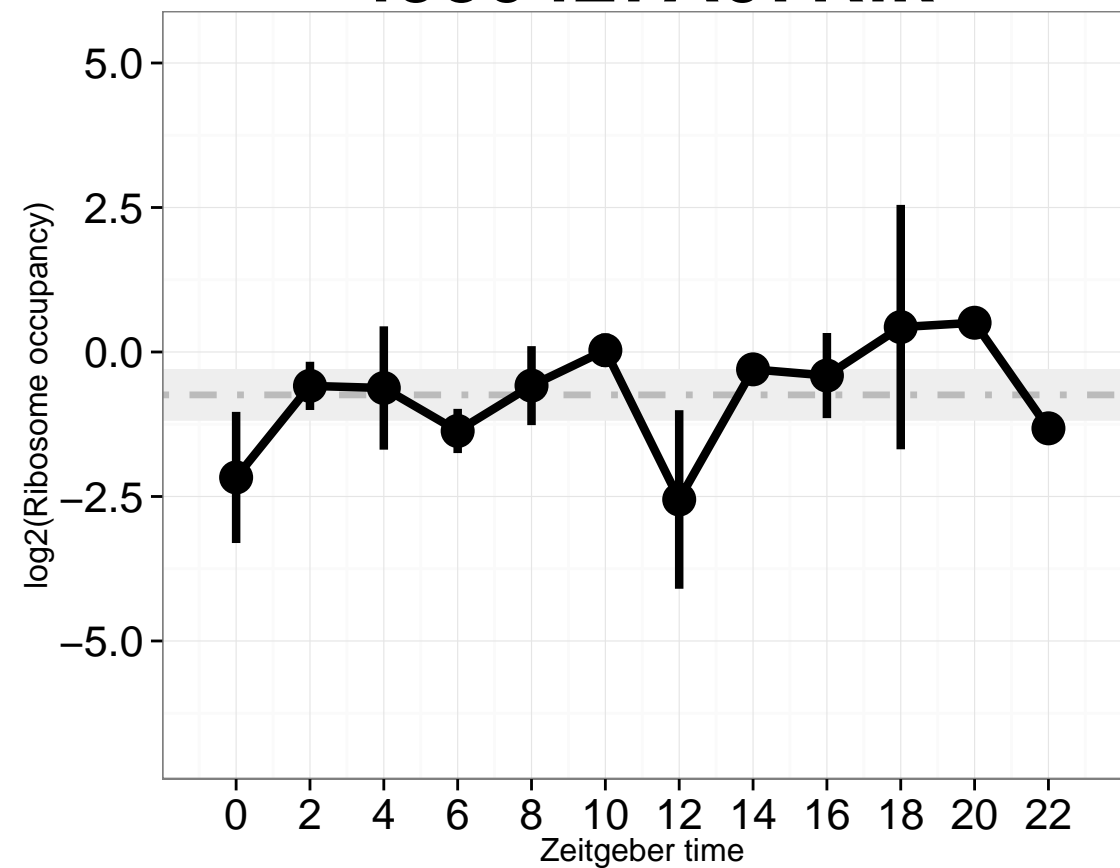

Supplement: Supplementary file 6 — Transcriptome-wide kidney RPF (blue) and RNA (orange) levels in the left panels (with “error bars” connecting the two replicates of each timepoint) and TE in the right panels. (ZIP 116896 kb) [file 13059_2017_1222_MOESM6_ESM.zip › Supp_Dataset_S1/A_RNA_non_rhythmic_RPF_non_rhythmic/4930427A07Rik_kidney_set_A.pdf]

## 4930430F08Rik

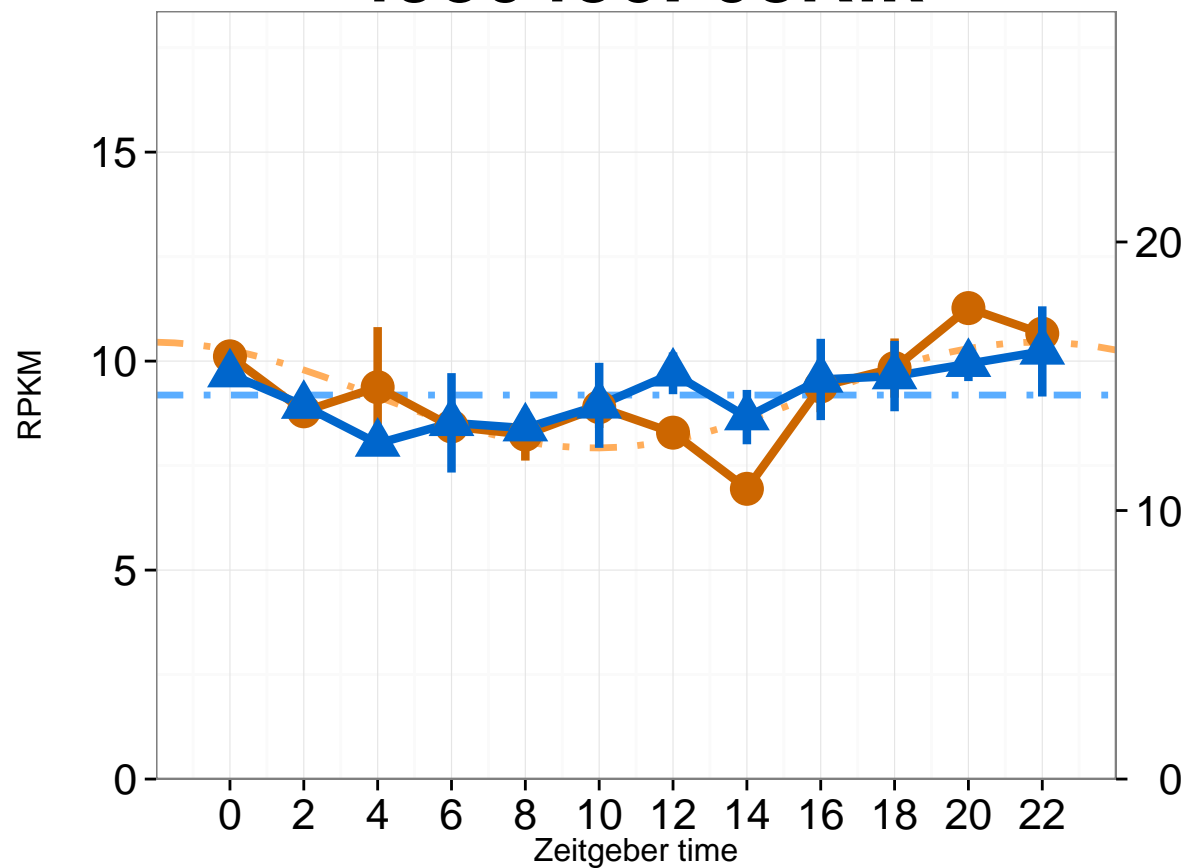

## 4930430F08Rik

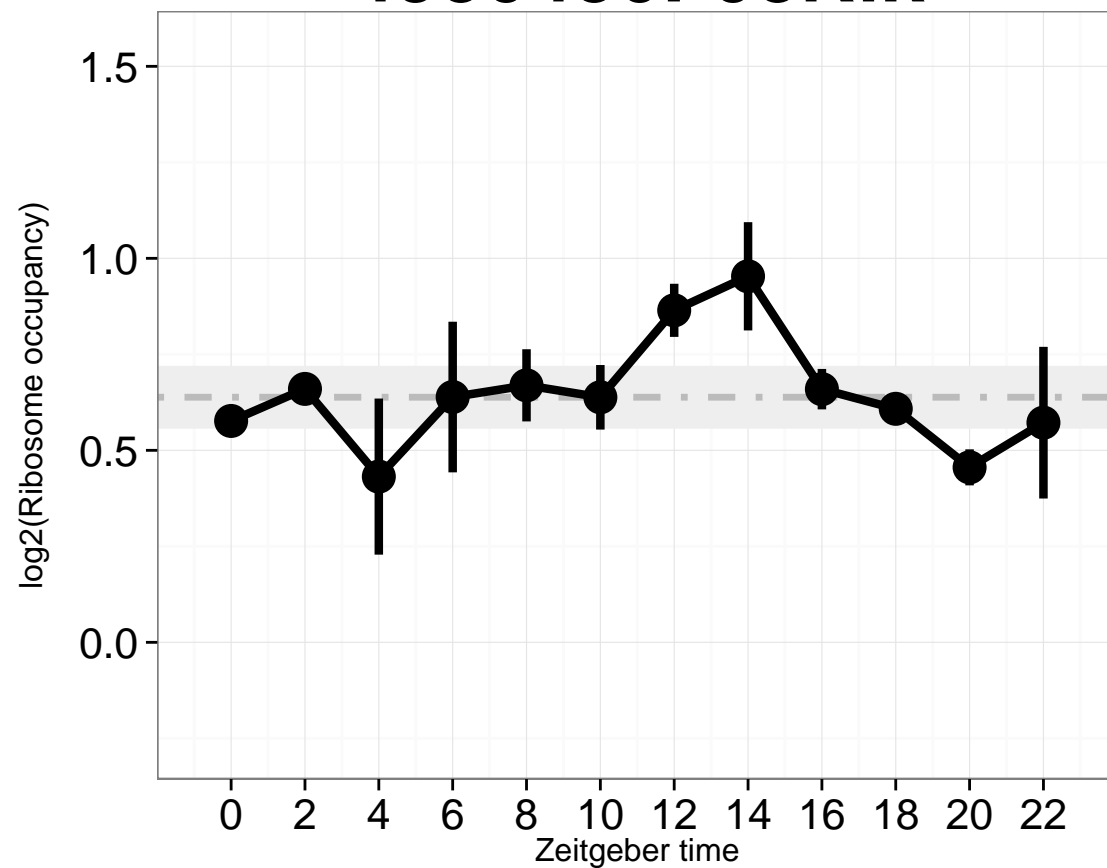

Supplement: Supplementary file 6 — Transcriptome-wide kidney RPF (blue) and RNA (orange) levels in the left panels (with “error bars” connecting the two replicates of each timepoint) and TE in the right panels. (ZIP 116896 kb) [file 13059_2017_1222_MOESM6_ESM.zip › Supp_Dataset_S1/A_RNA_non_rhythmic_RPF_non_rhythmic/4930430F08Rik_kidney_set_A.pdf]

4930431F12Rik

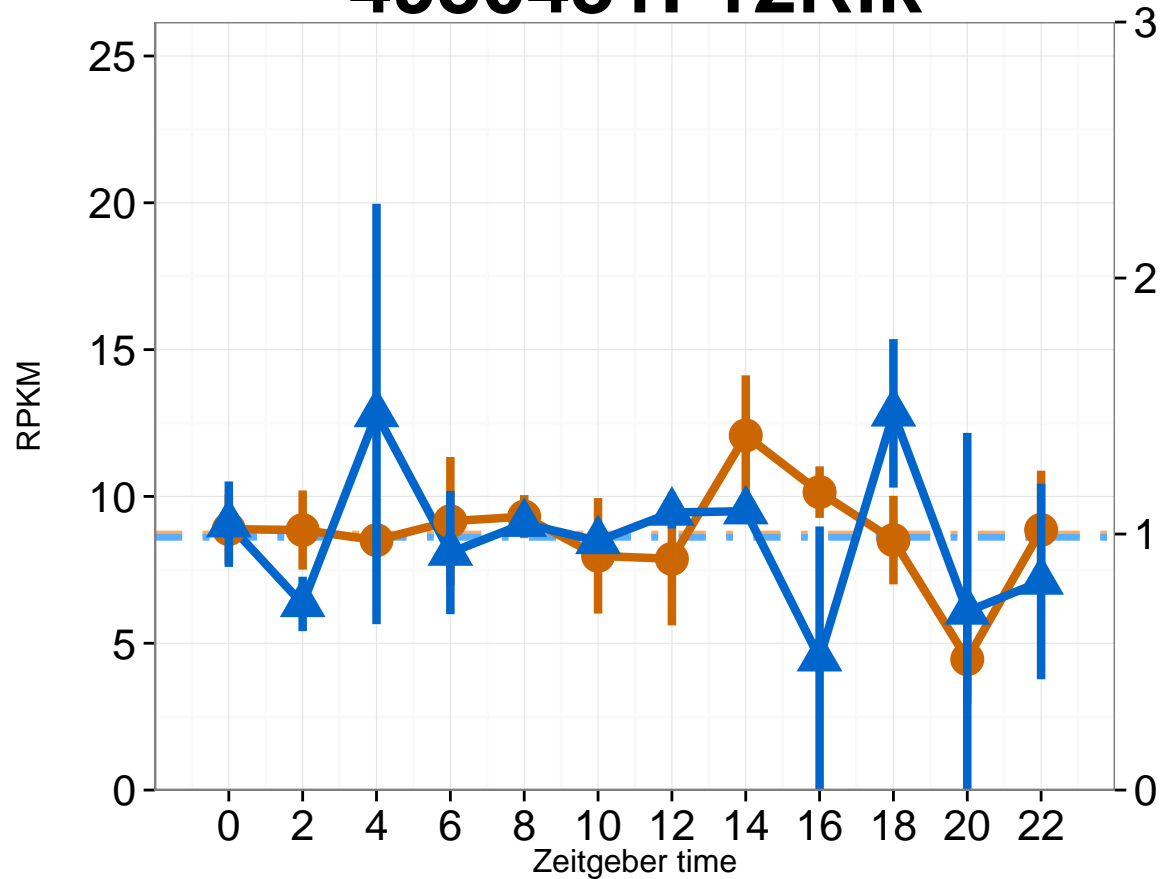

4930431F12Rik

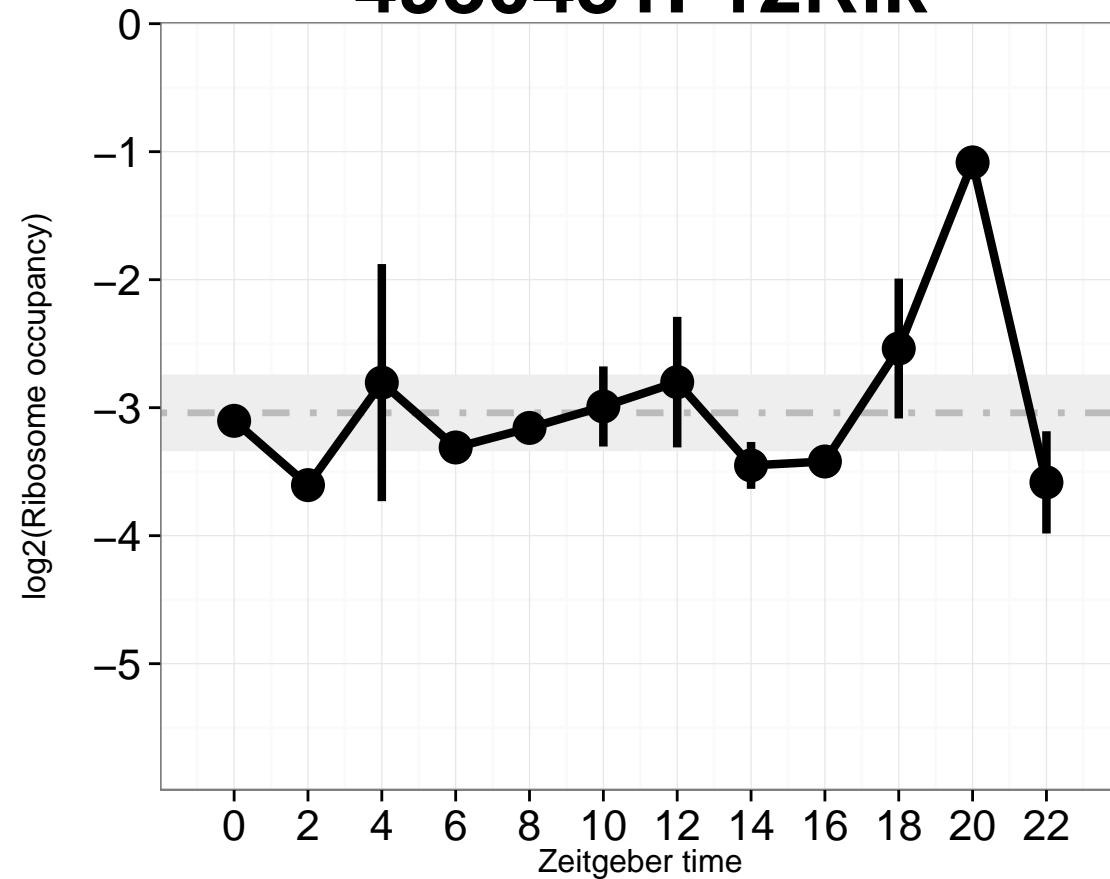

Supplement: Supplementary file 6 — Transcriptome-wide kidney RPF (blue) and RNA (orange) levels in the left panels (with “error bars” connecting the two replicates of each timepoint) and TE in the right panels. (ZIP 116896 kb) [file 13059_2017_1222_MOESM6_ESM.zip › Supp_Dataset_S1/A_RNA_non_rhythmic_RPF_non_rhythmic/4930431F12Rik_kidney_set_A.pdf]

## 4930451 G09Rik

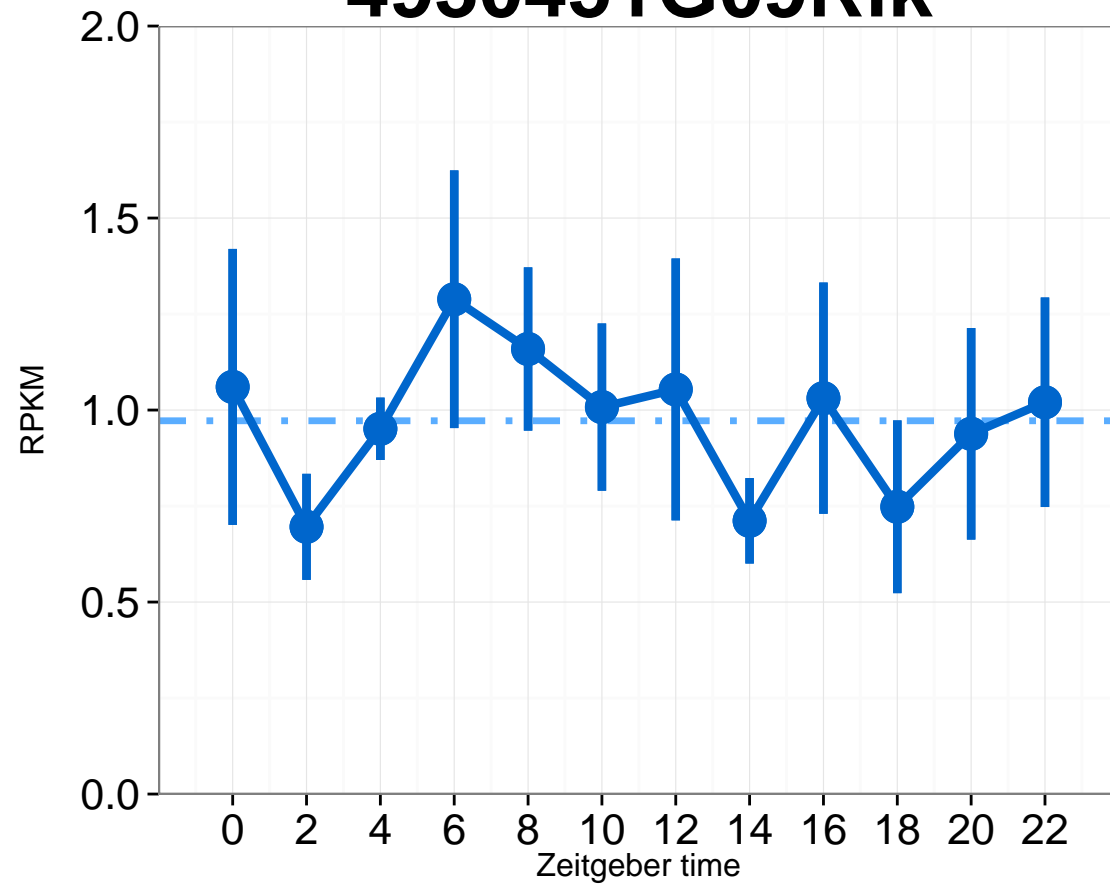

## 4930451 G09Rik log2(Ribosome occup

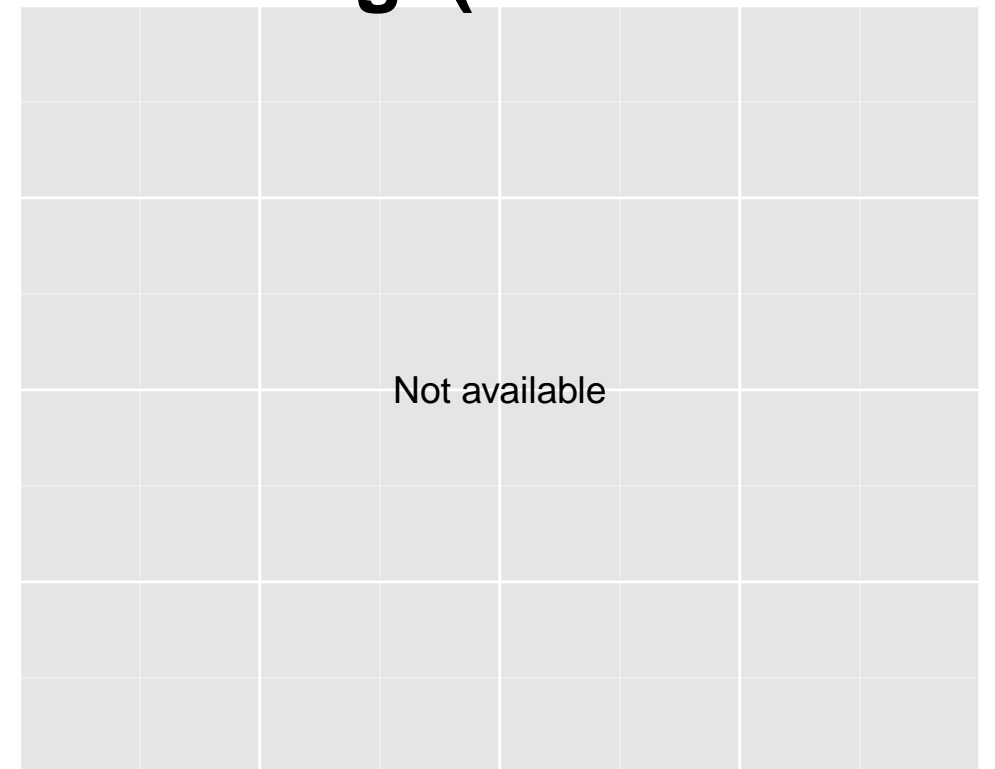

Supplement: Supplementary file 6 — Transcriptome-wide kidney RPF (blue) and RNA (orange) levels in the left panels (with “error bars” connecting the two replicates of each timepoint) and TE in the right panels. (ZIP 116896 kb) [file 13059_2017_1222_MOESM6_ESM.zip › Supp_Dataset_S1/A_RNA_non_rhythmic_RPF_non_rhythmic/4930451G09Rik_kidney_set_A.pdf]

# 4930453N24Rik

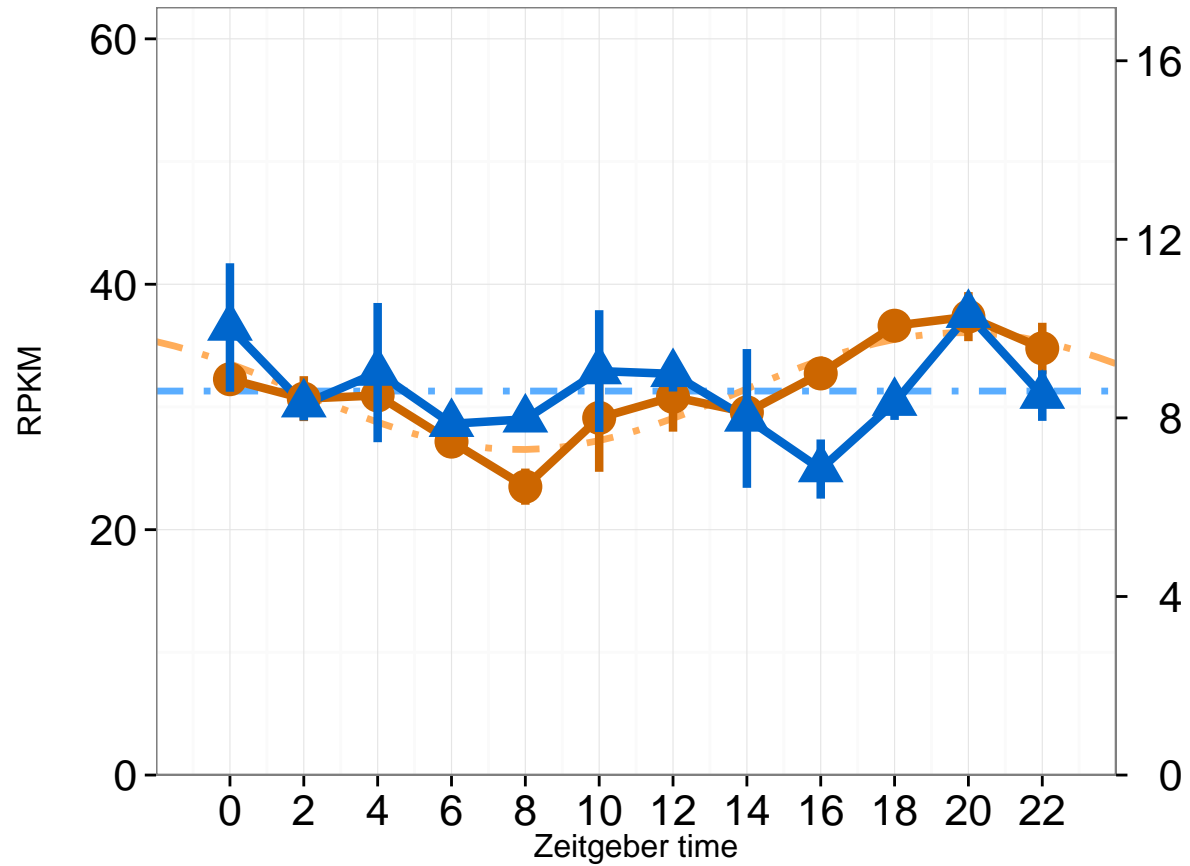

# 4930453N24Rik

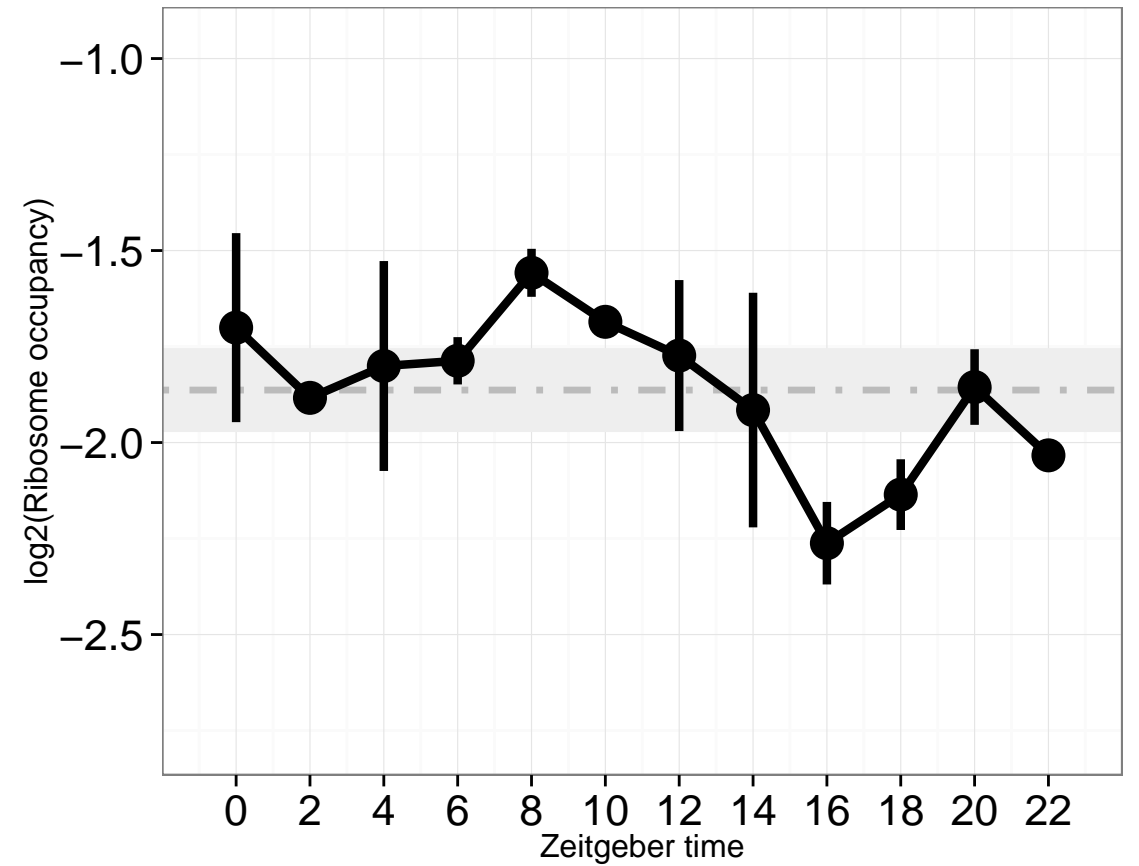

Supplement: Supplementary file 6 — Transcriptome-wide kidney RPF (blue) and RNA (orange) levels in the left panels (with “error bars” connecting the two replicates of each timepoint) and TE in the right panels. (ZIP 116896 kb) [file 13059_2017_1222_MOESM6_ESM.zip › Supp_Dataset_S1/A_RNA_non_rhythmic_RPF_non_rhythmic/4930453N24Rik_kidney_set_A.pdf]

# 4930502E18Rik

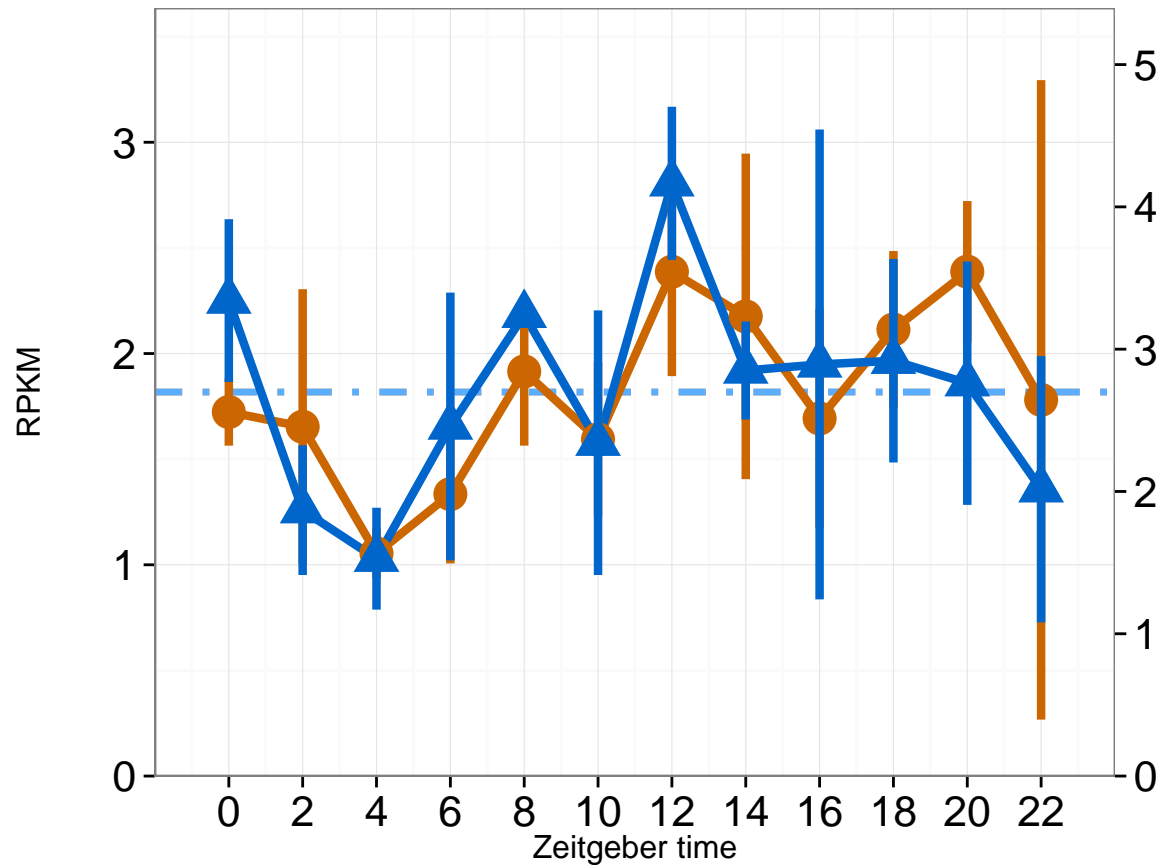

# 4930502E18Rik

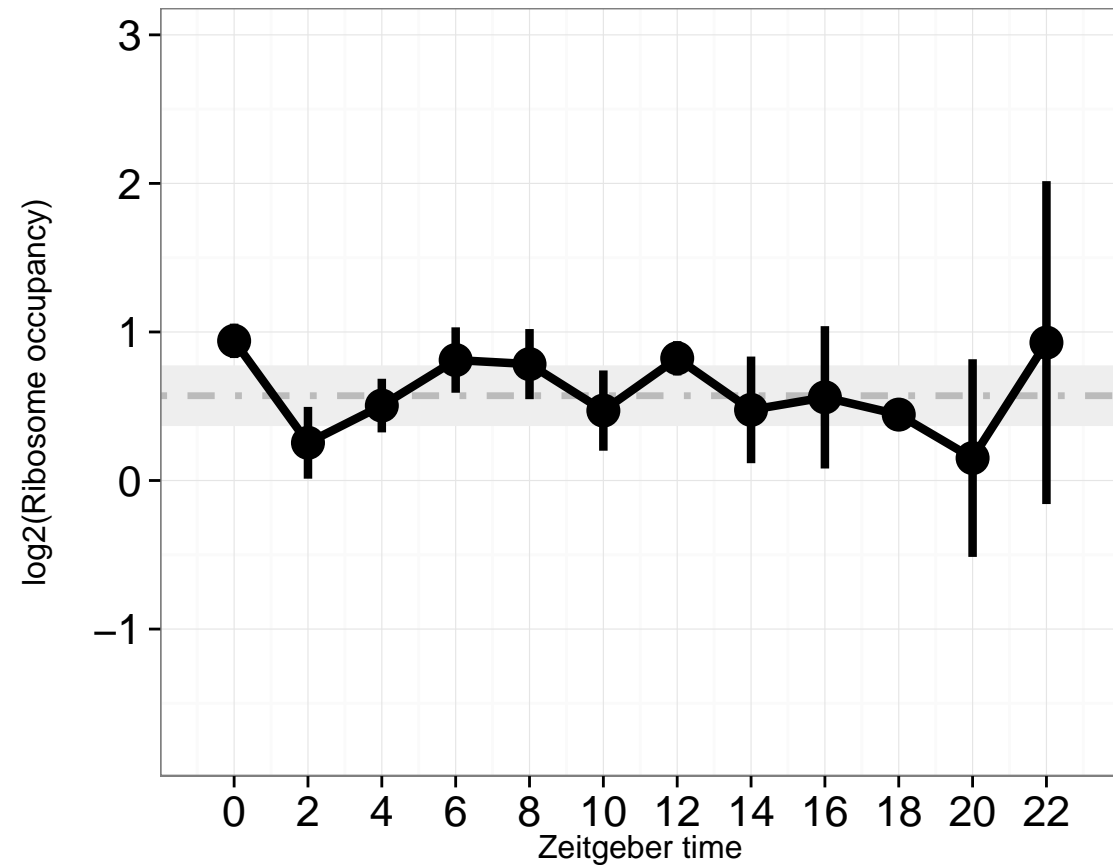

Supplement: Supplementary file 6 — Transcriptome-wide kidney RPF (blue) and RNA (orange) levels in the left panels (with “error bars” connecting the two replicates of each timepoint) and TE in the right panels. (ZIP 116896 kb) [file 13059_2017_1222_MOESM6_ESM.zip › Supp_Dataset_S1/A_RNA_non_rhythmic_RPF_non_rhythmic/4930502E18Rik_kidney_set_A.pdf]

## 4930503L19Rik

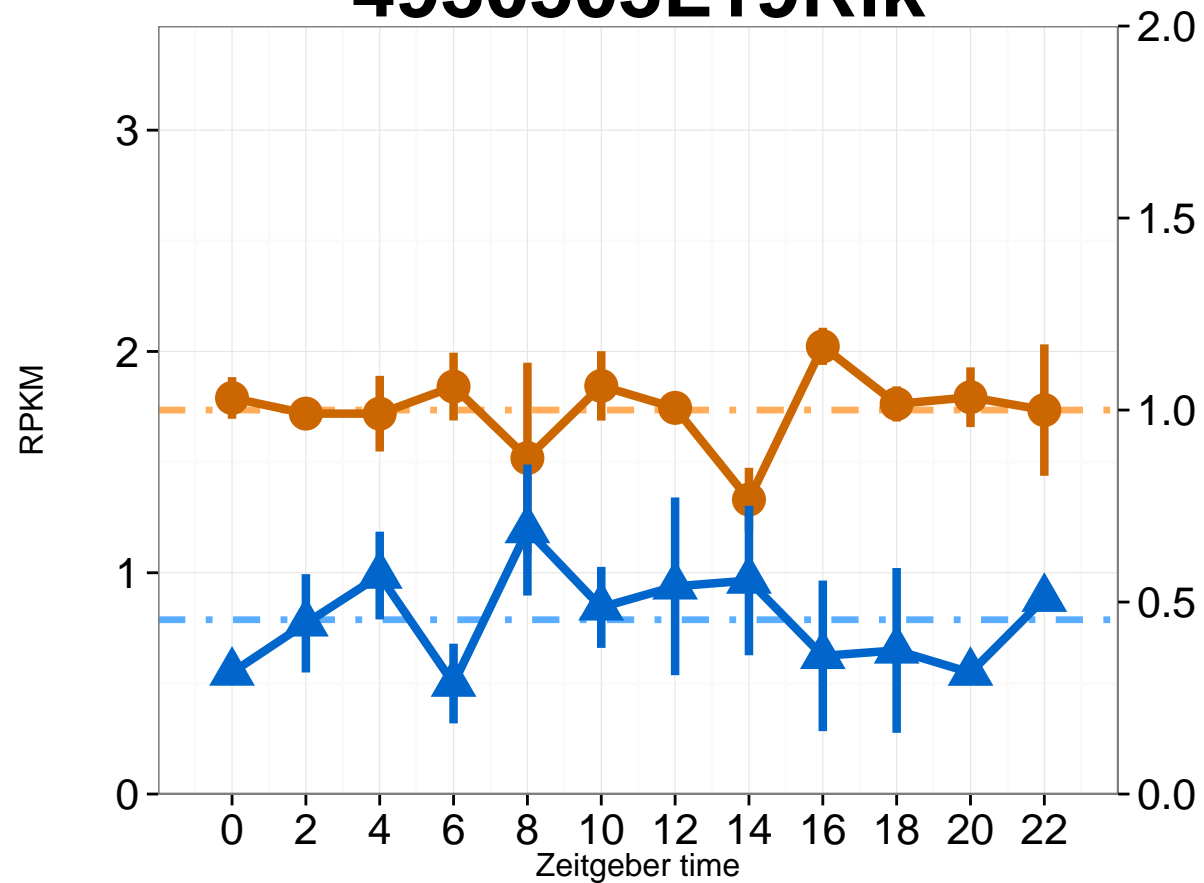

## 4930503L19Rik

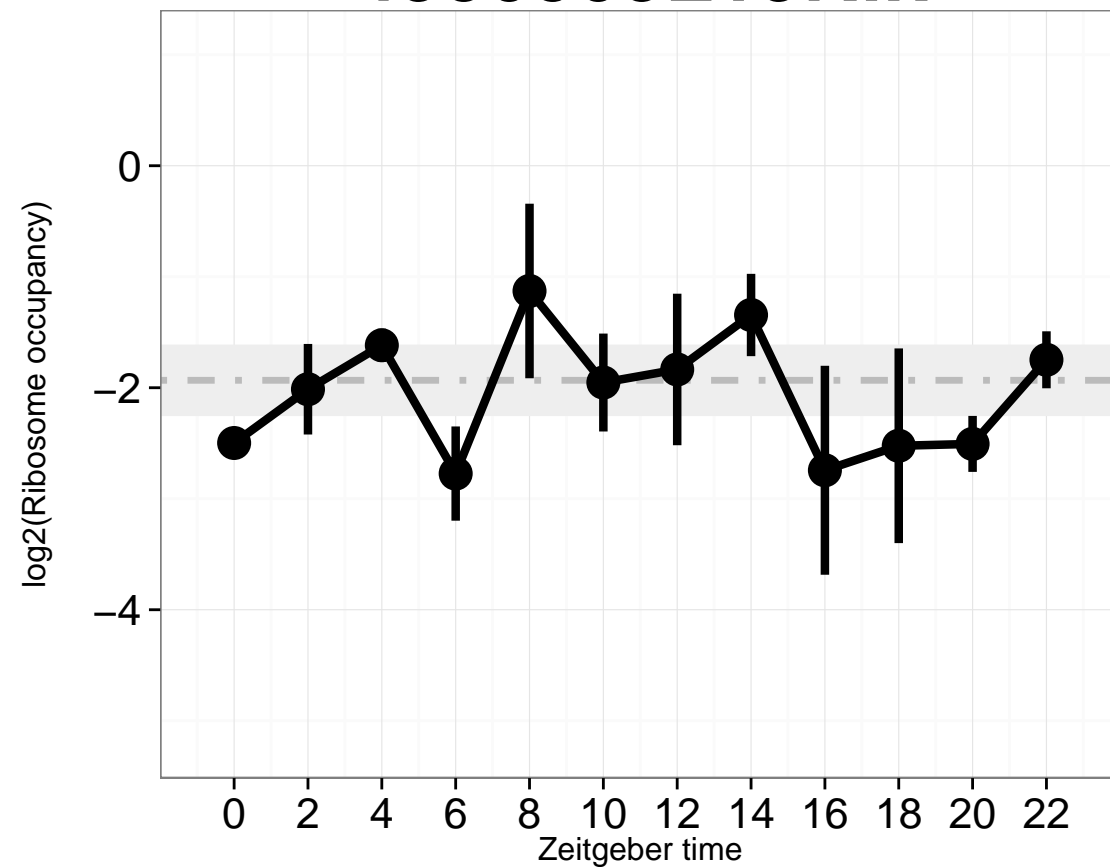

Supplement: Supplementary file 6 — Transcriptome-wide kidney RPF (blue) and RNA (orange) levels in the left panels (with “error bars” connecting the two replicates of each timepoint) and TE in the right panels. (ZIP 116896 kb) [file 13059_2017_1222_MOESM6_ESM.zip › Supp_Dataset_S1/A_RNA_non_rhythmic_RPF_non_rhythmic/4930503L19Rik_kidney_set_A.pdf]

# 4930523C07Rik

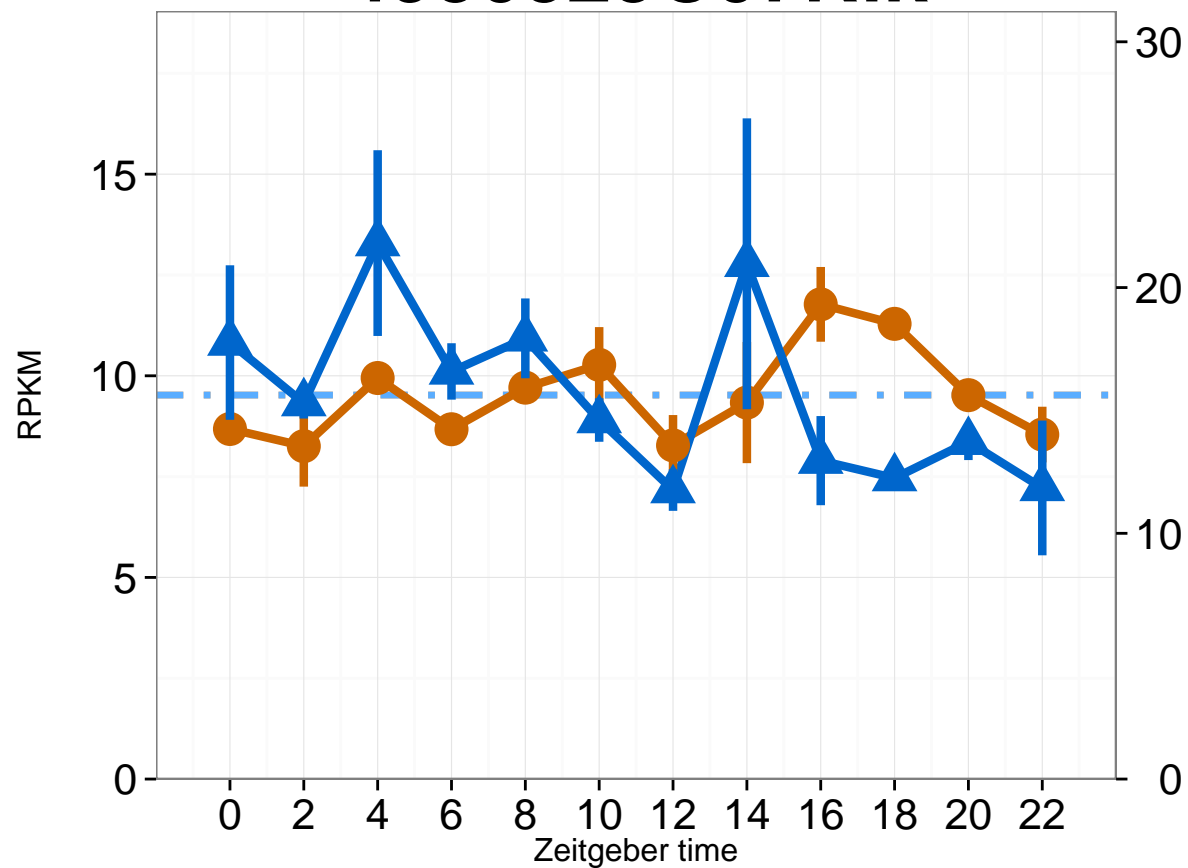

# 4930523C07Rik

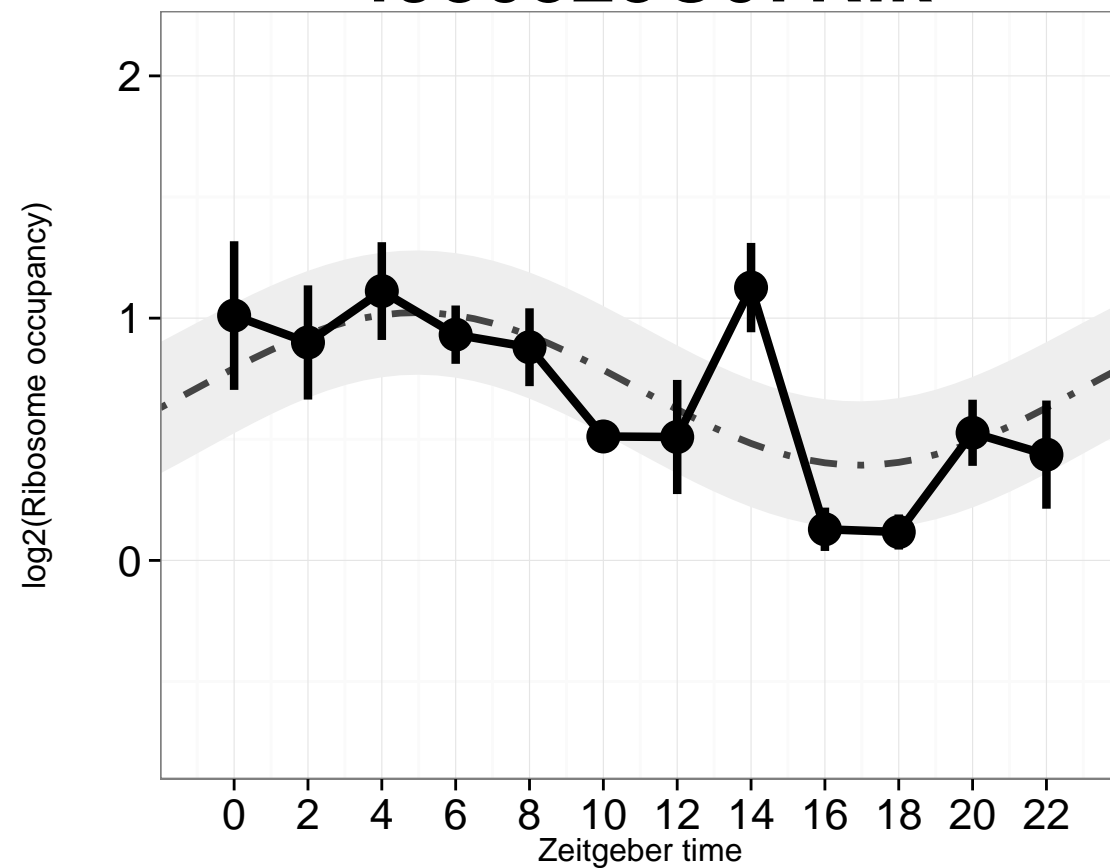

Supplement: Supplementary file 6 — Transcriptome-wide kidney RPF (blue) and RNA (orange) levels in the left panels (with “error bars” connecting the two replicates of each timepoint) and TE in the right panels. (ZIP 116896 kb) [file 13059_2017_1222_MOESM6_ESM.zip › Supp_Dataset_S1/A_RNA_non_rhythmic_RPF_non_rhythmic/4930523C07Rik_kidney_set_A.pdf]

## 4930546H06Rik

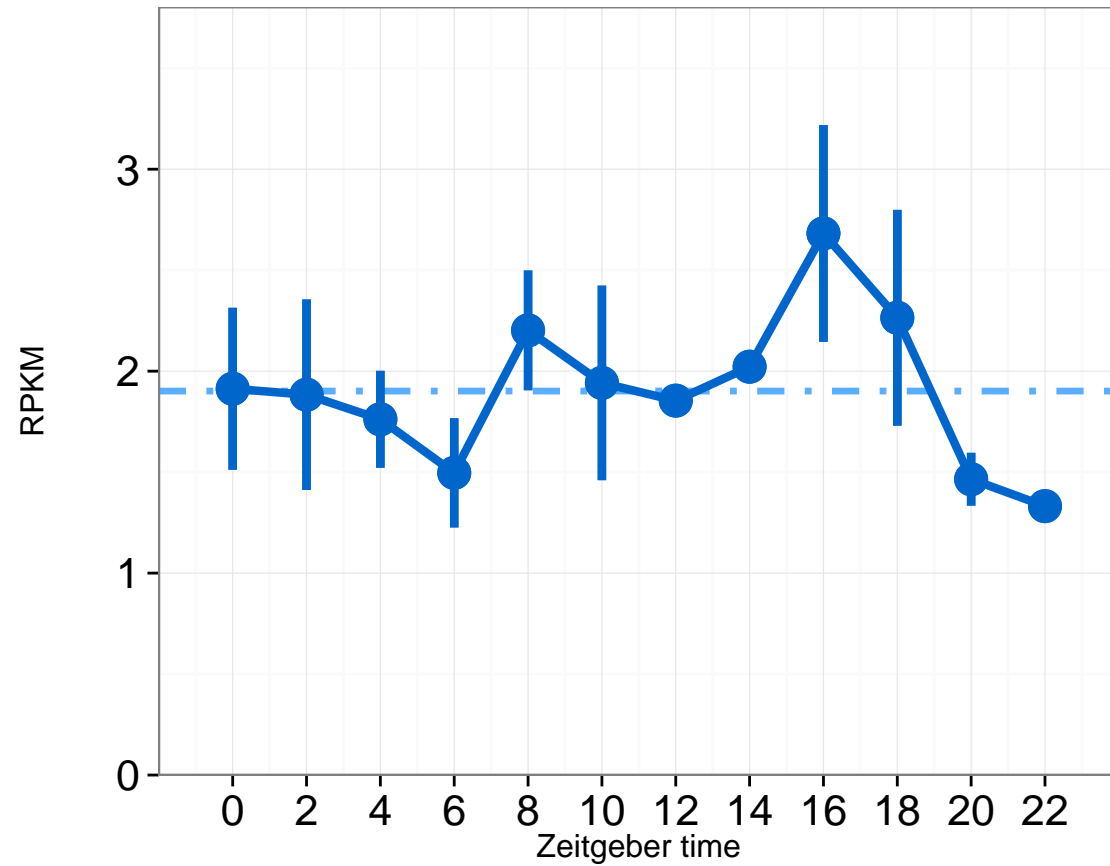

## 4930546H06Rik log2(Ribosome occup

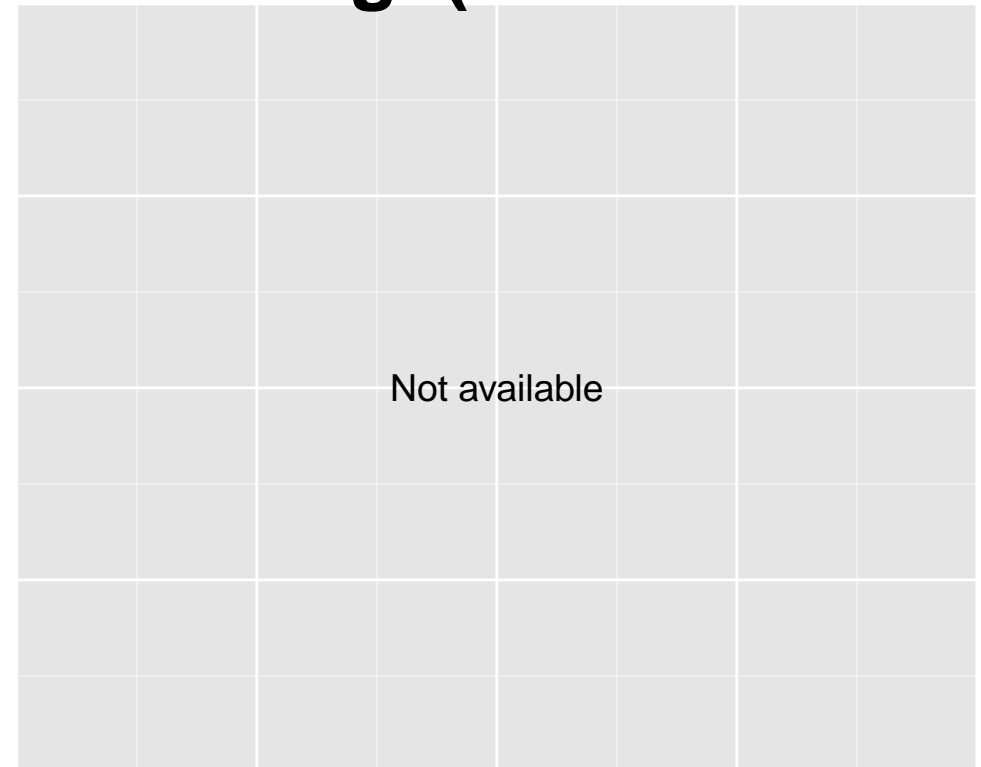

Supplement: Supplementary file 6 — Transcriptome-wide kidney RPF (blue) and RNA (orange) levels in the left panels (with “error bars” connecting the two replicates of each timepoint) and TE in the right panels. (ZIP 116896 kb) [file 13059_2017_1222_MOESM6_ESM.zip › Supp_Dataset_S1/A_RNA_non_rhythmic_RPF_non_rhythmic/4930546H06Rik_kidney_set_A.pdf]

## 4930562C15Rik

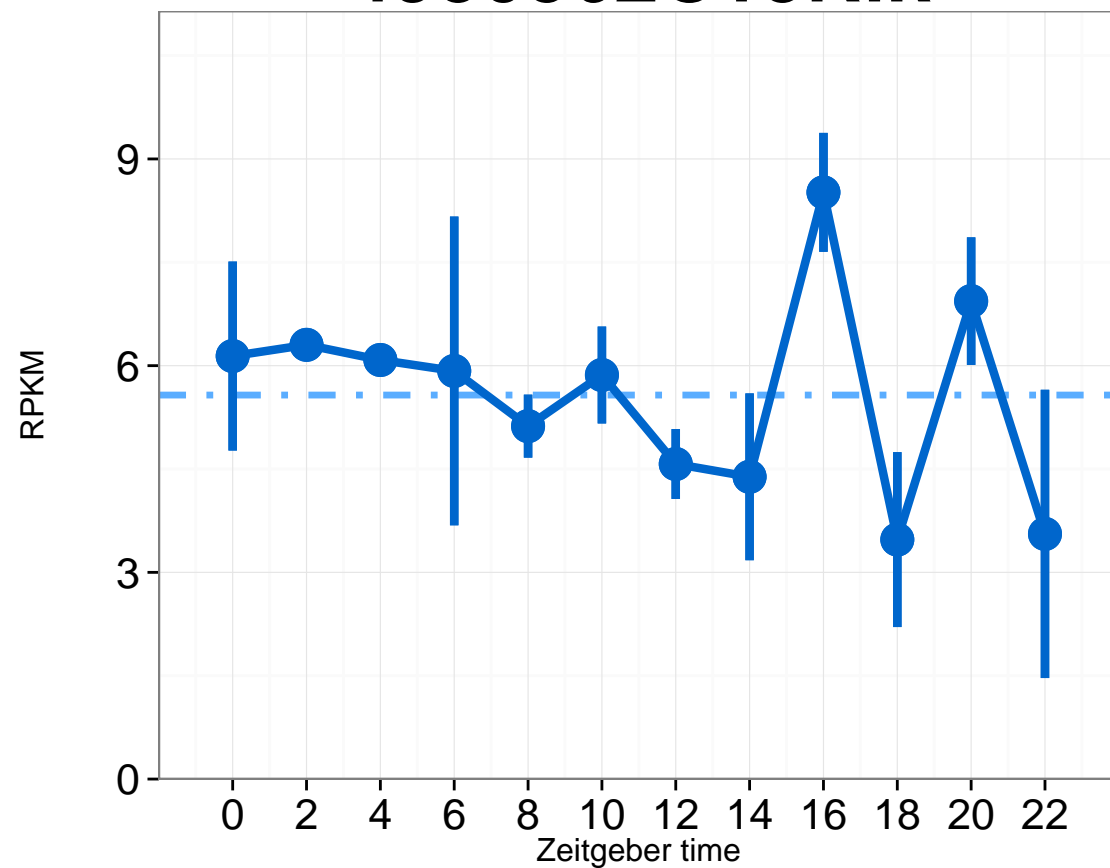

## 4930562C15Rik log2(Ribosome occup

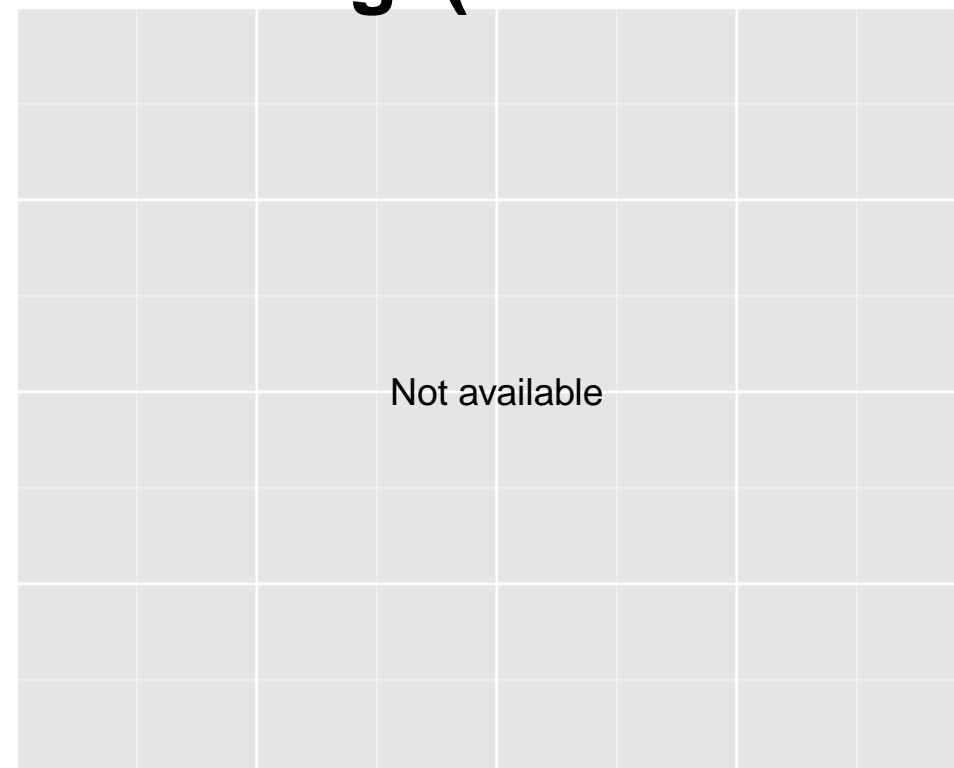

Supplement: Supplementary file 6 — Transcriptome-wide kidney RPF (blue) and RNA (orange) levels in the left panels (with “error bars” connecting the two replicates of each timepoint) and TE in the right panels. (ZIP 116896 kb) [file 13059_2017_1222_MOESM6_ESM.zip › Supp_Dataset_S1/A_RNA_non_rhythmic_RPF_non_rhythmic/4930562C15Rik_kidney_set_A.pdf]

## 4930579K19Rik

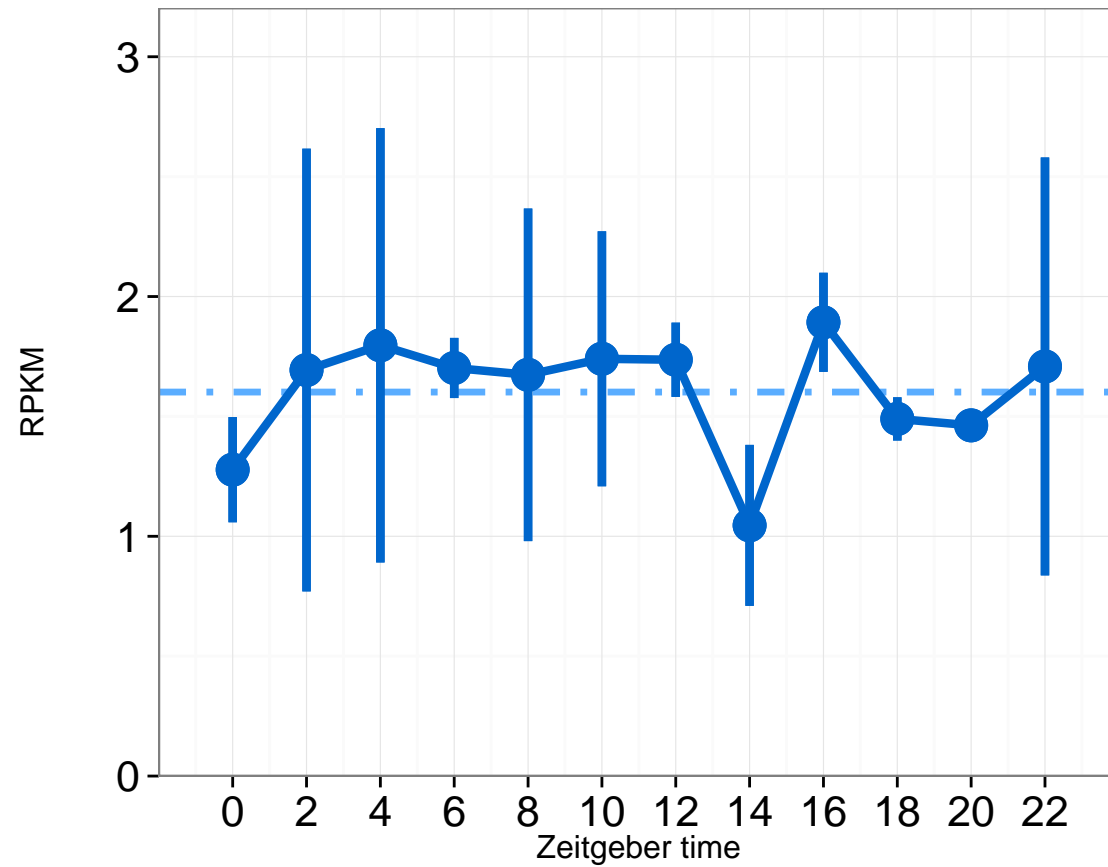

## 4930579K19Rik log2(Ribosome occup

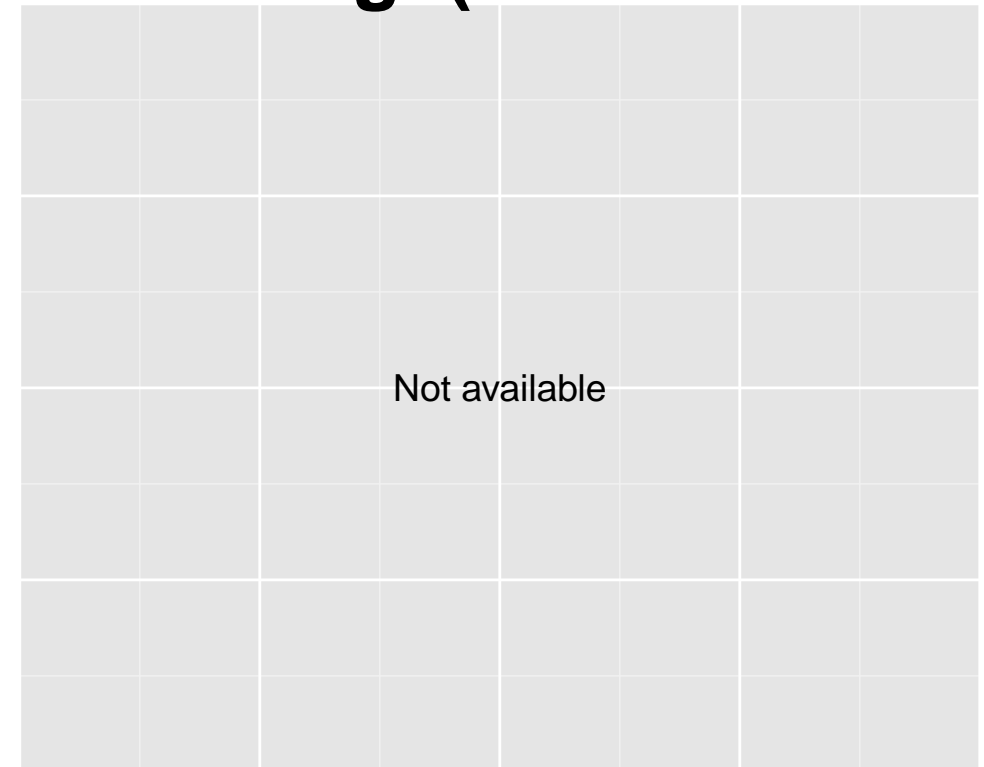

Supplement: Supplementary file 6 — Transcriptome-wide kidney RPF (blue) and RNA (orange) levels in the left panels (with “error bars” connecting the two replicates of each timepoint) and TE in the right panels. (ZIP 116896 kb) [file 13059_2017_1222_MOESM6_ESM.zip › Supp_Dataset_S1/A_RNA_non_rhythmic_RPF_non_rhythmic/4930579K19Rik_kidney_set_A.pdf]

## 4931406C07Rik

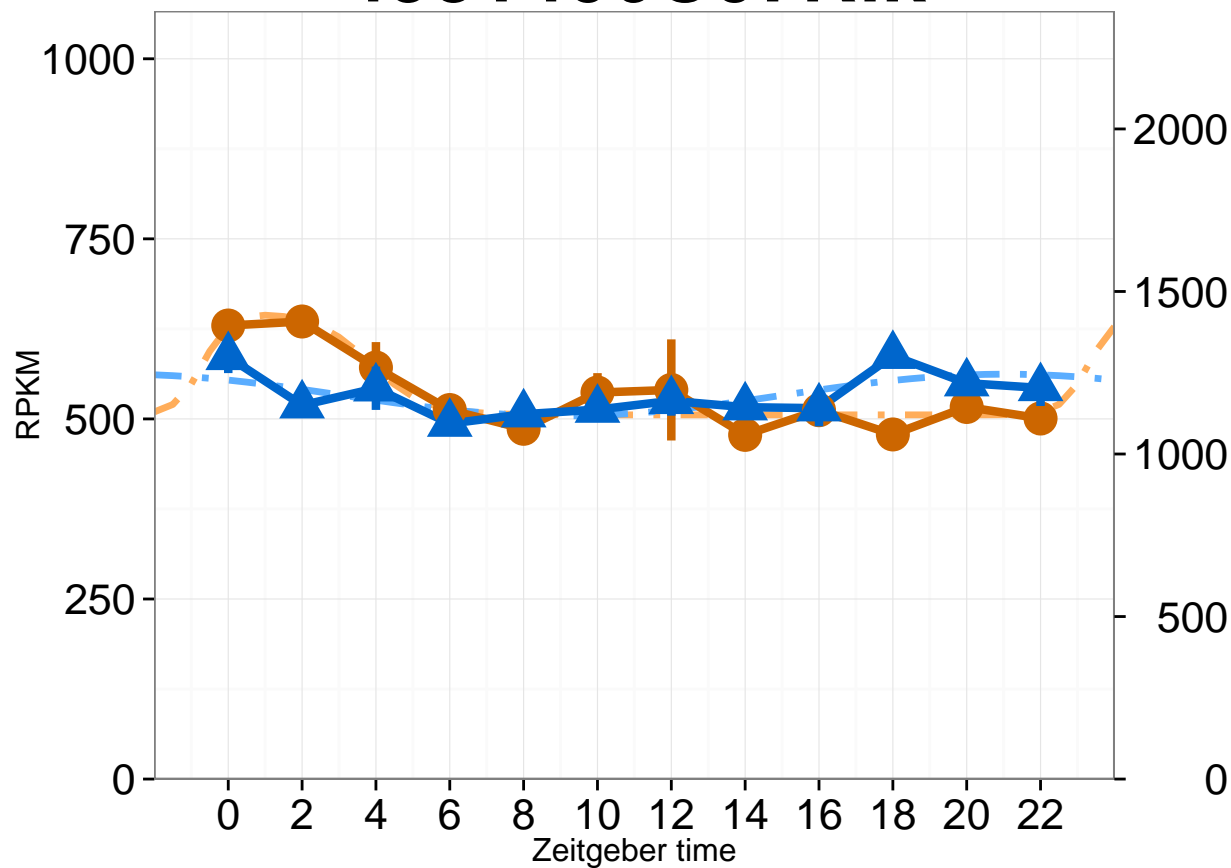

## 4931406C07Rik

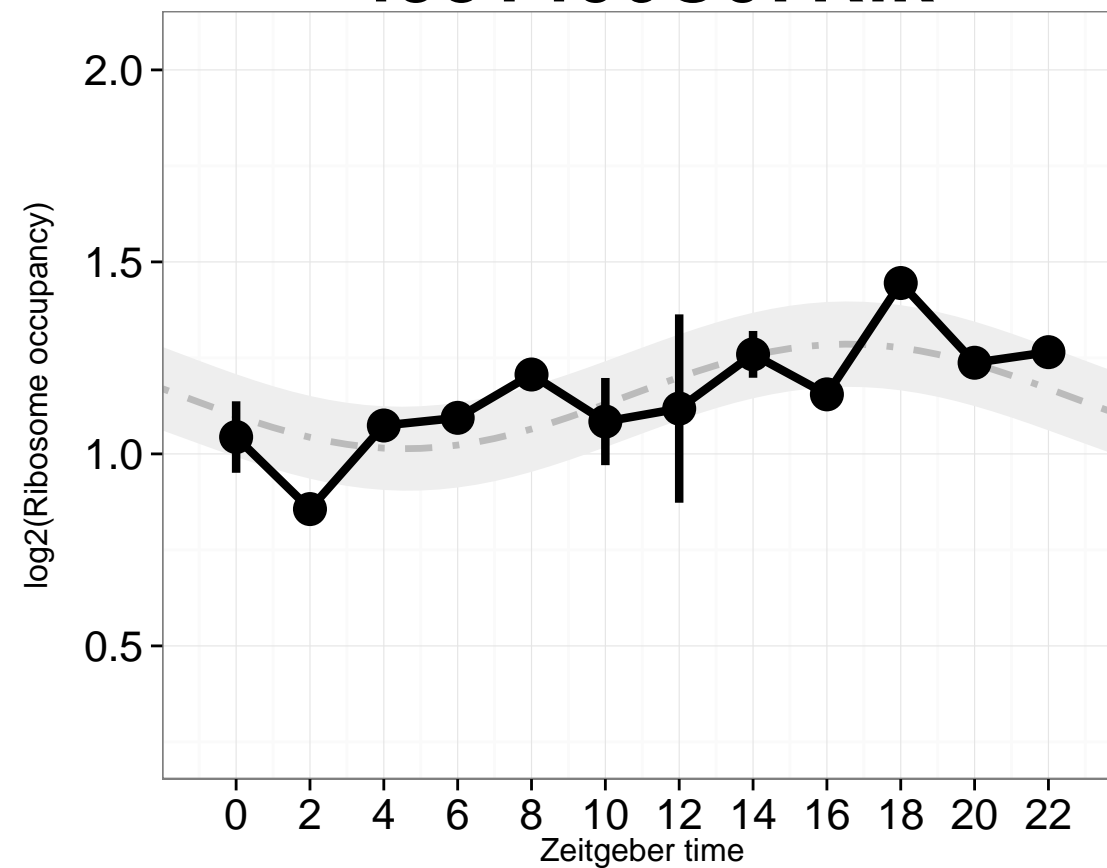

Supplement: Supplementary file 6 — Transcriptome-wide kidney RPF (blue) and RNA (orange) levels in the left panels (with “error bars” connecting the two replicates of each timepoint) and TE in the right panels. (ZIP 116896 kb) [file 13059_2017_1222_MOESM6_ESM.zip › Supp_Dataset_S1/A_RNA_non_rhythmic_RPF_non_rhythmic/4931406C07Rik_kidney_set_A.pdf]

# 4931406P16Rik

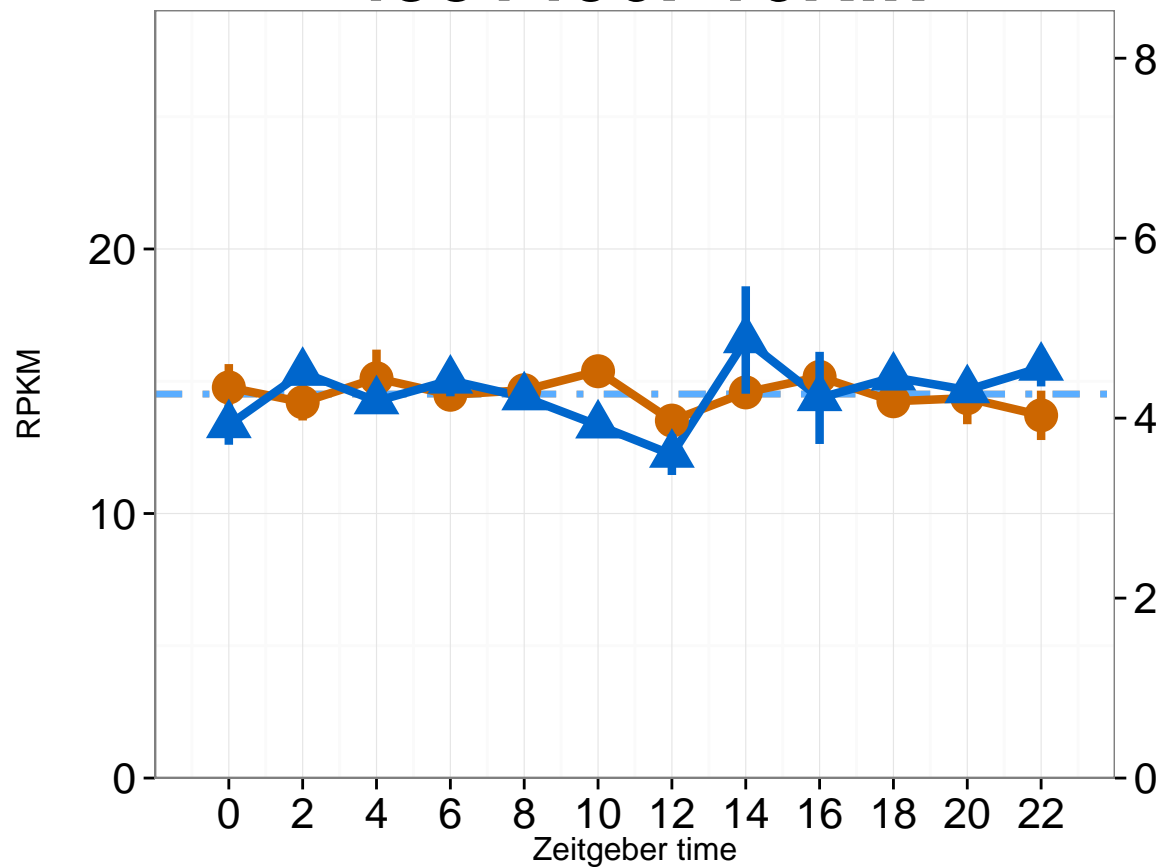

# 4931406P16Rik

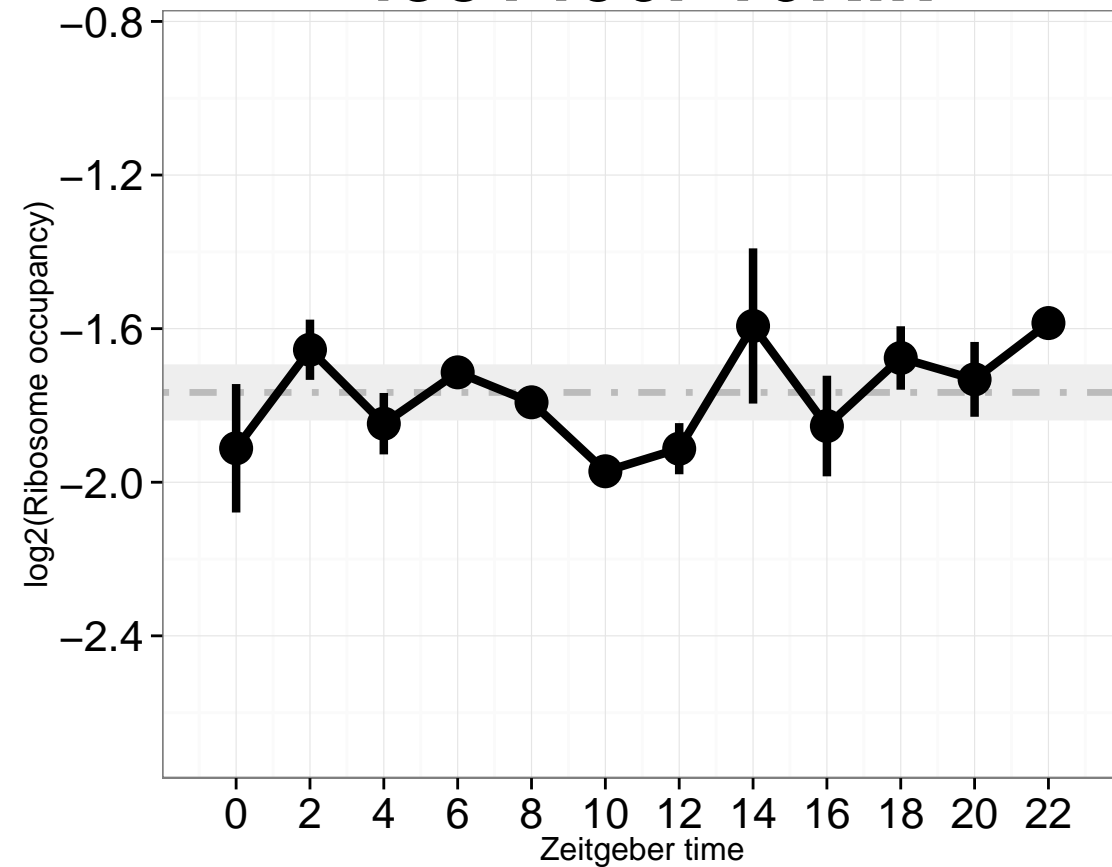

Supplement: Supplementary file 6 — Transcriptome-wide kidney RPF (blue) and RNA (orange) levels in the left panels (with “error bars” connecting the two replicates of each timepoint) and TE in the right panels. (ZIP 116896 kb) [file 13059_2017_1222_MOESM6_ESM.zip › Supp_Dataset_S1/A_RNA_non_rhythmic_RPF_non_rhythmic/4931406P16Rik_kidney_set_A.pdf]

**4931414P19Rik**

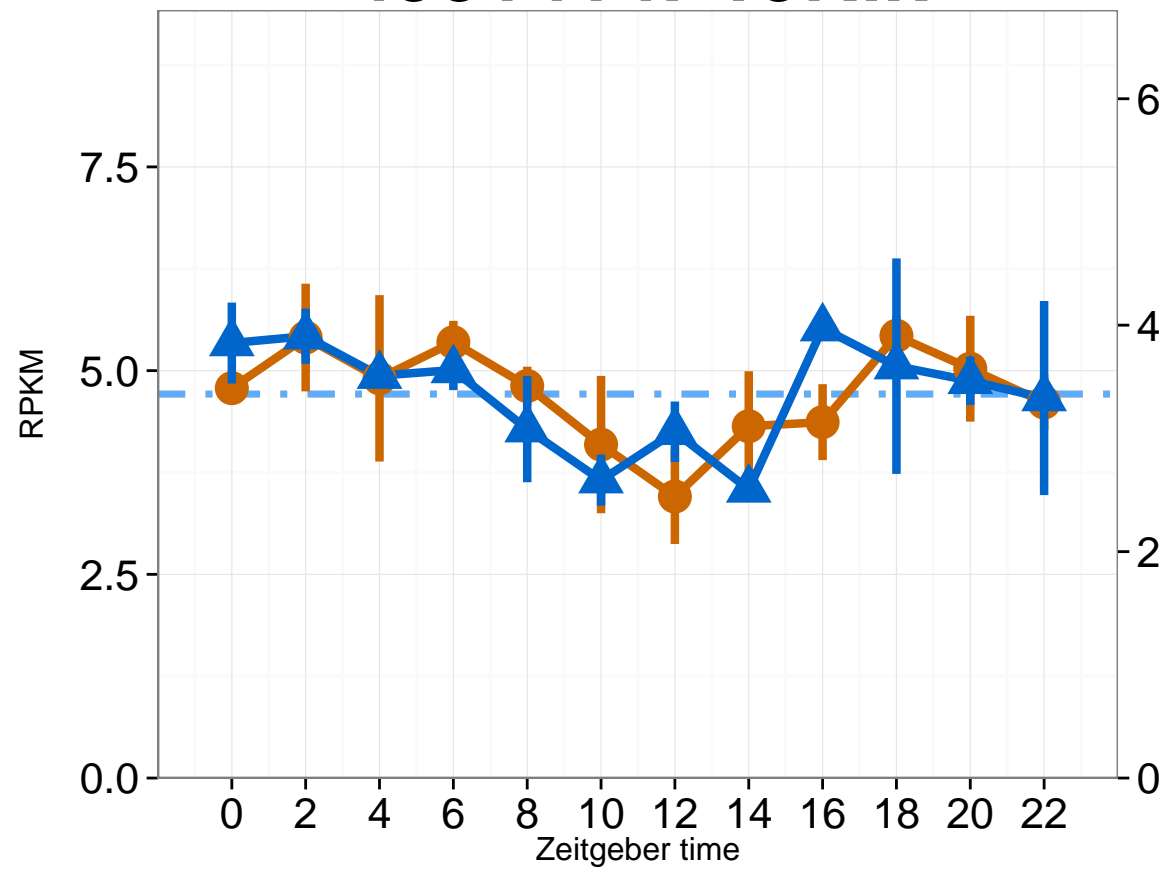

**4931414P19Rik**

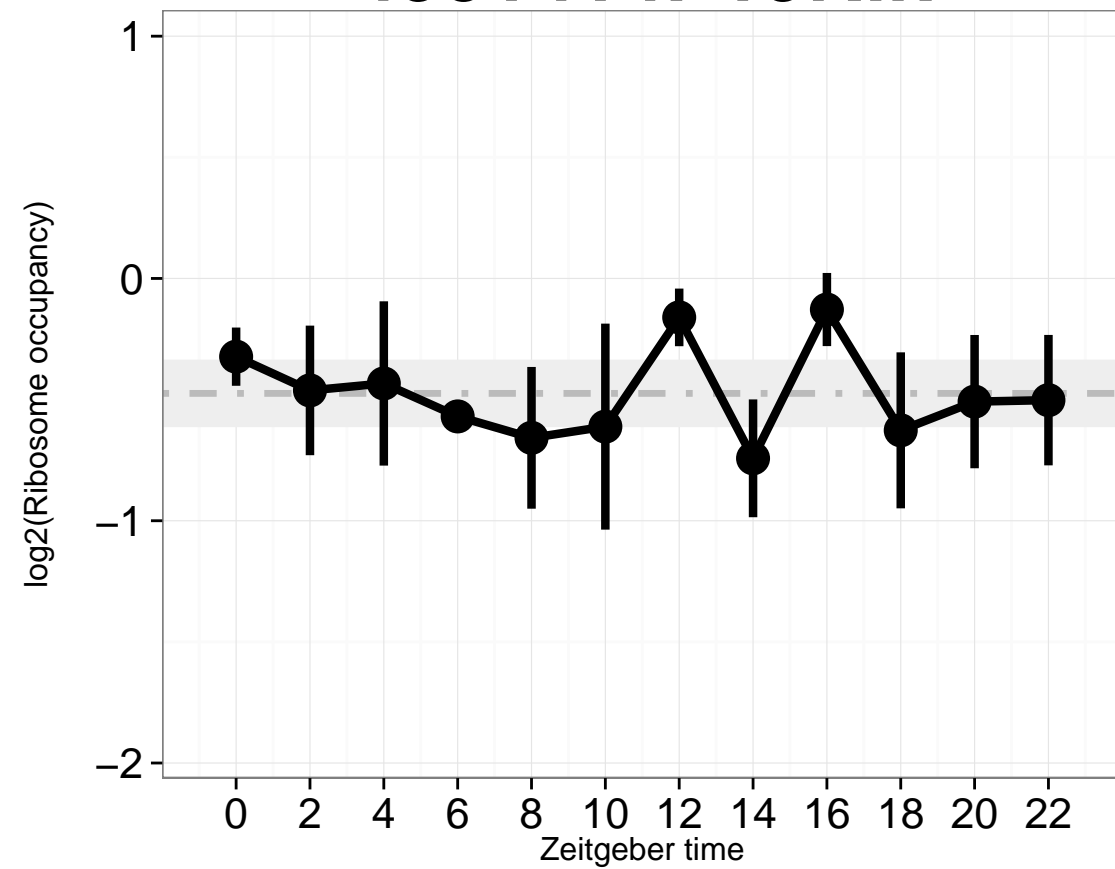

Supplement: Supplementary file 6 — Transcriptome-wide kidney RPF (blue) and RNA (orange) levels in the left panels (with “error bars” connecting the two replicates of each timepoint) and TE in the right panels. (ZIP 116896 kb) [file 13059_2017_1222_MOESM6_ESM.zip › Supp_Dataset_S1/A_RNA_non_rhythmic_RPF_non_rhythmic/4931414P19Rik_kidney_set_A.pdf]

## 4932438A13Rik

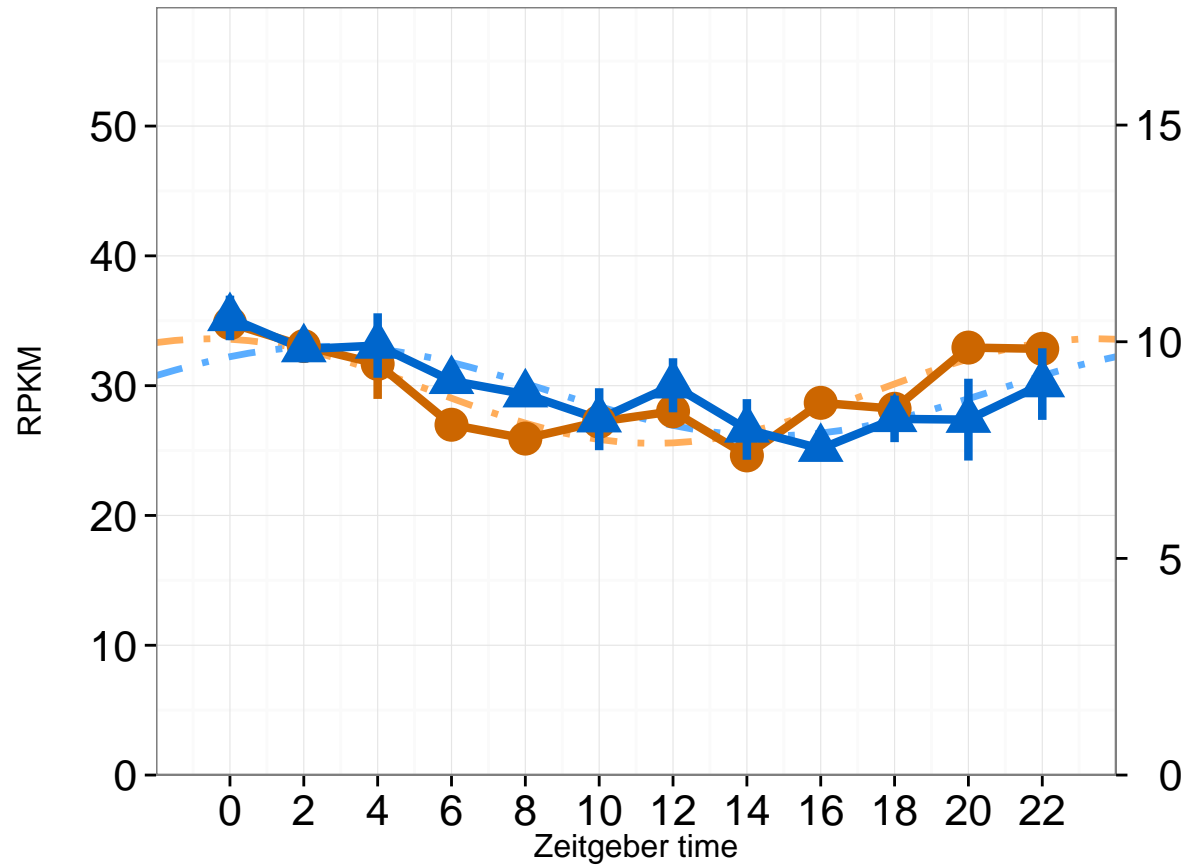

## 4932438A13Rik

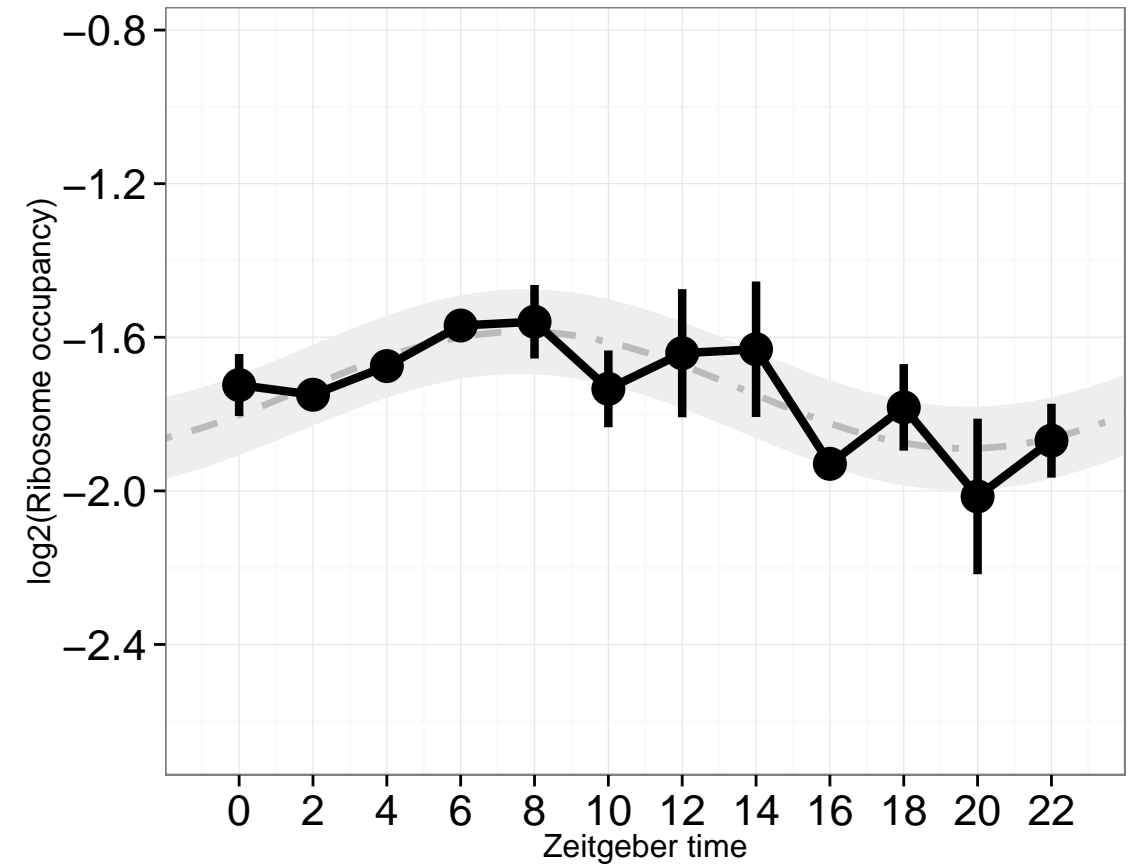

Supplement: Supplementary file 6 — Transcriptome-wide kidney RPF (blue) and RNA (orange) levels in the left panels (with “error bars” connecting the two replicates of each timepoint) and TE in the right panels. (ZIP 116896 kb) [file 13059_2017_1222_MOESM6_ESM.zip › Supp_Dataset_S1/A_RNA_non_rhythmic_RPF_non_rhythmic/4932438A13Rik_kidney_set_A.pdf]

## 4933411K16Rik

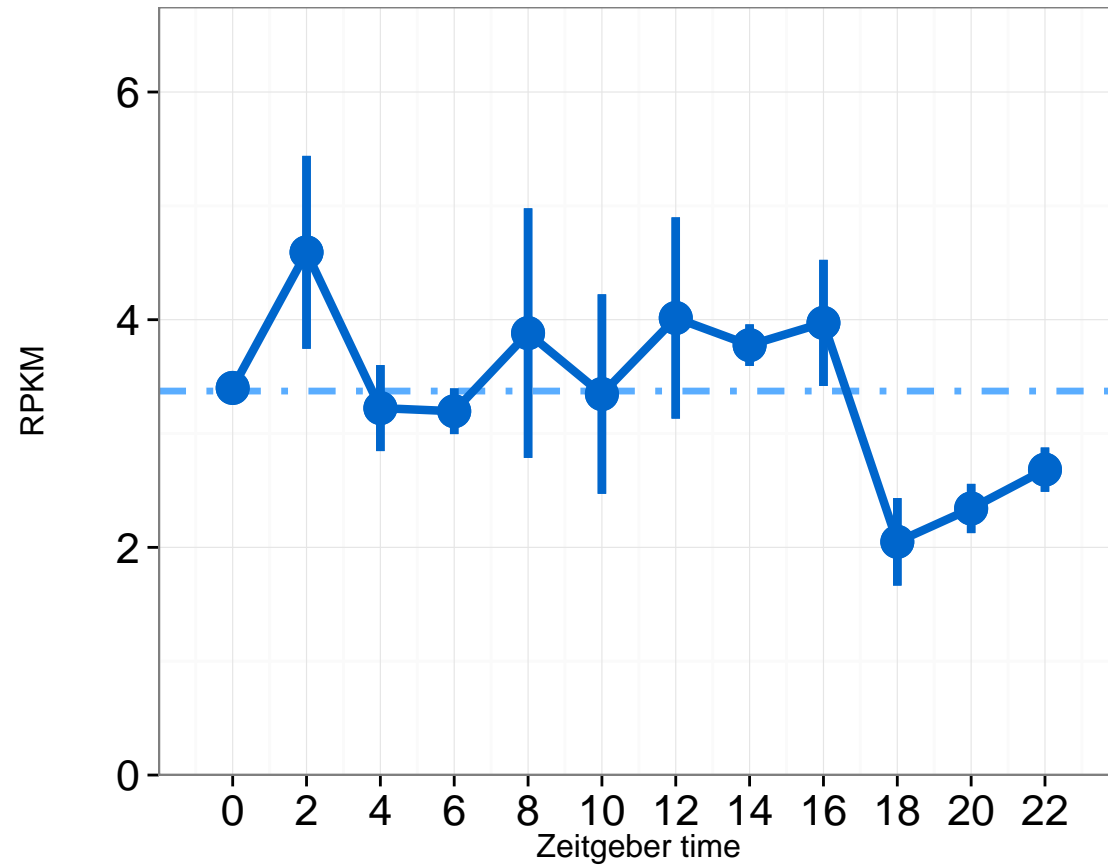

## 4933411K16Rik log2(Ribosome occup

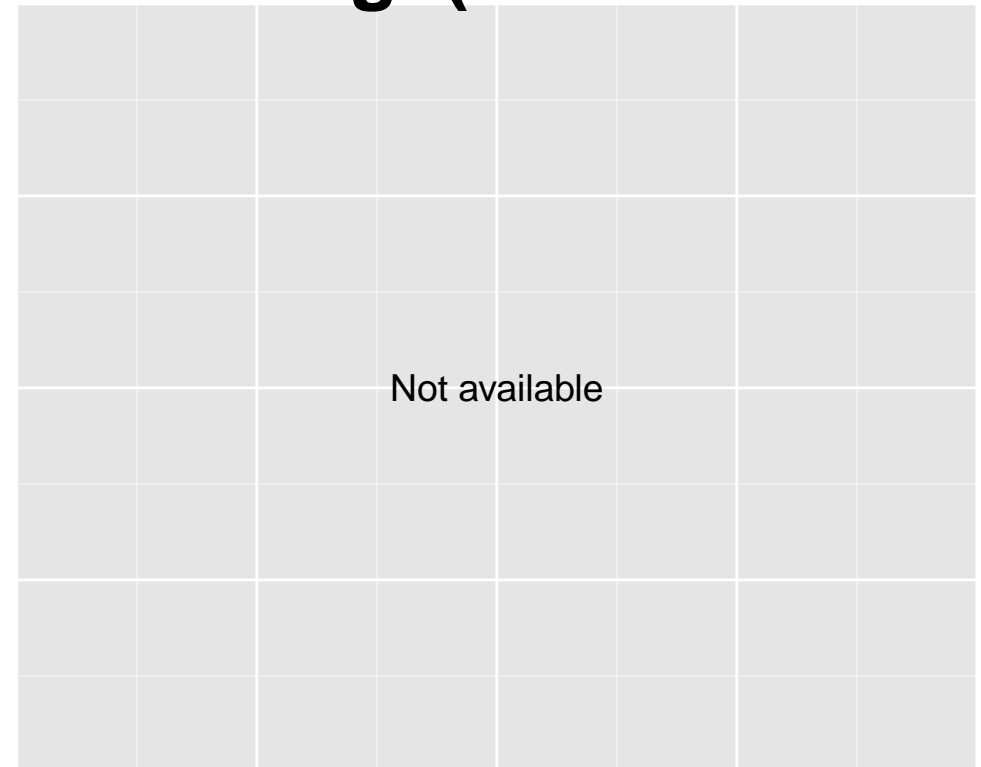

Supplement: Supplementary file 6 — Transcriptome-wide kidney RPF (blue) and RNA (orange) levels in the left panels (with “error bars” connecting the two replicates of each timepoint) and TE in the right panels. (ZIP 116896 kb) [file 13059_2017_1222_MOESM6_ESM.zip › Supp_Dataset_S1/A_RNA_non_rhythmic_RPF_non_rhythmic/4933411K16Rik_kidney_set_A.pdf]

# 4933411K20Rik

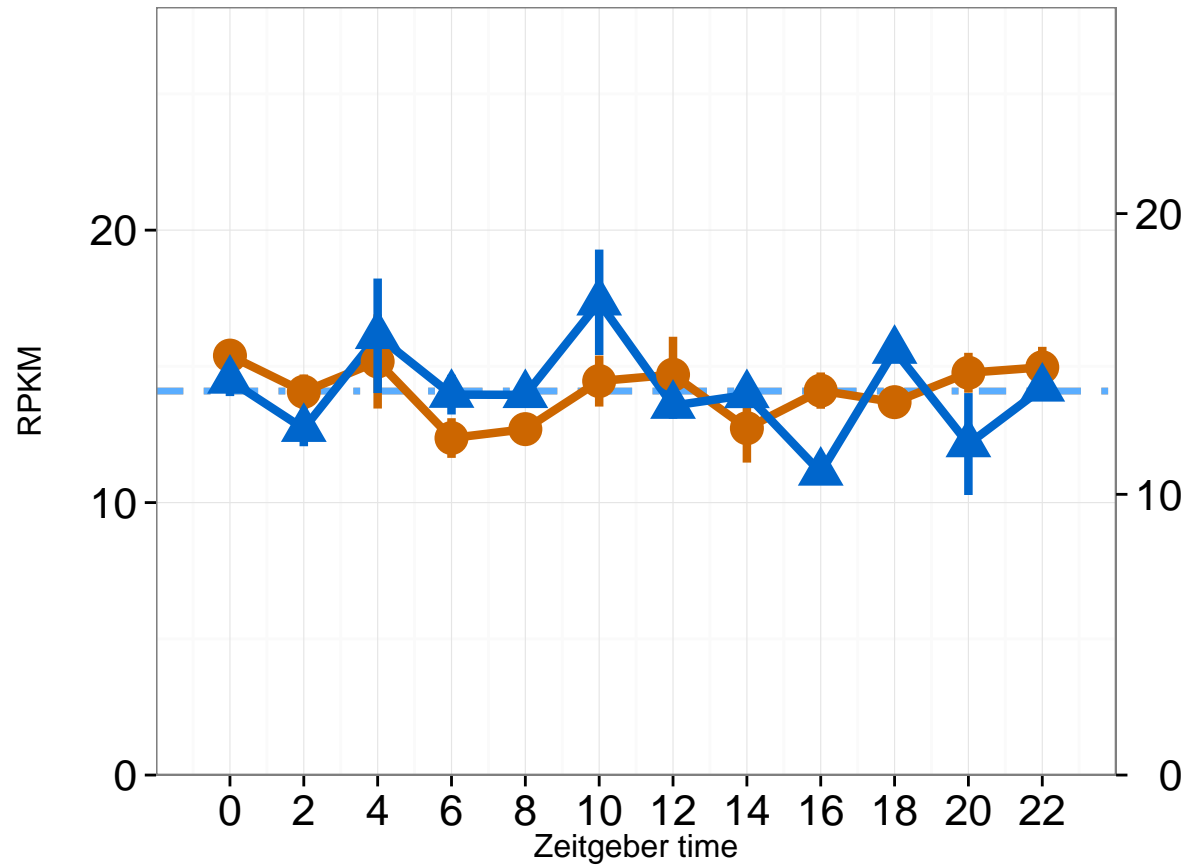

# 4933411K20Rik

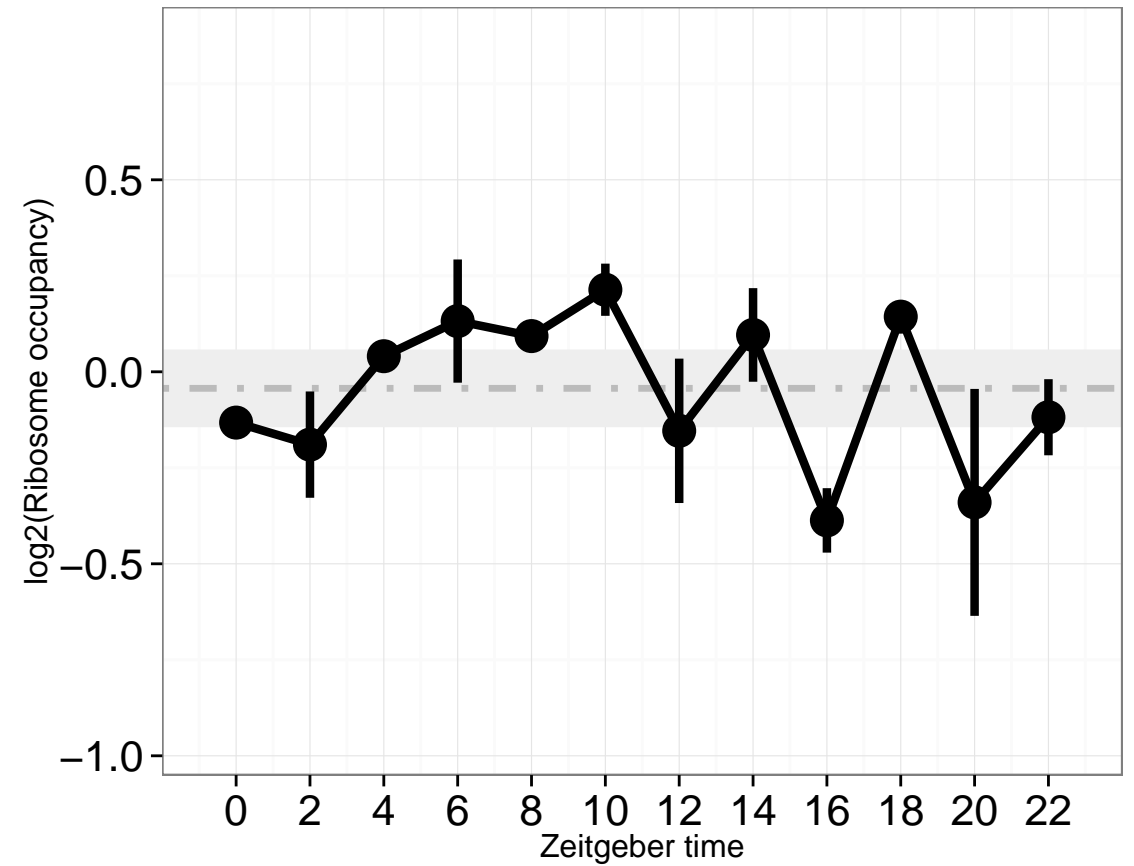

Supplement: Supplementary file 6 — Transcriptome-wide kidney RPF (blue) and RNA (orange) levels in the left panels (with “error bars” connecting the two replicates of each timepoint) and TE in the right panels. (ZIP 116896 kb) [file 13059_2017_1222_MOESM6_ESM.zip › Supp_Dataset_S1/A_RNA_non_rhythmic_RPF_non_rhythmic/4933411K20Rik_kidney_set_A.pdf]

## 4933426M11Rik

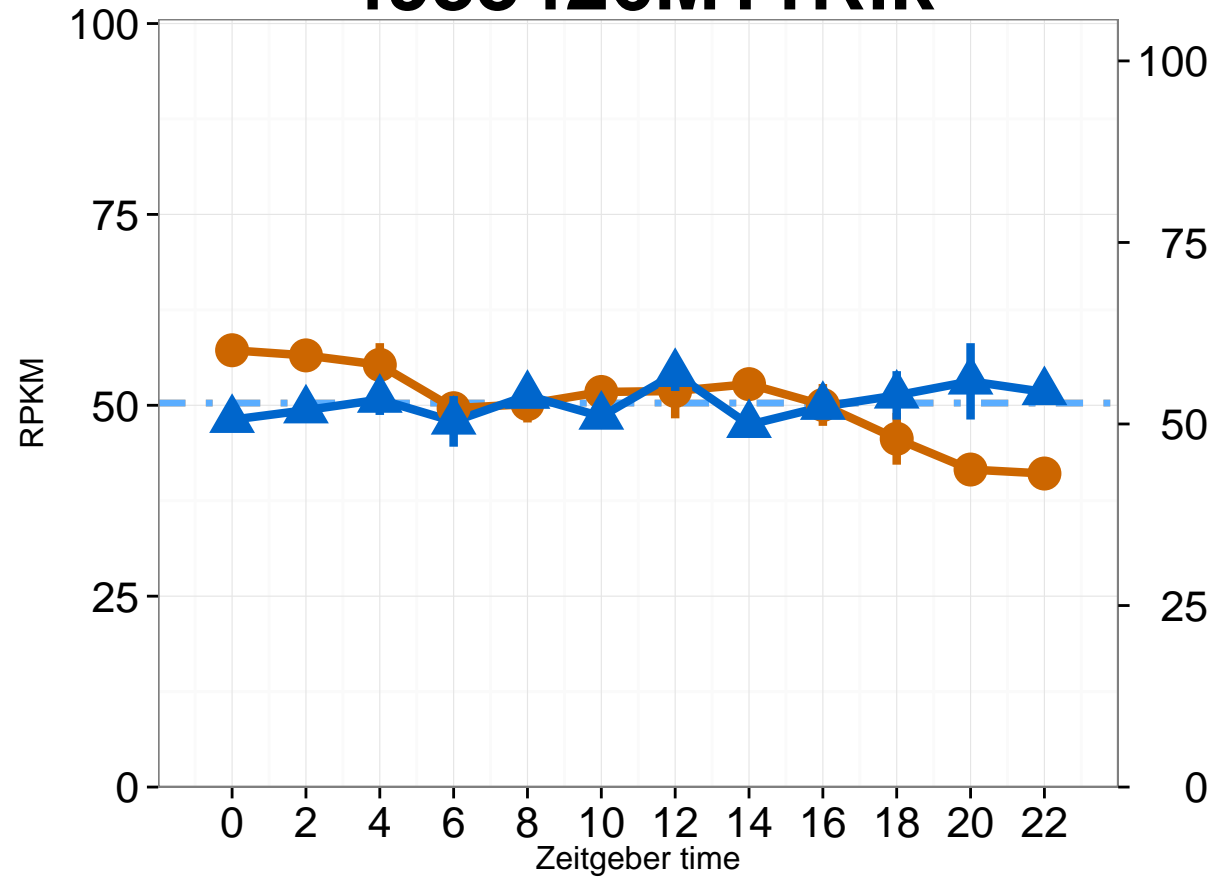

## 4933426M11Rik

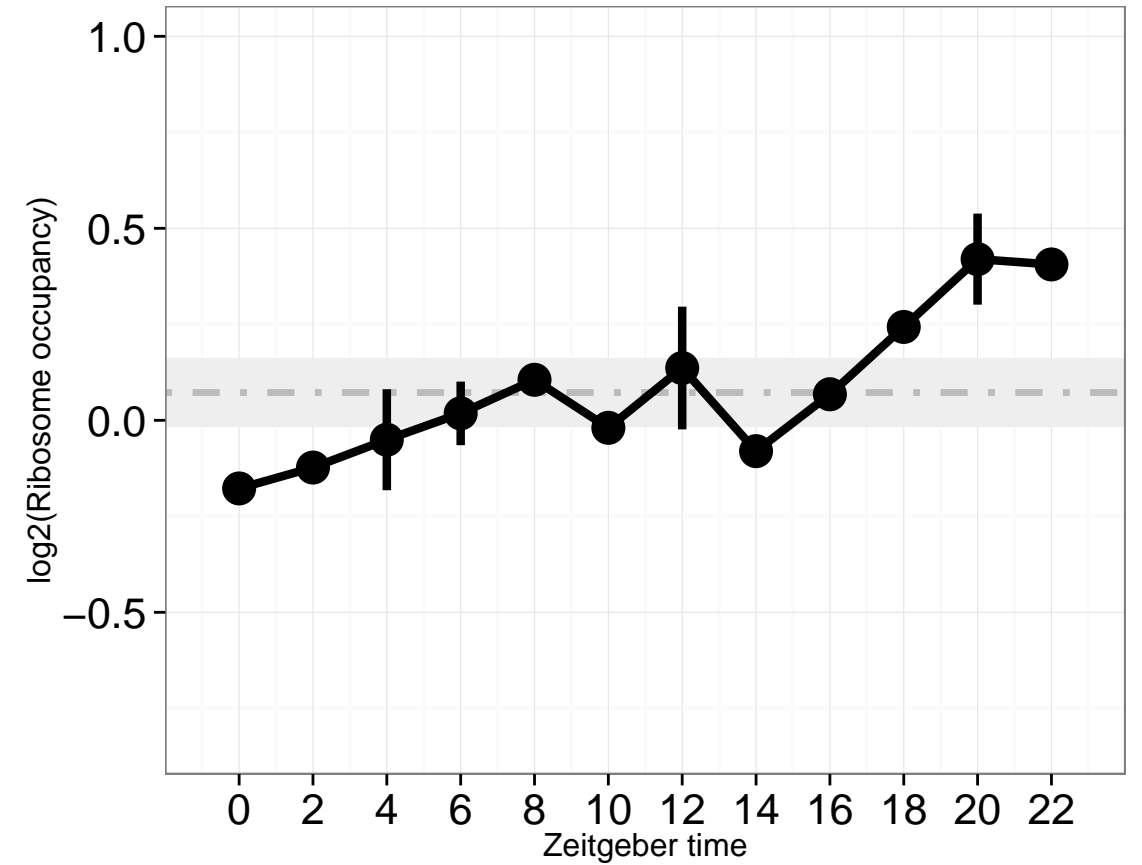

Supplement: Supplementary file 6 — Transcriptome-wide kidney RPF (blue) and RNA (orange) levels in the left panels (with “error bars” connecting the two replicates of each timepoint) and TE in the right panels. (ZIP 116896 kb) [file 13059_2017_1222_MOESM6_ESM.zip › Supp_Dataset_S1/A_RNA_non_rhythmic_RPF_non_rhythmic/4933426M11Rik_kidney_set_A.pdf]

# 4933427D14Rik

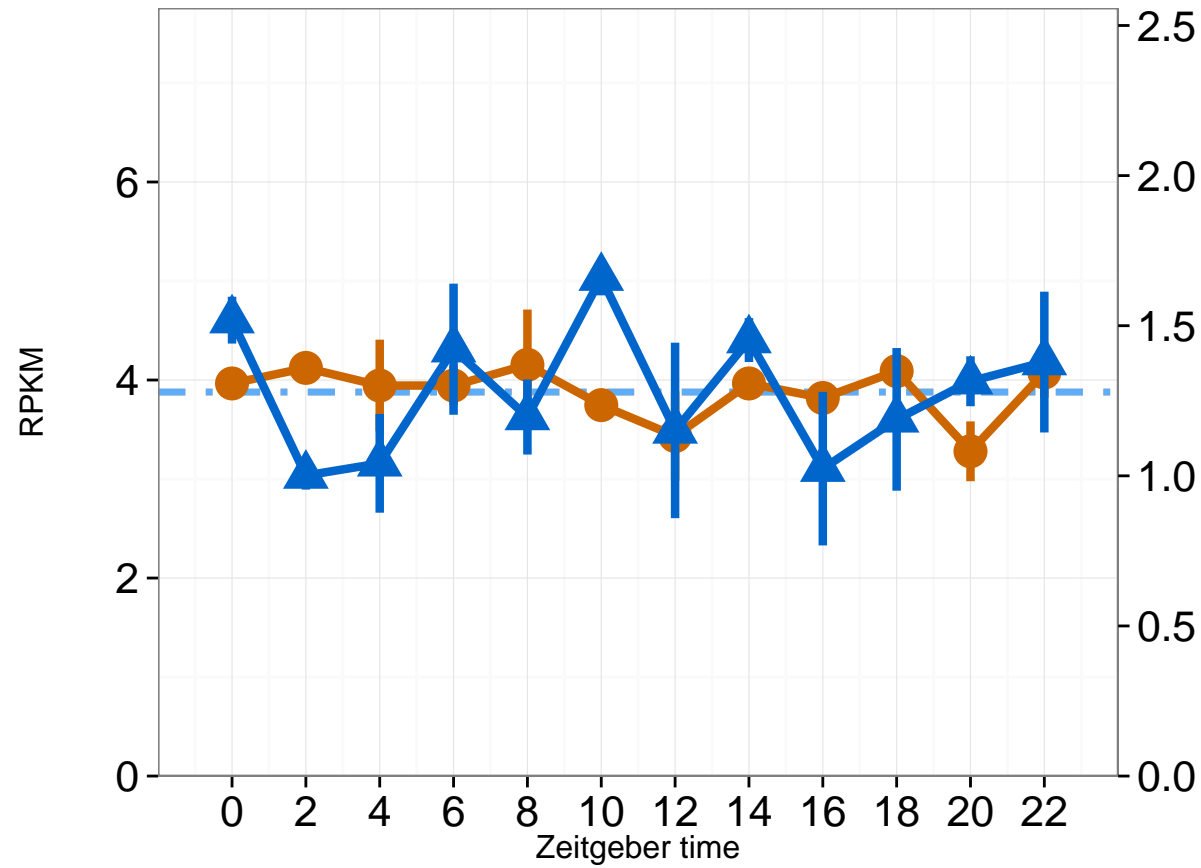

# 4933427D14Rik

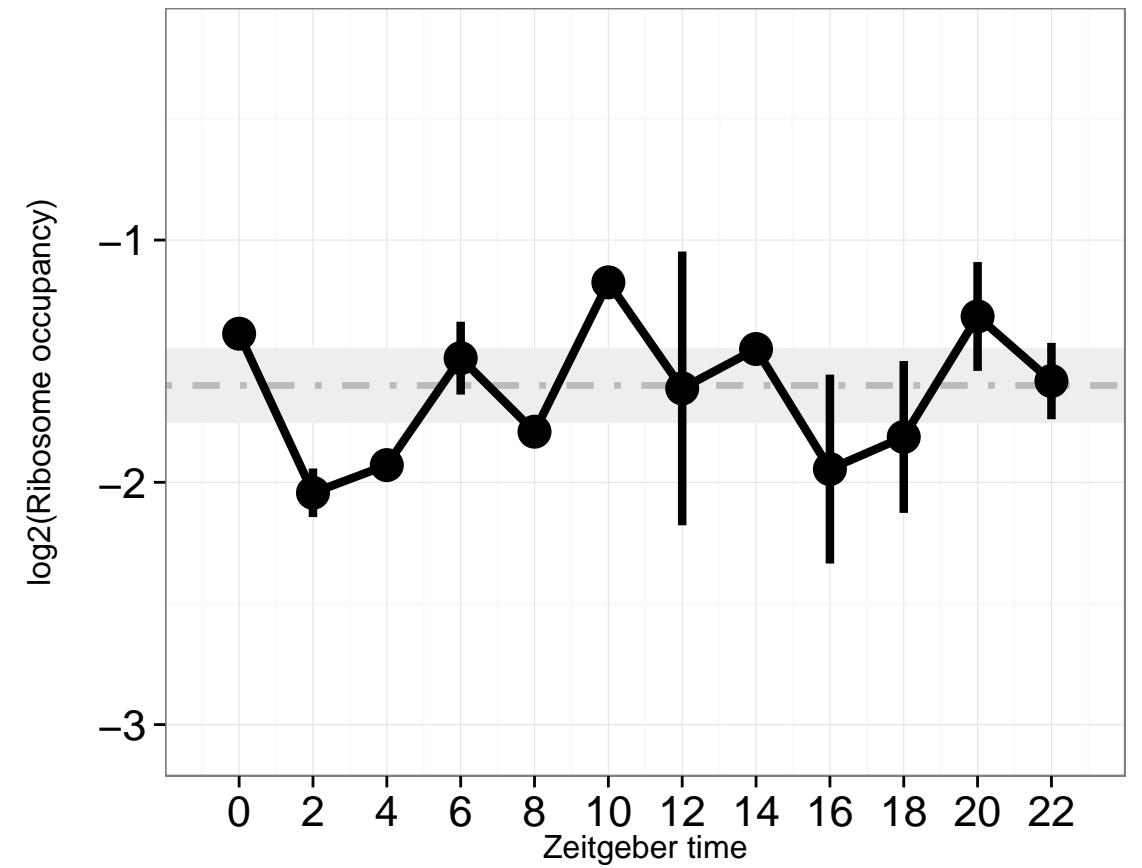

Supplement: Supplementary file 6 — Transcriptome-wide kidney RPF (blue) and RNA (orange) levels in the left panels (with “error bars” connecting the two replicates of each timepoint) and TE in the right panels. (ZIP 116896 kb) [file 13059_2017_1222_MOESM6_ESM.zip › Supp_Dataset_S1/A_RNA_non_rhythmic_RPF_non_rhythmic/4933427D14Rik_kidney_set_A.pdf]

4933434E20Rik

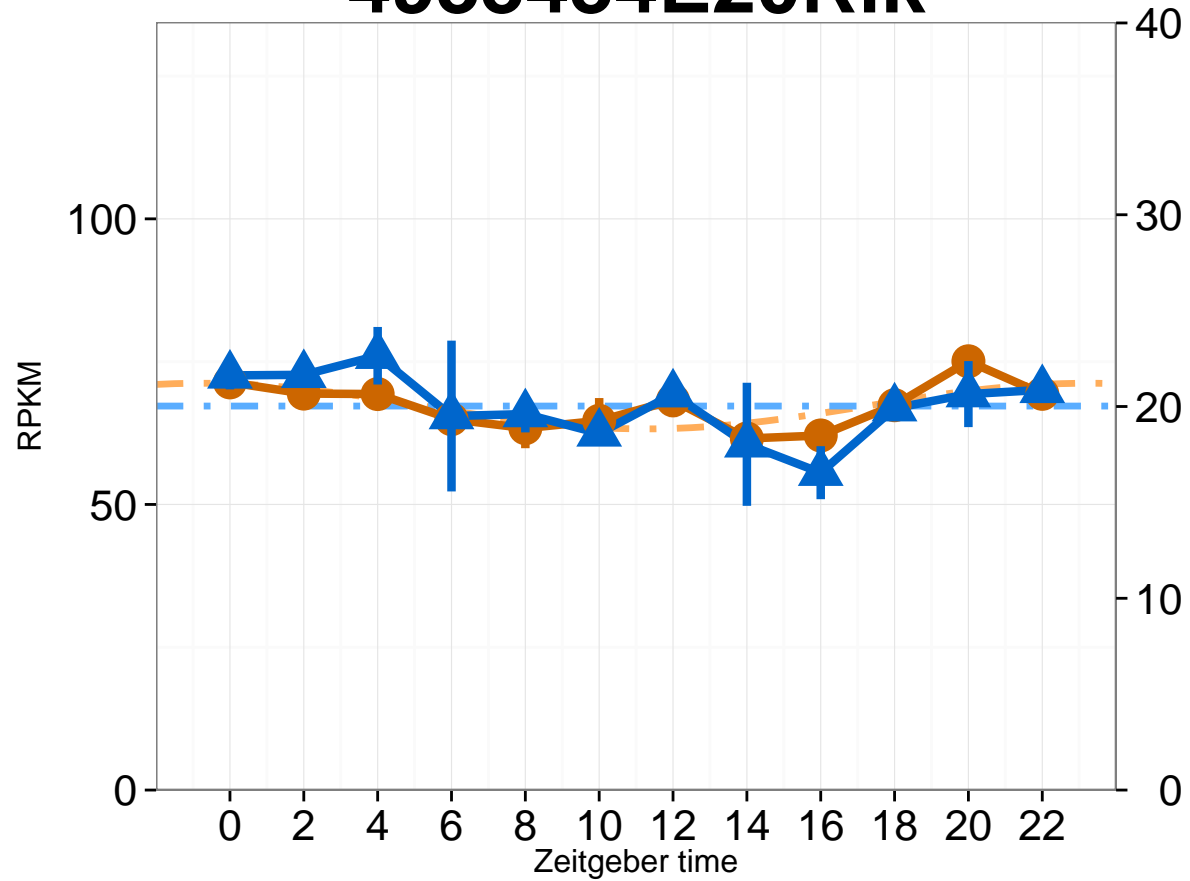

4933434E20Rik

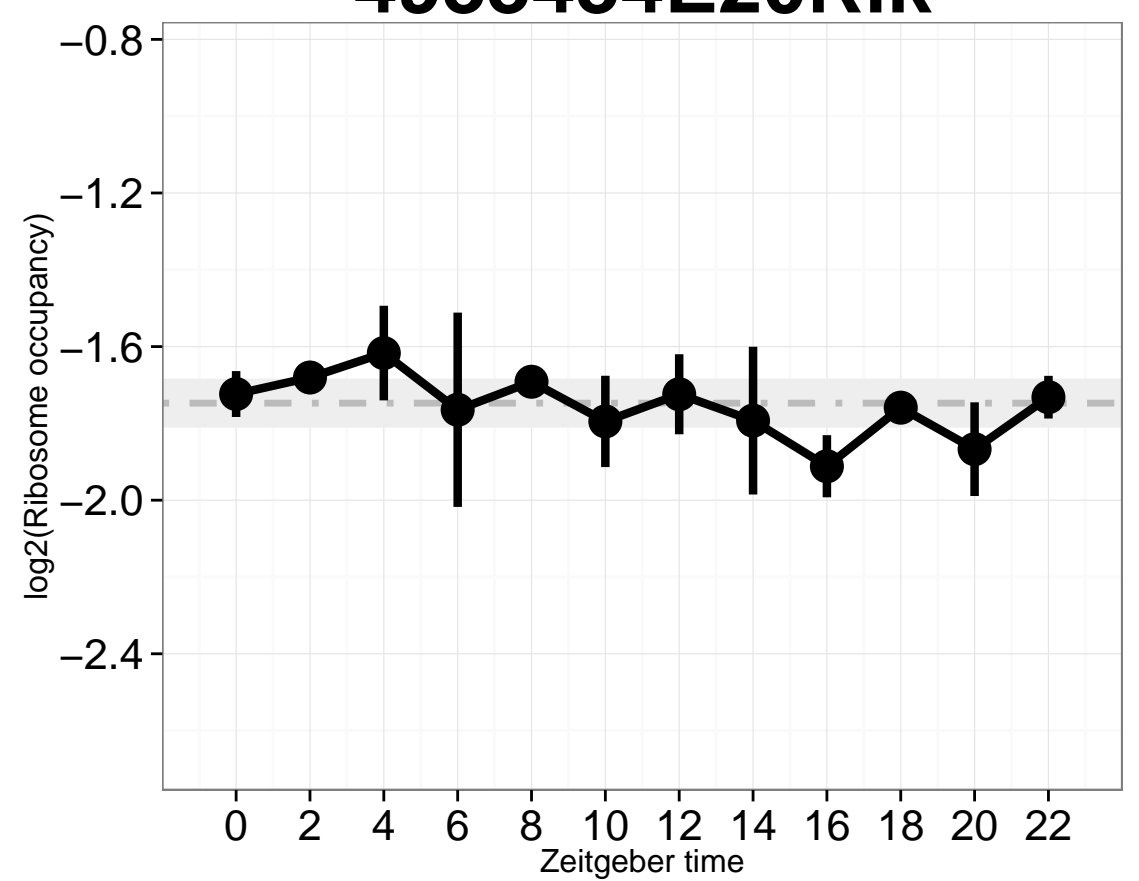

Supplement: Supplementary file 6 — Transcriptome-wide kidney RPF (blue) and RNA (orange) levels in the left panels (with “error bars” connecting the two replicates of each timepoint) and TE in the right panels. (ZIP 116896 kb) [file 13059_2017_1222_MOESM6_ESM.zip › Supp_Dataset_S1/A_RNA_non_rhythmic_RPF_non_rhythmic/4933434E20Rik_kidney_set_A.pdf]

## 5031414D18Rik

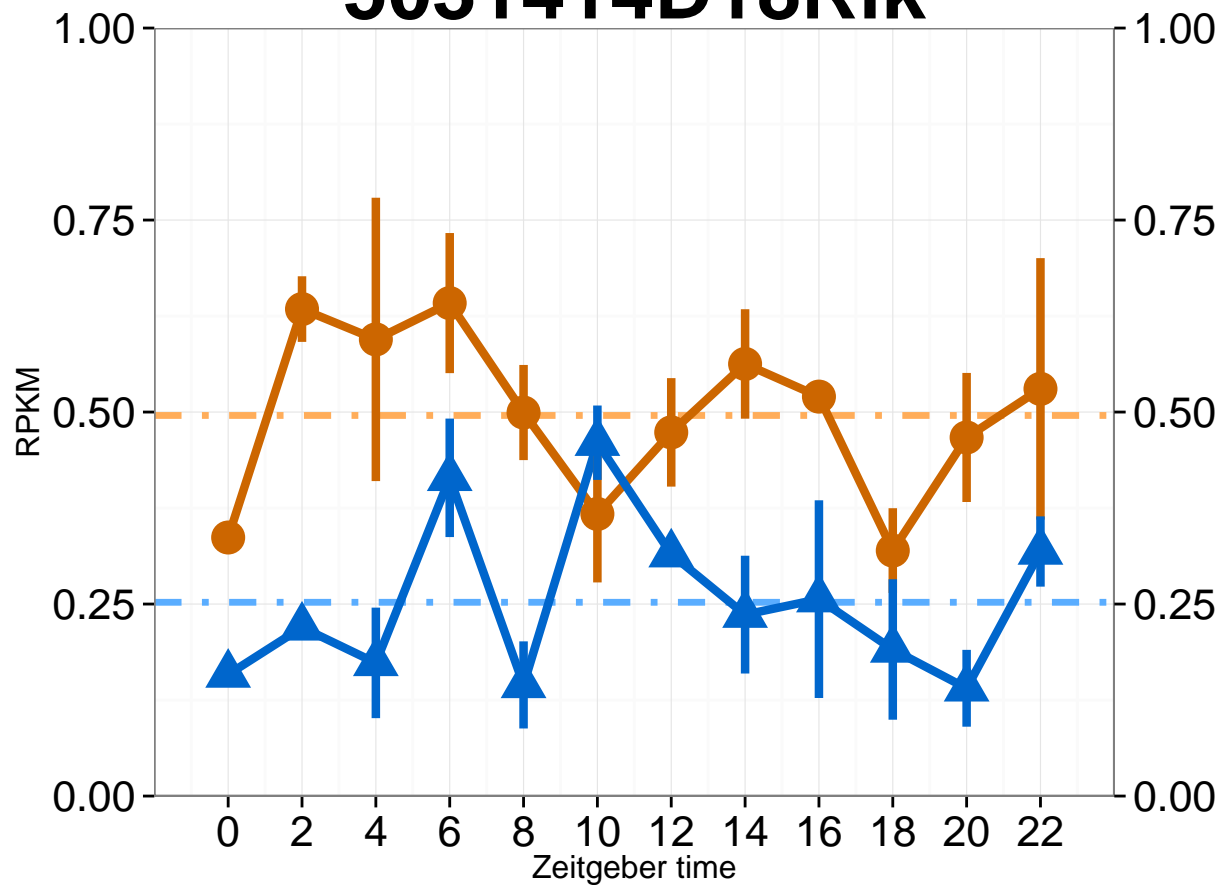

## 5031414D18Rik

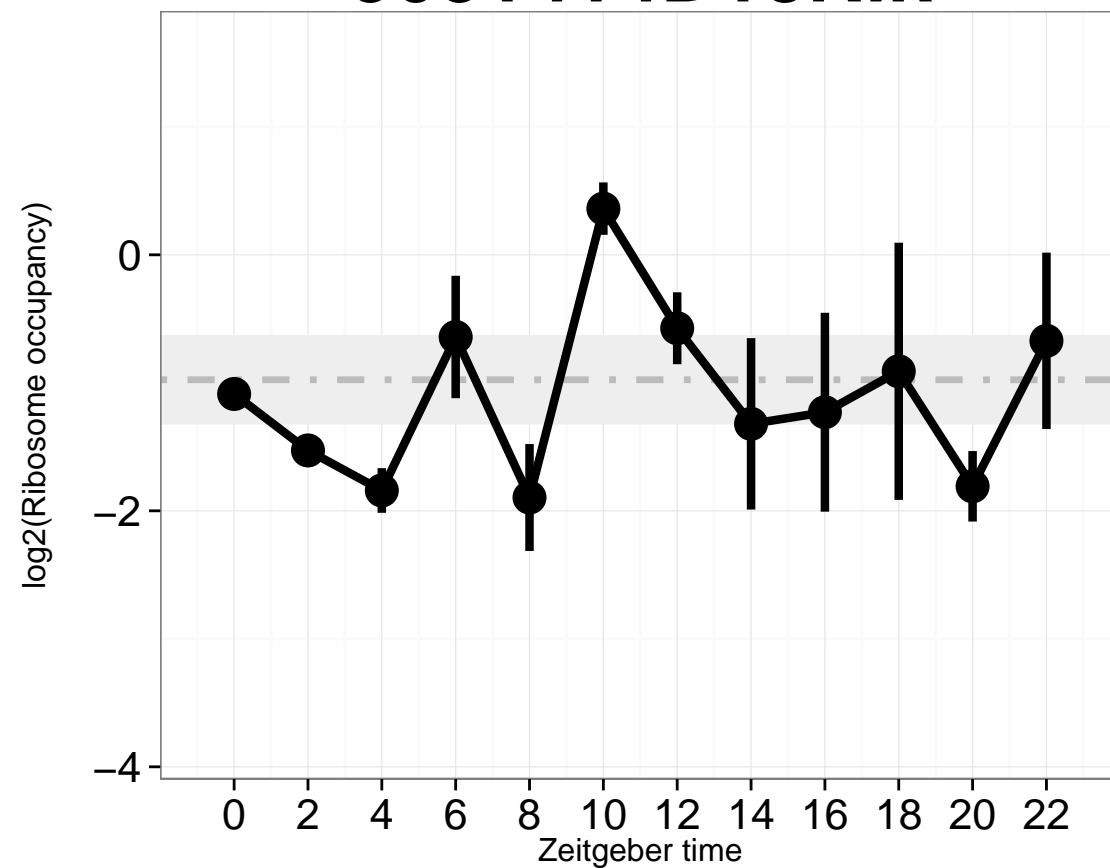

Supplement: Supplementary file 6 — Transcriptome-wide kidney RPF (blue) and RNA (orange) levels in the left panels (with “error bars” connecting the two replicates of each timepoint) and TE in the right panels. (ZIP 116896 kb) [file 13059_2017_1222_MOESM6_ESM.zip › Supp_Dataset_S1/A_RNA_non_rhythmic_RPF_non_rhythmic/5031414D18Rik_kidney_set_A.pdf]

# 5031425E22Rik

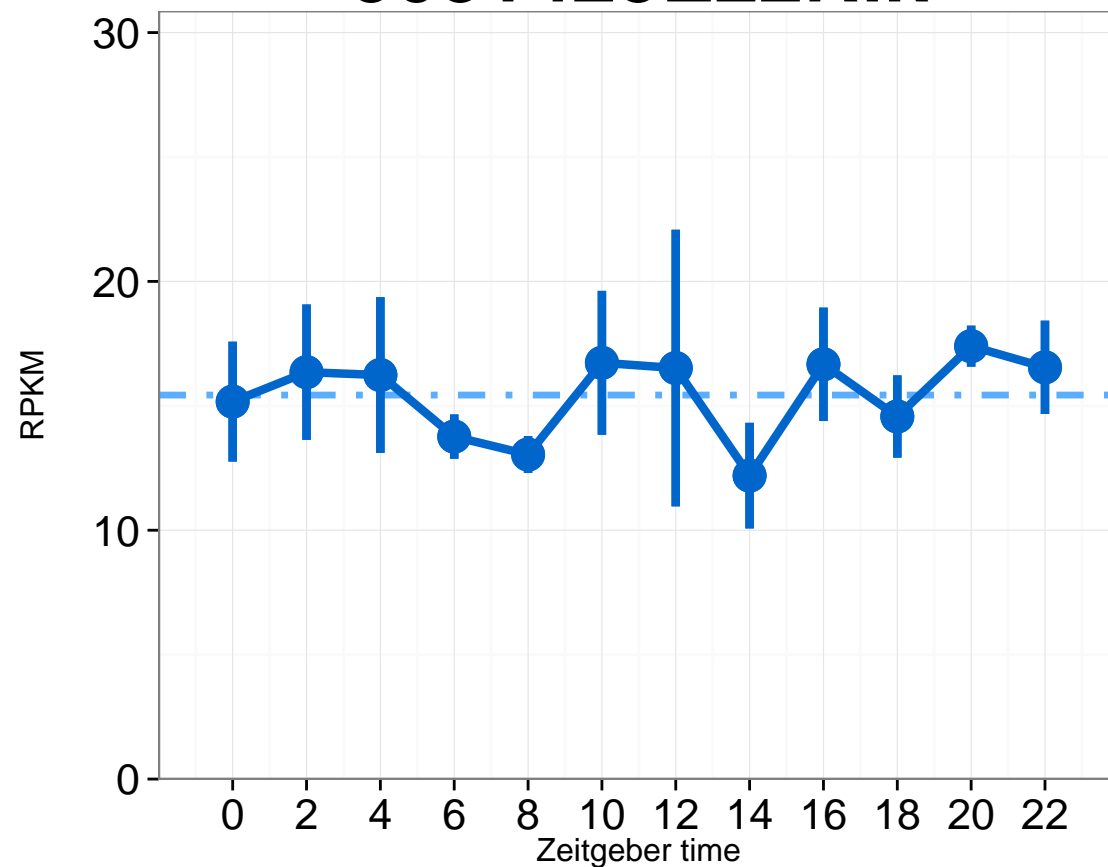

# 5031425E22Rik log2(Ribosome occup

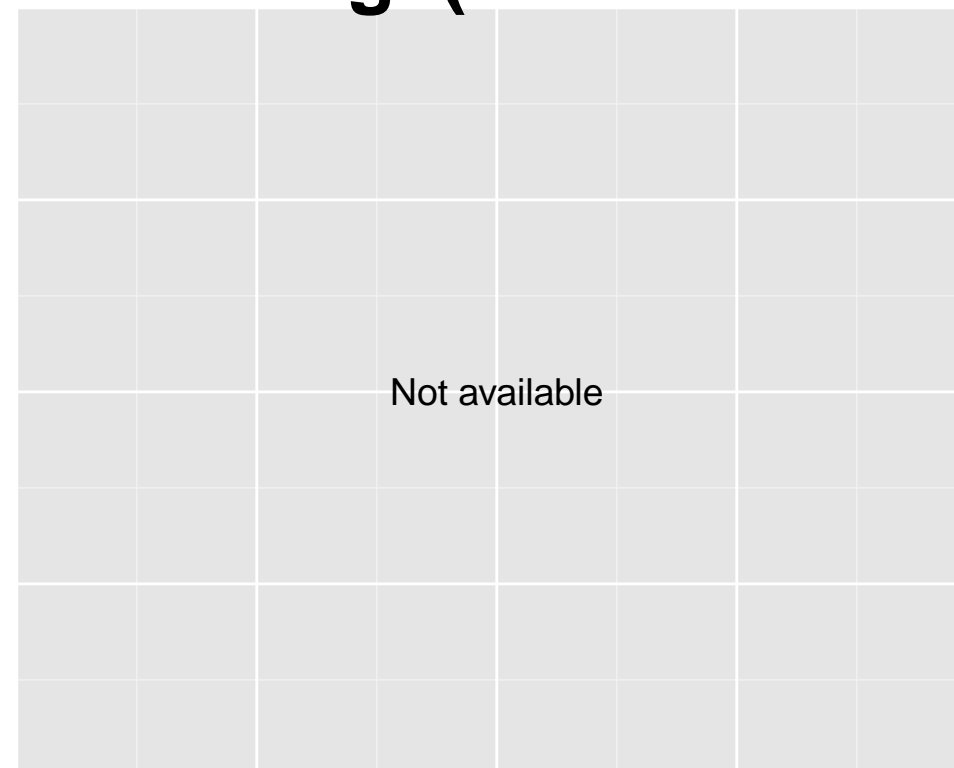

Supplement: Supplementary file 6 — Transcriptome-wide kidney RPF (blue) and RNA (orange) levels in the left panels (with “error bars” connecting the two replicates of each timepoint) and TE in the right panels. (ZIP 116896 kb) [file 13059_2017_1222_MOESM6_ESM.zip › Supp_Dataset_S1/A_RNA_non_rhythmic_RPF_non_rhythmic/5031425E22Rik_kidney_set_A.pdf]

# 5031439G07Rik

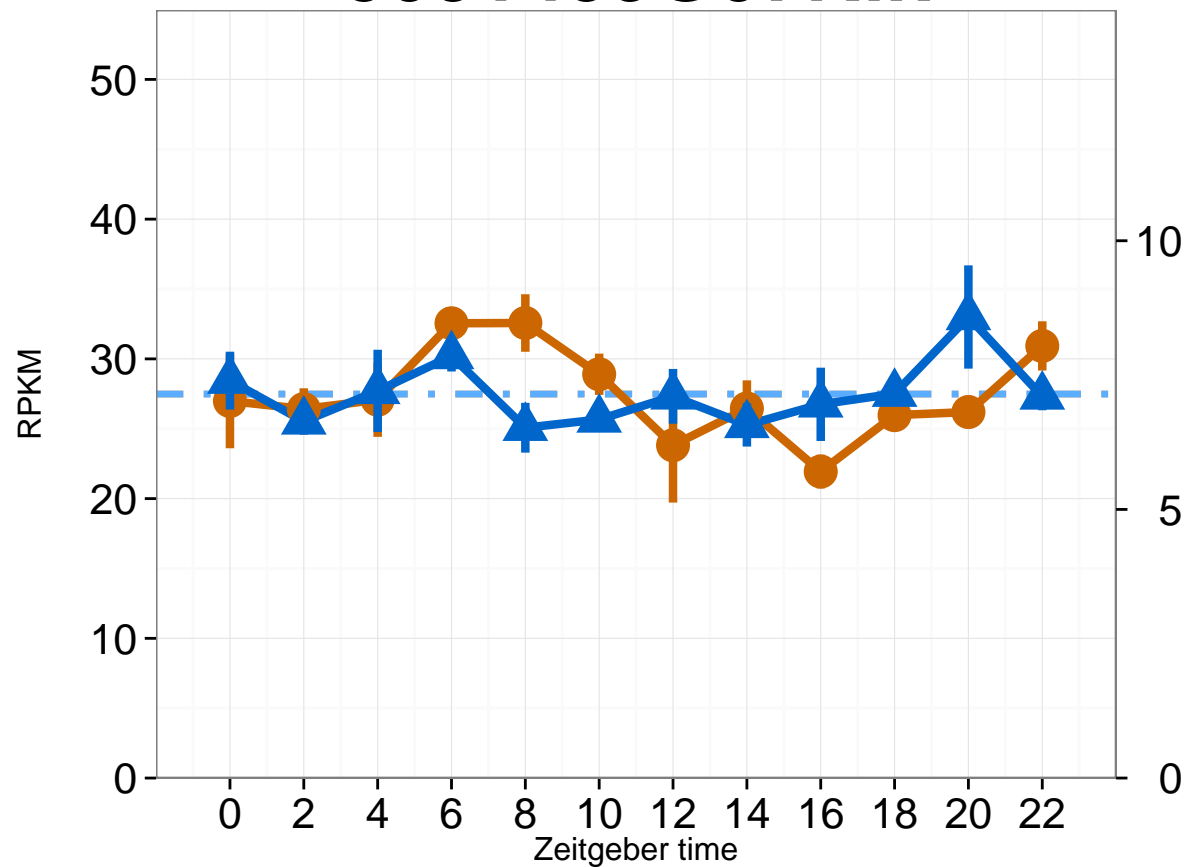

# 5031439G07Rik

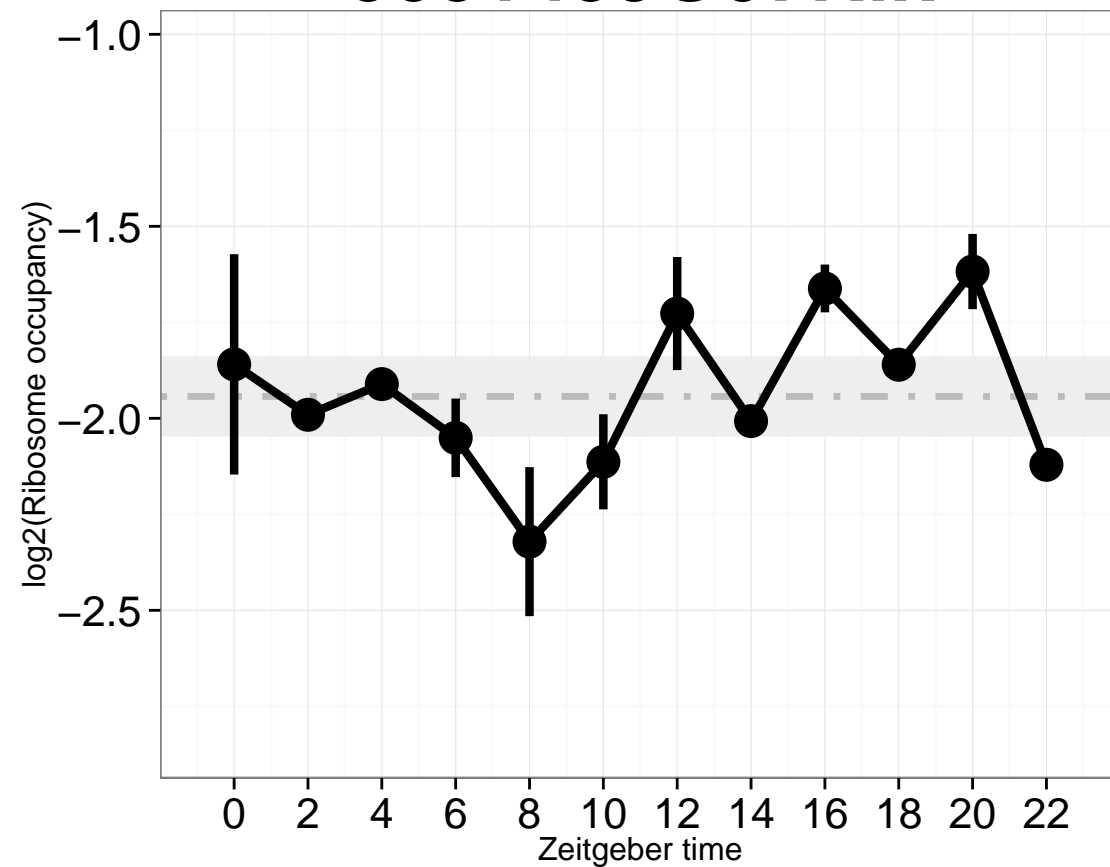

Supplement: Supplementary file 6 — Transcriptome-wide kidney RPF (blue) and RNA (orange) levels in the left panels (with “error bars” connecting the two replicates of each timepoint) and TE in the right panels. (ZIP 116896 kb) [file 13059_2017_1222_MOESM6_ESM.zip › Supp_Dataset_S1/A_RNA_non_rhythmic_RPF_non_rhythmic/5031439G07Rik_kidney_set_A.pdf]

## 5033411D12Rik

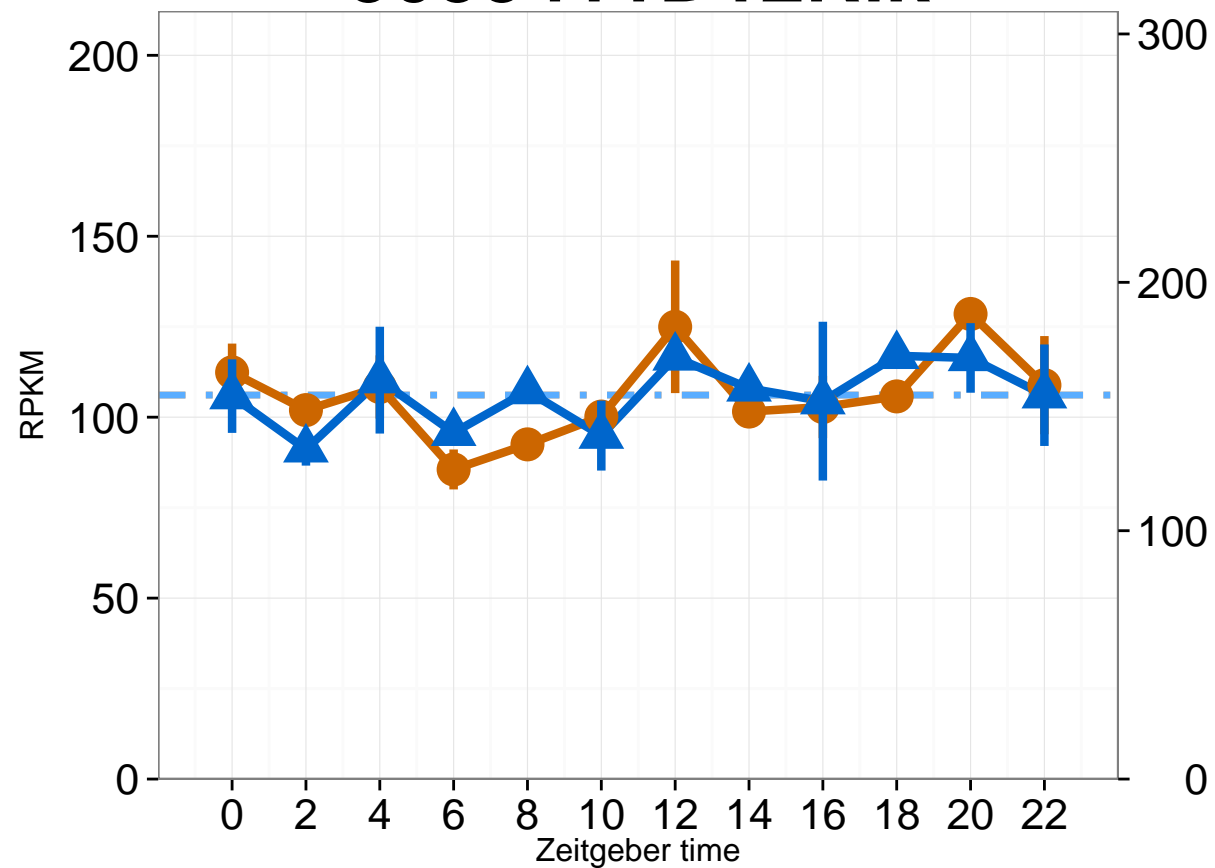

## 5033411D12Rik

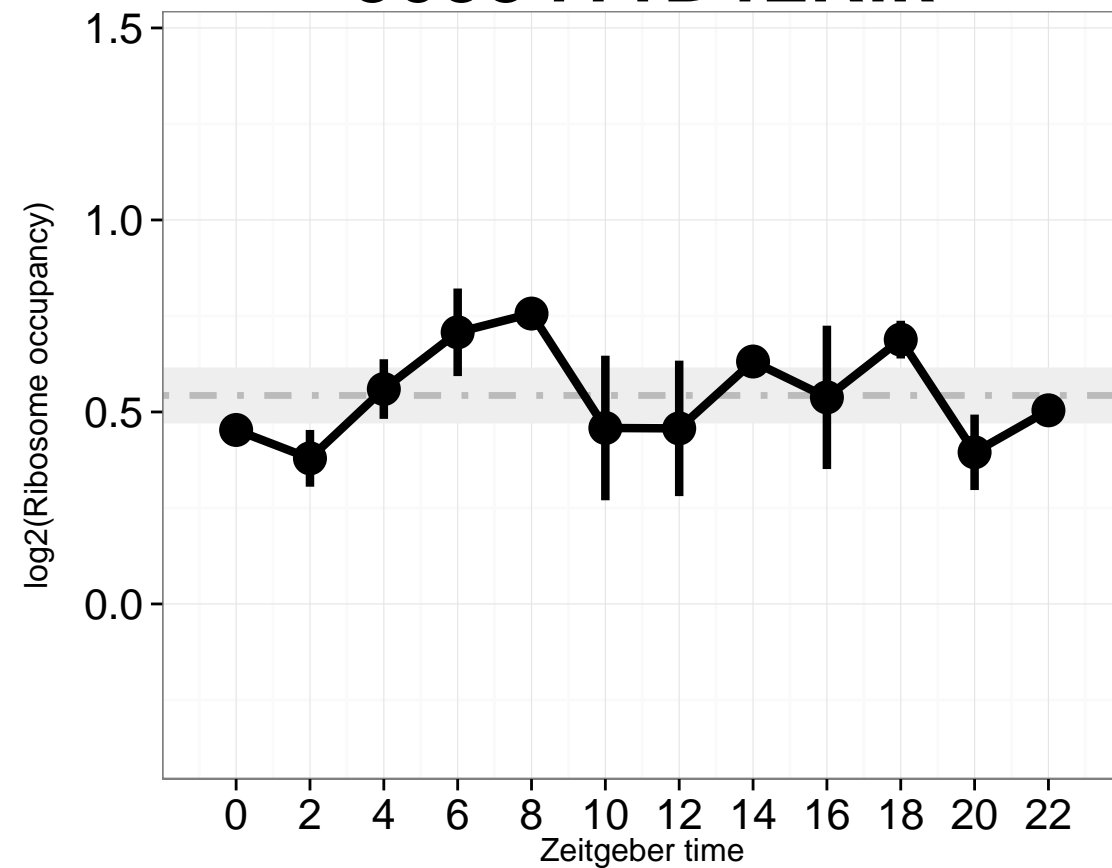

Supplement: Supplementary file 6 — Transcriptome-wide kidney RPF (blue) and RNA (orange) levels in the left panels (with “error bars” connecting the two replicates of each timepoint) and TE in the right panels. (ZIP 116896 kb) [file 13059_2017_1222_MOESM6_ESM.zip › Supp_Dataset_S1/A_RNA_non_rhythmic_RPF_non_rhythmic/5033411D12Rik_kidney_set_A.pdf]

## 5033430I15Rik

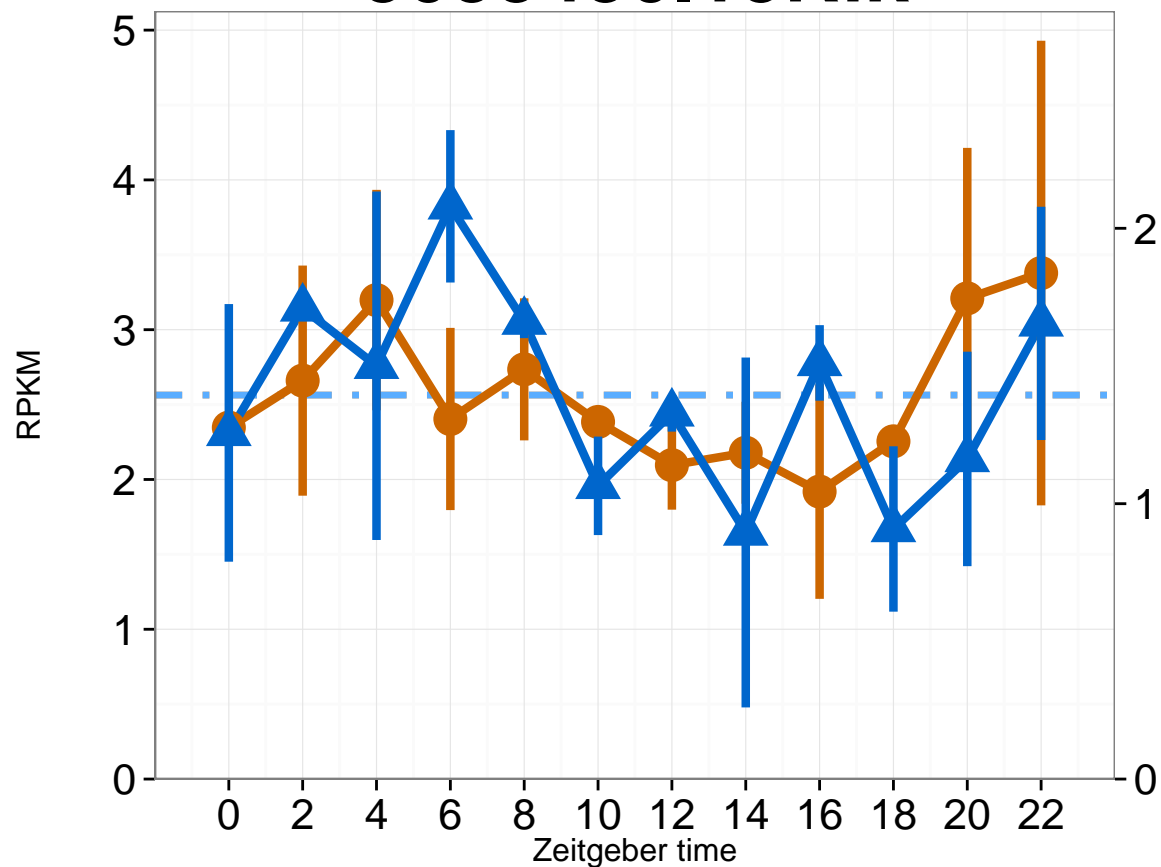

## 5033430I15Rik

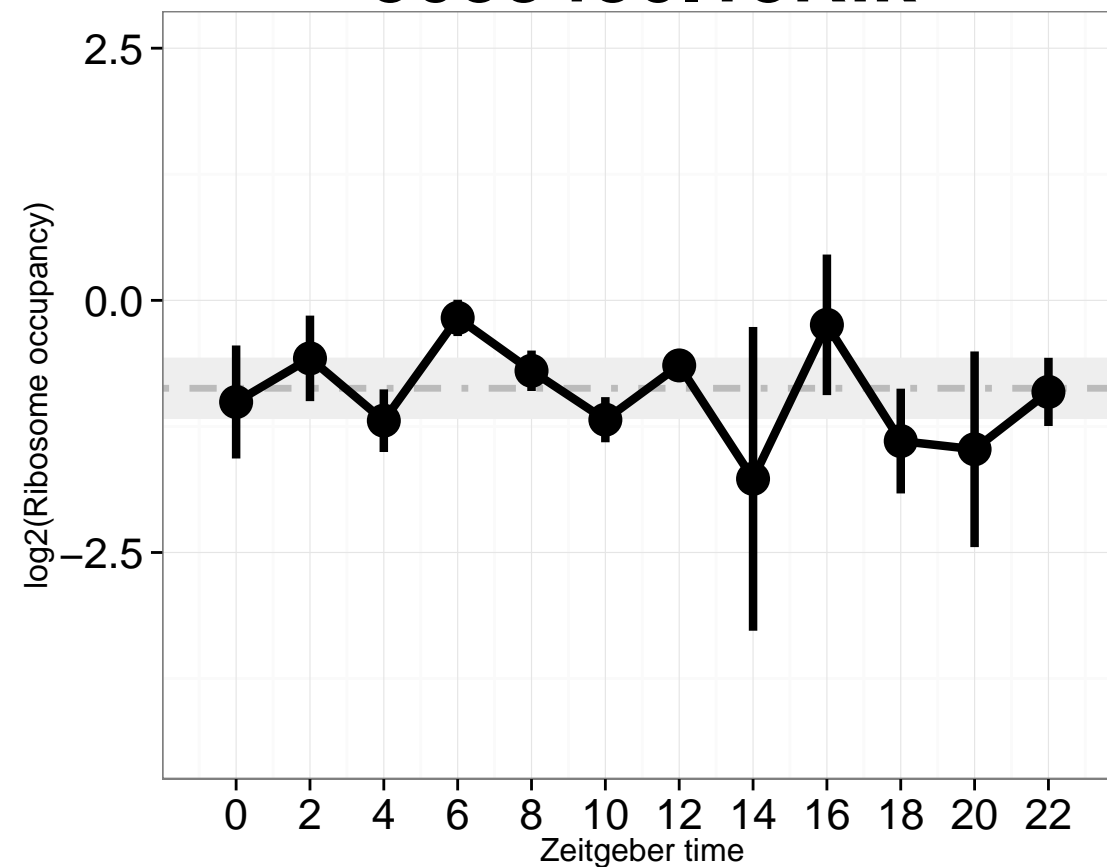

Supplement: Supplementary file 6 — Transcriptome-wide kidney RPF (blue) and RNA (orange) levels in the left panels (with “error bars” connecting the two replicates of each timepoint) and TE in the right panels. (ZIP 116896 kb) [file 13059_2017_1222_MOESM6_ESM.zip › Supp_Dataset_S1/A_RNA_non_rhythmic_RPF_non_rhythmic/5033430I15Rik_kidney_set_A.pdf]

## 5330426P16Rik

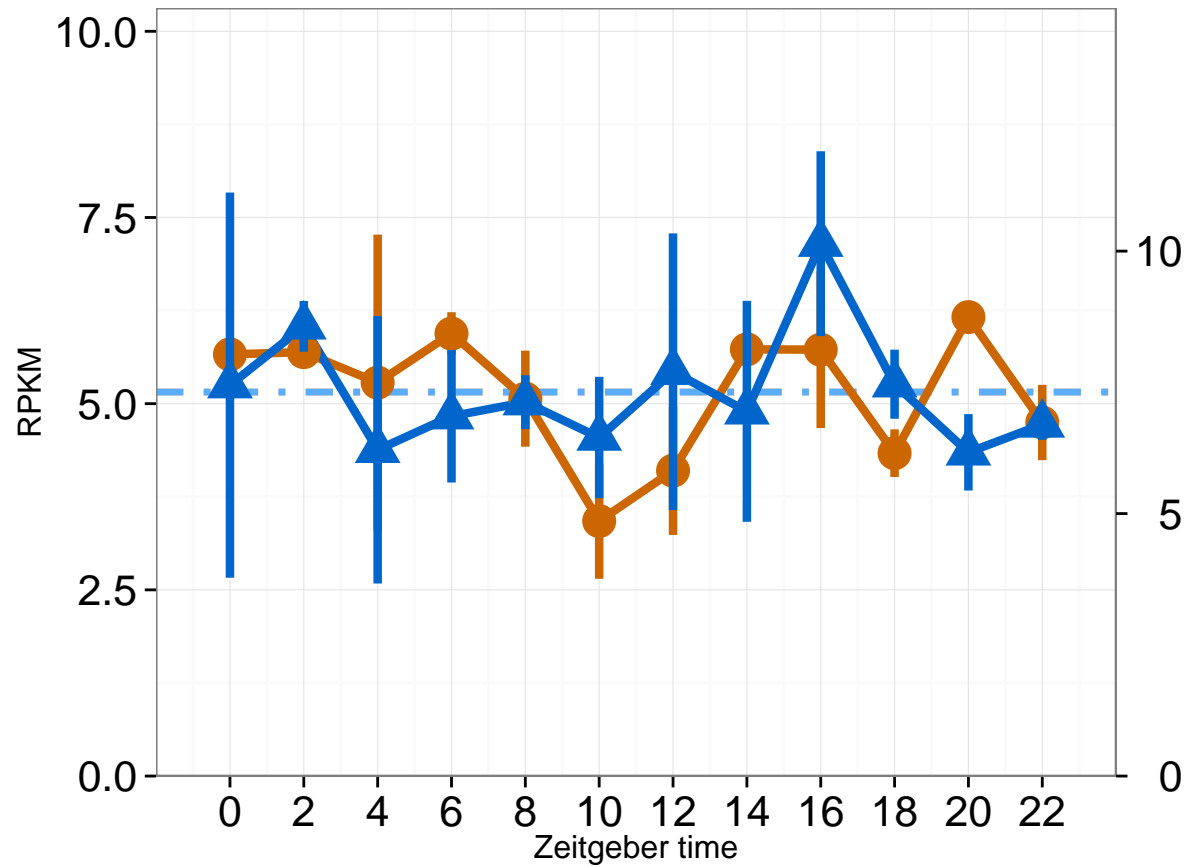

## 5330426P16Rik

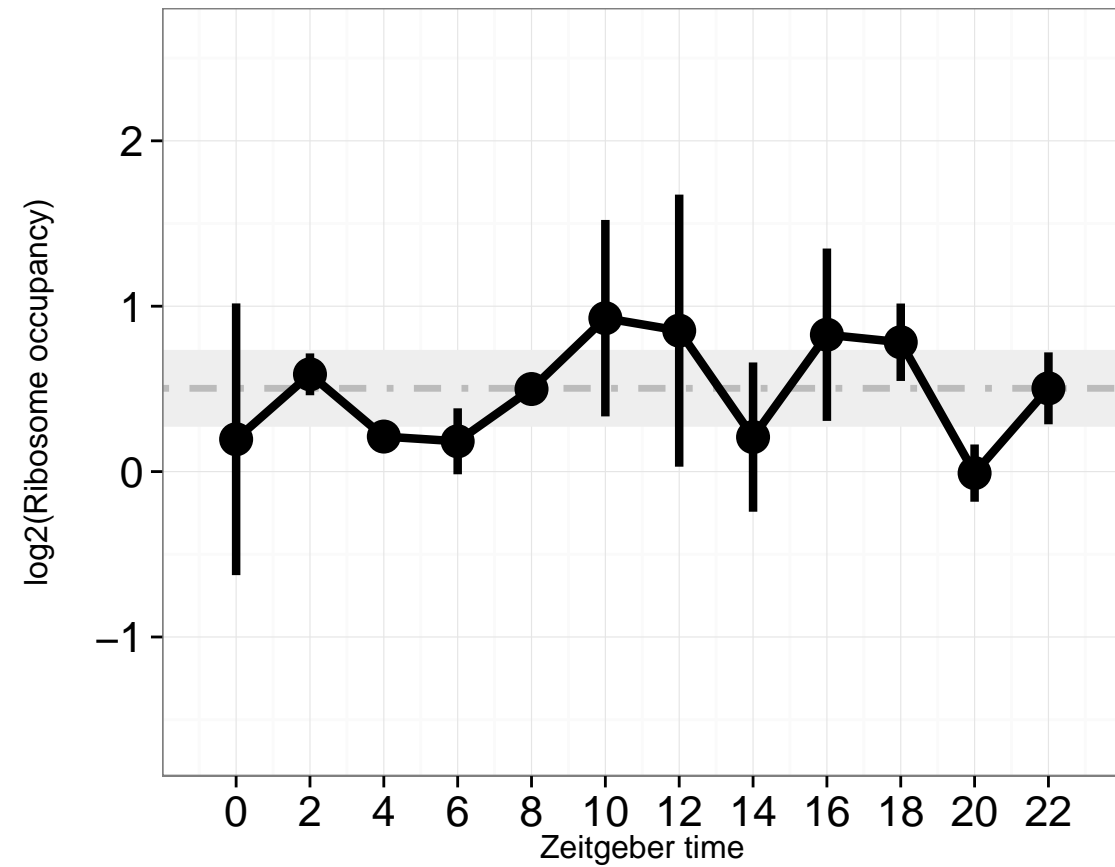

Supplement: Supplementary file 6 — Transcriptome-wide kidney RPF (blue) and RNA (orange) levels in the left panels (with “error bars” connecting the two replicates of each timepoint) and TE in the right panels. (ZIP 116896 kb) [file 13059_2017_1222_MOESM6_ESM.zip › Supp_Dataset_S1/A_RNA_non_rhythmic_RPF_non_rhythmic/5330426P16Rik_kidney_set_A.pdf]

**5430427O19Rik TR**

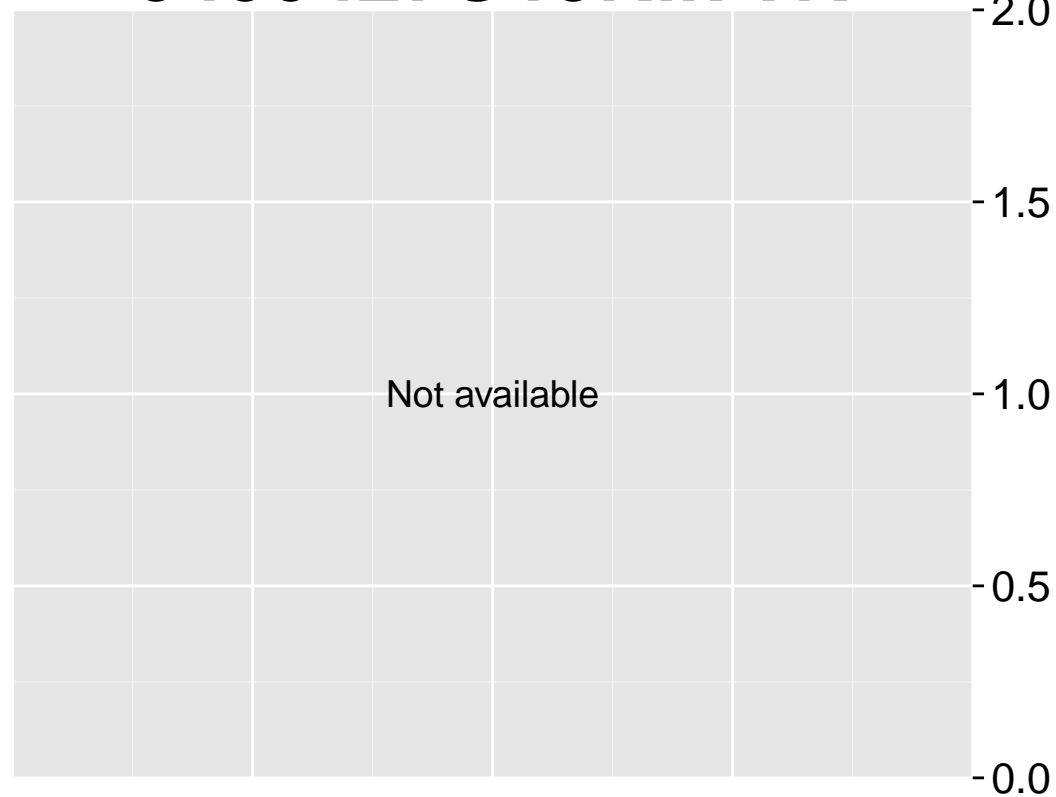

**5430427O19Rik log2(Ribosome occup**

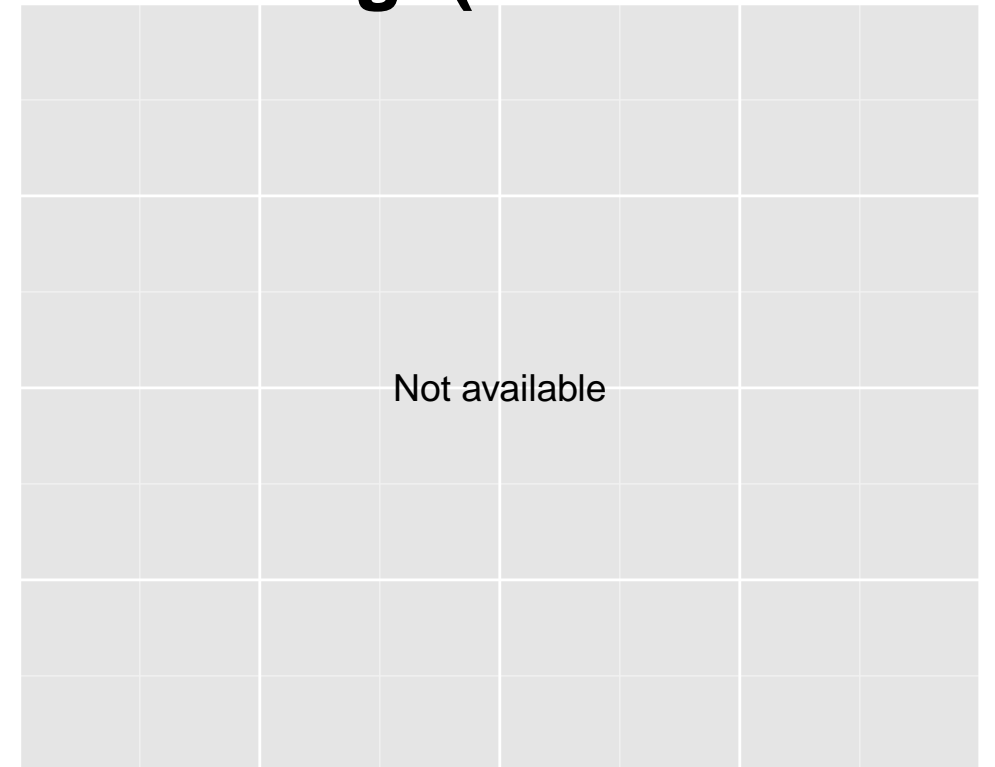

Supplement: Supplementary file 6 — Transcriptome-wide kidney RPF (blue) and RNA (orange) levels in the left panels (with “error bars” connecting the two replicates of each timepoint) and TE in the right panels. (ZIP 116896 kb) [file 13059_2017_1222_MOESM6_ESM.zip › Supp_Dataset_S1/A_RNA_non_rhythmic_RPF_non_rhythmic/5430427O19Rik_kidney_set_A.pdf]

## 5430435G22Rik

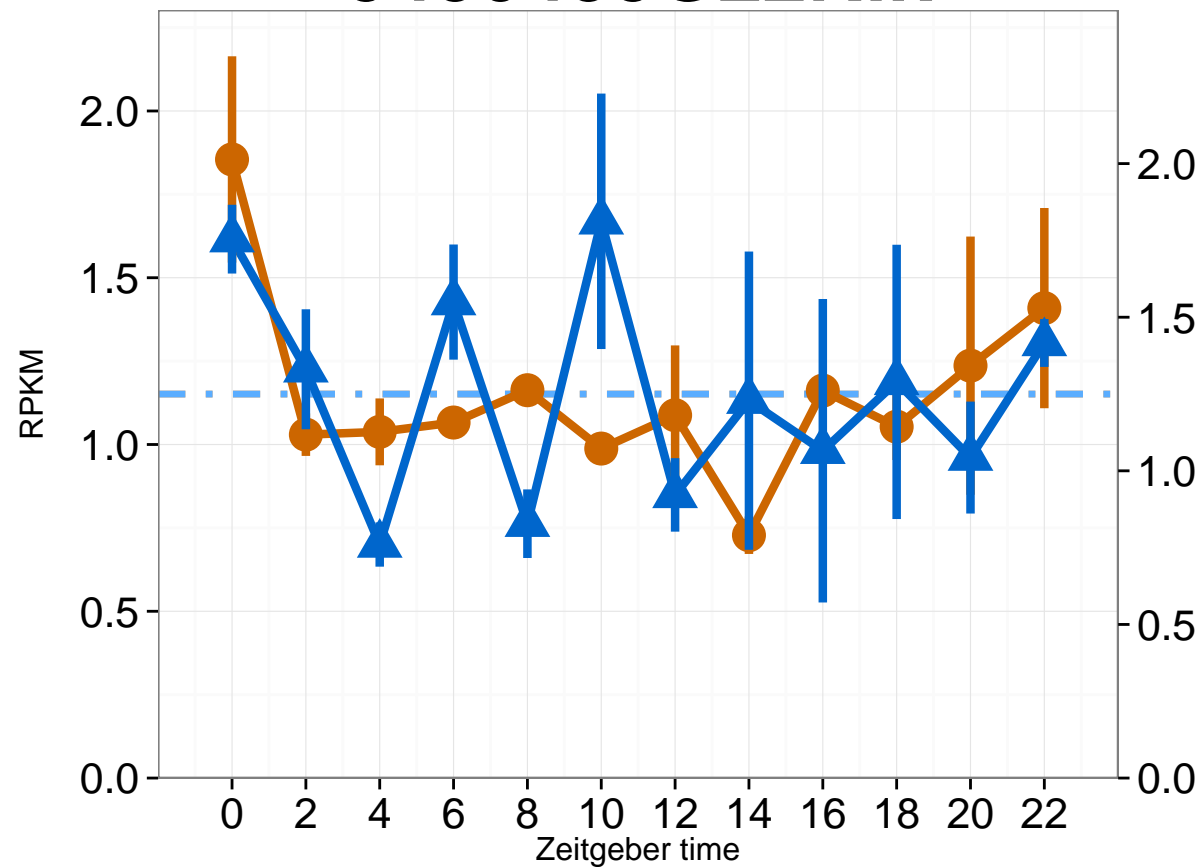

## 5430435G22Rik

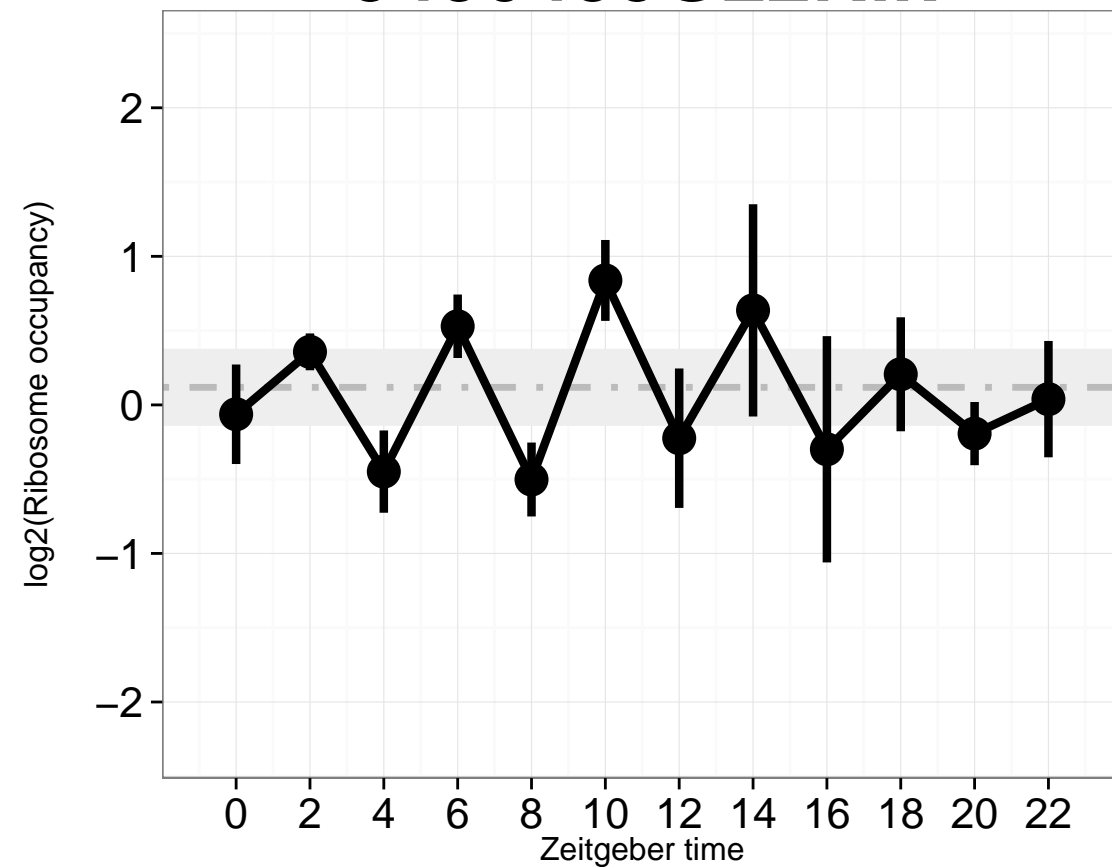

Supplement: Supplementary file 6 — Transcriptome-wide kidney RPF (blue) and RNA (orange) levels in the left panels (with “error bars” connecting the two replicates of each timepoint) and TE in the right panels. (ZIP 116896 kb) [file 13059_2017_1222_MOESM6_ESM.zip › Supp_Dataset_S1/A_RNA_non_rhythmic_RPF_non_rhythmic/5430435G22Rik_kidney_set_A.pdf]

## 5730455P16Rik

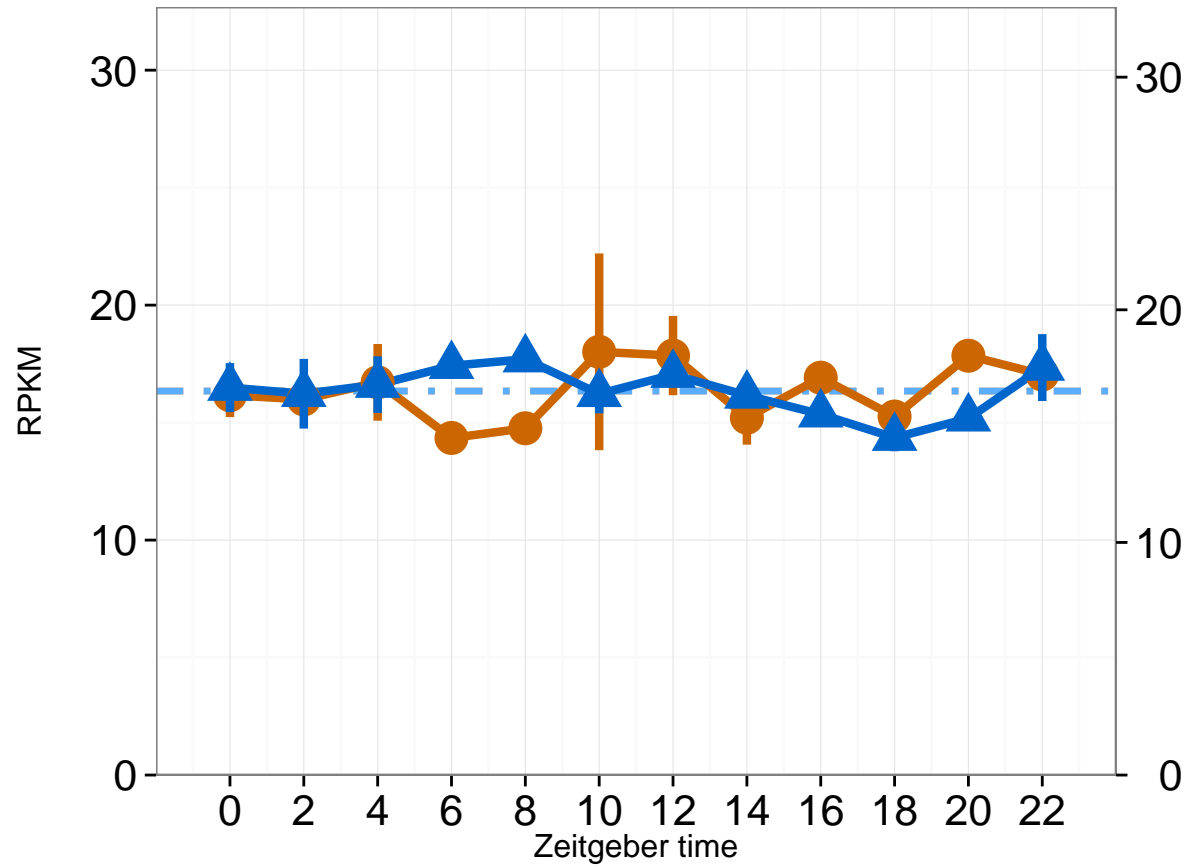

## 5730455P16Rik

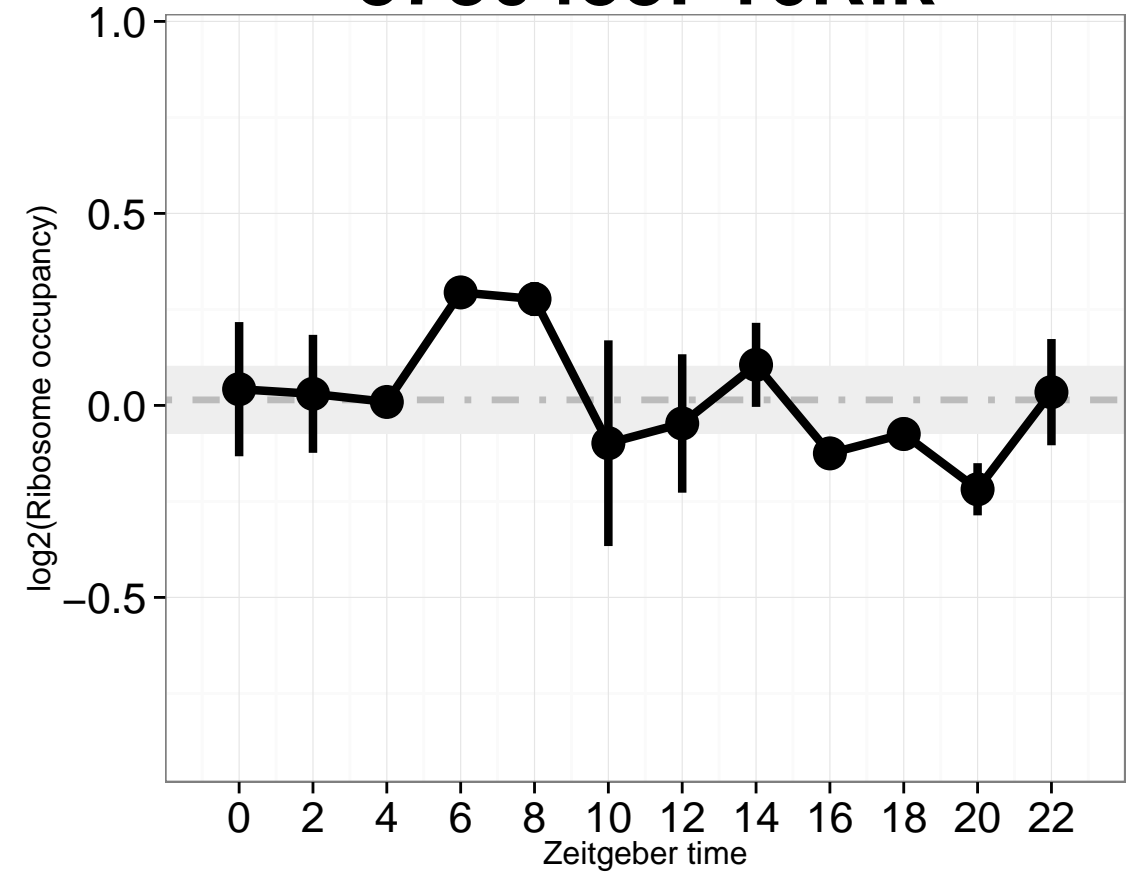

Supplement: Supplementary file 6 — Transcriptome-wide kidney RPF (blue) and RNA (orange) levels in the left panels (with “error bars” connecting the two replicates of each timepoint) and TE in the right panels. (ZIP 116896 kb) [file 13059_2017_1222_MOESM6_ESM.zip › Supp_Dataset_S1/A_RNA_non_rhythmic_RPF_non_rhythmic/5730455P16Rik_kidney_set_A.pdf]

# 5730507C01Rik

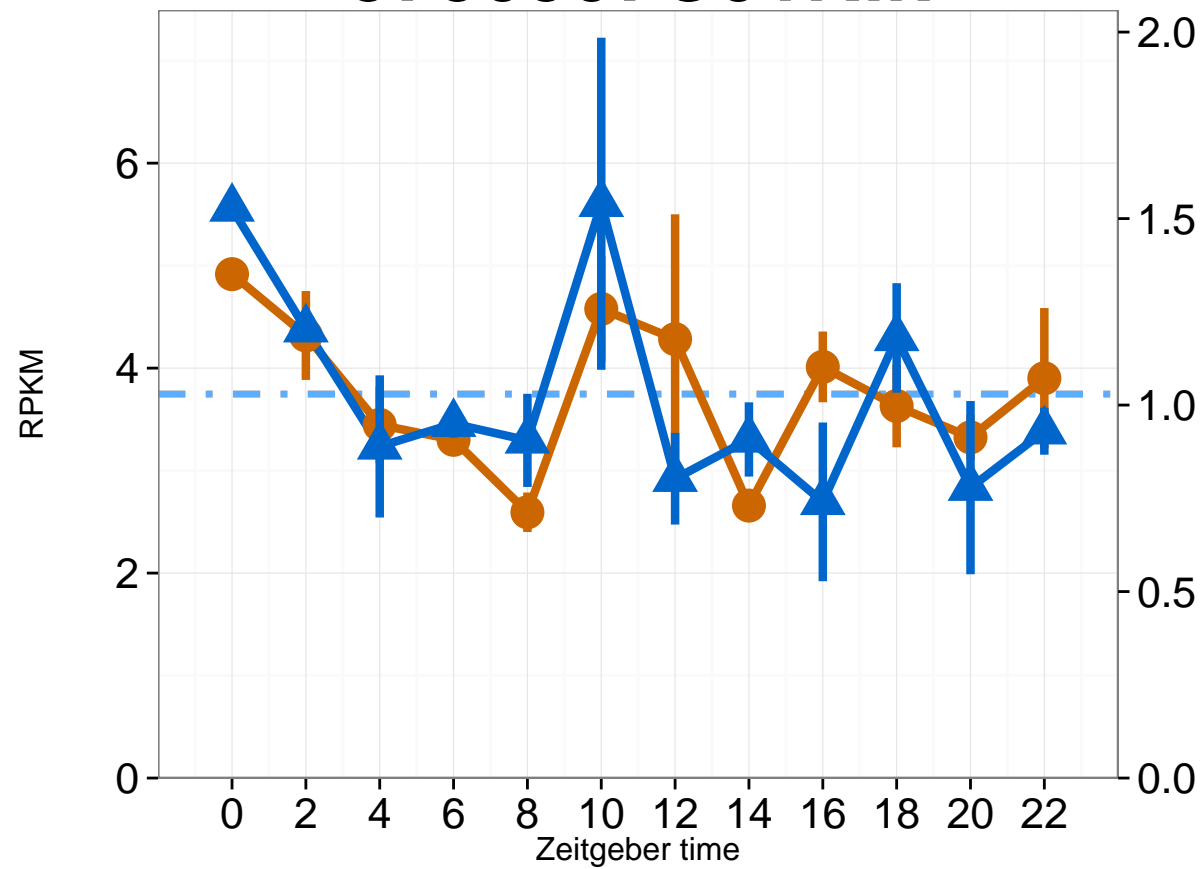

# 5730507C01Rik

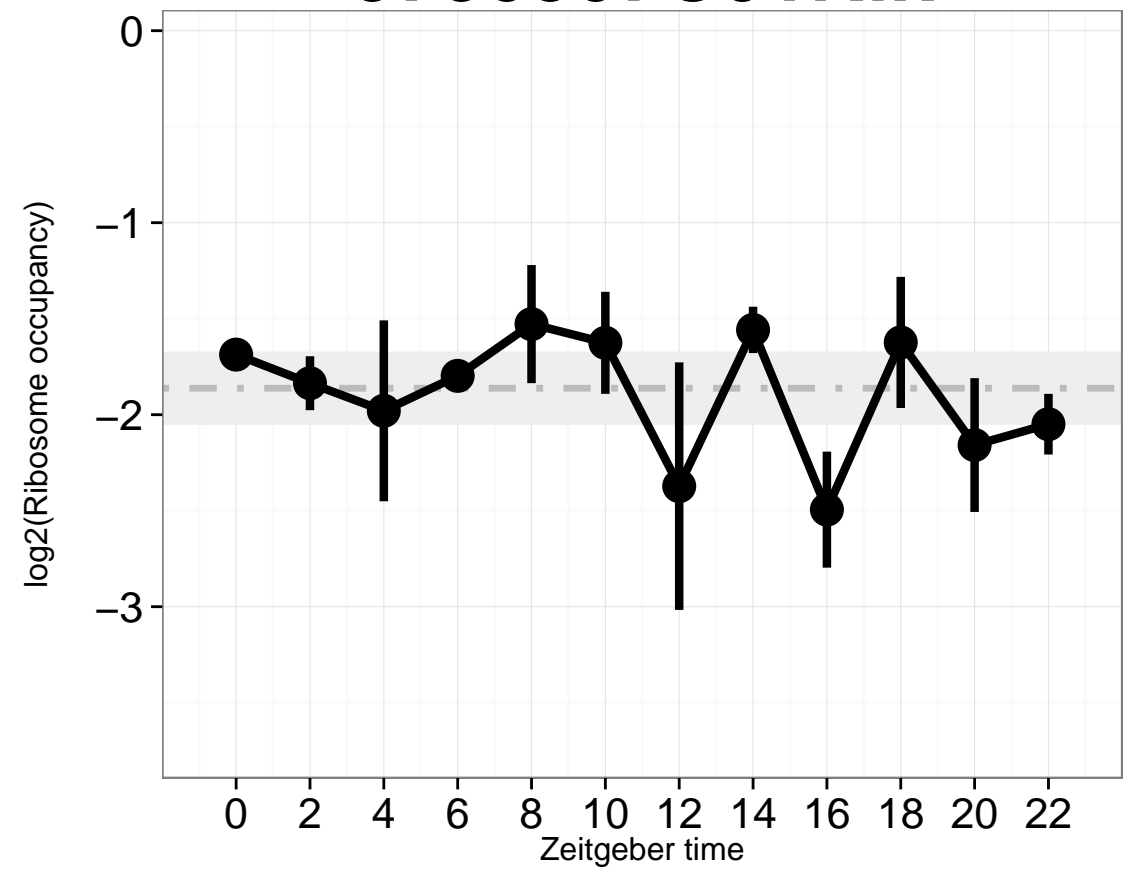

Supplement: Supplementary file 6 — Transcriptome-wide kidney RPF (blue) and RNA (orange) levels in the left panels (with “error bars” connecting the two replicates of each timepoint) and TE in the right panels. (ZIP 116896 kb) [file 13059_2017_1222_MOESM6_ESM.zip › Supp_Dataset_S1/A_RNA_non_rhythmic_RPF_non_rhythmic/5730507C01Rik_kidney_set_A.pdf]

# 5730508B09Rik

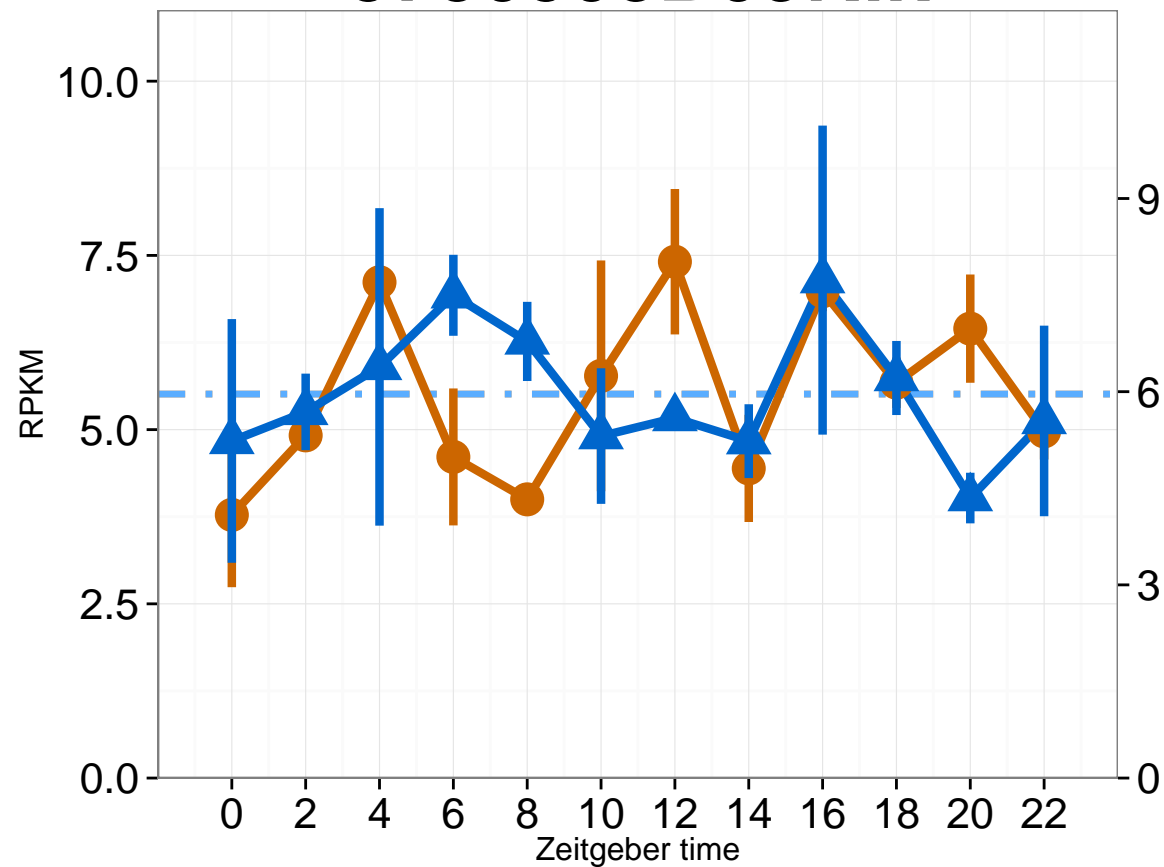

# 5730508B09Rik

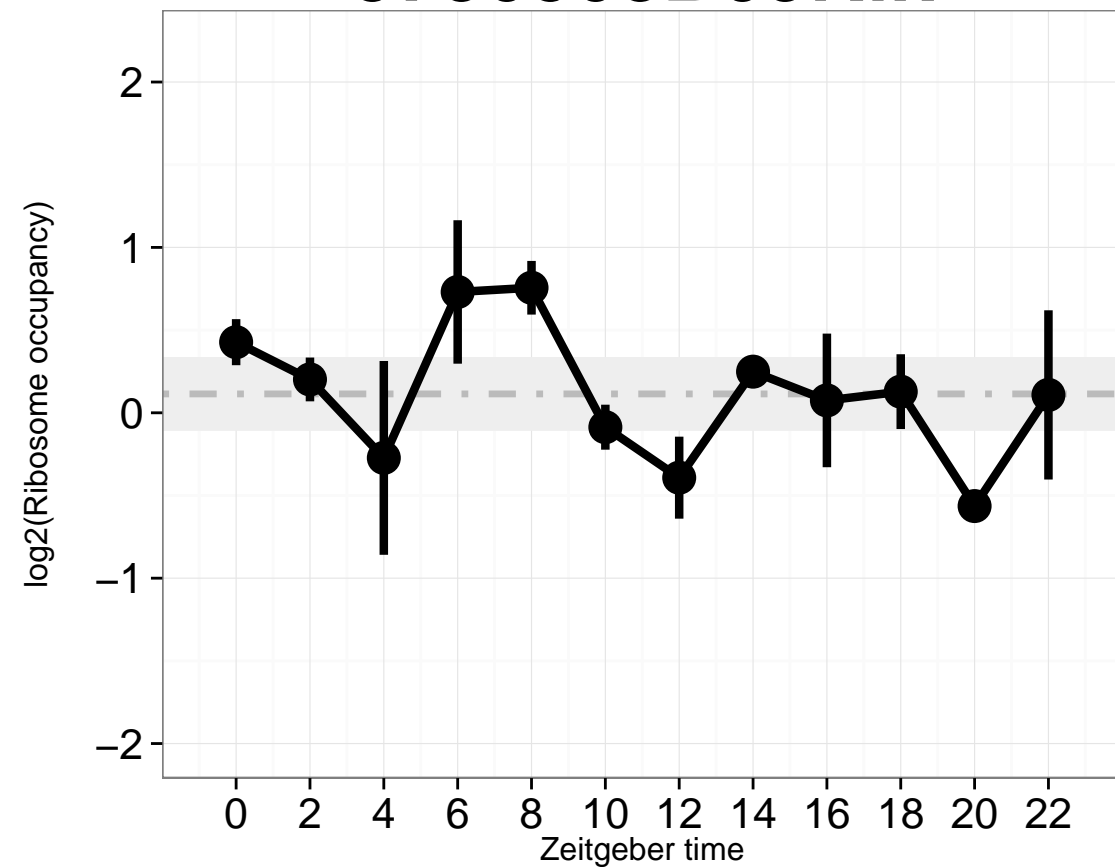

Supplement: Supplementary file 6 — Transcriptome-wide kidney RPF (blue) and RNA (orange) levels in the left panels (with “error bars” connecting the two replicates of each timepoint) and TE in the right panels. (ZIP 116896 kb) [file 13059_2017_1222_MOESM6_ESM.zip › Supp_Dataset_S1/A_RNA_non_rhythmic_RPF_non_rhythmic/5730508B09Rik_kidney_set_A.pdf]

# 5830411N06Rik

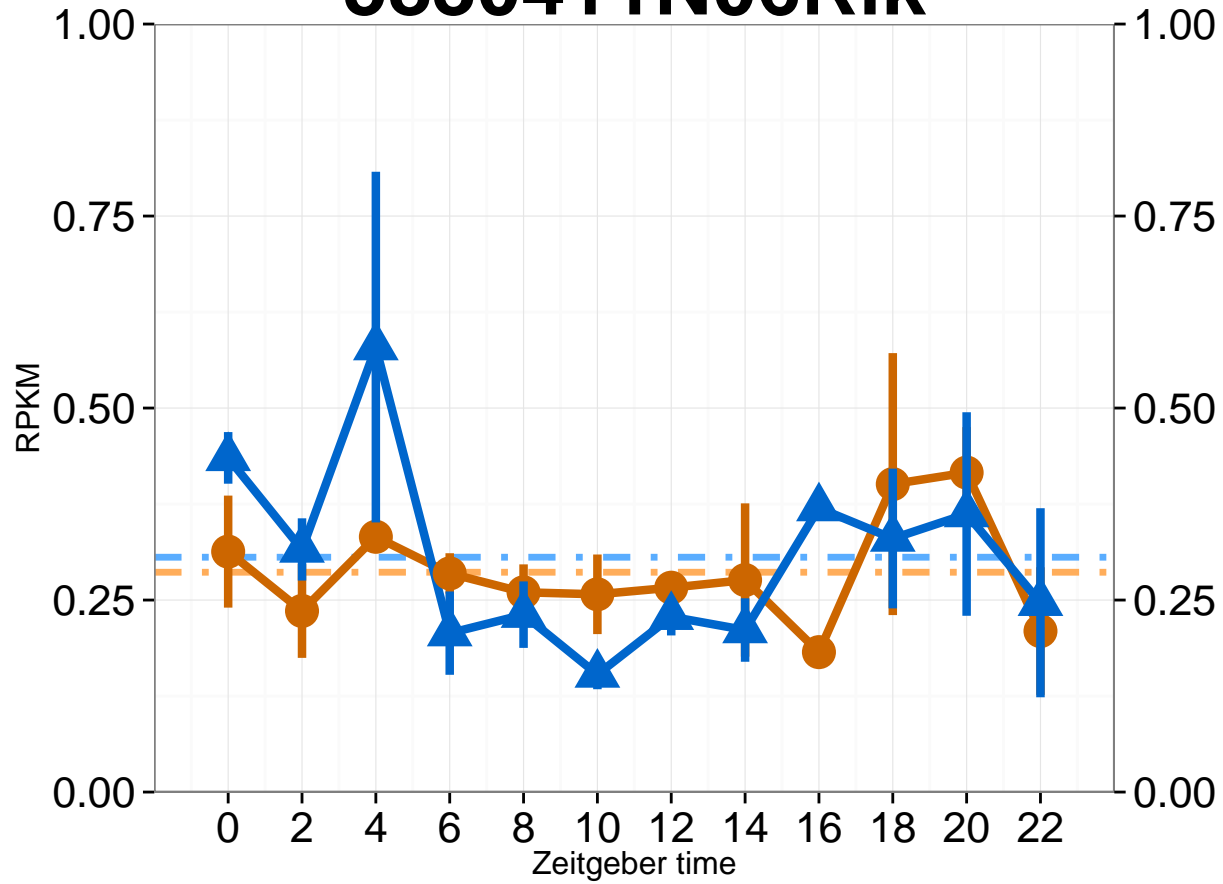

# 5830411N06Rik

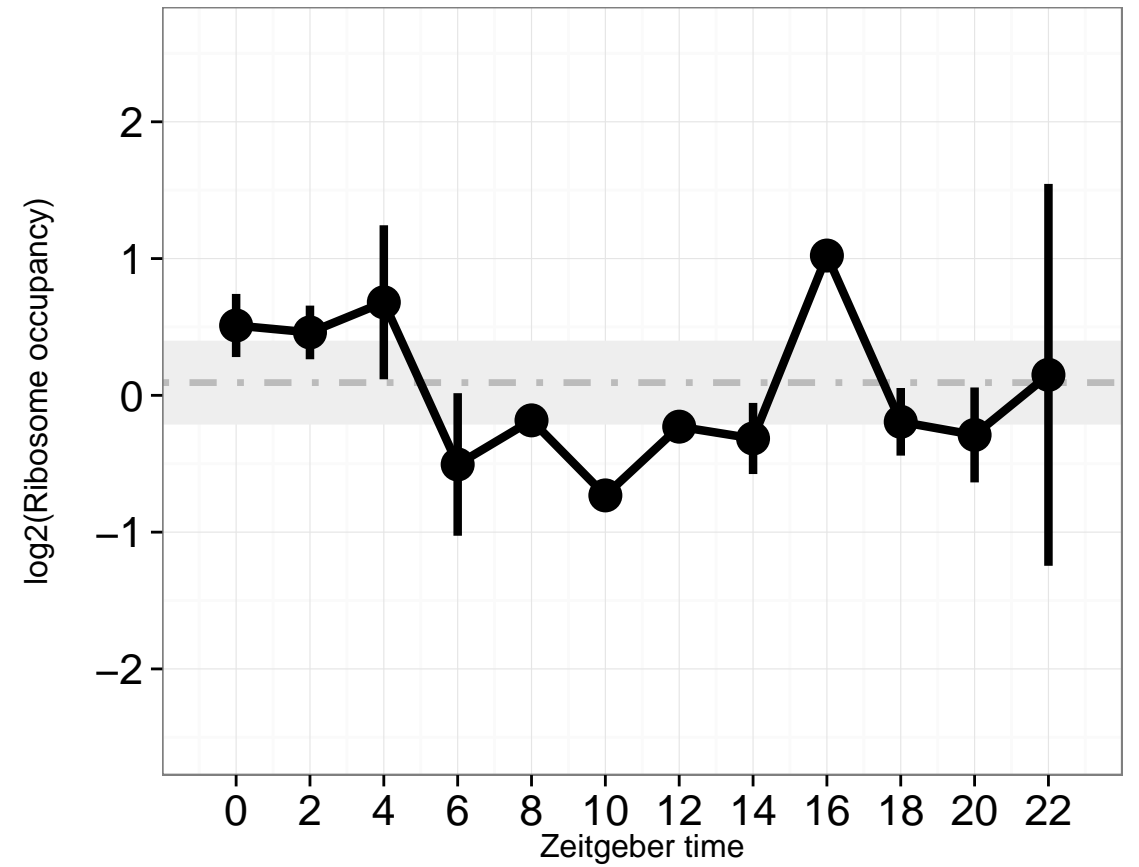

Supplement: Supplementary file 6 — Transcriptome-wide kidney RPF (blue) and RNA (orange) levels in the left panels (with “error bars” connecting the two replicates of each timepoint) and TE in the right panels. (ZIP 116896 kb) [file 13059_2017_1222_MOESM6_ESM.zip › Supp_Dataset_S1/A_RNA_non_rhythmic_RPF_non_rhythmic/5830411N06Rik_kidney_set_A.pdf]

# 5830415F09Rik

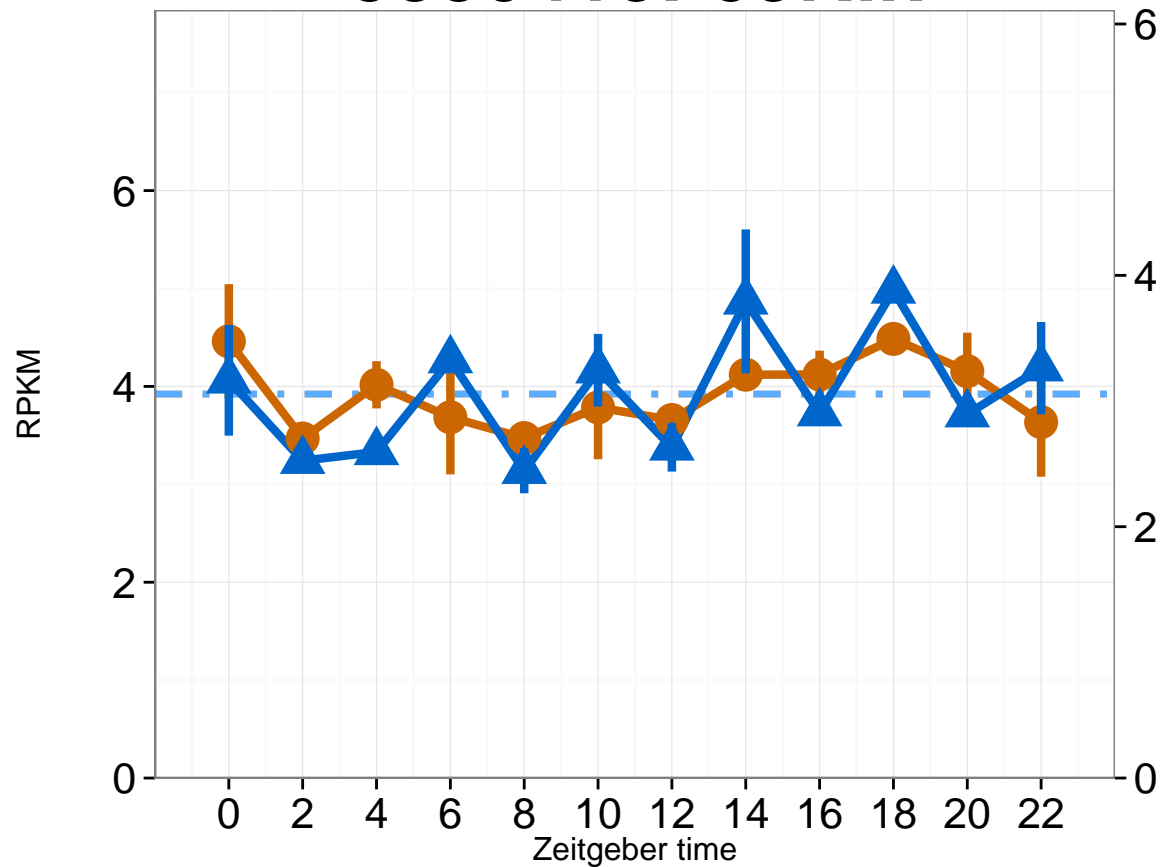

# 5830415F09Rik

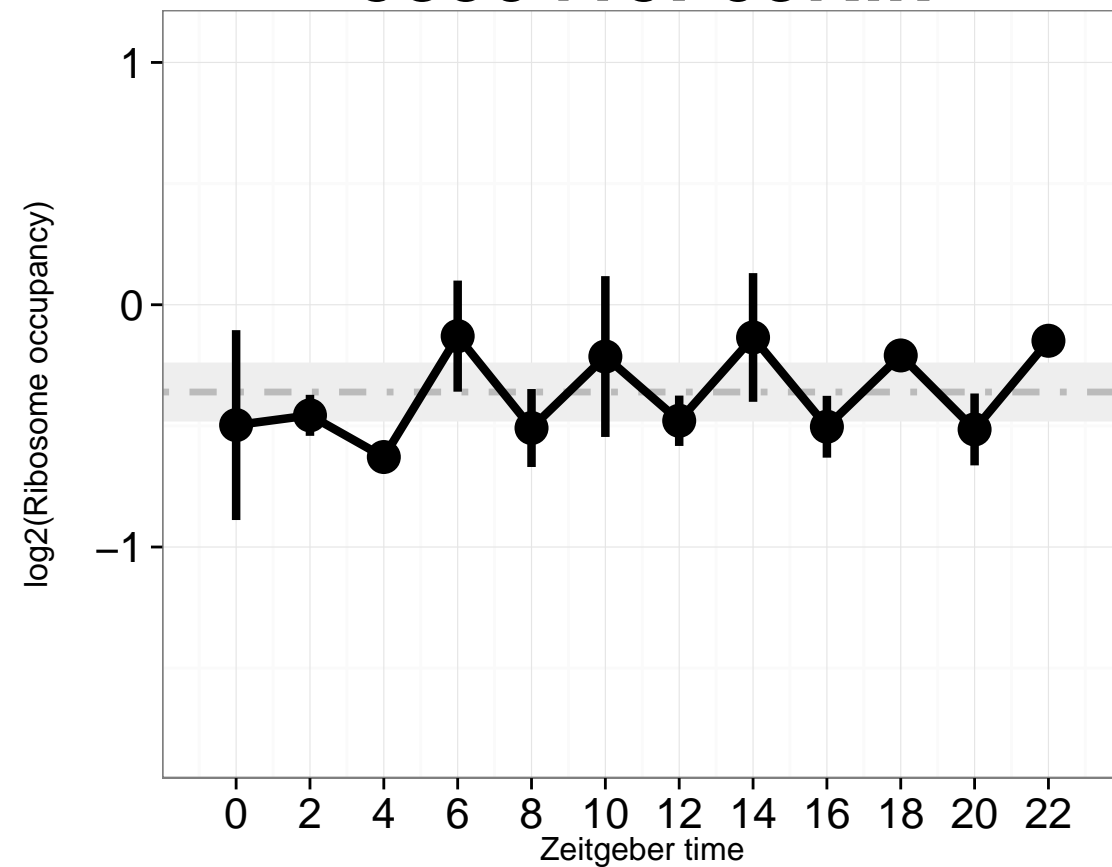

Supplement: Supplementary file 6 — Transcriptome-wide kidney RPF (blue) and RNA (orange) levels in the left panels (with “error bars” connecting the two replicates of each timepoint) and TE in the right panels. (ZIP 116896 kb) [file 13059_2017_1222_MOESM6_ESM.zip › Supp_Dataset_S1/A_RNA_non_rhythmic_RPF_non_rhythmic/5830415F09Rik_kidney_set_A.pdf]

## 5830418K08Rik

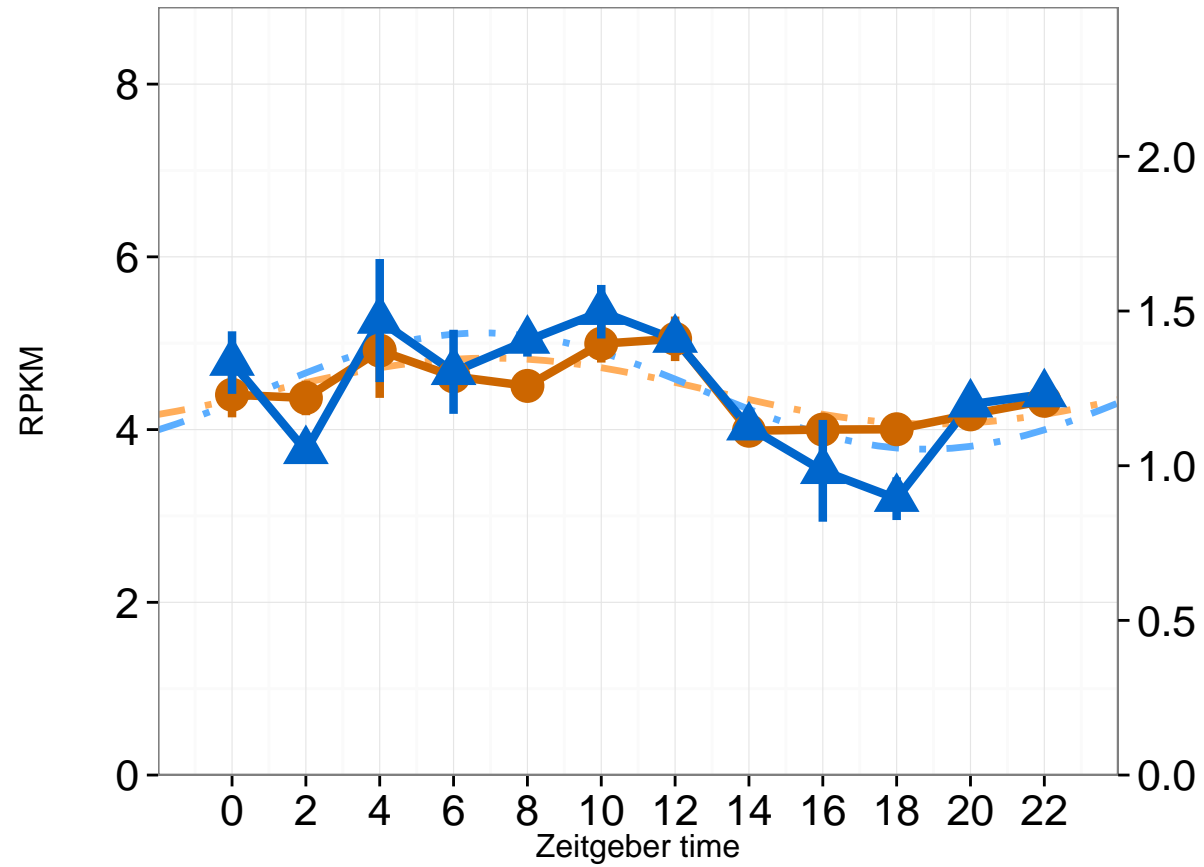

## 5830418K08Rik

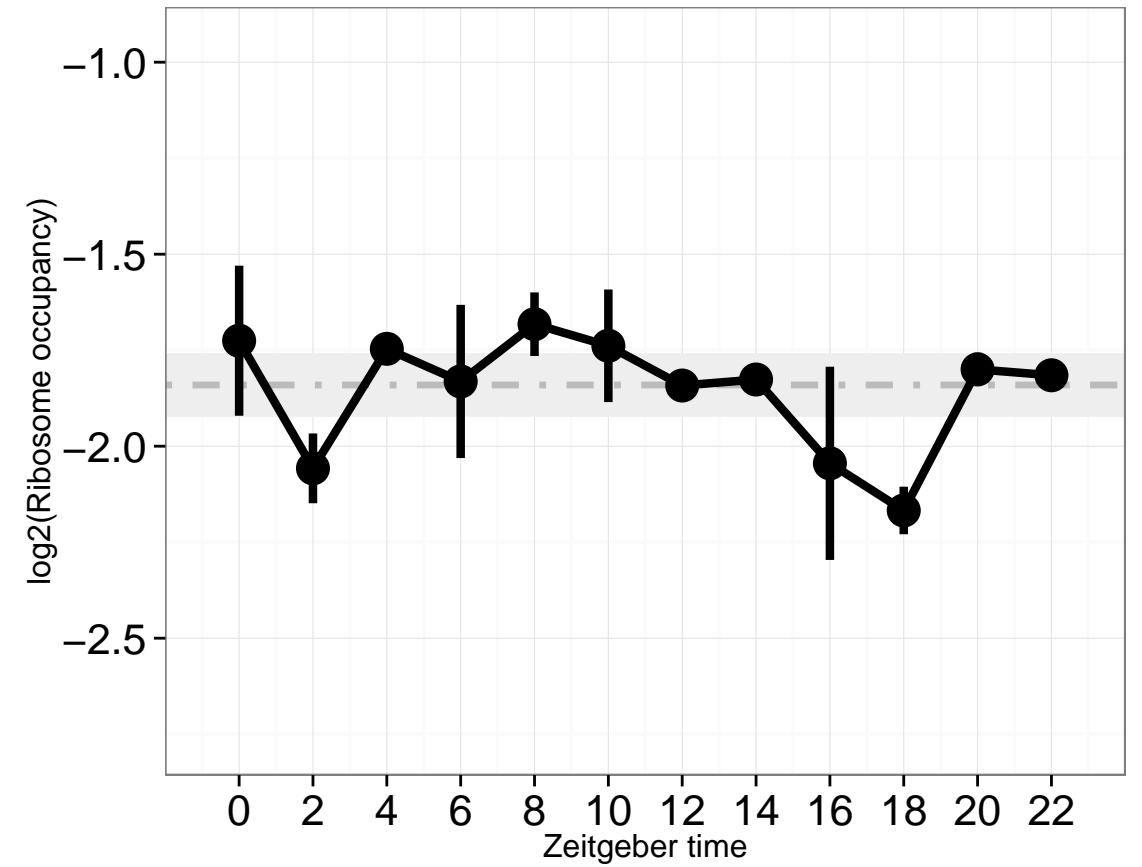

Supplement: Supplementary file 6 — Transcriptome-wide kidney RPF (blue) and RNA (orange) levels in the left panels (with “error bars” connecting the two replicates of each timepoint) and TE in the right panels. (ZIP 116896 kb) [file 13059_2017_1222_MOESM6_ESM.zip › Supp_Dataset_S1/A_RNA_non_rhythmic_RPF_non_rhythmic/5830418K08Rik_kidney_set_A.pdf]

**5830454E08Rik**

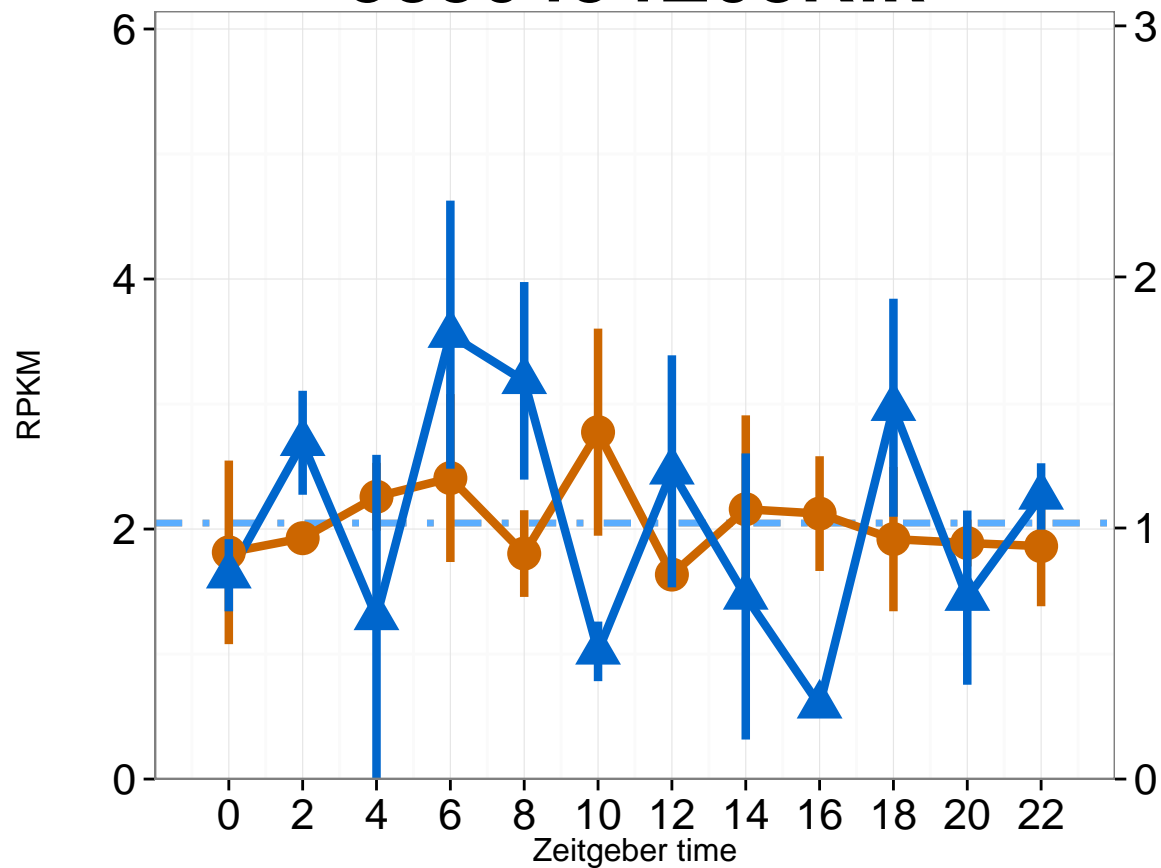

**5830454E08Rik**

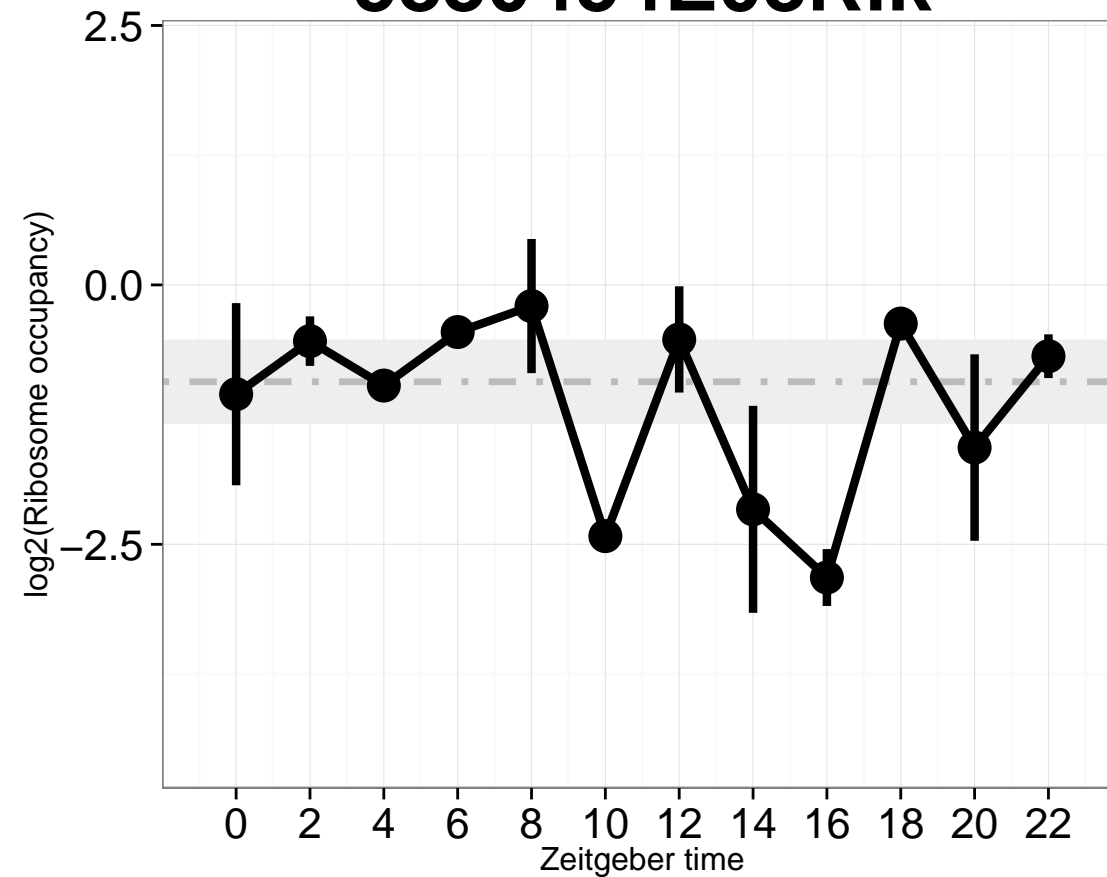

Supplement: Supplementary file 6 — Transcriptome-wide kidney RPF (blue) and RNA (orange) levels in the left panels (with “error bars” connecting the two replicates of each timepoint) and TE in the right panels. (ZIP 116896 kb) [file 13059_2017_1222_MOESM6_ESM.zip › Supp_Dataset_S1/A_RNA_non_rhythmic_RPF_non_rhythmic/5830454E08Rik_kidney_set_A.pdf]

## 5830473C10Rik

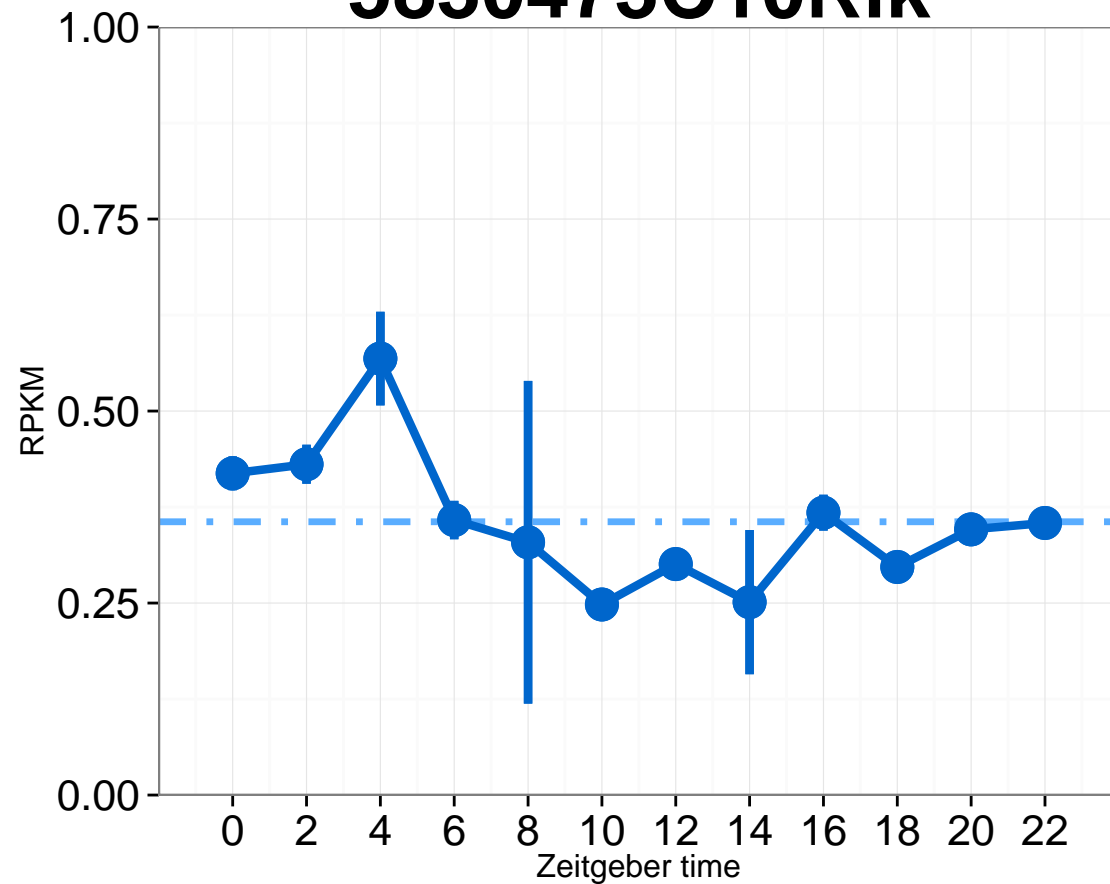

## 5830473C10Rik log2(Ribosome occup

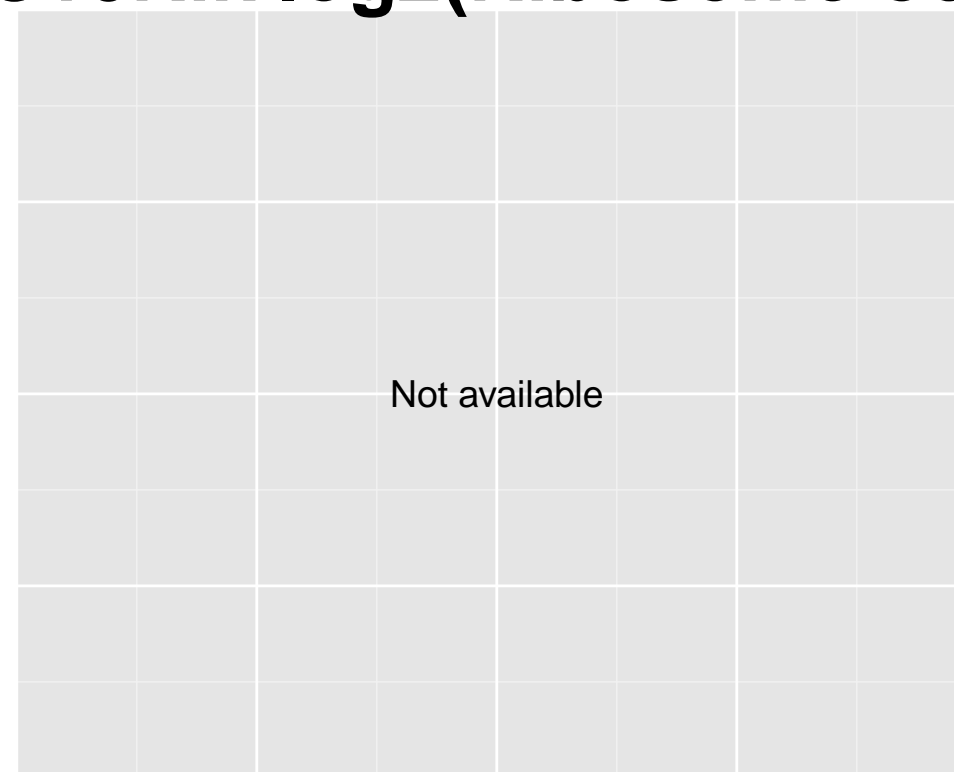

Supplement: Supplementary file 6 — Transcriptome-wide kidney RPF (blue) and RNA (orange) levels in the left panels (with “error bars” connecting the two replicates of each timepoint) and TE in the right panels. (ZIP 116896 kb) [file 13059_2017_1222_MOESM6_ESM.zip › Supp_Dataset_S1/A_RNA_non_rhythmic_RPF_non_rhythmic/5830473C10Rik_kidney_set_A.pdf]

## 6030419C18Rik

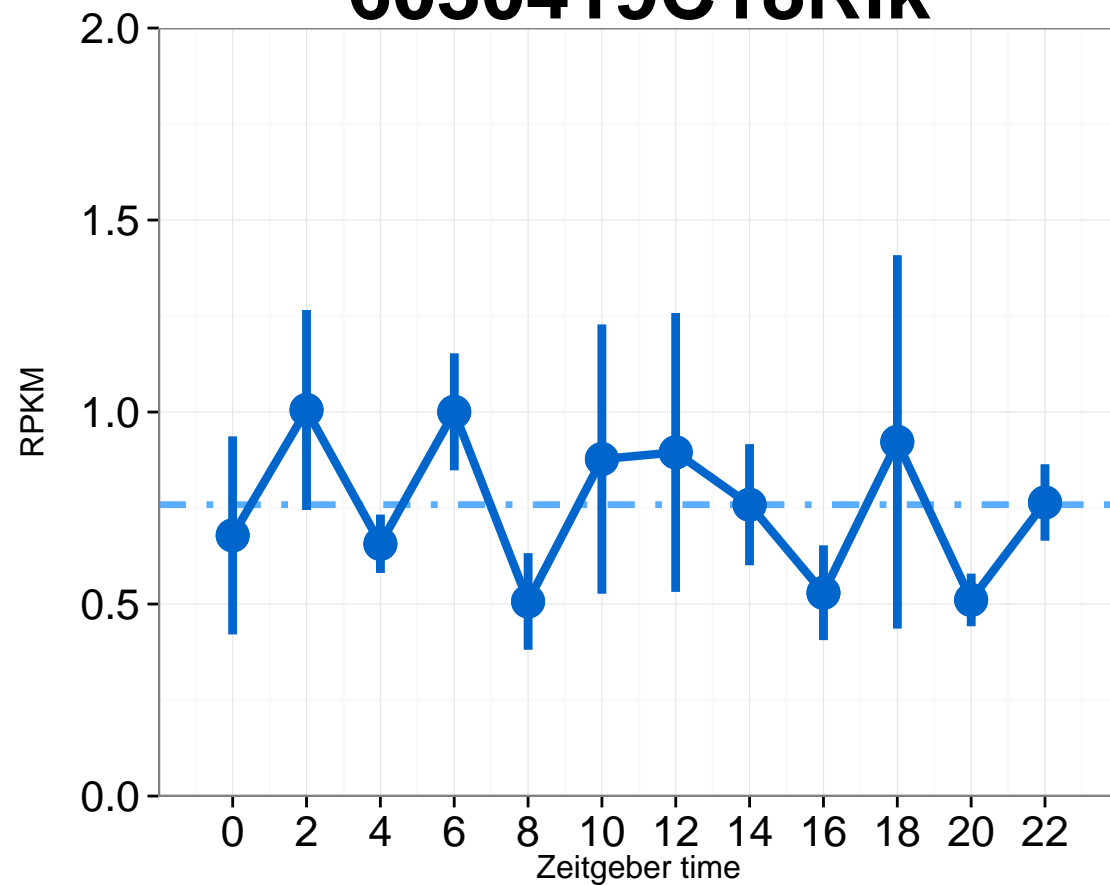

## 6030419C18Rik log<sub>2</sub>(Ribosome occup

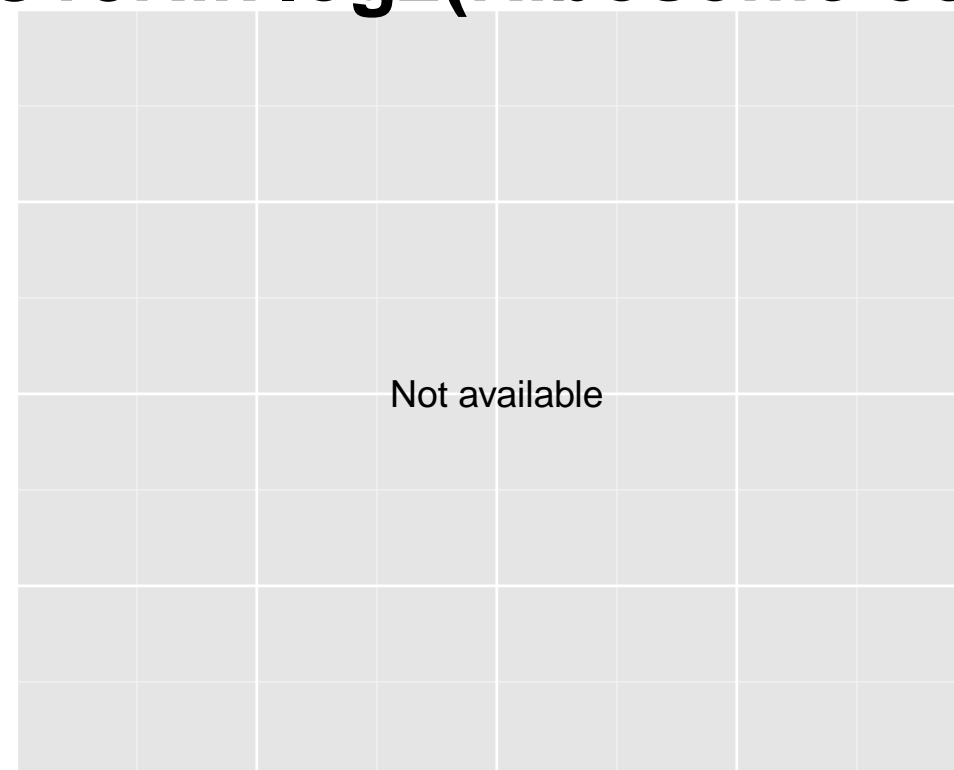

Supplement: Supplementary file 6 — Transcriptome-wide kidney RPF (blue) and RNA (orange) levels in the left panels (with “error bars” connecting the two replicates of each timepoint) and TE in the right panels. (ZIP 116896 kb) [file 13059_2017_1222_MOESM6_ESM.zip › Supp_Dataset_S1/A_RNA_non_rhythmic_RPF_non_rhythmic/6030419C18Rik_kidney_set_A.pdf]

# 6030458C11Rik

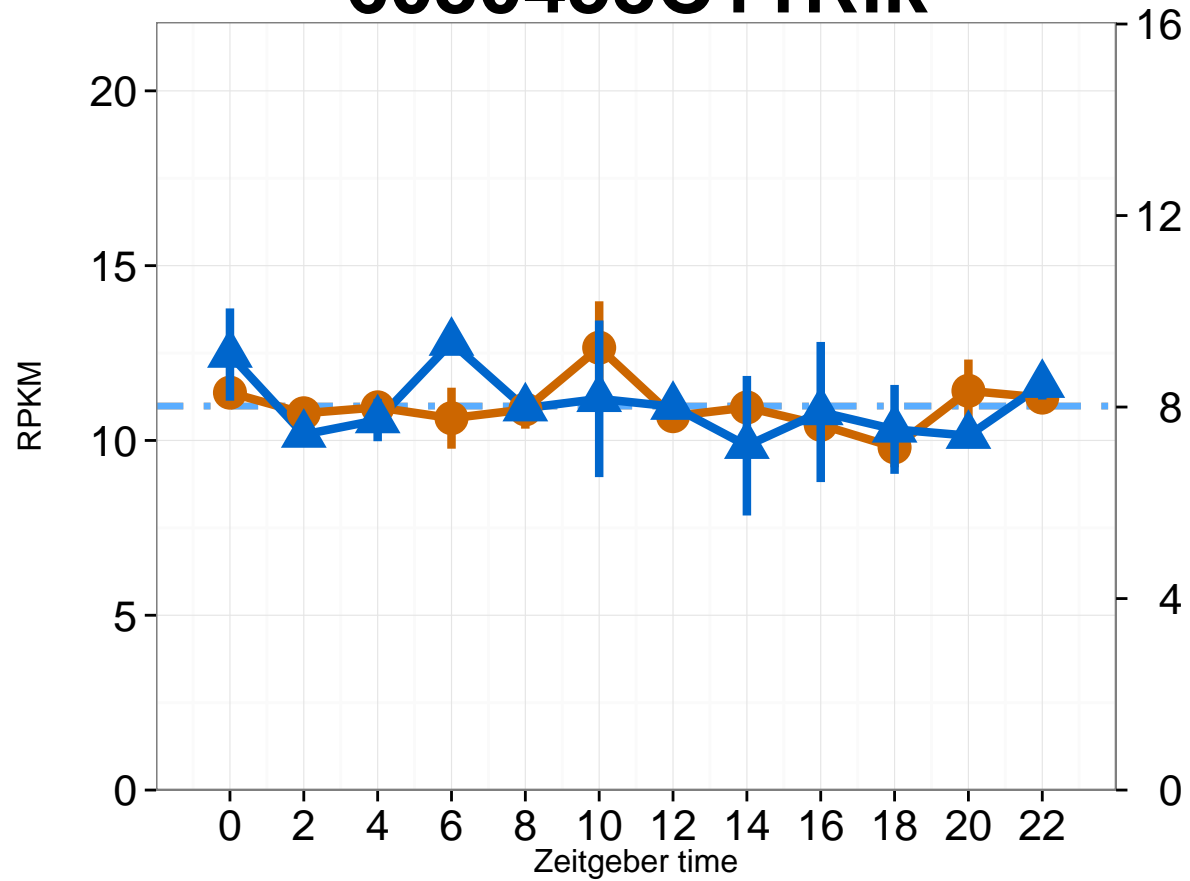

# 6030458C11Rik

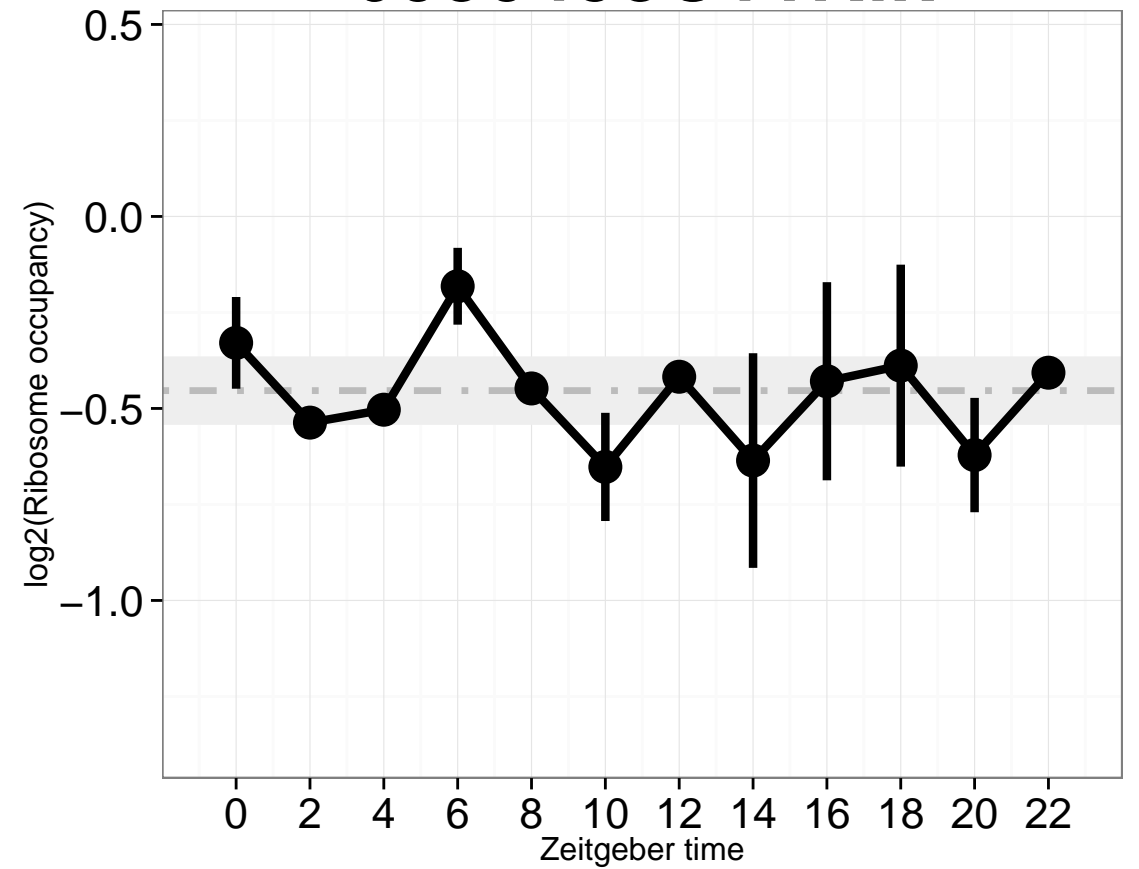

Supplement: Supplementary file 6 — Transcriptome-wide kidney RPF (blue) and RNA (orange) levels in the left panels (with “error bars” connecting the two replicates of each timepoint) and TE in the right panels. (ZIP 116896 kb) [file 13059_2017_1222_MOESM6_ESM.zip › Supp_Dataset_S1/A_RNA_non_rhythmic_RPF_non_rhythmic/6030458C11Rik_kidney_set_A.pdf]

# 6330403L08Rik

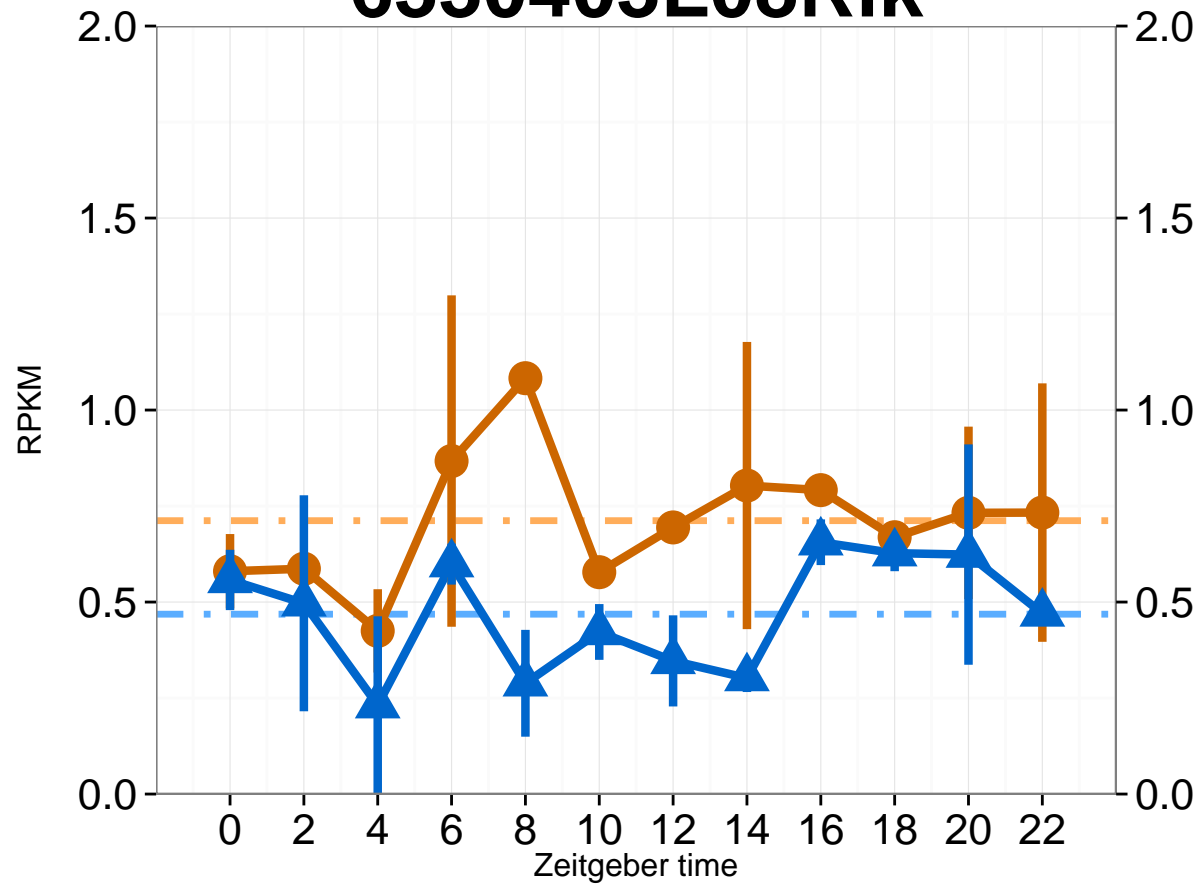

# 6330403L08Rik

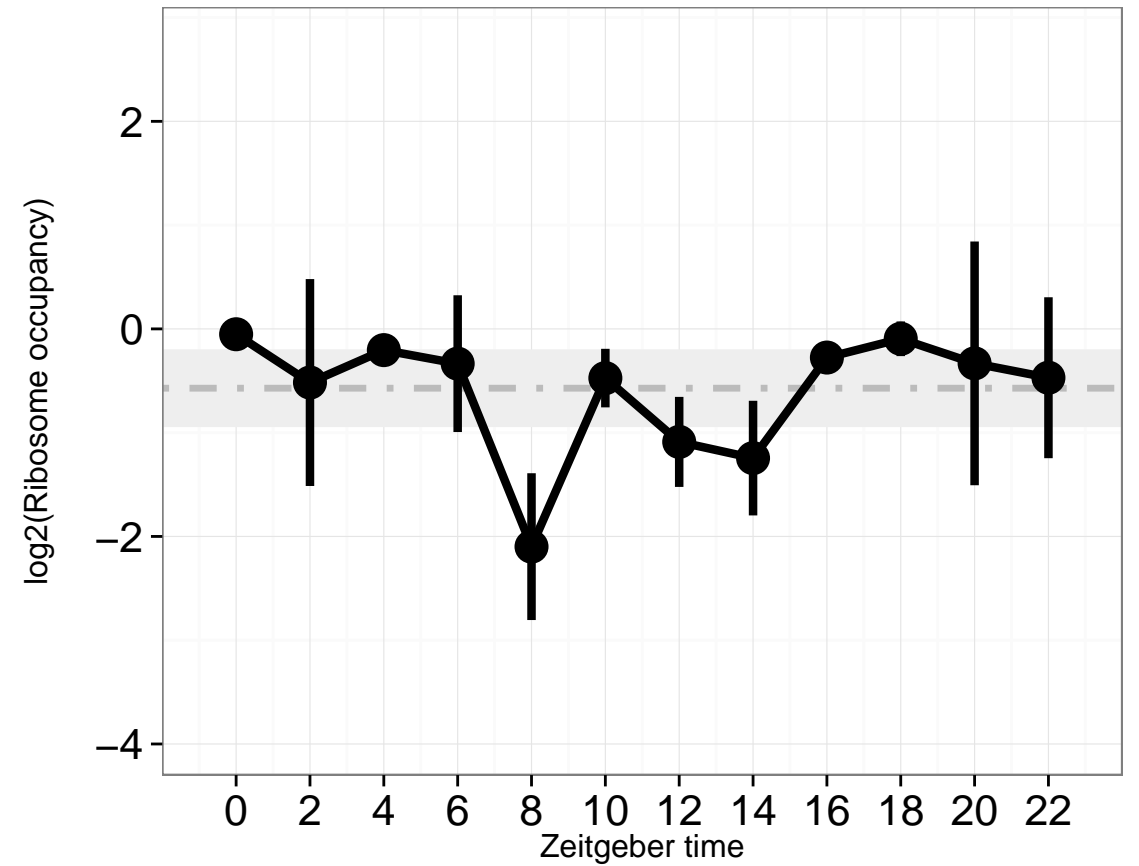

Supplement: Supplementary file 6 — Transcriptome-wide kidney RPF (blue) and RNA (orange) levels in the left panels (with “error bars” connecting the two replicates of each timepoint) and TE in the right panels. (ZIP 116896 kb) [file 13059_2017_1222_MOESM6_ESM.zip › Supp_Dataset_S1/A_RNA_non_rhythmic_RPF_non_rhythmic/6330403L08Rik_kidney_set_A.pdf]

# 6330408A02Rik

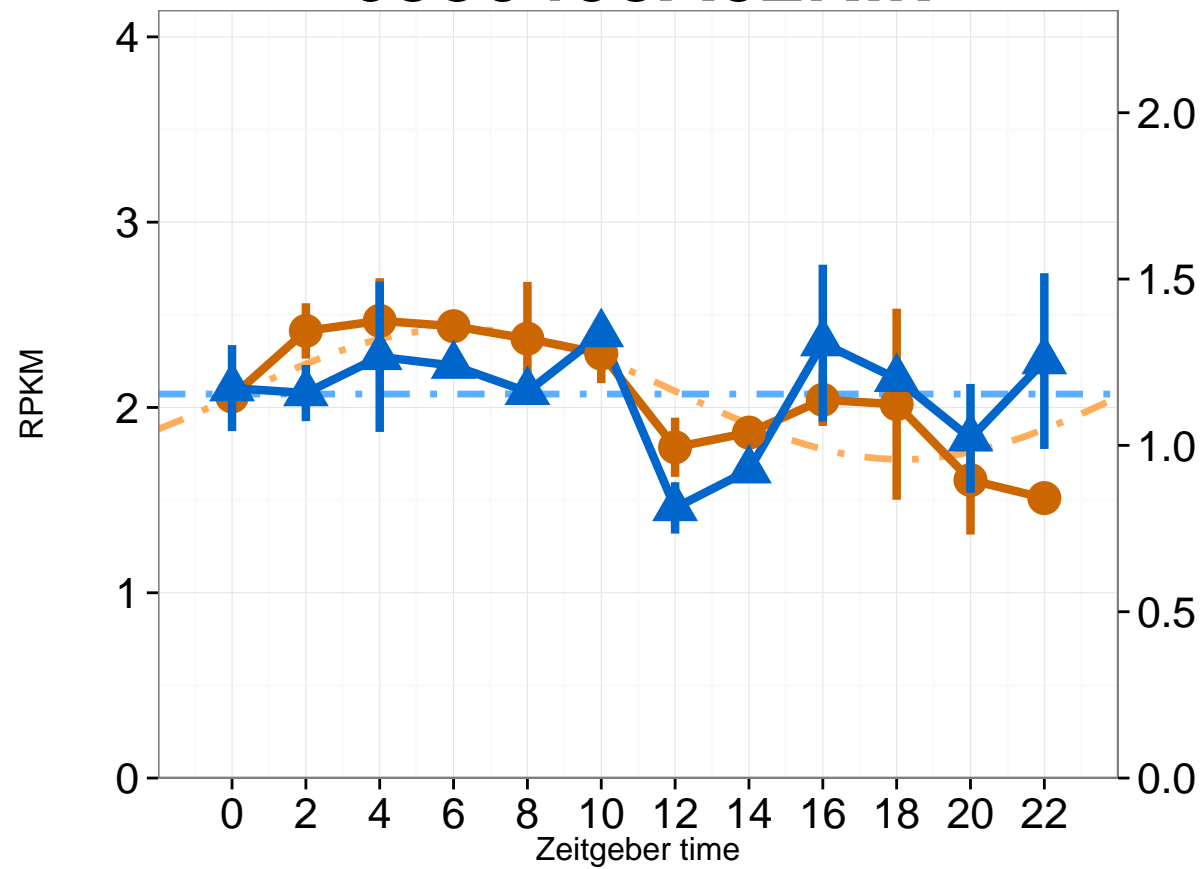

# 6330408A02Rik

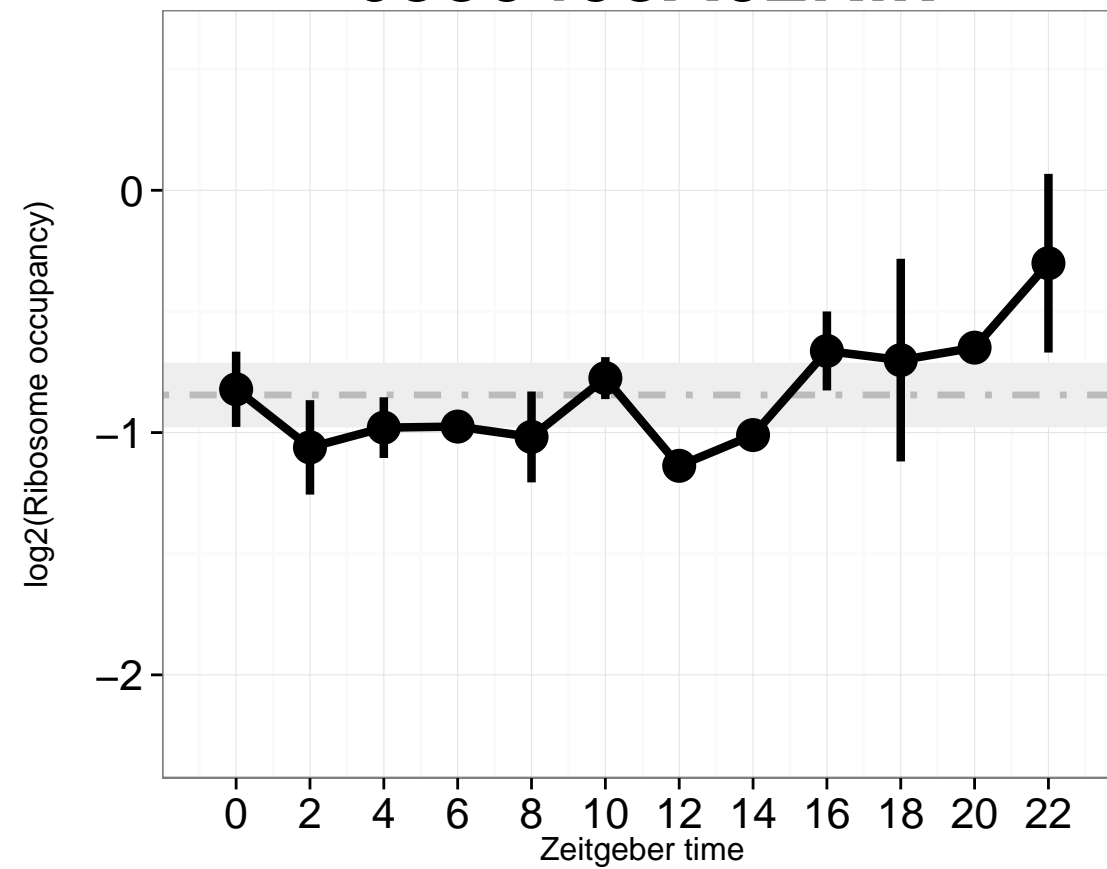

Supplement: Supplementary file 6 — Transcriptome-wide kidney RPF (blue) and RNA (orange) levels in the left panels (with “error bars” connecting the two replicates of each timepoint) and TE in the right panels. (ZIP 116896 kb) [file 13059_2017_1222_MOESM6_ESM.zip › Supp_Dataset_S1/A_RNA_non_rhythmic_RPF_non_rhythmic/6330408A02Rik_kidney_set_A.pdf]

# 6330416G13Rik

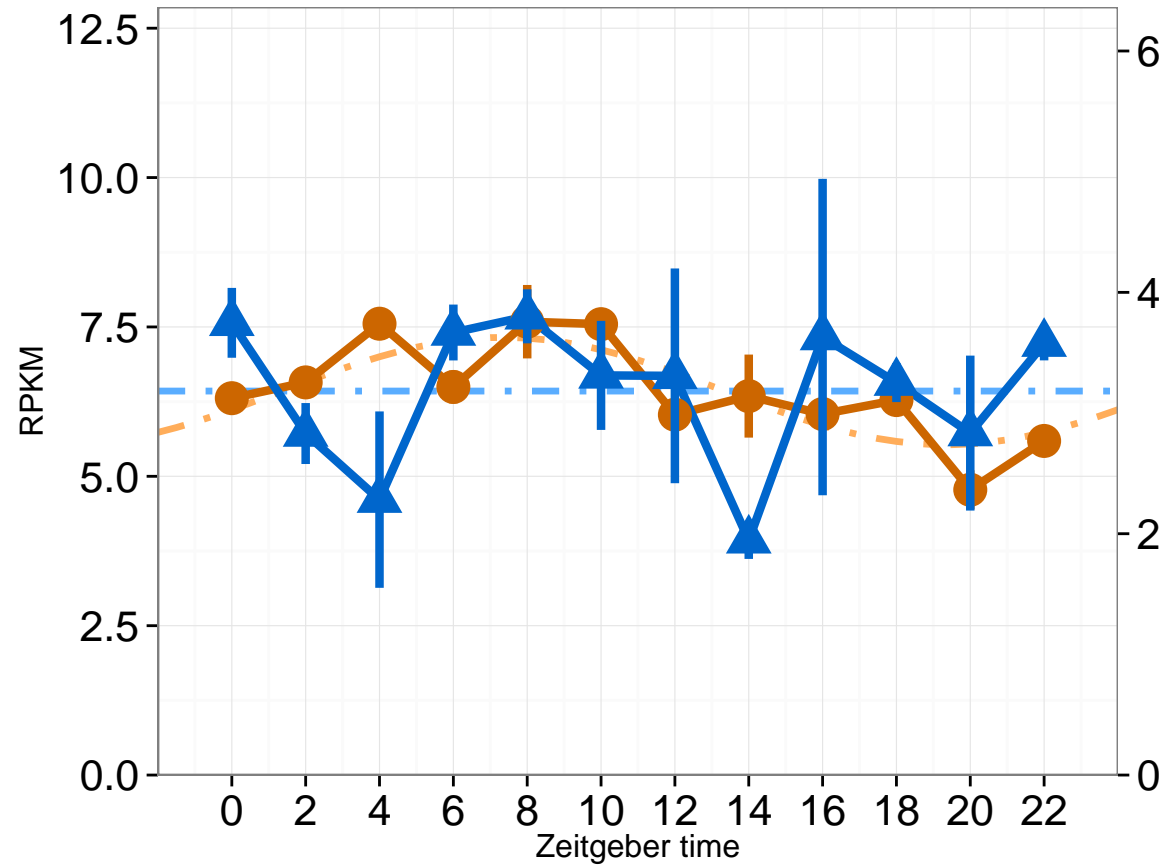

# 6330416G13Rik

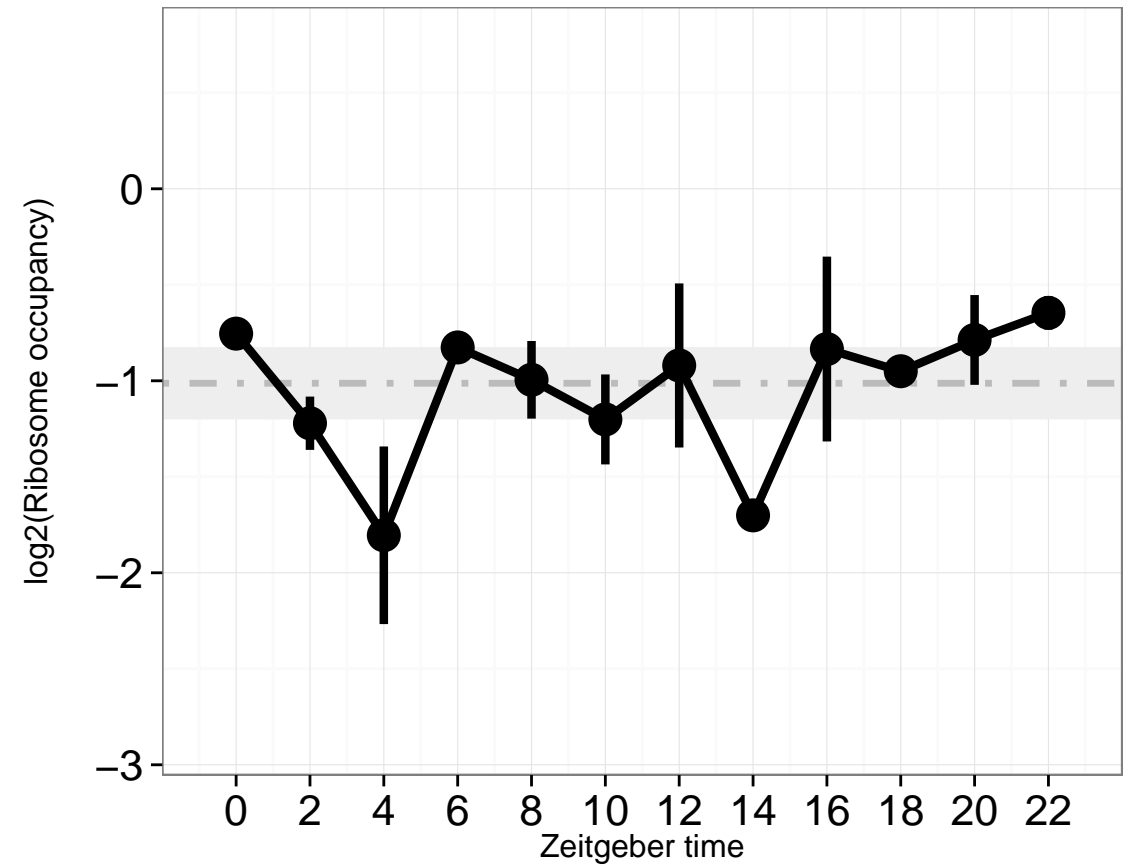

Supplement: Supplementary file 6 — Transcriptome-wide kidney RPF (blue) and RNA (orange) levels in the left panels (with “error bars” connecting the two replicates of each timepoint) and TE in the right panels. (ZIP 116896 kb) [file 13059_2017_1222_MOESM6_ESM.zip › Supp_Dataset_S1/A_RNA_non_rhythmic_RPF_non_rhythmic/6330416G13Rik_kidney_set_A.pdf]

# 6330419J24Rik

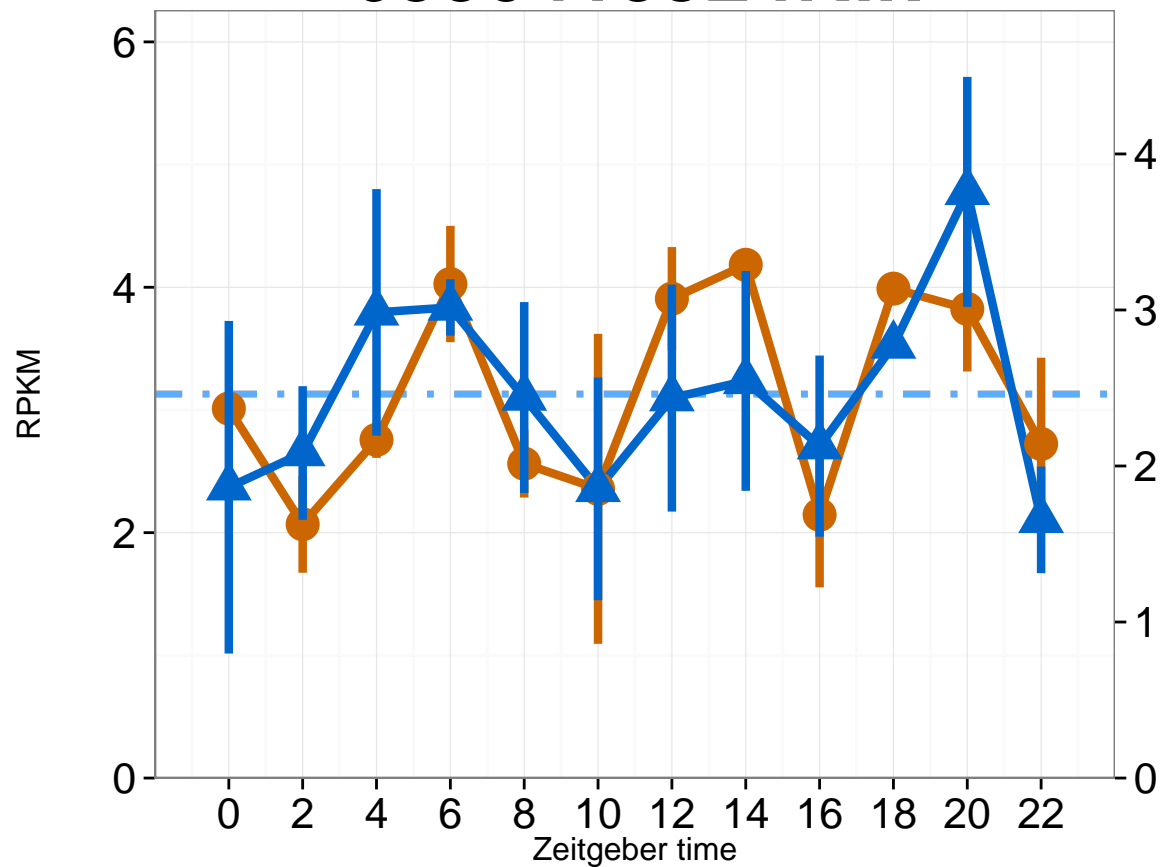

# 6330419J24Rik

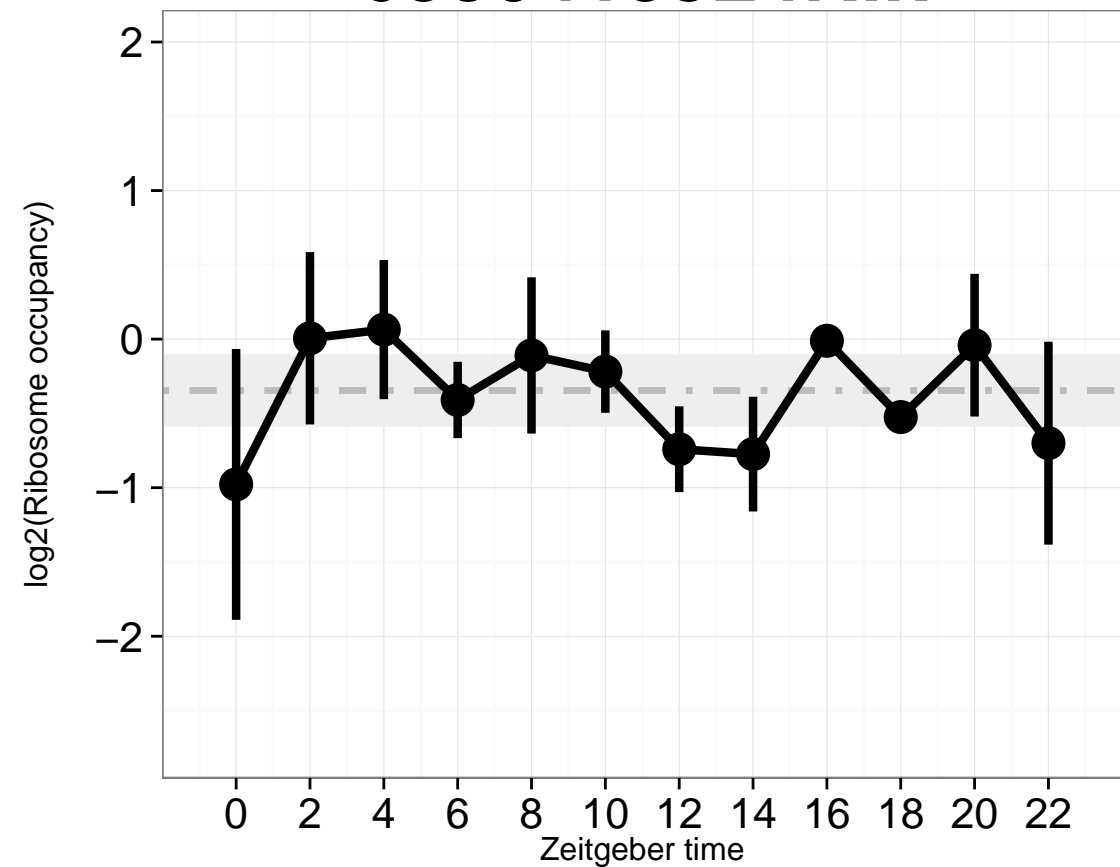

Supplement: Supplementary file 6 — Transcriptome-wide kidney RPF (blue) and RNA (orange) levels in the left panels (with “error bars” connecting the two replicates of each timepoint) and TE in the right panels. (ZIP 116896 kb) [file 13059_2017_1222_MOESM6_ESM.zip › Supp_Dataset_S1/A_RNA_non_rhythmic_RPF_non_rhythmic/6330419J24Rik_kidney_set_A.pdf]
