# Supplementary figures and images for: Translational contributions to tissue specificity in rhythmic and constitutive gene expression (part 1 of 4)
Source: Genome Biol. 2017 Jun 16;18:116. doi: 10.1186/s13059-017-1222-2 (PMC5473967; doi:10.1186/s13059-017-1222-2)

**0610007P14Rik**

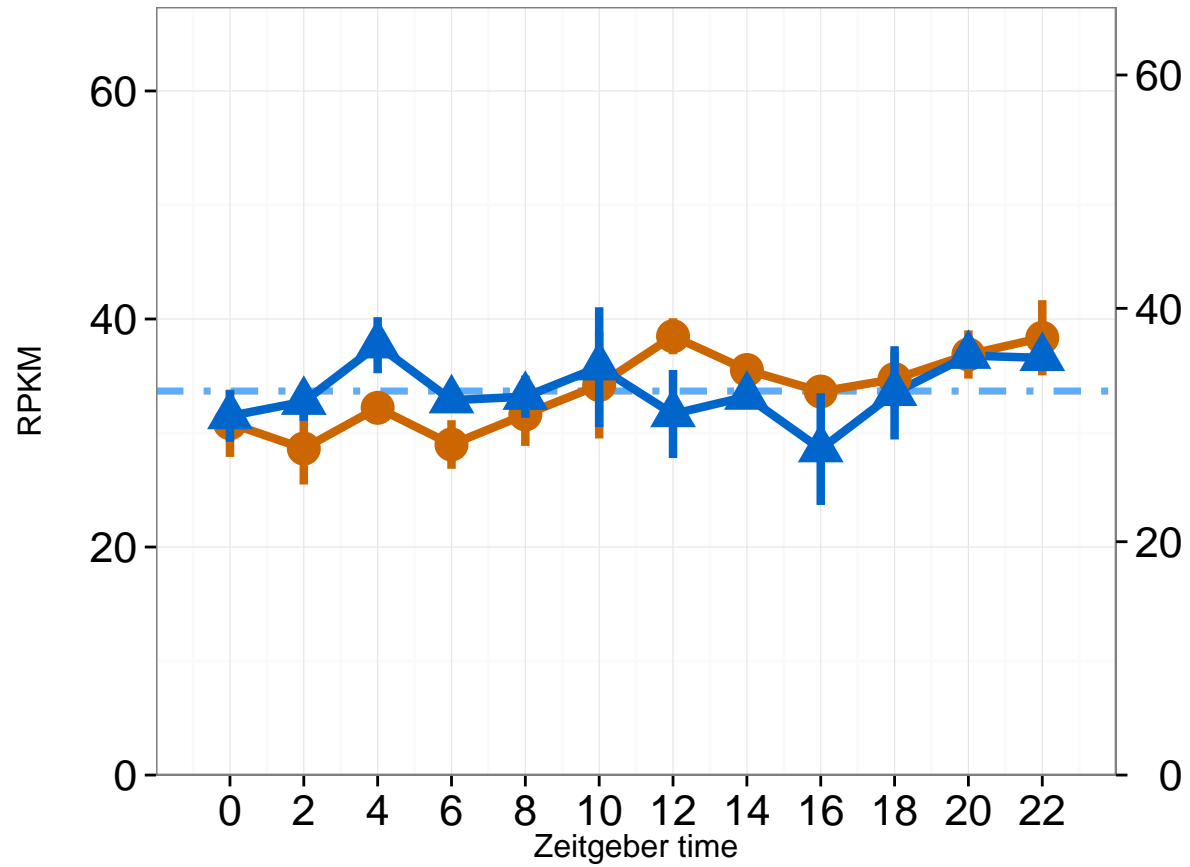

**0610007P14Rik**

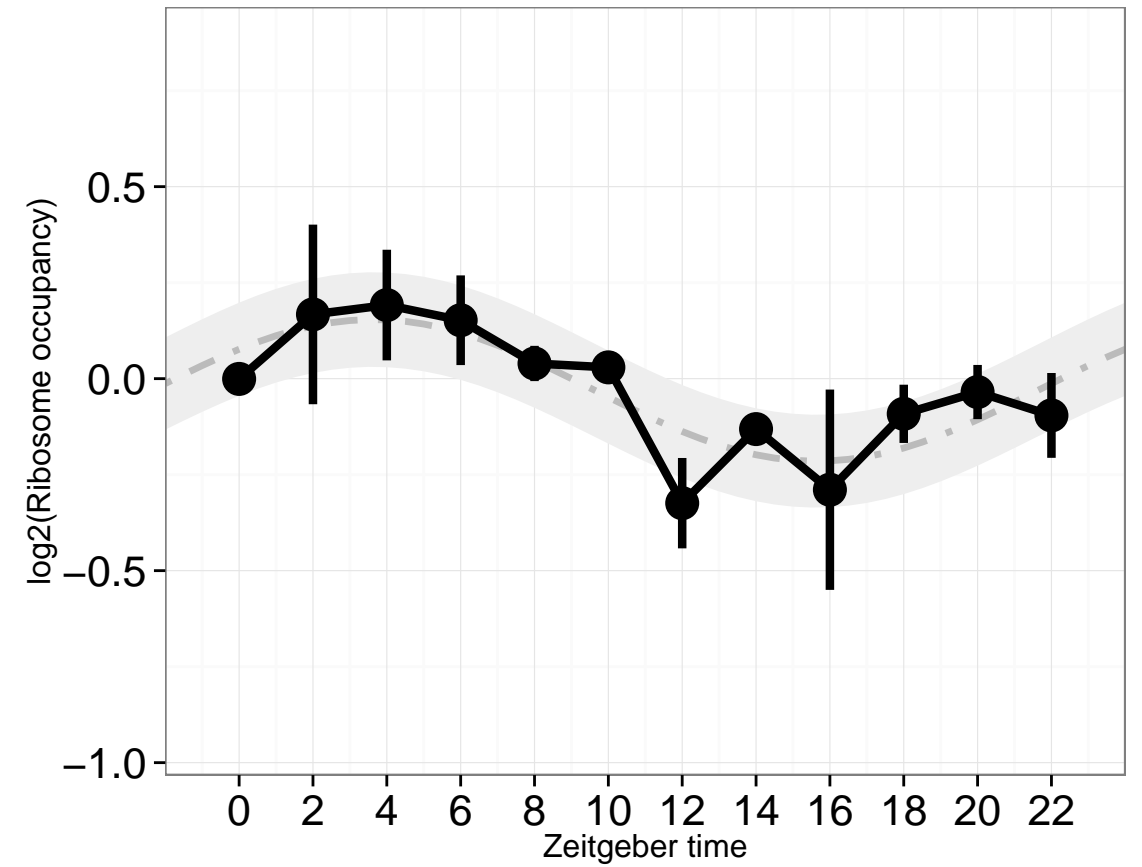

Supplement: Supplementary file 6 — Transcriptome-wide kidney RPF (blue) and RNA (orange) levels in the left panels (with “error bars” connecting the two replicates of each timepoint) and TE in the right panels. (ZIP 116896 kb) [file 13059_2017_1222_MOESM6_ESM.zip › Supp_Dataset_S1/A_RNA_non_rhythmic_RPF_non_rhythmic/0610007P14Rik_kidney_set_A.pdf]

## 0610009D07Rik

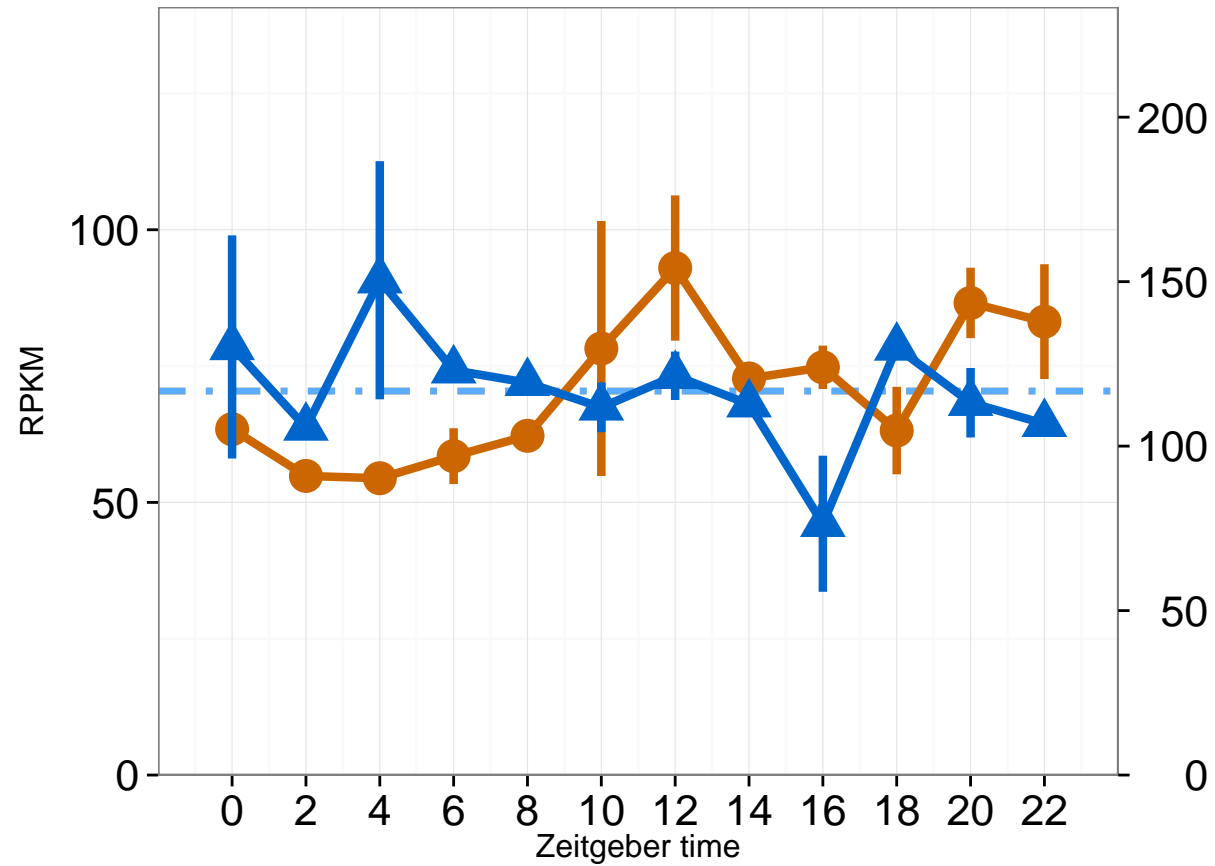

## 0610009D07Rik

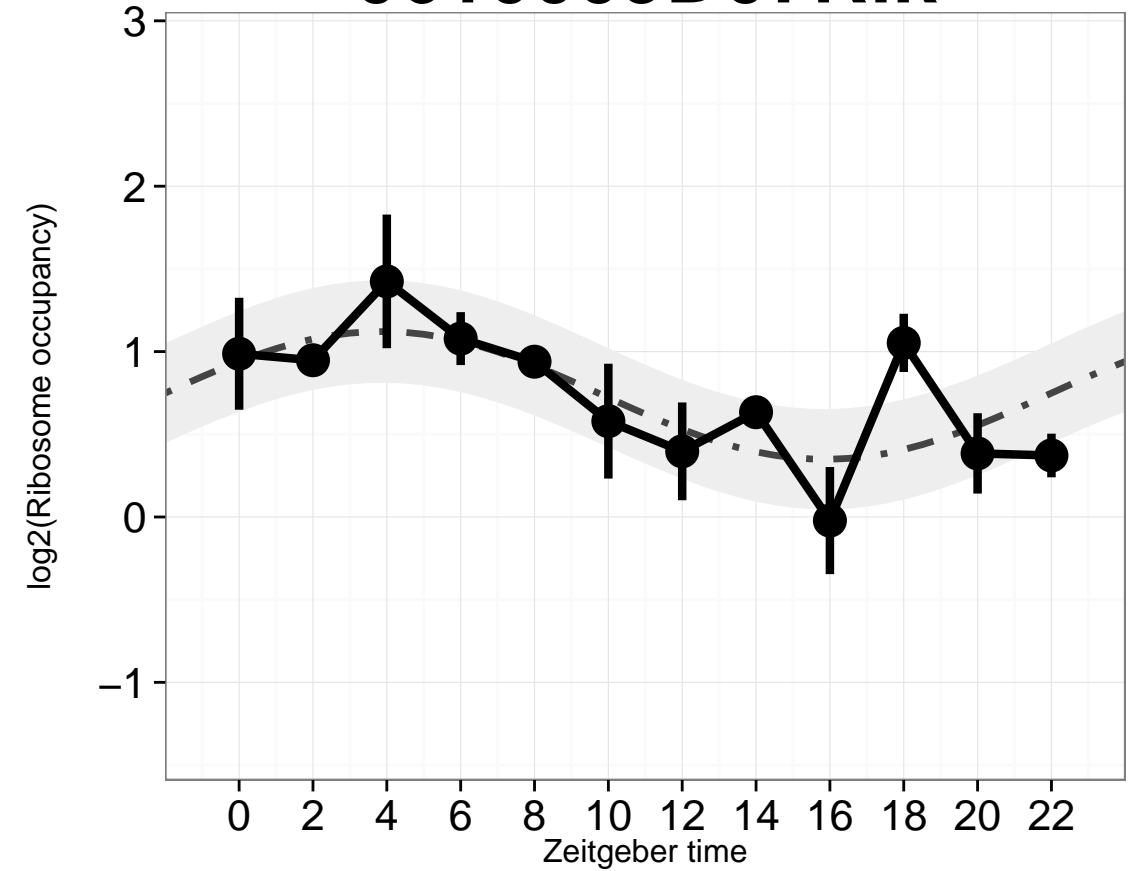

Supplement: Supplementary file 6 — Transcriptome-wide kidney RPF (blue) and RNA (orange) levels in the left panels (with “error bars” connecting the two replicates of each timepoint) and TE in the right panels. (ZIP 116896 kb) [file 13059_2017_1222_MOESM6_ESM.zip › Supp_Dataset_S1/A_RNA_non_rhythmic_RPF_non_rhythmic/0610009D07Rik_kidney_set_A.pdf]

**0610009L18Rik**

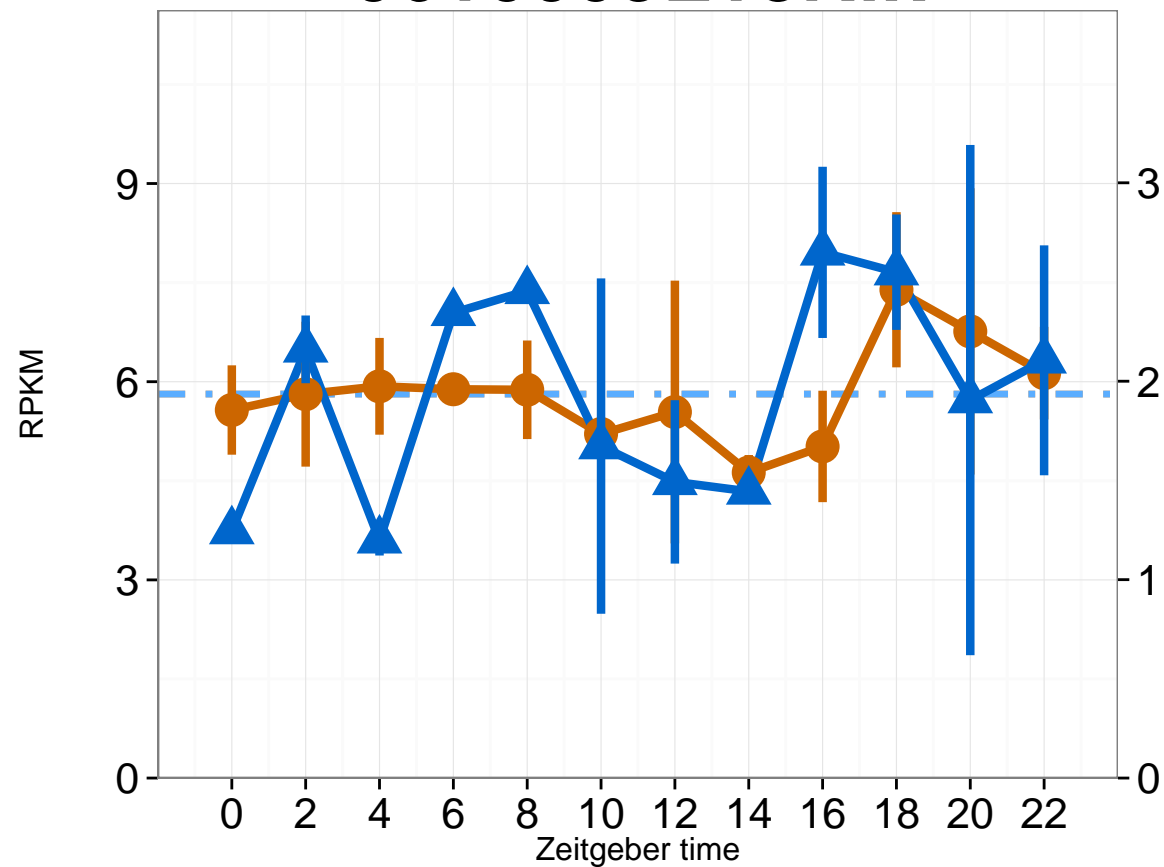

**0610009L18Rik**

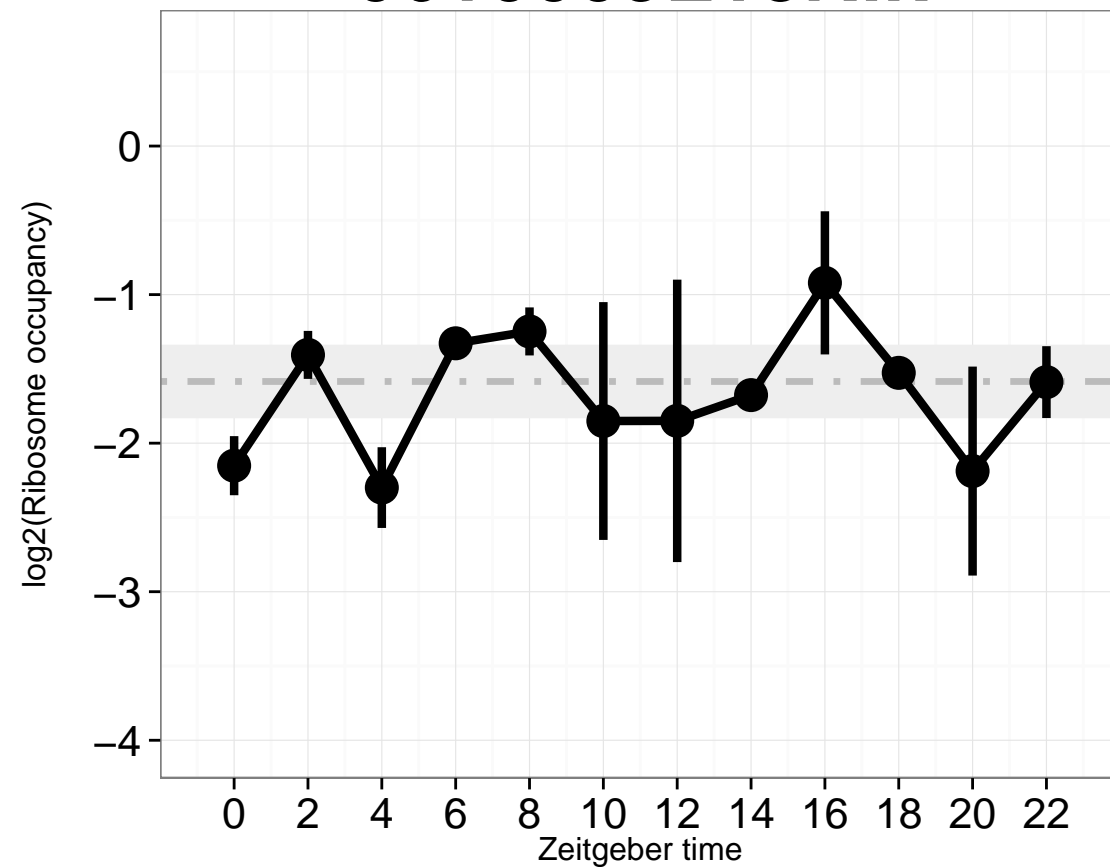

Supplement: Supplementary file 6 — Transcriptome-wide kidney RPF (blue) and RNA (orange) levels in the left panels (with “error bars” connecting the two replicates of each timepoint) and TE in the right panels. (ZIP 116896 kb) [file 13059_2017_1222_MOESM6_ESM.zip › Supp_Dataset_S1/A_RNA_non_rhythmic_RPF_non_rhythmic/0610009L18Rik_kidney_set_A.pdf]

## 0610009O20Rik

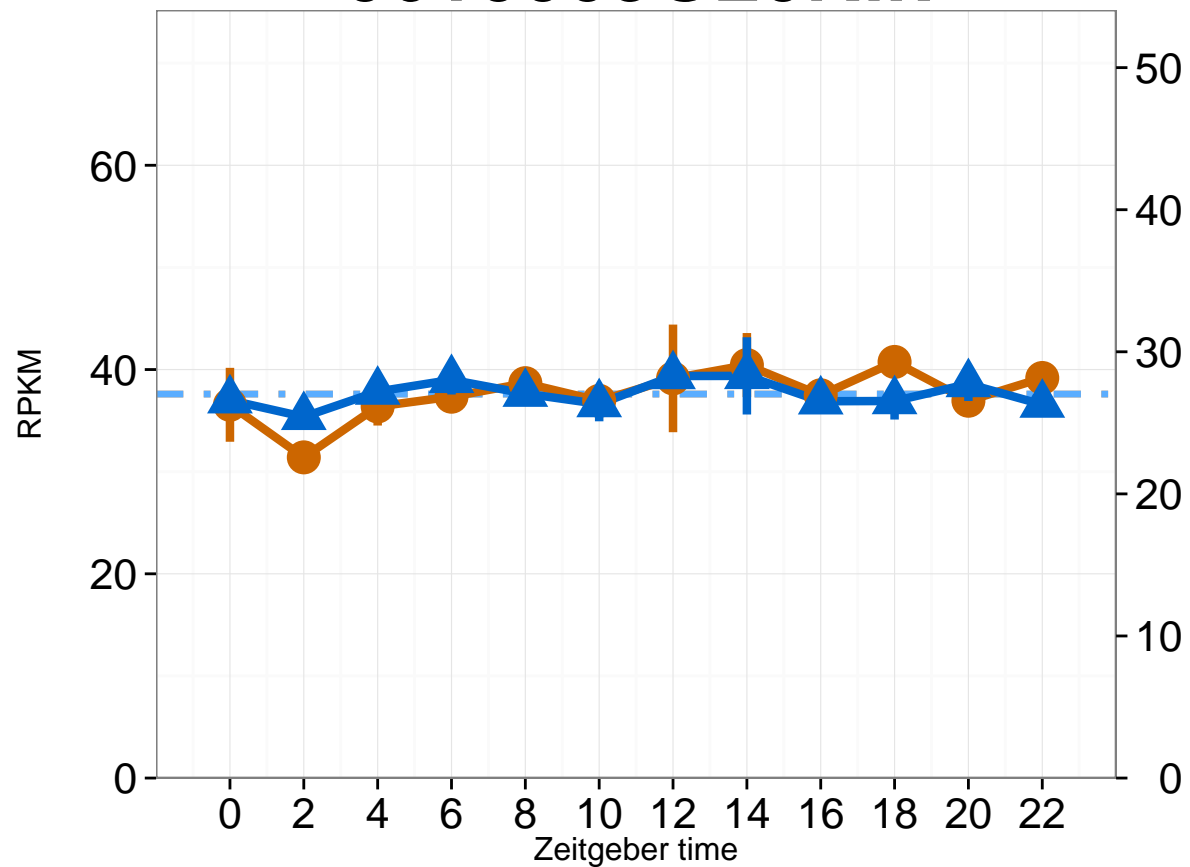

## 0610009O20Rik

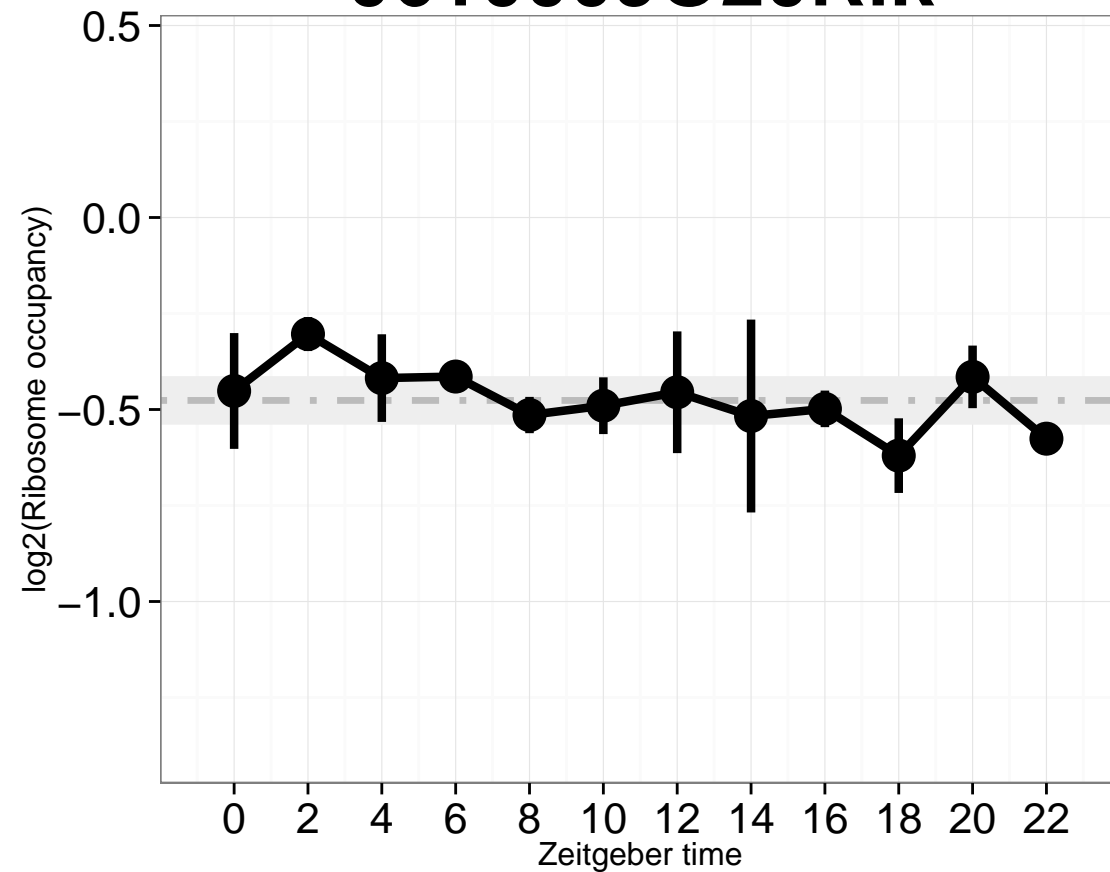

Supplement: Supplementary file 6 — Transcriptome-wide kidney RPF (blue) and RNA (orange) levels in the left panels (with “error bars” connecting the two replicates of each timepoint) and TE in the right panels. (ZIP 116896 kb) [file 13059_2017_1222_MOESM6_ESM.zip › Supp_Dataset_S1/A_RNA_non_rhythmic_RPF_non_rhythmic/0610009O20Rik_kidney_set_A.pdf]

## 0610010F05Rik

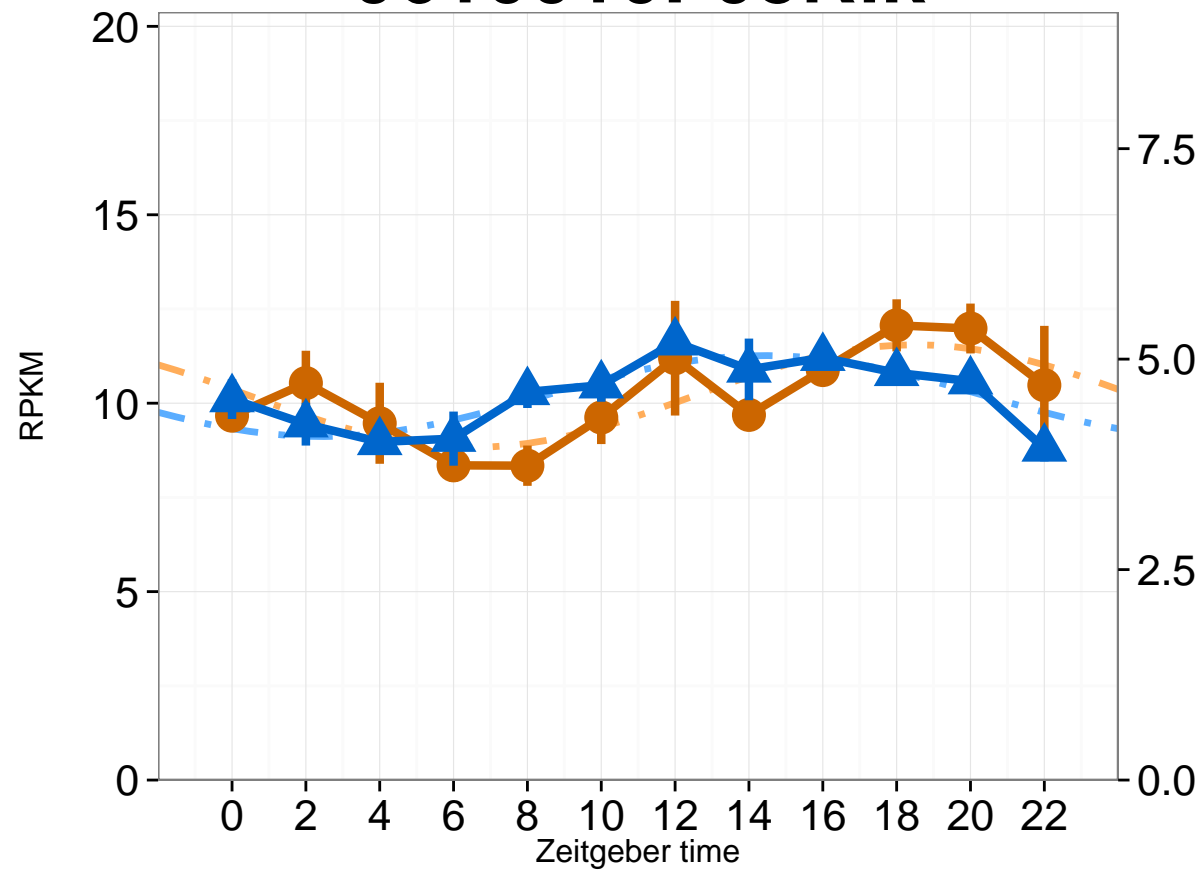

## 0610010F05Rik

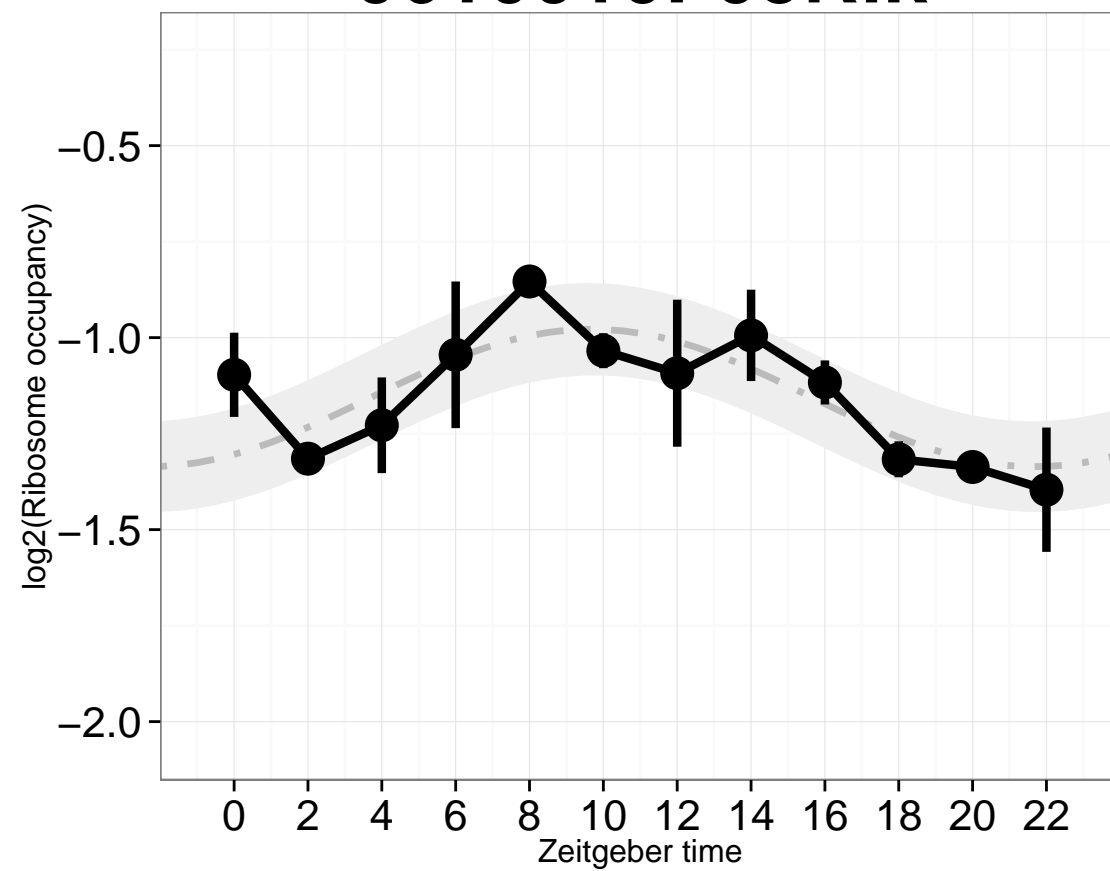

Supplement: Supplementary file 6 — Transcriptome-wide kidney RPF (blue) and RNA (orange) levels in the left panels (with “error bars” connecting the two replicates of each timepoint) and TE in the right panels. (ZIP 116896 kb) [file 13059_2017_1222_MOESM6_ESM.zip › Supp_Dataset_S1/A_RNA_non_rhythmic_RPF_non_rhythmic/0610010F05Rik_kidney_set_A.pdf]

**0610010K14Rik TR**

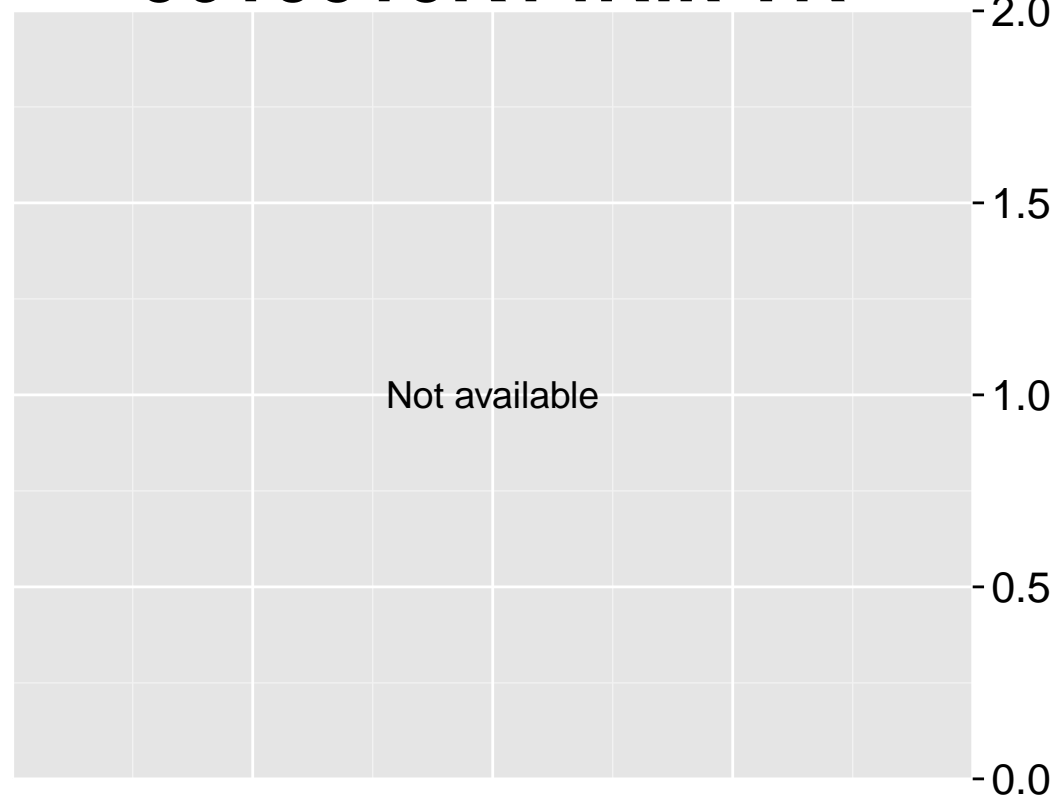

**0610010K14Rik log<sub>2</sub>(Ribosome occup**

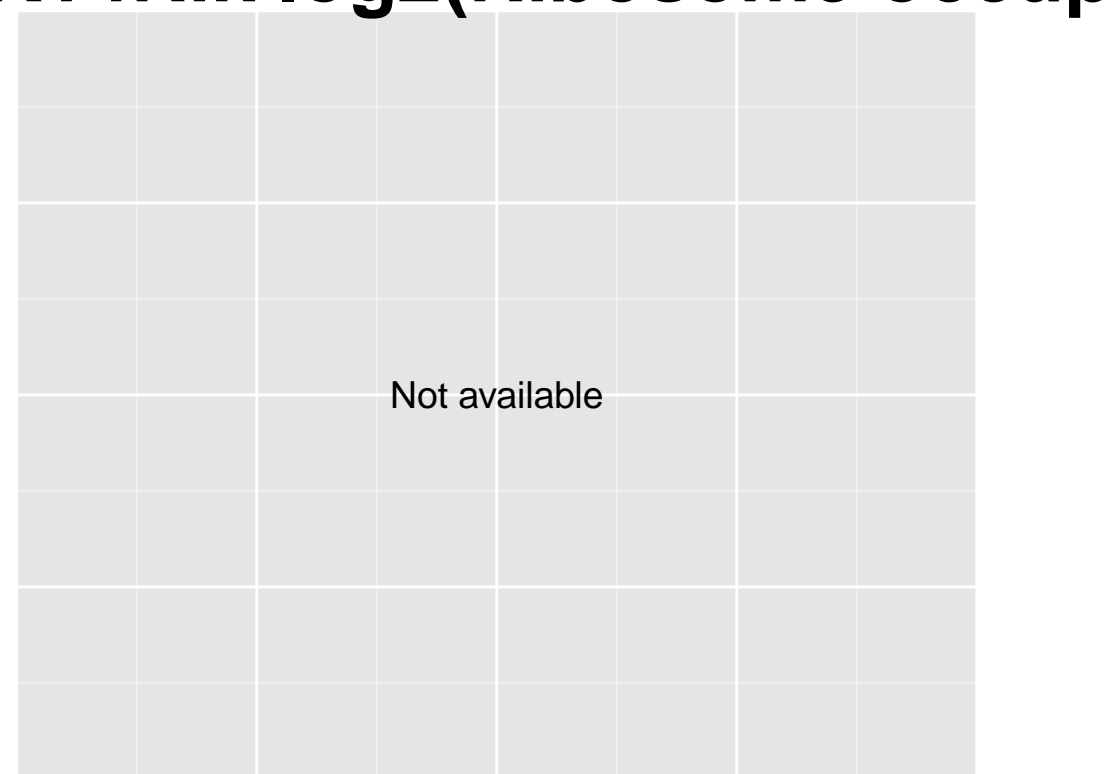

Supplement: Supplementary file 6 — Transcriptome-wide kidney RPF (blue) and RNA (orange) levels in the left panels (with “error bars” connecting the two replicates of each timepoint) and TE in the right panels. (ZIP 116896 kb) [file 13059_2017_1222_MOESM6_ESM.zip › Supp_Dataset_S1/A_RNA_non_rhythmic_RPF_non_rhythmic/0610010K14Rik_kidney_set_A.pdf]

# 0610011F06Rik

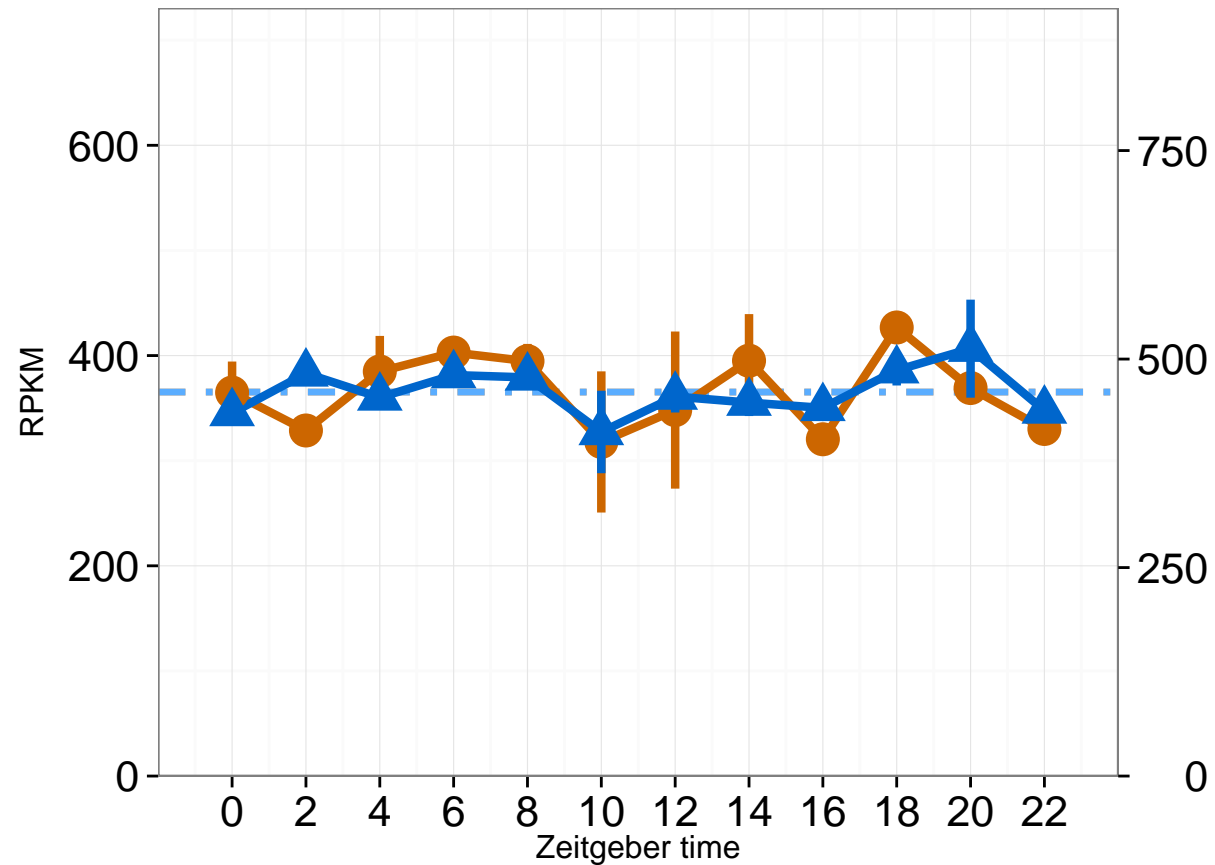

# 0610011F06Rik

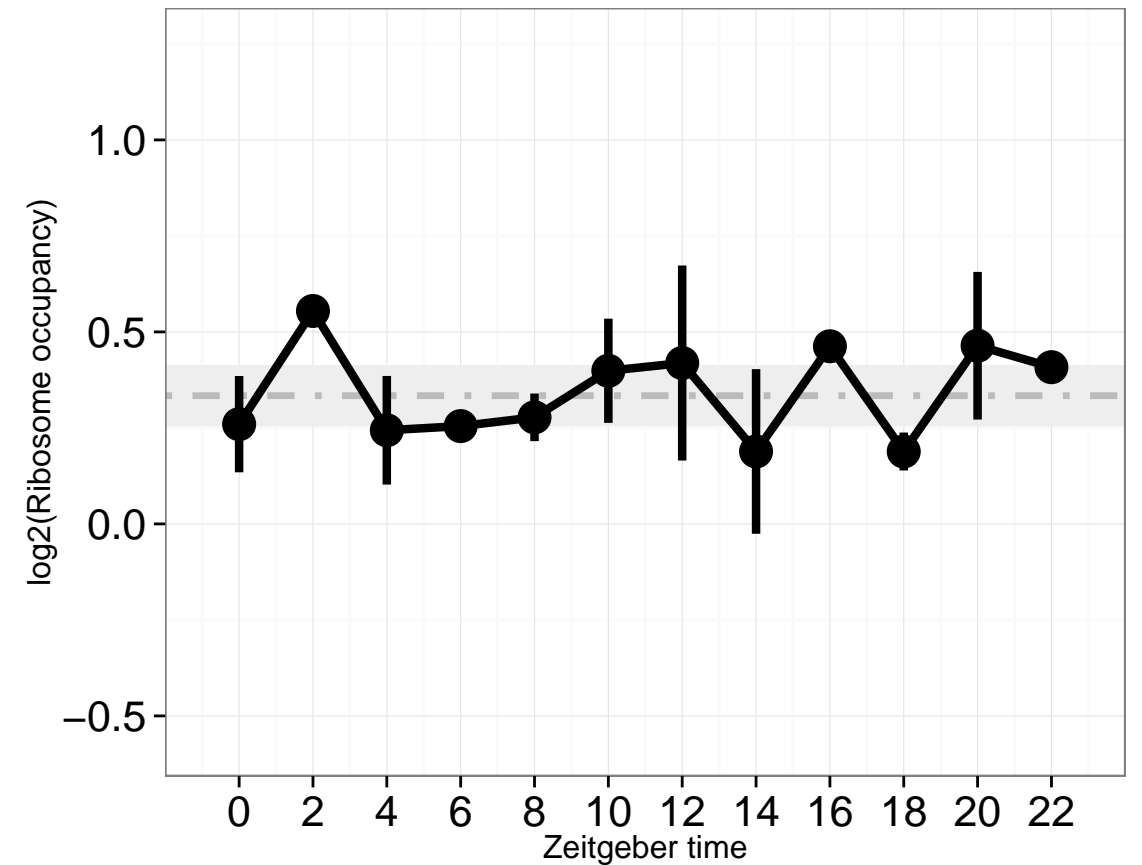

Supplement: Supplementary file 6 — Transcriptome-wide kidney RPF (blue) and RNA (orange) levels in the left panels (with “error bars” connecting the two replicates of each timepoint) and TE in the right panels. (ZIP 116896 kb) [file 13059_2017_1222_MOESM6_ESM.zip › Supp_Dataset_S1/A_RNA_non_rhythmic_RPF_non_rhythmic/0610011F06Rik_kidney_set_A.pdf]

## 0610012H03Rik

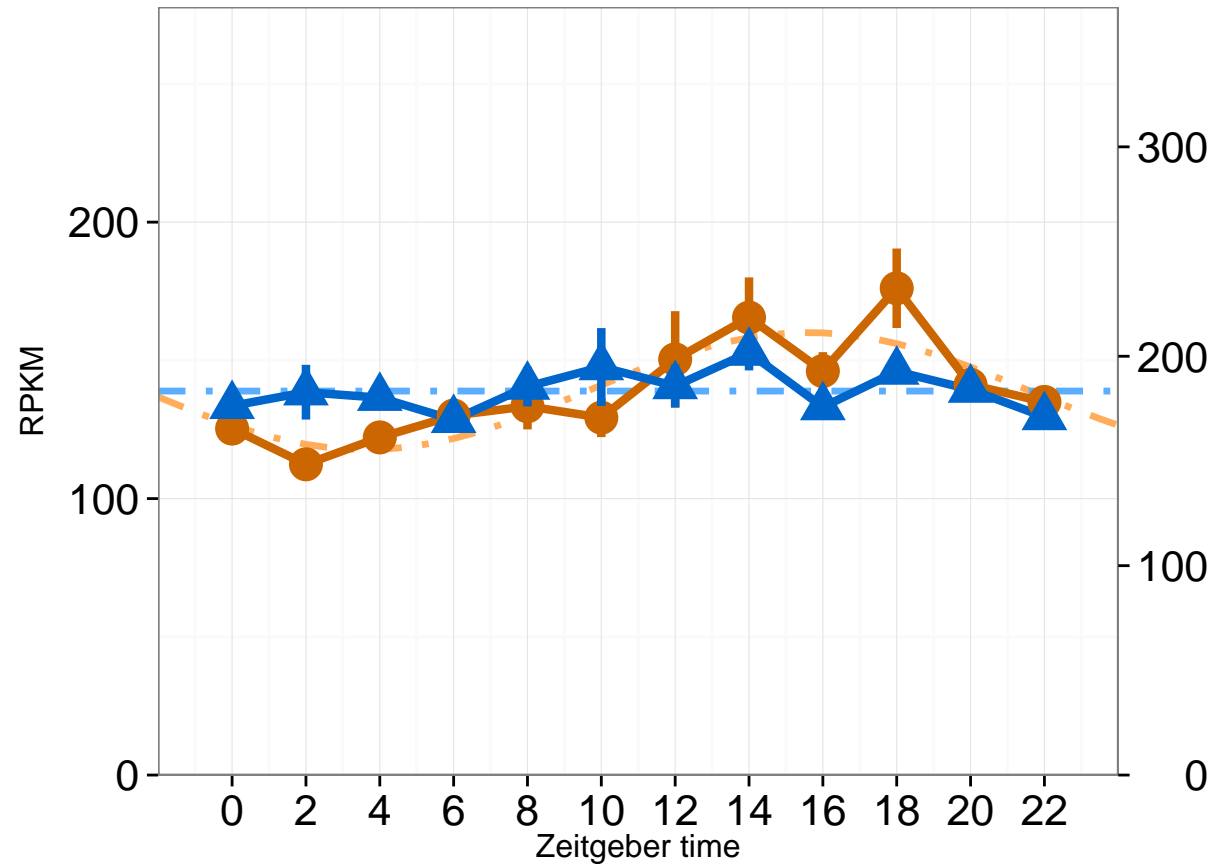

## 0610012H03Rik

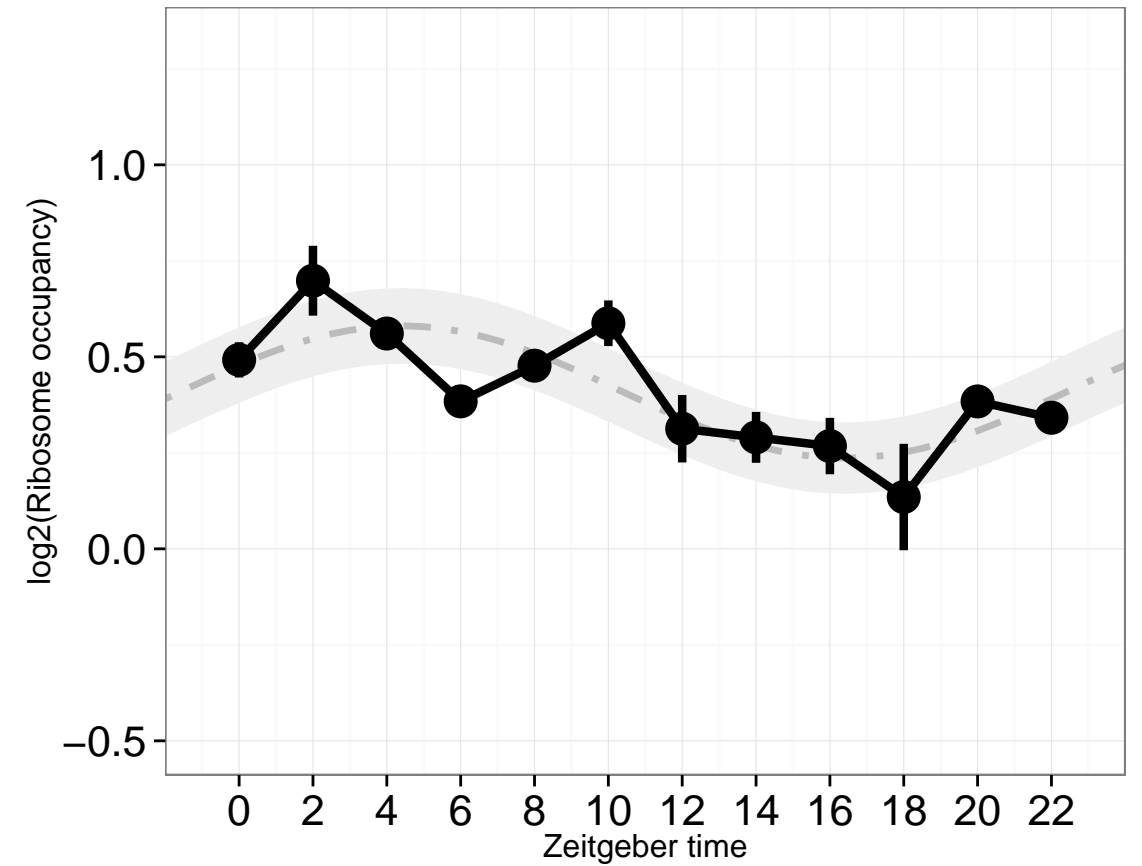

Supplement: Supplementary file 6 — Transcriptome-wide kidney RPF (blue) and RNA (orange) levels in the left panels (with “error bars” connecting the two replicates of each timepoint) and TE in the right panels. (ZIP 116896 kb) [file 13059_2017_1222_MOESM6_ESM.zip › Supp_Dataset_S1/A_RNA_non_rhythmic_RPF_non_rhythmic/0610012H03Rik_kidney_set_A.pdf]

# 0610030E20Rik

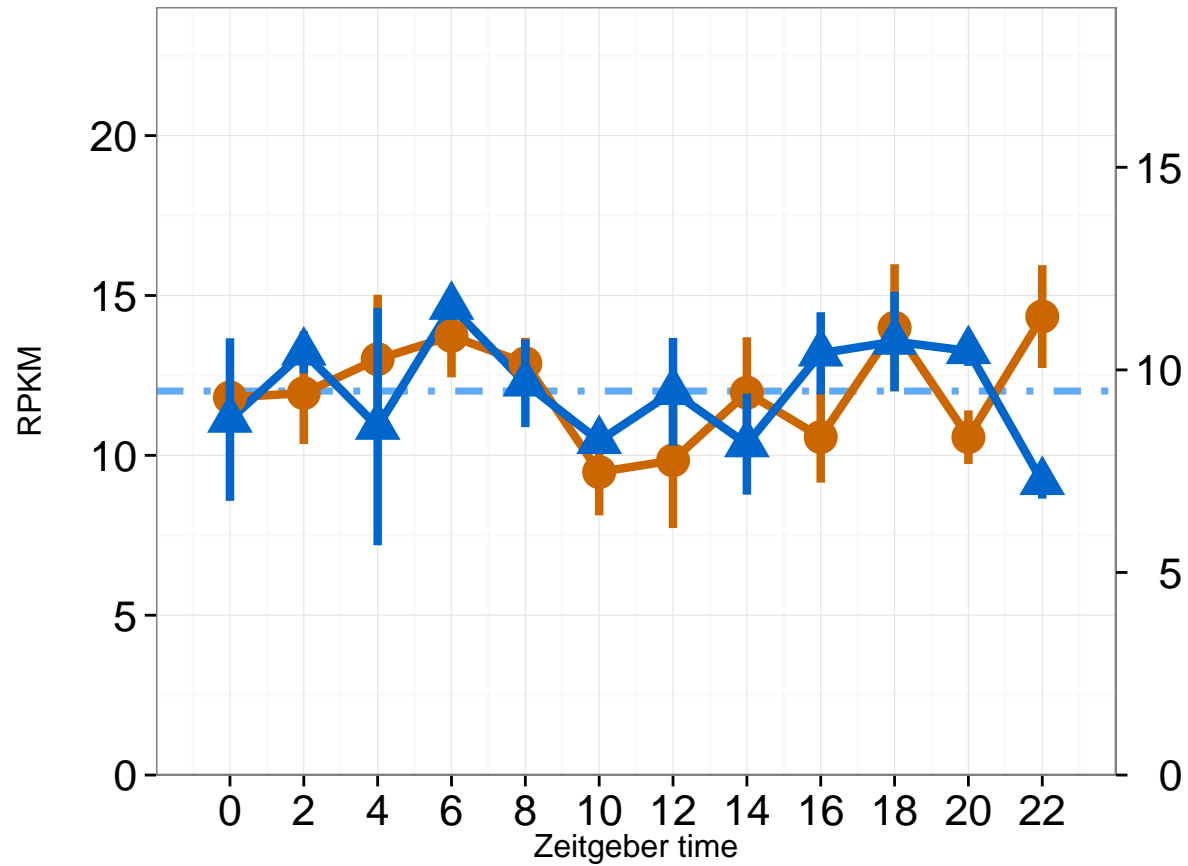

# 0610030E20Rik

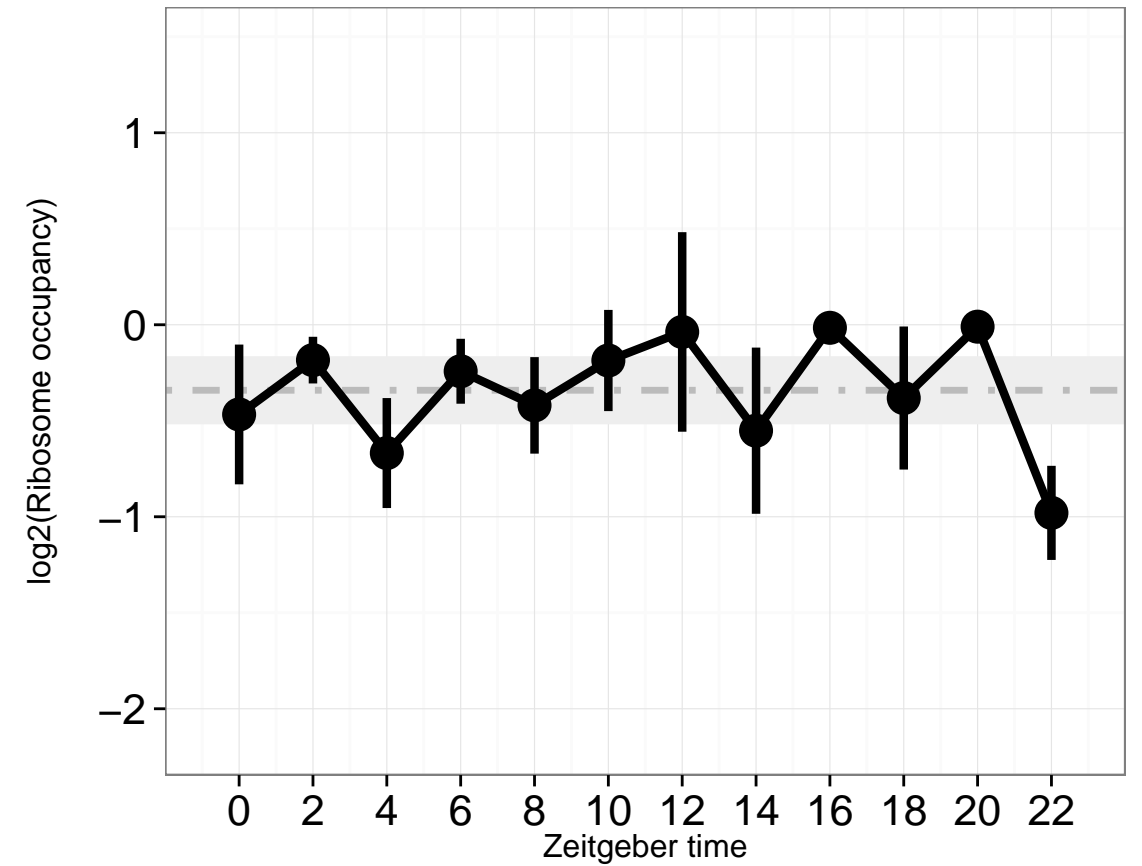

Supplement: Supplementary file 6 — Transcriptome-wide kidney RPF (blue) and RNA (orange) levels in the left panels (with “error bars” connecting the two replicates of each timepoint) and TE in the right panels. (ZIP 116896 kb) [file 13059_2017_1222_MOESM6_ESM.zip › Supp_Dataset_S1/A_RNA_non_rhythmic_RPF_non_rhythmic/0610030E20Rik_kidney_set_A.pdf]

# 0610031J06Rik

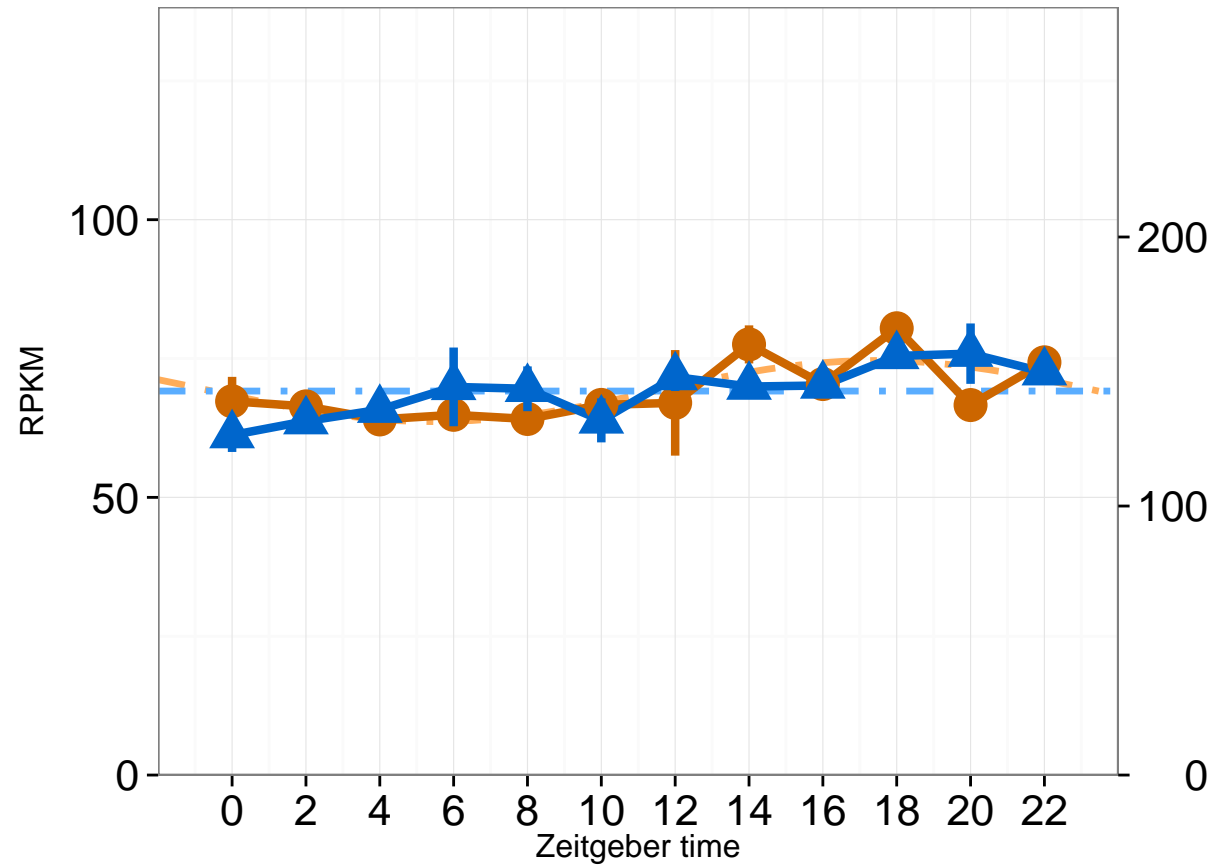

# 0610031J06Rik

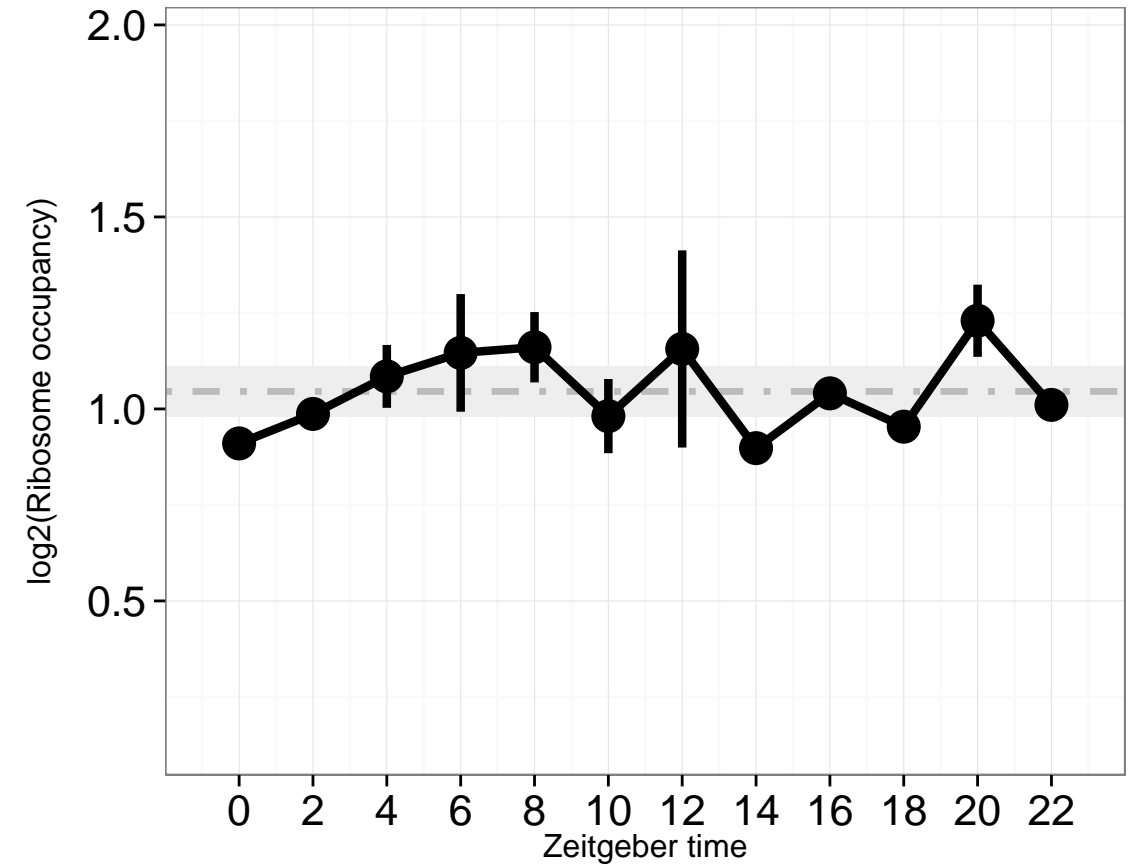

Supplement: Supplementary file 6 — Transcriptome-wide kidney RPF (blue) and RNA (orange) levels in the left panels (with “error bars” connecting the two replicates of each timepoint) and TE in the right panels. (ZIP 116896 kb) [file 13059_2017_1222_MOESM6_ESM.zip › Supp_Dataset_S1/A_RNA_non_rhythmic_RPF_non_rhythmic/0610031J06Rik_kidney_set_A.pdf]

**0610037L13Rik**

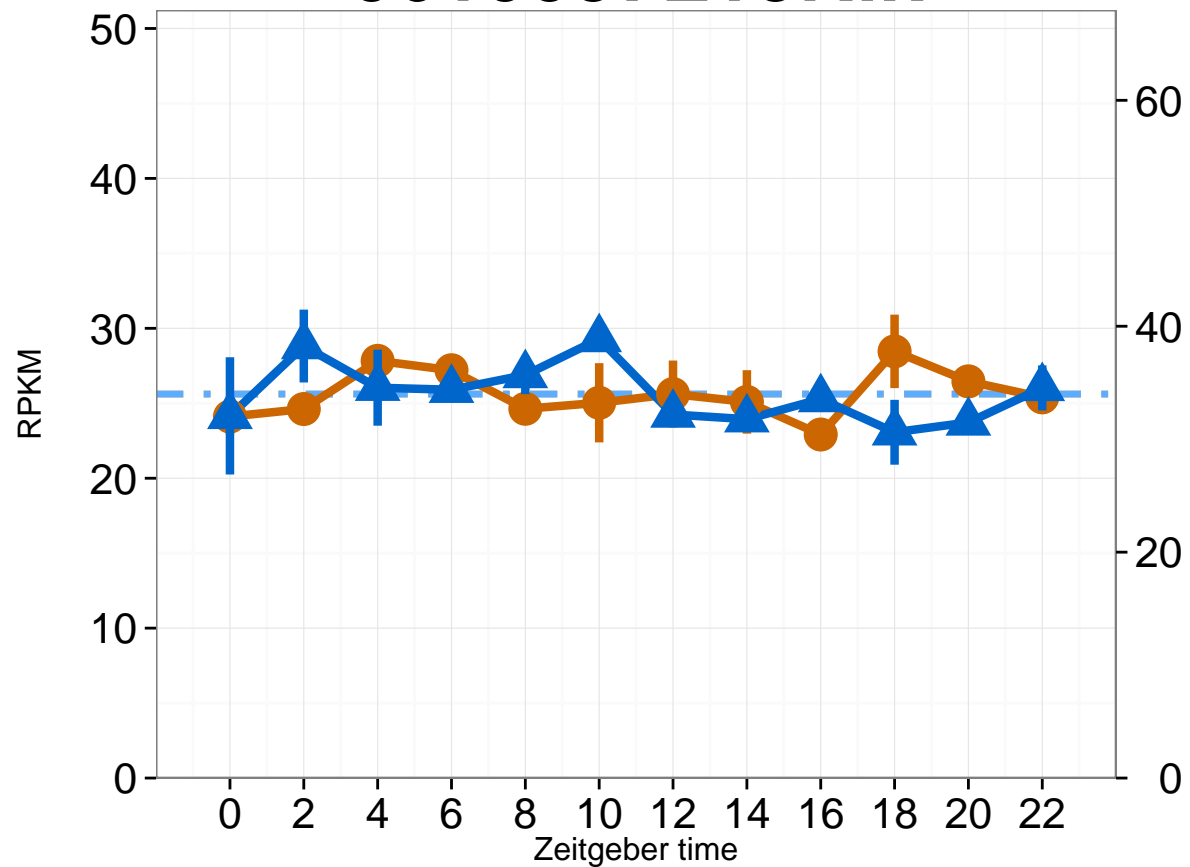

**0610037L13Rik**

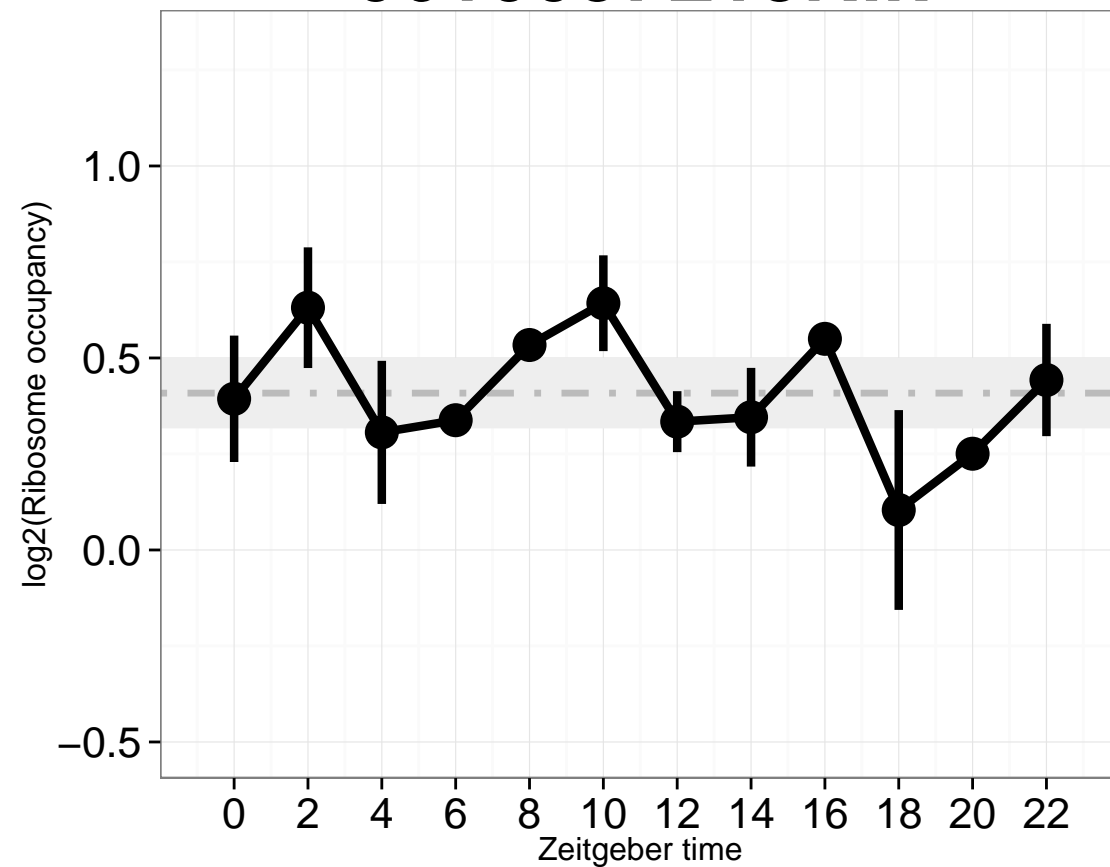

Supplement: Supplementary file 6 — Transcriptome-wide kidney RPF (blue) and RNA (orange) levels in the left panels (with “error bars” connecting the two replicates of each timepoint) and TE in the right panels. (ZIP 116896 kb) [file 13059_2017_1222_MOESM6_ESM.zip › Supp_Dataset_S1/A_RNA_non_rhythmic_RPF_non_rhythmic/0610037L13Rik_kidney_set_A.pdf]

# 0610040J01Rik

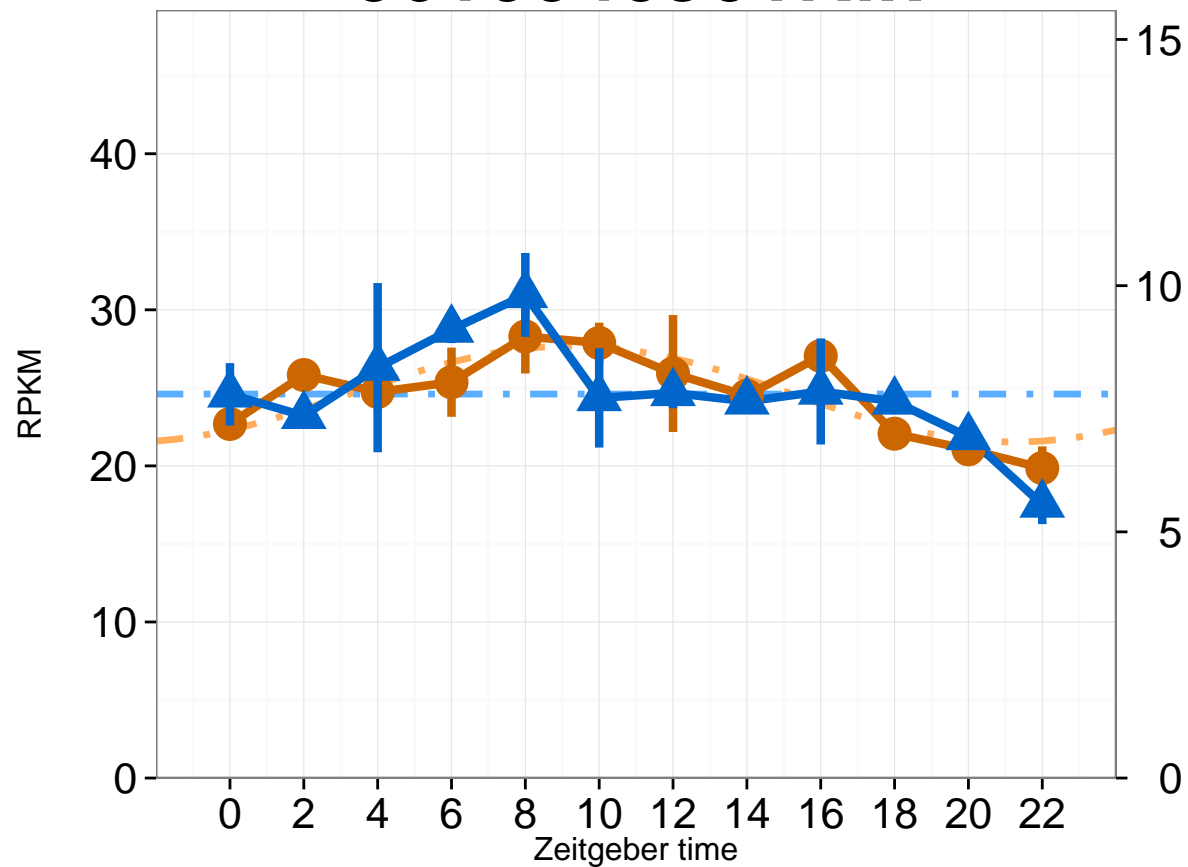

# 0610040J01Rik

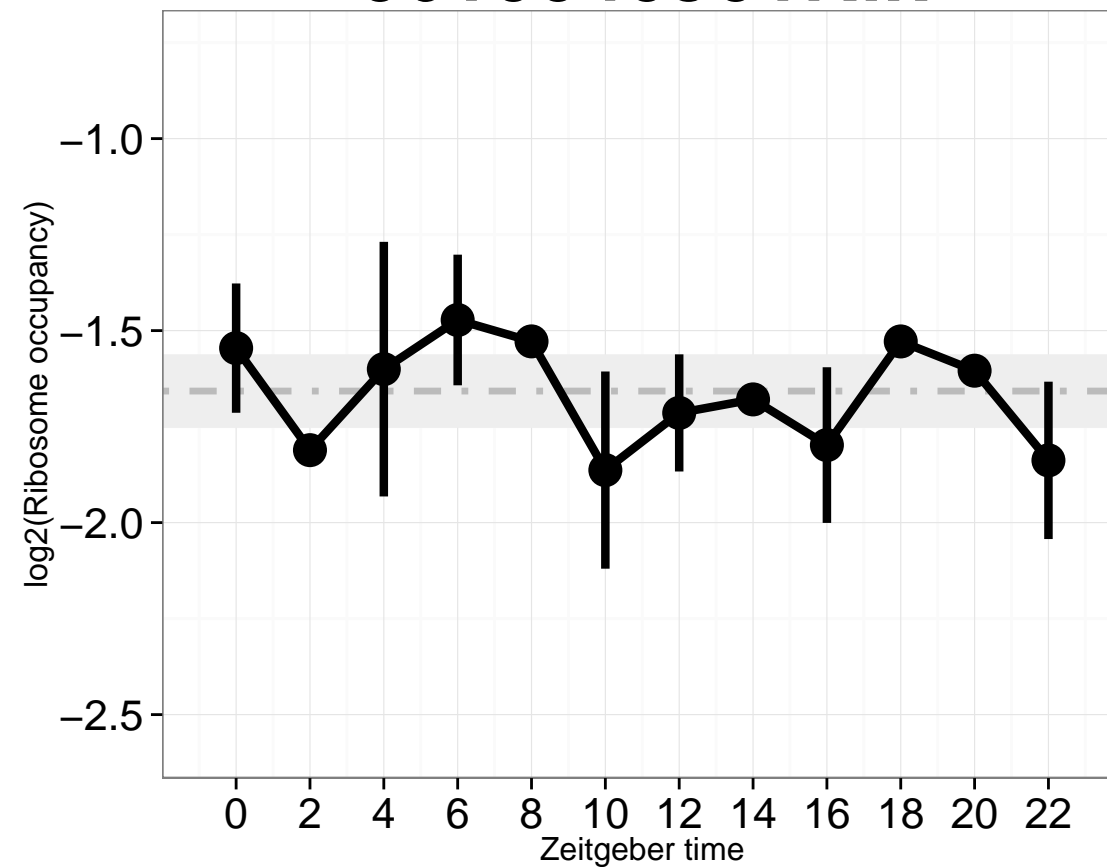

Supplement: Supplementary file 6 — Transcriptome-wide kidney RPF (blue) and RNA (orange) levels in the left panels (with “error bars” connecting the two replicates of each timepoint) and TE in the right panels. (ZIP 116896 kb) [file 13059_2017_1222_MOESM6_ESM.zip › Supp_Dataset_S1/A_RNA_non_rhythmic_RPF_non_rhythmic/0610040J01Rik_kidney_set_A.pdf]

# 1110001A16Rik

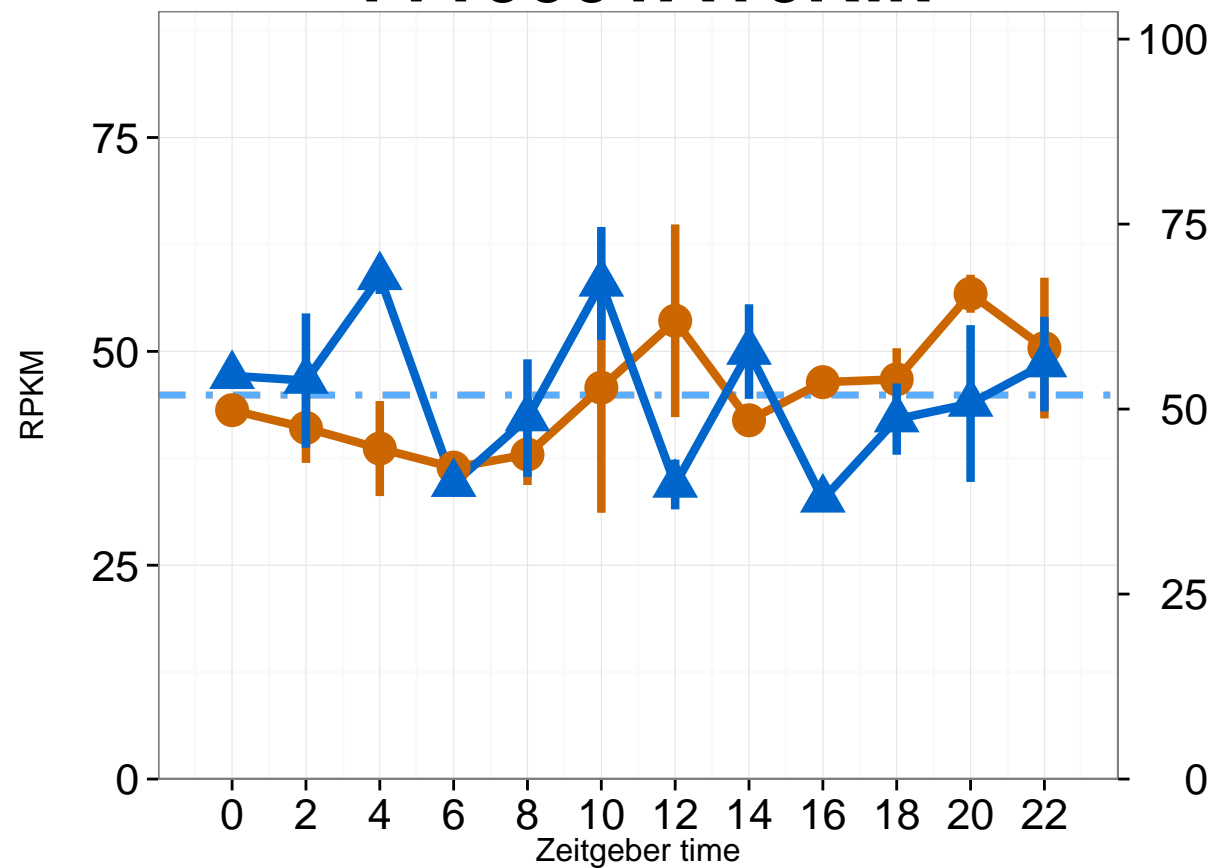

# 1110001A16Rik

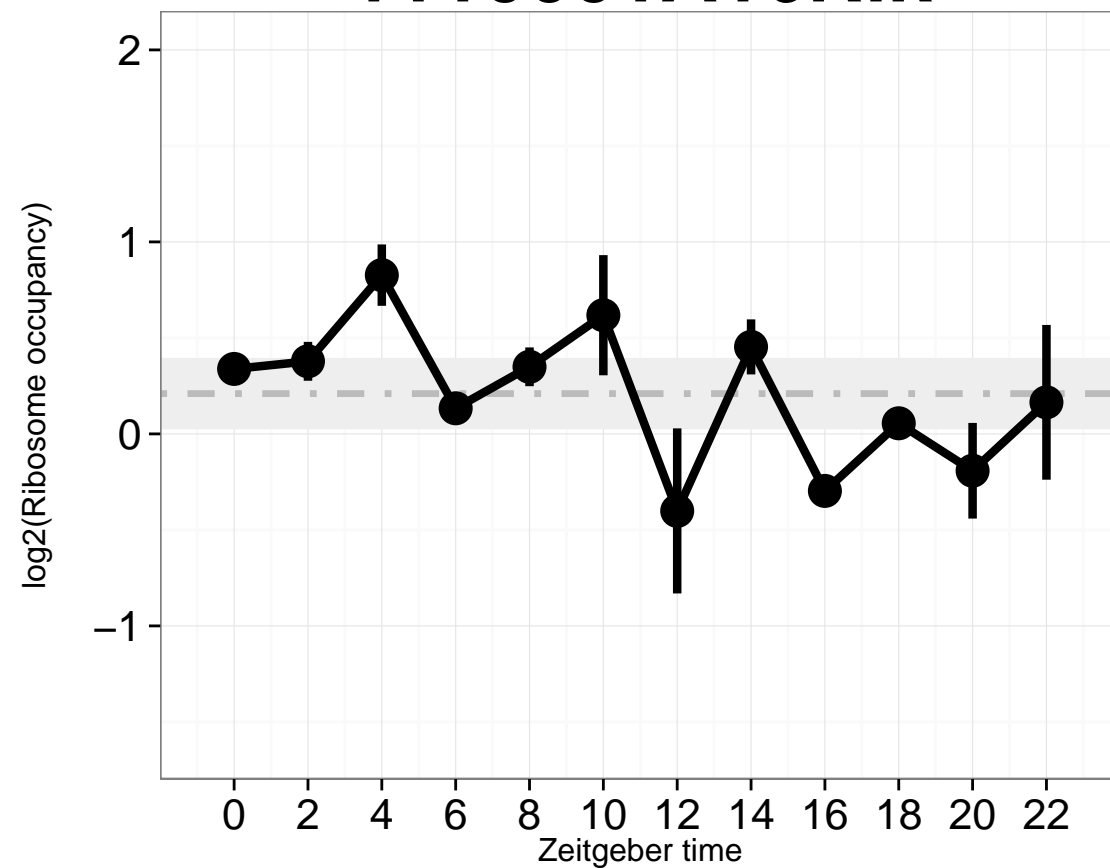

Supplement: Supplementary file 6 — Transcriptome-wide kidney RPF (blue) and RNA (orange) levels in the left panels (with “error bars” connecting the two replicates of each timepoint) and TE in the right panels. (ZIP 116896 kb) [file 13059_2017_1222_MOESM6_ESM.zip › Supp_Dataset_S1/A_RNA_non_rhythmic_RPF_non_rhythmic/1110001A16Rik_kidney_set_A.pdf]

# 1110002L01Rik

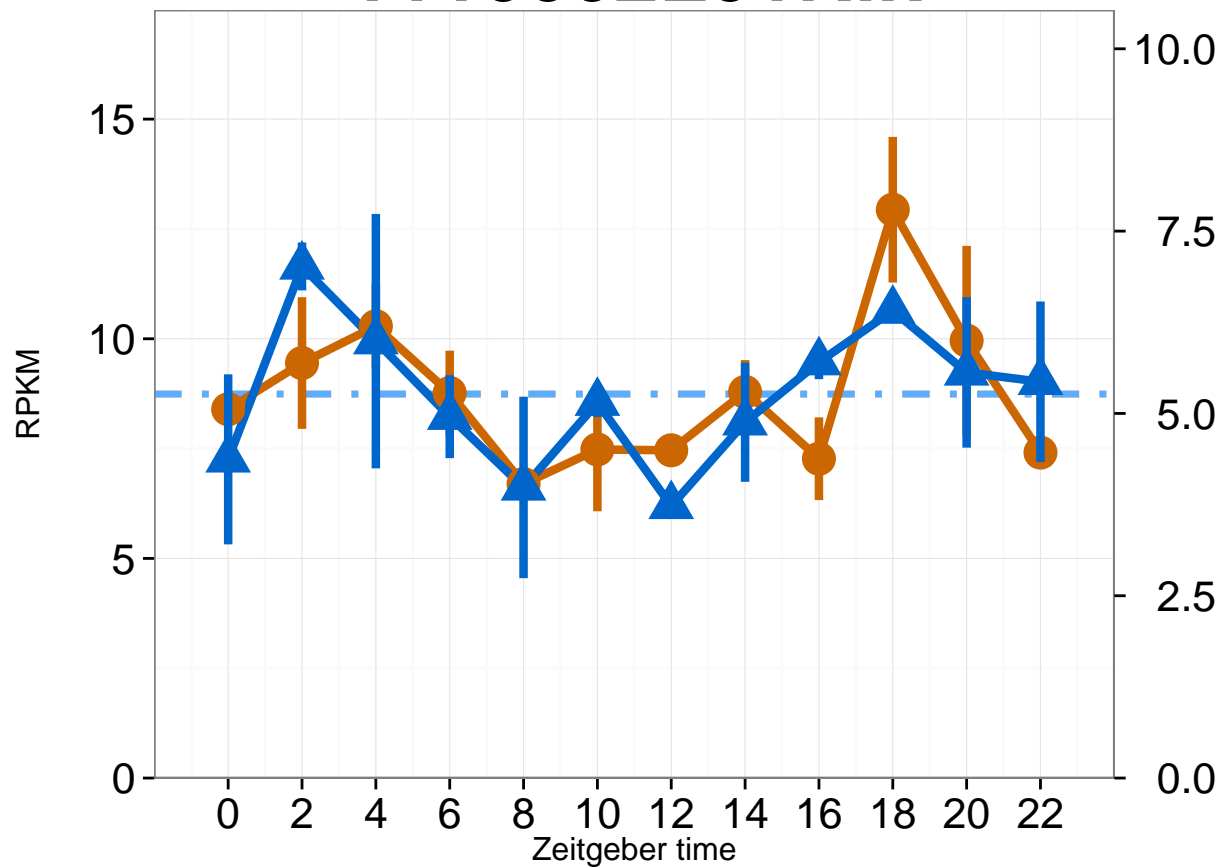

# 1110002L01Rik

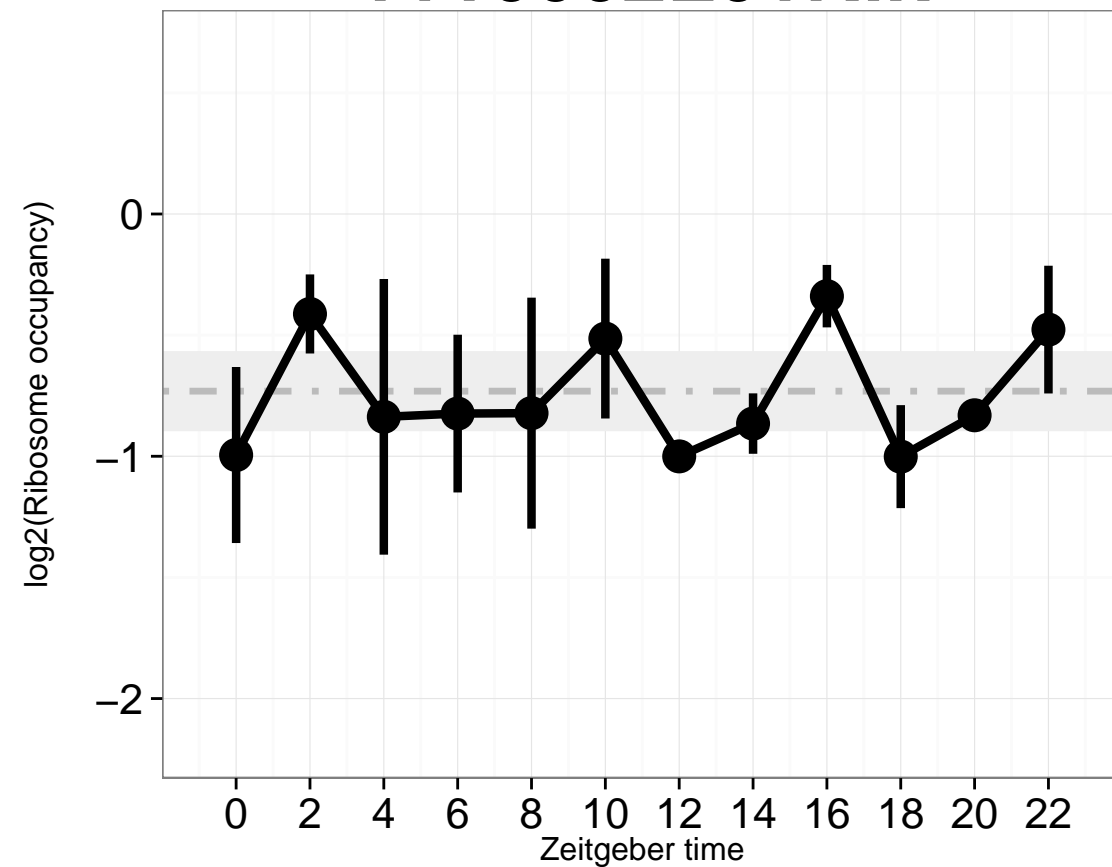

Supplement: Supplementary file 6 — Transcriptome-wide kidney RPF (blue) and RNA (orange) levels in the left panels (with “error bars” connecting the two replicates of each timepoint) and TE in the right panels. (ZIP 116896 kb) [file 13059_2017_1222_MOESM6_ESM.zip › Supp_Dataset_S1/A_RNA_non_rhythmic_RPF_non_rhythmic/1110002L01Rik_kidney_set_A.pdf]

# 1110004E09Rik

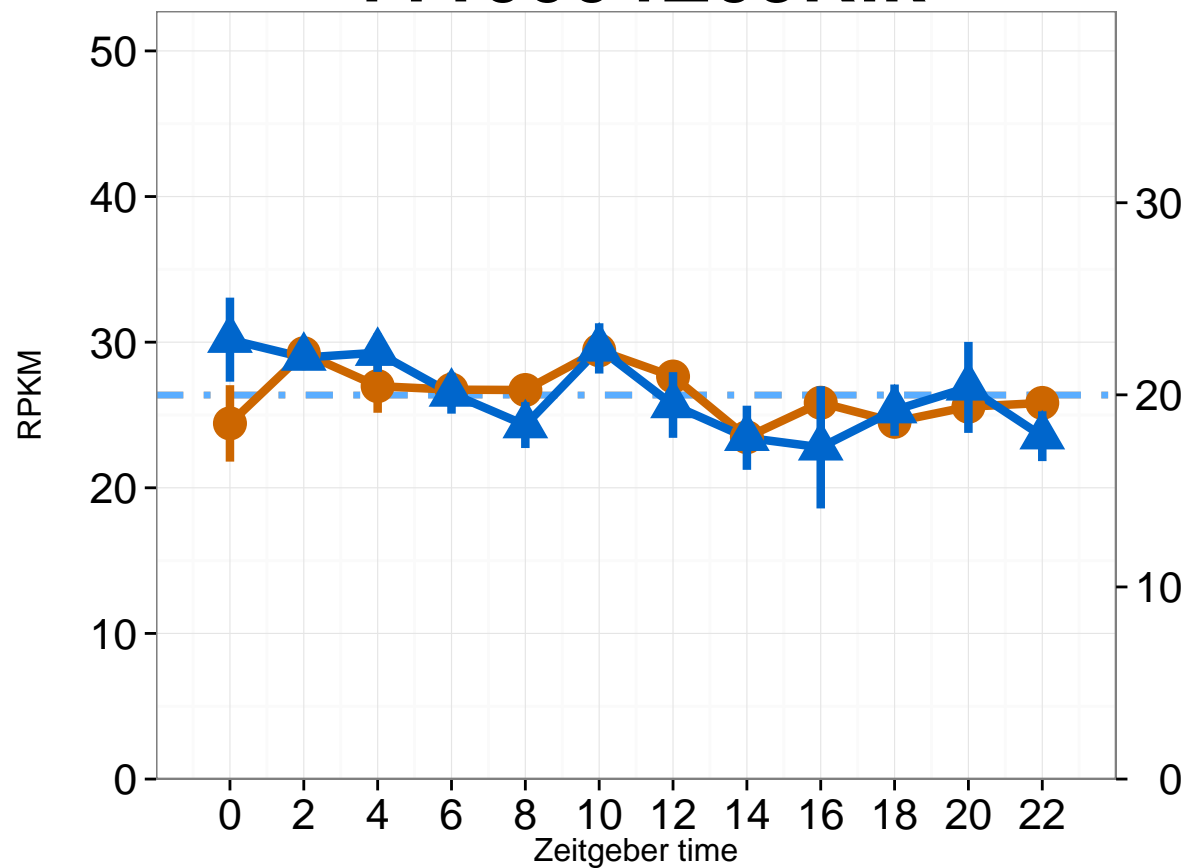

# 1110004E09Rik

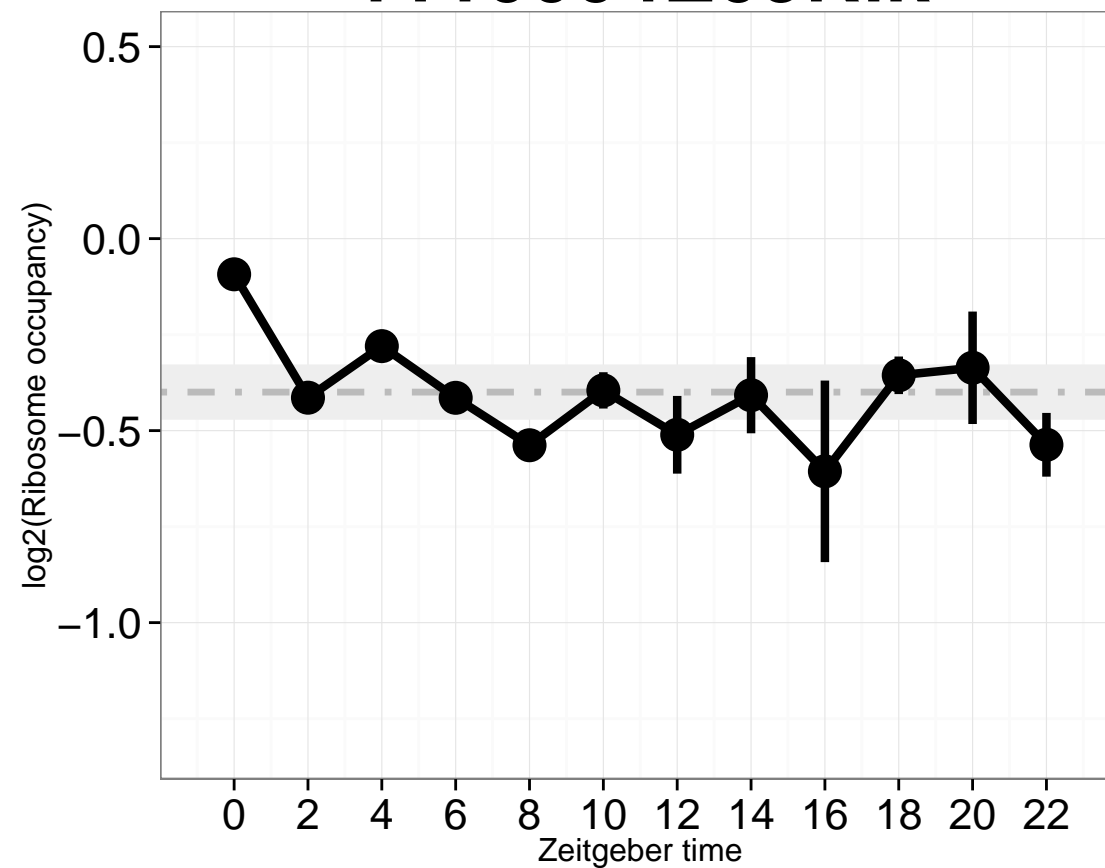

Supplement: Supplementary file 6 — Transcriptome-wide kidney RPF (blue) and RNA (orange) levels in the left panels (with “error bars” connecting the two replicates of each timepoint) and TE in the right panels. (ZIP 116896 kb) [file 13059_2017_1222_MOESM6_ESM.zip › Supp_Dataset_S1/A_RNA_non_rhythmic_RPF_non_rhythmic/1110004E09Rik_kidney_set_A.pdf]

# 1110004F10Rik

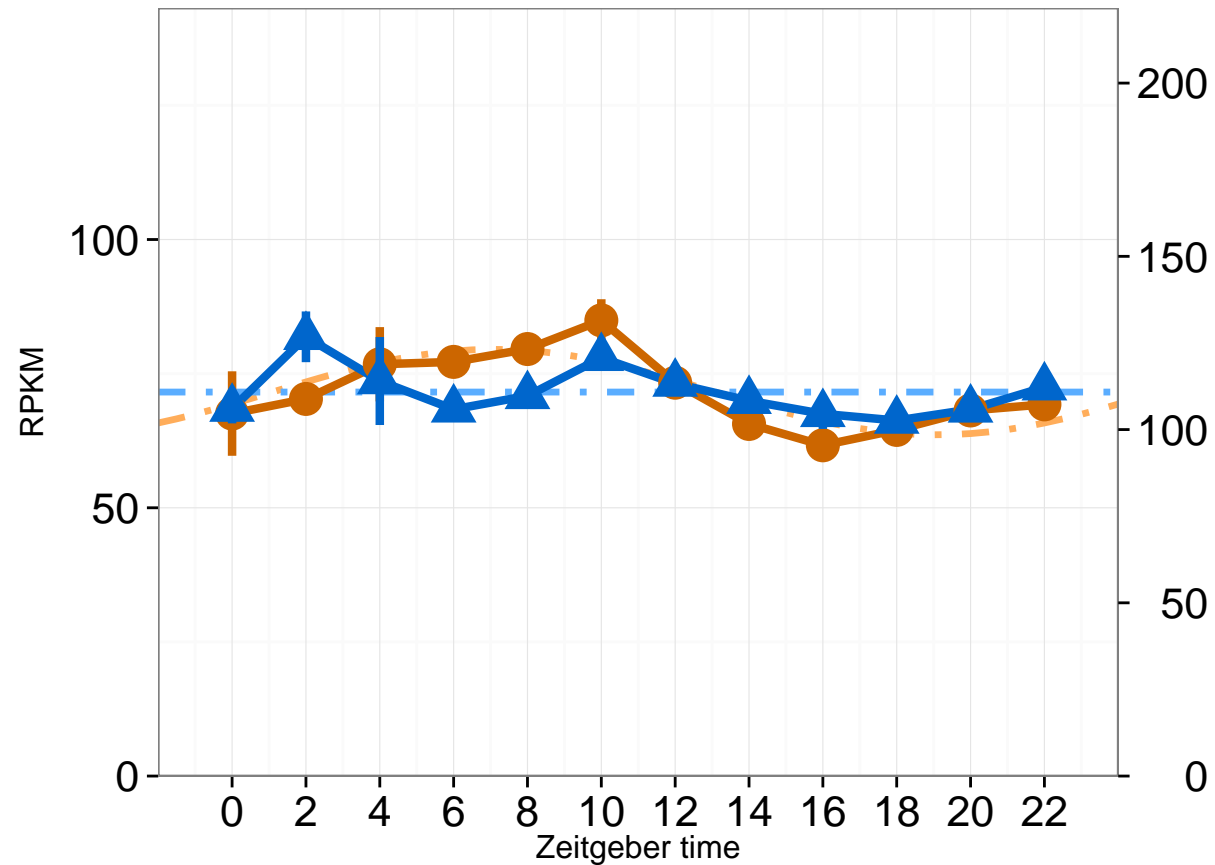

# 1110004F10Rik

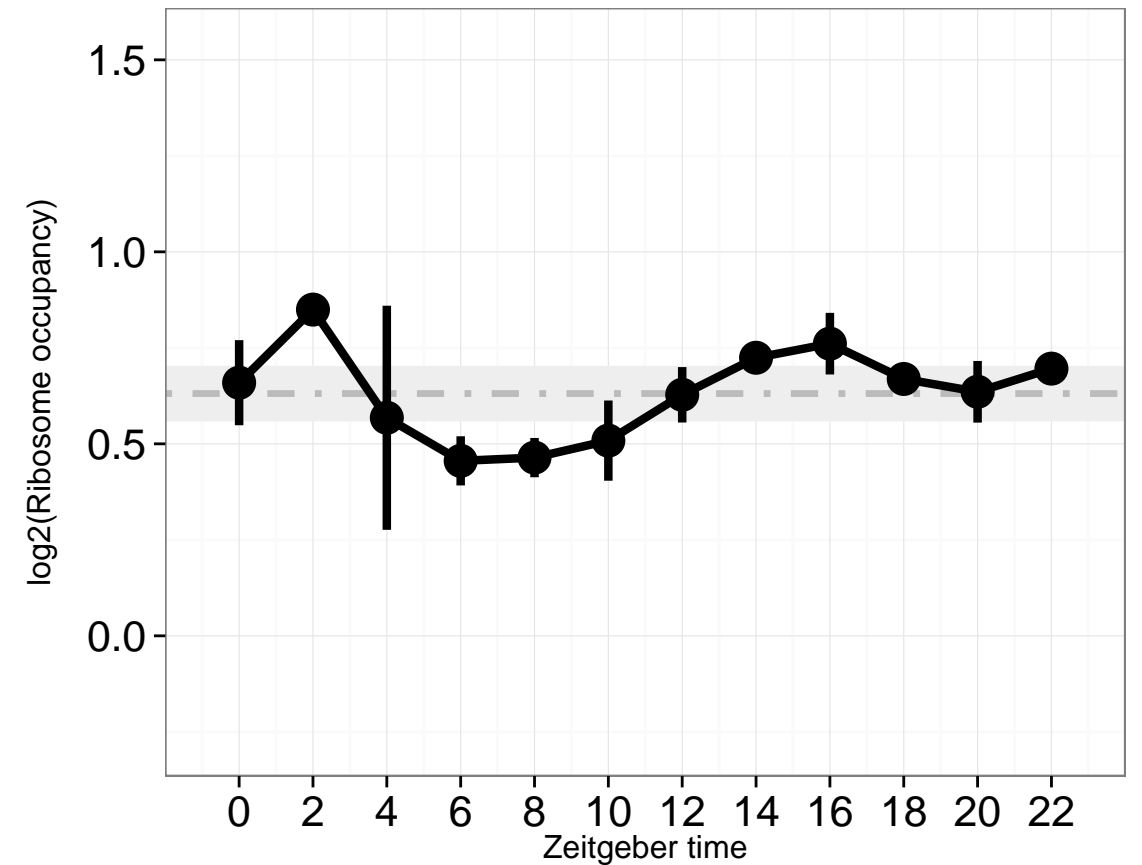

Supplement: Supplementary file 6 — Transcriptome-wide kidney RPF (blue) and RNA (orange) levels in the left panels (with “error bars” connecting the two replicates of each timepoint) and TE in the right panels. (ZIP 116896 kb) [file 13059_2017_1222_MOESM6_ESM.zip › Supp_Dataset_S1/A_RNA_non_rhythmic_RPF_non_rhythmic/1110004F10Rik_kidney_set_A.pdf]

# 1110007C09Rik

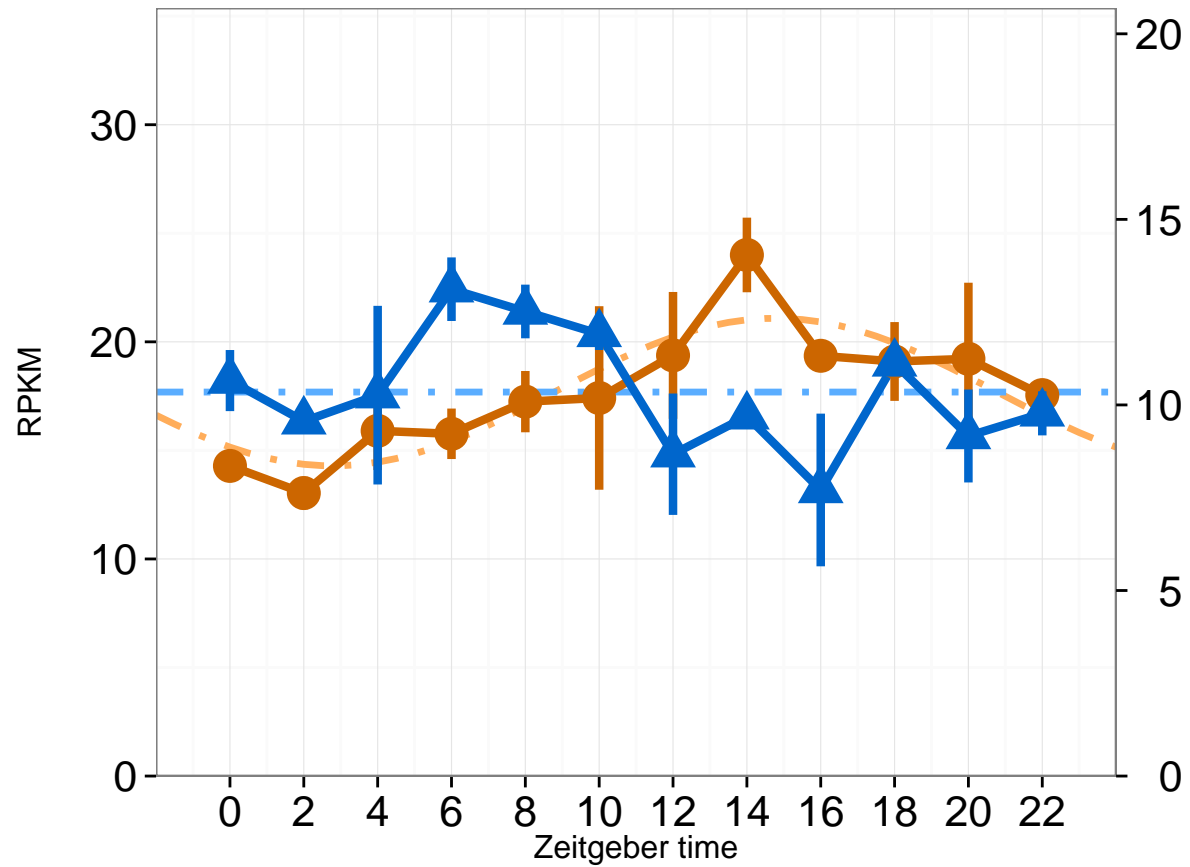

# 1110007C09Rik

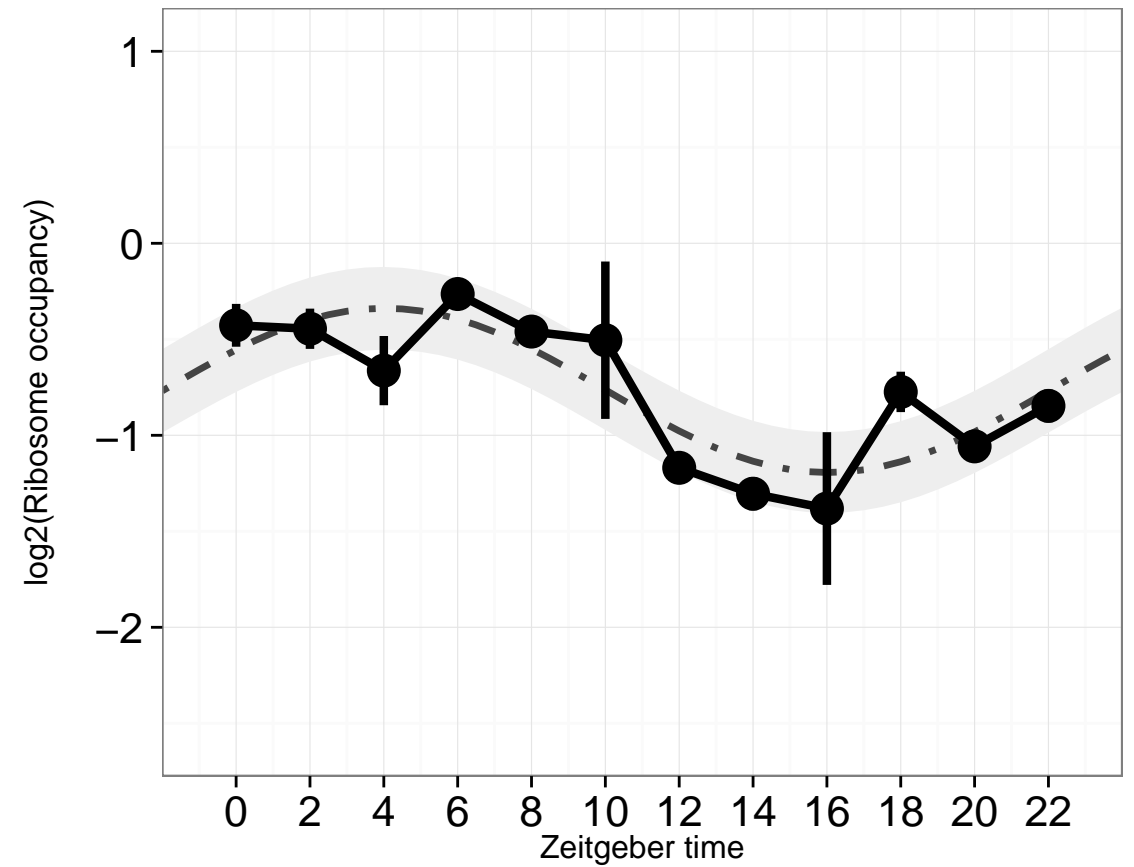

Supplement: Supplementary file 6 — Transcriptome-wide kidney RPF (blue) and RNA (orange) levels in the left panels (with “error bars” connecting the two replicates of each timepoint) and TE in the right panels. (ZIP 116896 kb) [file 13059_2017_1222_MOESM6_ESM.zip › Supp_Dataset_S1/A_RNA_non_rhythmic_RPF_non_rhythmic/1110007C09Rik_kidney_set_A.pdf]

# 1110008F13Rik

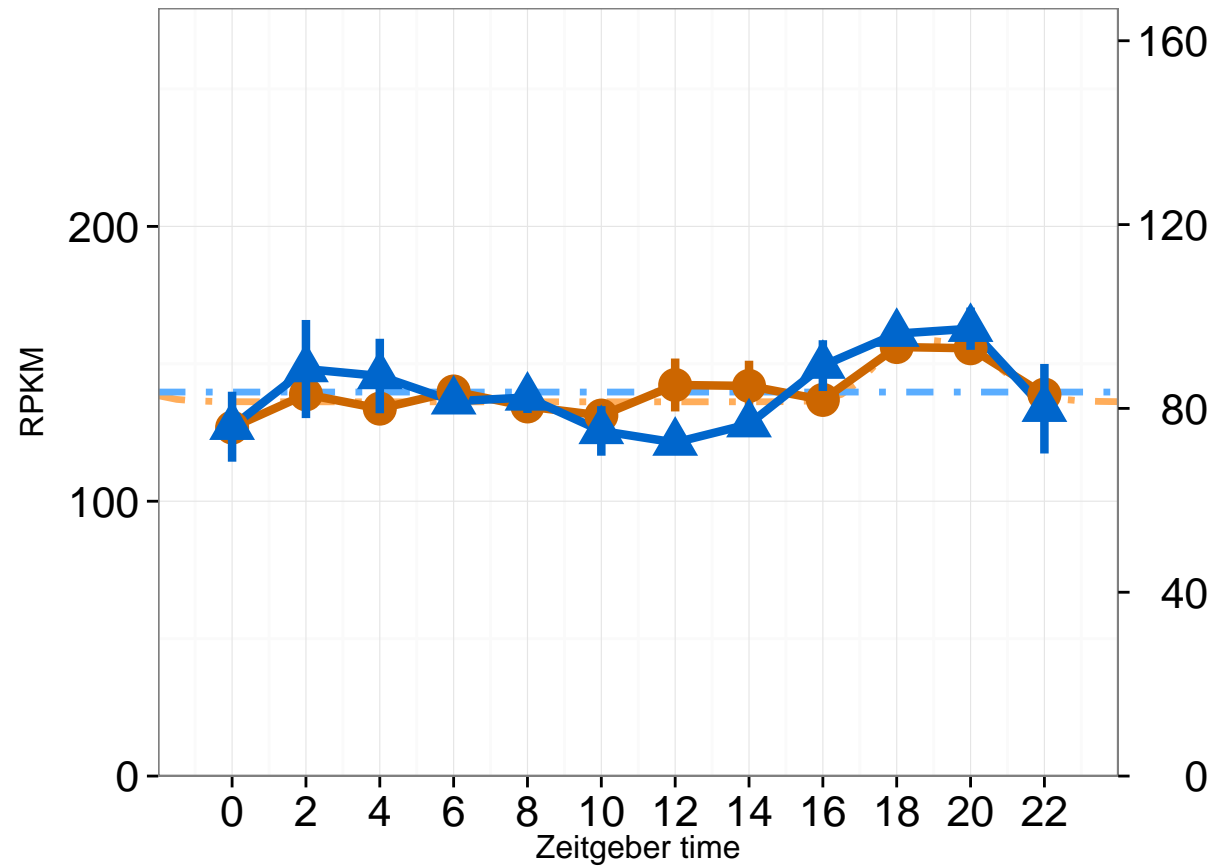

# 1110008F13Rik

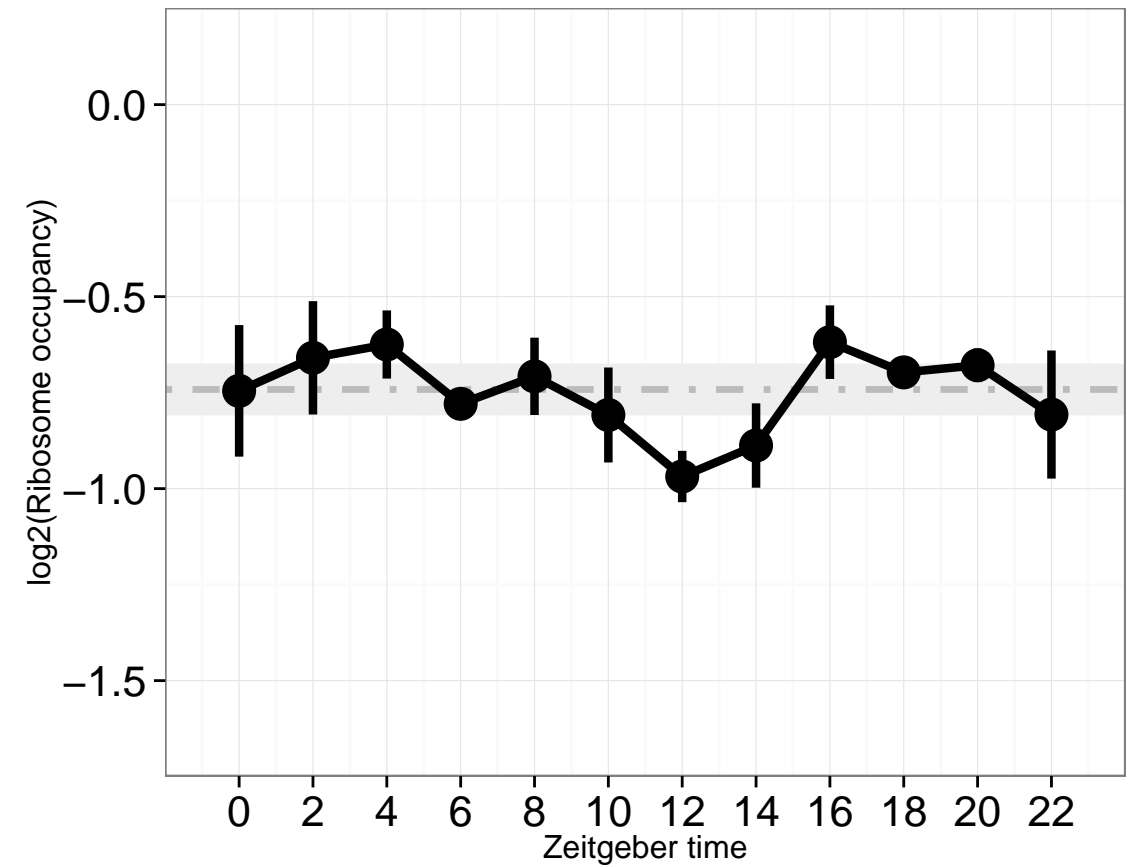

Supplement: Supplementary file 6 — Transcriptome-wide kidney RPF (blue) and RNA (orange) levels in the left panels (with “error bars” connecting the two replicates of each timepoint) and TE in the right panels. (ZIP 116896 kb) [file 13059_2017_1222_MOESM6_ESM.zip › Supp_Dataset_S1/A_RNA_non_rhythmic_RPF_non_rhythmic/1110008F13Rik_kidney_set_A.pdf]

# 1110008J03Rik

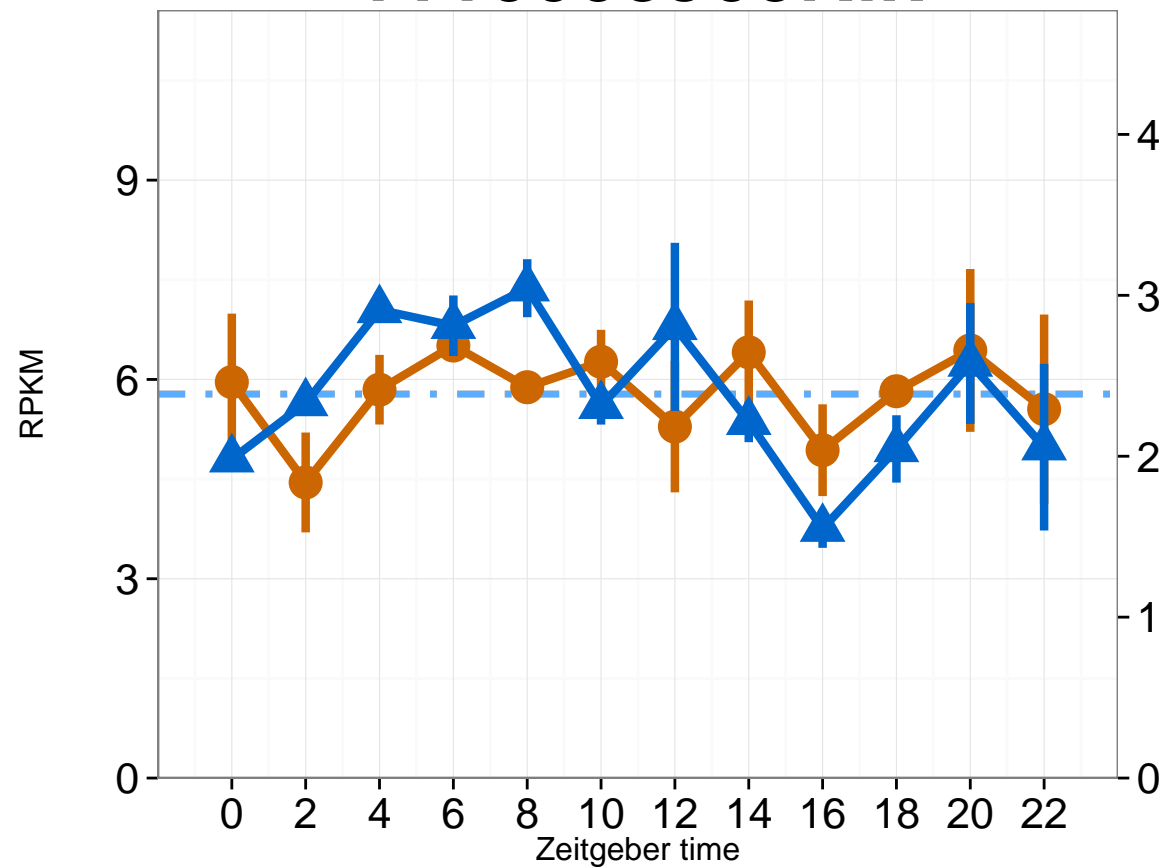

# 1110008J03Rik

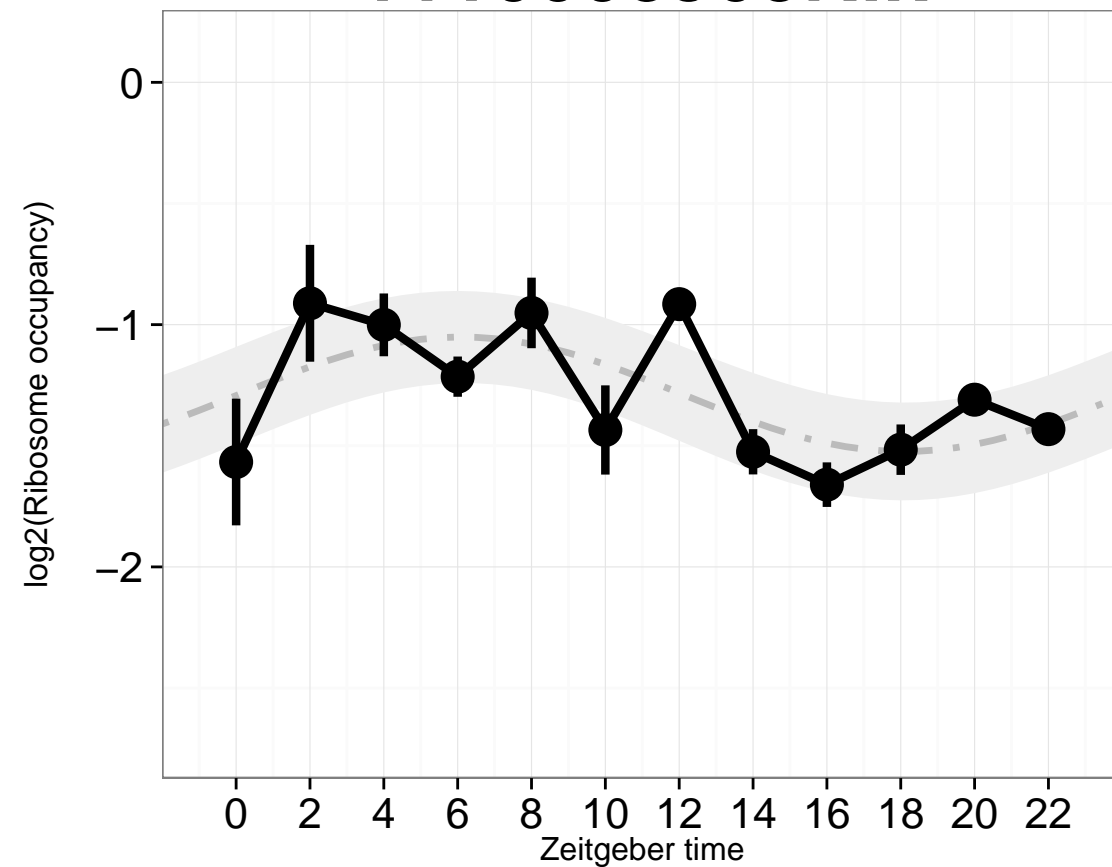

Supplement: Supplementary file 6 — Transcriptome-wide kidney RPF (blue) and RNA (orange) levels in the left panels (with “error bars” connecting the two replicates of each timepoint) and TE in the right panels. (ZIP 116896 kb) [file 13059_2017_1222_MOESM6_ESM.zip › Supp_Dataset_S1/A_RNA_non_rhythmic_RPF_non_rhythmic/1110008J03Rik_kidney_set_A.pdf]

# 1110008L16Rik

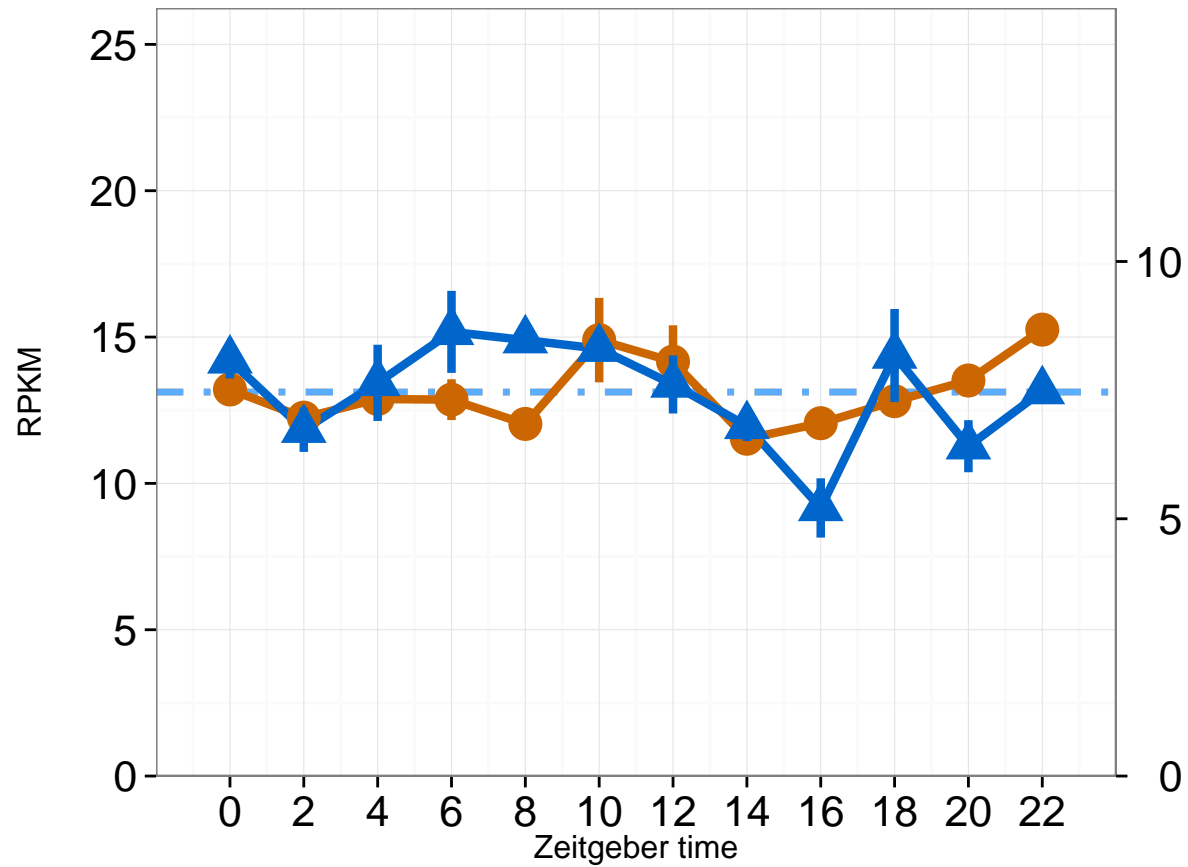

# 1110008L16Rik

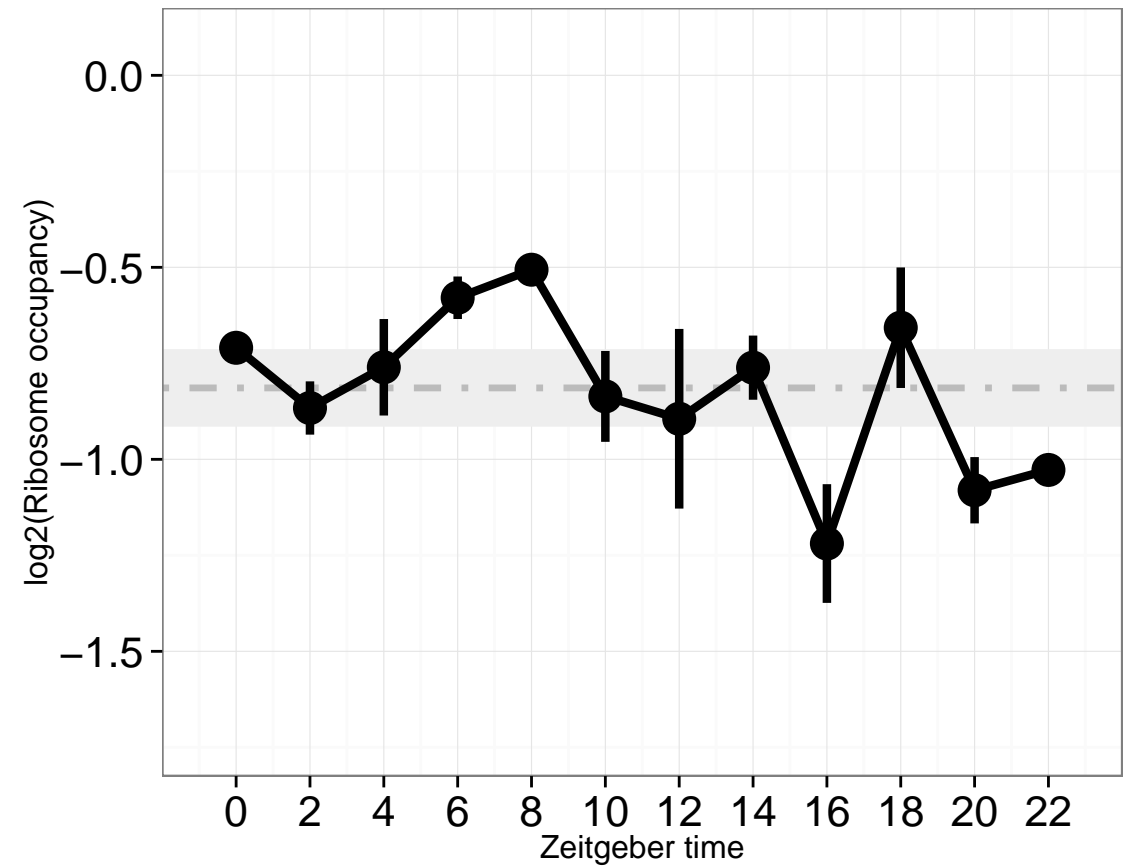

Supplement: Supplementary file 6 — Transcriptome-wide kidney RPF (blue) and RNA (orange) levels in the left panels (with “error bars” connecting the two replicates of each timepoint) and TE in the right panels. (ZIP 116896 kb) [file 13059_2017_1222_MOESM6_ESM.zip › Supp_Dataset_S1/A_RNA_non_rhythmic_RPF_non_rhythmic/1110008L16Rik_kidney_set_A.pdf]

# 1110008P14Rik

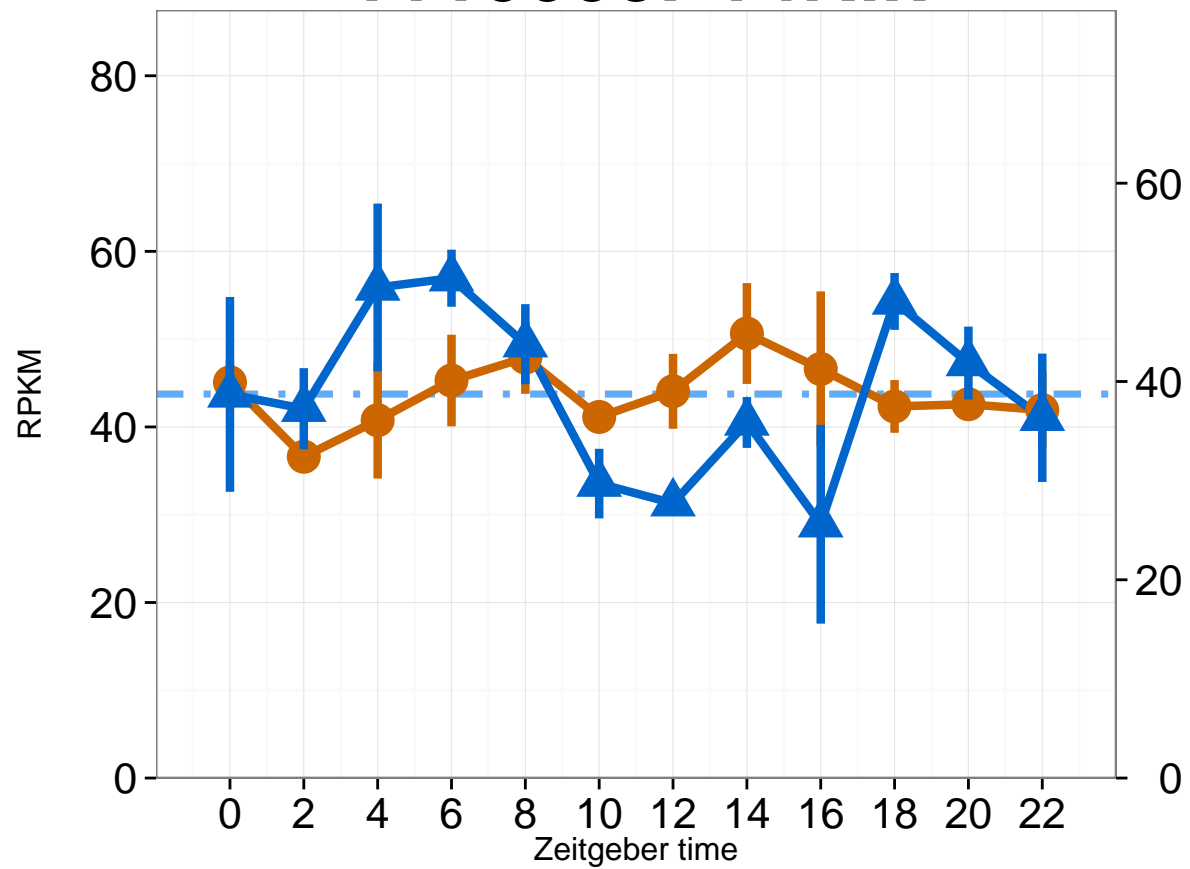

# 1110008P14Rik

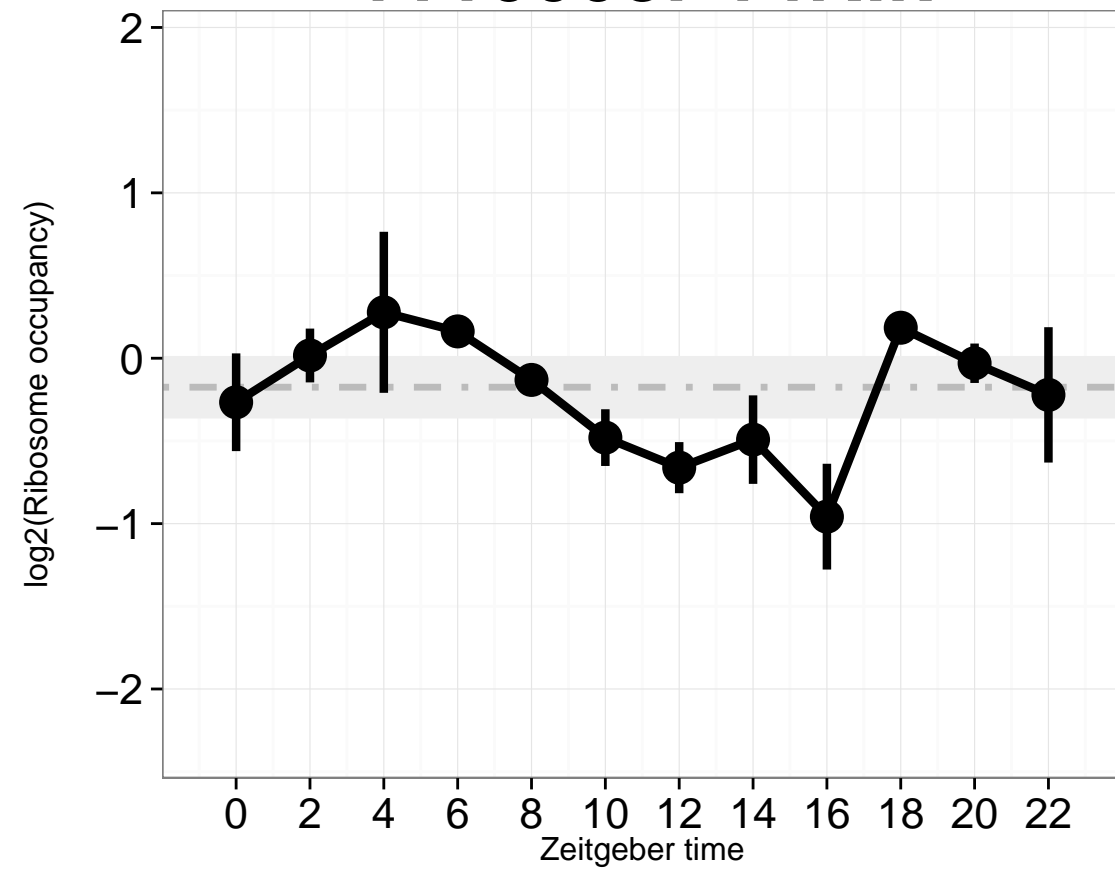

Supplement: Supplementary file 6 — Transcriptome-wide kidney RPF (blue) and RNA (orange) levels in the left panels (with “error bars” connecting the two replicates of each timepoint) and TE in the right panels. (ZIP 116896 kb) [file 13059_2017_1222_MOESM6_ESM.zip › Supp_Dataset_S1/A_RNA_non_rhythmic_RPF_non_rhythmic/1110008P14Rik_kidney_set_A.pdf]

# 1110012L19Rik

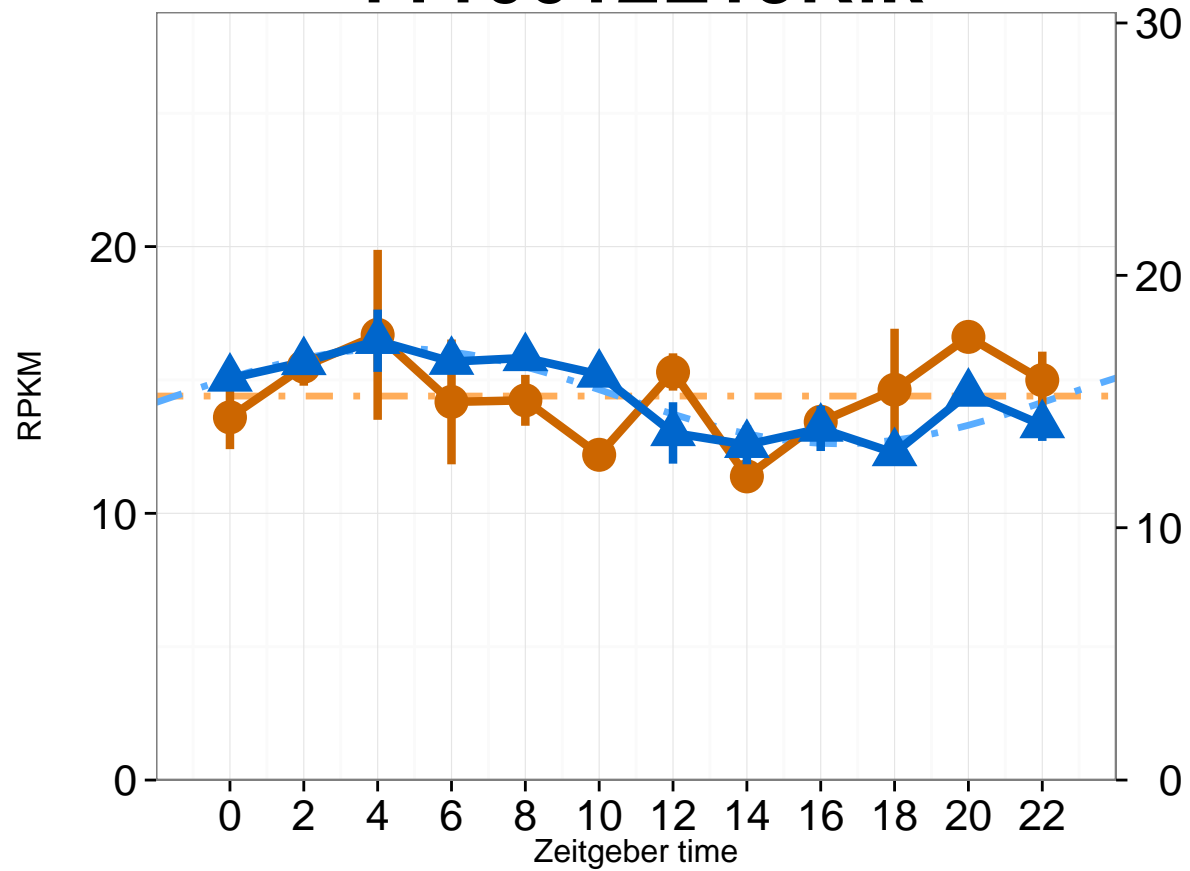

# 1110012L19Rik

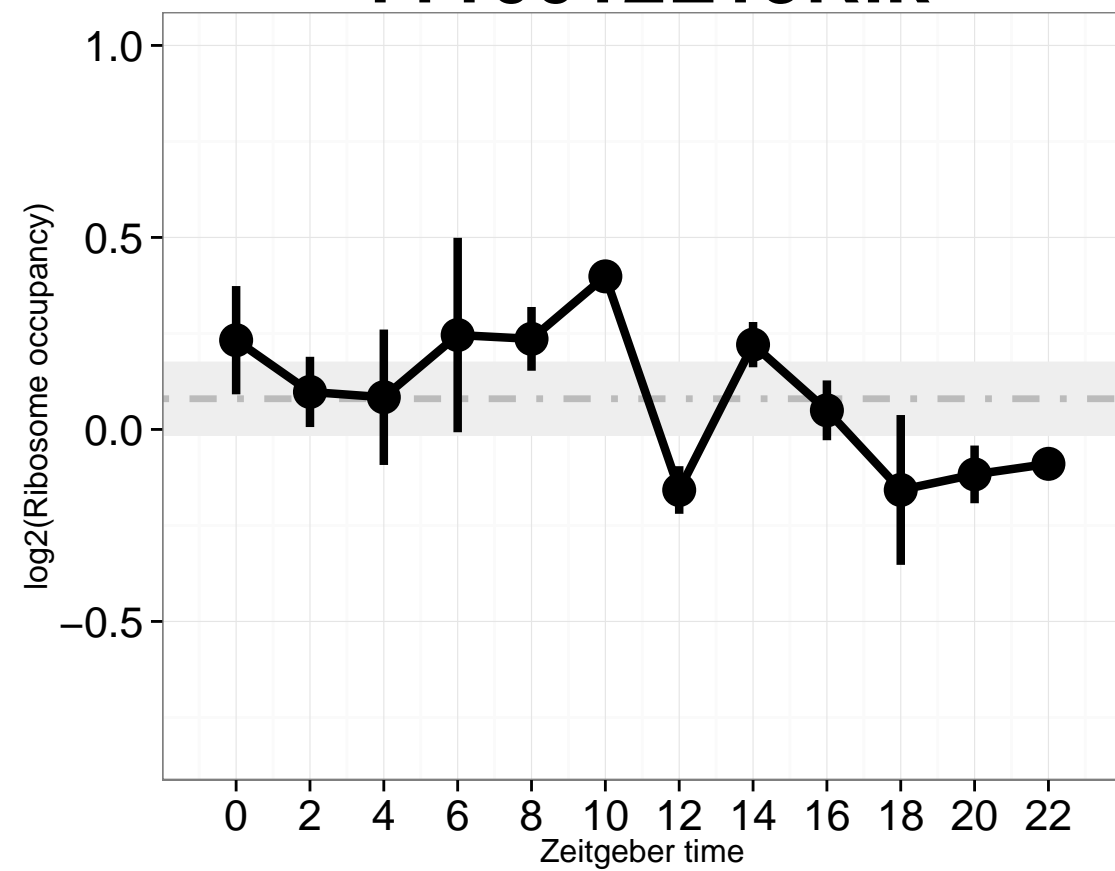

Supplement: Supplementary file 6 — Transcriptome-wide kidney RPF (blue) and RNA (orange) levels in the left panels (with “error bars” connecting the two replicates of each timepoint) and TE in the right panels. (ZIP 116896 kb) [file 13059_2017_1222_MOESM6_ESM.zip › Supp_Dataset_S1/A_RNA_non_rhythmic_RPF_non_rhythmic/1110012L19Rik_kidney_set_A.pdf]

# 1110032A03Rik

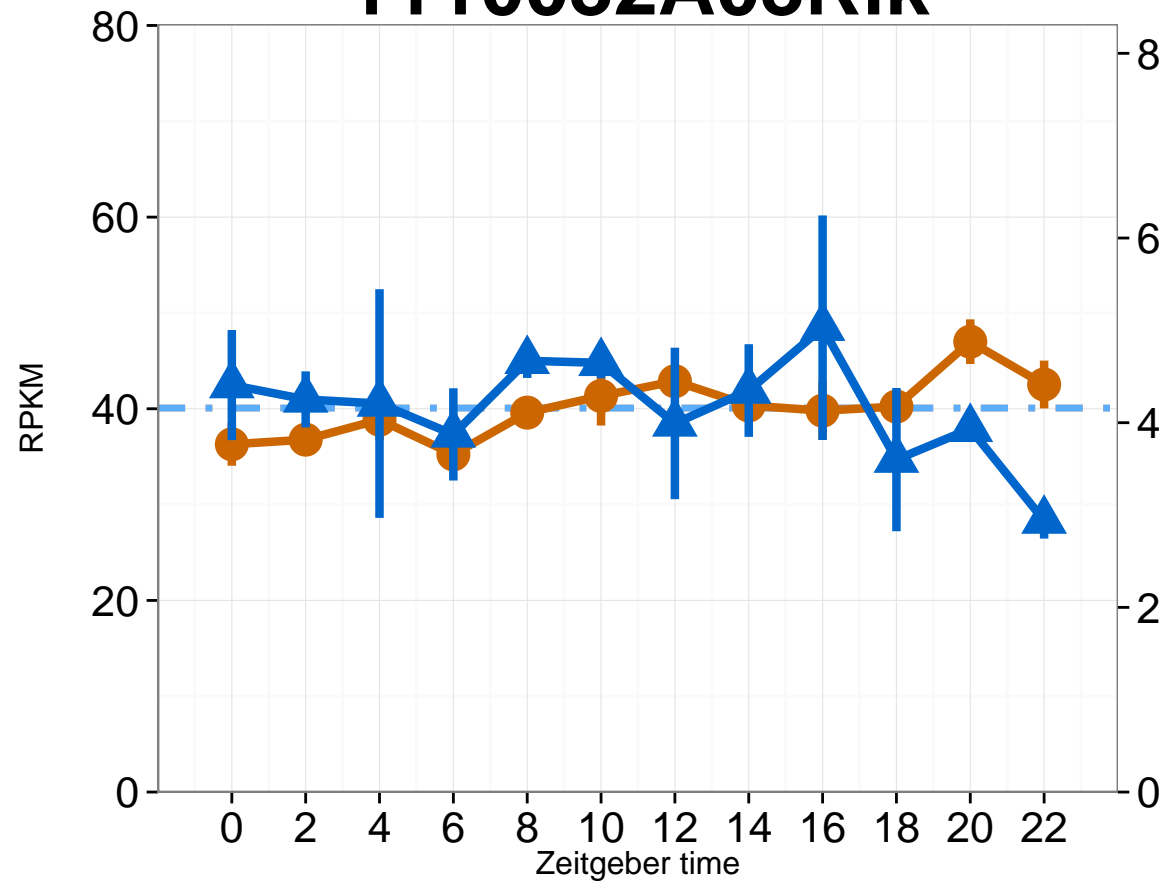

# 1110032A03Rik

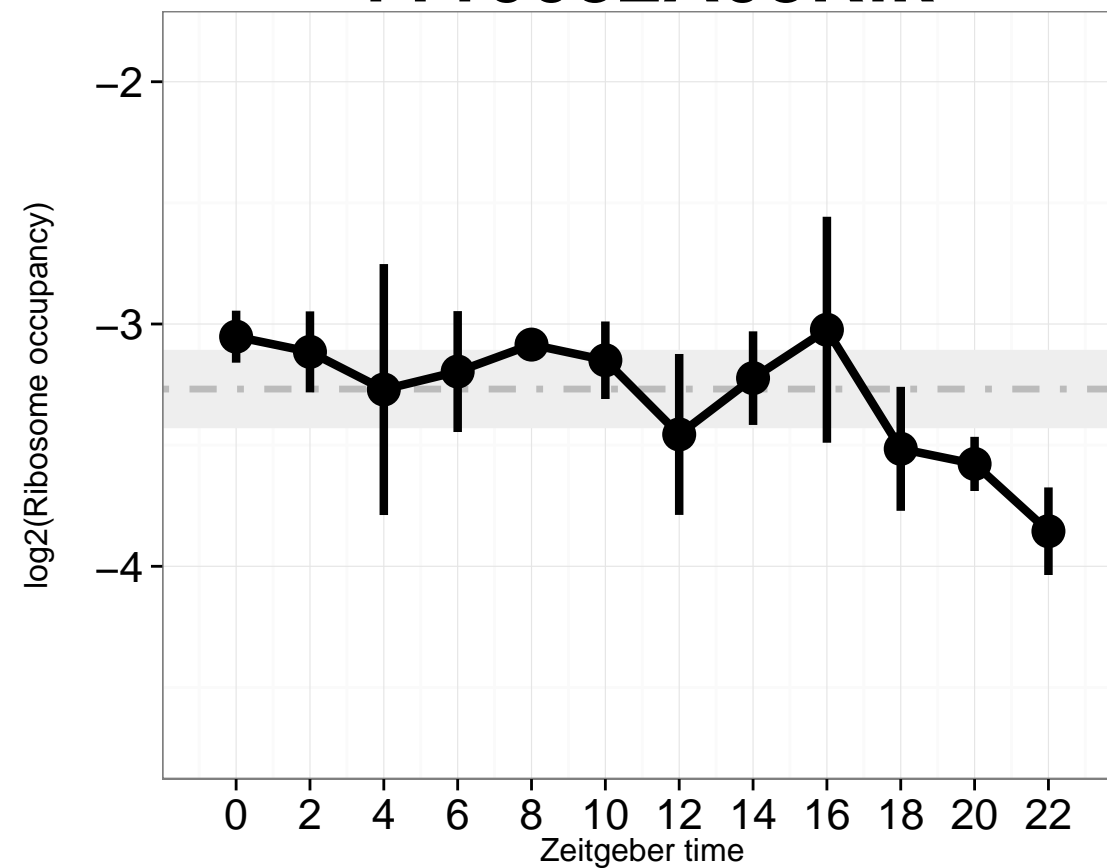

Supplement: Supplementary file 6 — Transcriptome-wide kidney RPF (blue) and RNA (orange) levels in the left panels (with “error bars” connecting the two replicates of each timepoint) and TE in the right panels. (ZIP 116896 kb) [file 13059_2017_1222_MOESM6_ESM.zip › Supp_Dataset_S1/A_RNA_non_rhythmic_RPF_non_rhythmic/1110032A03Rik_kidney_set_A.pdf]

# 1110032F04Rik

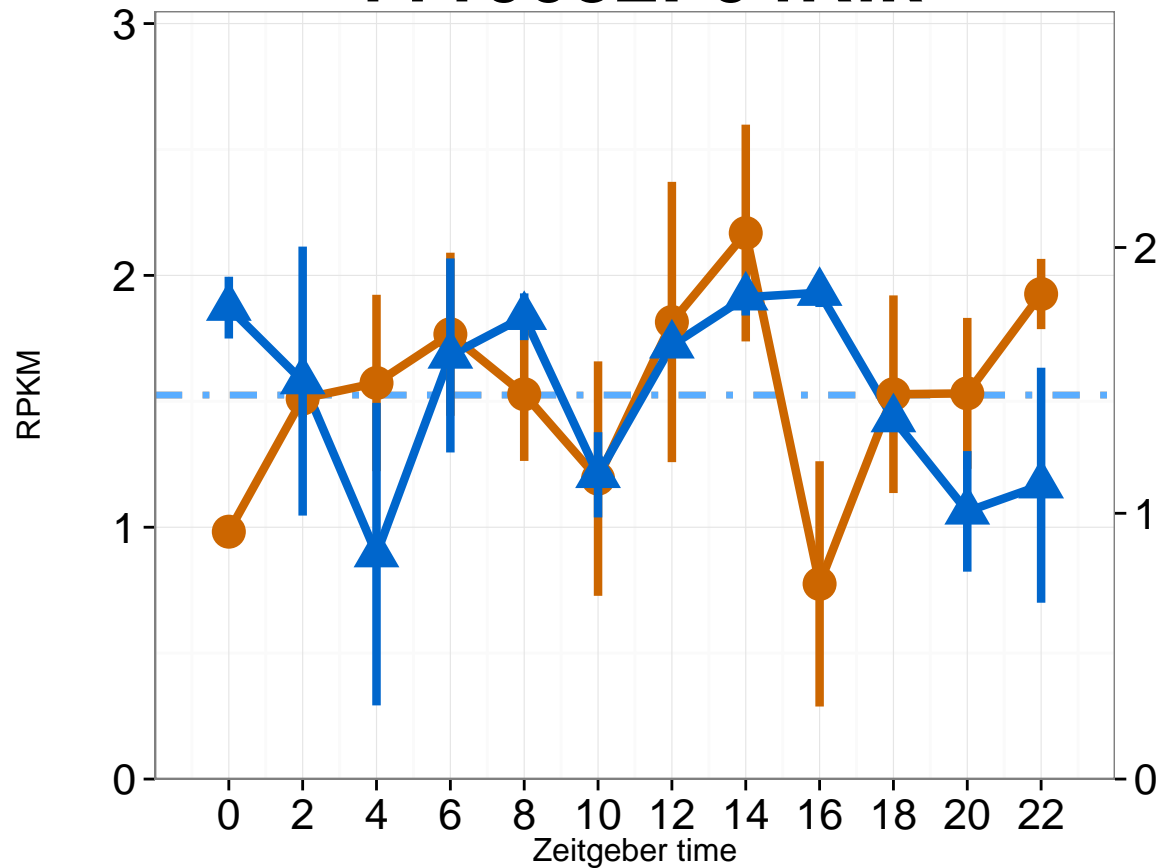

# 1110032F04Rik

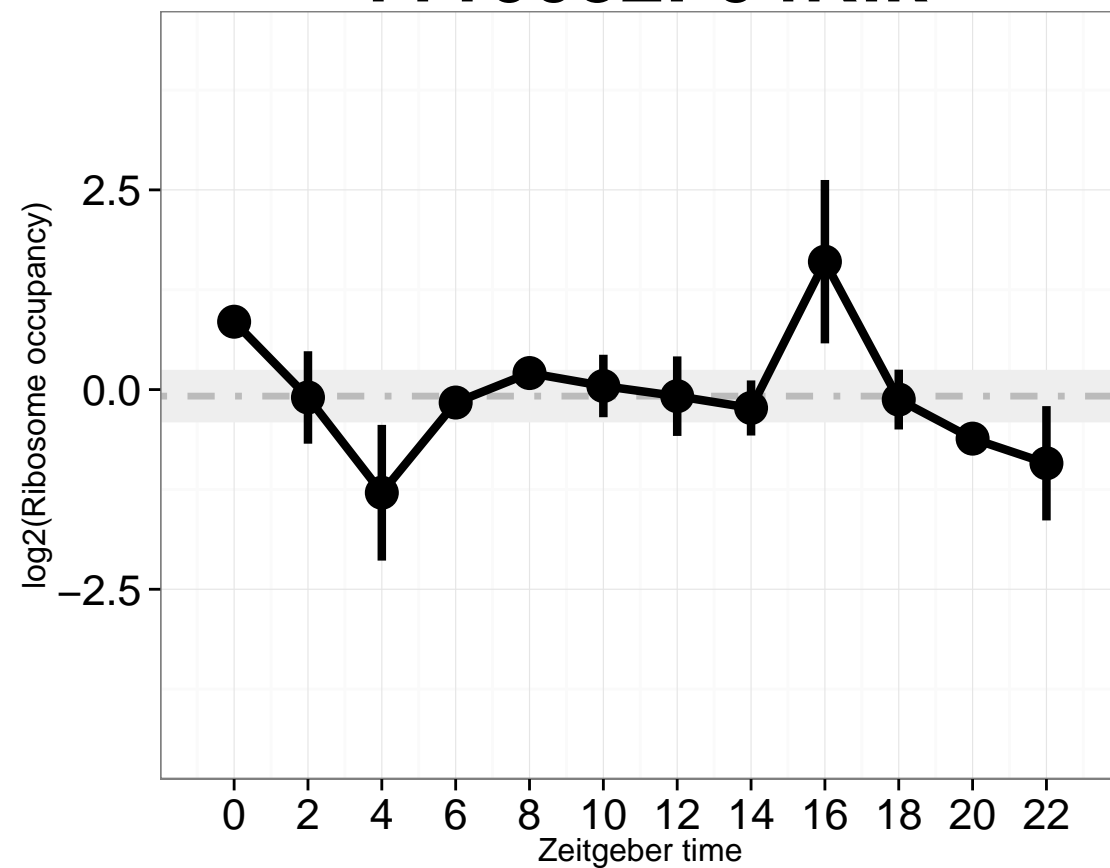

Supplement: Supplementary file 6 — Transcriptome-wide kidney RPF (blue) and RNA (orange) levels in the left panels (with “error bars” connecting the two replicates of each timepoint) and TE in the right panels. (ZIP 116896 kb) [file 13059_2017_1222_MOESM6_ESM.zip › Supp_Dataset_S1/A_RNA_non_rhythmic_RPF_non_rhythmic/1110032F04Rik_kidney_set_A.pdf]

# 1110034G24Rik

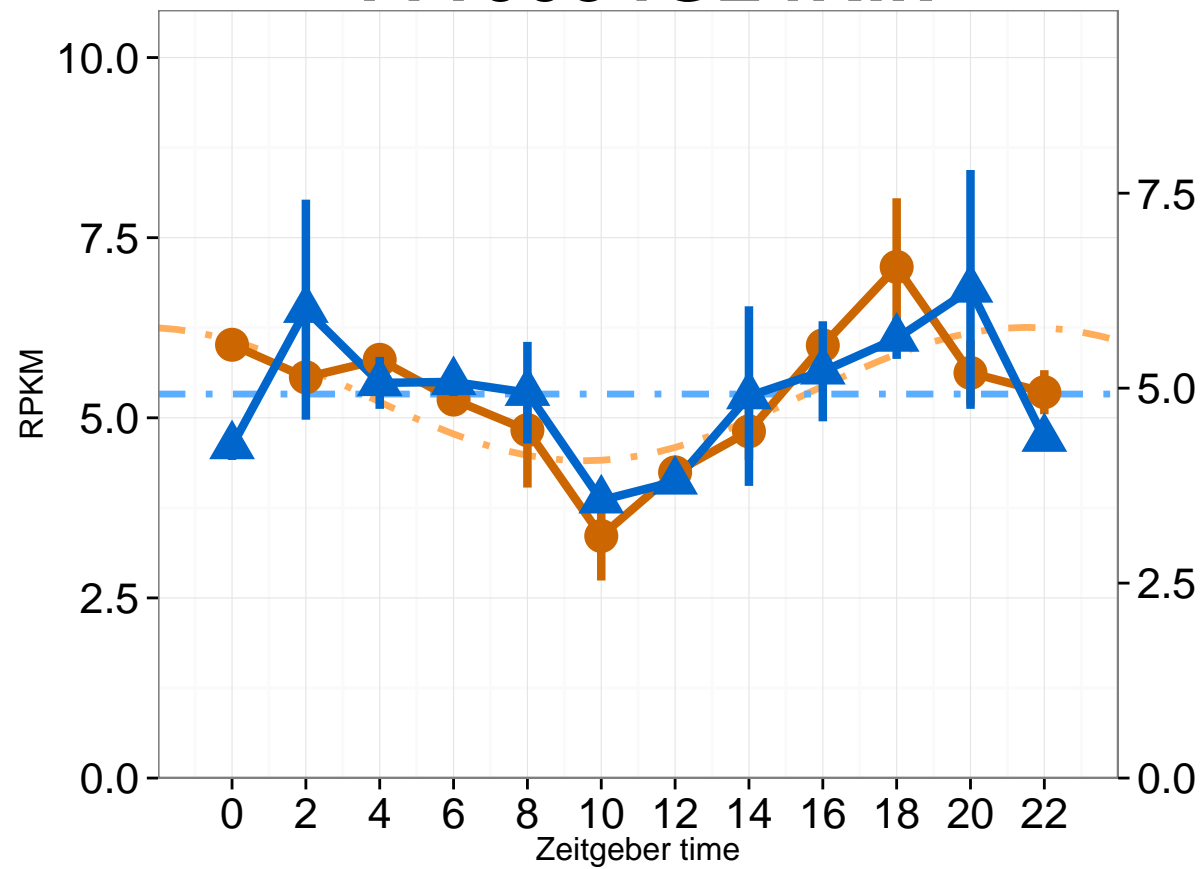

# 1110034G24Rik

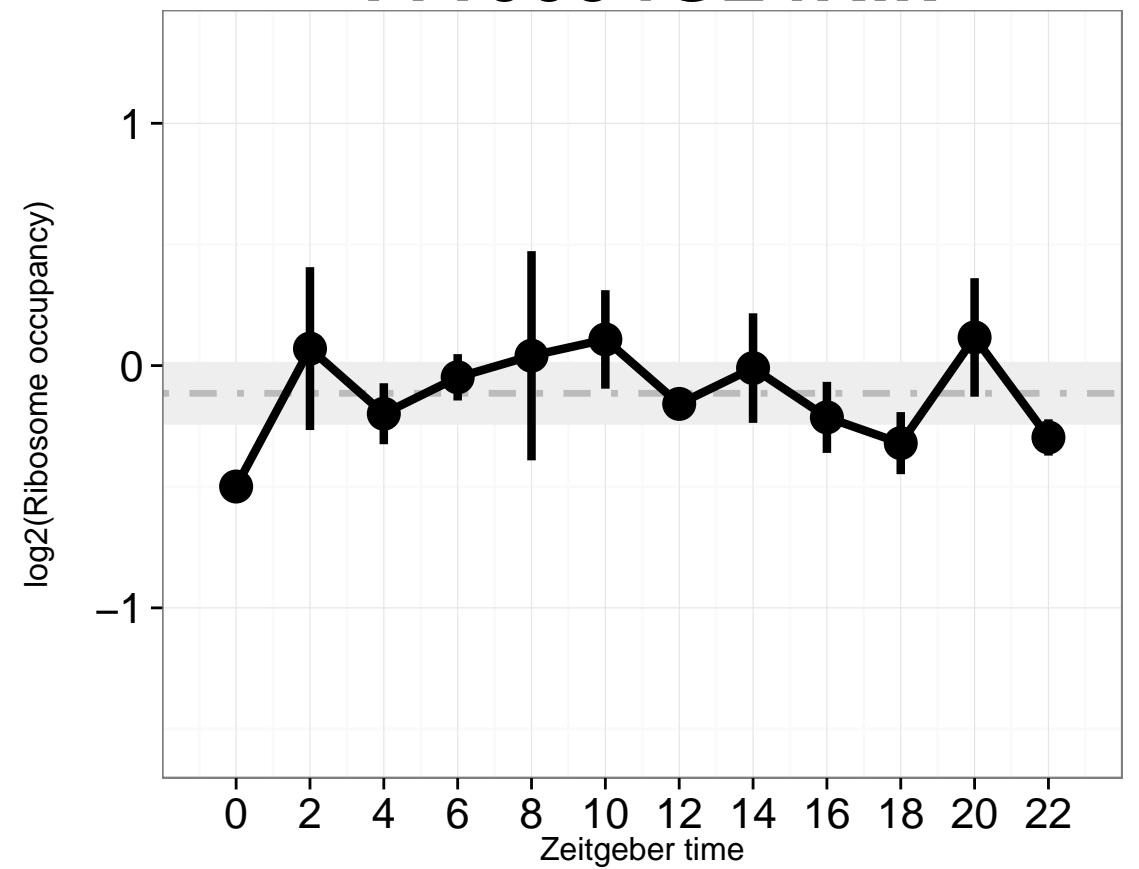

Supplement: Supplementary file 6 — Transcriptome-wide kidney RPF (blue) and RNA (orange) levels in the left panels (with “error bars” connecting the two replicates of each timepoint) and TE in the right panels. (ZIP 116896 kb) [file 13059_2017_1222_MOESM6_ESM.zip › Supp_Dataset_S1/A_RNA_non_rhythmic_RPF_non_rhythmic/1110034G24Rik_kidney_set_A.pdf]

# 1110037F02Rik

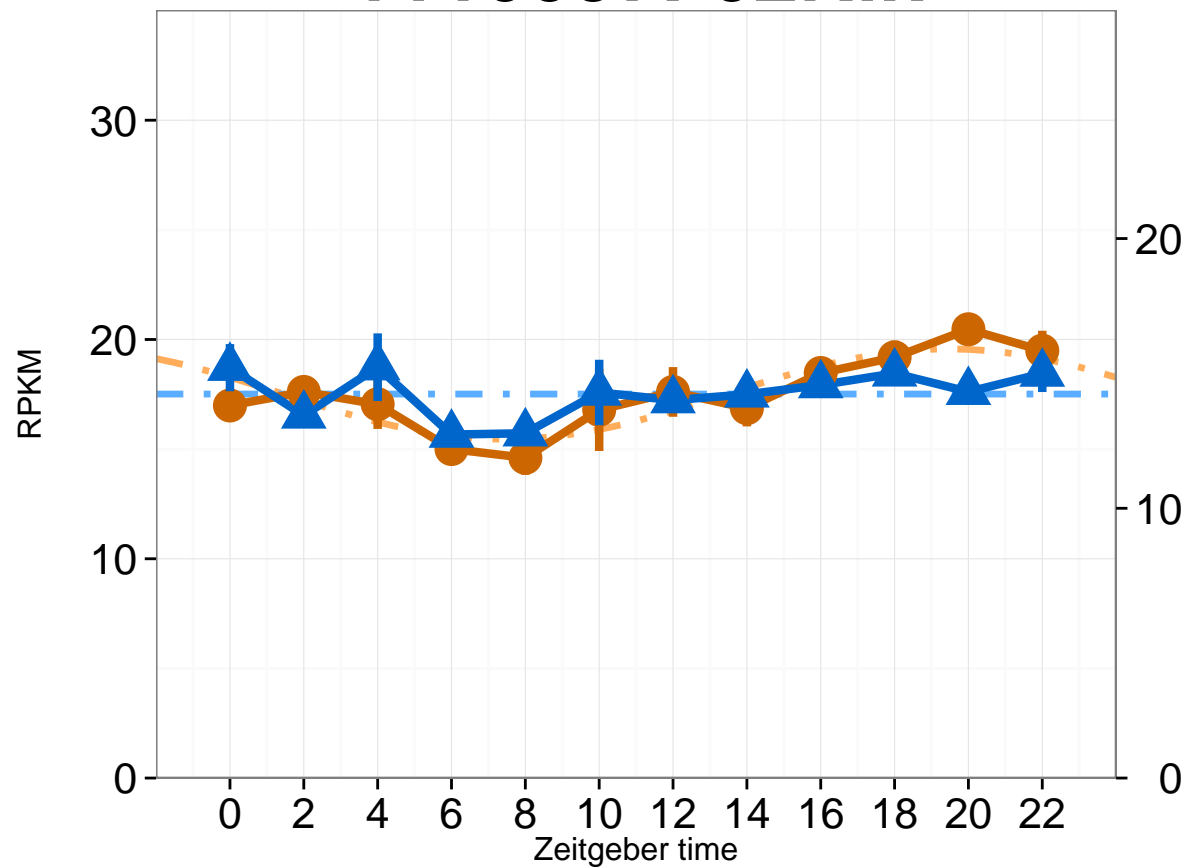

# 1110037F02Rik

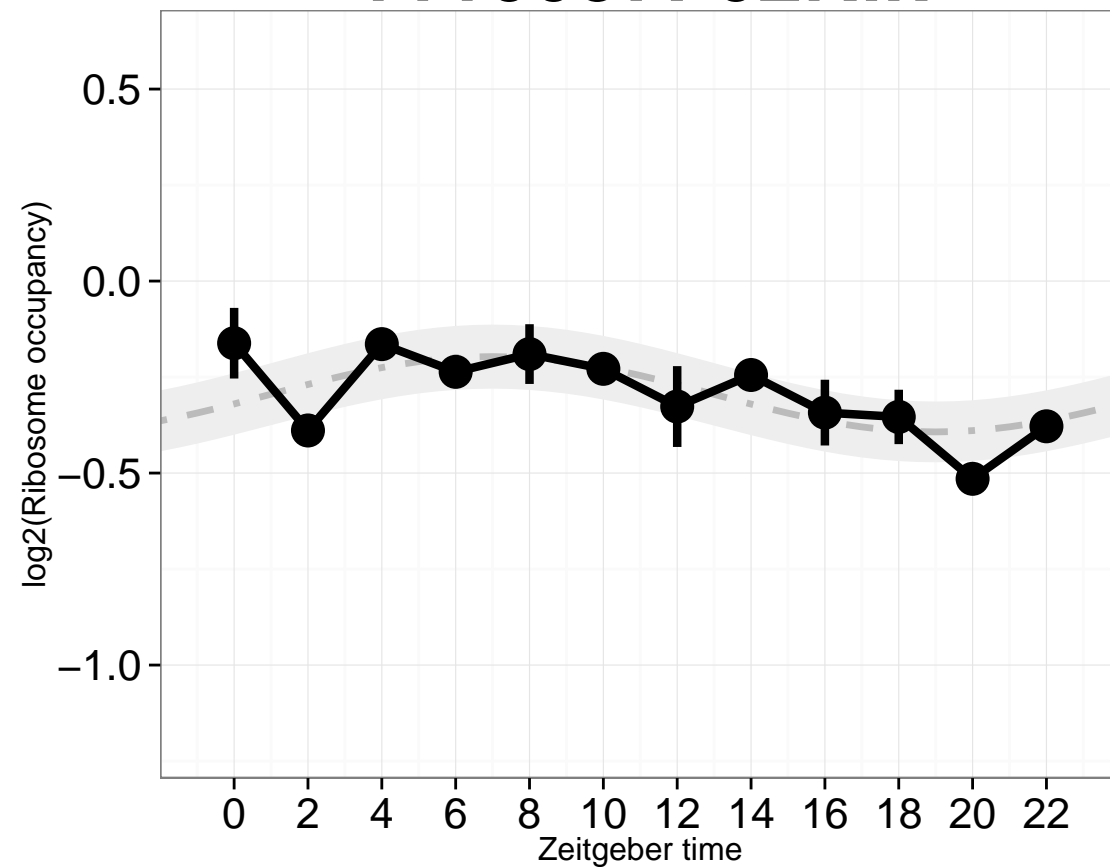

Supplement: Supplementary file 6 — Transcriptome-wide kidney RPF (blue) and RNA (orange) levels in the left panels (with “error bars” connecting the two replicates of each timepoint) and TE in the right panels. (ZIP 116896 kb) [file 13059_2017_1222_MOESM6_ESM.zip › Supp_Dataset_S1/A_RNA_non_rhythmic_RPF_non_rhythmic/1110037F02Rik_kidney_set_A.pdf]

# 1110038F14Rik

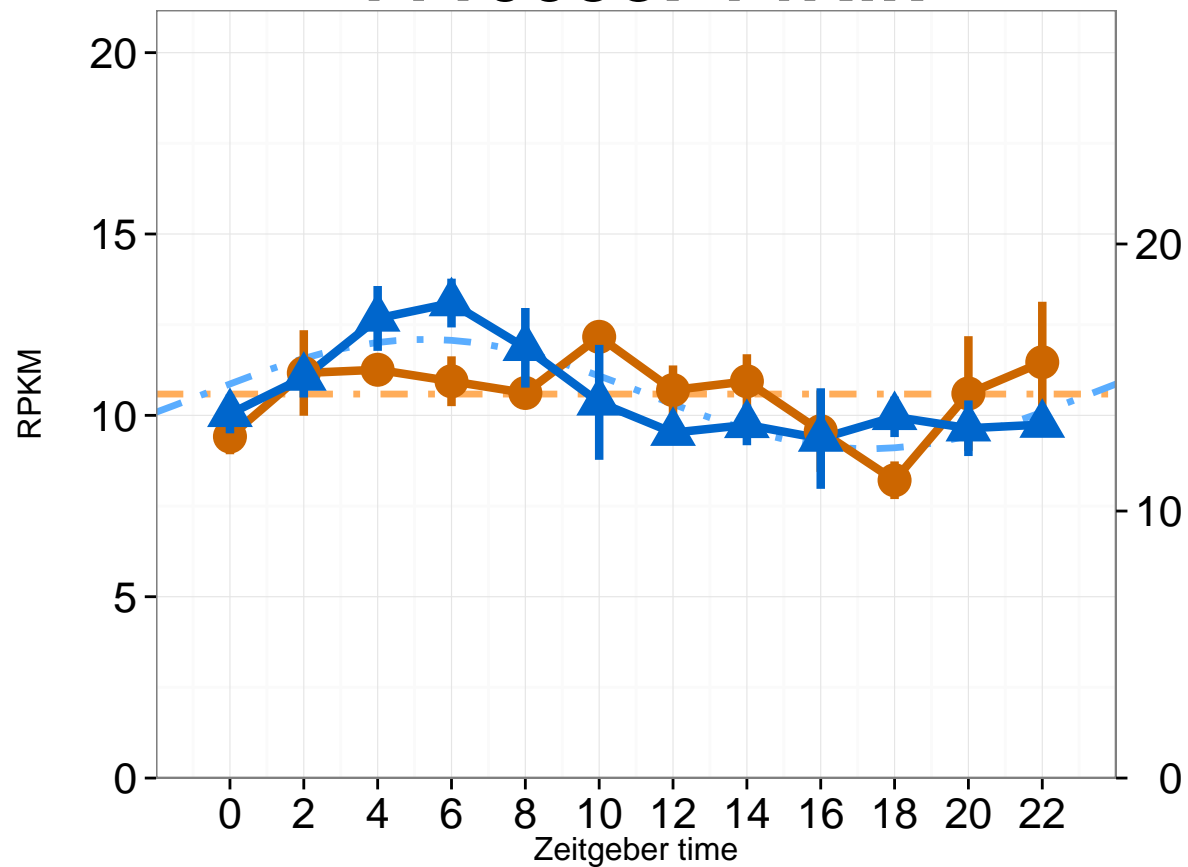

# 1110038F14Rik

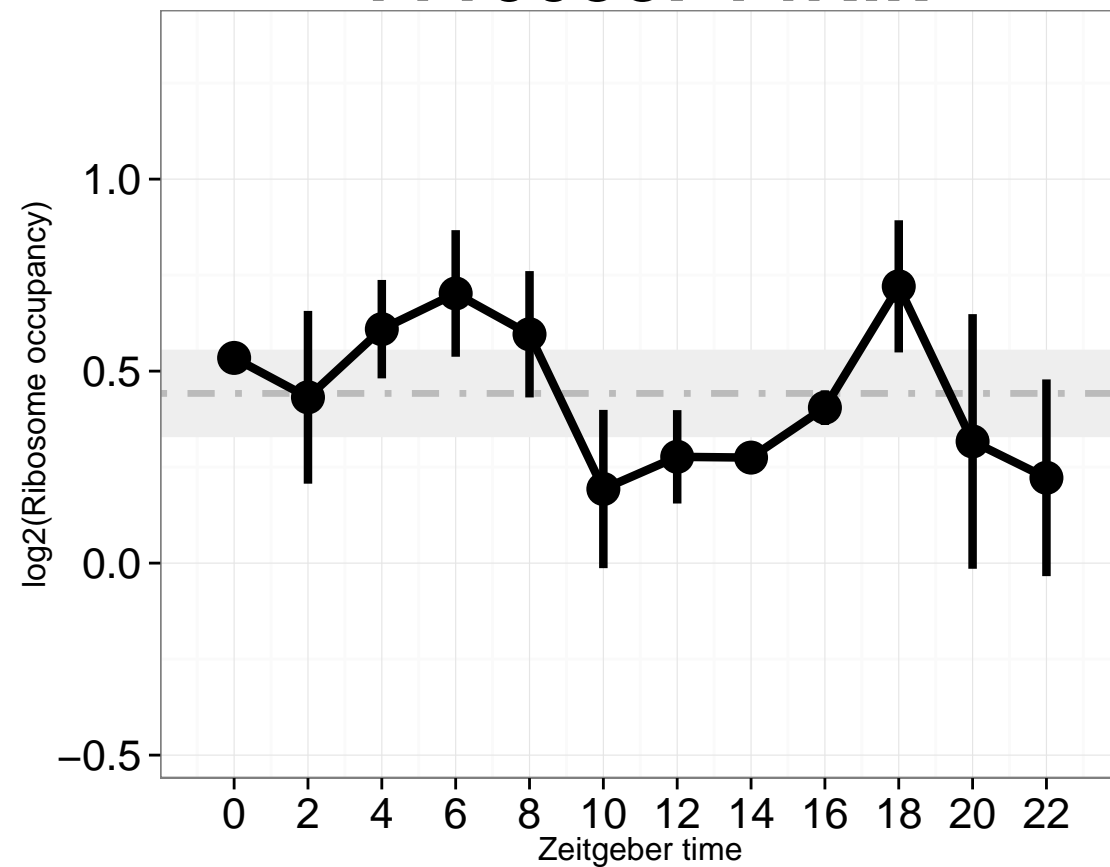

Supplement: Supplementary file 6 — Transcriptome-wide kidney RPF (blue) and RNA (orange) levels in the left panels (with “error bars” connecting the two replicates of each timepoint) and TE in the right panels. (ZIP 116896 kb) [file 13059_2017_1222_MOESM6_ESM.zip › Supp_Dataset_S1/A_RNA_non_rhythmic_RPF_non_rhythmic/1110038F14Rik_kidney_set_A.pdf]

# 1110051M20Rik

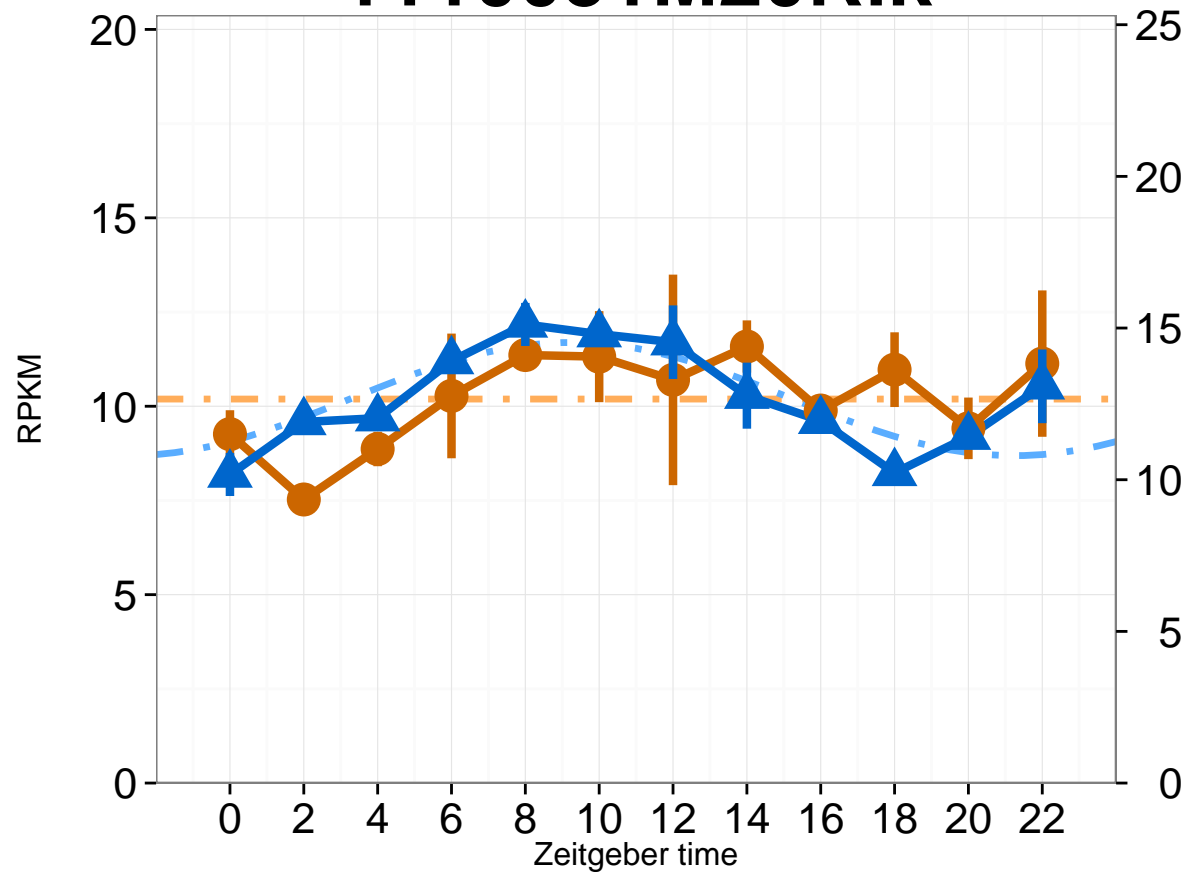

# 1110051M20Rik

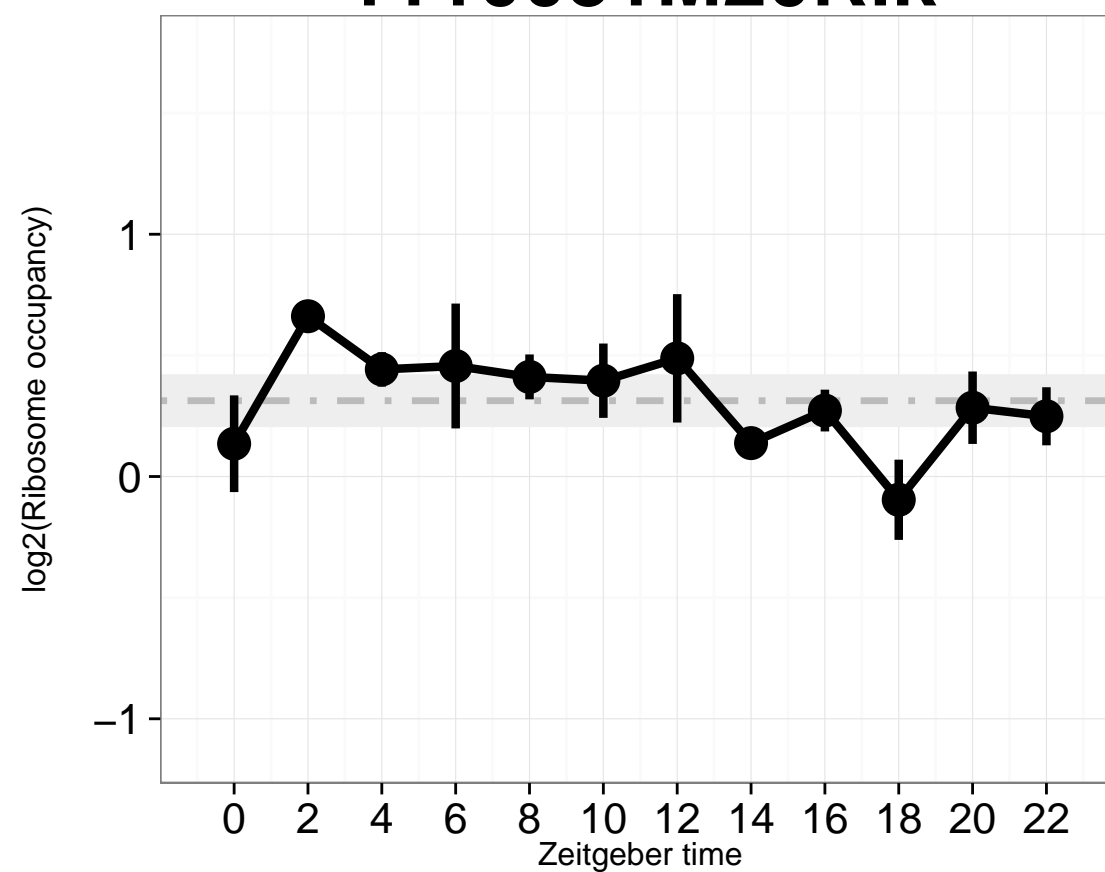

Supplement: Supplementary file 6 — Transcriptome-wide kidney RPF (blue) and RNA (orange) levels in the left panels (with “error bars” connecting the two replicates of each timepoint) and TE in the right panels. (ZIP 116896 kb) [file 13059_2017_1222_MOESM6_ESM.zip › Supp_Dataset_S1/A_RNA_non_rhythmic_RPF_non_rhythmic/1110051M20Rik_kidney_set_A.pdf]

# 1110058L19Rik

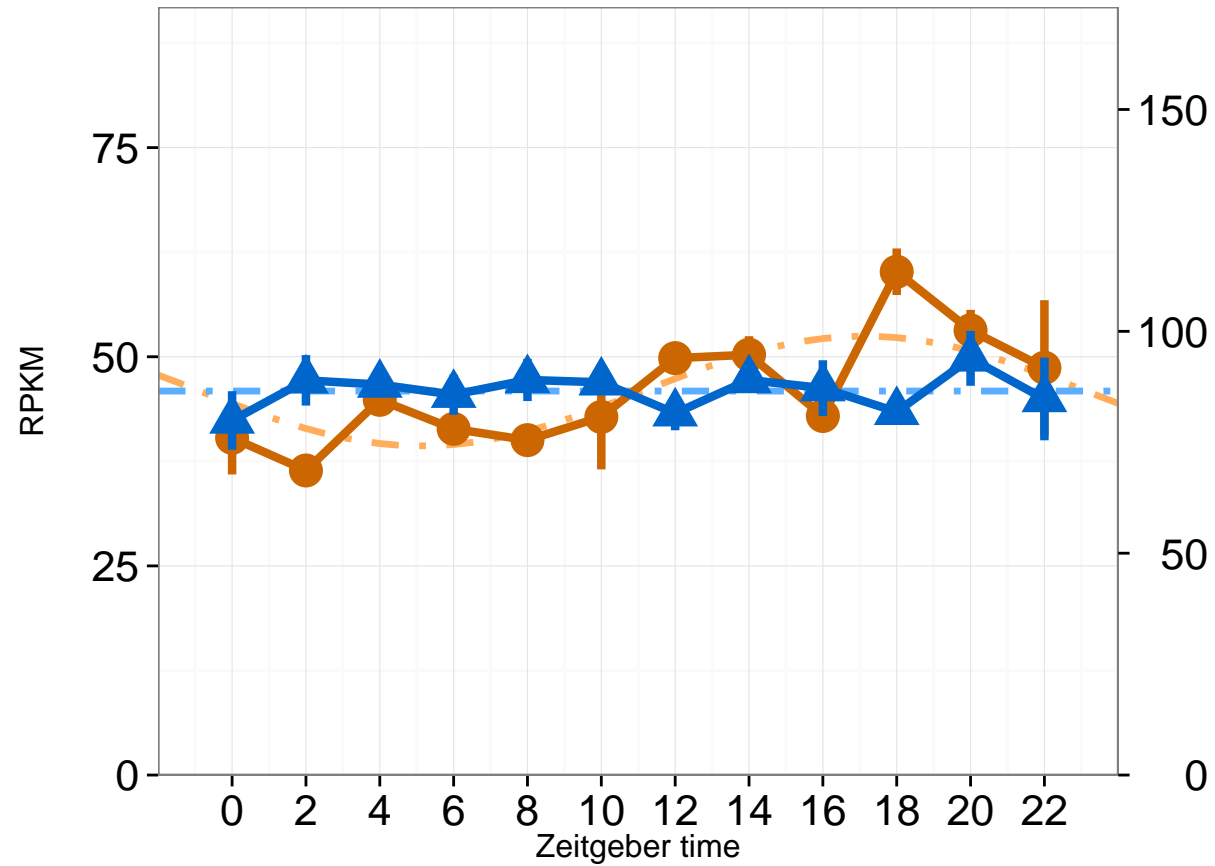

# 1110058L19Rik

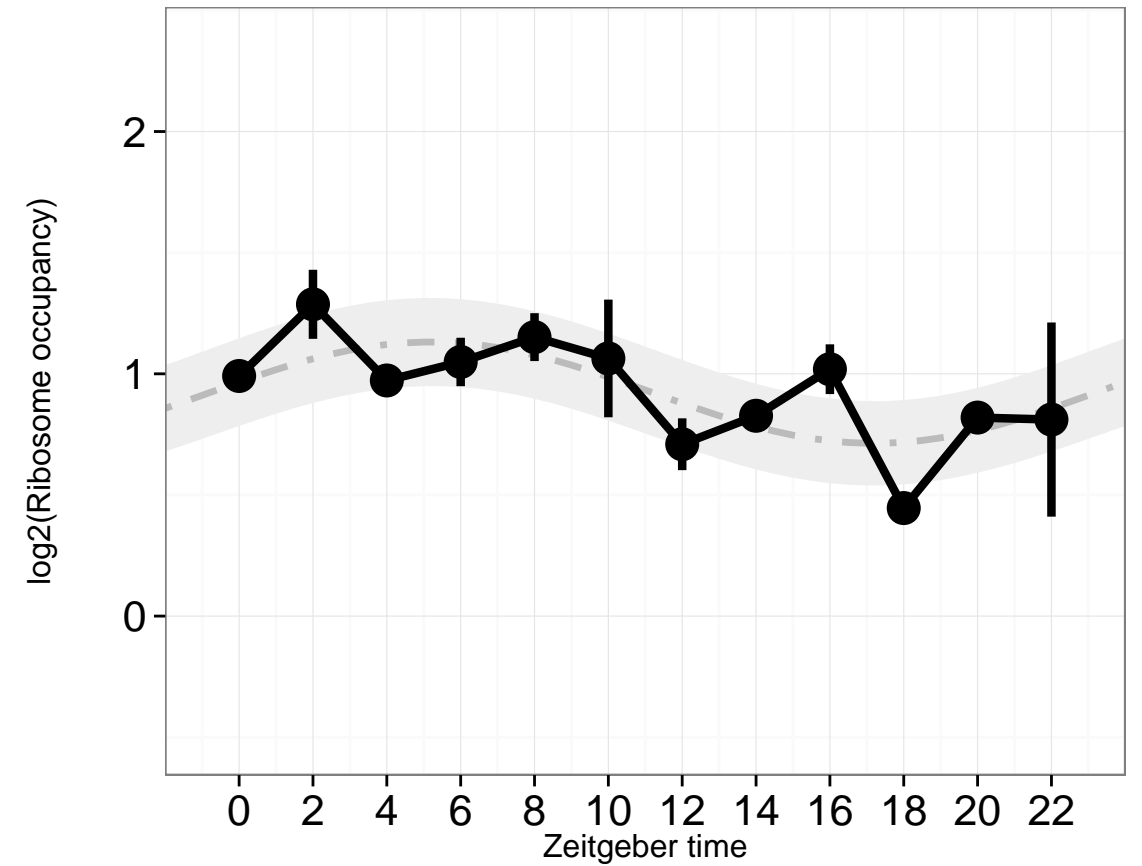

Supplement: Supplementary file 6 — Transcriptome-wide kidney RPF (blue) and RNA (orange) levels in the left panels (with “error bars” connecting the two replicates of each timepoint) and TE in the right panels. (ZIP 116896 kb) [file 13059_2017_1222_MOESM6_ESM.zip › Supp_Dataset_S1/A_RNA_non_rhythmic_RPF_non_rhythmic/1110058L19Rik_kidney_set_A.pdf]

# 1110059E24Rik

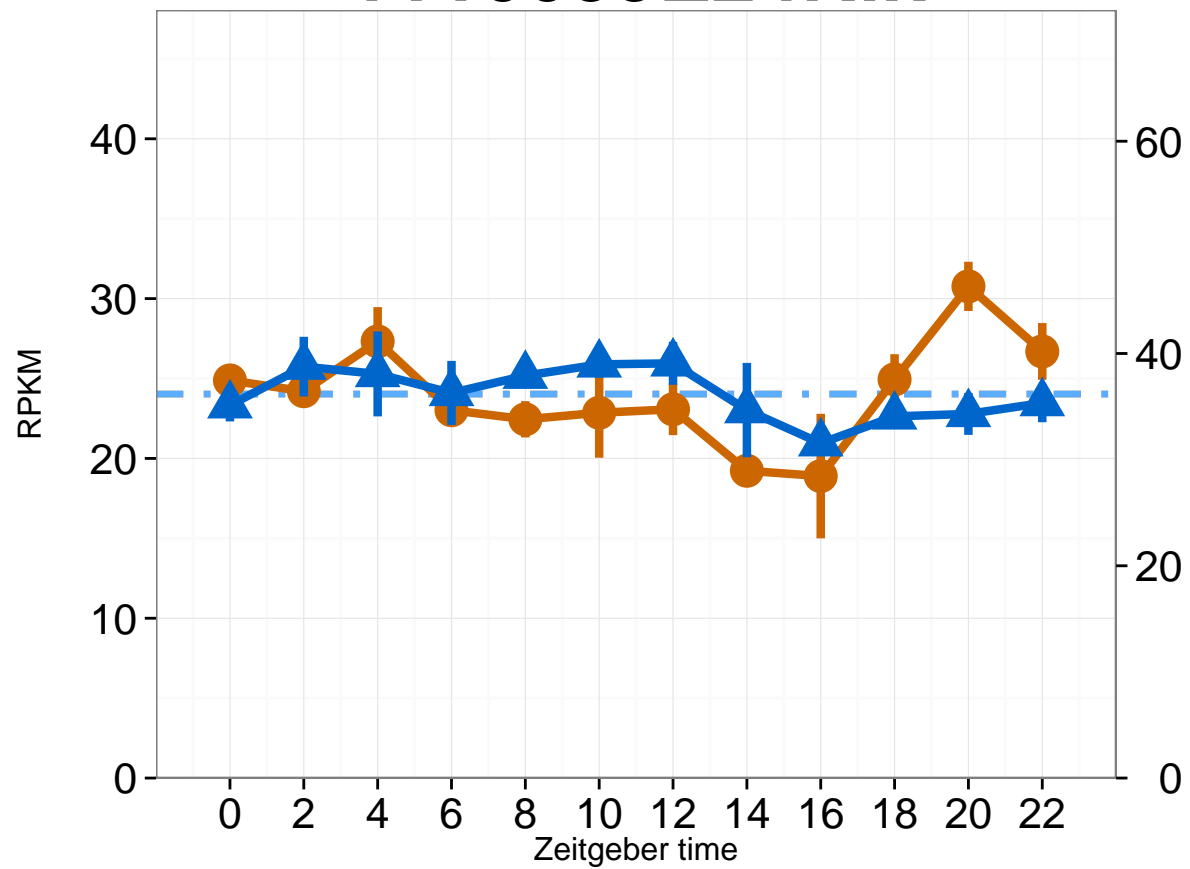

# 1110059E24Rik

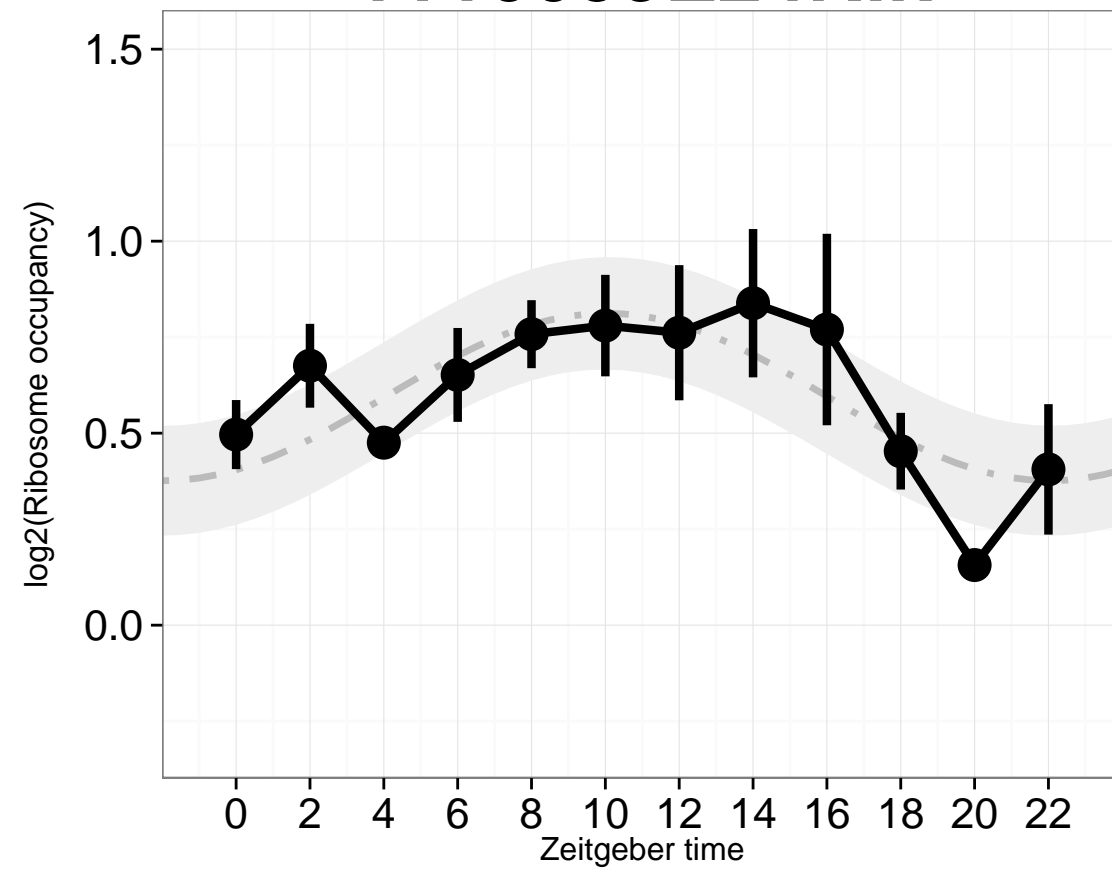

Supplement: Supplementary file 6 — Transcriptome-wide kidney RPF (blue) and RNA (orange) levels in the left panels (with “error bars” connecting the two replicates of each timepoint) and TE in the right panels. (ZIP 116896 kb) [file 13059_2017_1222_MOESM6_ESM.zip › Supp_Dataset_S1/A_RNA_non_rhythmic_RPF_non_rhythmic/1110059E24Rik_kidney_set_A.pdf]

# 1110065P20Rik

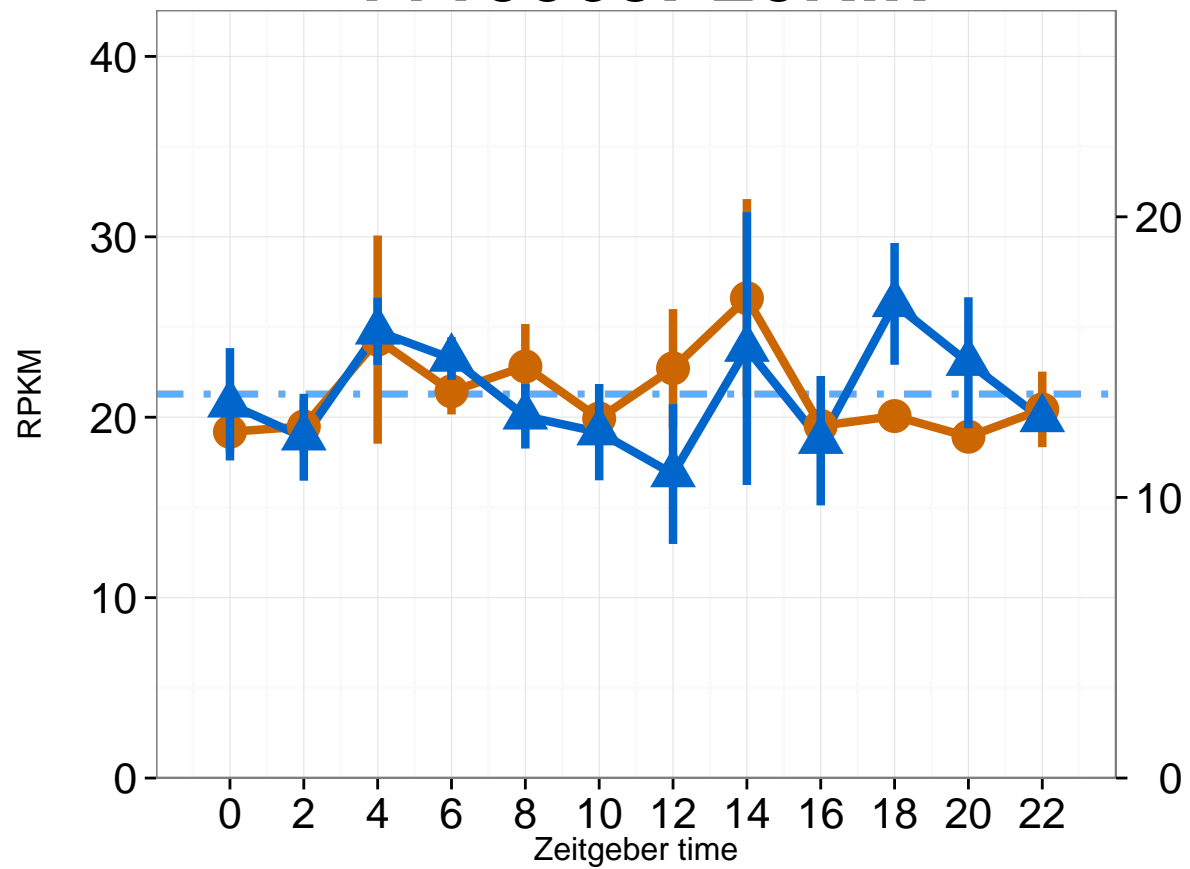

# 1110065P20Rik

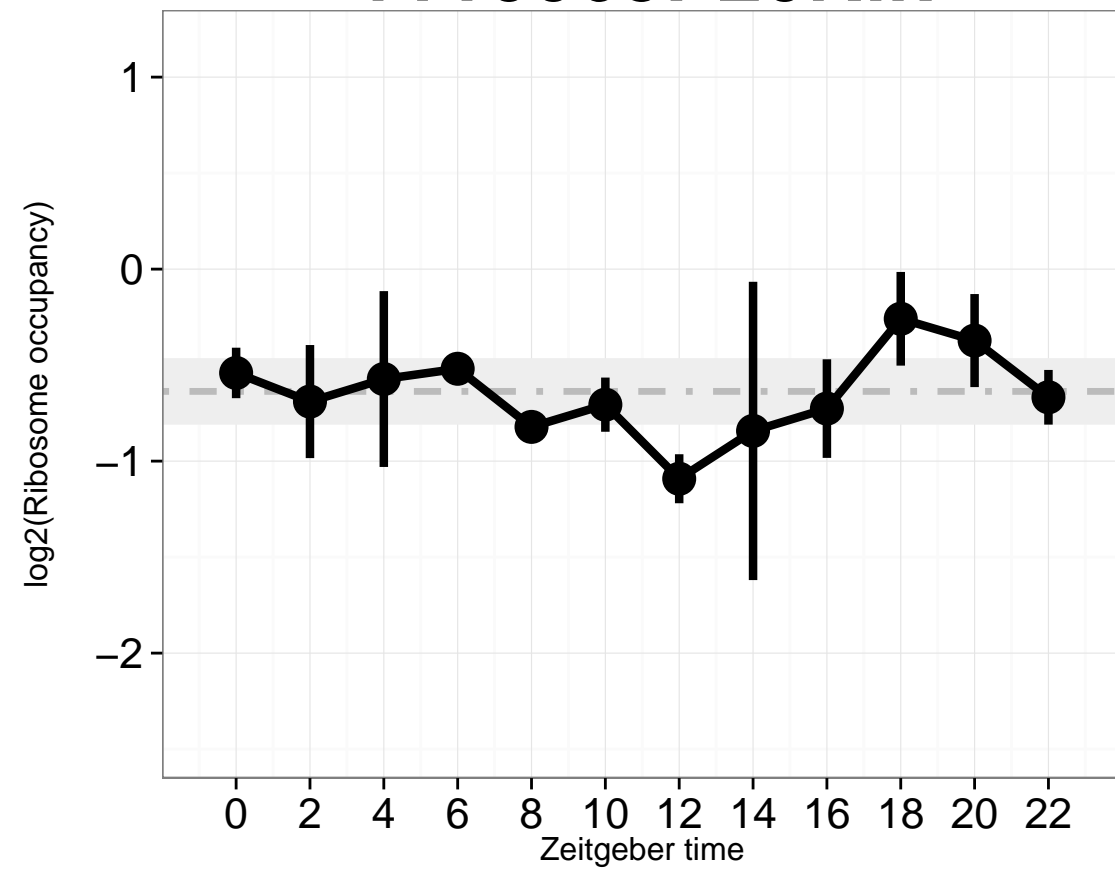

Supplement: Supplementary file 6 — Transcriptome-wide kidney RPF (blue) and RNA (orange) levels in the left panels (with “error bars” connecting the two replicates of each timepoint) and TE in the right panels. (ZIP 116896 kb) [file 13059_2017_1222_MOESM6_ESM.zip › Supp_Dataset_S1/A_RNA_non_rhythmic_RPF_non_rhythmic/1110065P20Rik_kidney_set_A.pdf]

# 1190002N15Rik

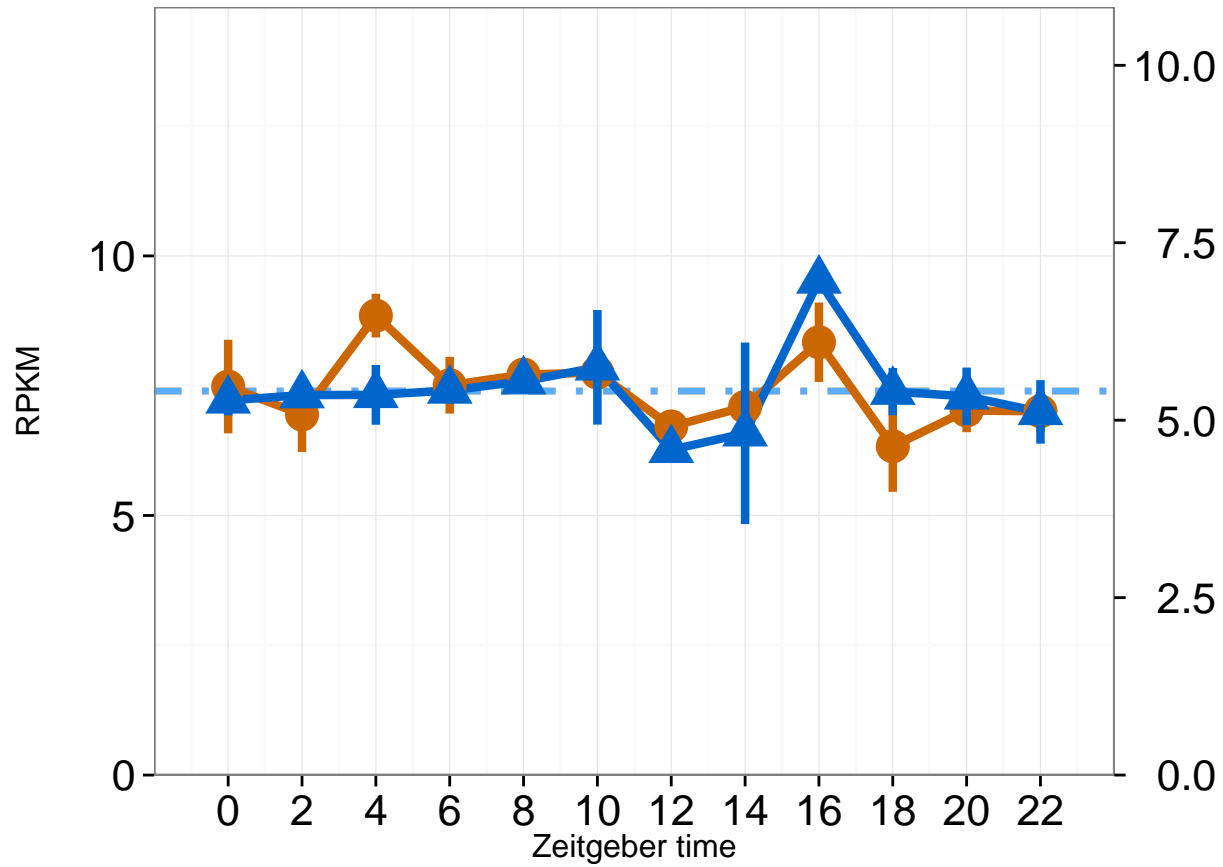

# 1190002N15Rik

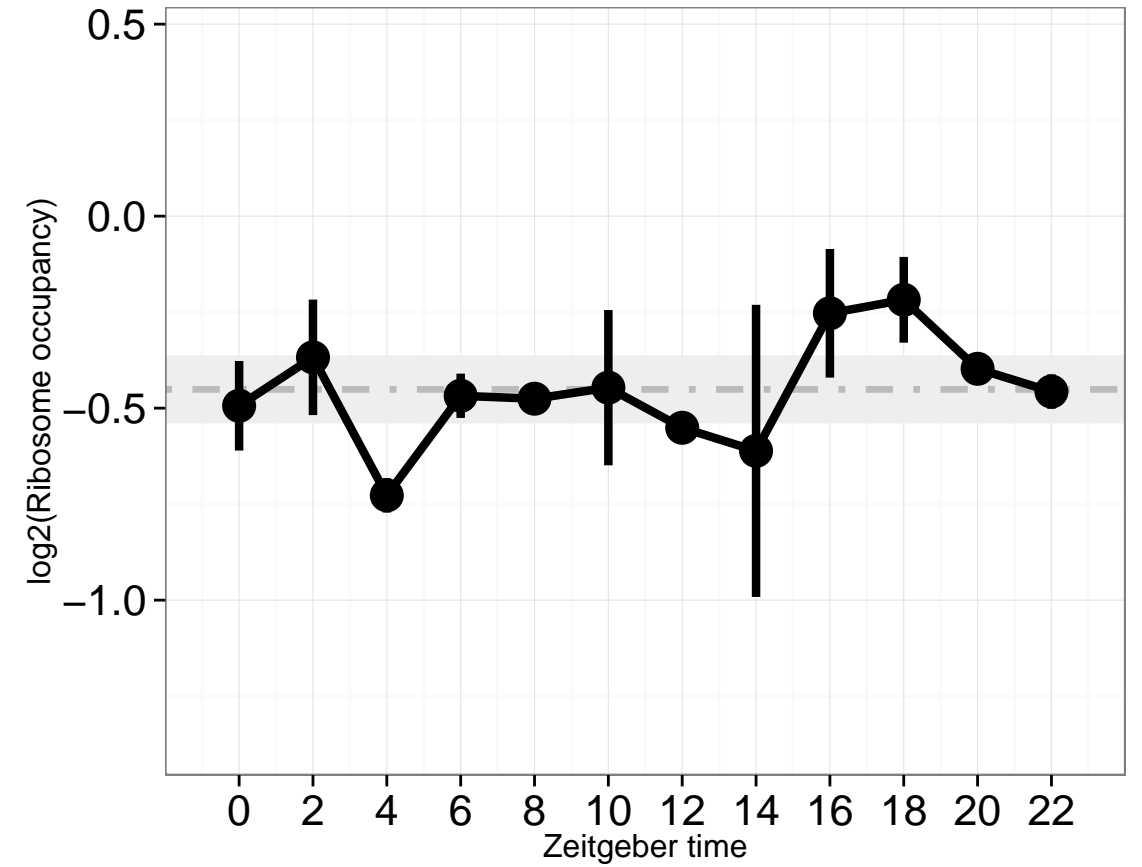

Supplement: Supplementary file 6 — Transcriptome-wide kidney RPF (blue) and RNA (orange) levels in the left panels (with “error bars” connecting the two replicates of each timepoint) and TE in the right panels. (ZIP 116896 kb) [file 13059_2017_1222_MOESM6_ESM.zip › Supp_Dataset_S1/A_RNA_non_rhythmic_RPF_non_rhythmic/1190002N15Rik_kidney_set_A.pdf]

# 1190007I07Rik

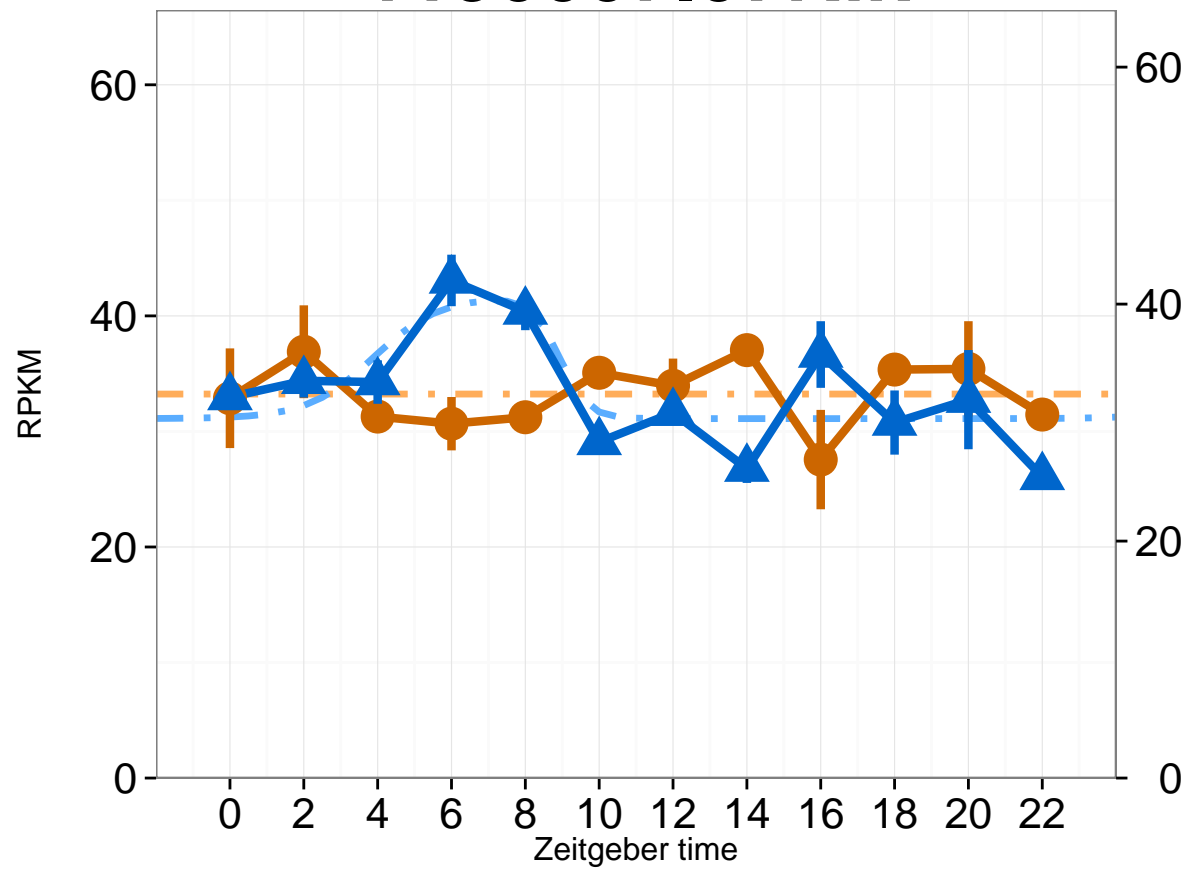

# 1190007I07Rik

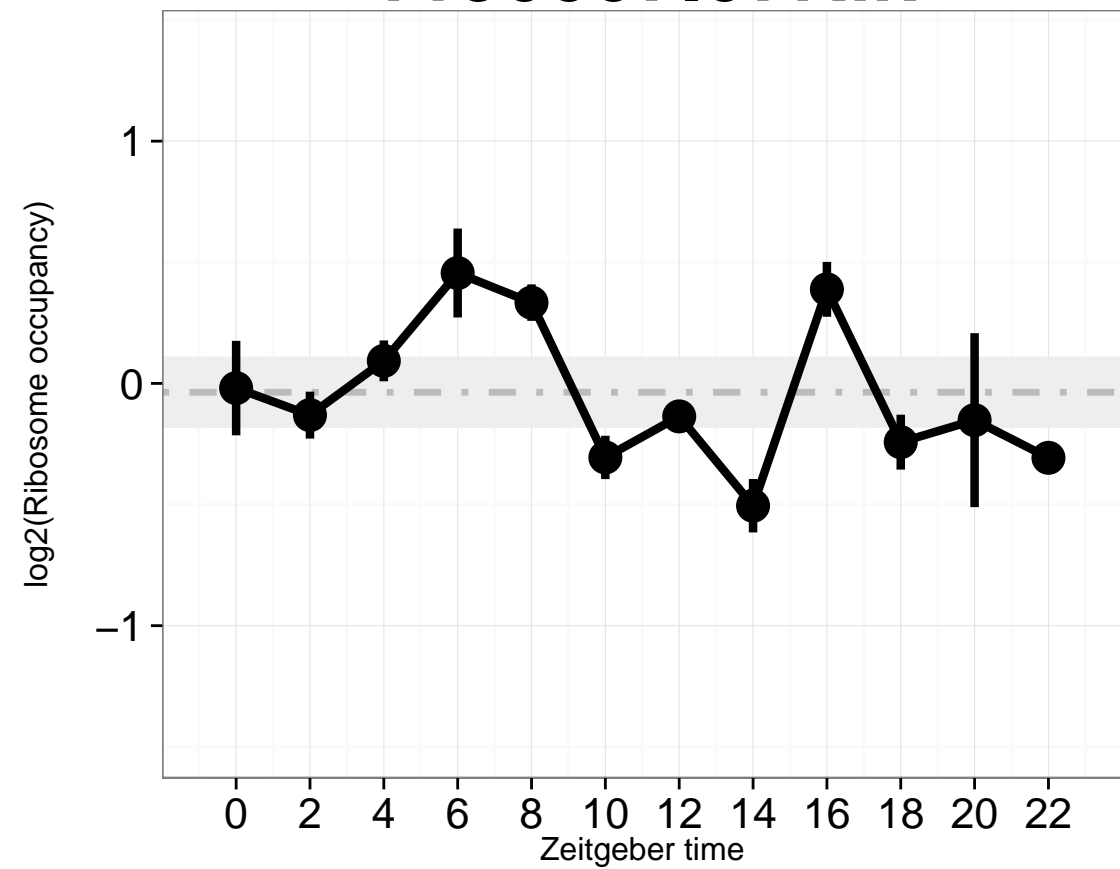

Supplement: Supplementary file 6 — Transcriptome-wide kidney RPF (blue) and RNA (orange) levels in the left panels (with “error bars” connecting the two replicates of each timepoint) and TE in the right panels. (ZIP 116896 kb) [file 13059_2017_1222_MOESM6_ESM.zip › Supp_Dataset_S1/A_RNA_non_rhythmic_RPF_non_rhythmic/1190007I07Rik_kidney_set_A.pdf]

# 1200014J11Rik

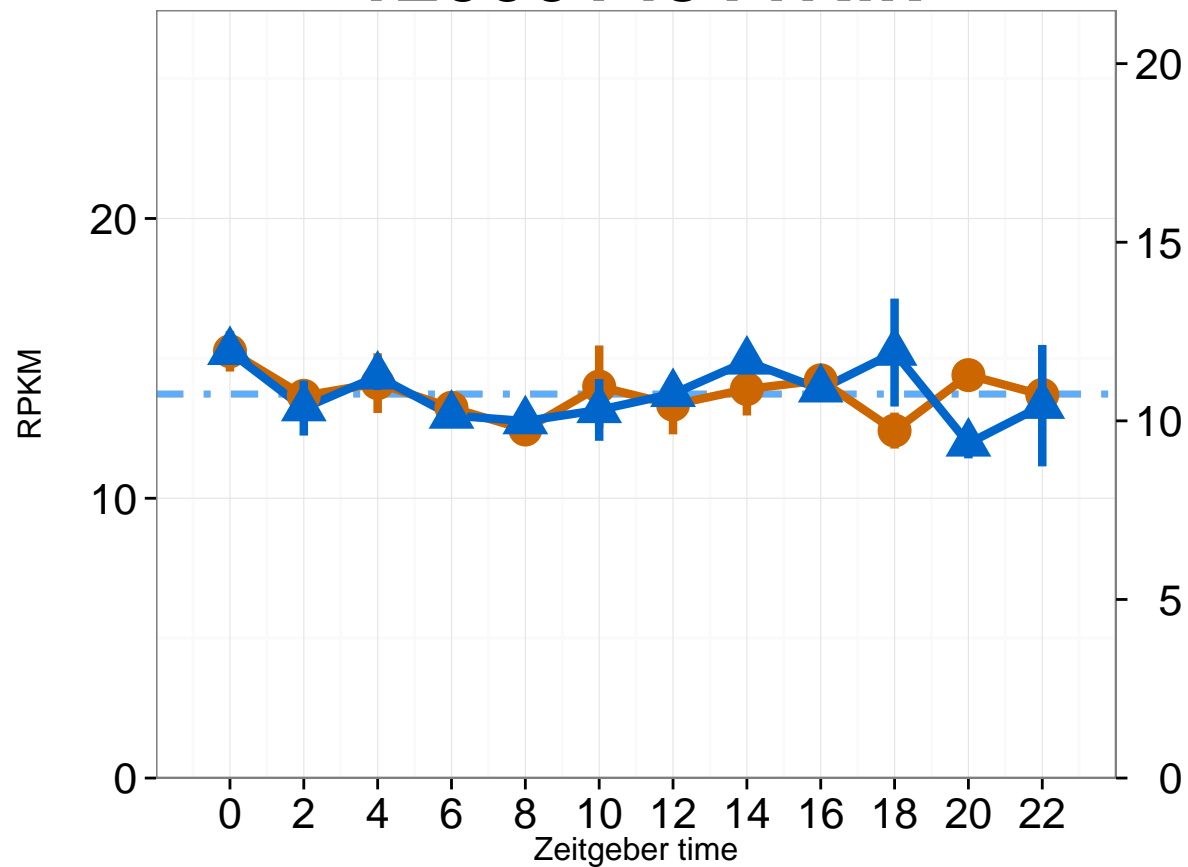

# 1200014J11Rik

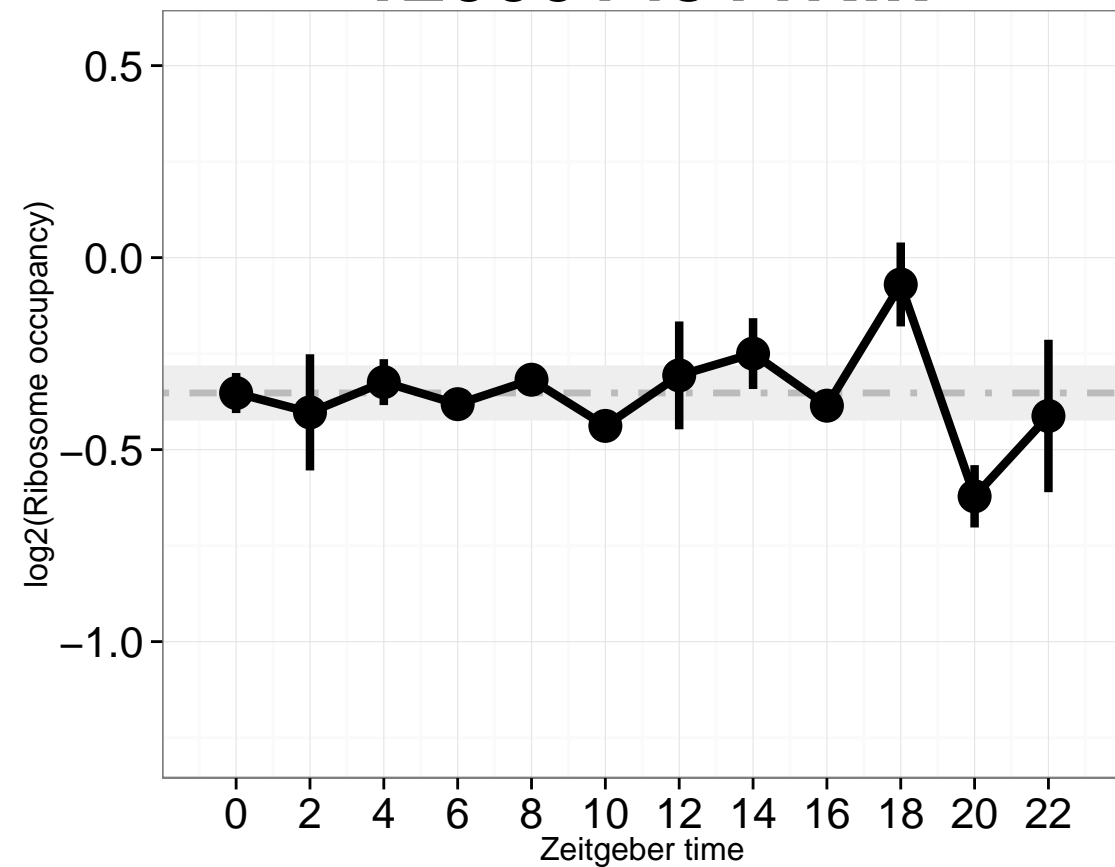

Supplement: Supplementary file 6 — Transcriptome-wide kidney RPF (blue) and RNA (orange) levels in the left panels (with “error bars” connecting the two replicates of each timepoint) and TE in the right panels. (ZIP 116896 kb) [file 13059_2017_1222_MOESM6_ESM.zip › Supp_Dataset_S1/A_RNA_non_rhythmic_RPF_non_rhythmic/1200014J11Rik_kidney_set_A.pdf]

1500012F01Rik

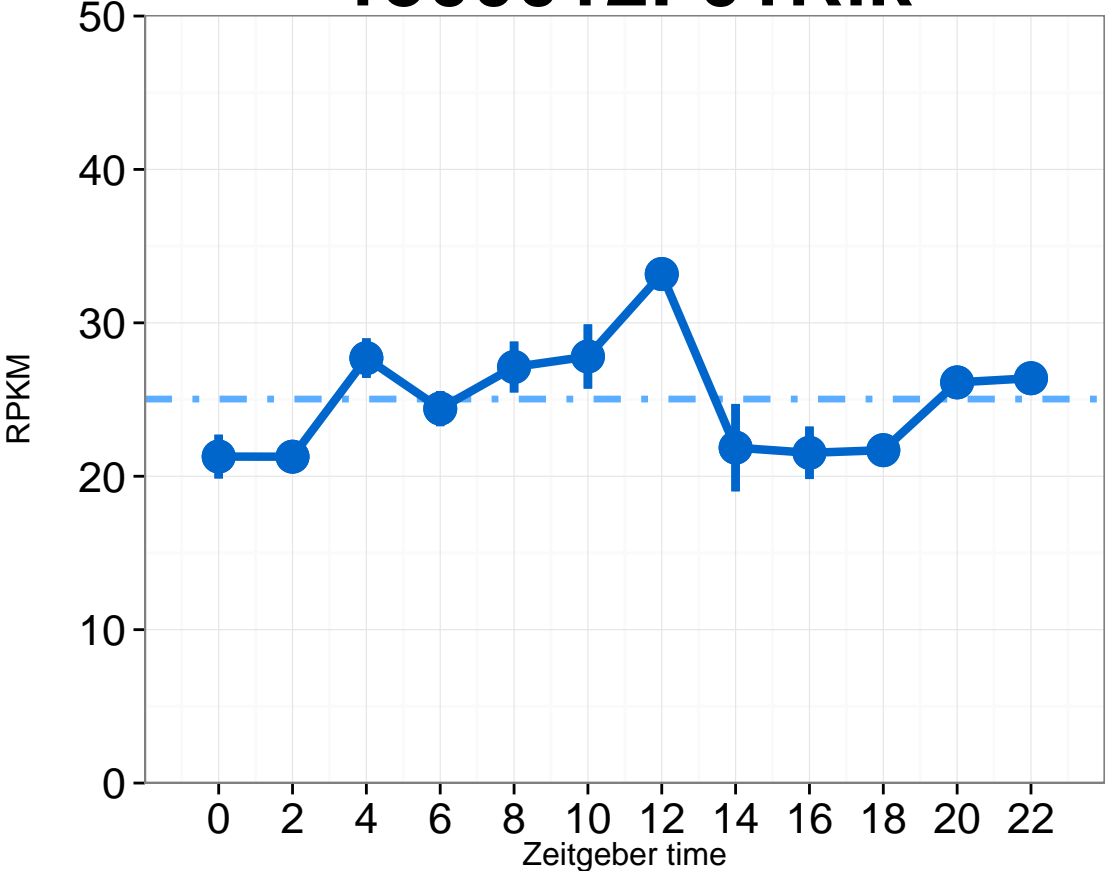

1500012F01Rik log2(Ribosome occupancy)

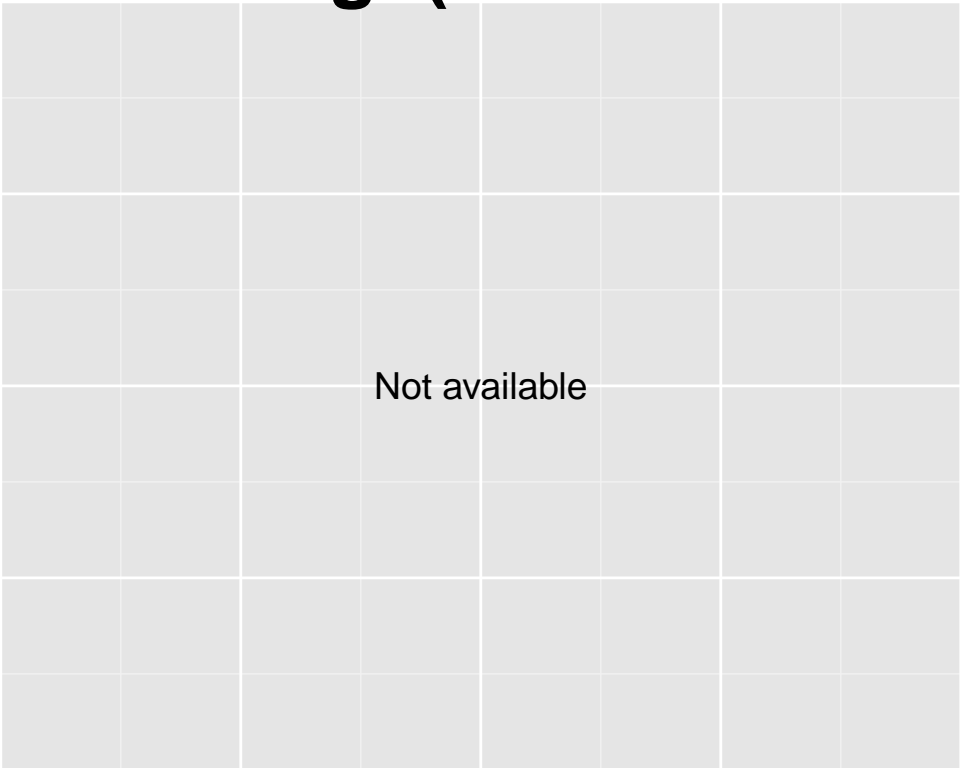

Supplement: Supplementary file 6 — Transcriptome-wide kidney RPF (blue) and RNA (orange) levels in the left panels (with “error bars” connecting the two replicates of each timepoint) and TE in the right panels. (ZIP 116896 kb) [file 13059_2017_1222_MOESM6_ESM.zip › Supp_Dataset_S1/A_RNA_non_rhythmic_RPF_non_rhythmic/1500012F01Rik_kidney_set_A.pdf]

1500015O10Rik TR

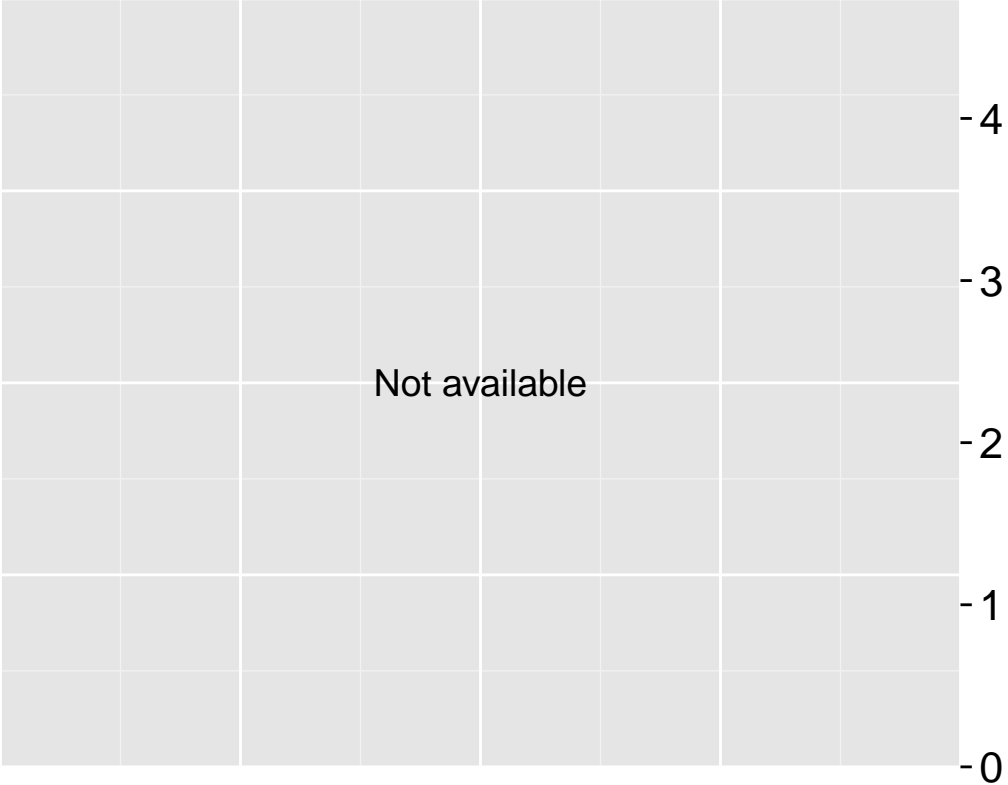

1500015O10Rik log2(Ribosome occup

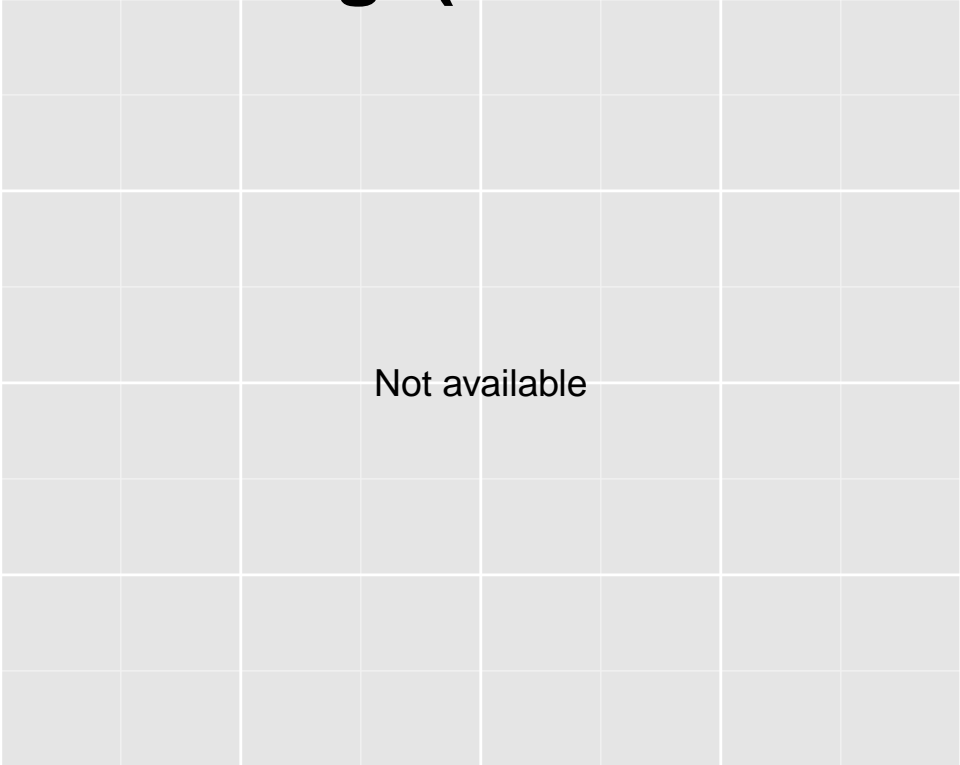

Supplement: Supplementary file 6 — Transcriptome-wide kidney RPF (blue) and RNA (orange) levels in the left panels (with “error bars” connecting the two replicates of each timepoint) and TE in the right panels. (ZIP 116896 kb) [file 13059_2017_1222_MOESM6_ESM.zip › Supp_Dataset_S1/A_RNA_non_rhythmic_RPF_non_rhythmic/1500015O10Rik_kidney_set_A.pdf]

# 1600002H07Rik

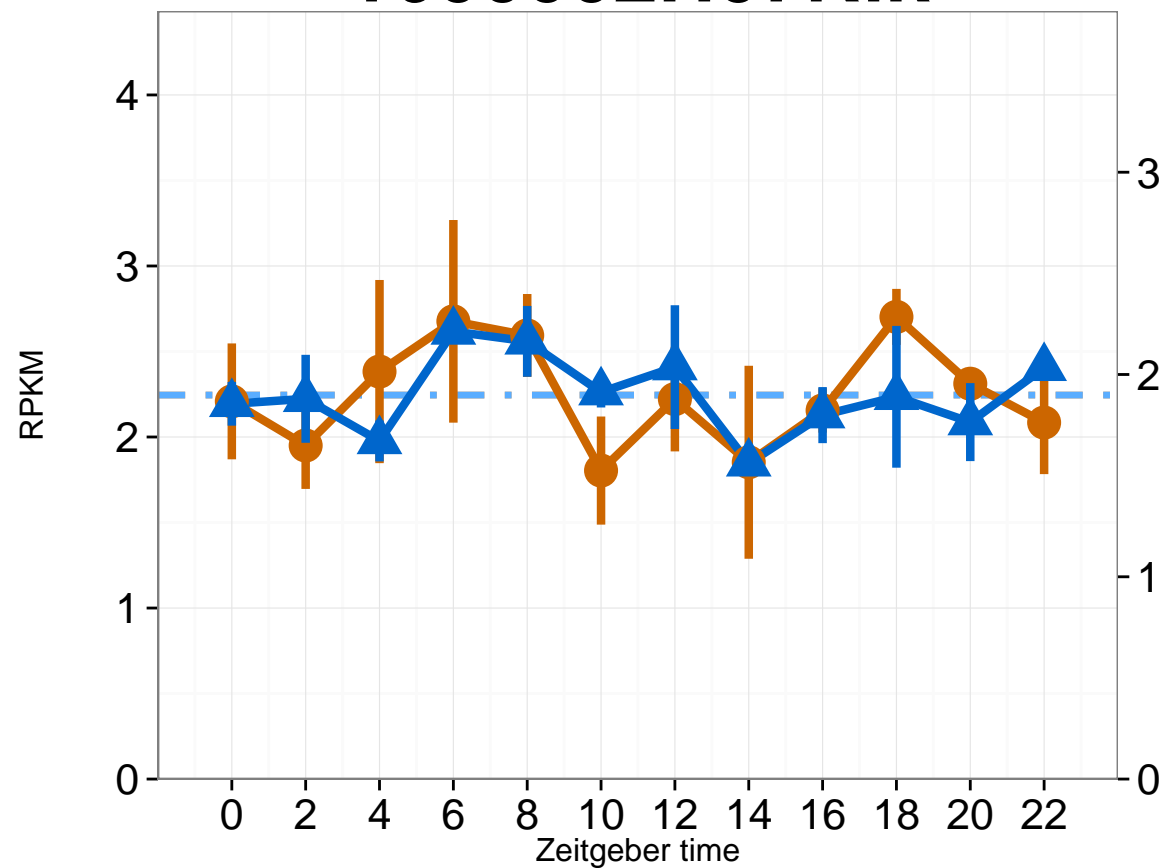

# 1600002H07Rik

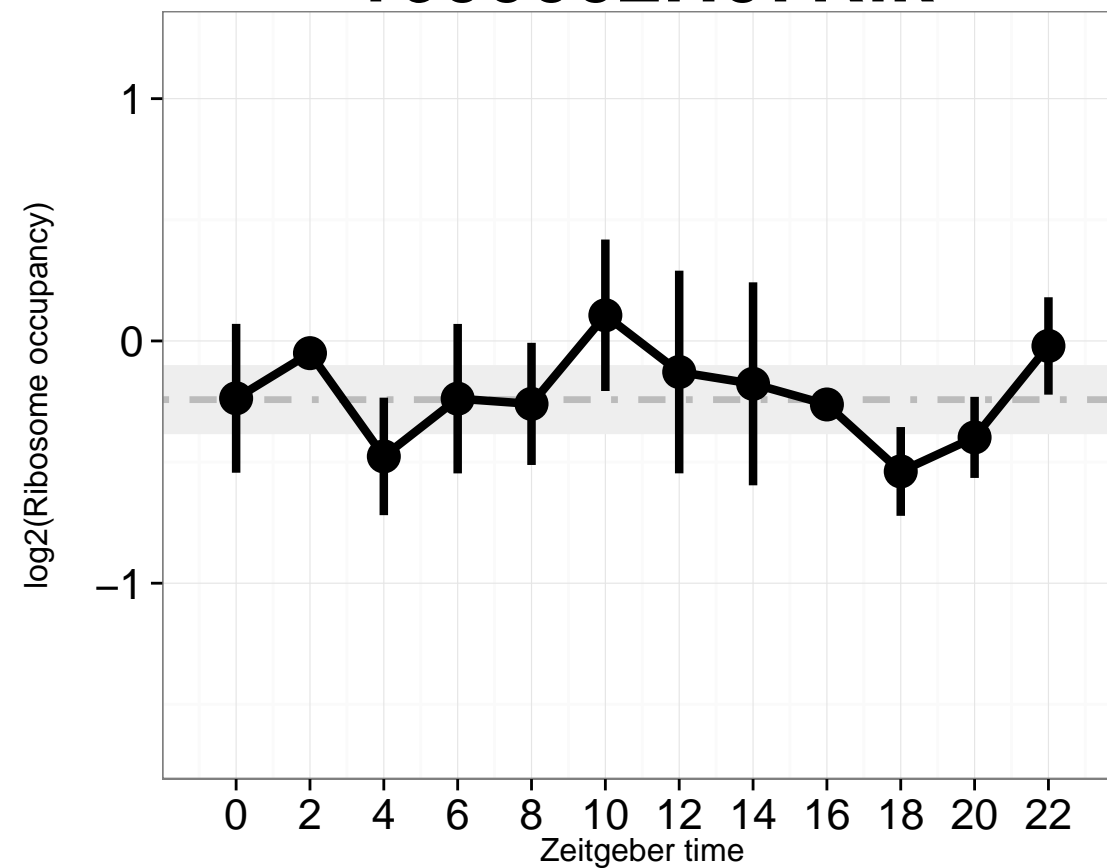

Supplement: Supplementary file 6 — Transcriptome-wide kidney RPF (blue) and RNA (orange) levels in the left panels (with “error bars” connecting the two replicates of each timepoint) and TE in the right panels. (ZIP 116896 kb) [file 13059_2017_1222_MOESM6_ESM.zip › Supp_Dataset_S1/A_RNA_non_rhythmic_RPF_non_rhythmic/1600002H07Rik_kidney_set_A.pdf]

# 1600012H06Rik

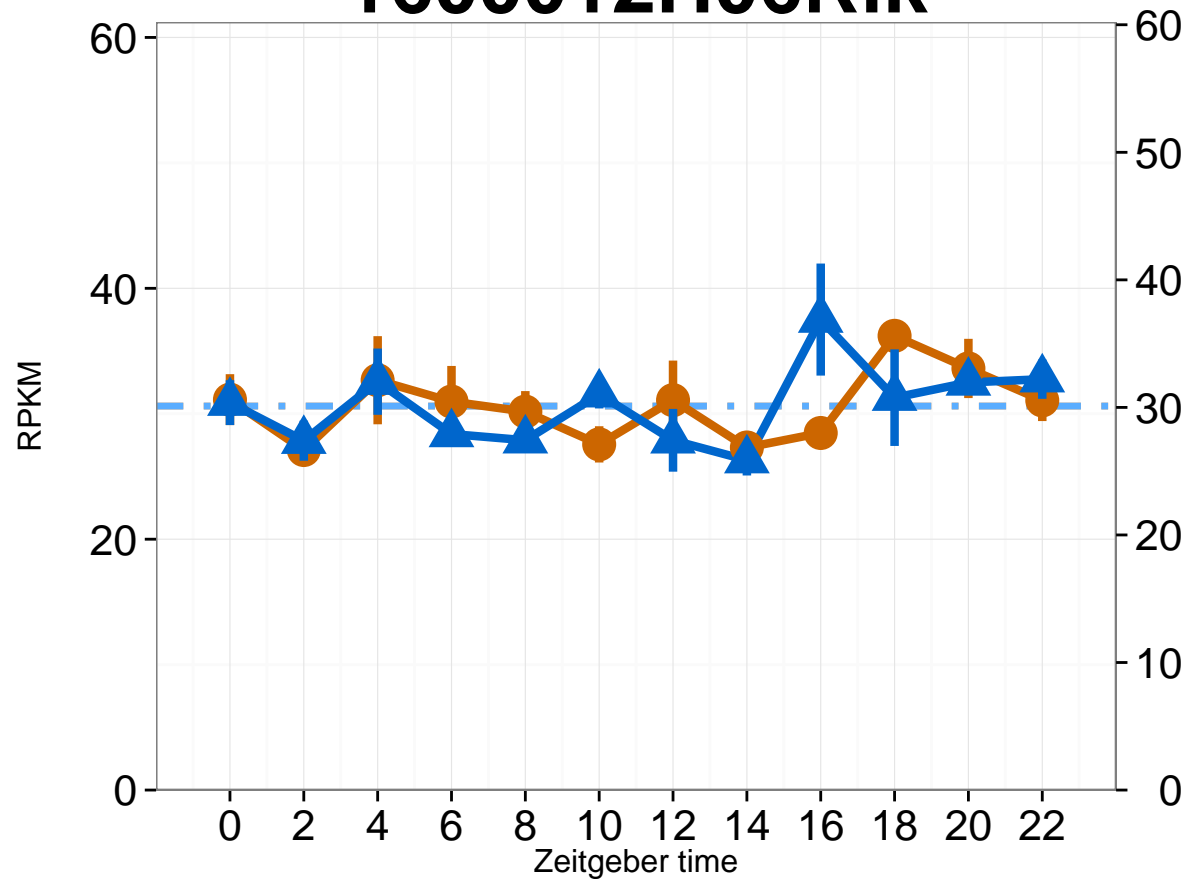

# 1600012H06Rik

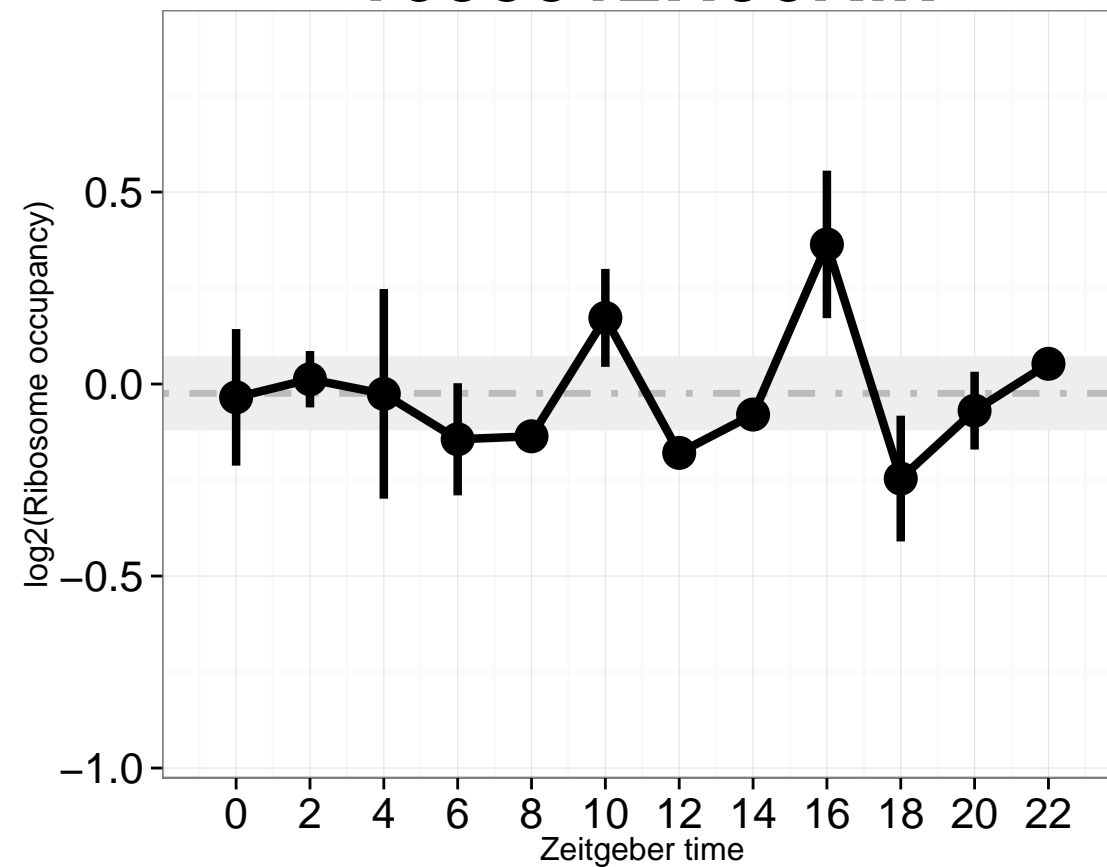

Supplement: Supplementary file 6 — Transcriptome-wide kidney RPF (blue) and RNA (orange) levels in the left panels (with “error bars” connecting the two replicates of each timepoint) and TE in the right panels. (ZIP 116896 kb) [file 13059_2017_1222_MOESM6_ESM.zip › Supp_Dataset_S1/A_RNA_non_rhythmic_RPF_non_rhythmic/1600012H06Rik_kidney_set_A.pdf]

# 1600029D21Rik

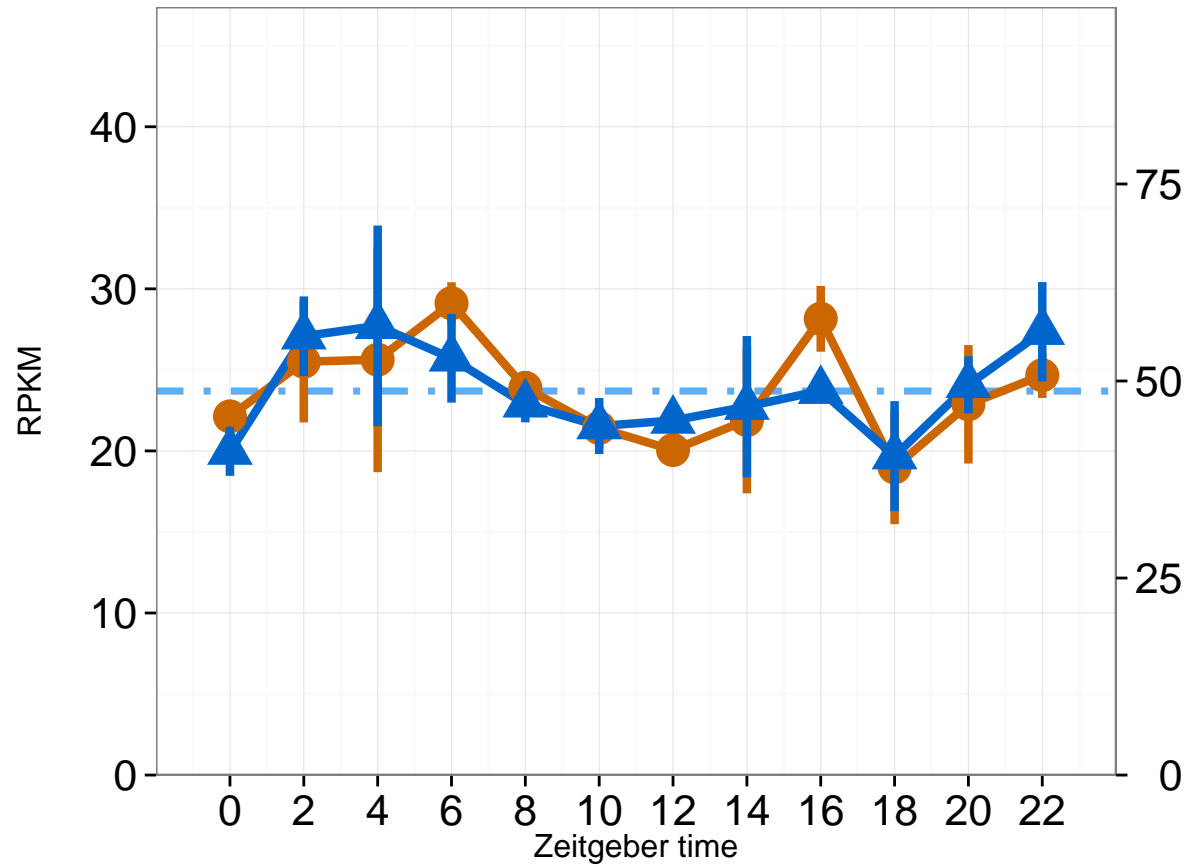

# 1600029D21Rik

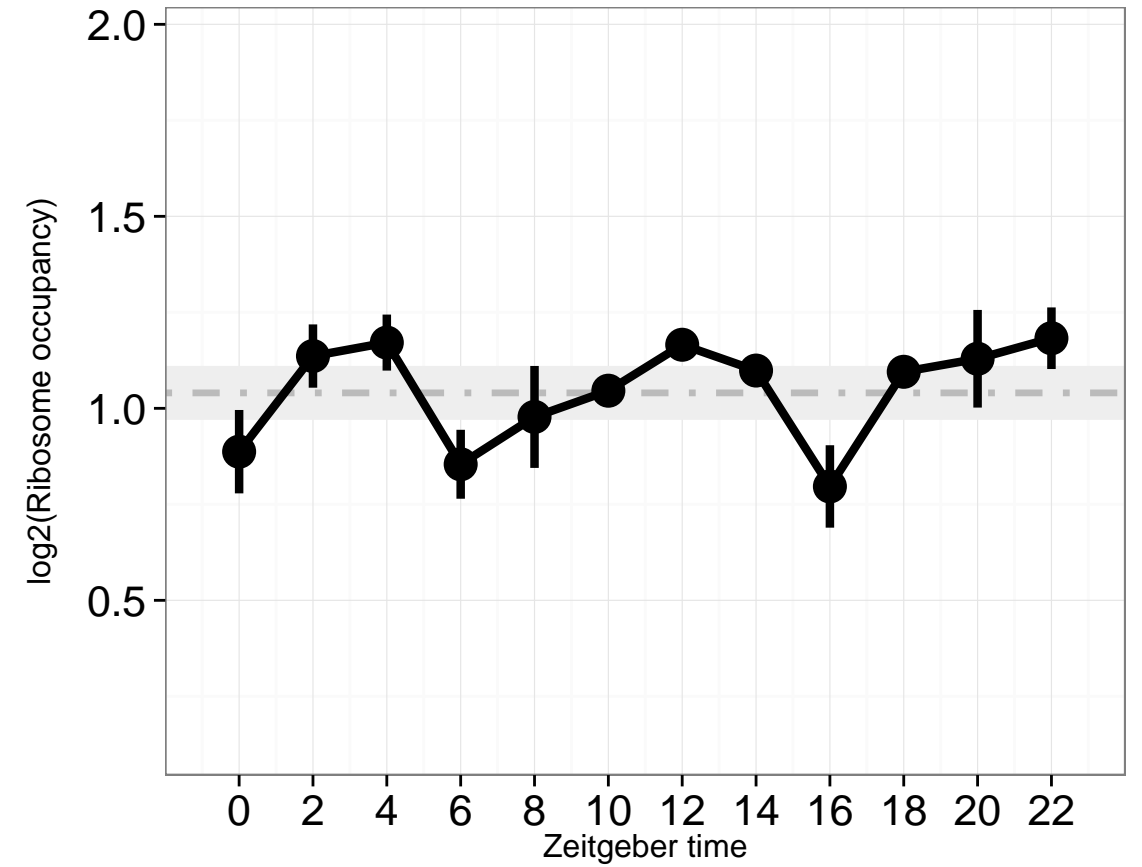

Supplement: Supplementary file 6 — Transcriptome-wide kidney RPF (blue) and RNA (orange) levels in the left panels (with “error bars” connecting the two replicates of each timepoint) and TE in the right panels. (ZIP 116896 kb) [file 13059_2017_1222_MOESM6_ESM.zip › Supp_Dataset_S1/A_RNA_non_rhythmic_RPF_non_rhythmic/1600029D21Rik_kidney_set_A.pdf]

# 1700003E16Rik

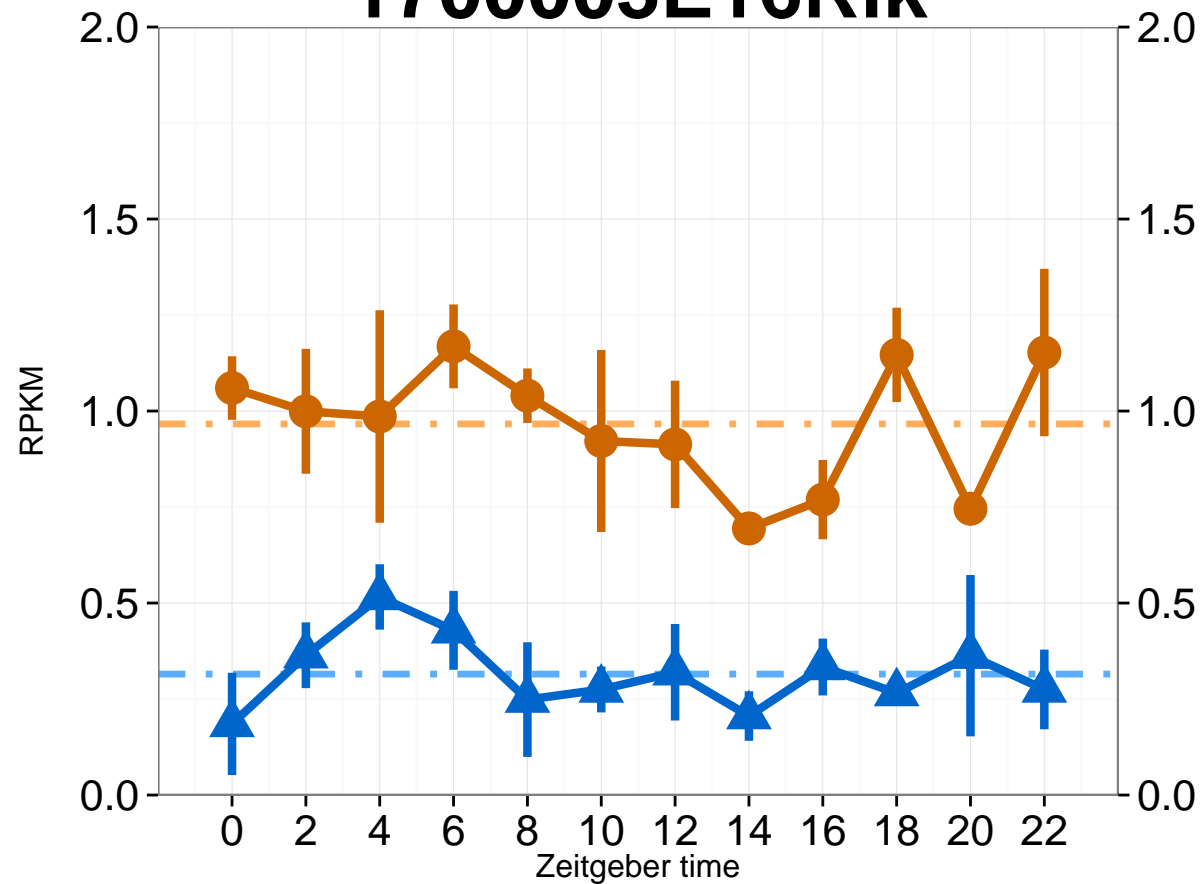

# 1700003E16Rik

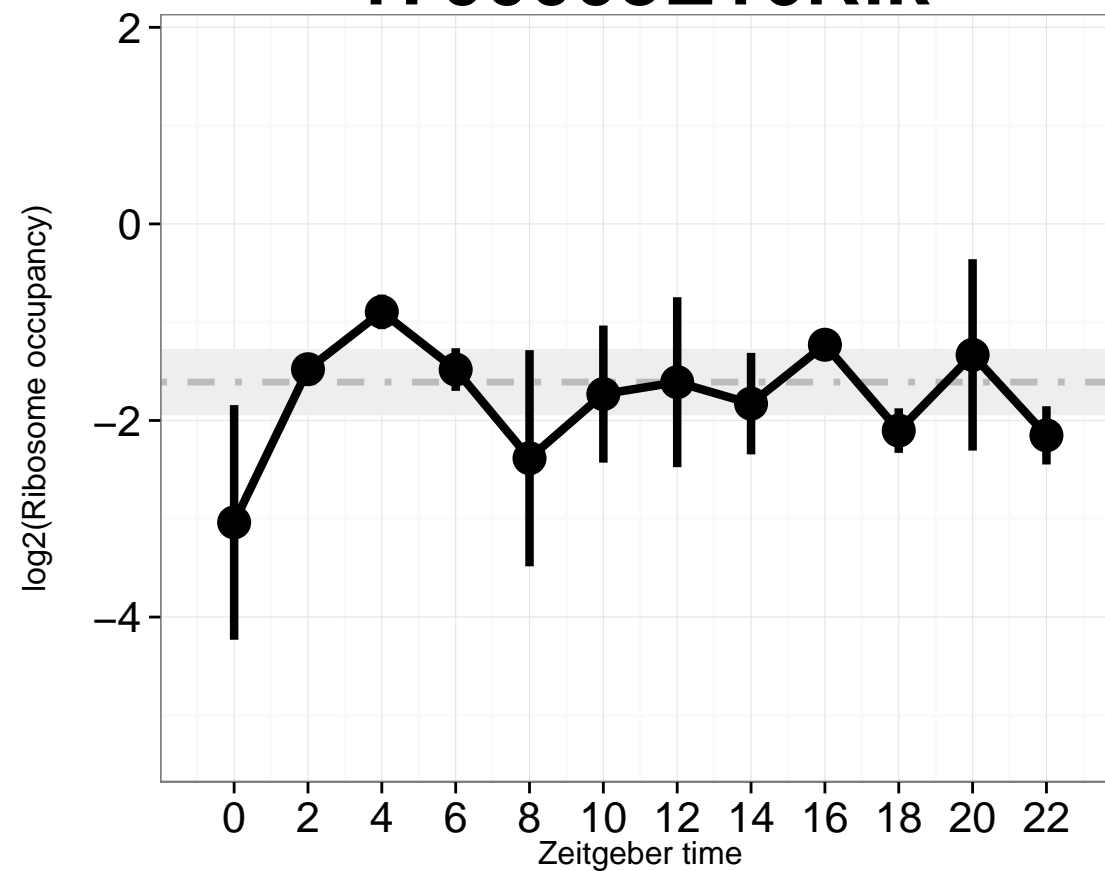

Supplement: Supplementary file 6 — Transcriptome-wide kidney RPF (blue) and RNA (orange) levels in the left panels (with “error bars” connecting the two replicates of each timepoint) and TE in the right panels. (ZIP 116896 kb) [file 13059_2017_1222_MOESM6_ESM.zip › Supp_Dataset_S1/A_RNA_non_rhythmic_RPF_non_rhythmic/1700003E16Rik_kidney_set_A.pdf]

**1700009P17Rik TR**

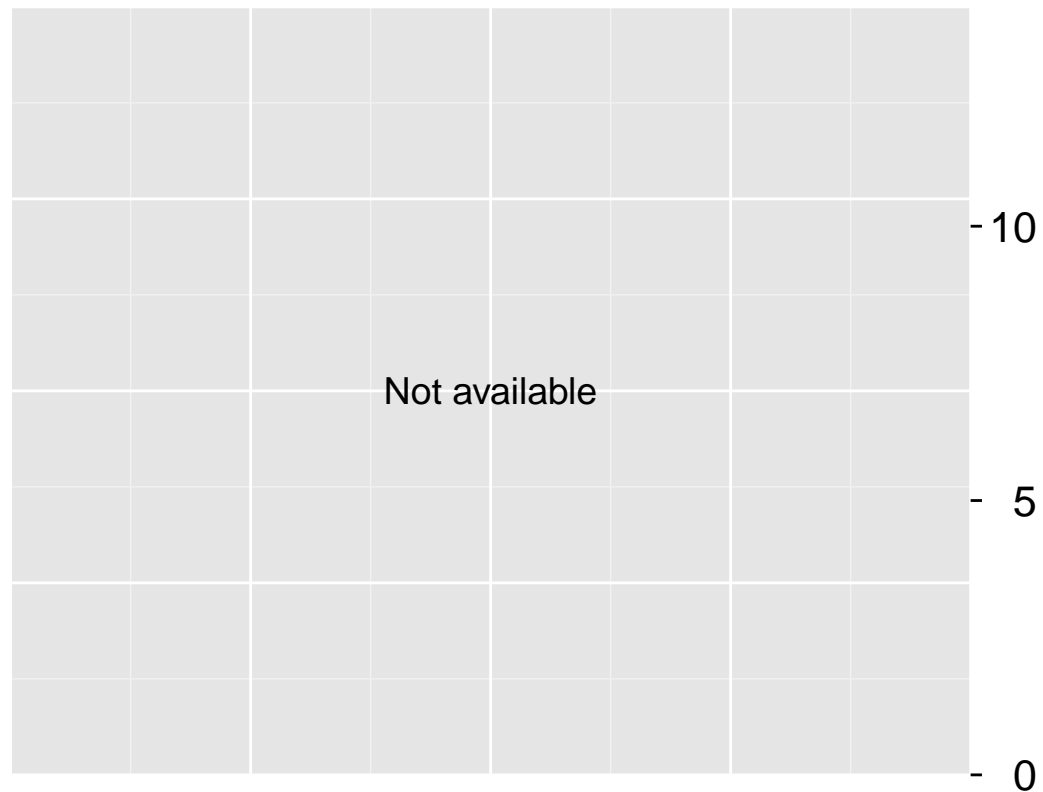

**1700009P17Rik log2(Ribosome occup**

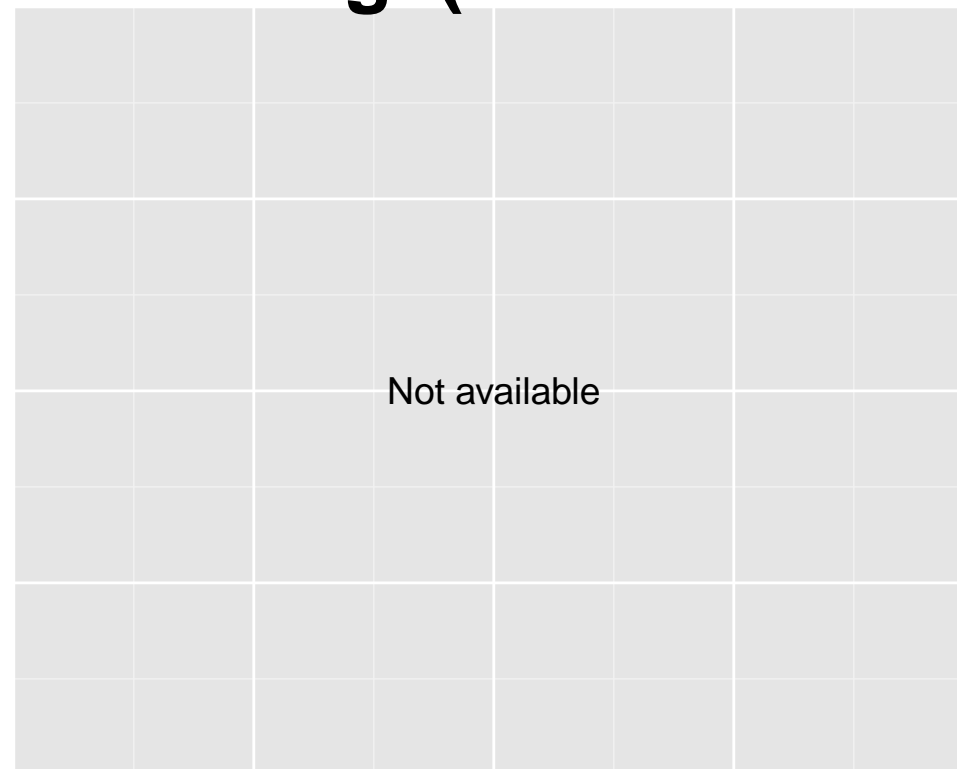

Supplement: Supplementary file 6 — Transcriptome-wide kidney RPF (blue) and RNA (orange) levels in the left panels (with “error bars” connecting the two replicates of each timepoint) and TE in the right panels. (ZIP 116896 kb) [file 13059_2017_1222_MOESM6_ESM.zip › Supp_Dataset_S1/A_RNA_non_rhythmic_RPF_non_rhythmic/1700009P17Rik_kidney_set_A.pdf]

# 1700011H14Rik

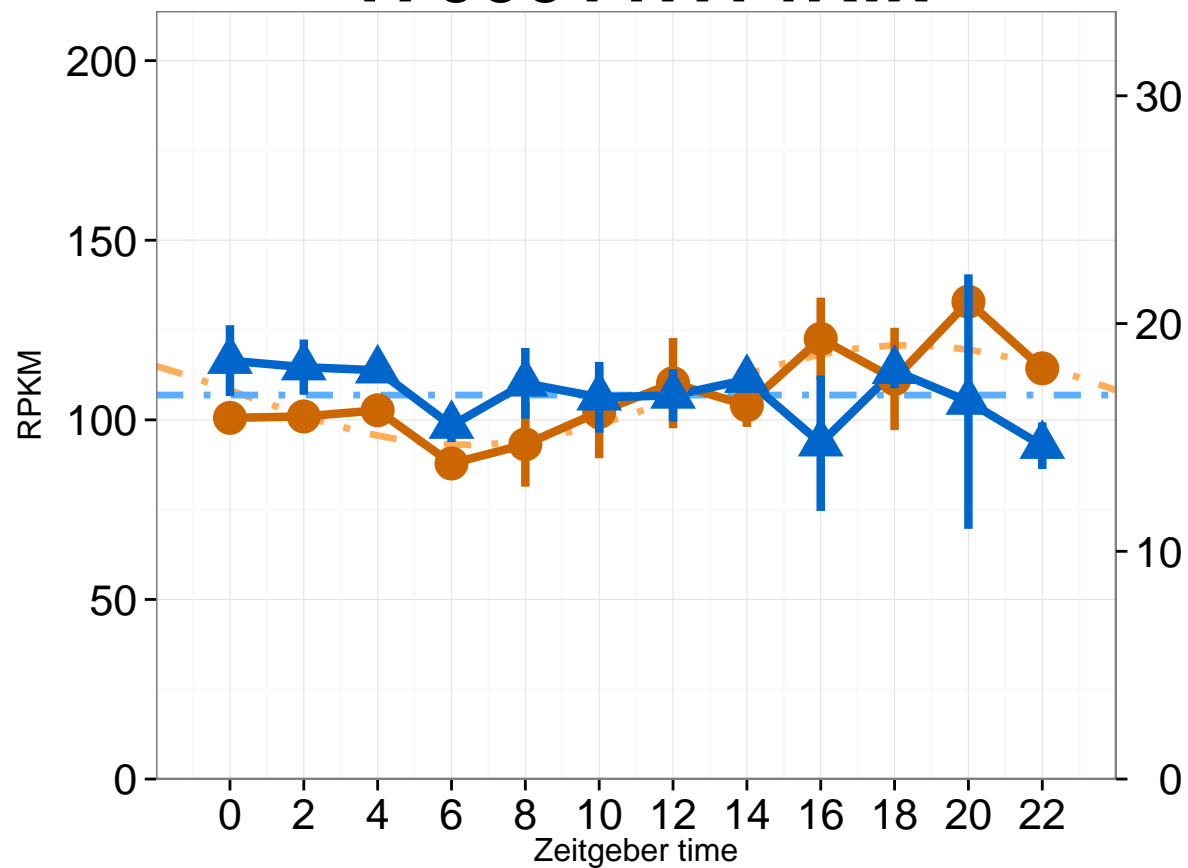

# 1700011H14Rik

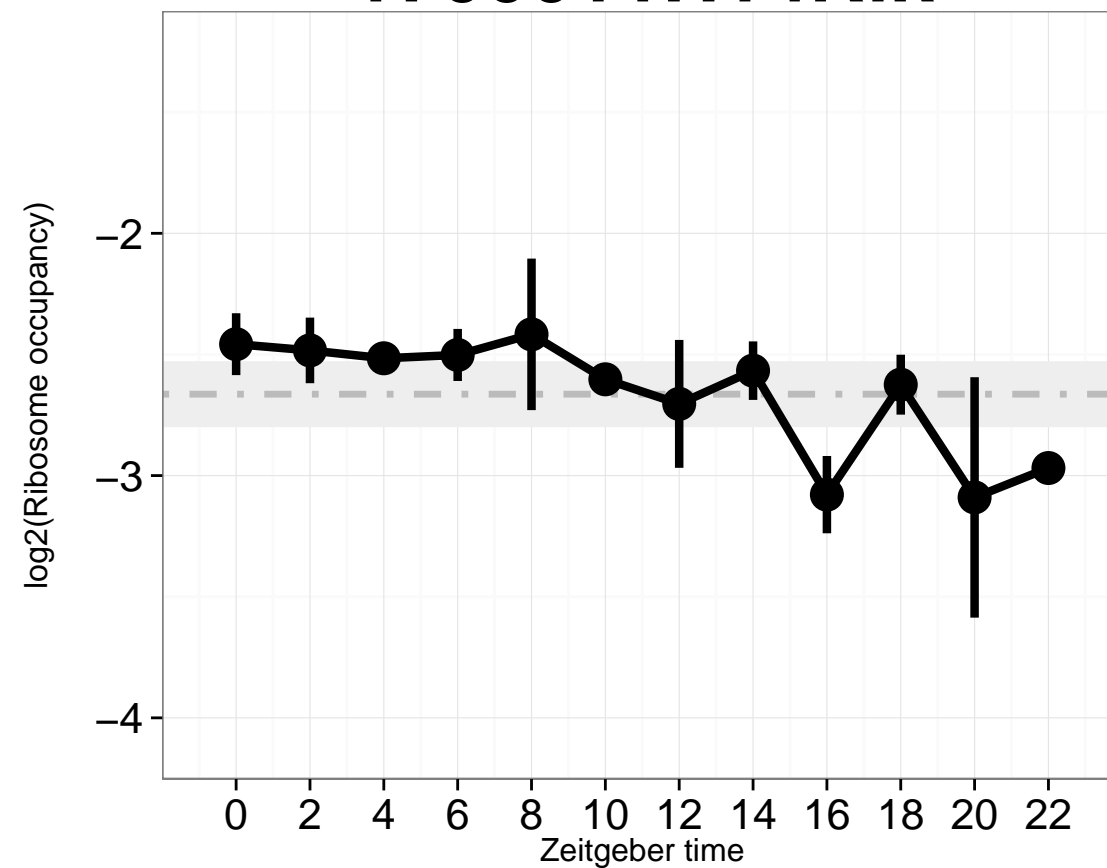

Supplement: Supplementary file 6 — Transcriptome-wide kidney RPF (blue) and RNA (orange) levels in the left panels (with “error bars” connecting the two replicates of each timepoint) and TE in the right panels. (ZIP 116896 kb) [file 13059_2017_1222_MOESM6_ESM.zip › Supp_Dataset_S1/A_RNA_non_rhythmic_RPF_non_rhythmic/1700011H14Rik_kidney_set_A.pdf]

# 1700021F05Rik

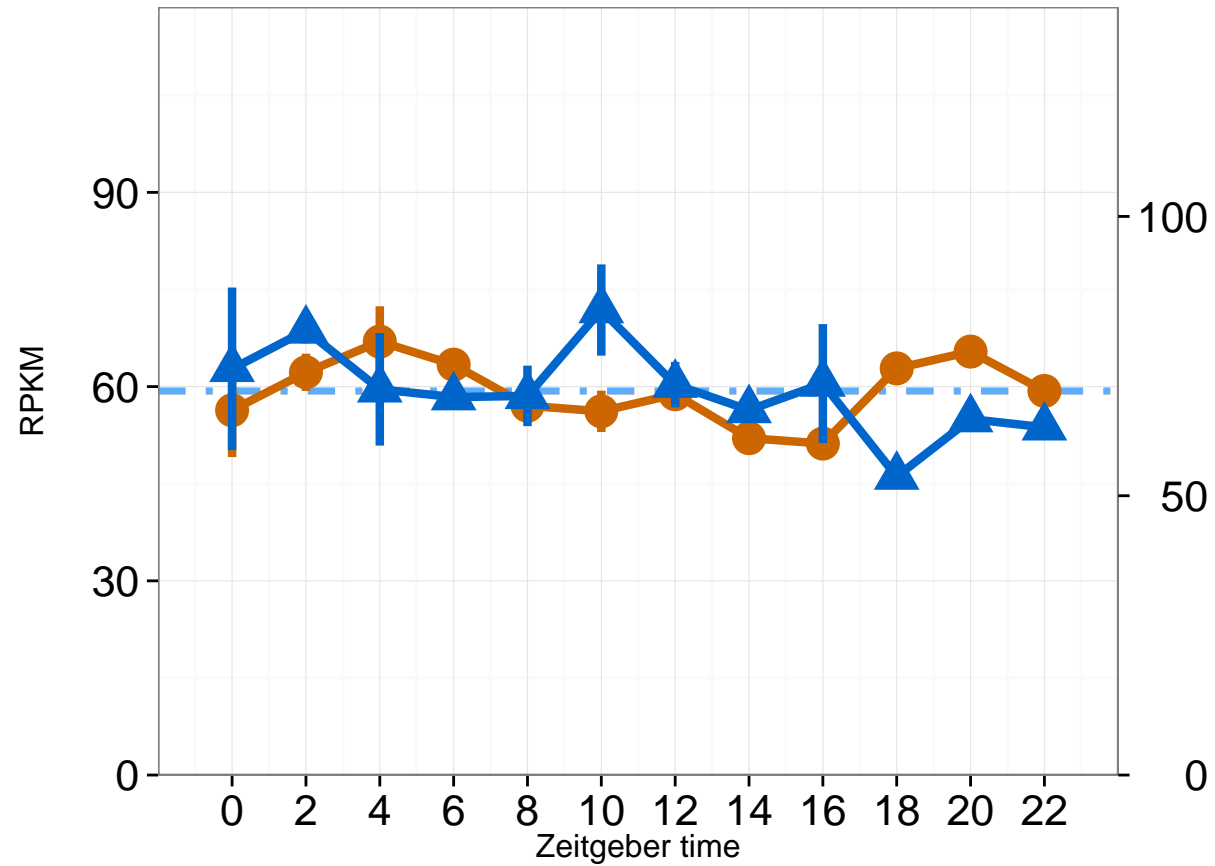

# 1700021F05Rik

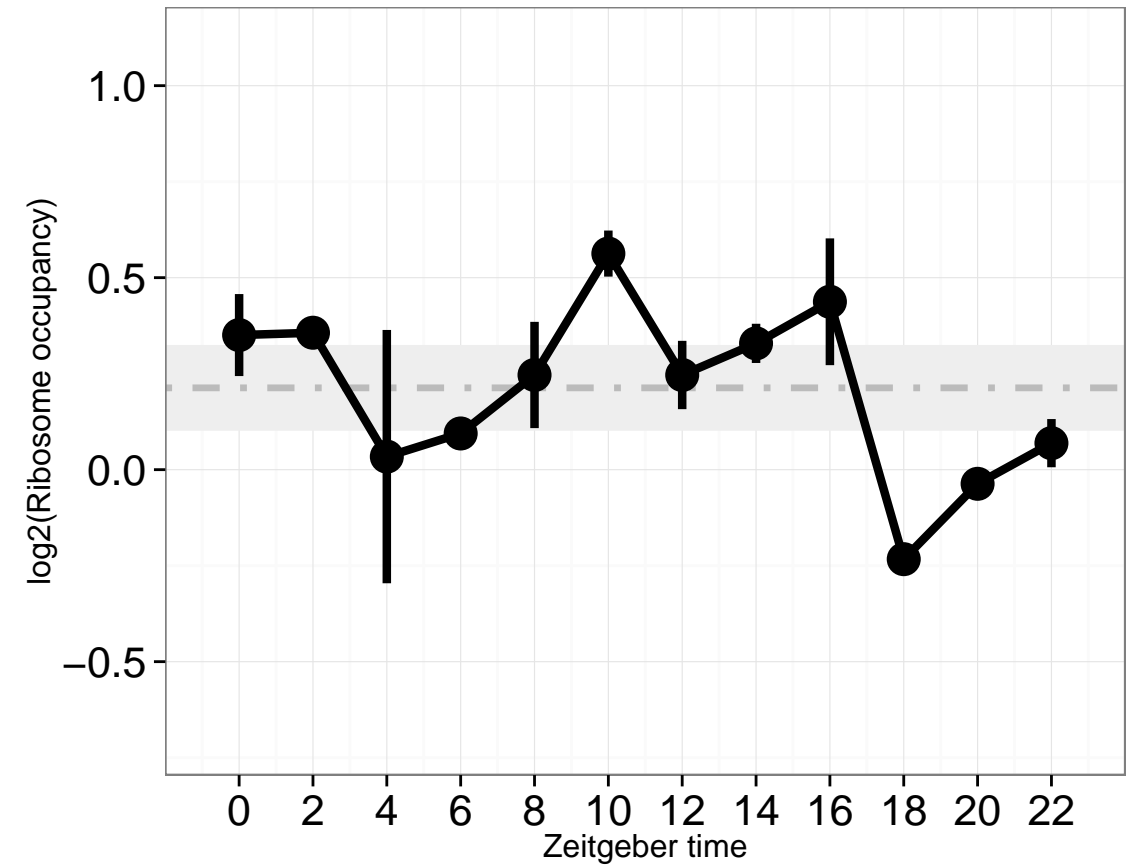

Supplement: Supplementary file 6 — Transcriptome-wide kidney RPF (blue) and RNA (orange) levels in the left panels (with “error bars” connecting the two replicates of each timepoint) and TE in the right panels. (ZIP 116896 kb) [file 13059_2017_1222_MOESM6_ESM.zip › Supp_Dataset_S1/A_RNA_non_rhythmic_RPF_non_rhythmic/1700021F05Rik_kidney_set_A.pdf]

# 1700021K19Rik

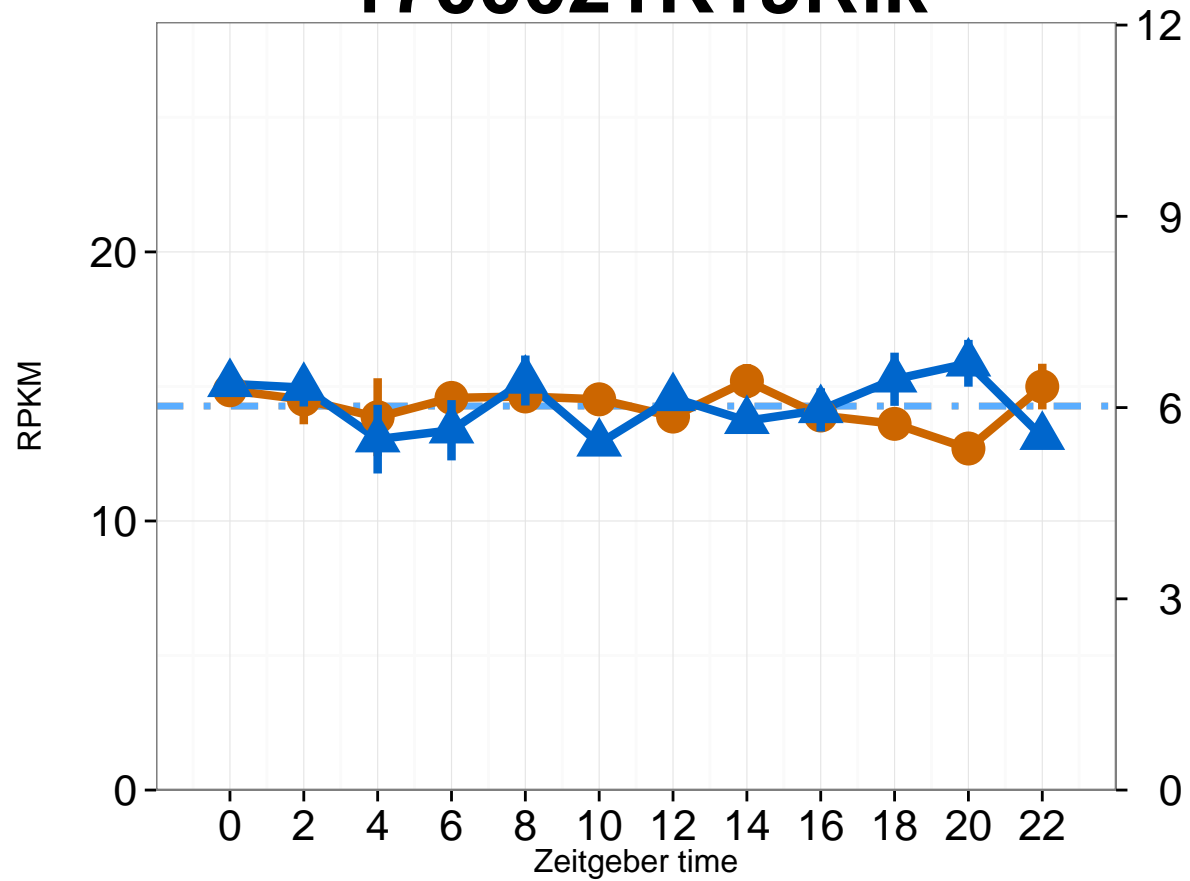

# 1700021K19Rik

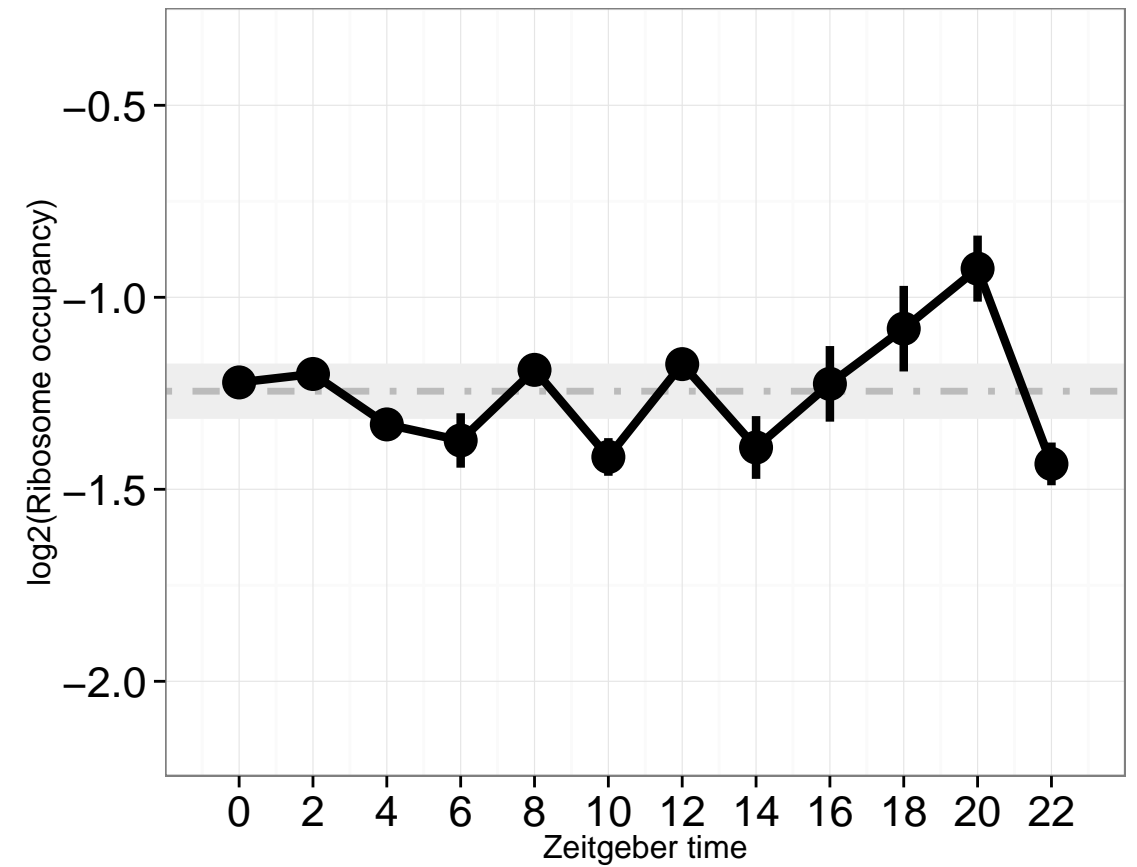

Supplement: Supplementary file 6 — Transcriptome-wide kidney RPF (blue) and RNA (orange) levels in the left panels (with “error bars” connecting the two replicates of each timepoint) and TE in the right panels. (ZIP 116896 kb) [file 13059_2017_1222_MOESM6_ESM.zip › Supp_Dataset_S1/A_RNA_non_rhythmic_RPF_non_rhythmic/1700021K19Rik_kidney_set_A.pdf]

# 1700024P16Rik

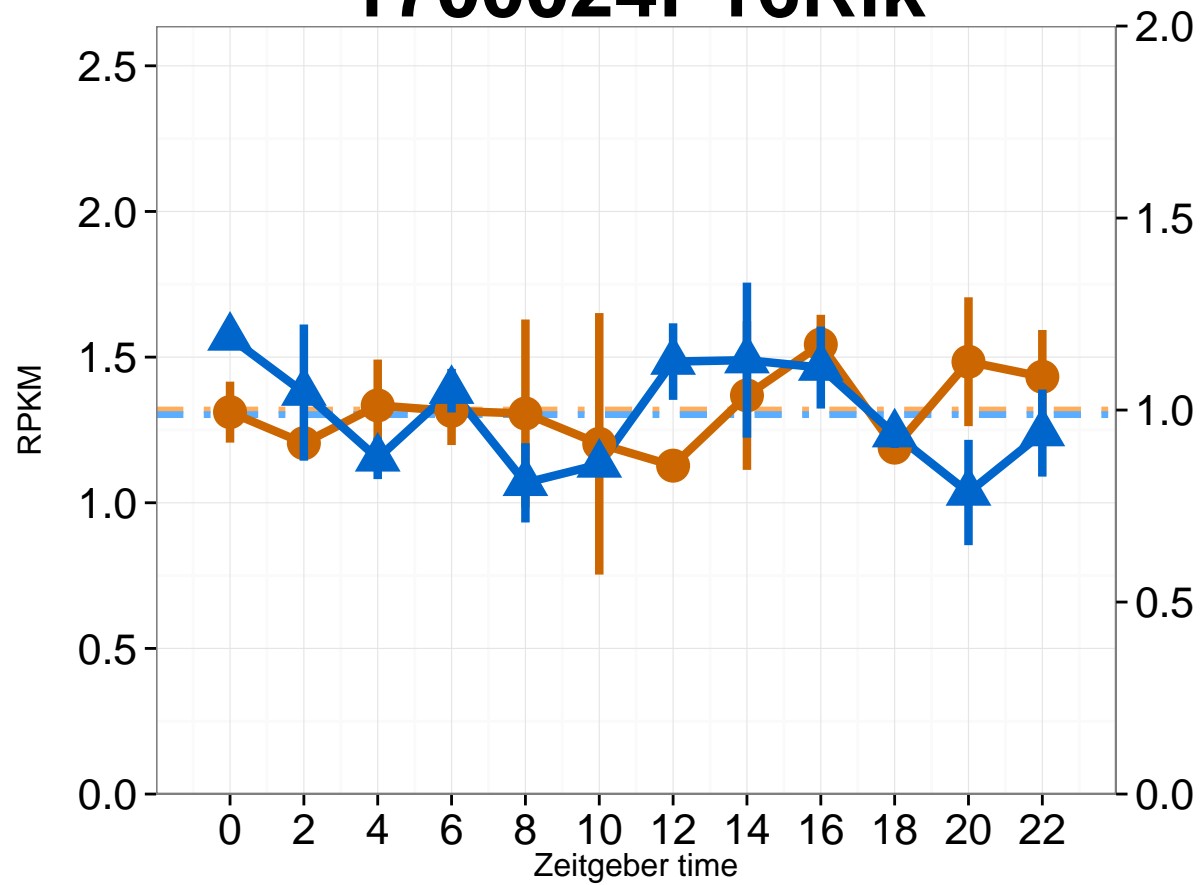

# 1700024P16Rik

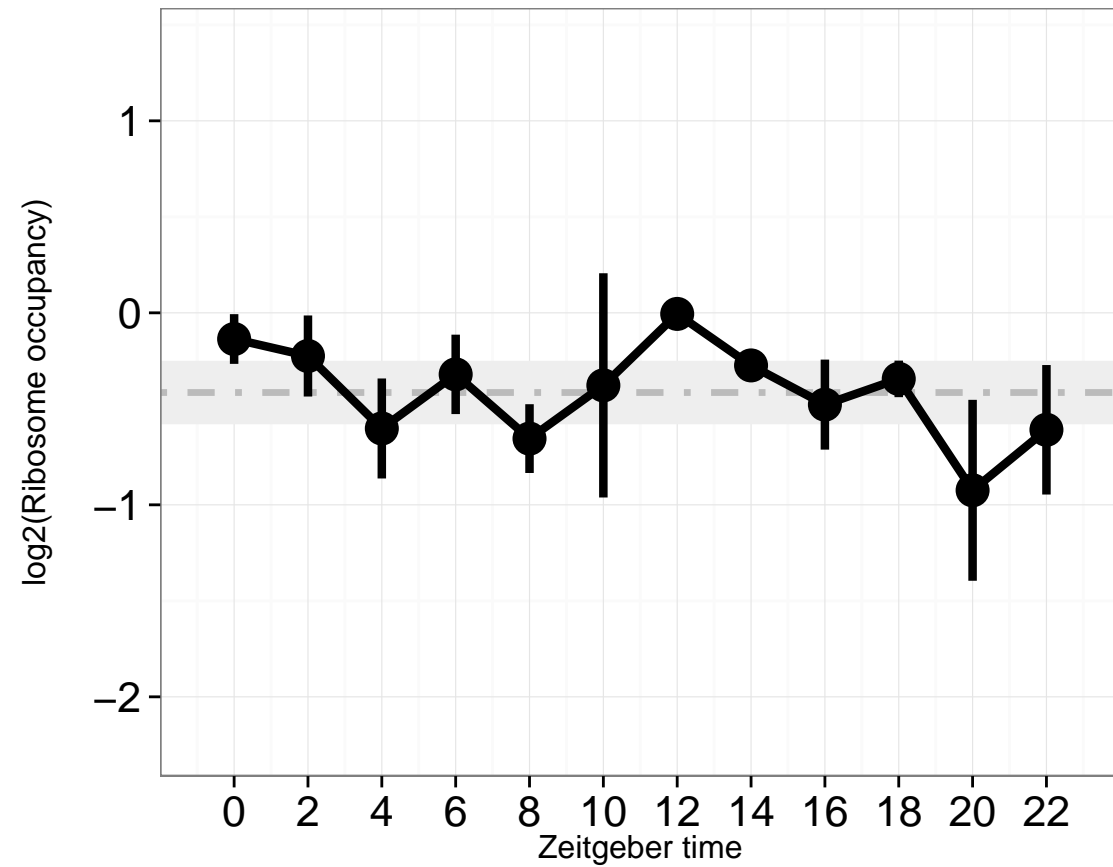

Supplement: Supplementary file 6 — Transcriptome-wide kidney RPF (blue) and RNA (orange) levels in the left panels (with “error bars” connecting the two replicates of each timepoint) and TE in the right panels. (ZIP 116896 kb) [file 13059_2017_1222_MOESM6_ESM.zip › Supp_Dataset_S1/A_RNA_non_rhythmic_RPF_non_rhythmic/1700024P16Rik_kidney_set_A.pdf]

# 1700025G04Rik

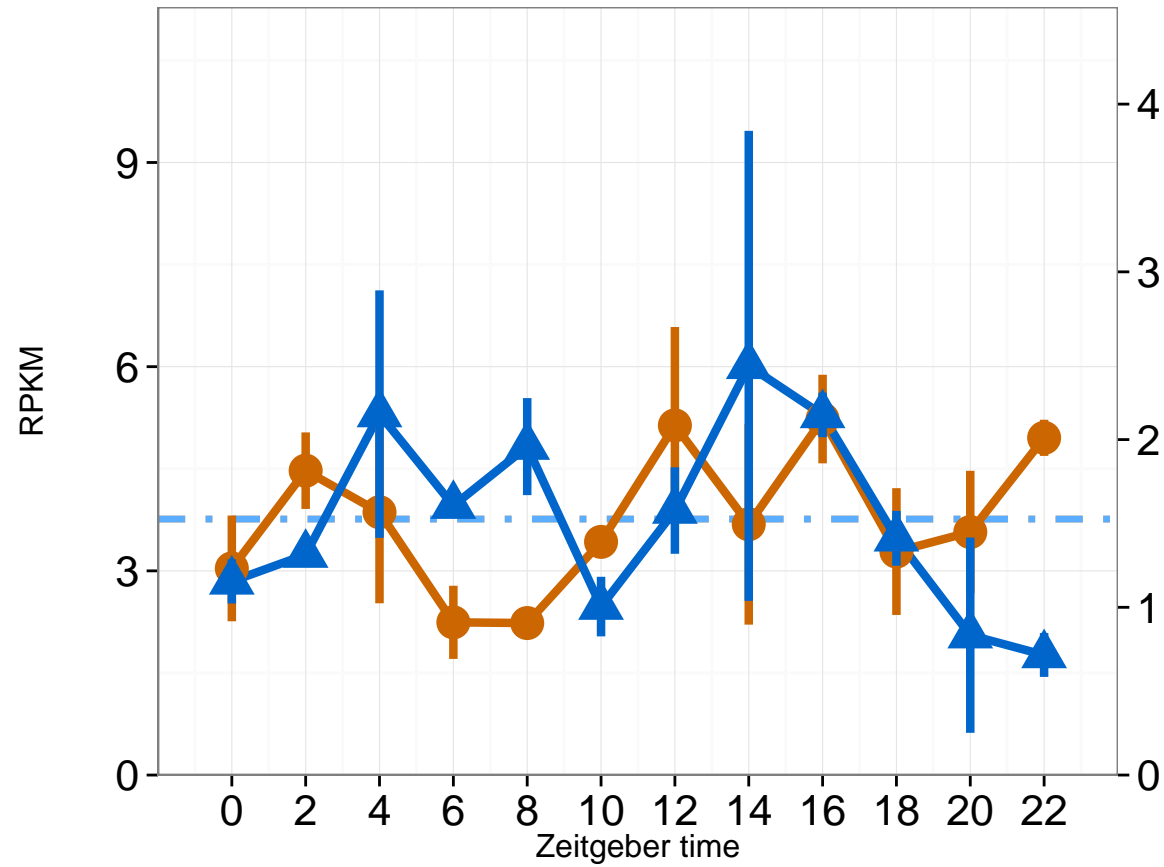

# 1700025G04Rik

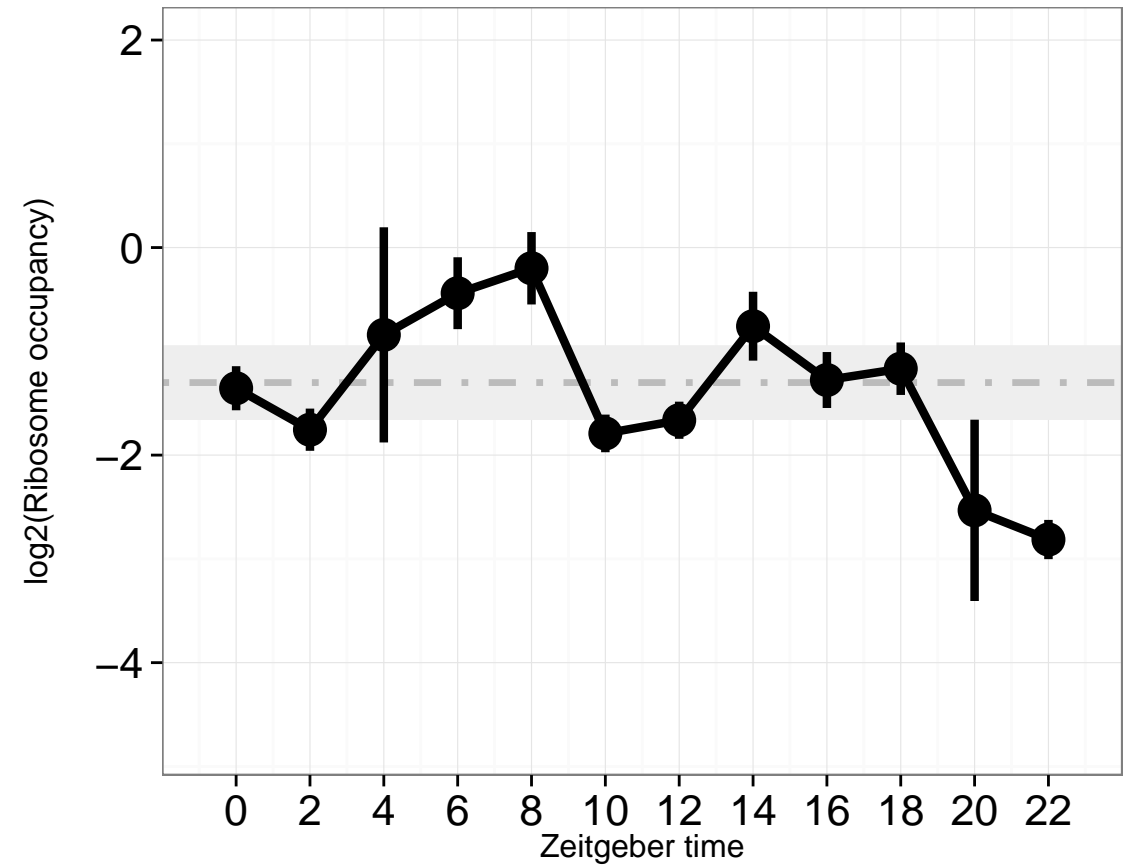

Supplement: Supplementary file 6 — Transcriptome-wide kidney RPF (blue) and RNA (orange) levels in the left panels (with “error bars” connecting the two replicates of each timepoint) and TE in the right panels. (ZIP 116896 kb) [file 13059_2017_1222_MOESM6_ESM.zip › Supp_Dataset_S1/A_RNA_non_rhythmic_RPF_non_rhythmic/1700025G04Rik_kidney_set_A.pdf]

# 1700029J07Rik

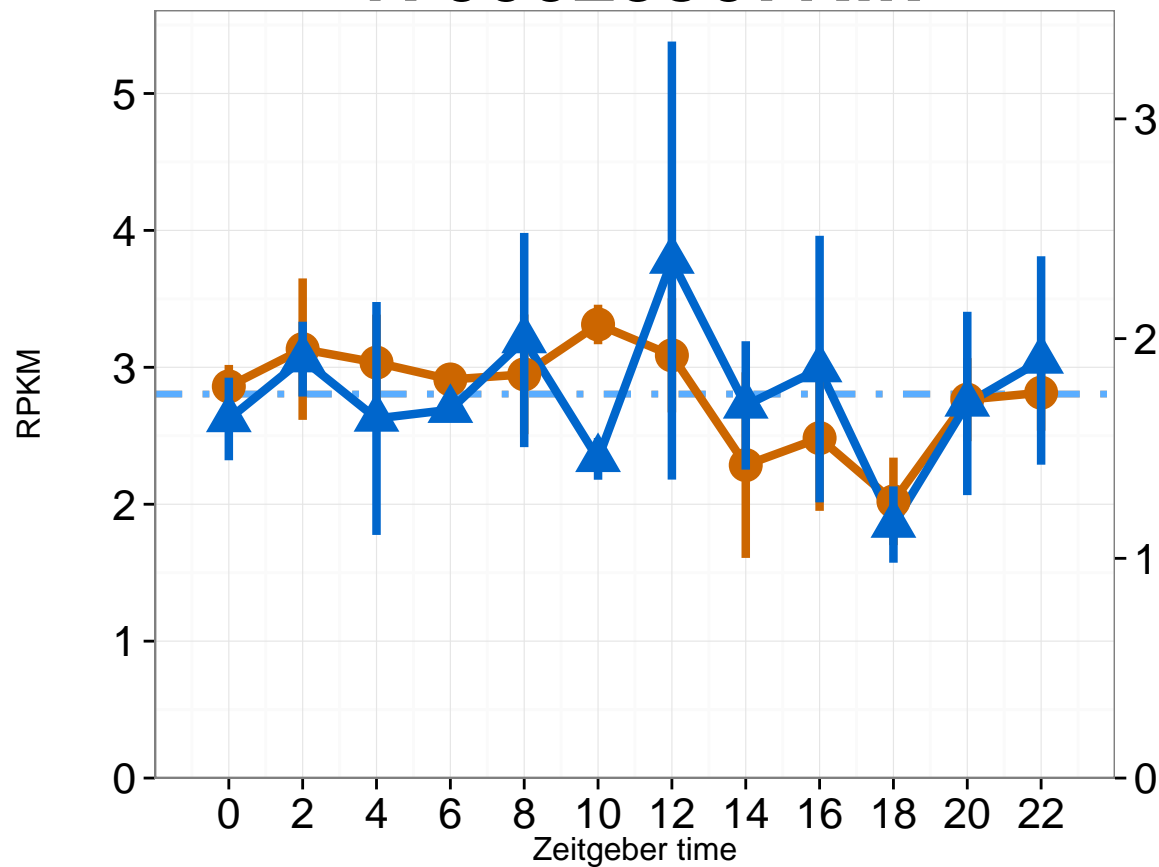

# 1700029J07Rik

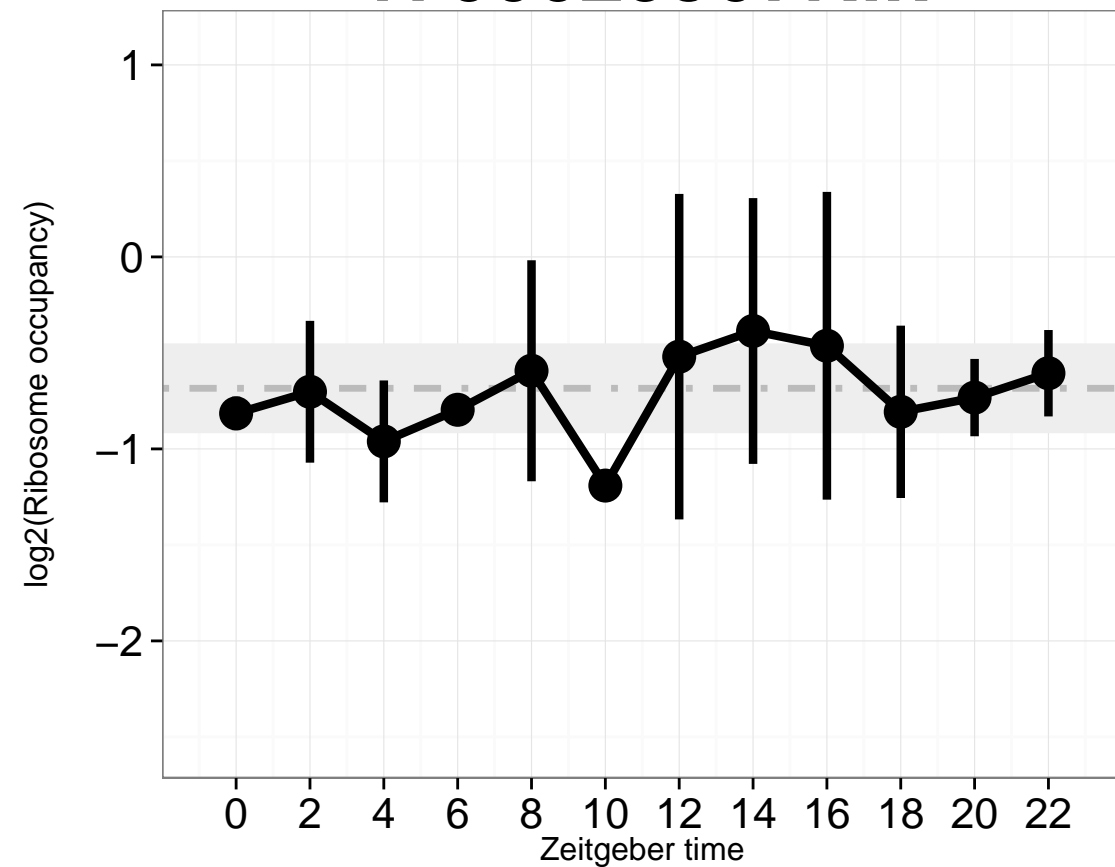

Supplement: Supplementary file 6 — Transcriptome-wide kidney RPF (blue) and RNA (orange) levels in the left panels (with “error bars” connecting the two replicates of each timepoint) and TE in the right panels. (ZIP 116896 kb) [file 13059_2017_1222_MOESM6_ESM.zip › Supp_Dataset_S1/A_RNA_non_rhythmic_RPF_non_rhythmic/1700029J07Rik_kidney_set_A.pdf]

# 1700030J22Rik

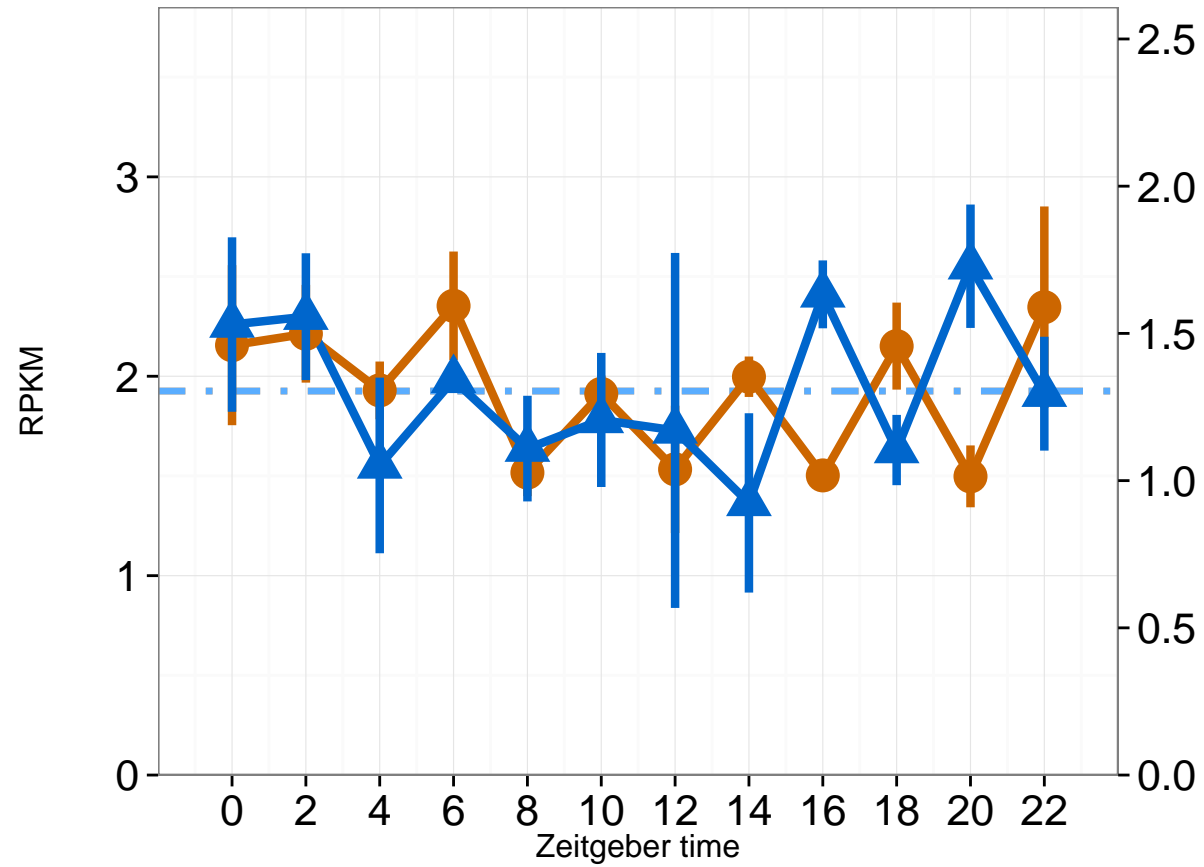

# 1700030J22Rik

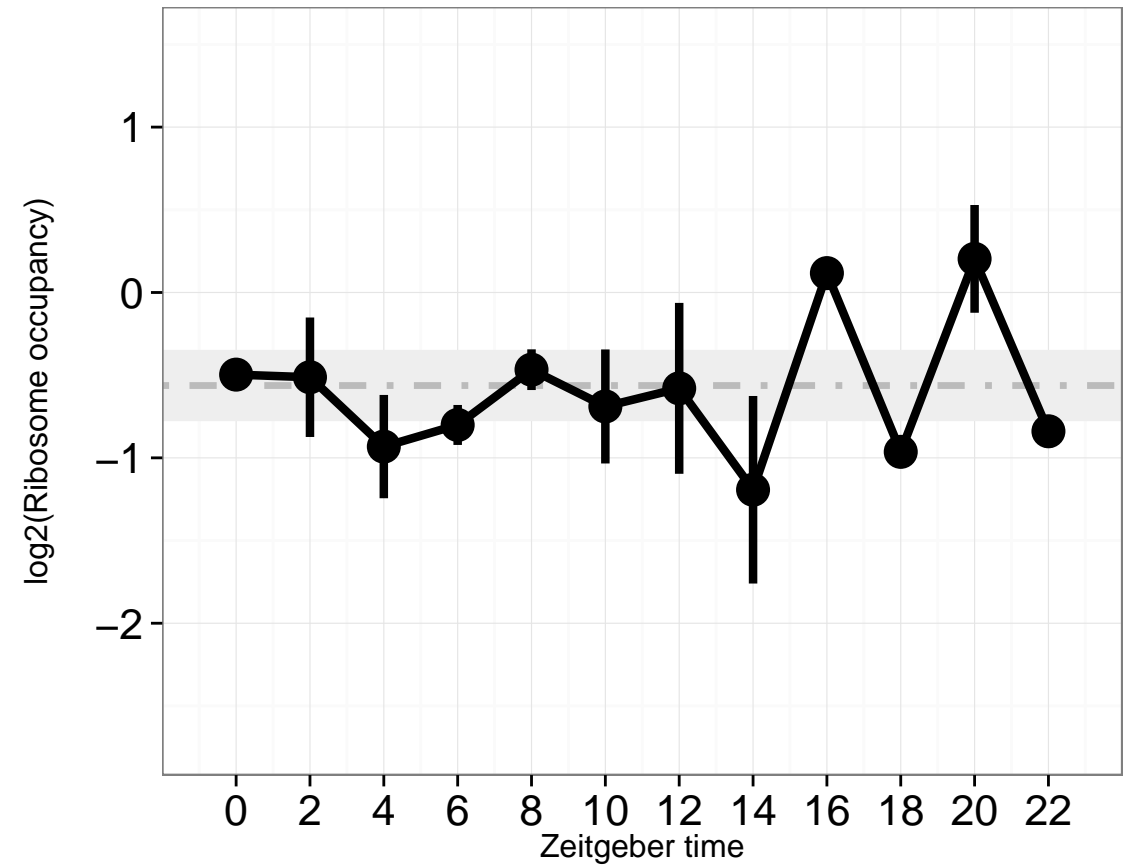

Supplement: Supplementary file 6 — Transcriptome-wide kidney RPF (blue) and RNA (orange) levels in the left panels (with “error bars” connecting the two replicates of each timepoint) and TE in the right panels. (ZIP 116896 kb) [file 13059_2017_1222_MOESM6_ESM.zip › Supp_Dataset_S1/A_RNA_non_rhythmic_RPF_non_rhythmic/1700030J22Rik_kidney_set_A.pdf]

# 1700030K09Rik

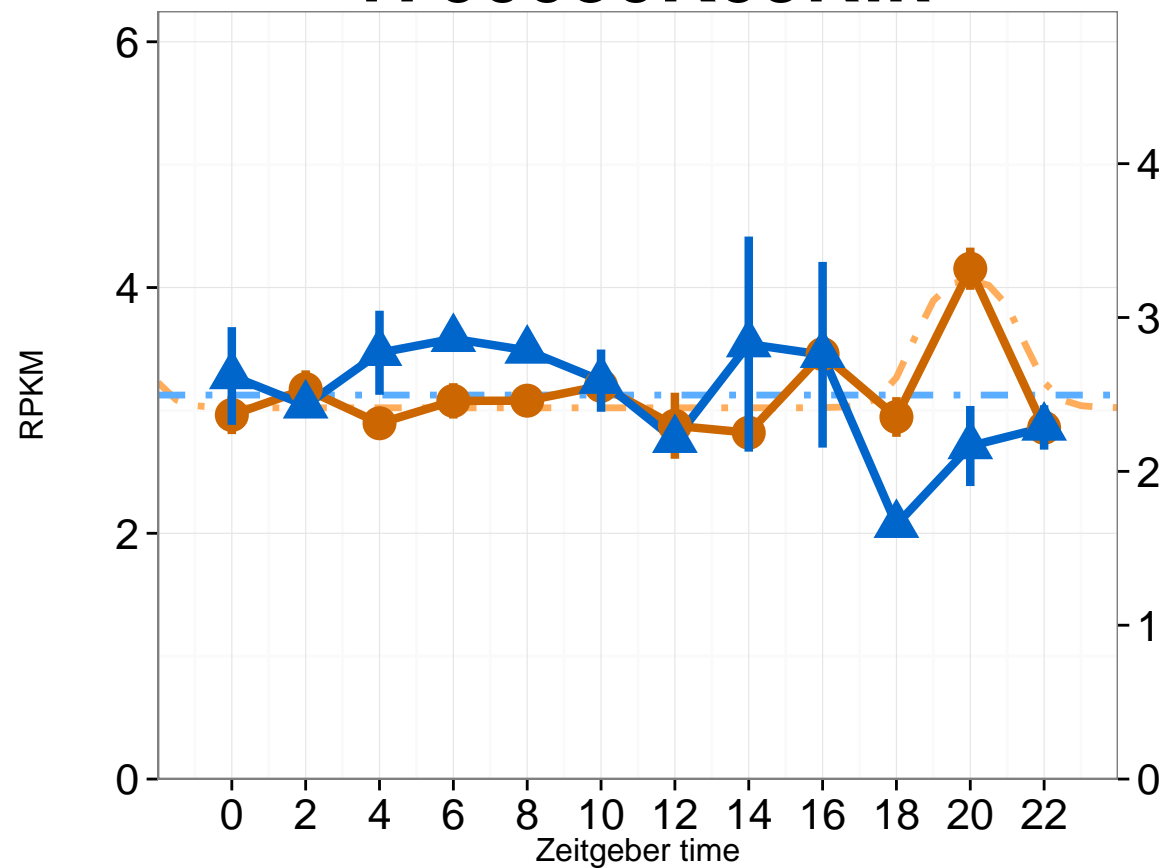

# 1700030K09Rik

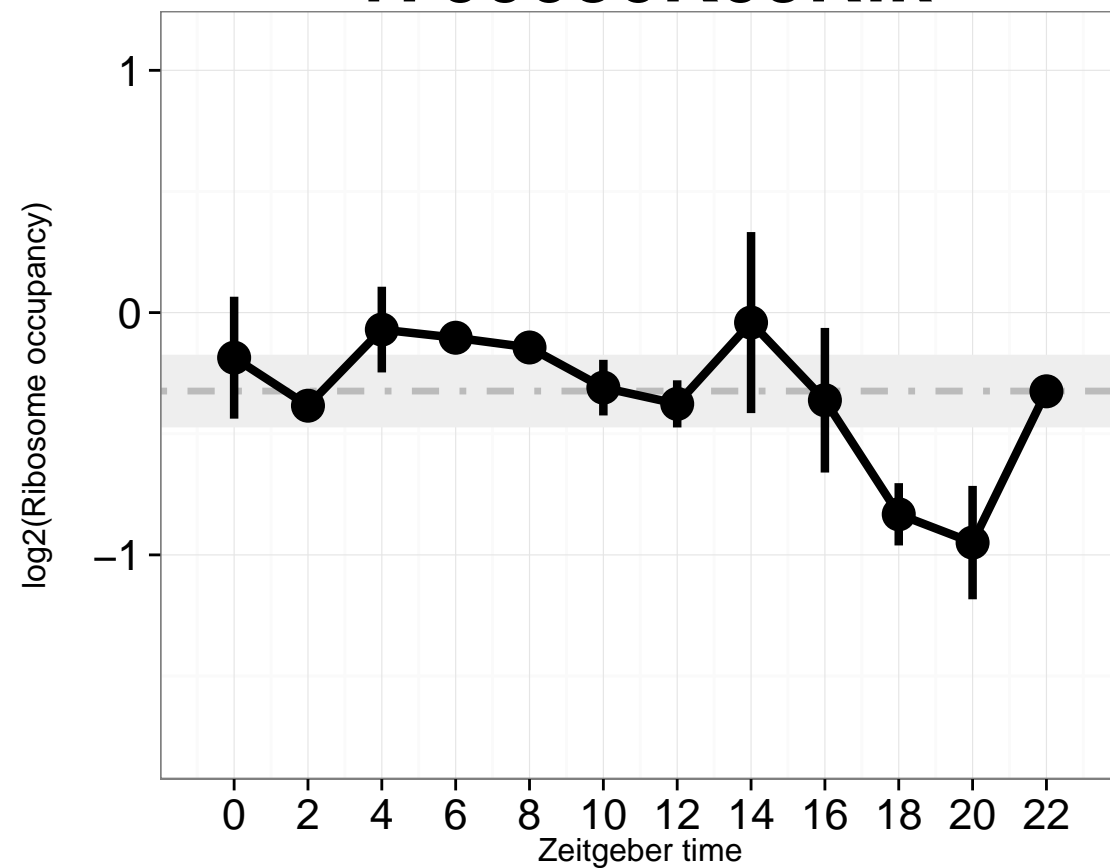

Supplement: Supplementary file 6 — Transcriptome-wide kidney RPF (blue) and RNA (orange) levels in the left panels (with “error bars” connecting the two replicates of each timepoint) and TE in the right panels. (ZIP 116896 kb) [file 13059_2017_1222_MOESM6_ESM.zip › Supp_Dataset_S1/A_RNA_non_rhythmic_RPF_non_rhythmic/1700030K09Rik_kidney_set_A.pdf]

# 1700037H04Rik

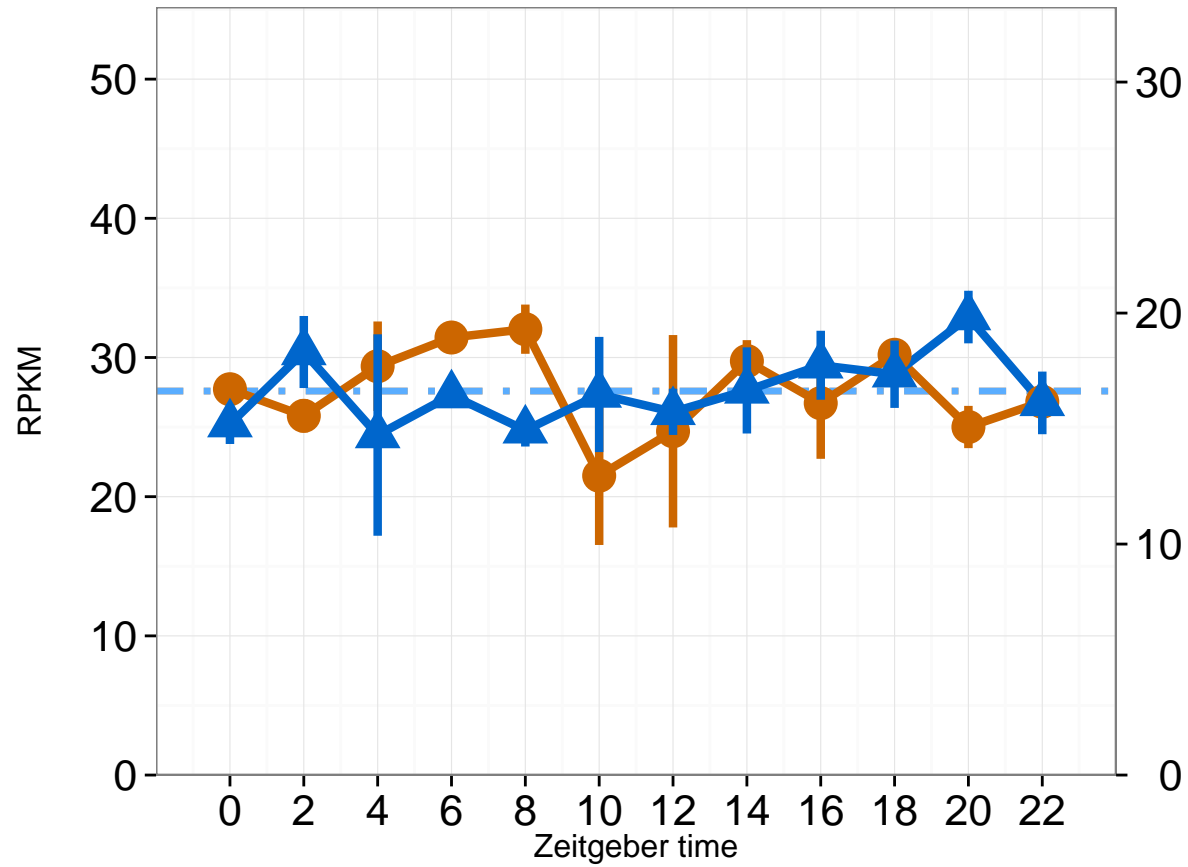

# 1700037H04Rik

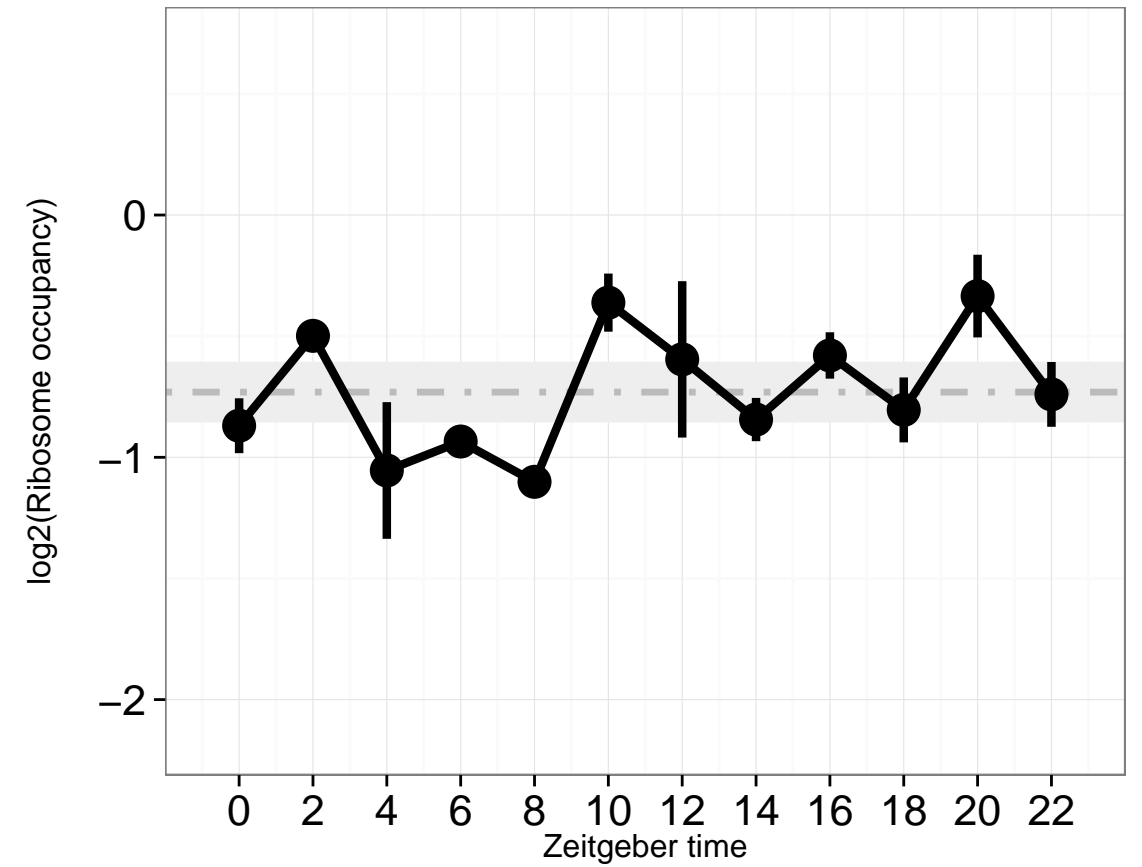

Supplement: Supplementary file 6 — Transcriptome-wide kidney RPF (blue) and RNA (orange) levels in the left panels (with “error bars” connecting the two replicates of each timepoint) and TE in the right panels. (ZIP 116896 kb) [file 13059_2017_1222_MOESM6_ESM.zip › Supp_Dataset_S1/A_RNA_non_rhythmic_RPF_non_rhythmic/1700037H04Rik_kidney_set_A.pdf]

## 1700040L02Rik

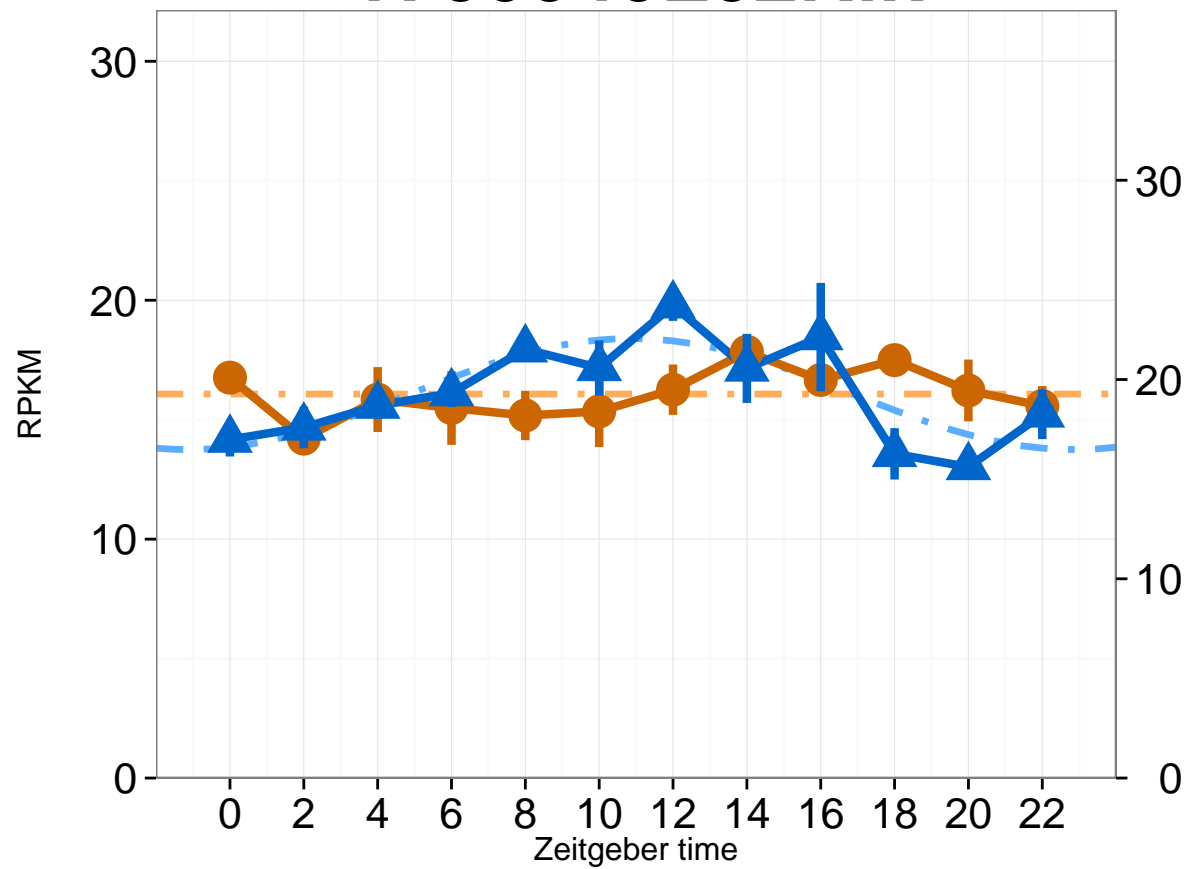

## 1700040L02Rik

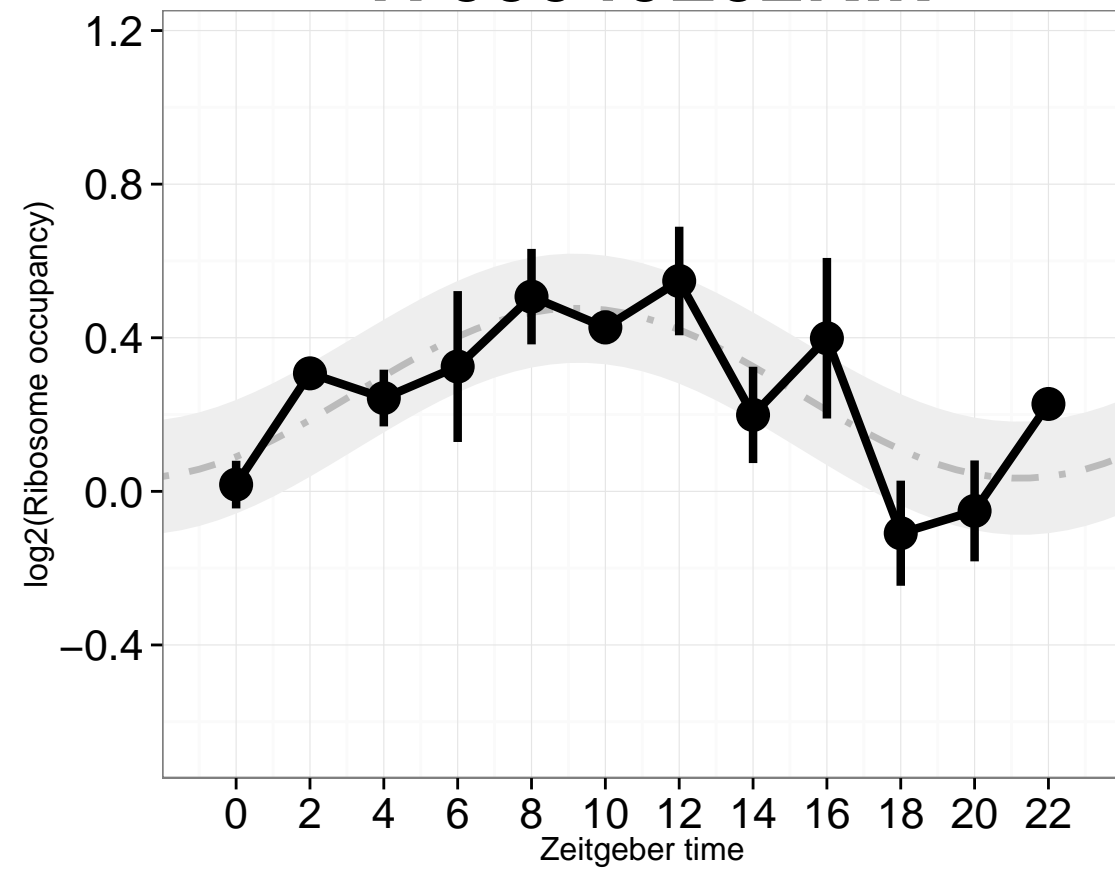

Supplement: Supplementary file 6 — Transcriptome-wide kidney RPF (blue) and RNA (orange) levels in the left panels (with “error bars” connecting the two replicates of each timepoint) and TE in the right panels. (ZIP 116896 kb) [file 13059_2017_1222_MOESM6_ESM.zip › Supp_Dataset_S1/A_RNA_non_rhythmic_RPF_non_rhythmic/1700040L02Rik_kidney_set_A.pdf]

# 1700049G17Rik

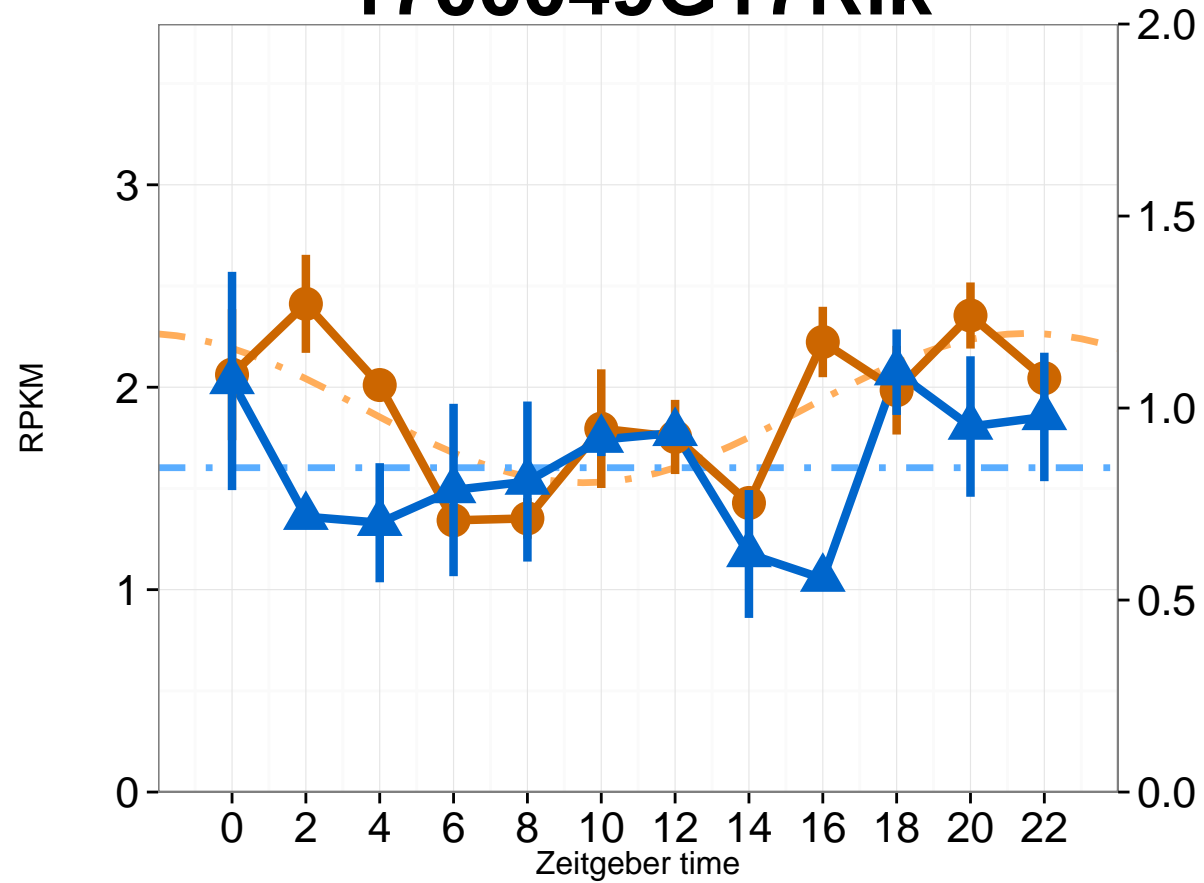

# 1700049G17Rik

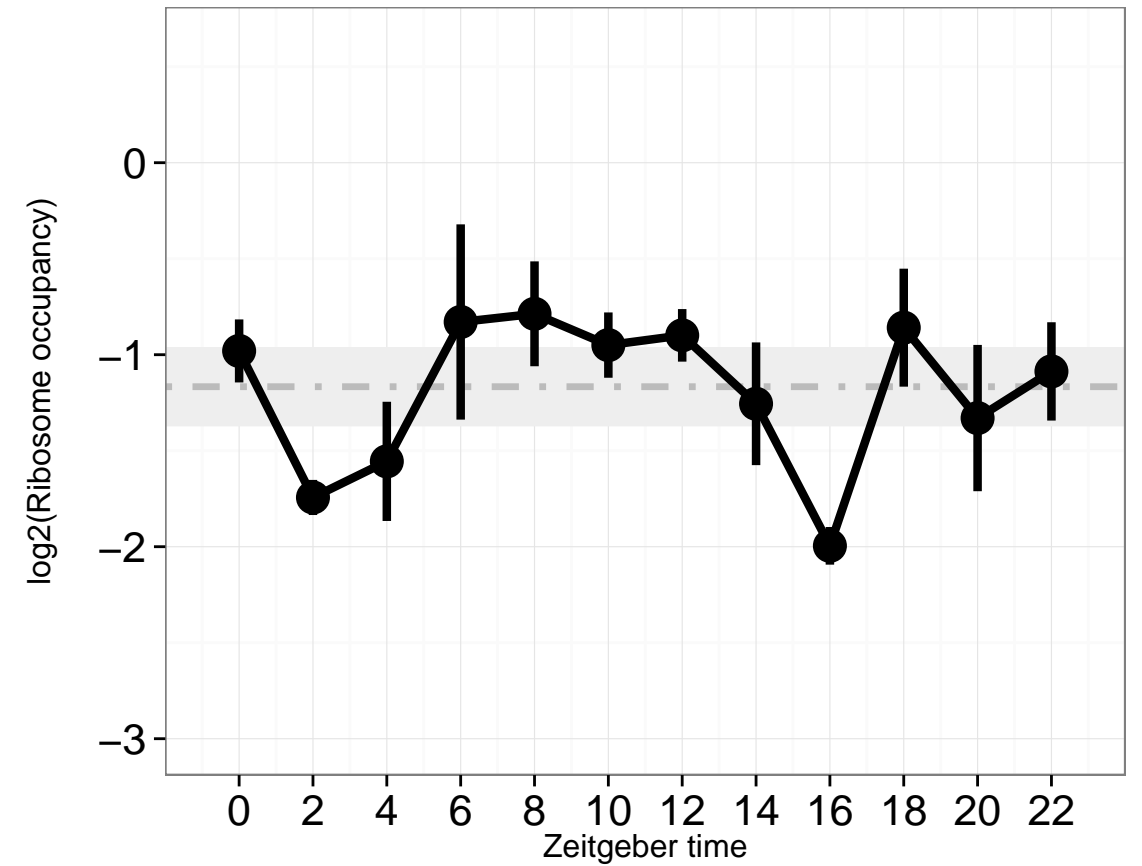

Supplement: Supplementary file 6 — Transcriptome-wide kidney RPF (blue) and RNA (orange) levels in the left panels (with “error bars” connecting the two replicates of each timepoint) and TE in the right panels. (ZIP 116896 kb) [file 13059_2017_1222_MOESM6_ESM.zip › Supp_Dataset_S1/A_RNA_non_rhythmic_RPF_non_rhythmic/1700049G17Rik_kidney_set_A.pdf]

# 1700052N19Rik

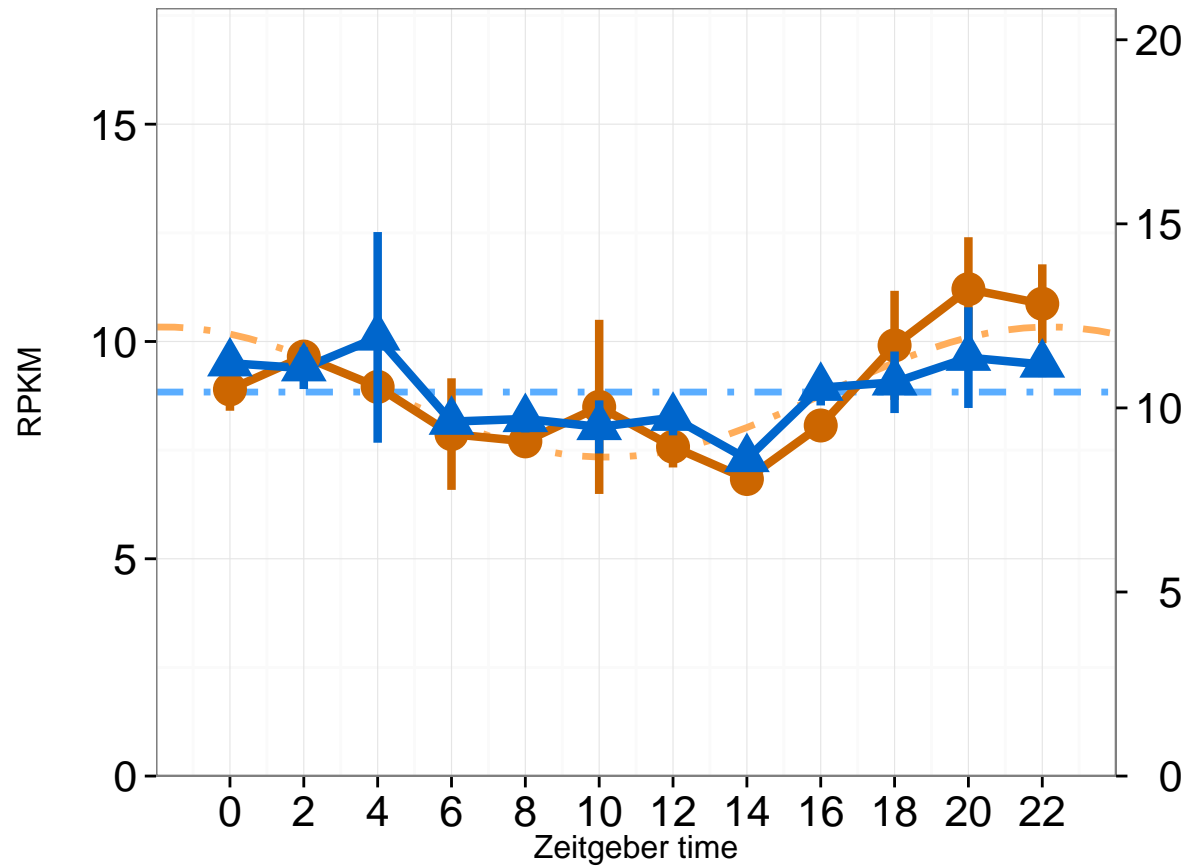

# 1700052N19Rik

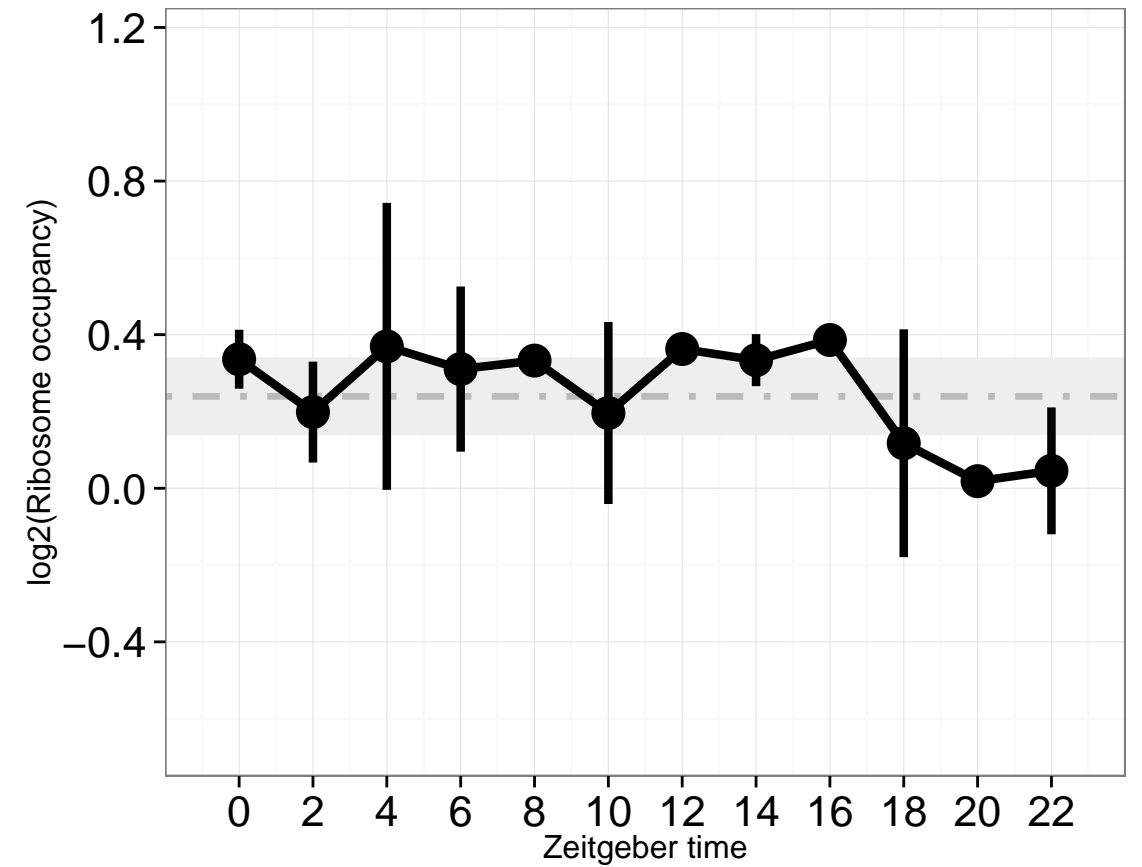

Supplement: Supplementary file 6 — Transcriptome-wide kidney RPF (blue) and RNA (orange) levels in the left panels (with “error bars” connecting the two replicates of each timepoint) and TE in the right panels. (ZIP 116896 kb) [file 13059_2017_1222_MOESM6_ESM.zip › Supp_Dataset_S1/A_RNA_non_rhythmic_RPF_non_rhythmic/1700052N19Rik_kidney_set_A.pdf]

## 1700055D18Rik

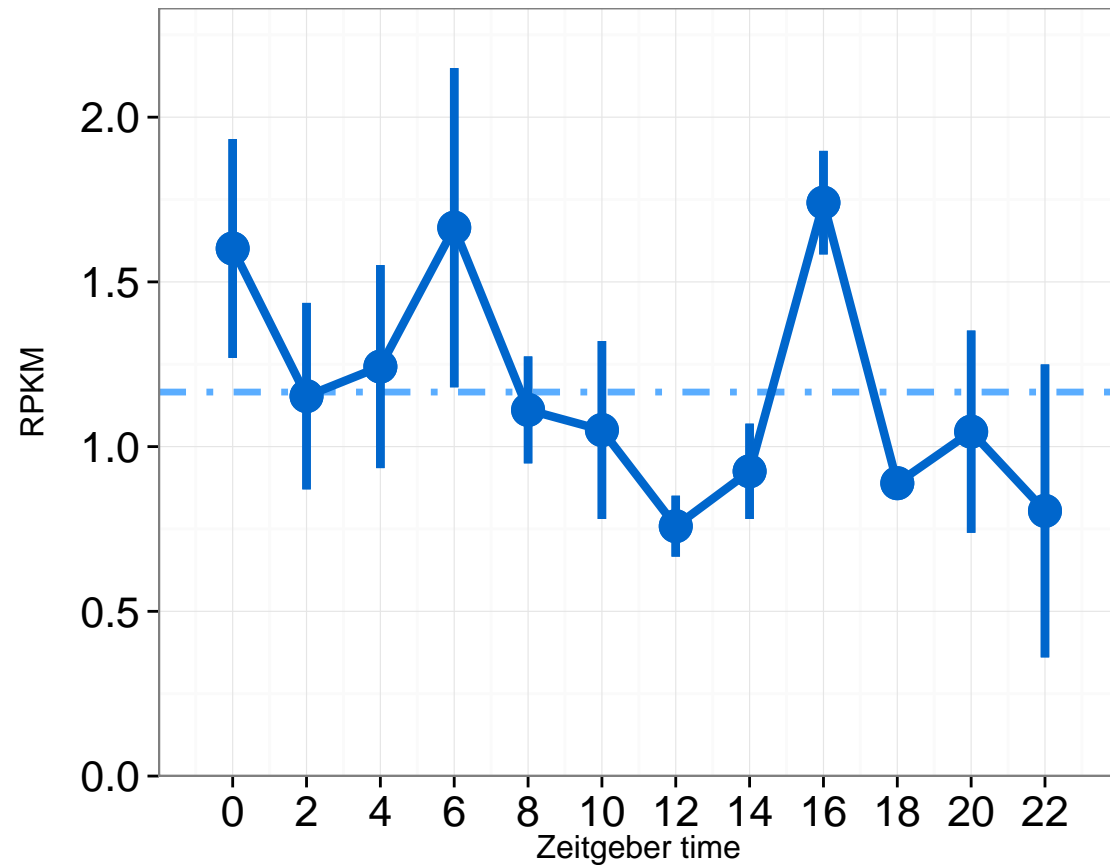

## 1700055D18Rik log2(Ribosome occup

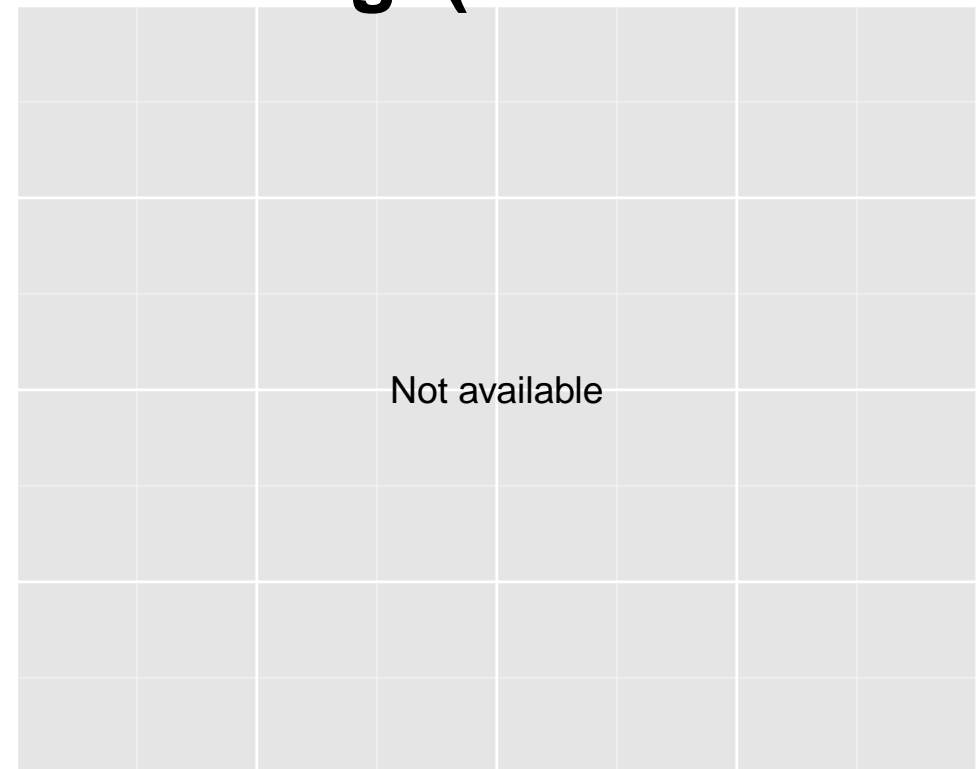

Supplement: Supplementary file 6 — Transcriptome-wide kidney RPF (blue) and RNA (orange) levels in the left panels (with “error bars” connecting the two replicates of each timepoint) and TE in the right panels. (ZIP 116896 kb) [file 13059_2017_1222_MOESM6_ESM.zip › Supp_Dataset_S1/A_RNA_non_rhythmic_RPF_non_rhythmic/1700055D18Rik_kidney_set_A.pdf]

# 1700055N04Rik

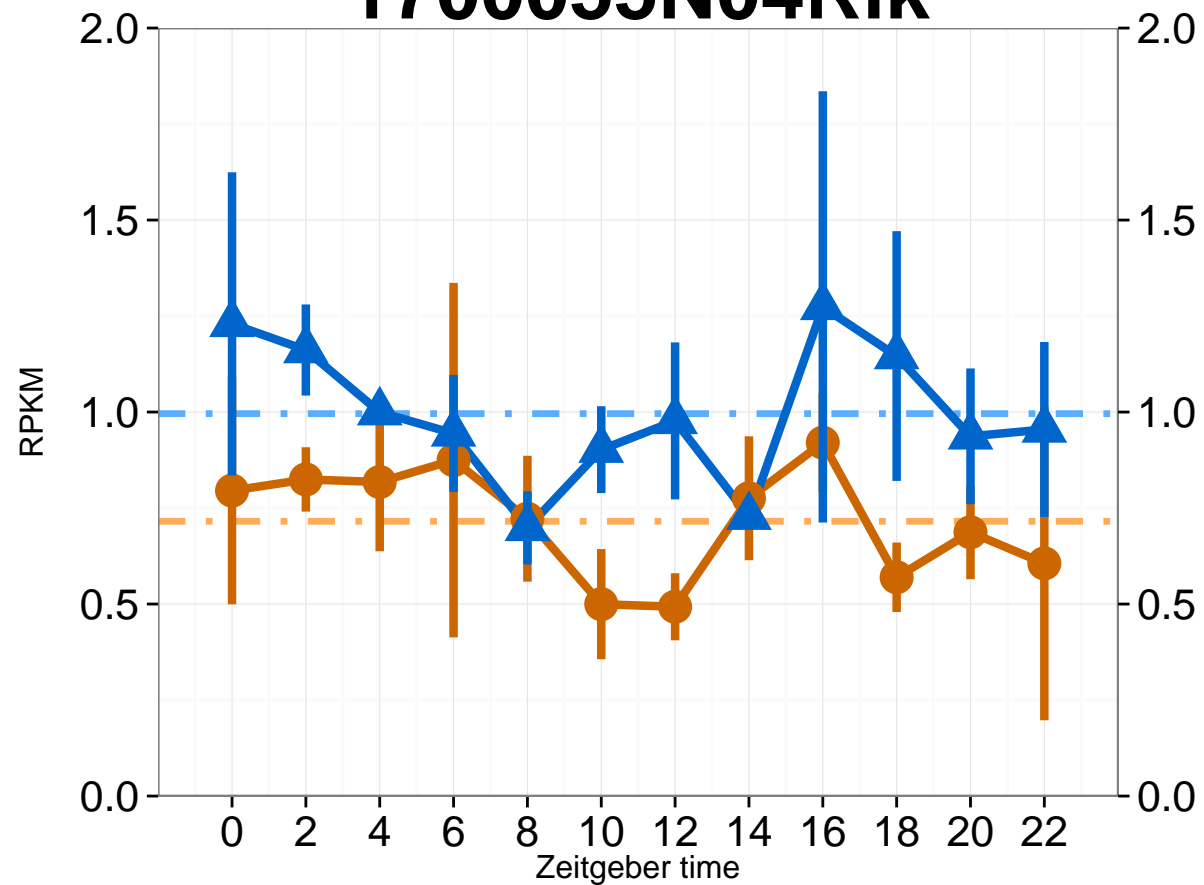

# 1700055N04Rik

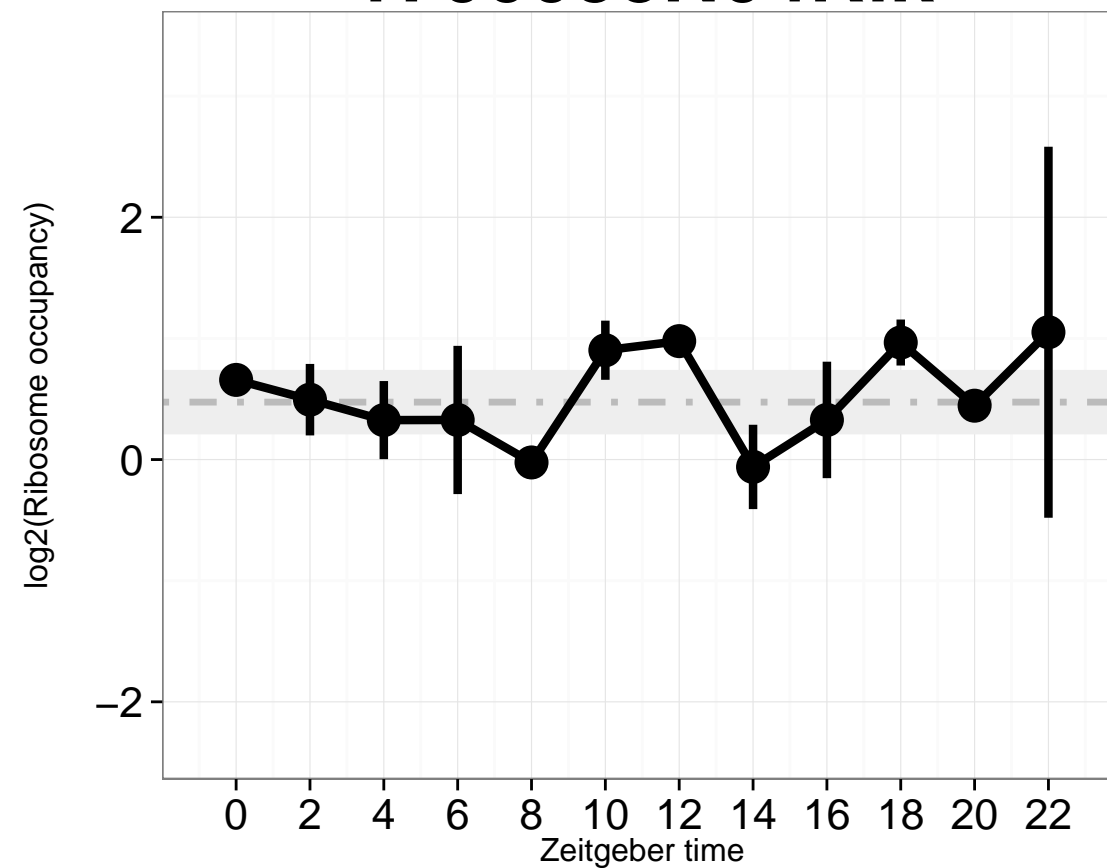

Supplement: Supplementary file 6 — Transcriptome-wide kidney RPF (blue) and RNA (orange) levels in the left panels (with “error bars” connecting the two replicates of each timepoint) and TE in the right panels. (ZIP 116896 kb) [file 13059_2017_1222_MOESM6_ESM.zip › Supp_Dataset_S1/A_RNA_non_rhythmic_RPF_non_rhythmic/1700055N04Rik_kidney_set_A.pdf]

# 1700066M21Rik

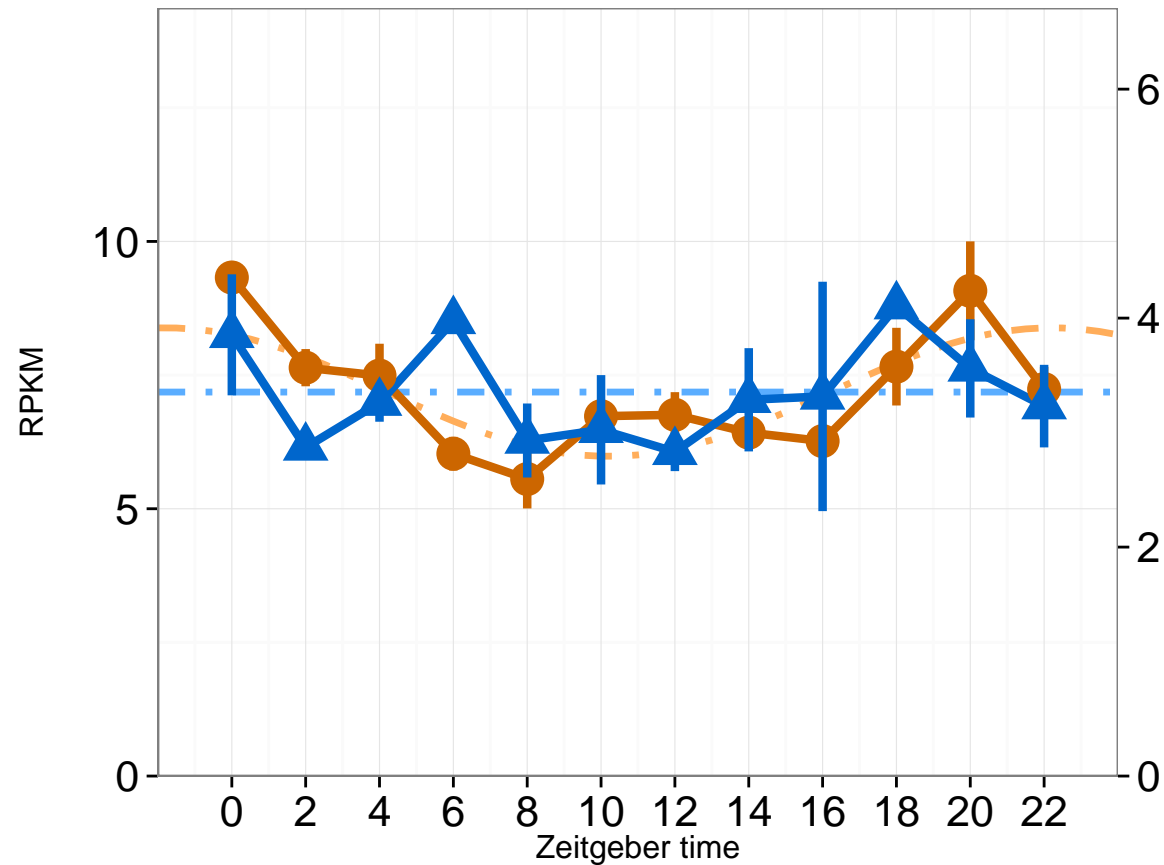

# 1700066M21Rik

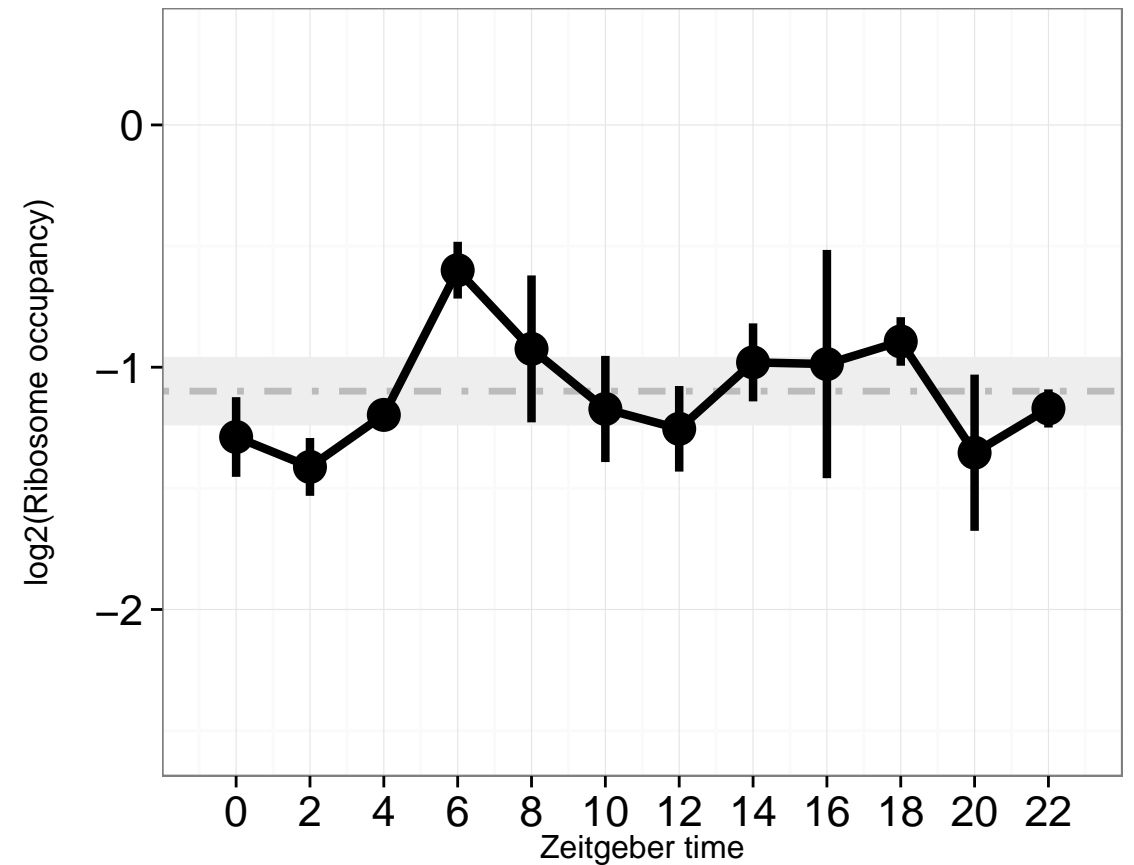

Supplement: Supplementary file 6 — Transcriptome-wide kidney RPF (blue) and RNA (orange) levels in the left panels (with “error bars” connecting the two replicates of each timepoint) and TE in the right panels. (ZIP 116896 kb) [file 13059_2017_1222_MOESM6_ESM.zip › Supp_Dataset_S1/A_RNA_non_rhythmic_RPF_non_rhythmic/1700066M21Rik_kidney_set_A.pdf]

# 1700067K01Rik

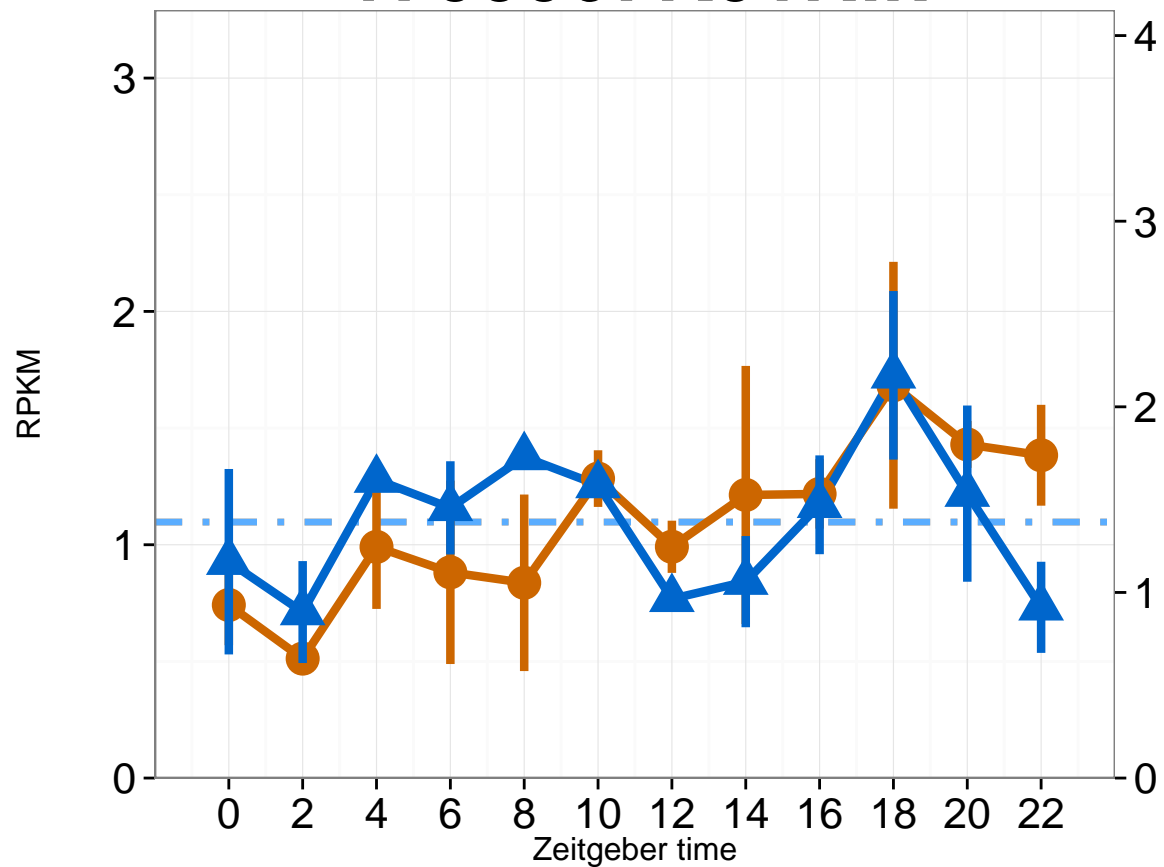

# 1700067K01Rik

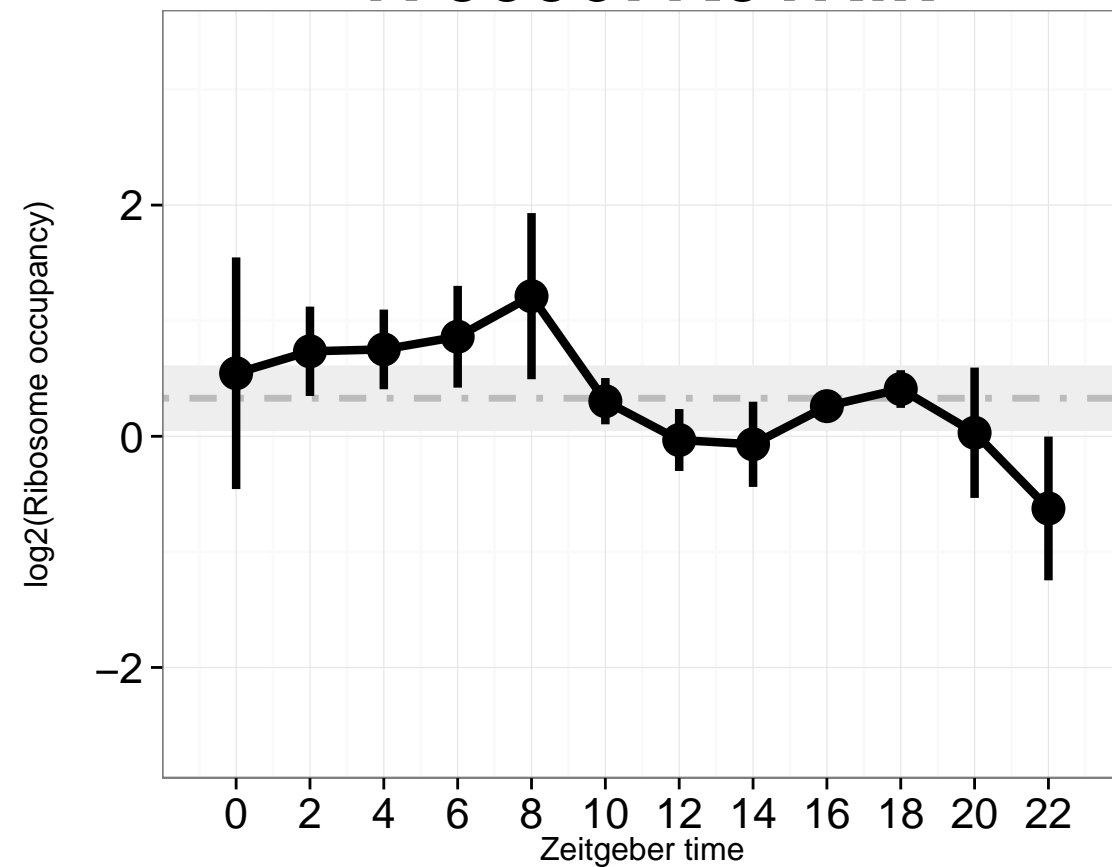

Supplement: Supplementary file 6 — Transcriptome-wide kidney RPF (blue) and RNA (orange) levels in the left panels (with “error bars” connecting the two replicates of each timepoint) and TE in the right panels. (ZIP 116896 kb) [file 13059_2017_1222_MOESM6_ESM.zip › Supp_Dataset_S1/A_RNA_non_rhythmic_RPF_non_rhythmic/1700067K01Rik_kidney_set_A.pdf]

# 1700088E04Rik

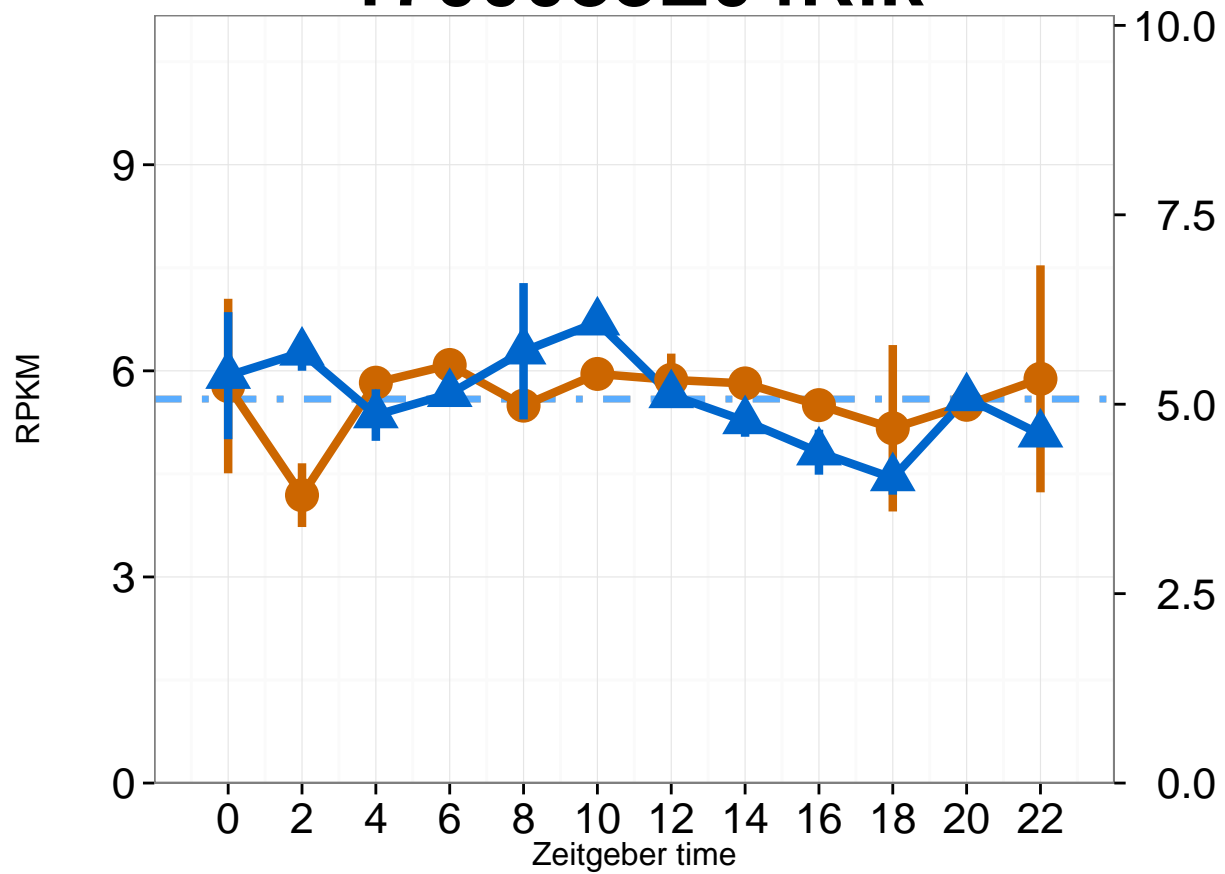

# 1700088E04Rik

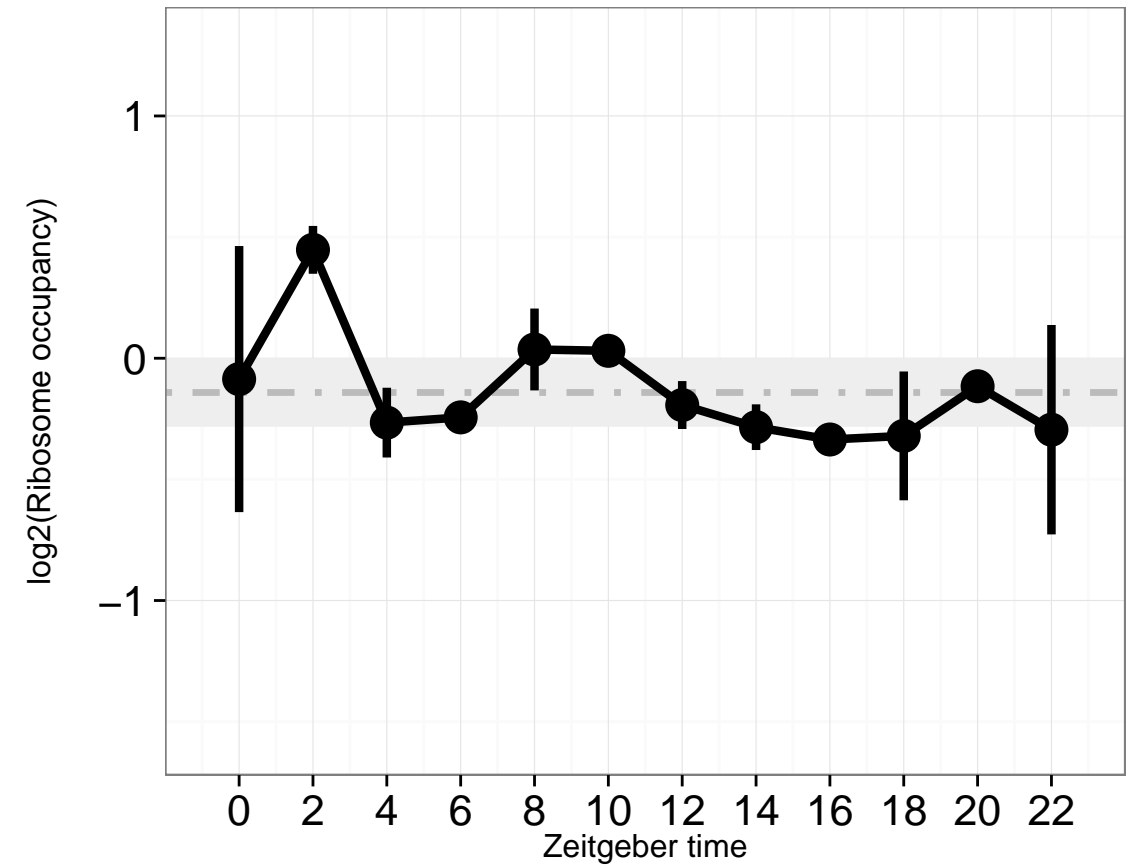

Supplement: Supplementary file 6 — Transcriptome-wide kidney RPF (blue) and RNA (orange) levels in the left panels (with “error bars” connecting the two replicates of each timepoint) and TE in the right panels. (ZIP 116896 kb) [file 13059_2017_1222_MOESM6_ESM.zip › Supp_Dataset_S1/A_RNA_non_rhythmic_RPF_non_rhythmic/1700088E04Rik_kidney_set_A.pdf]

# 1700094D03Rik

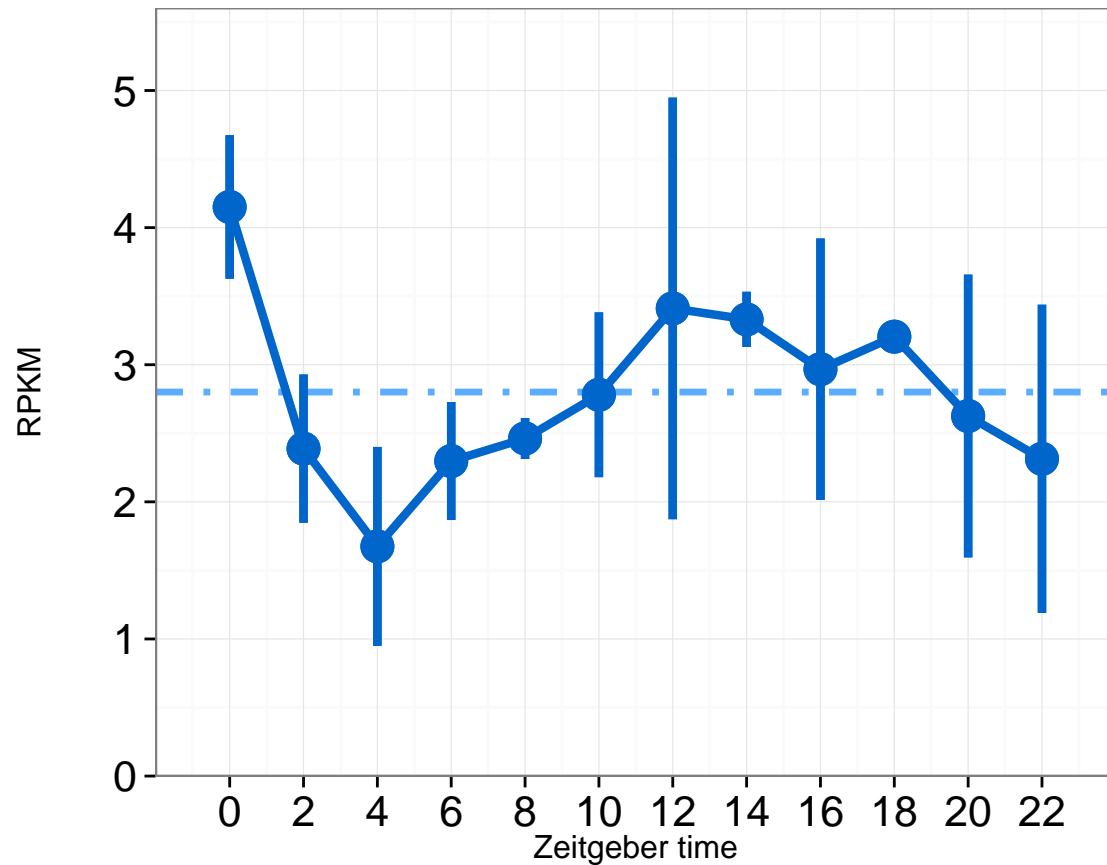

# 1700094D03Rik log2(Ribosome occup

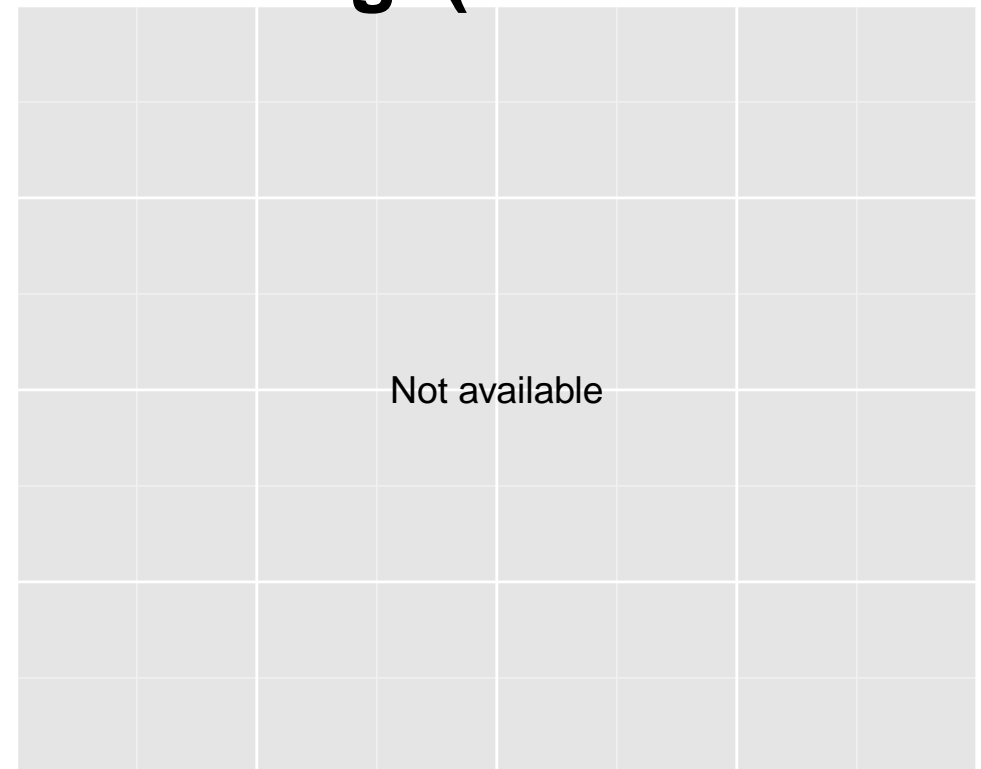

Supplement: Supplementary file 6 — Transcriptome-wide kidney RPF (blue) and RNA (orange) levels in the left panels (with “error bars” connecting the two replicates of each timepoint) and TE in the right panels. (ZIP 116896 kb) [file 13059_2017_1222_MOESM6_ESM.zip › Supp_Dataset_S1/A_RNA_non_rhythmic_RPF_non_rhythmic/1700094D03Rik_kidney_set_A.pdf]

# 1700109H08Rik

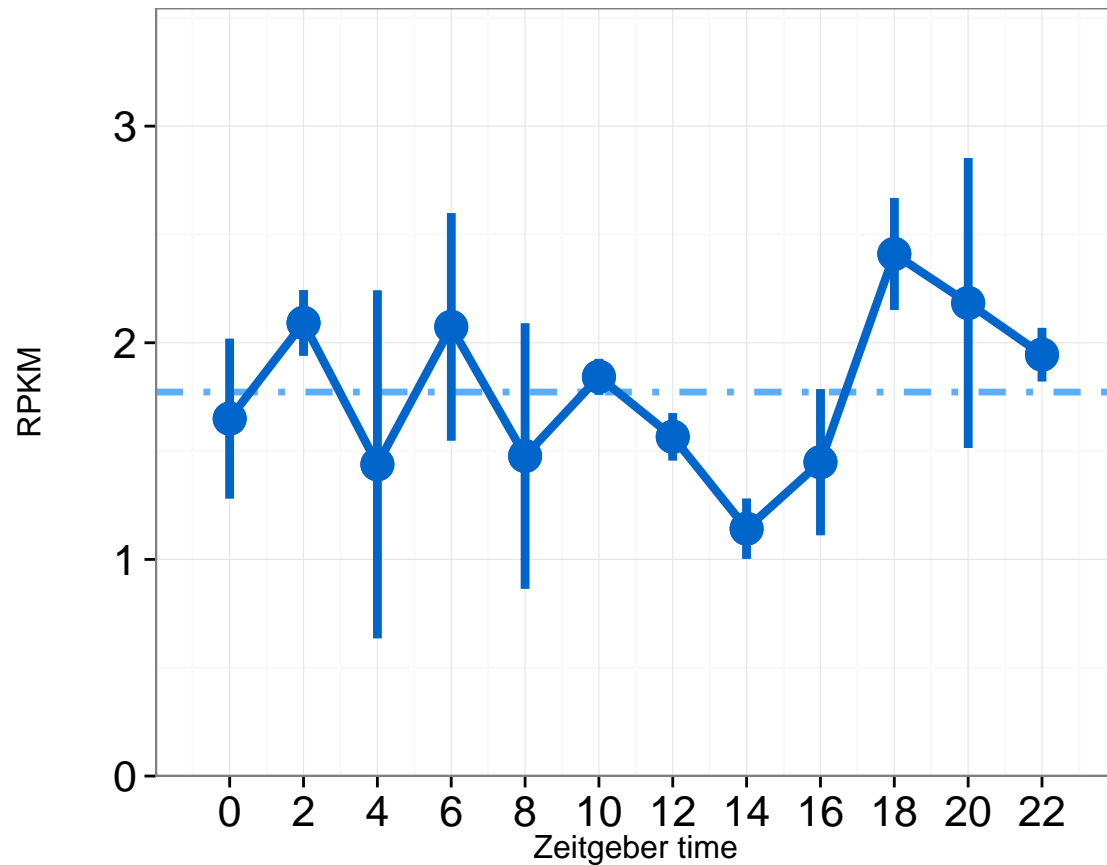

# 1700109H08Rik log2(Ribosome occup

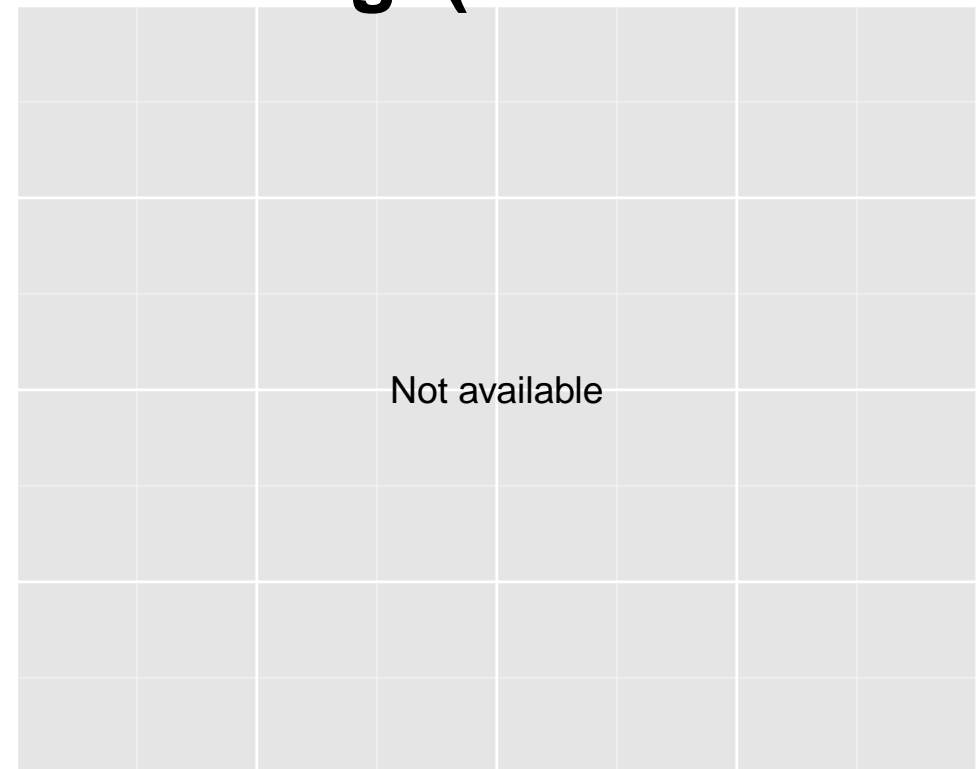

Supplement: Supplementary file 6 — Transcriptome-wide kidney RPF (blue) and RNA (orange) levels in the left panels (with “error bars” connecting the two replicates of each timepoint) and TE in the right panels. (ZIP 116896 kb) [file 13059_2017_1222_MOESM6_ESM.zip › Supp_Dataset_S1/A_RNA_non_rhythmic_RPF_non_rhythmic/1700109H08Rik_kidney_set_A.pdf]

# 1700123O20Rik

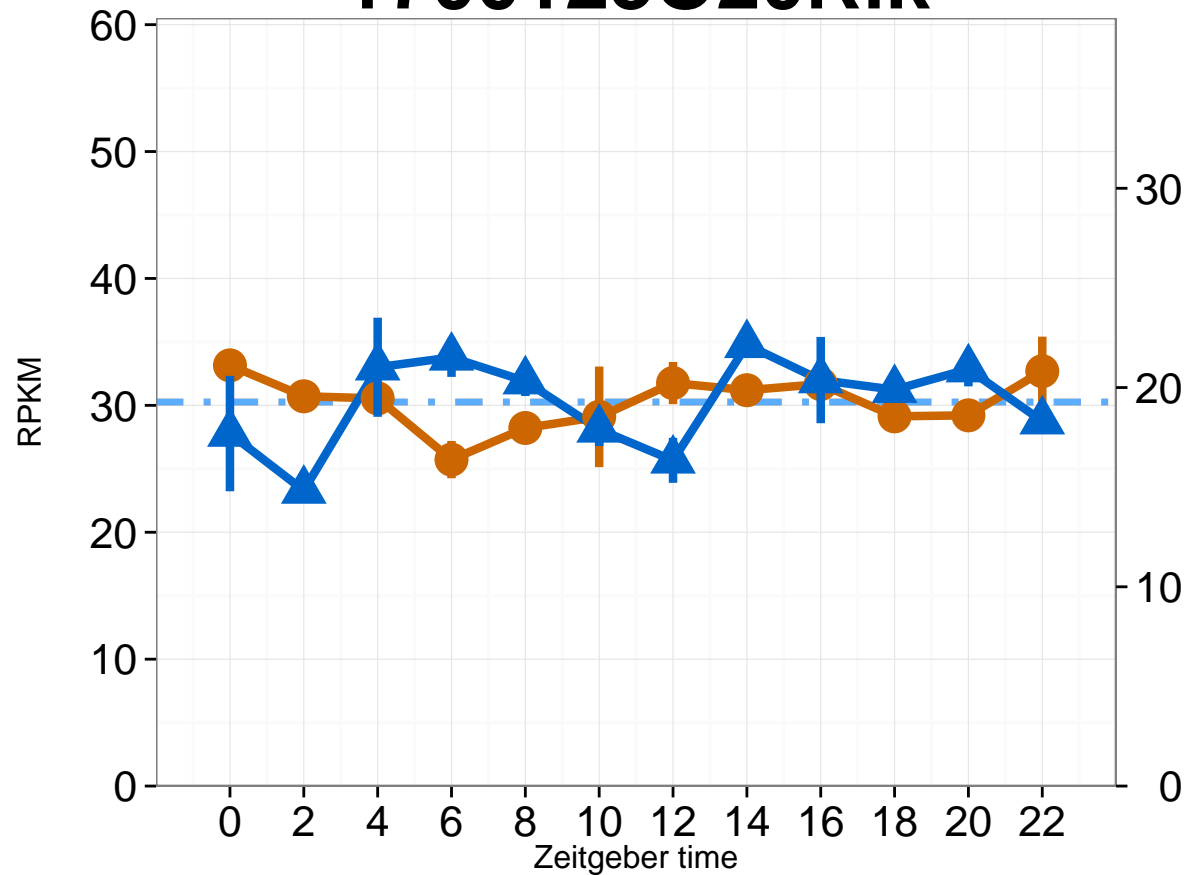

# 1700123O20Rik

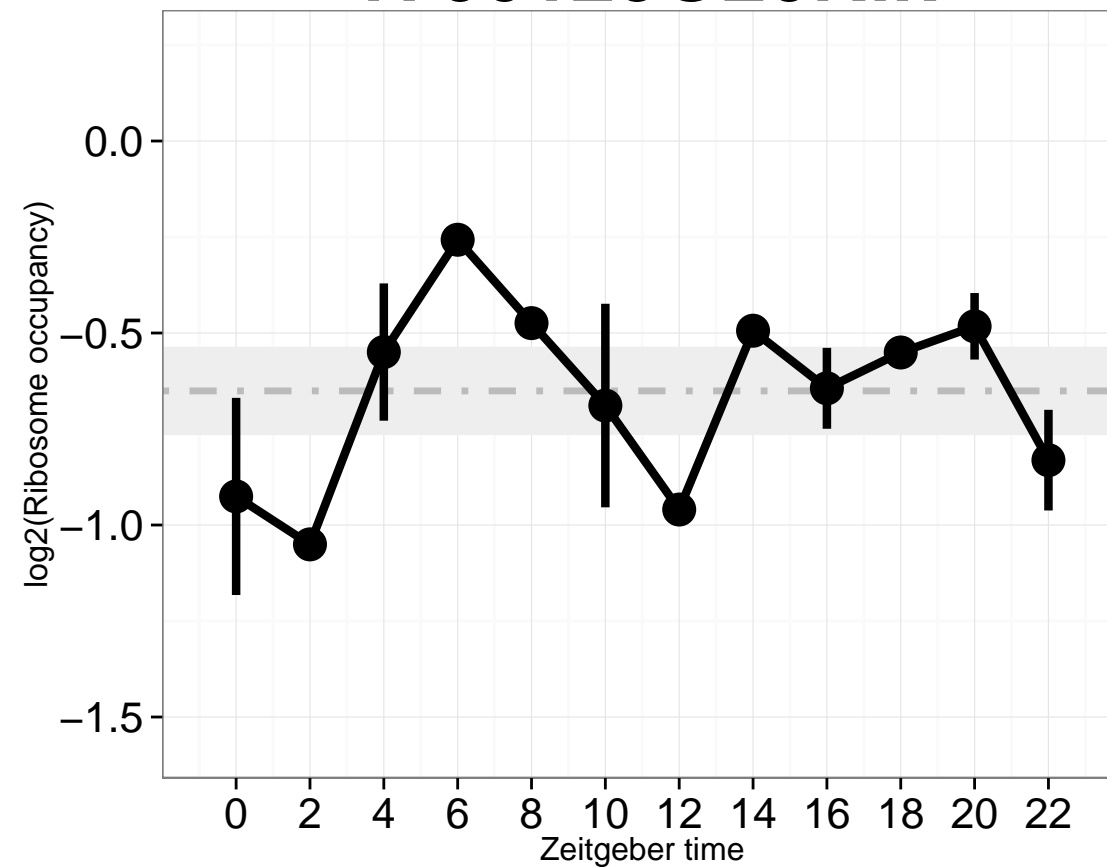

Supplement: Supplementary file 6 — Transcriptome-wide kidney RPF (blue) and RNA (orange) levels in the left panels (with “error bars” connecting the two replicates of each timepoint) and TE in the right panels. (ZIP 116896 kb) [file 13059_2017_1222_MOESM6_ESM.zip › Supp_Dataset_S1/A_RNA_non_rhythmic_RPF_non_rhythmic/1700123O20Rik_kidney_set_A.pdf]

1700128F08Rik

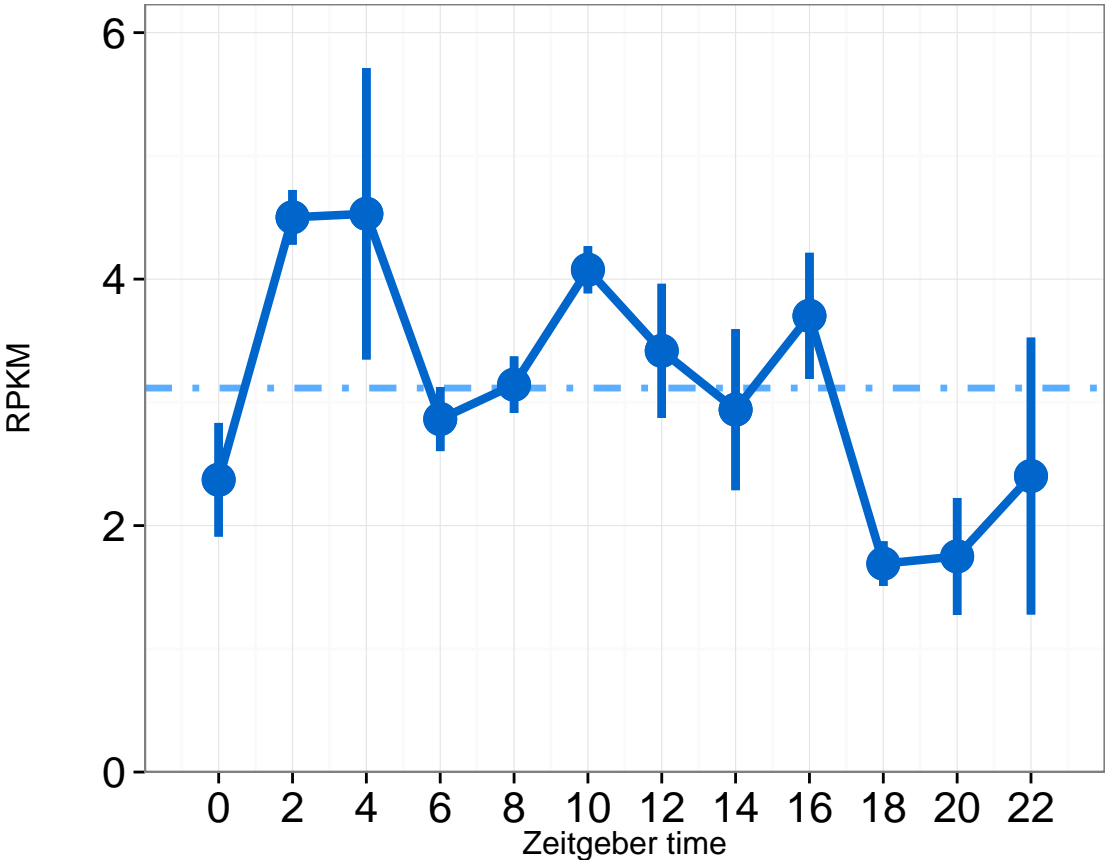

1700128F08Rik log2(Ribosome occupancy)

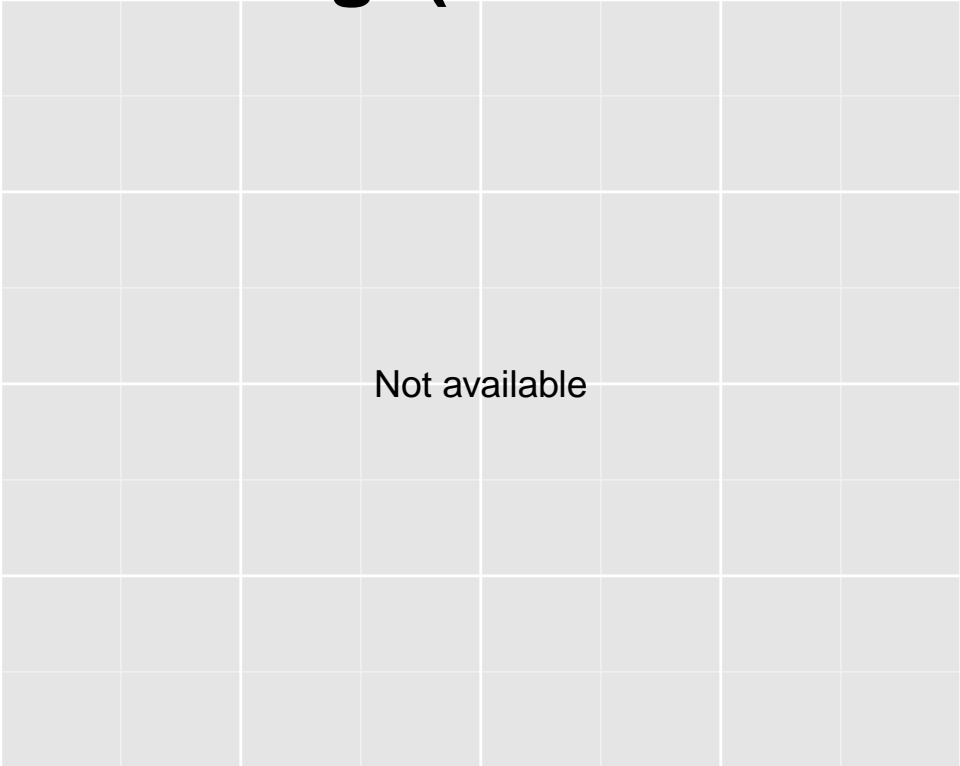

Supplement: Supplementary file 6 — Transcriptome-wide kidney RPF (blue) and RNA (orange) levels in the left panels (with “error bars” connecting the two replicates of each timepoint) and TE in the right panels. (ZIP 116896 kb) [file 13059_2017_1222_MOESM6_ESM.zip › Supp_Dataset_S1/A_RNA_non_rhythmic_RPF_non_rhythmic/1700128F08Rik_kidney_set_A.pdf]

# 1810009A15Rik

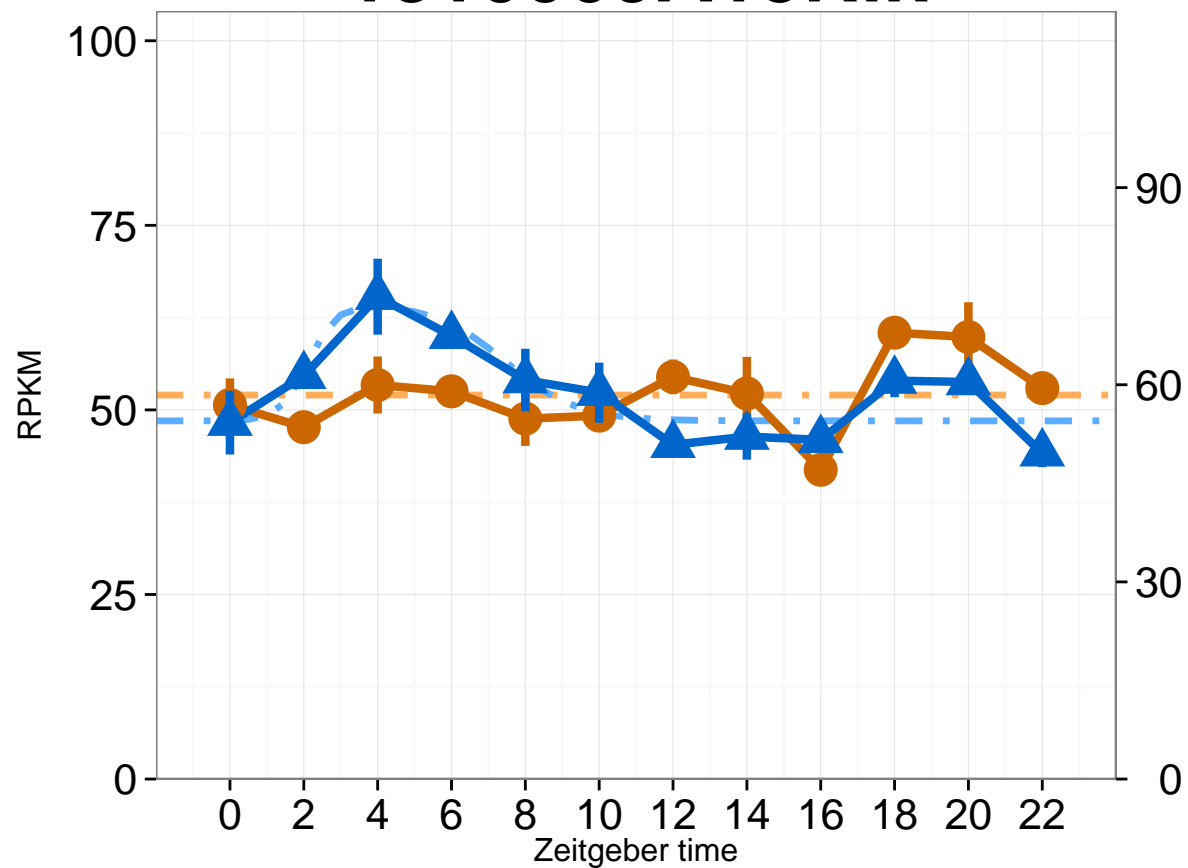

# 1810009A15Rik

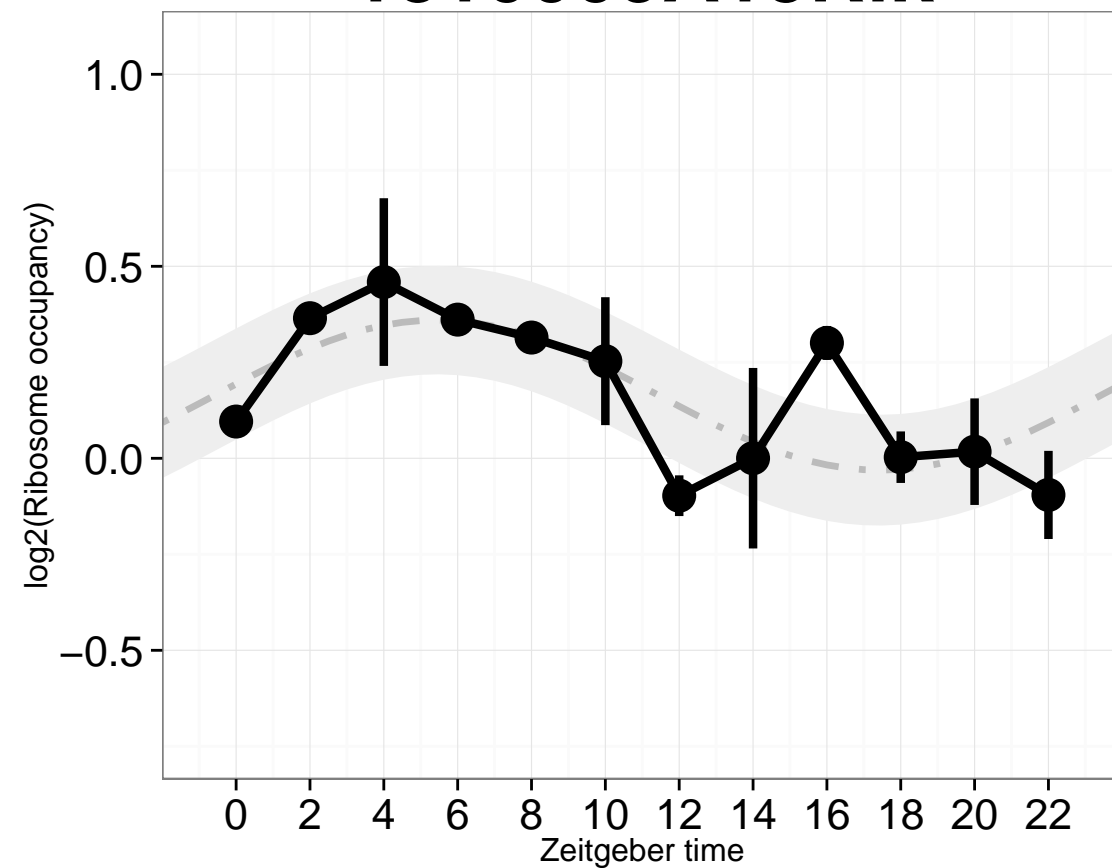

Supplement: Supplementary file 6 — Transcriptome-wide kidney RPF (blue) and RNA (orange) levels in the left panels (with “error bars” connecting the two replicates of each timepoint) and TE in the right panels. (ZIP 116896 kb) [file 13059_2017_1222_MOESM6_ESM.zip › Supp_Dataset_S1/A_RNA_non_rhythmic_RPF_non_rhythmic/1810009A15Rik_kidney_set_A.pdf]

# 1810013L24Rik

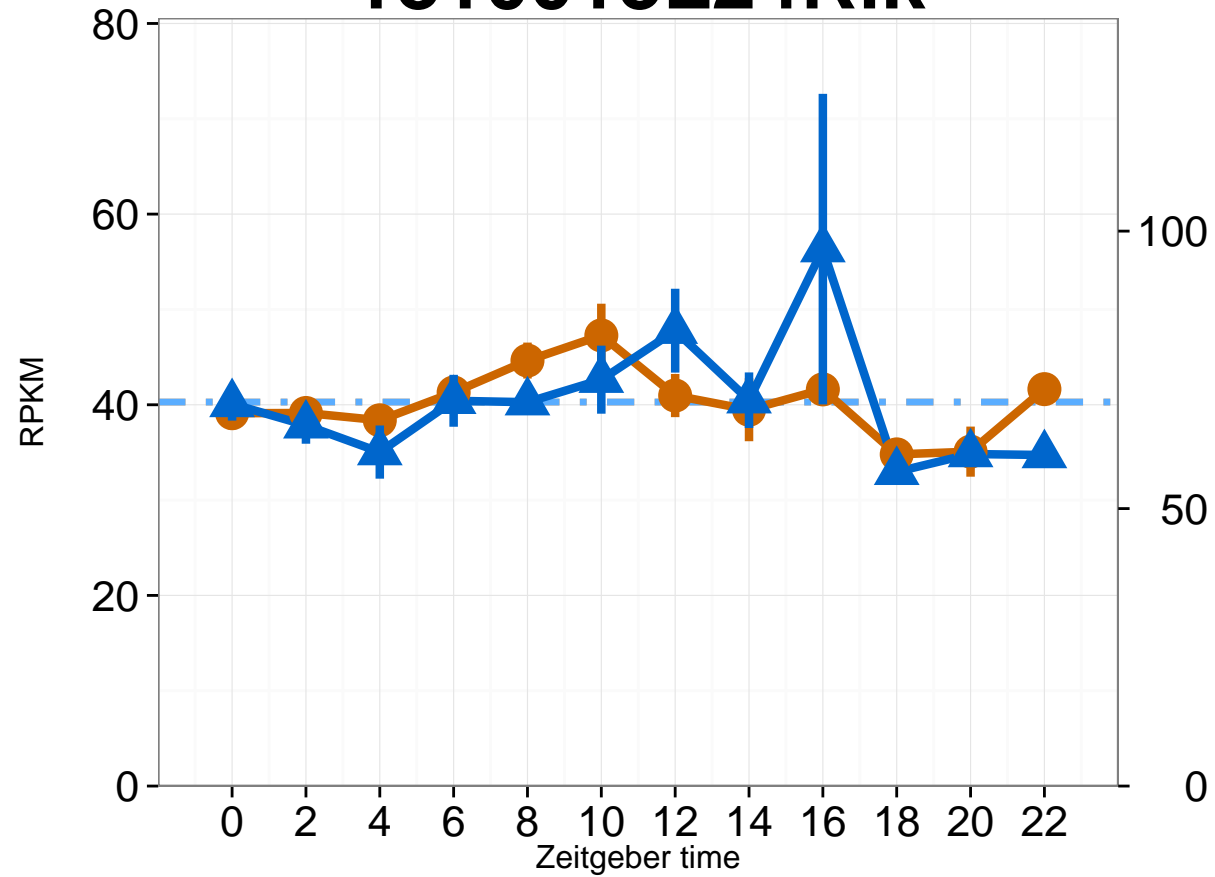

# 1810013L24Rik

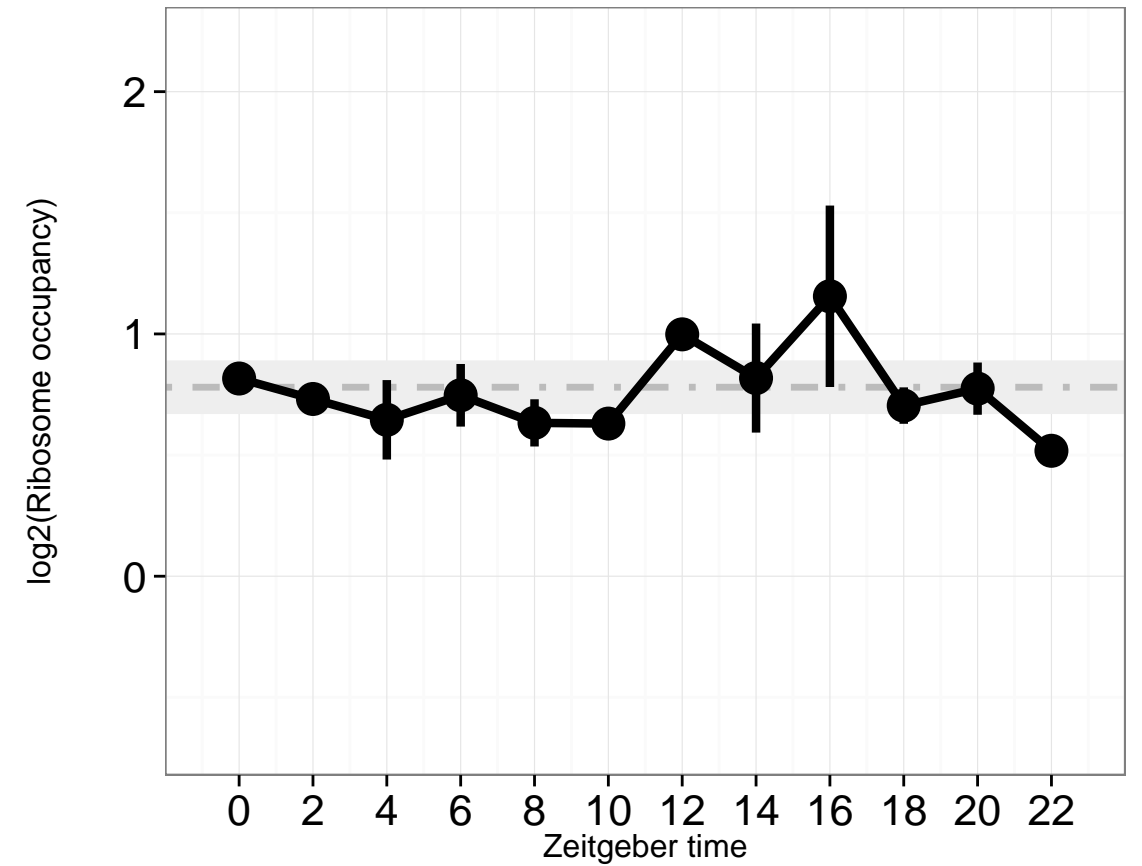

Supplement: Supplementary file 6 — Transcriptome-wide kidney RPF (blue) and RNA (orange) levels in the left panels (with “error bars” connecting the two replicates of each timepoint) and TE in the right panels. (ZIP 116896 kb) [file 13059_2017_1222_MOESM6_ESM.zip › Supp_Dataset_S1/A_RNA_non_rhythmic_RPF_non_rhythmic/1810013L24Rik_kidney_set_A.pdf]

# 1810019J16Rik

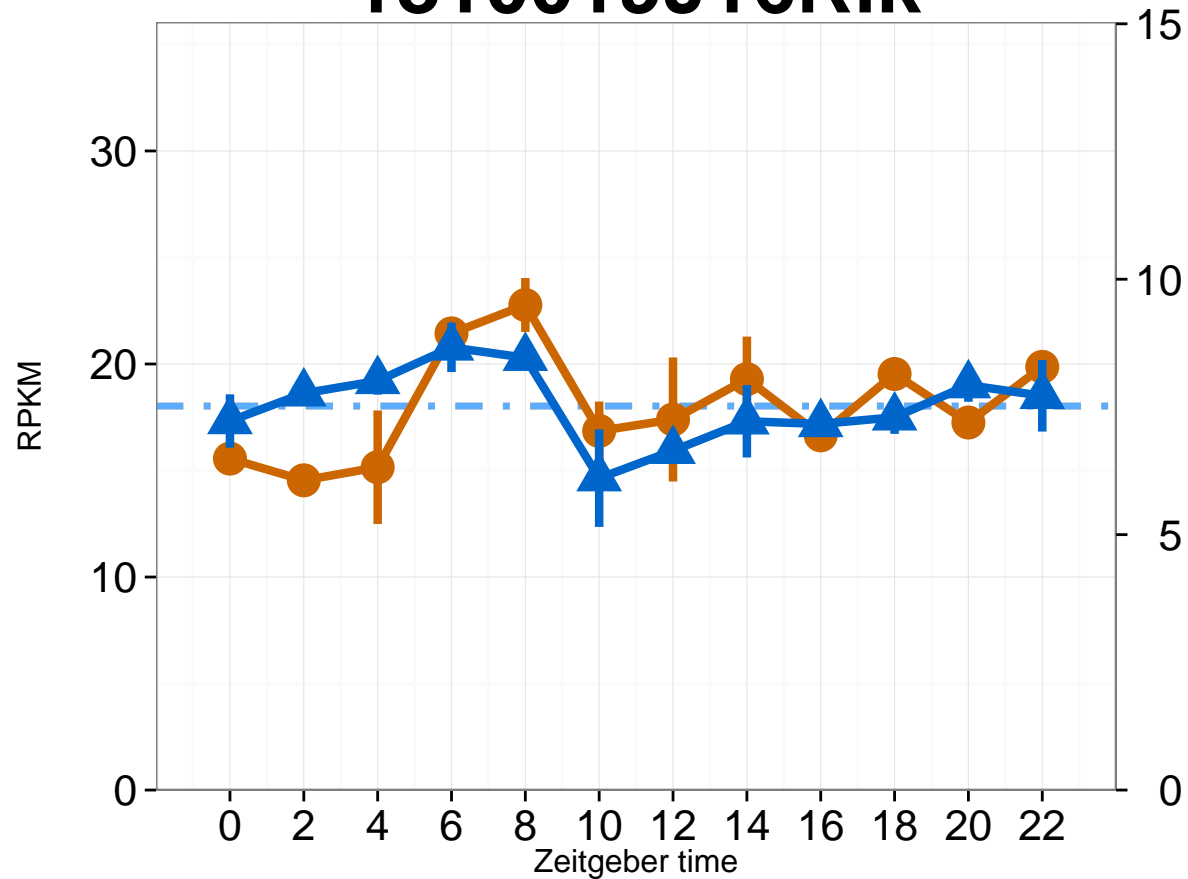

# 1810019J16Rik

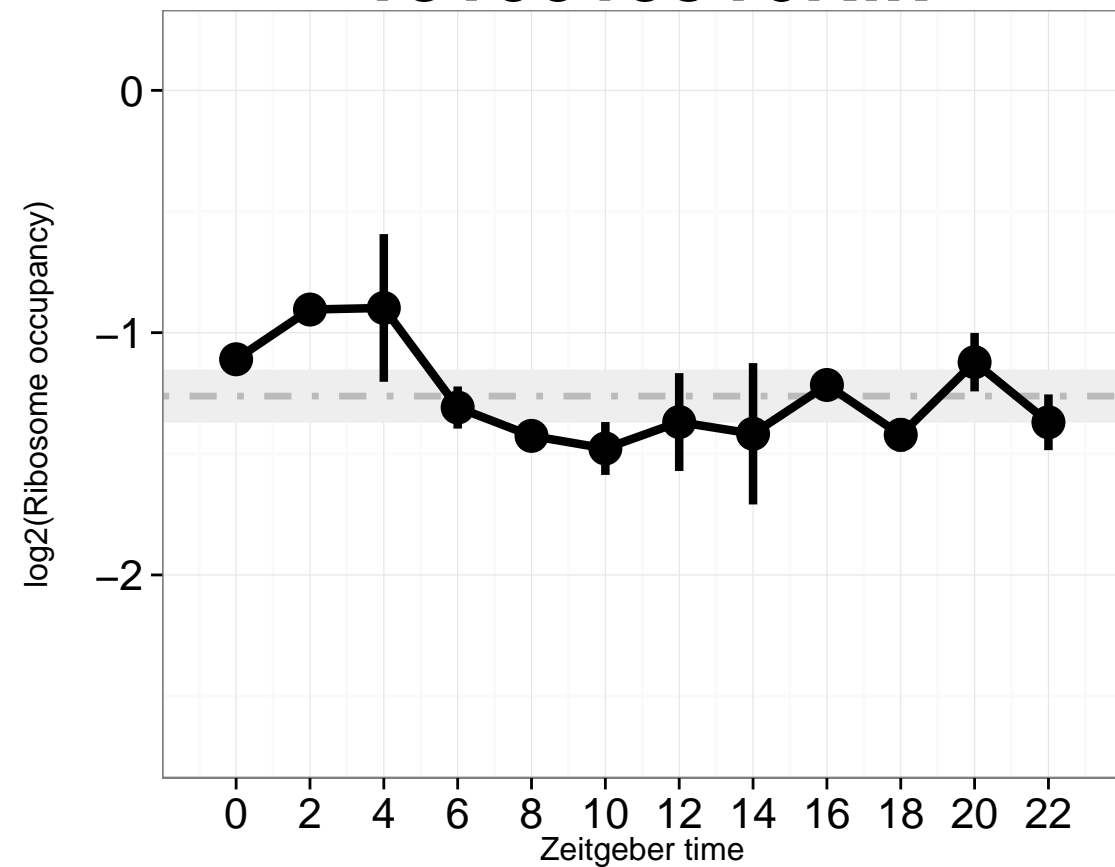

Supplement: Supplementary file 6 — Transcriptome-wide kidney RPF (blue) and RNA (orange) levels in the left panels (with “error bars” connecting the two replicates of each timepoint) and TE in the right panels. (ZIP 116896 kb) [file 13059_2017_1222_MOESM6_ESM.zip › Supp_Dataset_S1/A_RNA_non_rhythmic_RPF_non_rhythmic/1810019J16Rik_kidney_set_A.pdf]

# 1810026J23Rik

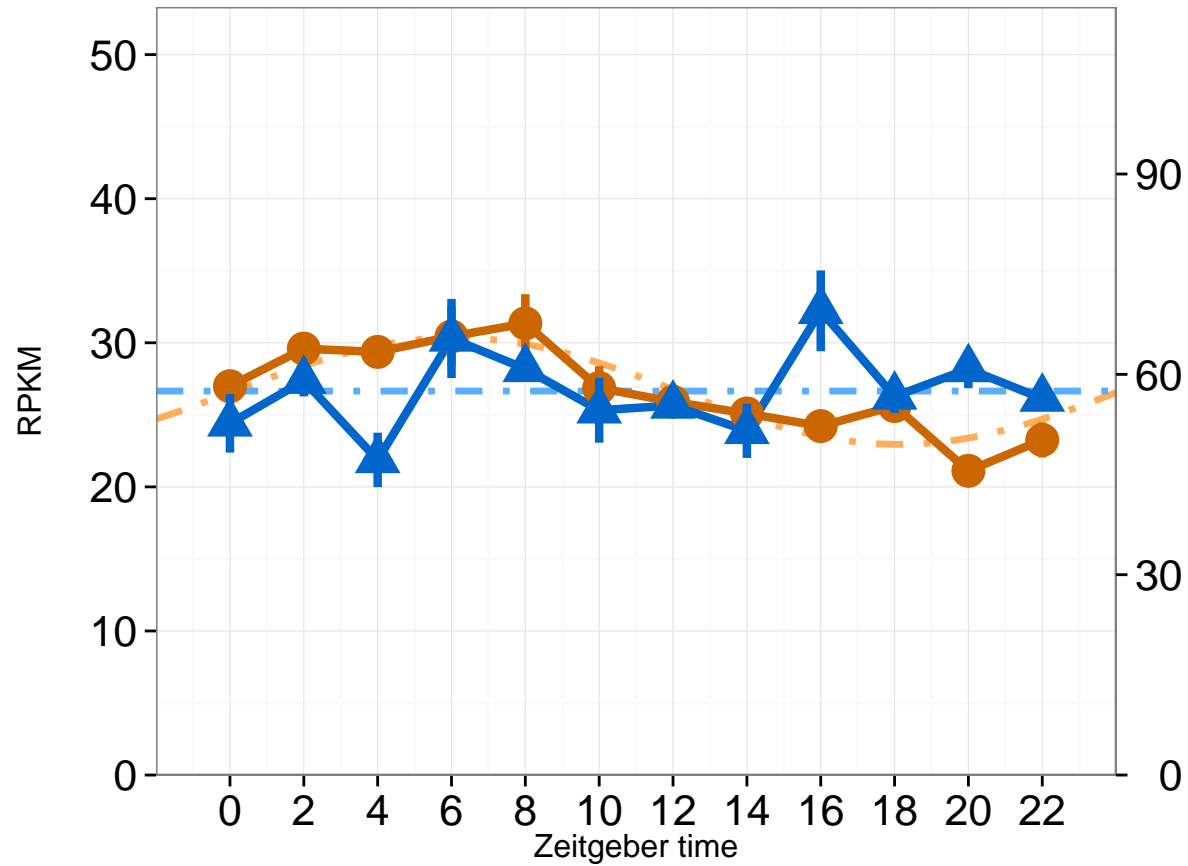

# 1810026J23Rik

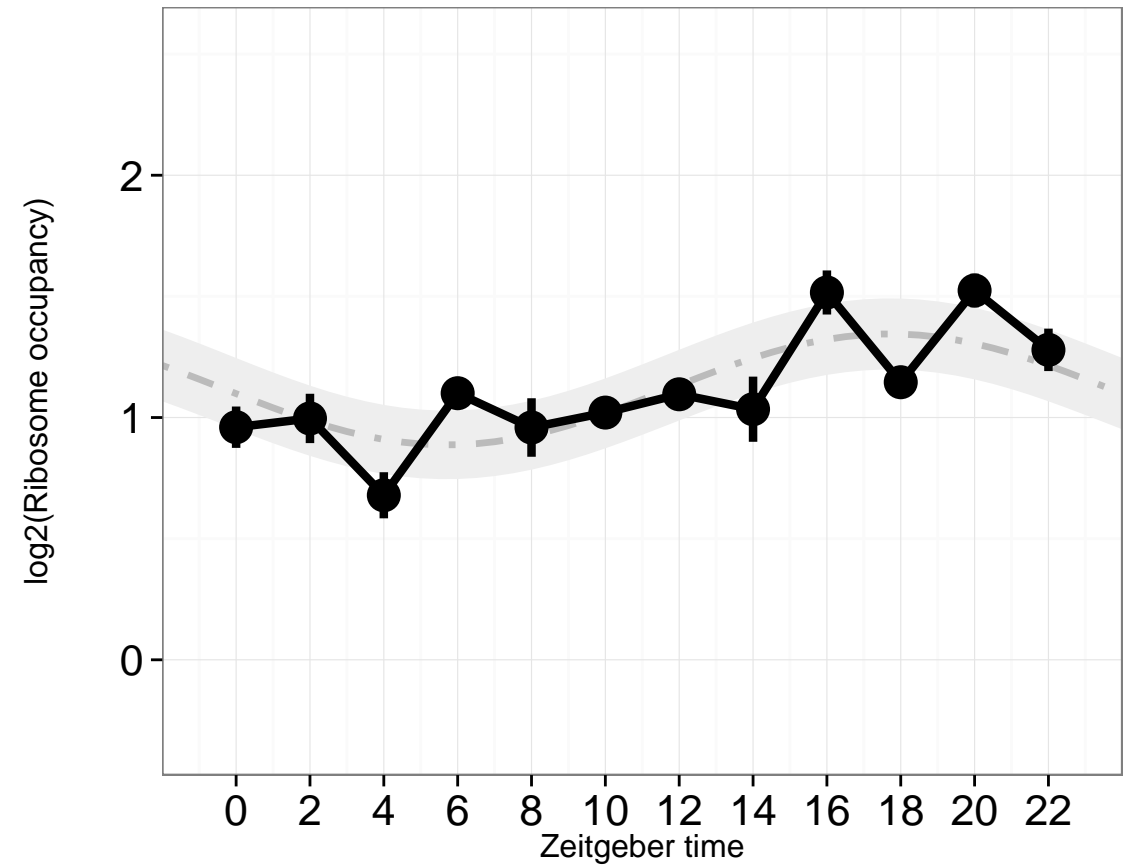

Supplement: Supplementary file 6 — Transcriptome-wide kidney RPF (blue) and RNA (orange) levels in the left panels (with “error bars” connecting the two replicates of each timepoint) and TE in the right panels. (ZIP 116896 kb) [file 13059_2017_1222_MOESM6_ESM.zip › Supp_Dataset_S1/A_RNA_non_rhythmic_RPF_non_rhythmic/1810026J23Rik_kidney_set_A.pdf]

# 1810032O08Rik

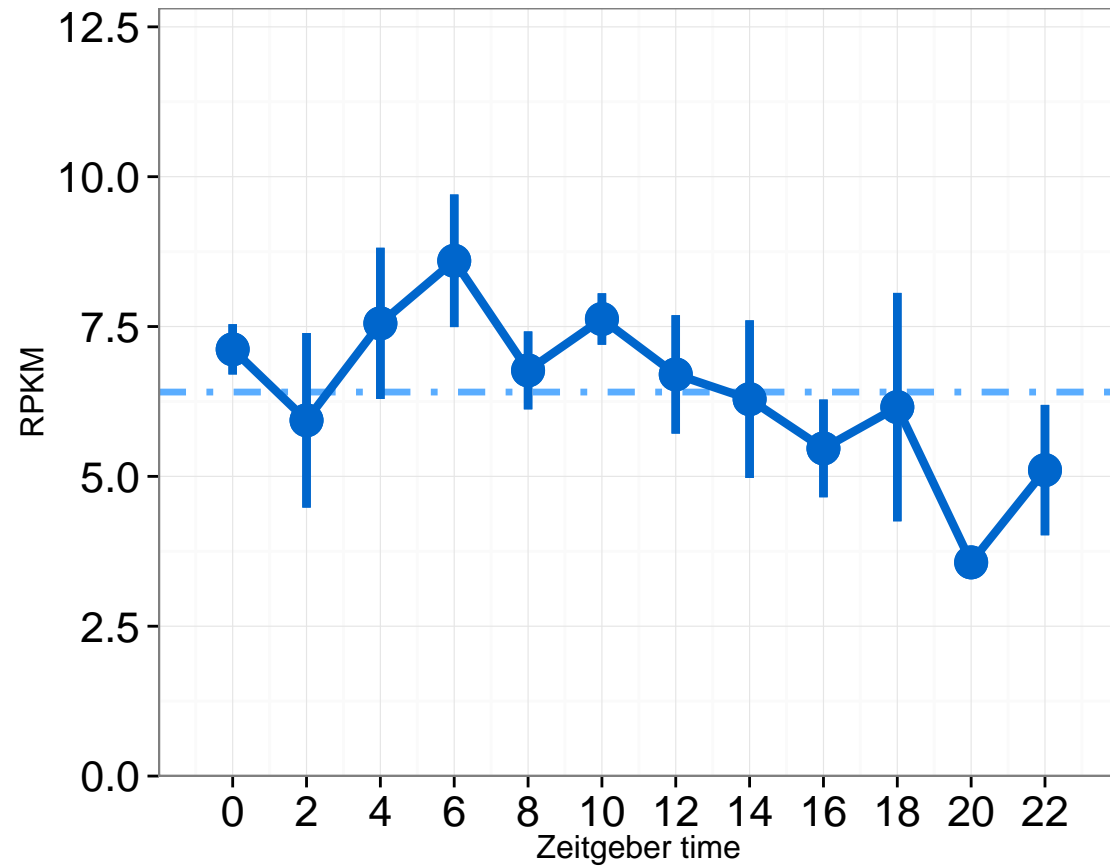

# 1810032O08Rik log2(Ribosome occup

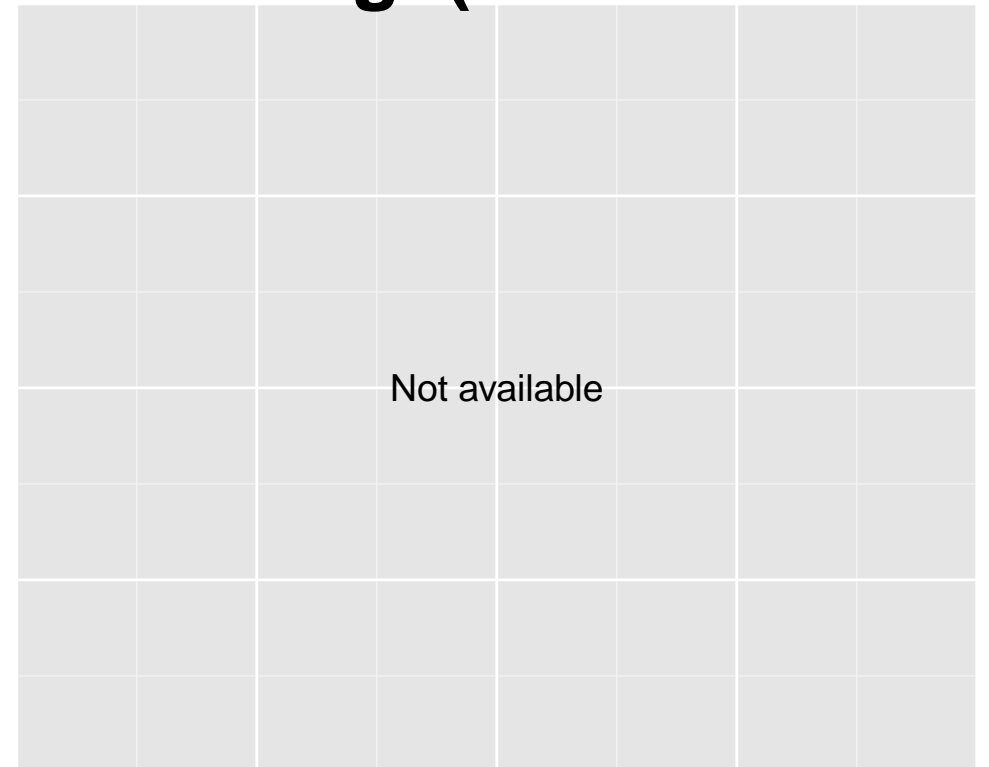

Supplement: Supplementary file 6 — Transcriptome-wide kidney RPF (blue) and RNA (orange) levels in the left panels (with “error bars” connecting the two replicates of each timepoint) and TE in the right panels. (ZIP 116896 kb) [file 13059_2017_1222_MOESM6_ESM.zip › Supp_Dataset_S1/A_RNA_non_rhythmic_RPF_non_rhythmic/1810032O08Rik_kidney_set_A.pdf]

# 1810037I17Rik

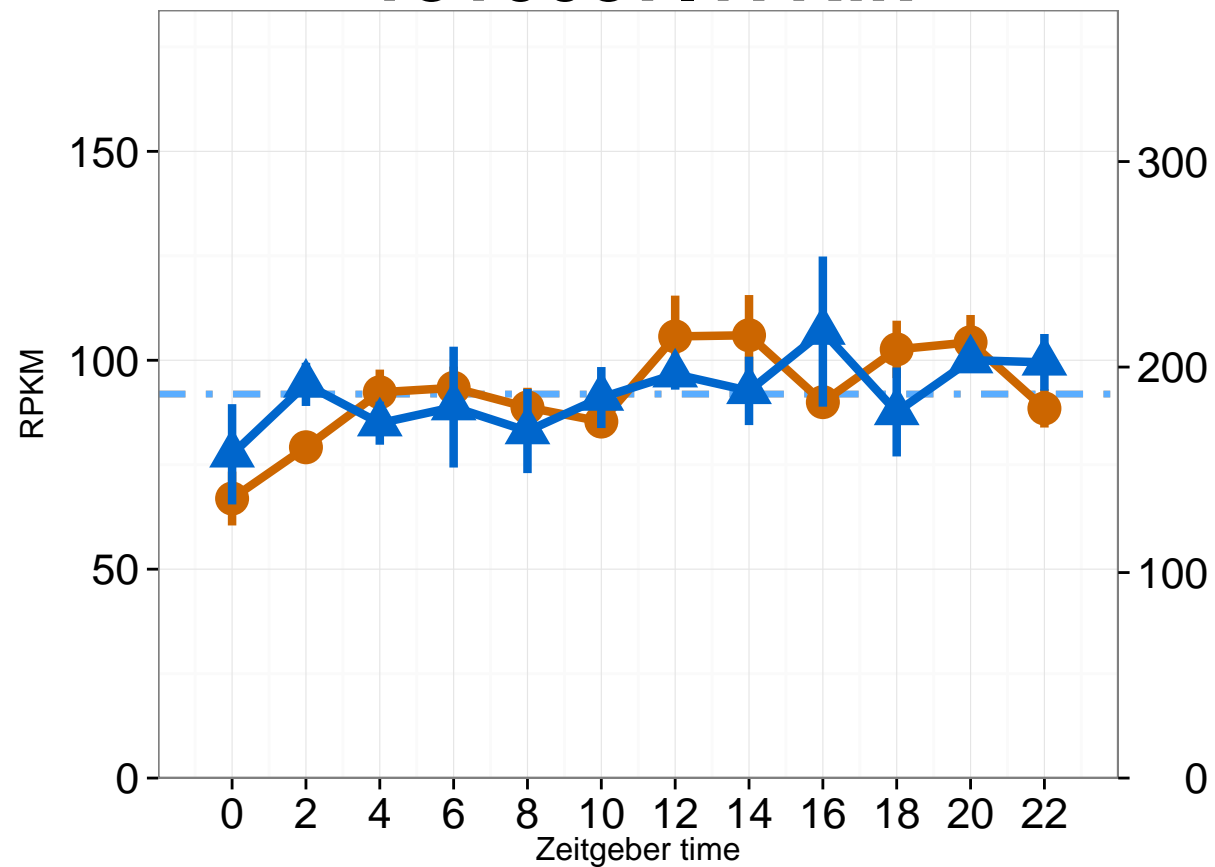

# 1810037I17Rik

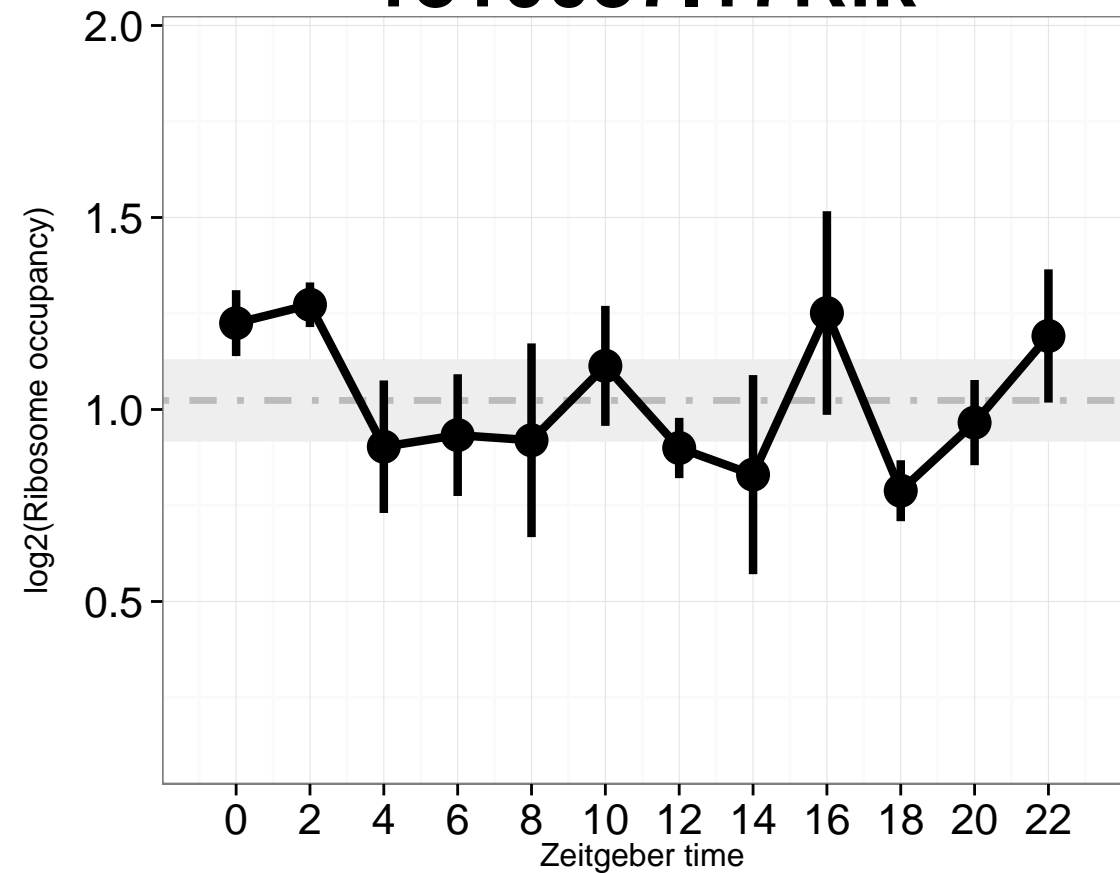

Supplement: Supplementary file 6 — Transcriptome-wide kidney RPF (blue) and RNA (orange) levels in the left panels (with “error bars” connecting the two replicates of each timepoint) and TE in the right panels. (ZIP 116896 kb) [file 13059_2017_1222_MOESM6_ESM.zip › Supp_Dataset_S1/A_RNA_non_rhythmic_RPF_non_rhythmic/1810037I17Rik_kidney_set_A.pdf]

## 1810055G02Rik

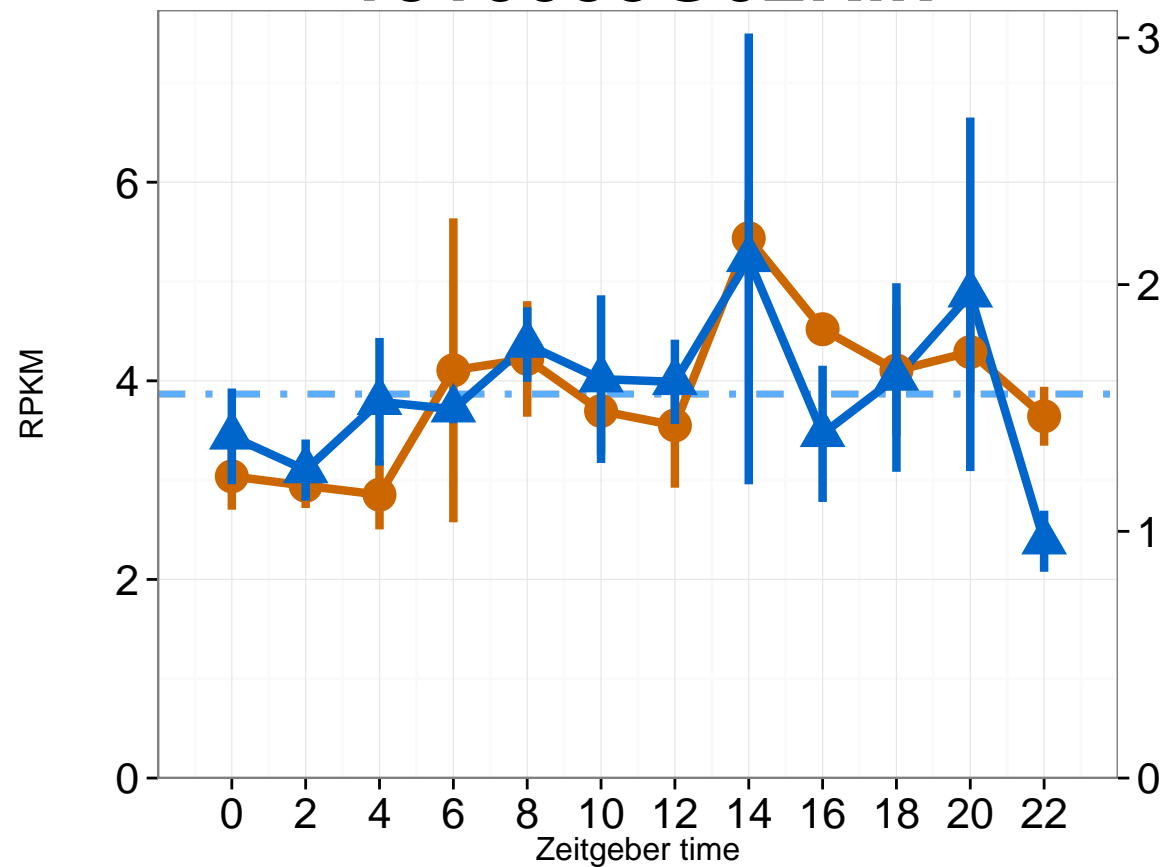

## 1810055G02Rik

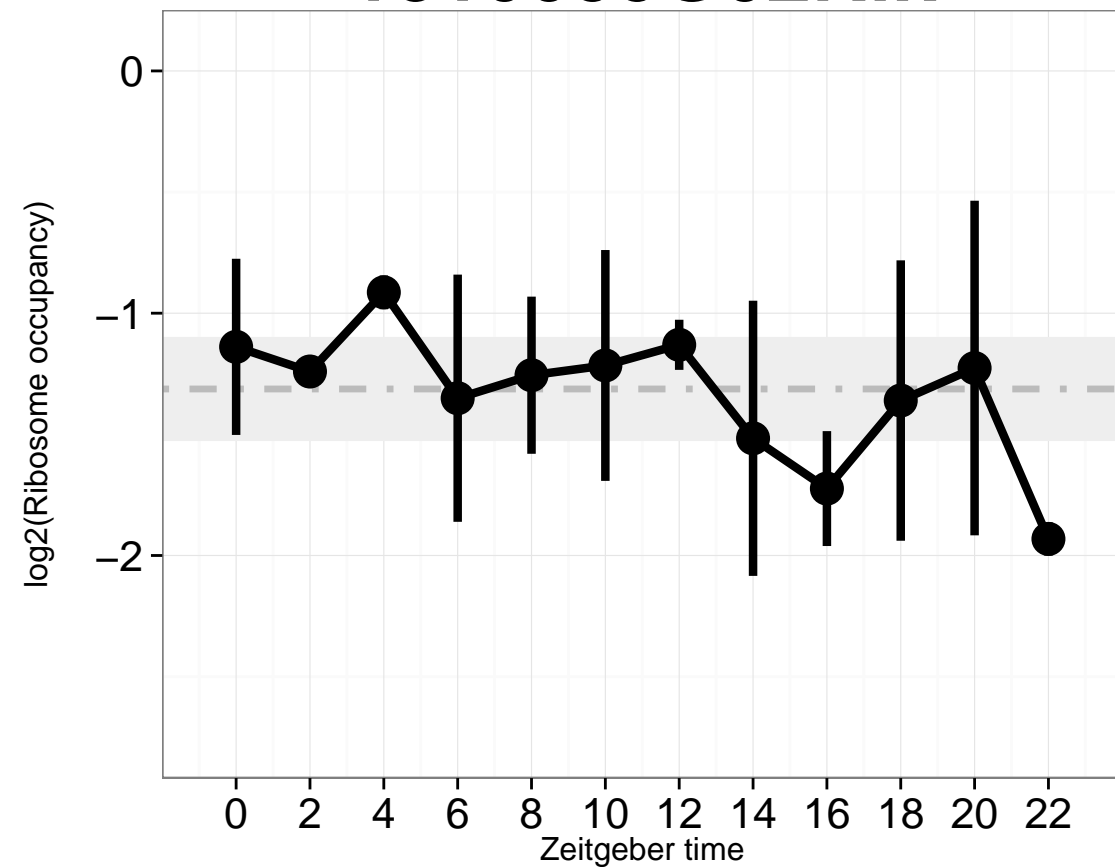

Supplement: Supplementary file 6 — Transcriptome-wide kidney RPF (blue) and RNA (orange) levels in the left panels (with “error bars” connecting the two replicates of each timepoint) and TE in the right panels. (ZIP 116896 kb) [file 13059_2017_1222_MOESM6_ESM.zip › Supp_Dataset_S1/A_RNA_non_rhythmic_RPF_non_rhythmic/1810055G02Rik_kidney_set_A.pdf]

# 2010003K11Rik

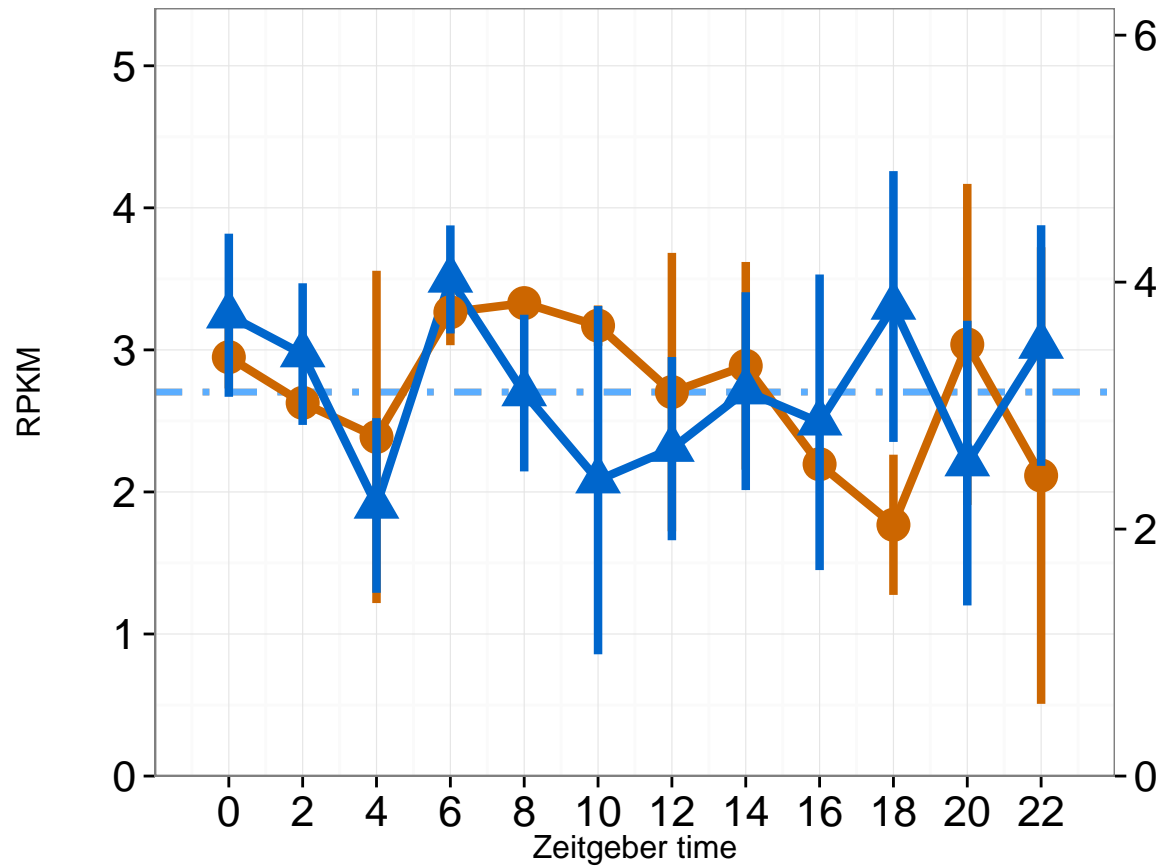

# 2010003K11Rik

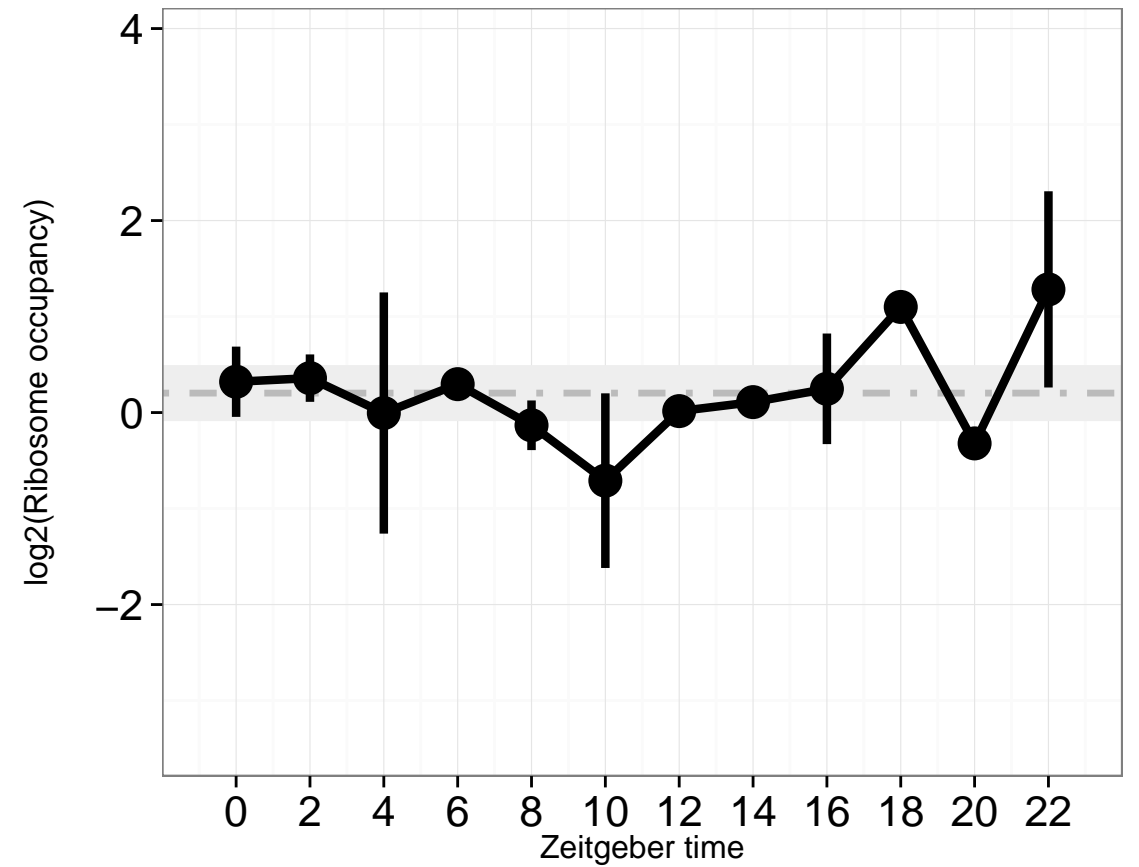

Supplement: Supplementary file 6 — Transcriptome-wide kidney RPF (blue) and RNA (orange) levels in the left panels (with “error bars” connecting the two replicates of each timepoint) and TE in the right panels. (ZIP 116896 kb) [file 13059_2017_1222_MOESM6_ESM.zip › Supp_Dataset_S1/A_RNA_non_rhythmic_RPF_non_rhythmic/2010003K11Rik_kidney_set_A.pdf]

## 2010012005Rik

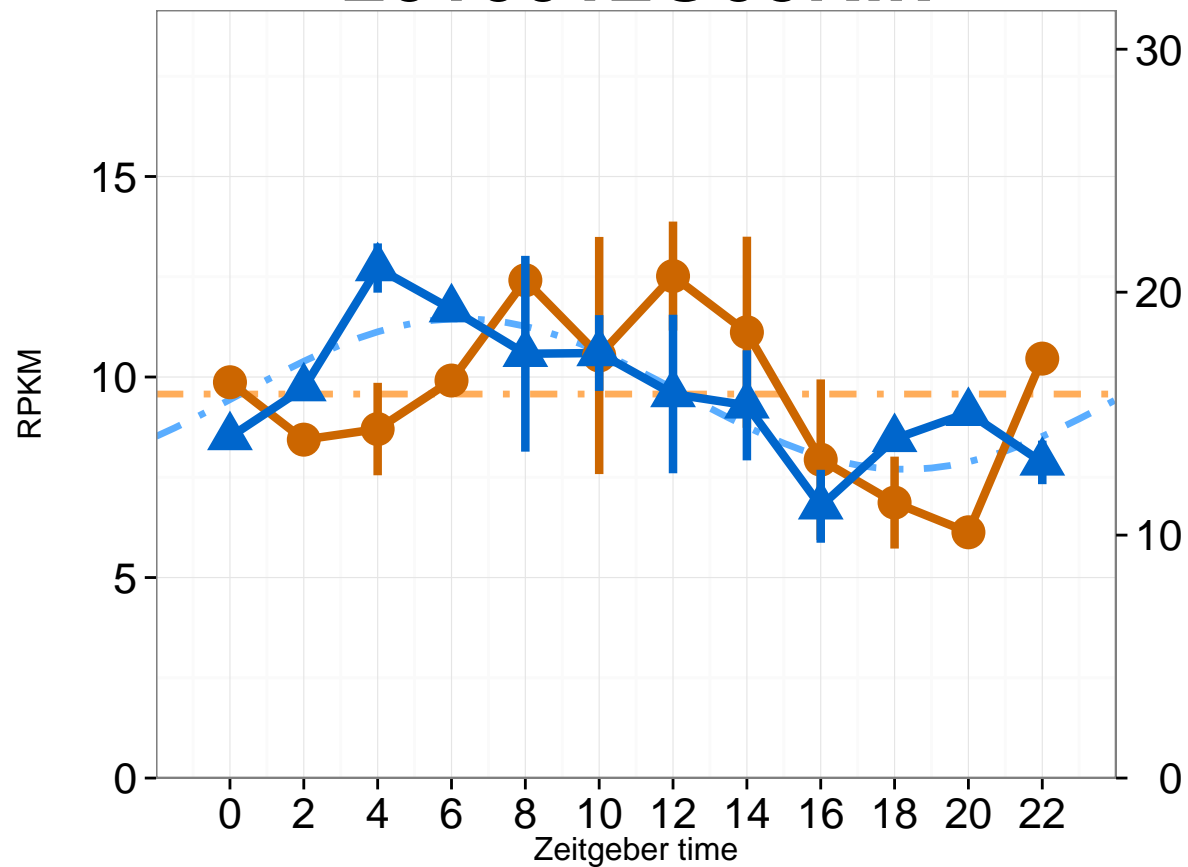

## 2010012005Rik

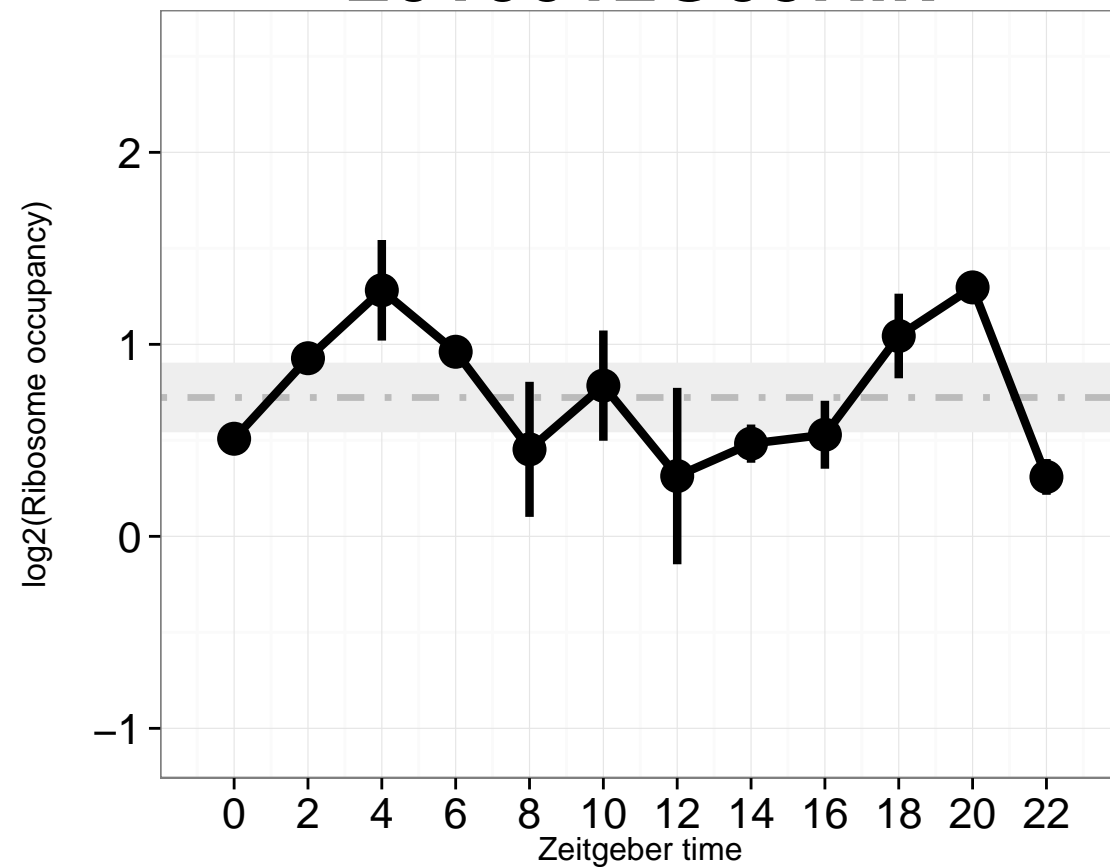

Supplement: Supplementary file 6 — Transcriptome-wide kidney RPF (blue) and RNA (orange) levels in the left panels (with “error bars” connecting the two replicates of each timepoint) and TE in the right panels. (ZIP 116896 kb) [file 13059_2017_1222_MOESM6_ESM.zip › Supp_Dataset_S1/A_RNA_non_rhythmic_RPF_non_rhythmic/2010012O05Rik_kidney_set_A.pdf]

# 2010107E04Rik

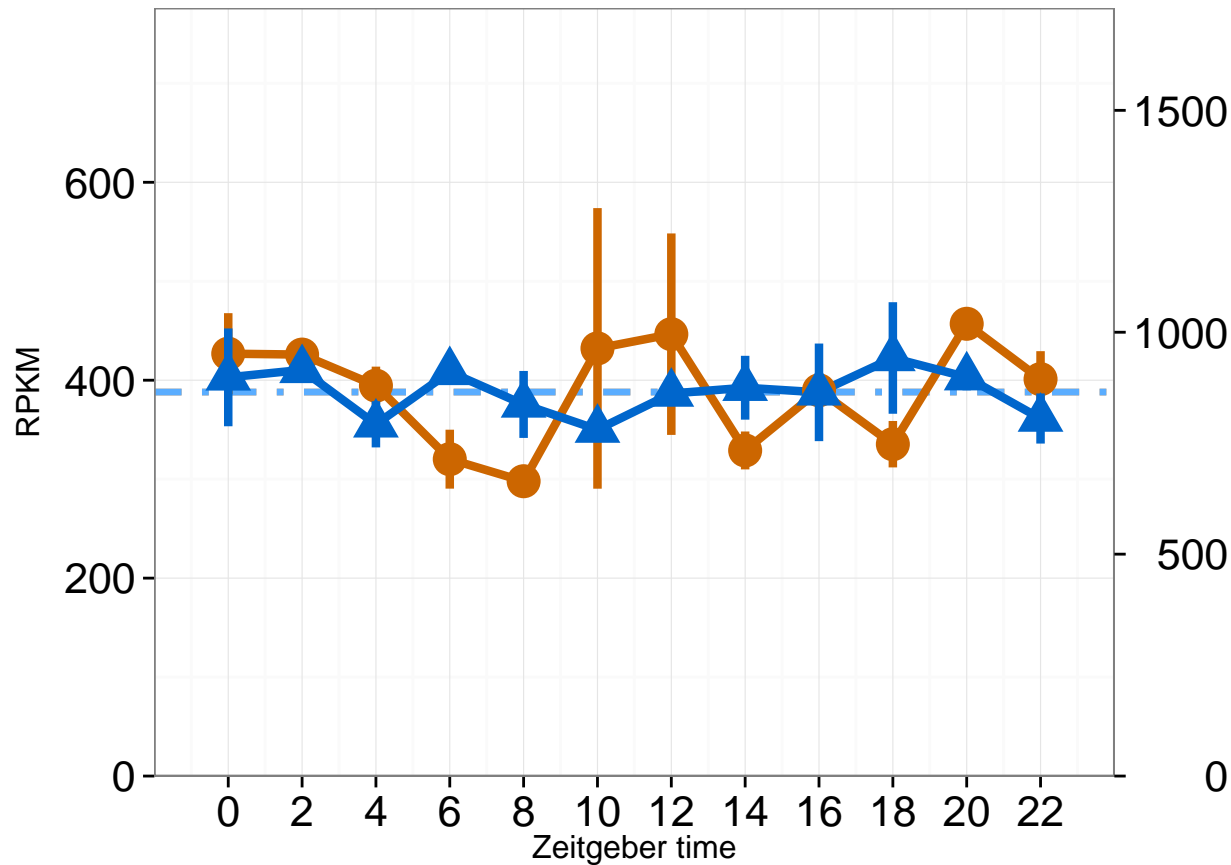

# 2010107E04Rik

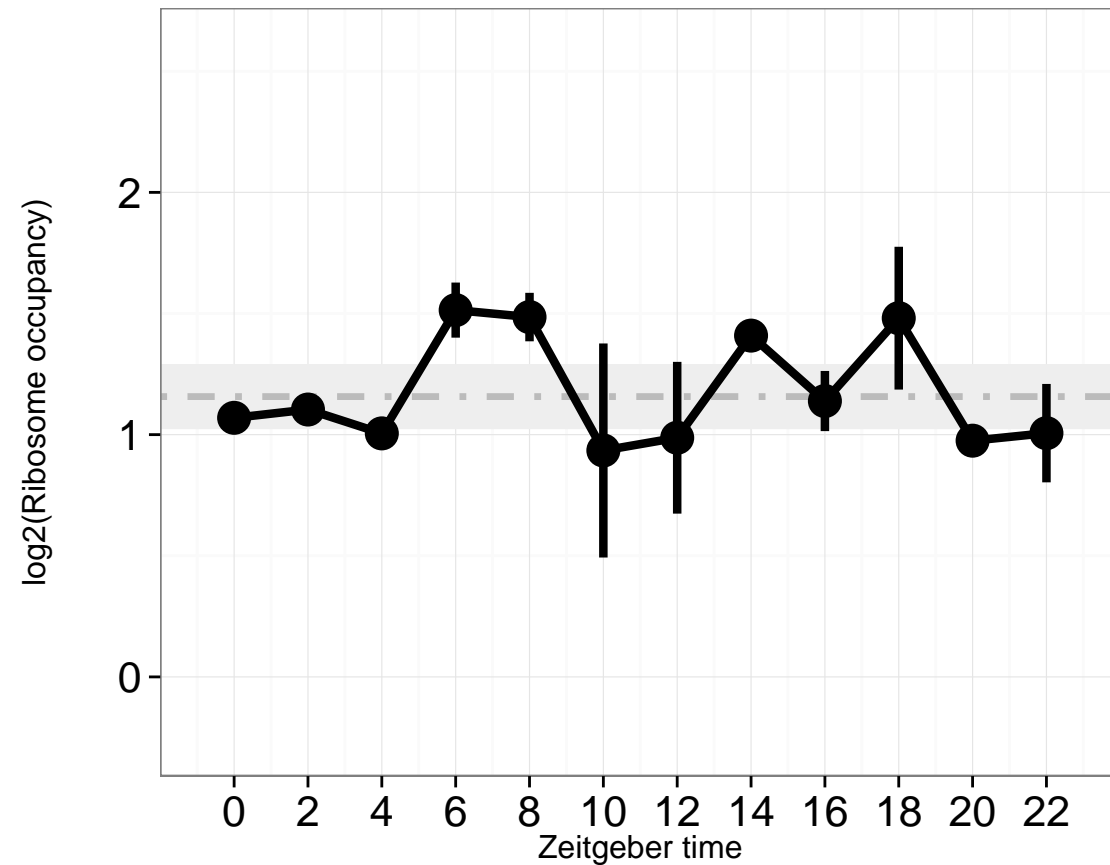

Supplement: Supplementary file 6 — Transcriptome-wide kidney RPF (blue) and RNA (orange) levels in the left panels (with “error bars” connecting the two replicates of each timepoint) and TE in the right panels. (ZIP 116896 kb) [file 13059_2017_1222_MOESM6_ESM.zip › Supp_Dataset_S1/A_RNA_non_rhythmic_RPF_non_rhythmic/2010107E04Rik_kidney_set_A.pdf]

# 2010107G23Rik

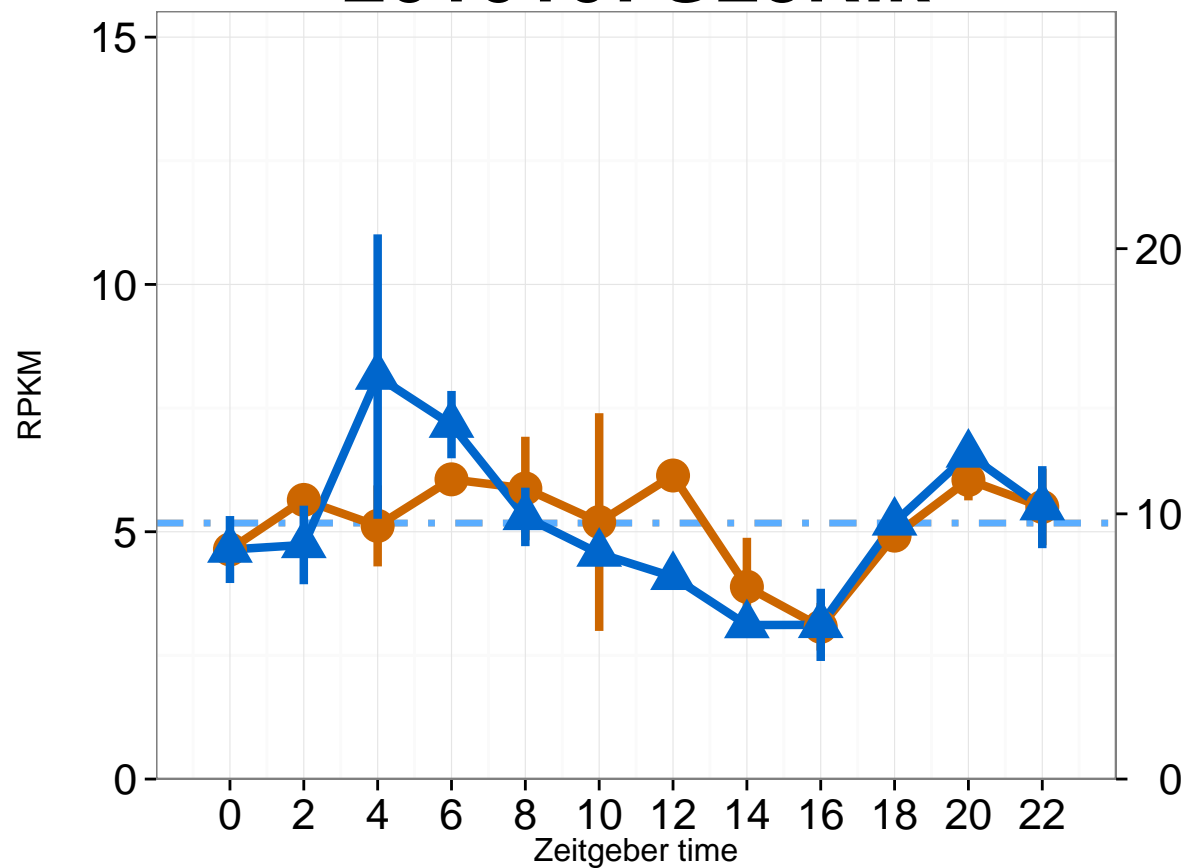

# 2010107G23Rik

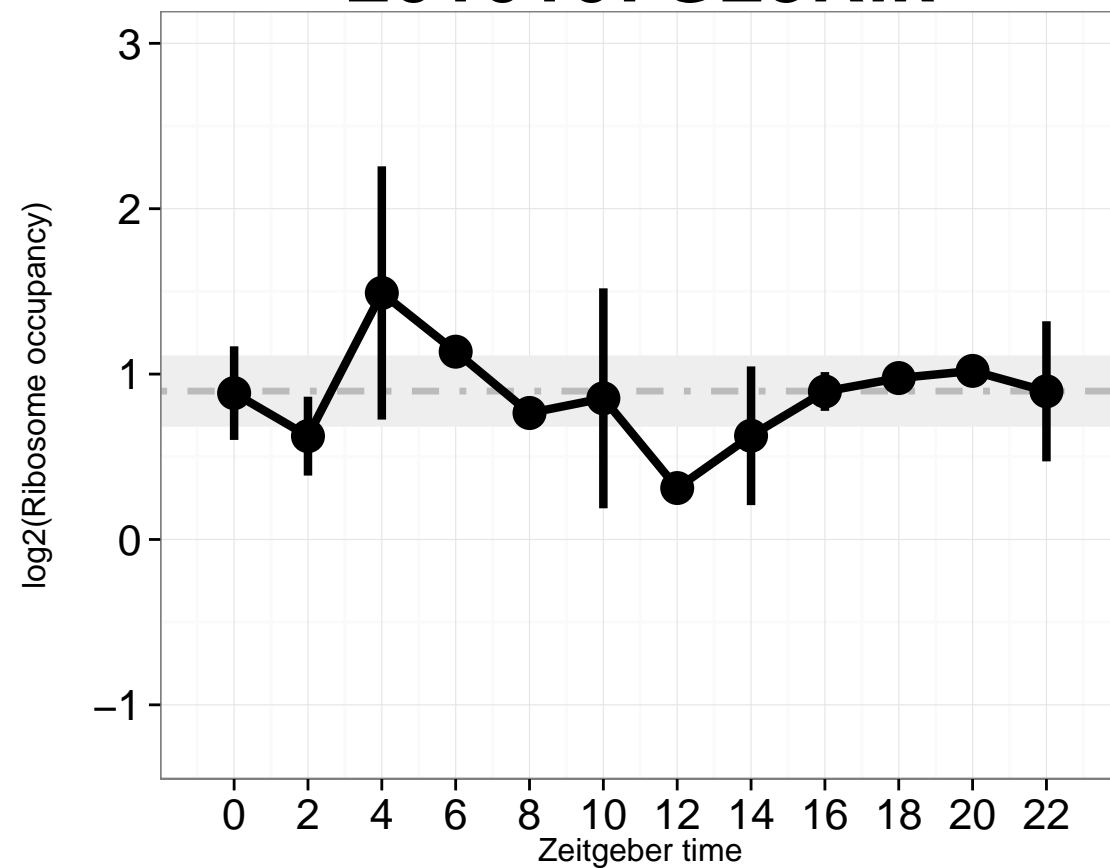

Supplement: Supplementary file 6 — Transcriptome-wide kidney RPF (blue) and RNA (orange) levels in the left panels (with “error bars” connecting the two replicates of each timepoint) and TE in the right panels. (ZIP 116896 kb) [file 13059_2017_1222_MOESM6_ESM.zip › Supp_Dataset_S1/A_RNA_non_rhythmic_RPF_non_rhythmic/2010107G23Rik_kidney_set_A.pdf]

2010111I01Rik

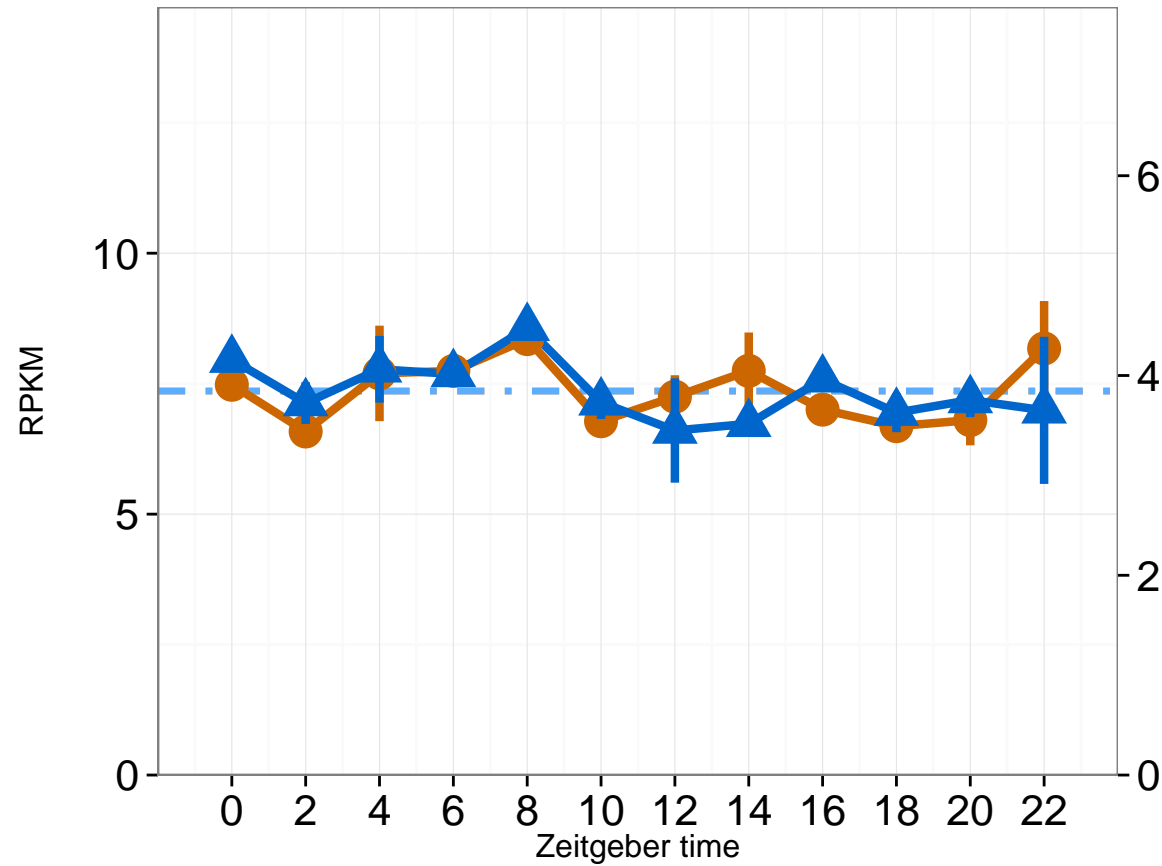

2010111I01Rik

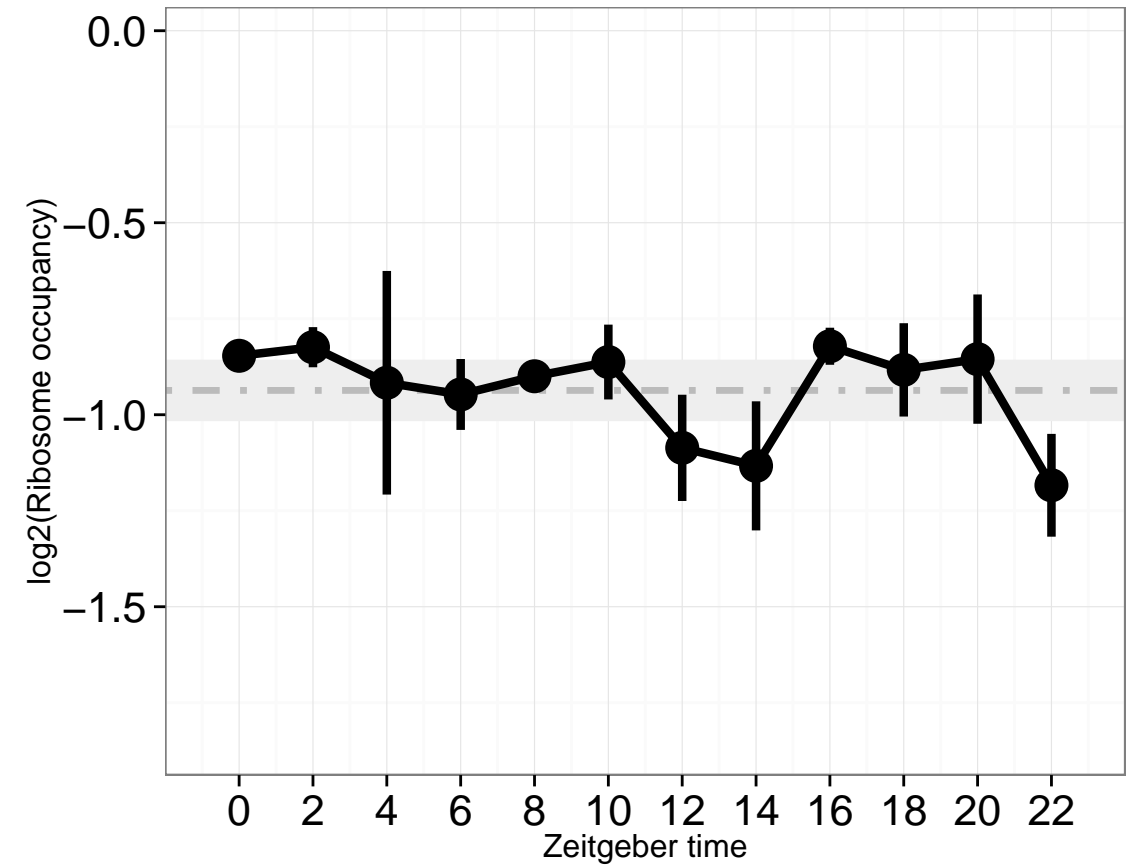

Supplement: Supplementary file 6 — Transcriptome-wide kidney RPF (blue) and RNA (orange) levels in the left panels (with “error bars” connecting the two replicates of each timepoint) and TE in the right panels. (ZIP 116896 kb) [file 13059_2017_1222_MOESM6_ESM.zip › Supp_Dataset_S1/A_RNA_non_rhythmic_RPF_non_rhythmic/2010111I01Rik_kidney_set_A.pdf]

## 2210016F16Rik

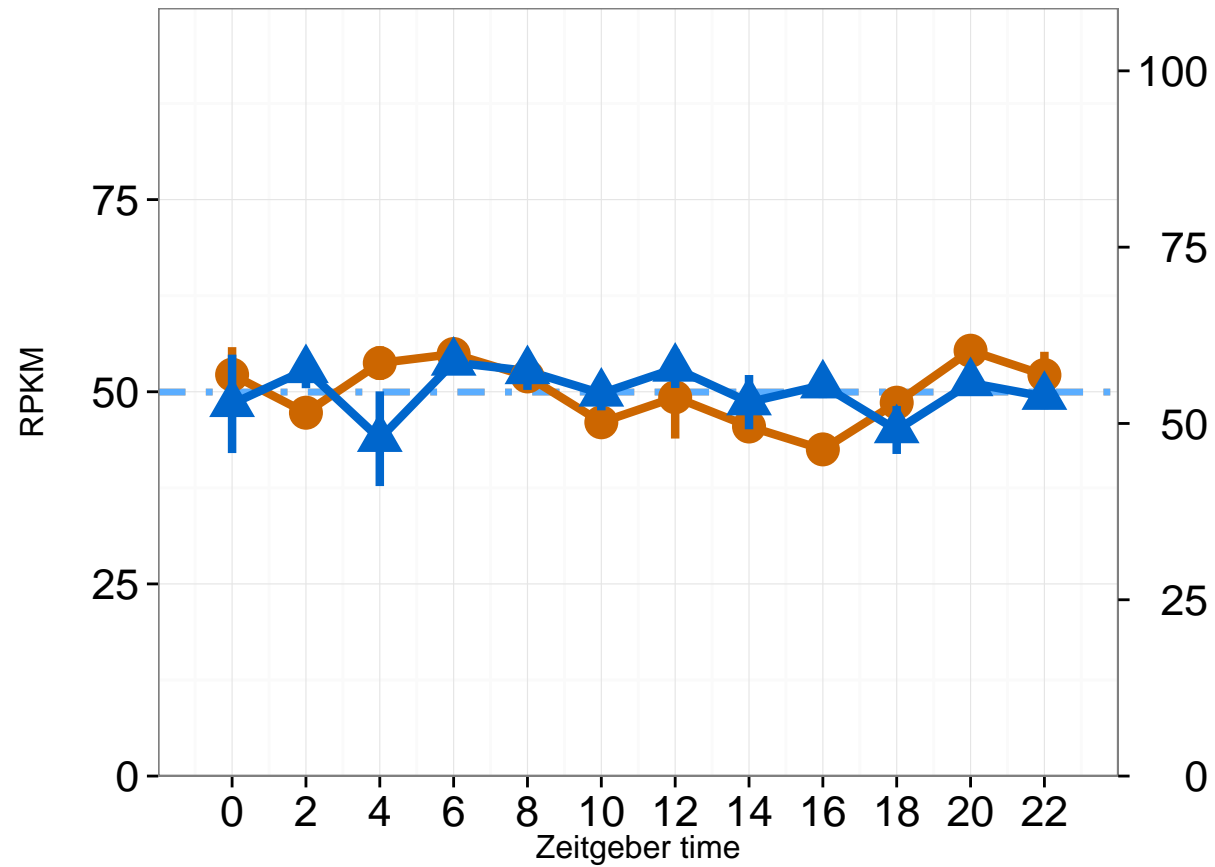

## 2210016F16Rik

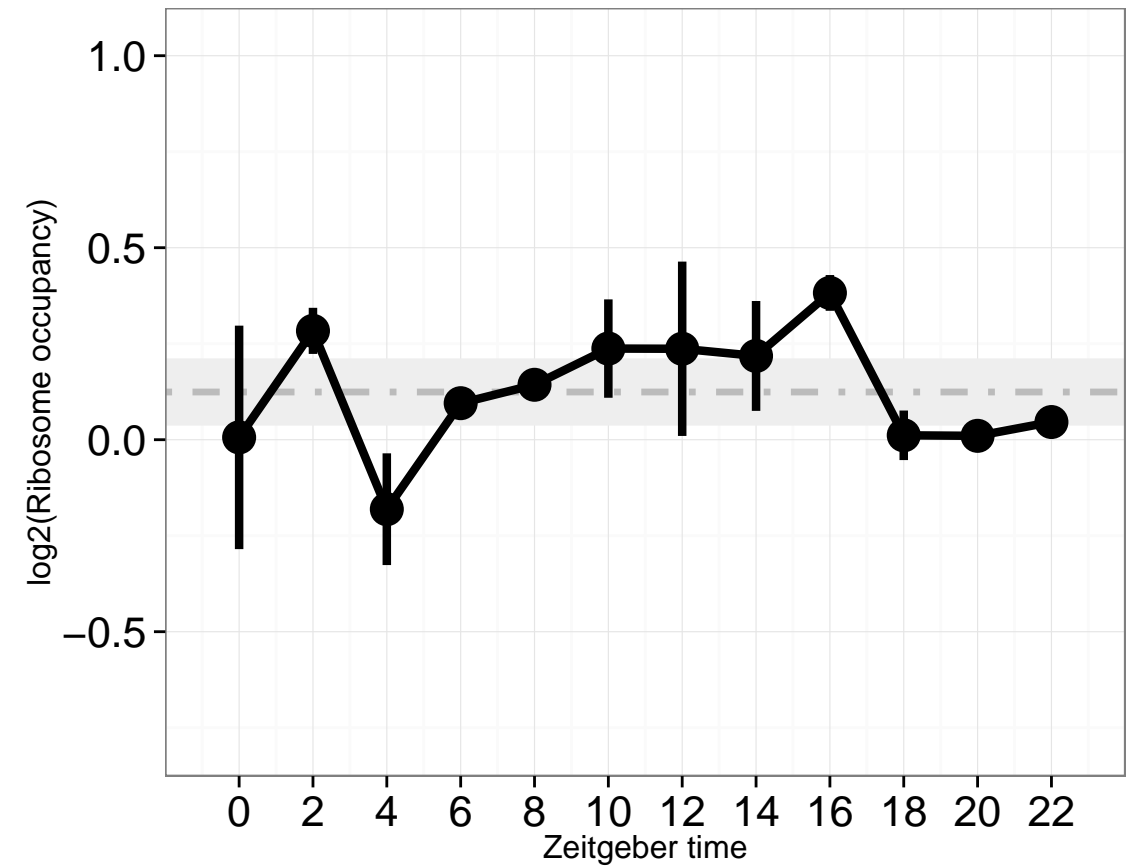

Supplement: Supplementary file 6 — Transcriptome-wide kidney RPF (blue) and RNA (orange) levels in the left panels (with “error bars” connecting the two replicates of each timepoint) and TE in the right panels. (ZIP 116896 kb) [file 13059_2017_1222_MOESM6_ESM.zip › Supp_Dataset_S1/A_RNA_non_rhythmic_RPF_non_rhythmic/2210016F16Rik_kidney_set_A.pdf]

## 2210016L21Rik

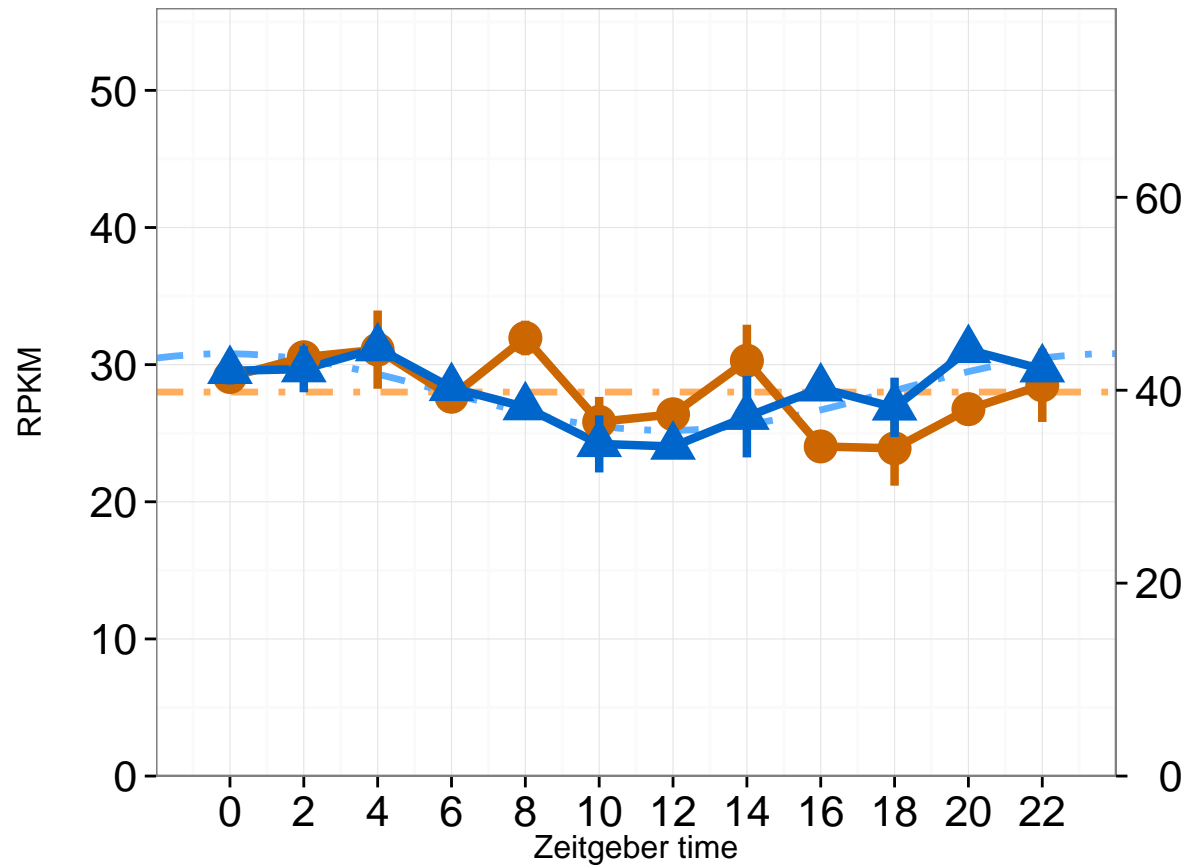

## 2210016L21Rik

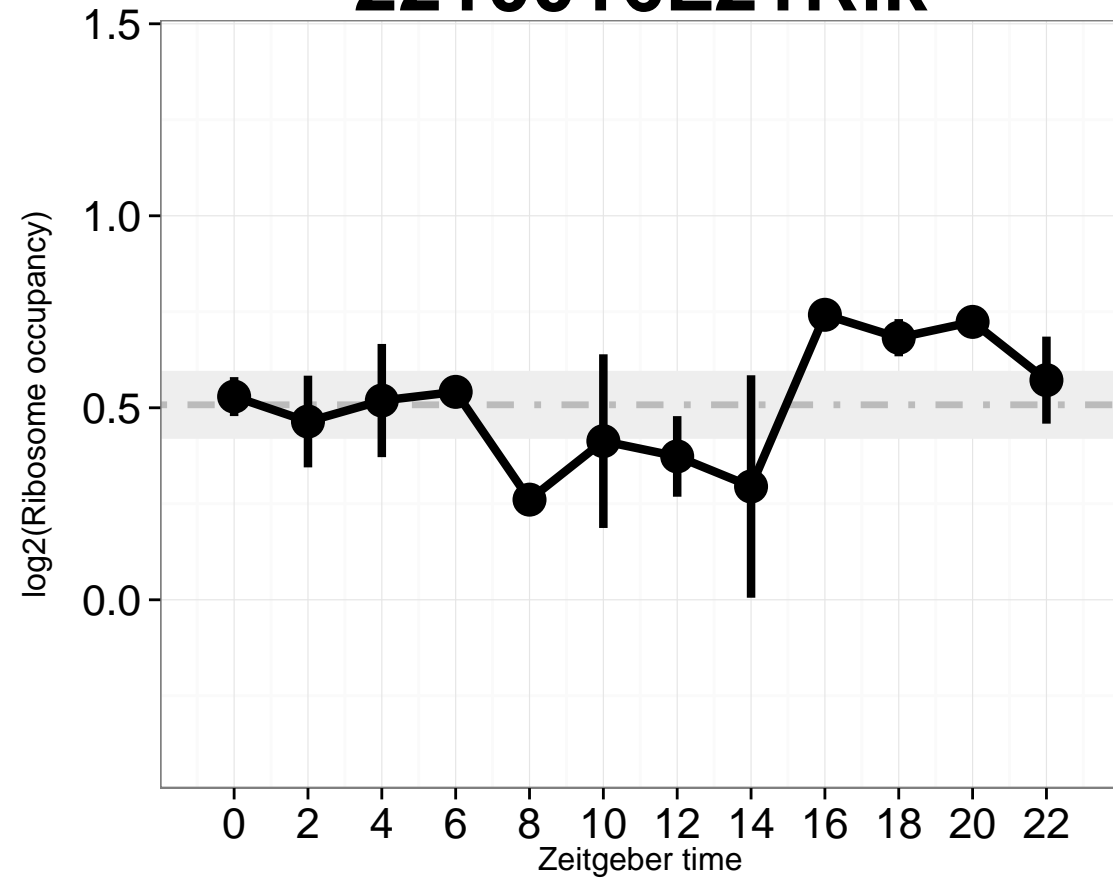

Supplement: Supplementary file 6 — Transcriptome-wide kidney RPF (blue) and RNA (orange) levels in the left panels (with “error bars” connecting the two replicates of each timepoint) and TE in the right panels. (ZIP 116896 kb) [file 13059_2017_1222_MOESM6_ESM.zip › Supp_Dataset_S1/A_RNA_non_rhythmic_RPF_non_rhythmic/2210016L21Rik_kidney_set_A.pdf]

# 2210018M11Rik

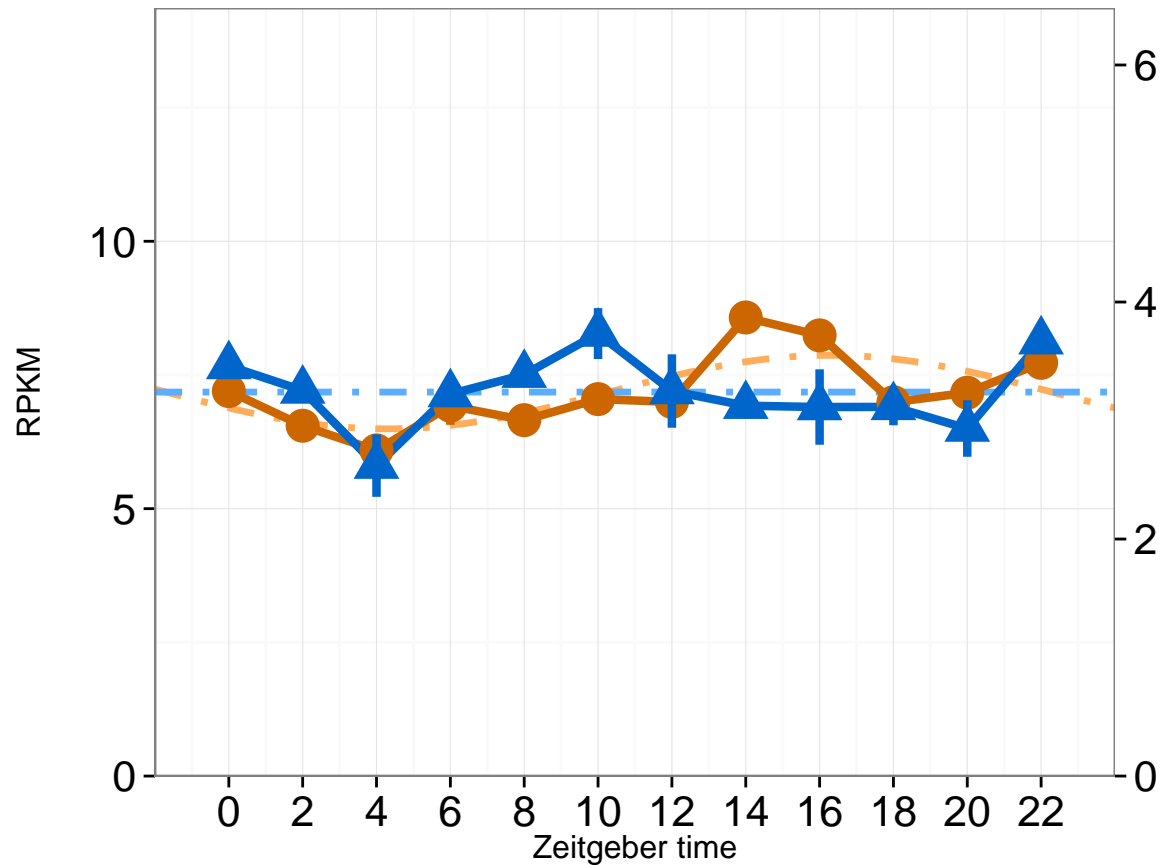

# 2210018M11Rik

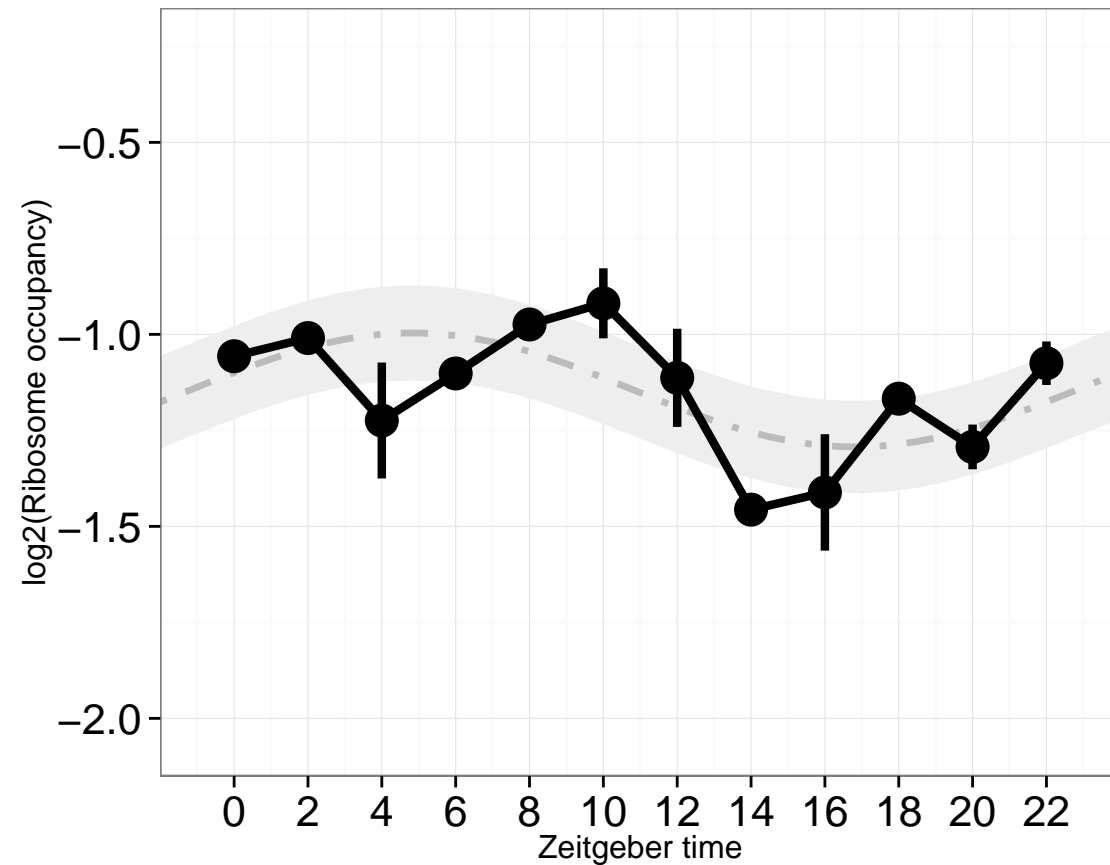

Supplement: Supplementary file 6 — Transcriptome-wide kidney RPF (blue) and RNA (orange) levels in the left panels (with “error bars” connecting the two replicates of each timepoint) and TE in the right panels. (ZIP 116896 kb) [file 13059_2017_1222_MOESM6_ESM.zip › Supp_Dataset_S1/A_RNA_non_rhythmic_RPF_non_rhythmic/2210018M11Rik_kidney_set_A.pdf]

## 2210404O09Rik

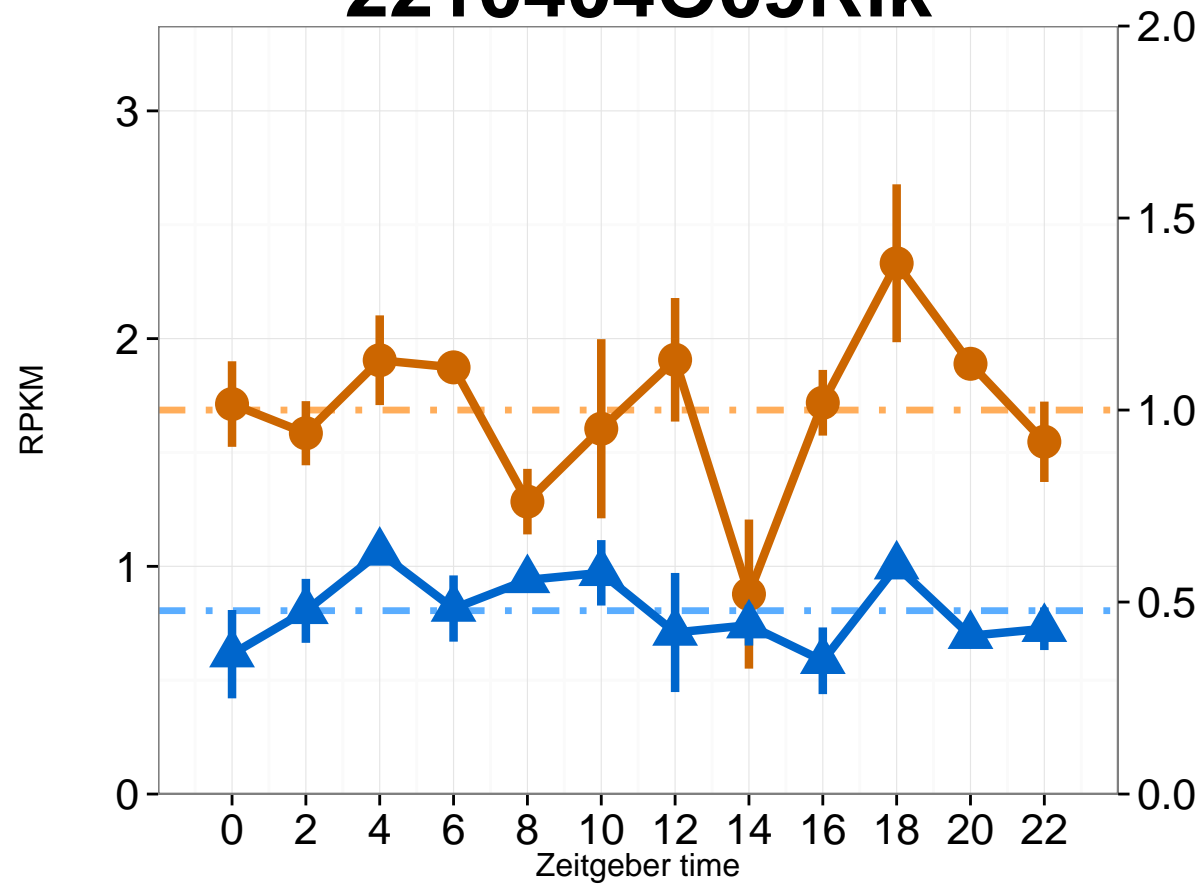

## 2210404O09Rik

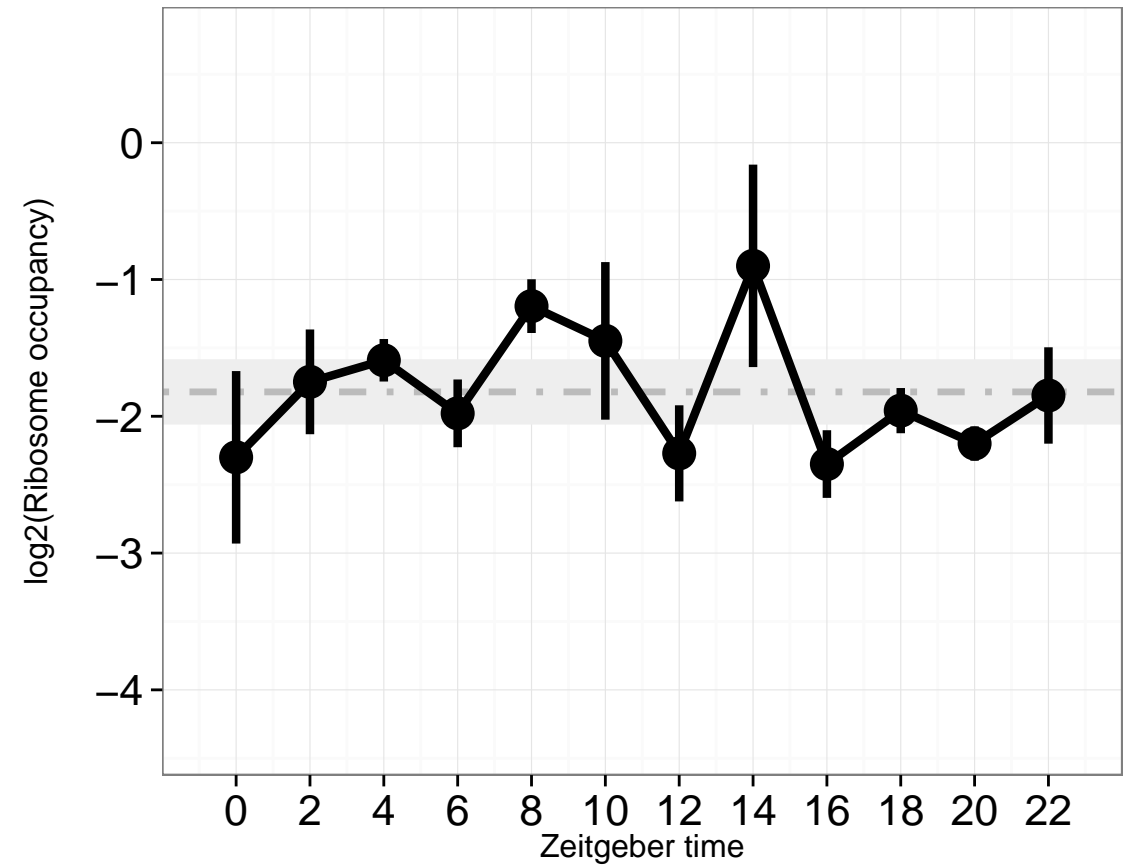

Supplement: Supplementary file 6 — Transcriptome-wide kidney RPF (blue) and RNA (orange) levels in the left panels (with “error bars” connecting the two replicates of each timepoint) and TE in the right panels. (ZIP 116896 kb) [file 13059_2017_1222_MOESM6_ESM.zip › Supp_Dataset_S1/A_RNA_non_rhythmic_RPF_non_rhythmic/2210404O09Rik_kidney_set_A.pdf]

## 2210408I21Rik

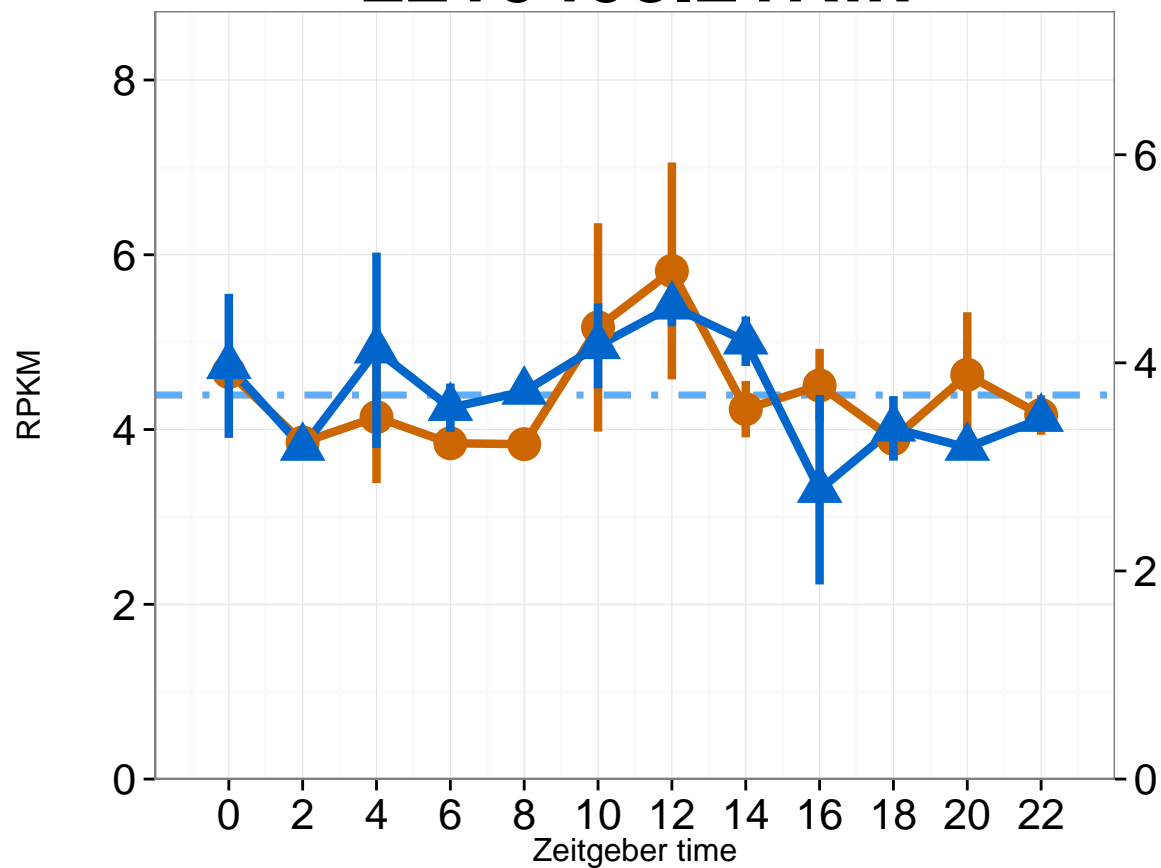

## 2210408I21Rik

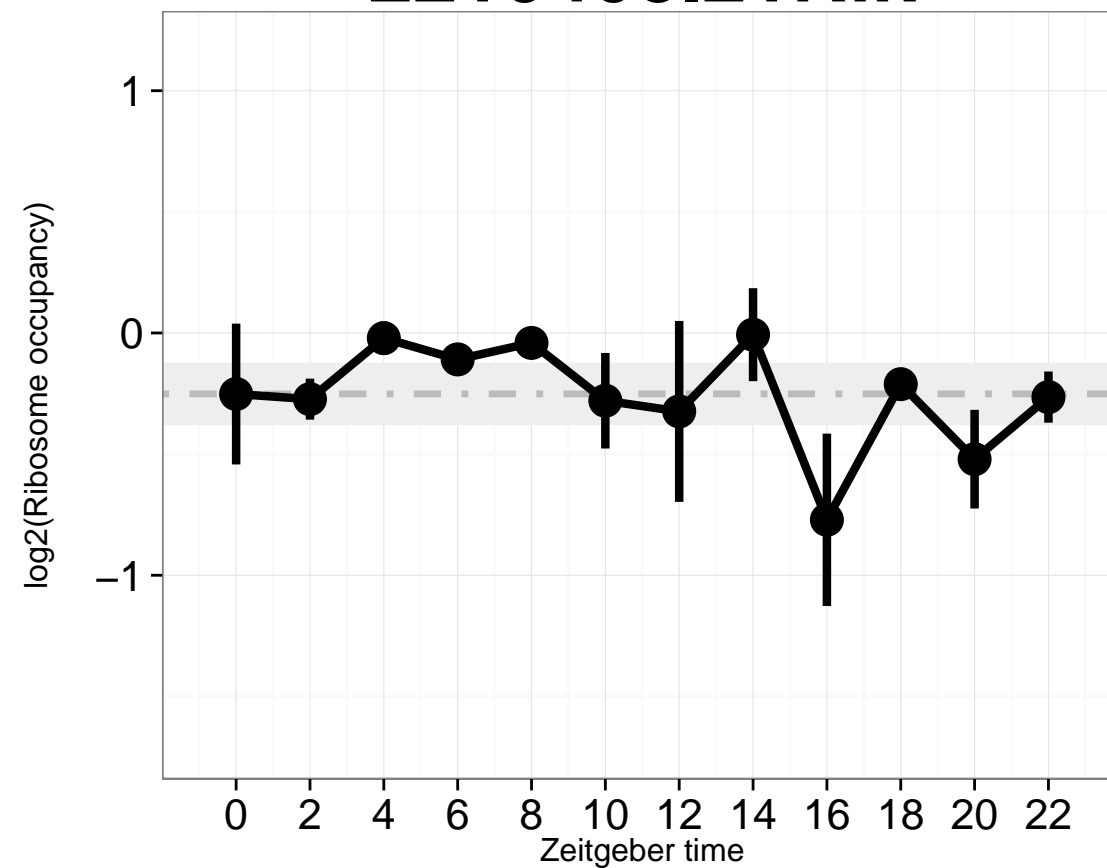

Supplement: Supplementary file 6 — Transcriptome-wide kidney RPF (blue) and RNA (orange) levels in the left panels (with “error bars” connecting the two replicates of each timepoint) and TE in the right panels. (ZIP 116896 kb) [file 13059_2017_1222_MOESM6_ESM.zip › Supp_Dataset_S1/A_RNA_non_rhythmic_RPF_non_rhythmic/2210408I21Rik_kidney_set_A.pdf]

**2310007B03Rik**

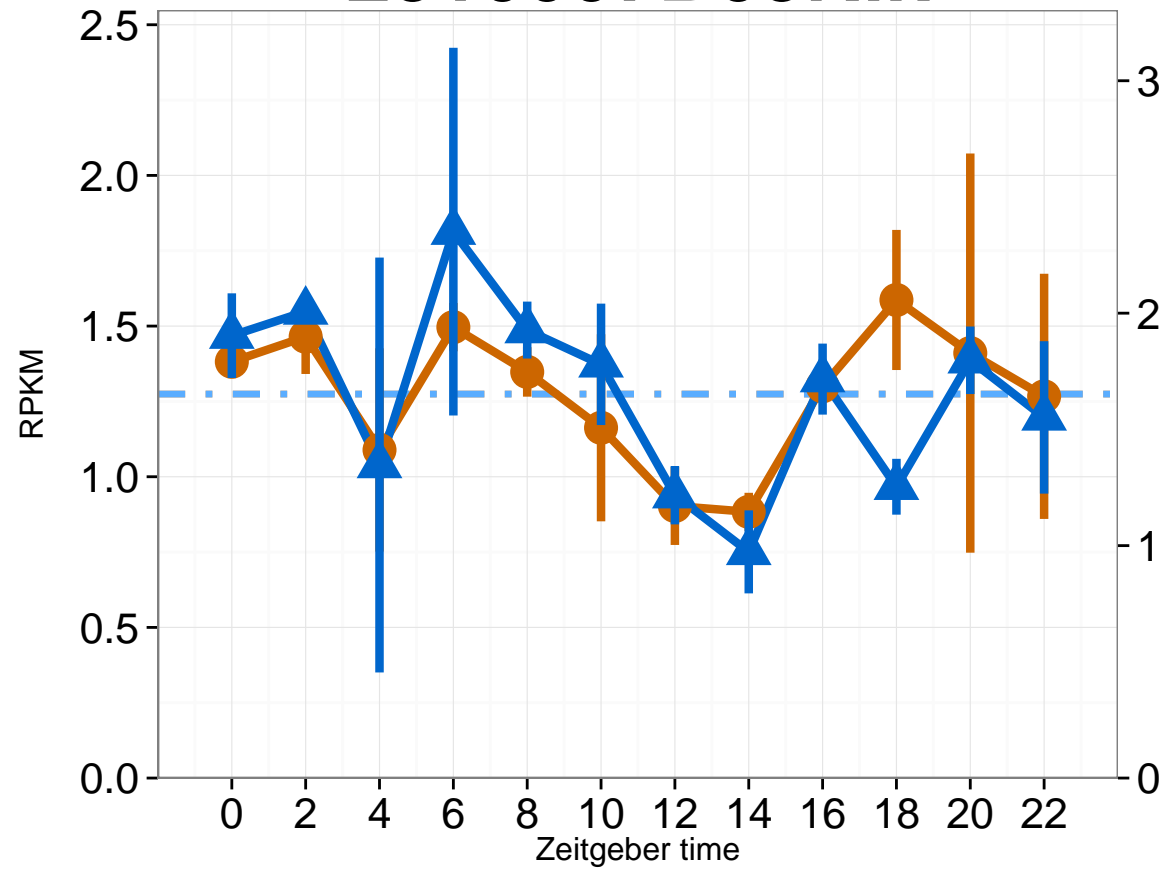

**2310007B03Rik**

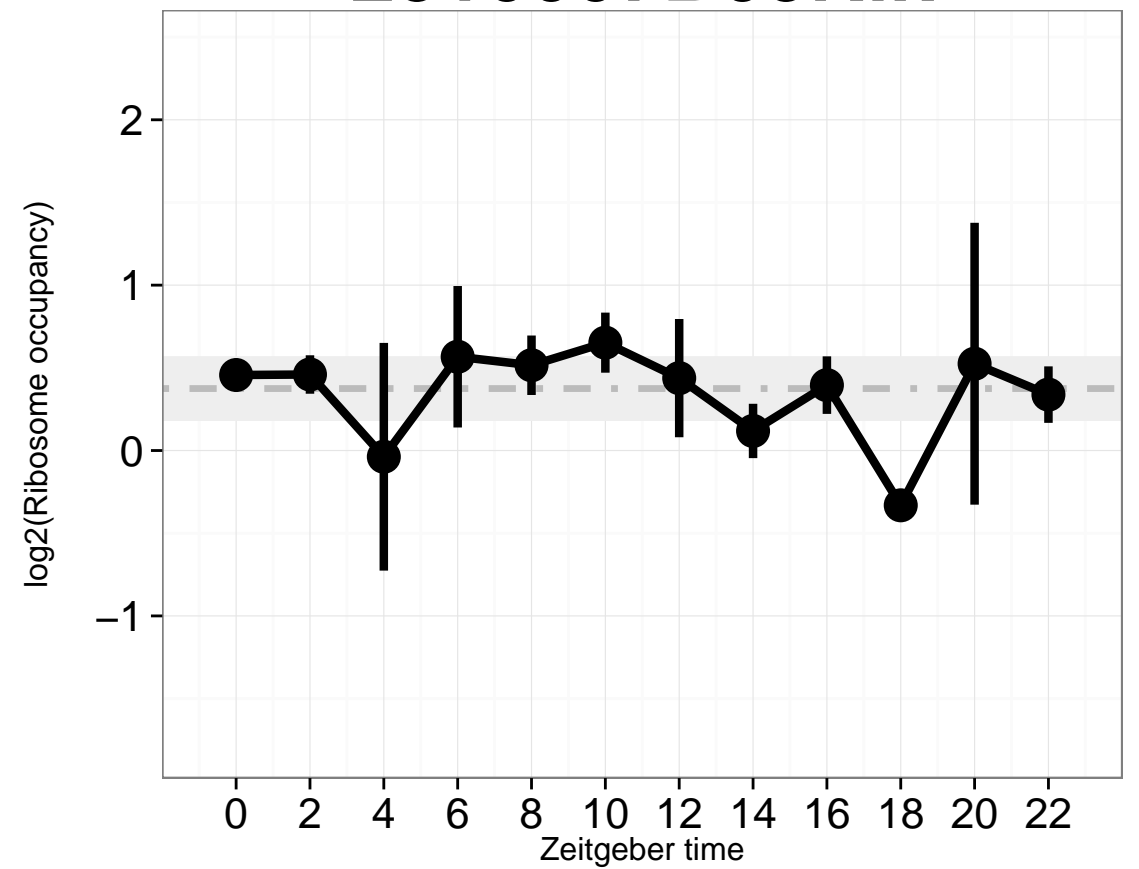

Supplement: Supplementary file 6 — Transcriptome-wide kidney RPF (blue) and RNA (orange) levels in the left panels (with “error bars” connecting the two replicates of each timepoint) and TE in the right panels. (ZIP 116896 kb) [file 13059_2017_1222_MOESM6_ESM.zip › Supp_Dataset_S1/A_RNA_non_rhythmic_RPF_non_rhythmic/2310007B03Rik_kidney_set_A.pdf]

## 2310009B15Rik

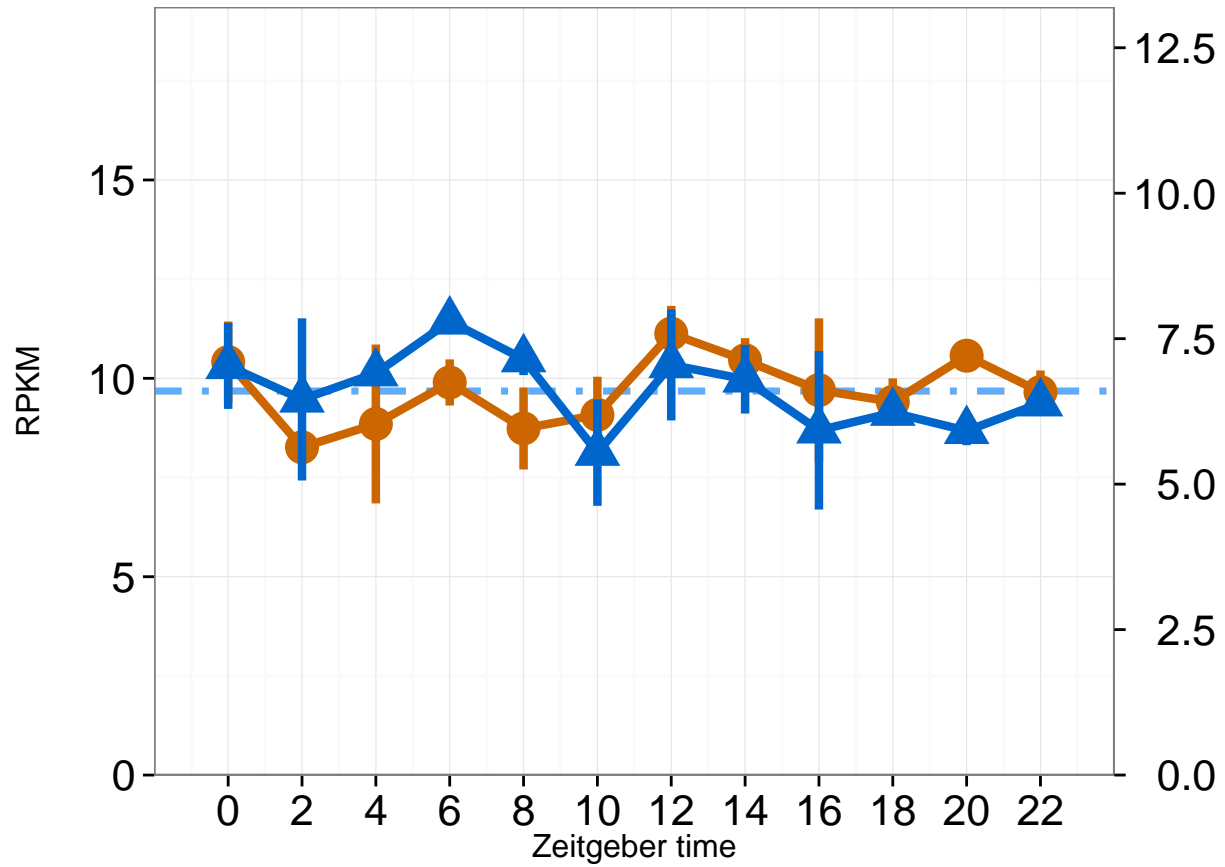

## 2310009B15Rik

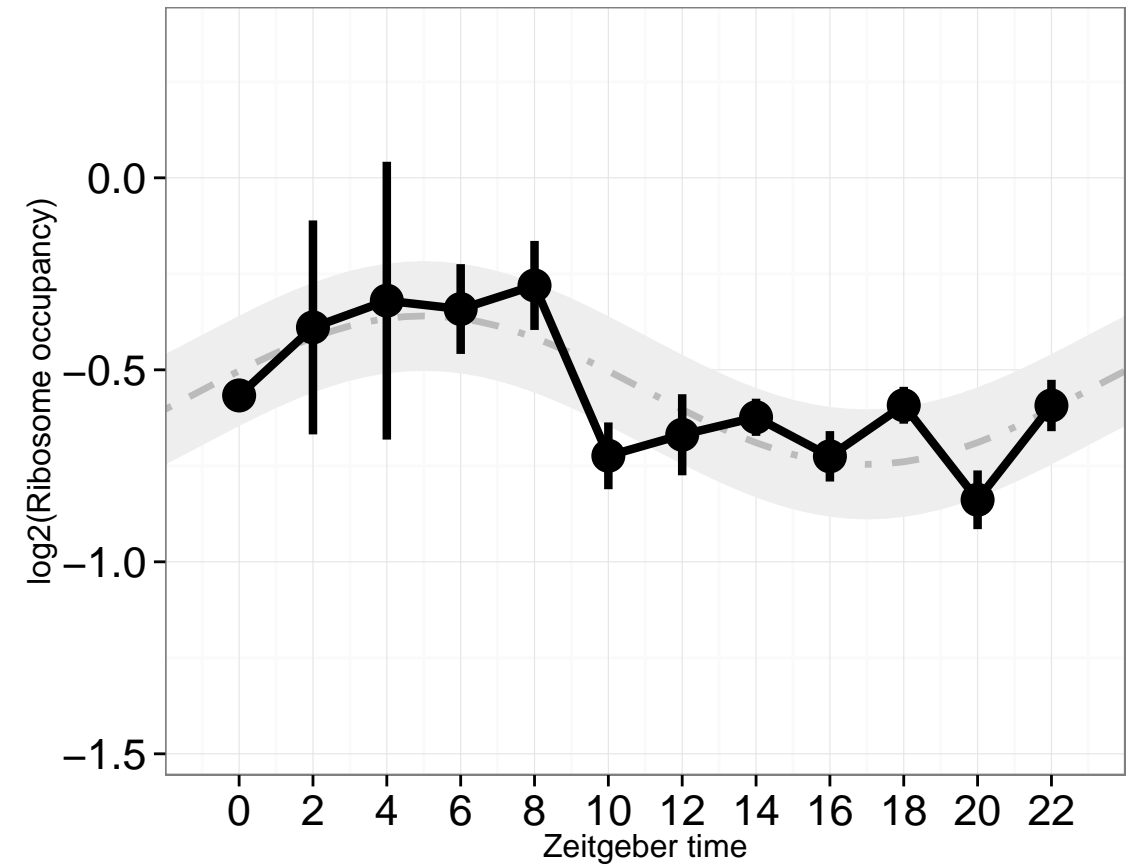

Supplement: Supplementary file 6 — Transcriptome-wide kidney RPF (blue) and RNA (orange) levels in the left panels (with “error bars” connecting the two replicates of each timepoint) and TE in the right panels. (ZIP 116896 kb) [file 13059_2017_1222_MOESM6_ESM.zip › Supp_Dataset_S1/A_RNA_non_rhythmic_RPF_non_rhythmic/2310009B15Rik_kidney_set_A.pdf]

## 2310011J03Rik

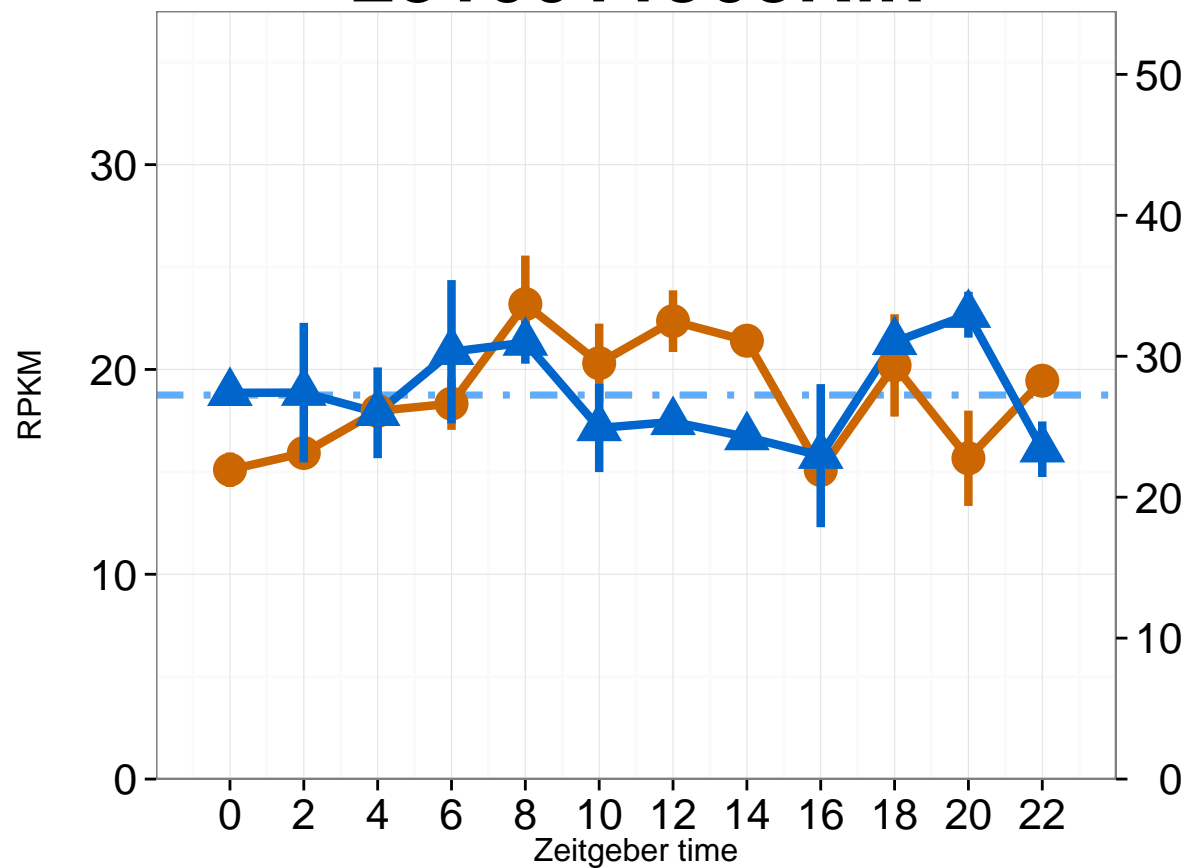

## 2310011J03Rik

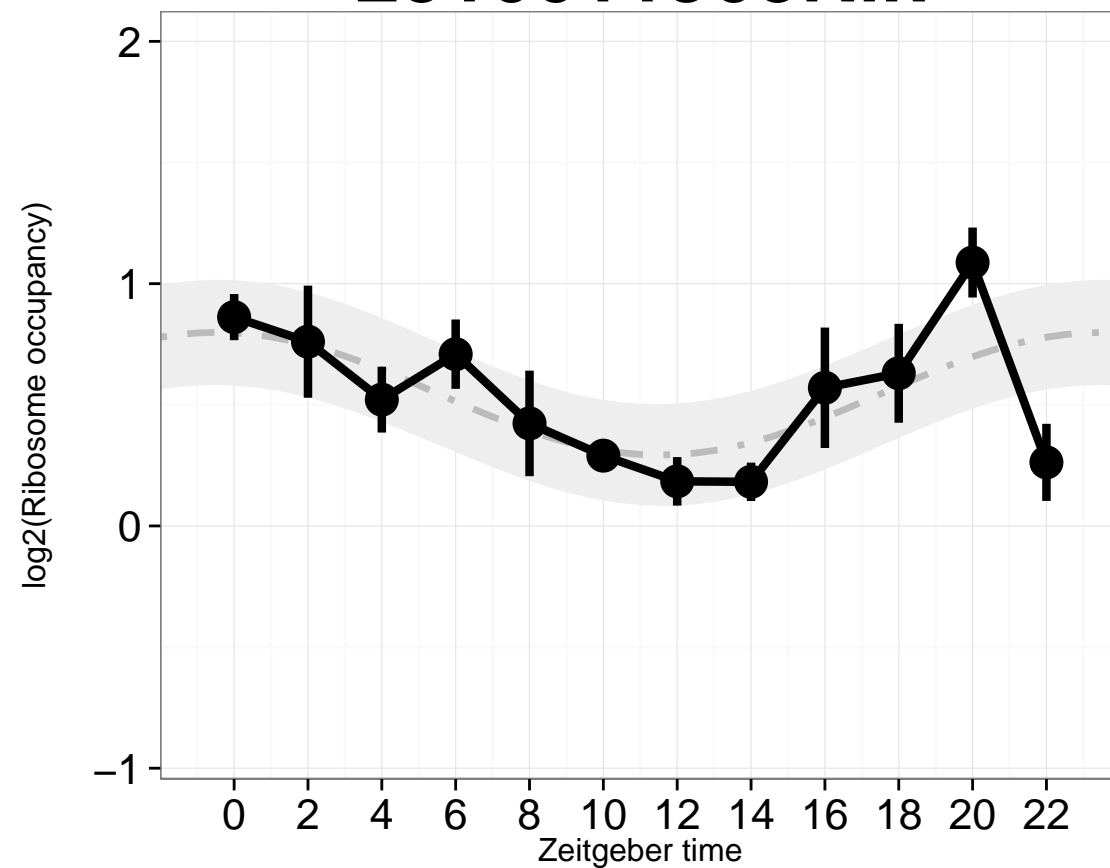

Supplement: Supplementary file 6 — Transcriptome-wide kidney RPF (blue) and RNA (orange) levels in the left panels (with “error bars” connecting the two replicates of each timepoint) and TE in the right panels. (ZIP 116896 kb) [file 13059_2017_1222_MOESM6_ESM.zip › Supp_Dataset_S1/A_RNA_non_rhythmic_RPF_non_rhythmic/2310011J03Rik_kidney_set_A.pdf]

## 2310016G11Rik

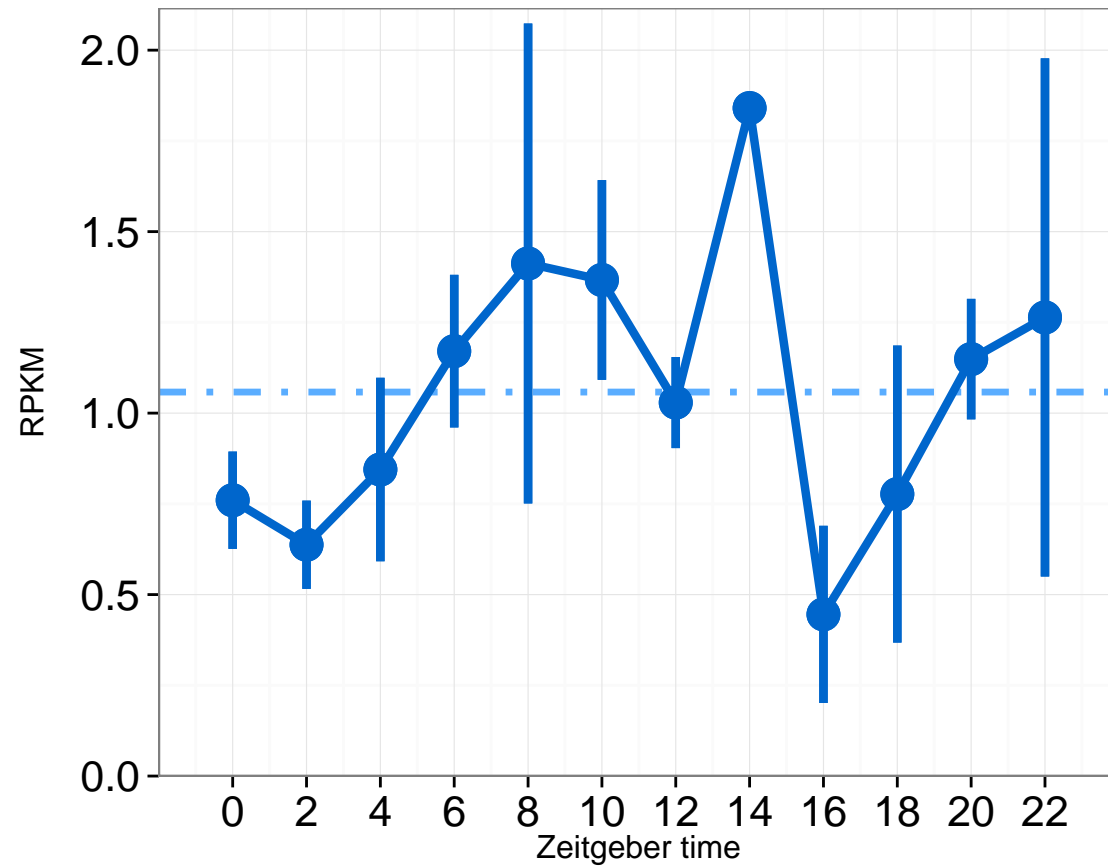

## 2310016G11Rik log2(Ribosome occup

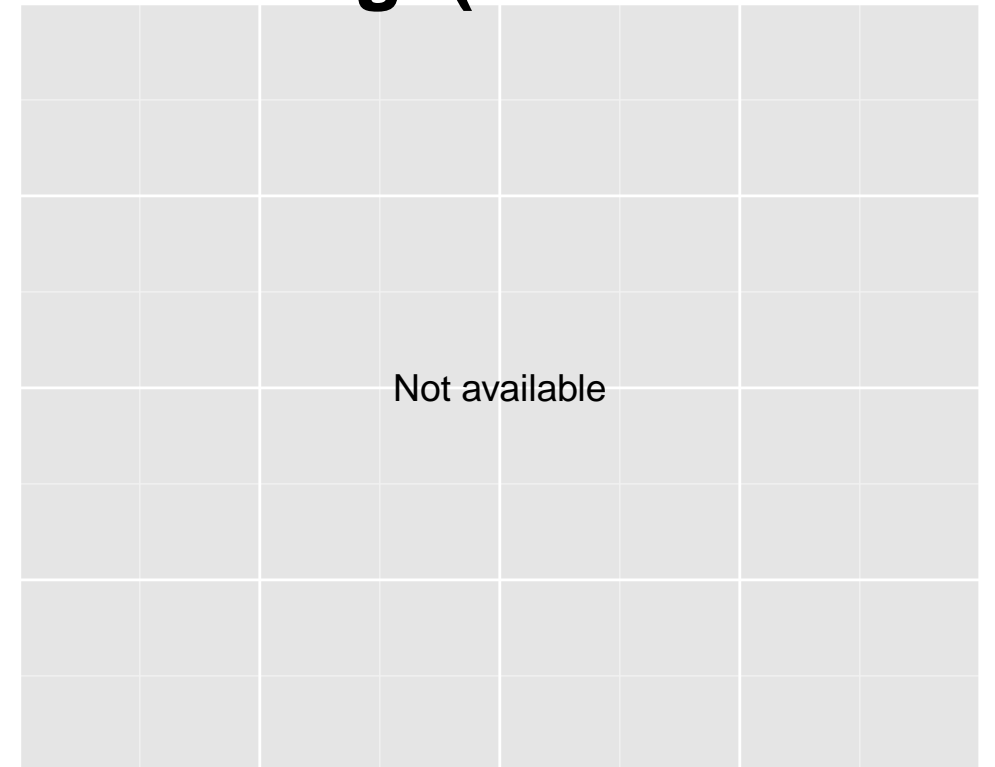

Supplement: Supplementary file 6 — Transcriptome-wide kidney RPF (blue) and RNA (orange) levels in the left panels (with “error bars” connecting the two replicates of each timepoint) and TE in the right panels. (ZIP 116896 kb) [file 13059_2017_1222_MOESM6_ESM.zip › Supp_Dataset_S1/A_RNA_non_rhythmic_RPF_non_rhythmic/2310016G11Rik_kidney_set_A.pdf]

## 2310022B05Rik

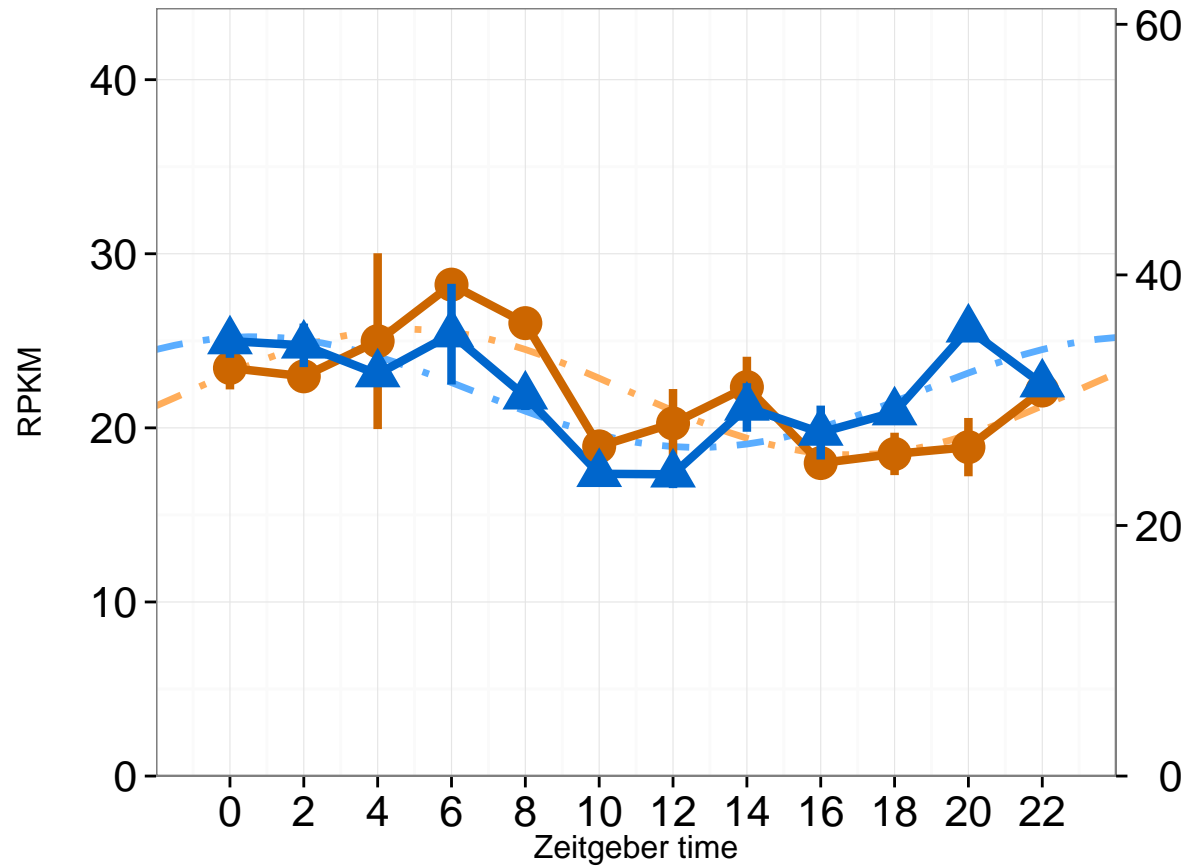

## 2310022B05Rik

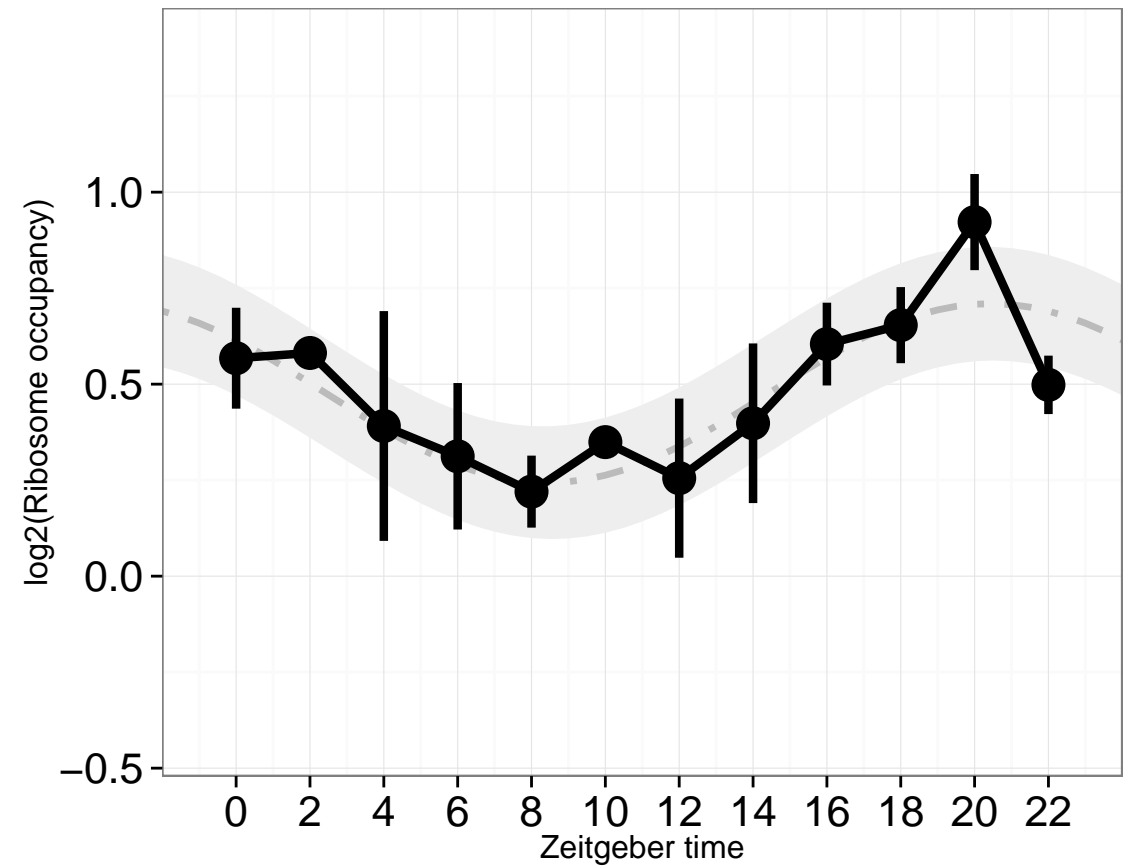

Supplement: Supplementary file 6 — Transcriptome-wide kidney RPF (blue) and RNA (orange) levels in the left panels (with “error bars” connecting the two replicates of each timepoint) and TE in the right panels. (ZIP 116896 kb) [file 13059_2017_1222_MOESM6_ESM.zip › Supp_Dataset_S1/A_RNA_non_rhythmic_RPF_non_rhythmic/2310022B05Rik_kidney_set_A.pdf]

# 2310030G06Rik

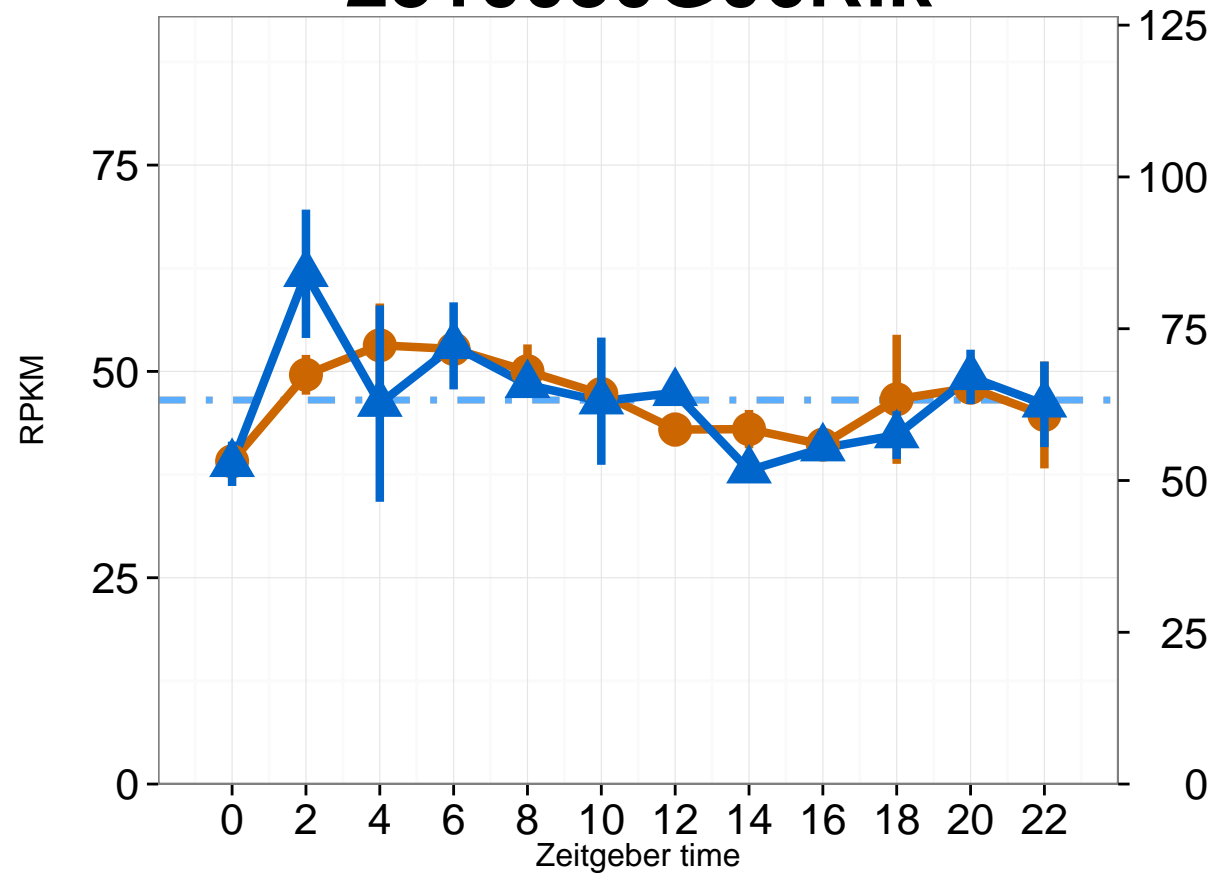

# 2310030G06Rik

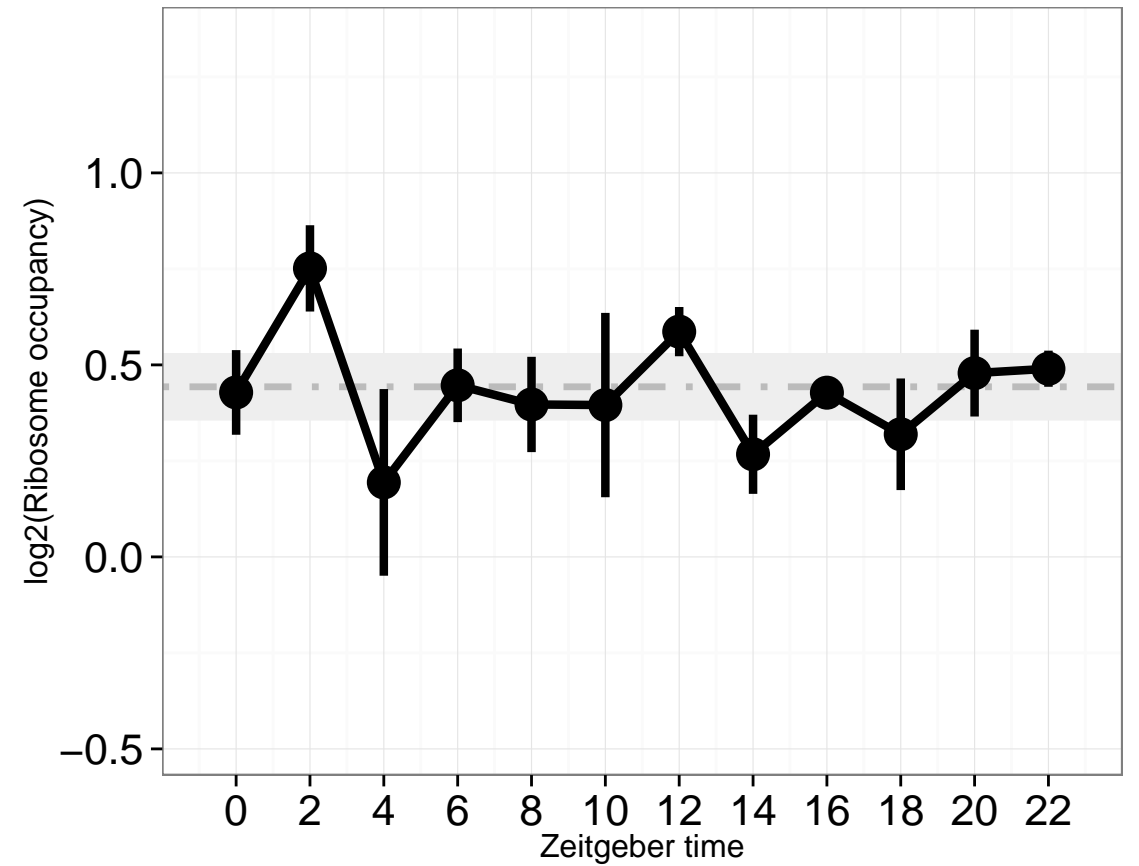

Supplement: Supplementary file 6 — Transcriptome-wide kidney RPF (blue) and RNA (orange) levels in the left panels (with “error bars” connecting the two replicates of each timepoint) and TE in the right panels. (ZIP 116896 kb) [file 13059_2017_1222_MOESM6_ESM.zip › Supp_Dataset_S1/A_RNA_non_rhythmic_RPF_non_rhythmic/2310030G06Rik_kidney_set_A.pdf]

## 2310035C23Rik

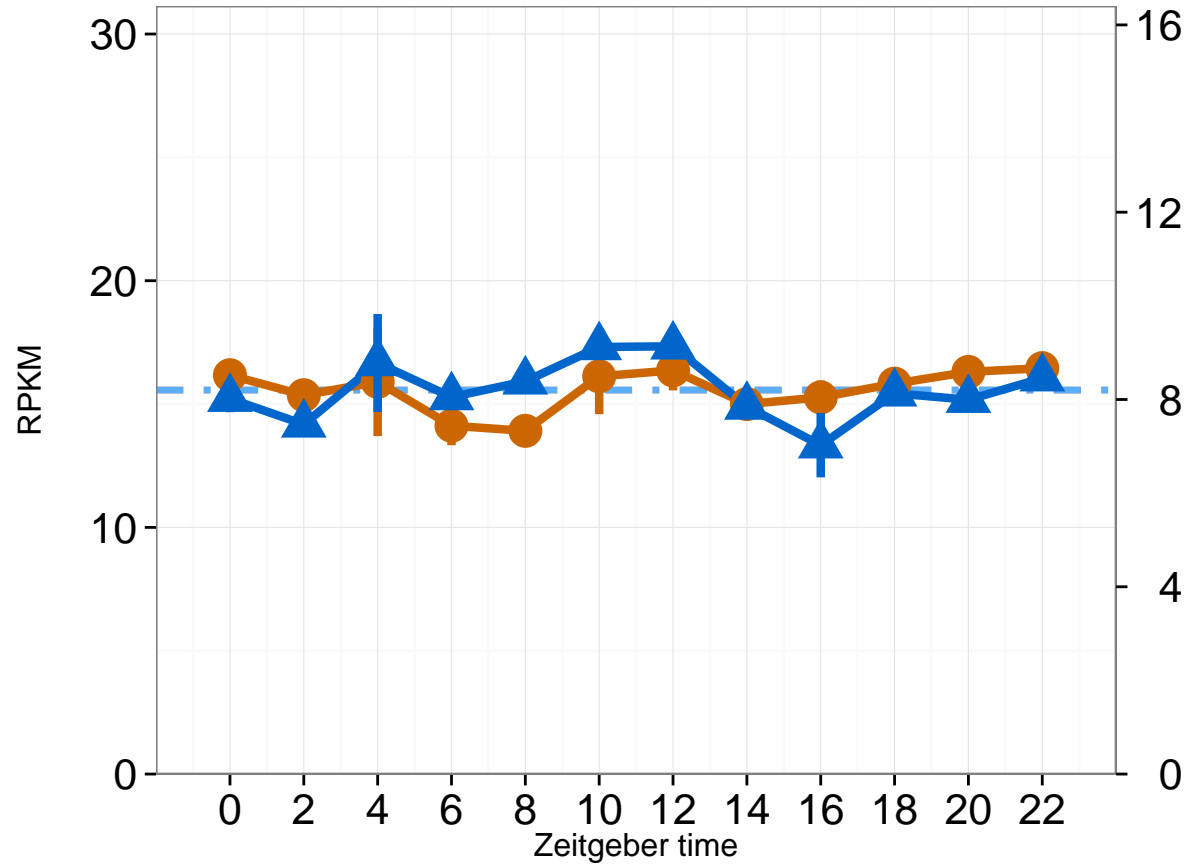

## 2310035C23Rik

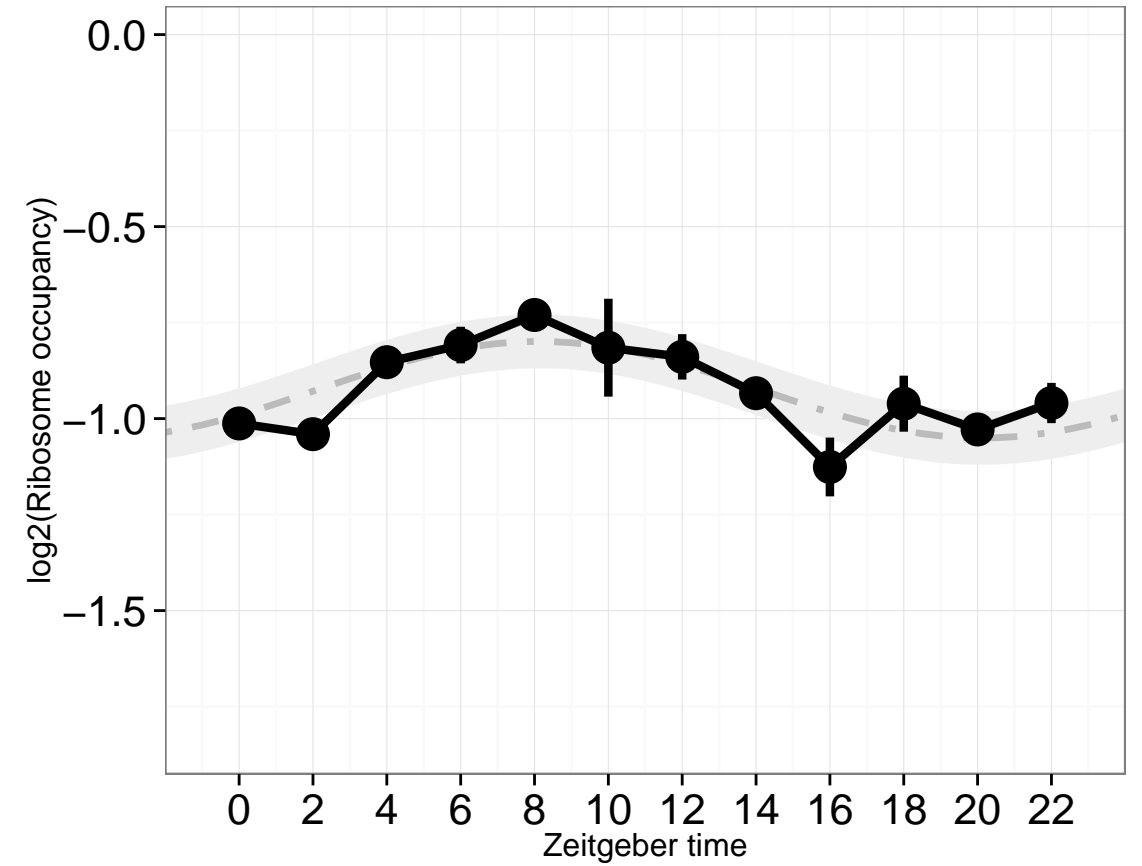

Supplement: Supplementary file 6 — Transcriptome-wide kidney RPF (blue) and RNA (orange) levels in the left panels (with “error bars” connecting the two replicates of each timepoint) and TE in the right panels. (ZIP 116896 kb) [file 13059_2017_1222_MOESM6_ESM.zip › Supp_Dataset_S1/A_RNA_non_rhythmic_RPF_non_rhythmic/2310035C23Rik_kidney_set_A.pdf]

# 2310036O22Rik

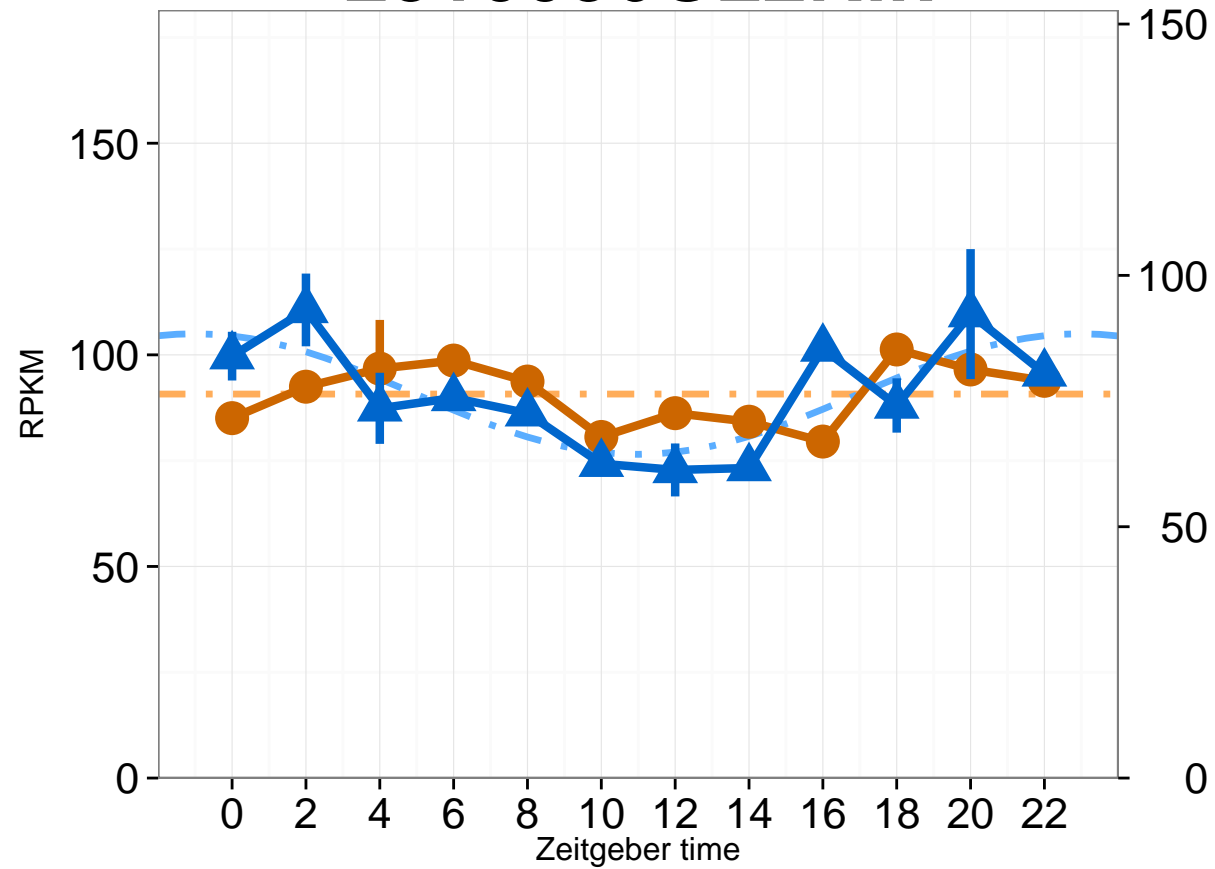

# 2310036O22Rik

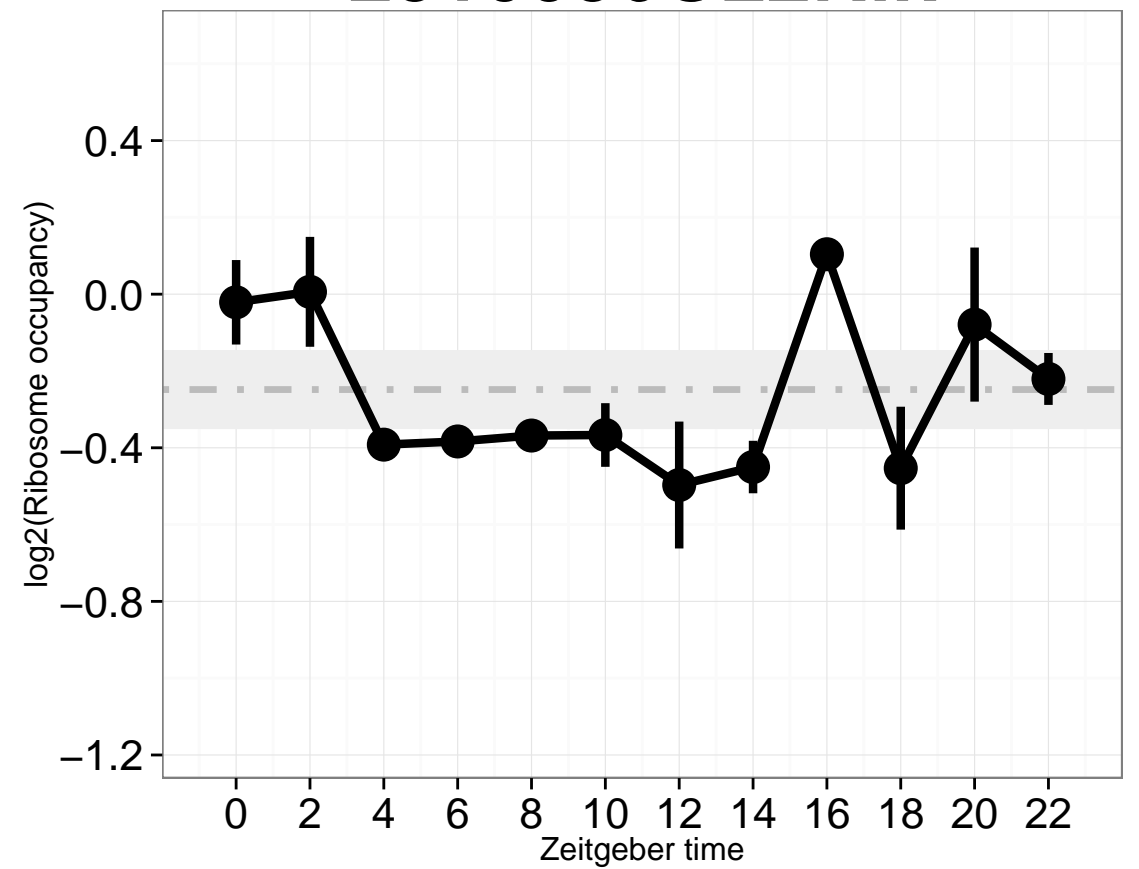

Supplement: Supplementary file 6 — Transcriptome-wide kidney RPF (blue) and RNA (orange) levels in the left panels (with “error bars” connecting the two replicates of each timepoint) and TE in the right panels. (ZIP 116896 kb) [file 13059_2017_1222_MOESM6_ESM.zip › Supp_Dataset_S1/A_RNA_non_rhythmic_RPF_non_rhythmic/2310036O22Rik_kidney_set_A.pdf]

## 2310039H08Rik

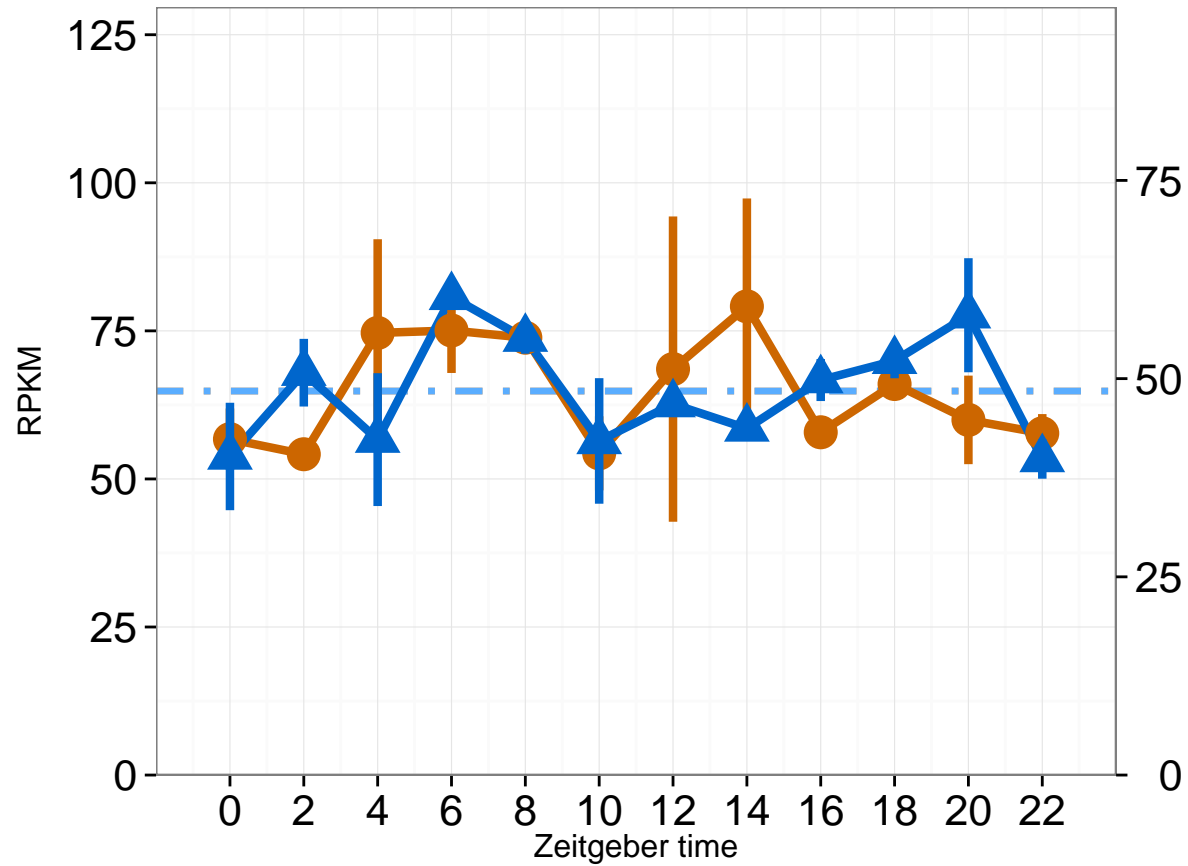

## 2310039H08Rik

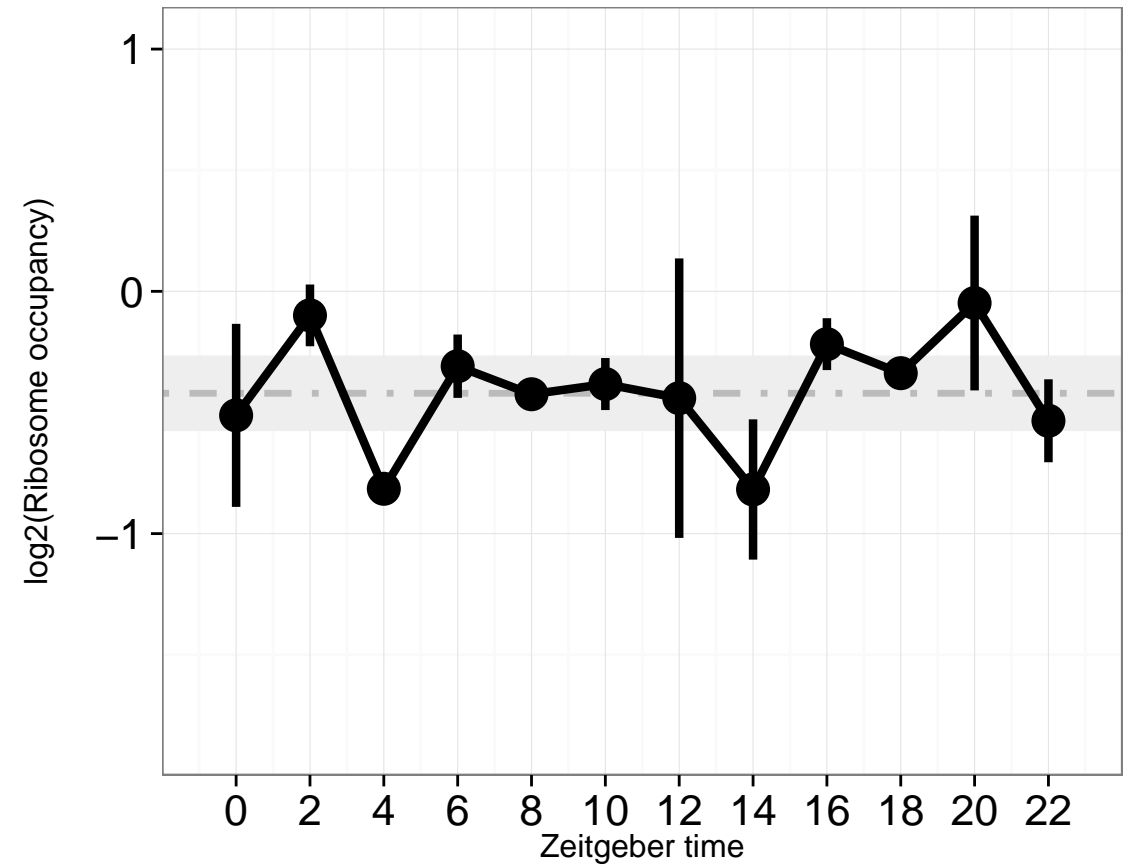

Supplement: Supplementary file 6 — Transcriptome-wide kidney RPF (blue) and RNA (orange) levels in the left panels (with “error bars” connecting the two replicates of each timepoint) and TE in the right panels. (ZIP 116896 kb) [file 13059_2017_1222_MOESM6_ESM.zip › Supp_Dataset_S1/A_RNA_non_rhythmic_RPF_non_rhythmic/2310039H08Rik_kidney_set_A.pdf]

# 2310042D19Rik

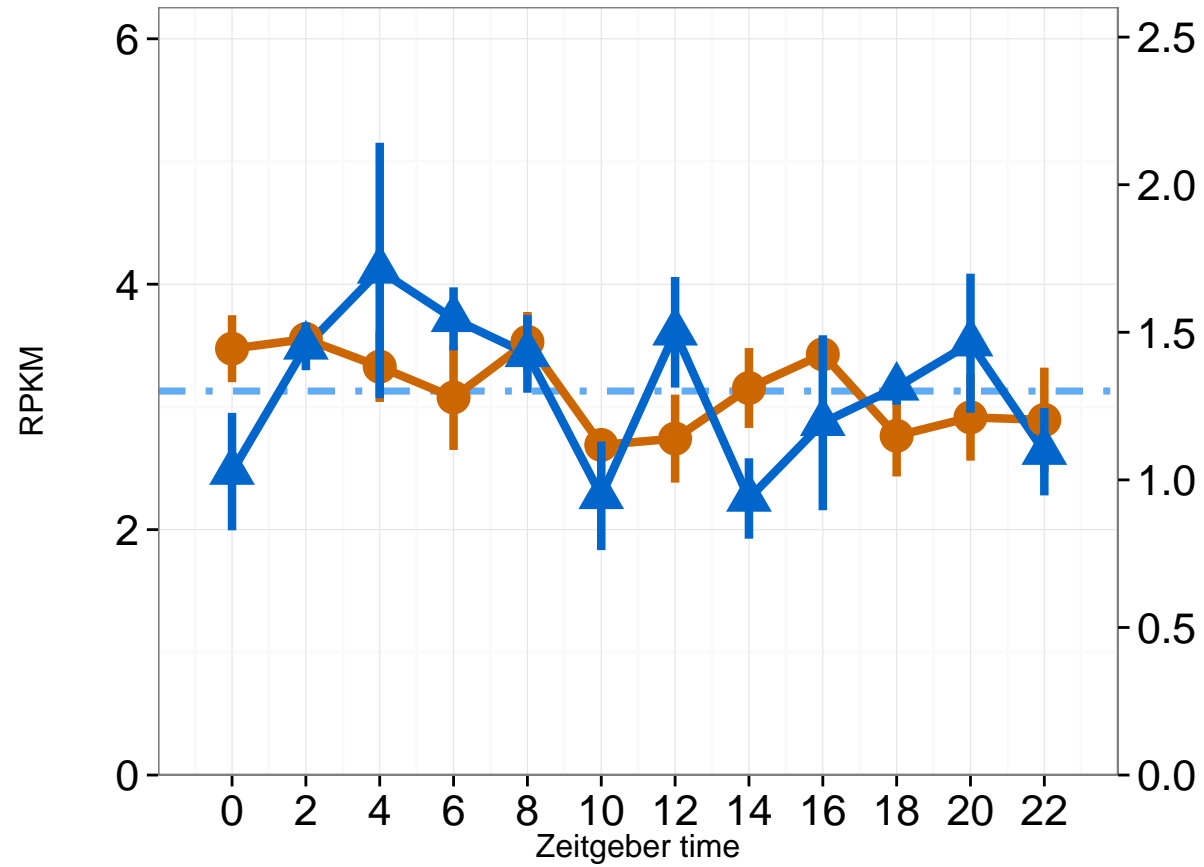

# 2310042D19Rik

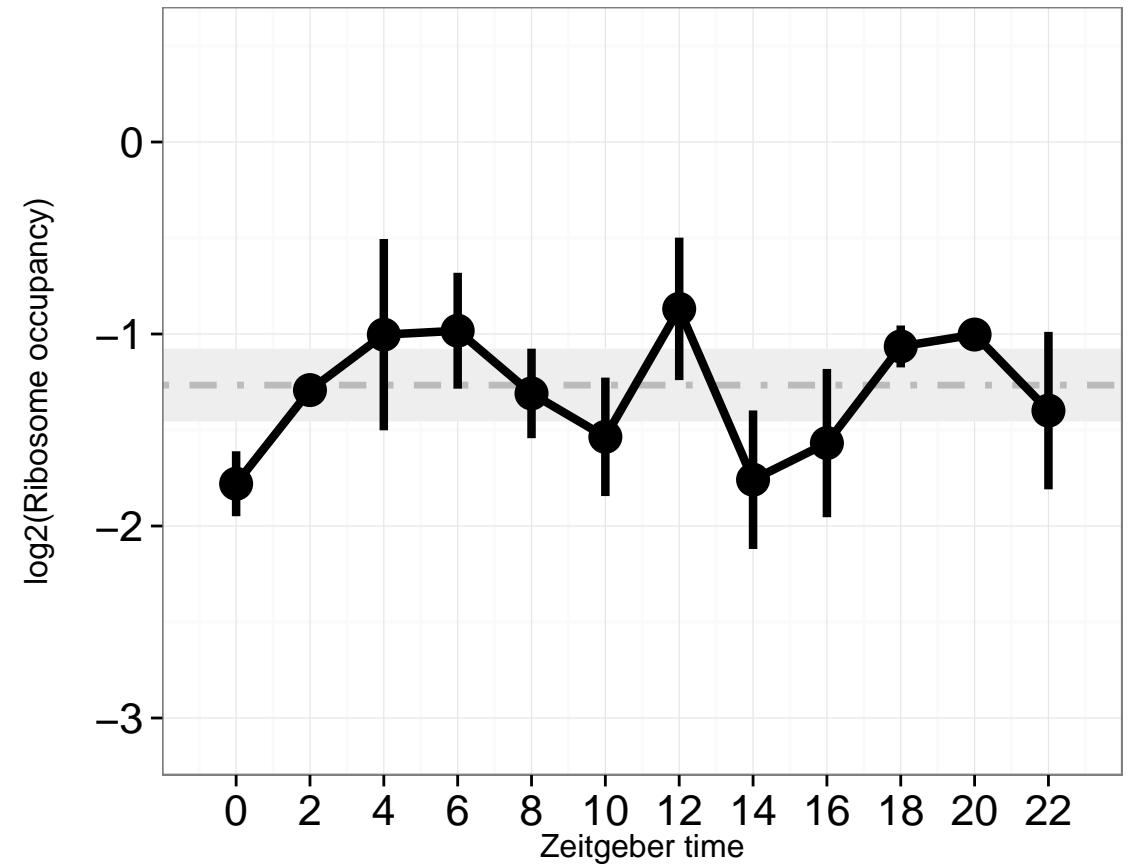

Supplement: Supplementary file 6 — Transcriptome-wide kidney RPF (blue) and RNA (orange) levels in the left panels (with “error bars” connecting the two replicates of each timepoint) and TE in the right panels. (ZIP 116896 kb) [file 13059_2017_1222_MOESM6_ESM.zip › Supp_Dataset_S1/A_RNA_non_rhythmic_RPF_non_rhythmic/2310042D19Rik_kidney_set_A.pdf]

## 2310045N01Rik

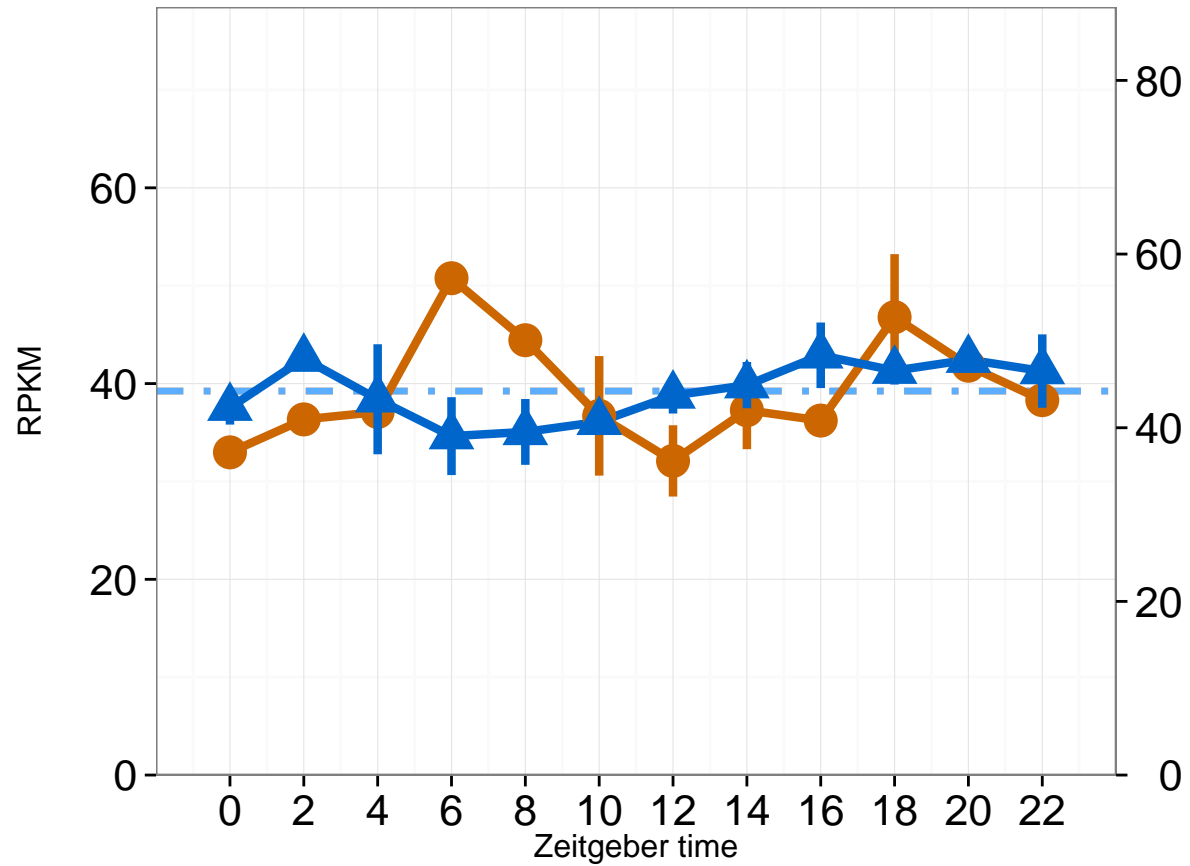

## 2310045N01Rik

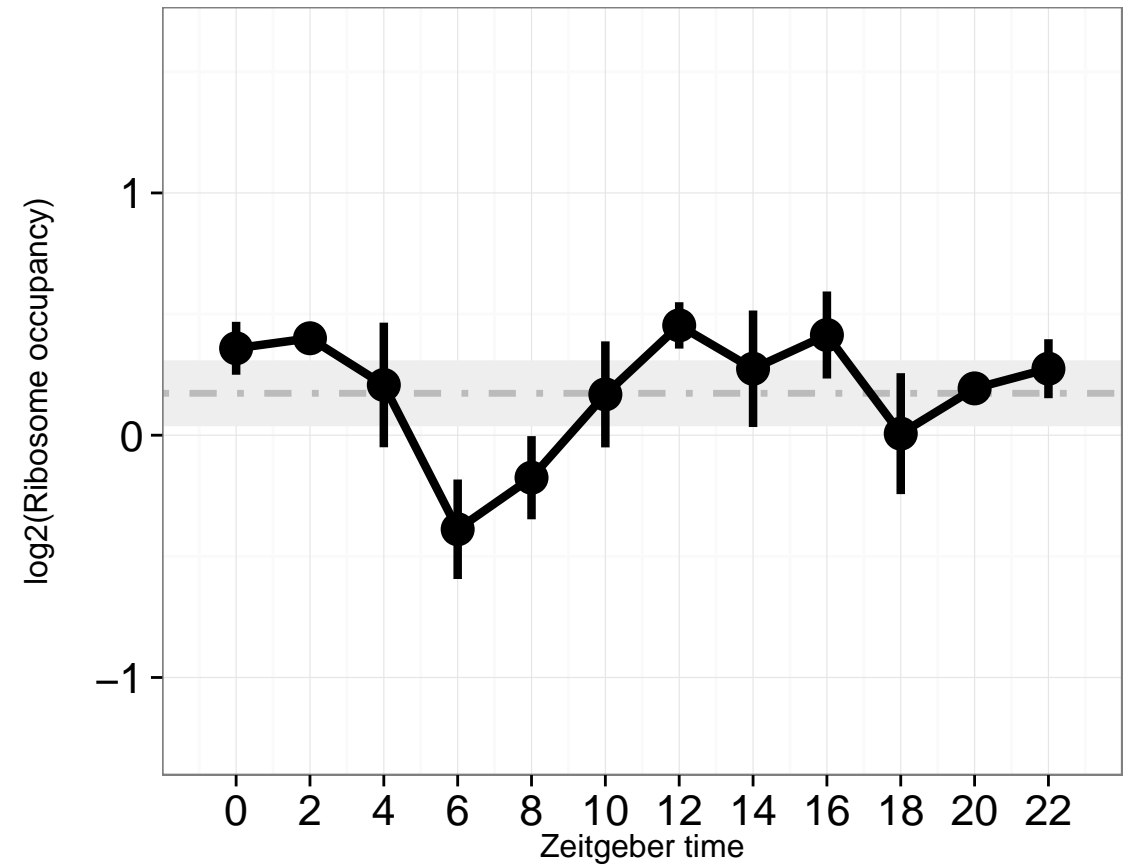

Supplement: Supplementary file 6 — Transcriptome-wide kidney RPF (blue) and RNA (orange) levels in the left panels (with “error bars” connecting the two replicates of each timepoint) and TE in the right panels. (ZIP 116896 kb) [file 13059_2017_1222_MOESM6_ESM.zip › Supp_Dataset_S1/A_RNA_non_rhythmic_RPF_non_rhythmic/2310045N01Rik_kidney_set_A.pdf]

## 2310047M10Rik

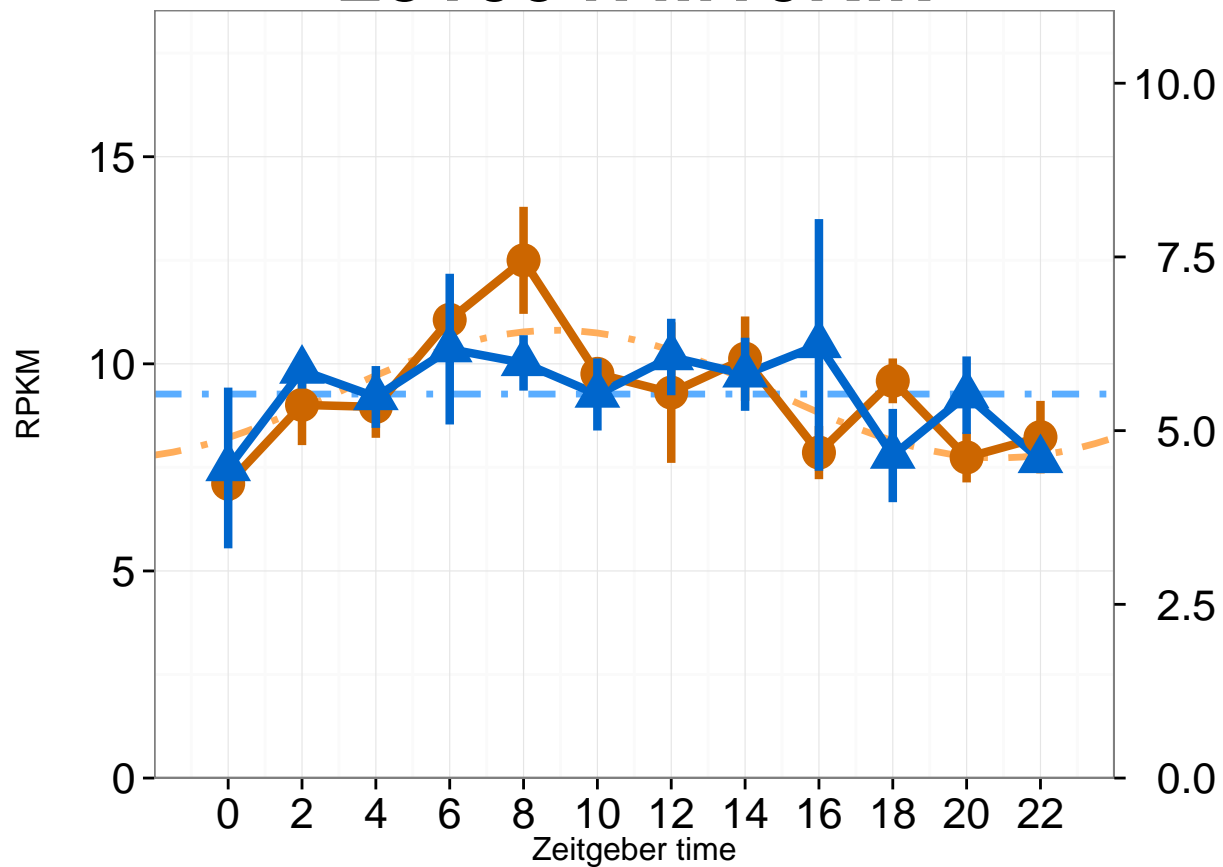

## 2310047M10Rik

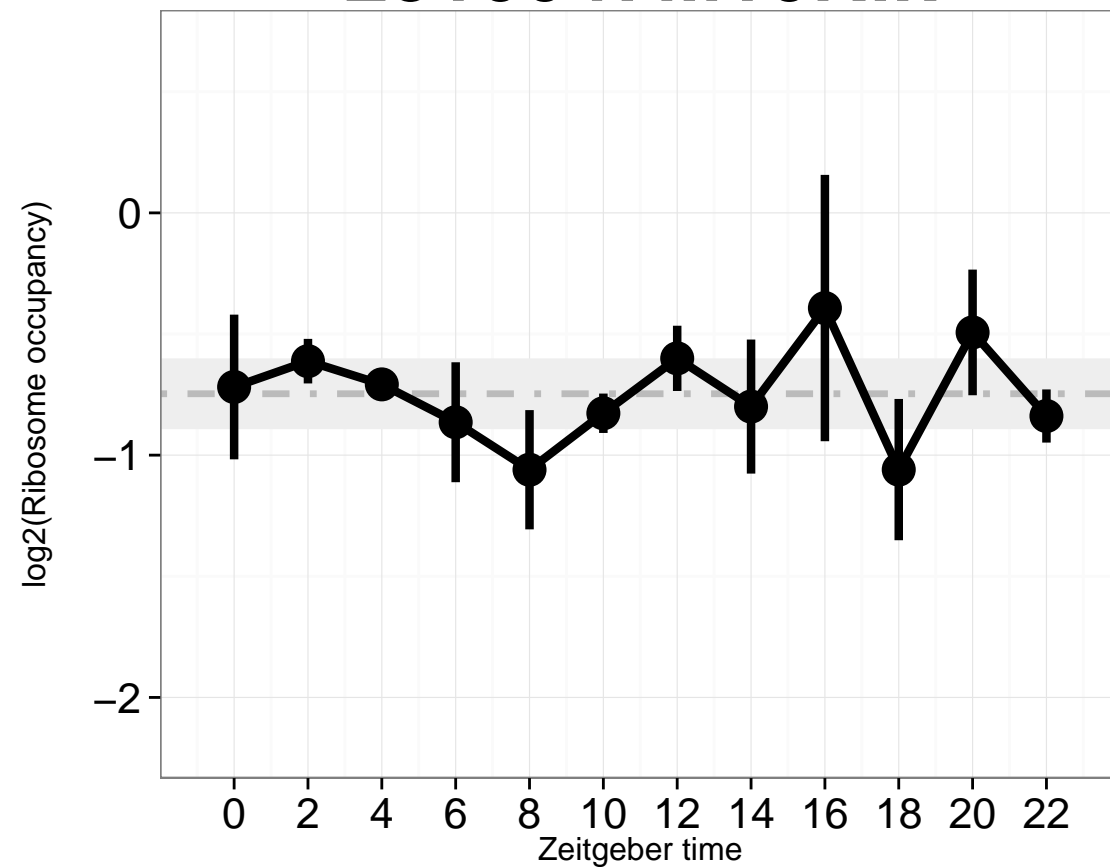

Supplement: Supplementary file 6 — Transcriptome-wide kidney RPF (blue) and RNA (orange) levels in the left panels (with “error bars” connecting the two replicates of each timepoint) and TE in the right panels. (ZIP 116896 kb) [file 13059_2017_1222_MOESM6_ESM.zip › Supp_Dataset_S1/A_RNA_non_rhythmic_RPF_non_rhythmic/2310047M10Rik_kidney_set_A.pdf]

# 2310057M21Rik

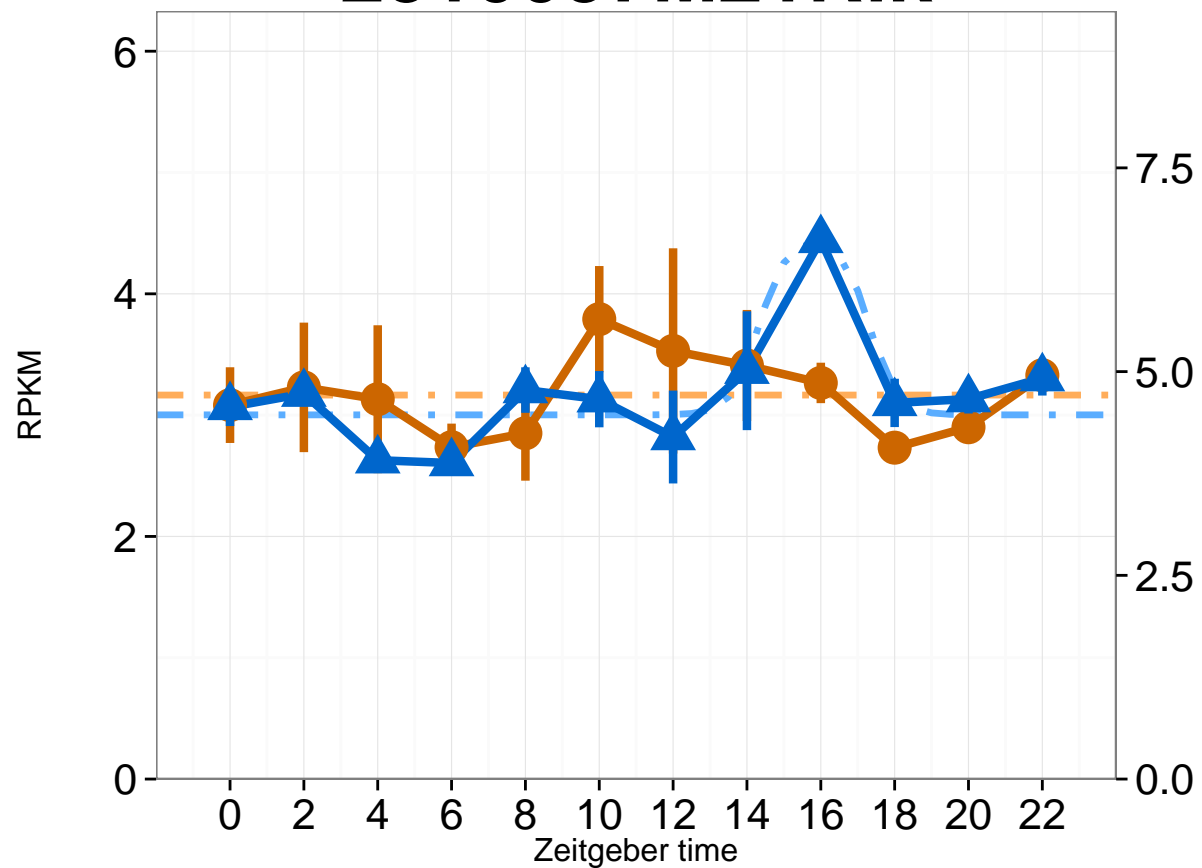

# 2310057M21Rik

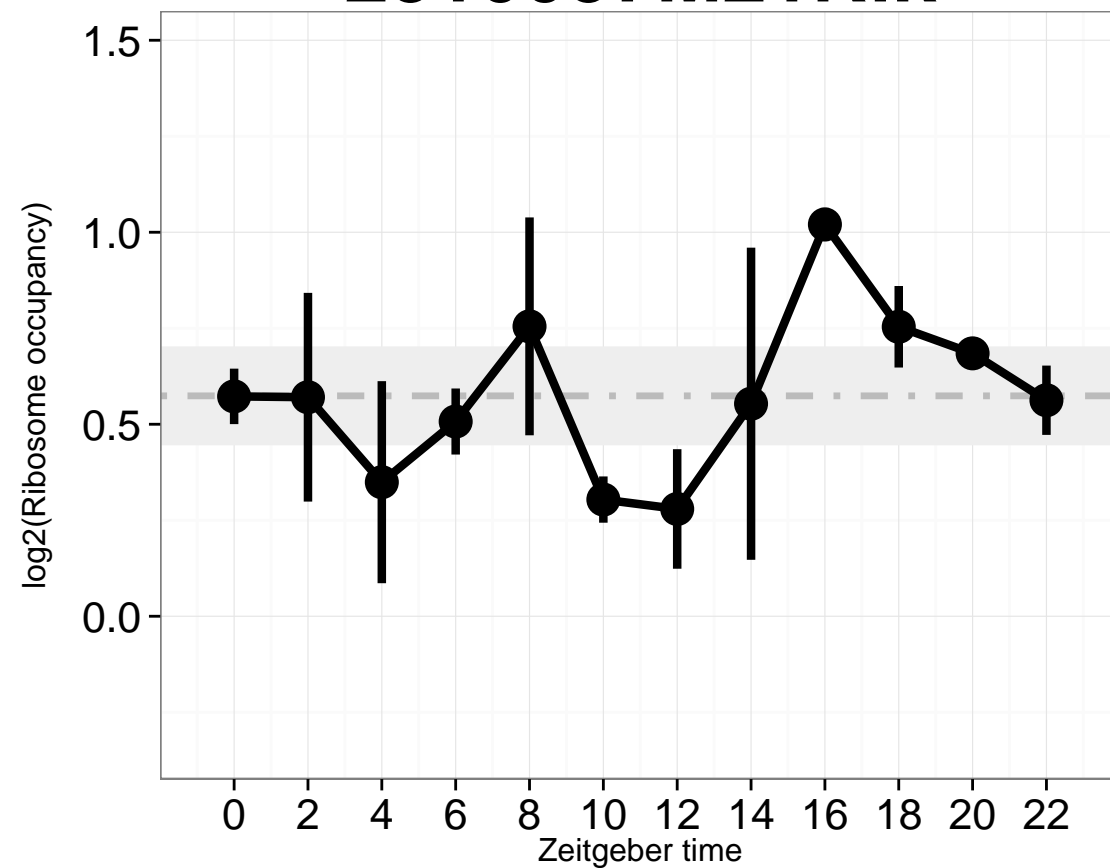

Supplement: Supplementary file 6 — Transcriptome-wide kidney RPF (blue) and RNA (orange) levels in the left panels (with “error bars” connecting the two replicates of each timepoint) and TE in the right panels. (ZIP 116896 kb) [file 13059_2017_1222_MOESM6_ESM.zip › Supp_Dataset_S1/A_RNA_non_rhythmic_RPF_non_rhythmic/2310057M21Rik_kidney_set_A.pdf]

## 2310061I04Rik

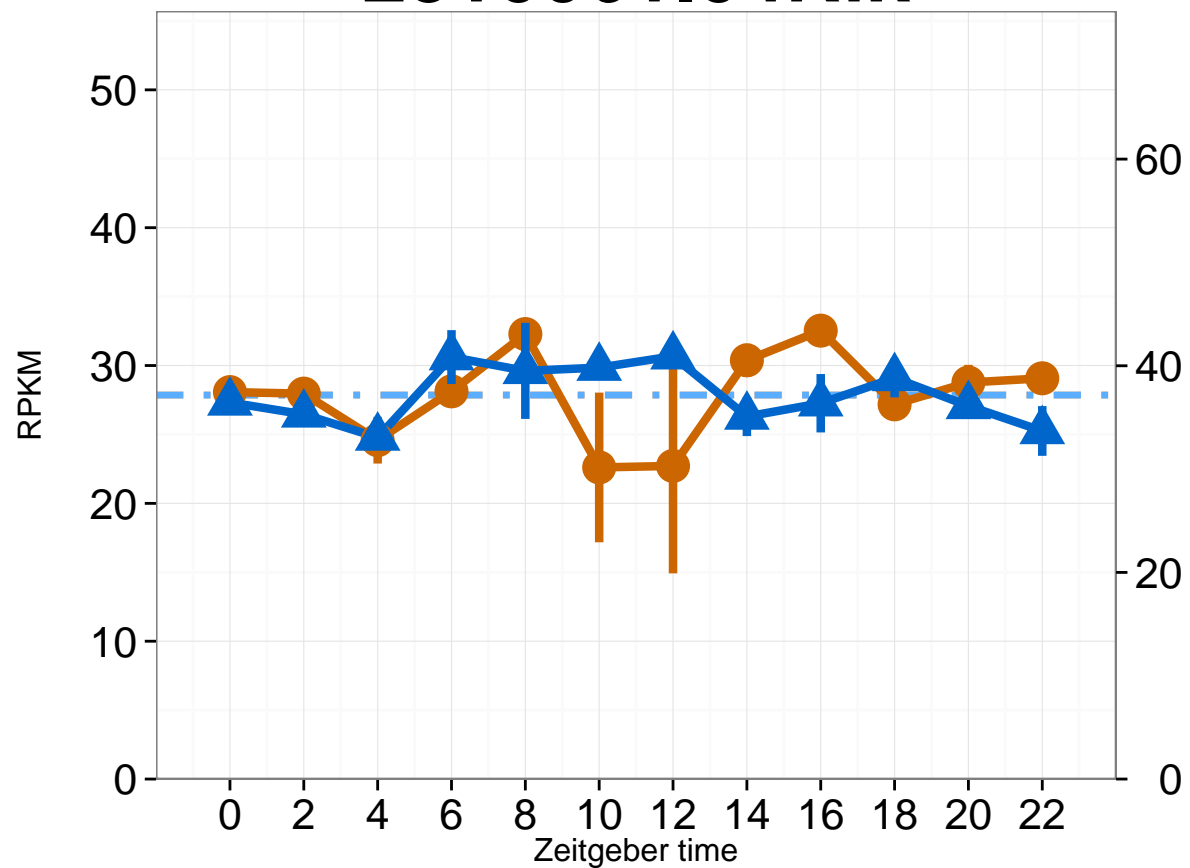

## 2310061I04Rik

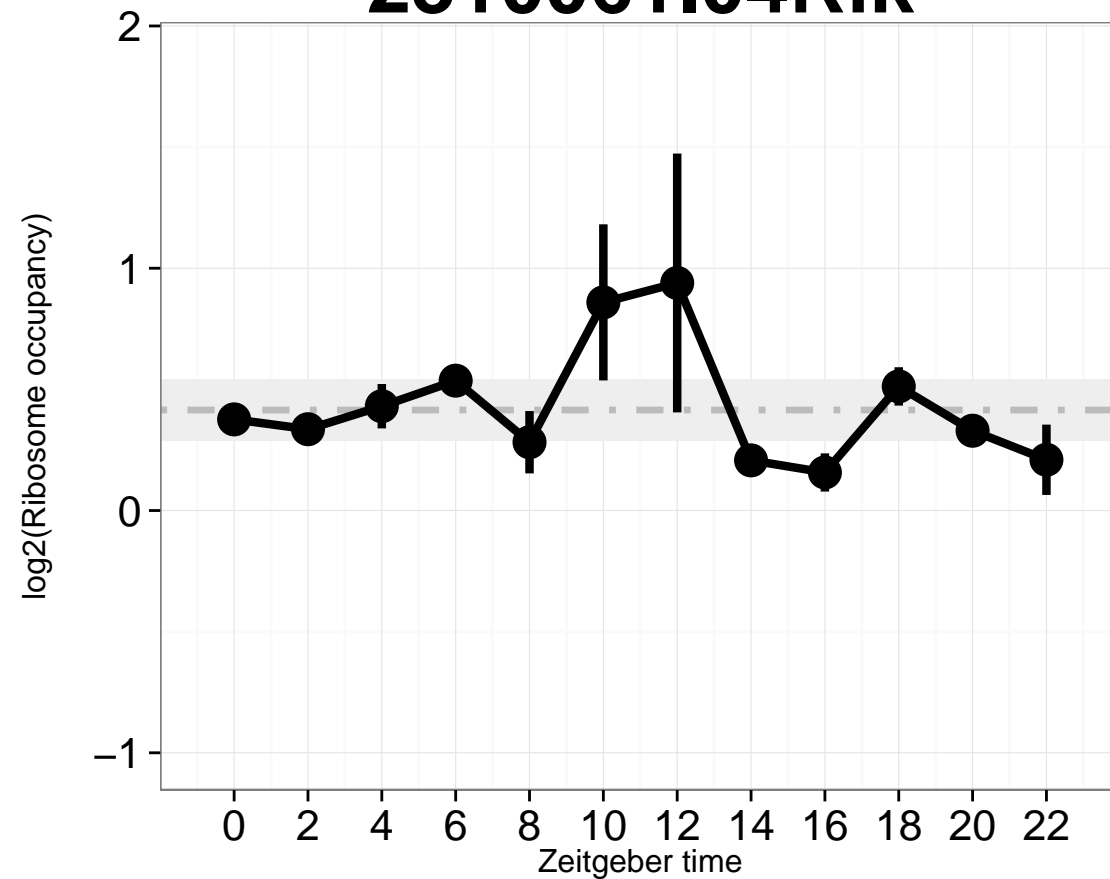

Supplement: Supplementary file 6 — Transcriptome-wide kidney RPF (blue) and RNA (orange) levels in the left panels (with “error bars” connecting the two replicates of each timepoint) and TE in the right panels. (ZIP 116896 kb) [file 13059_2017_1222_MOESM6_ESM.zip › Supp_Dataset_S1/A_RNA_non_rhythmic_RPF_non_rhythmic/2310061I04Rik_kidney_set_A.pdf]

## 2310067B10Rik

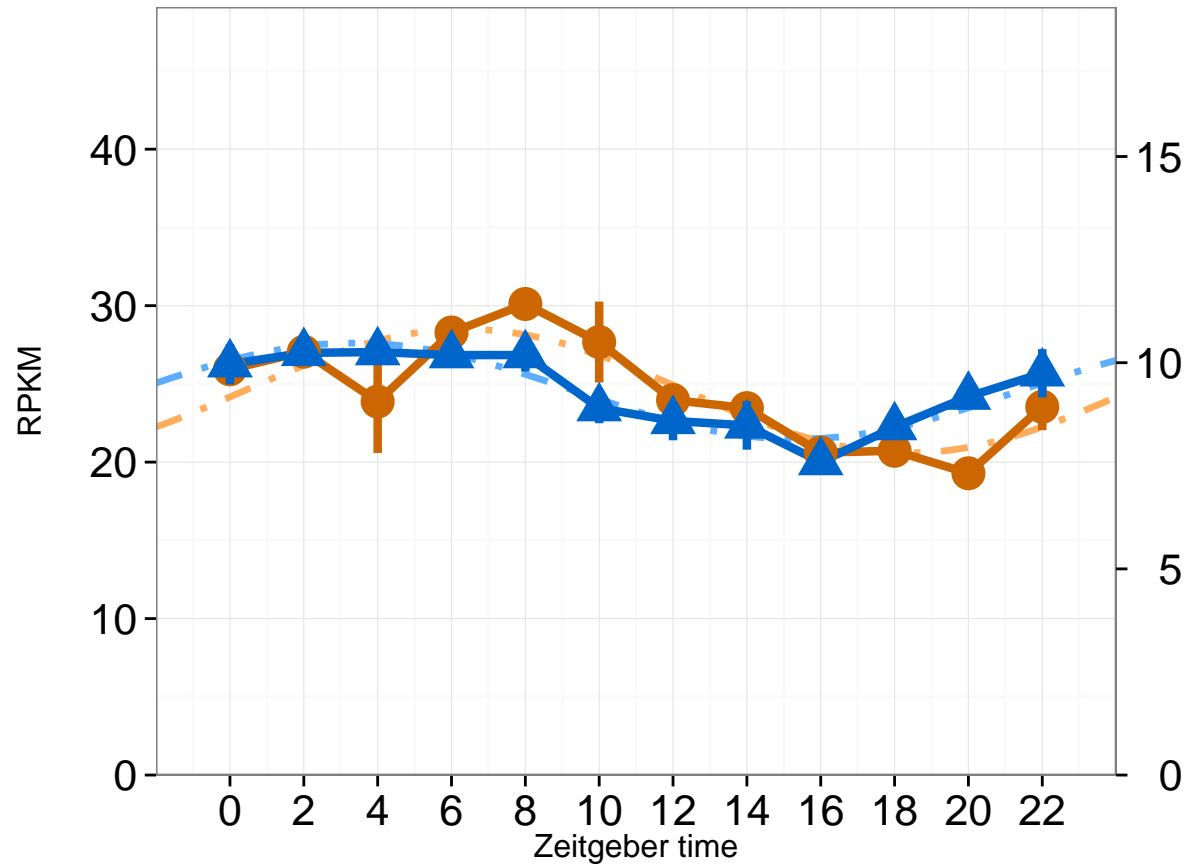

## 2310067B10Rik

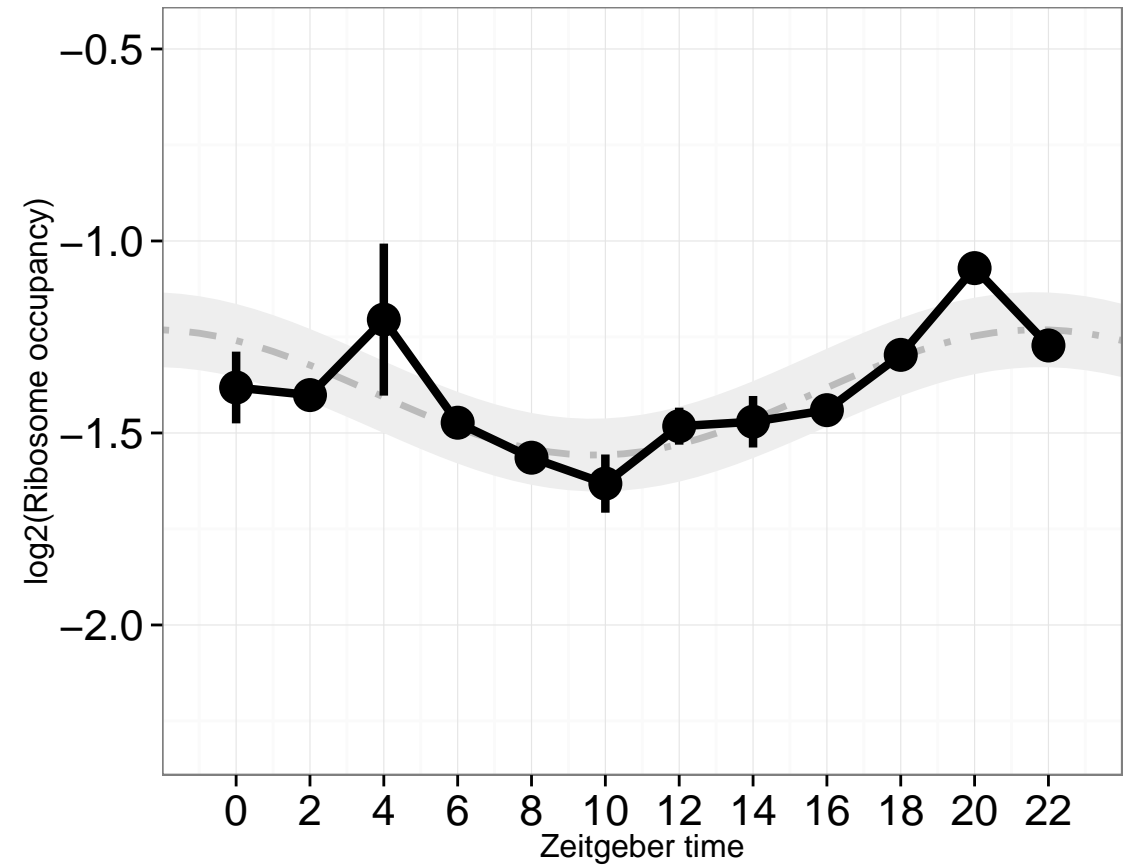

Supplement: Supplementary file 6 — Transcriptome-wide kidney RPF (blue) and RNA (orange) levels in the left panels (with “error bars” connecting the two replicates of each timepoint) and TE in the right panels. (ZIP 116896 kb) [file 13059_2017_1222_MOESM6_ESM.zip › Supp_Dataset_S1/A_RNA_non_rhythmic_RPF_non_rhythmic/2310067B10Rik_kidney_set_A.pdf]

# 2410004B18Rik

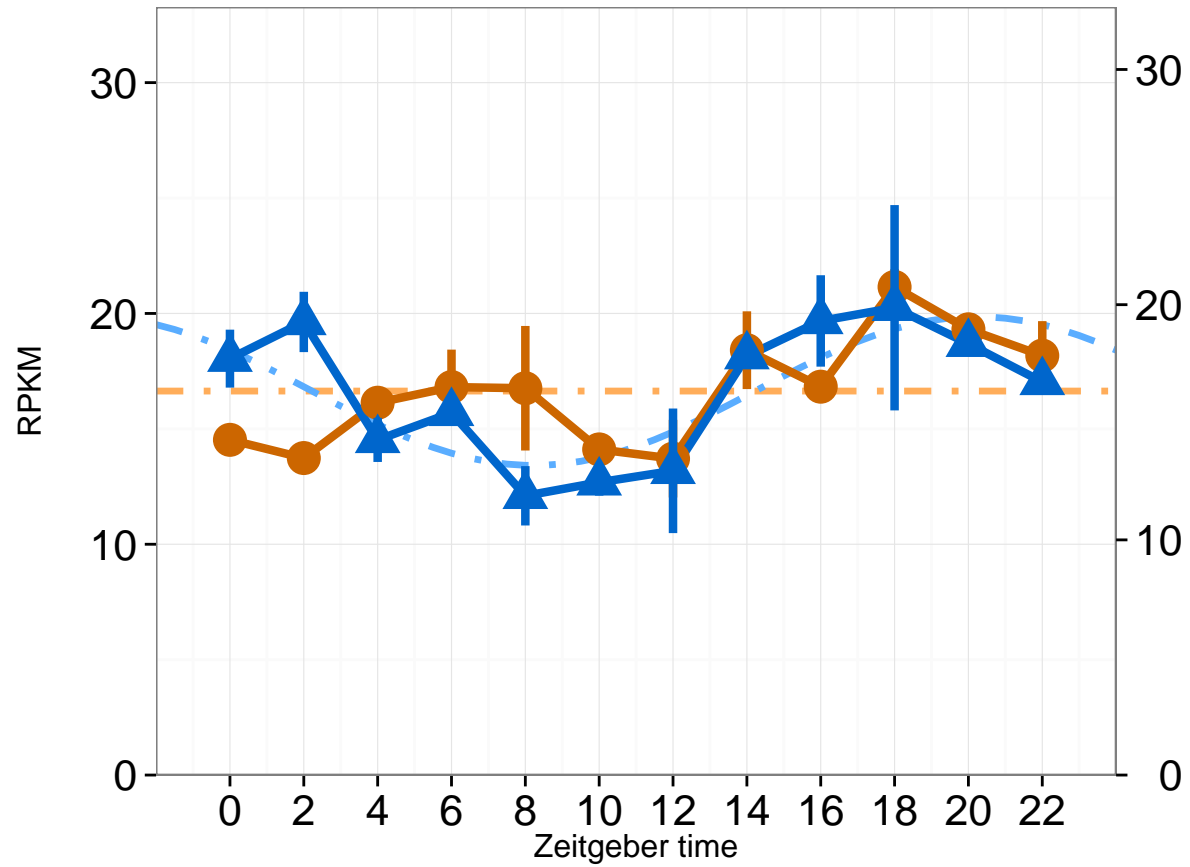

# 2410004B18Rik

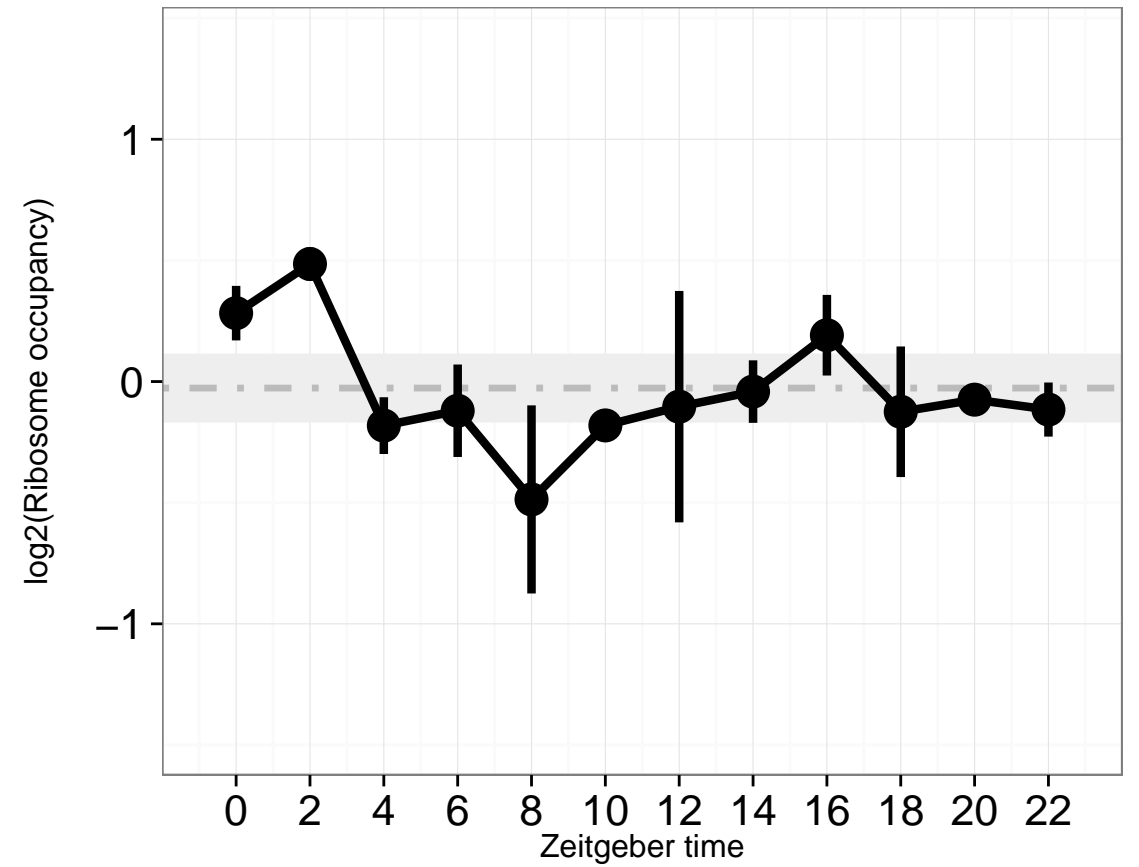

Supplement: Supplementary file 6 — Transcriptome-wide kidney RPF (blue) and RNA (orange) levels in the left panels (with “error bars” connecting the two replicates of each timepoint) and TE in the right panels. (ZIP 116896 kb) [file 13059_2017_1222_MOESM6_ESM.zip › Supp_Dataset_S1/A_RNA_non_rhythmic_RPF_non_rhythmic/2410004B18Rik_kidney_set_A.pdf]

## 2410015M20Rik

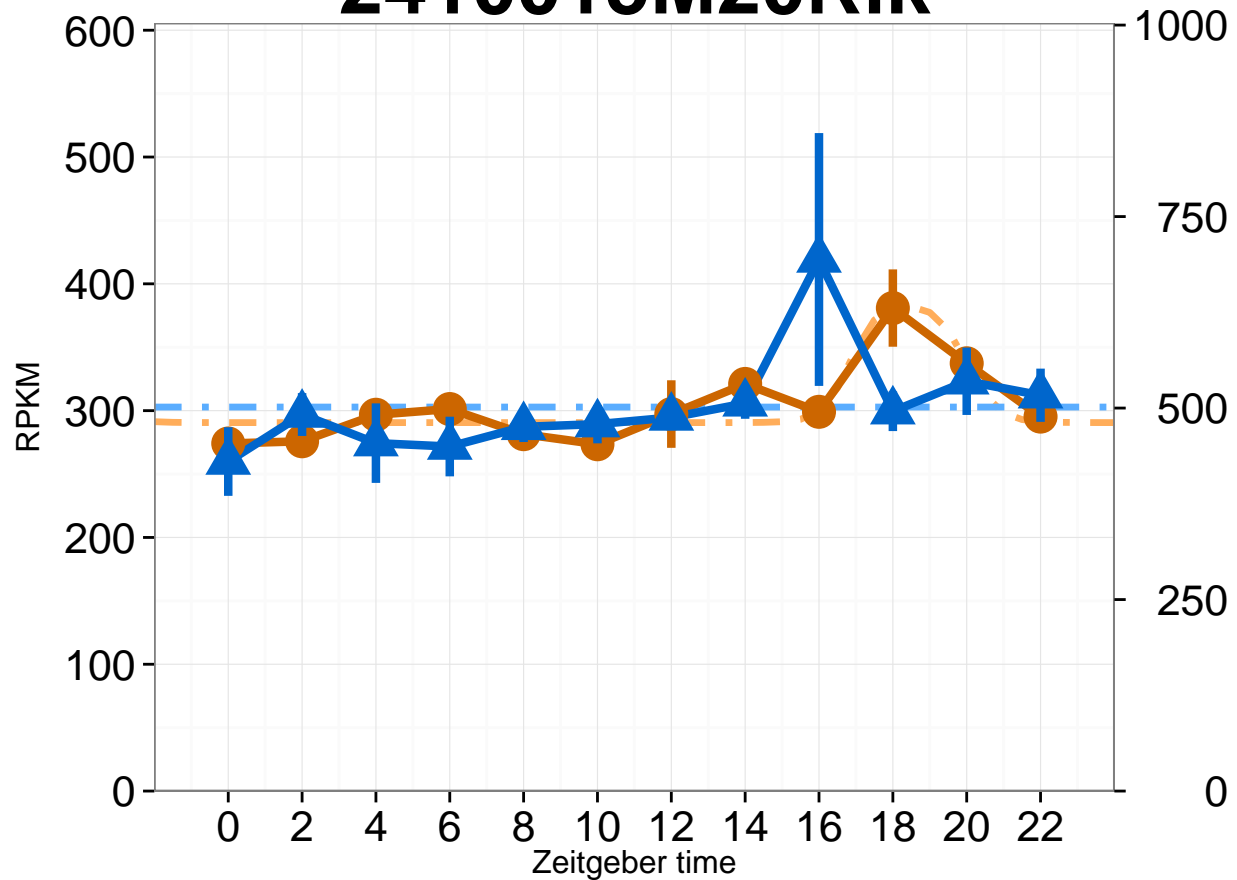

## 2410015M20Rik

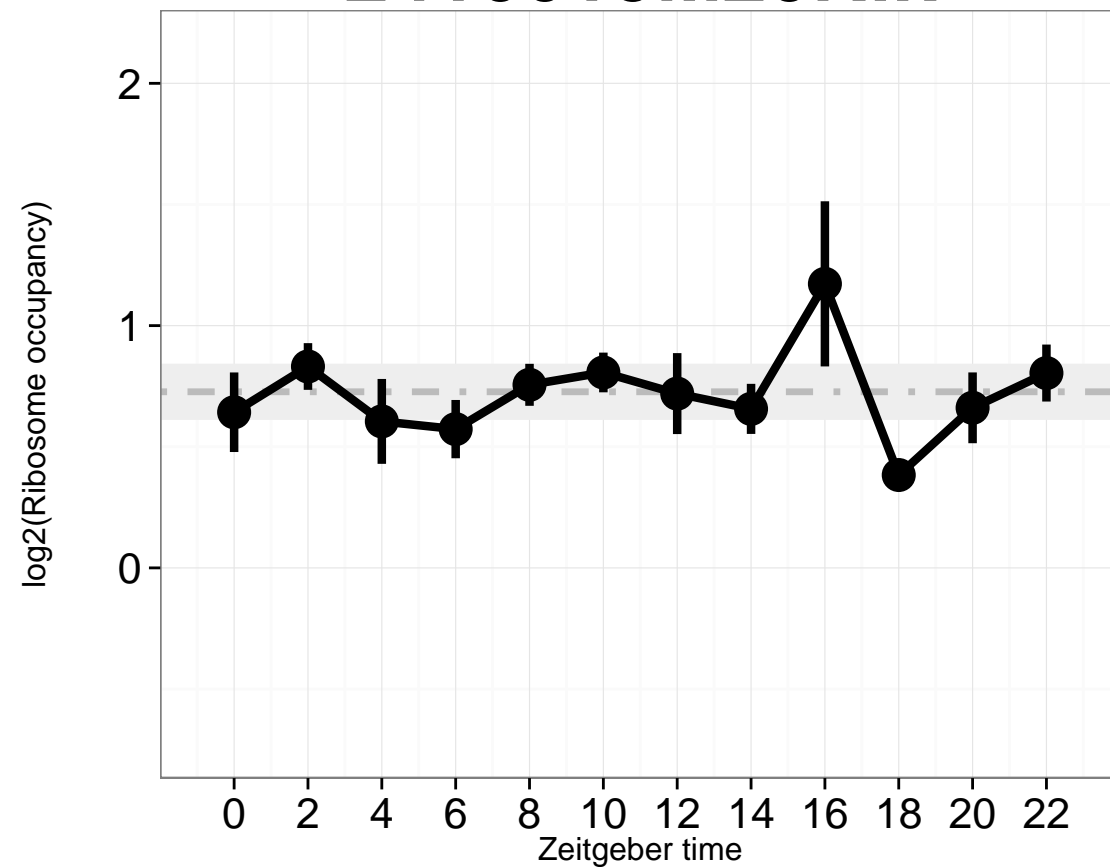

Supplement: Supplementary file 6 — Transcriptome-wide kidney RPF (blue) and RNA (orange) levels in the left panels (with “error bars” connecting the two replicates of each timepoint) and TE in the right panels. (ZIP 116896 kb) [file 13059_2017_1222_MOESM6_ESM.zip › Supp_Dataset_S1/A_RNA_non_rhythmic_RPF_non_rhythmic/2410015M20Rik_kidney_set_A.pdf]

## 2410016O06Rik

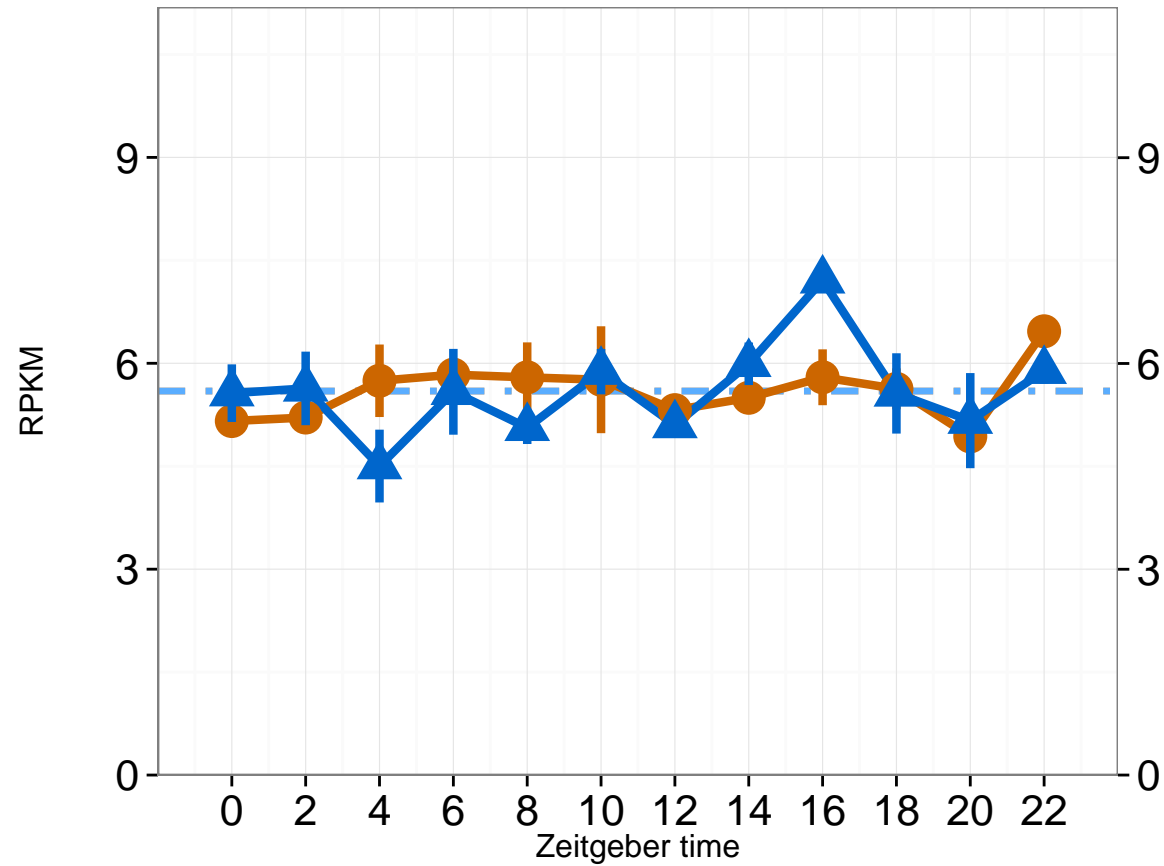

## 2410016O06Rik

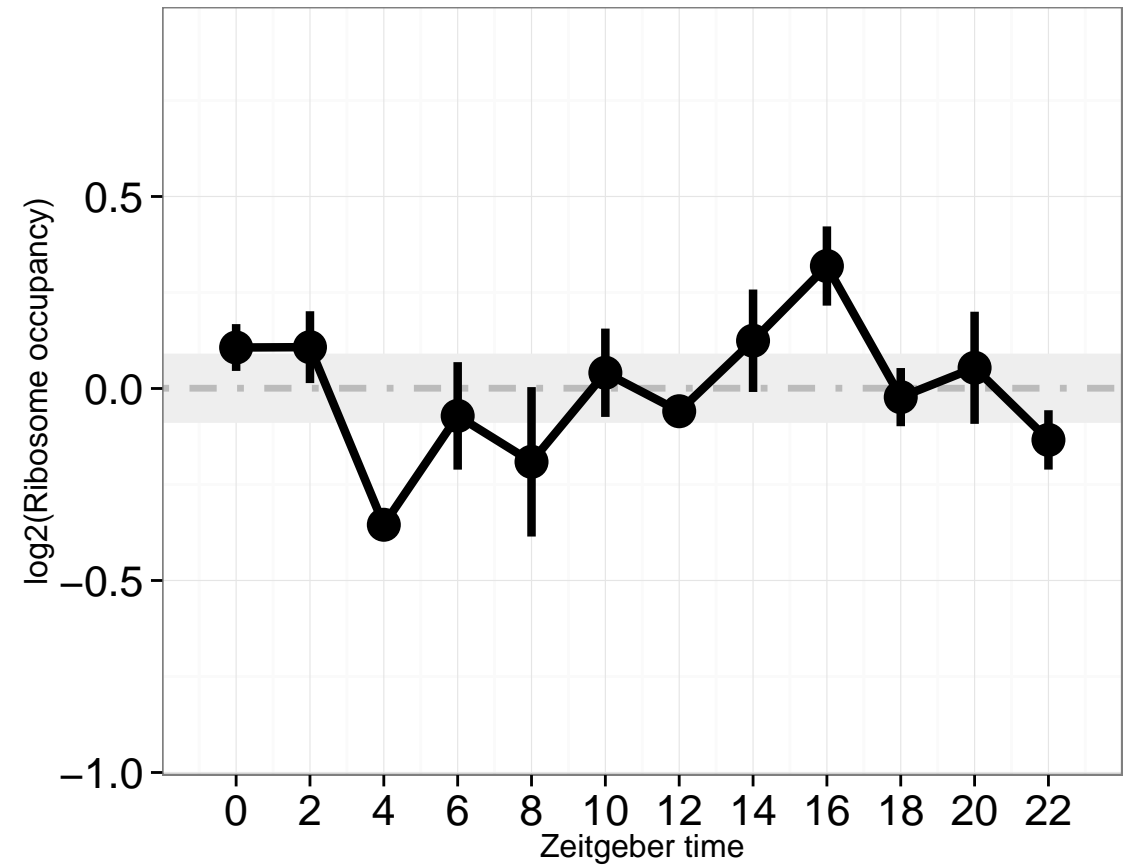

Supplement: Supplementary file 6 — Transcriptome-wide kidney RPF (blue) and RNA (orange) levels in the left panels (with “error bars” connecting the two replicates of each timepoint) and TE in the right panels. (ZIP 116896 kb) [file 13059_2017_1222_MOESM6_ESM.zip › Supp_Dataset_S1/A_RNA_non_rhythmic_RPF_non_rhythmic/2410016O06Rik_kidney_set_A.pdf]

## 2410066E13Rik

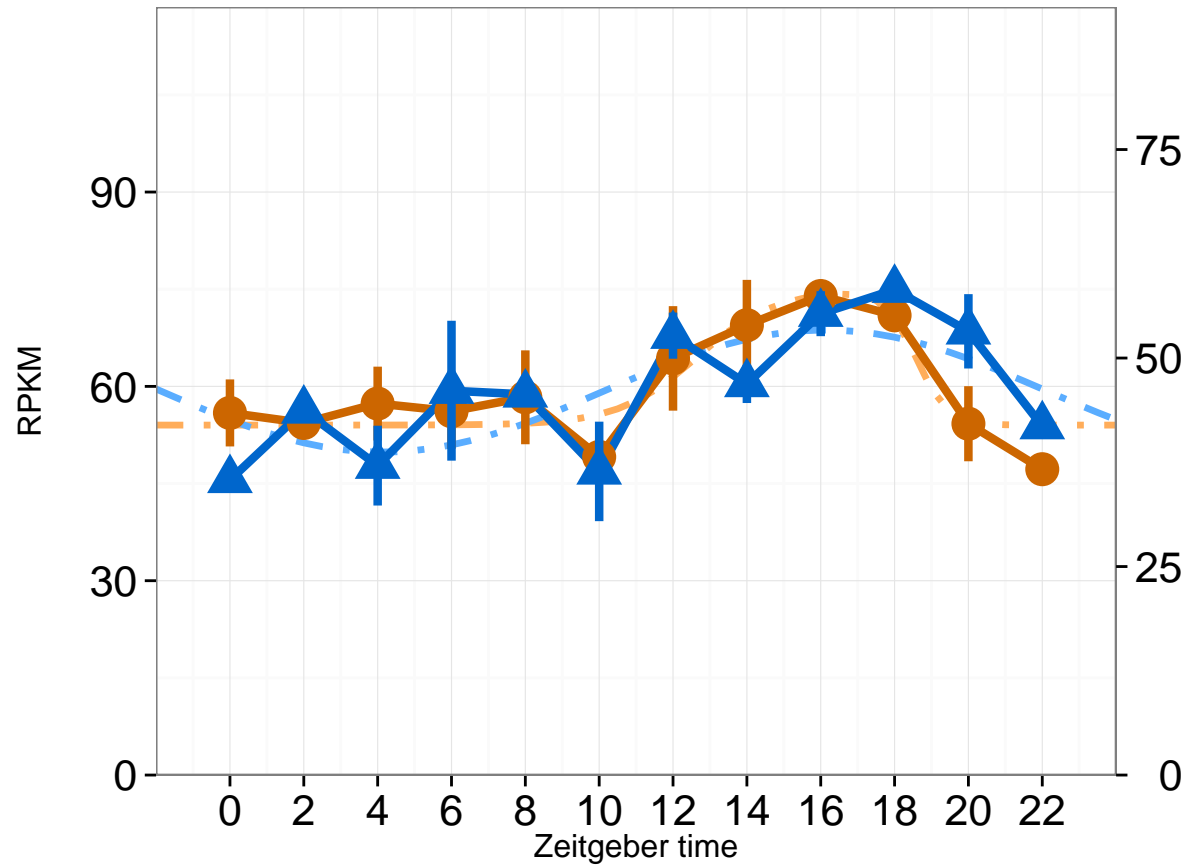

## 2410066E13Rik

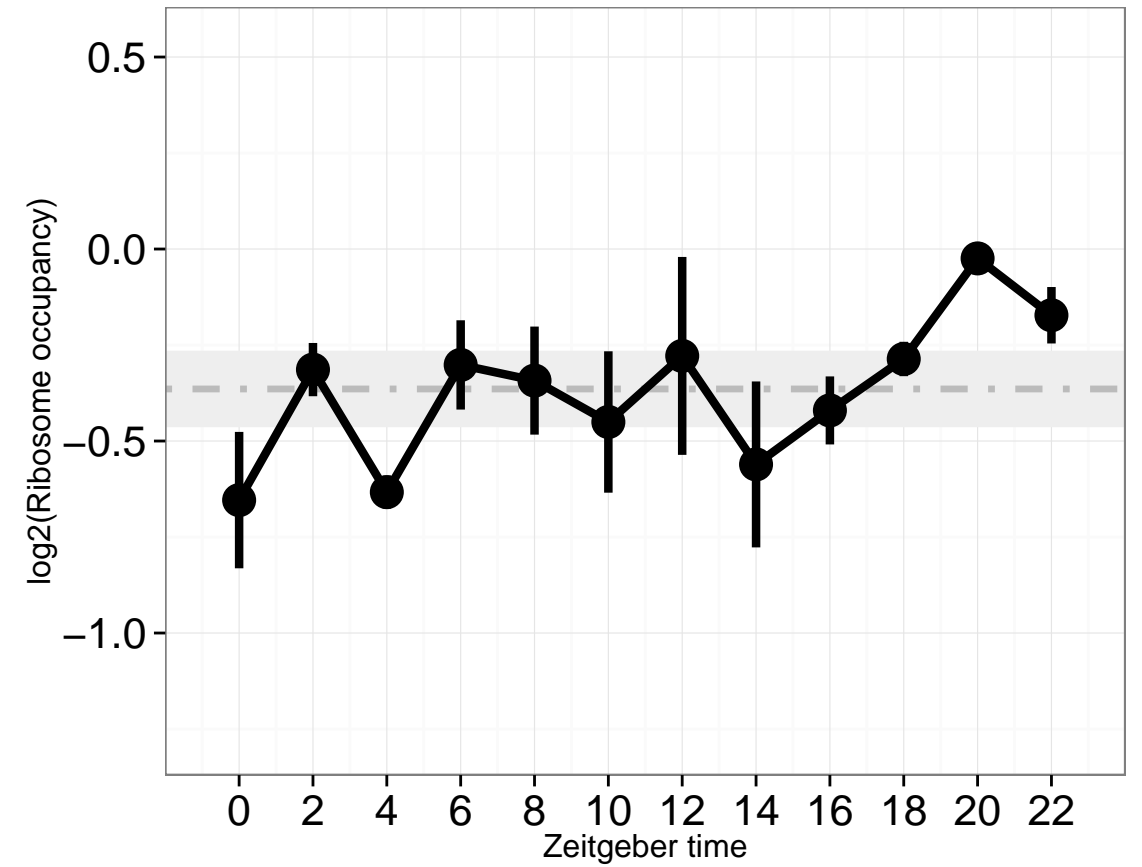

Supplement: Supplementary file 6 — Transcriptome-wide kidney RPF (blue) and RNA (orange) levels in the left panels (with “error bars” connecting the two replicates of each timepoint) and TE in the right panels. (ZIP 116896 kb) [file 13059_2017_1222_MOESM6_ESM.zip › Supp_Dataset_S1/A_RNA_non_rhythmic_RPF_non_rhythmic/2410066E13Rik_kidney_set_A.pdf]

## 2410127L17Rik

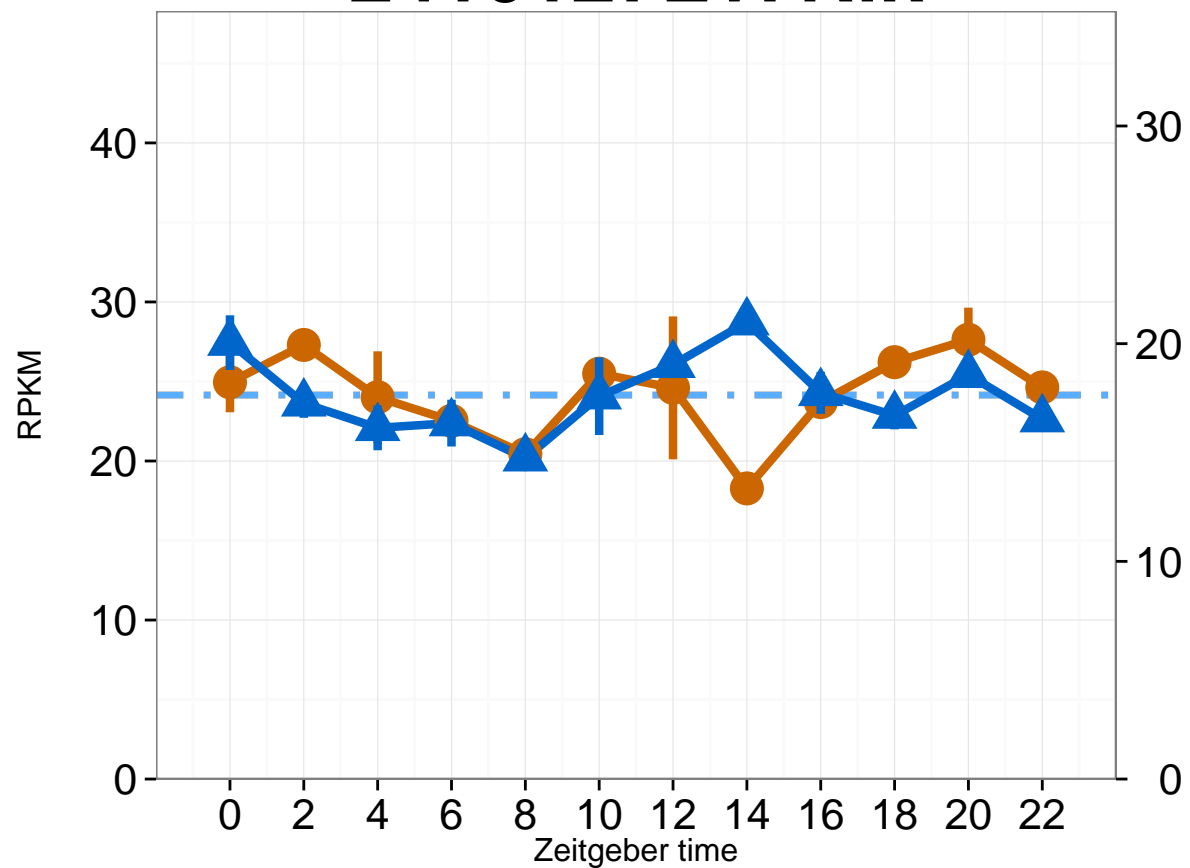

## 2410127L17Rik

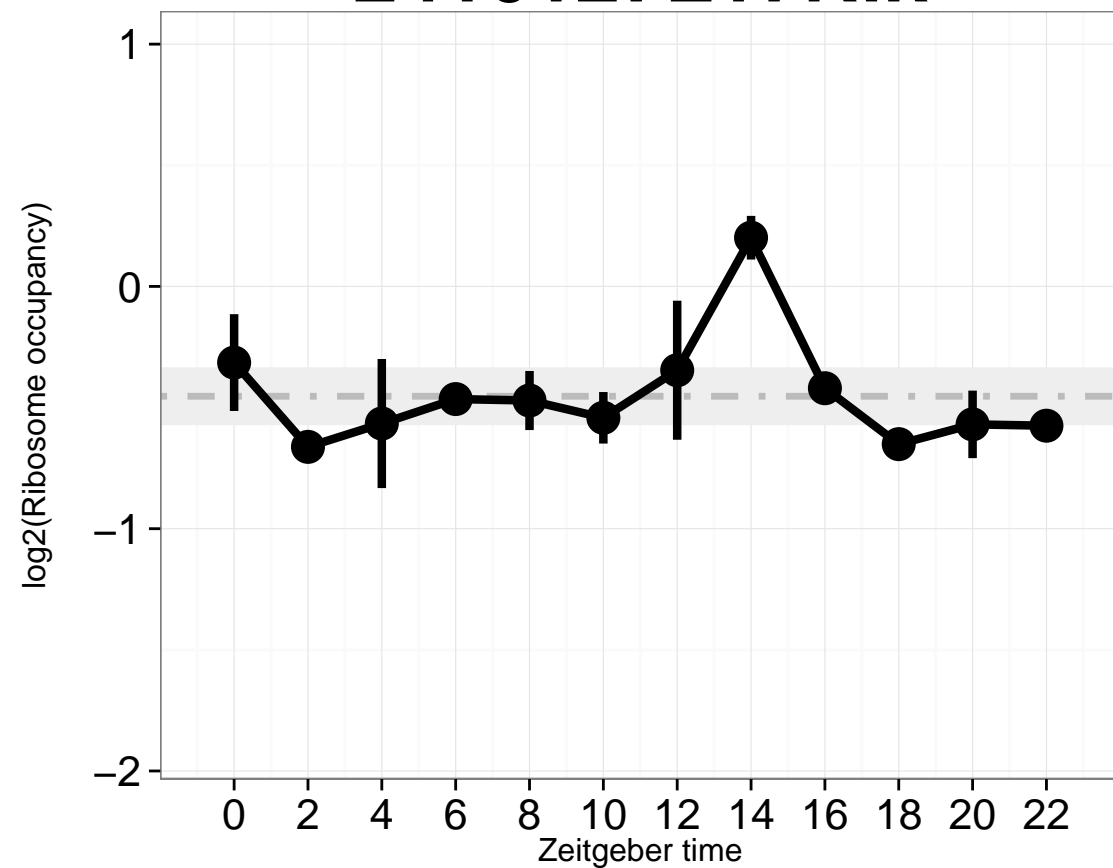

Supplement: Supplementary file 6 — Transcriptome-wide kidney RPF (blue) and RNA (orange) levels in the left panels (with “error bars” connecting the two replicates of each timepoint) and TE in the right panels. (ZIP 116896 kb) [file 13059_2017_1222_MOESM6_ESM.zip › Supp_Dataset_S1/A_RNA_non_rhythmic_RPF_non_rhythmic/2410127L17Rik_kidney_set_A.pdf]

## 2410131K14Rik

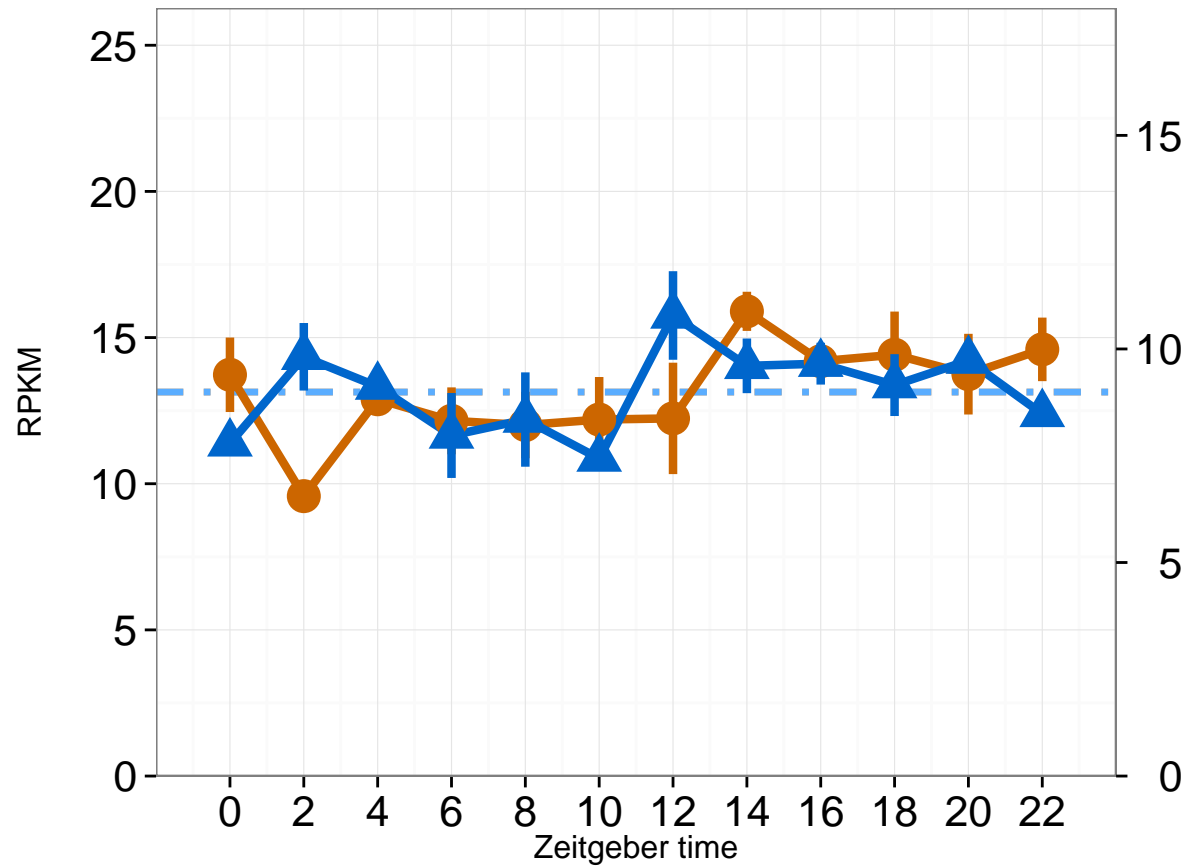

## 2410131K14Rik

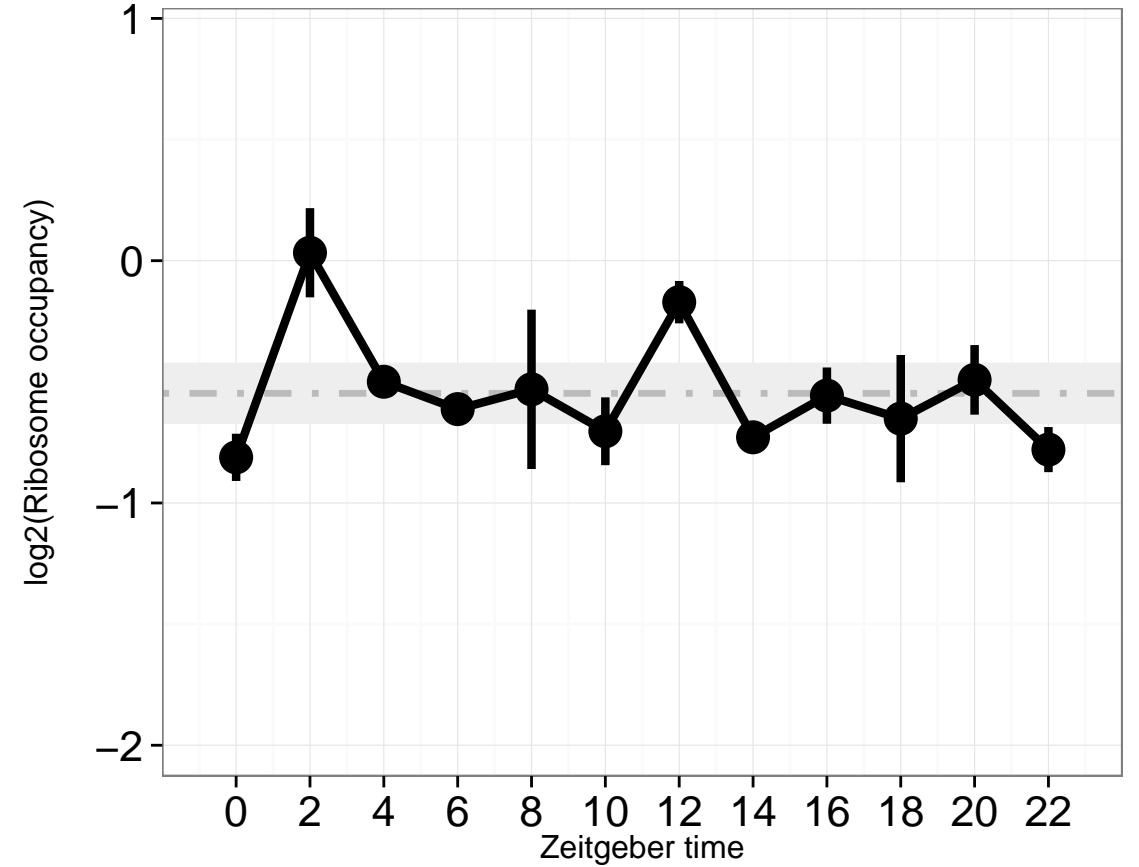

Supplement: Supplementary file 6 — Transcriptome-wide kidney RPF (blue) and RNA (orange) levels in the left panels (with “error bars” connecting the two replicates of each timepoint) and TE in the right panels. (ZIP 116896 kb) [file 13059_2017_1222_MOESM6_ESM.zip › Supp_Dataset_S1/A_RNA_non_rhythmic_RPF_non_rhythmic/2410131K14Rik_kidney_set_A.pdf]
