# Supplementary figures and images for: Translational contributions to tissue specificity in rhythmic and constitutive gene expression (part 4 of 4)
Source: Genome Biol. 2017 Jun 16;18:116. doi: 10.1186/s13059-017-1222-2 (PMC5473967; doi:10.1186/s13059-017-1222-2)

## Dbp

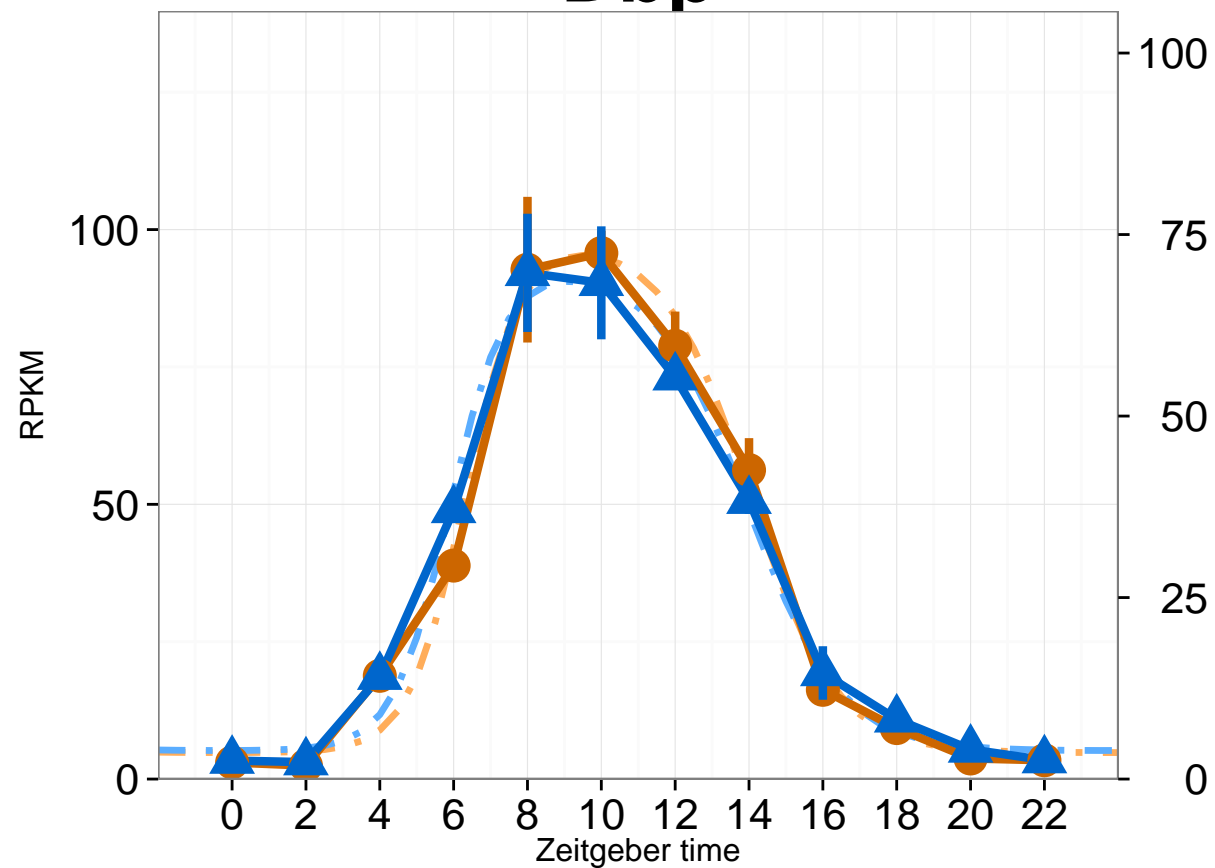

## Dbp

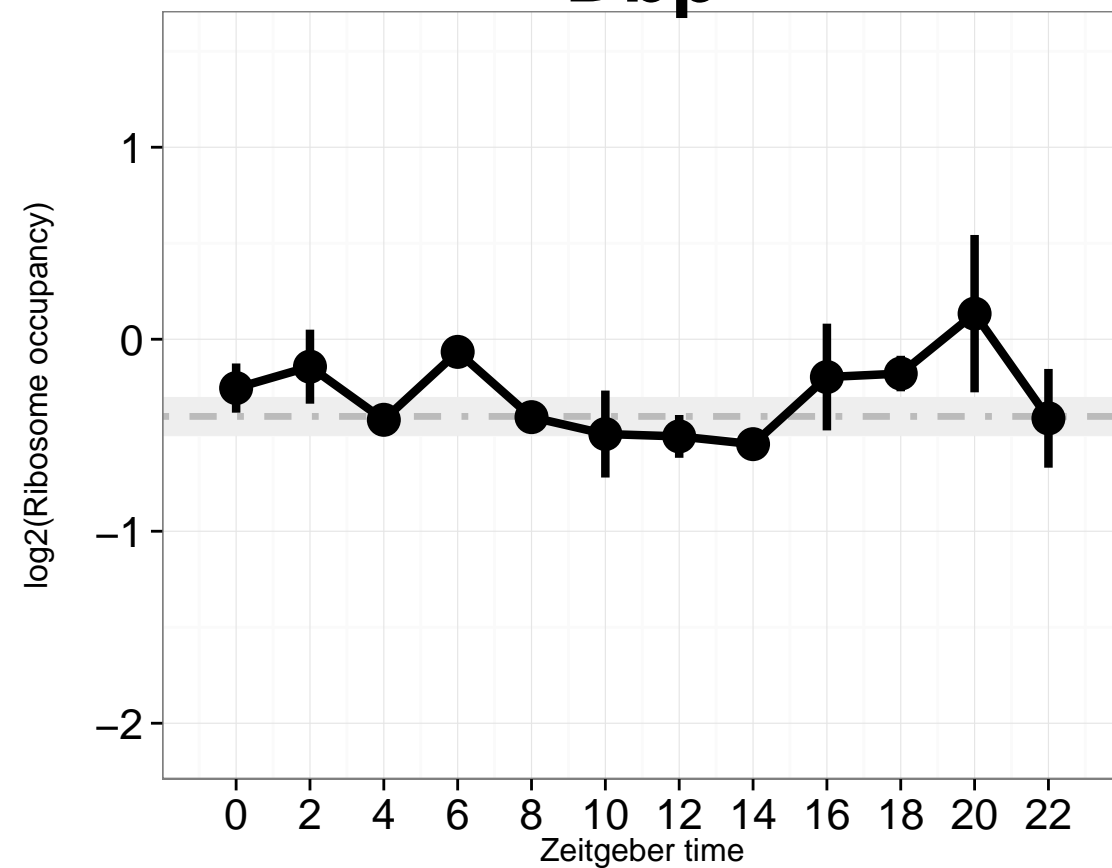

Supplement: Supplementary file 7 — Expression plots for kidney and liver for the 178 common rhythmic genes of Fig. 3c. (ZIP 3338.28 kb) [file 13059_2017_1222_MOESM7_ESM.zip › set_D_shared(178)/Dbp_kidney_set_D.pdf]

## Dbp

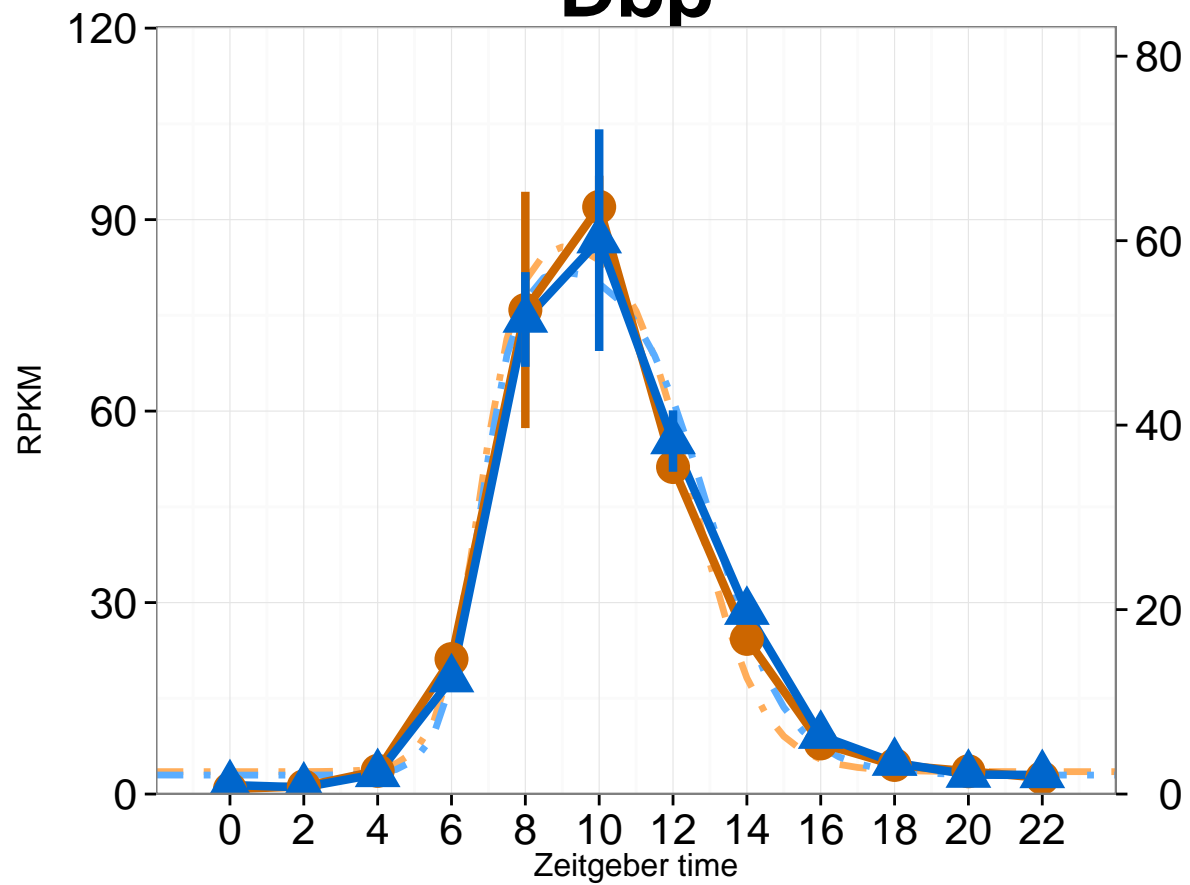

## Dbp

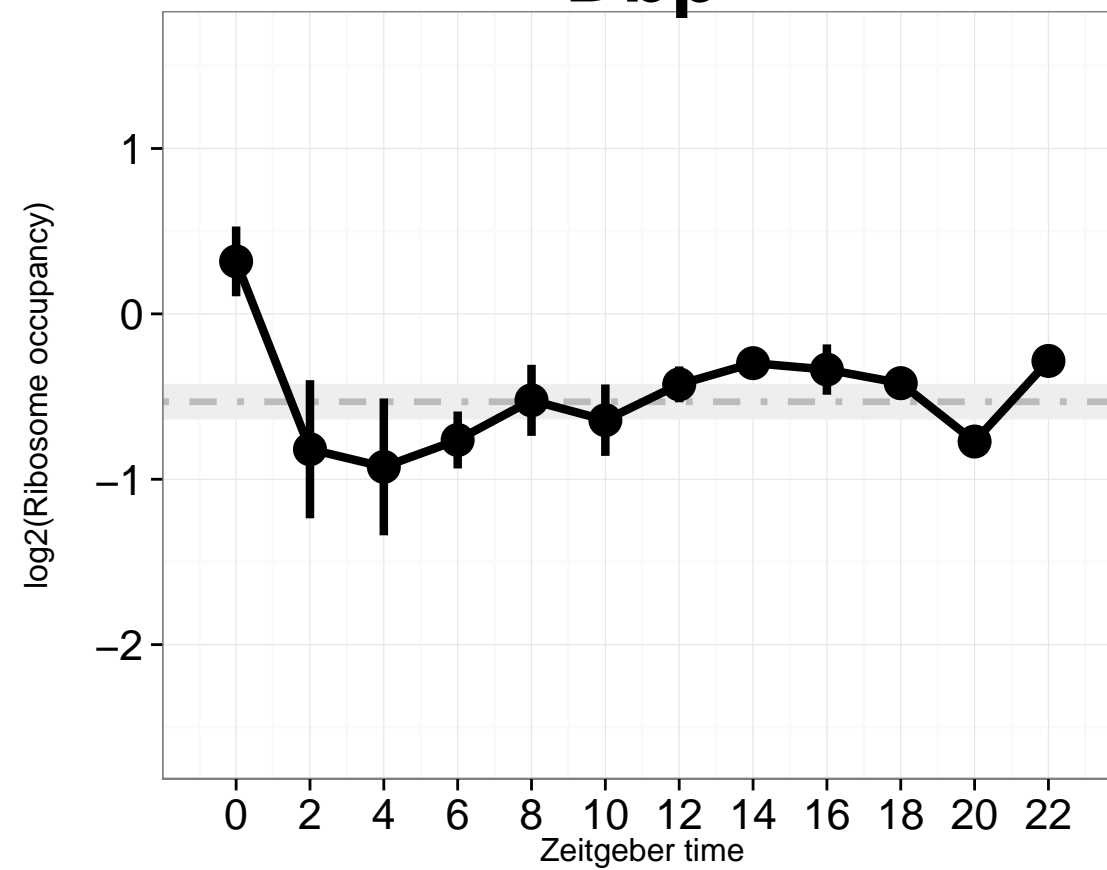

Supplement: Supplementary file 7 — Expression plots for kidney and liver for the 178 common rhythmic genes of Fig. 3c. (ZIP 3338.28 kb) [file 13059_2017_1222_MOESM7_ESM.zip › set_D_shared(178)/Dbp_liver_set_D.pdf]

## Ddc

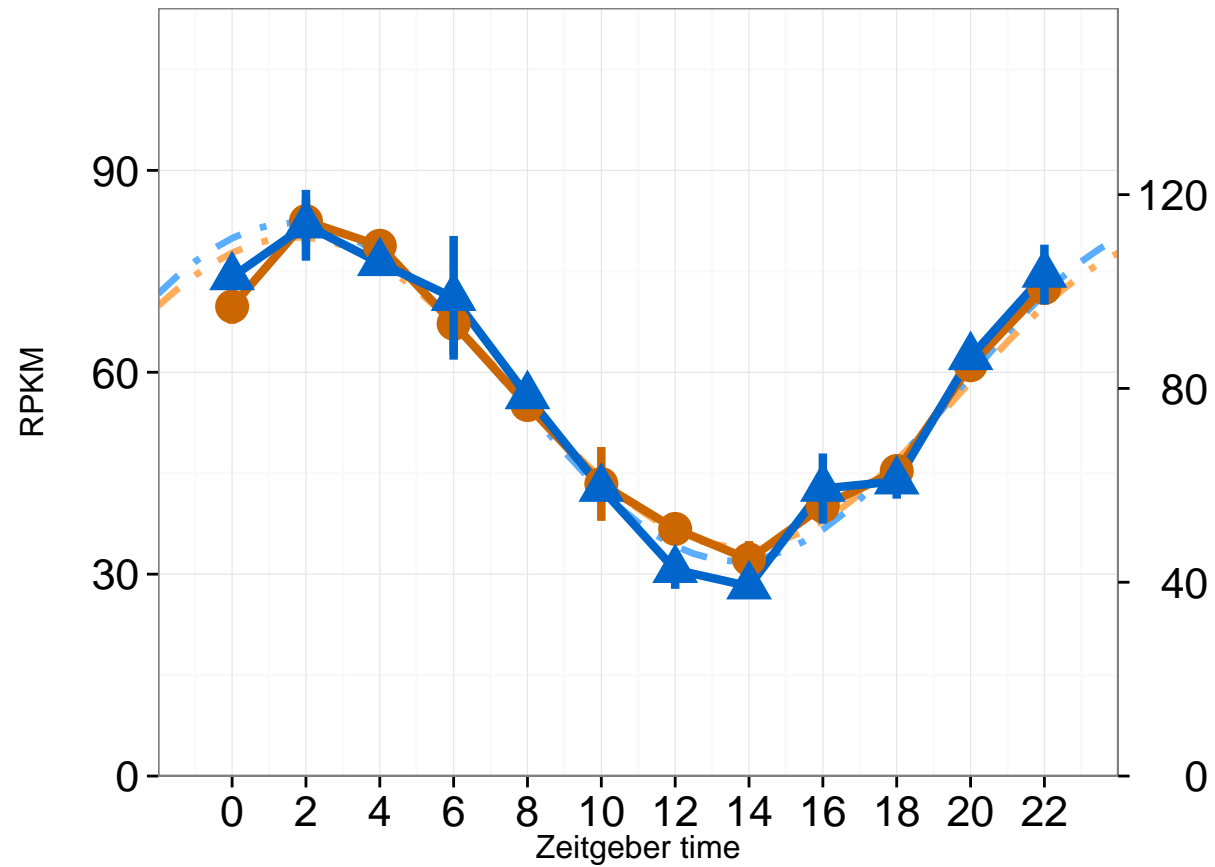

## Ddc

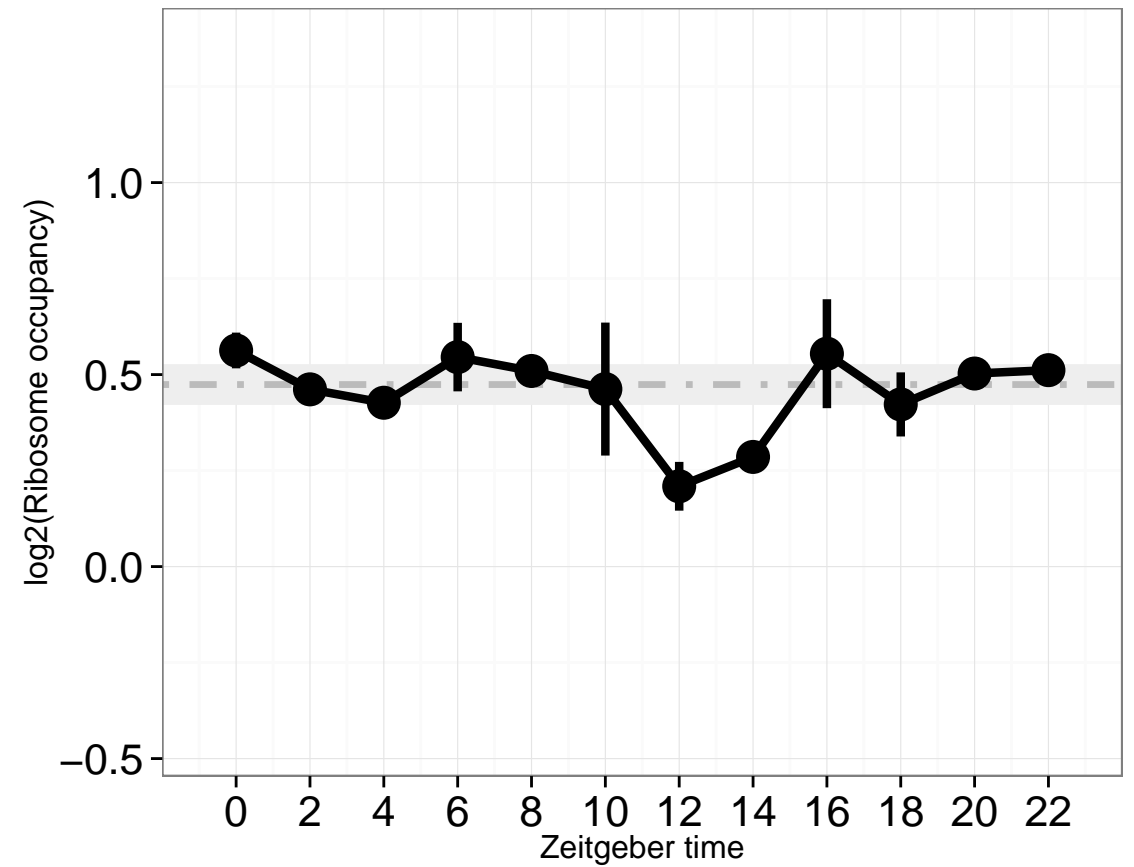

Supplement: Supplementary file 7 — Expression plots for kidney and liver for the 178 common rhythmic genes of Fig. 3c. (ZIP 3338.28 kb) [file 13059_2017_1222_MOESM7_ESM.zip › set_D_shared(178)/Ddc_kidney_set_D.pdf]

## Ddc

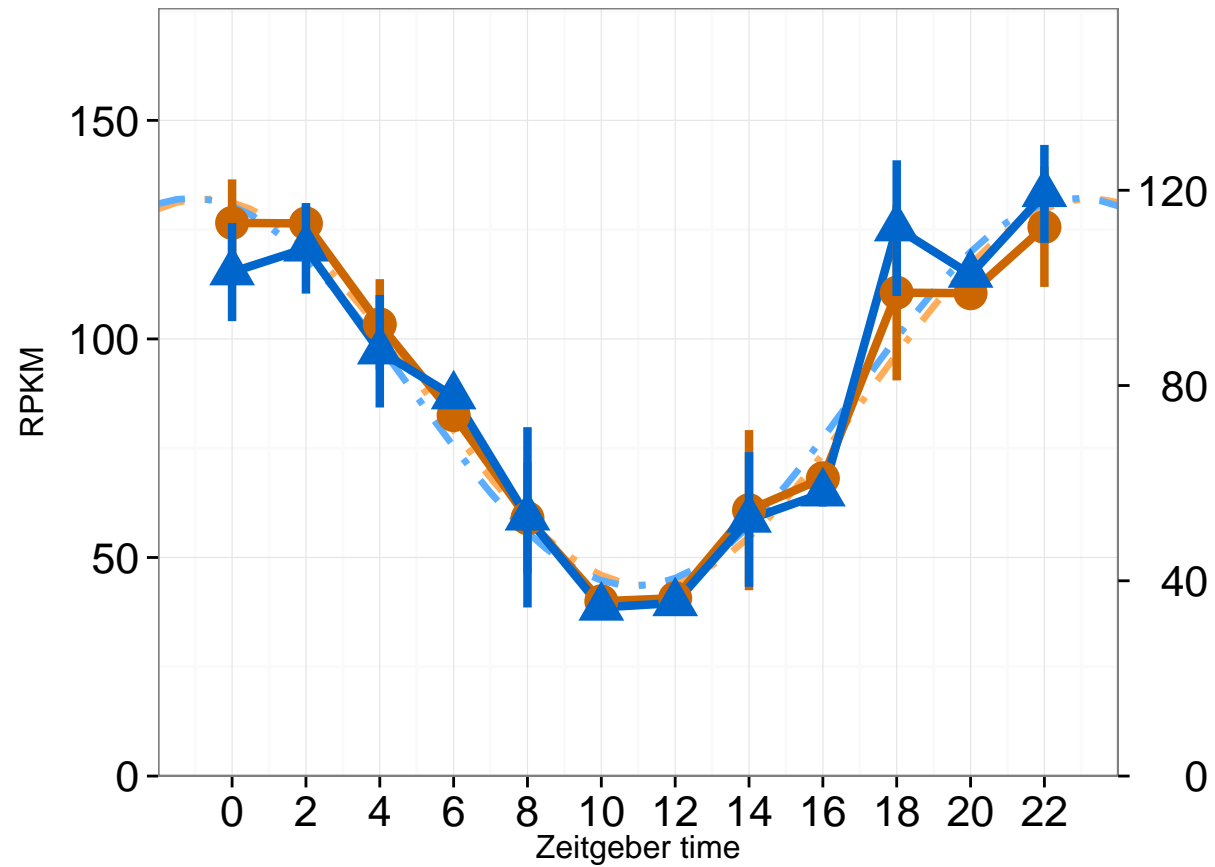

## Ddc

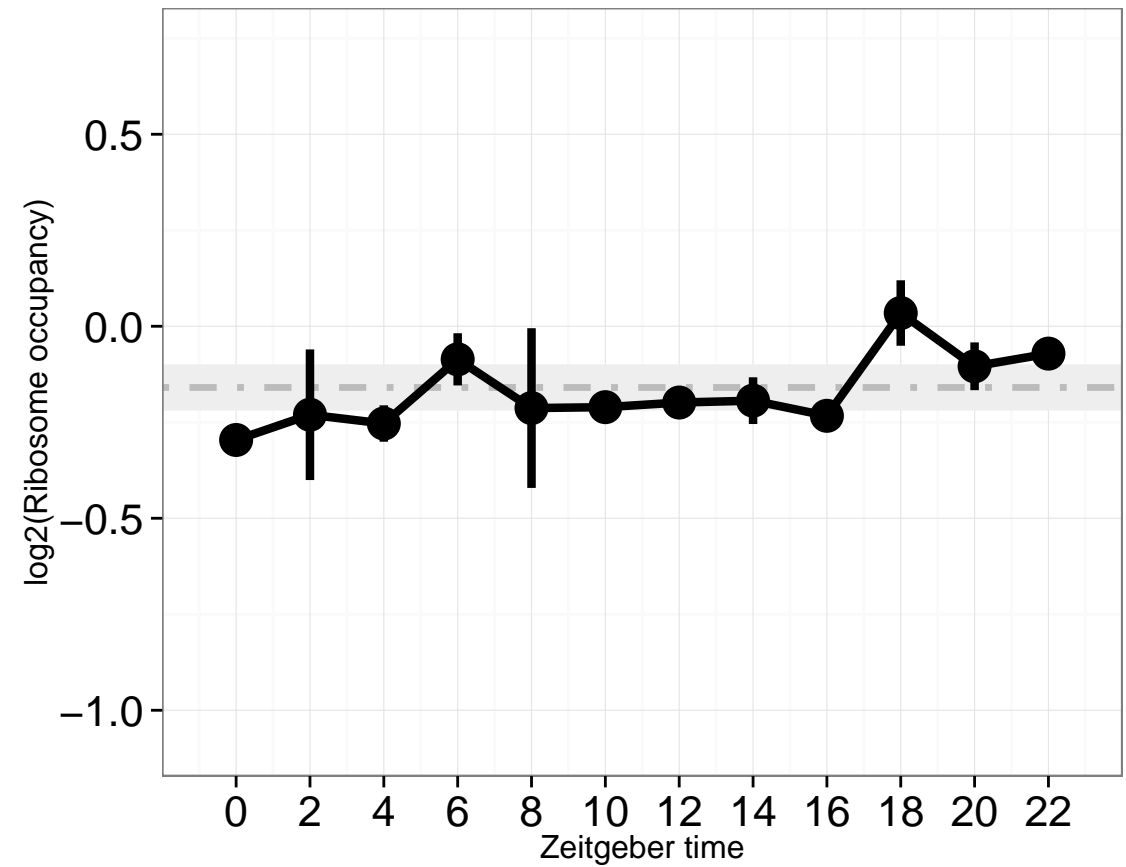

Supplement: Supplementary file 7 — Expression plots for kidney and liver for the 178 common rhythmic genes of Fig. 3c. (ZIP 3338.28 kb) [file 13059_2017_1222_MOESM7_ESM.zip › set_D_shared(178)/Ddc_liver_set_D.pdf]

## Ddhd1

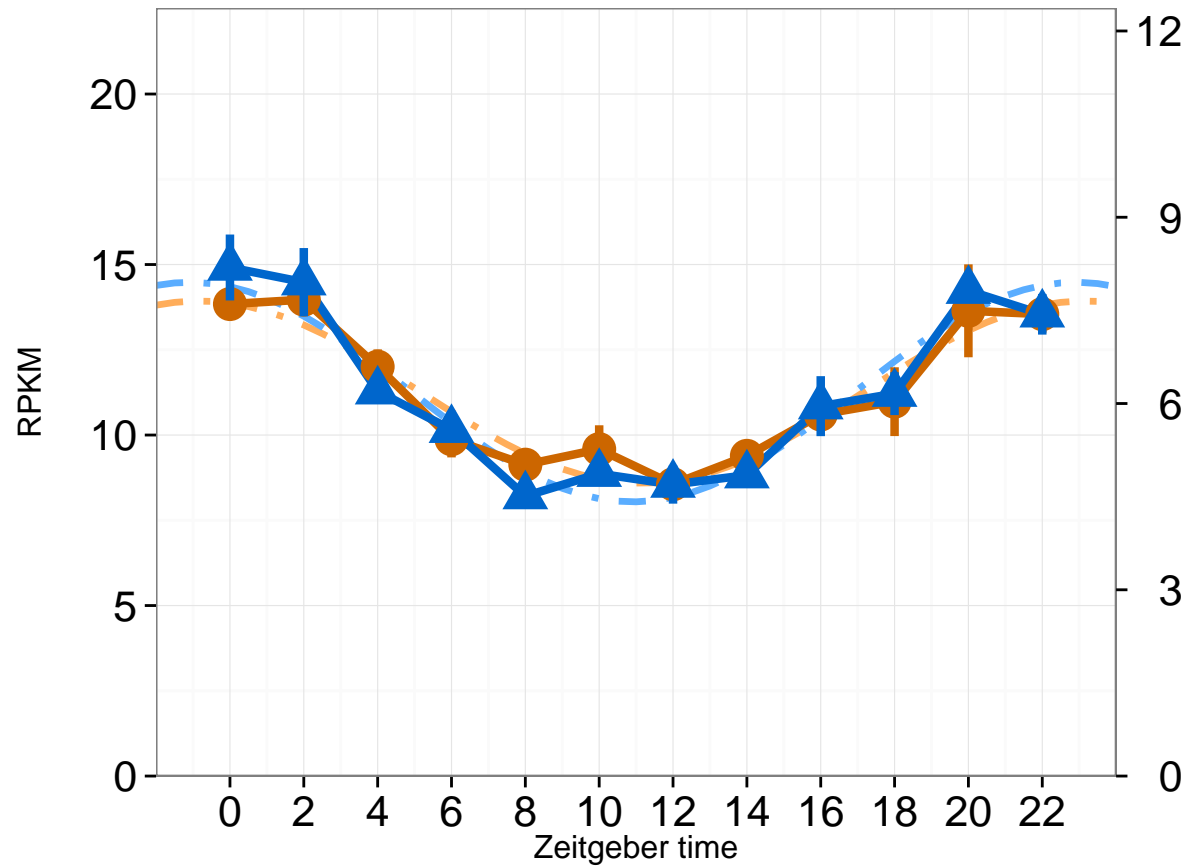

## Ddhd1

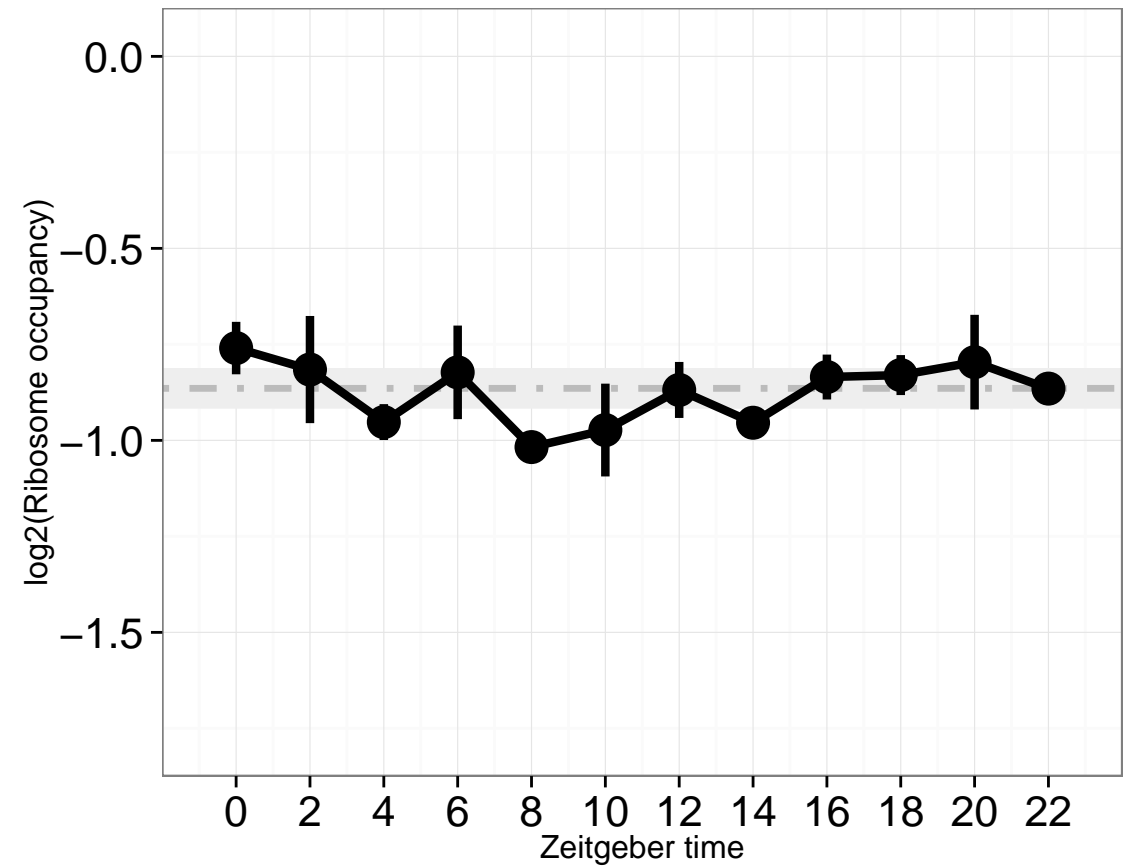

Supplement: Supplementary file 7 — Expression plots for kidney and liver for the 178 common rhythmic genes of Fig. 3c. (ZIP 3338.28 kb) [file 13059_2017_1222_MOESM7_ESM.zip › set_D_shared(178)/Ddhd1_kidney_set_D.pdf]

# Ddhd1

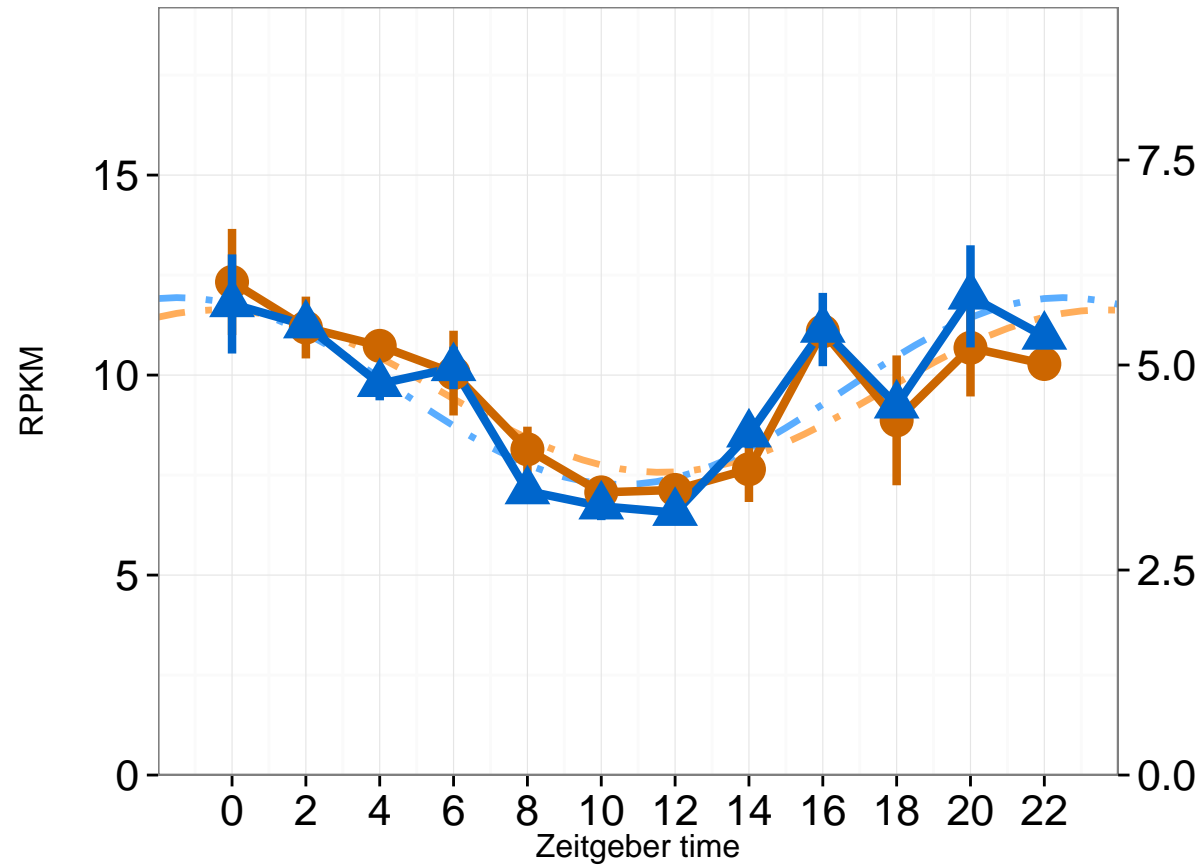

# Ddhd1

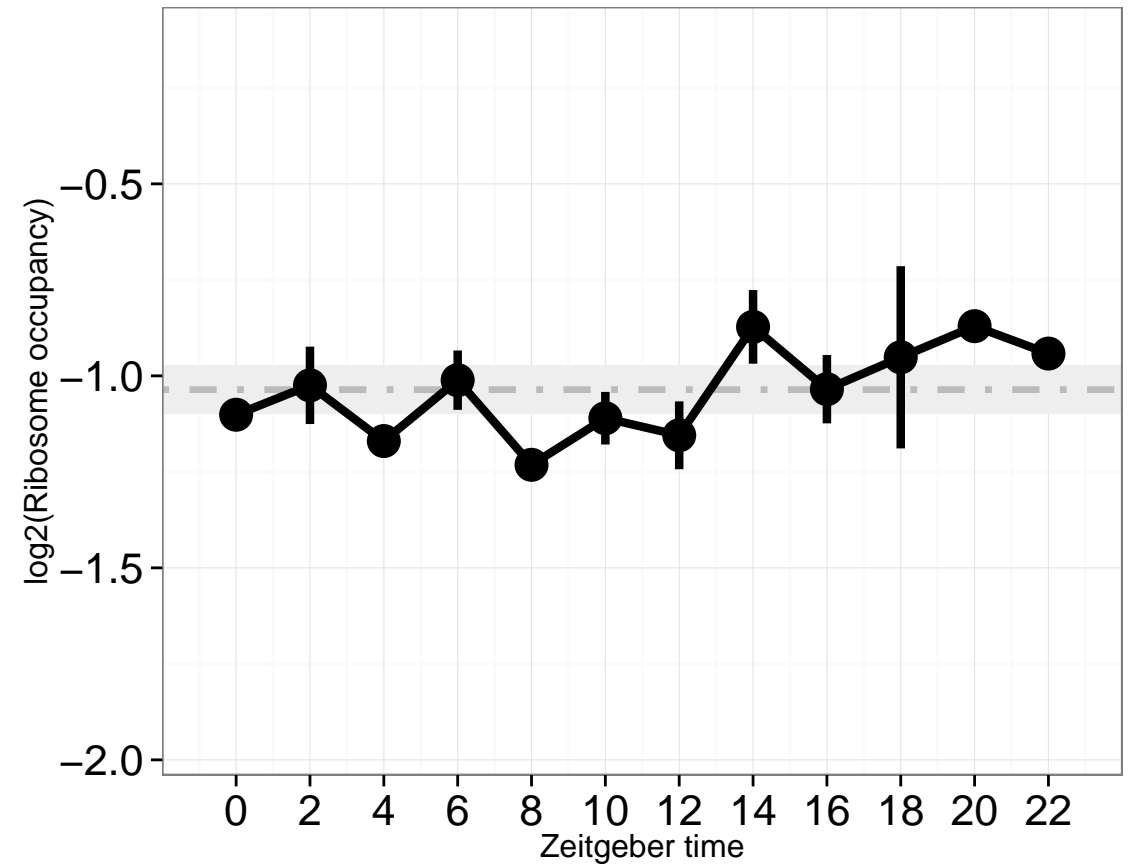

Supplement: Supplementary file 7 — Expression plots for kidney and liver for the 178 common rhythmic genes of Fig. 3c. (ZIP 3338.28 kb) [file 13059_2017_1222_MOESM7_ESM.zip › set_D_shared(178)/Ddhd1_liver_set_D.pdf]

## Ddx17

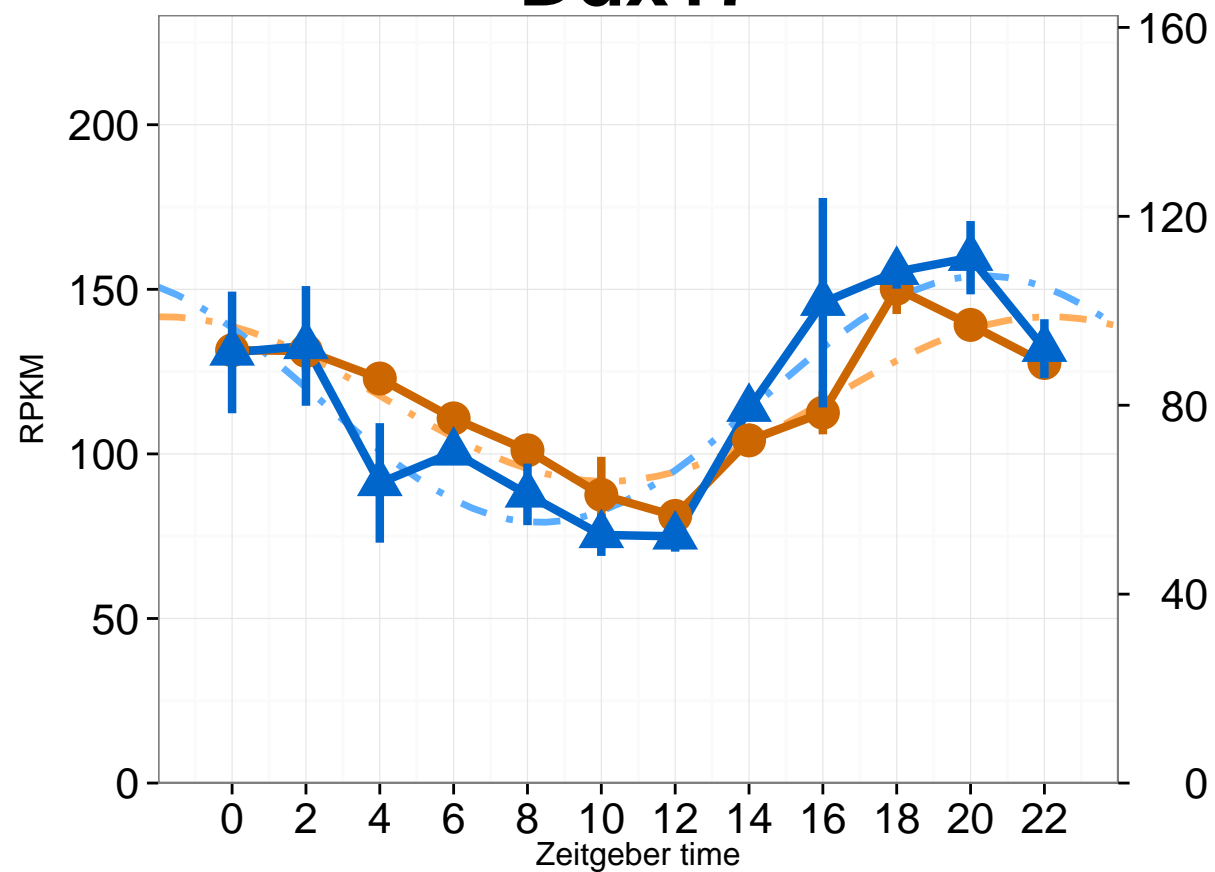

## Ddx17

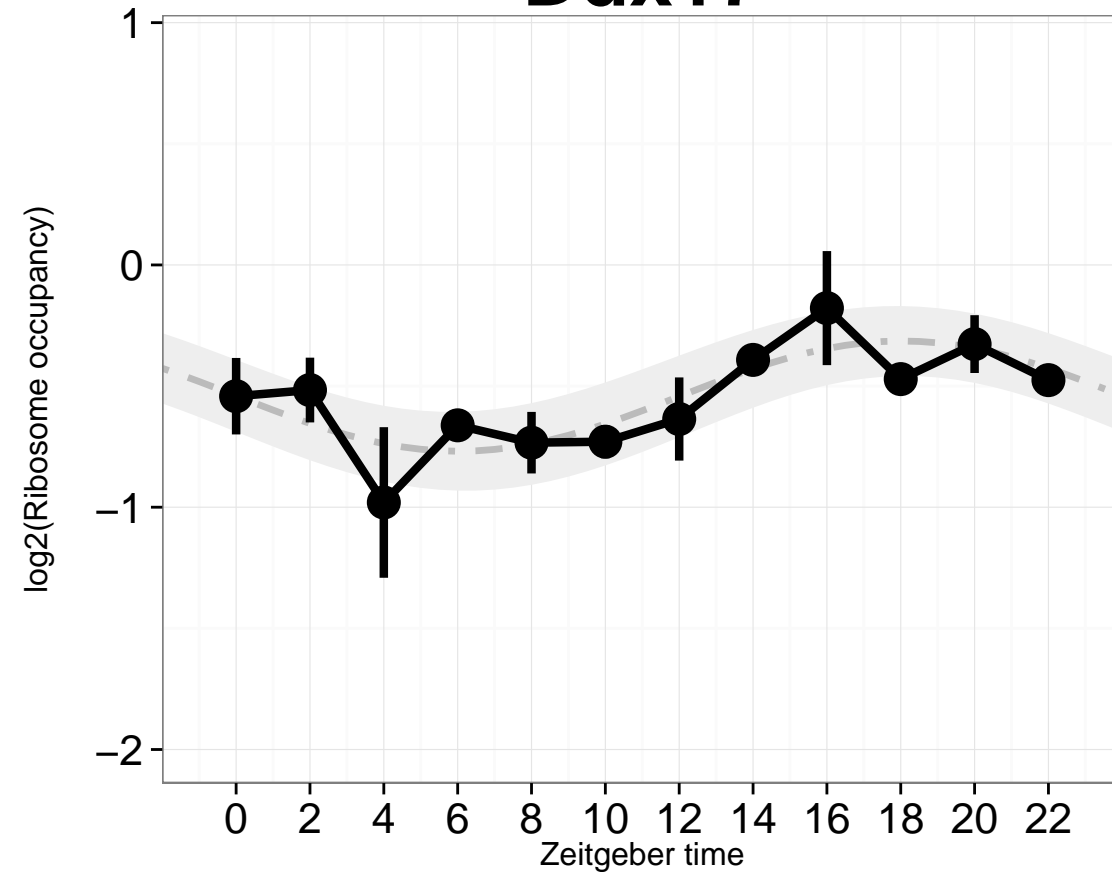

Supplement: Supplementary file 7 — Expression plots for kidney and liver for the 178 common rhythmic genes of Fig. 3c. (ZIP 3338.28 kb) [file 13059_2017_1222_MOESM7_ESM.zip › set_D_shared(178)/Ddx17_kidney_set_D.pdf]

# Ddx17

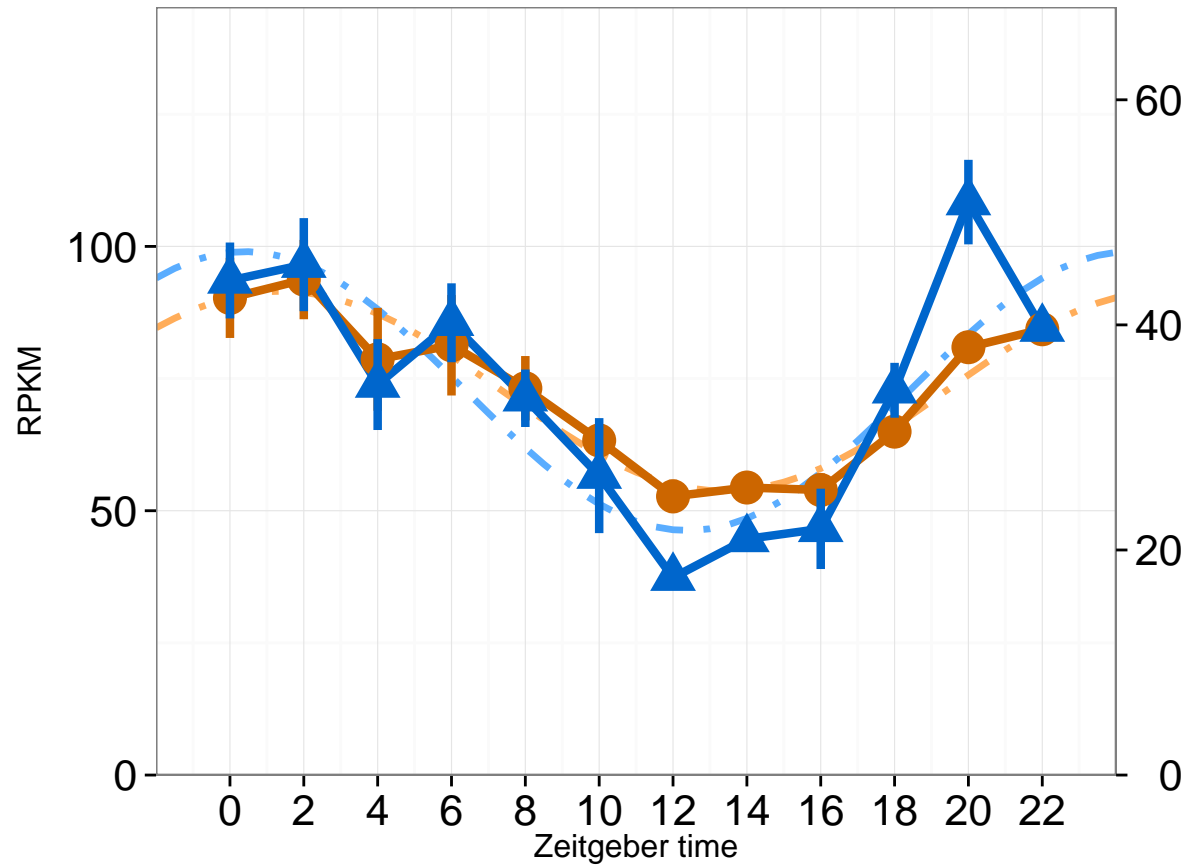

# Ddx17

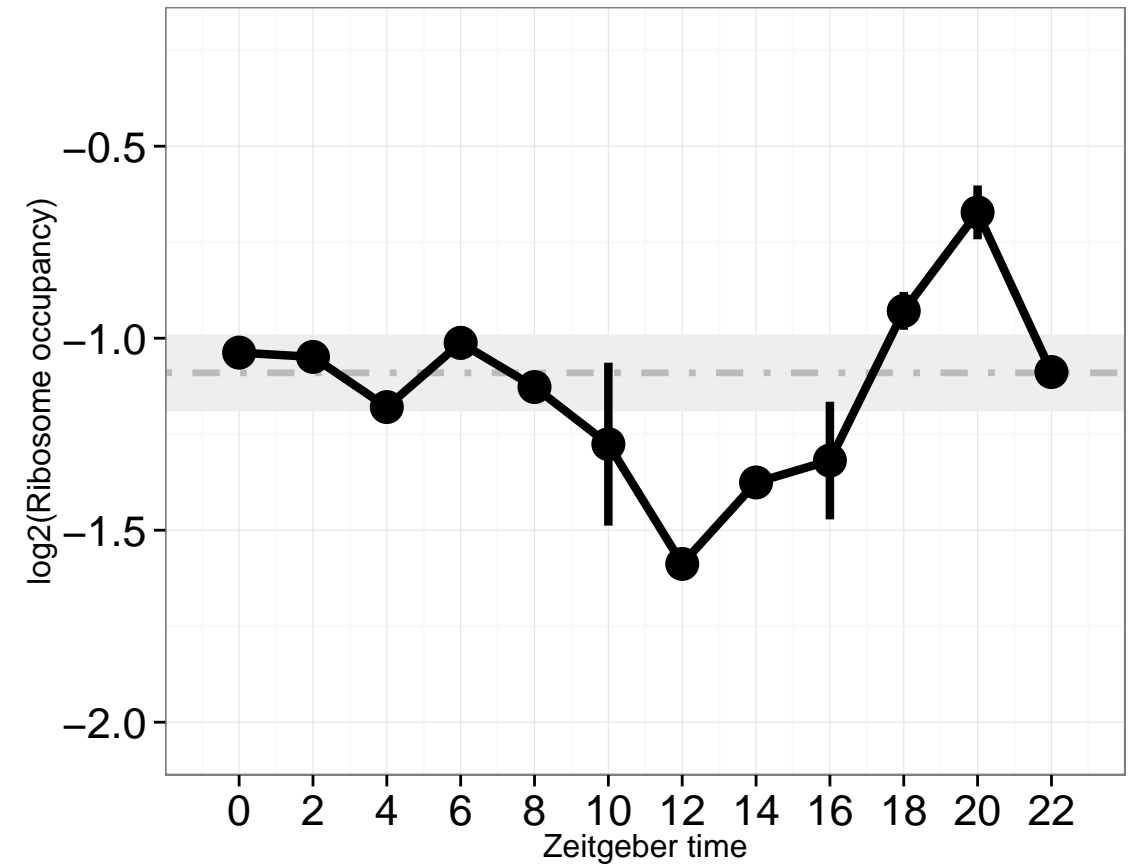

Supplement: Supplementary file 7 — Expression plots for kidney and liver for the 178 common rhythmic genes of Fig. 3c. (ZIP 3338.28 kb) [file 13059_2017_1222_MOESM7_ESM.zip › set_D_shared(178)/Ddx17_liver_set_D.pdf]

# Ddx60

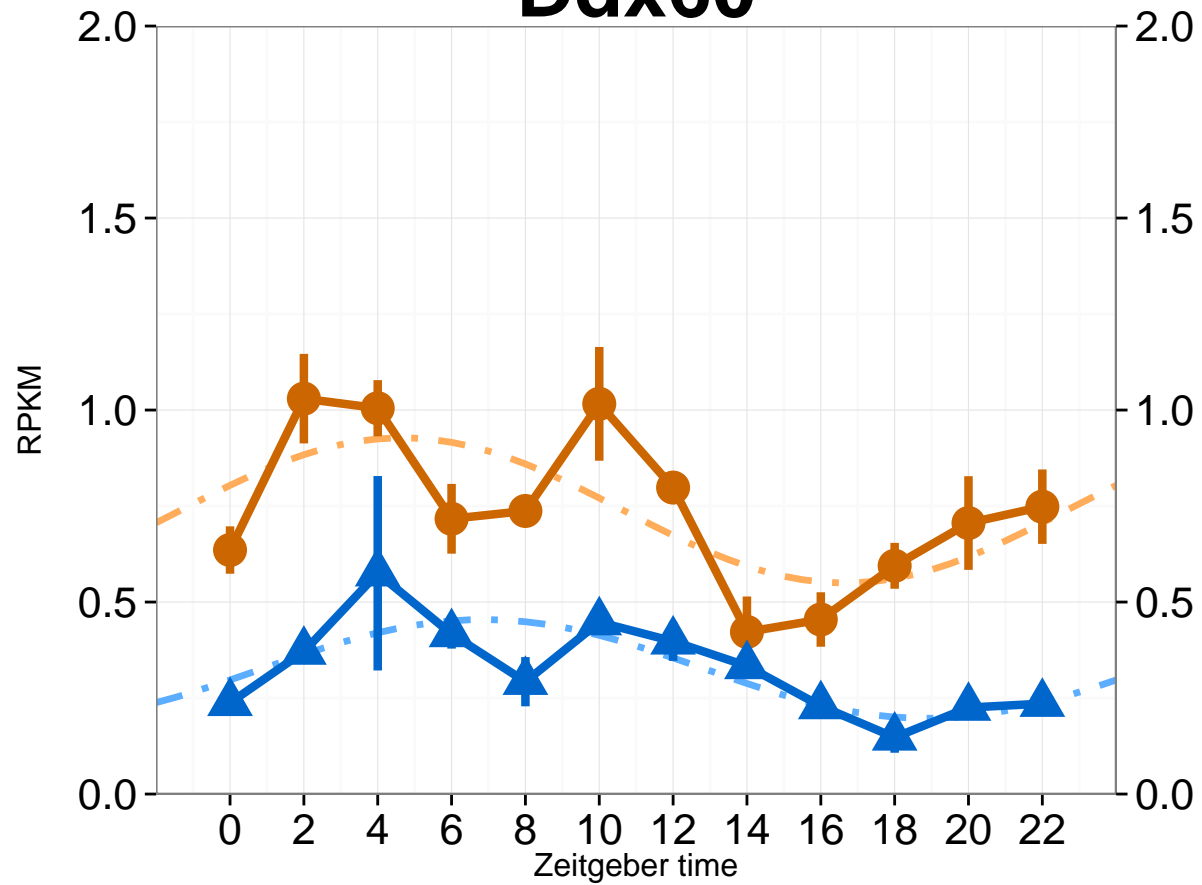

# Ddx60

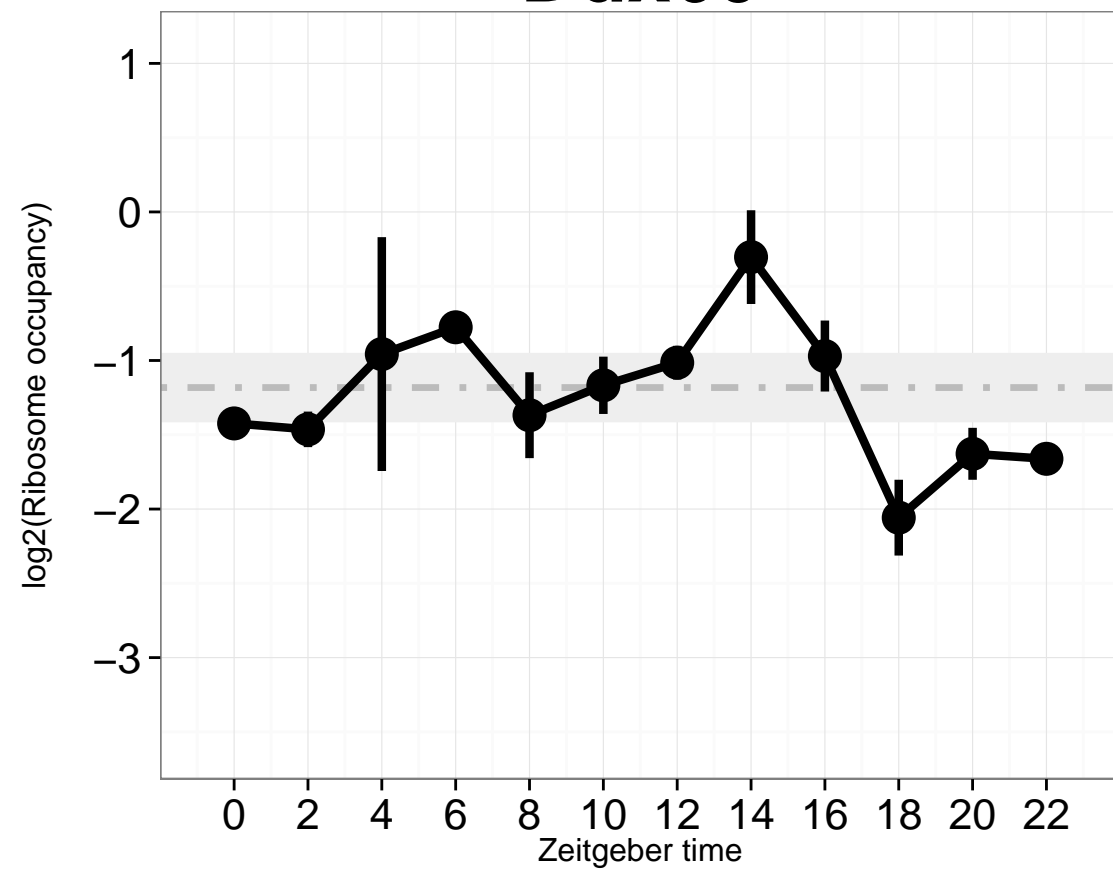

Supplement: Supplementary file 7 — Expression plots for kidney and liver for the 178 common rhythmic genes of Fig. 3c. (ZIP 3338.28 kb) [file 13059_2017_1222_MOESM7_ESM.zip › set_D_shared(178)/Ddx60_kidney_set_D.pdf]

# Ddx60

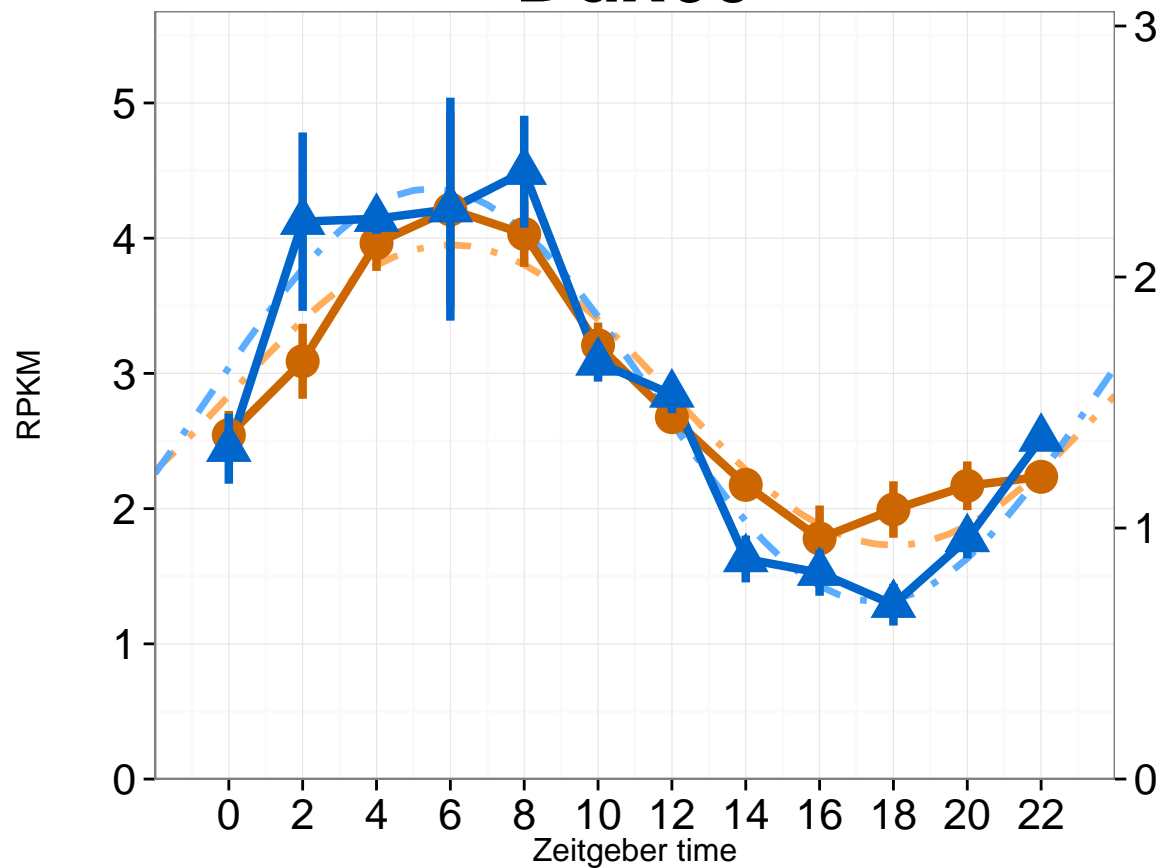

# Ddx60

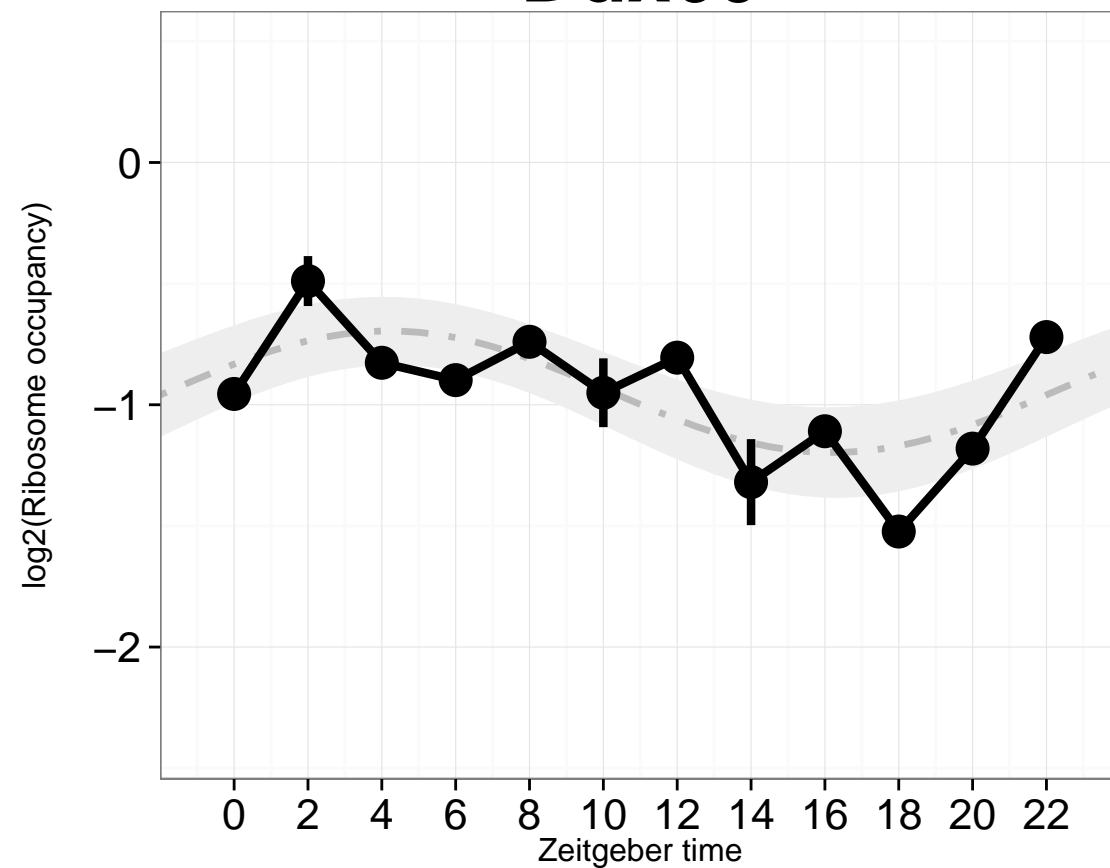

Supplement: Supplementary file 7 — Expression plots for kidney and liver for the 178 common rhythmic genes of Fig. 3c. (ZIP 3338.28 kb) [file 13059_2017_1222_MOESM7_ESM.zip › set_D_shared(178)/Ddx60_liver_set_D.pdf]

## Derl3

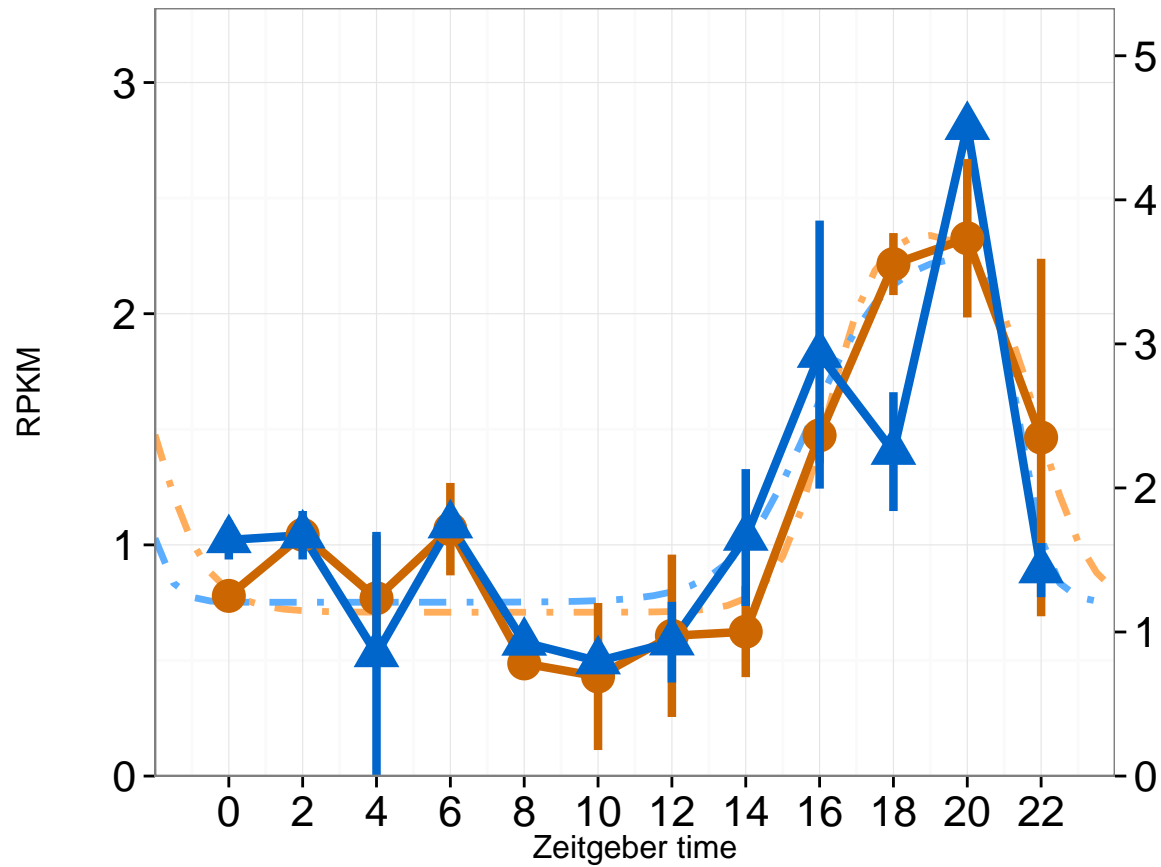

## Derl3

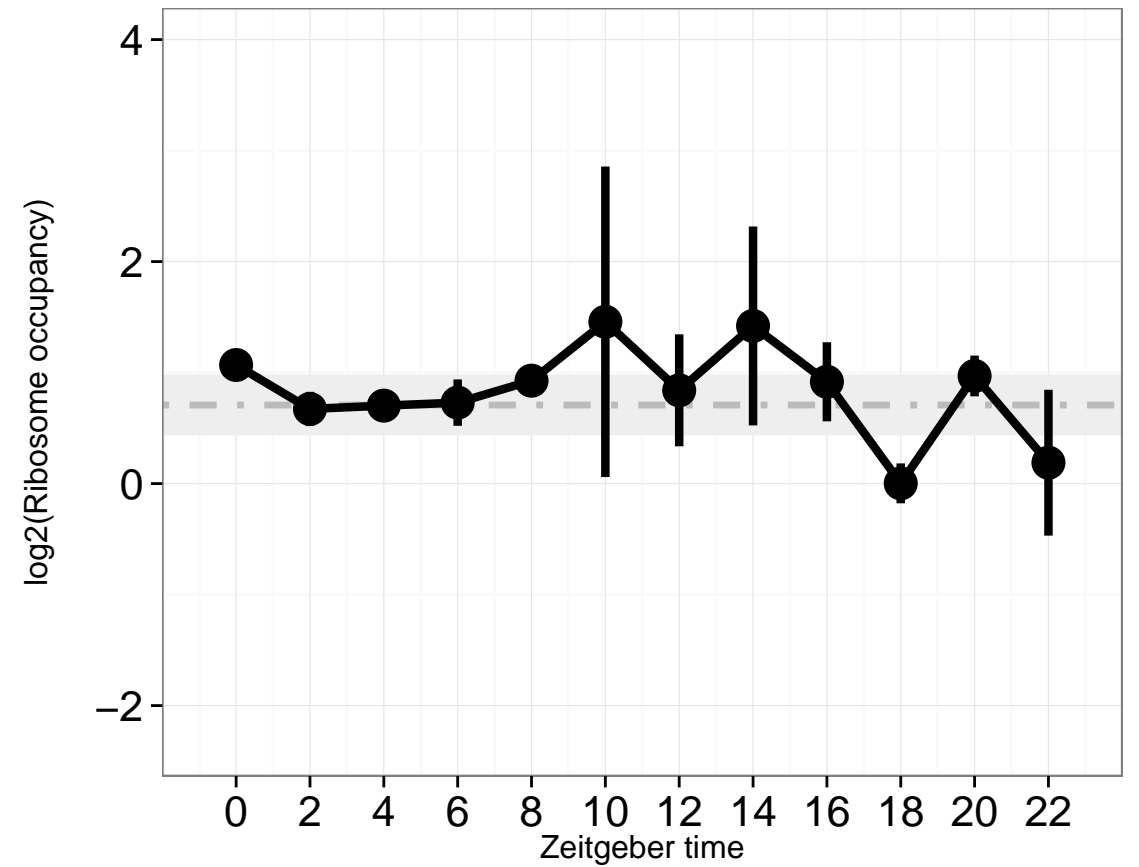

Supplement: Supplementary file 7 — Expression plots for kidney and liver for the 178 common rhythmic genes of Fig. 3c. (ZIP 3338.28 kb) [file 13059_2017_1222_MOESM7_ESM.zip › set_D_shared(178)/Derl3_kidney_set_D.pdf]

# Derl3

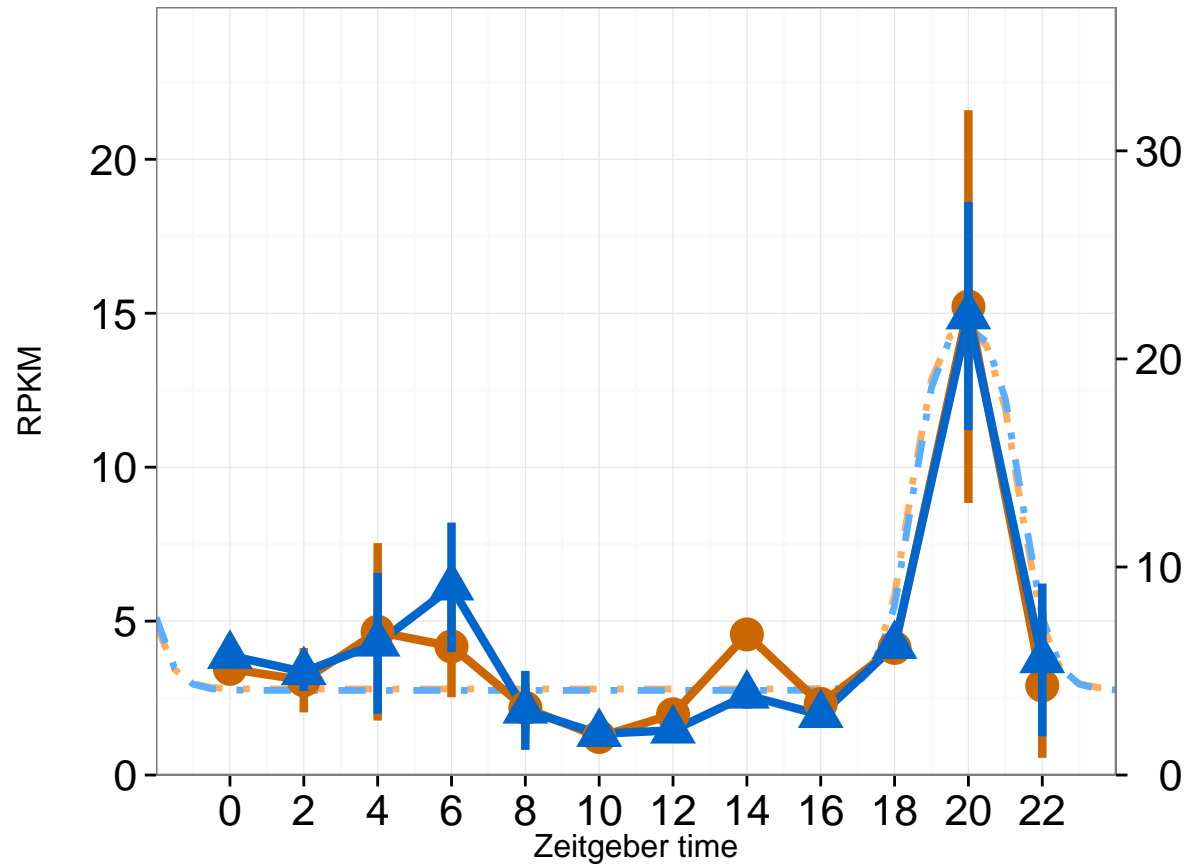

# Derl3

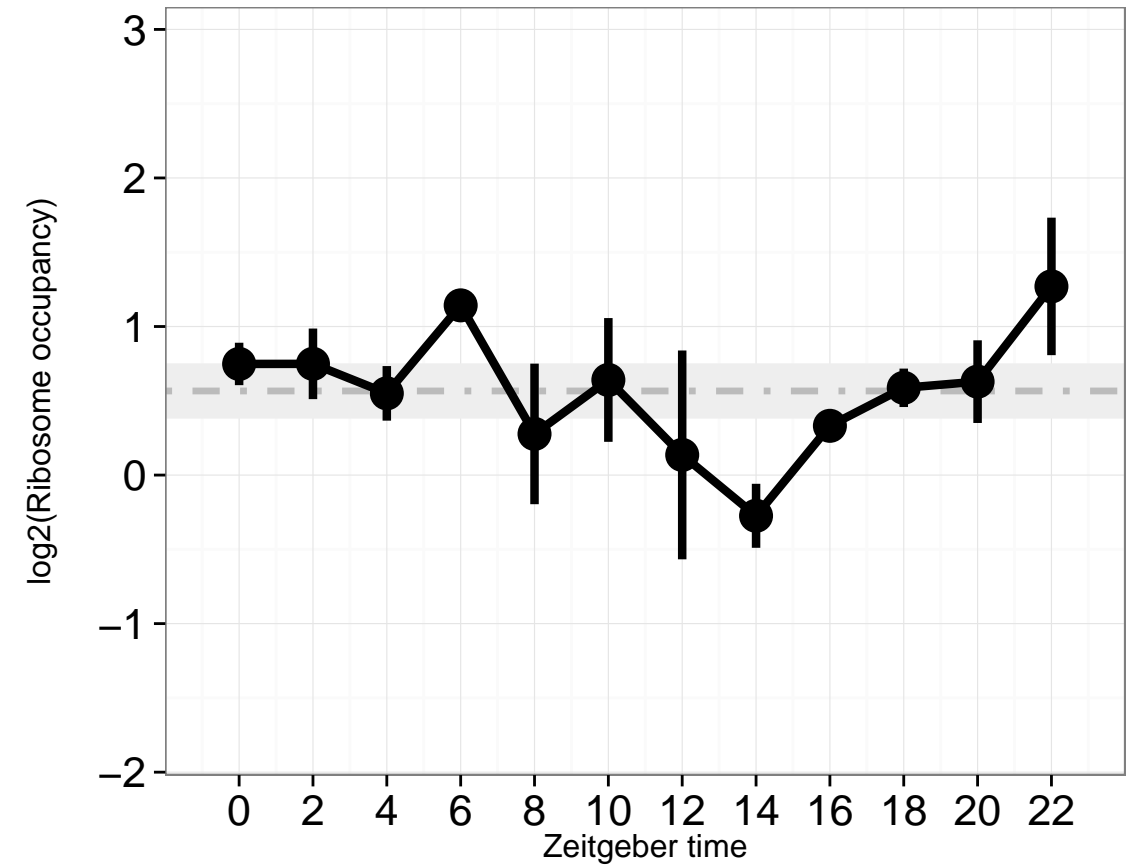

Supplement: Supplementary file 7 — Expression plots for kidney and liver for the 178 common rhythmic genes of Fig. 3c. (ZIP 3338.28 kb) [file 13059_2017_1222_MOESM7_ESM.zip › set_D_shared(178)/Derl3_liver_set_D.pdf]

# Dnaja1

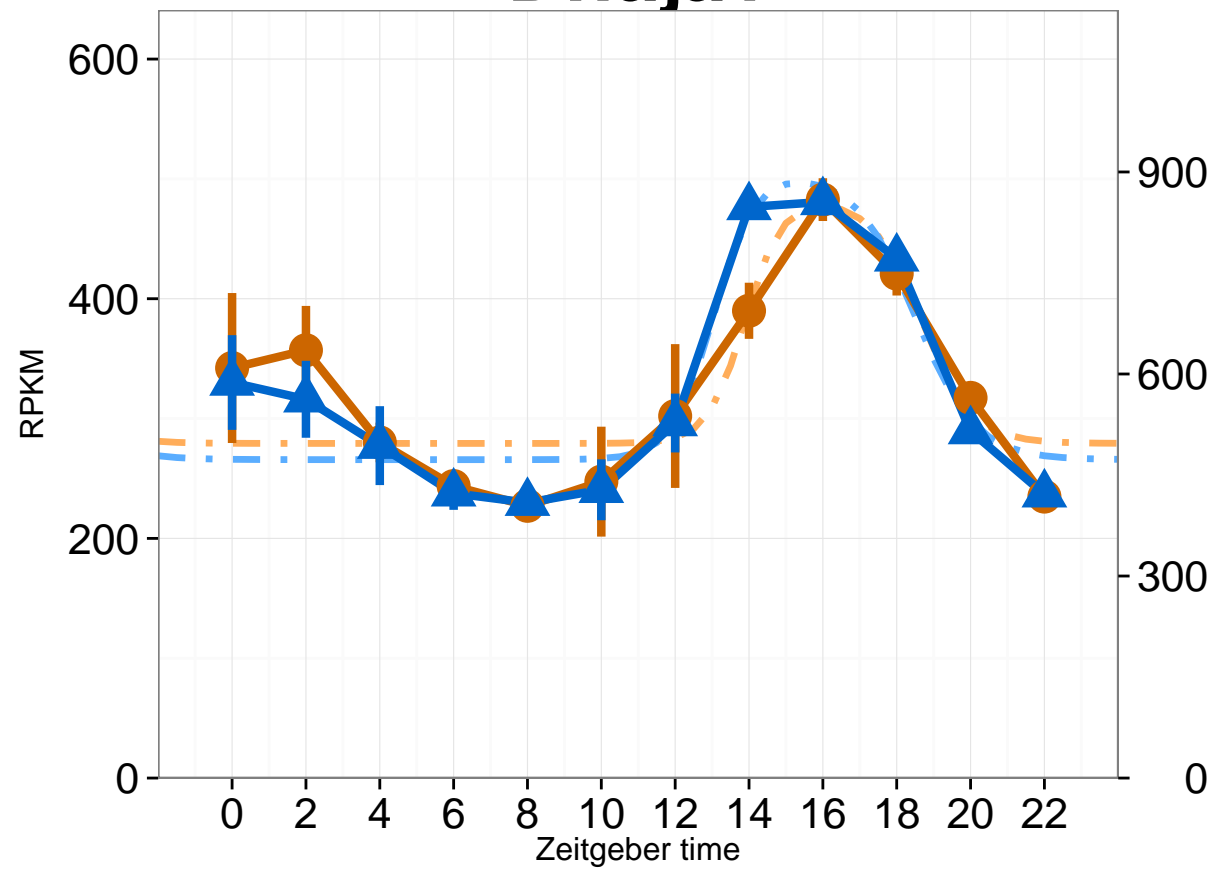

# Dnaja1

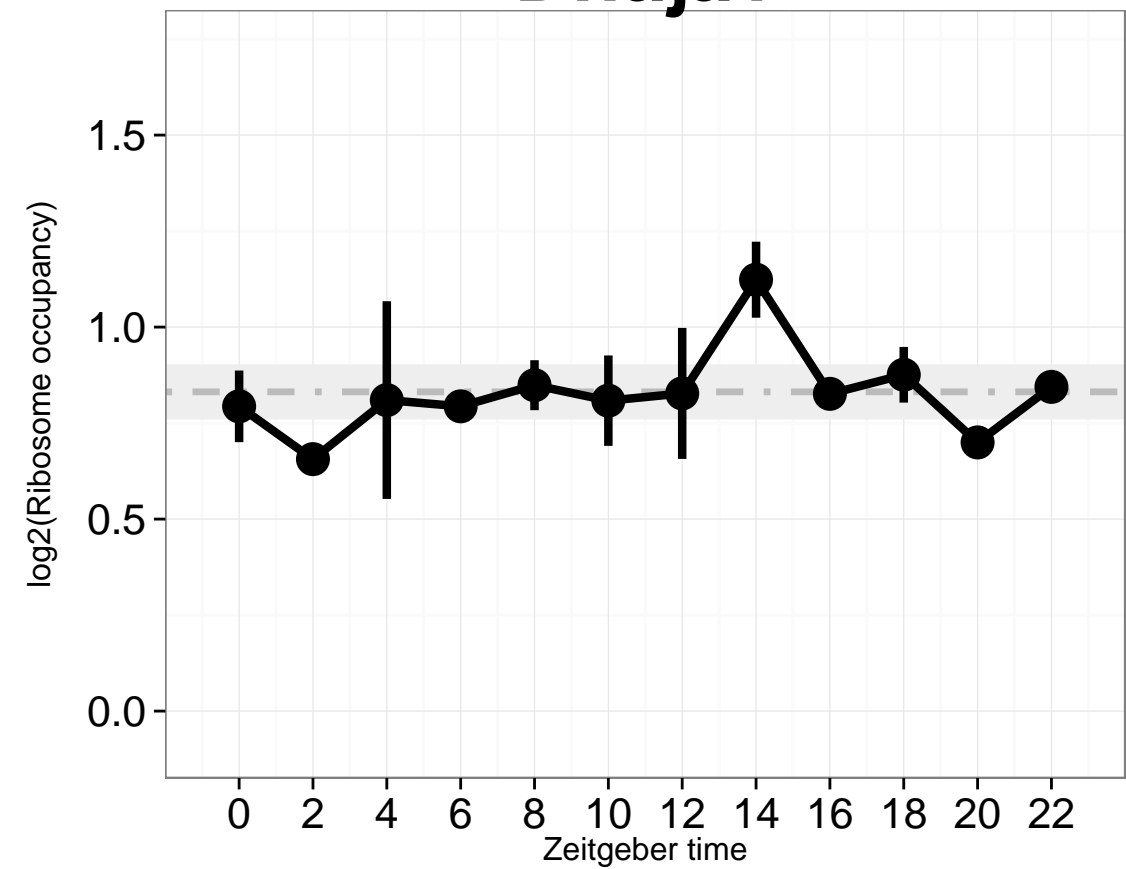

Supplement: Supplementary file 7 — Expression plots for kidney and liver for the 178 common rhythmic genes of Fig. 3c. (ZIP 3338.28 kb) [file 13059_2017_1222_MOESM7_ESM.zip › set_D_shared(178)/Dnaja1_kidney_set_D.pdf]

# Dnaja1

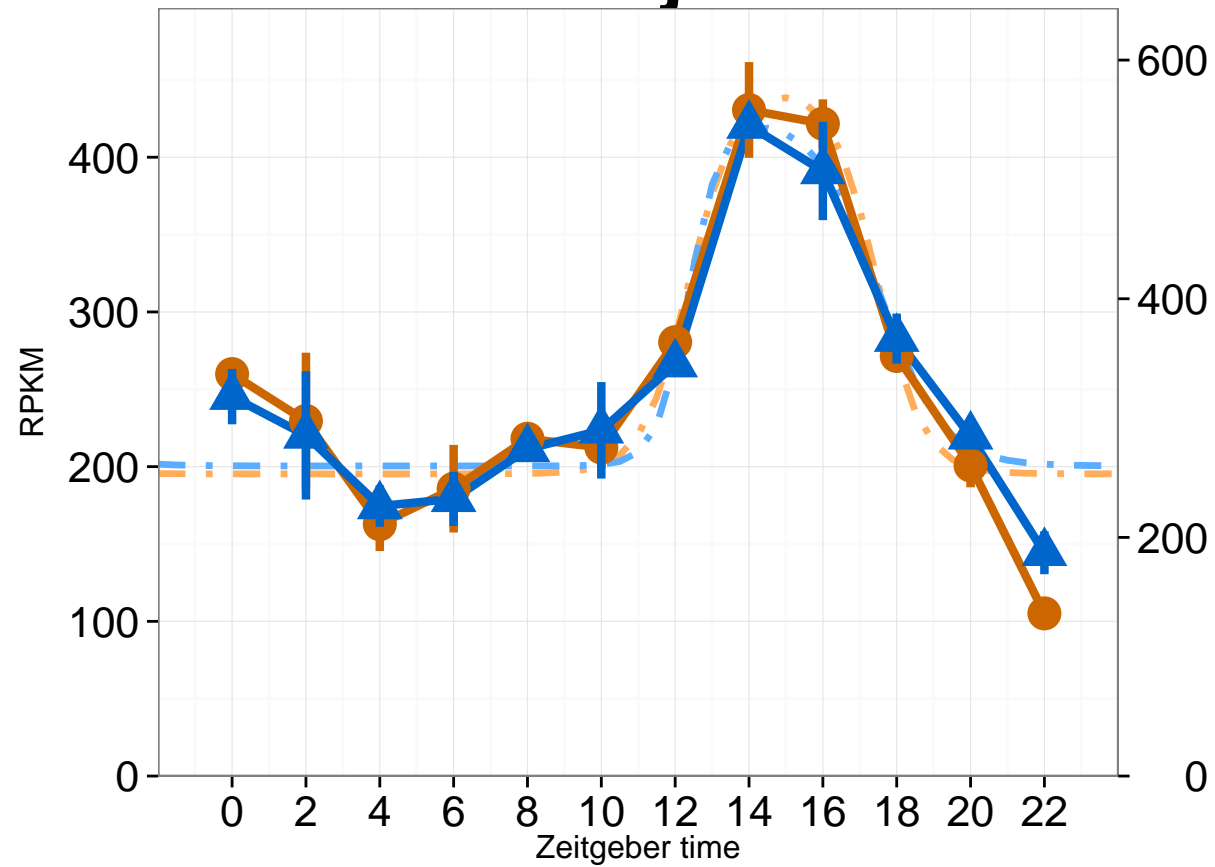

# Dnaja1

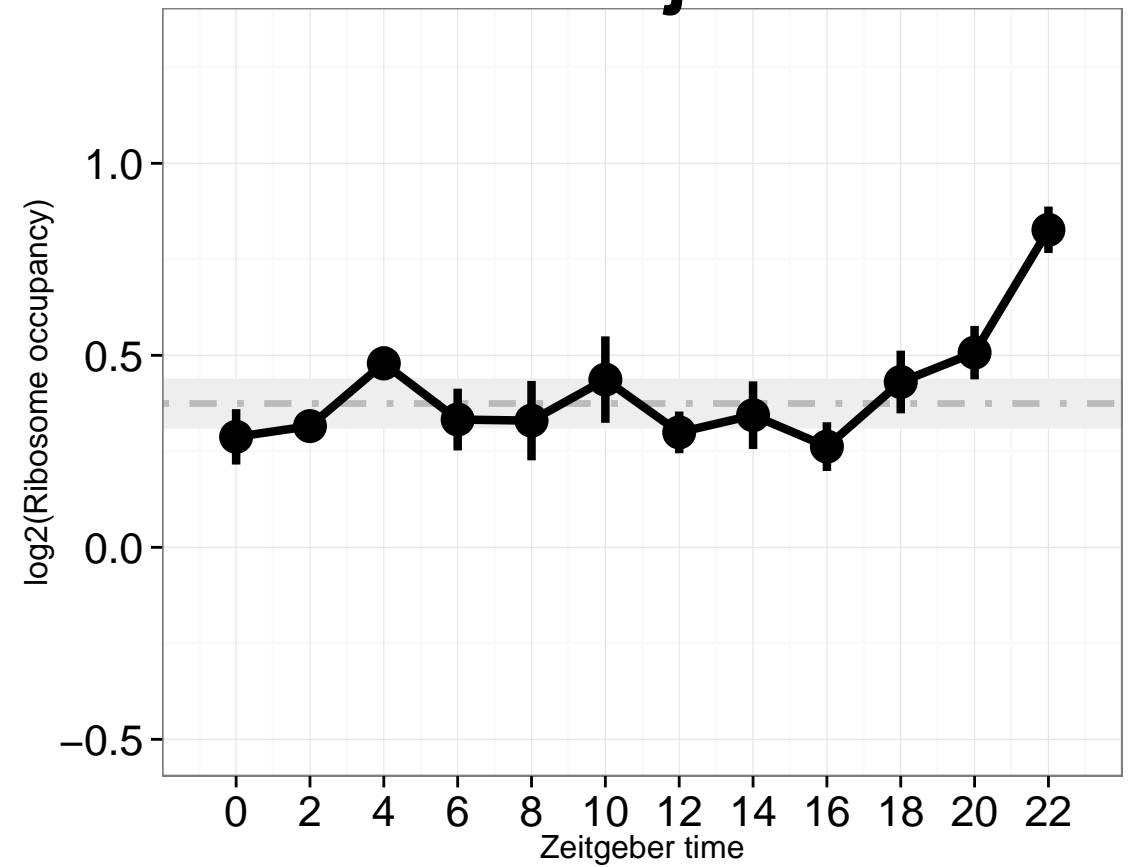

Supplement: Supplementary file 7 — Expression plots for kidney and liver for the 178 common rhythmic genes of Fig. 3c. (ZIP 3338.28 kb) [file 13059_2017_1222_MOESM7_ESM.zip › set_D_shared(178)/Dnaja1_liver_set_D.pdf]

## Dnajb1

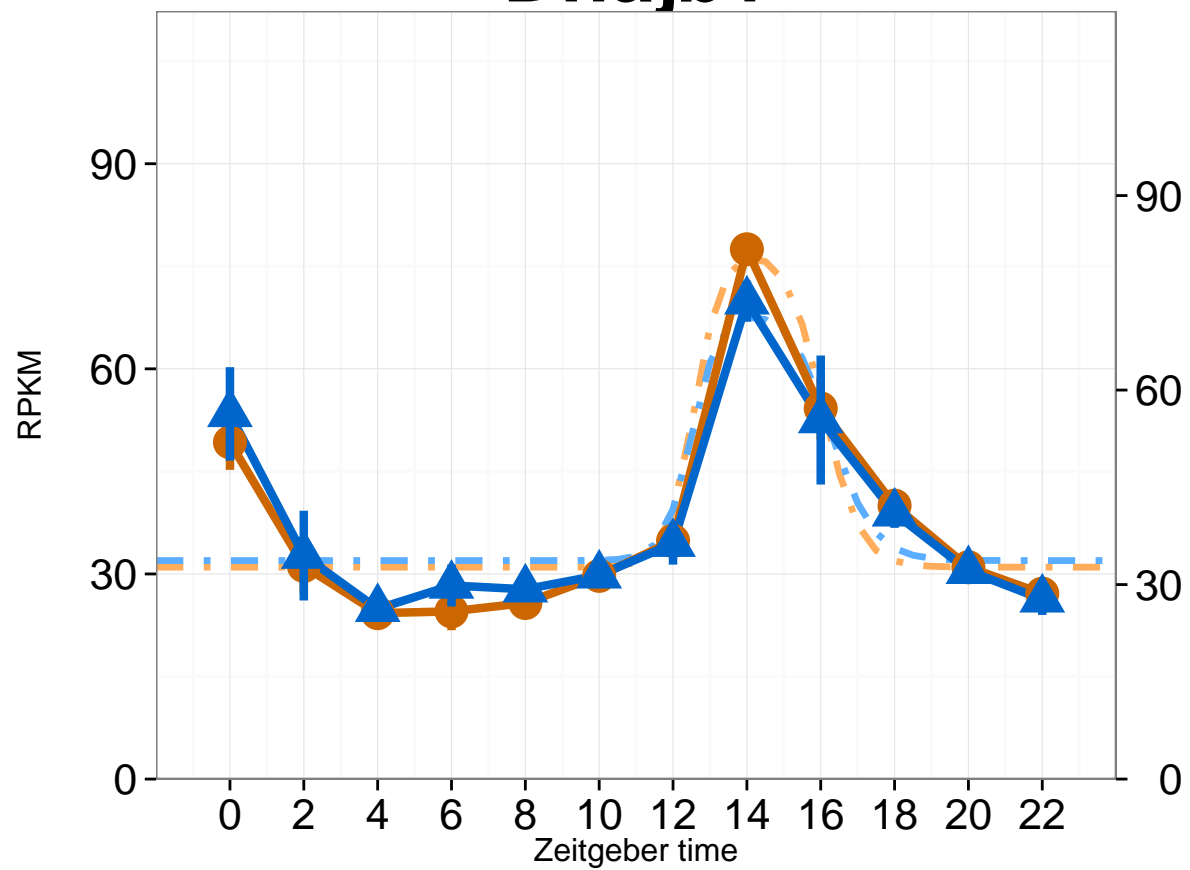

## Dnajb1

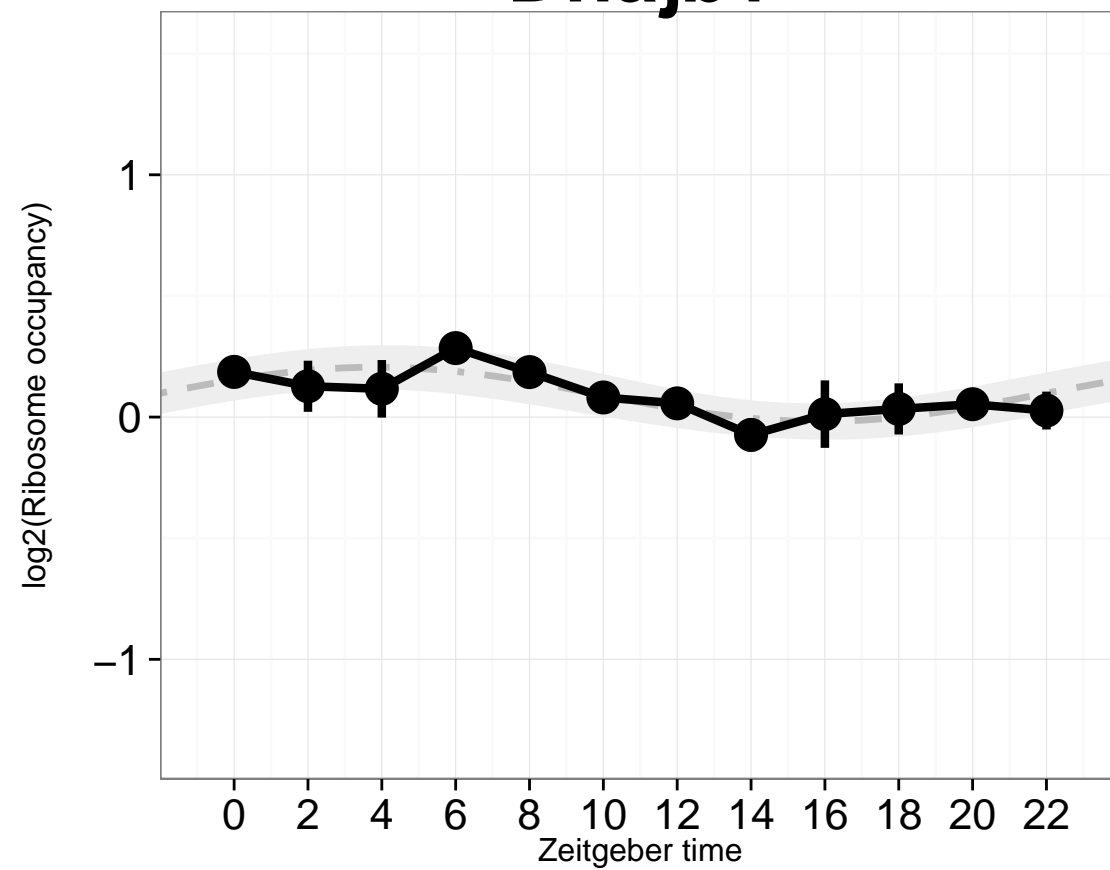

Supplement: Supplementary file 7 — Expression plots for kidney and liver for the 178 common rhythmic genes of Fig. 3c. (ZIP 3338.28 kb) [file 13059_2017_1222_MOESM7_ESM.zip › set_D_shared(178)/Dnajb1_kidney_set_D.pdf]

# Dnajb1

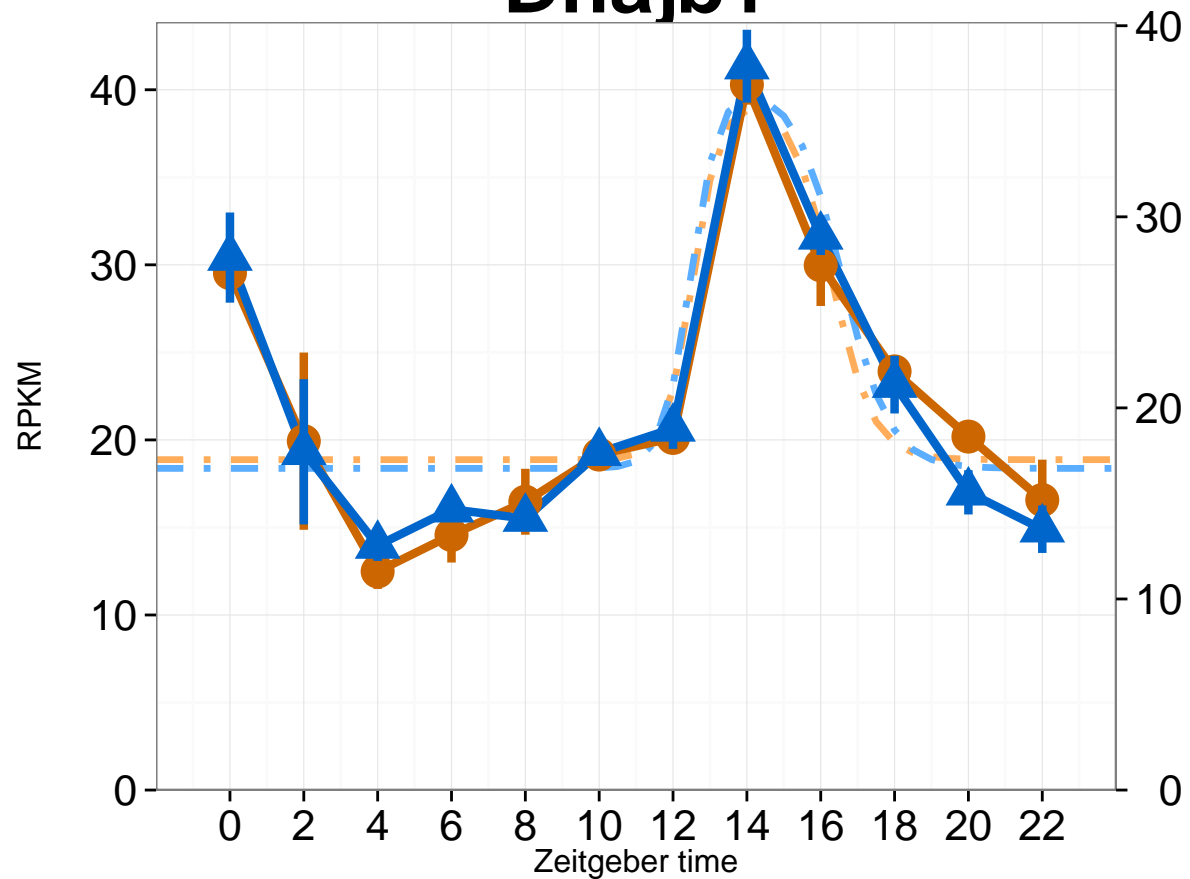

# Dnajb1

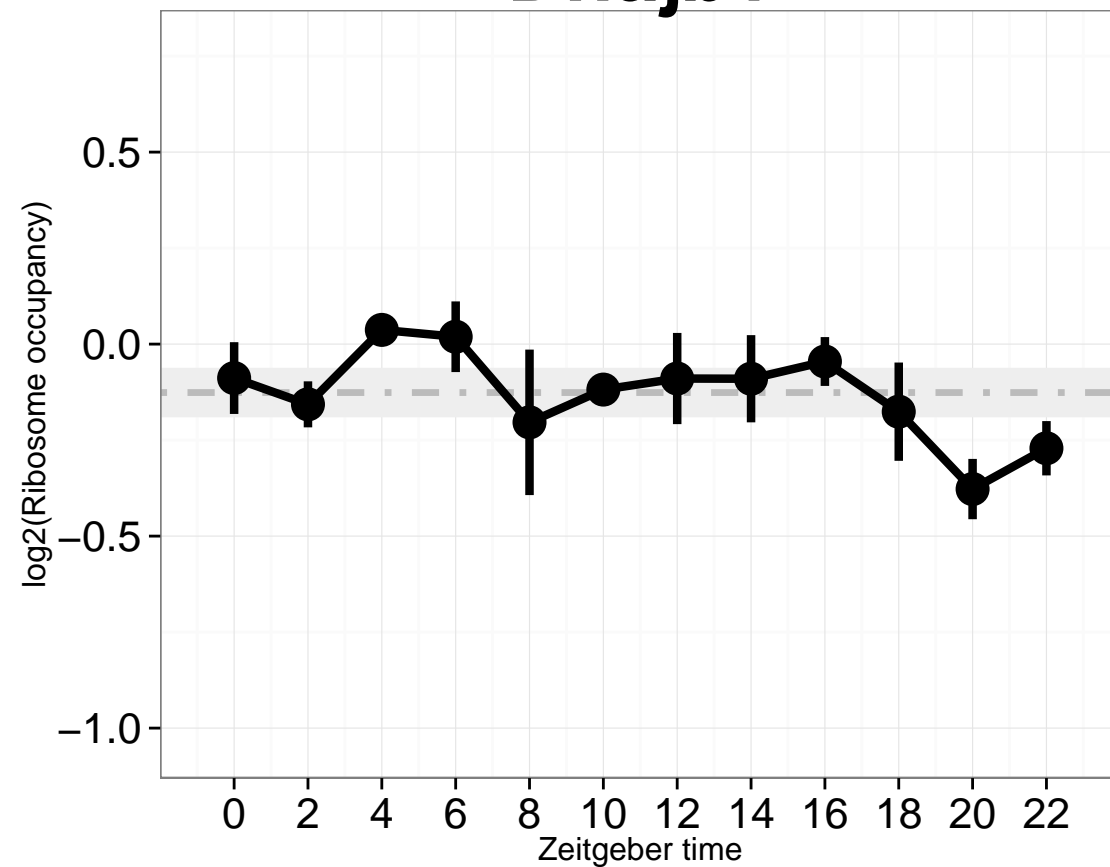

Supplement: Supplementary file 7 — Expression plots for kidney and liver for the 178 common rhythmic genes of Fig. 3c. (ZIP 3338.28 kb) [file 13059_2017_1222_MOESM7_ESM.zip › set_D_shared(178)/Dnajb1_liver_set_D.pdf]

# Dnajb4

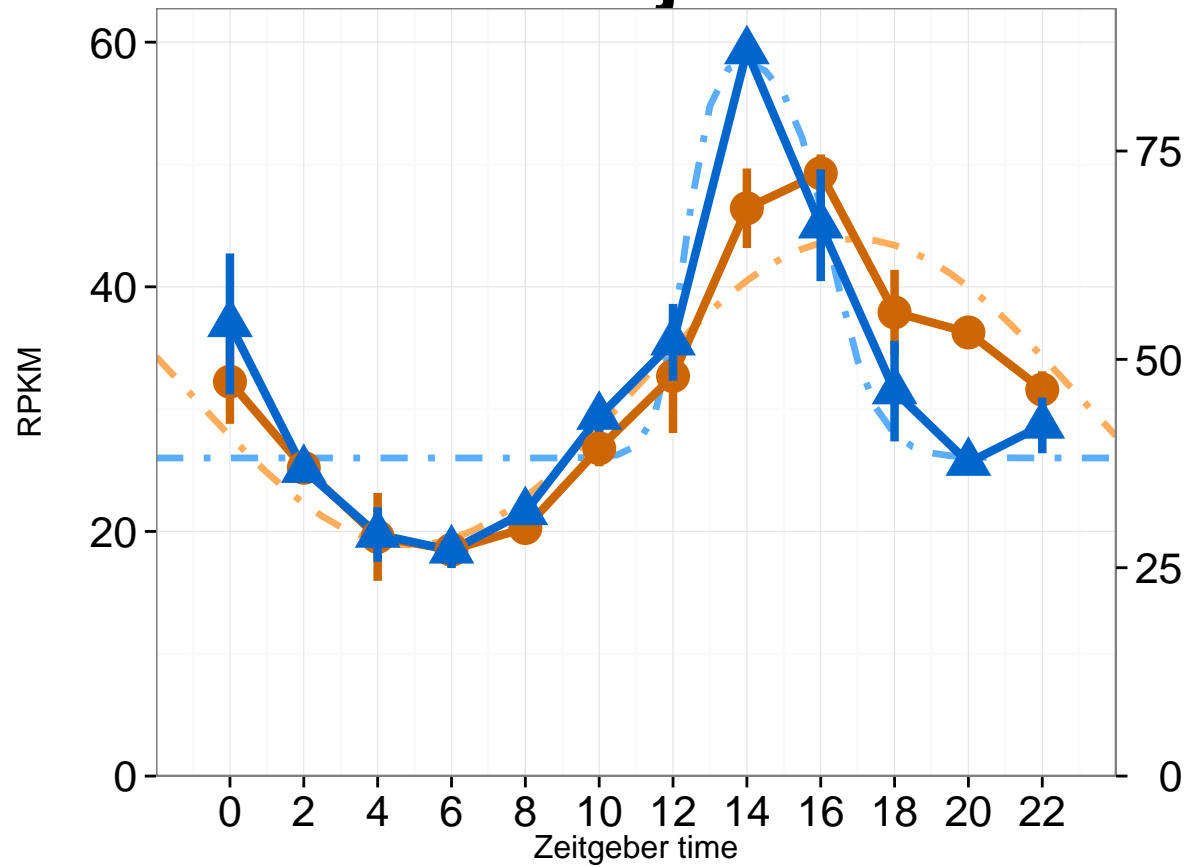

# Dnajb4

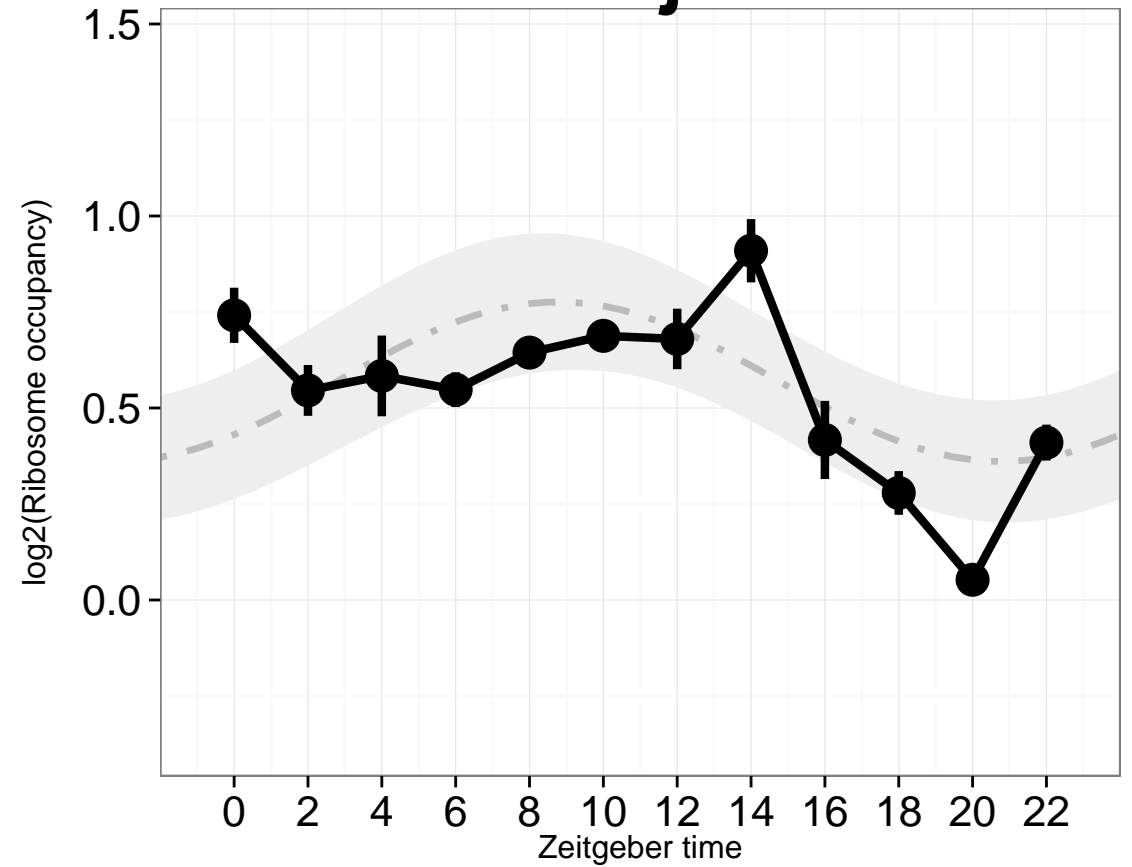

Supplement: Supplementary file 7 — Expression plots for kidney and liver for the 178 common rhythmic genes of Fig. 3c. (ZIP 3338.28 kb) [file 13059_2017_1222_MOESM7_ESM.zip › set_D_shared(178)/Dnajb4_kidney_set_D.pdf]

# Dnajb4

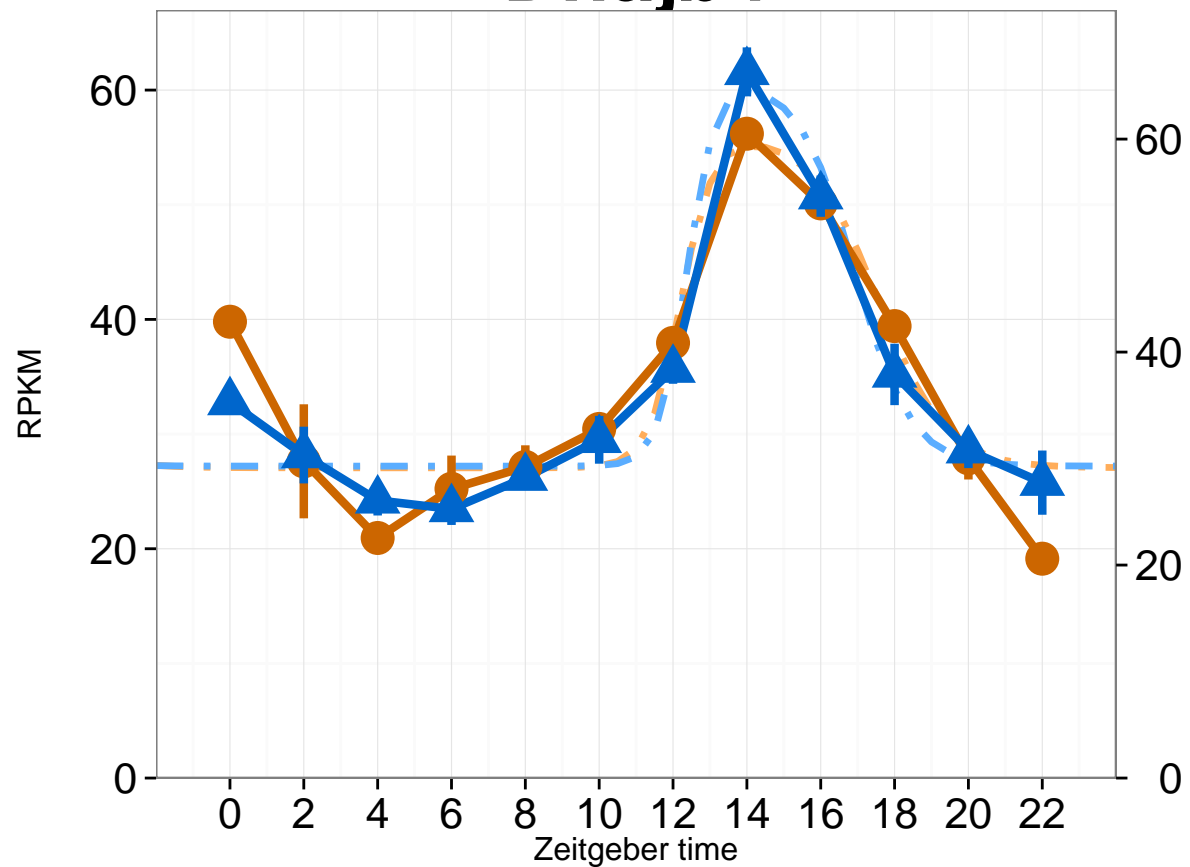

# Dnajb4

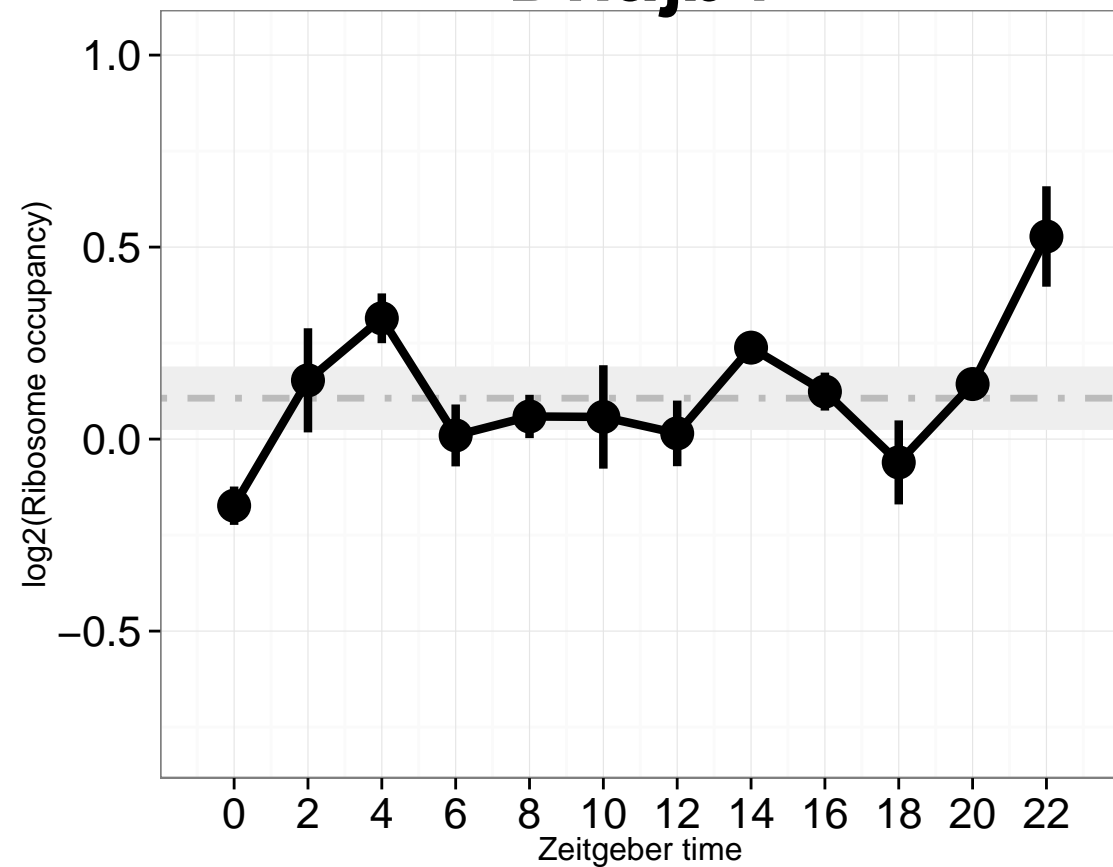

Supplement: Supplementary file 7 — Expression plots for kidney and liver for the 178 common rhythmic genes of Fig. 3c. (ZIP 3338.28 kb) [file 13059_2017_1222_MOESM7_ESM.zip › set_D_shared(178)/Dnajb4_liver_set_D.pdf]

# Dnajb9

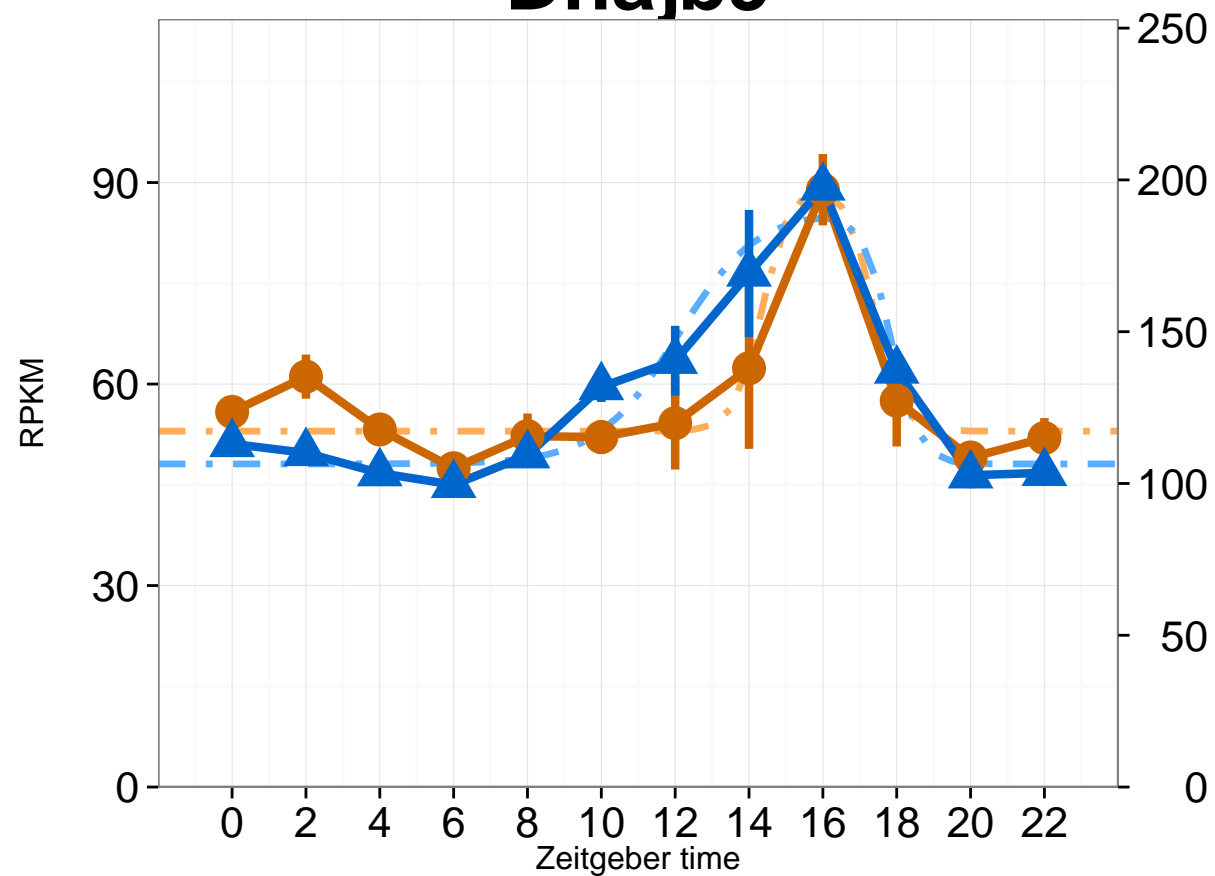

# Dnajb9

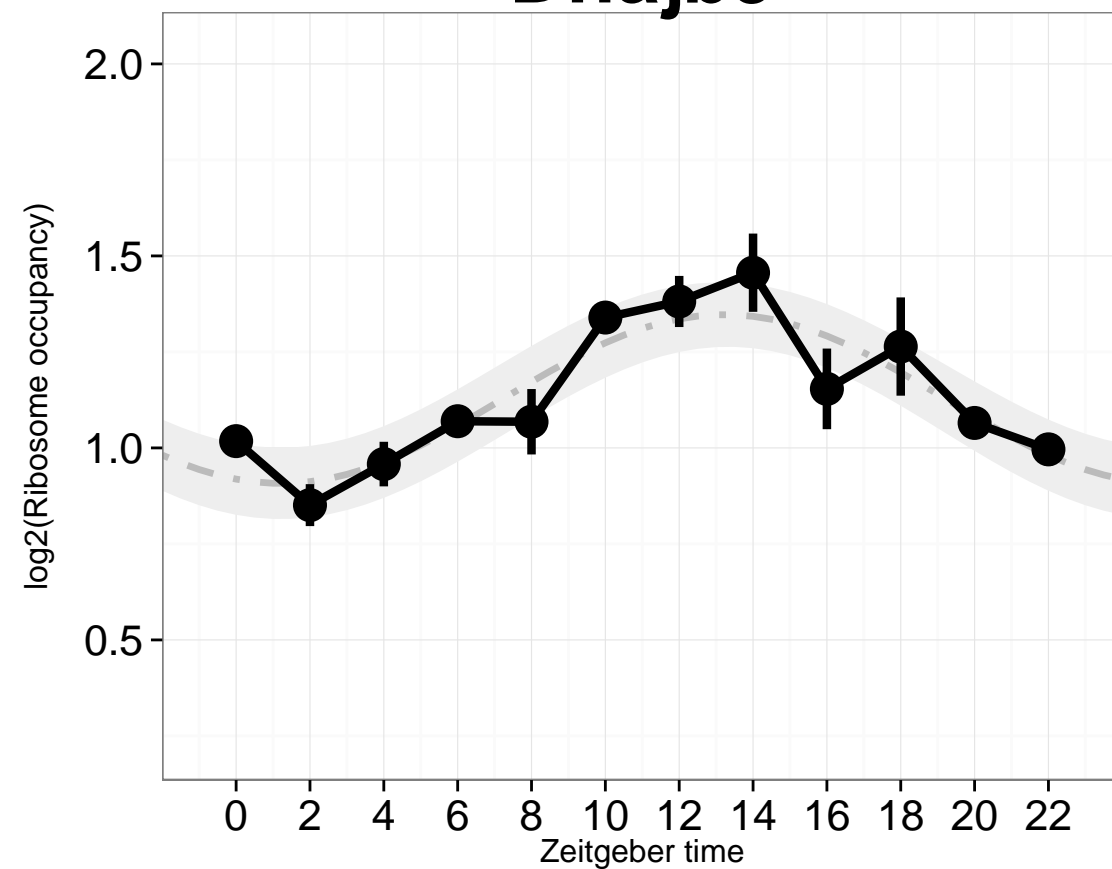

Supplement: Supplementary file 7 — Expression plots for kidney and liver for the 178 common rhythmic genes of Fig. 3c. (ZIP 3338.28 kb) [file 13059_2017_1222_MOESM7_ESM.zip › set_D_shared(178)/Dnajb9_kidney_set_D.pdf]

# Dnajb9

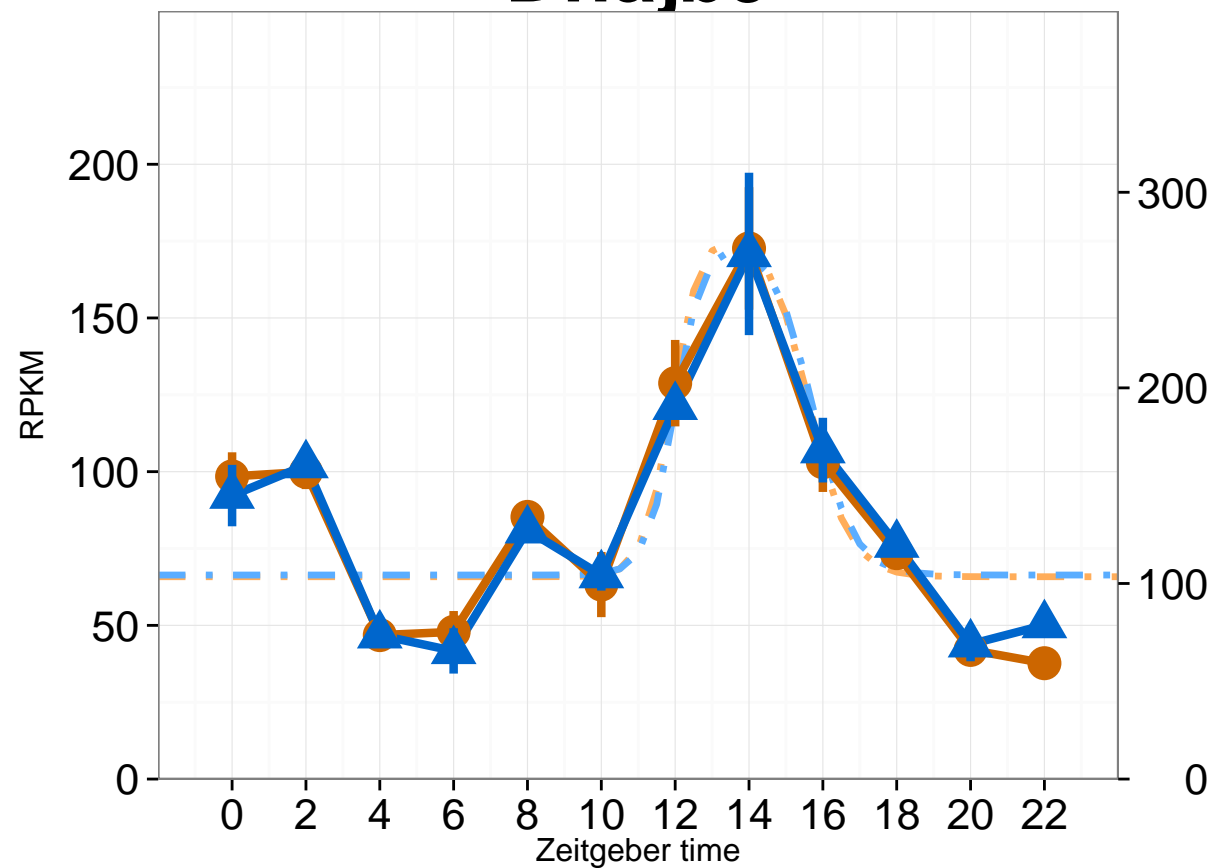

# Dnajb9

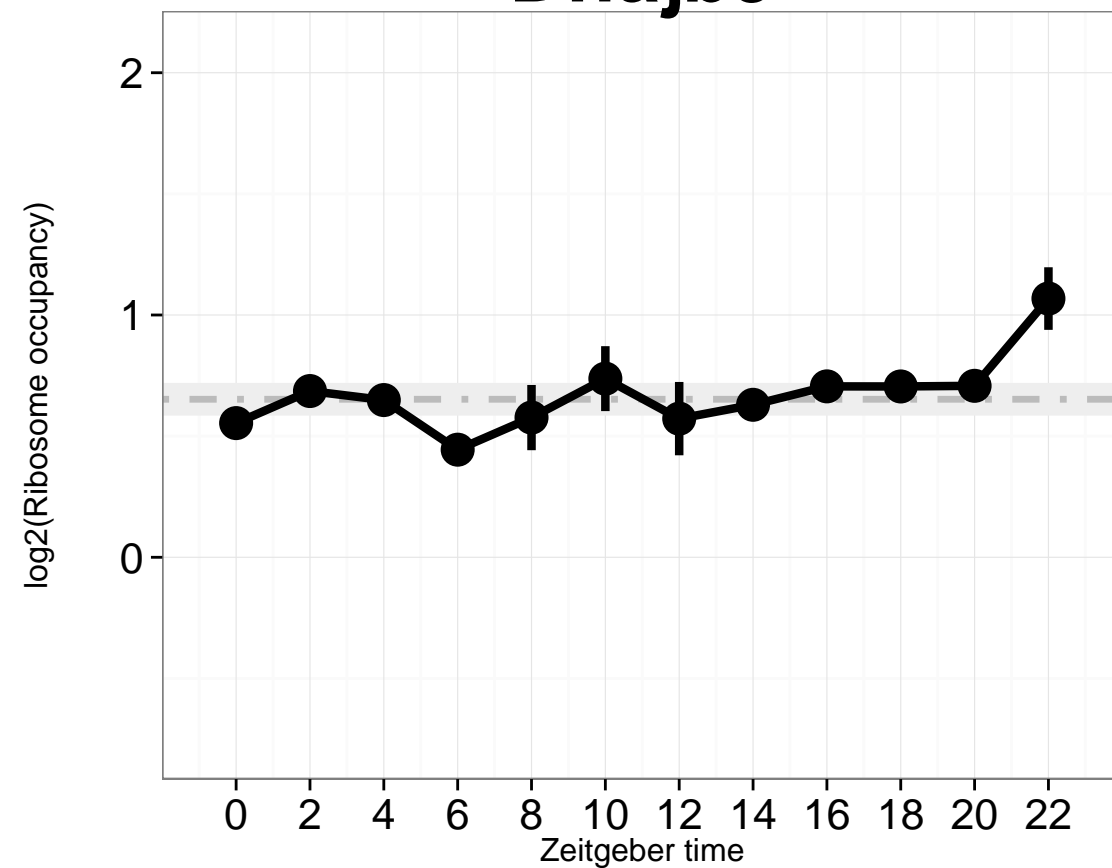

Supplement: Supplementary file 7 — Expression plots for kidney and liver for the 178 common rhythmic genes of Fig. 3c. (ZIP 3338.28 kb) [file 13059_2017_1222_MOESM7_ESM.zip › set_D_shared(178)/Dnajb9_liver_set_D.pdf]

# Dtx4

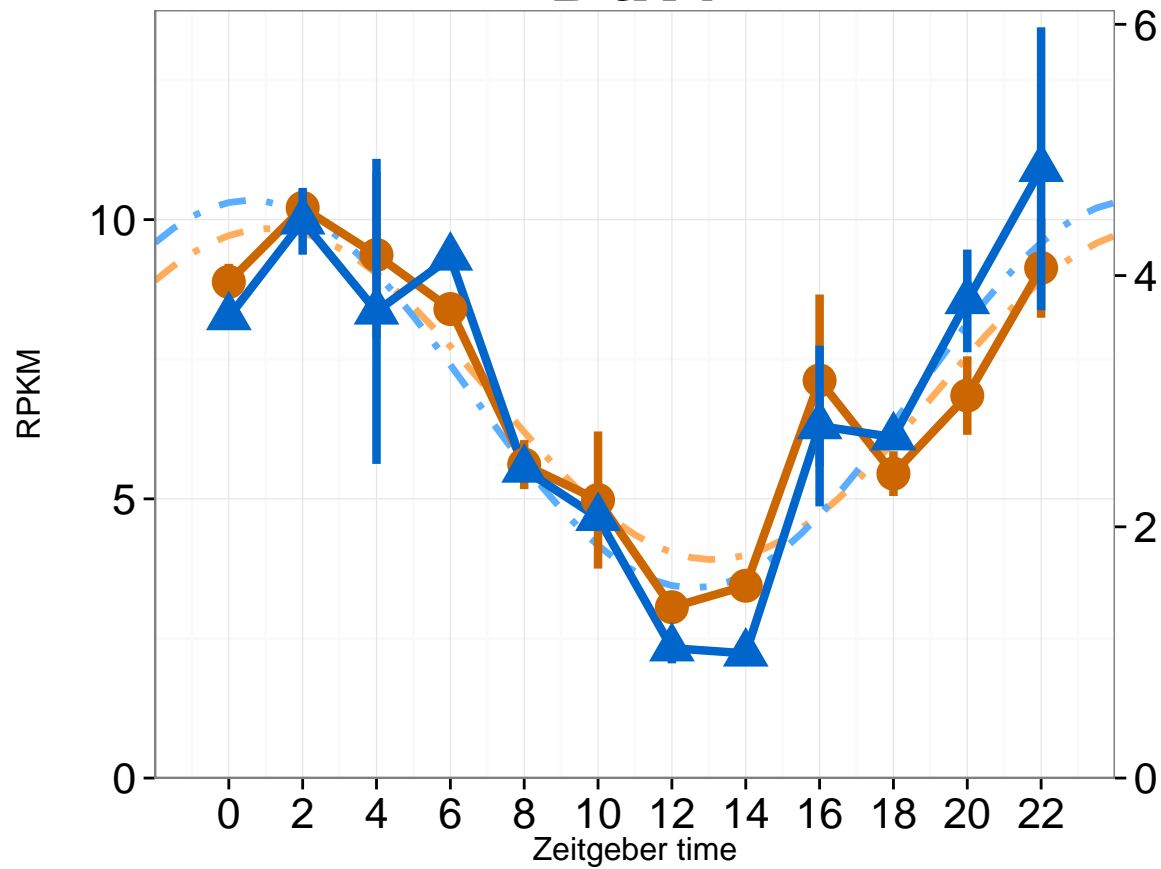

# Dtx4

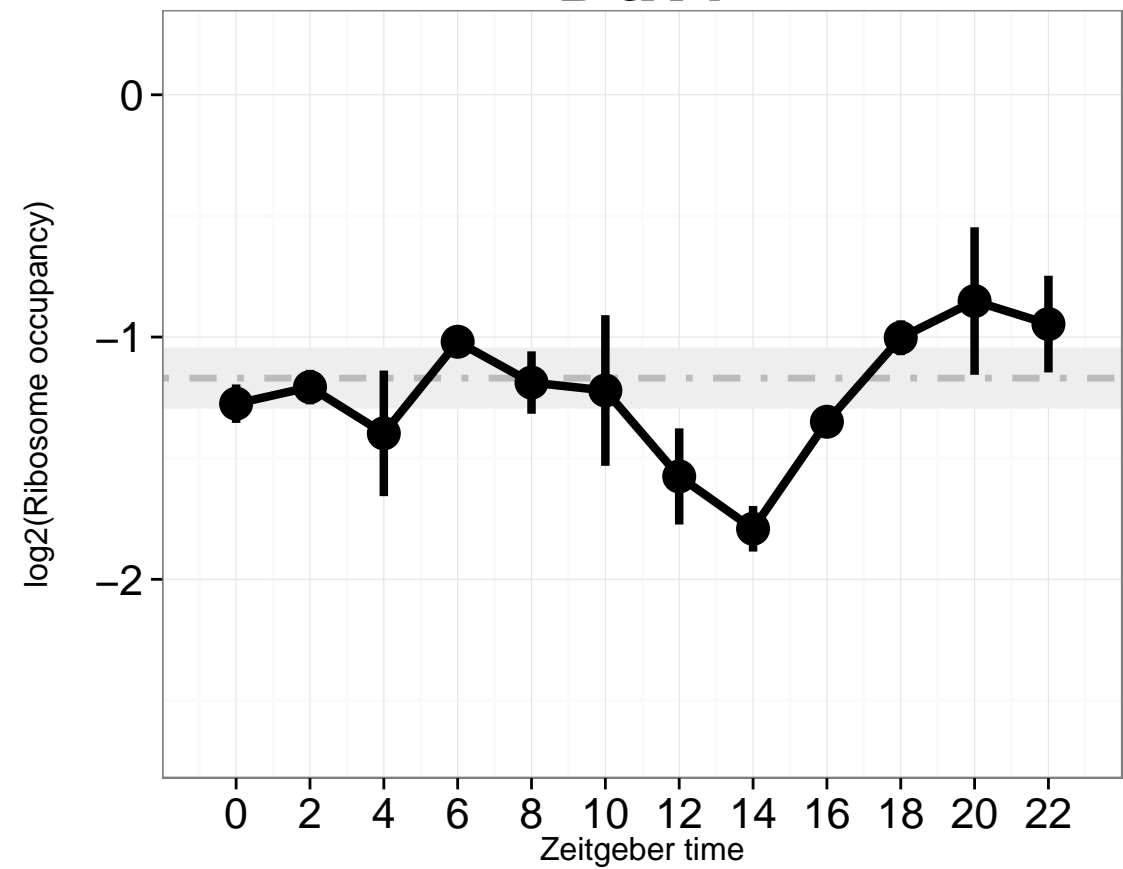

Supplement: Supplementary file 7 — Expression plots for kidney and liver for the 178 common rhythmic genes of Fig. 3c. (ZIP 3338.28 kb) [file 13059_2017_1222_MOESM7_ESM.zip › set_D_shared(178)/Dtx4_kidney_set_D.pdf]

# Dtx4

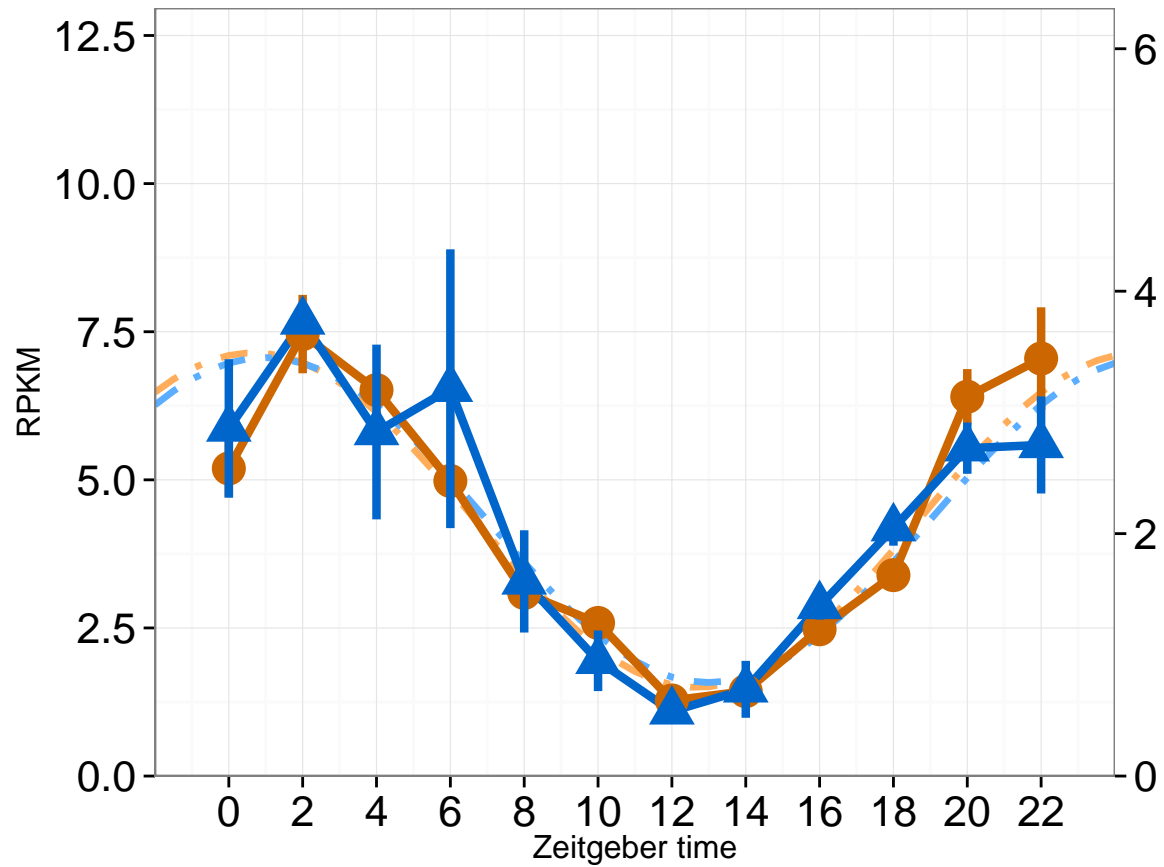

# Dtx4

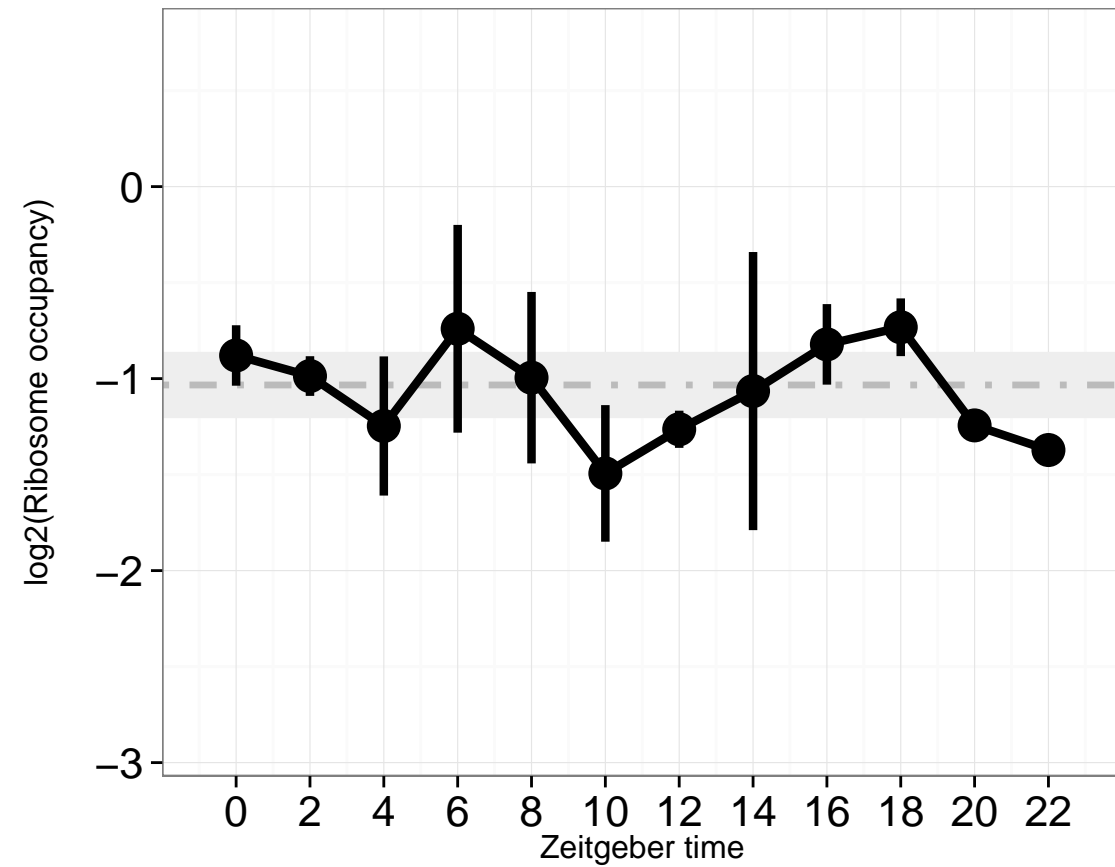

Supplement: Supplementary file 7 — Expression plots for kidney and liver for the 178 common rhythmic genes of Fig. 3c. (ZIP 3338.28 kb) [file 13059_2017_1222_MOESM7_ESM.zip › set_D_shared(178)/Dtx4_liver_set_D.pdf]

# Dyrk1b

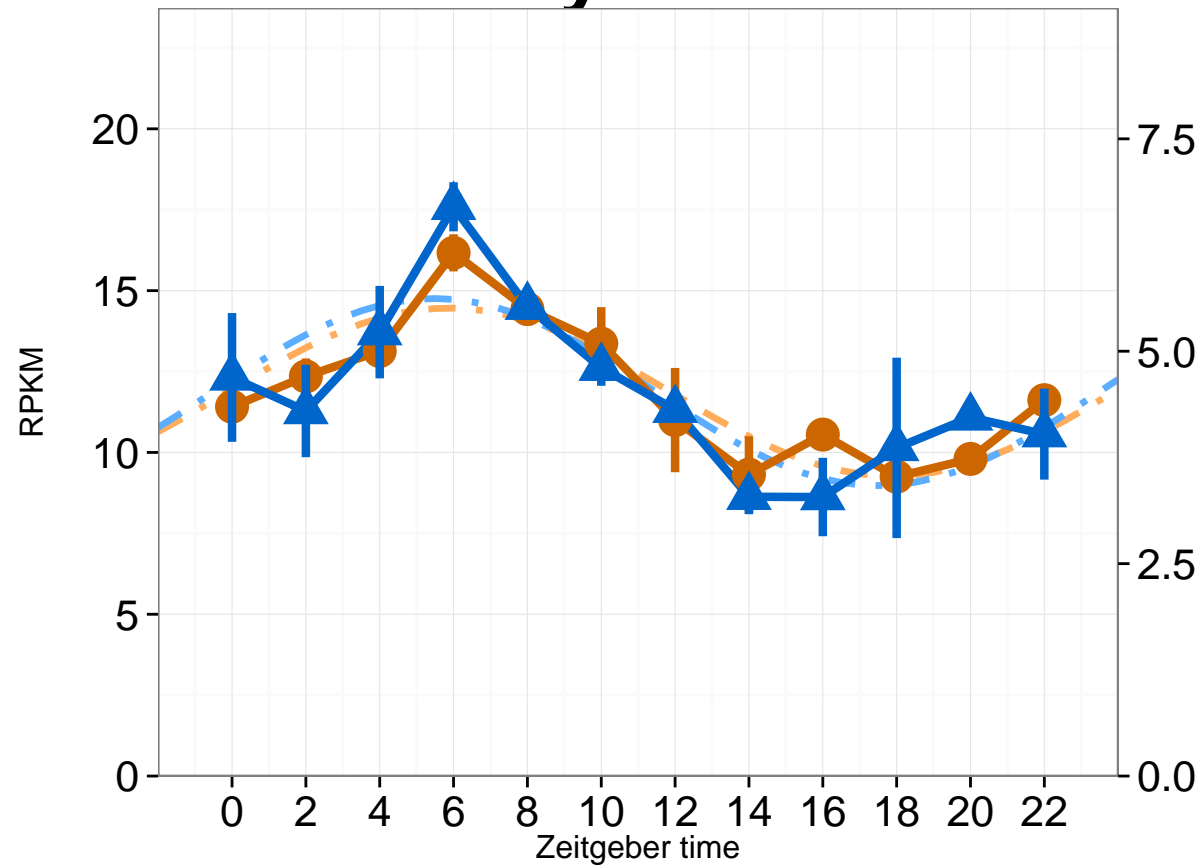

# Dyrk1b

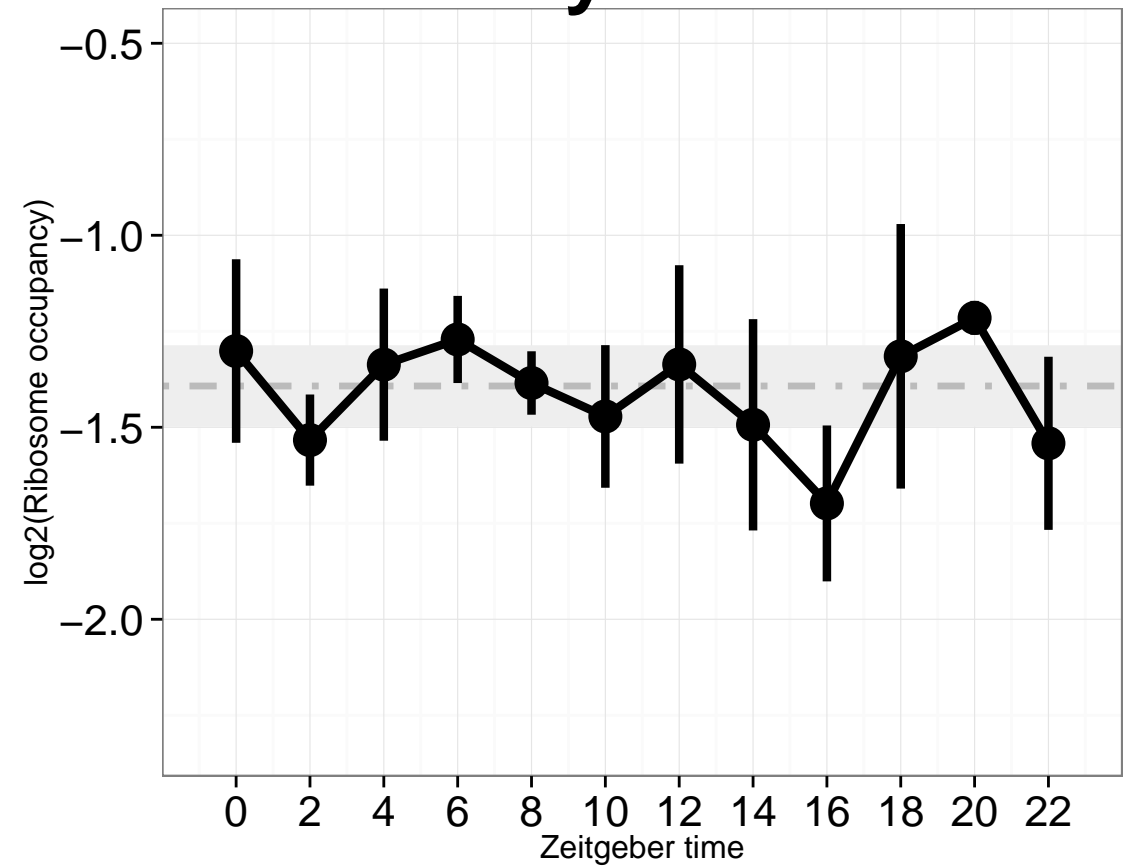

Supplement: Supplementary file 7 — Expression plots for kidney and liver for the 178 common rhythmic genes of Fig. 3c. (ZIP 3338.28 kb) [file 13059_2017_1222_MOESM7_ESM.zip › set_D_shared(178)/Dyrk1b_kidney_set_D.pdf]

# Dyrk1b

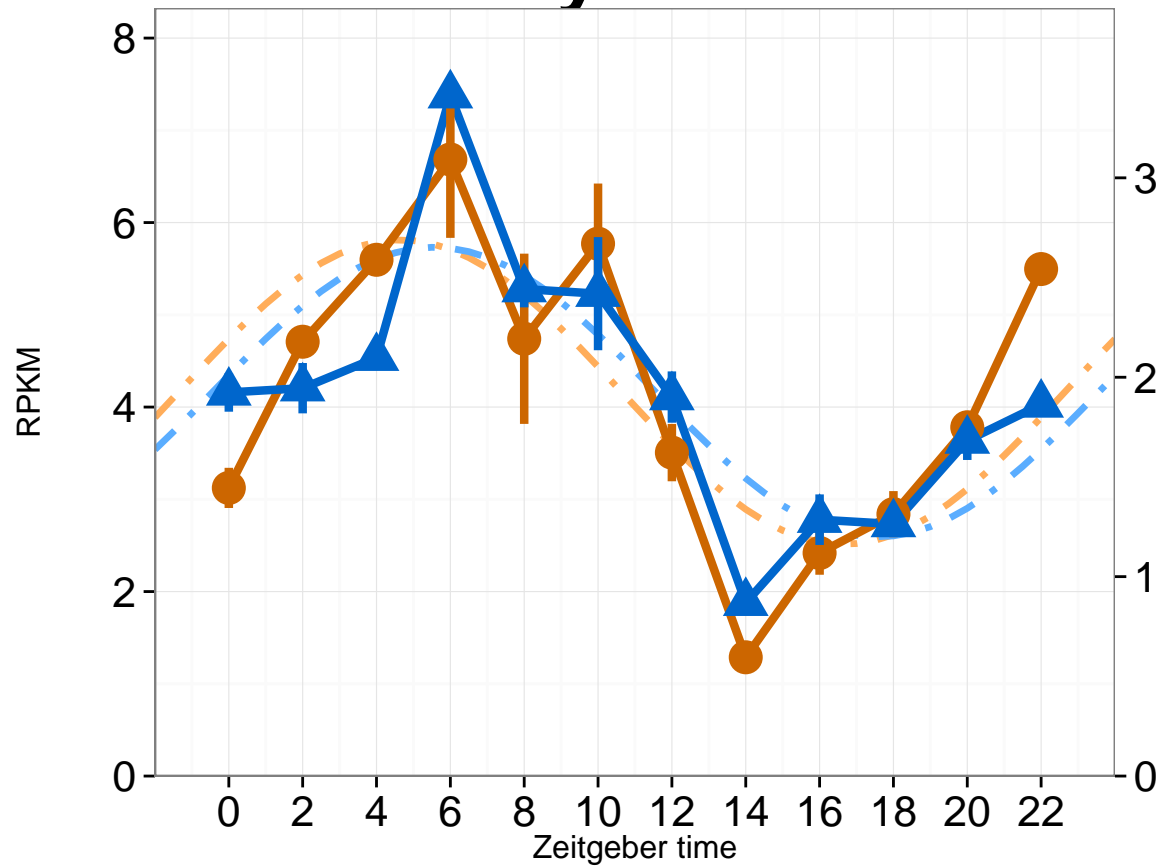

# Dyrk1b

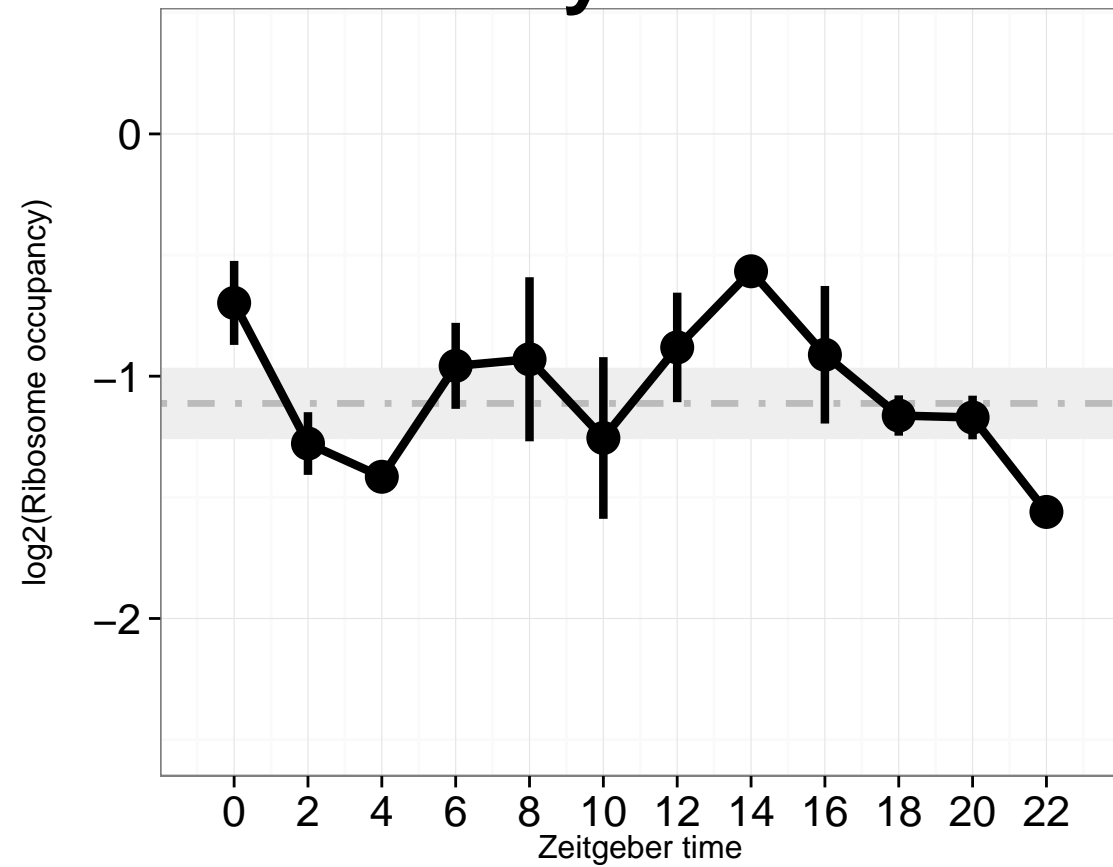

Supplement: Supplementary file 7 — Expression plots for kidney and liver for the 178 common rhythmic genes of Fig. 3c. (ZIP 3338.28 kb) [file 13059_2017_1222_MOESM7_ESM.zip › set_D_shared(178)/Dyrk1b_liver_set_D.pdf]

## Enpp2

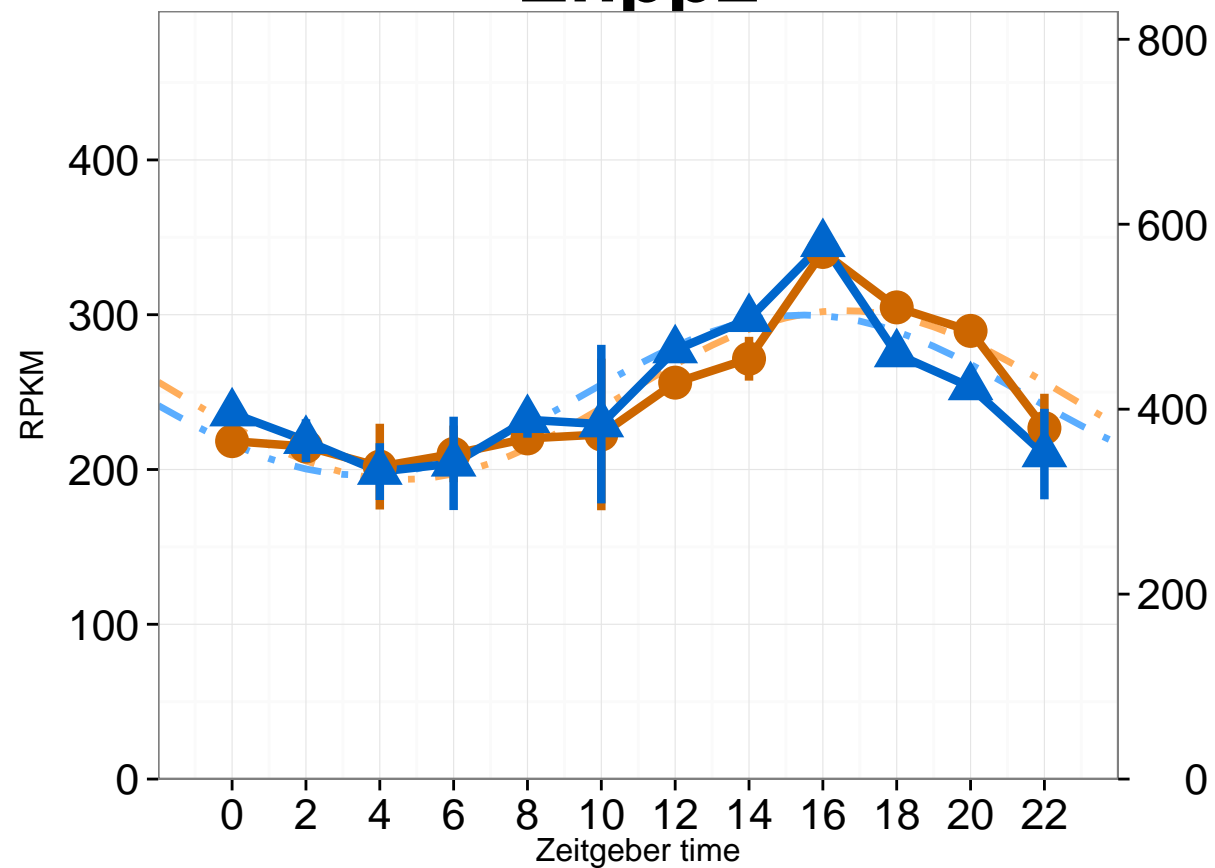

## Enpp2

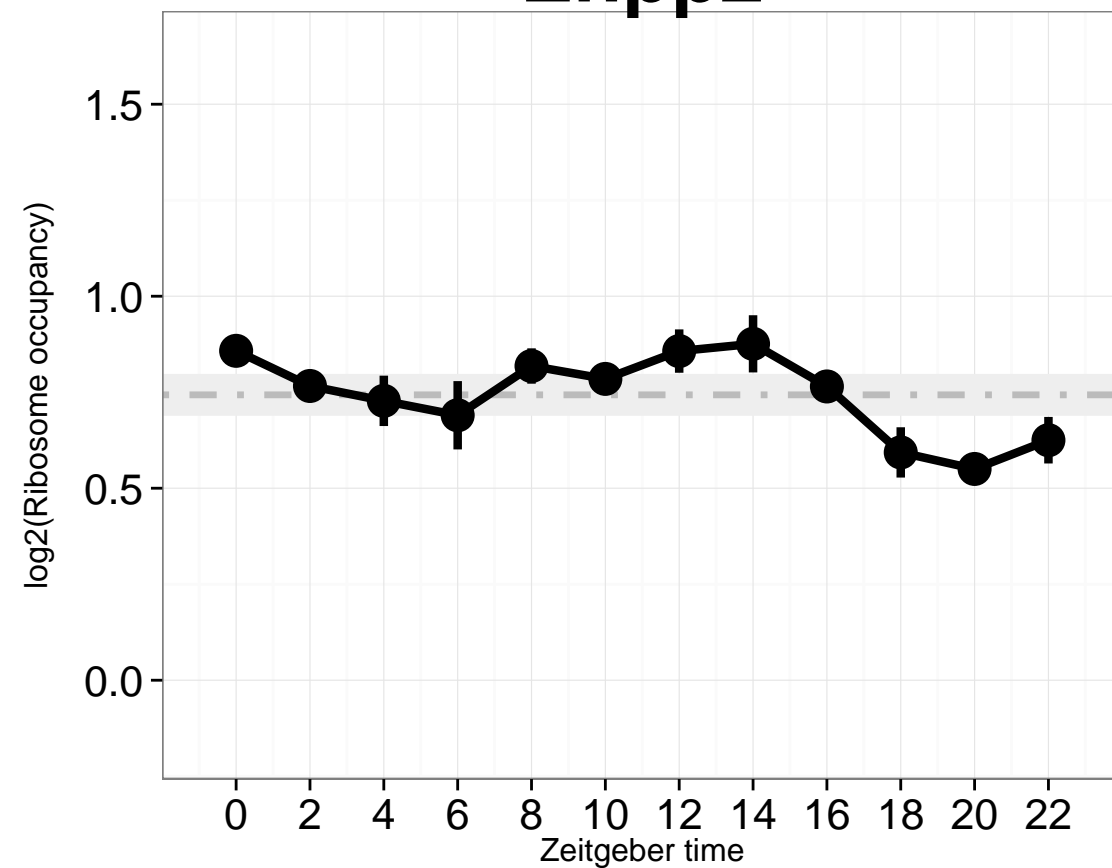

Supplement: Supplementary file 7 — Expression plots for kidney and liver for the 178 common rhythmic genes of Fig. 3c. (ZIP 3338.28 kb) [file 13059_2017_1222_MOESM7_ESM.zip › set_D_shared(178)/Enpp2_kidney_set_D.pdf]

## Enpp2

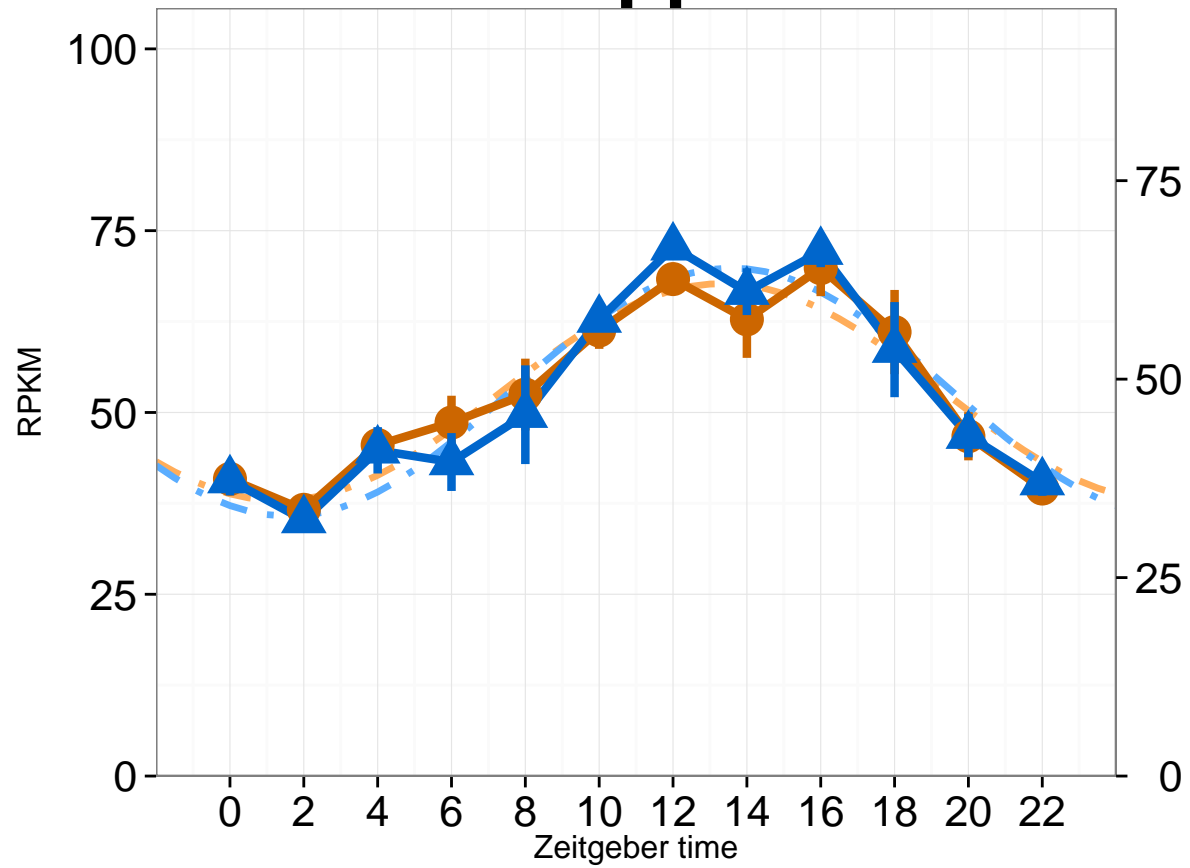

## Enpp2

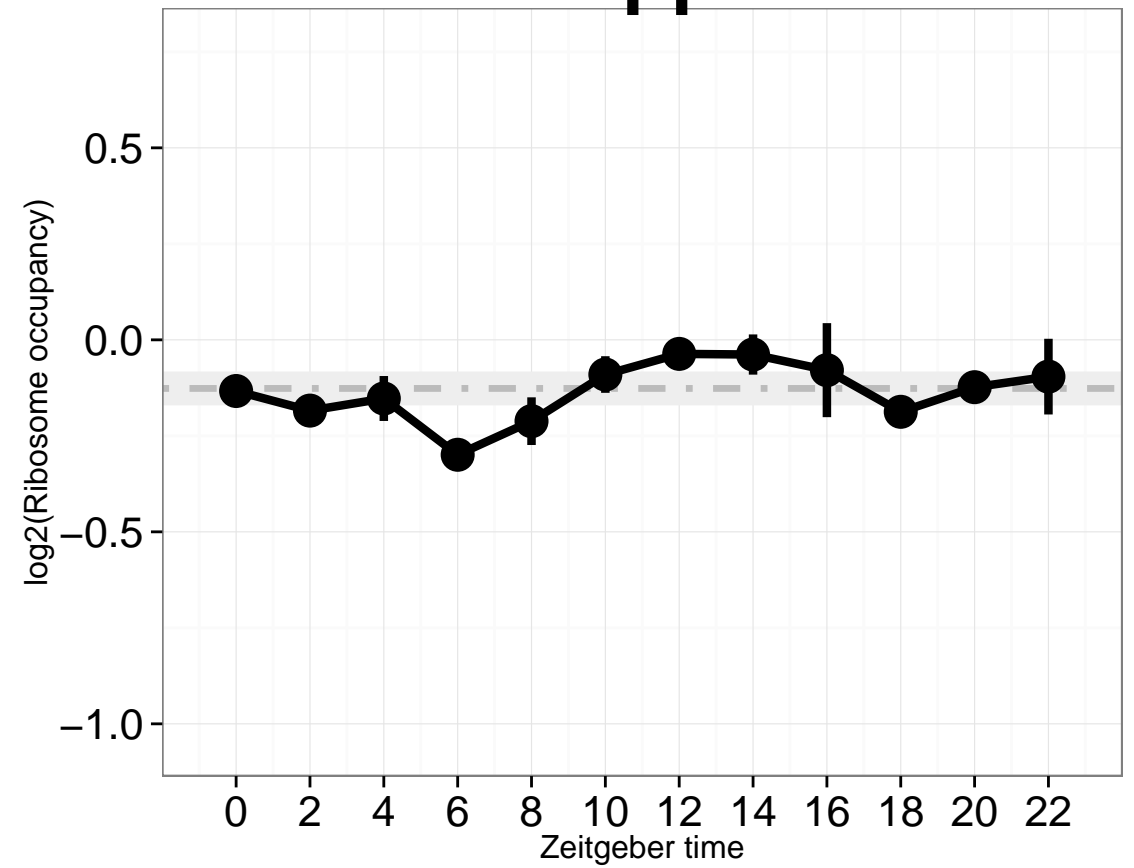

Supplement: Supplementary file 7 — Expression plots for kidney and liver for the 178 common rhythmic genes of Fig. 3c. (ZIP 3338.28 kb) [file 13059_2017_1222_MOESM7_ESM.zip › set_D_shared(178)/Enpp2_liver_set_D.pdf]

## ErbB3

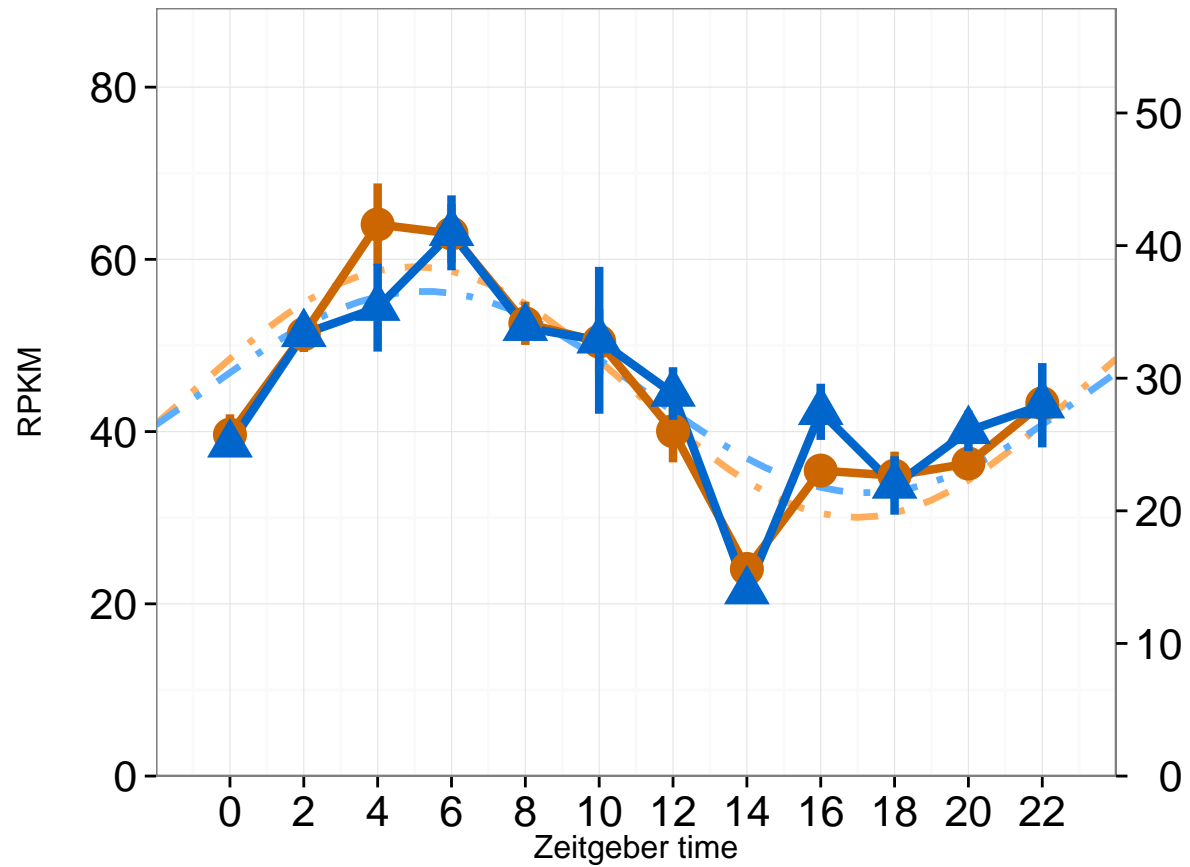

## ErbB3

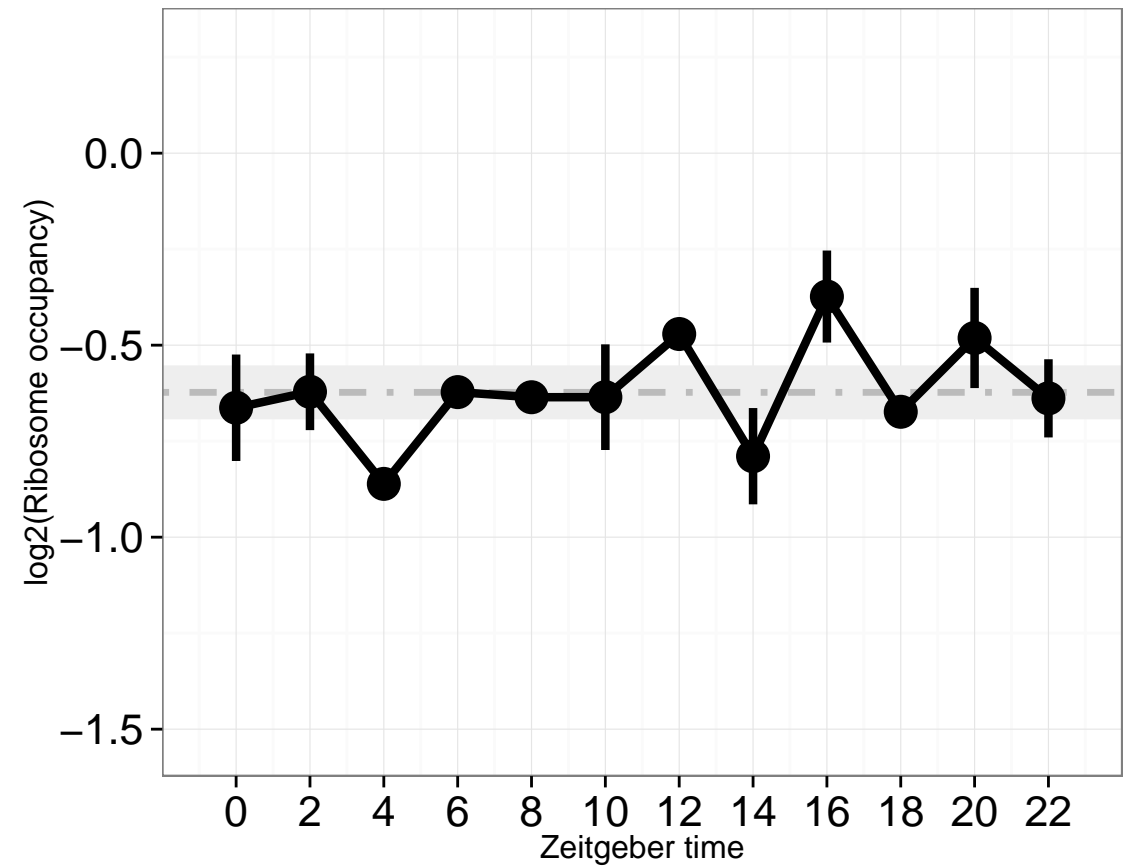

Supplement: Supplementary file 7 — Expression plots for kidney and liver for the 178 common rhythmic genes of Fig. 3c. (ZIP 3338.28 kb) [file 13059_2017_1222_MOESM7_ESM.zip › set_D_shared(178)/Erbb3_kidney_set_D.pdf]

## ErbB3

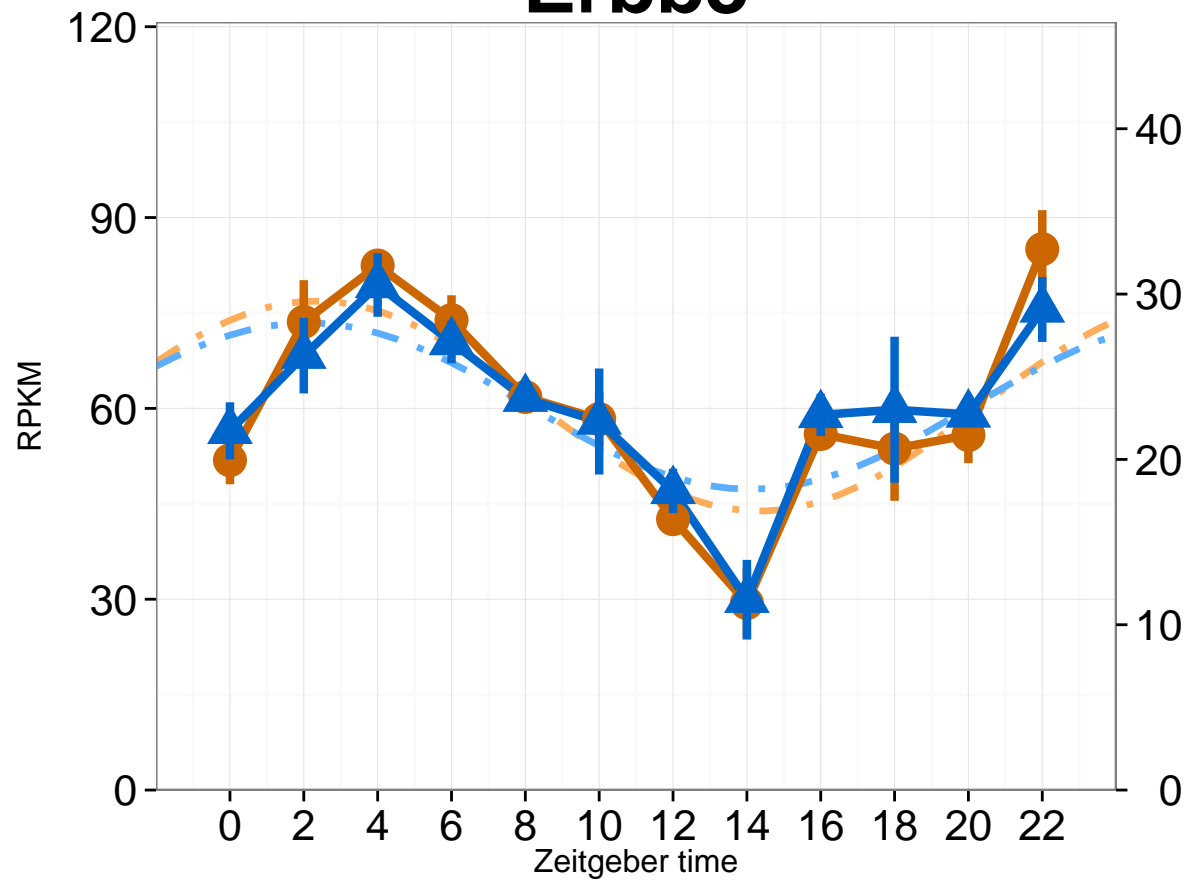

## ErbB3

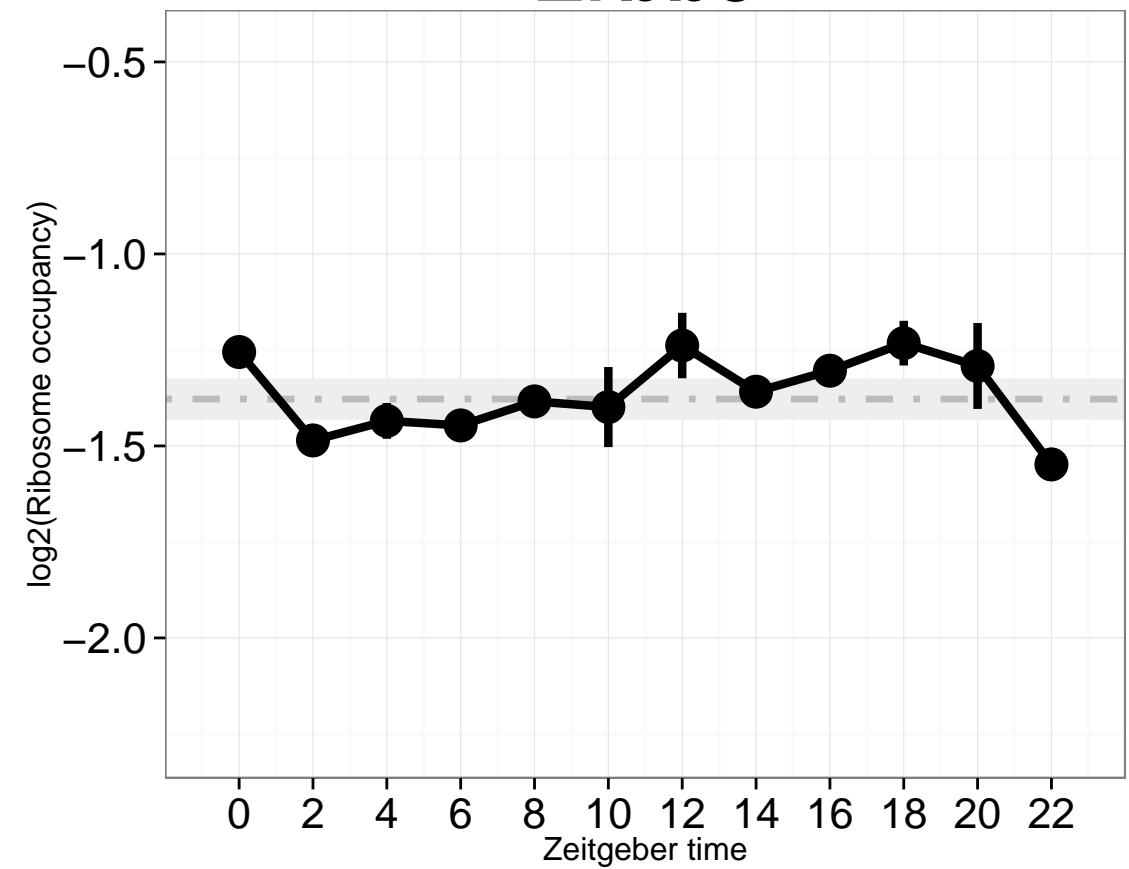

Supplement: Supplementary file 7 — Expression plots for kidney and liver for the 178 common rhythmic genes of Fig. 3c. (ZIP 3338.28 kb) [file 13059_2017_1222_MOESM7_ESM.zip › set_D_shared(178)/Erbb3_liver_set_D.pdf]

## Ethe1

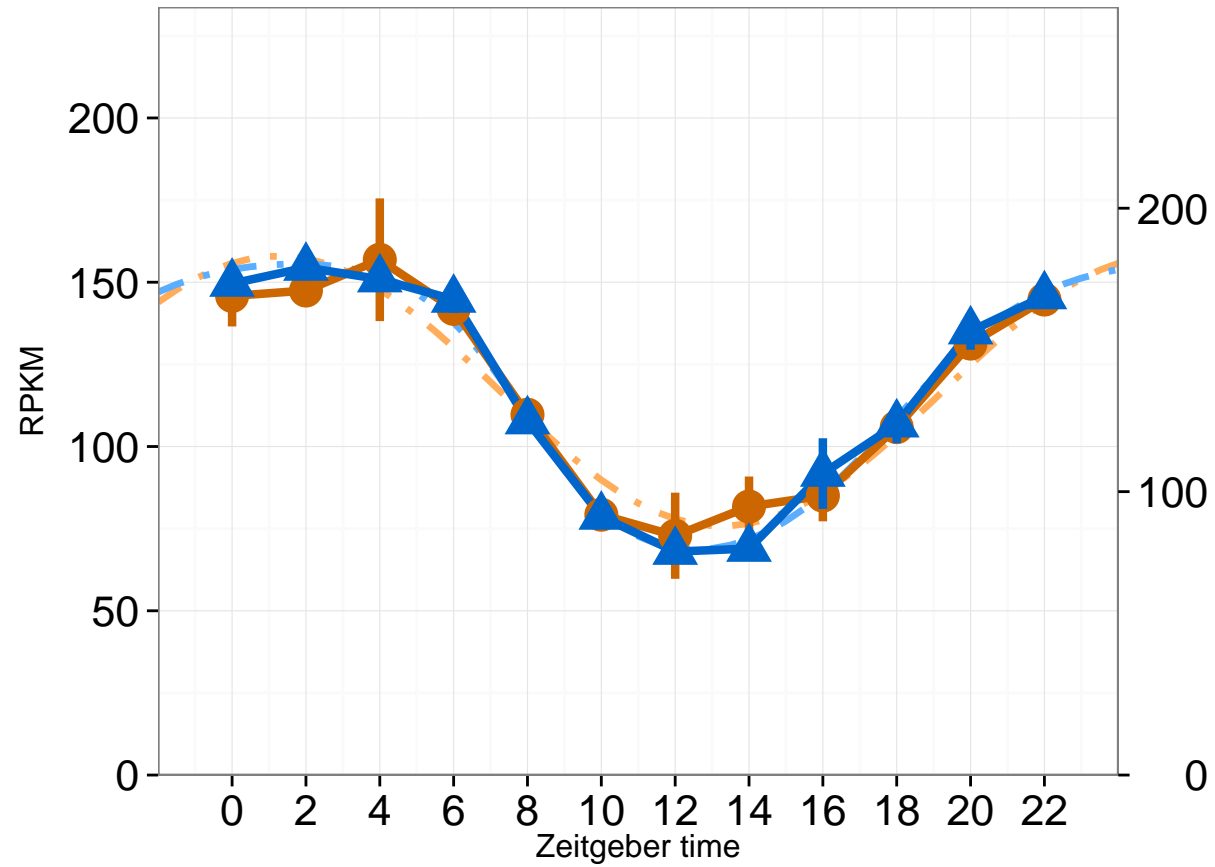

## Ethe1

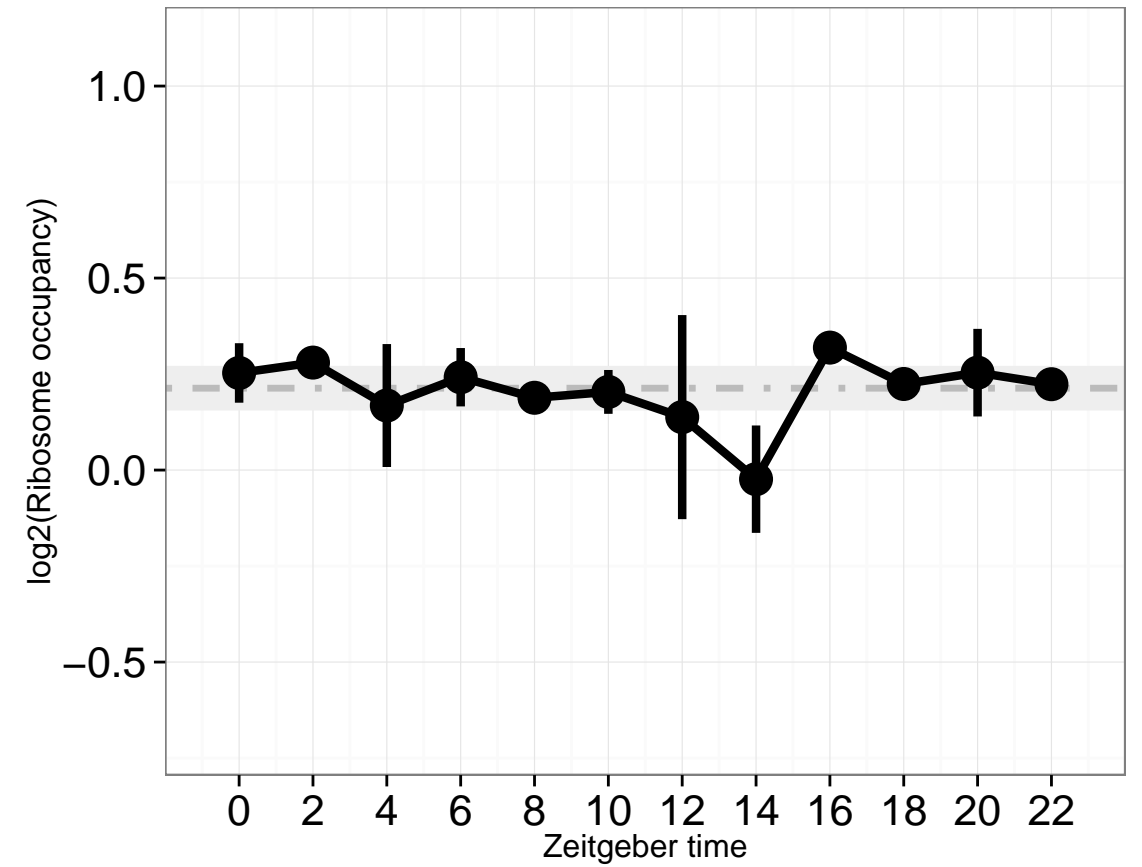

Supplement: Supplementary file 7 — Expression plots for kidney and liver for the 178 common rhythmic genes of Fig. 3c. (ZIP 3338.28 kb) [file 13059_2017_1222_MOESM7_ESM.zip › set_D_shared(178)/Ethe1_kidney_set_D.pdf]

## Ethe1

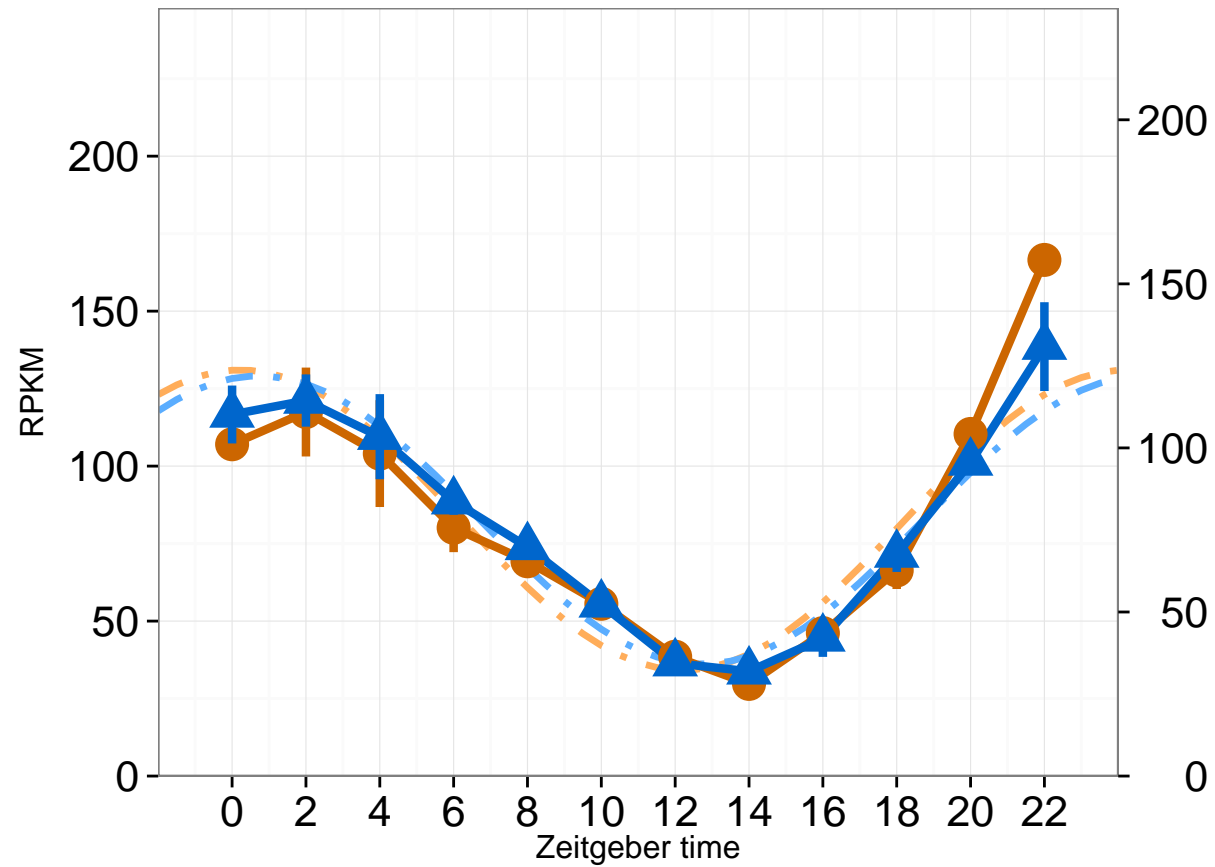

## Ethe1

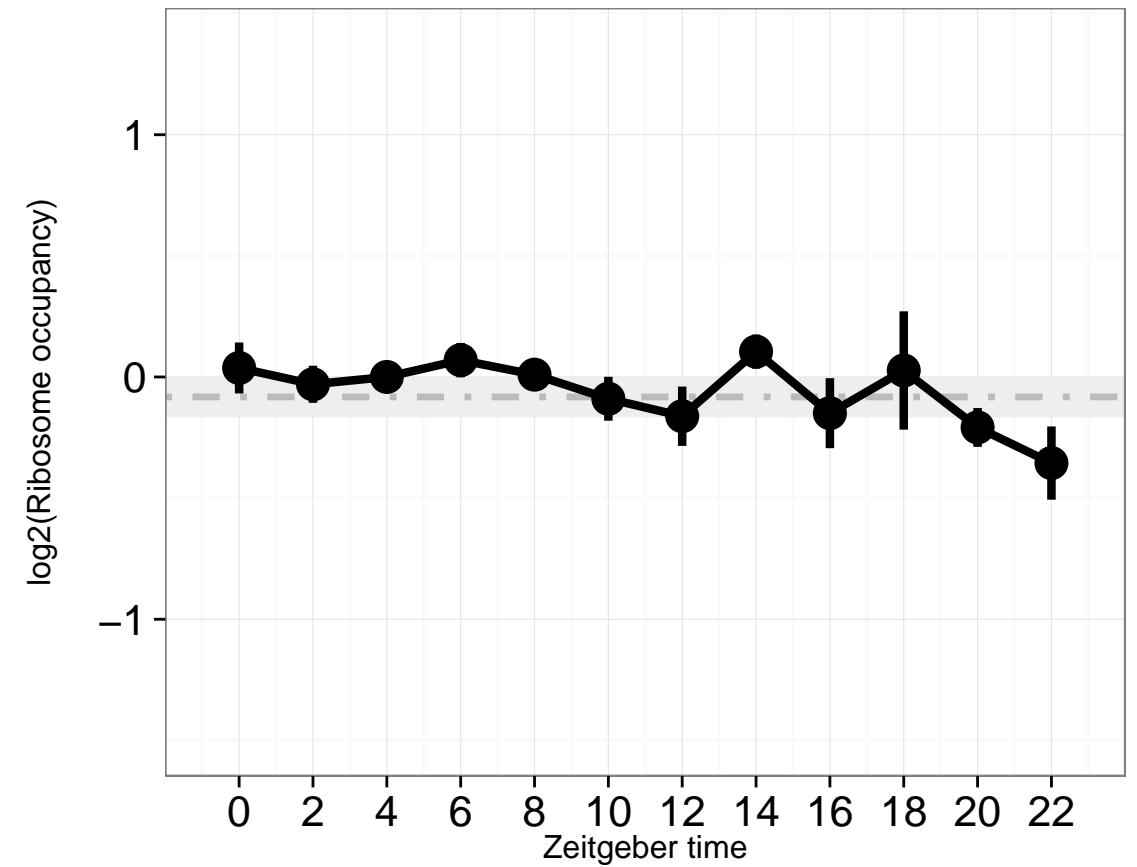

Supplement: Supplementary file 7 — Expression plots for kidney and liver for the 178 common rhythmic genes of Fig. 3c. (ZIP 3338.28 kb) [file 13059_2017_1222_MOESM7_ESM.zip › set_D_shared(178)/Ethe1_liver_set_D.pdf]

## Etnk2

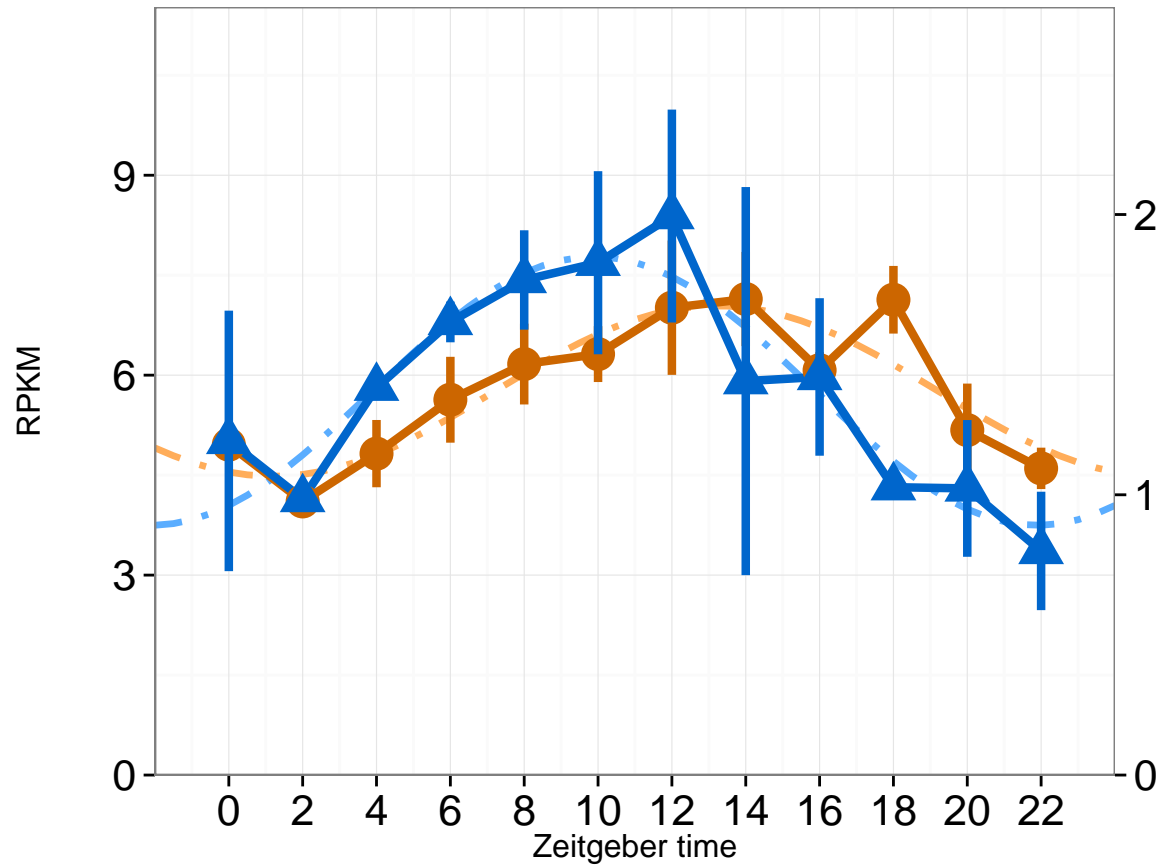

## Etnk2

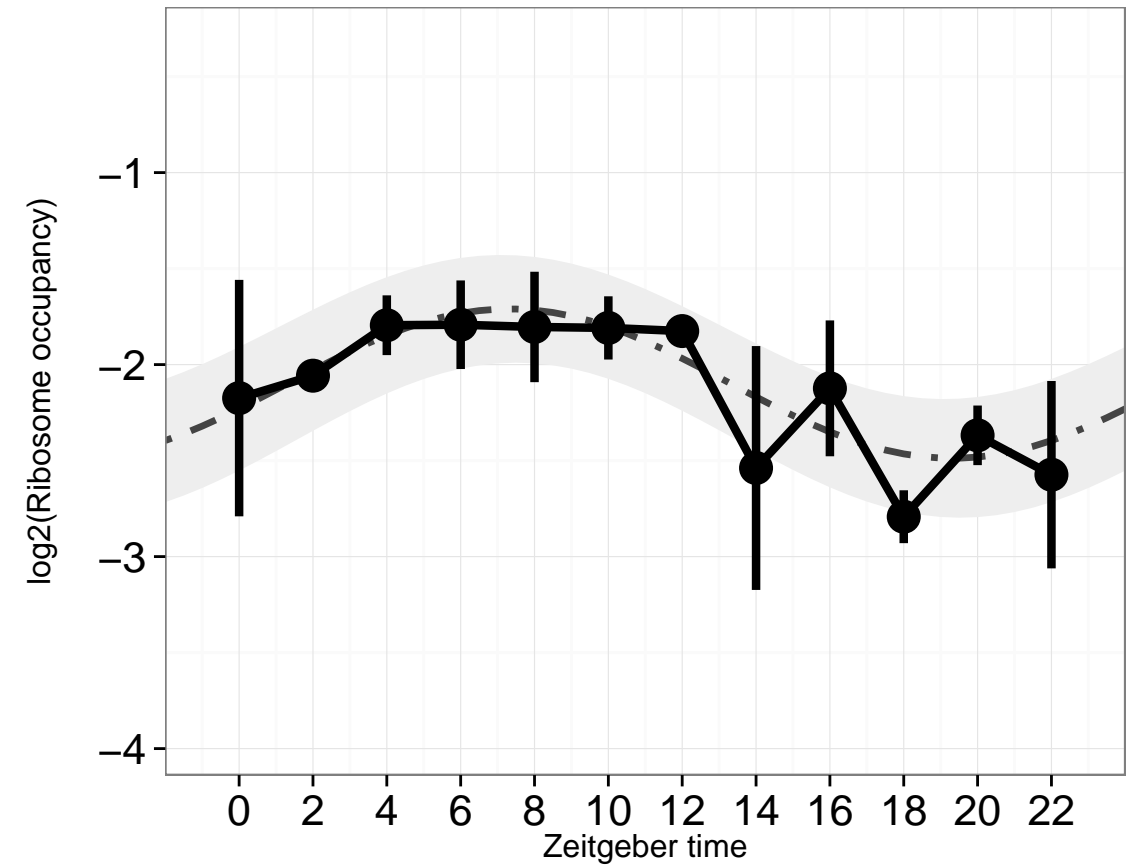

Supplement: Supplementary file 7 — Expression plots for kidney and liver for the 178 common rhythmic genes of Fig. 3c. (ZIP 3338.28 kb) [file 13059_2017_1222_MOESM7_ESM.zip › set_D_shared(178)/Etnk2_kidney_set_D.pdf]

## Etnk2

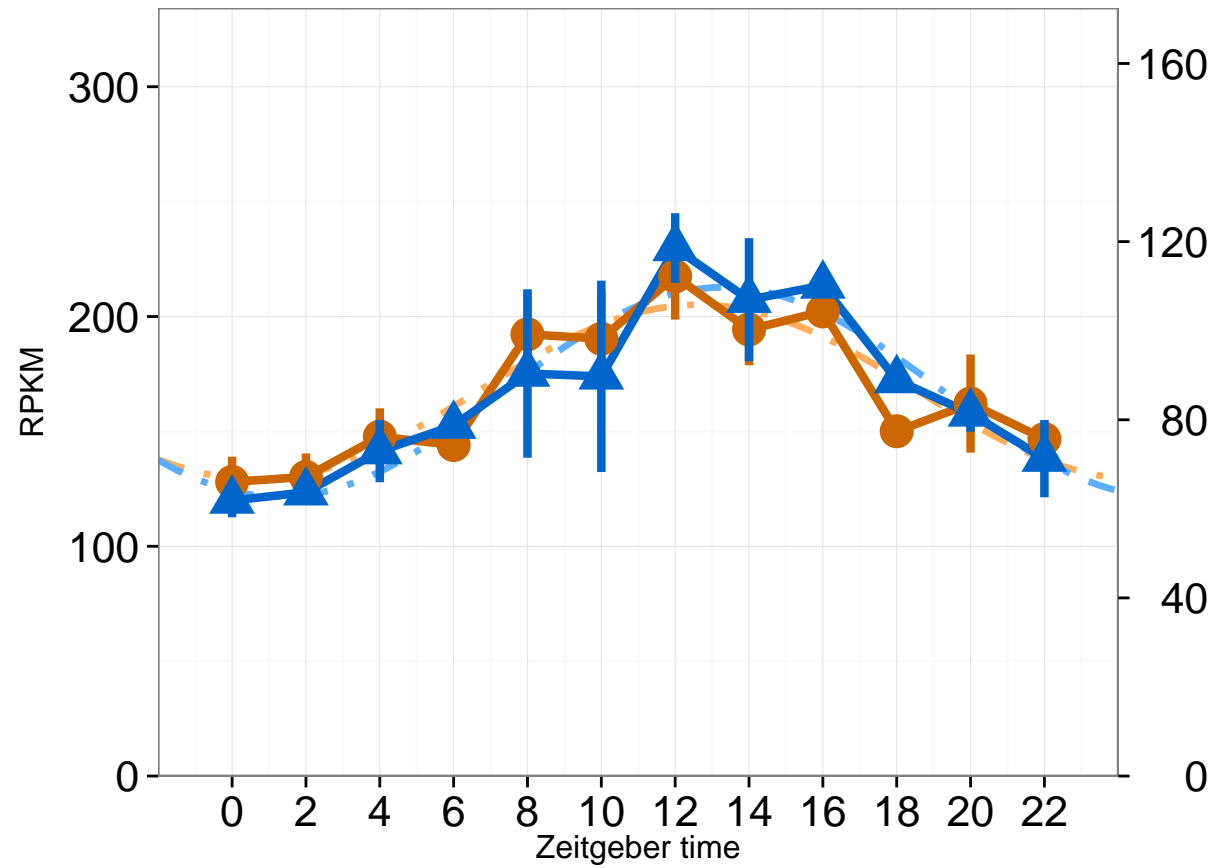

## Etnk2

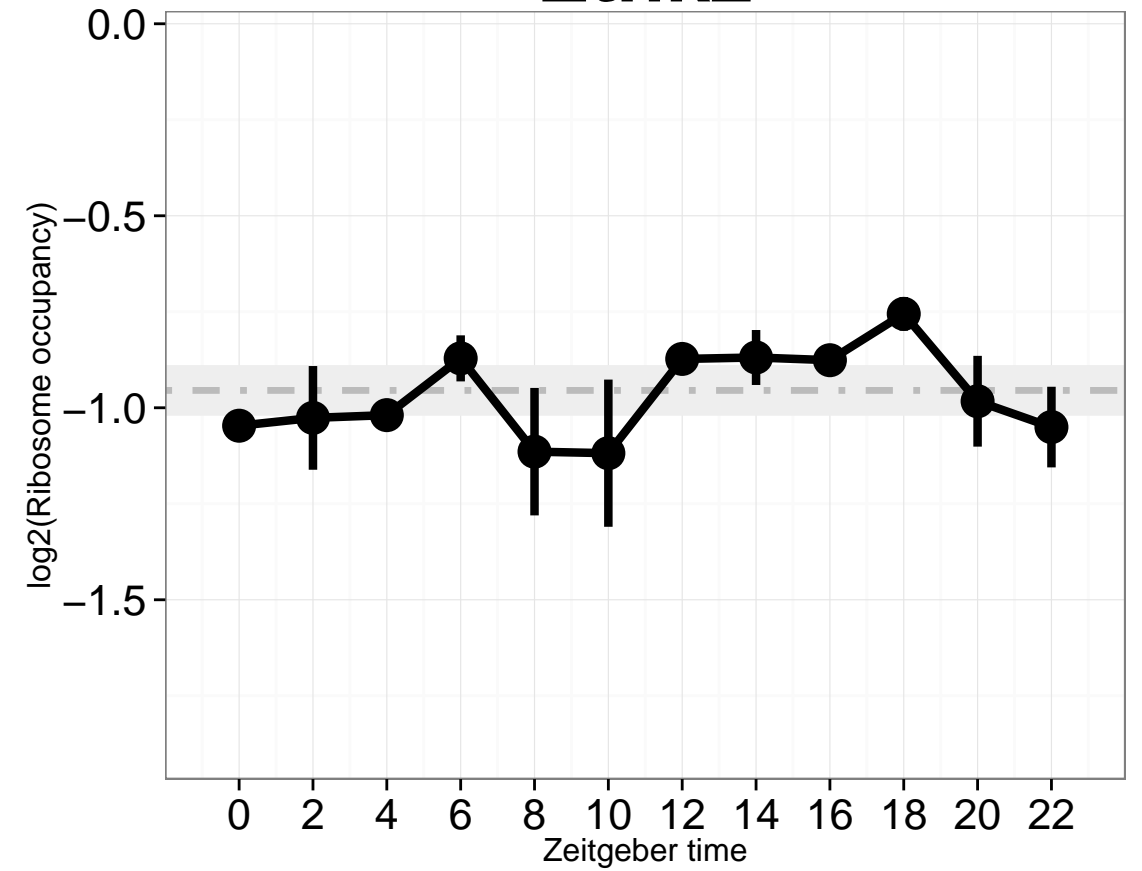

Supplement: Supplementary file 7 — Expression plots for kidney and liver for the 178 common rhythmic genes of Fig. 3c. (ZIP 3338.28 kb) [file 13059_2017_1222_MOESM7_ESM.zip › set_D_shared(178)/Etnk2_liver_set_D.pdf]

# Fam102a

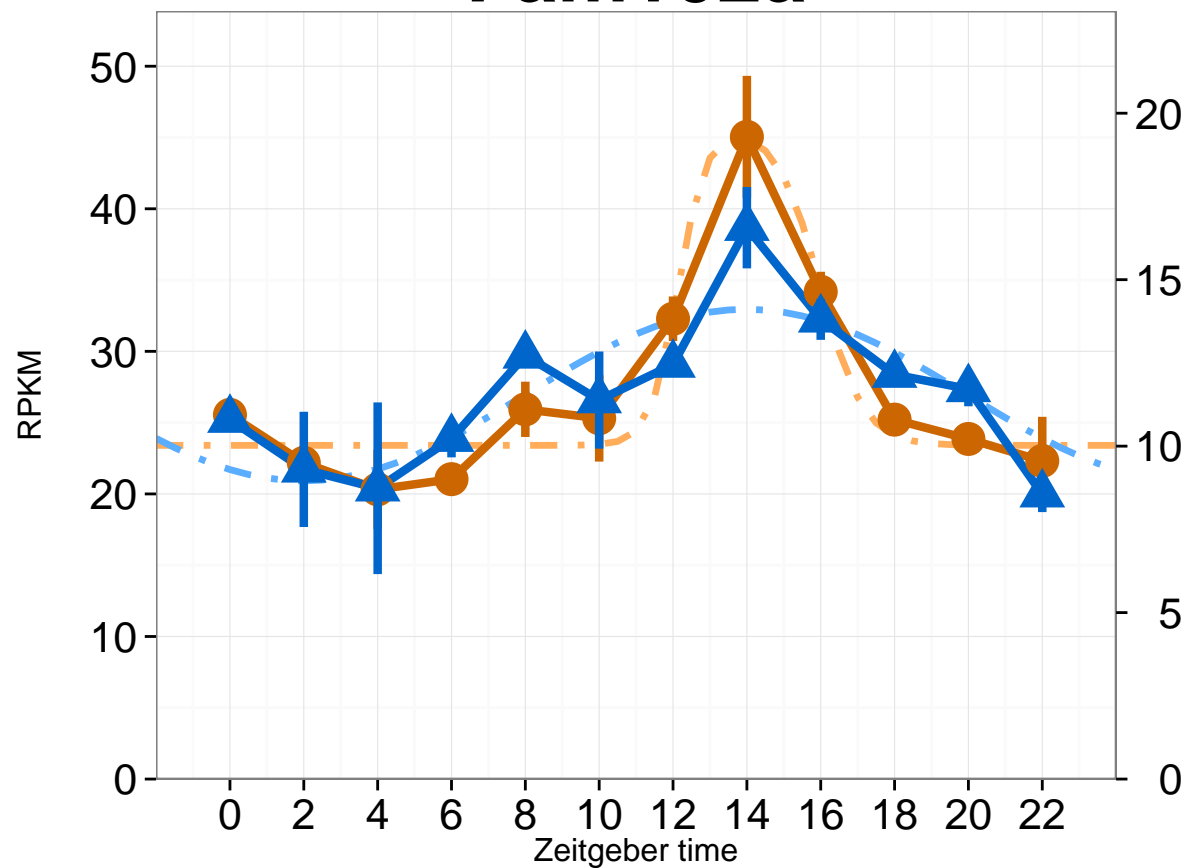

# Fam102a

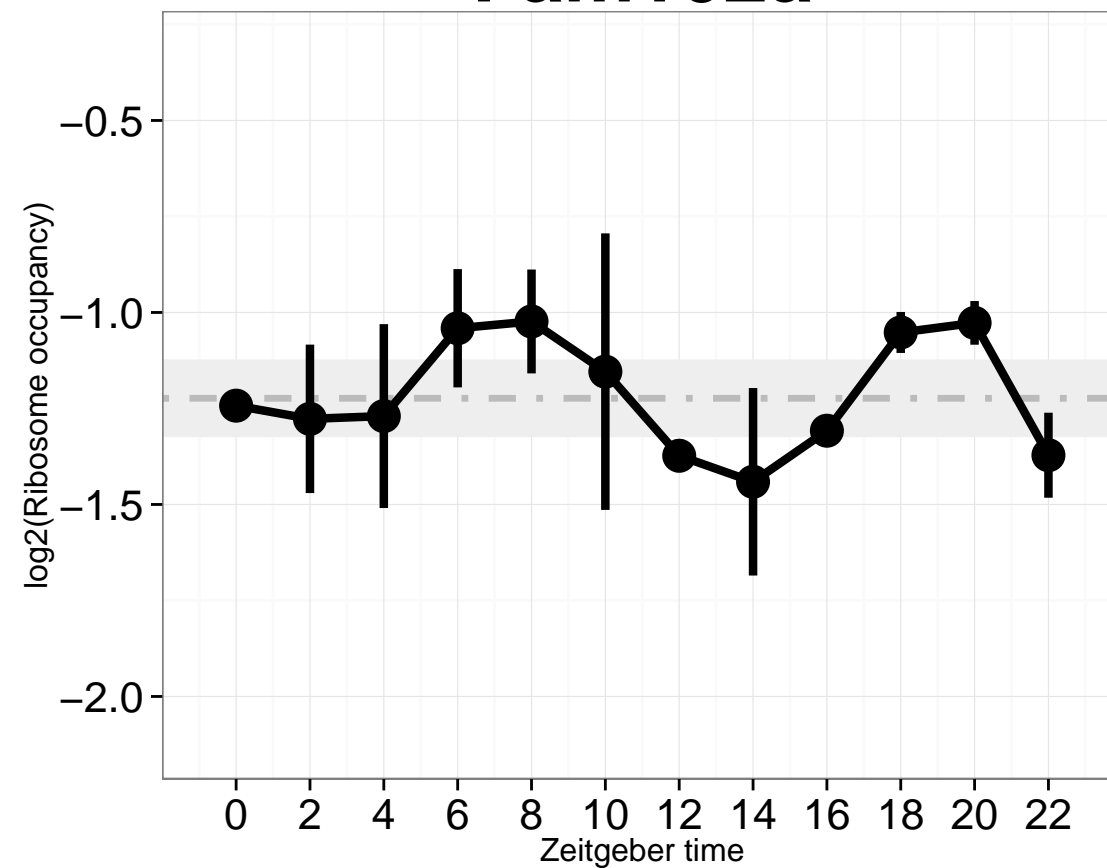

Supplement: Supplementary file 7 — Expression plots for kidney and liver for the 178 common rhythmic genes of Fig. 3c. (ZIP 3338.28 kb) [file 13059_2017_1222_MOESM7_ESM.zip › set_D_shared(178)/Fam102a_kidney_set_D.pdf]

# Fam102a

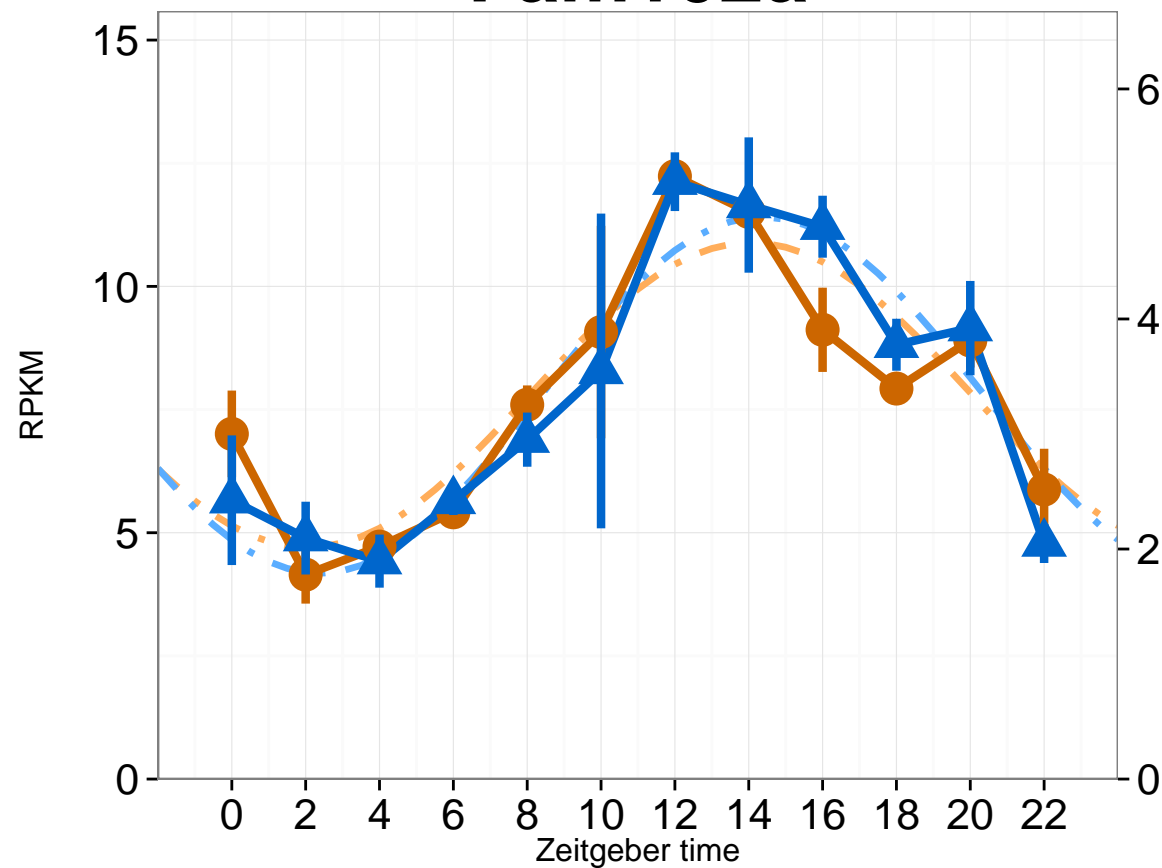

# Fam102a

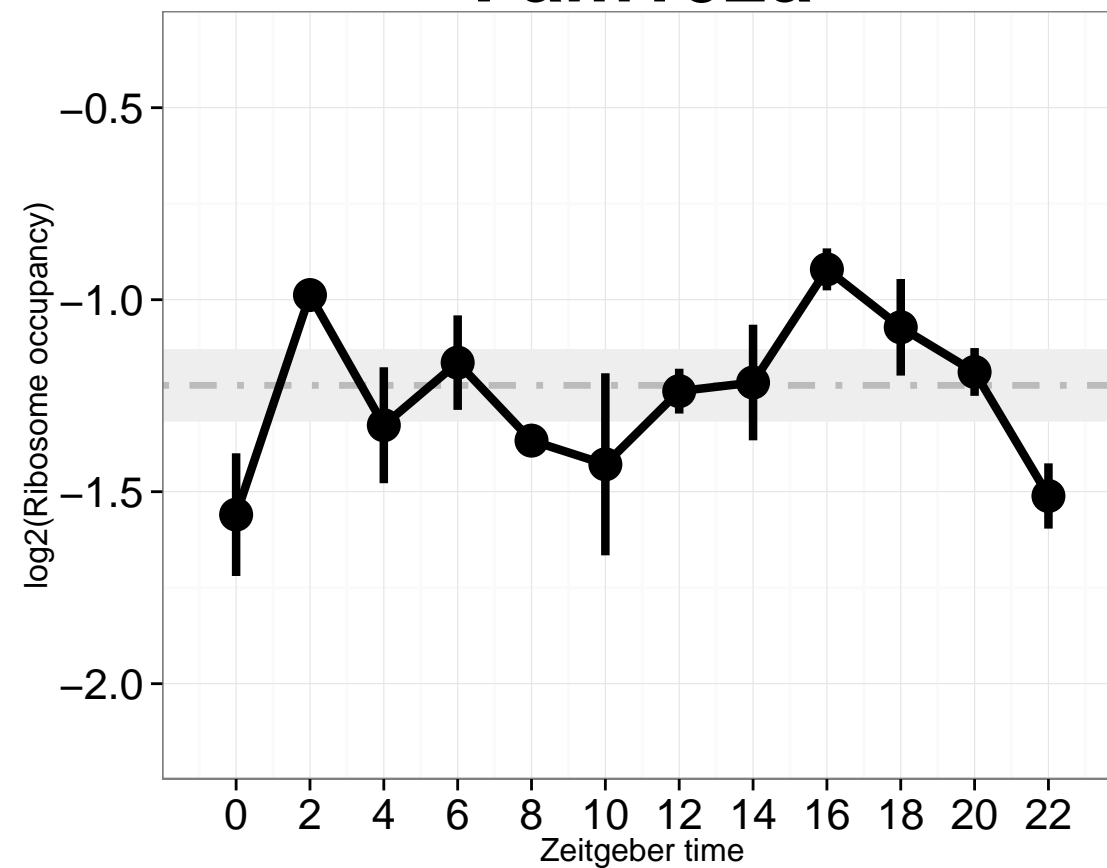

Supplement: Supplementary file 7 — Expression plots for kidney and liver for the 178 common rhythmic genes of Fig. 3c. (ZIP 3338.28 kb) [file 13059_2017_1222_MOESM7_ESM.zip › set_D_shared(178)/Fam102a_liver_set_D.pdf]

## Fam20a

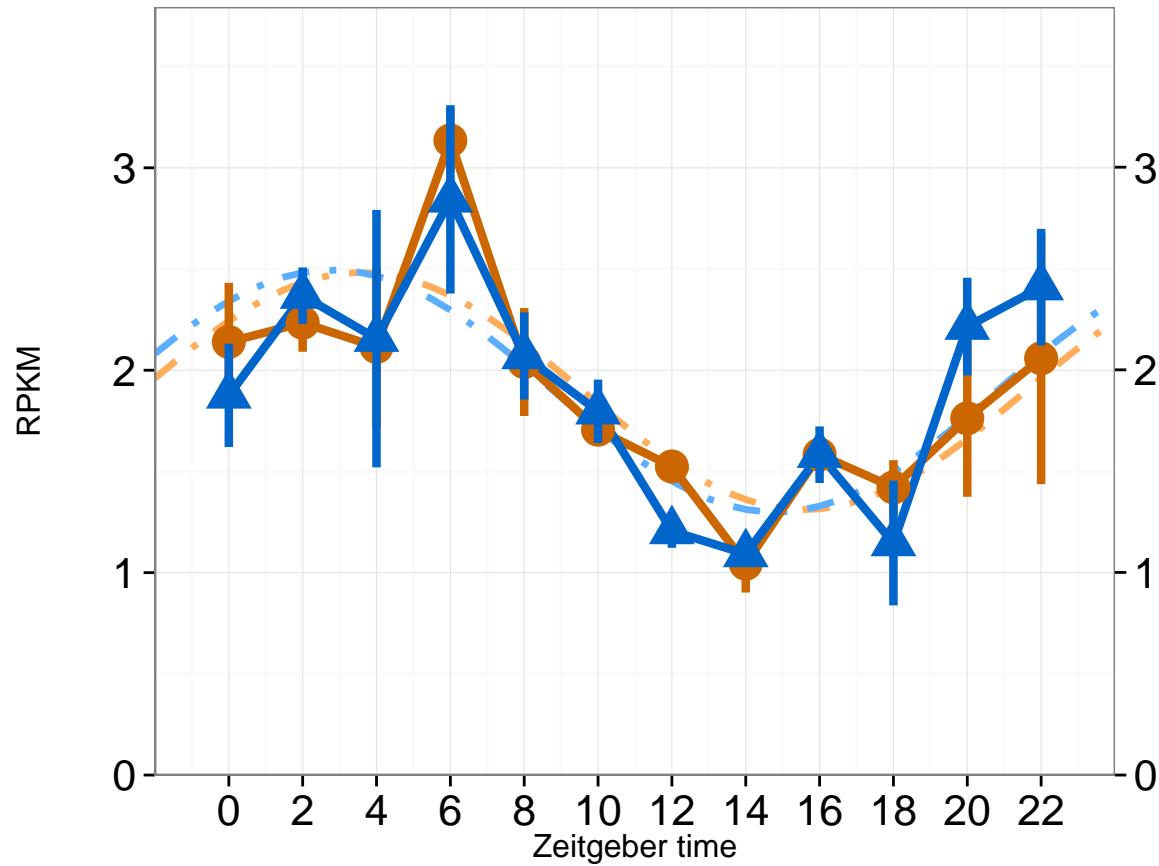

## Fam20a

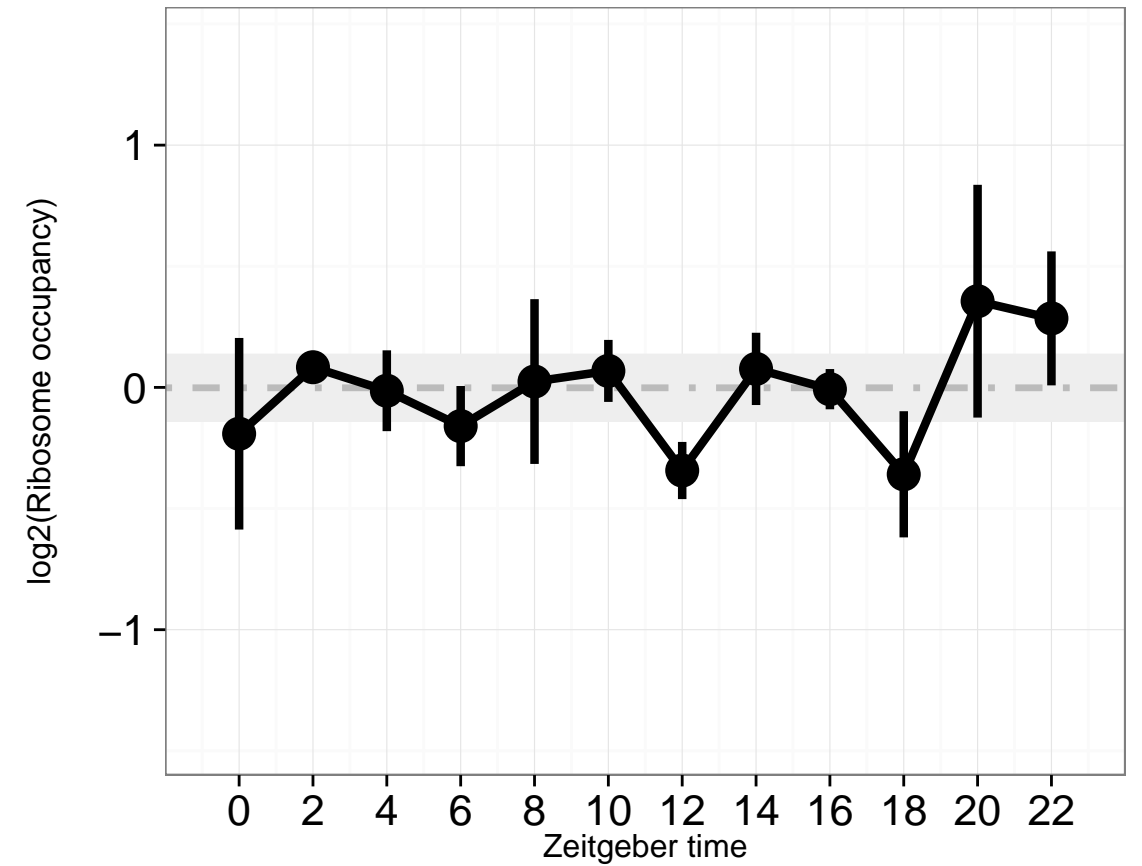

Supplement: Supplementary file 7 — Expression plots for kidney and liver for the 178 common rhythmic genes of Fig. 3c. (ZIP 3338.28 kb) [file 13059_2017_1222_MOESM7_ESM.zip › set_D_shared(178)/Fam20a_kidney_set_D.pdf]

# Fam20a

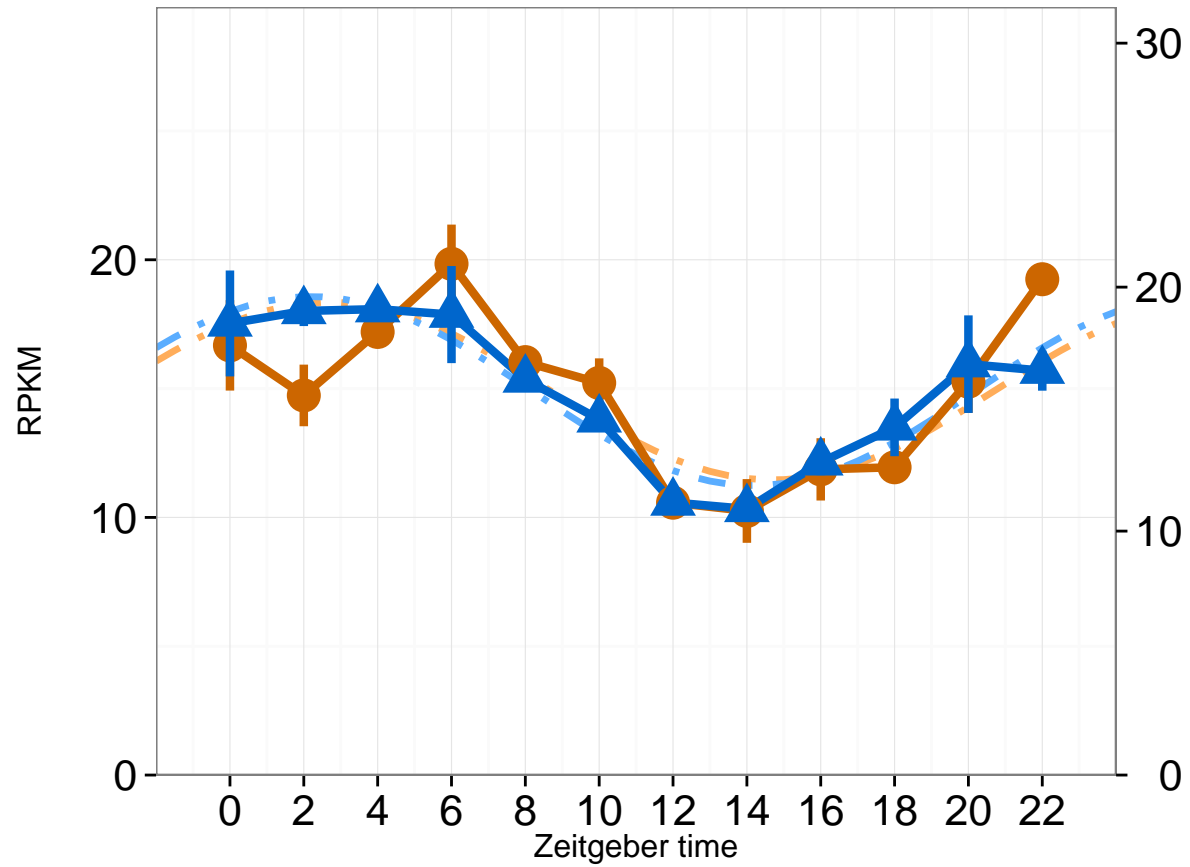

# Fam20a

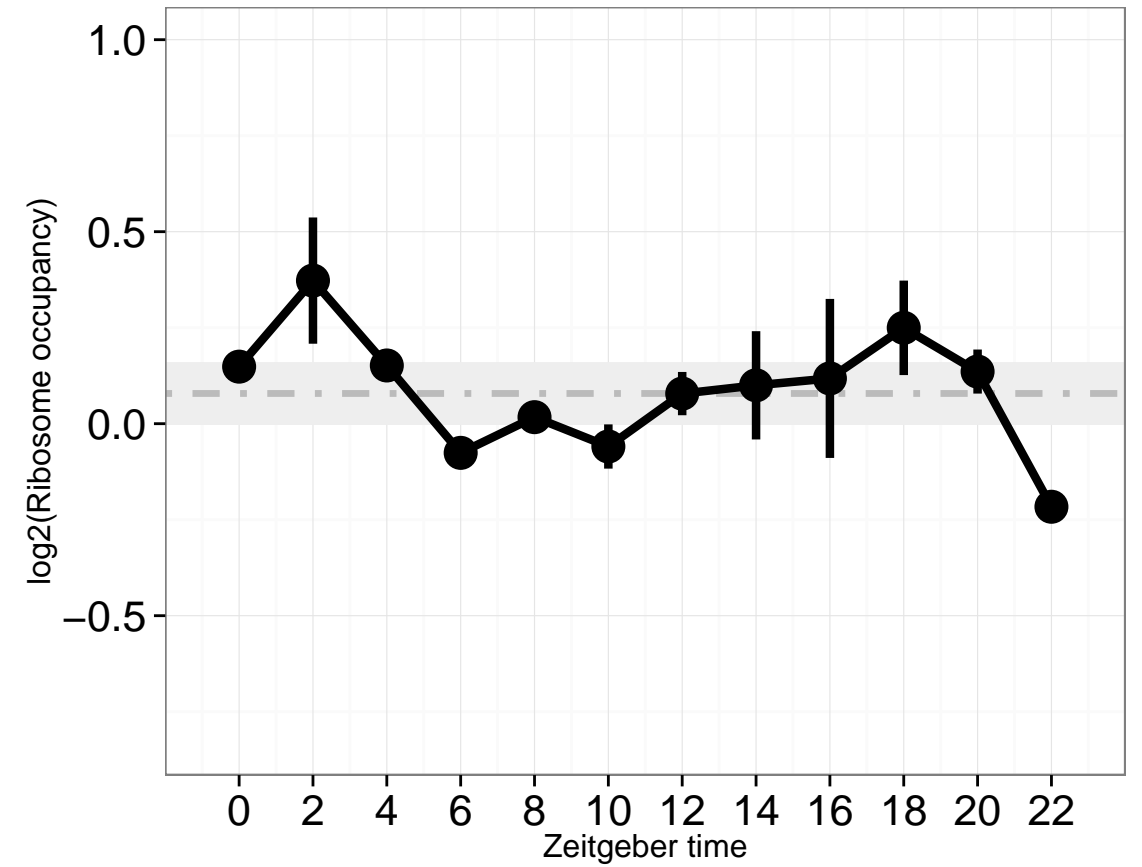

Supplement: Supplementary file 7 — Expression plots for kidney and liver for the 178 common rhythmic genes of Fig. 3c. (ZIP 3338.28 kb) [file 13059_2017_1222_MOESM7_ESM.zip › set_D_shared(178)/Fam20a_liver_set_D.pdf]

## Fdps

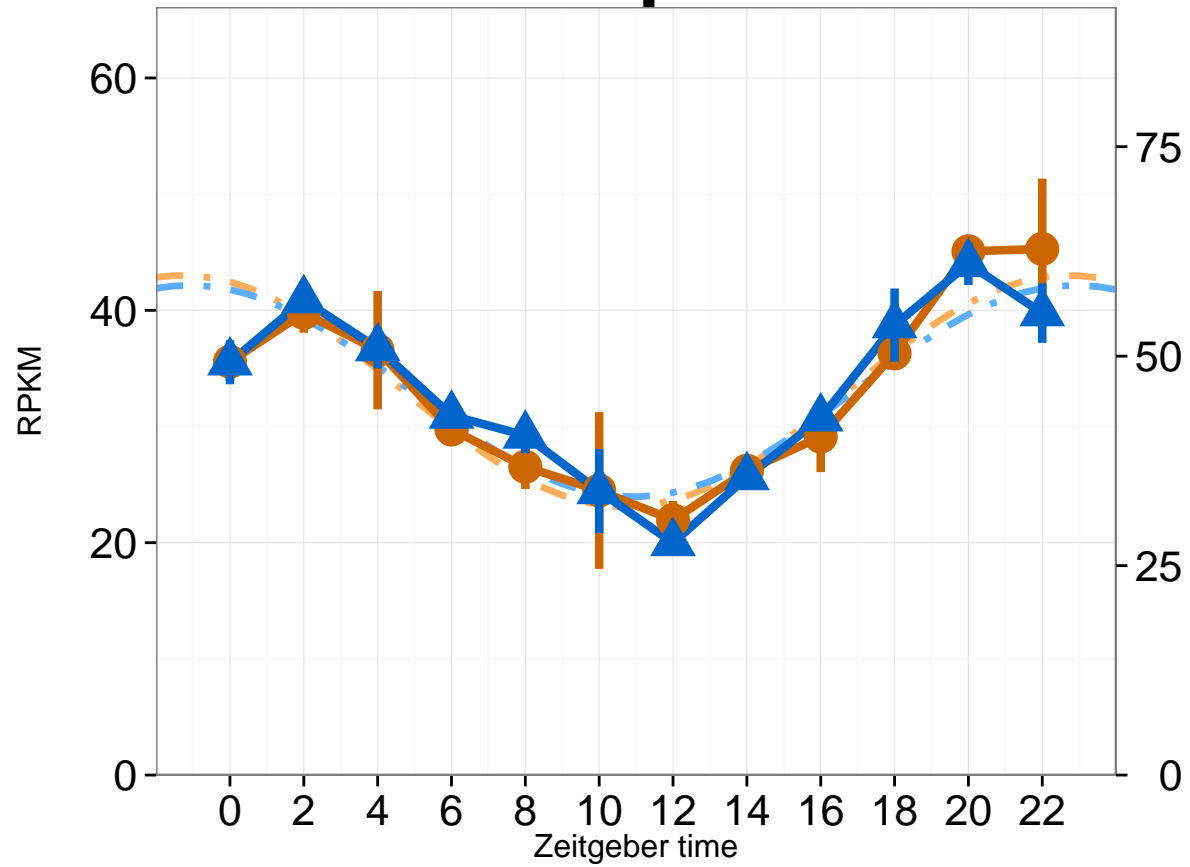

## Fdps

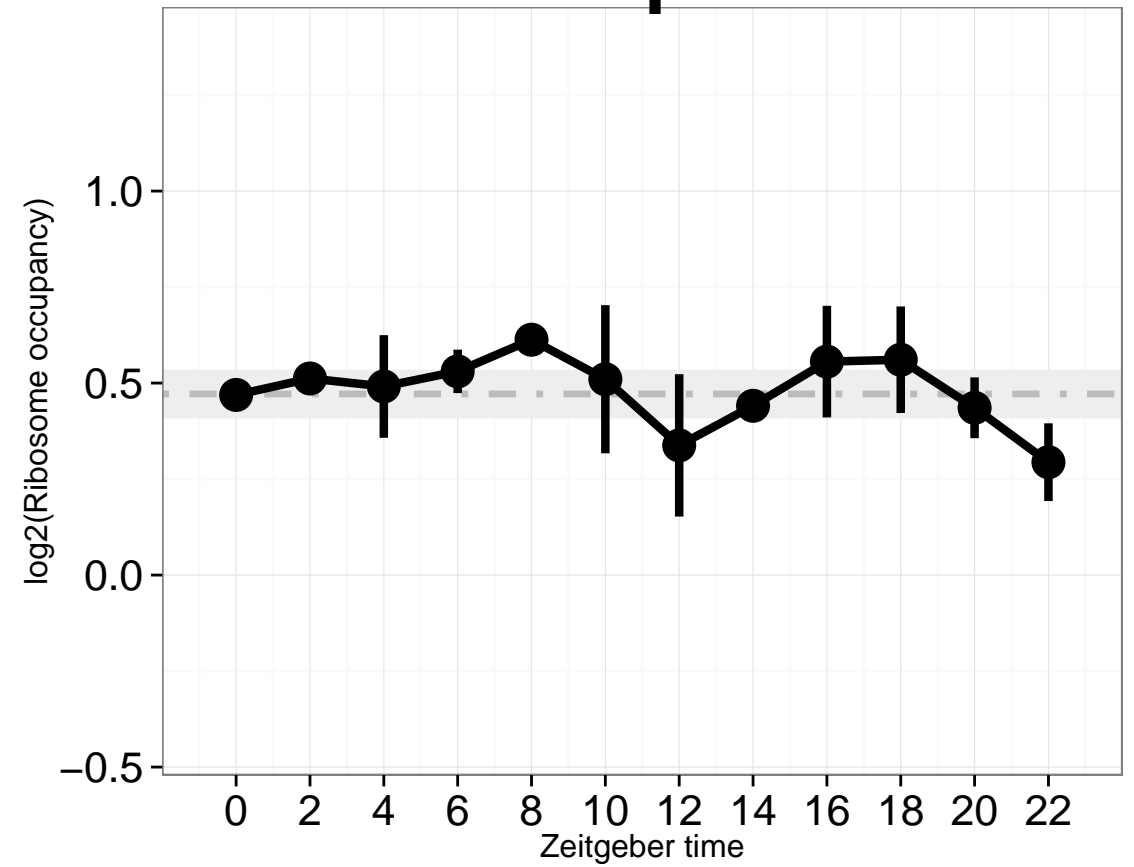

Supplement: Supplementary file 7 — Expression plots for kidney and liver for the 178 common rhythmic genes of Fig. 3c. (ZIP 3338.28 kb) [file 13059_2017_1222_MOESM7_ESM.zip › set_D_shared(178)/Fdps_kidney_set_D.pdf]

## Fdps

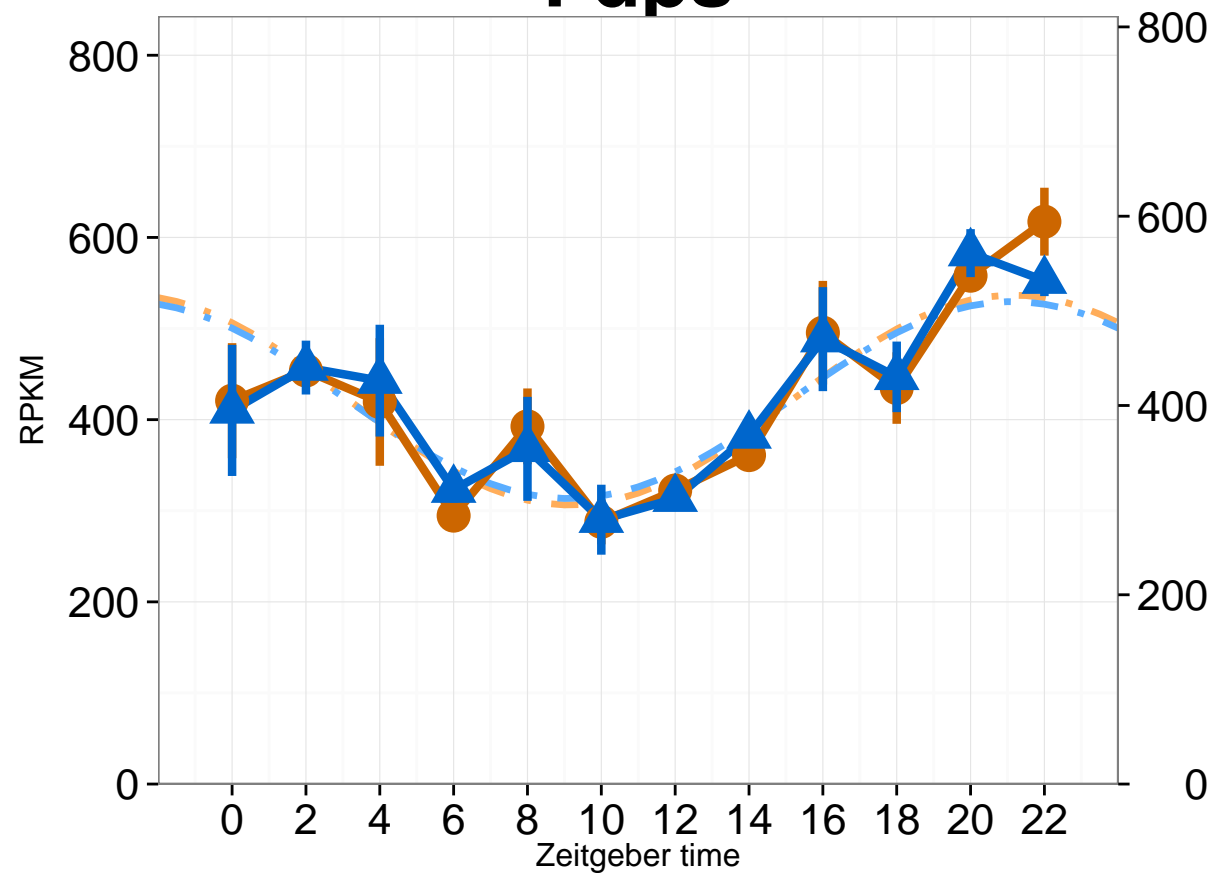

## Fdps

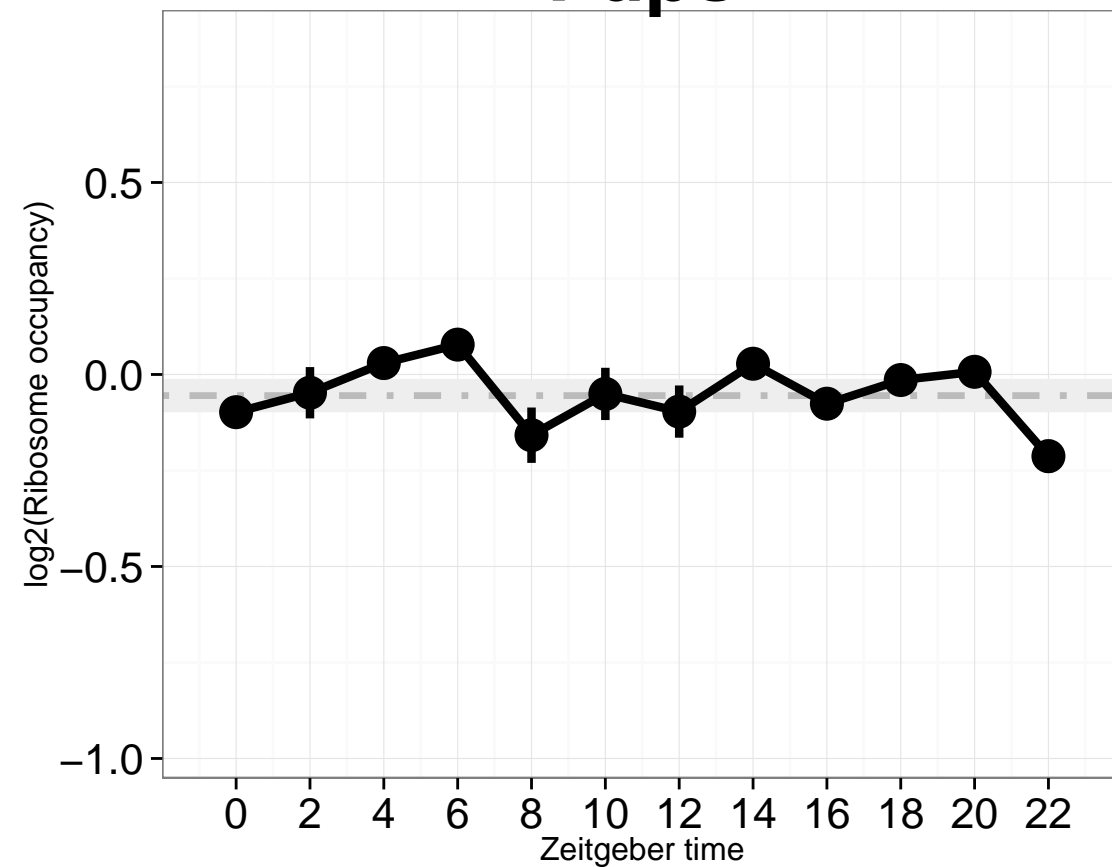

Supplement: Supplementary file 7 — Expression plots for kidney and liver for the 178 common rhythmic genes of Fig. 3c. (ZIP 3338.28 kb) [file 13059_2017_1222_MOESM7_ESM.zip › set_D_shared(178)/Fdps_liver_set_D.pdf]

## Fkbp5

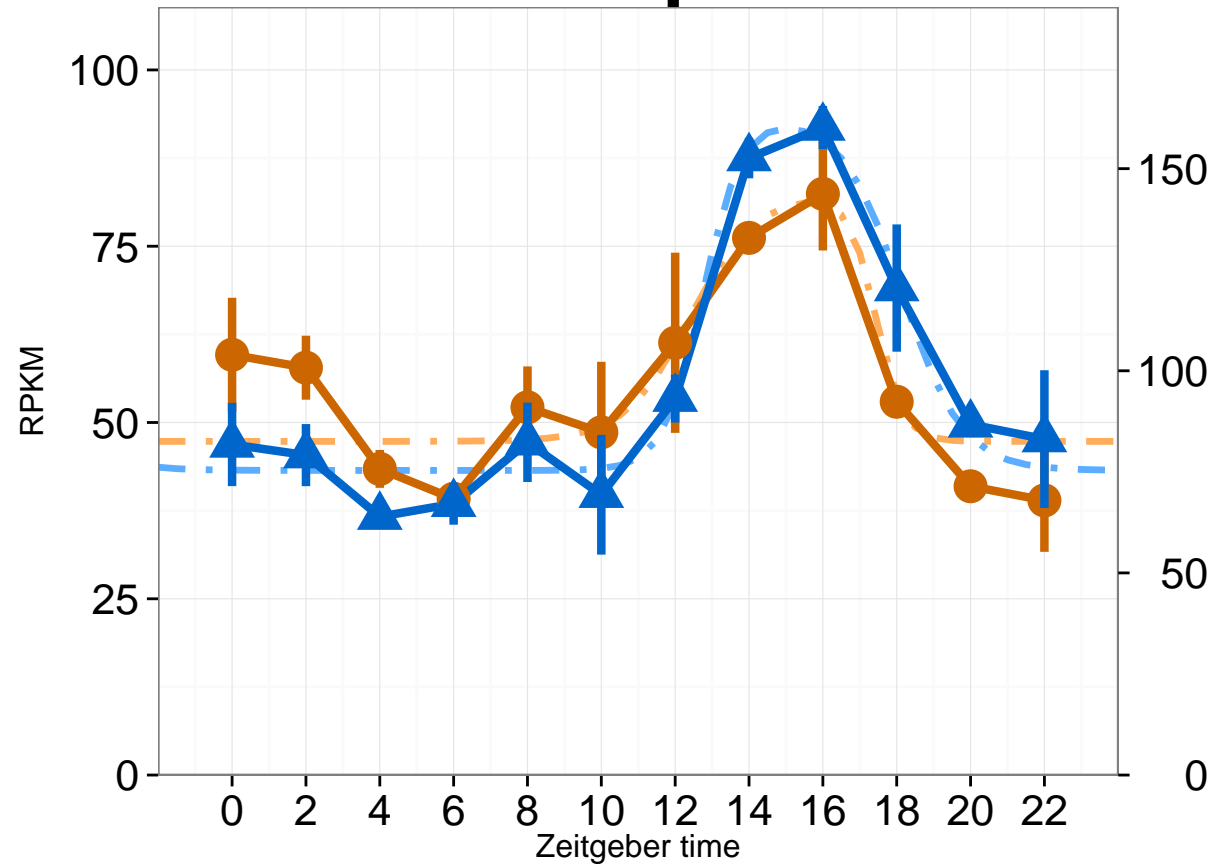

## Fkbp5

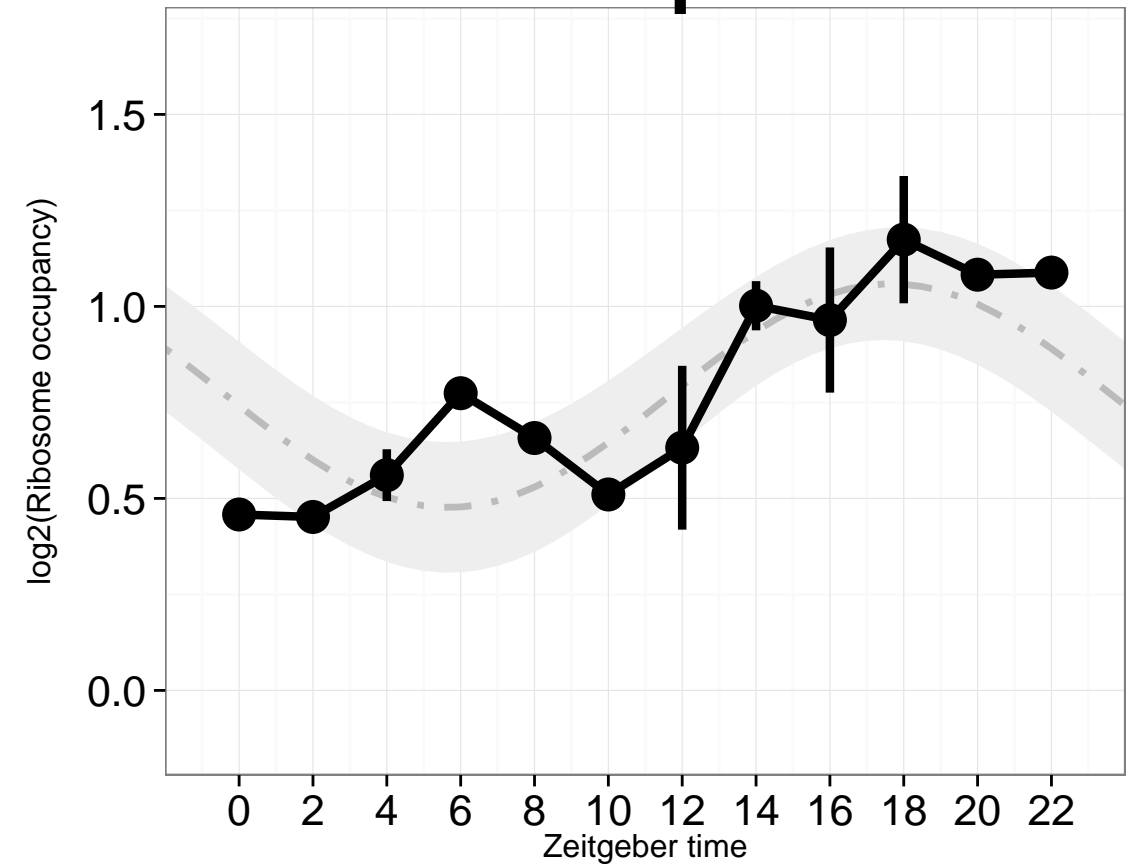

Supplement: Supplementary file 7 — Expression plots for kidney and liver for the 178 common rhythmic genes of Fig. 3c. (ZIP 3338.28 kb) [file 13059_2017_1222_MOESM7_ESM.zip › set_D_shared(178)/Fkbp5_kidney_set_D.pdf]

## Fkbp5

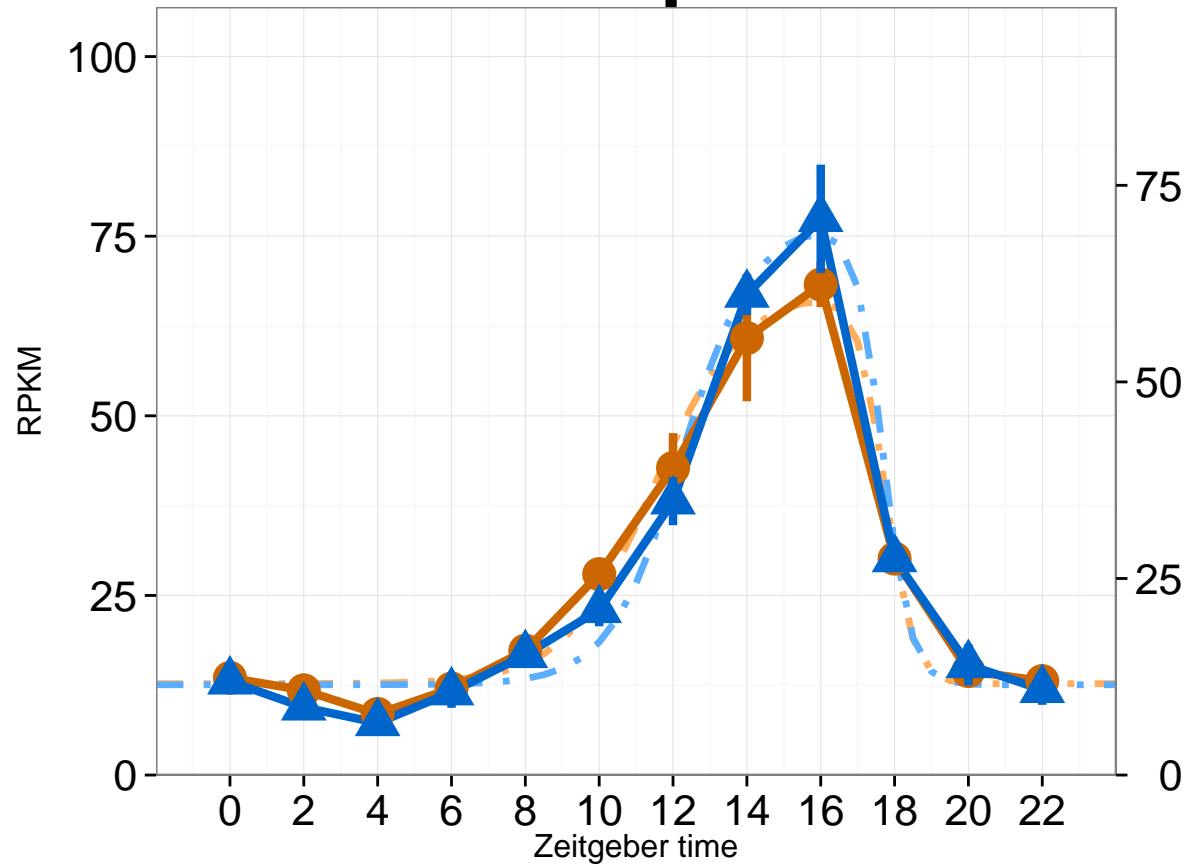

## Fkbp5

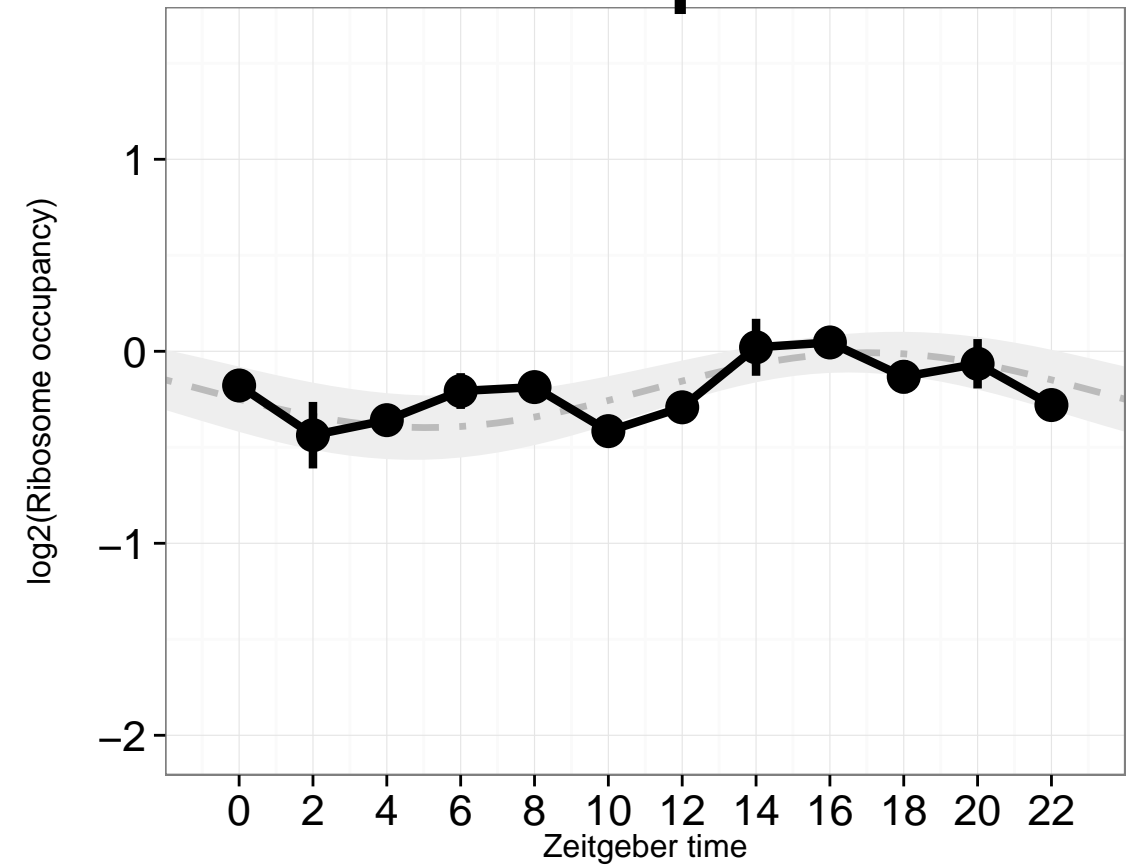

Supplement: Supplementary file 7 — Expression plots for kidney and liver for the 178 common rhythmic genes of Fig. 3c. (ZIP 3338.28 kb) [file 13059_2017_1222_MOESM7_ESM.zip › set_D_shared(178)/Fkbp5_liver_set_D.pdf]

## Fus

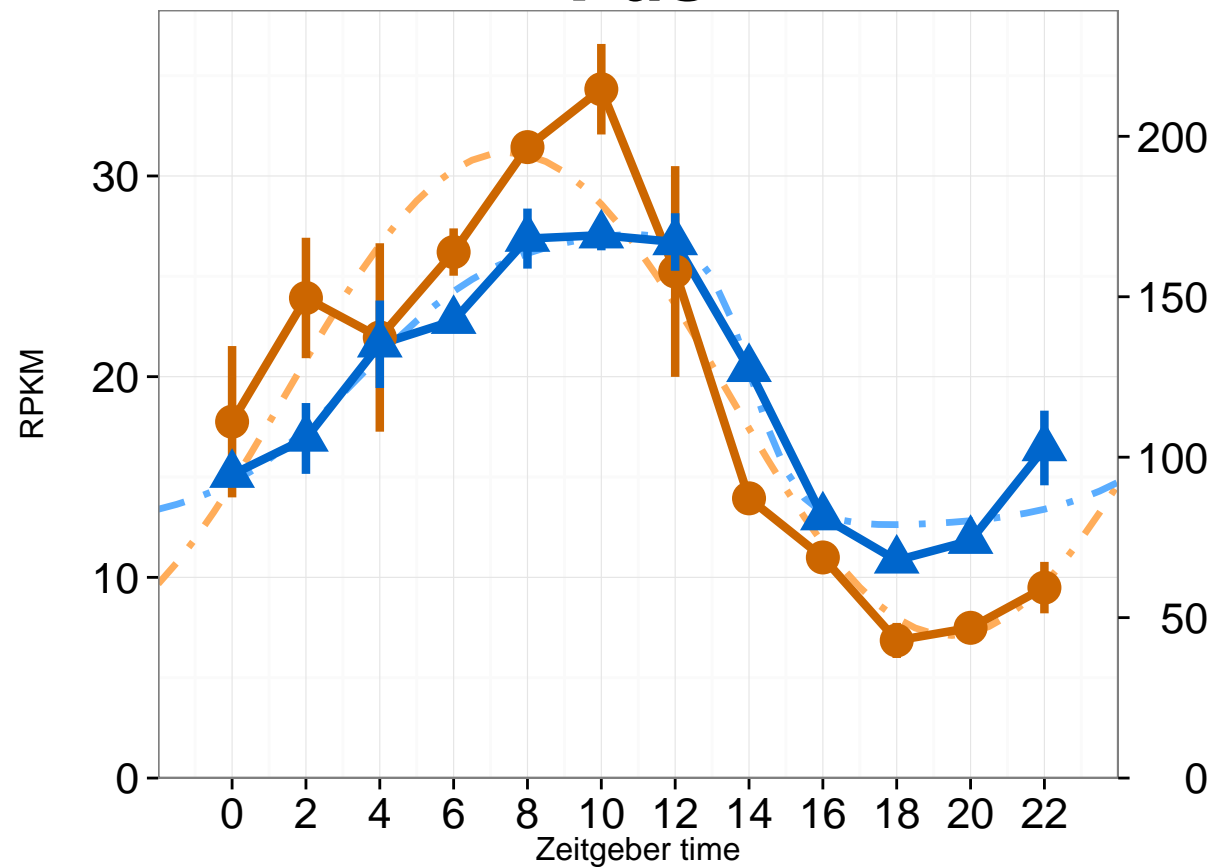

## Fus

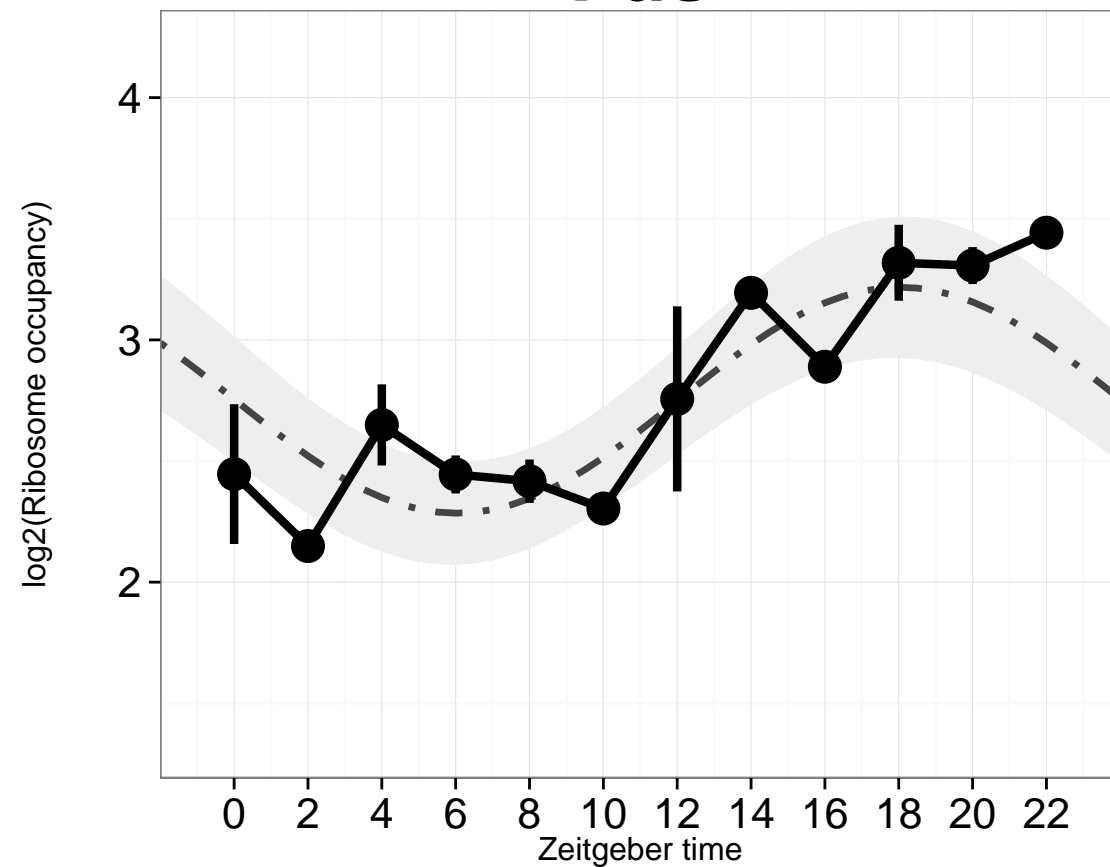

Supplement: Supplementary file 7 — Expression plots for kidney and liver for the 178 common rhythmic genes of Fig. 3c. (ZIP 3338.28 kb) [file 13059_2017_1222_MOESM7_ESM.zip › set_D_shared(178)/Fus_kidney_set_D.pdf]

## Fus

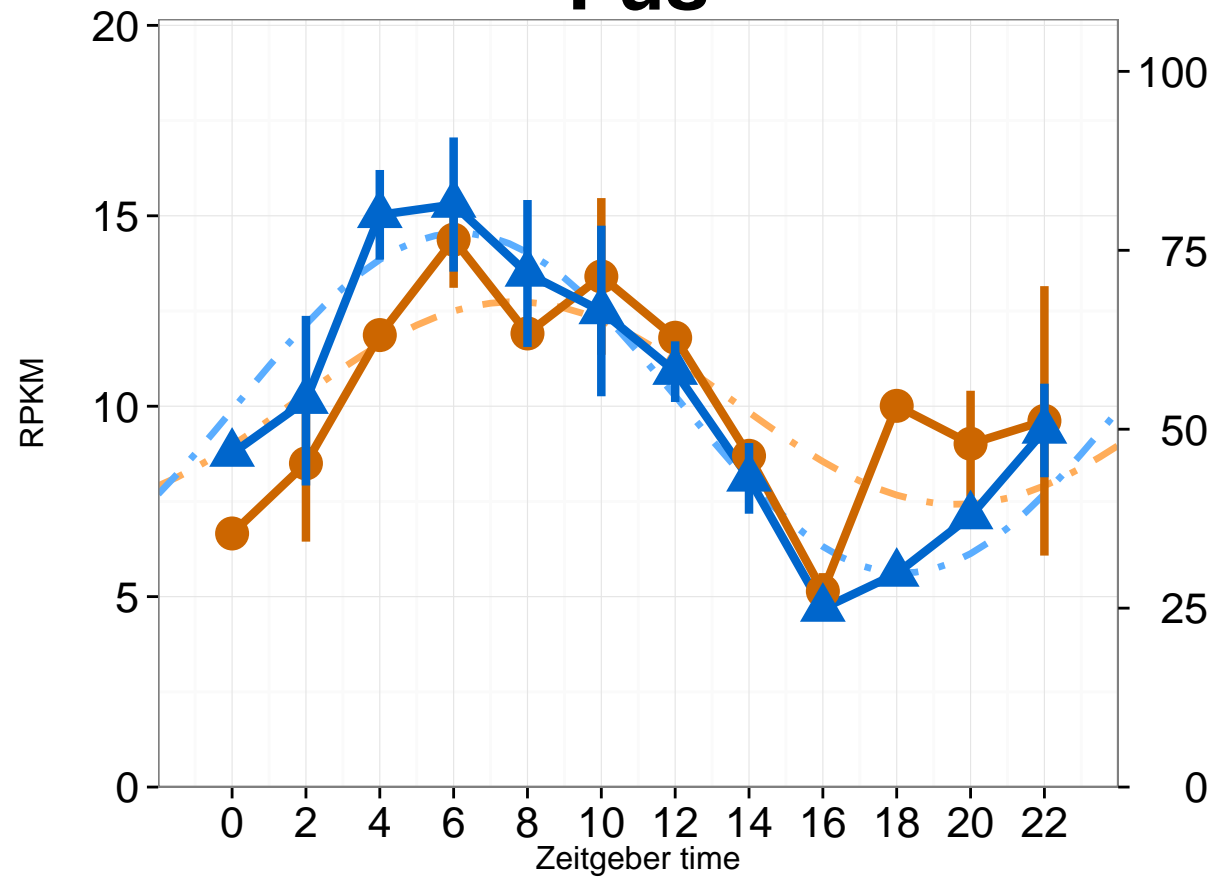

## Fus

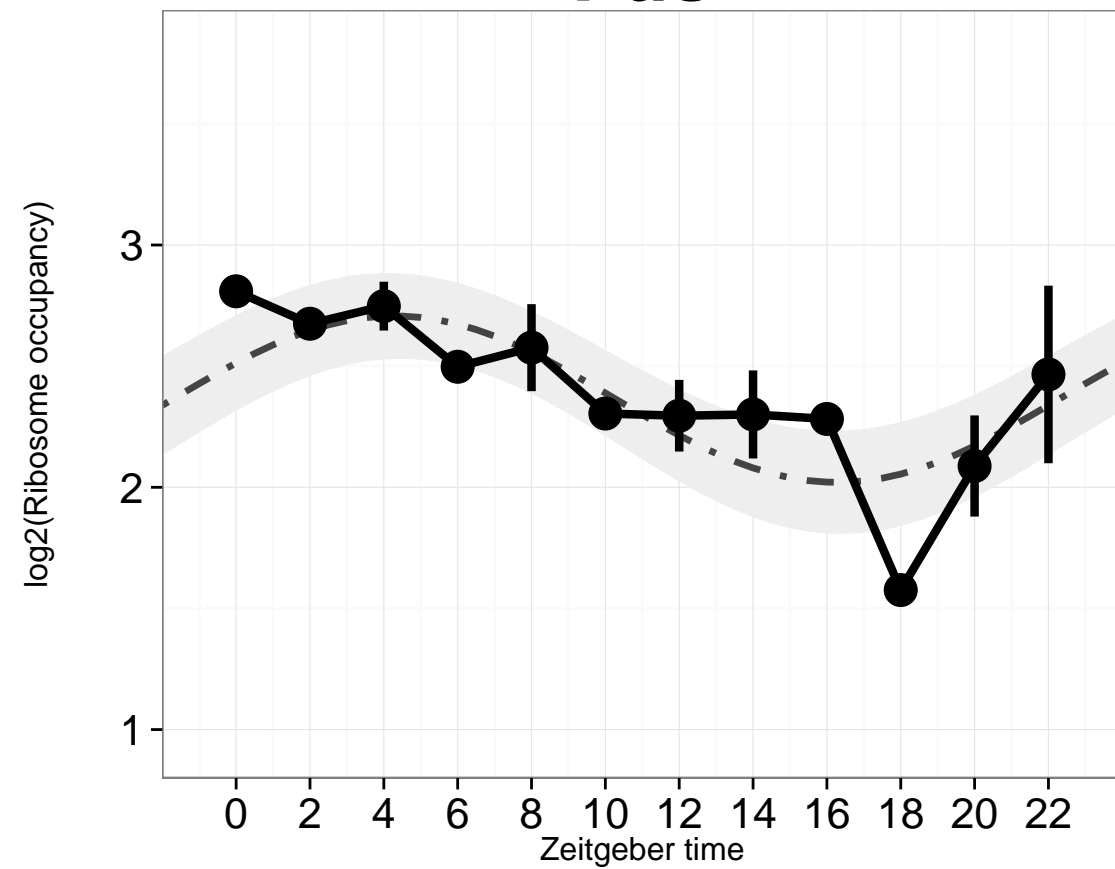

Supplement: Supplementary file 7 — Expression plots for kidney and liver for the 178 common rhythmic genes of Fig. 3c. (ZIP 3338.28 kb) [file 13059_2017_1222_MOESM7_ESM.zip › set_D_shared(178)/Fus_liver_set_D.pdf]

## Gatsl3

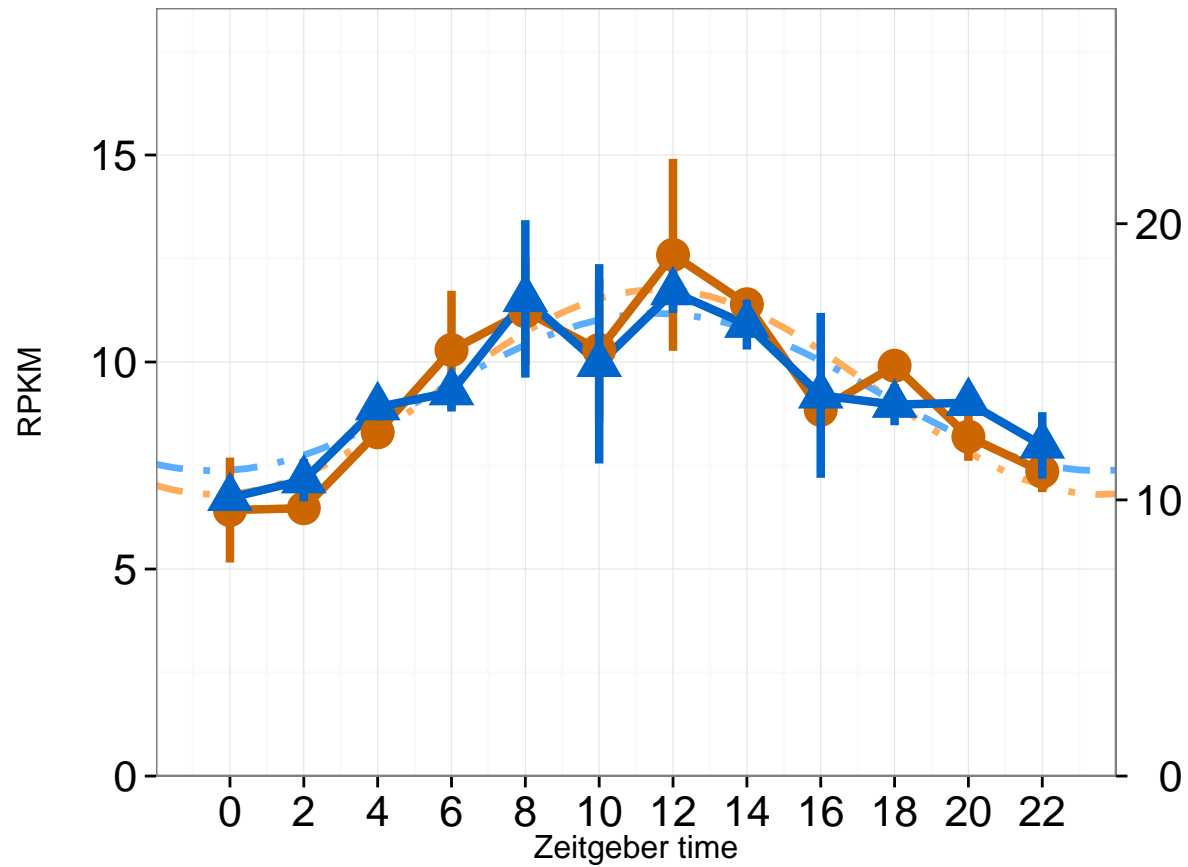

## Gatsl3

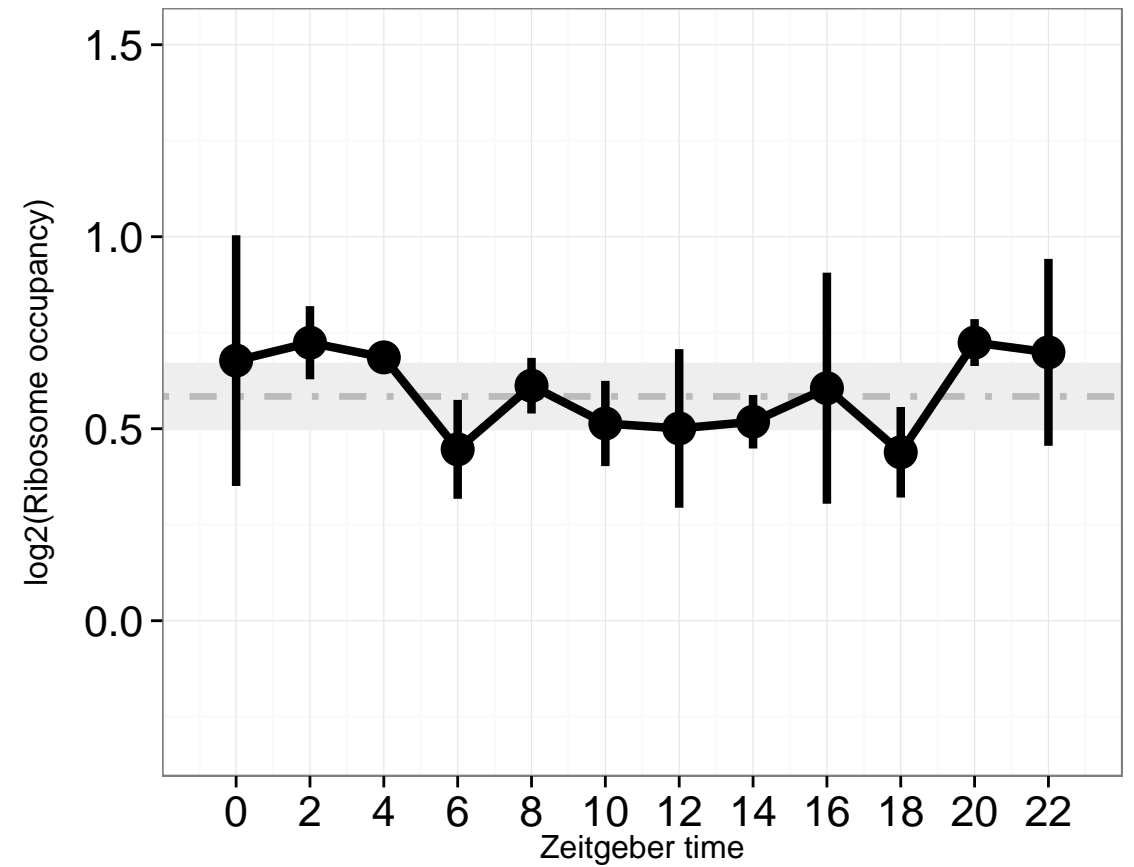

Supplement: Supplementary file 7 — Expression plots for kidney and liver for the 178 common rhythmic genes of Fig. 3c. (ZIP 3338.28 kb) [file 13059_2017_1222_MOESM7_ESM.zip › set_D_shared(178)/Gatsl3_kidney_set_D.pdf]

## Gatsl3

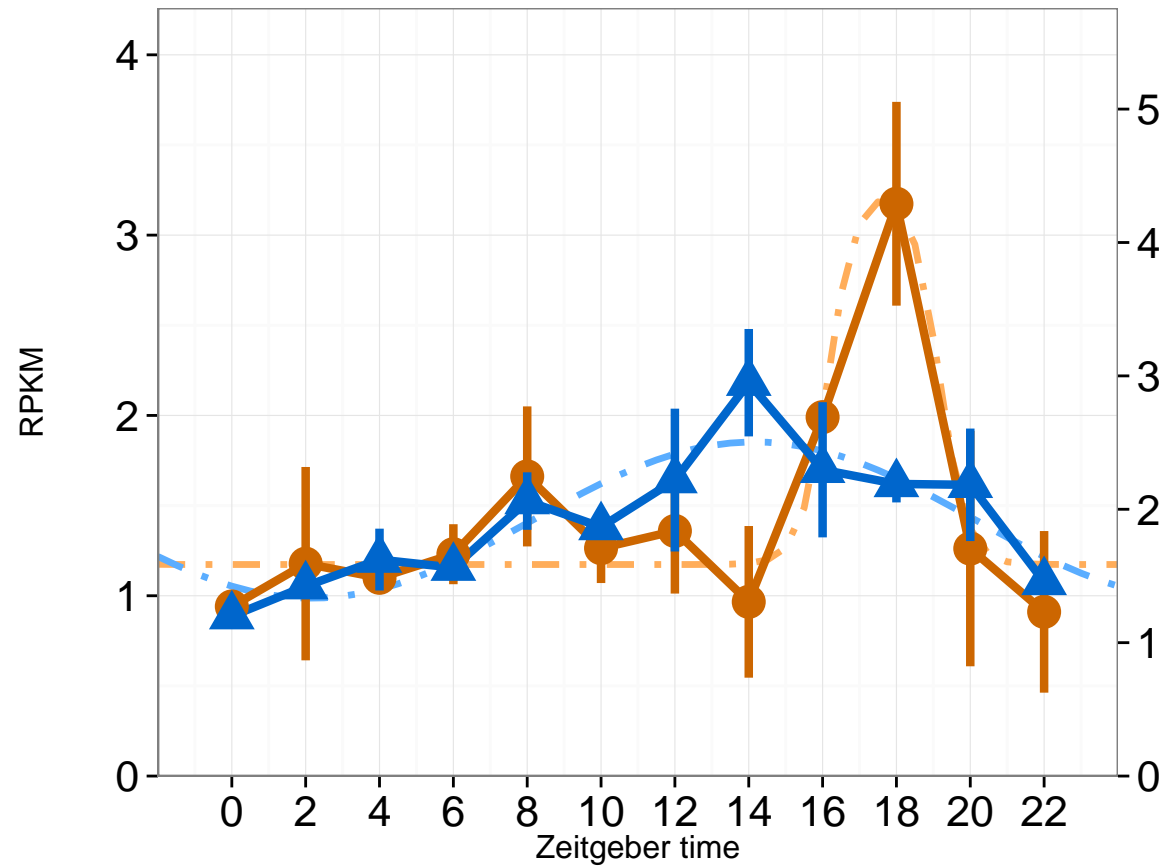

## Gatsl3

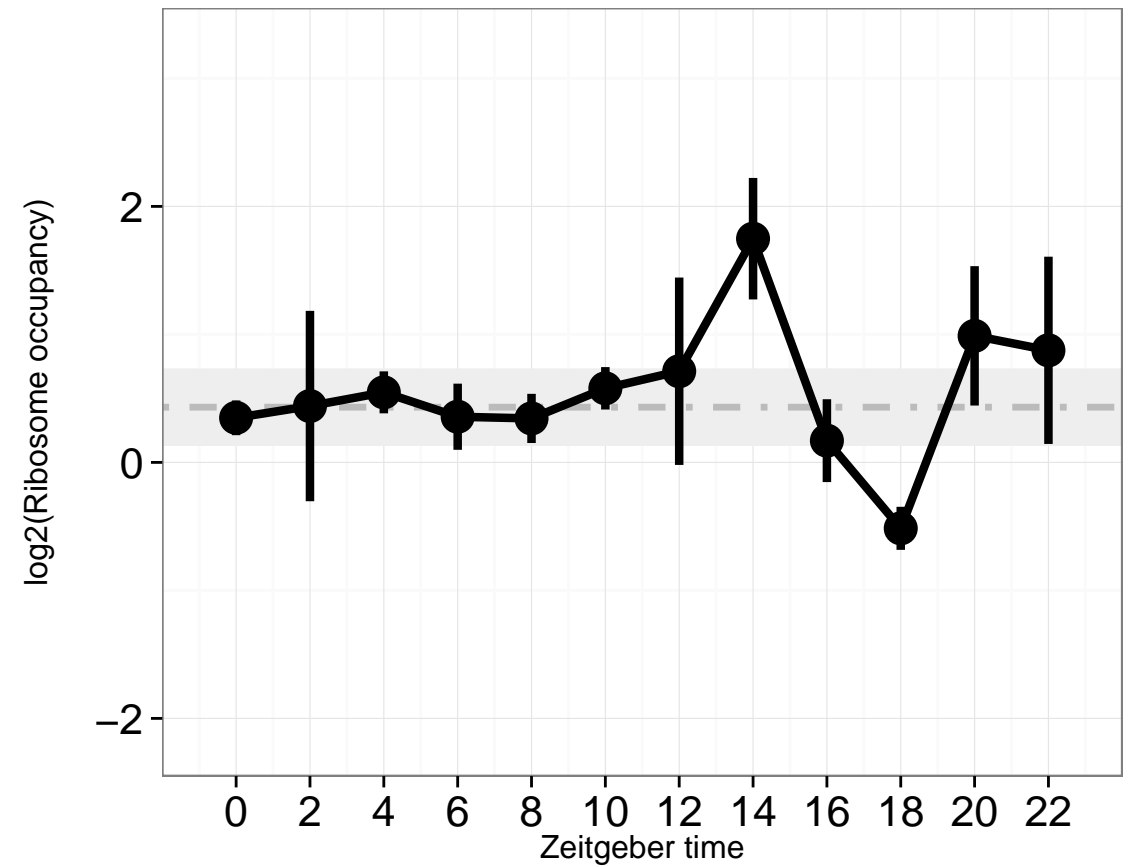

Supplement: Supplementary file 7 — Expression plots for kidney and liver for the 178 common rhythmic genes of Fig. 3c. (ZIP 3338.28 kb) [file 13059_2017_1222_MOESM7_ESM.zip › set_D_shared(178)/Gatsl3_liver_set_D.pdf]

## Ggct

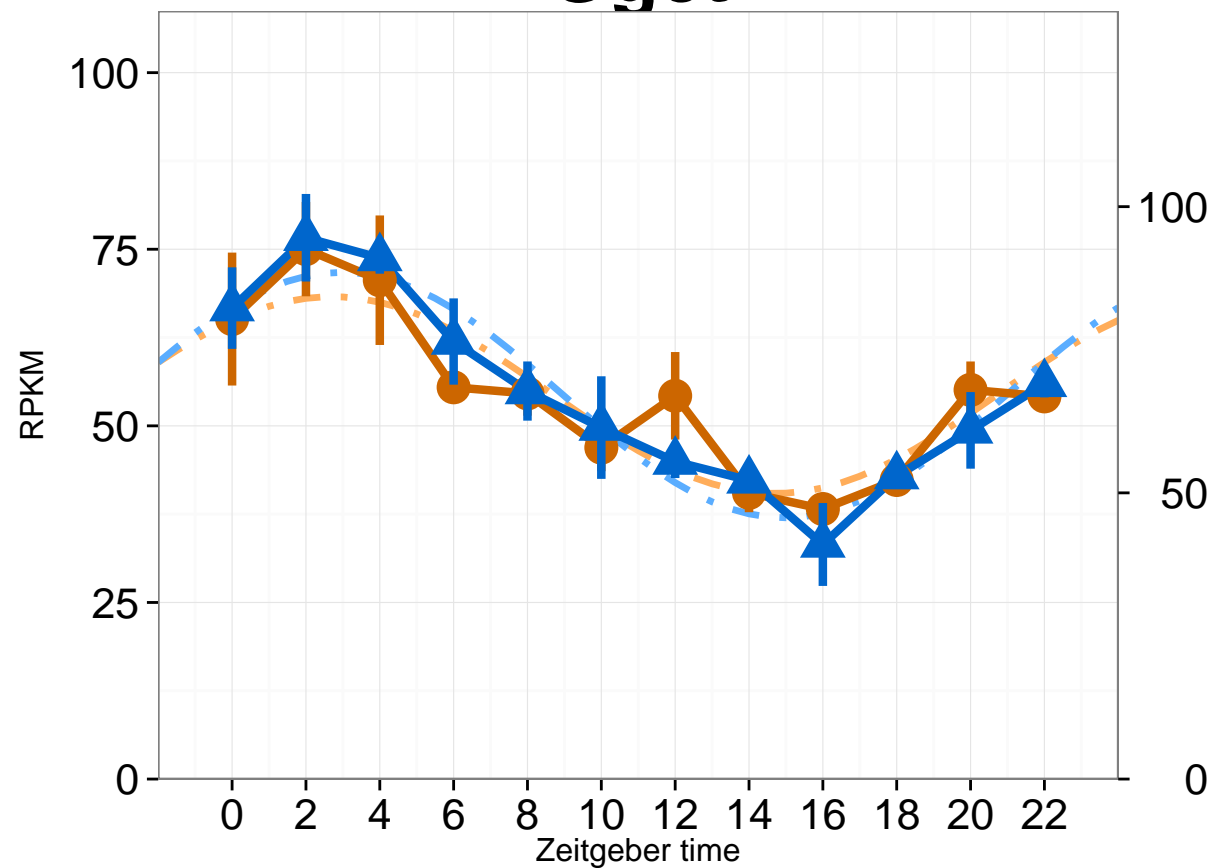

## Ggct

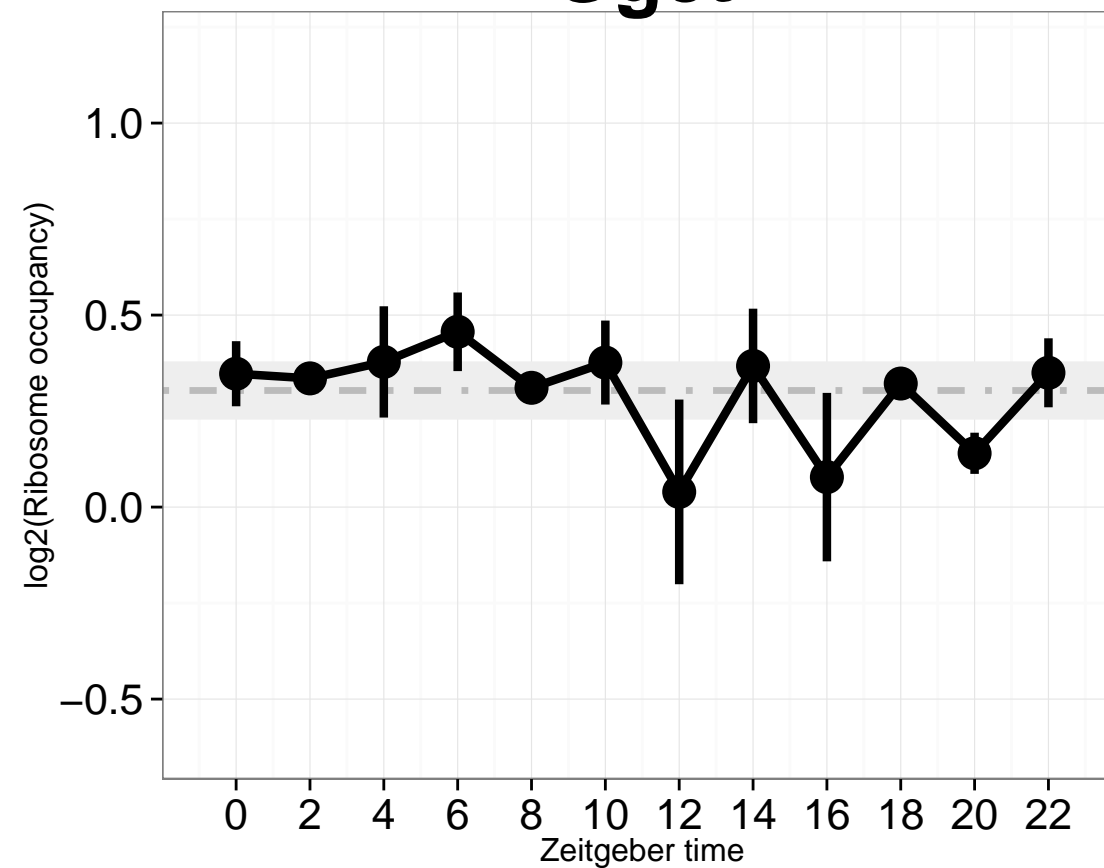

Supplement: Supplementary file 7 — Expression plots for kidney and liver for the 178 common rhythmic genes of Fig. 3c. (ZIP 3338.28 kb) [file 13059_2017_1222_MOESM7_ESM.zip › set_D_shared(178)/Ggct_kidney_set_D.pdf]

## Ggct

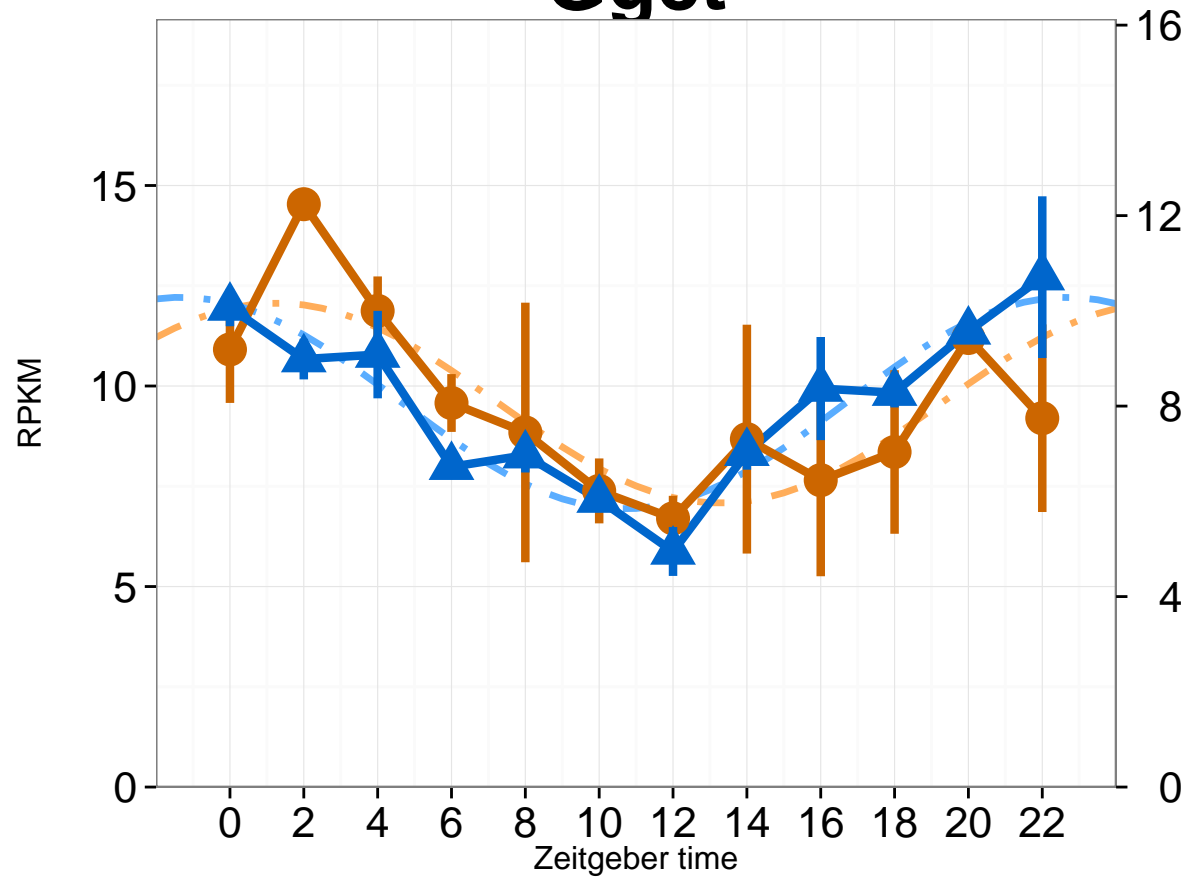

## Ggct

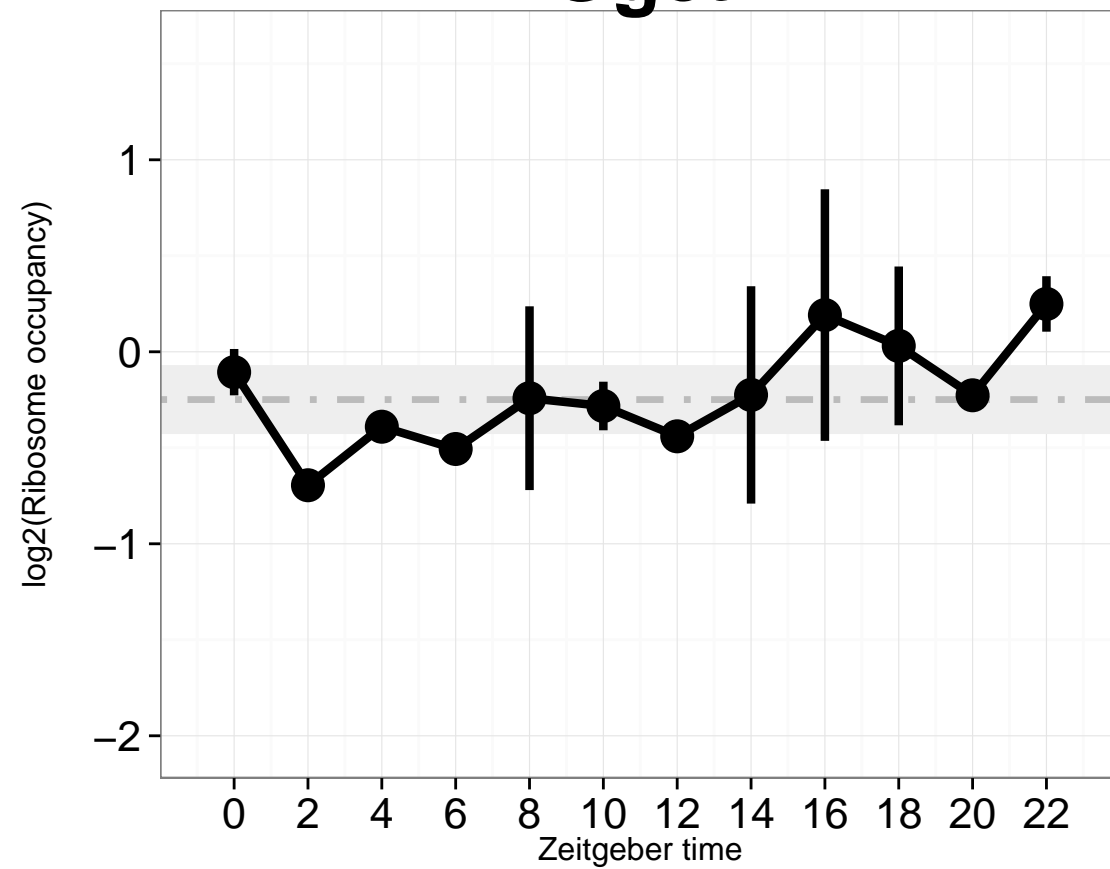

Supplement: Supplementary file 7 — Expression plots for kidney and liver for the 178 common rhythmic genes of Fig. 3c. (ZIP 3338.28 kb) [file 13059_2017_1222_MOESM7_ESM.zip › set_D_shared(178)/Ggct_liver_set_D.pdf]

## Gldc

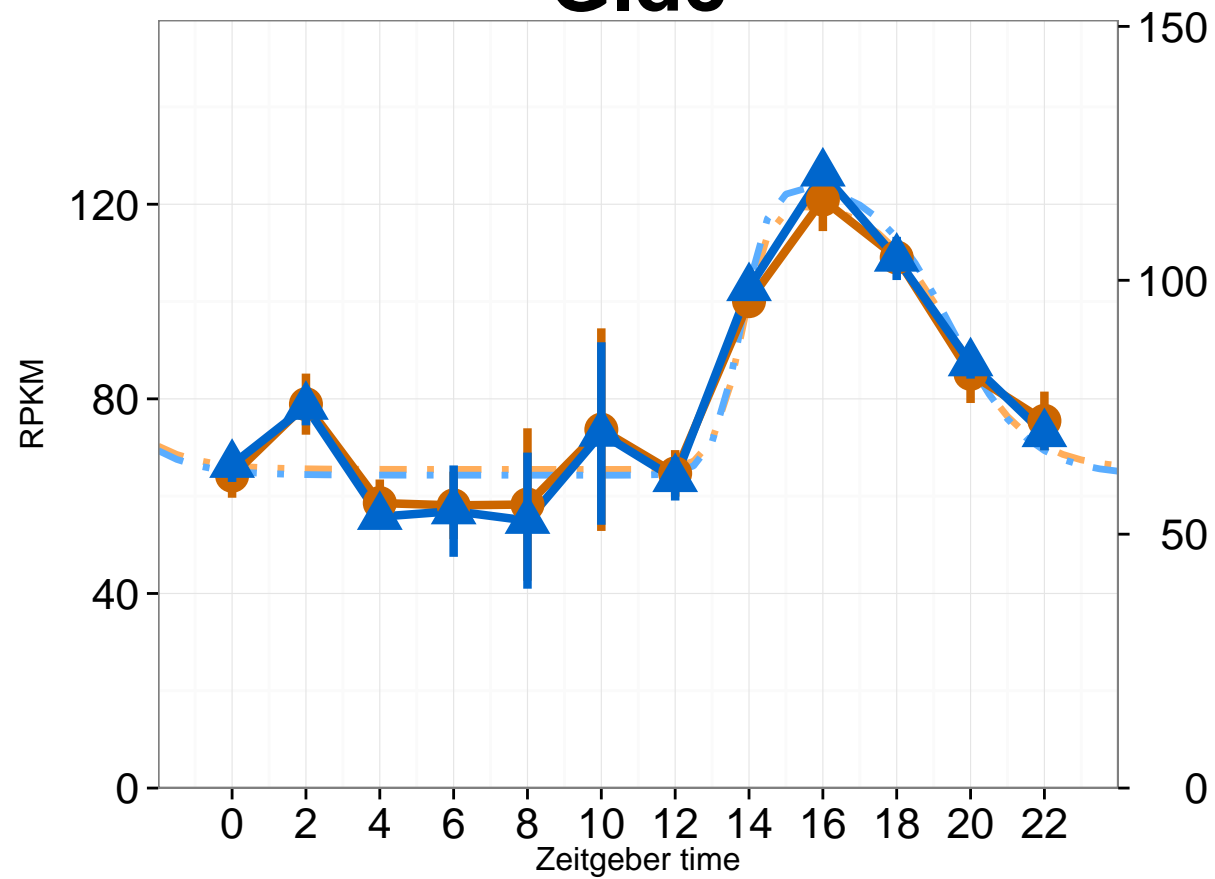

## Gldc

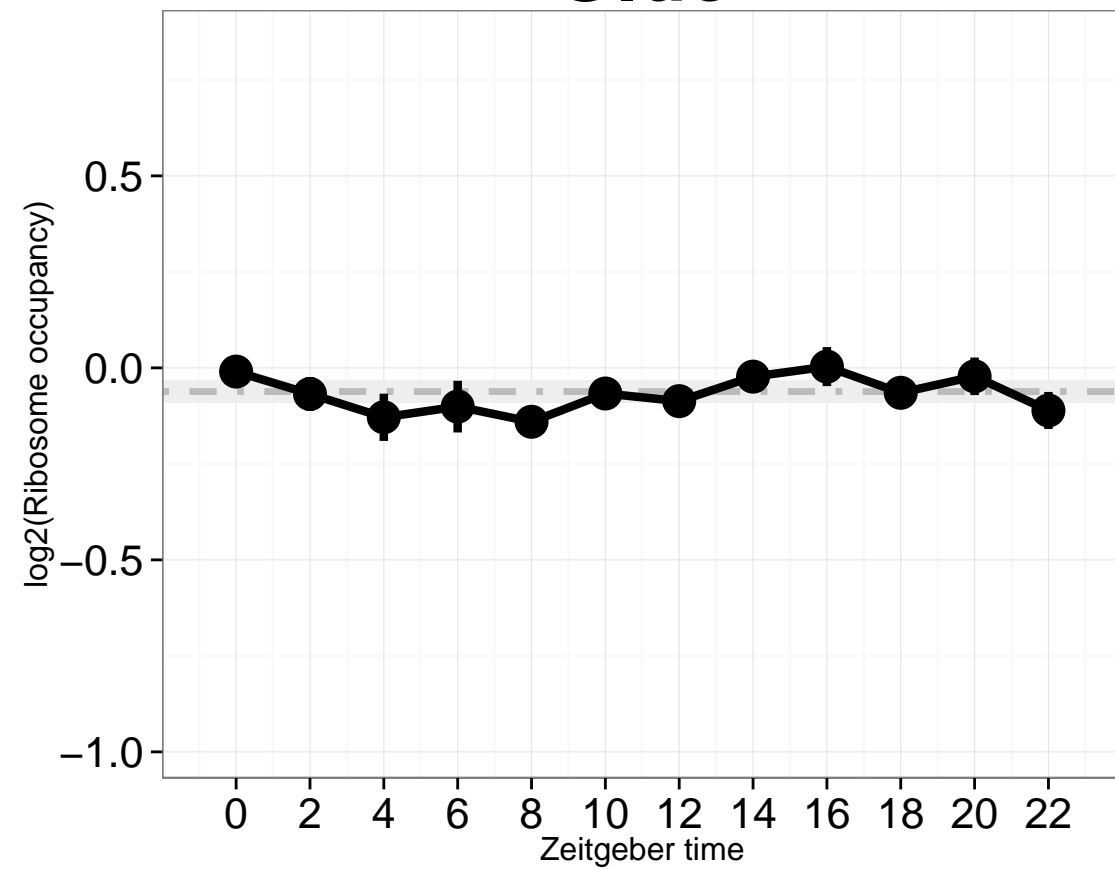

Supplement: Supplementary file 7 — Expression plots for kidney and liver for the 178 common rhythmic genes of Fig. 3c. (ZIP 3338.28 kb) [file 13059_2017_1222_MOESM7_ESM.zip › set_D_shared(178)/Gldc_kidney_set_D.pdf]

## Gldc

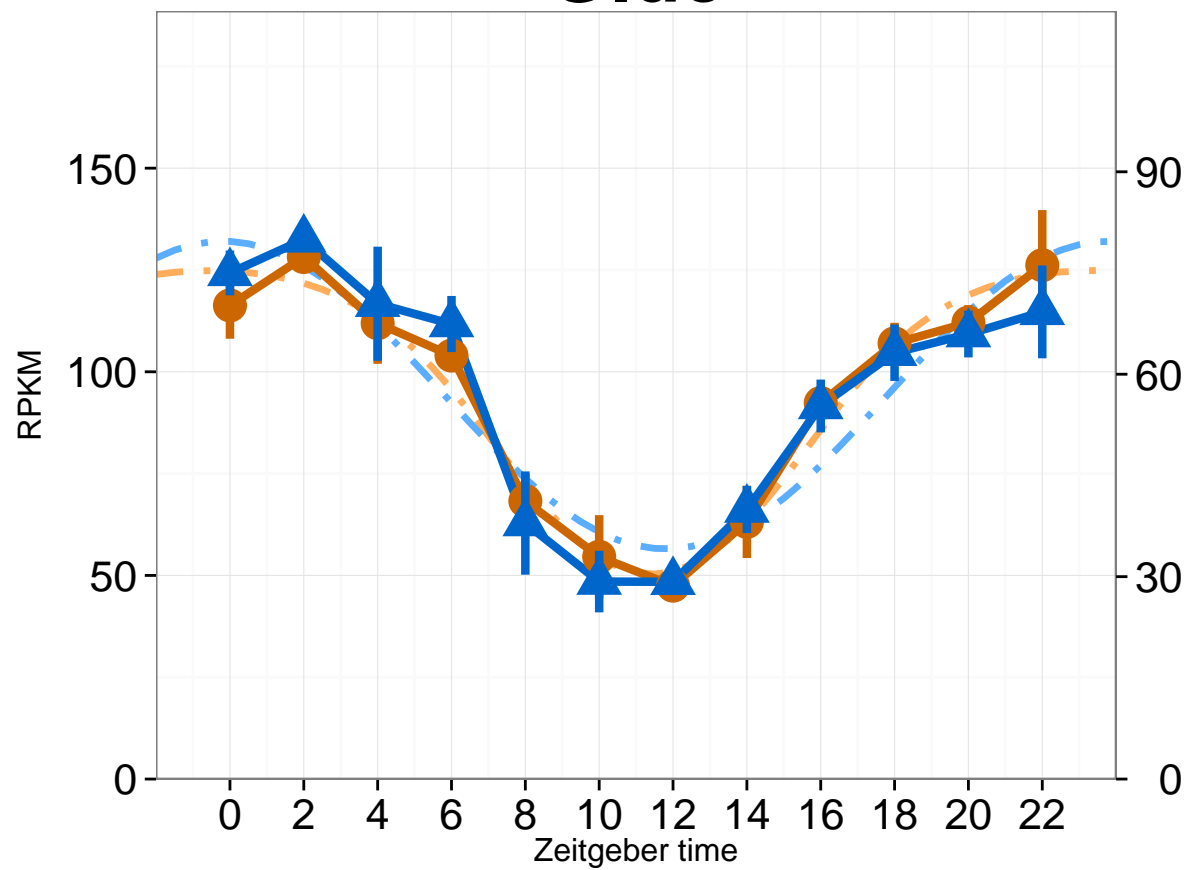

## Gldc

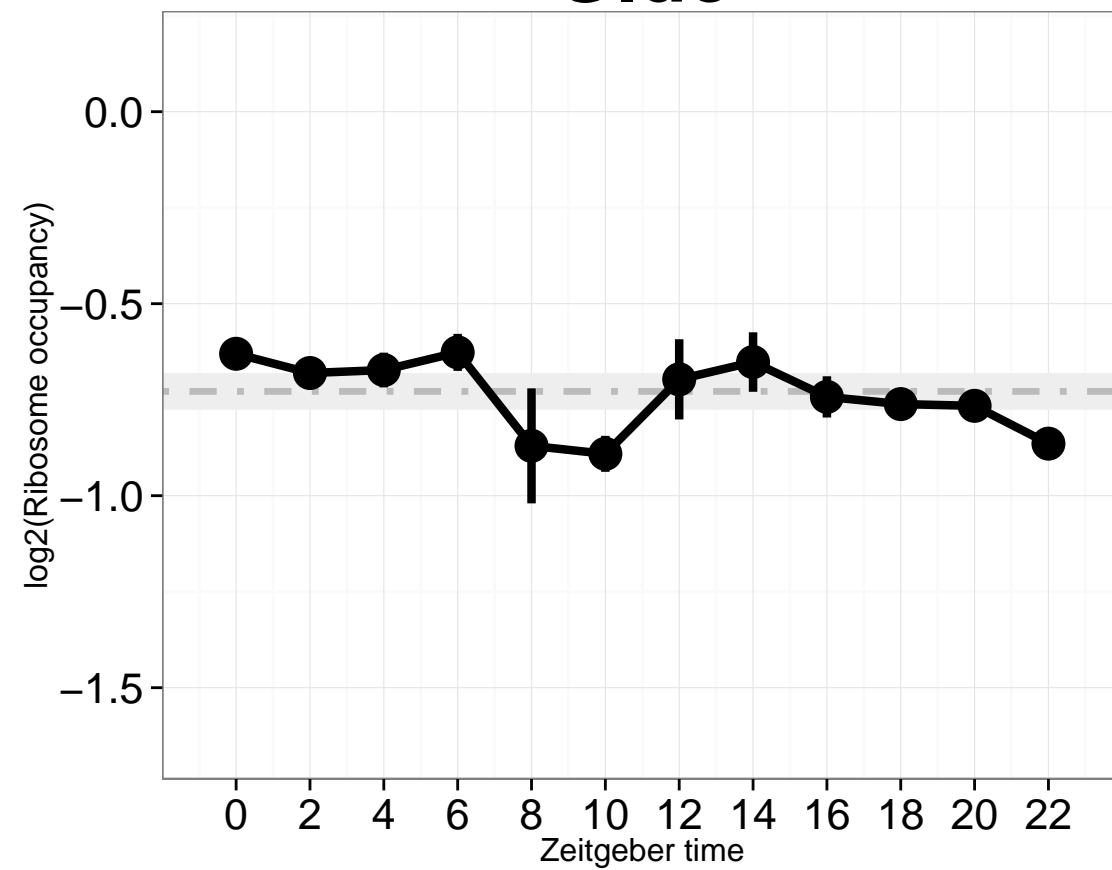

Supplement: Supplementary file 7 — Expression plots for kidney and liver for the 178 common rhythmic genes of Fig. 3c. (ZIP 3338.28 kb) [file 13059_2017_1222_MOESM7_ESM.zip › set_D_shared(178)/Gldc_liver_set_D.pdf]

# Gm129

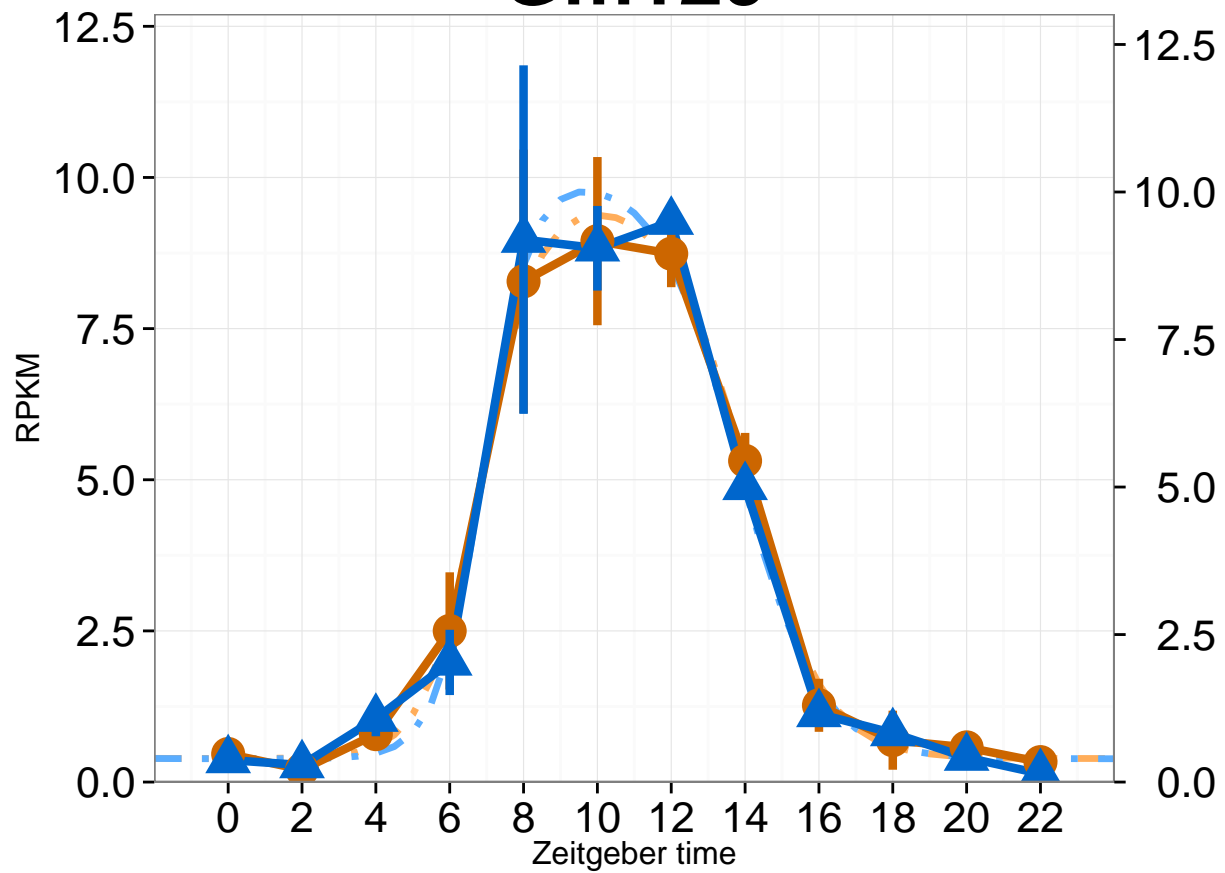

# Gm129

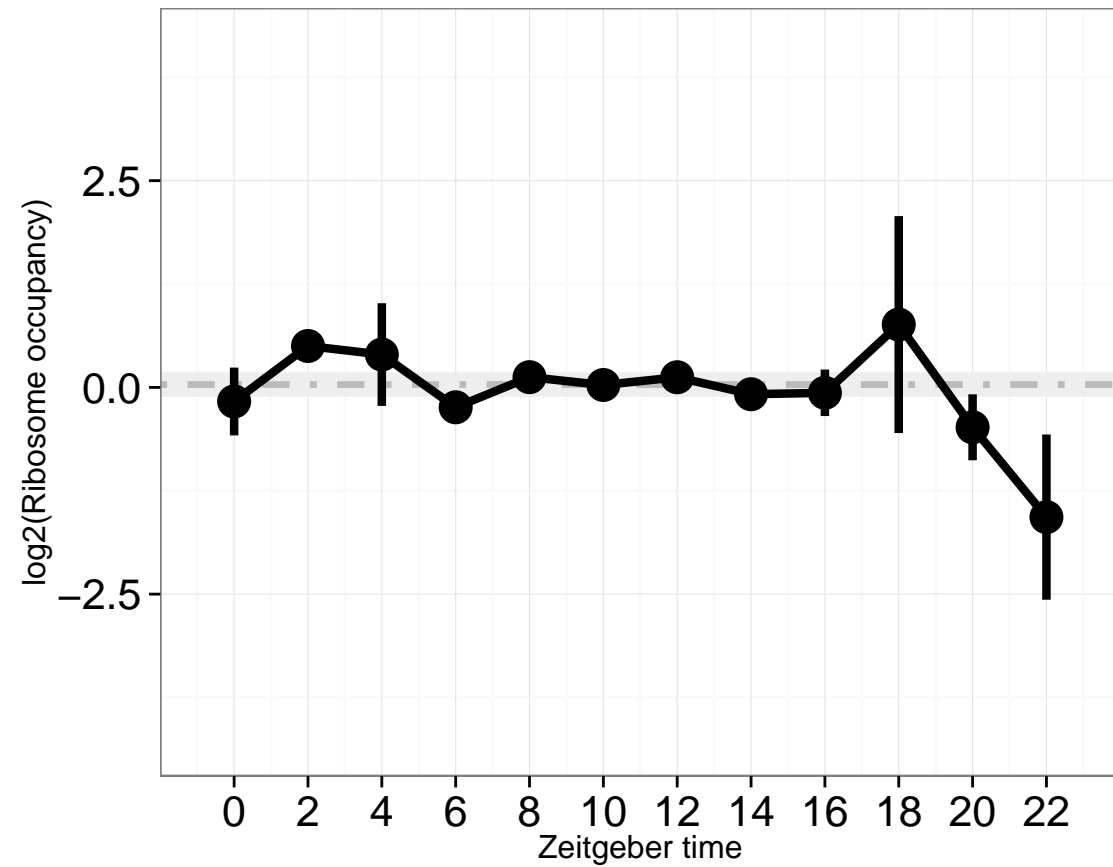

Supplement: Supplementary file 7 — Expression plots for kidney and liver for the 178 common rhythmic genes of Fig. 3c. (ZIP 3338.28 kb) [file 13059_2017_1222_MOESM7_ESM.zip › set_D_shared(178)/Gm129_kidney_set_D.pdf]

# Gm129

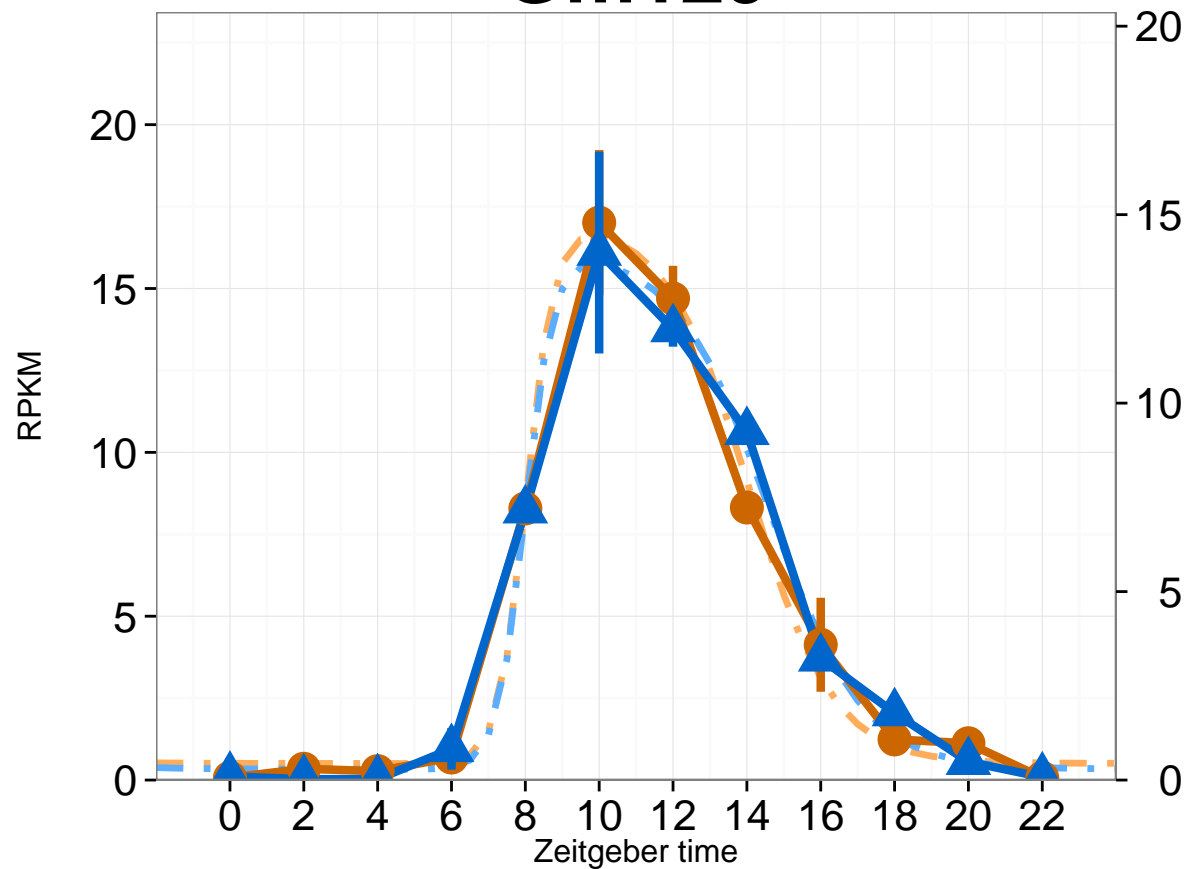

# Gm129

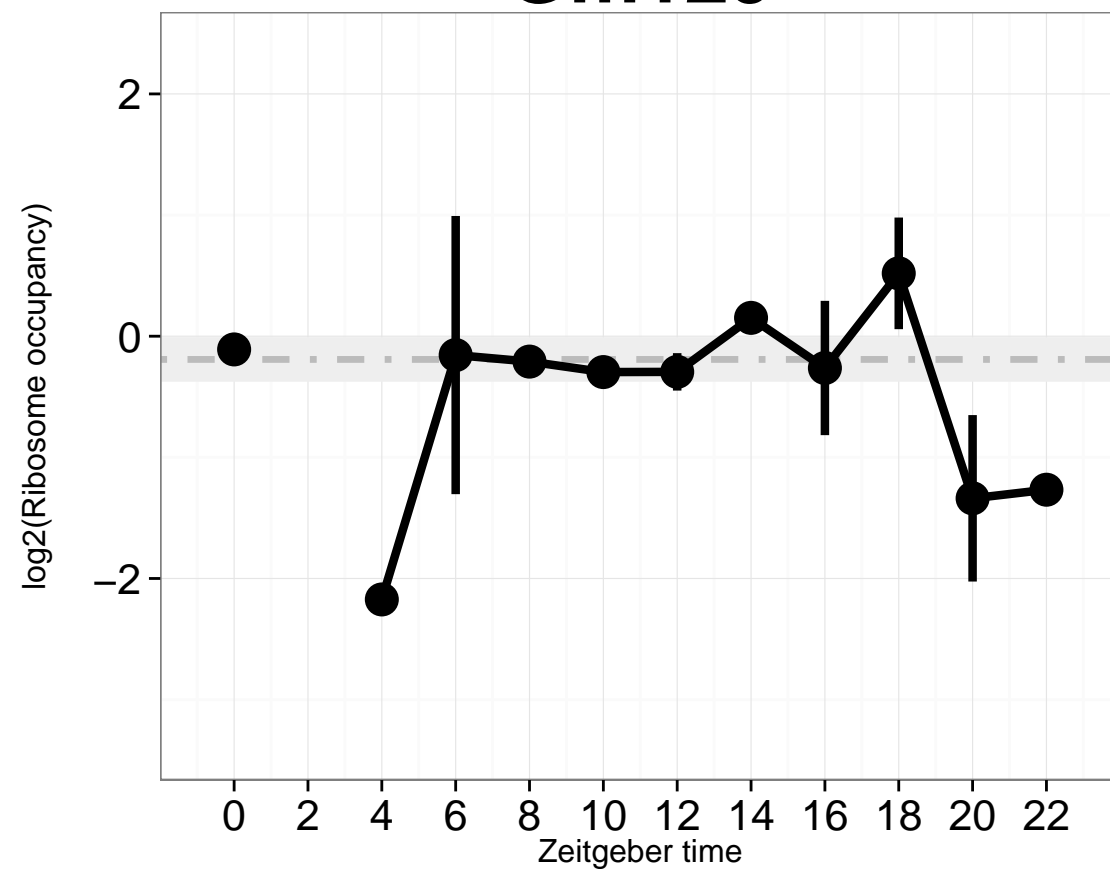

Supplement: Supplementary file 7 — Expression plots for kidney and liver for the 178 common rhythmic genes of Fig. 3c. (ZIP 3338.28 kb) [file 13059_2017_1222_MOESM7_ESM.zip › set_D_shared(178)/Gm129_liver_set_D.pdf]

## Gm4952

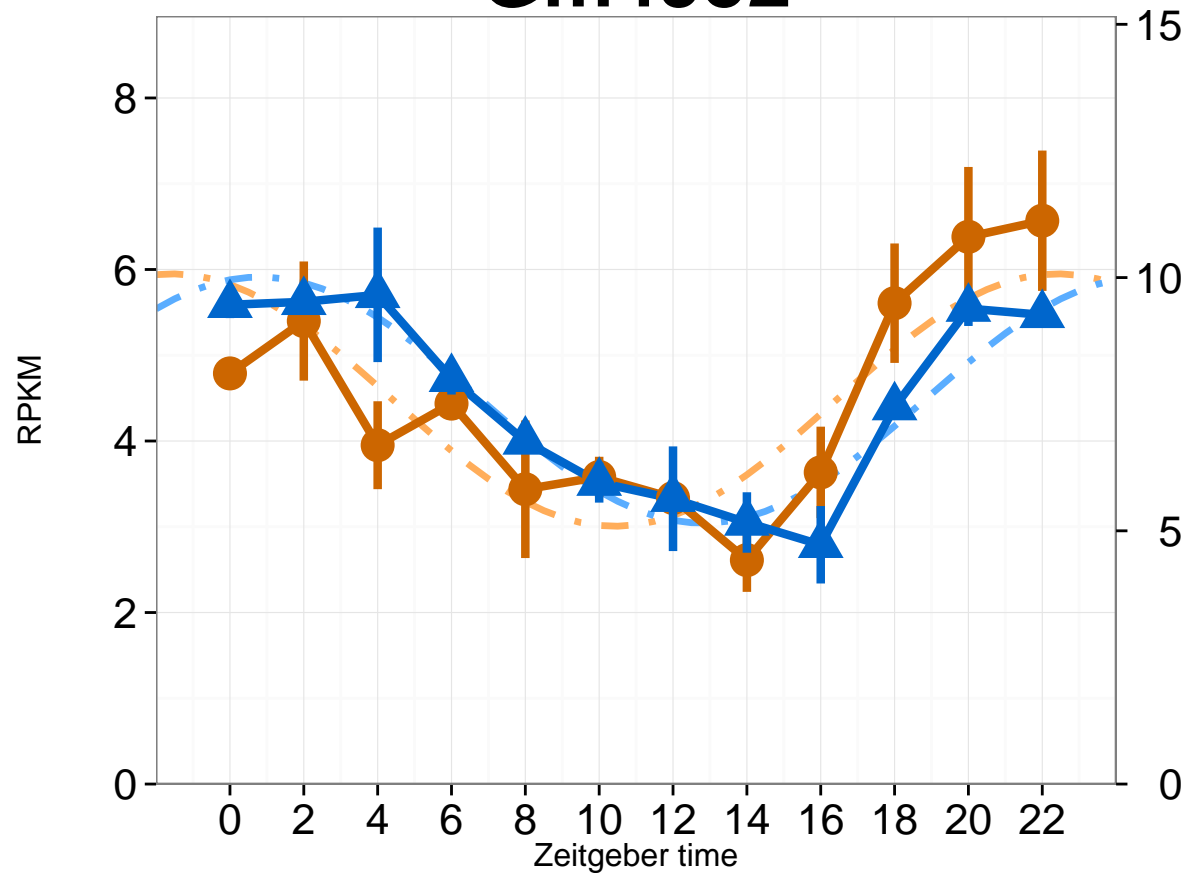

## Gm4952

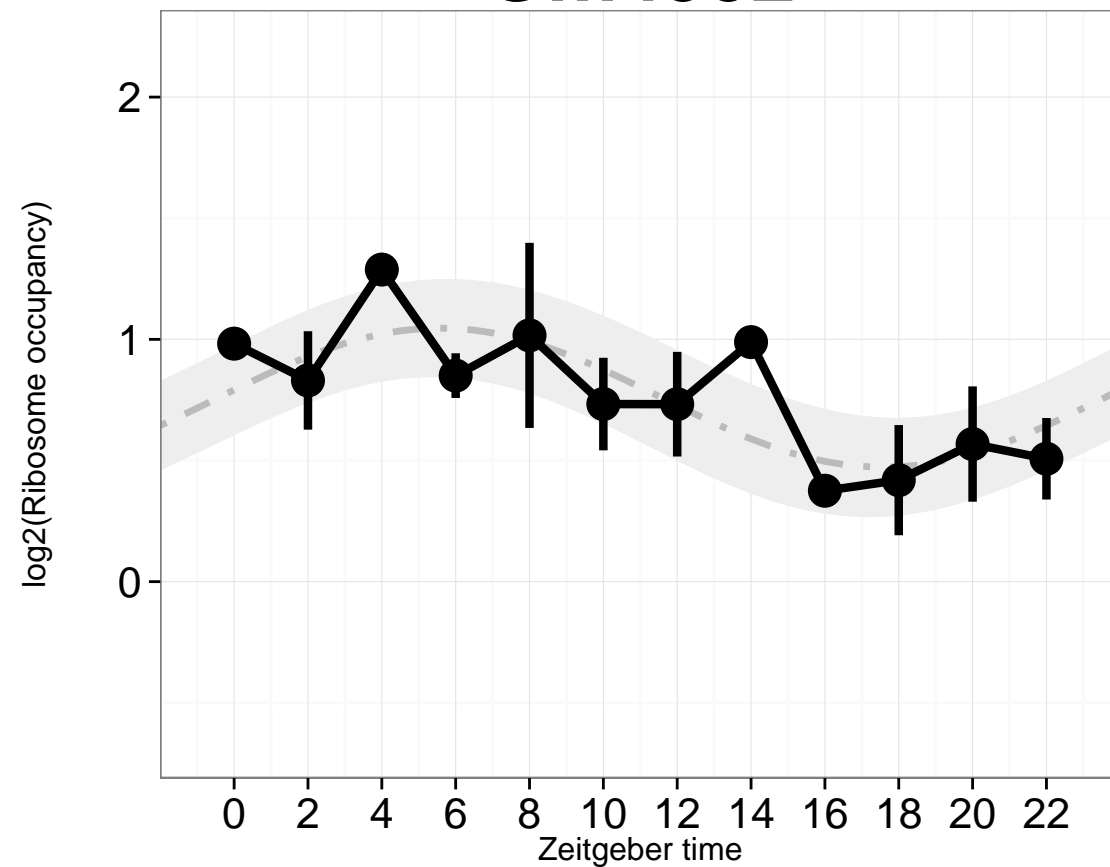

Supplement: Supplementary file 7 — Expression plots for kidney and liver for the 178 common rhythmic genes of Fig. 3c. (ZIP 3338.28 kb) [file 13059_2017_1222_MOESM7_ESM.zip › set_D_shared(178)/Gm4952_kidney_set_D.pdf]

# Gm4952

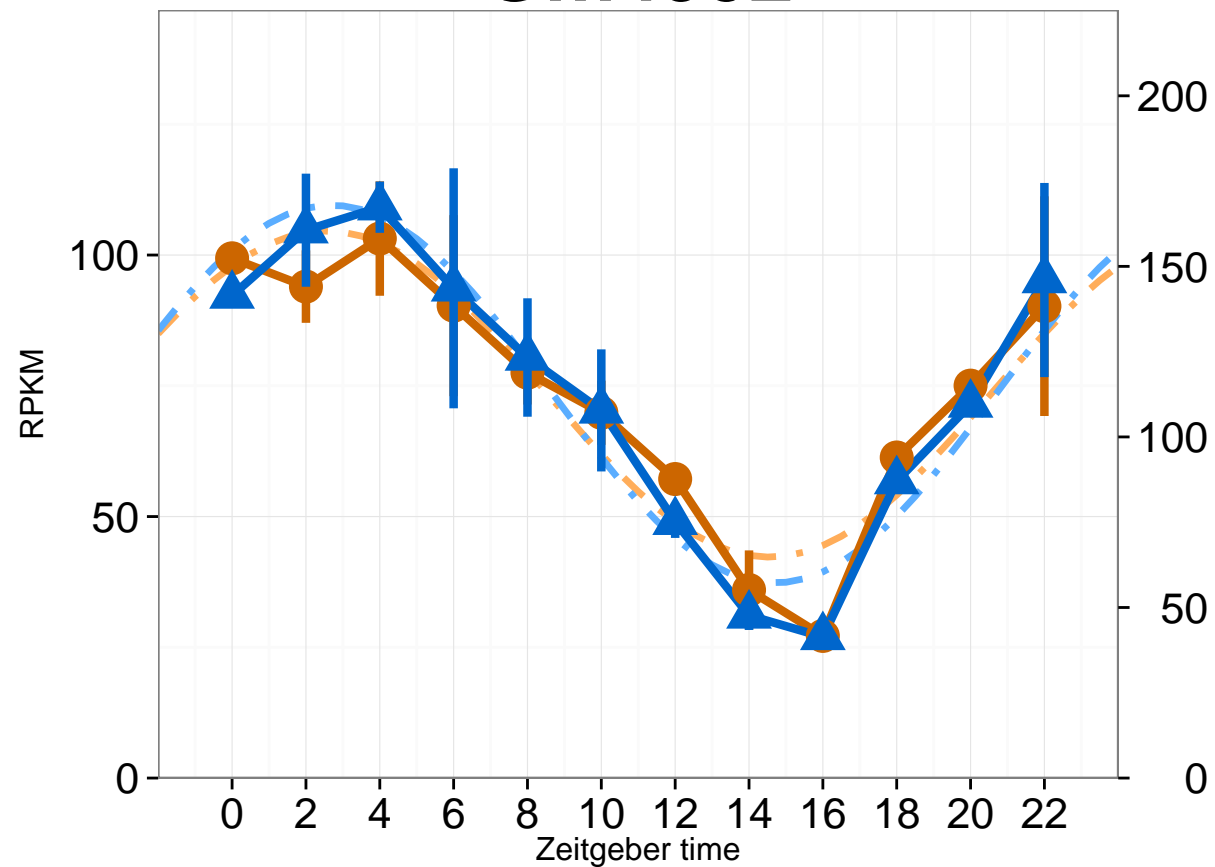

# Gm4952

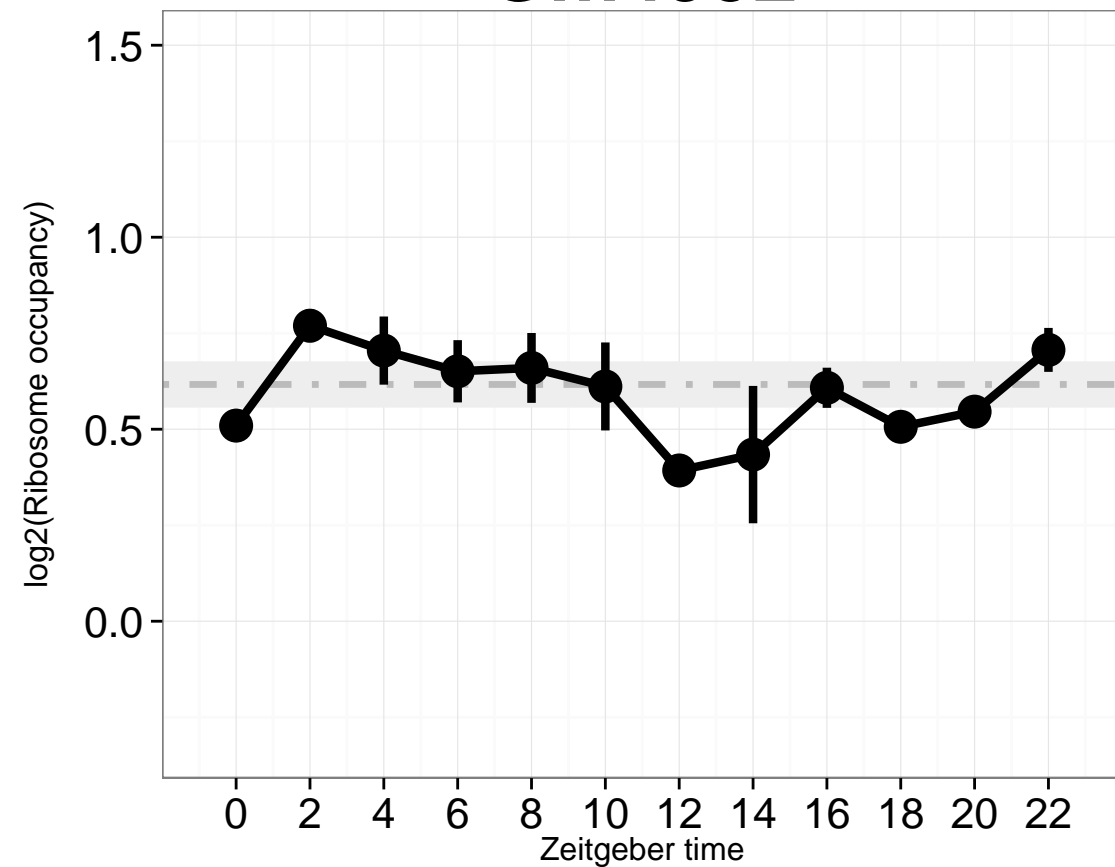

Supplement: Supplementary file 7 — Expression plots for kidney and liver for the 178 common rhythmic genes of Fig. 3c. (ZIP 3338.28 kb) [file 13059_2017_1222_MOESM7_ESM.zip › set_D_shared(178)/Gm4952_liver_set_D.pdf]

## Gpcpd1

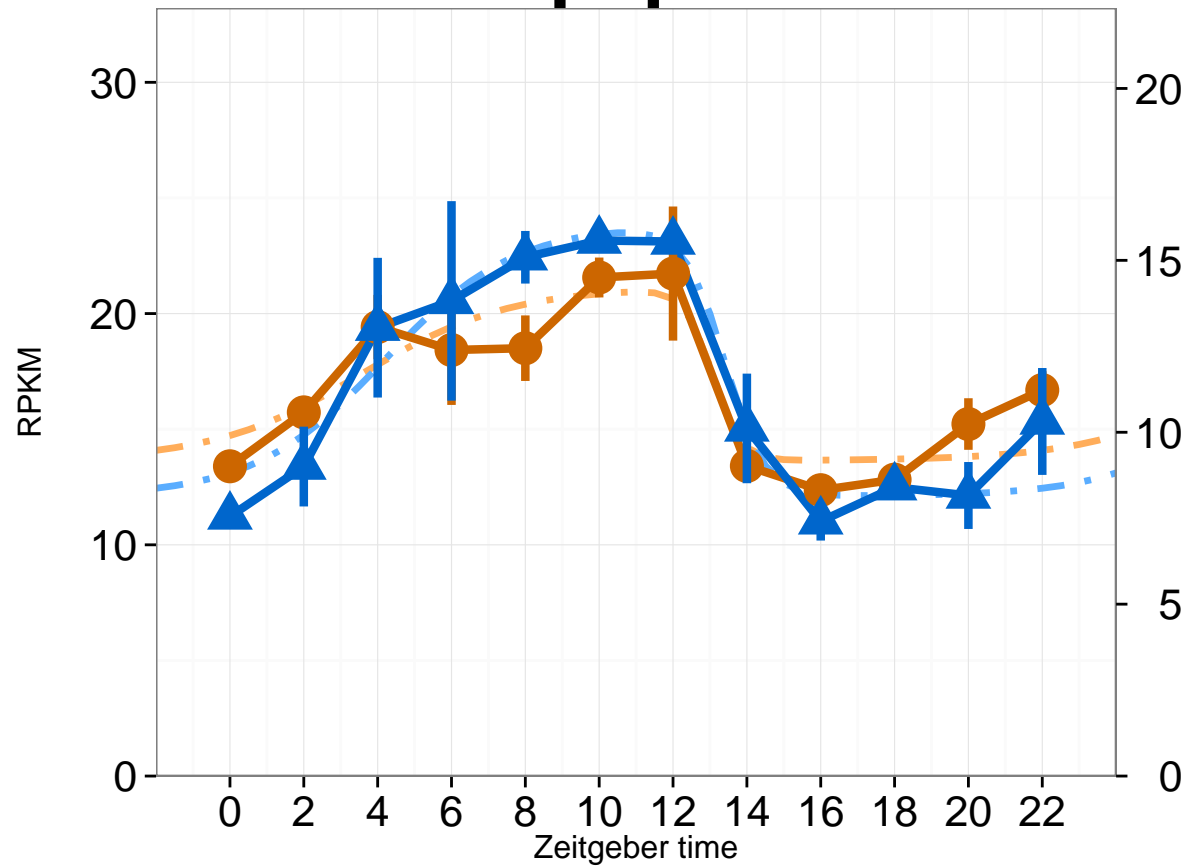

## Gpcpd1

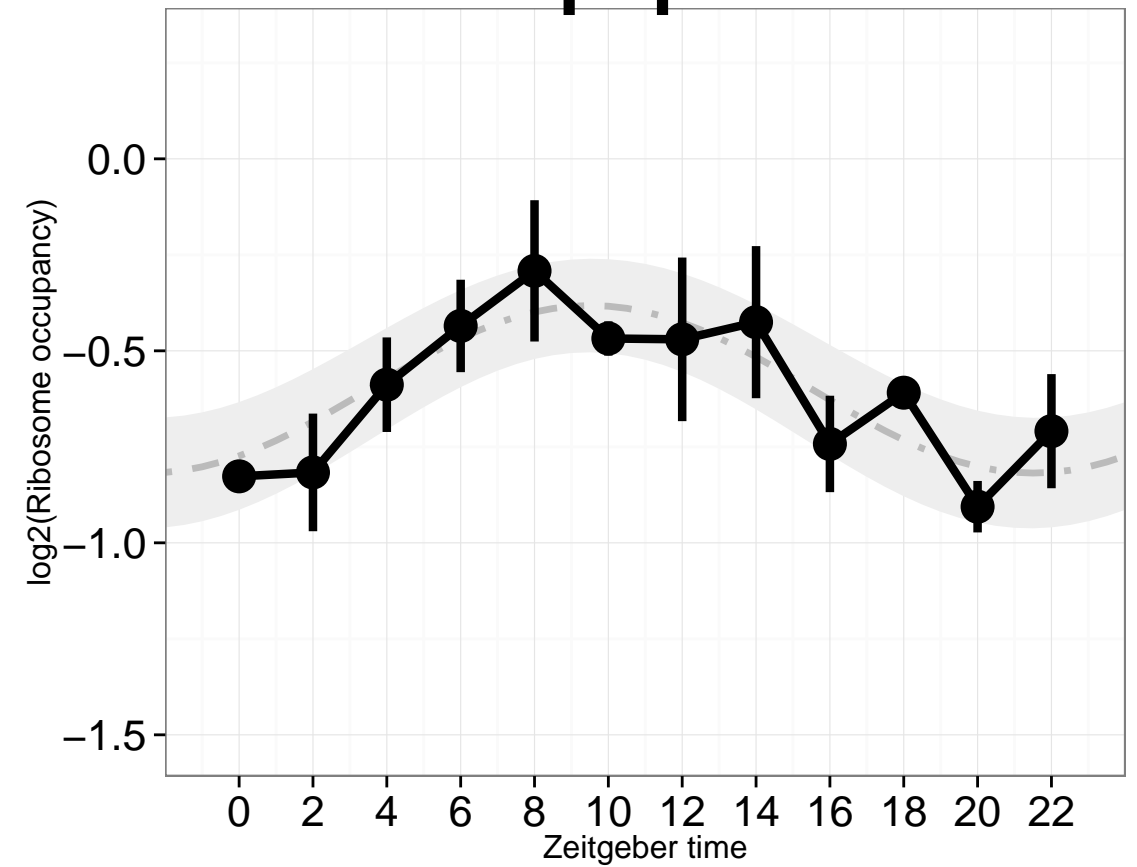

Supplement: Supplementary file 7 — Expression plots for kidney and liver for the 178 common rhythmic genes of Fig. 3c. (ZIP 3338.28 kb) [file 13059_2017_1222_MOESM7_ESM.zip › set_D_shared(178)/Gpcpd1_kidney_set_D.pdf]

# Gpcpd1

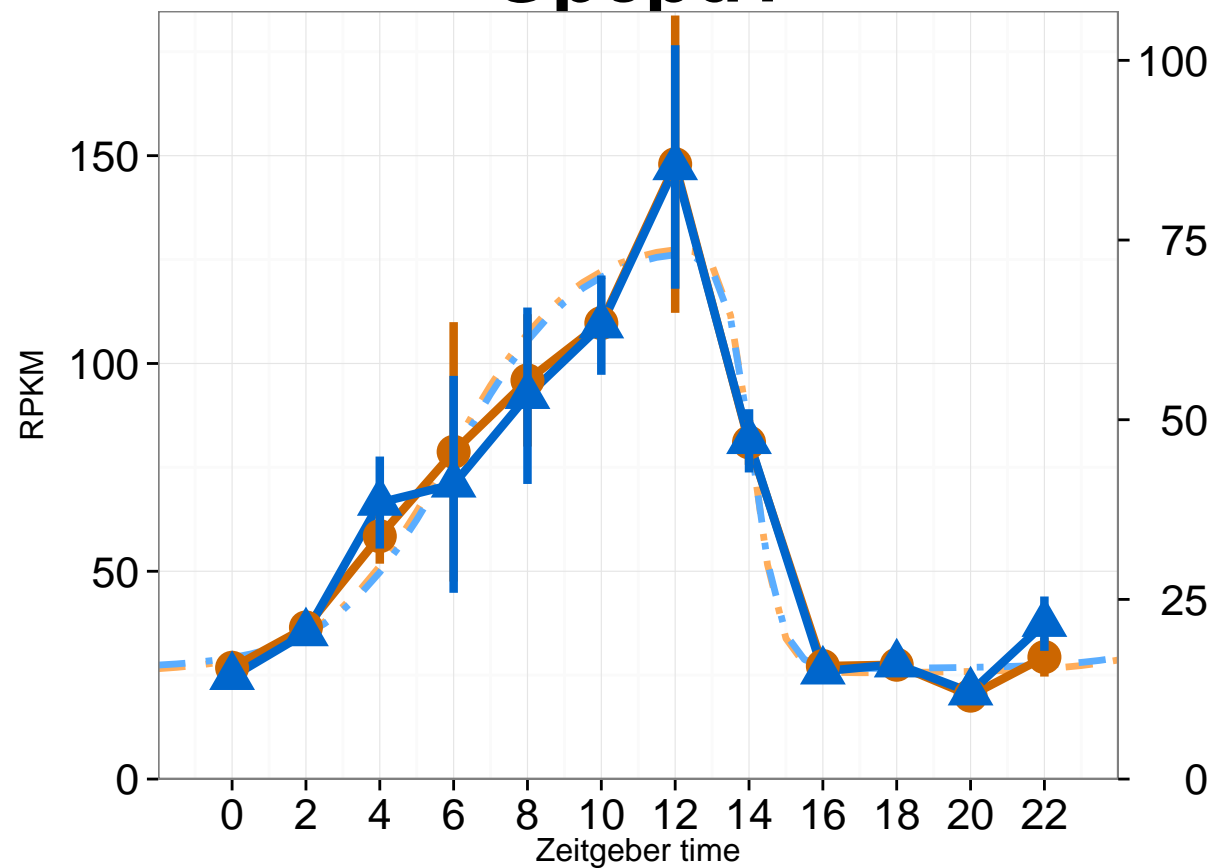

# Gpcpd1

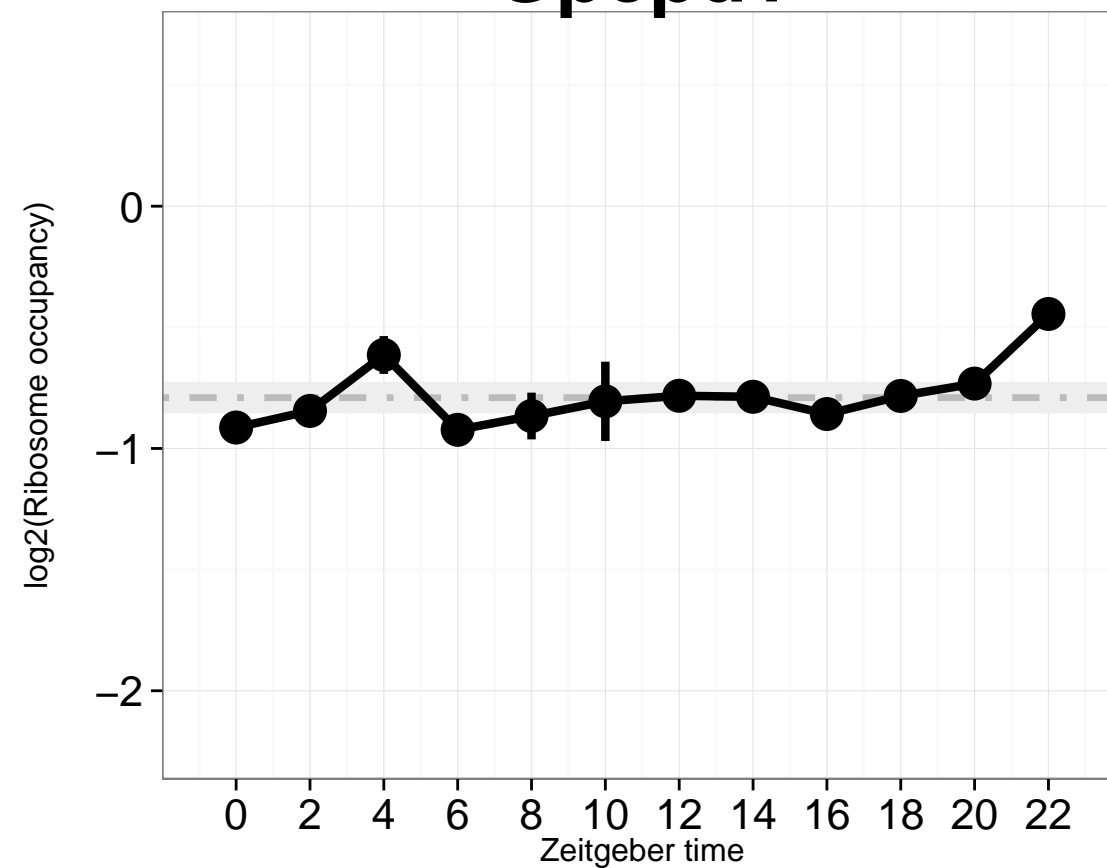

Supplement: Supplementary file 7 — Expression plots for kidney and liver for the 178 common rhythmic genes of Fig. 3c. (ZIP 3338.28 kb) [file 13059_2017_1222_MOESM7_ESM.zip › set_D_shared(178)/Gpcpd1_liver_set_D.pdf]

# Gprin3

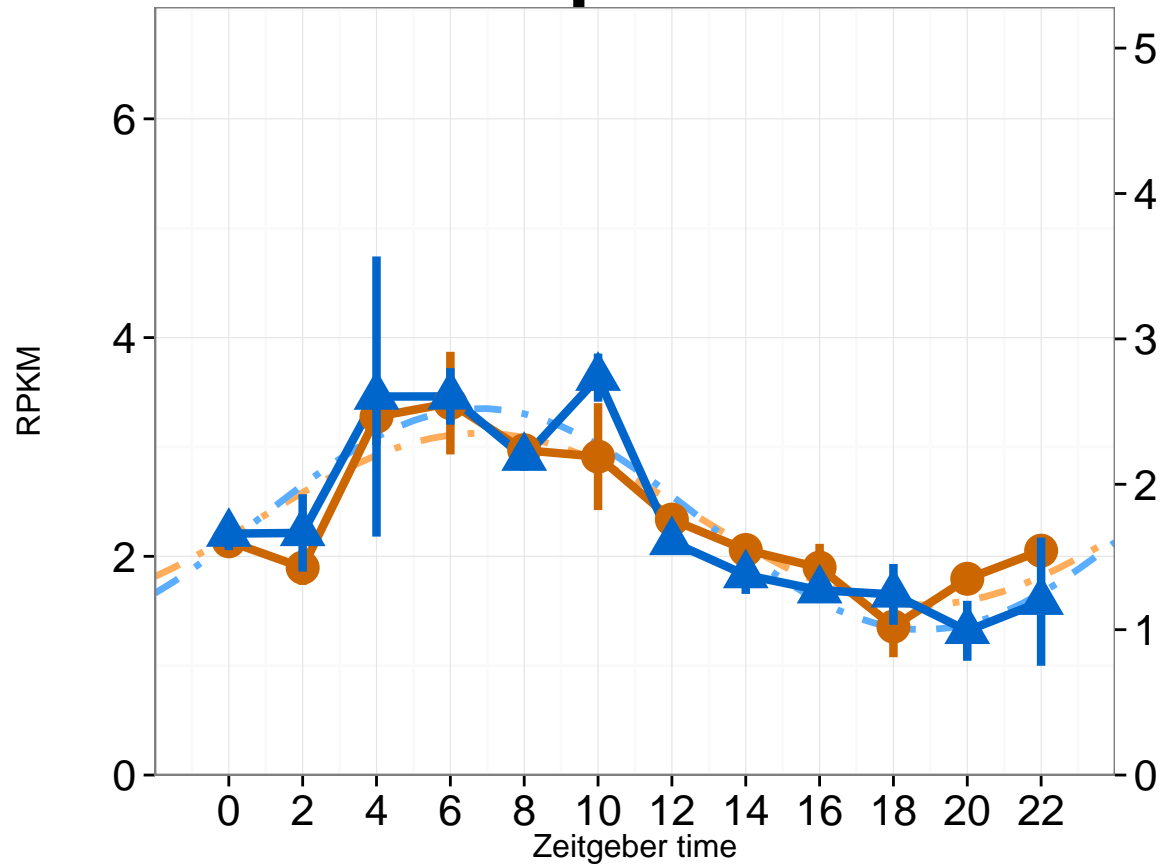

# Gprin3

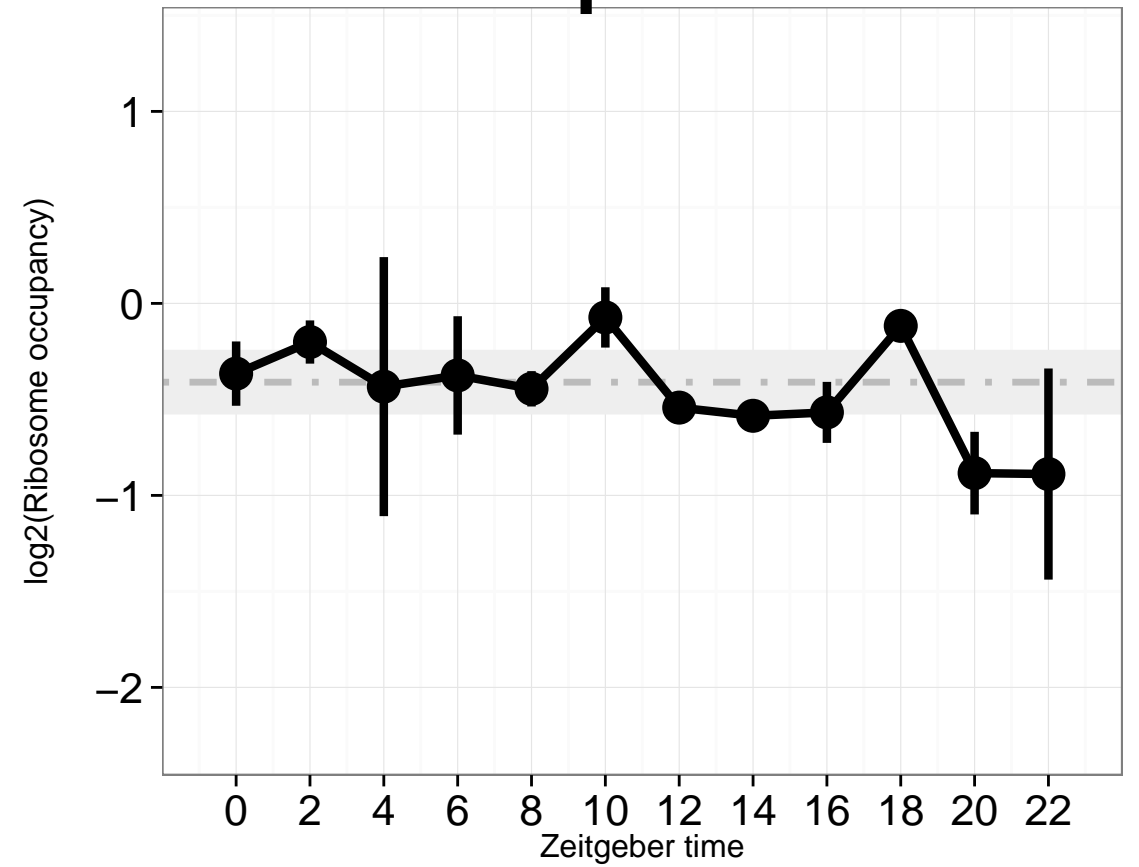

Supplement: Supplementary file 7 — Expression plots for kidney and liver for the 178 common rhythmic genes of Fig. 3c. (ZIP 3338.28 kb) [file 13059_2017_1222_MOESM7_ESM.zip › set_D_shared(178)/Gprin3_kidney_set_D.pdf]

# Gprin3

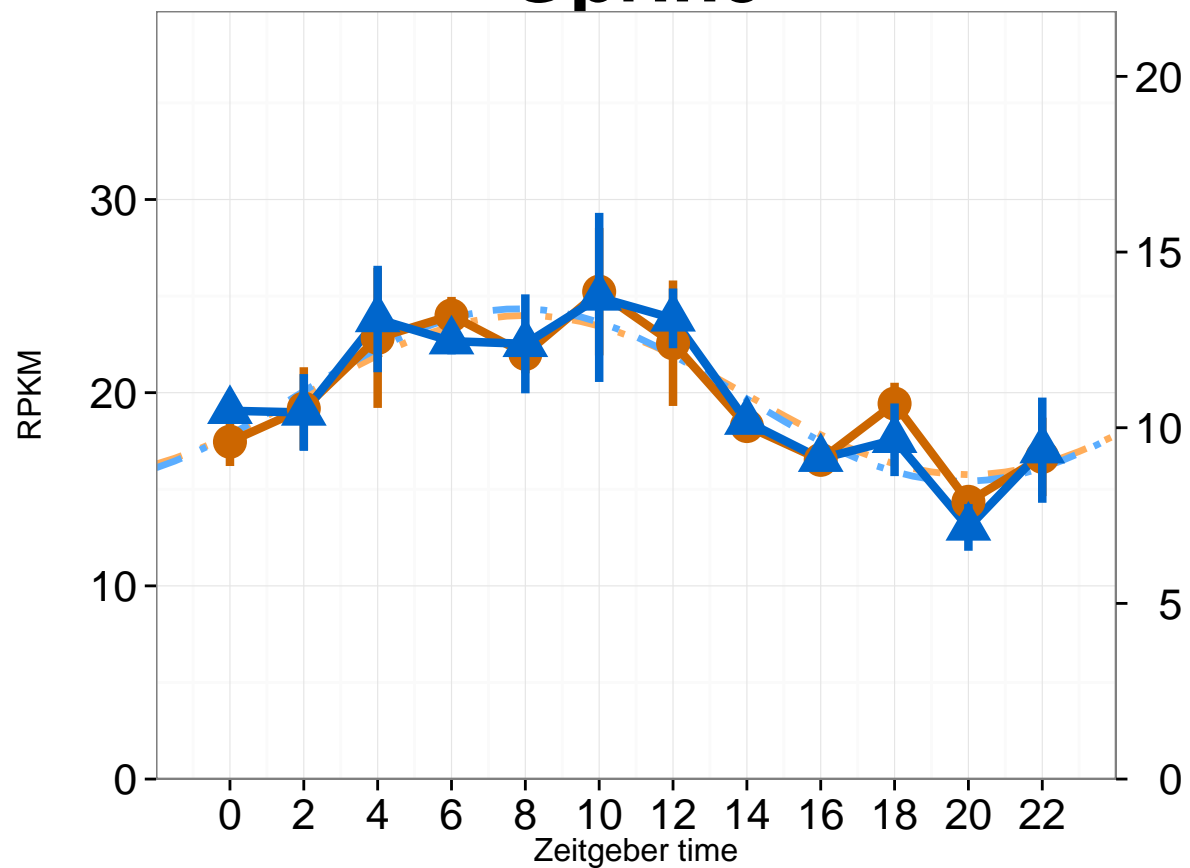

# Gprin3

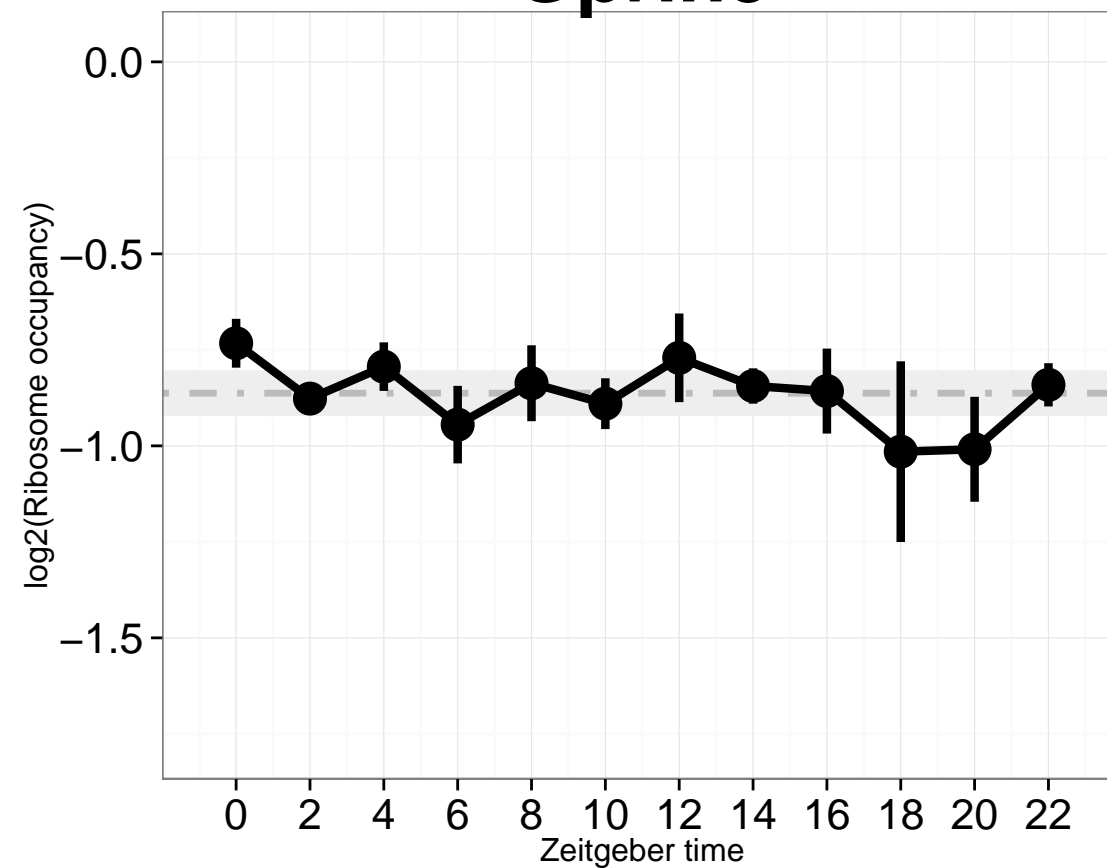

Supplement: Supplementary file 7 — Expression plots for kidney and liver for the 178 common rhythmic genes of Fig. 3c. (ZIP 3338.28 kb) [file 13059_2017_1222_MOESM7_ESM.zip › set_D_shared(178)/Gprin3_liver_set_D.pdf]

## Herc3

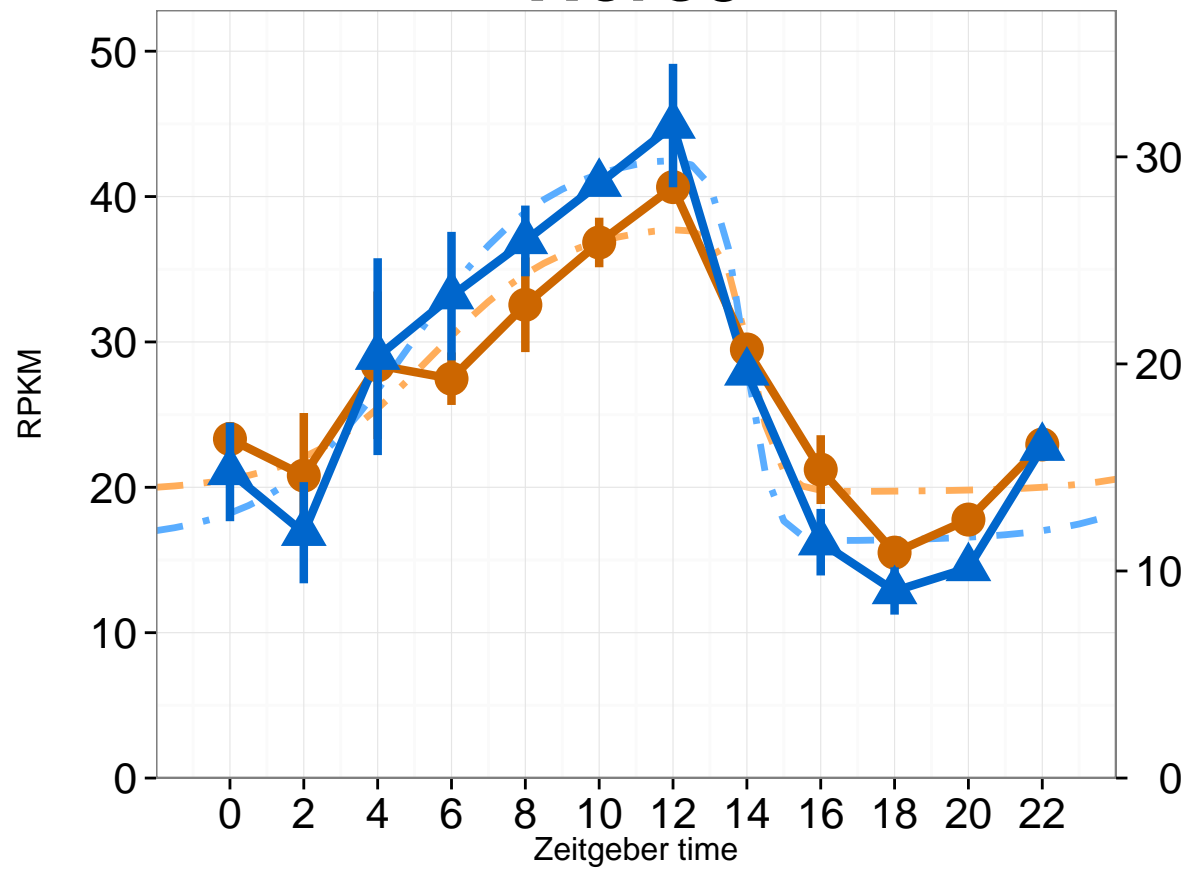

## Herc3

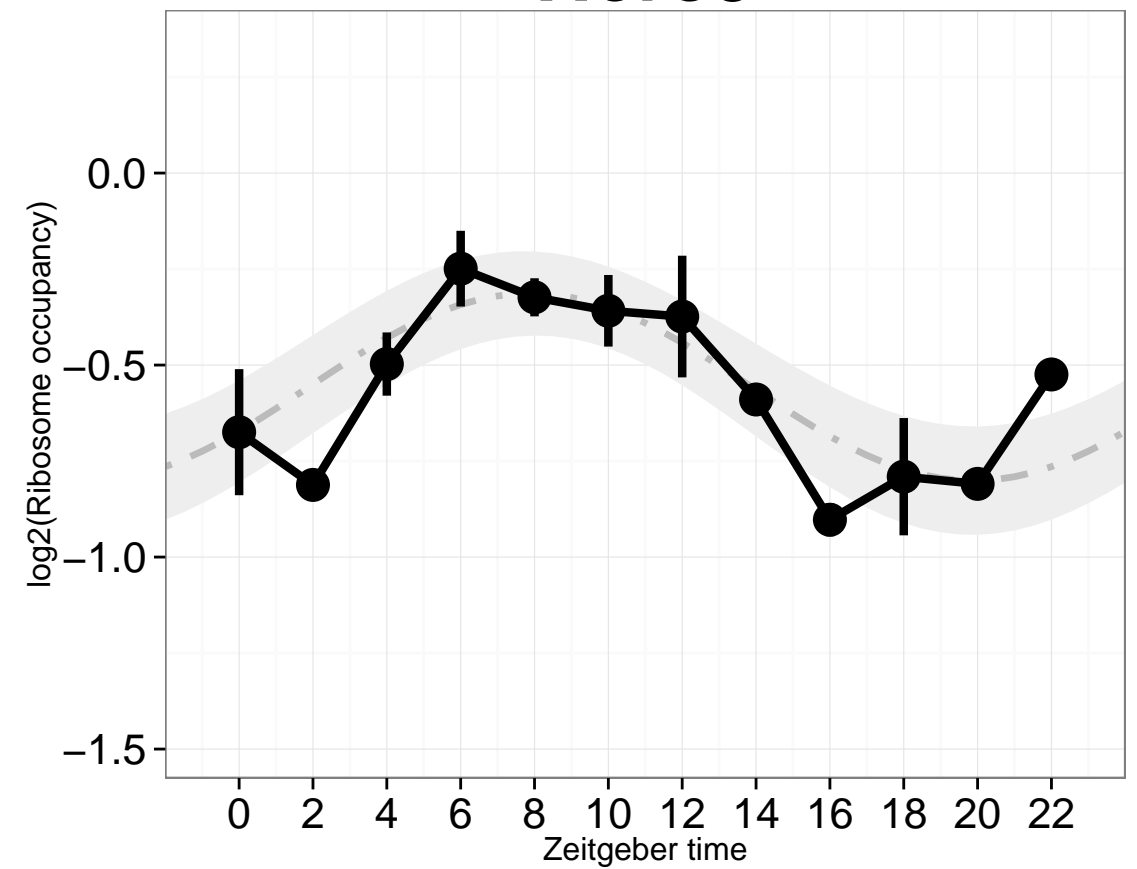

Supplement: Supplementary file 7 — Expression plots for kidney and liver for the 178 common rhythmic genes of Fig. 3c. (ZIP 3338.28 kb) [file 13059_2017_1222_MOESM7_ESM.zip › set_D_shared(178)/Herc3_kidney_set_D.pdf]

## Herc3

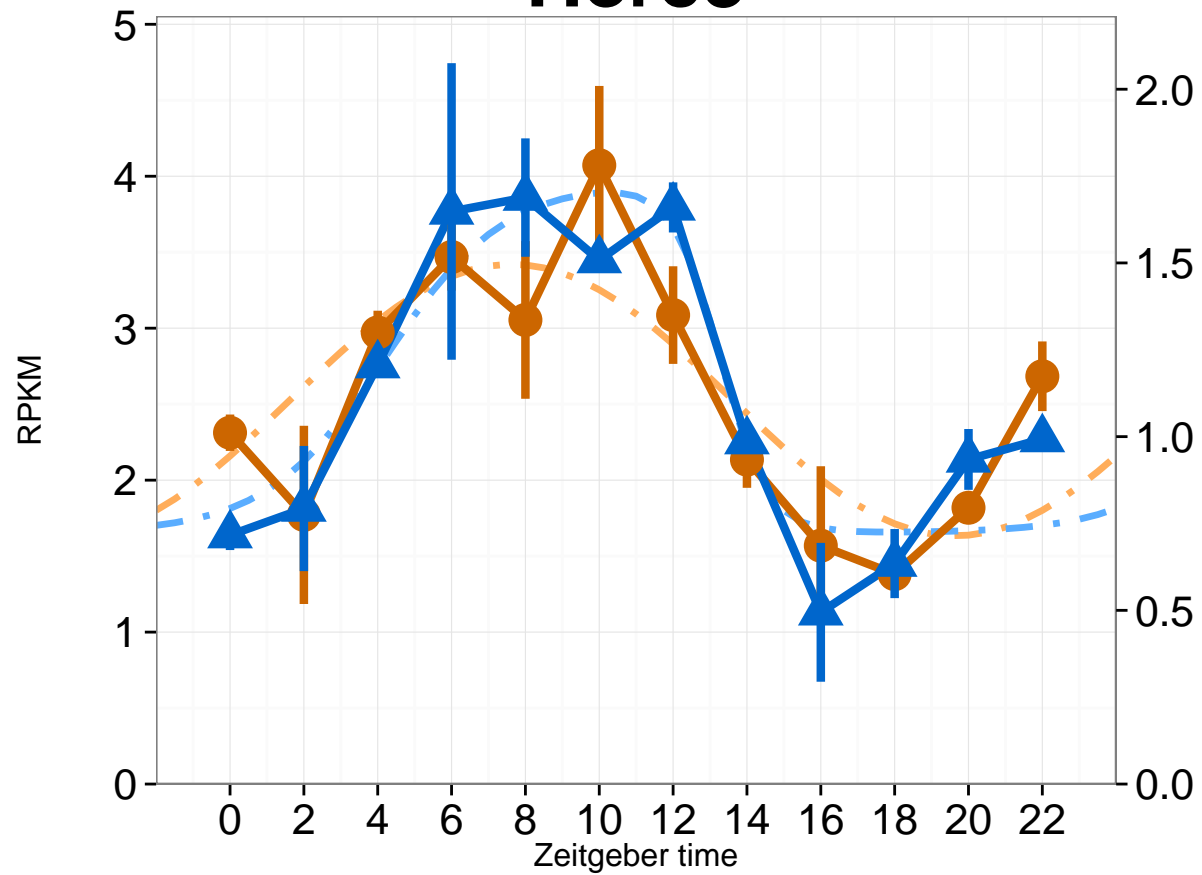

## Herc3

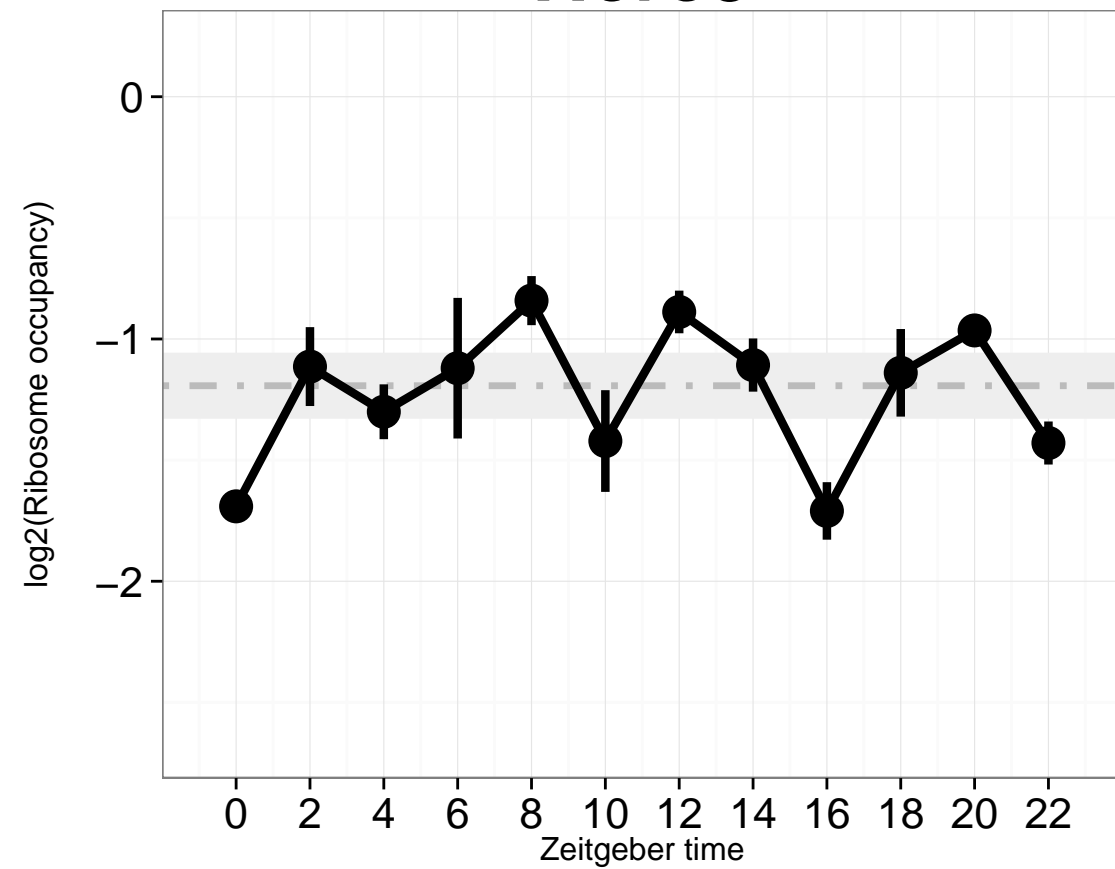

Supplement: Supplementary file 7 — Expression plots for kidney and liver for the 178 common rhythmic genes of Fig. 3c. (ZIP 3338.28 kb) [file 13059_2017_1222_MOESM7_ESM.zip › set_D_shared(178)/Herc3_liver_set_D.pdf]

# Herpud1

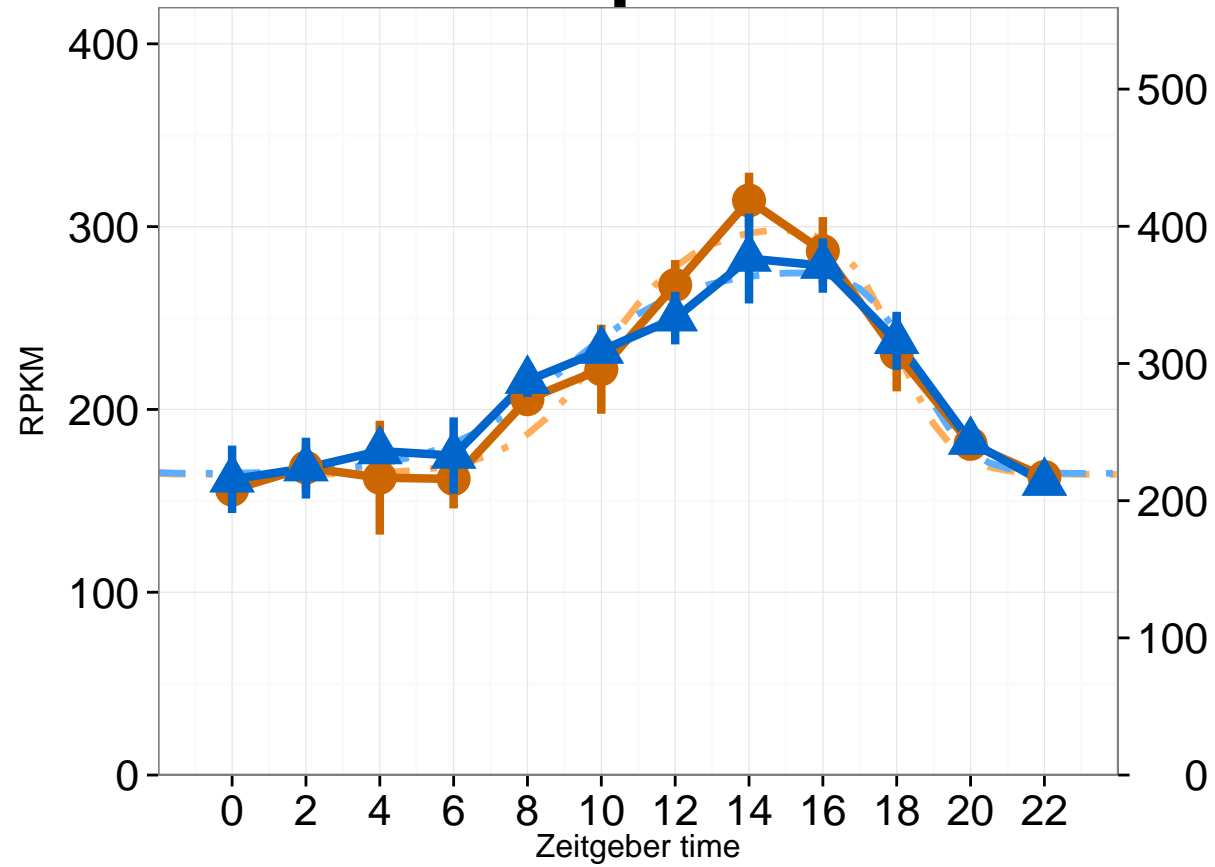

# Herpud1

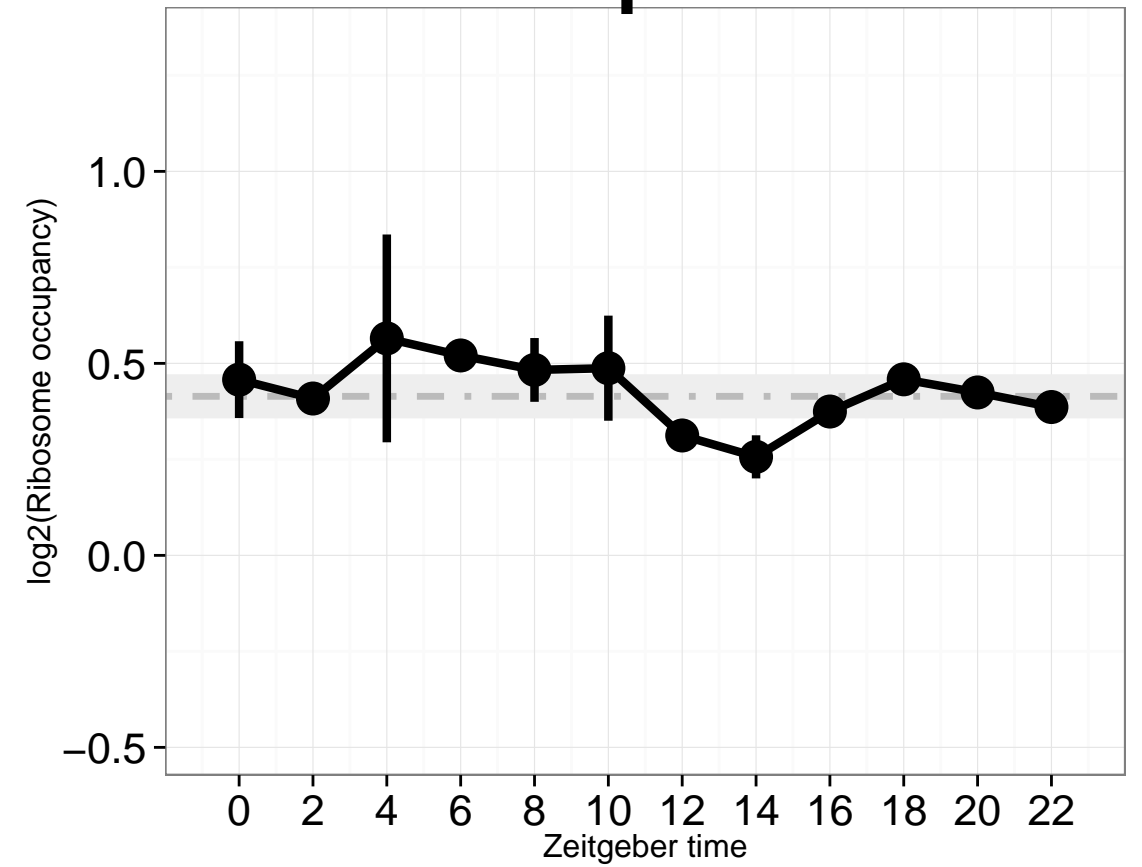

Supplement: Supplementary file 7 — Expression plots for kidney and liver for the 178 common rhythmic genes of Fig. 3c. (ZIP 3338.28 kb) [file 13059_2017_1222_MOESM7_ESM.zip › set_D_shared(178)/Herpud1_kidney_set_D.pdf]

# Herpud1

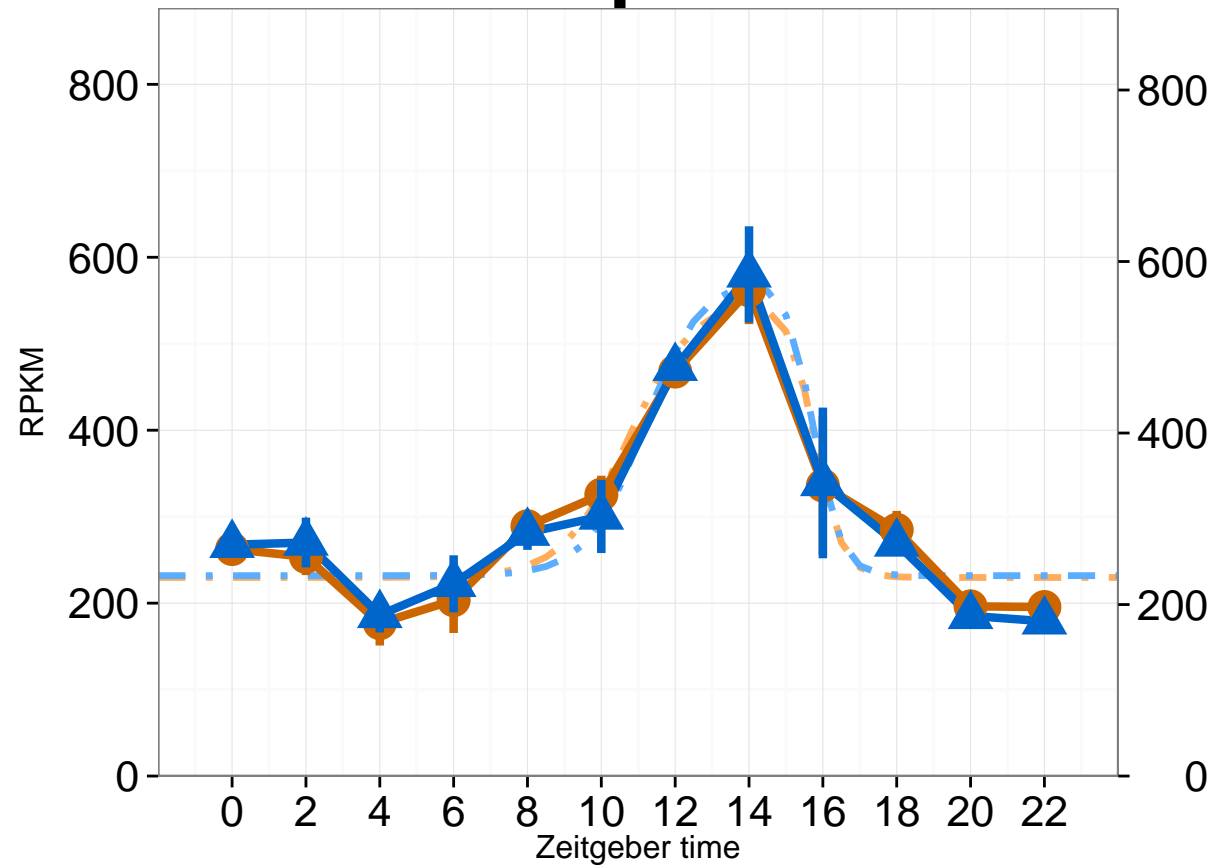

# Herpud1

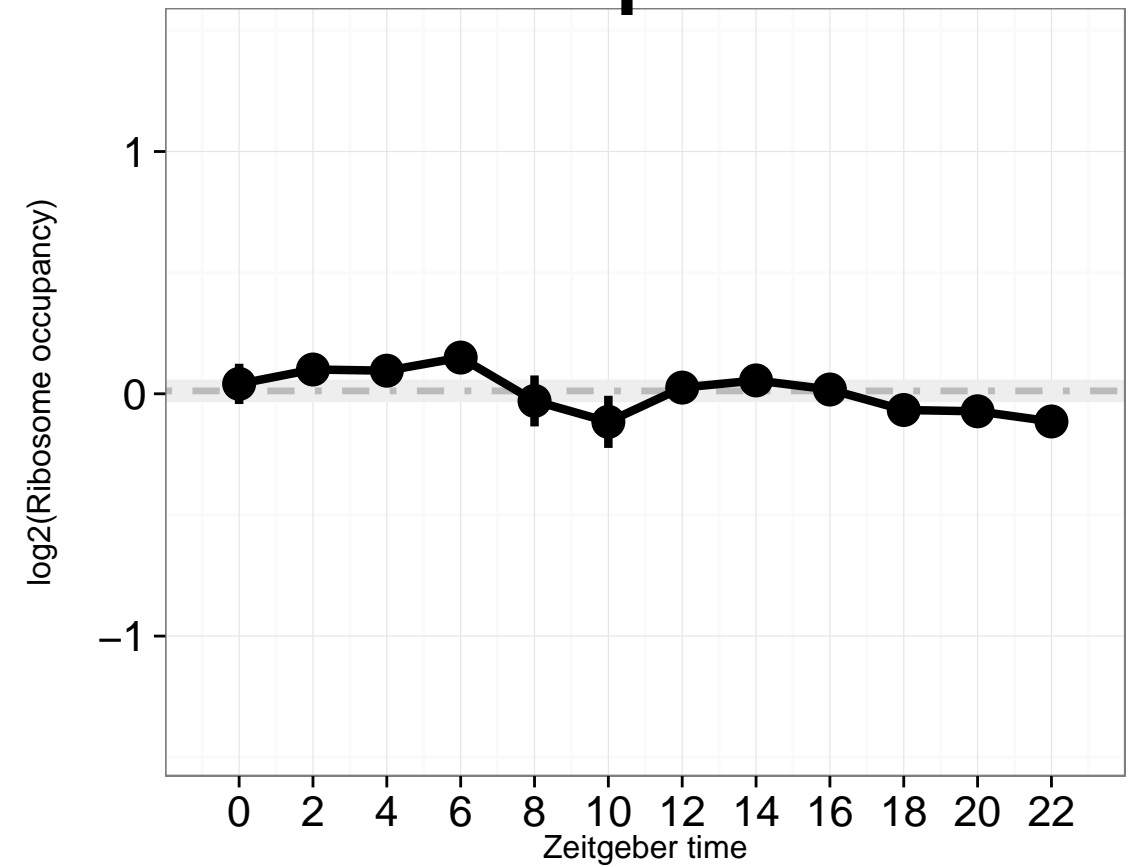

Supplement: Supplementary file 7 — Expression plots for kidney and liver for the 178 common rhythmic genes of Fig. 3c. (ZIP 3338.28 kb) [file 13059_2017_1222_MOESM7_ESM.zip › set_D_shared(178)/Herpud1_liver_set_D.pdf]

# Hist1h1c

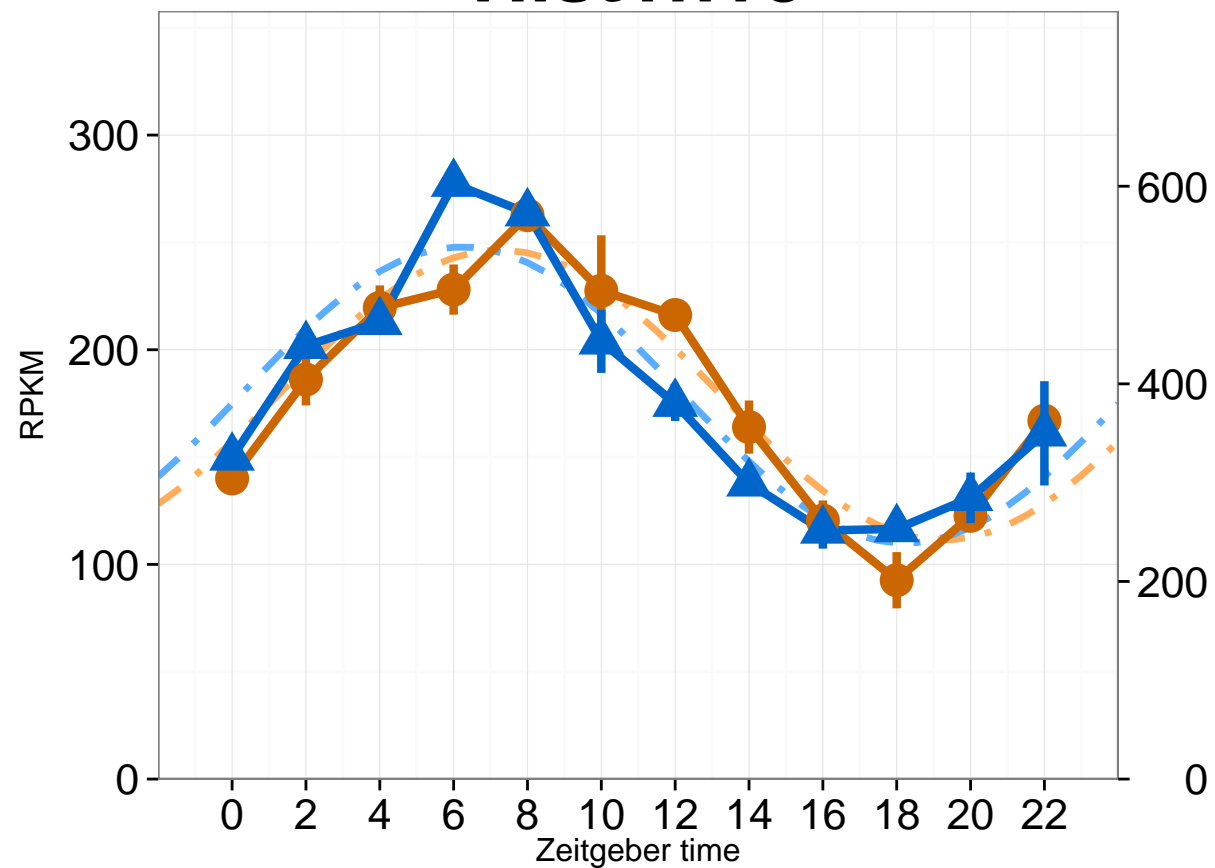

# Hist1h1c

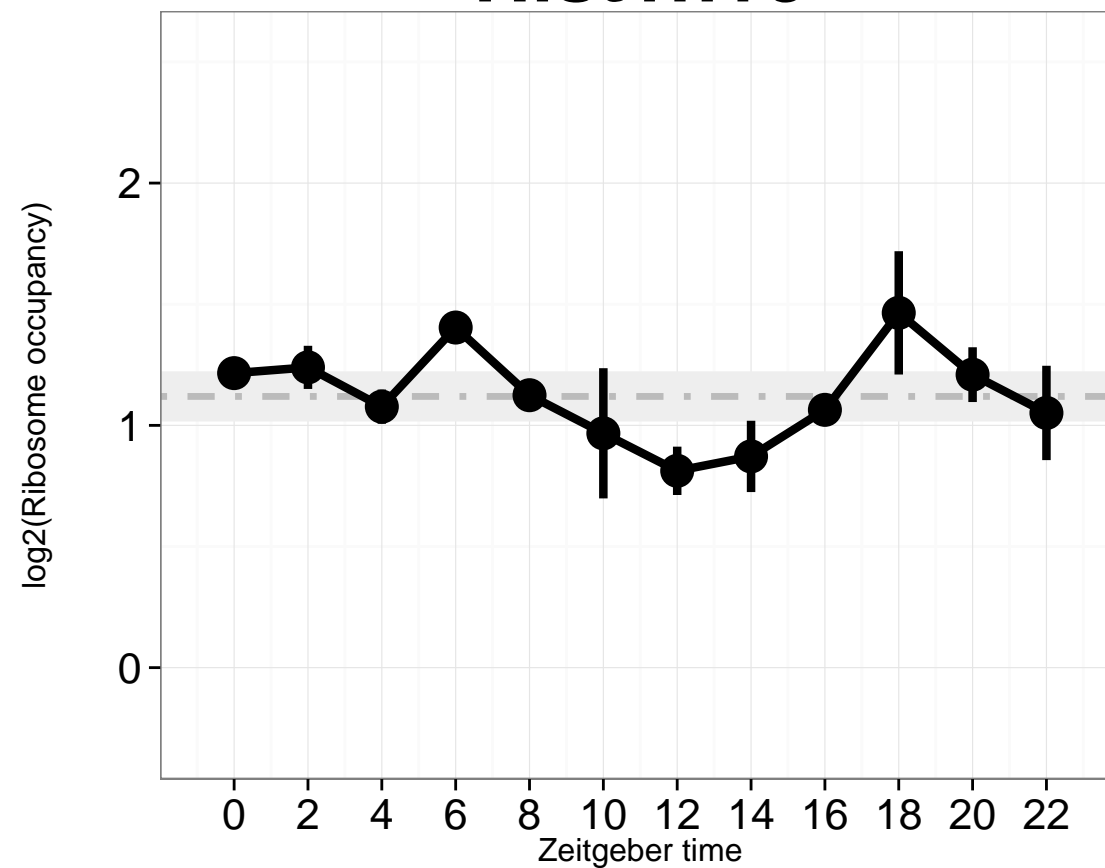

Supplement: Supplementary file 7 — Expression plots for kidney and liver for the 178 common rhythmic genes of Fig. 3c. (ZIP 3338.28 kb) [file 13059_2017_1222_MOESM7_ESM.zip › set_D_shared(178)/Hist1h1c_kidney_set_D.pdf]

# Hist1h1c

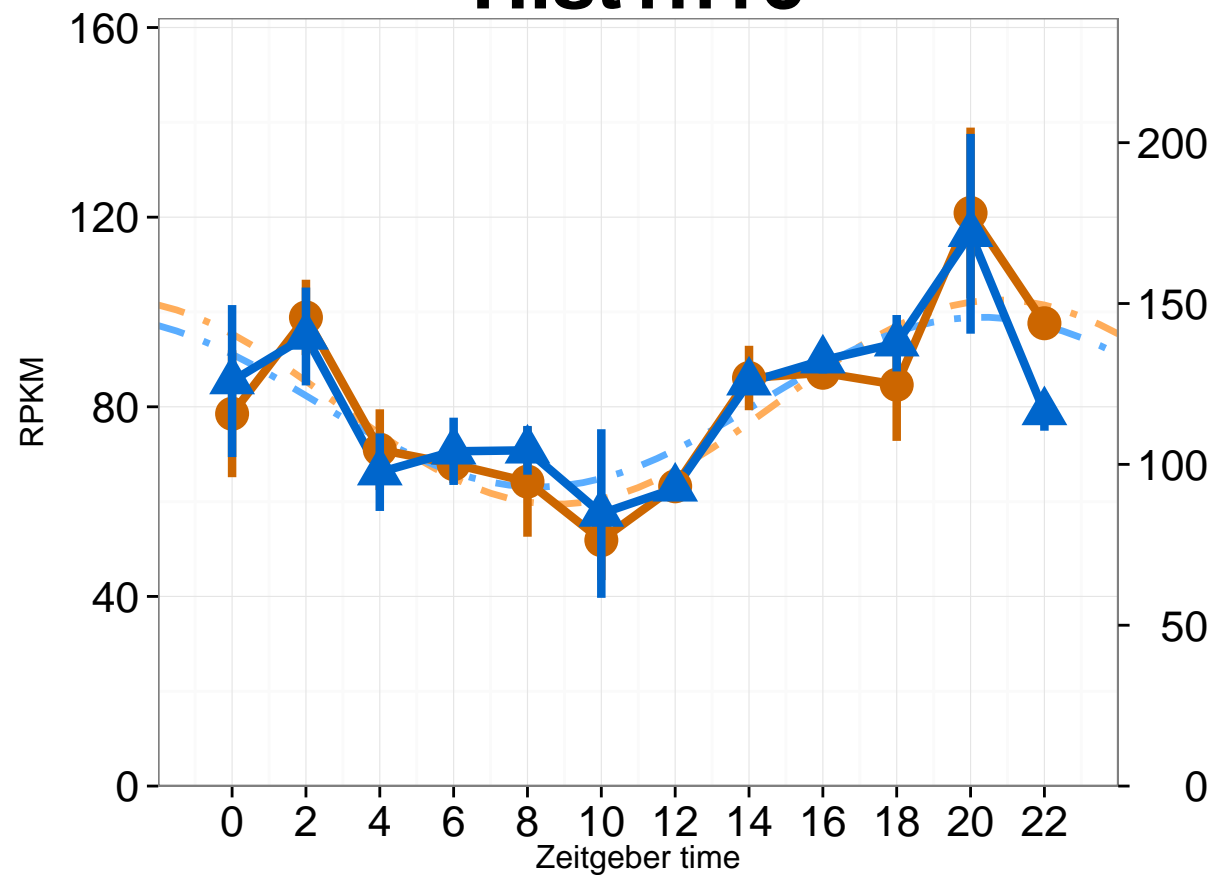

# Hist1h1c

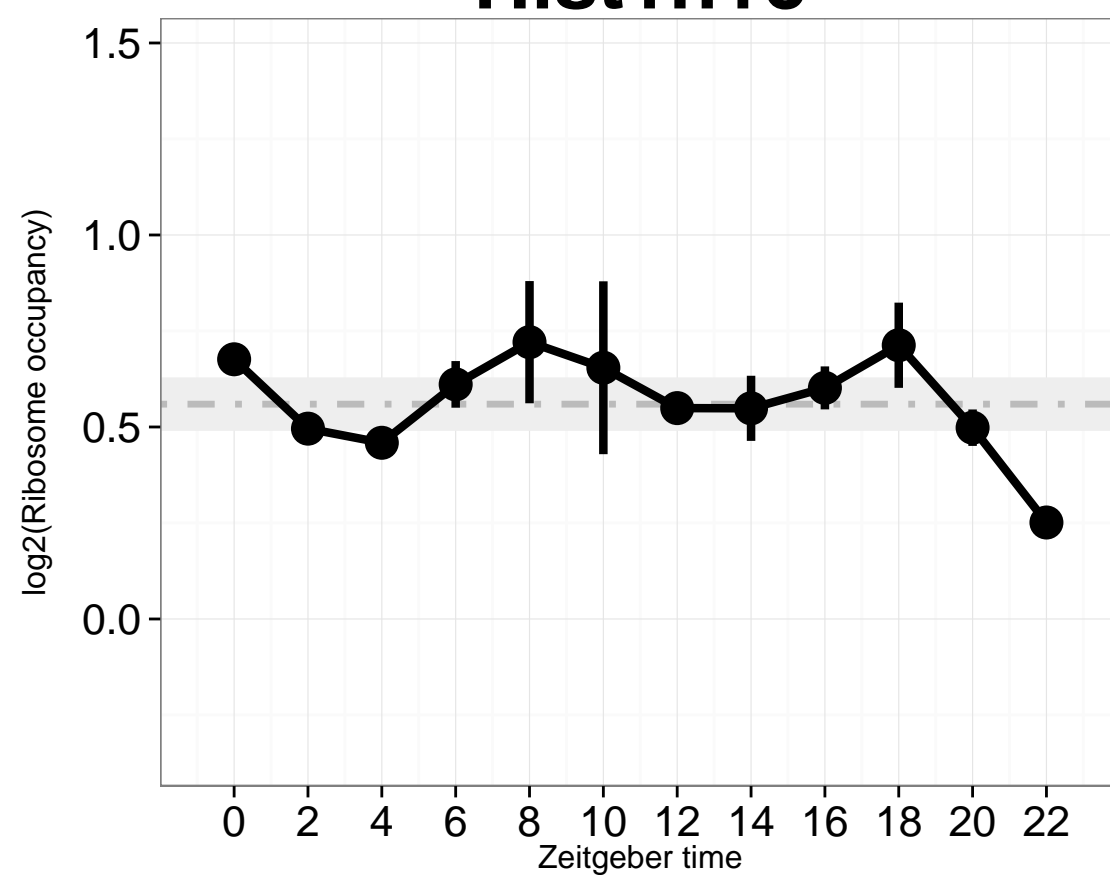

Supplement: Supplementary file 7 — Expression plots for kidney and liver for the 178 common rhythmic genes of Fig. 3c. (ZIP 3338.28 kb) [file 13059_2017_1222_MOESM7_ESM.zip › set_D_shared(178)/Hist1h1c_liver_set_D.pdf]

# Hlf

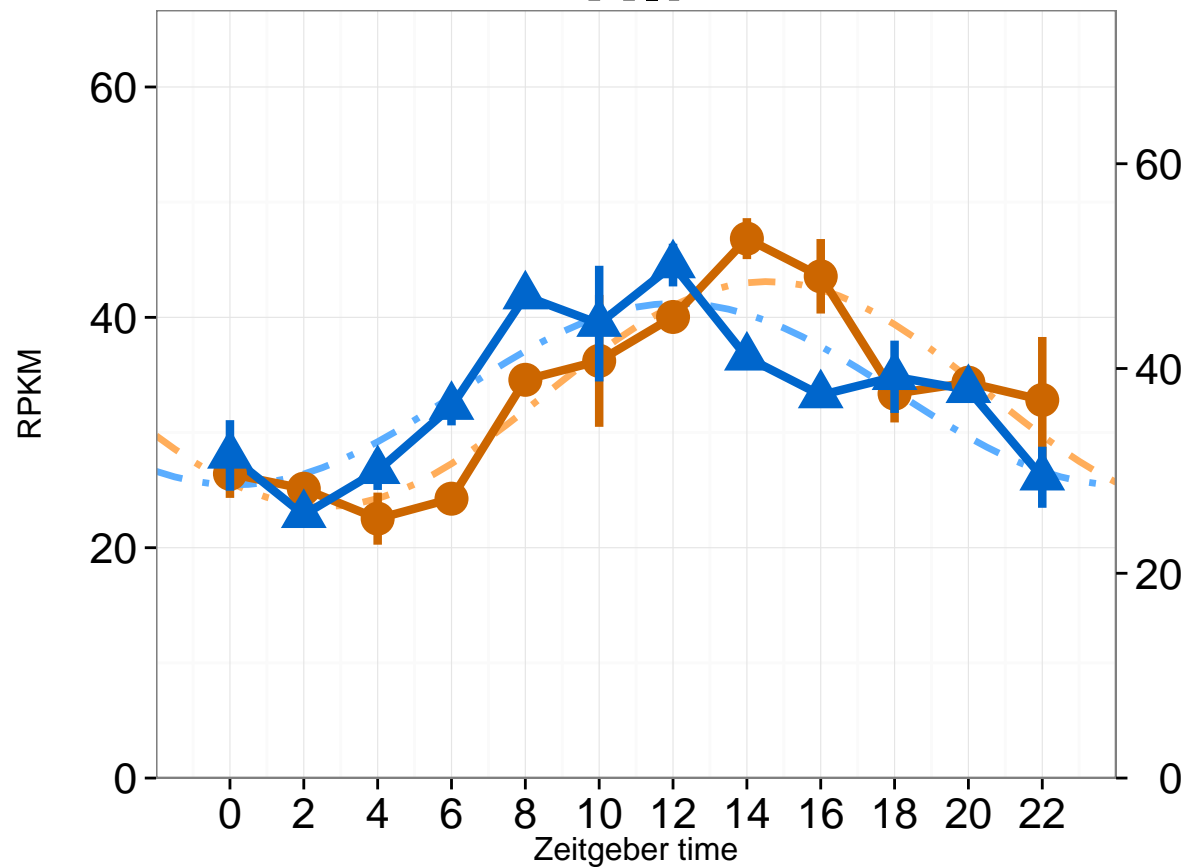

# Hlf

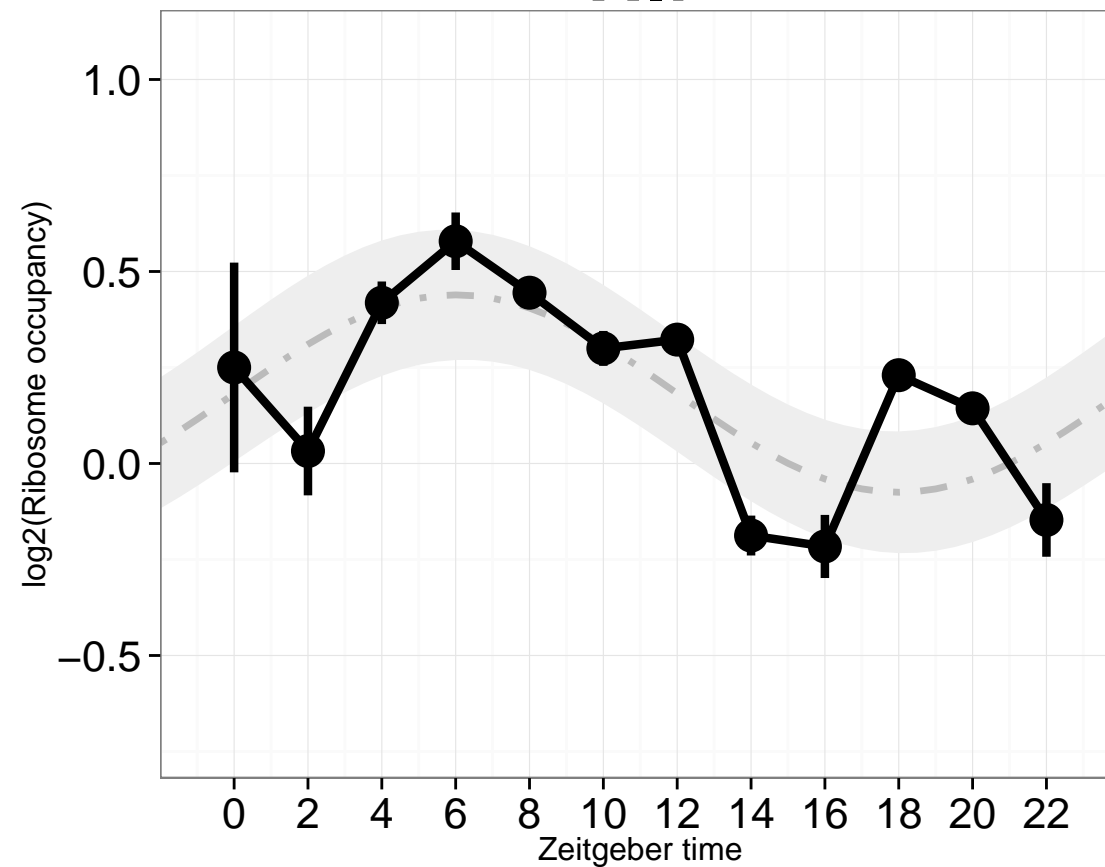

Supplement: Supplementary file 7 — Expression plots for kidney and liver for the 178 common rhythmic genes of Fig. 3c. (ZIP 3338.28 kb) [file 13059_2017_1222_MOESM7_ESM.zip › set_D_shared(178)/Hlf_kidney_set_D.pdf]

# Hif

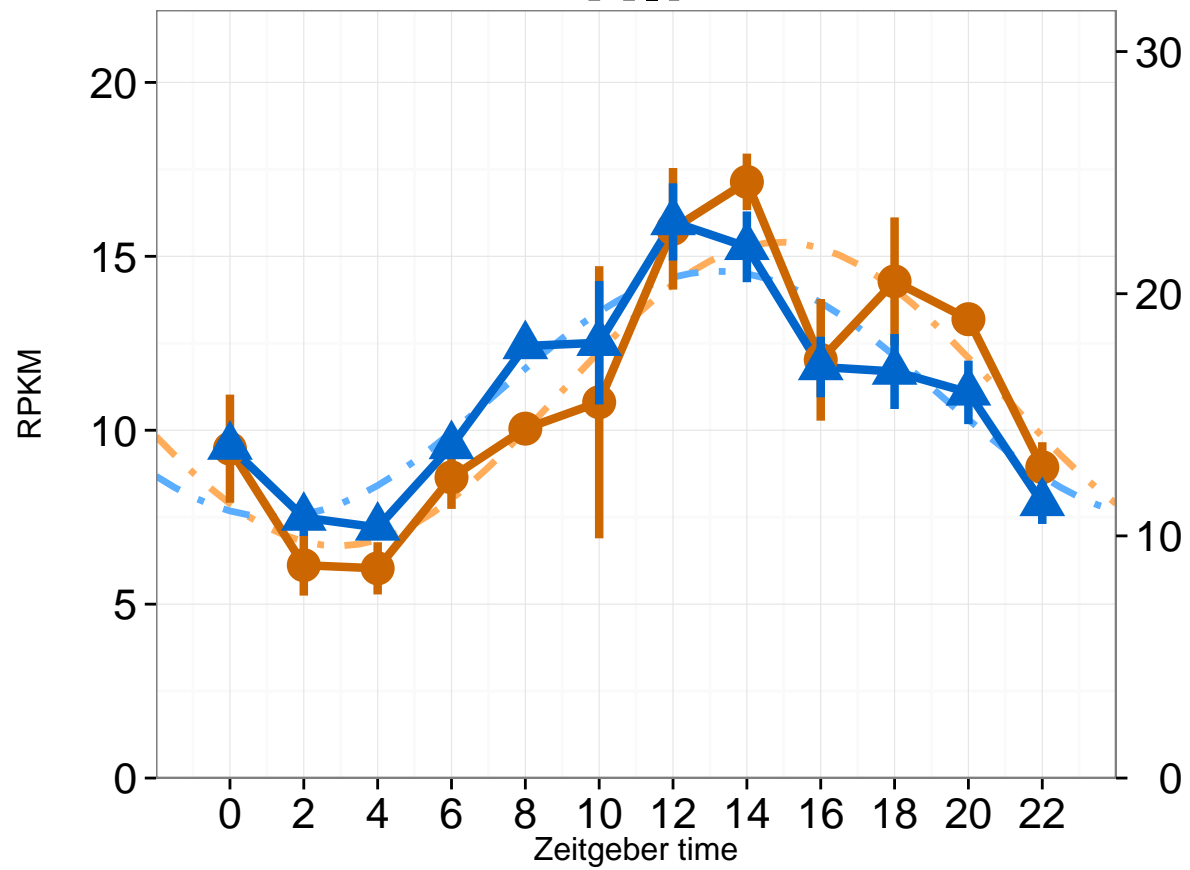

# Hif

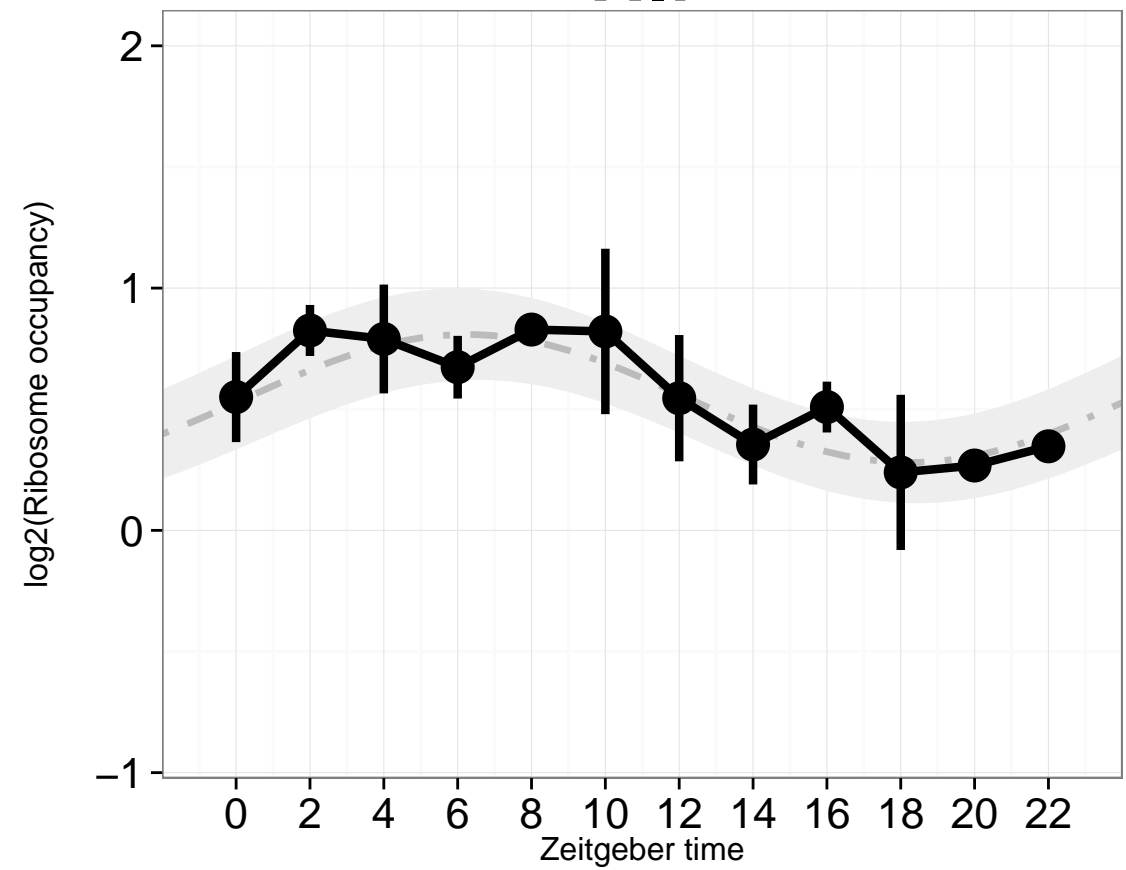

Supplement: Supplementary file 7 — Expression plots for kidney and liver for the 178 common rhythmic genes of Fig. 3c. (ZIP 3338.28 kb) [file 13059_2017_1222_MOESM7_ESM.zip › set_D_shared(178)/Hlf_liver_set_D.pdf]

# Hnf1b

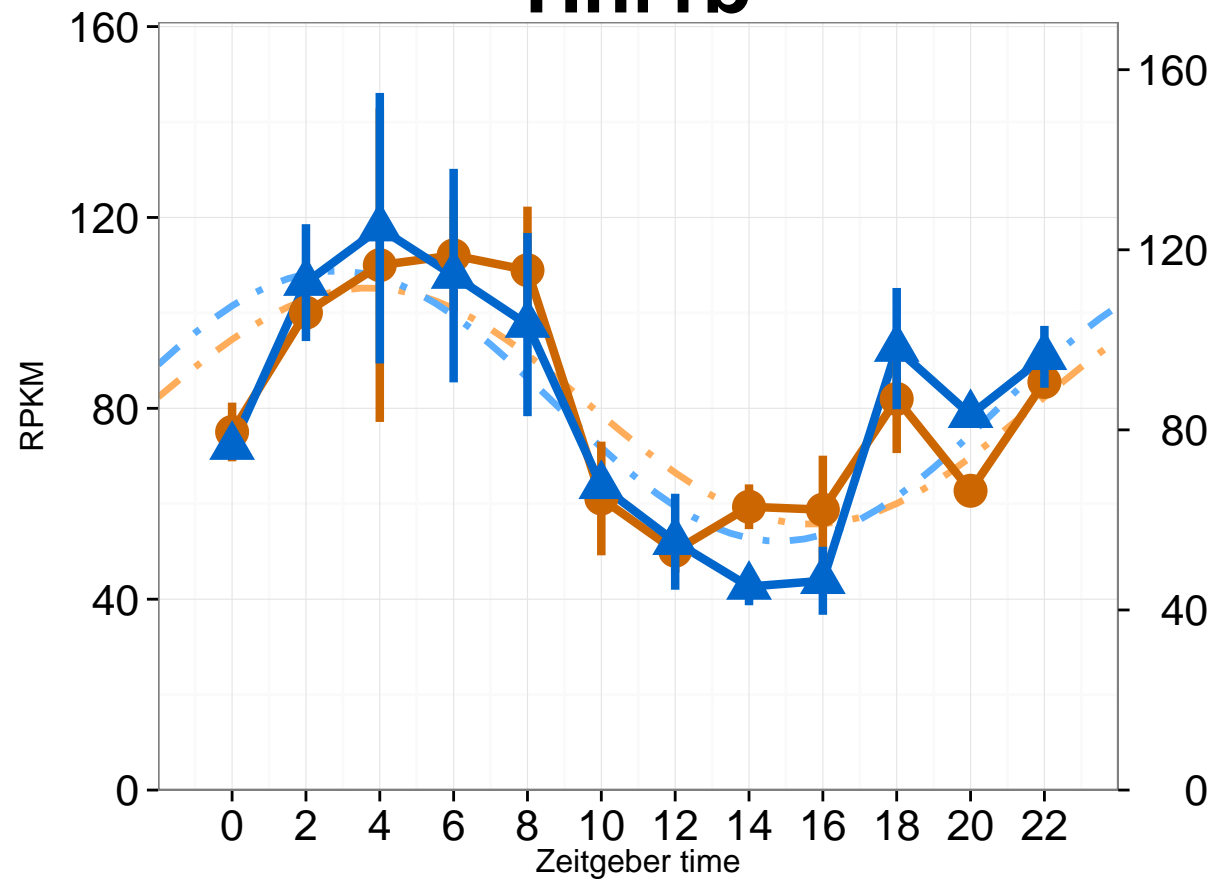

# Hnf1b

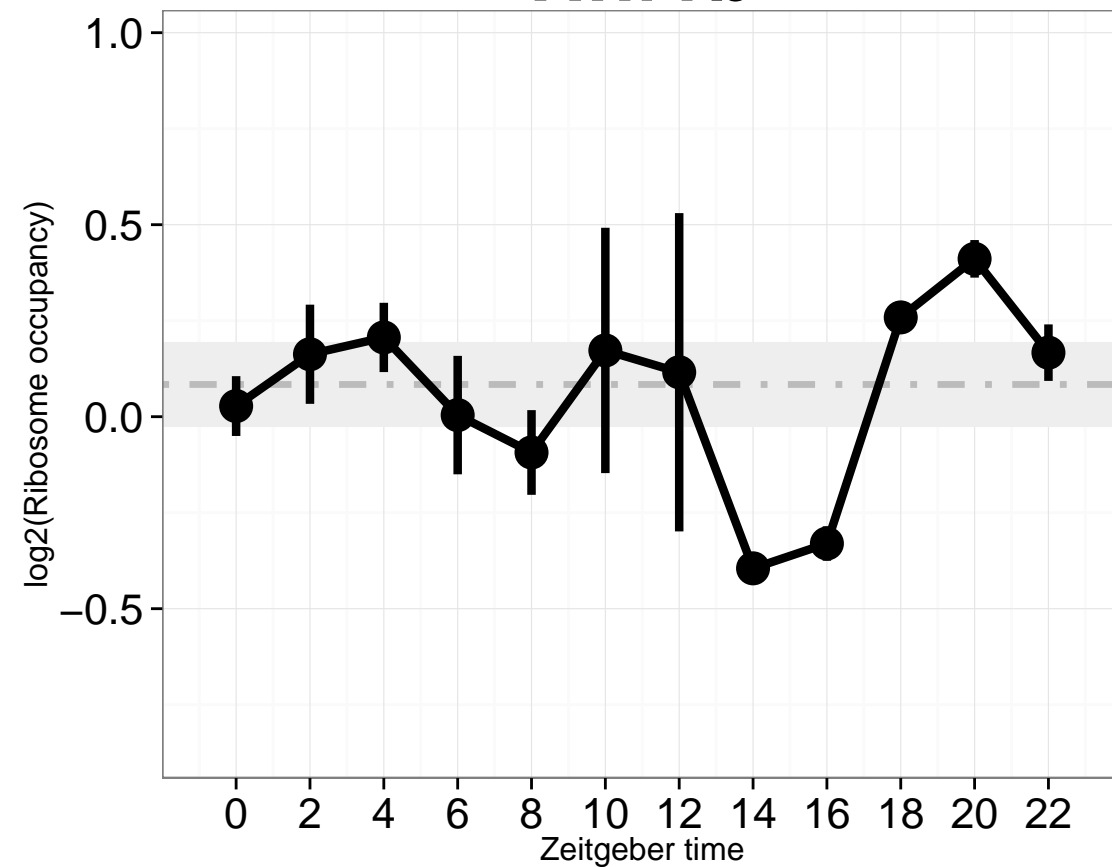

Supplement: Supplementary file 7 — Expression plots for kidney and liver for the 178 common rhythmic genes of Fig. 3c. (ZIP 3338.28 kb) [file 13059_2017_1222_MOESM7_ESM.zip › set_D_shared(178)/Hnf1b_kidney_set_D.pdf]

# Hnf1b

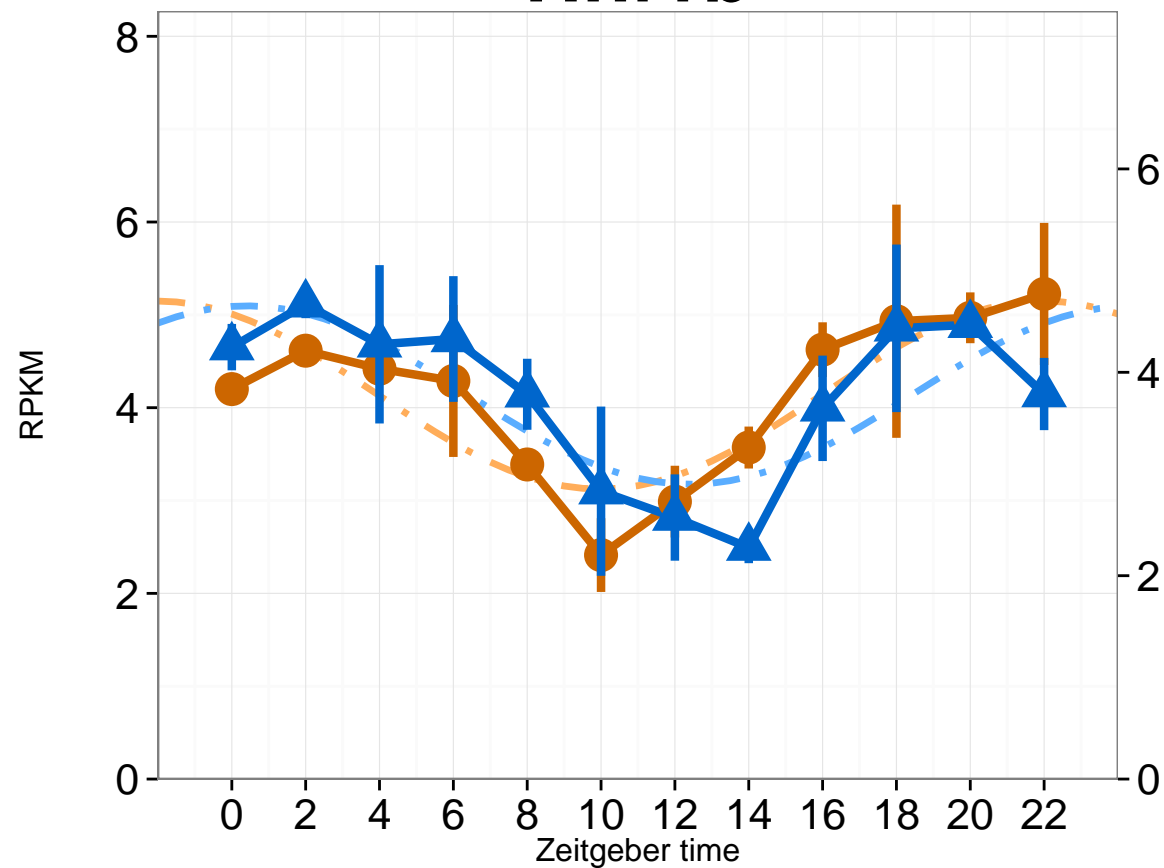

# Hnf1b

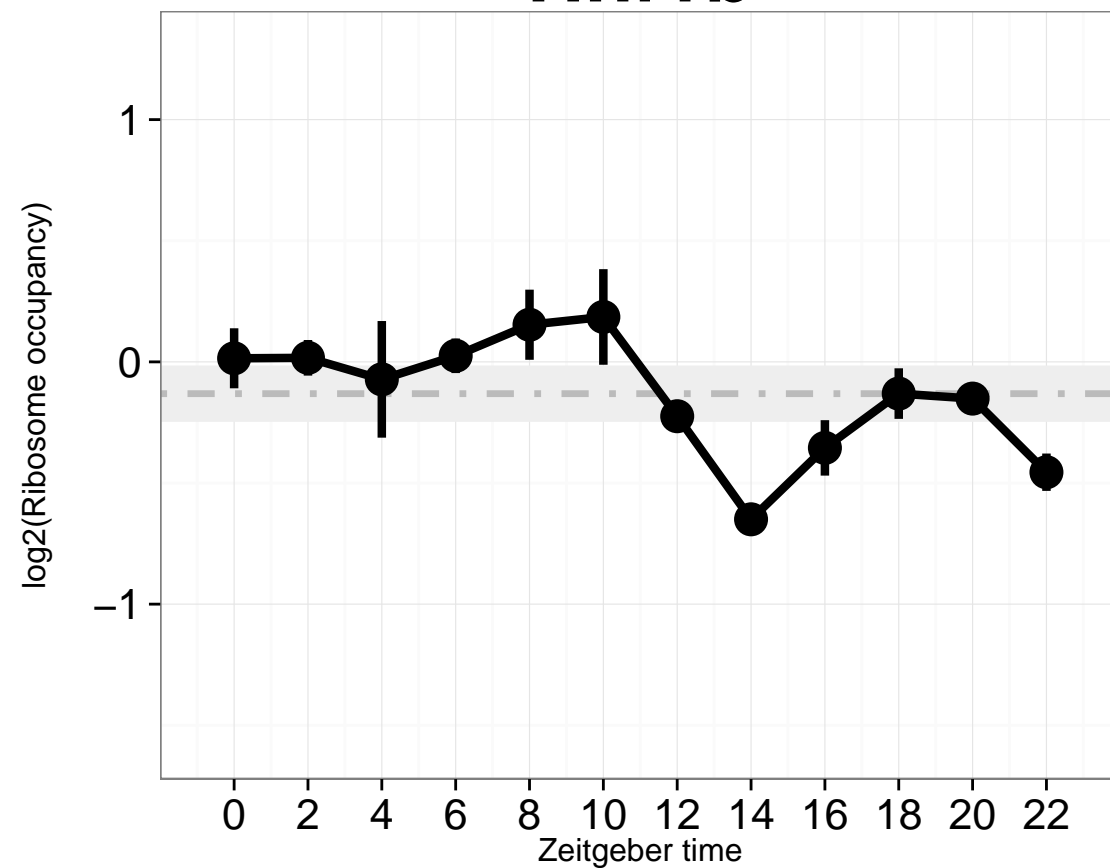

Supplement: Supplementary file 7 — Expression plots for kidney and liver for the 178 common rhythmic genes of Fig. 3c. (ZIP 3338.28 kb) [file 13059_2017_1222_MOESM7_ESM.zip › set_D_shared(178)/Hnf1b_liver_set_D.pdf]

## Hnrnpdl

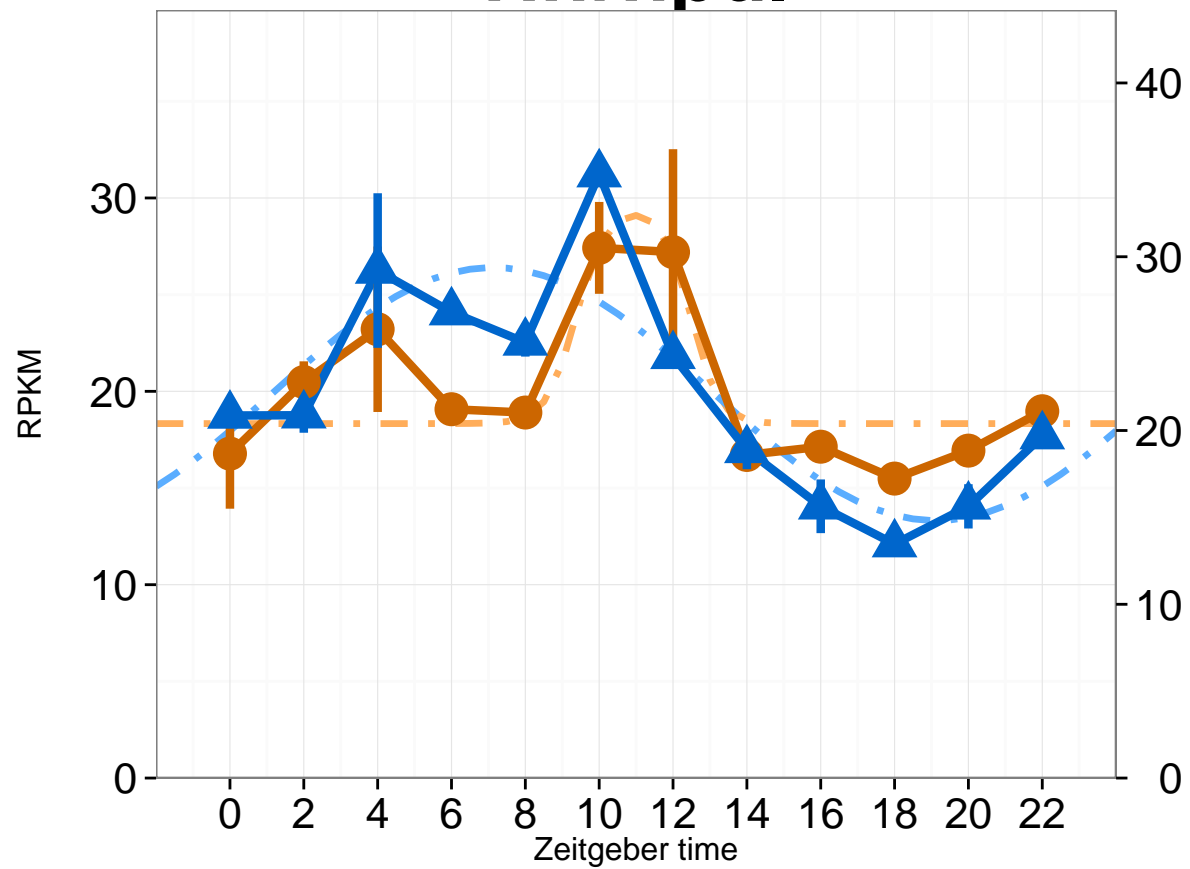

## Hnrnpdl

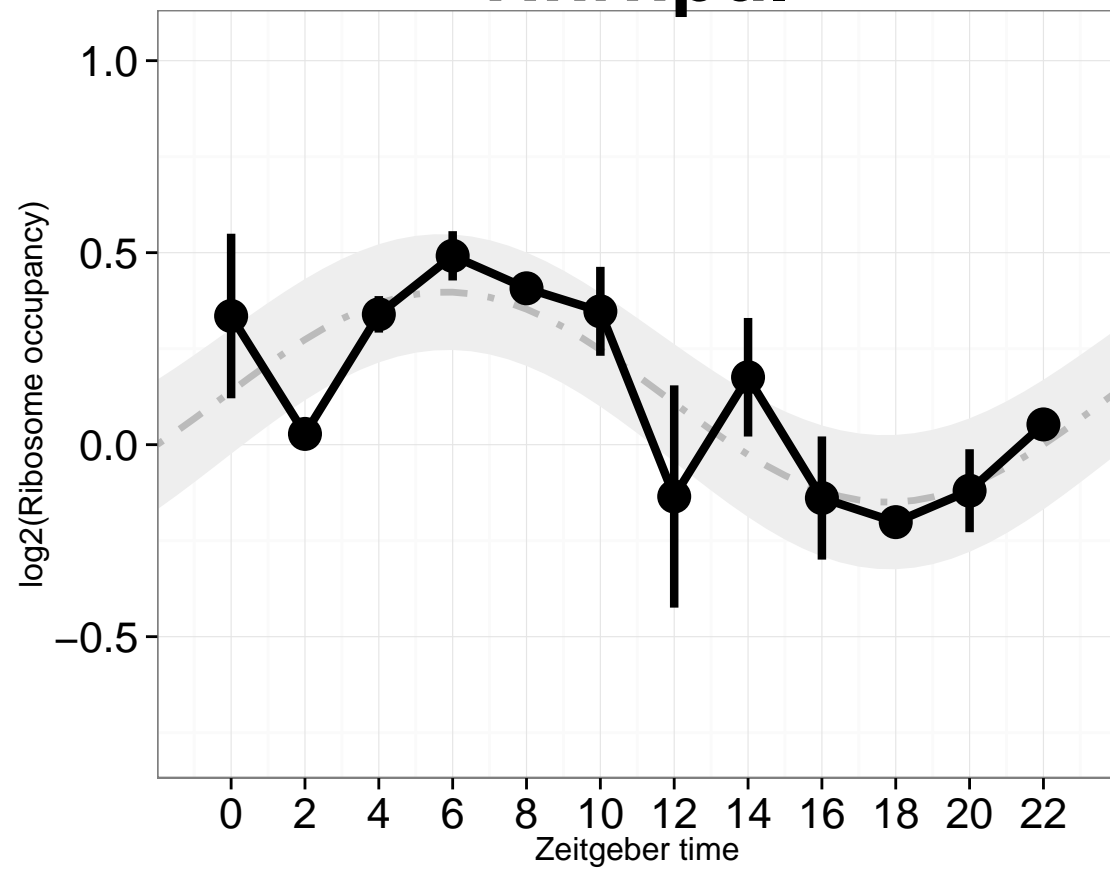

Supplement: Supplementary file 7 — Expression plots for kidney and liver for the 178 common rhythmic genes of Fig. 3c. (ZIP 3338.28 kb) [file 13059_2017_1222_MOESM7_ESM.zip › set_D_shared(178)/Hnrnpdl_kidney_set_D.pdf]

# Hnrnpdl

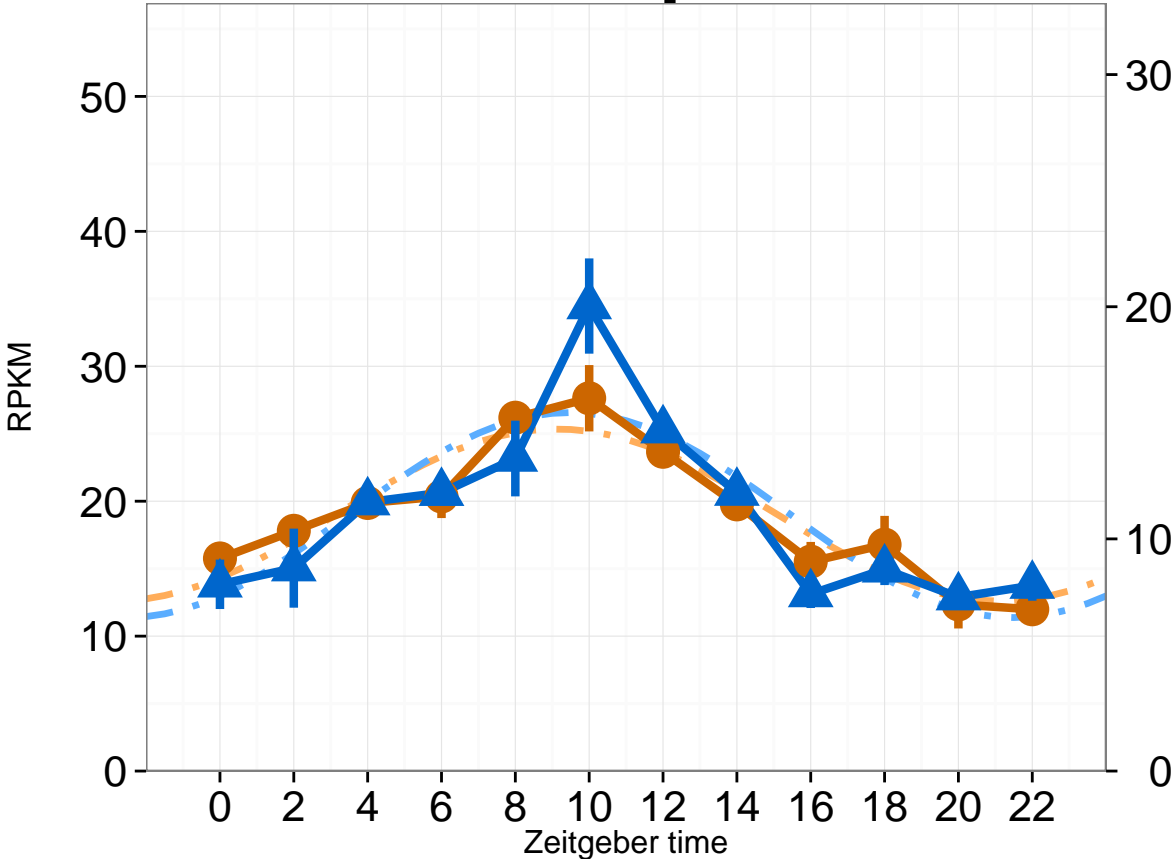

# Hnrnpdl

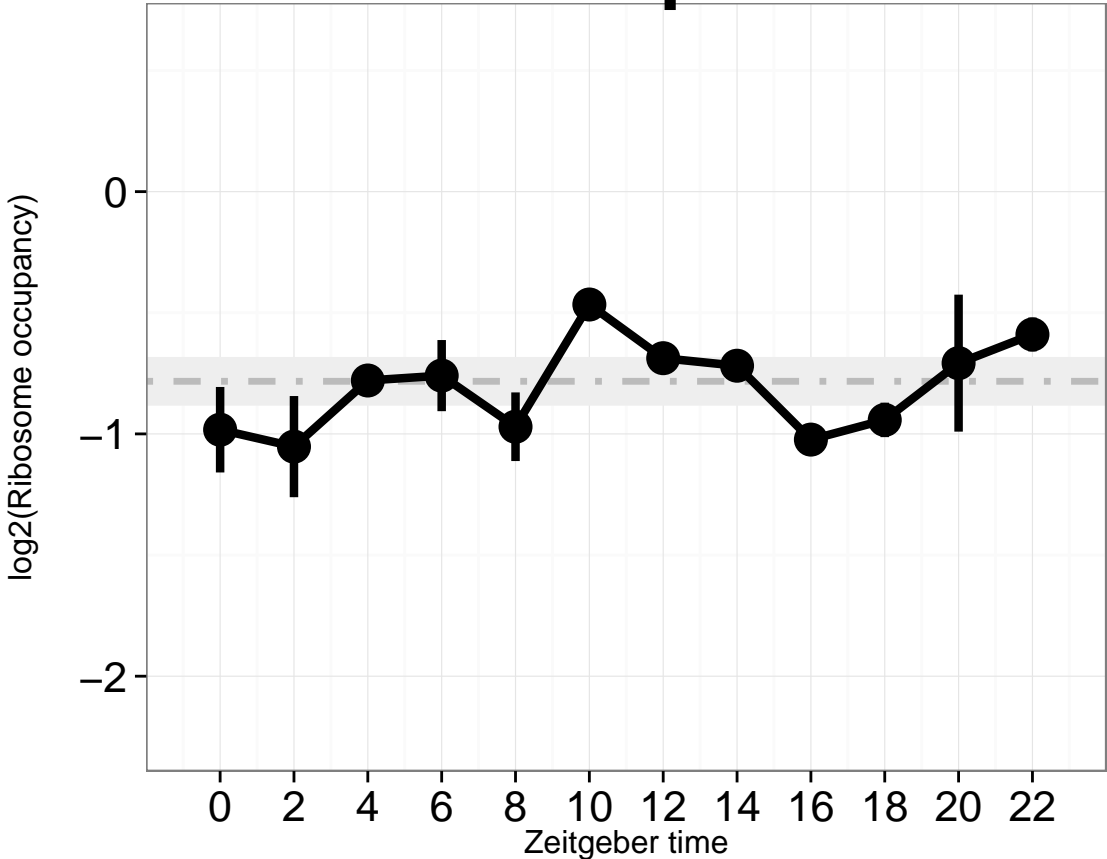

Supplement: Supplementary file 7 — Expression plots for kidney and liver for the 178 common rhythmic genes of Fig. 3c. (ZIP 3338.28 kb) [file 13059_2017_1222_MOESM7_ESM.zip › set_D_shared(178)/Hnrnpdl_liver_set_D.pdf]

## Hpx

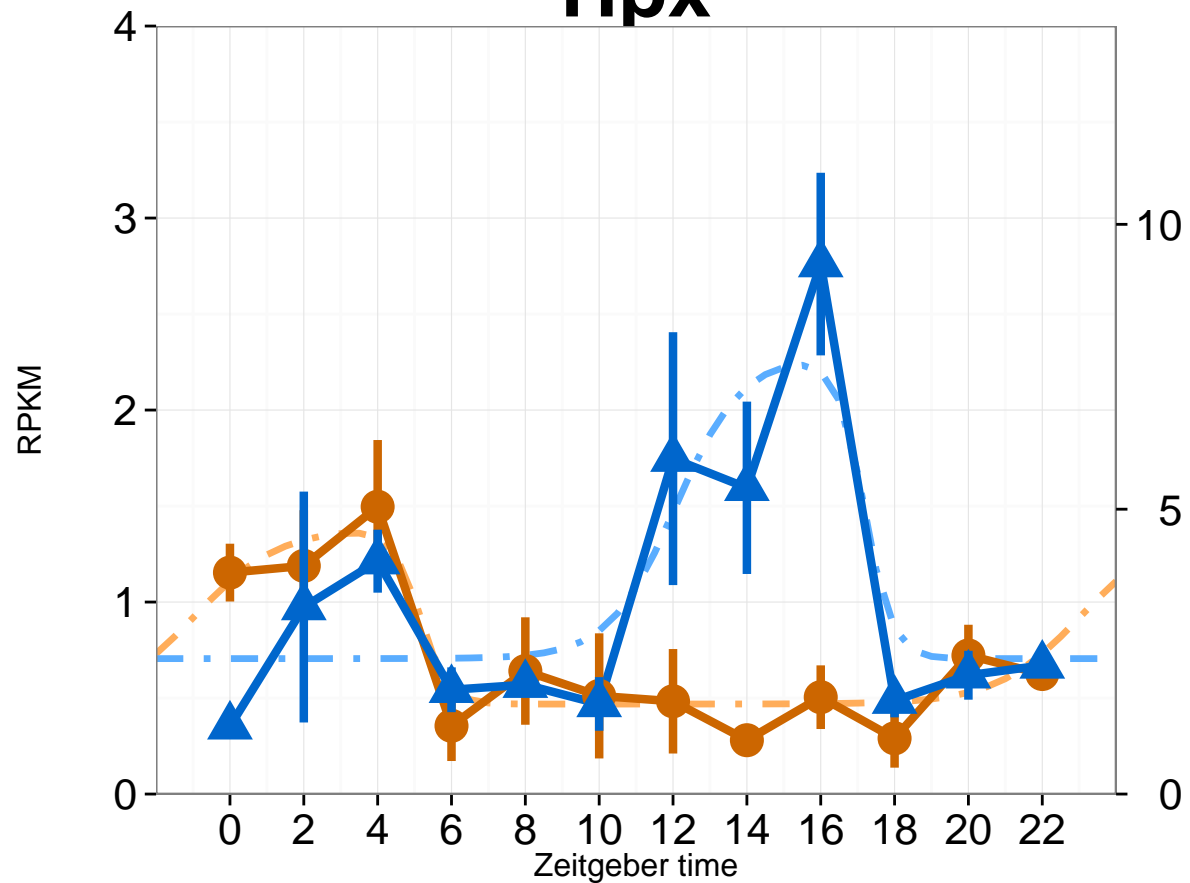

## Hpx

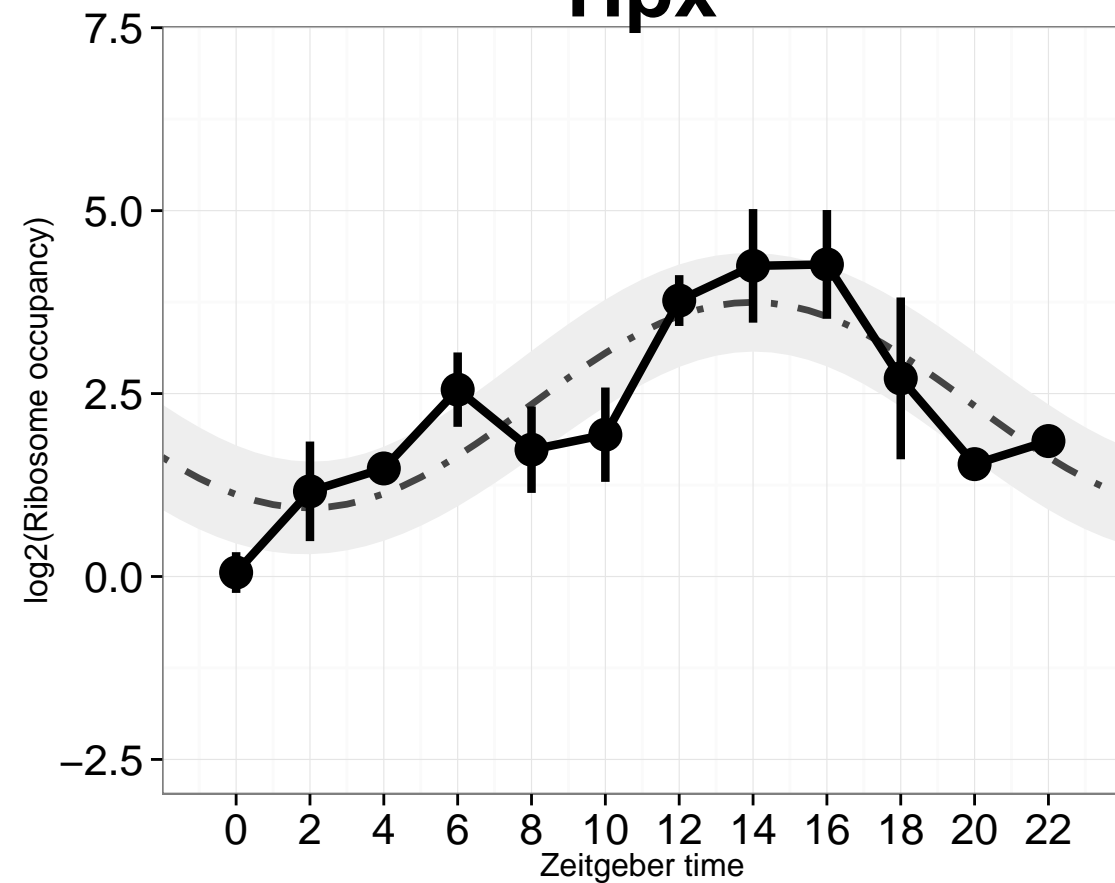

Supplement: Supplementary file 7 — Expression plots for kidney and liver for the 178 common rhythmic genes of Fig. 3c. (ZIP 3338.28 kb) [file 13059_2017_1222_MOESM7_ESM.zip › set_D_shared(178)/Hpx_kidney_set_D.pdf]

## Hpx

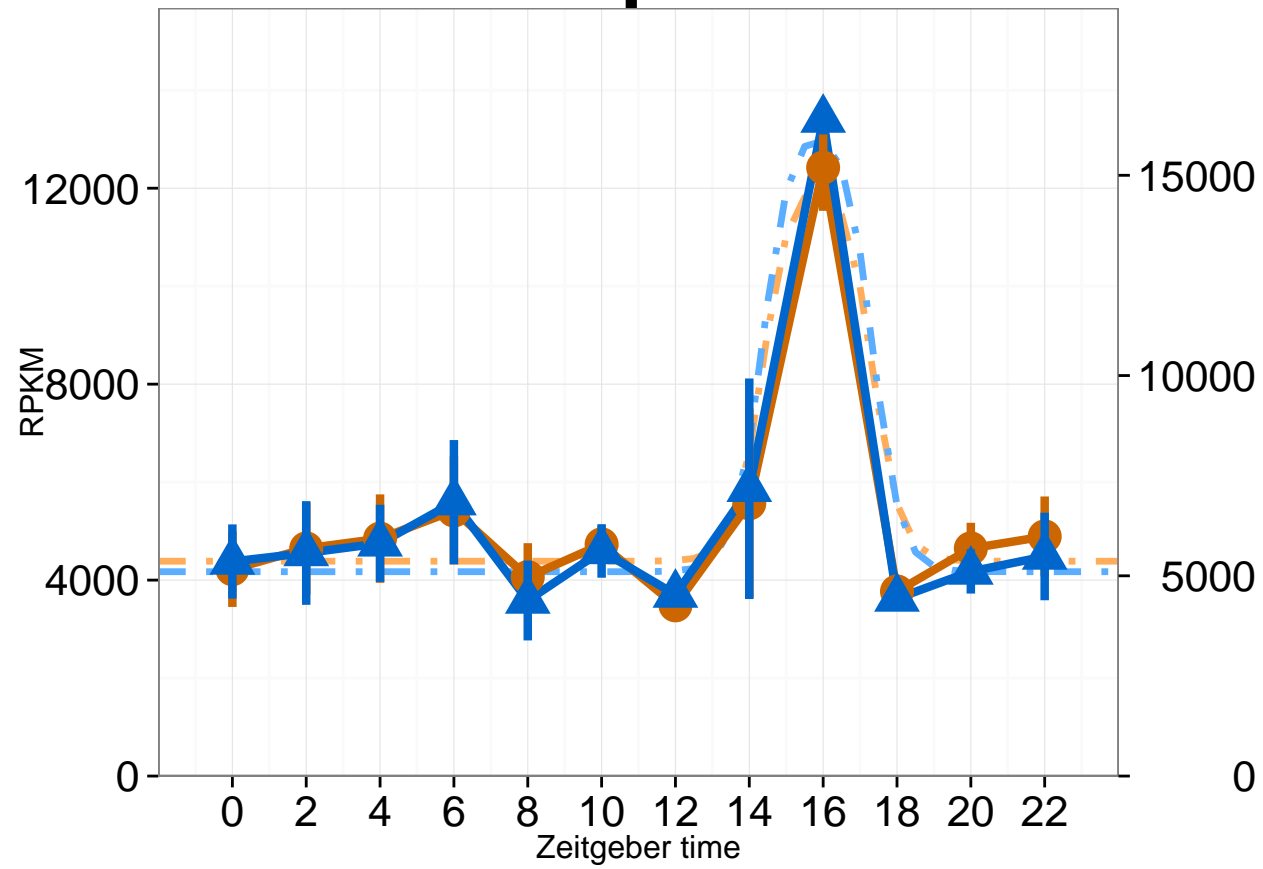

## Hpx

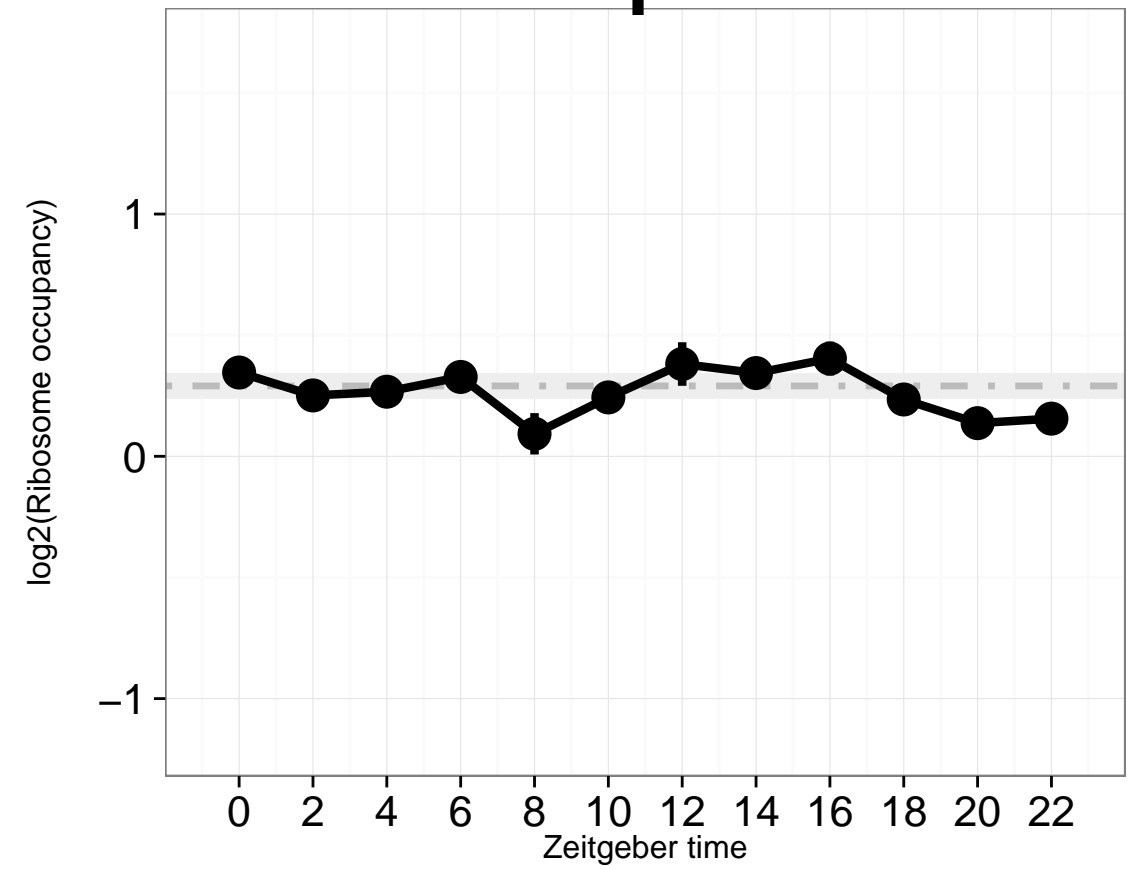

Supplement: Supplementary file 7 — Expression plots for kidney and liver for the 178 common rhythmic genes of Fig. 3c. (ZIP 3338.28 kb) [file 13059_2017_1222_MOESM7_ESM.zip › set_D_shared(178)/Hpx_liver_set_D.pdf]

# Hsp90aa1

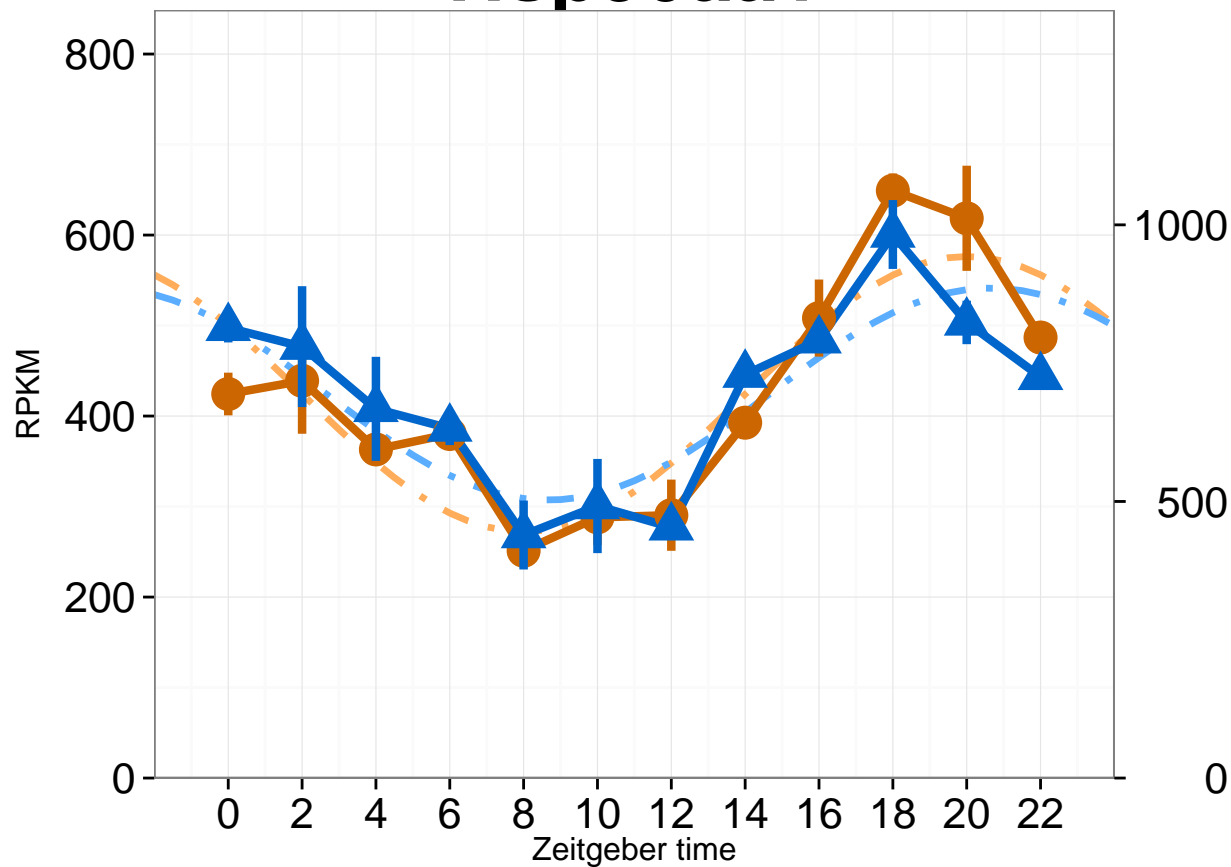

# Hsp90aa1

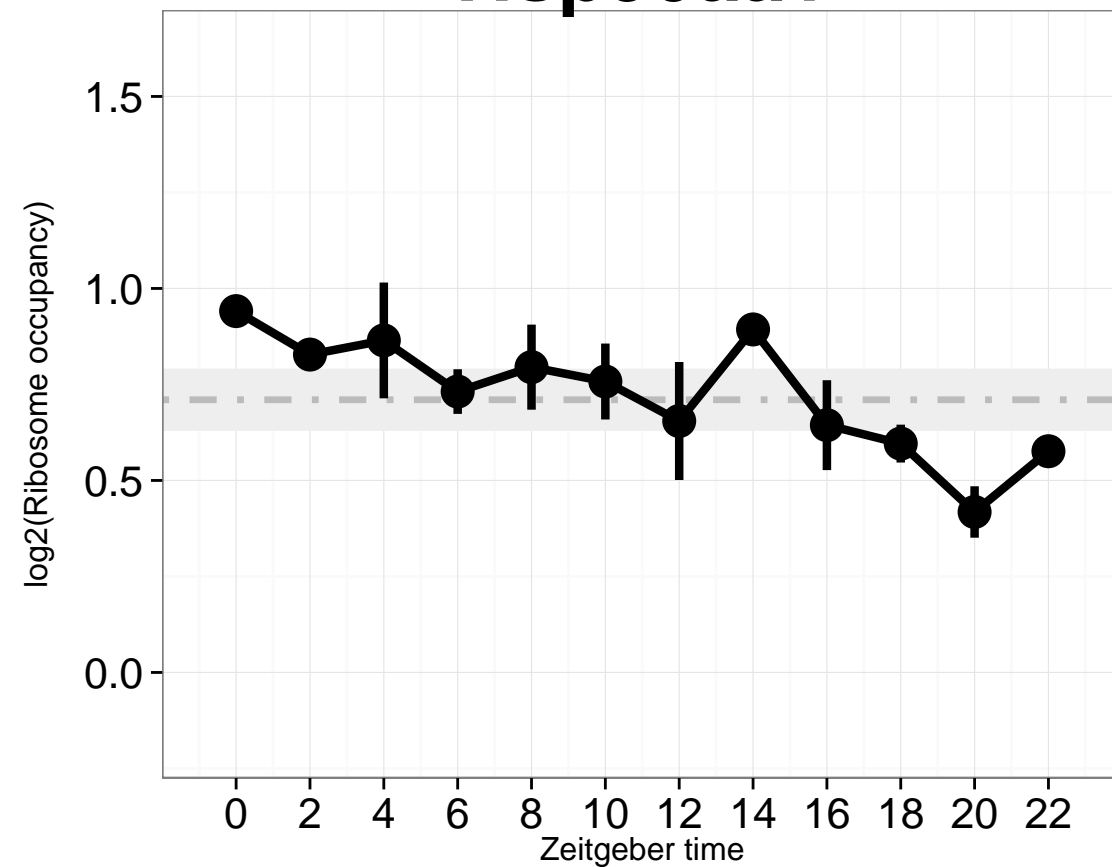

Supplement: Supplementary file 7 — Expression plots for kidney and liver for the 178 common rhythmic genes of Fig. 3c. (ZIP 3338.28 kb) [file 13059_2017_1222_MOESM7_ESM.zip › set_D_shared(178)/Hsp90aa1_kidney_set_D.pdf]

# Hsp90aa1

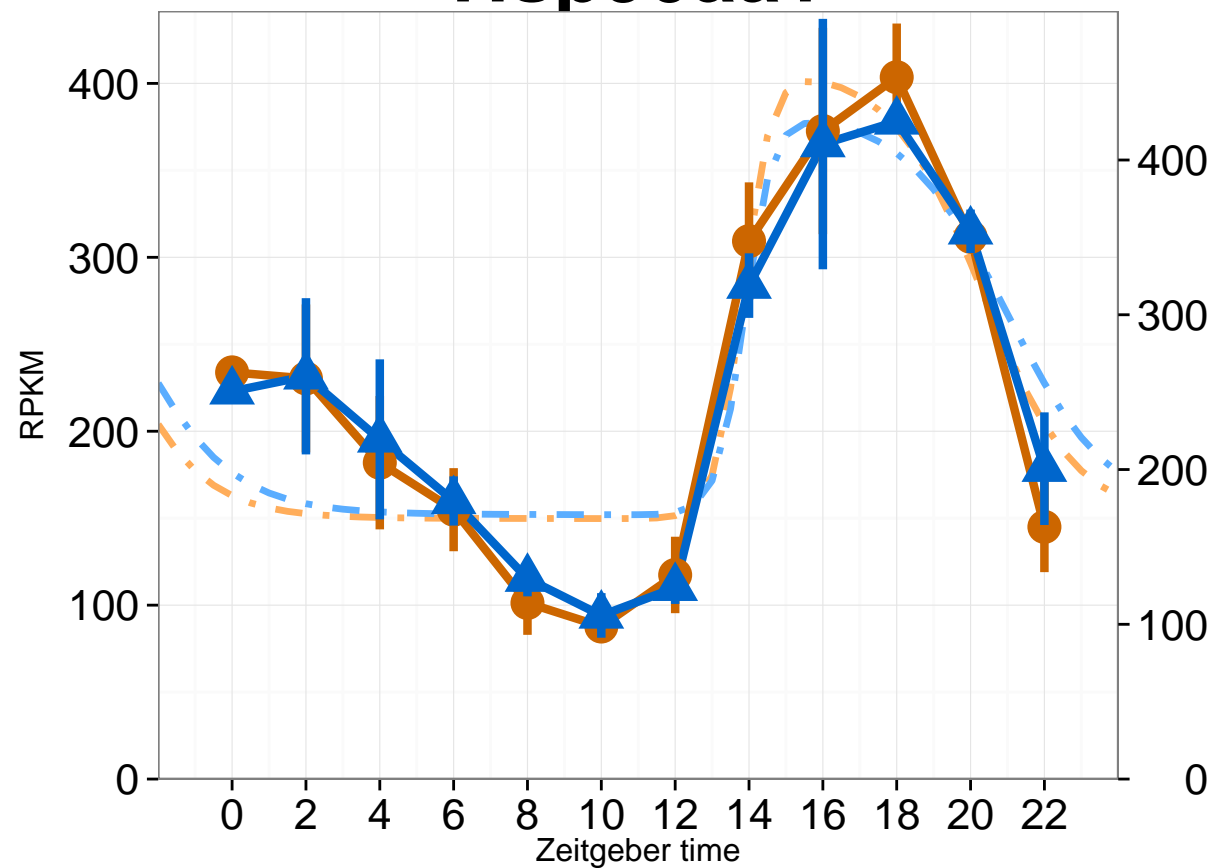

# Hsp90aa1

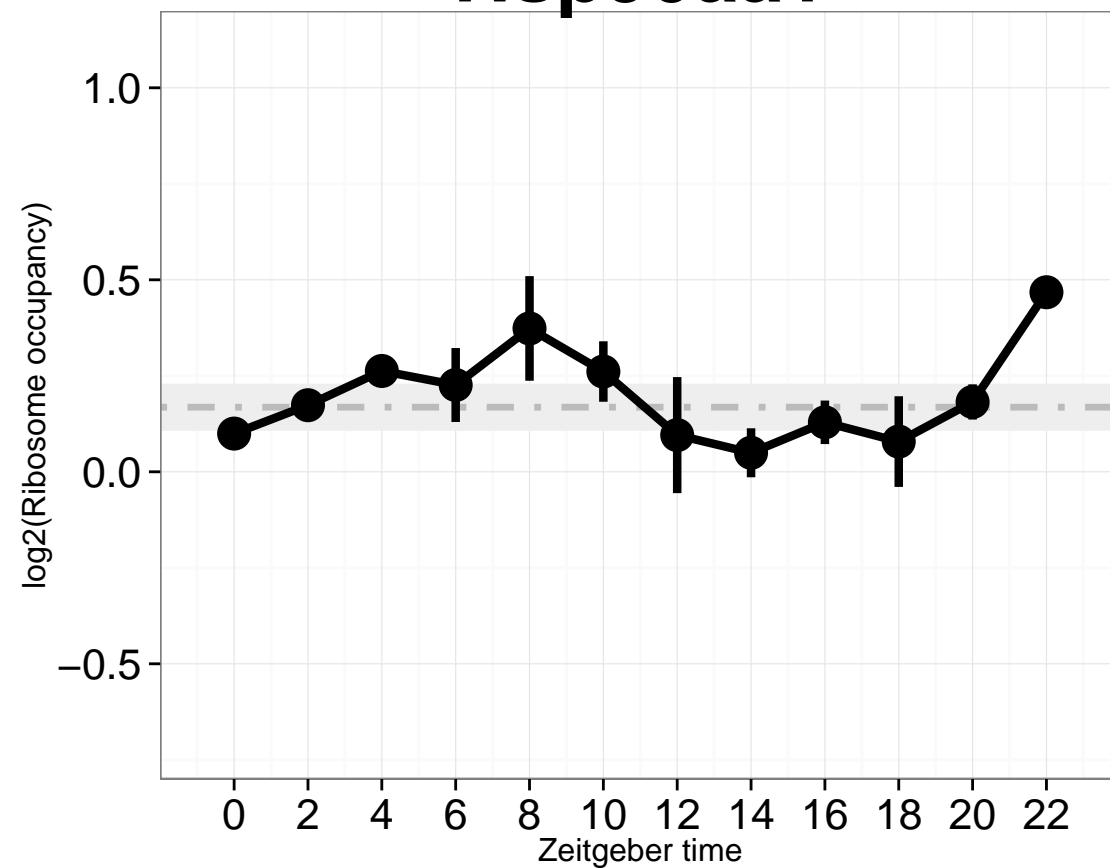

Supplement: Supplementary file 7 — Expression plots for kidney and liver for the 178 common rhythmic genes of Fig. 3c. (ZIP 3338.28 kb) [file 13059_2017_1222_MOESM7_ESM.zip › set_D_shared(178)/Hsp90aa1_liver_set_D.pdf]

# Hspa1b

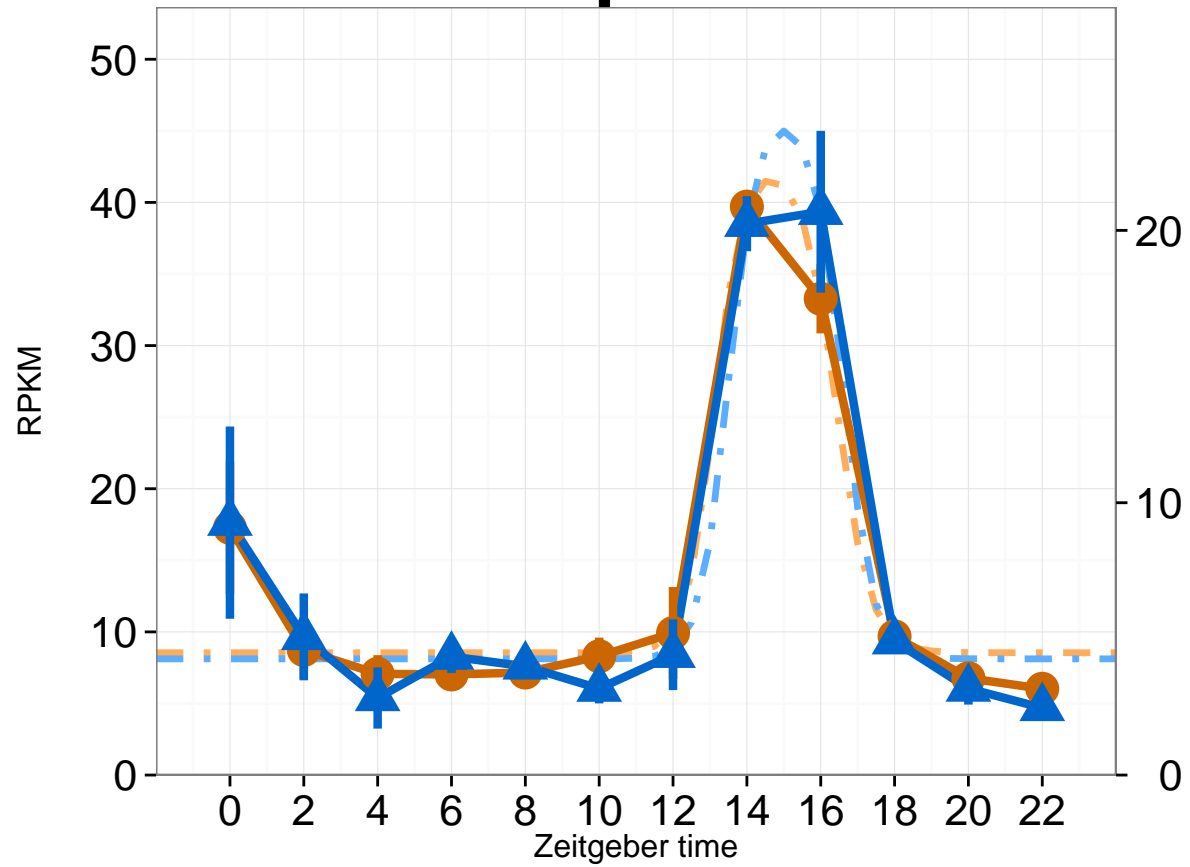

# Hspa1b

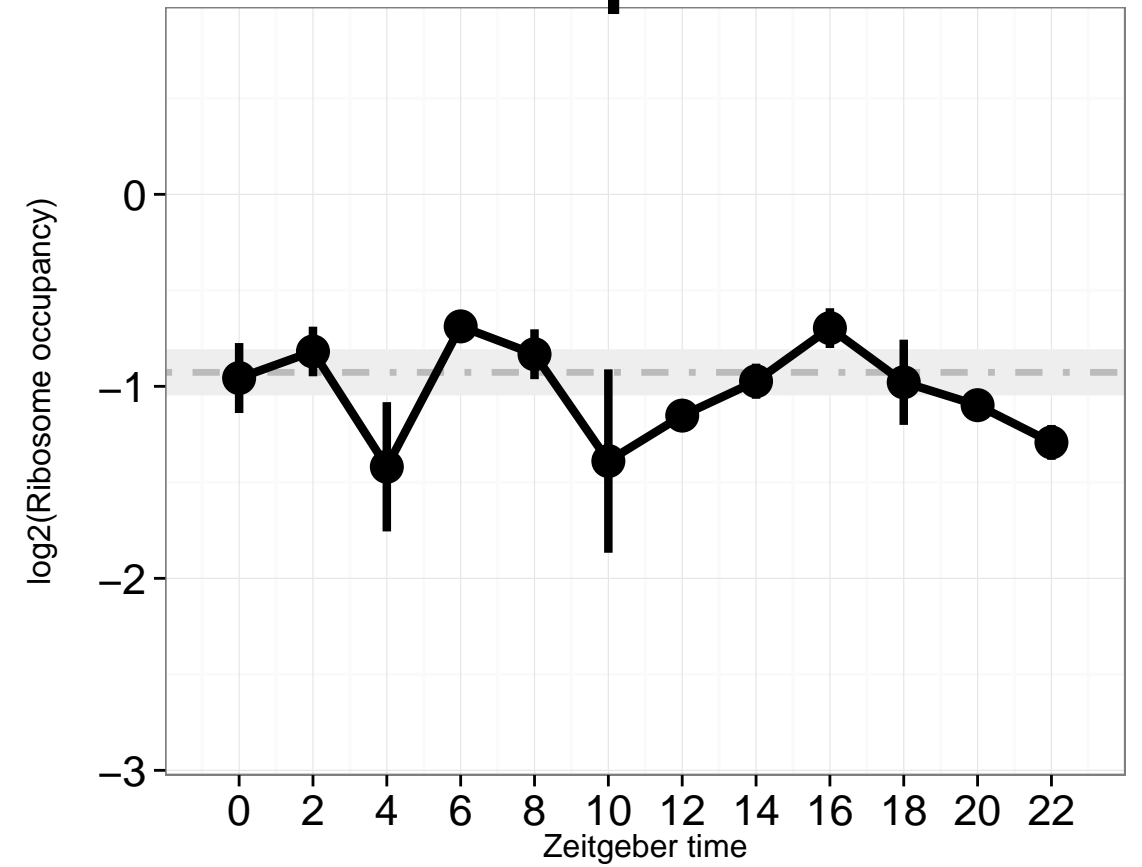

Supplement: Supplementary file 7 — Expression plots for kidney and liver for the 178 common rhythmic genes of Fig. 3c. (ZIP 3338.28 kb) [file 13059_2017_1222_MOESM7_ESM.zip › set_D_shared(178)/Hspa1b_kidney_set_D.pdf]

# Hspa1b

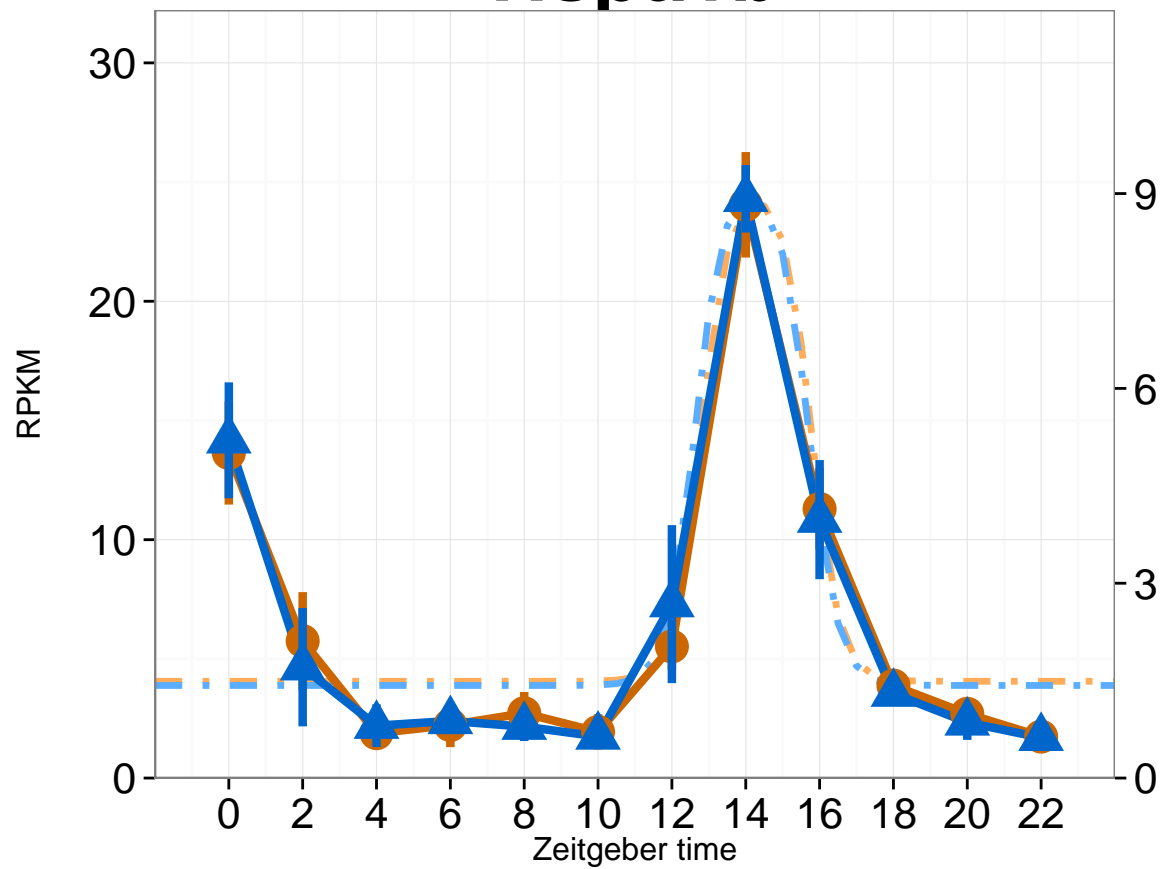

# Hspa1b

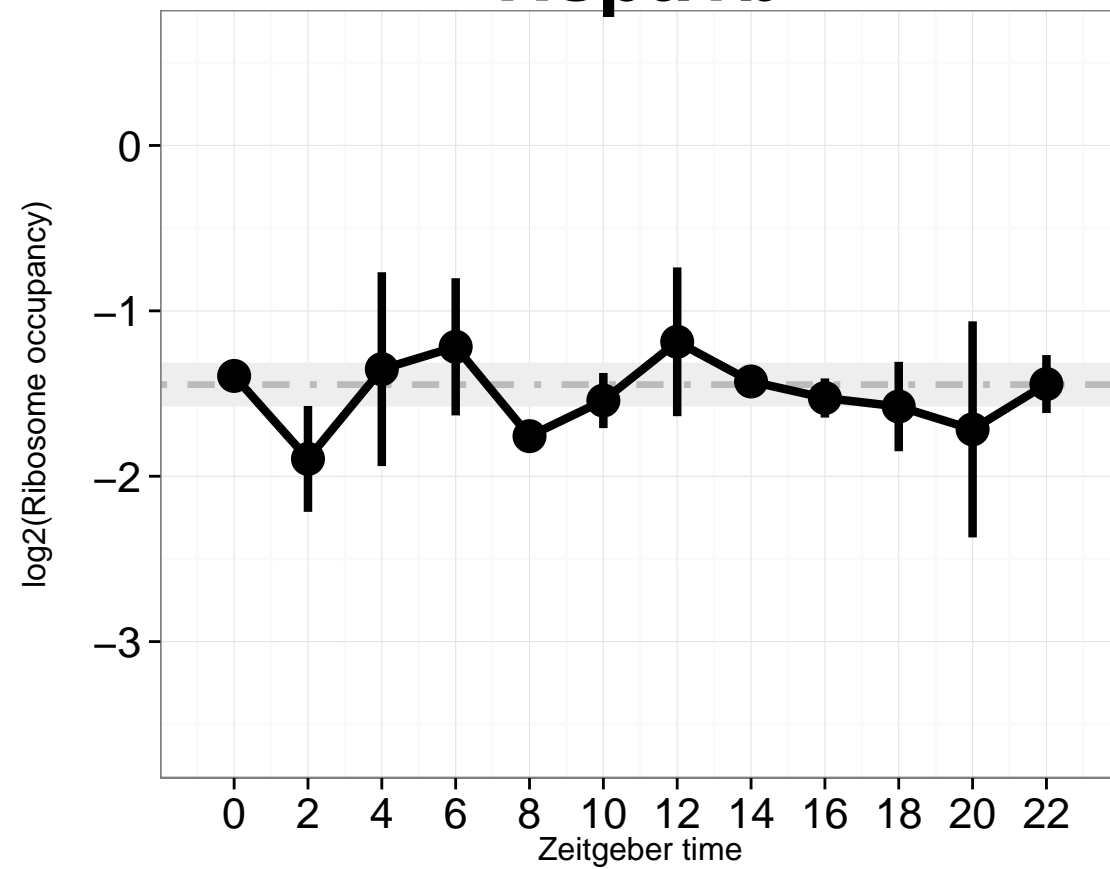

Supplement: Supplementary file 7 — Expression plots for kidney and liver for the 178 common rhythmic genes of Fig. 3c. (ZIP 3338.28 kb) [file 13059_2017_1222_MOESM7_ESM.zip › set_D_shared(178)/Hspa1b_liver_set_D.pdf]

# Hspa2

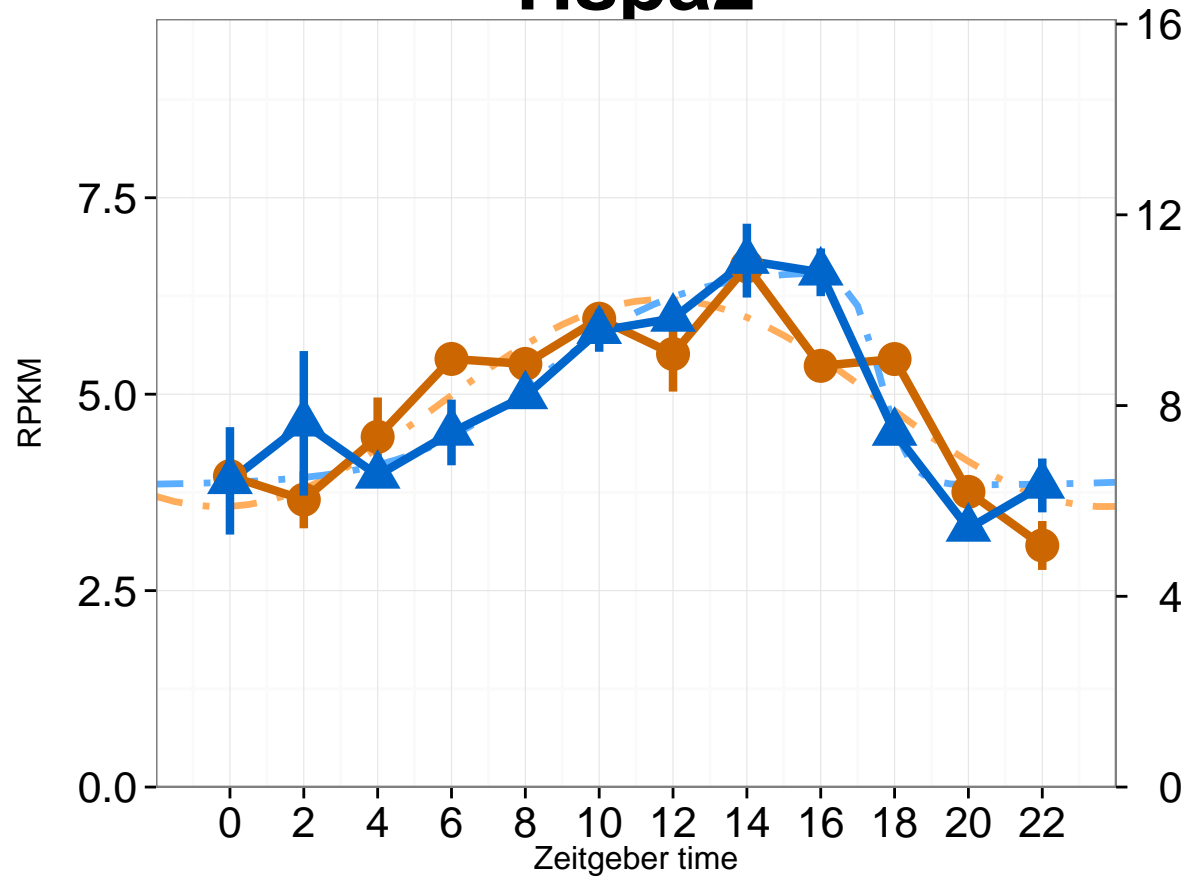

# Hspa2

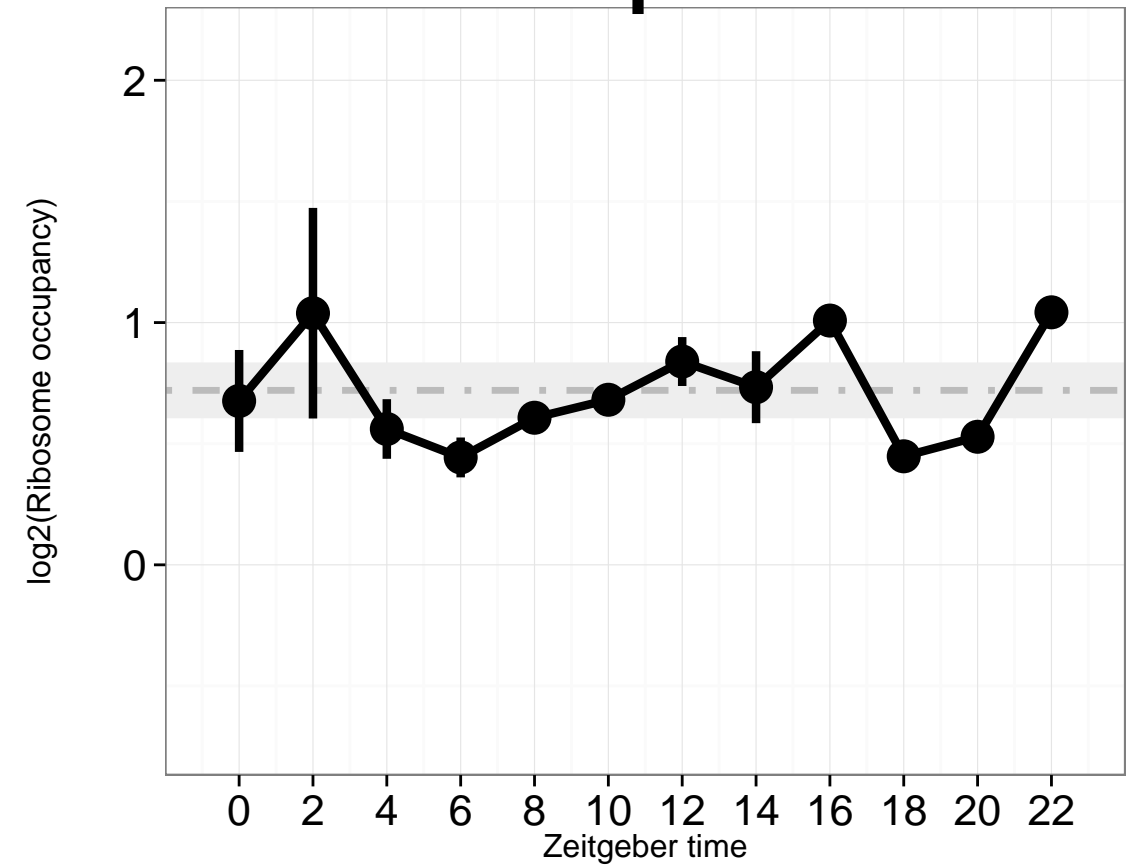

Supplement: Supplementary file 7 — Expression plots for kidney and liver for the 178 common rhythmic genes of Fig. 3c. (ZIP 3338.28 kb) [file 13059_2017_1222_MOESM7_ESM.zip › set_D_shared(178)/Hspa2_kidney_set_D.pdf]

# Hspa2

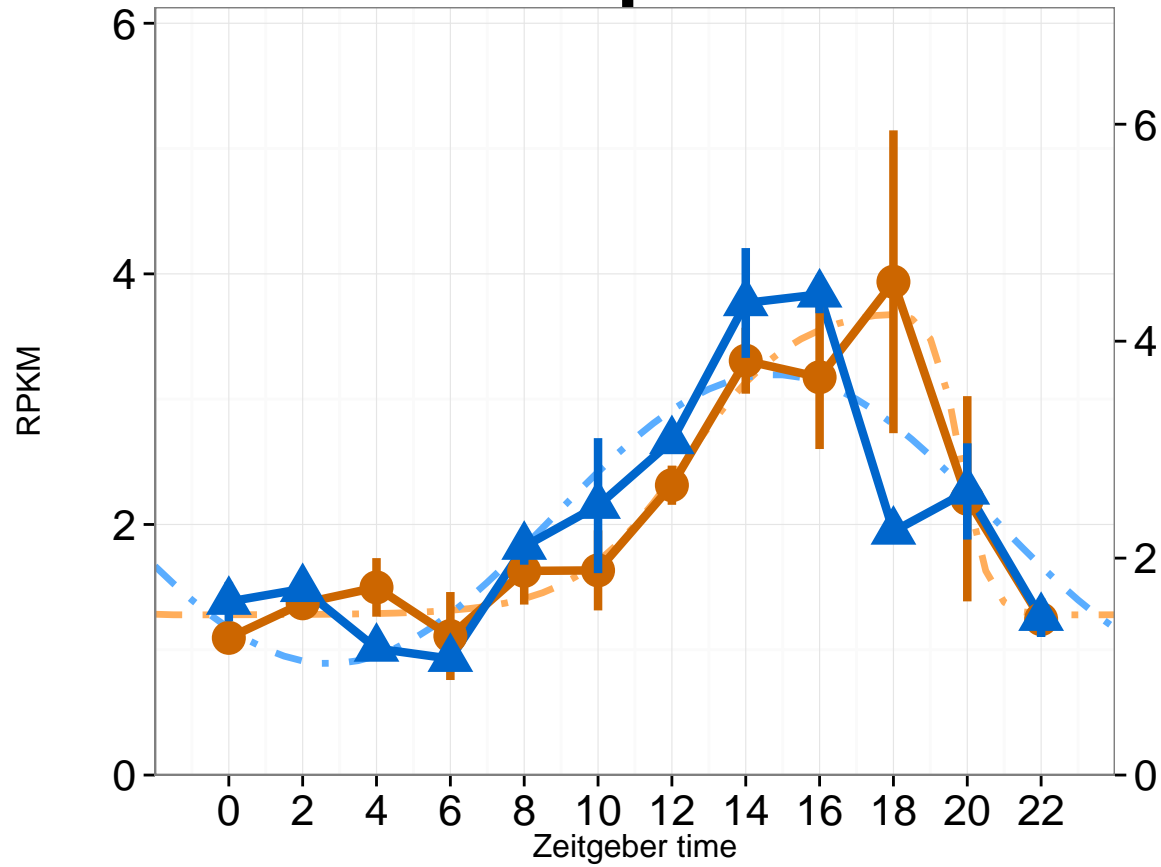

# Hspa2

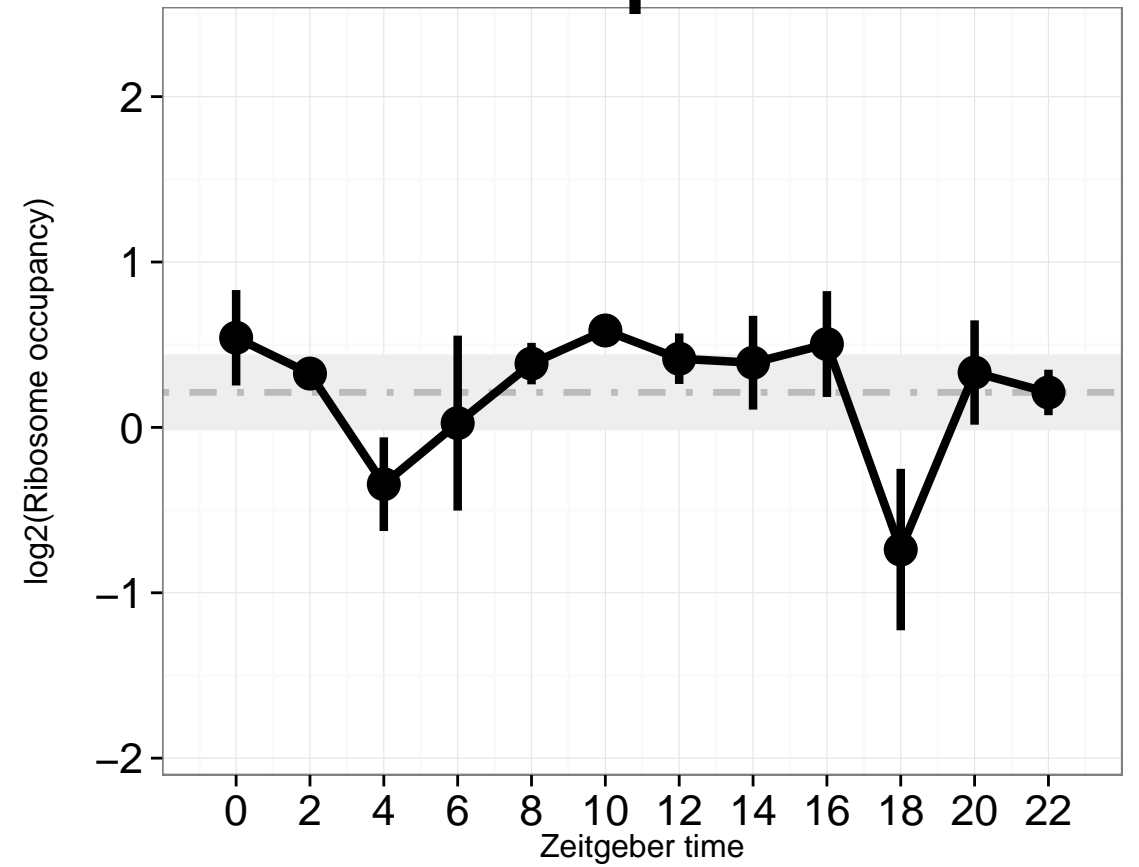

Supplement: Supplementary file 7 — Expression plots for kidney and liver for the 178 common rhythmic genes of Fig. 3c. (ZIP 3338.28 kb) [file 13059_2017_1222_MOESM7_ESM.zip › set_D_shared(178)/Hspa2_liver_set_D.pdf]

# Hspa8

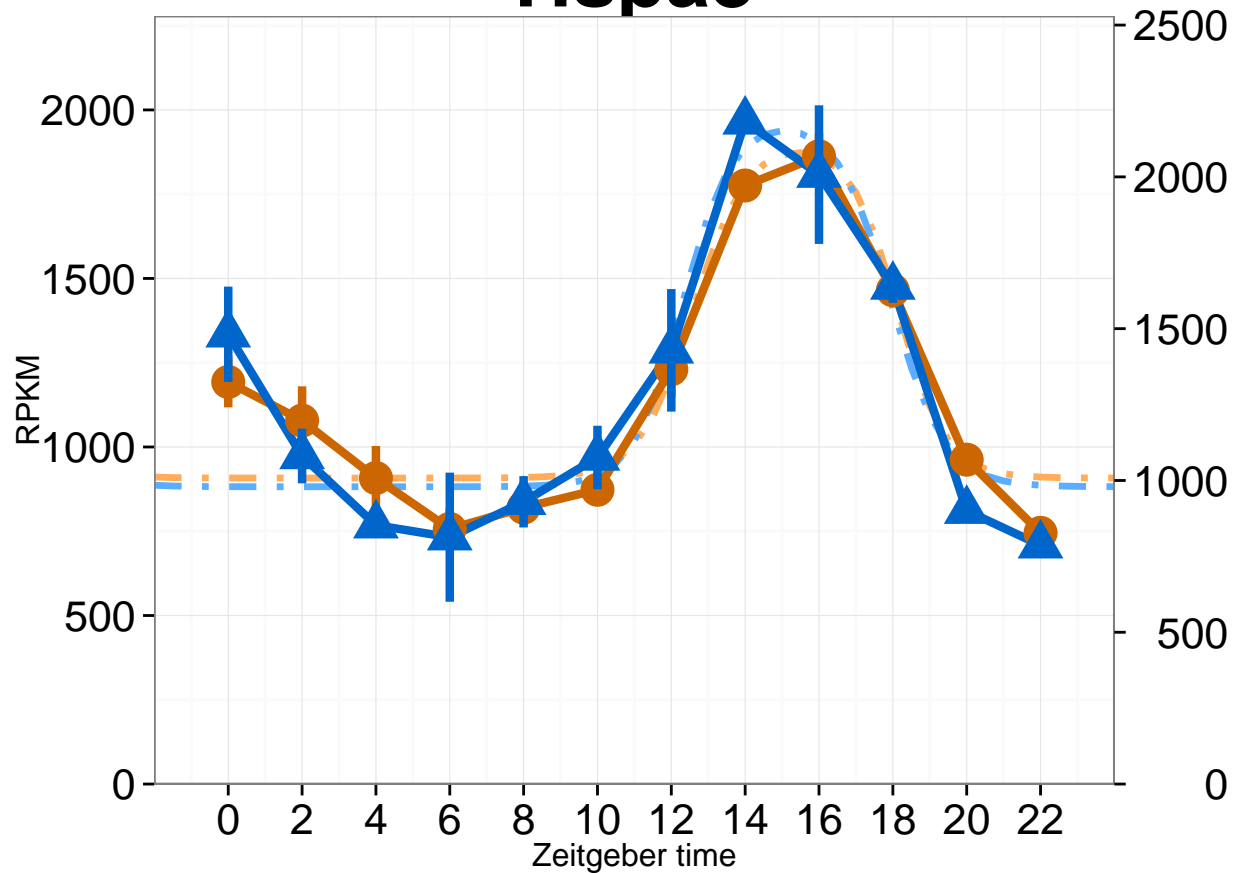

# Hspa8

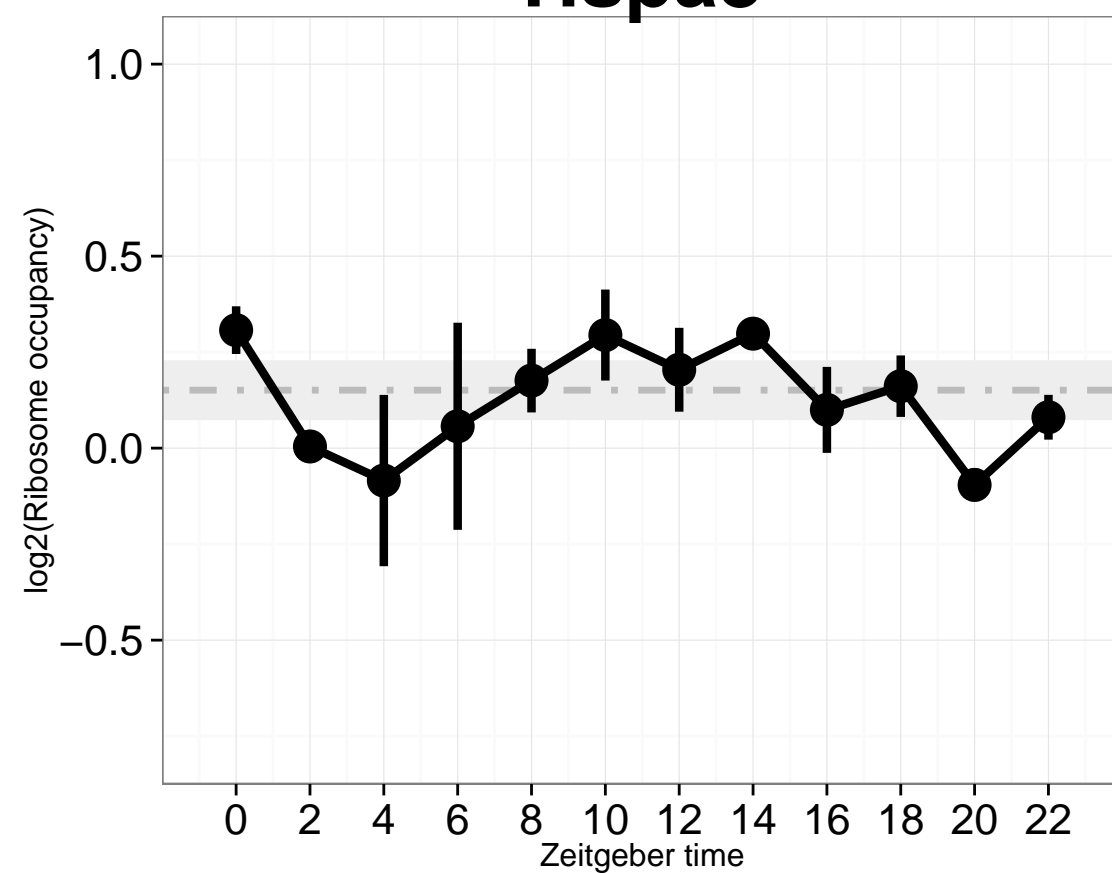

Supplement: Supplementary file 7 — Expression plots for kidney and liver for the 178 common rhythmic genes of Fig. 3c. (ZIP 3338.28 kb) [file 13059_2017_1222_MOESM7_ESM.zip › set_D_shared(178)/Hspa8_kidney_set_D.pdf]

# Hspa8

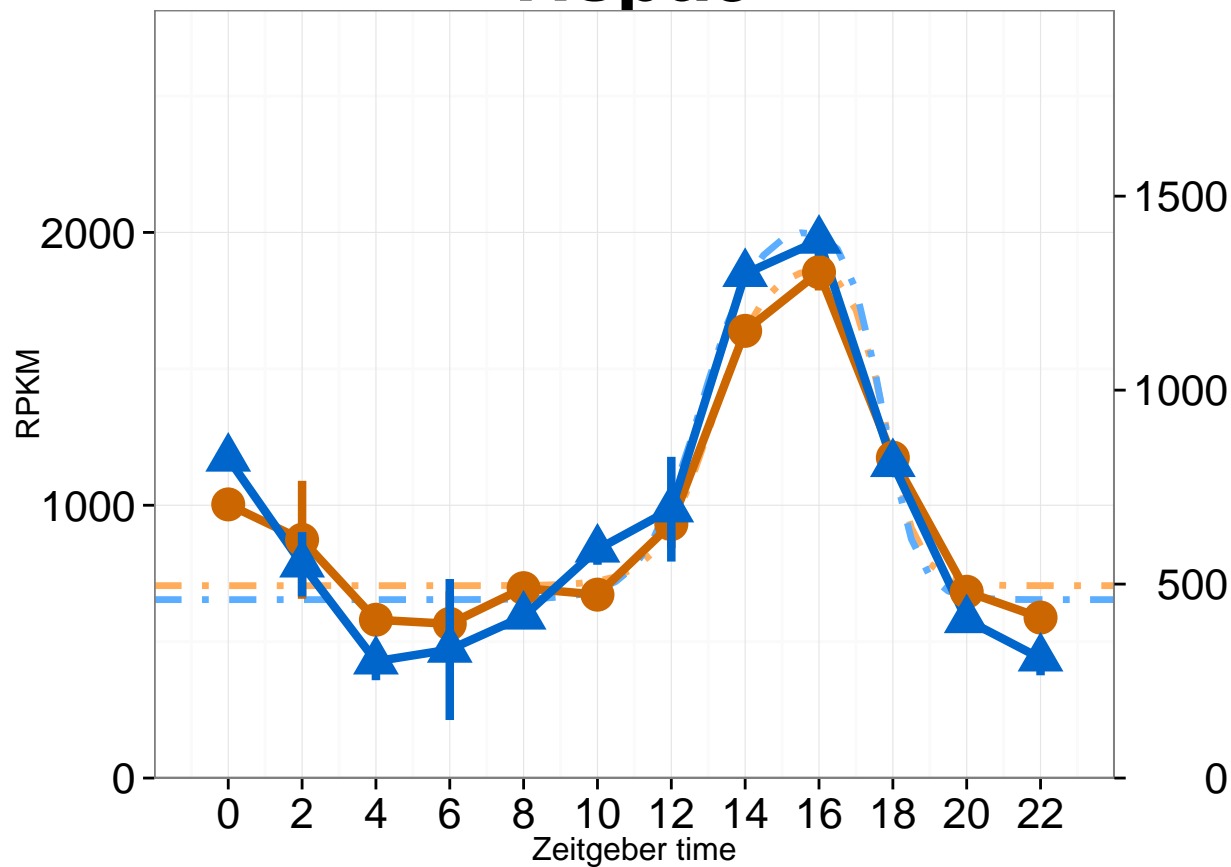

# Hspa8

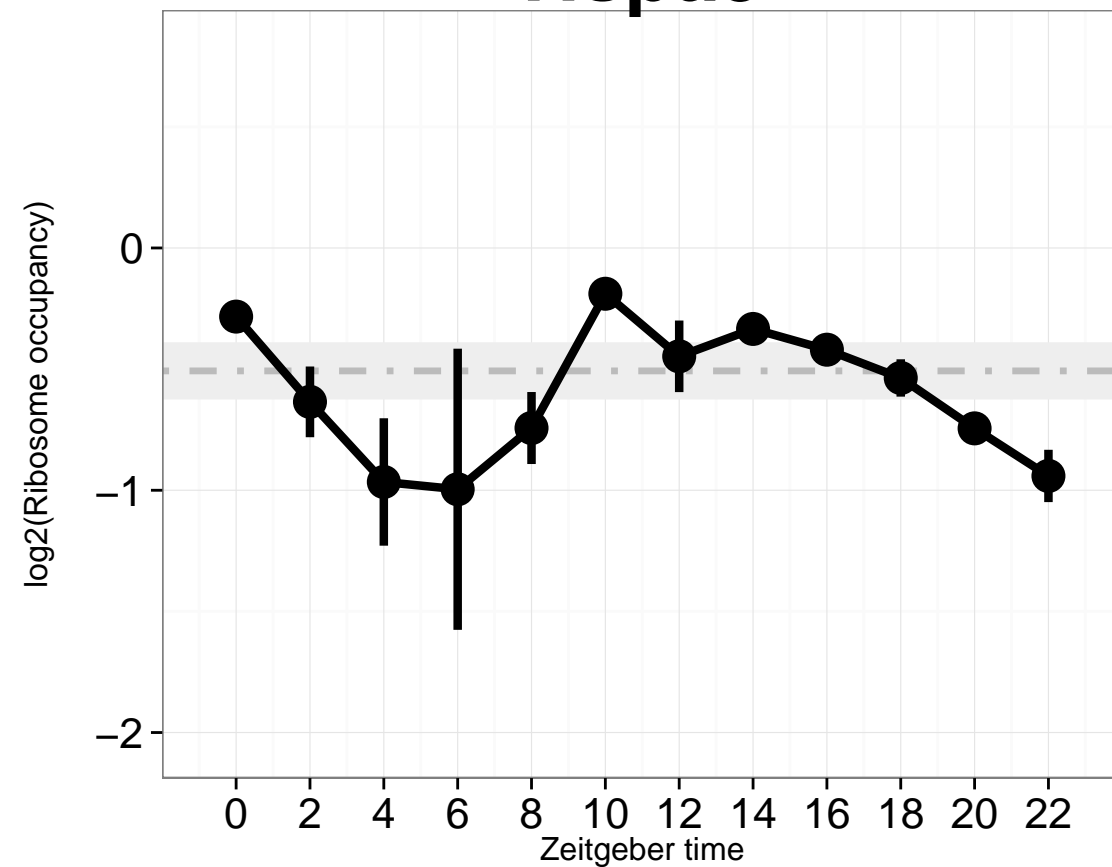

Supplement: Supplementary file 7 — Expression plots for kidney and liver for the 178 common rhythmic genes of Fig. 3c. (ZIP 3338.28 kb) [file 13059_2017_1222_MOESM7_ESM.zip › set_D_shared(178)/Hspa8_liver_set_D.pdf]

## Hsph1

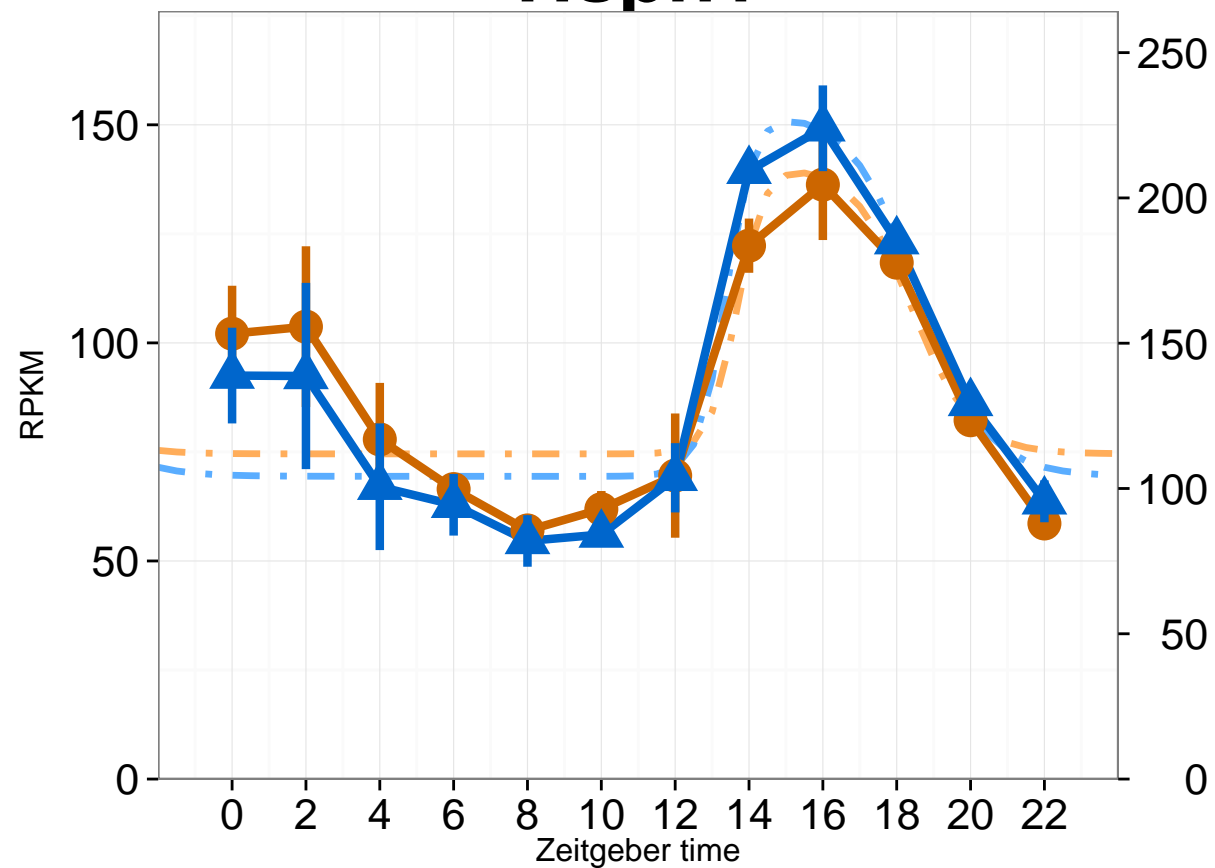

## Hsph1

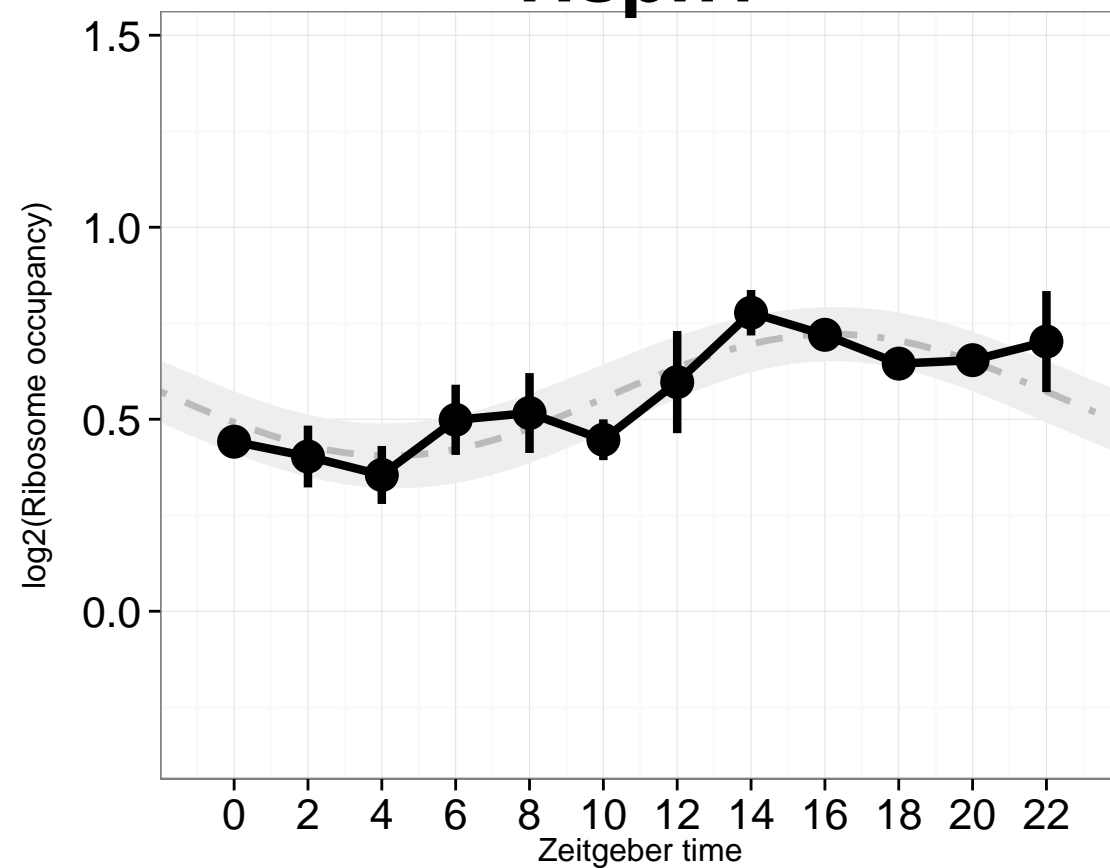

Supplement: Supplementary file 7 — Expression plots for kidney and liver for the 178 common rhythmic genes of Fig. 3c. (ZIP 3338.28 kb) [file 13059_2017_1222_MOESM7_ESM.zip › set_D_shared(178)/Hsph1_kidney_set_D.pdf]

# Hsph1

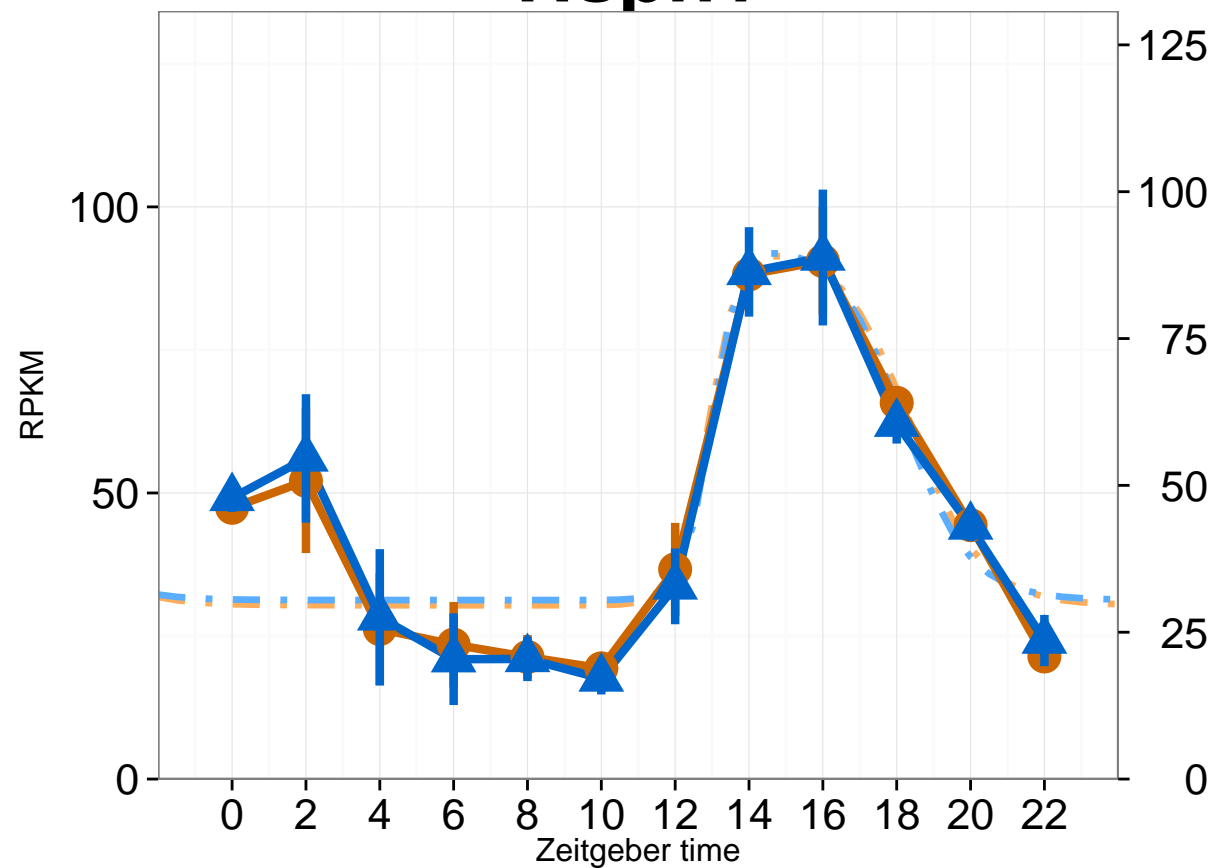

# Hsph1

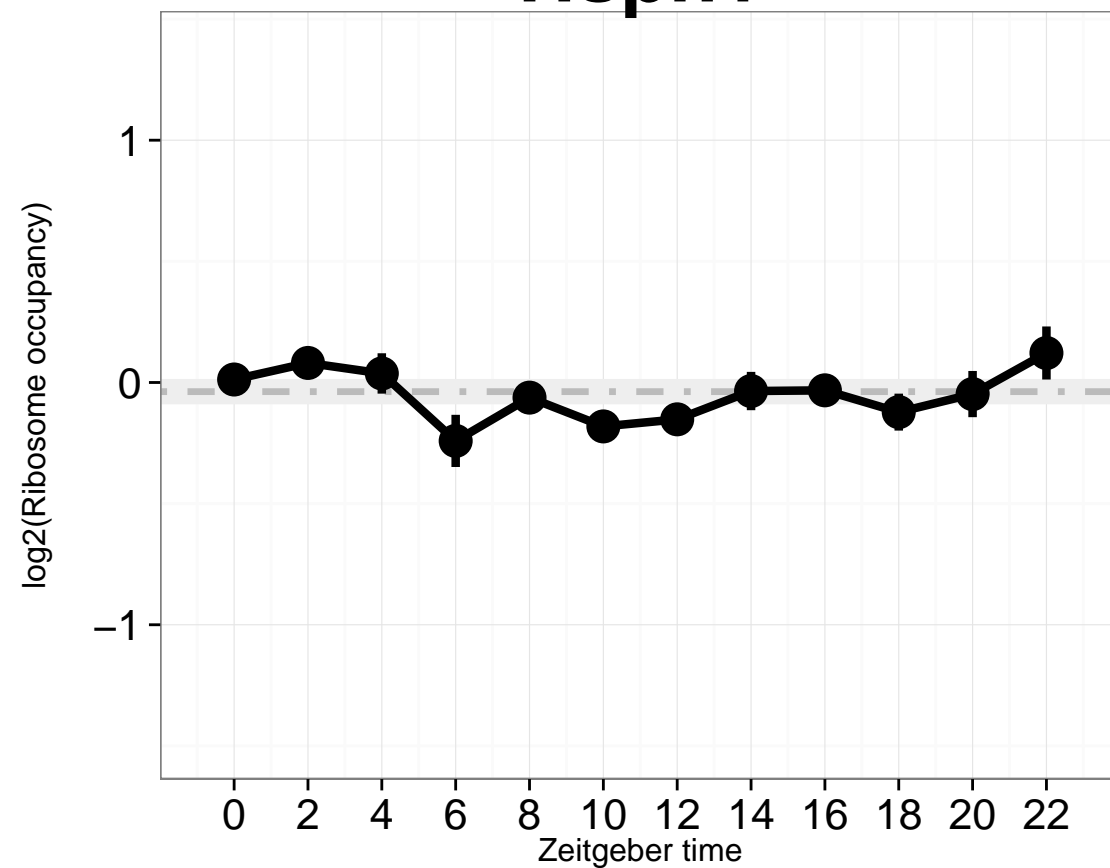

Supplement: Supplementary file 7 — Expression plots for kidney and liver for the 178 common rhythmic genes of Fig. 3c. (ZIP 3338.28 kb) [file 13059_2017_1222_MOESM7_ESM.zip › set_D_shared(178)/Hsph1_liver_set_D.pdf]

# Itga8

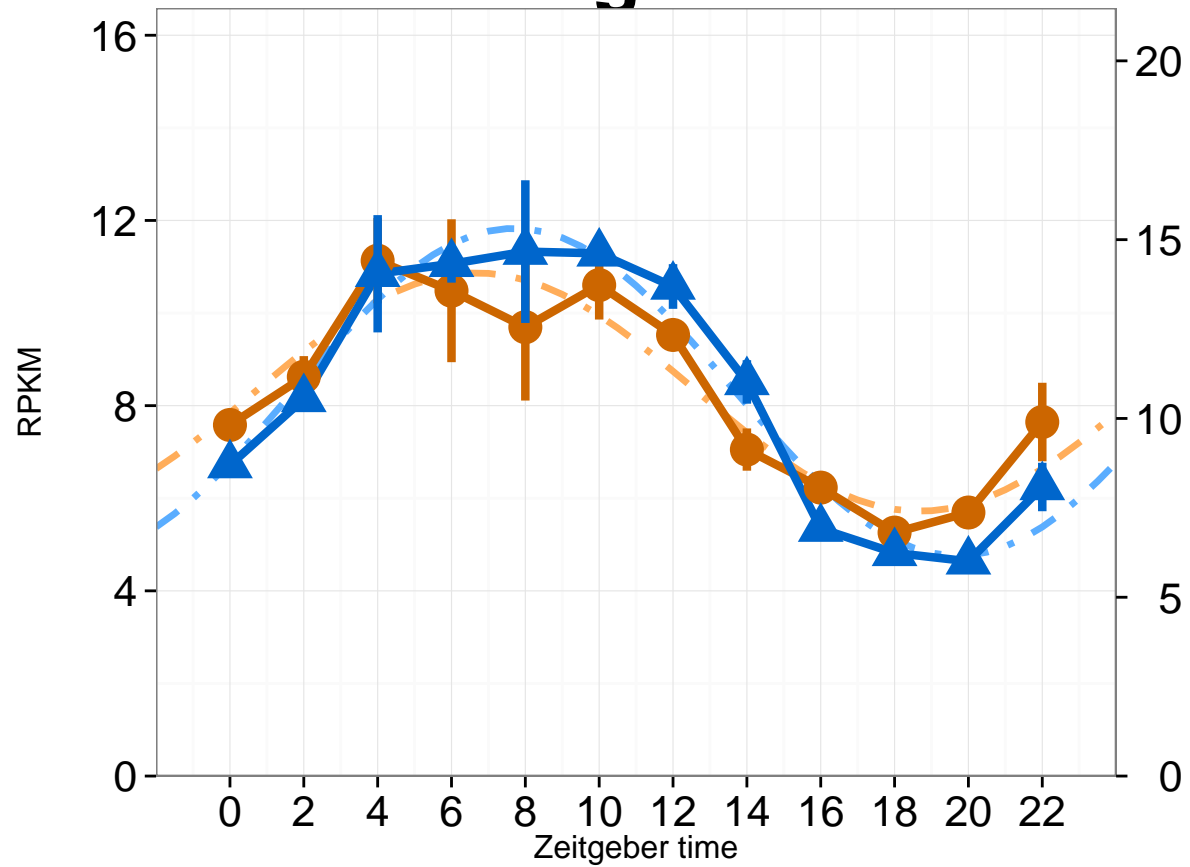

# Itga8

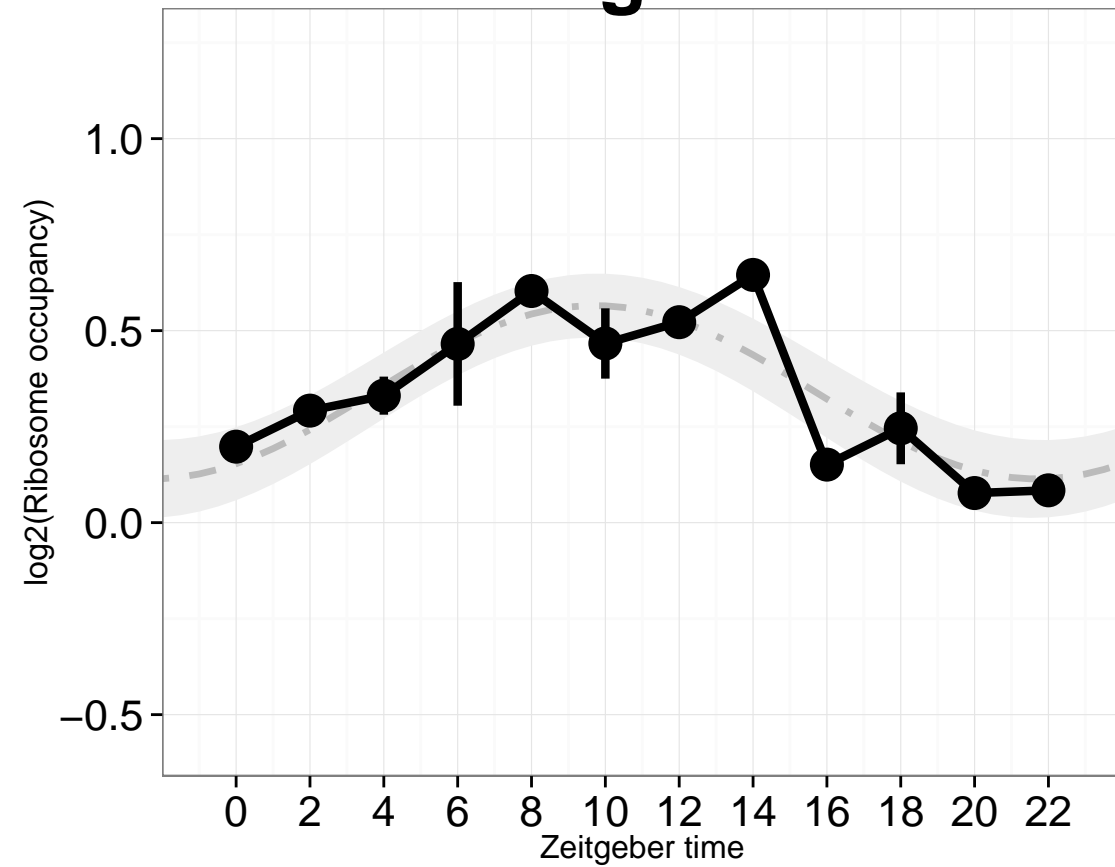

Supplement: Supplementary file 7 — Expression plots for kidney and liver for the 178 common rhythmic genes of Fig. 3c. (ZIP 3338.28 kb) [file 13059_2017_1222_MOESM7_ESM.zip › set_D_shared(178)/Itga8_kidney_set_D.pdf]

# Itga8

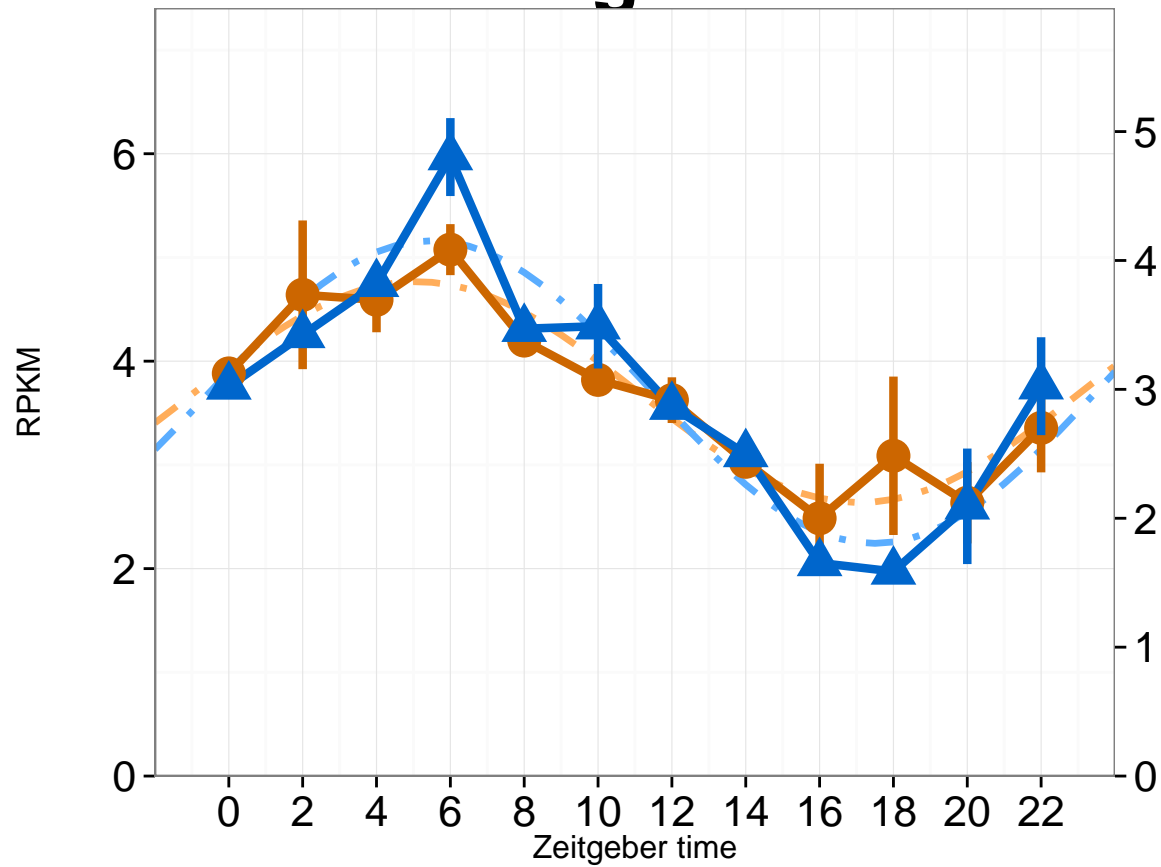

# Itga8

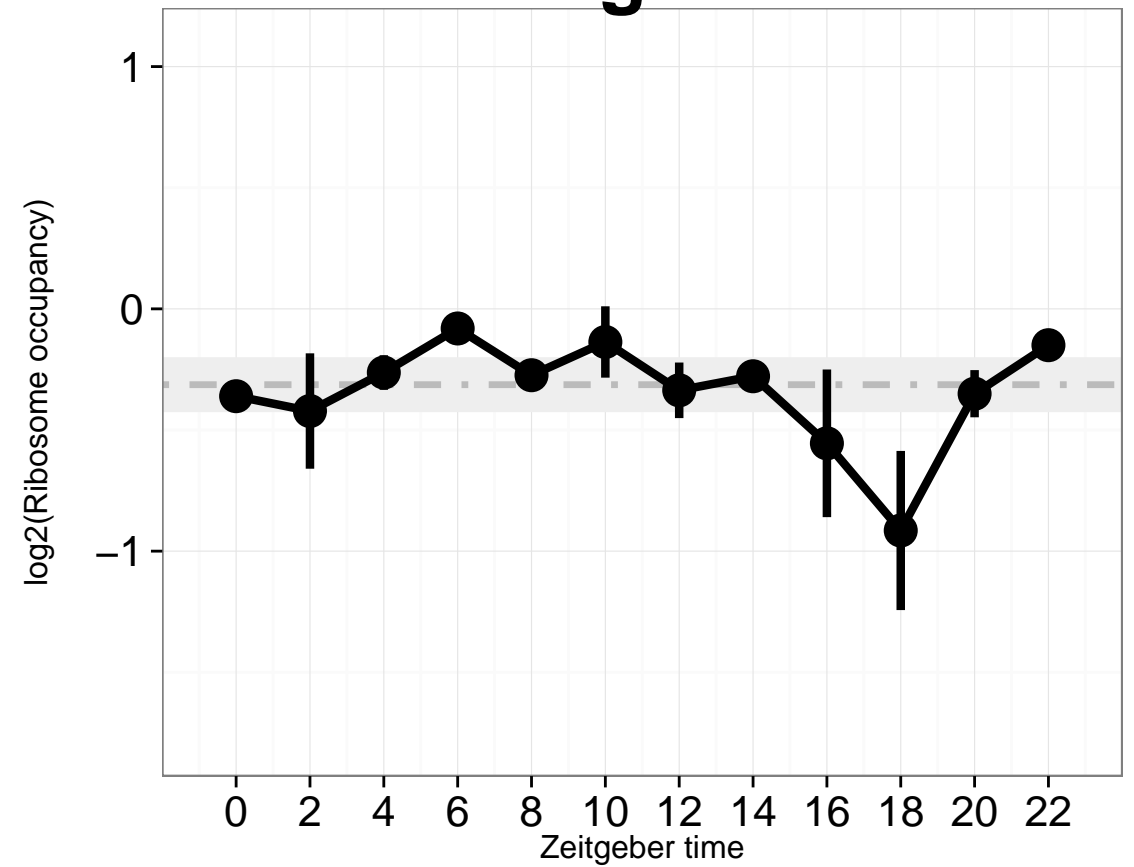

Supplement: Supplementary file 7 — Expression plots for kidney and liver for the 178 common rhythmic genes of Fig. 3c. (ZIP 3338.28 kb) [file 13059_2017_1222_MOESM7_ESM.zip › set_D_shared(178)/Itga8_liver_set_D.pdf]

## Junb

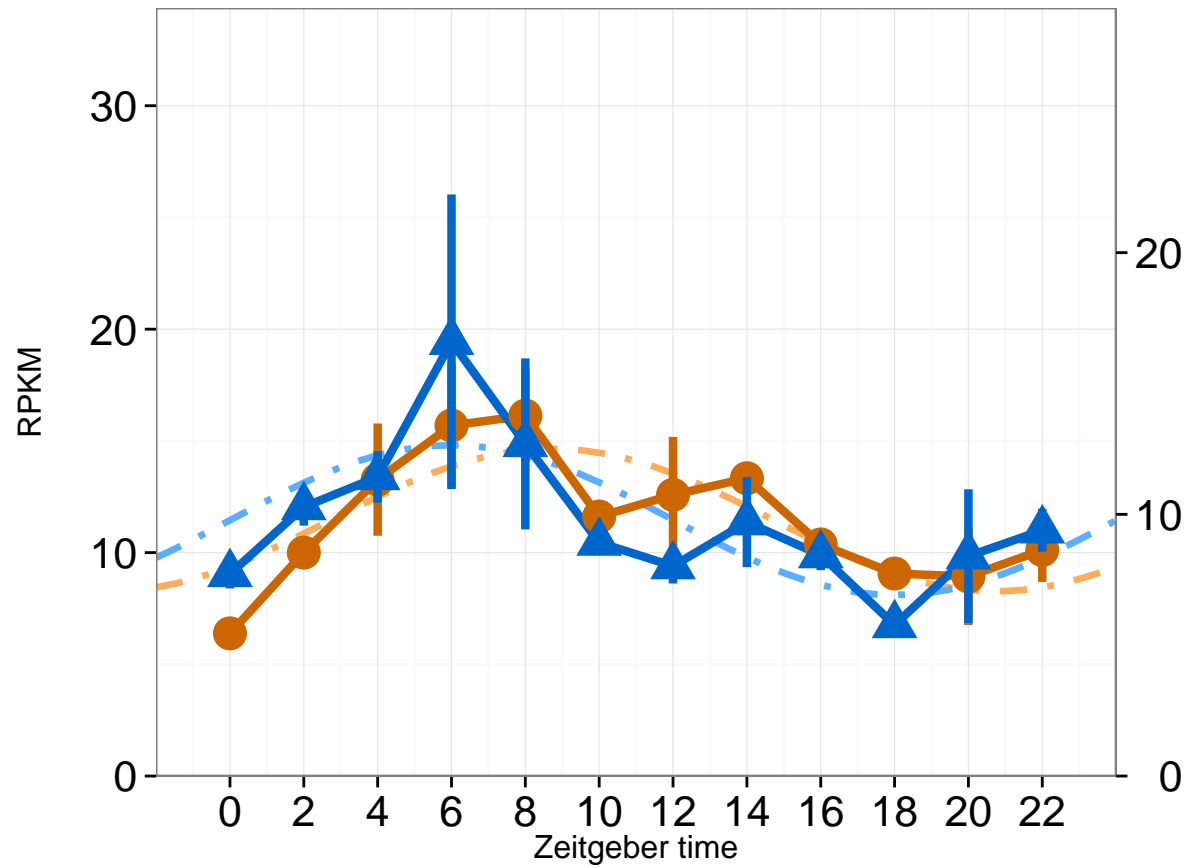

## Junb

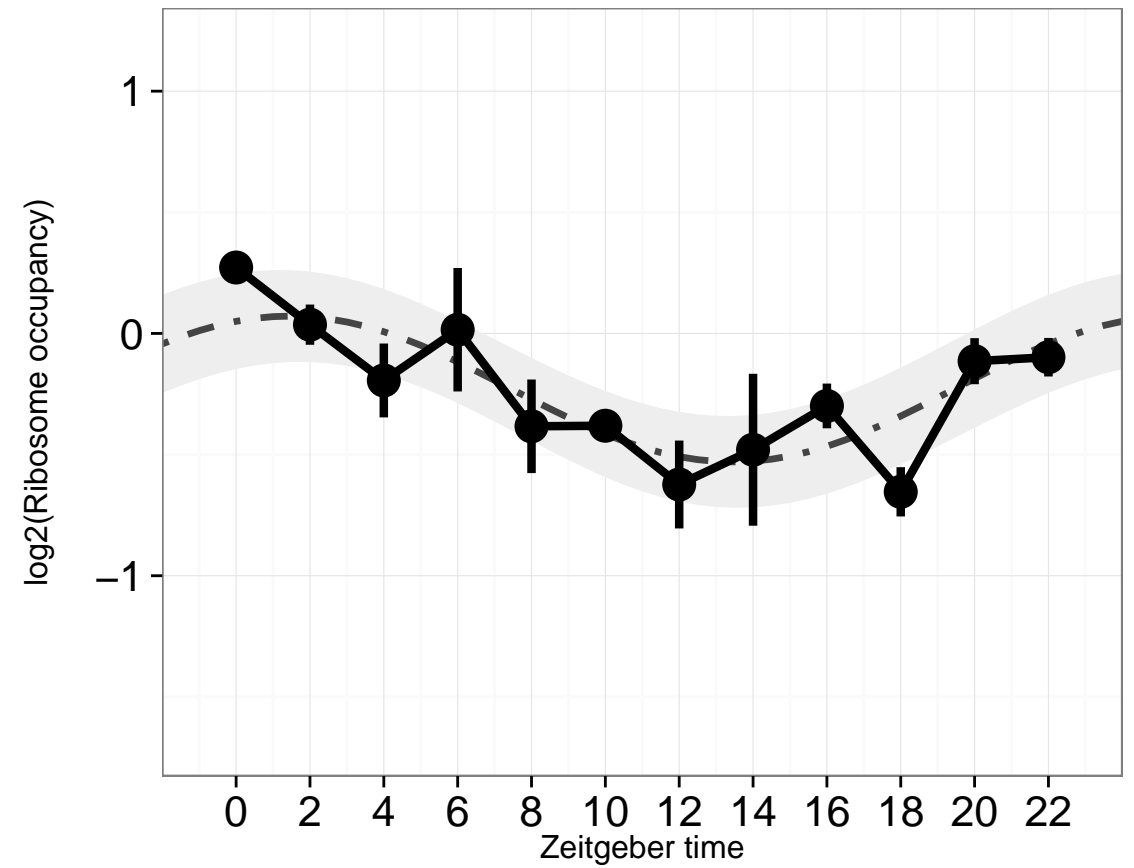

Supplement: Supplementary file 7 — Expression plots for kidney and liver for the 178 common rhythmic genes of Fig. 3c. (ZIP 3338.28 kb) [file 13059_2017_1222_MOESM7_ESM.zip › set_D_shared(178)/Junb_kidney_set_D.pdf]

## Junb

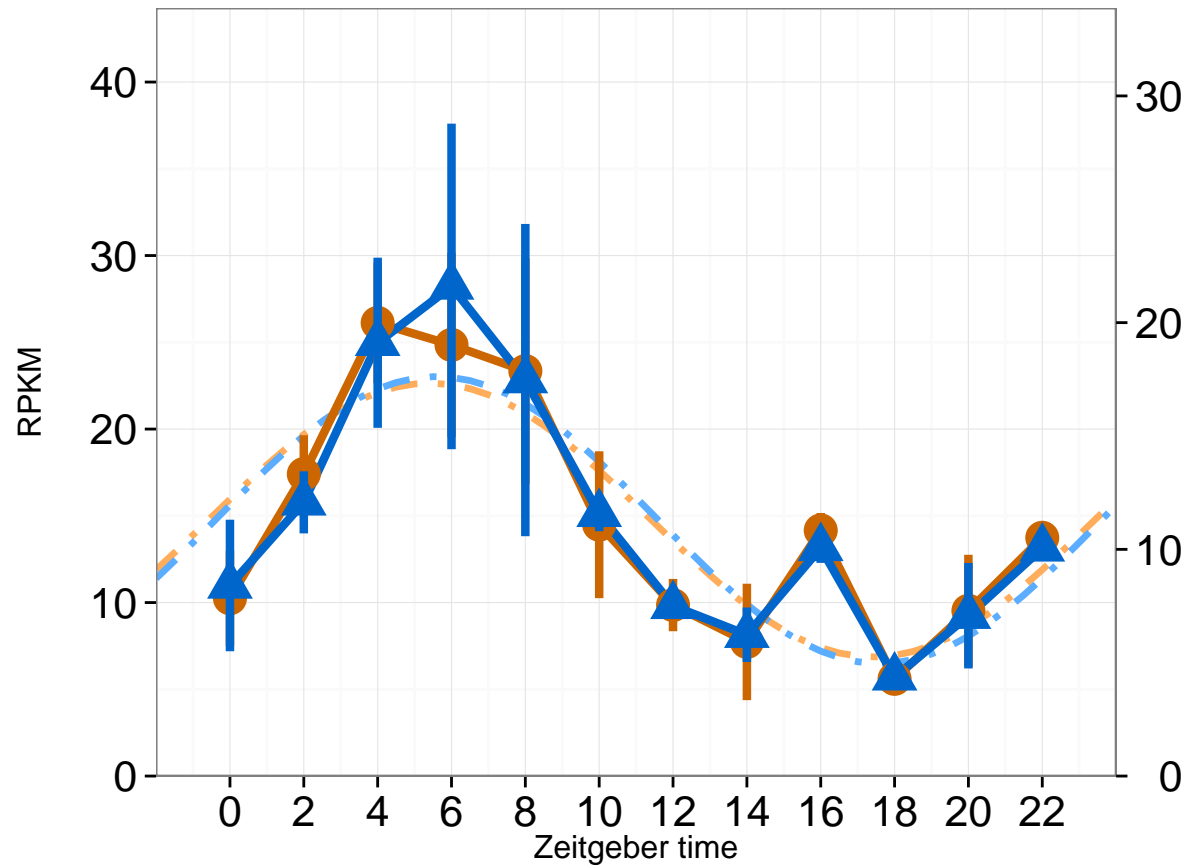

## Junb

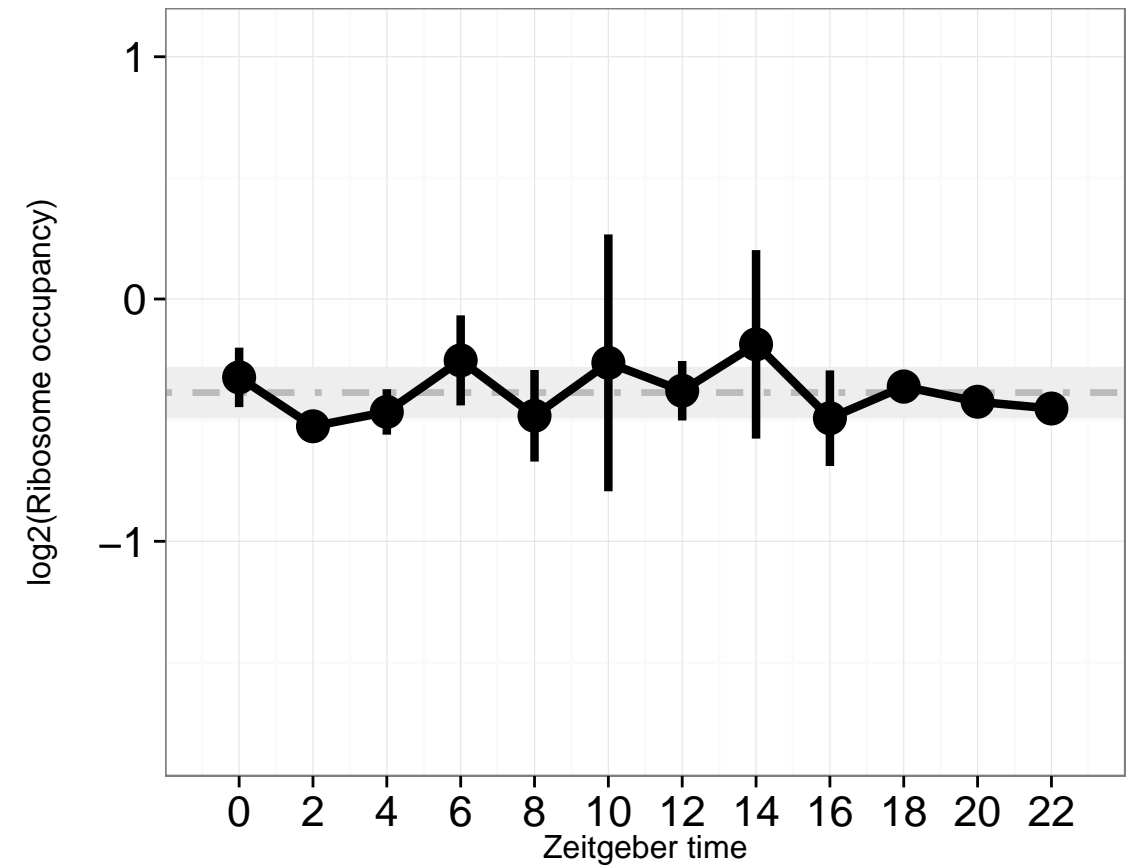

Supplement: Supplementary file 7 — Expression plots for kidney and liver for the 178 common rhythmic genes of Fig. 3c. (ZIP 3338.28 kb) [file 13059_2017_1222_MOESM7_ESM.zip › set_D_shared(178)/Junb_liver_set_D.pdf]

# Kbtbd8

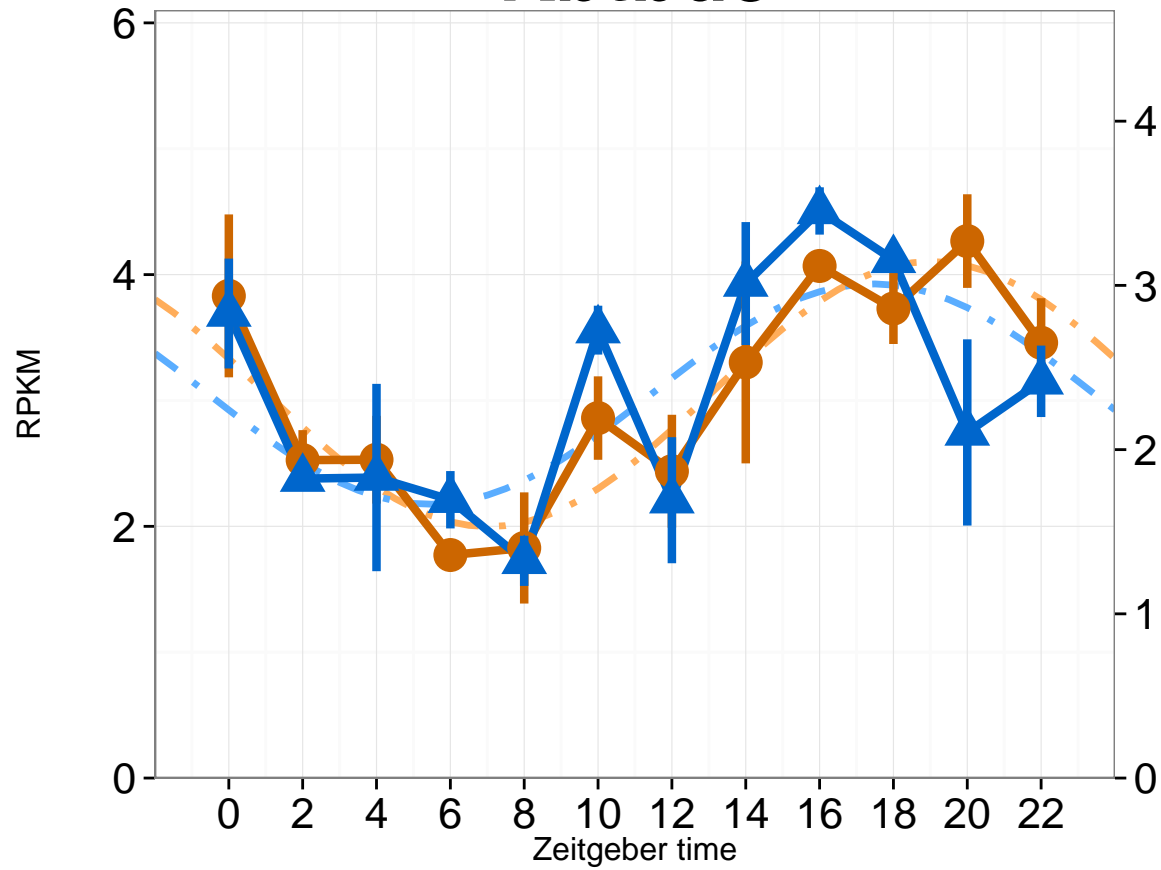

# Kbtbd8

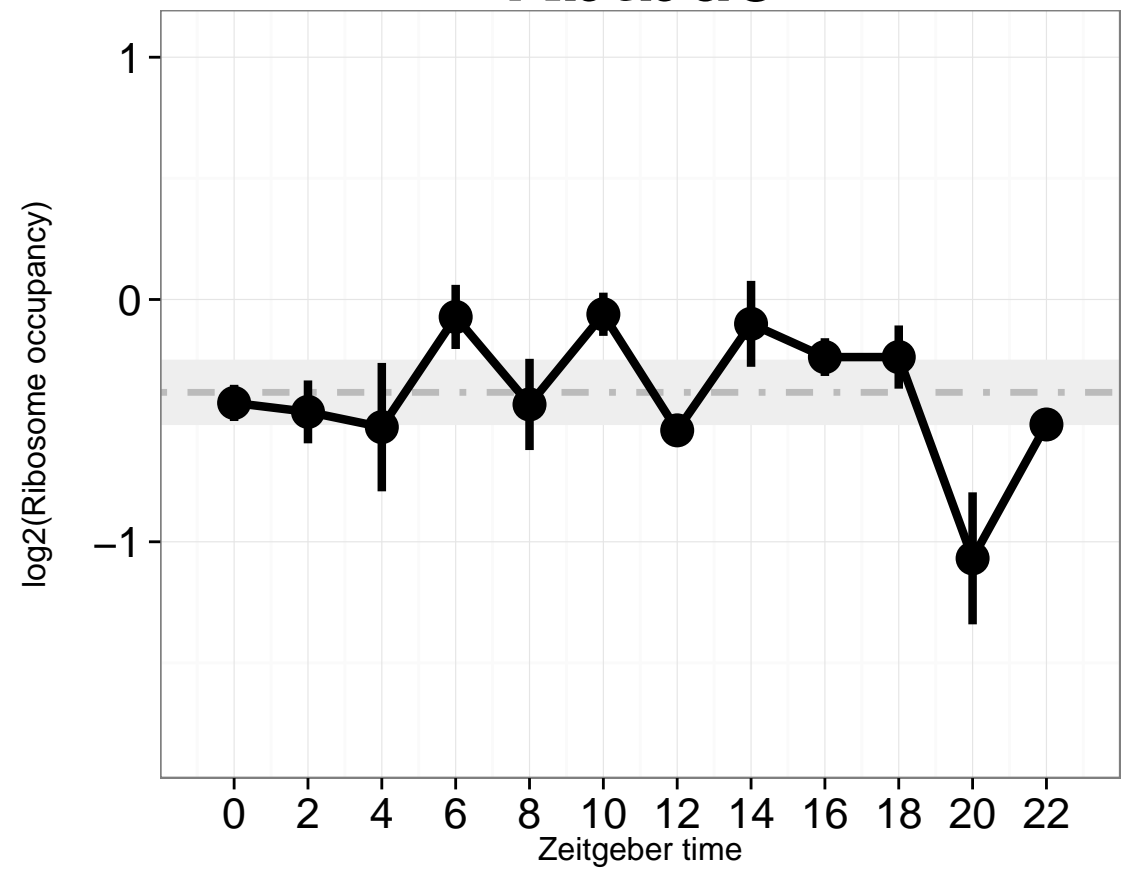

Supplement: Supplementary file 7 — Expression plots for kidney and liver for the 178 common rhythmic genes of Fig. 3c. (ZIP 3338.28 kb) [file 13059_2017_1222_MOESM7_ESM.zip › set_D_shared(178)/Kbtbd8_kidney_set_D.pdf]

# Kbtbd8

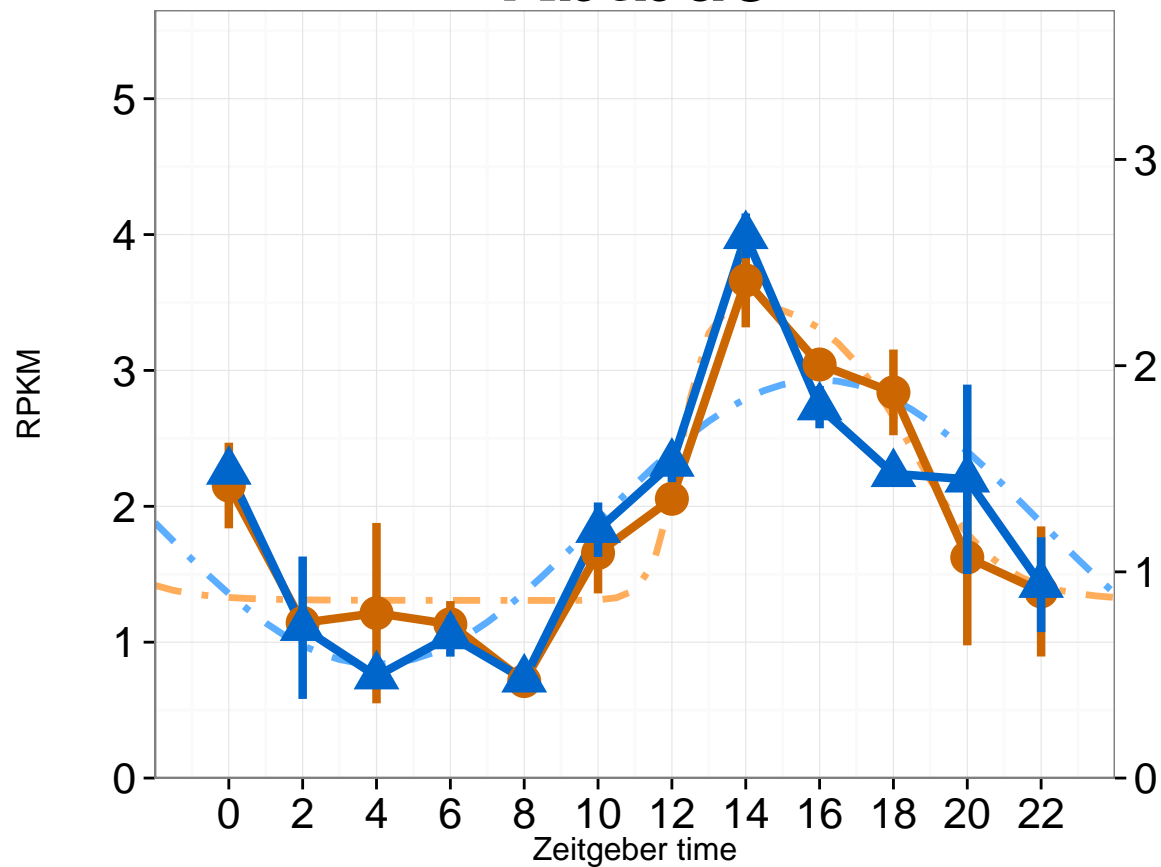

# Kbtbd8

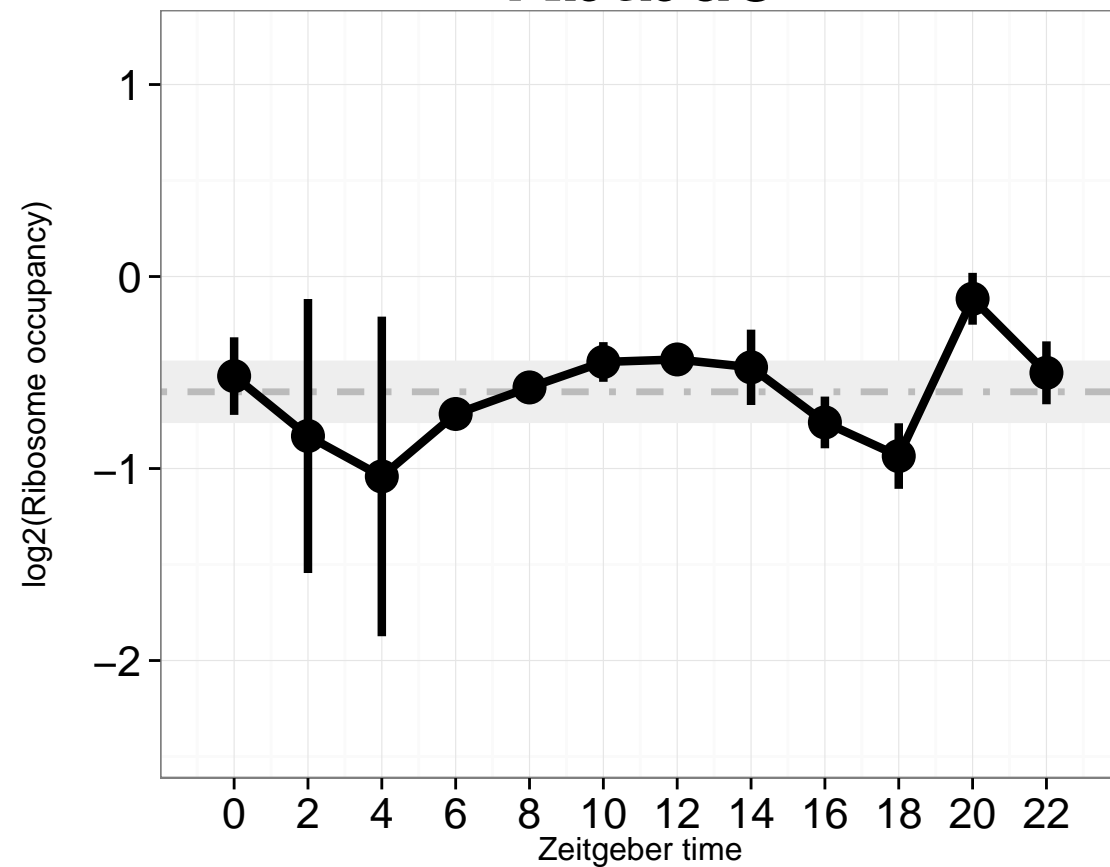

Supplement: Supplementary file 7 — Expression plots for kidney and liver for the 178 common rhythmic genes of Fig. 3c. (ZIP 3338.28 kb) [file 13059_2017_1222_MOESM7_ESM.zip › set_D_shared(178)/Kbtbd8_liver_set_D.pdf]

## Klf13

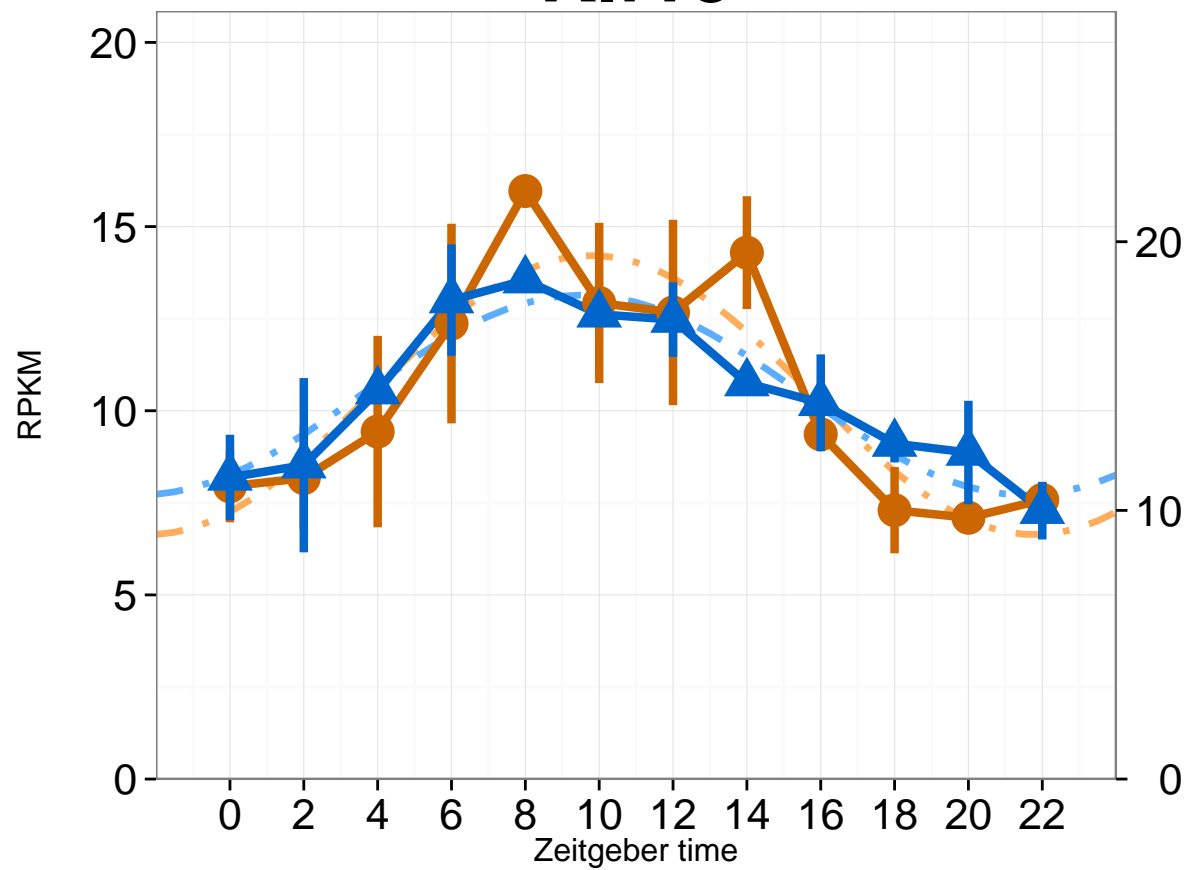

## Klf13

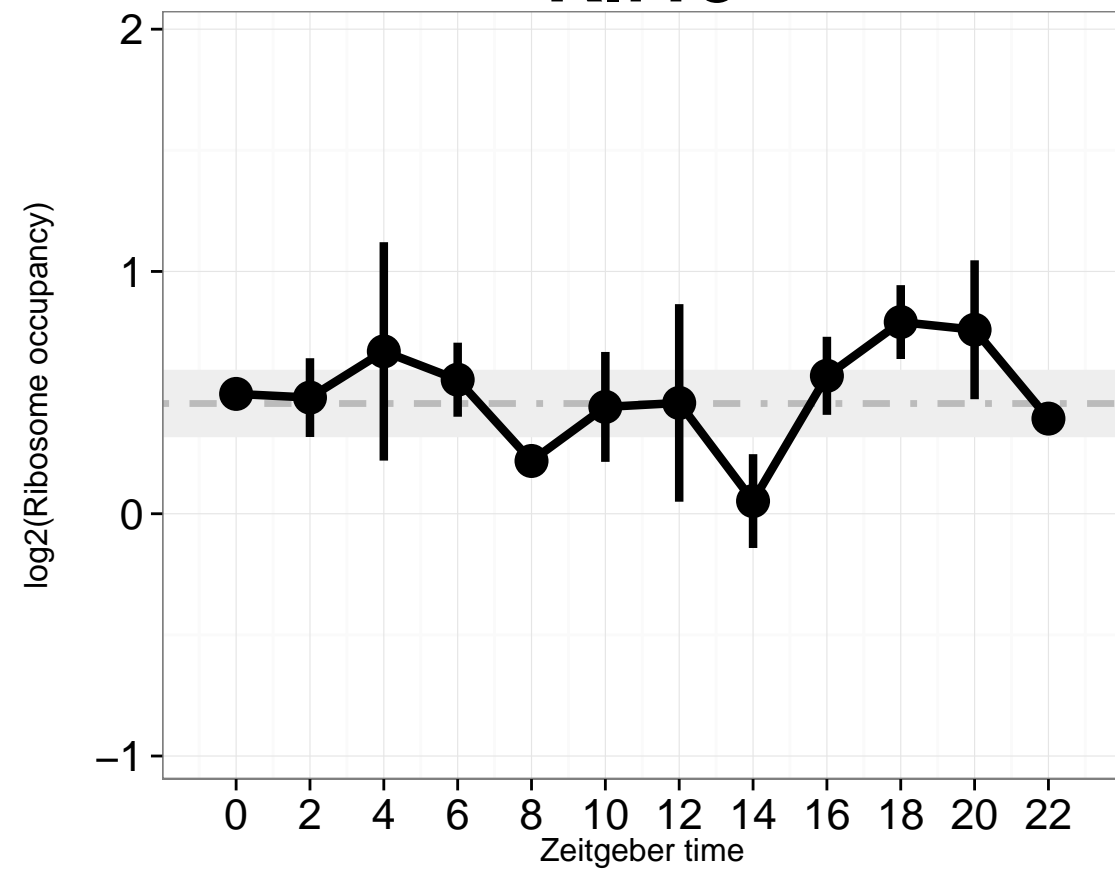

Supplement: Supplementary file 7 — Expression plots for kidney and liver for the 178 common rhythmic genes of Fig. 3c. (ZIP 3338.28 kb) [file 13059_2017_1222_MOESM7_ESM.zip › set_D_shared(178)/Klf13_kidney_set_D.pdf]

## Klf13

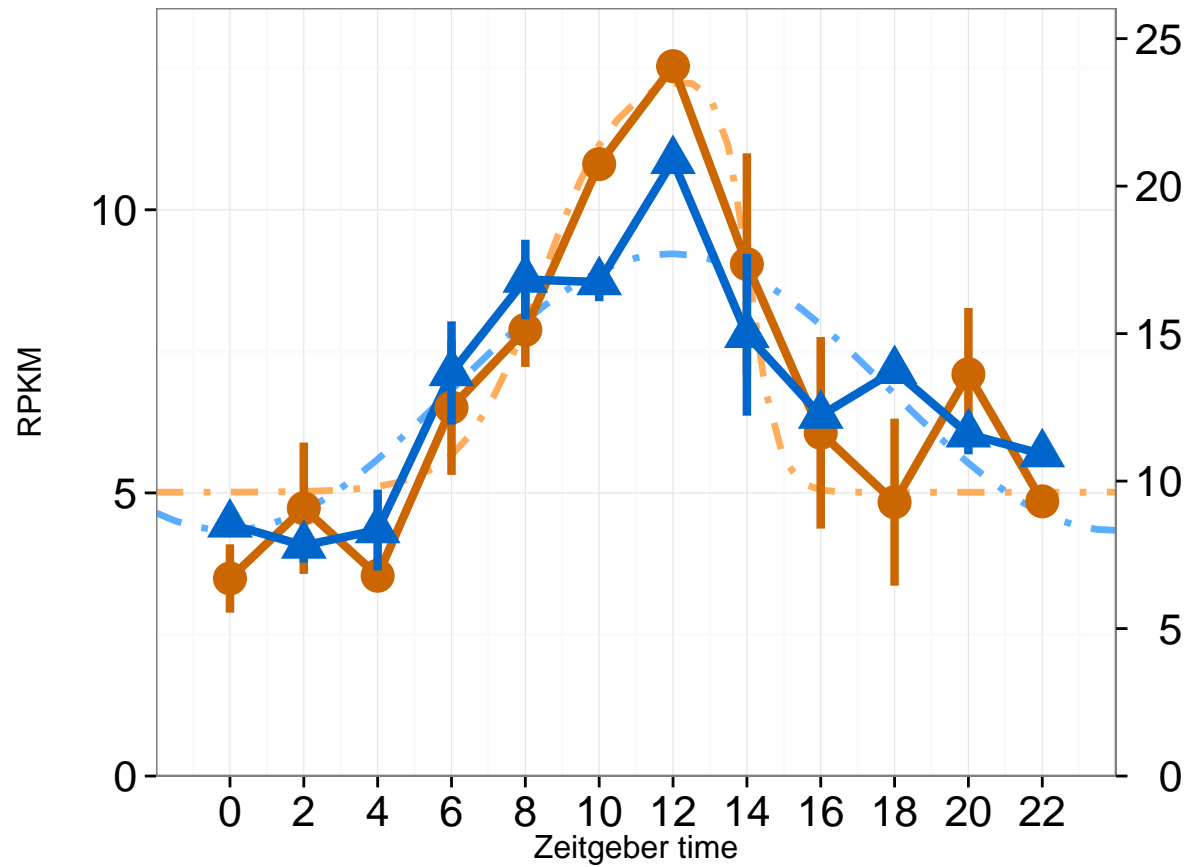

## Klf13

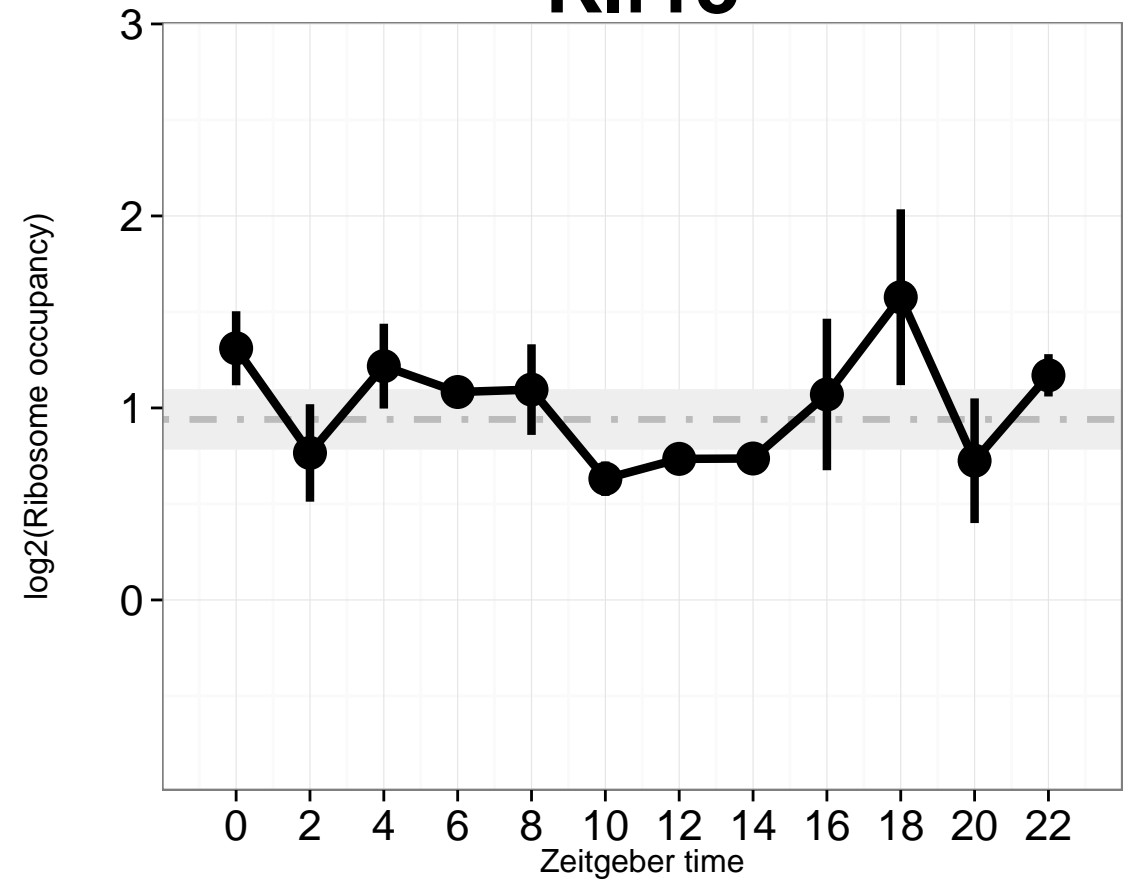

Supplement: Supplementary file 7 — Expression plots for kidney and liver for the 178 common rhythmic genes of Fig. 3c. (ZIP 3338.28 kb) [file 13059_2017_1222_MOESM7_ESM.zip › set_D_shared(178)/Klf13_liver_set_D.pdf]

# Lcn2

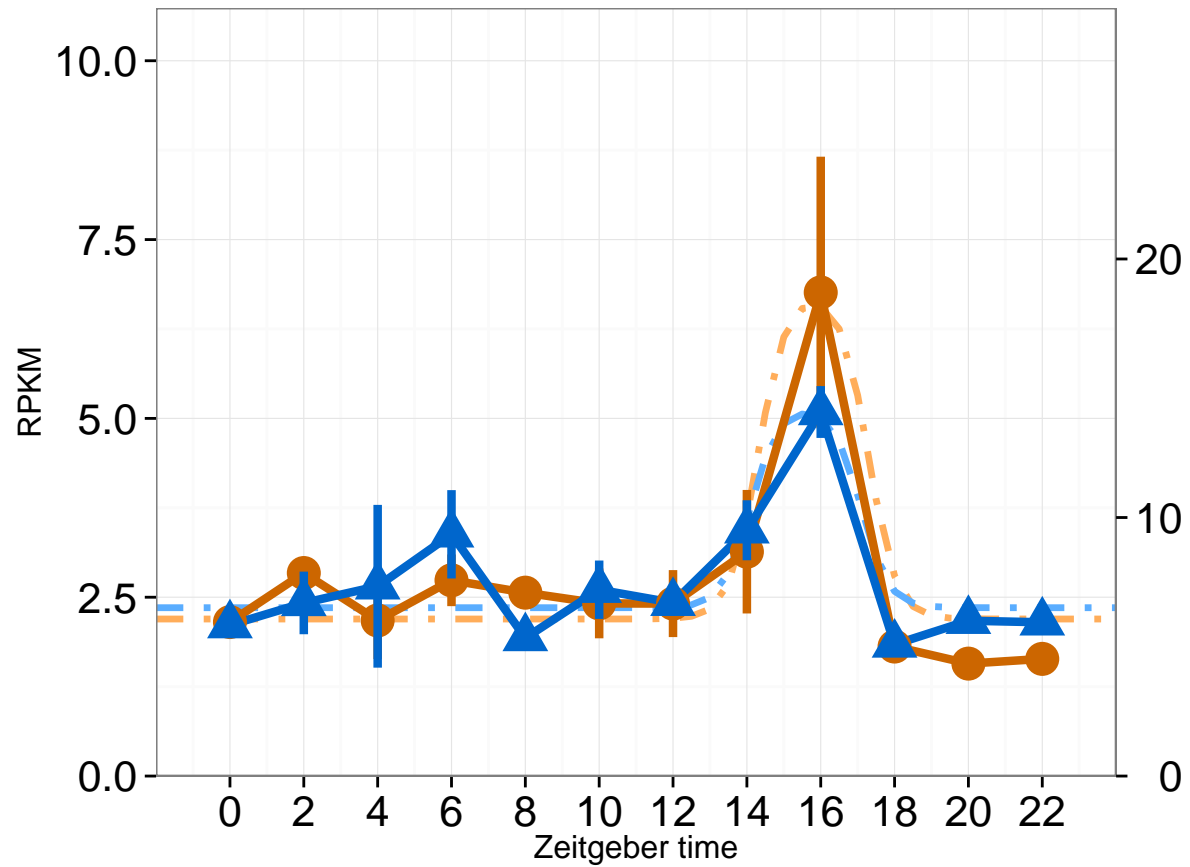

# Lcn2

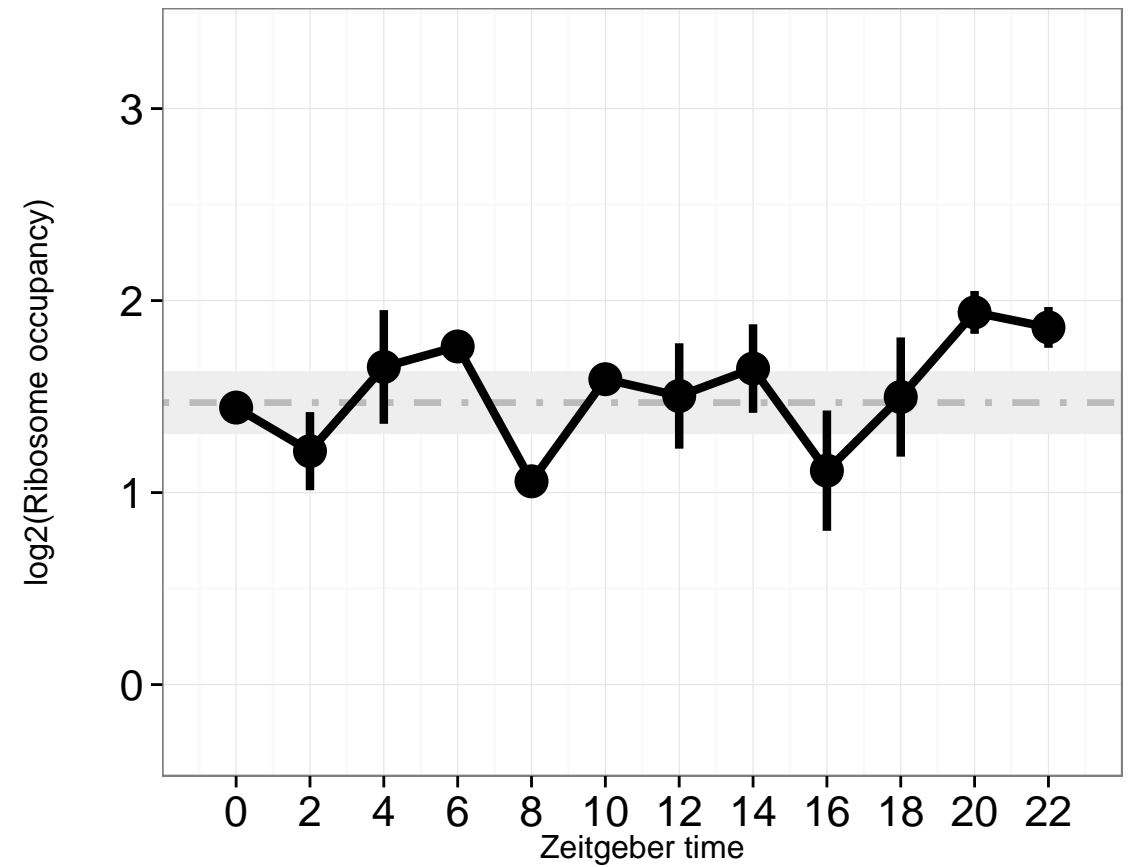

Supplement: Supplementary file 7 — Expression plots for kidney and liver for the 178 common rhythmic genes of Fig. 3c. (ZIP 3338.28 kb) [file 13059_2017_1222_MOESM7_ESM.zip › set_D_shared(178)/Lcn2_kidney_set_D.pdf]

## Lcn2

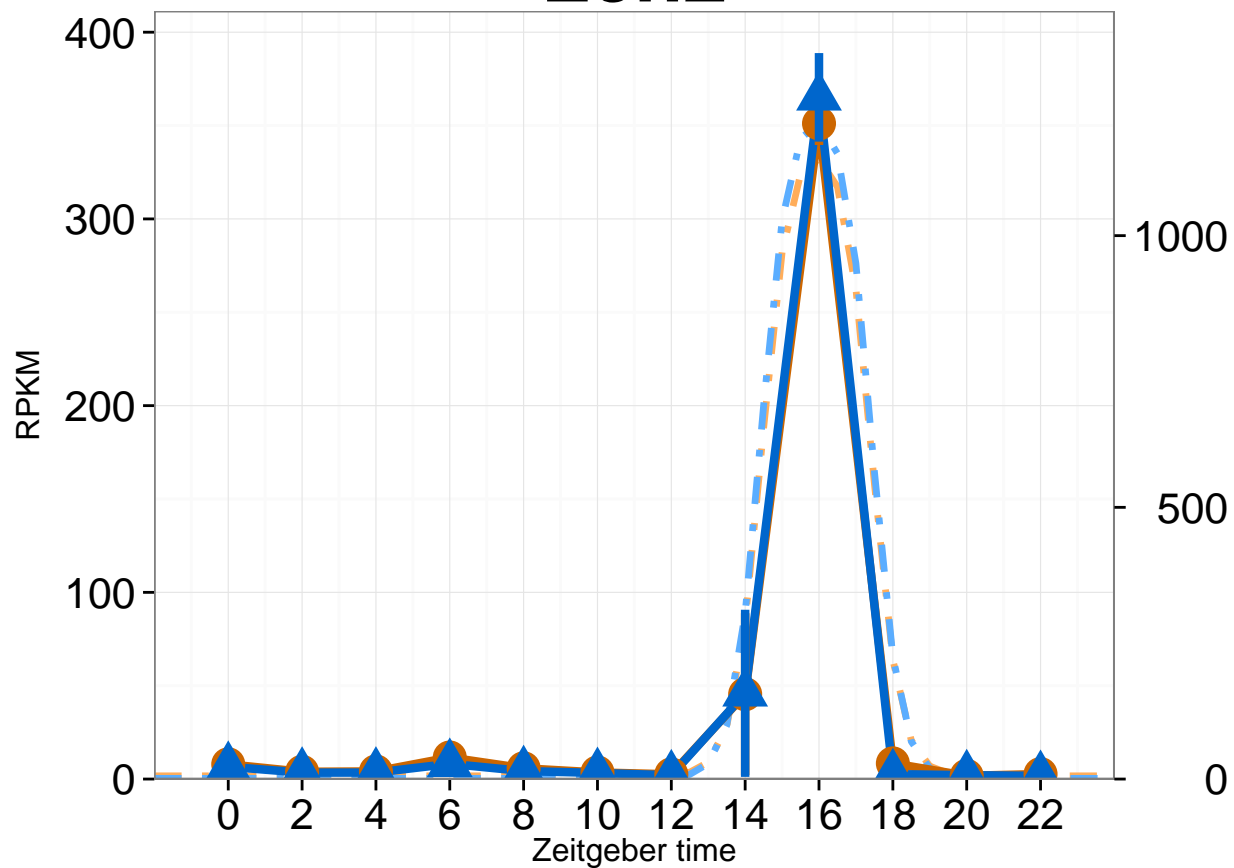

## Lcn2

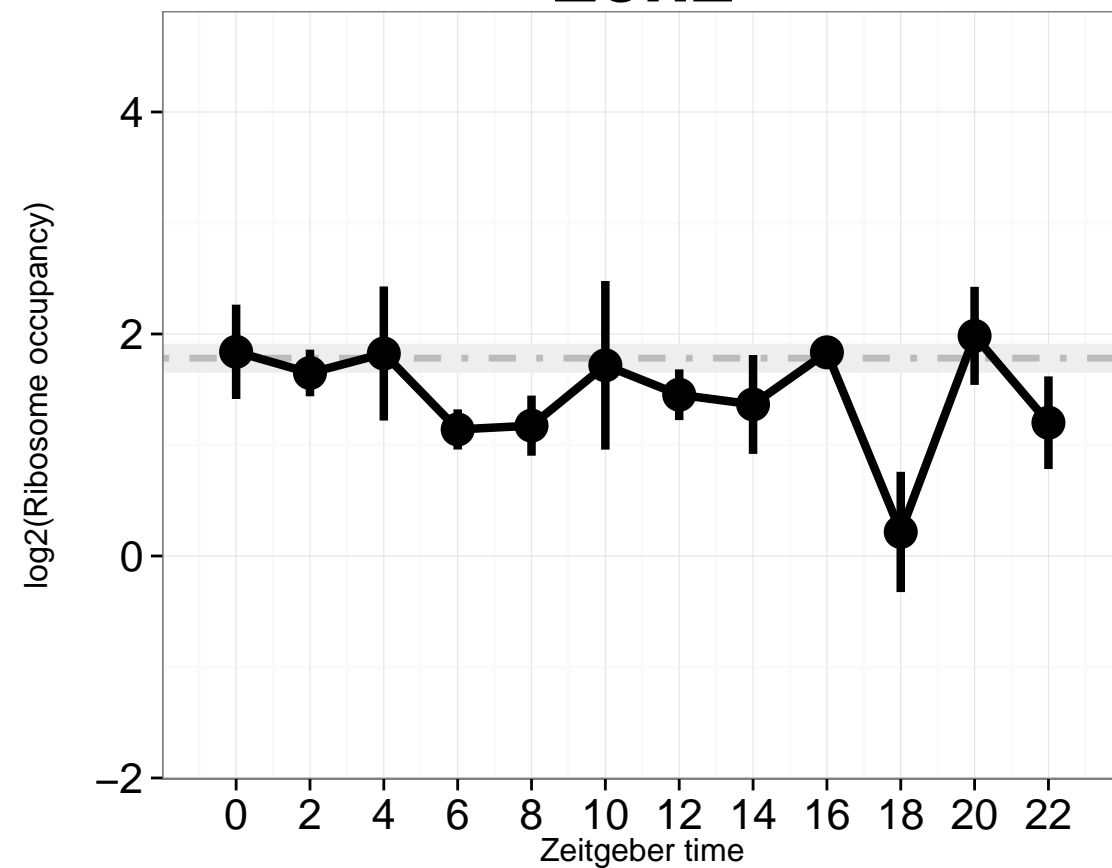

Supplement: Supplementary file 7 — Expression plots for kidney and liver for the 178 common rhythmic genes of Fig. 3c. (ZIP 3338.28 kb) [file 13059_2017_1222_MOESM7_ESM.zip › set_D_shared(178)/Lcn2_liver_set_D.pdf]

## Leo1

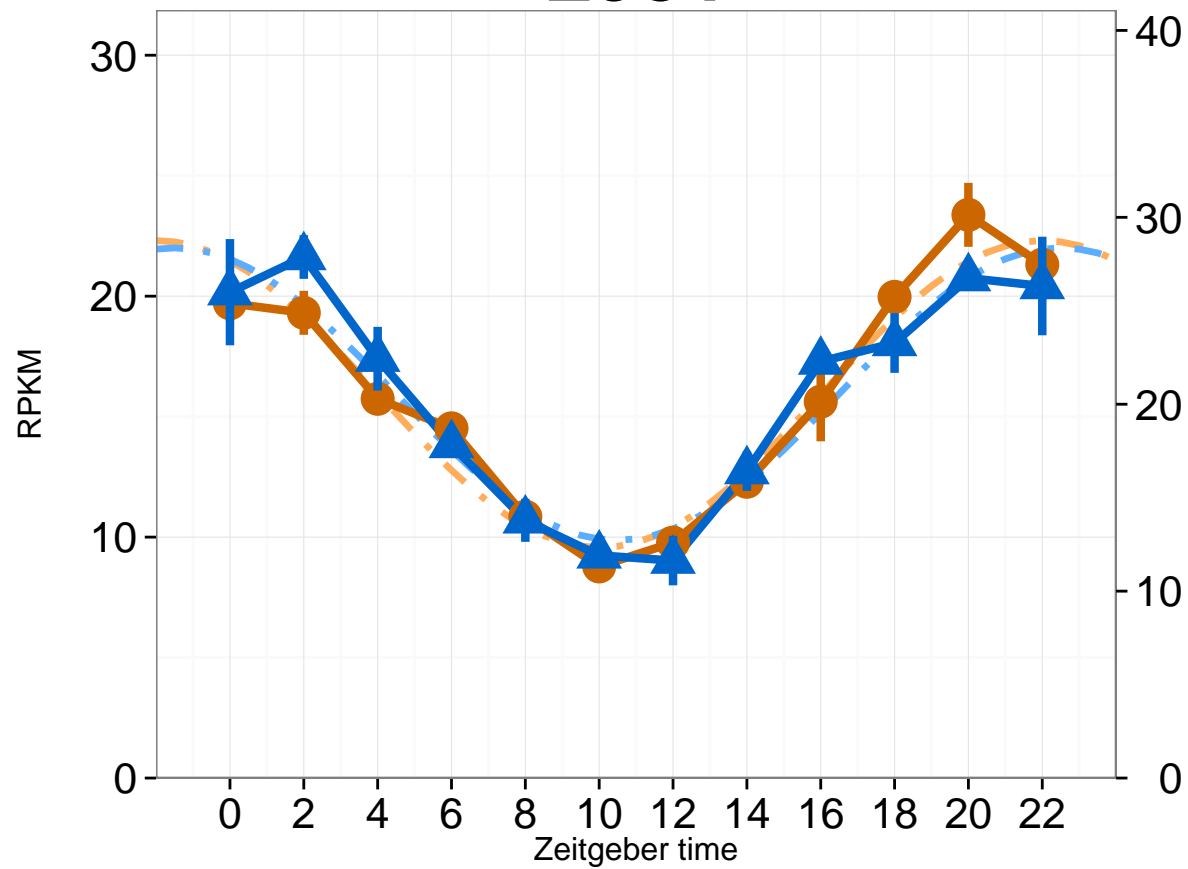

## Leo1

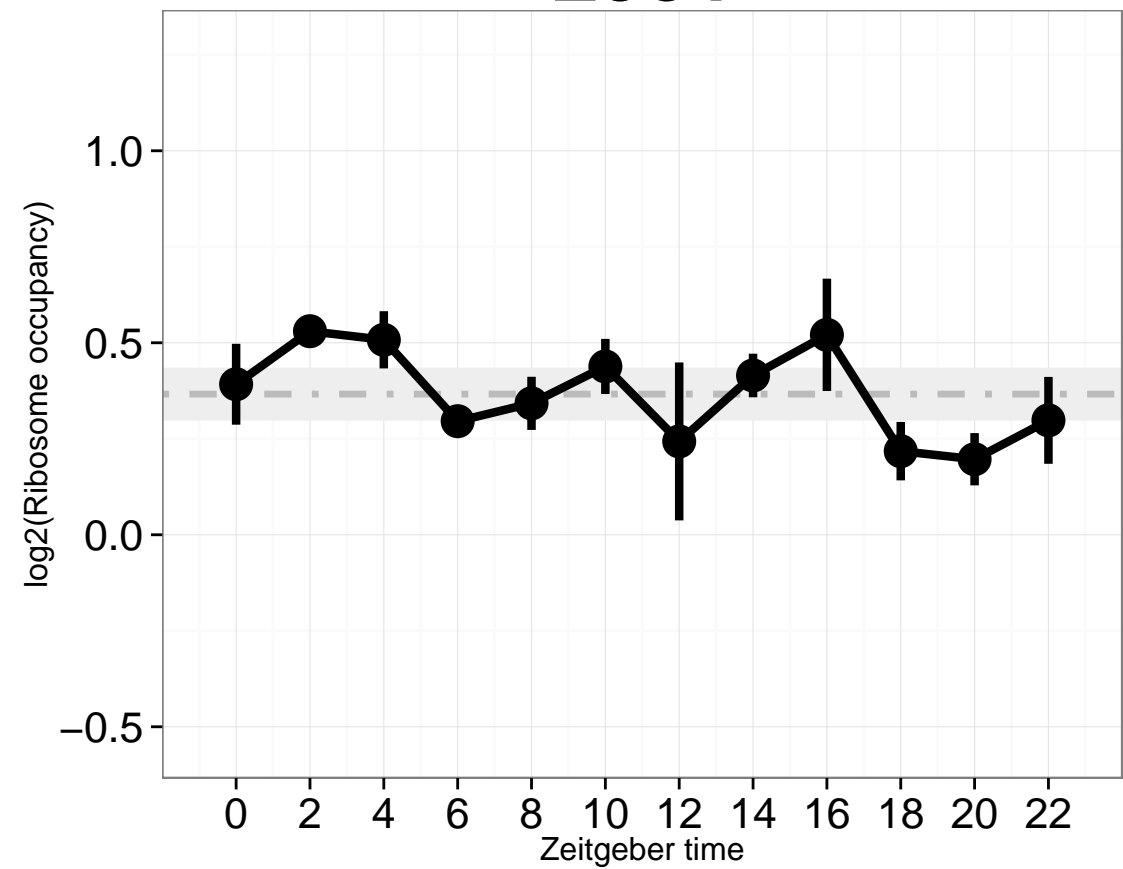

Supplement: Supplementary file 7 — Expression plots for kidney and liver for the 178 common rhythmic genes of Fig. 3c. (ZIP 3338.28 kb) [file 13059_2017_1222_MOESM7_ESM.zip › set_D_shared(178)/Leo1_kidney_set_D.pdf]

## Leo1

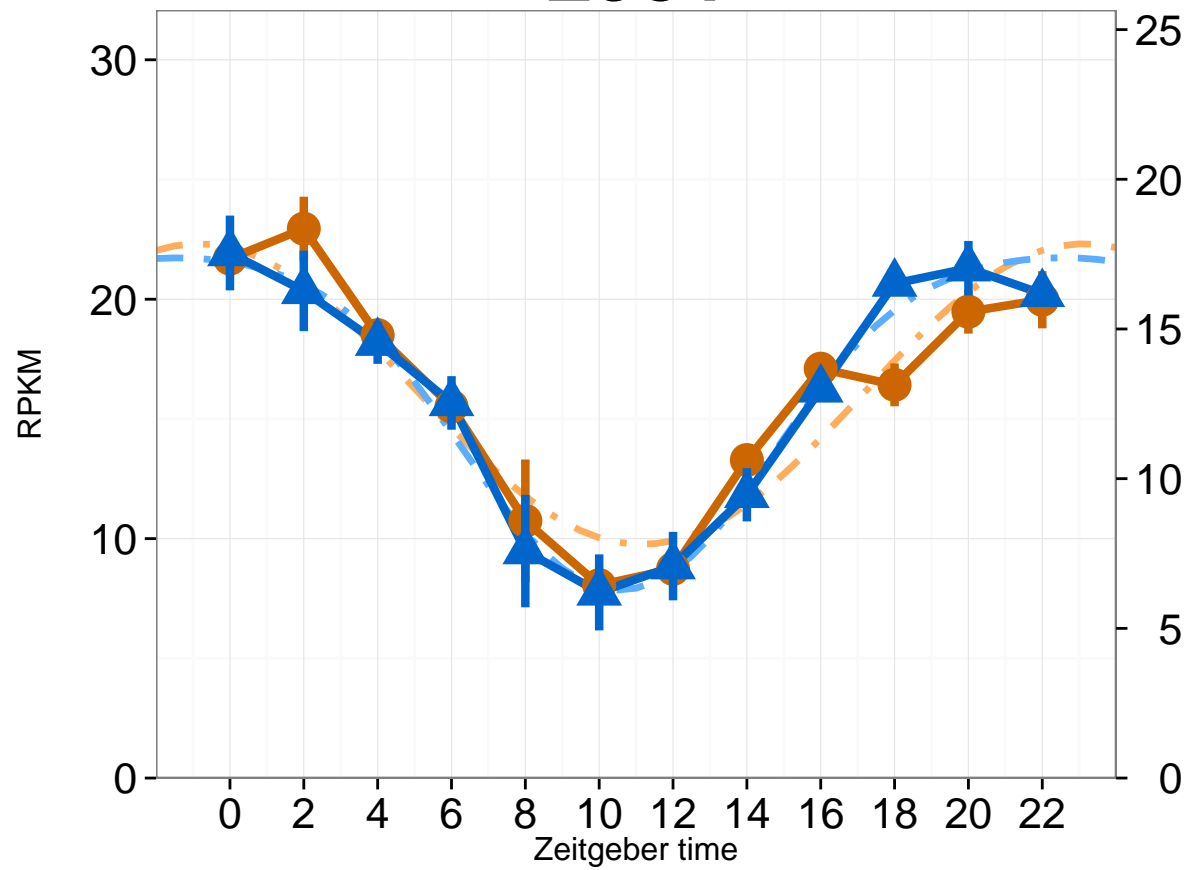

## Leo1

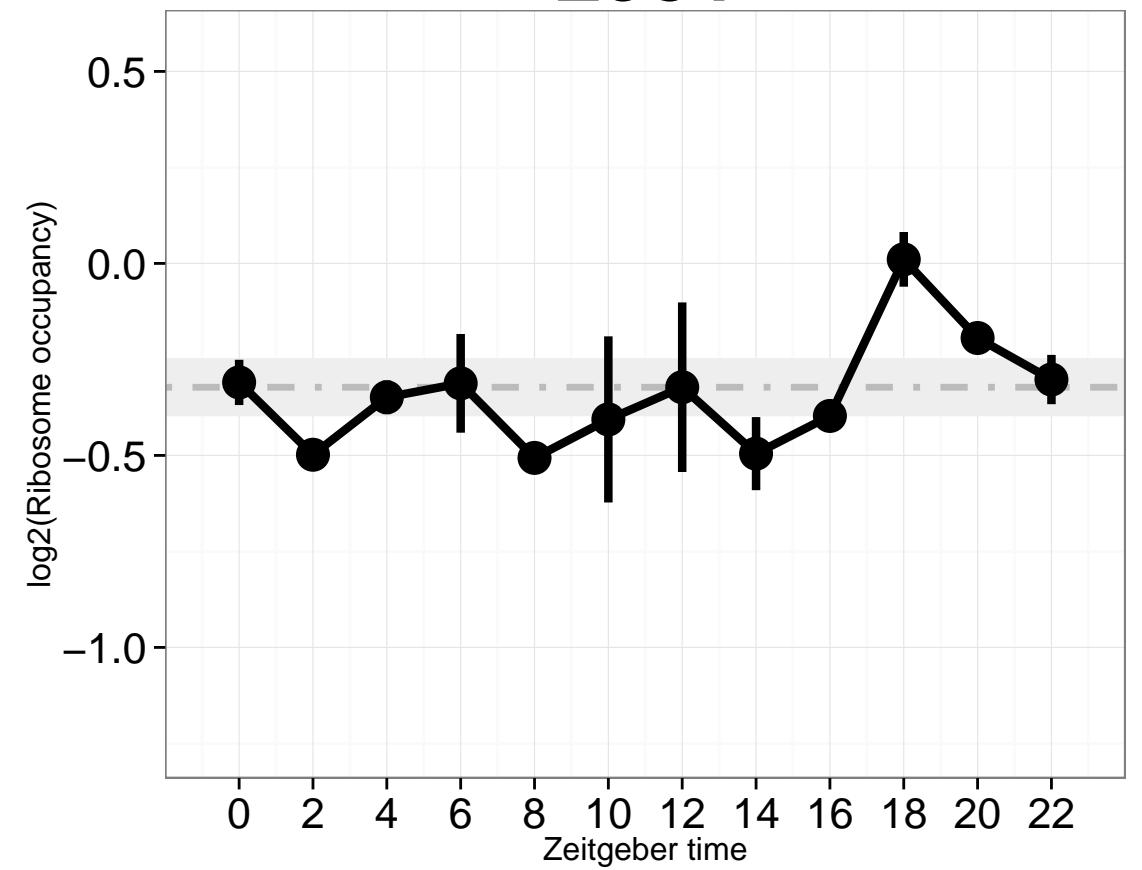

Supplement: Supplementary file 7 — Expression plots for kidney and liver for the 178 common rhythmic genes of Fig. 3c. (ZIP 3338.28 kb) [file 13059_2017_1222_MOESM7_ESM.zip › set_D_shared(178)/Leo1_liver_set_D.pdf]

## Lipg

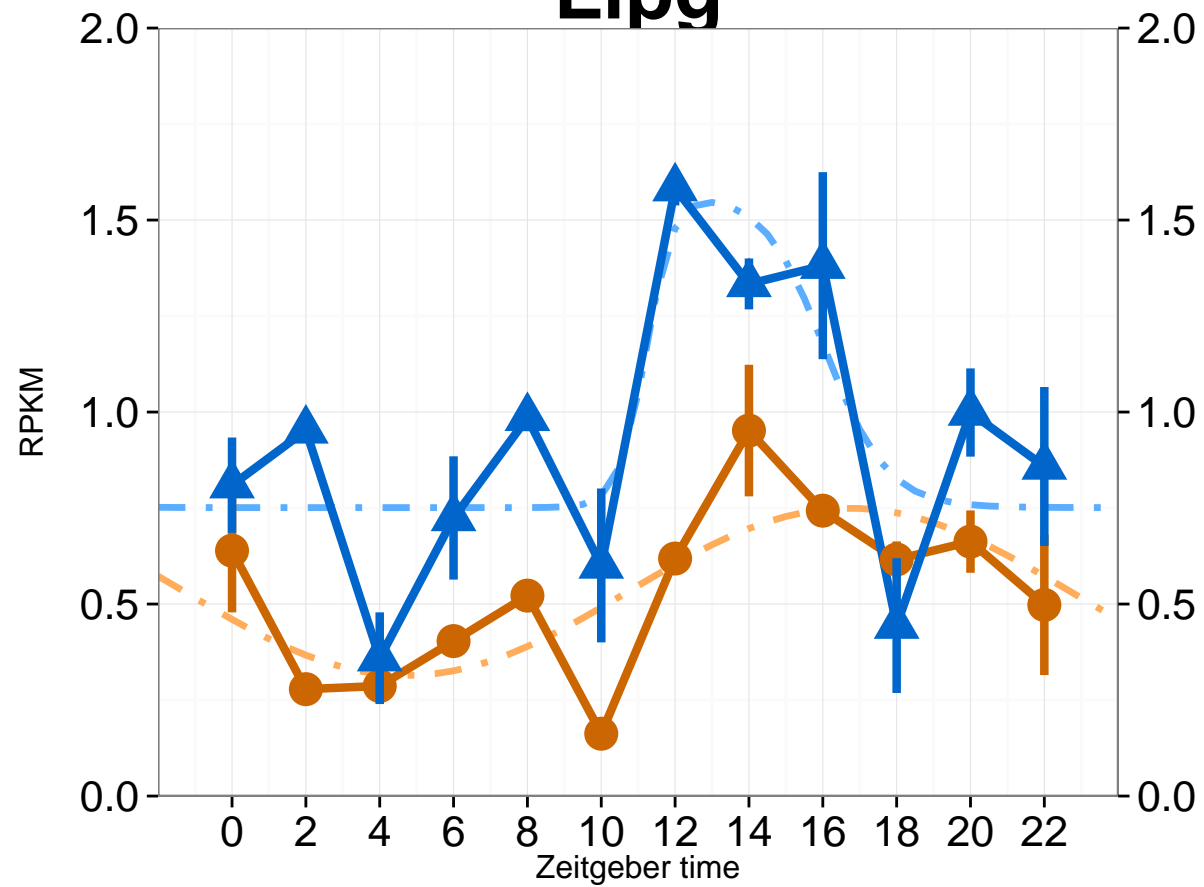

## Lipg

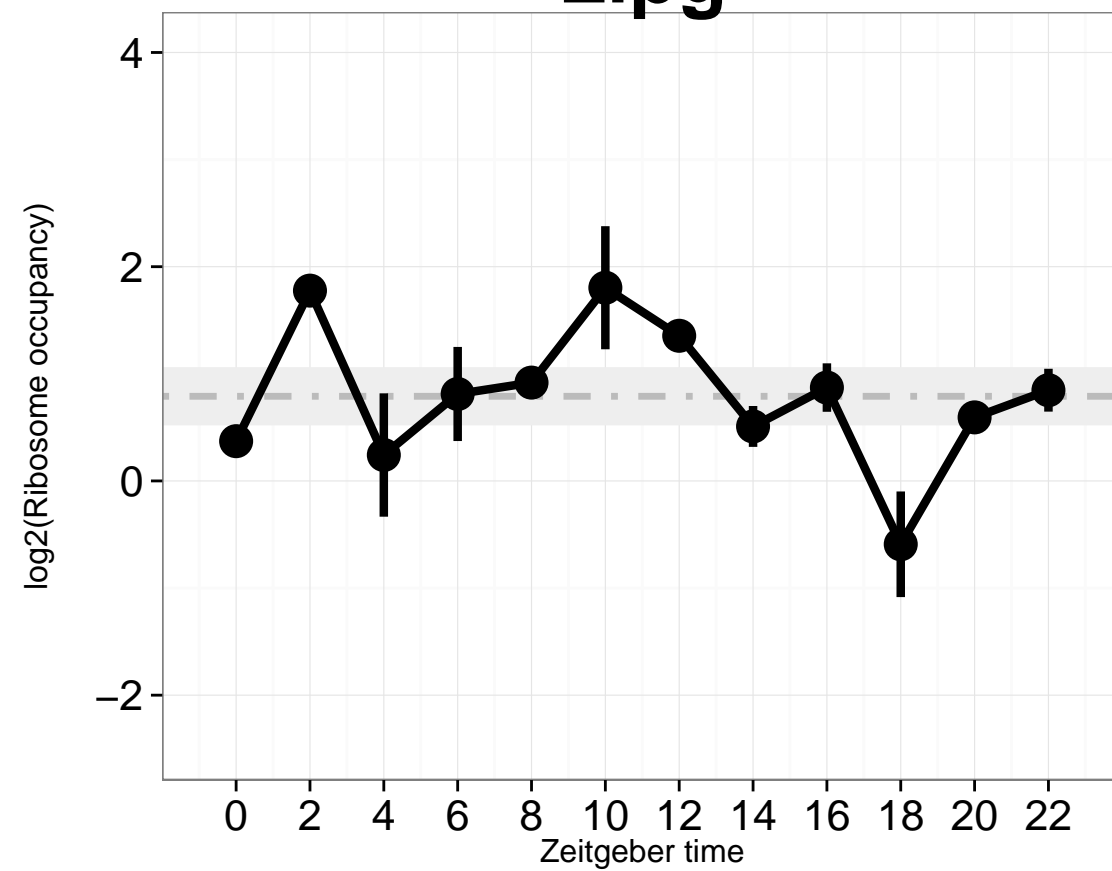

Supplement: Supplementary file 7 — Expression plots for kidney and liver for the 178 common rhythmic genes of Fig. 3c. (ZIP 3338.28 kb) [file 13059_2017_1222_MOESM7_ESM.zip › set_D_shared(178)/Lipg_kidney_set_D.pdf]

# Lipg

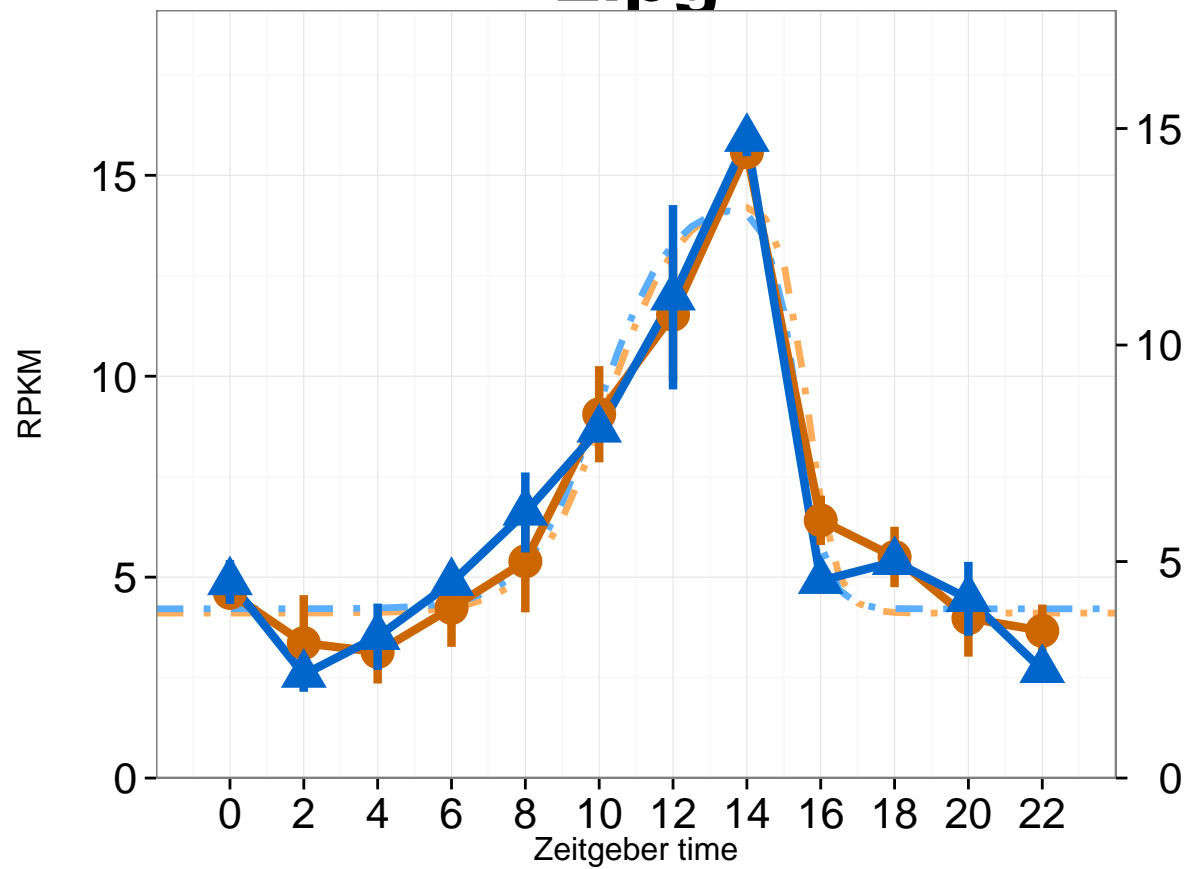

# Lipg

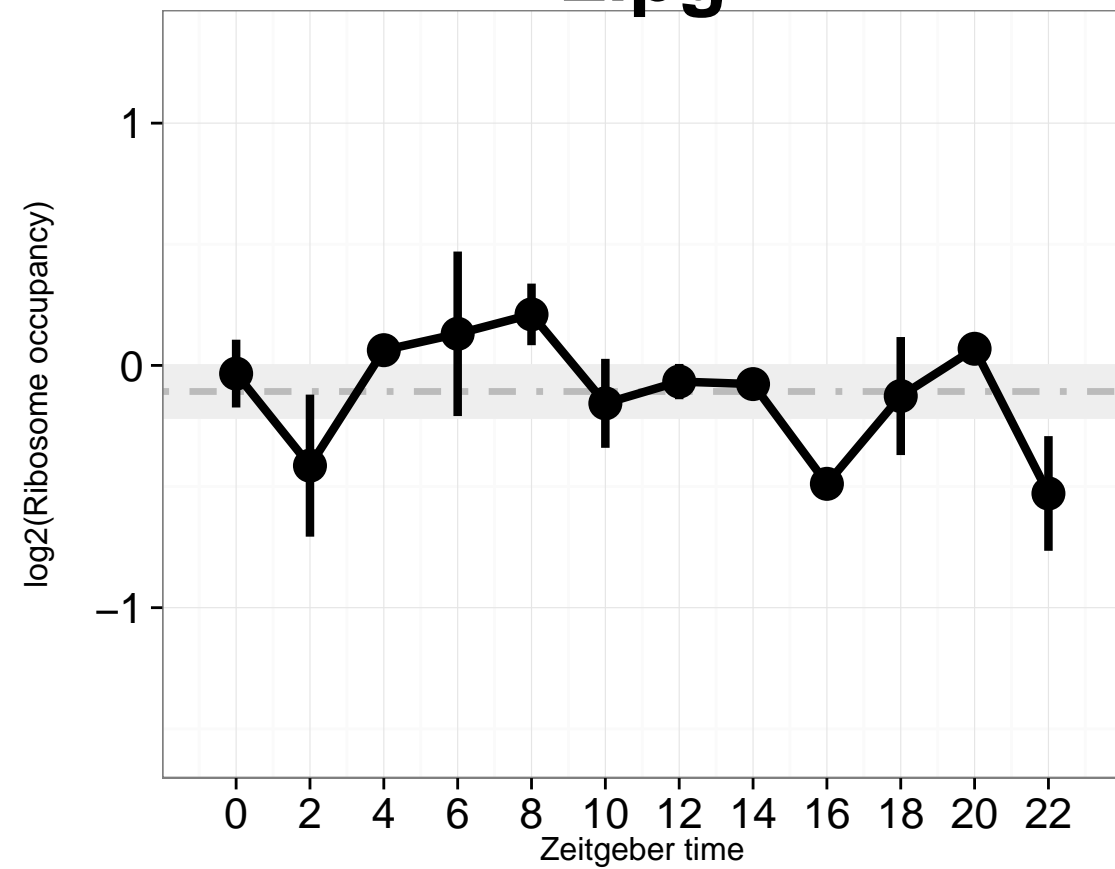

Supplement: Supplementary file 7 — Expression plots for kidney and liver for the 178 common rhythmic genes of Fig. 3c. (ZIP 3338.28 kb) [file 13059_2017_1222_MOESM7_ESM.zip › set_D_shared(178)/Lipg_liver_set_D.pdf]

# Lonrf1

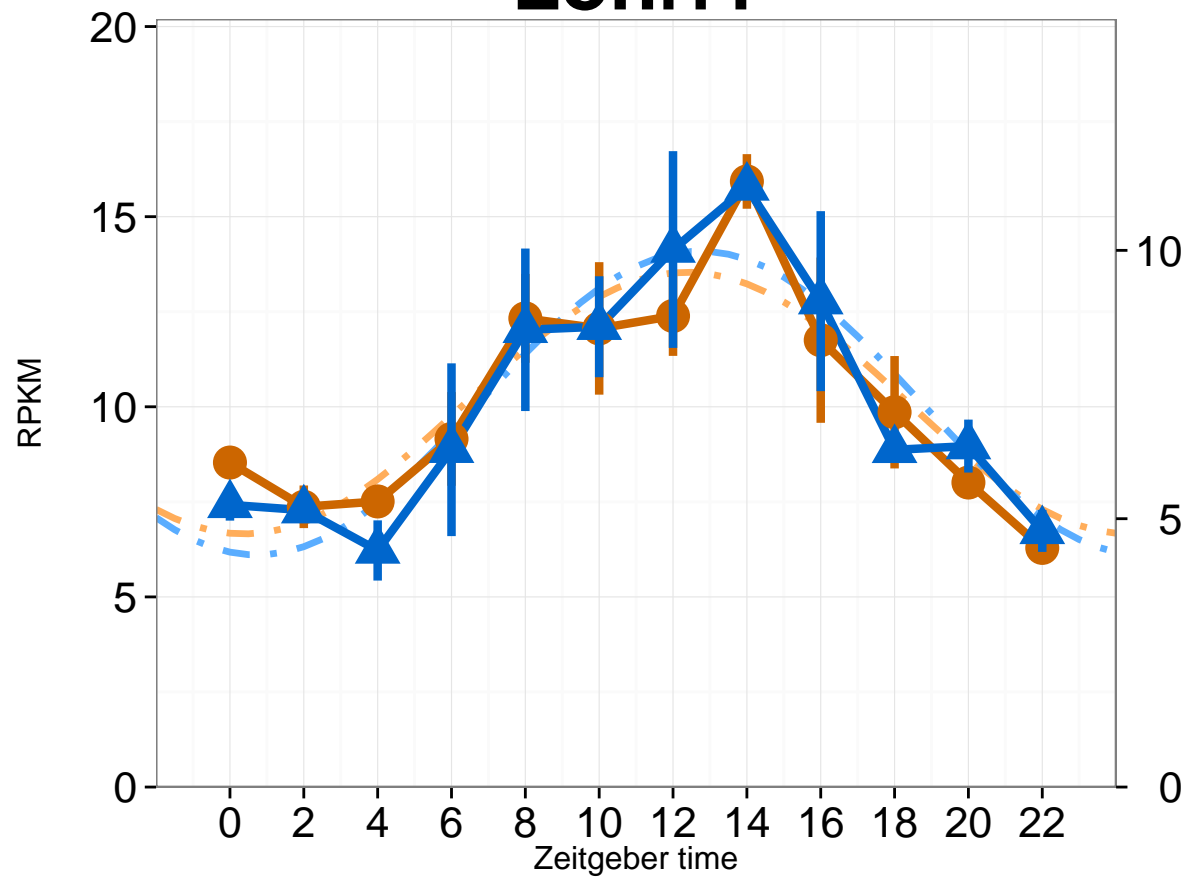

# Lonrf1

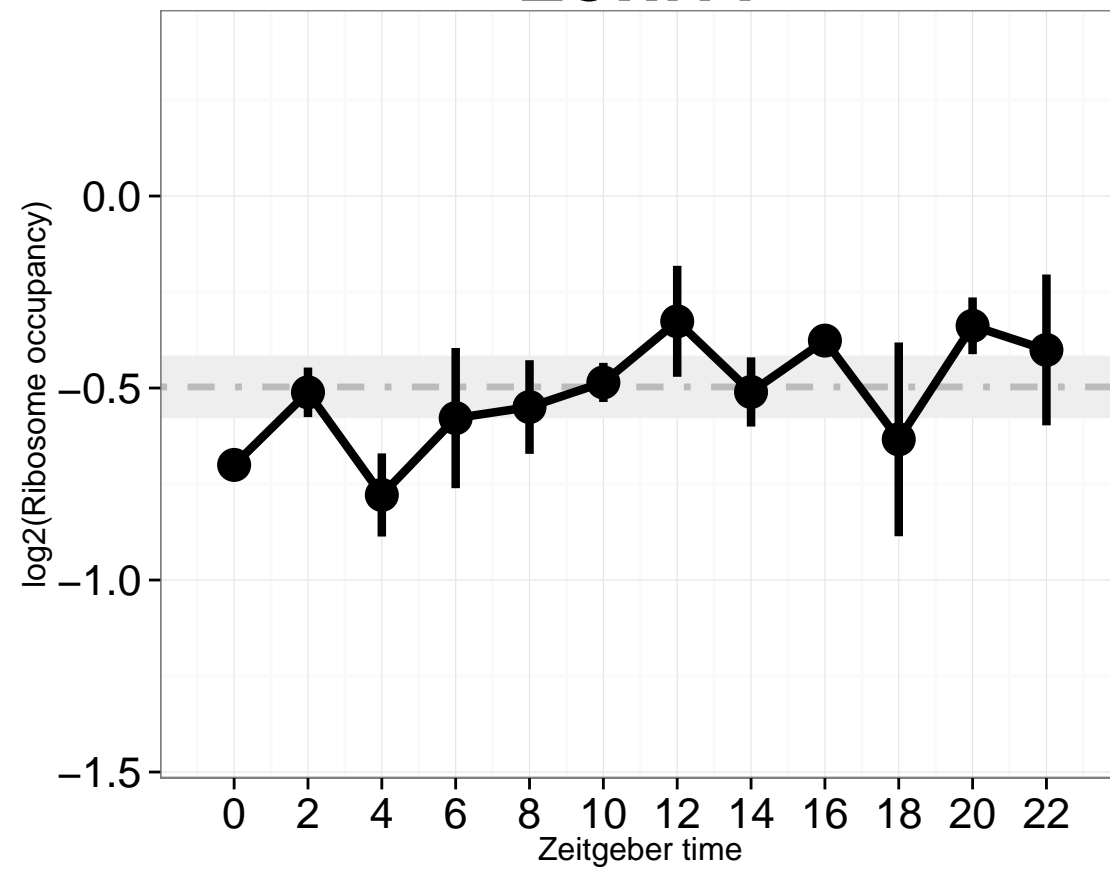

Supplement: Supplementary file 7 — Expression plots for kidney and liver for the 178 common rhythmic genes of Fig. 3c. (ZIP 3338.28 kb) [file 13059_2017_1222_MOESM7_ESM.zip › set_D_shared(178)/Lonrf1_kidney_set_D.pdf]

# Lonrf1

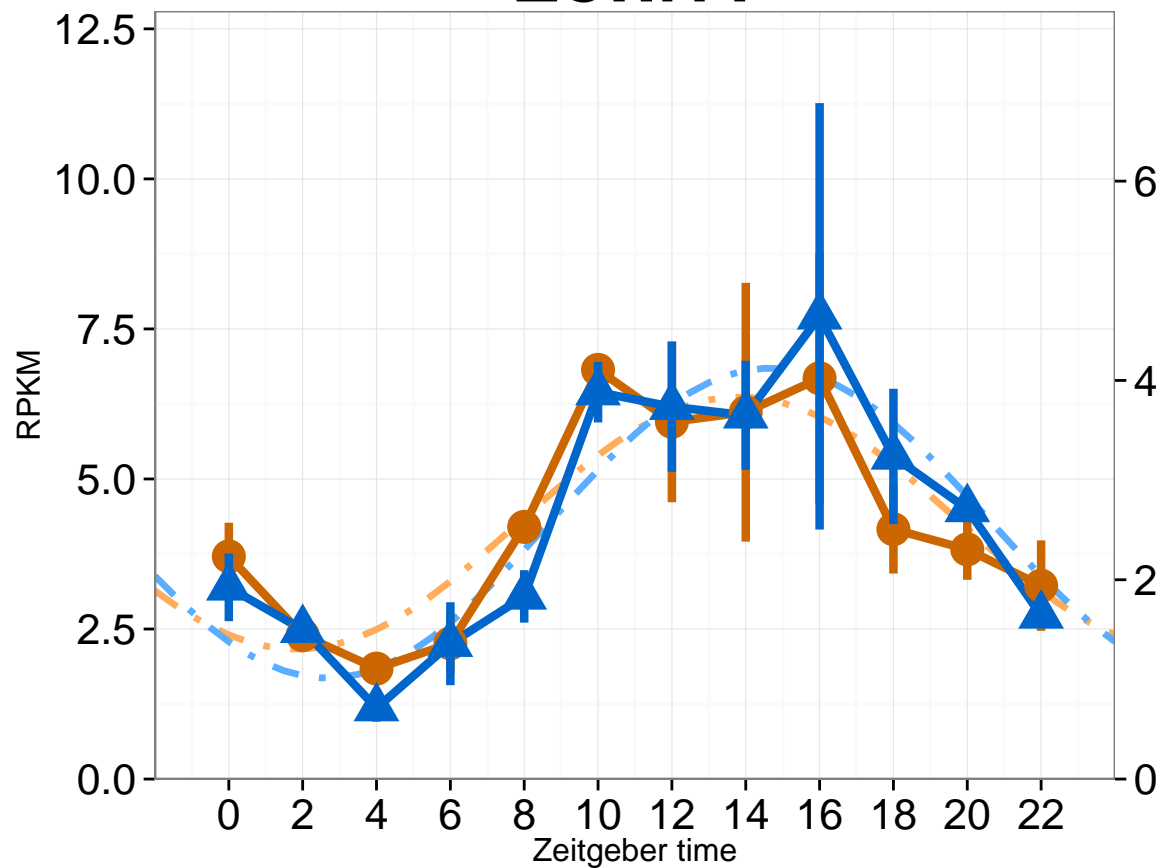

# Lonrf1

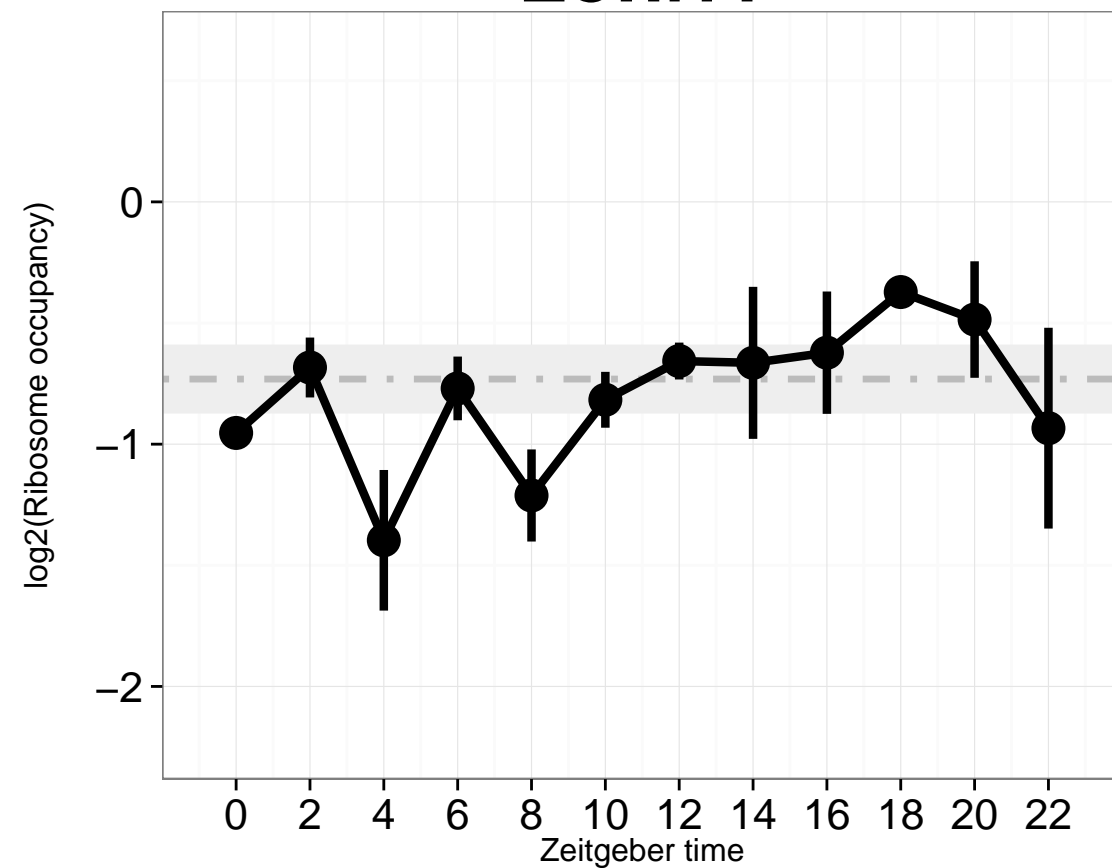

Supplement: Supplementary file 7 — Expression plots for kidney and liver for the 178 common rhythmic genes of Fig. 3c. (ZIP 3338.28 kb) [file 13059_2017_1222_MOESM7_ESM.zip › set_D_shared(178)/Lonrf1_liver_set_D.pdf]

## Loxl4

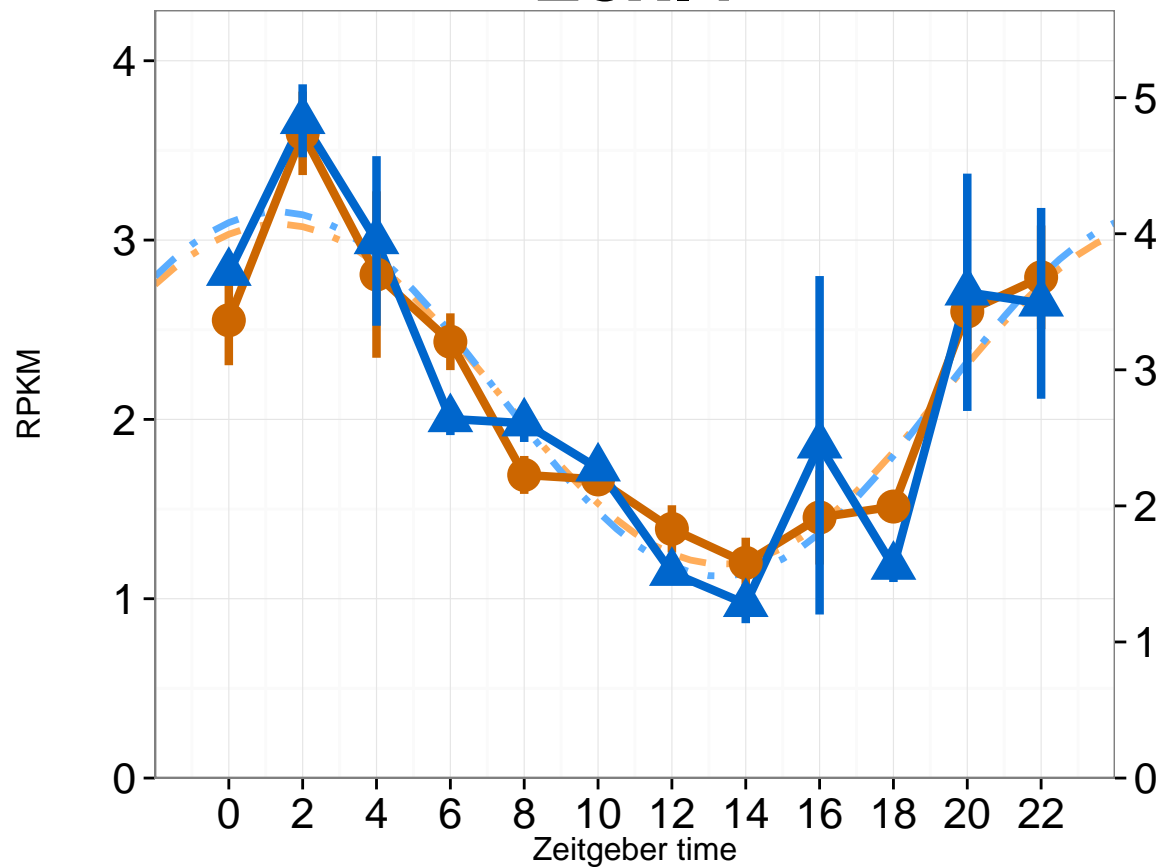

## Loxl4

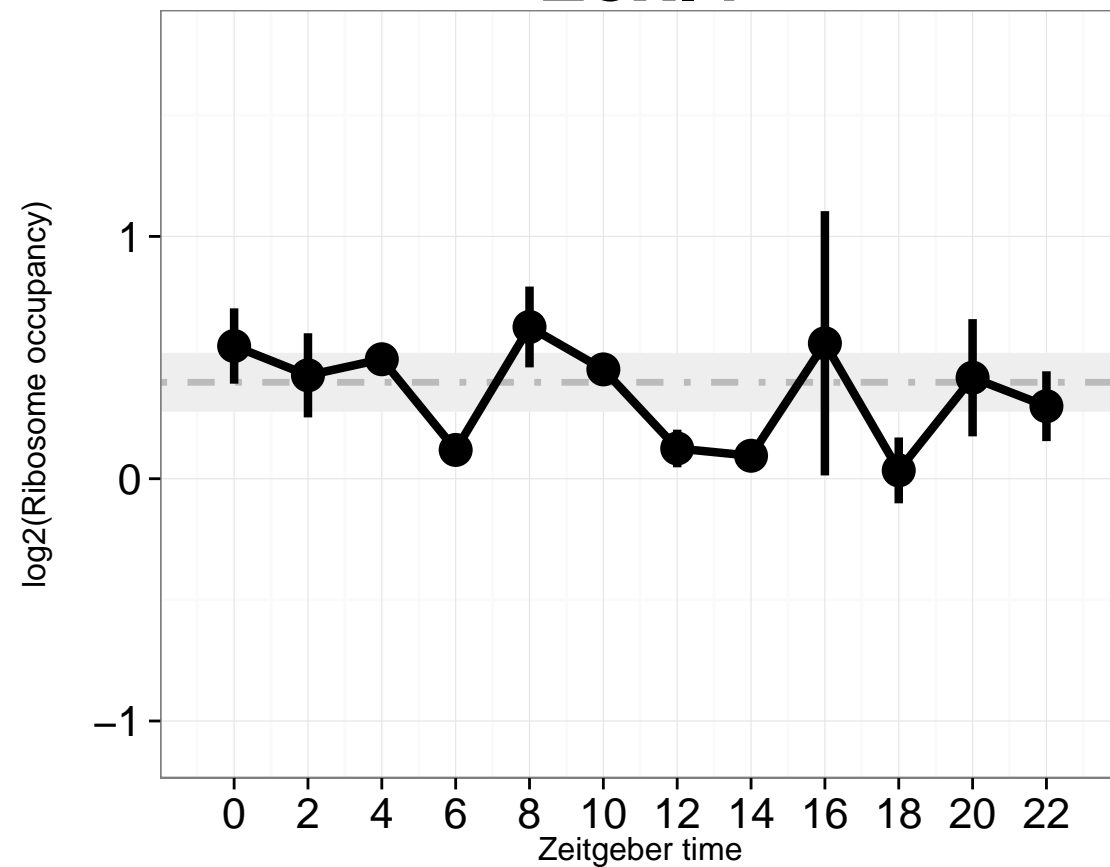

Supplement: Supplementary file 7 — Expression plots for kidney and liver for the 178 common rhythmic genes of Fig. 3c. (ZIP 3338.28 kb) [file 13059_2017_1222_MOESM7_ESM.zip › set_D_shared(178)/Loxl4_kidney_set_D.pdf]

## Loxl4

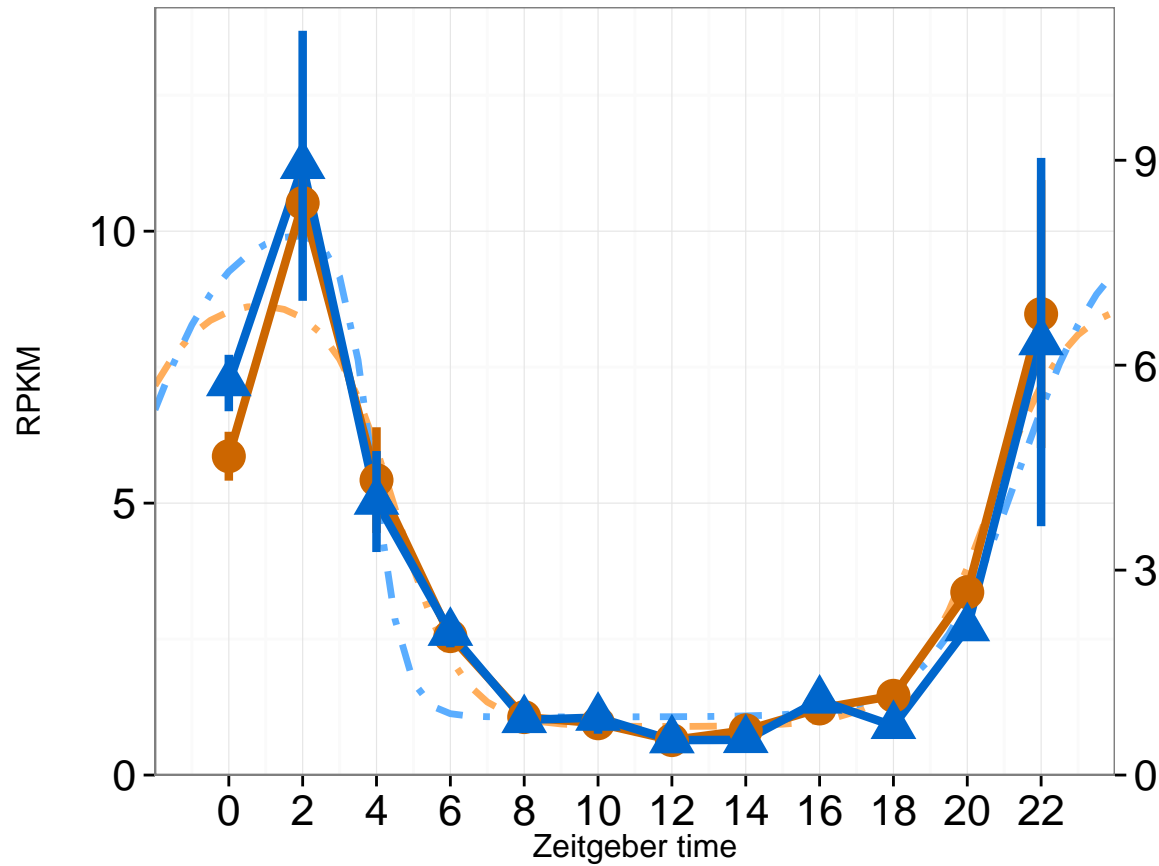

## Loxl4

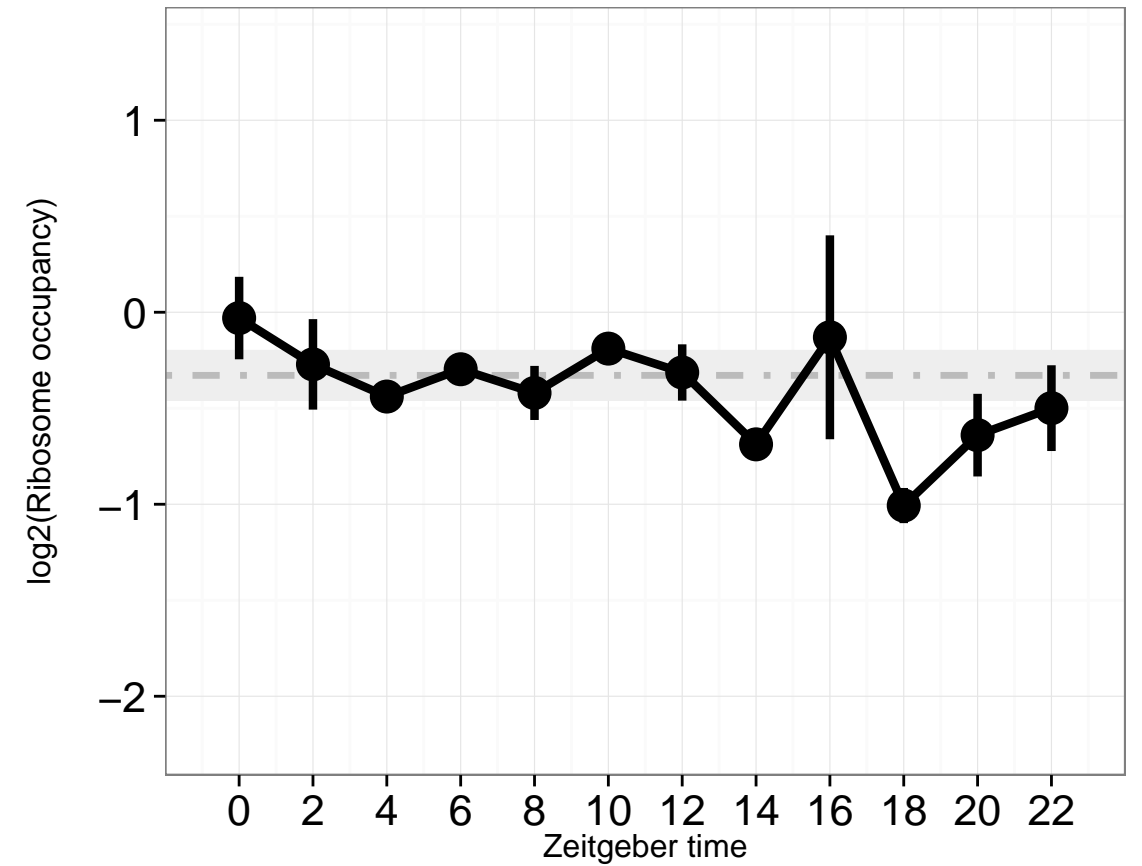

Supplement: Supplementary file 7 — Expression plots for kidney and liver for the 178 common rhythmic genes of Fig. 3c. (ZIP 3338.28 kb) [file 13059_2017_1222_MOESM7_ESM.zip › set_D_shared(178)/Loxl4_liver_set_D.pdf]

# Map2k6

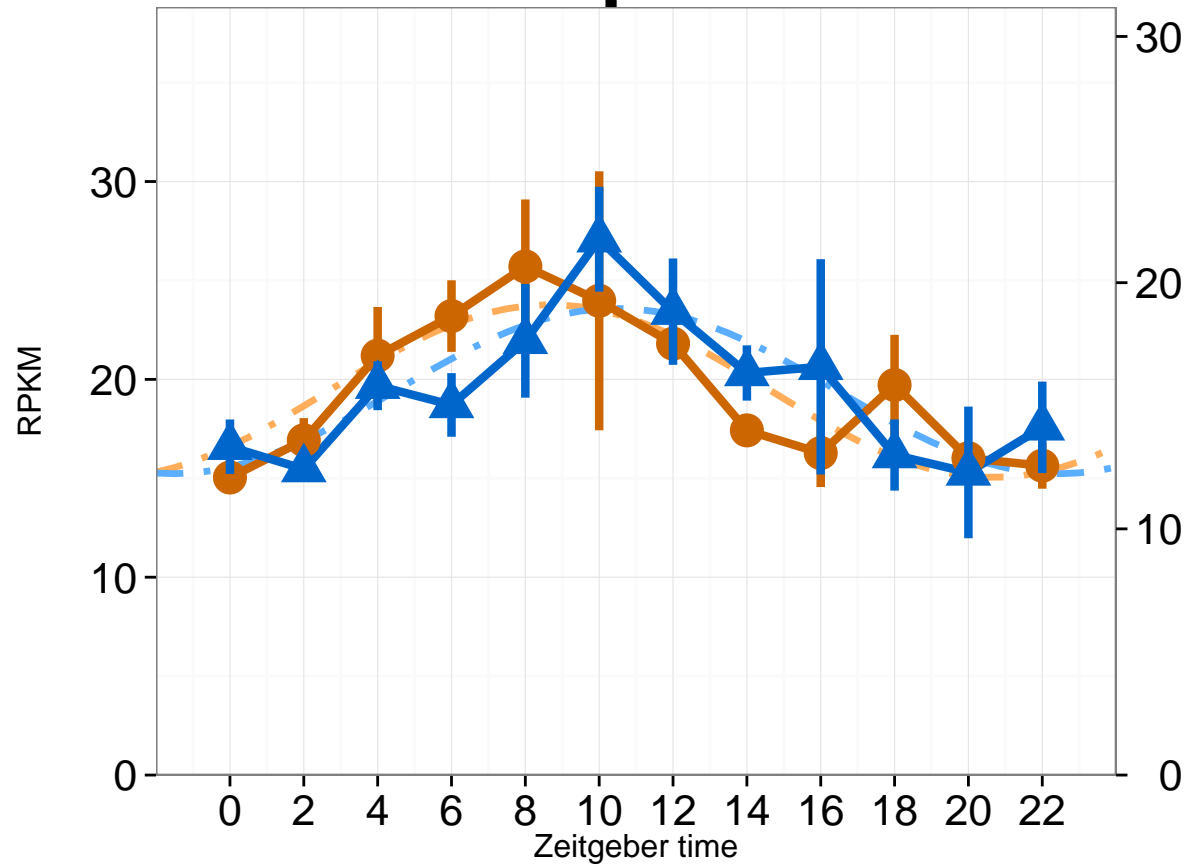

# Map2k6

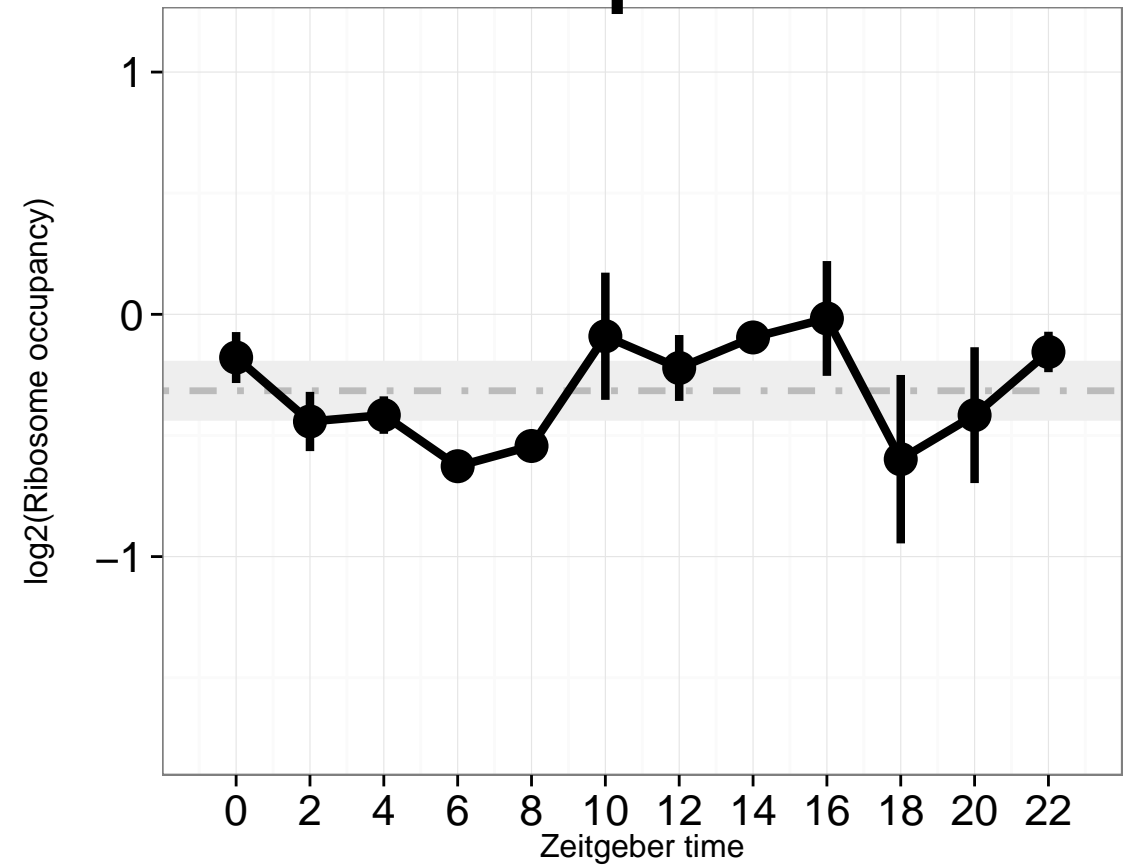

Supplement: Supplementary file 7 — Expression plots for kidney and liver for the 178 common rhythmic genes of Fig. 3c. (ZIP 3338.28 kb) [file 13059_2017_1222_MOESM7_ESM.zip › set_D_shared(178)/Map2k6_kidney_set_D.pdf]

# Map2k6

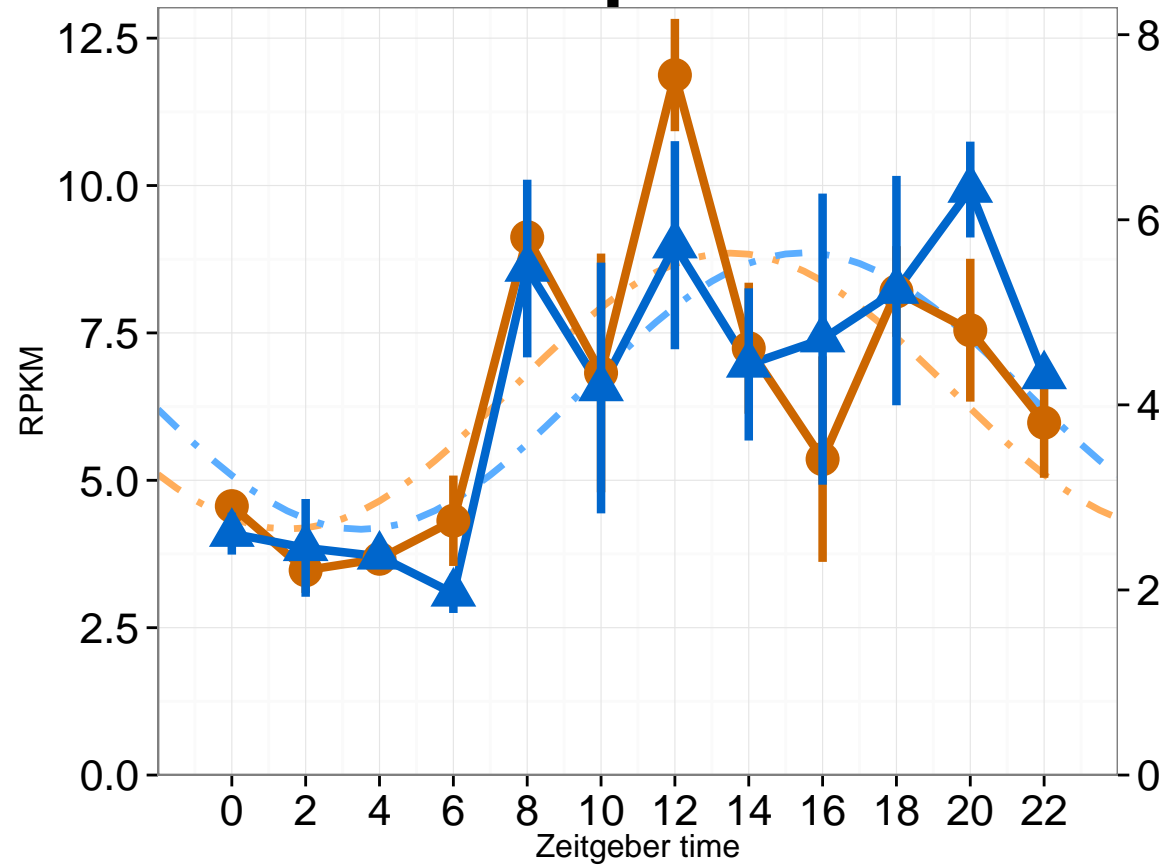

# Map2k6

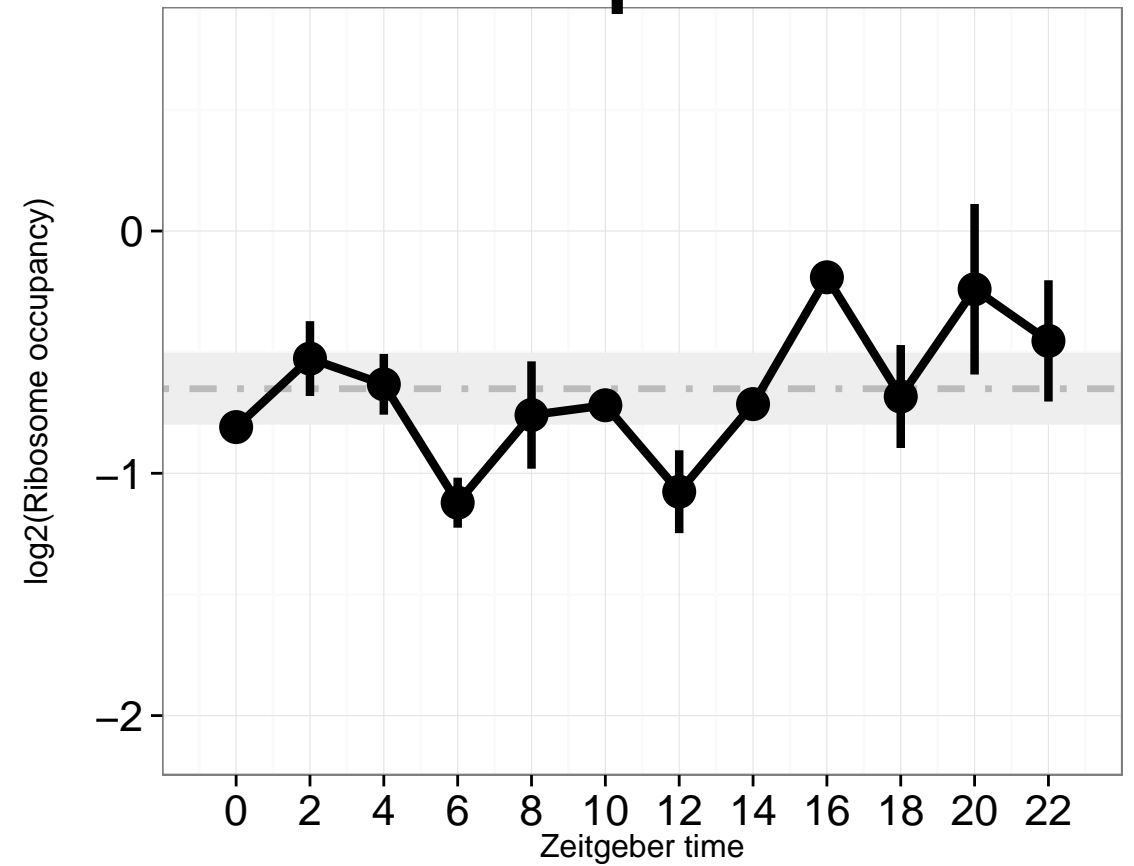

Supplement: Supplementary file 7 — Expression plots for kidney and liver for the 178 common rhythmic genes of Fig. 3c. (ZIP 3338.28 kb) [file 13059_2017_1222_MOESM7_ESM.zip › set_D_shared(178)/Map2k6_liver_set_D.pdf]
