# Supplementary figures and images for: Translational contributions to tissue specificity in rhythmic and constitutive gene expression (part 3 of 4)
Source: Genome Biol. 2017 Jun 16;18:116. doi: 10.1186/s13059-017-1222-2 (PMC5473967; doi:10.1186/s13059-017-1222-2)

# 1300002K09Rik

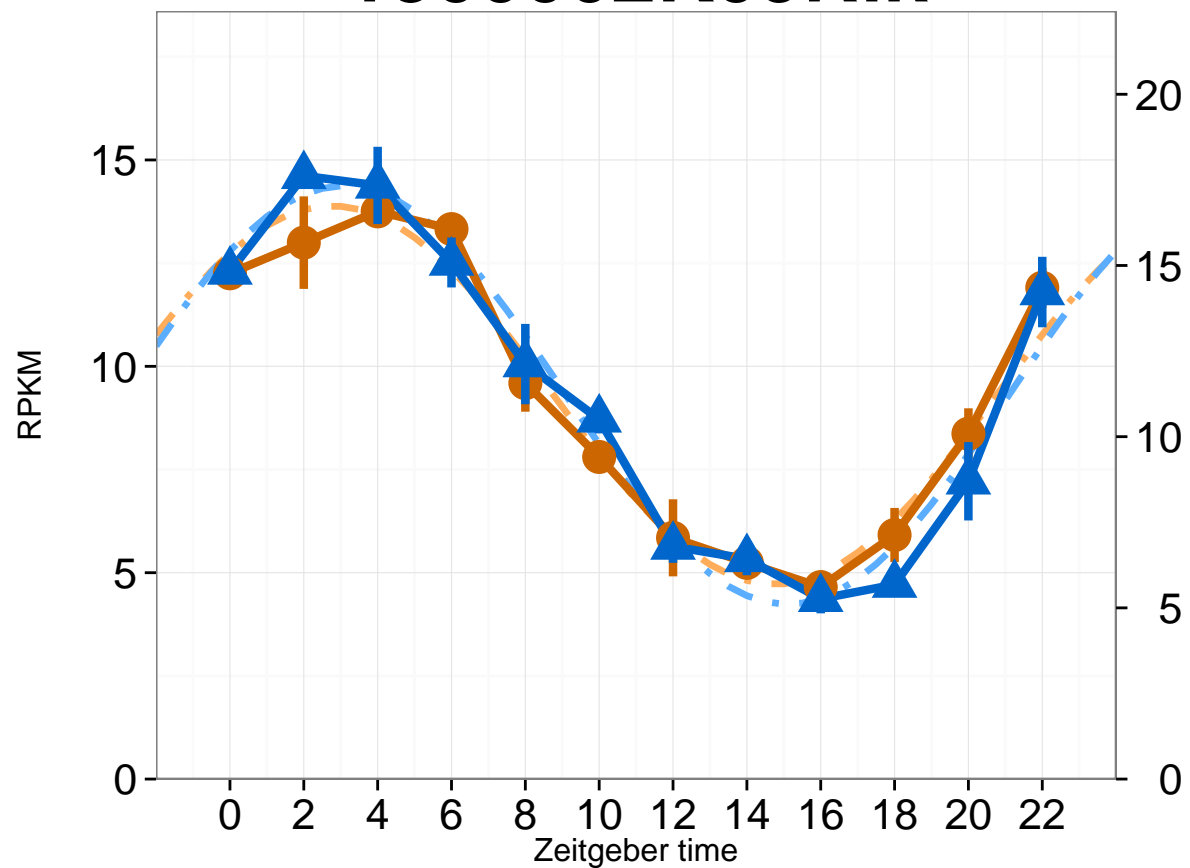

# 1300002K09Rik

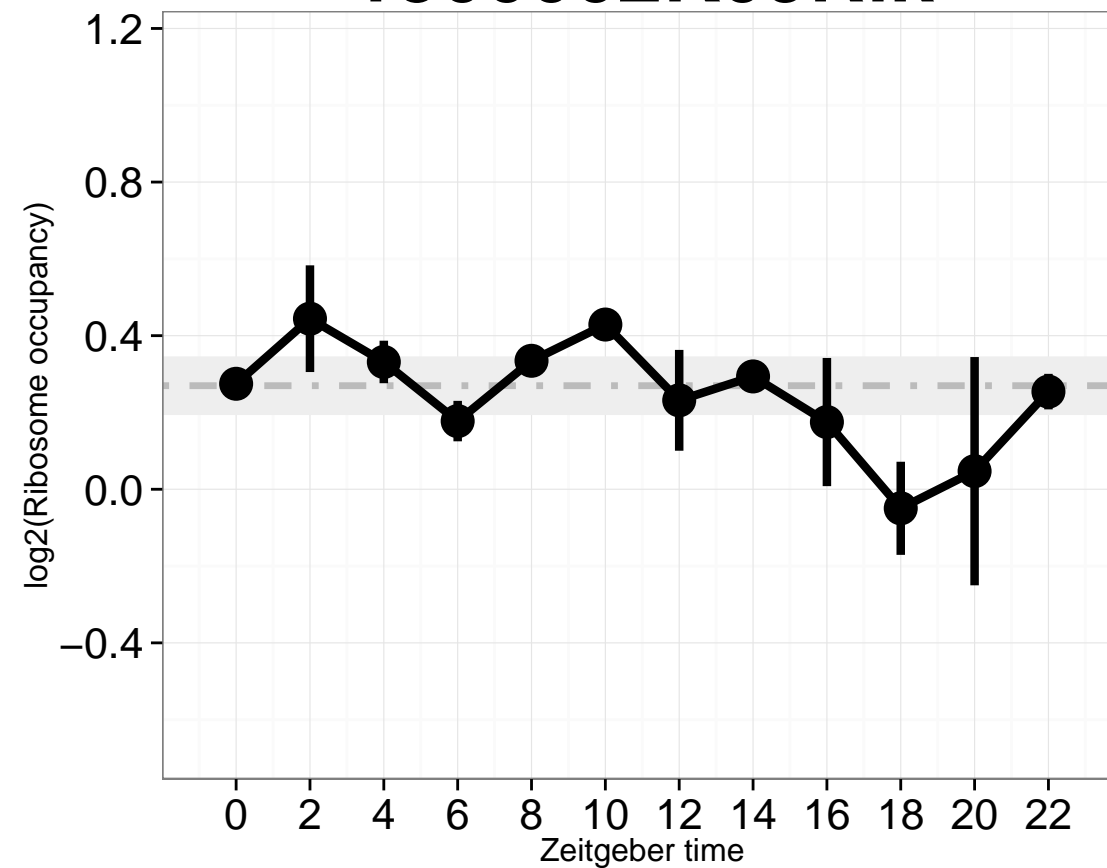

Supplement: Supplementary file 7 — Expression plots for kidney and liver for the 178 common rhythmic genes of Fig. 3c. (ZIP 3338.28 kb) [file 13059_2017_1222_MOESM7_ESM.zip › set_D_shared(178)/1300002K09Rik_kidney_set_D.pdf]

# 1300002K09Rik

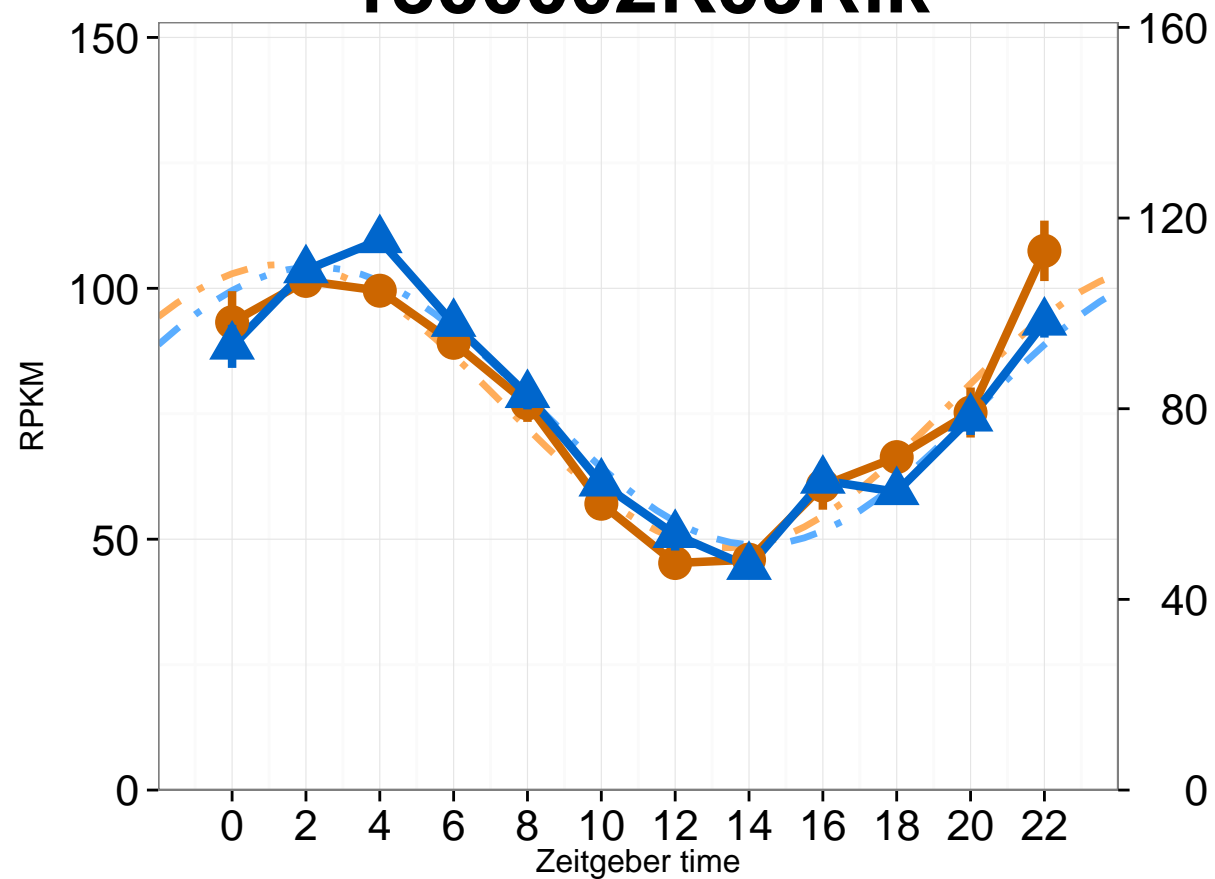

# 1300002K09Rik

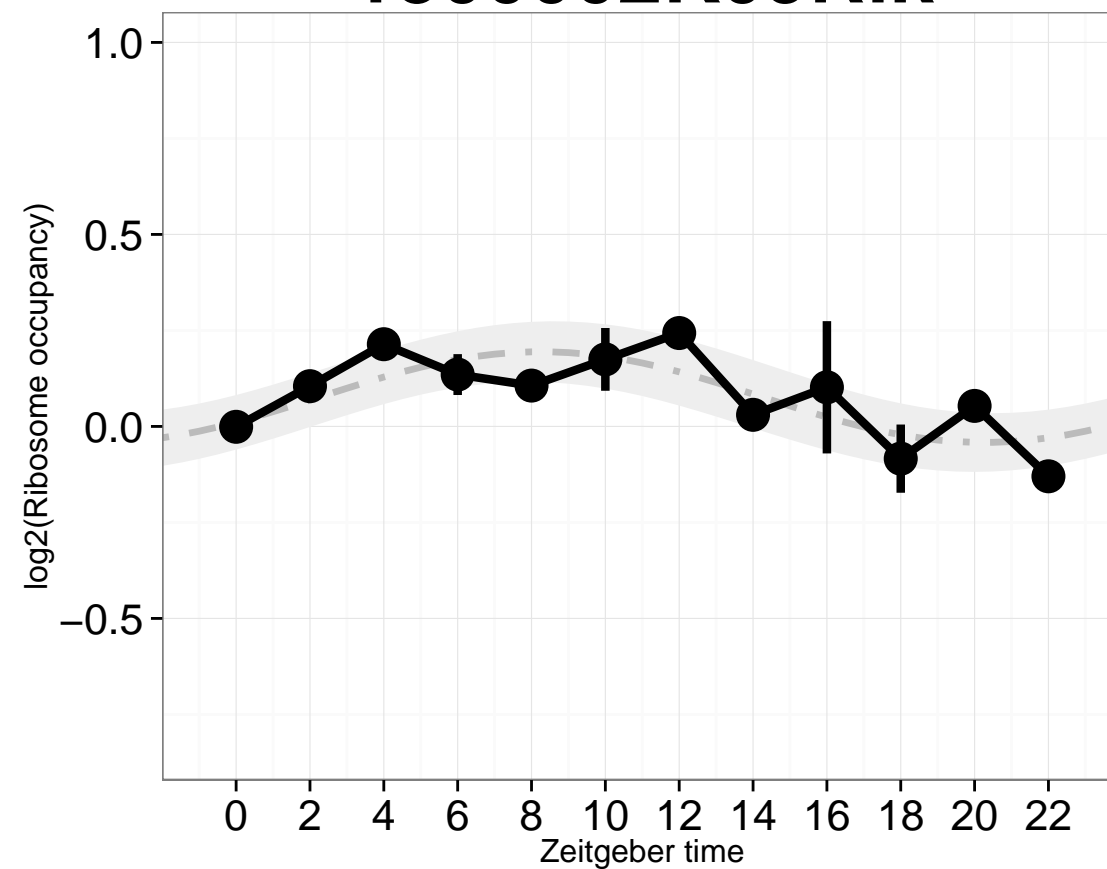

Supplement: Supplementary file 7 — Expression plots for kidney and liver for the 178 common rhythmic genes of Fig. 3c. (ZIP 3338.28 kb) [file 13059_2017_1222_MOESM7_ESM.zip › set_D_shared(178)/1300002K09Rik_liver_set_D.pdf]

# 3010026O09Rik

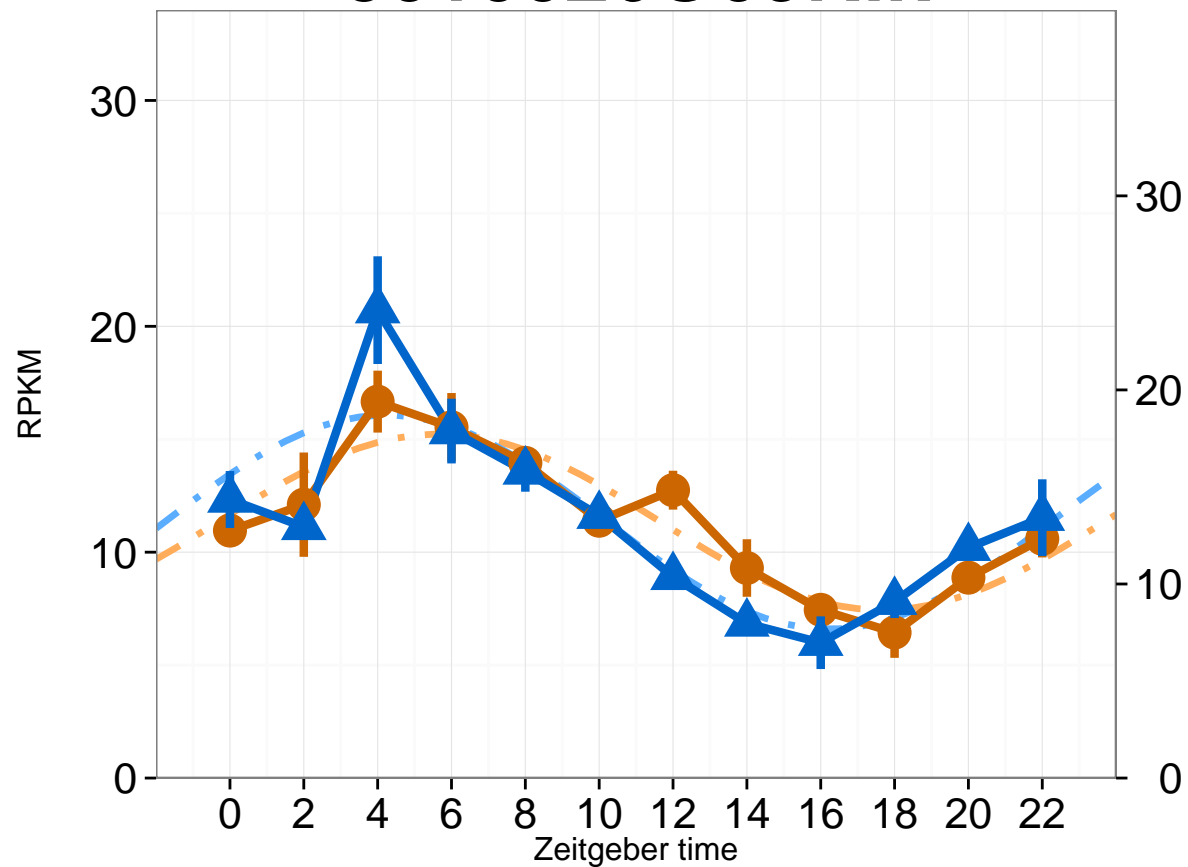

# 3010026O09Rik

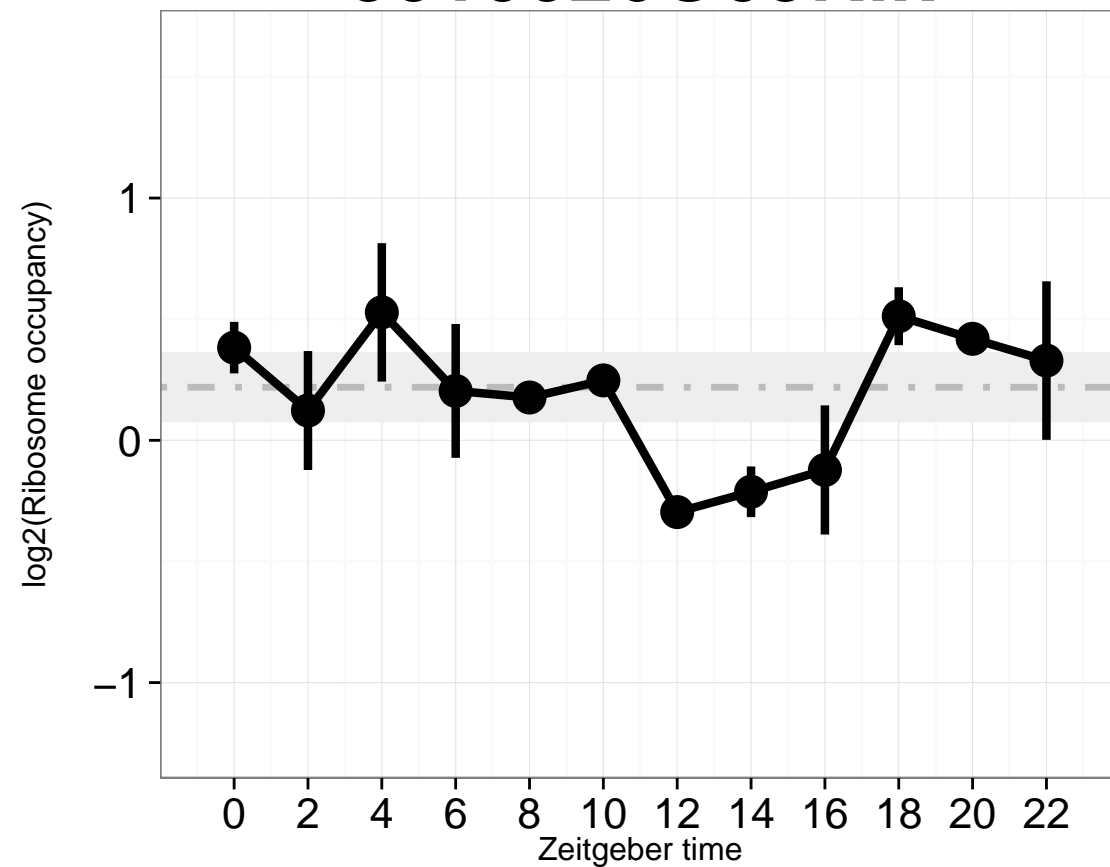

Supplement: Supplementary file 7 — Expression plots for kidney and liver for the 178 common rhythmic genes of Fig. 3c. (ZIP 3338.28 kb) [file 13059_2017_1222_MOESM7_ESM.zip › set_D_shared(178)/3010026O09Rik_kidney_set_D.pdf]

# 3010026O09Rik

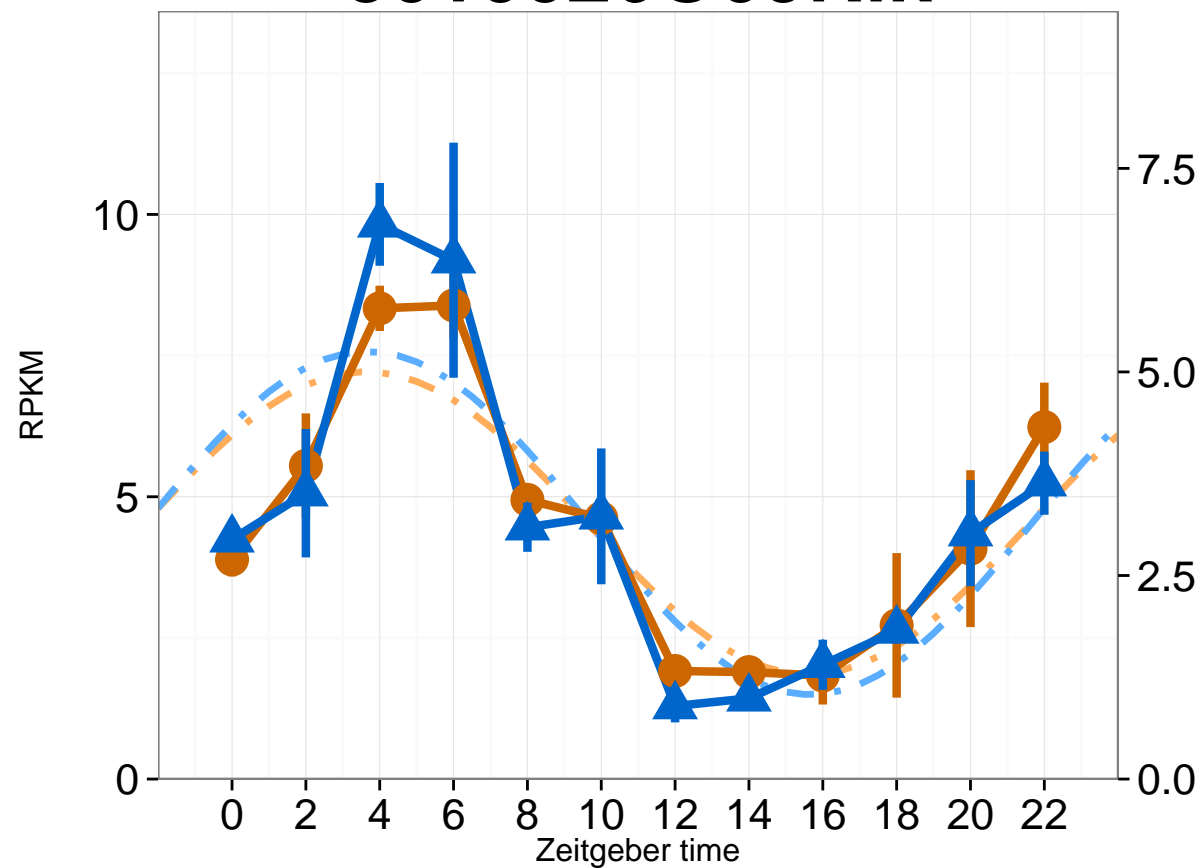

# 3010026O09Rik

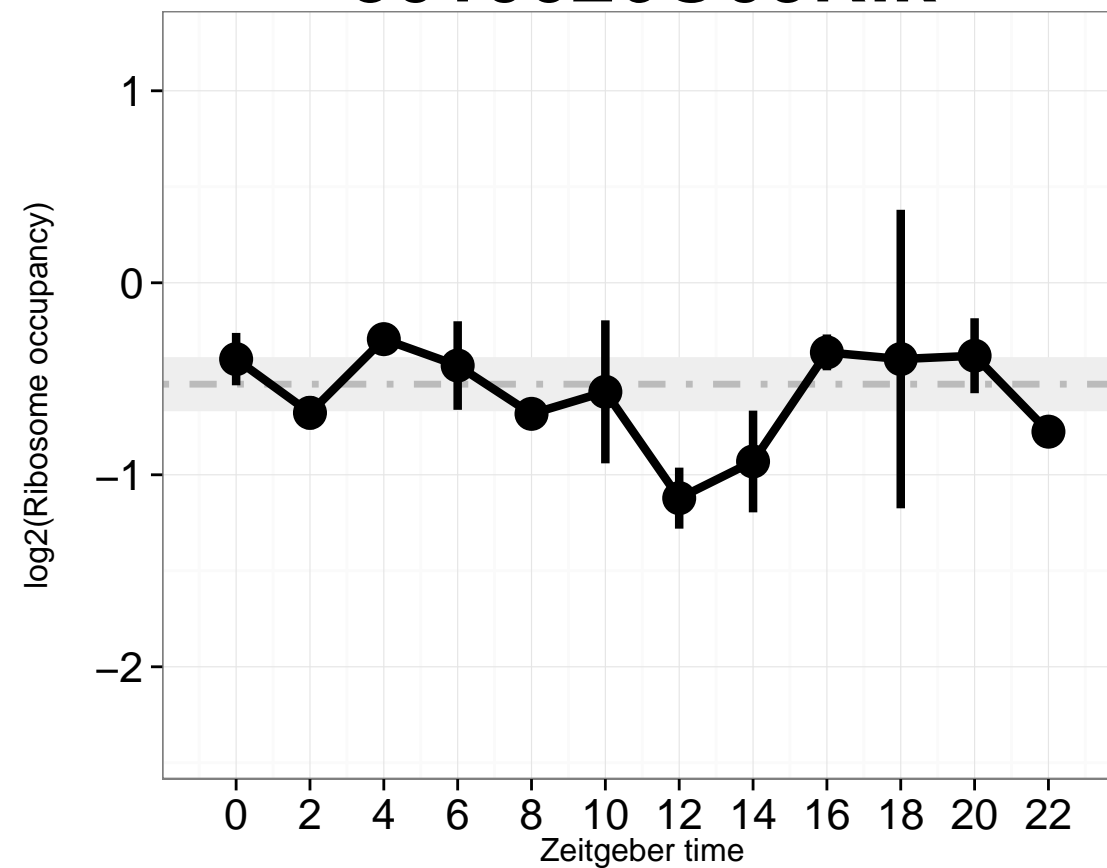

Supplement: Supplementary file 7 — Expression plots for kidney and liver for the 178 common rhythmic genes of Fig. 3c. (ZIP 3338.28 kb) [file 13059_2017_1222_MOESM7_ESM.zip › set_D_shared(178)/3010026O09Rik_liver_set_D.pdf]

## Acmsd

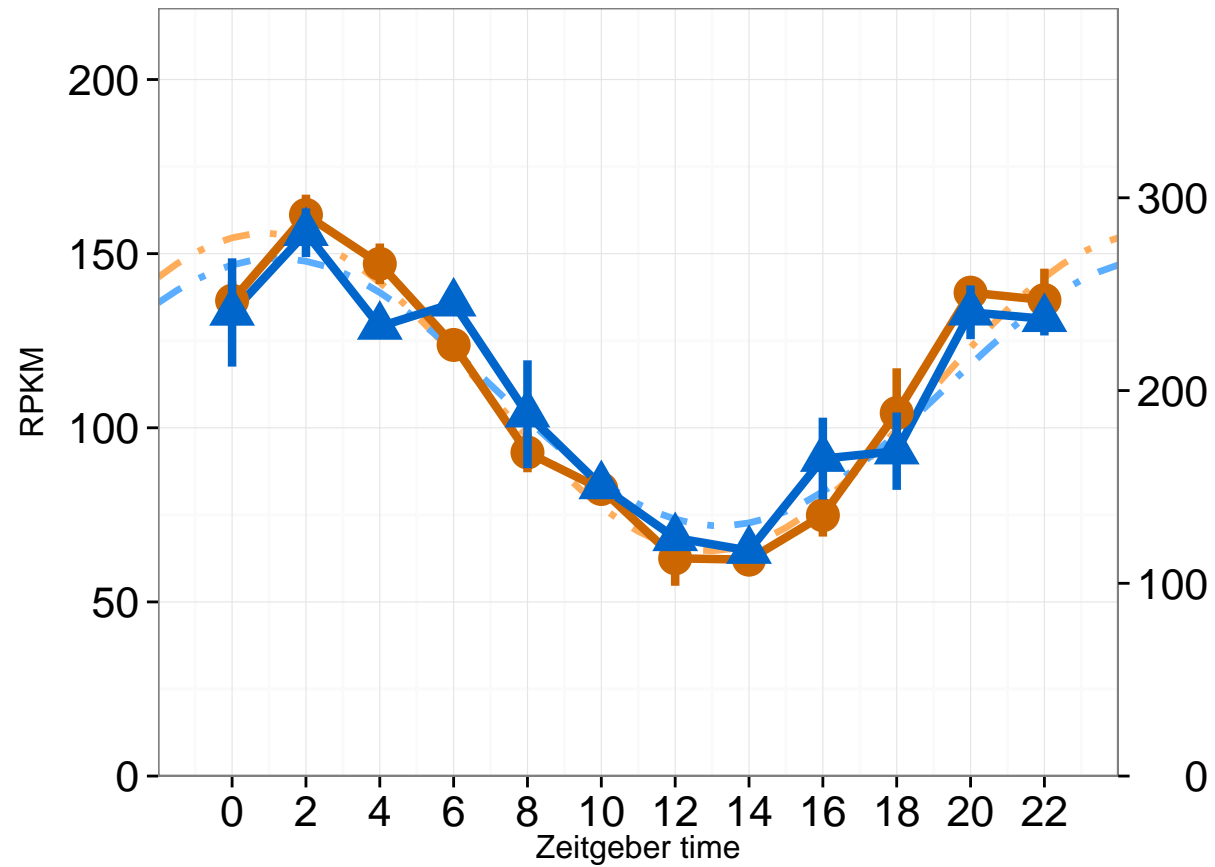

## Acmsd

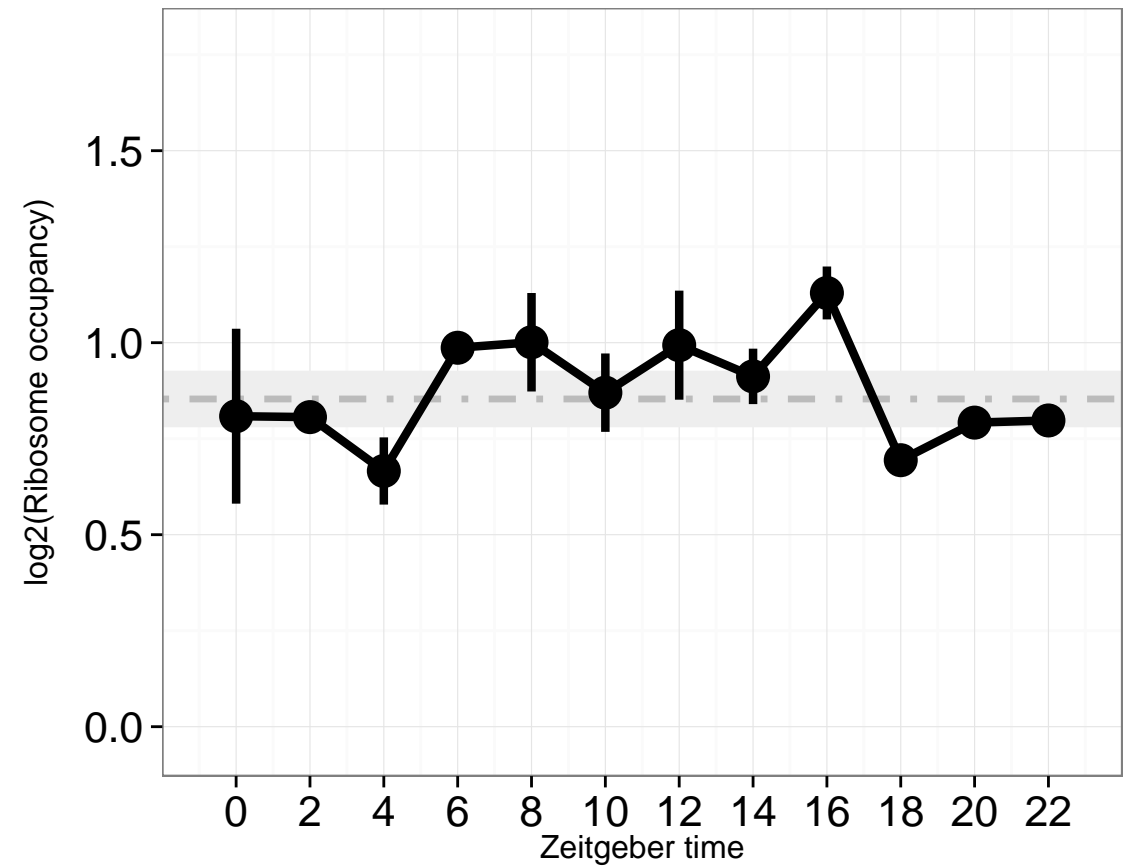

Supplement: Supplementary file 7 — Expression plots for kidney and liver for the 178 common rhythmic genes of Fig. 3c. (ZIP 3338.28 kb) [file 13059_2017_1222_MOESM7_ESM.zip › set_D_shared(178)/Acmsd_kidney_set_D.pdf]

# Acmsd

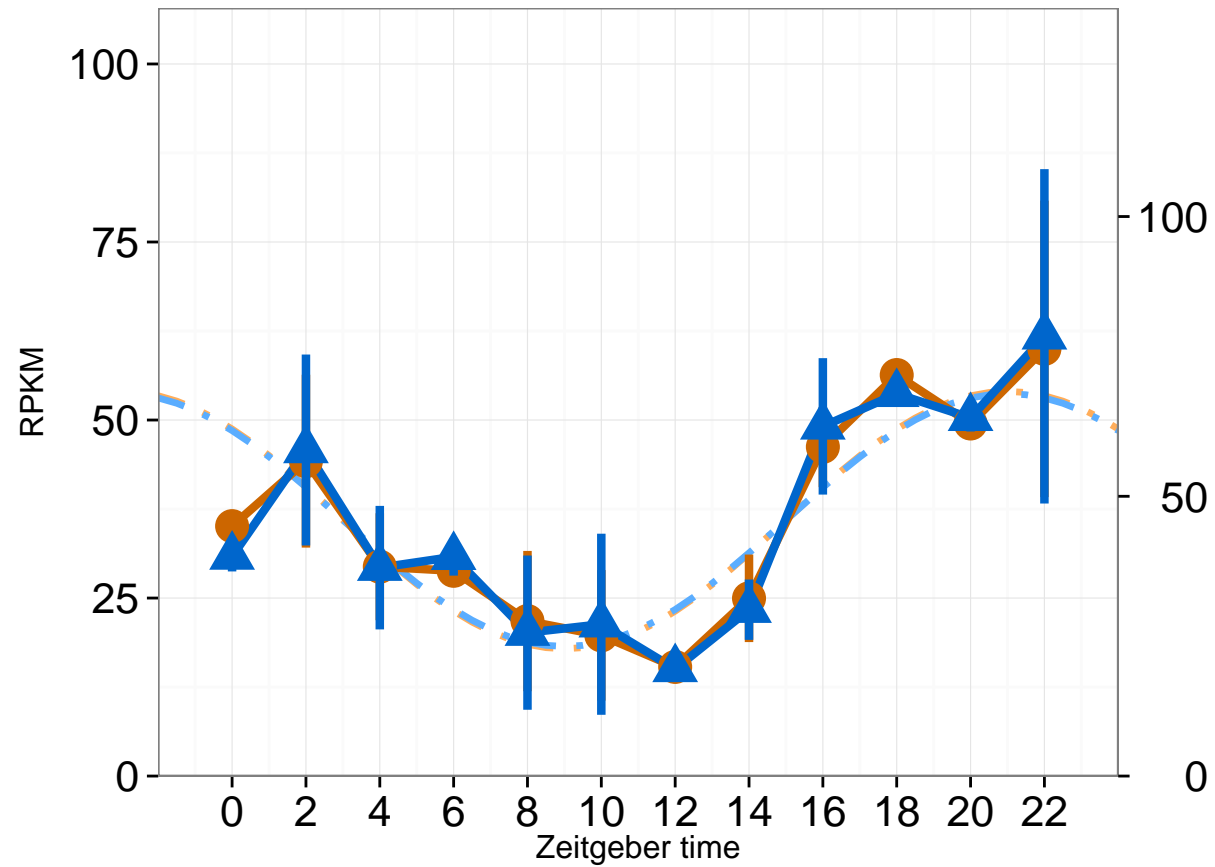

# Acmsd

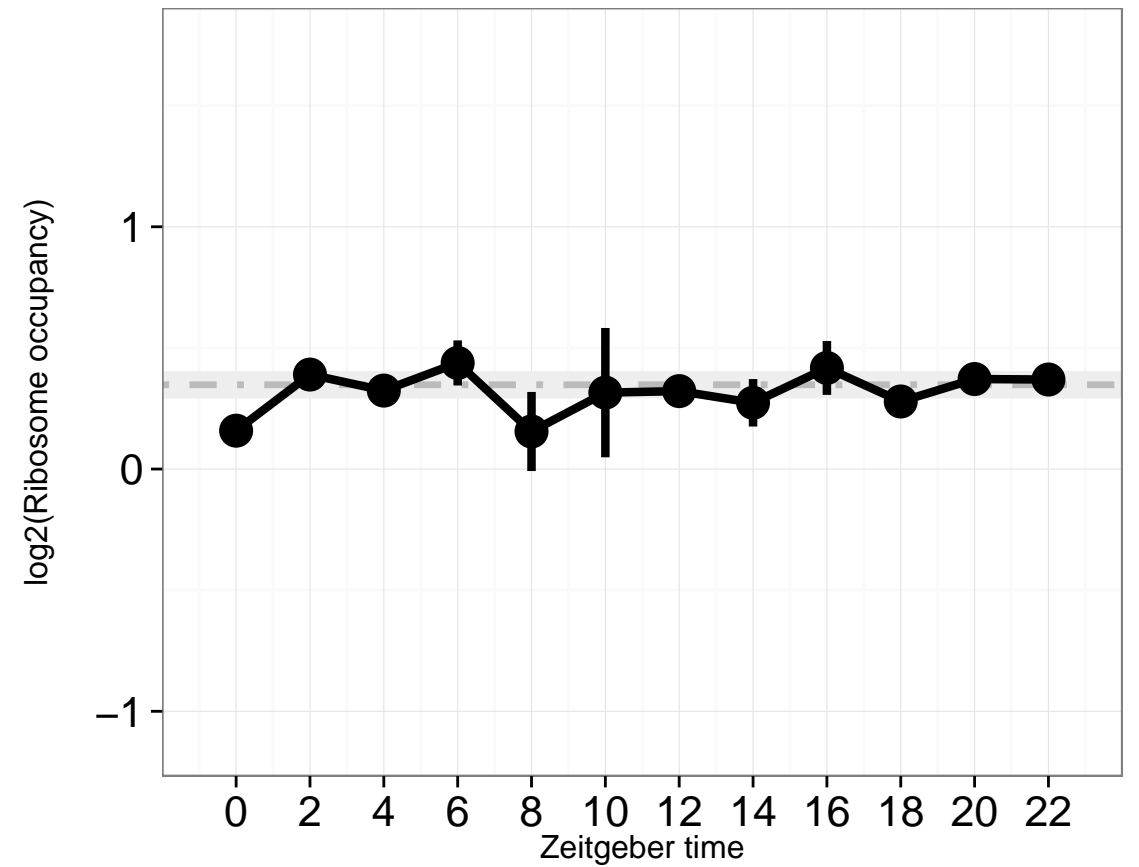

Supplement: Supplementary file 7 — Expression plots for kidney and liver for the 178 common rhythmic genes of Fig. 3c. (ZIP 3338.28 kb) [file 13059_2017_1222_MOESM7_ESM.zip › set_D_shared(178)/Acmsd_liver_set_D.pdf]

# Acnat1

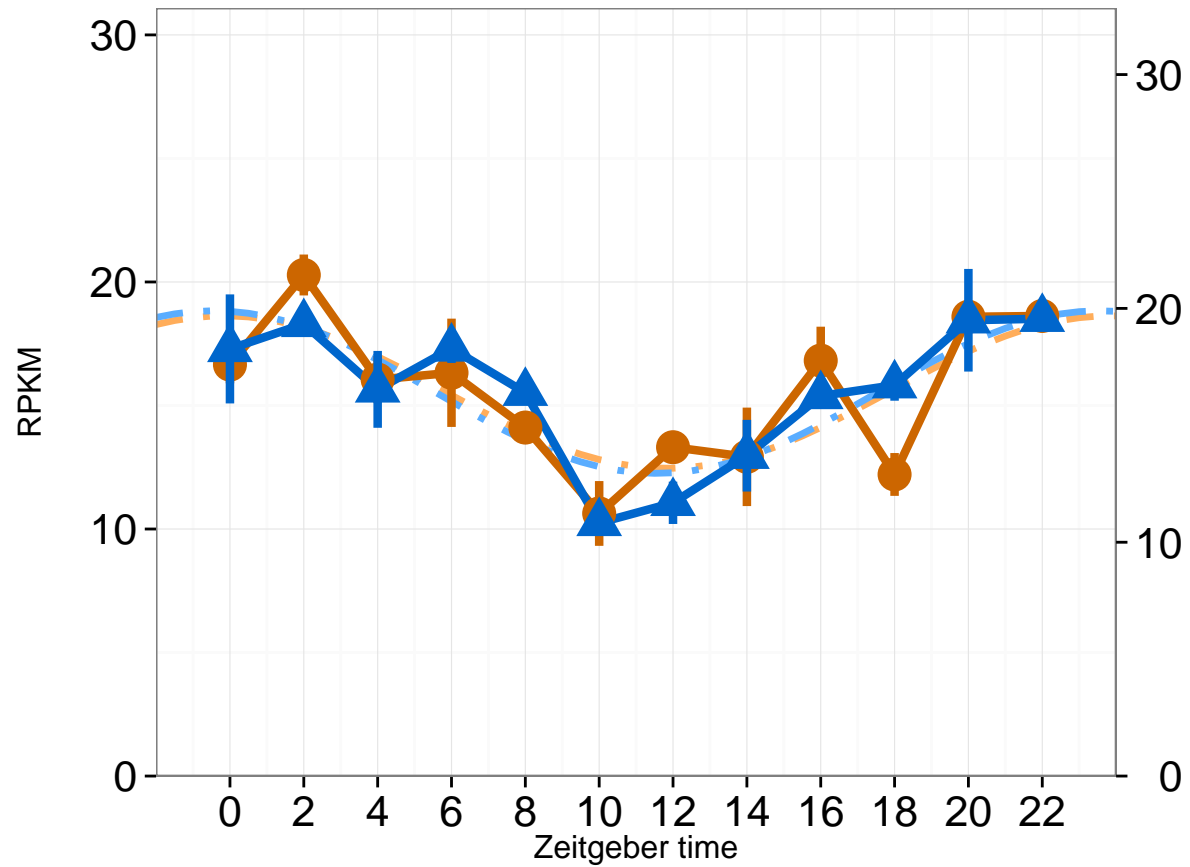

# Acnat1

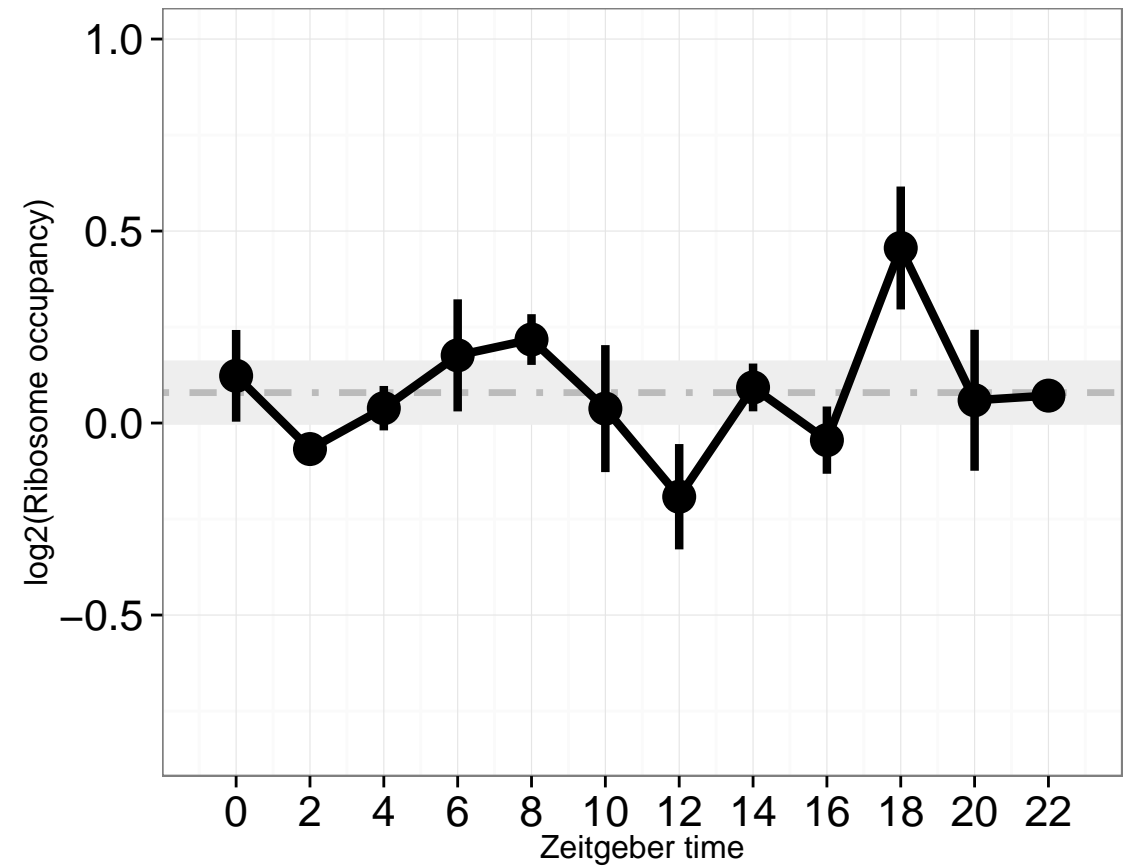

Supplement: Supplementary file 7 — Expression plots for kidney and liver for the 178 common rhythmic genes of Fig. 3c. (ZIP 3338.28 kb) [file 13059_2017_1222_MOESM7_ESM.zip › set_D_shared(178)/Acnat1_kidney_set_D.pdf]

# Acnat1

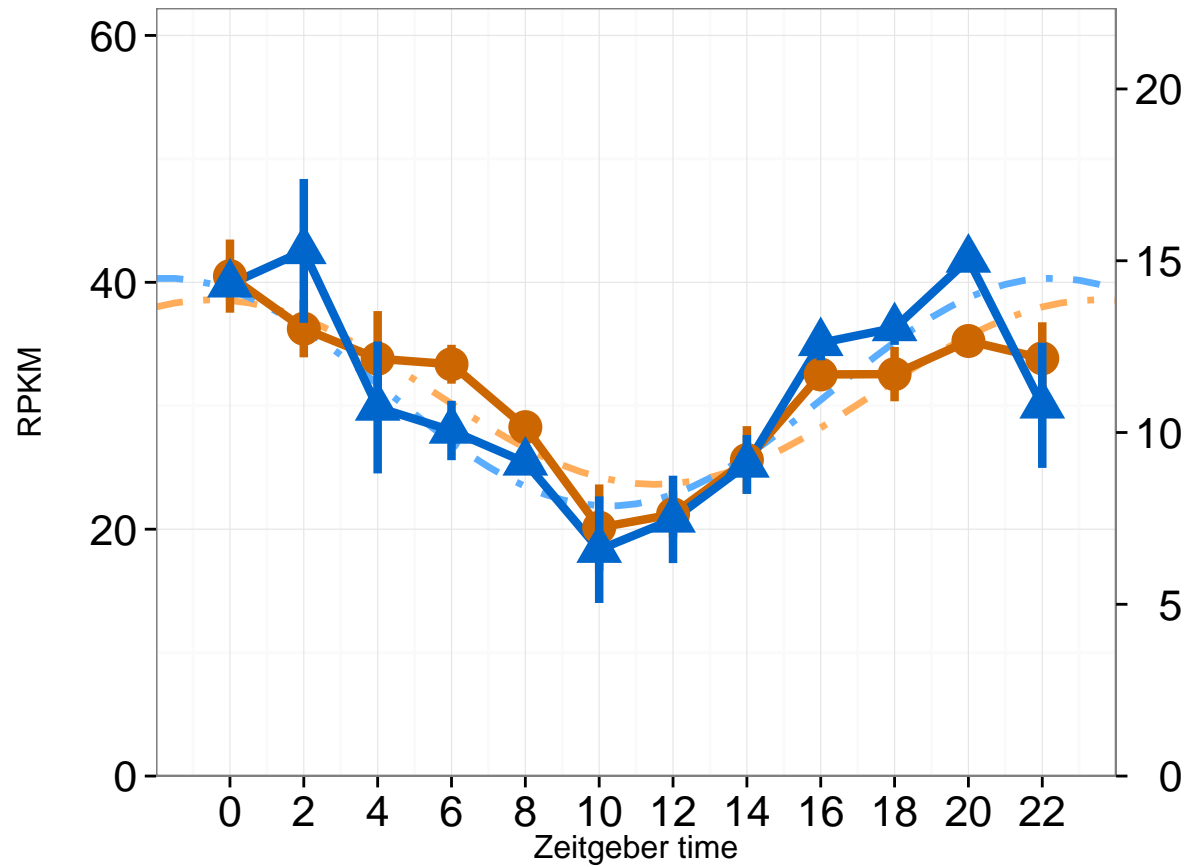

# Acnat1

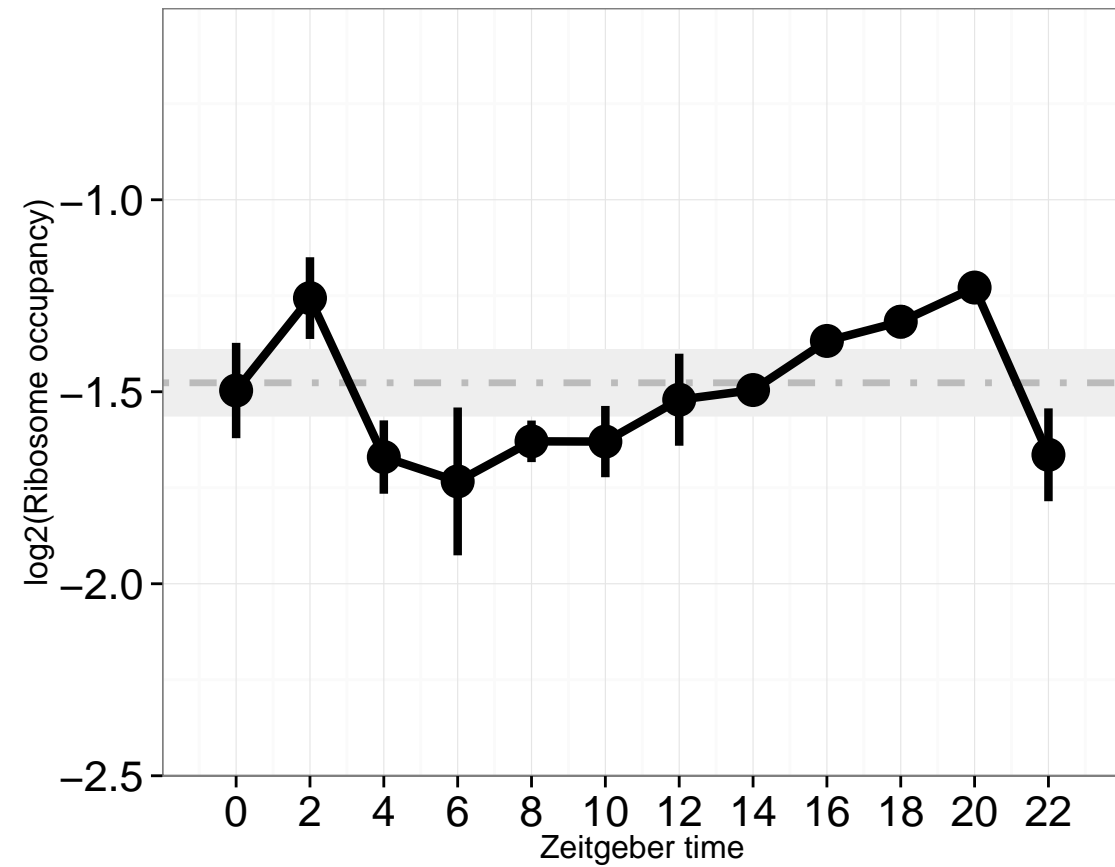

Supplement: Supplementary file 7 — Expression plots for kidney and liver for the 178 common rhythmic genes of Fig. 3c. (ZIP 3338.28 kb) [file 13059_2017_1222_MOESM7_ESM.zip › set_D_shared(178)/Acnat1_liver_set_D.pdf]

# Acnat2

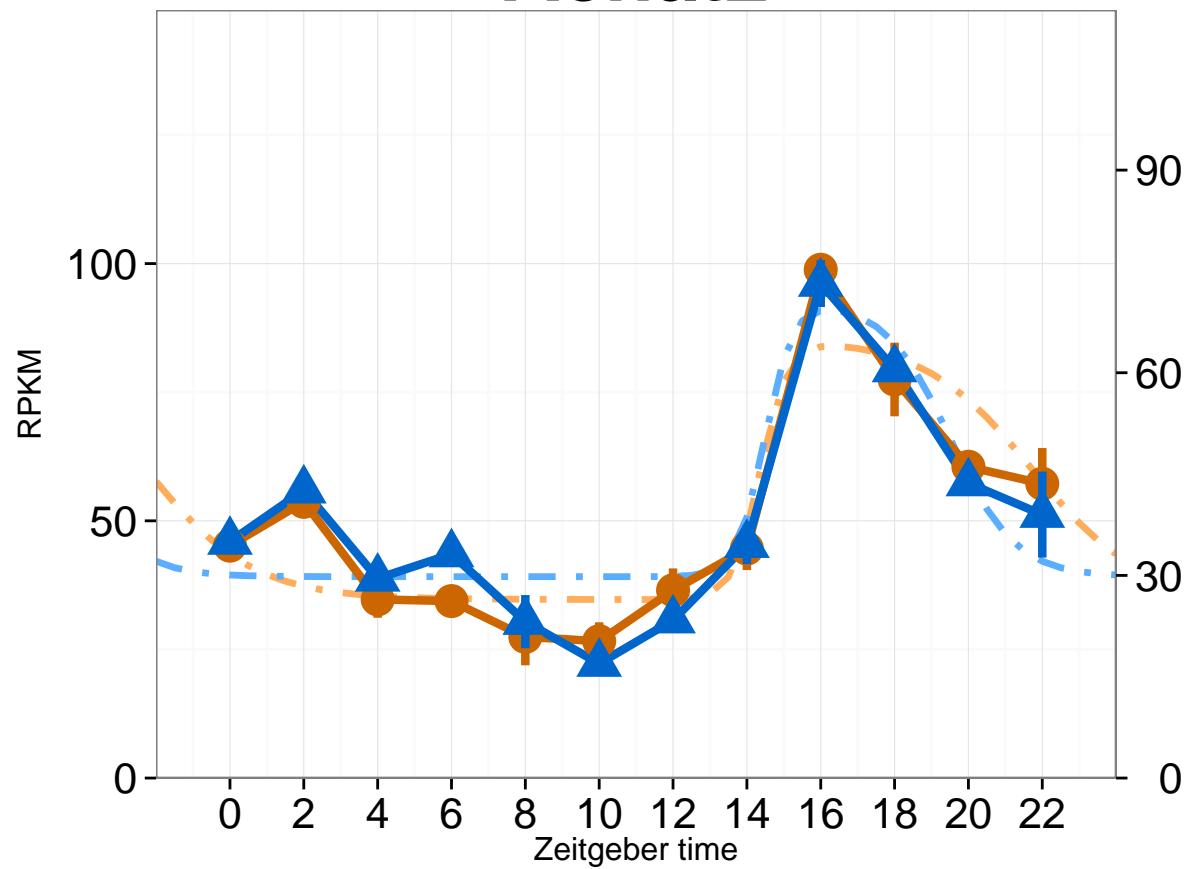

# Acnat2

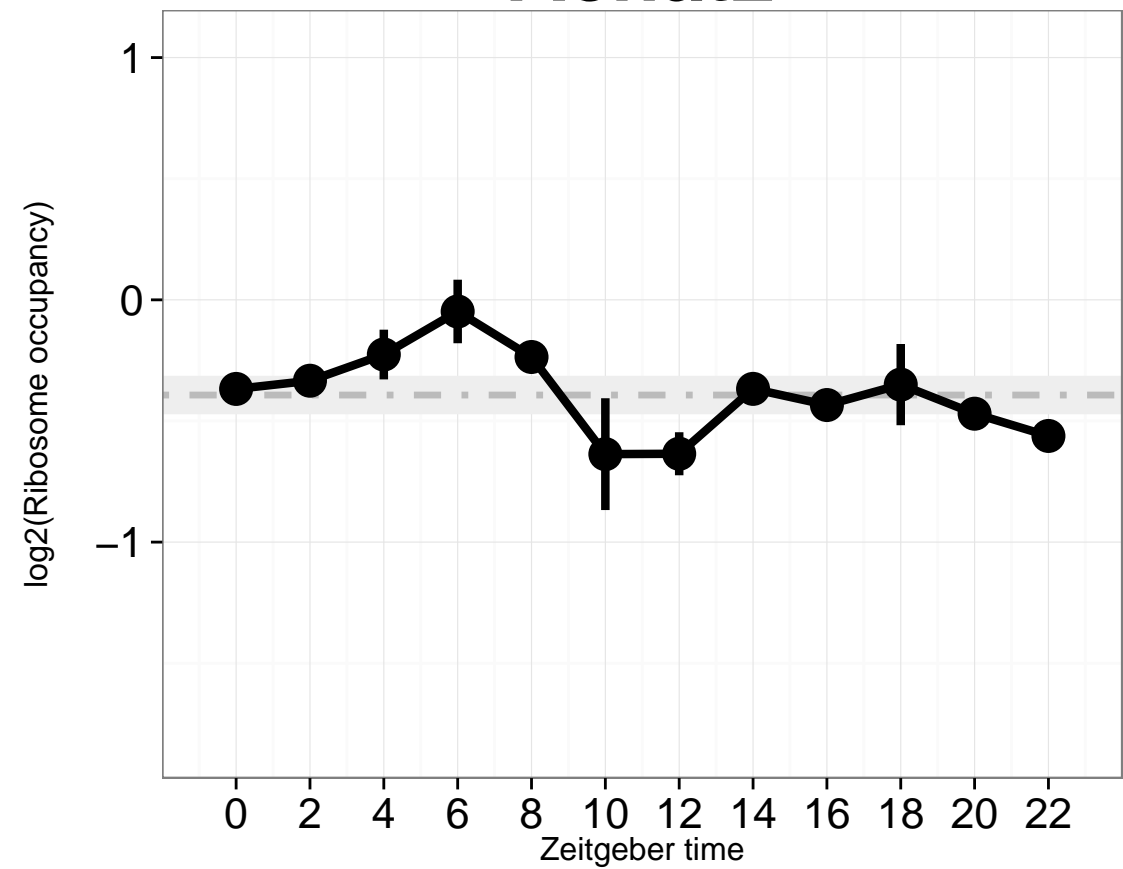

Supplement: Supplementary file 7 — Expression plots for kidney and liver for the 178 common rhythmic genes of Fig. 3c. (ZIP 3338.28 kb) [file 13059_2017_1222_MOESM7_ESM.zip › set_D_shared(178)/Acnat2_kidney_set_D.pdf]

## Acnat2

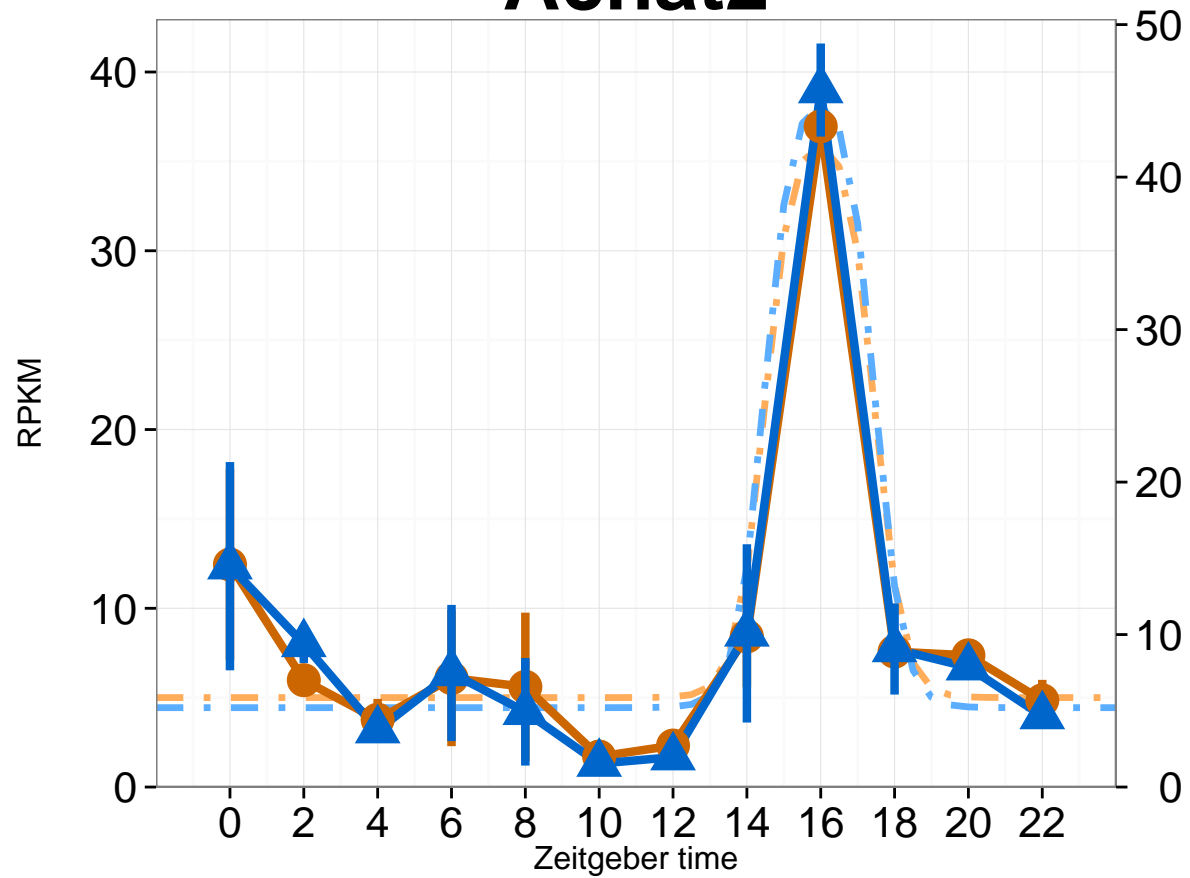

## Acnat2

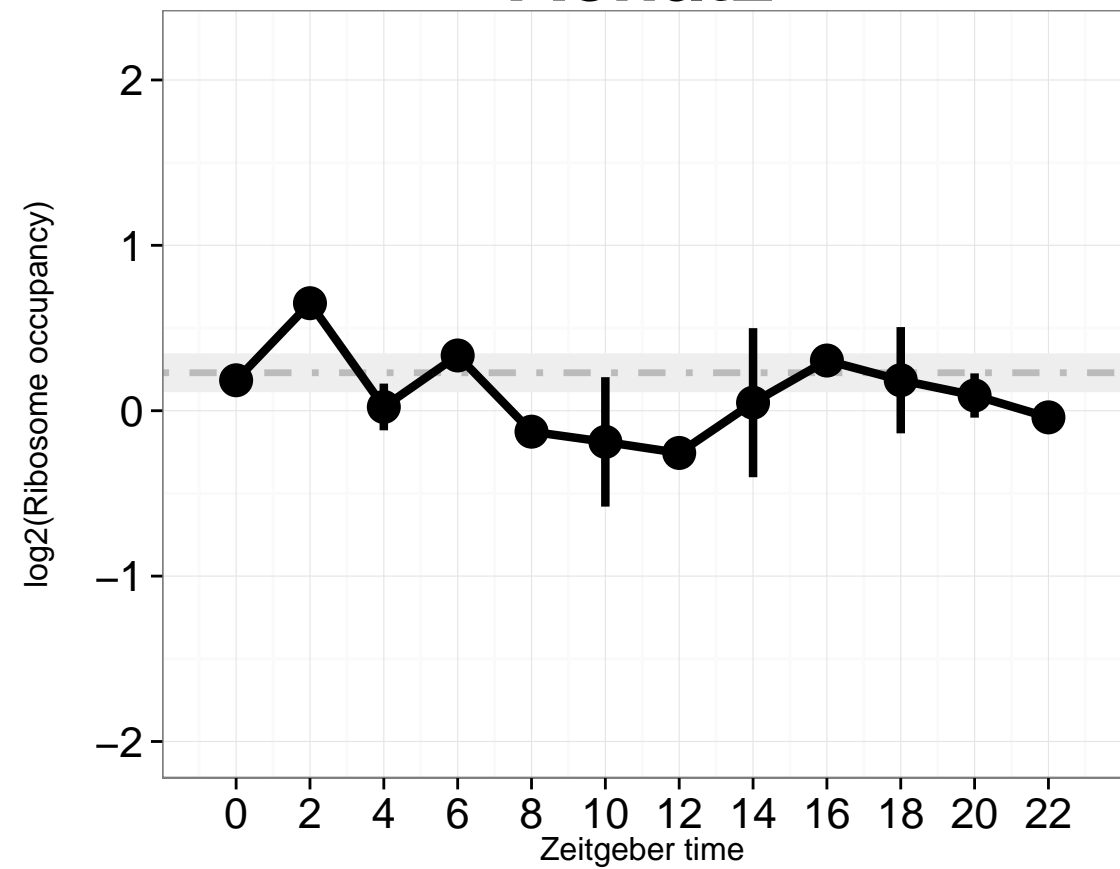

Supplement: Supplementary file 7 — Expression plots for kidney and liver for the 178 common rhythmic genes of Fig. 3c. (ZIP 3338.28 kb) [file 13059_2017_1222_MOESM7_ESM.zip › set_D_shared(178)/Acnat2_liver_set_D.pdf]

## Adck5

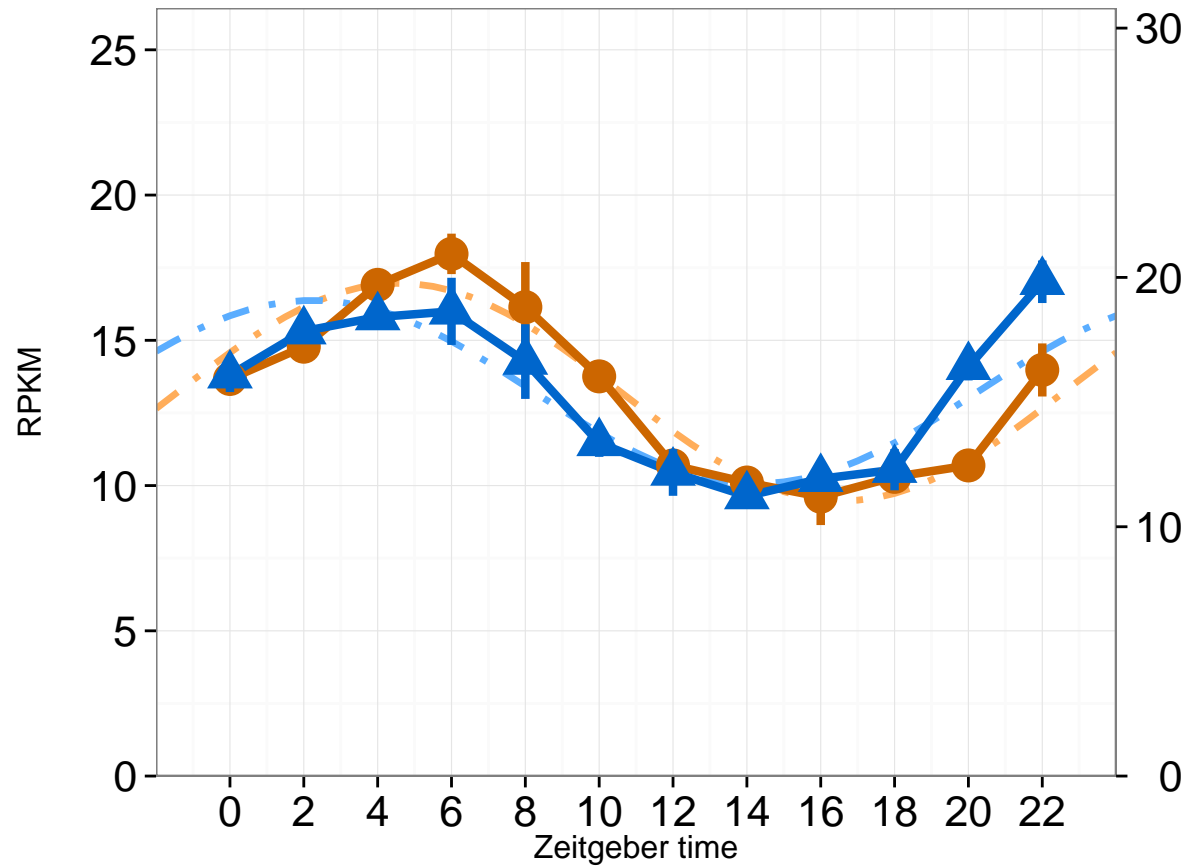

## Adck5

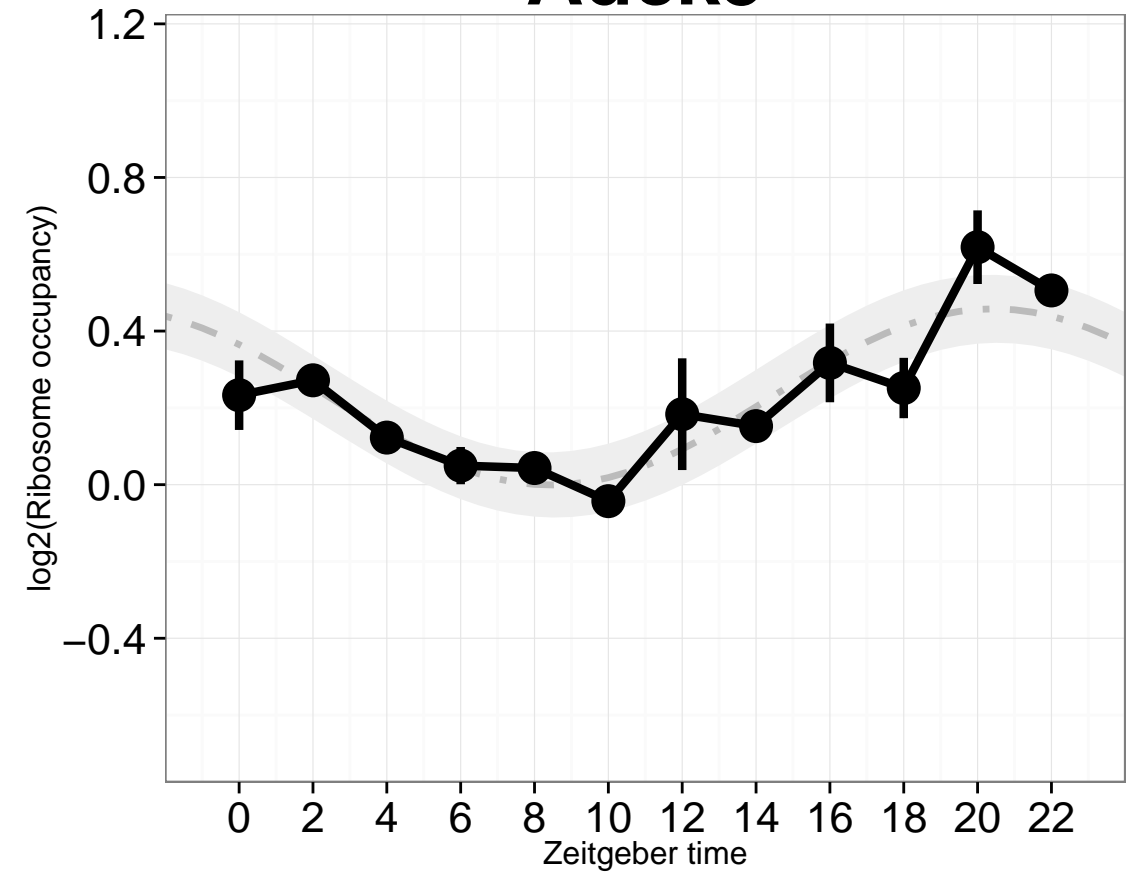

Supplement: Supplementary file 7 — Expression plots for kidney and liver for the 178 common rhythmic genes of Fig. 3c. (ZIP 3338.28 kb) [file 13059_2017_1222_MOESM7_ESM.zip › set_D_shared(178)/Adck5_kidney_set_D.pdf]

## Adck5

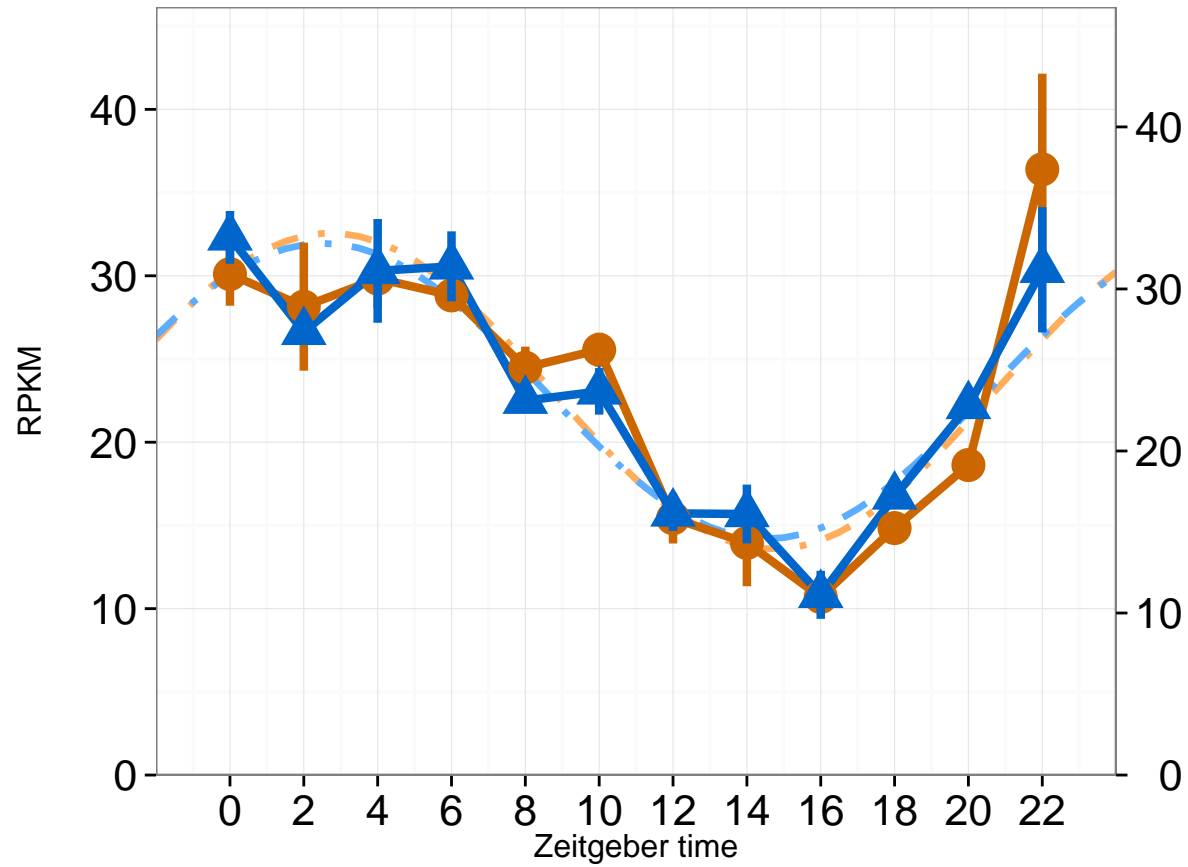

## Adck5

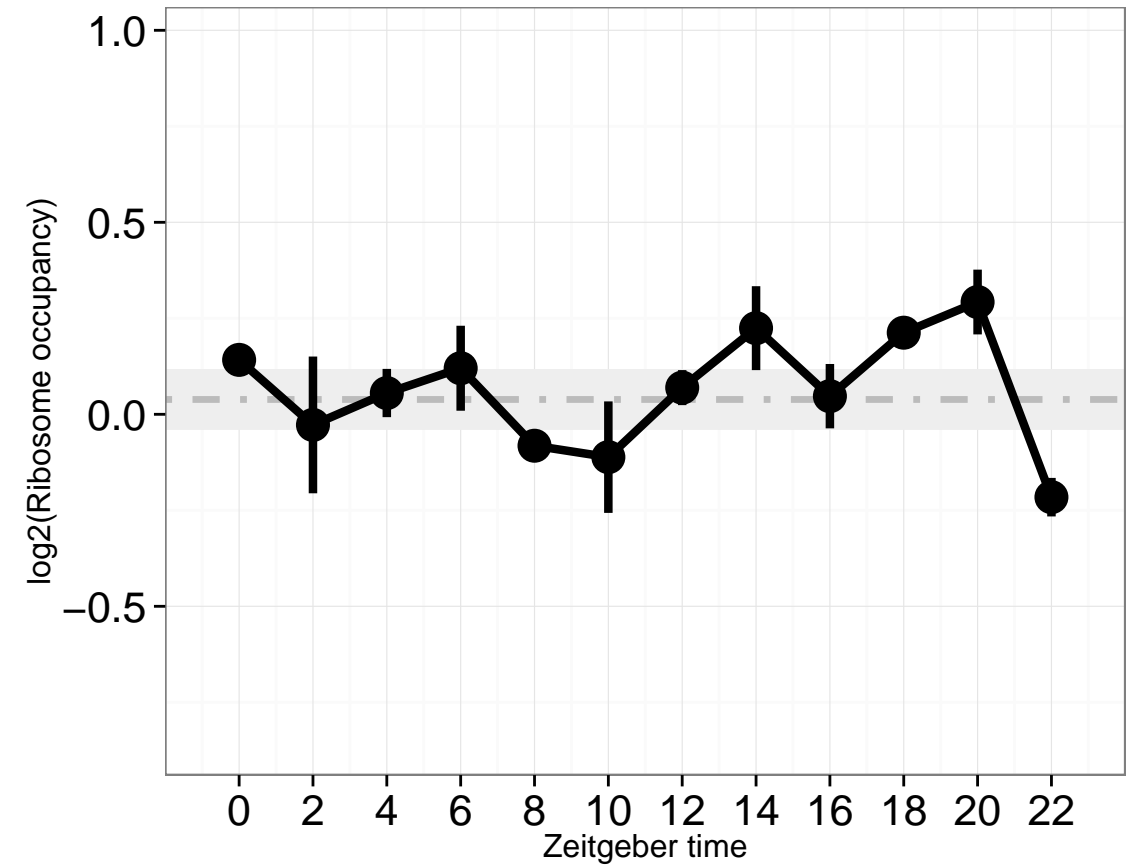

Supplement: Supplementary file 7 — Expression plots for kidney and liver for the 178 common rhythmic genes of Fig. 3c. (ZIP 3338.28 kb) [file 13059_2017_1222_MOESM7_ESM.zip › set_D_shared(178)/Adck5_liver_set_D.pdf]

## Adra2b

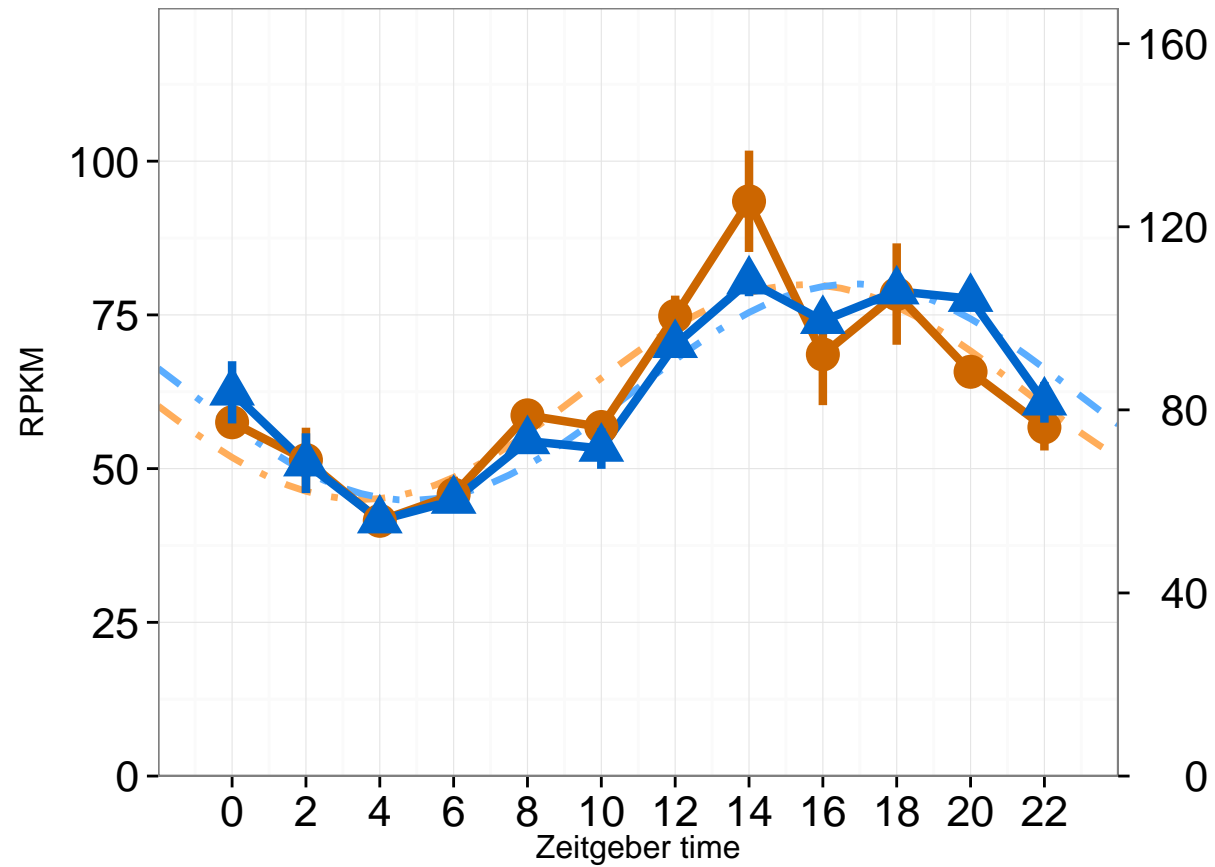

## Adra2b

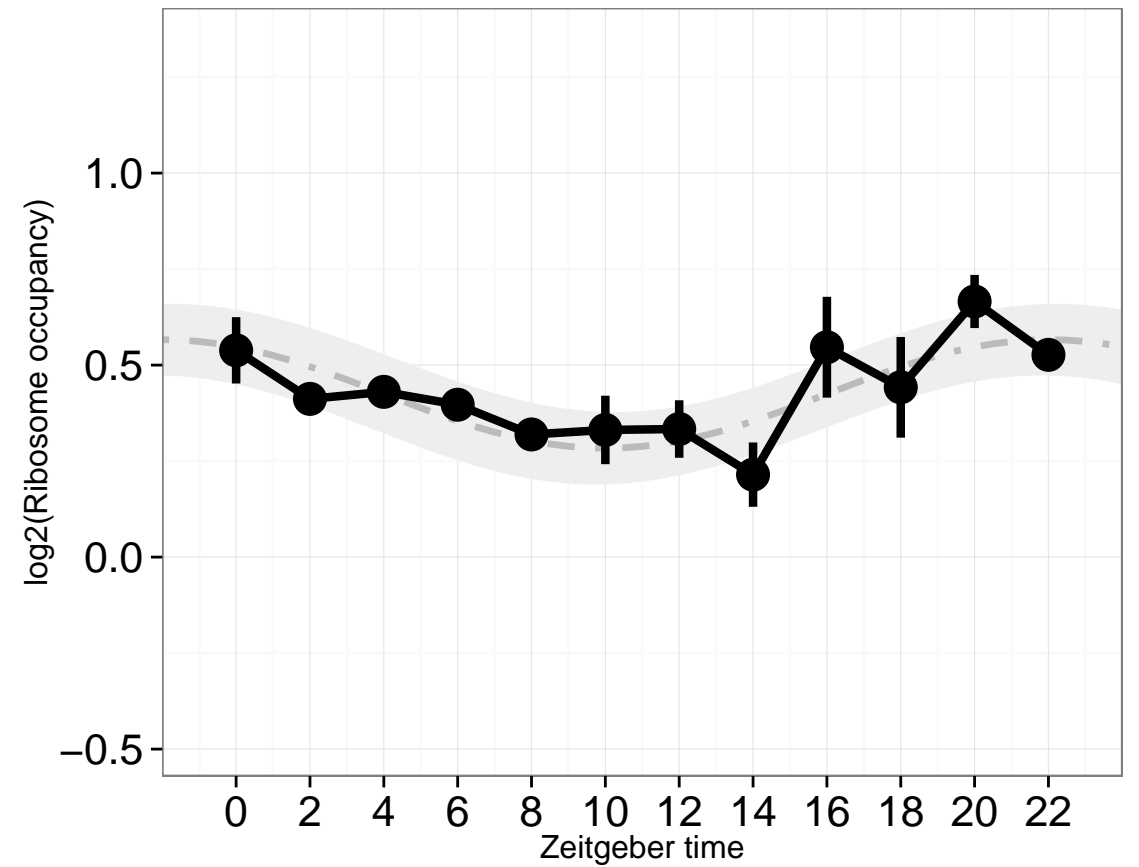

Supplement: Supplementary file 7 — Expression plots for kidney and liver for the 178 common rhythmic genes of Fig. 3c. (ZIP 3338.28 kb) [file 13059_2017_1222_MOESM7_ESM.zip › set_D_shared(178)/Adra2b_kidney_set_D.pdf]

# Adra2b

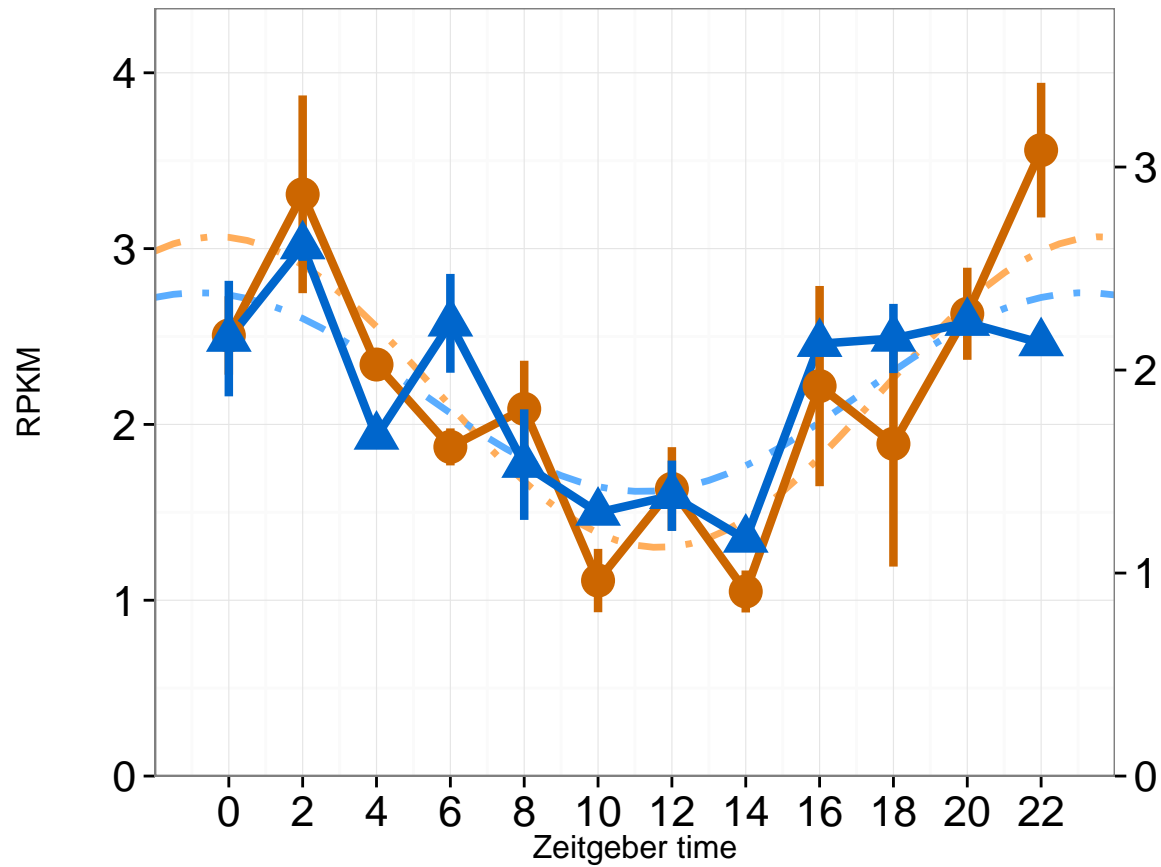

# Adra2b

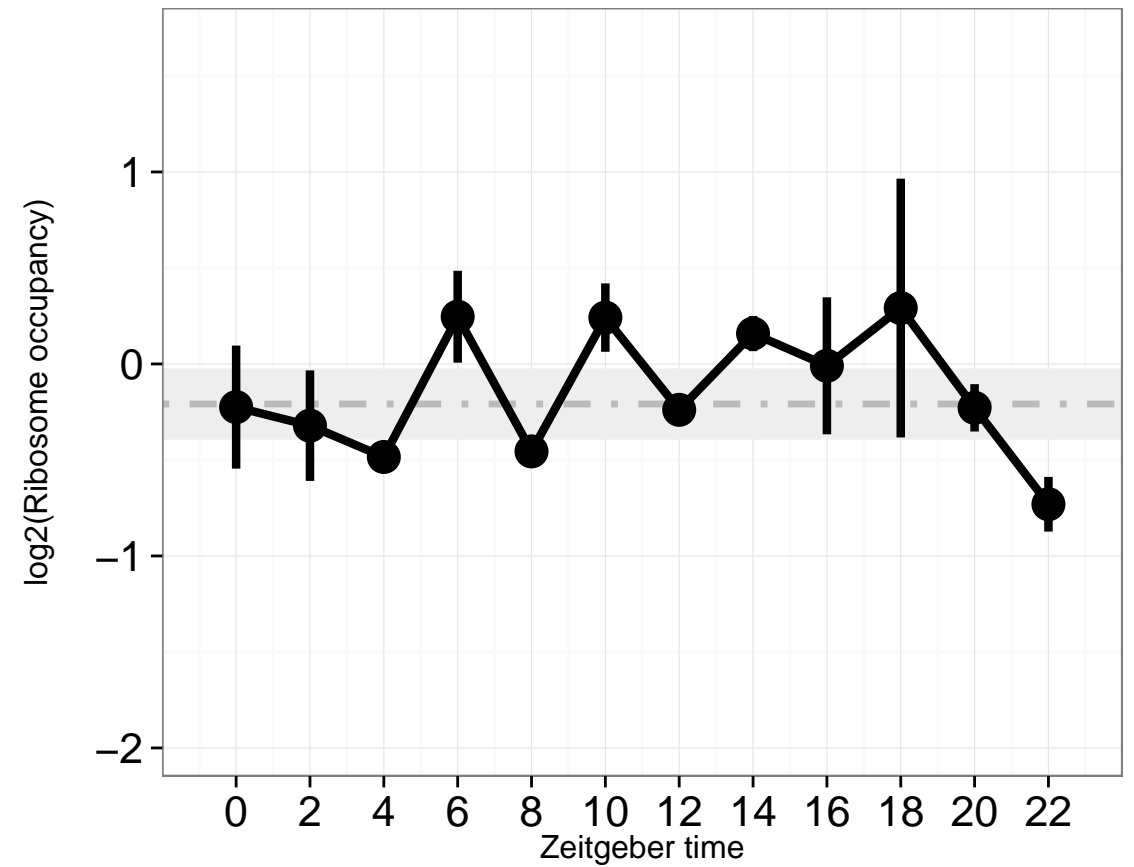

Supplement: Supplementary file 7 — Expression plots for kidney and liver for the 178 common rhythmic genes of Fig. 3c. (ZIP 3338.28 kb) [file 13059_2017_1222_MOESM7_ESM.zip › set_D_shared(178)/Adra2b_liver_set_D.pdf]

## Ahsa2

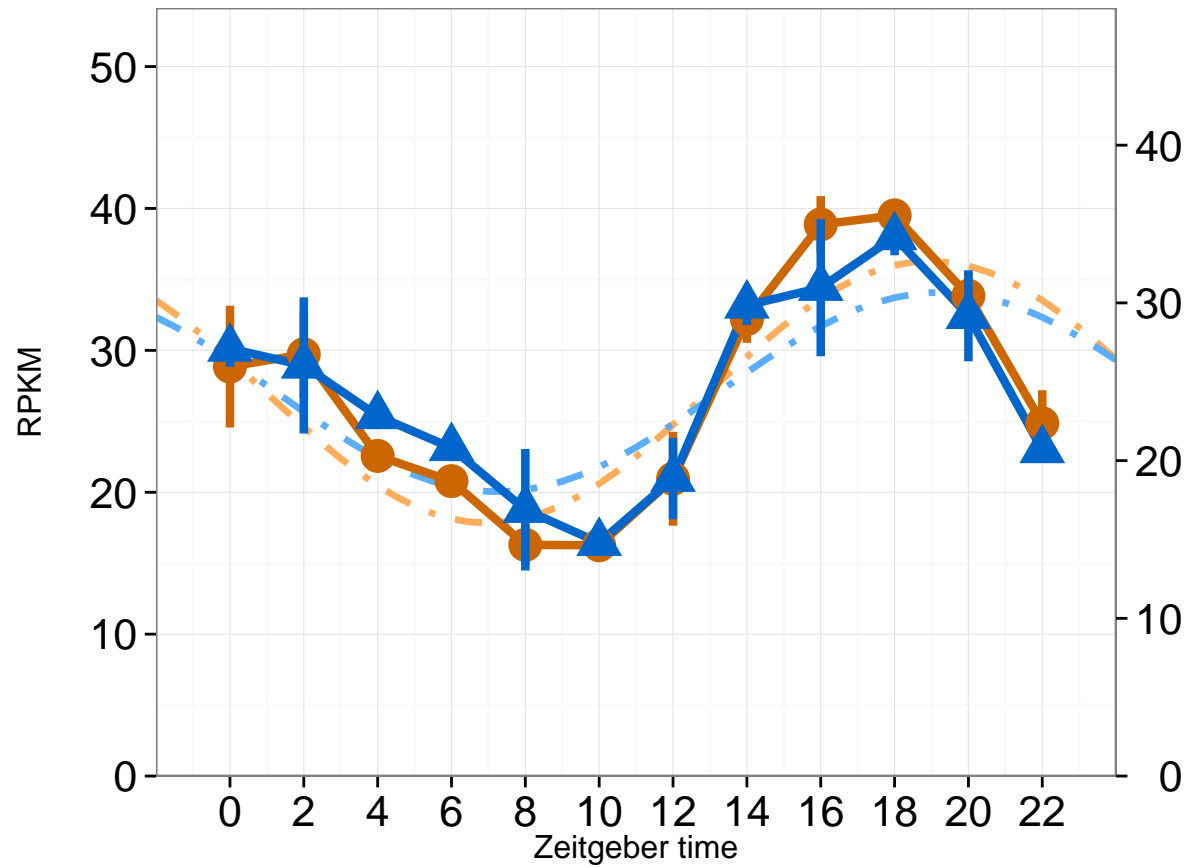

## Ahsa2

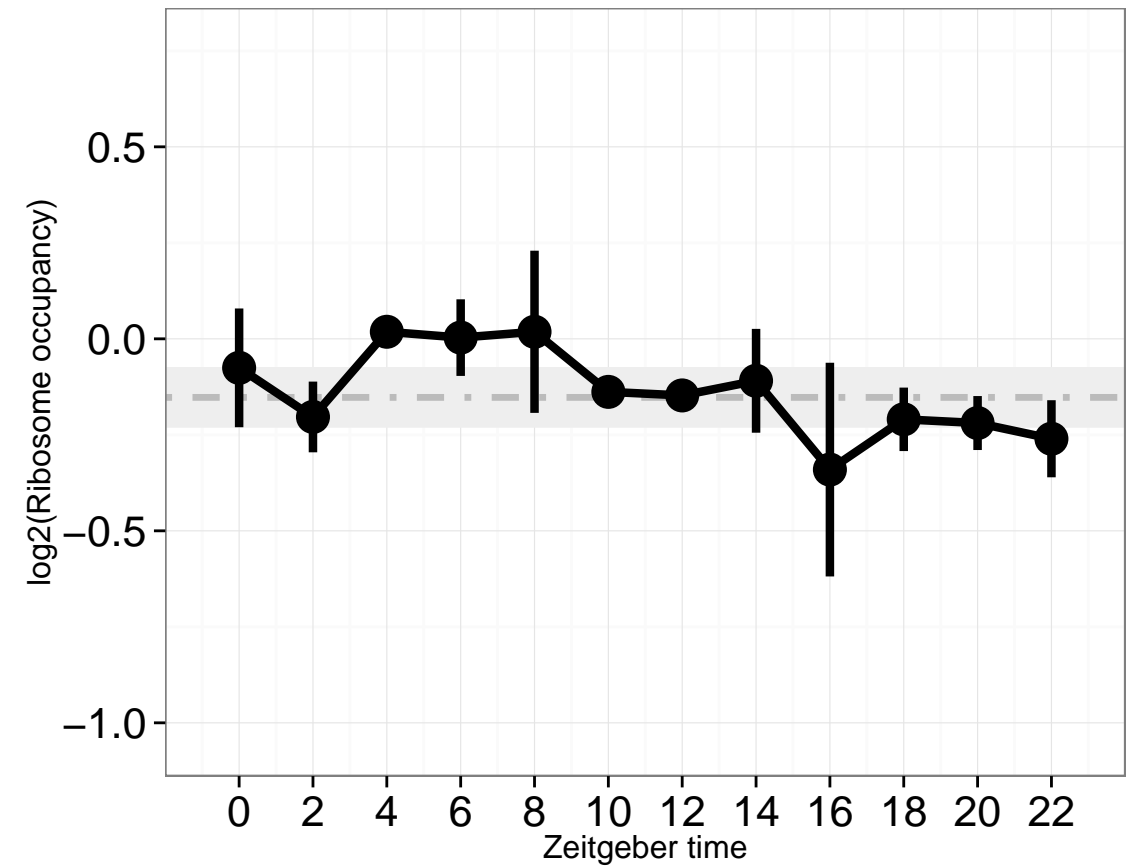

Supplement: Supplementary file 7 — Expression plots for kidney and liver for the 178 common rhythmic genes of Fig. 3c. (ZIP 3338.28 kb) [file 13059_2017_1222_MOESM7_ESM.zip › set_D_shared(178)/Ahsa2_kidney_set_D.pdf]

## Ahsa2

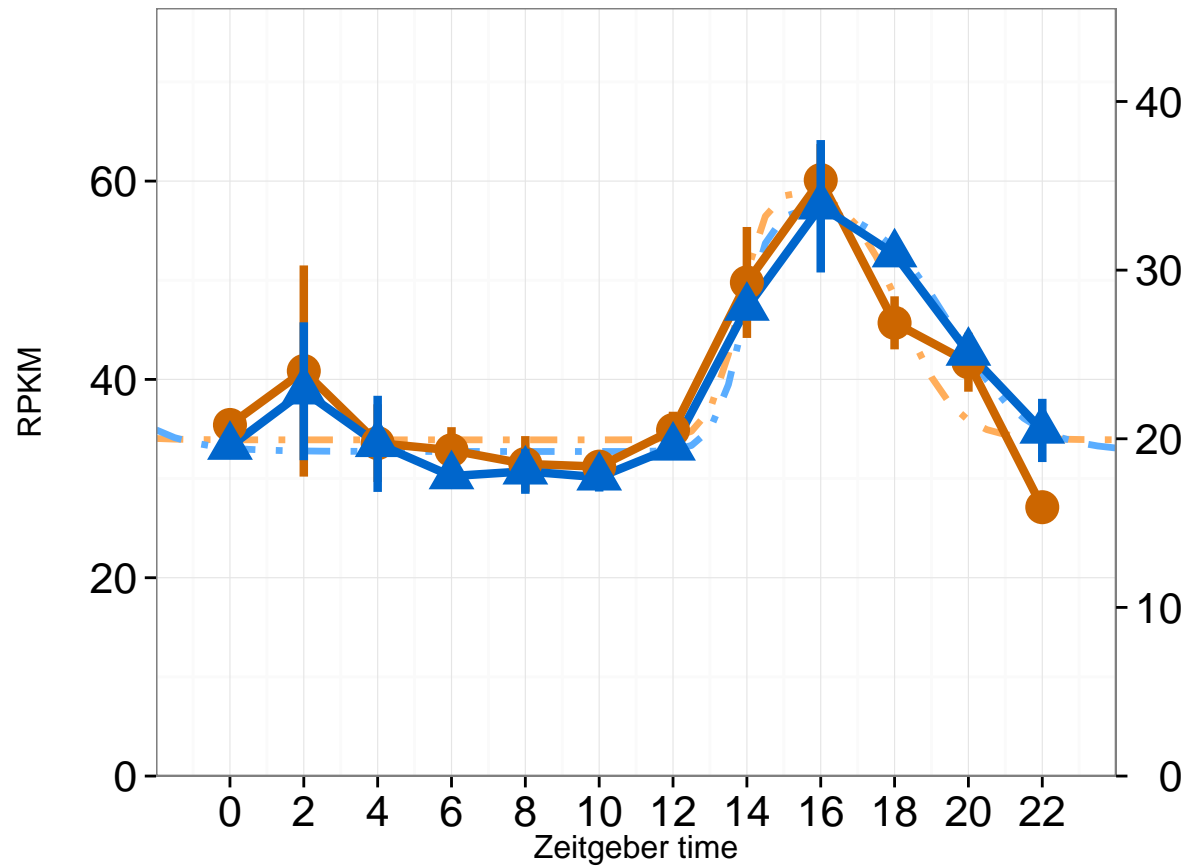

## Ahsa2

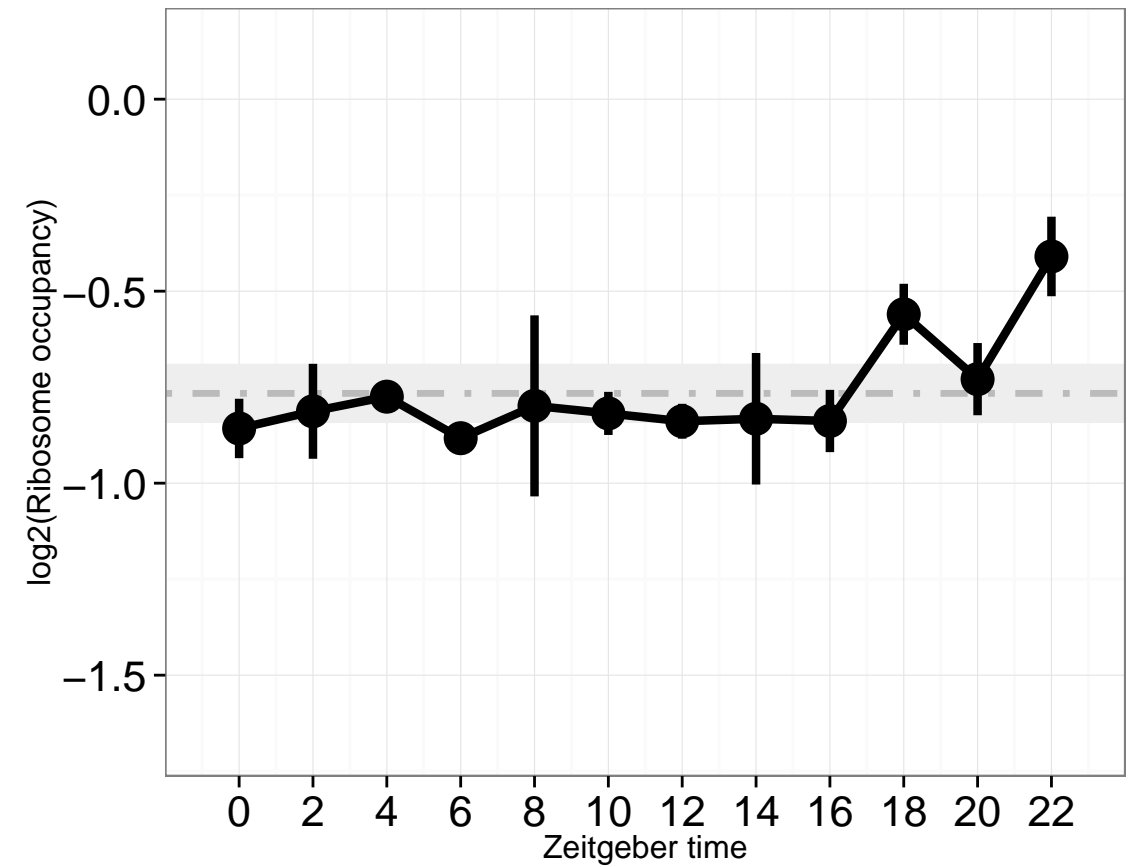

Supplement: Supplementary file 7 — Expression plots for kidney and liver for the 178 common rhythmic genes of Fig. 3c. (ZIP 3338.28 kb) [file 13059_2017_1222_MOESM7_ESM.zip › set_D_shared(178)/Ahsa2_liver_set_D.pdf]

## Ak4

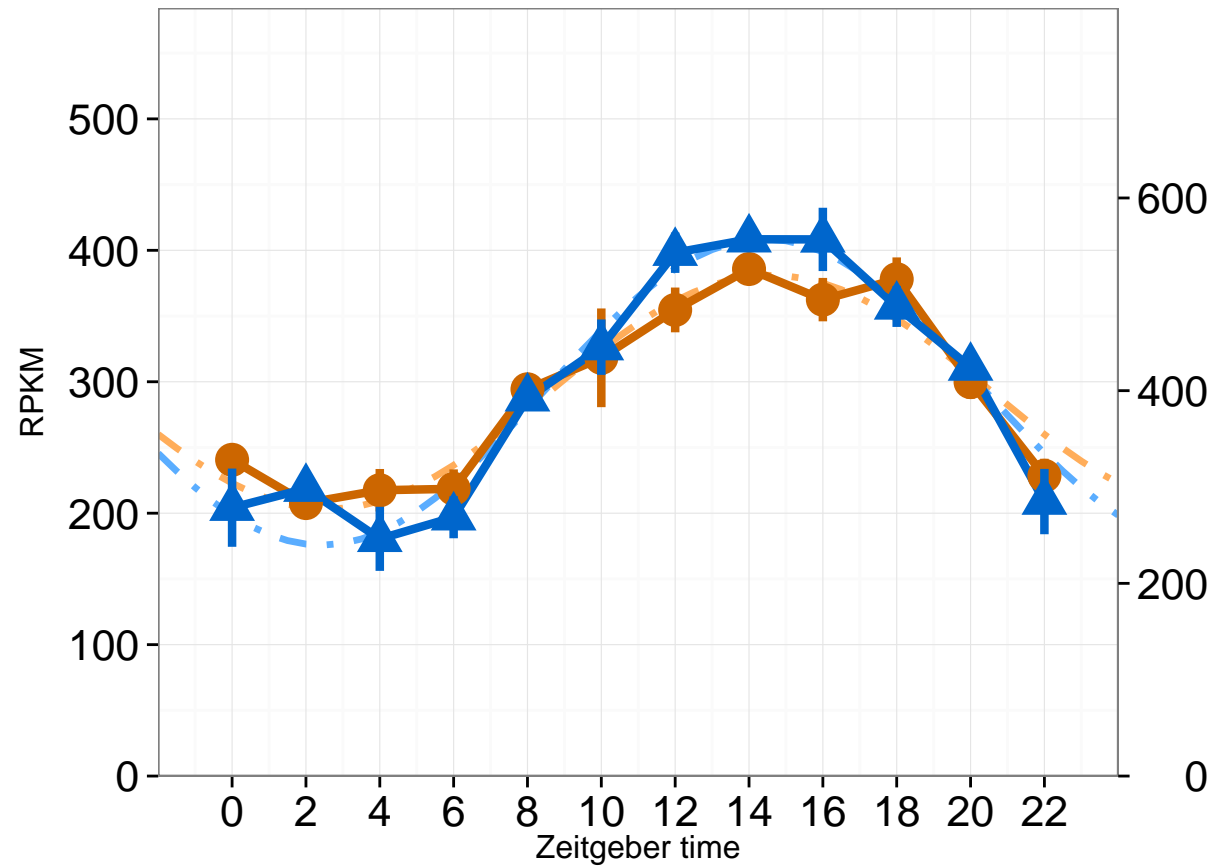

## Ak4

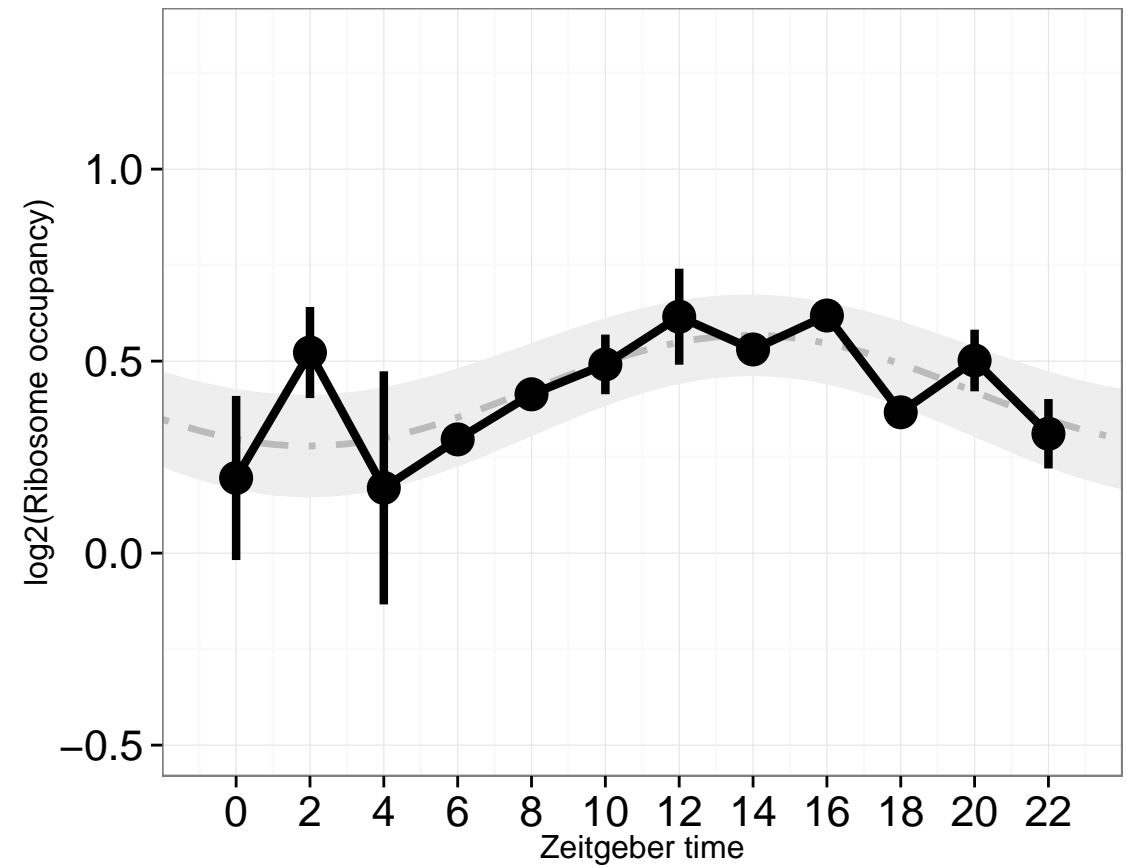

Supplement: Supplementary file 7 — Expression plots for kidney and liver for the 178 common rhythmic genes of Fig. 3c. (ZIP 3338.28 kb) [file 13059_2017_1222_MOESM7_ESM.zip › set_D_shared(178)/Ak4_kidney_set_D.pdf]

## Ak4

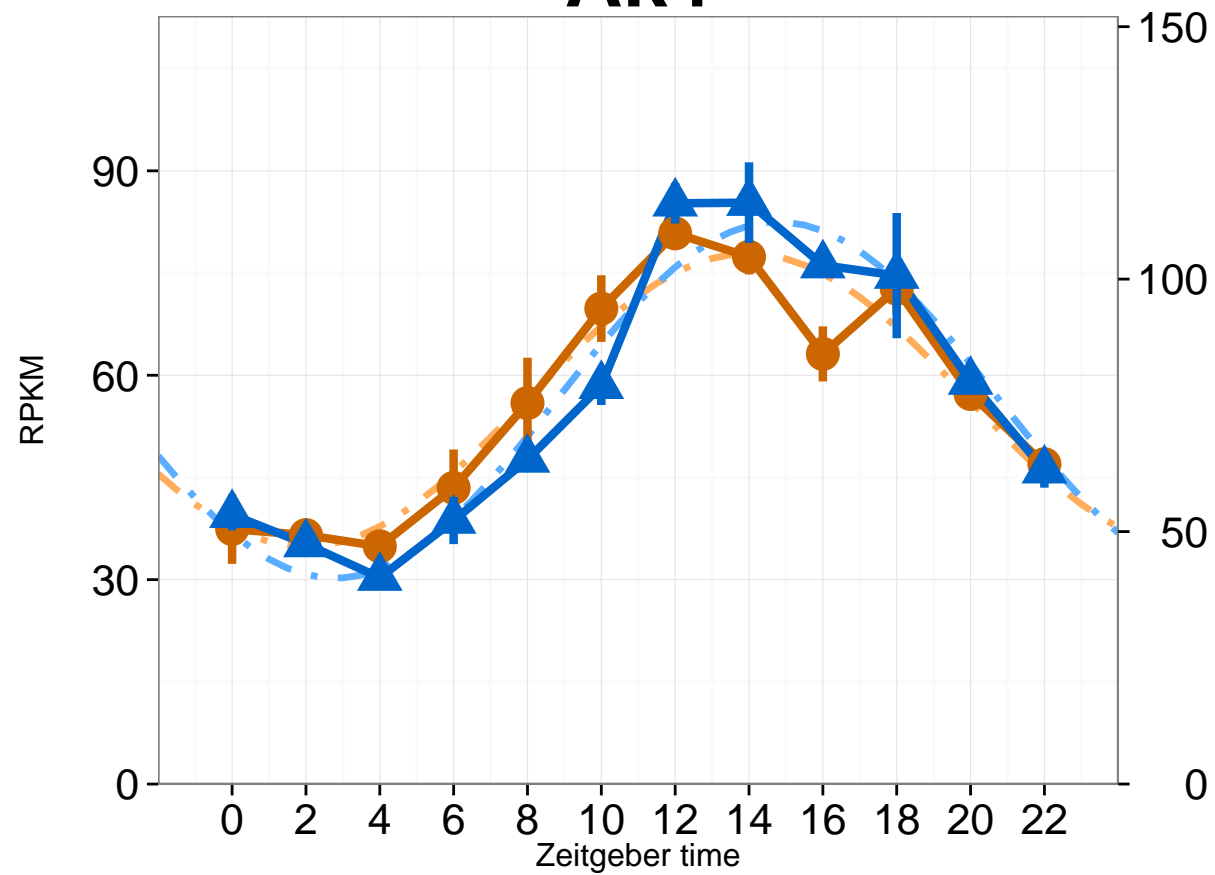

## Ak4

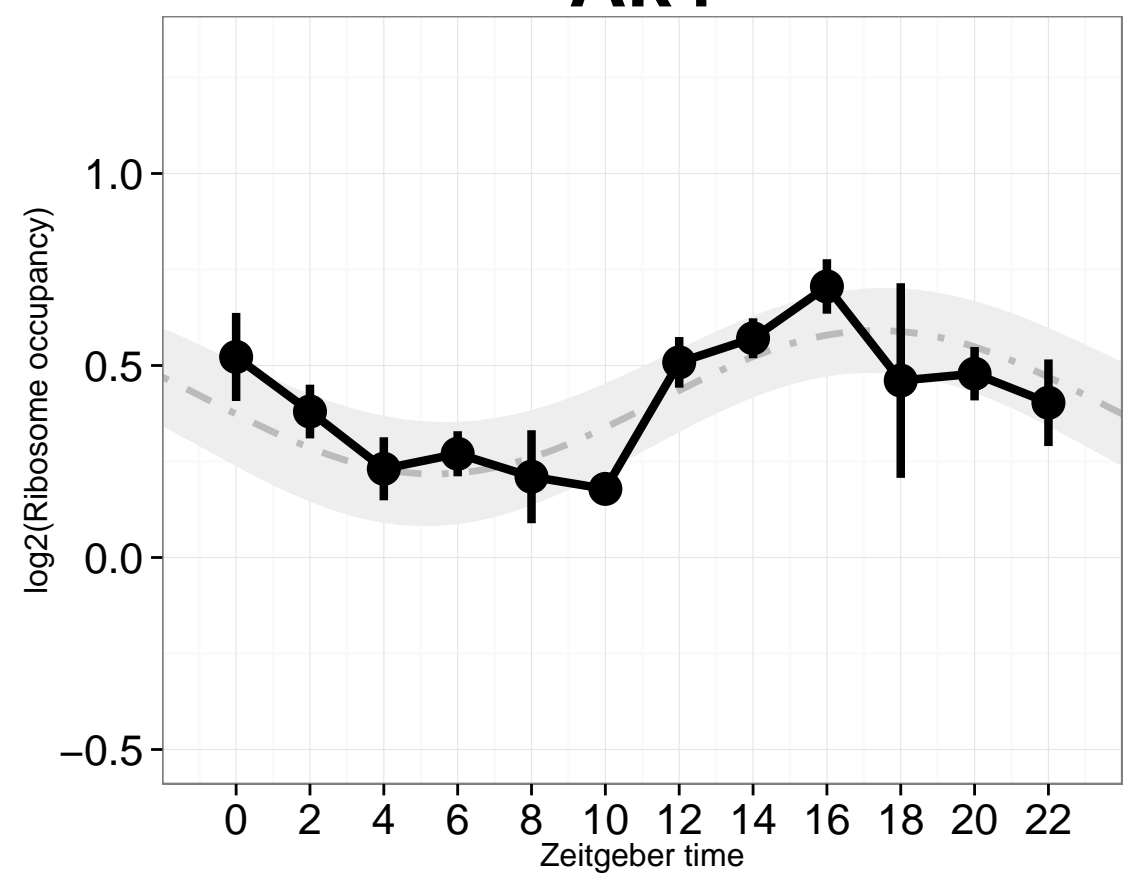

Supplement: Supplementary file 7 — Expression plots for kidney and liver for the 178 common rhythmic genes of Fig. 3c. (ZIP 3338.28 kb) [file 13059_2017_1222_MOESM7_ESM.zip › set_D_shared(178)/Ak4_liver_set_D.pdf]

# Akr1c19

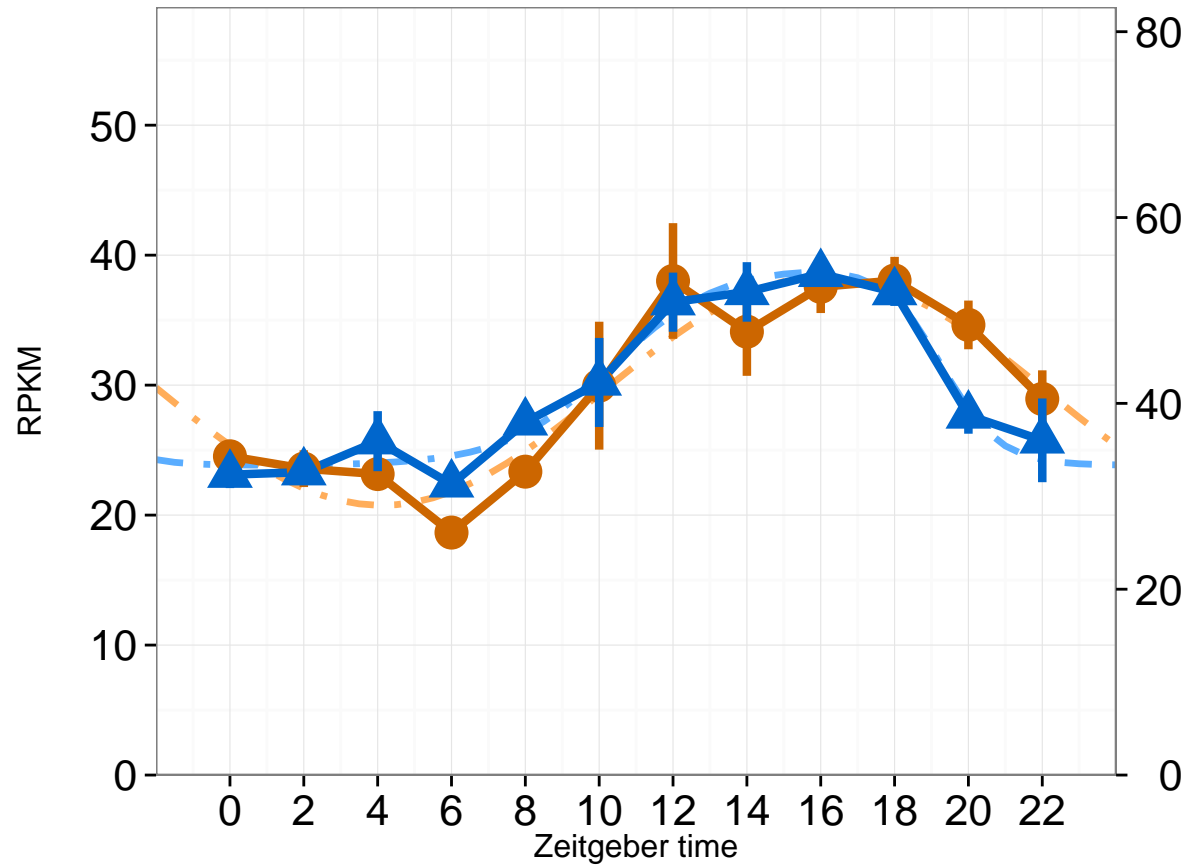

# Akr1c19

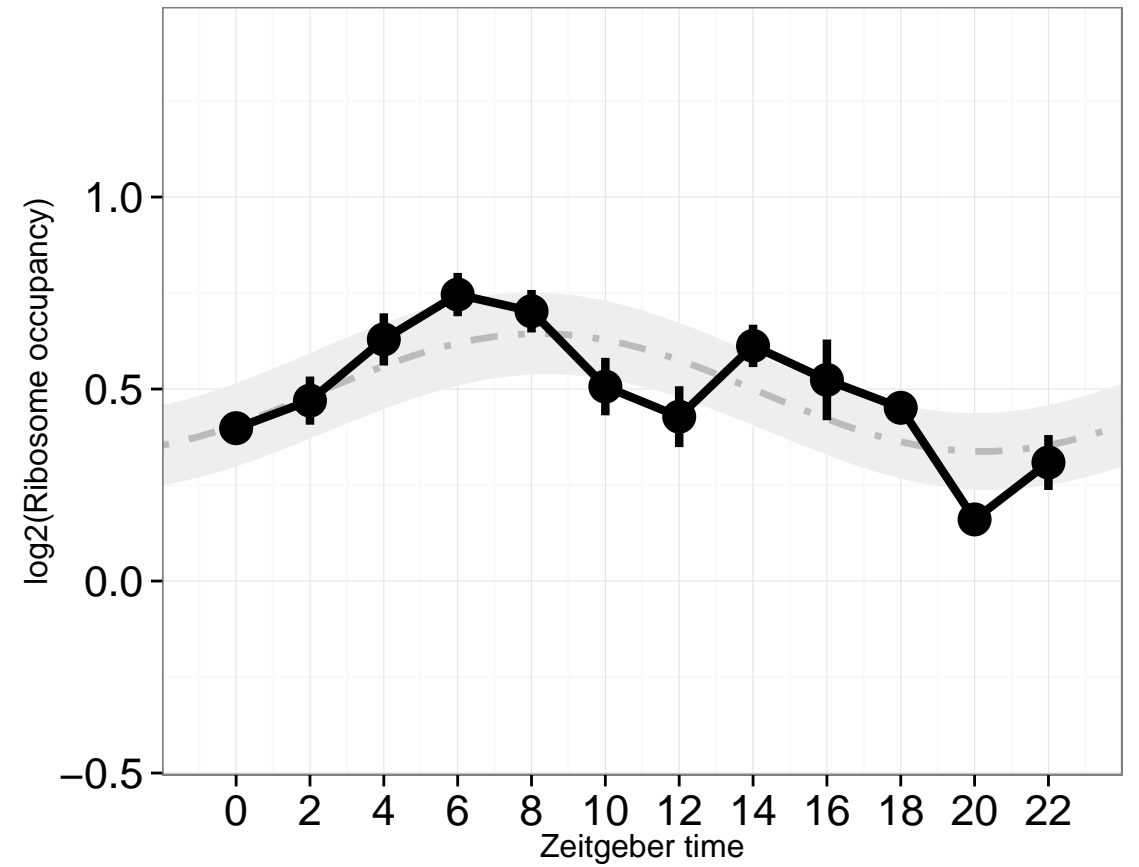

Supplement: Supplementary file 7 — Expression plots for kidney and liver for the 178 common rhythmic genes of Fig. 3c. (ZIP 3338.28 kb) [file 13059_2017_1222_MOESM7_ESM.zip › set_D_shared(178)/Akr1c19_kidney_set_D.pdf]

# Akr1c19

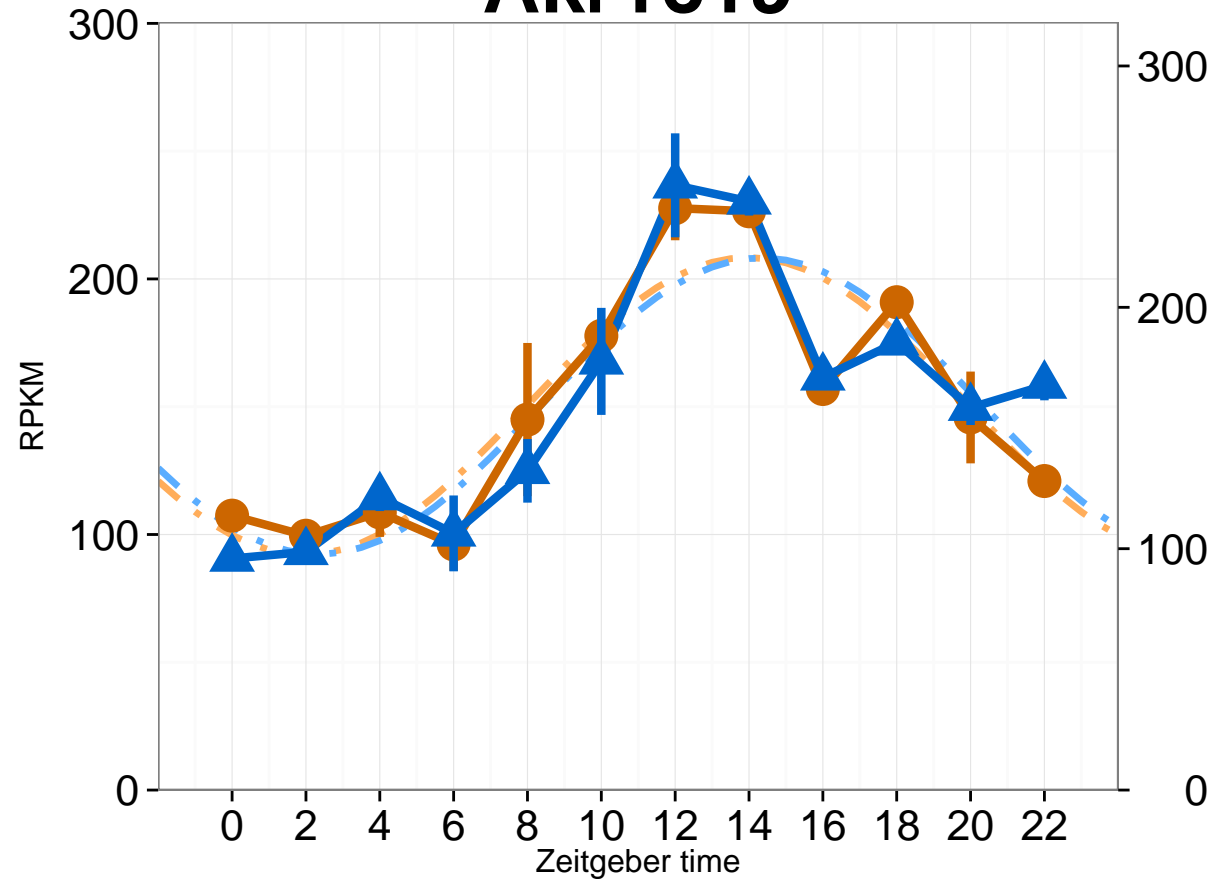

# Akr1c19

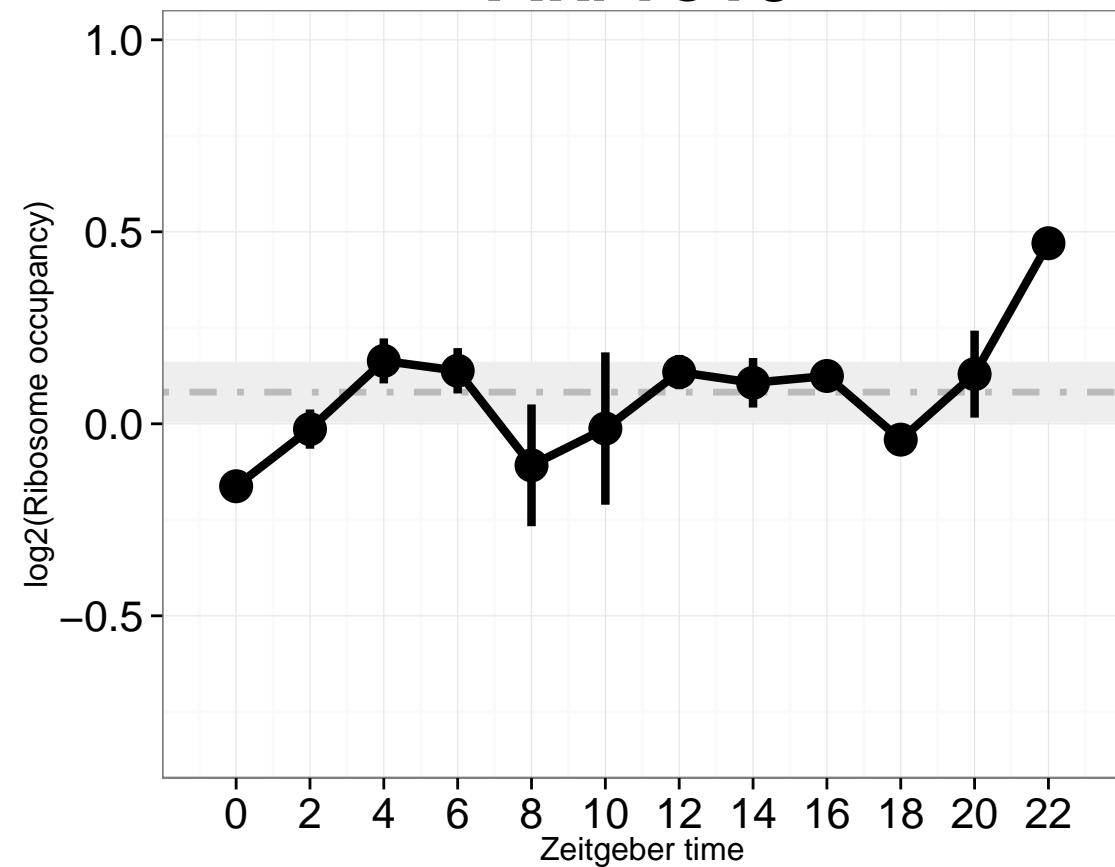

Supplement: Supplementary file 7 — Expression plots for kidney and liver for the 178 common rhythmic genes of Fig. 3c. (ZIP 3338.28 kb) [file 13059_2017_1222_MOESM7_ESM.zip › set_D_shared(178)/Akr1c19_liver_set_D.pdf]

## Alas1

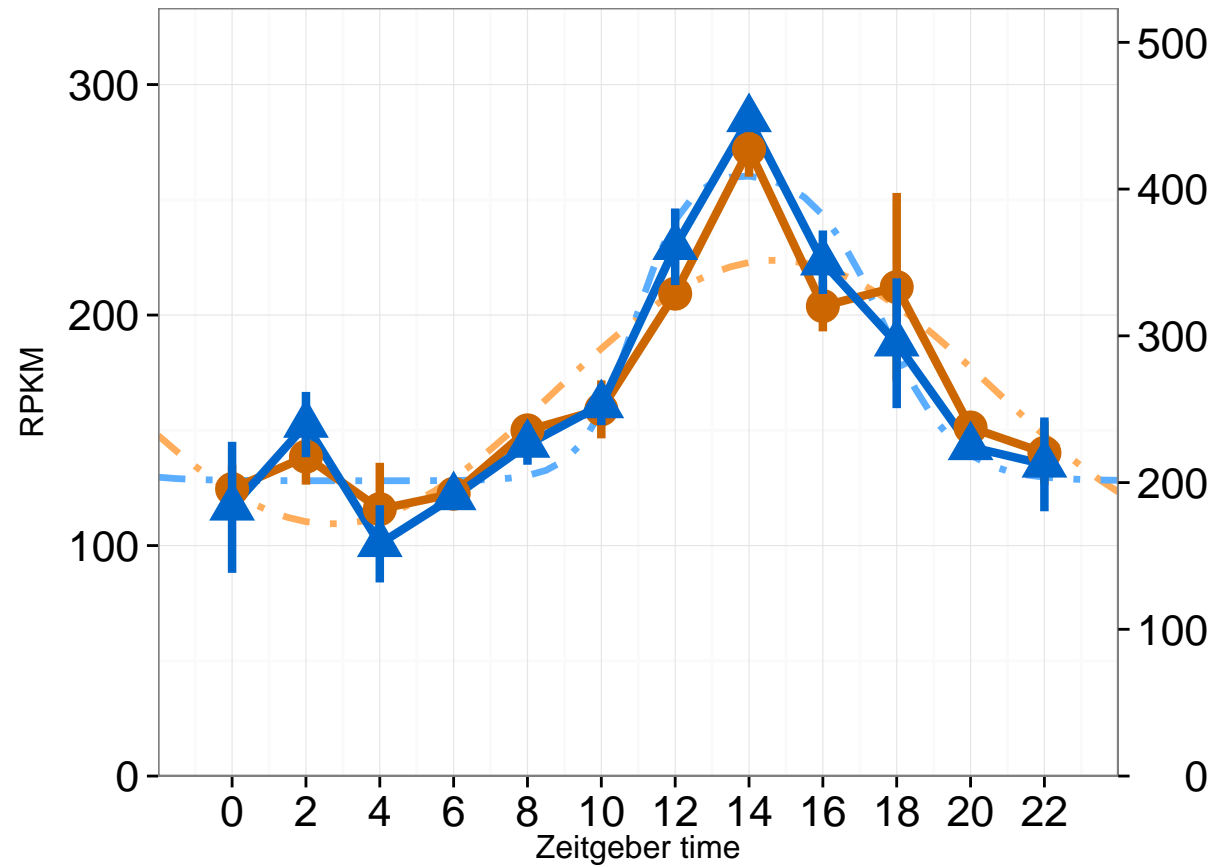

## Alas1

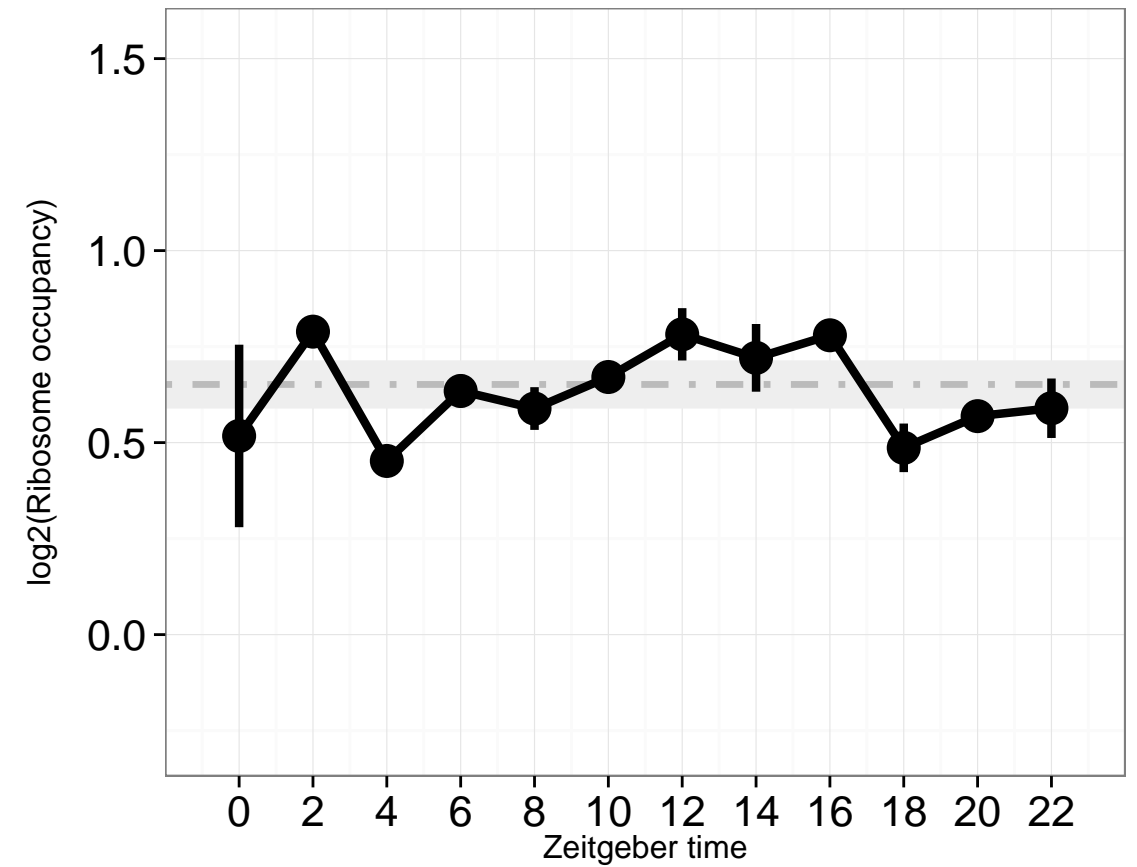

Supplement: Supplementary file 7 — Expression plots for kidney and liver for the 178 common rhythmic genes of Fig. 3c. (ZIP 3338.28 kb) [file 13059_2017_1222_MOESM7_ESM.zip › set_D_shared(178)/Alas1_kidney_set_D.pdf]

# Alas1

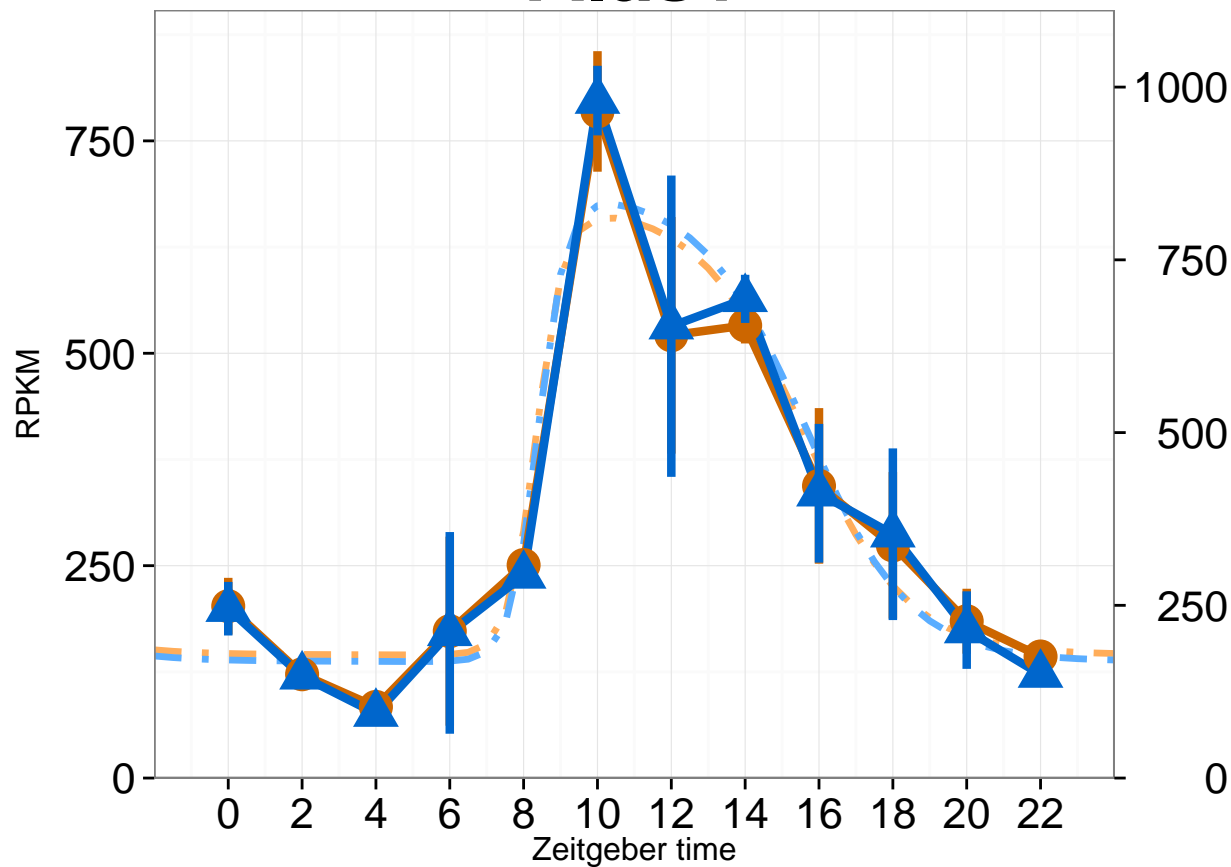

# Alas1

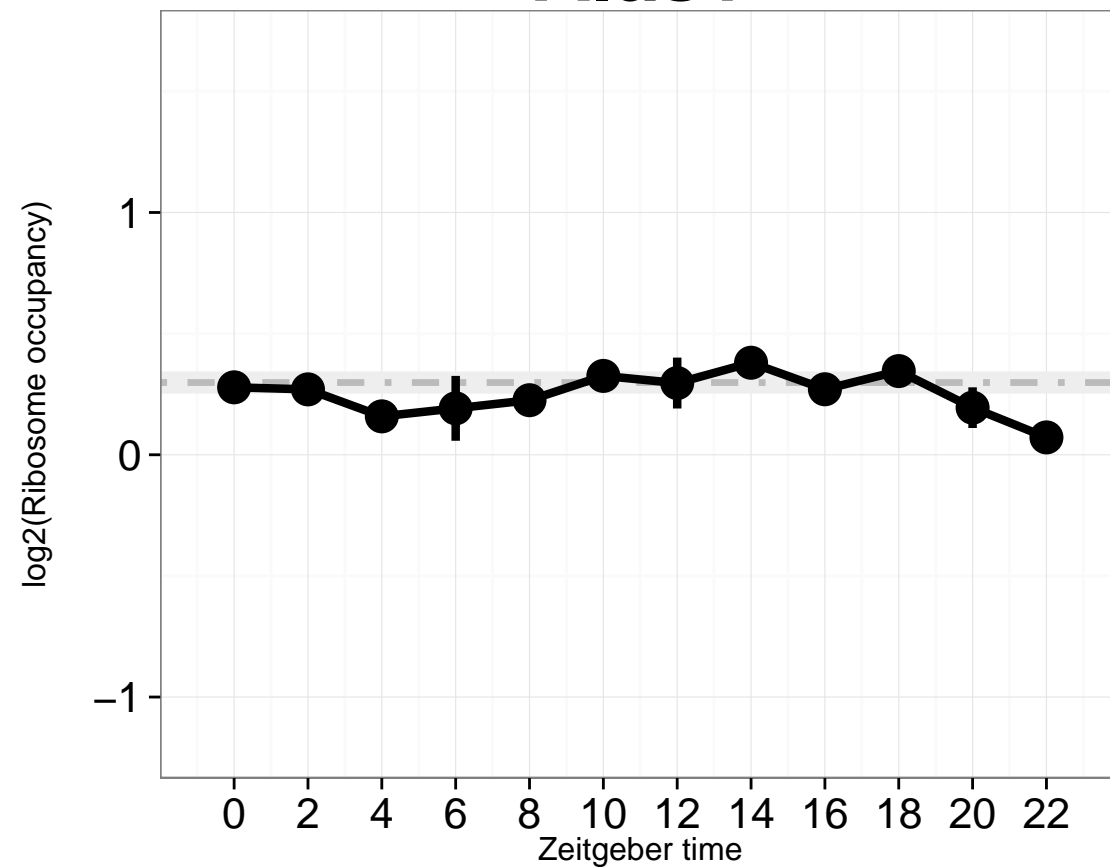

Supplement: Supplementary file 7 — Expression plots for kidney and liver for the 178 common rhythmic genes of Fig. 3c. (ZIP 3338.28 kb) [file 13059_2017_1222_MOESM7_ESM.zip › set_D_shared(178)/Alas1_liver_set_D.pdf]

## Amd1

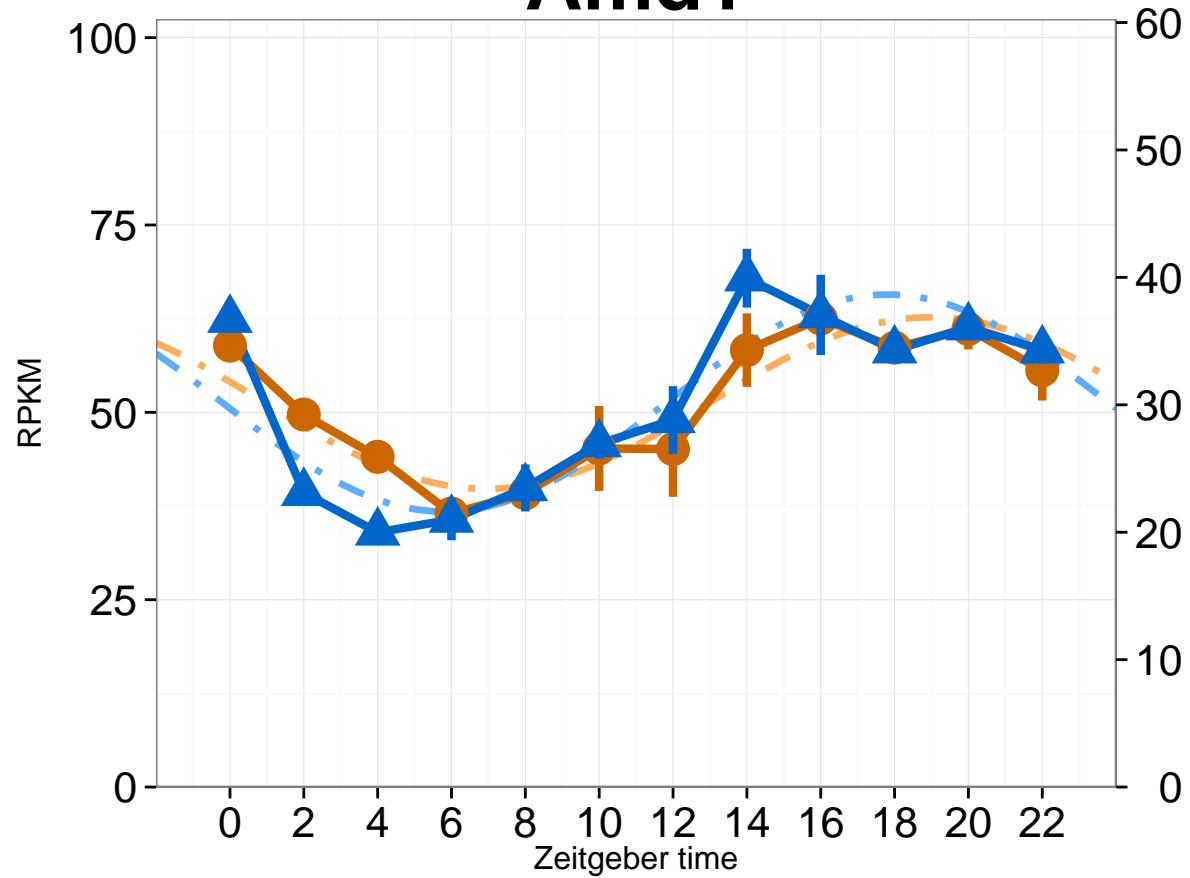

## Amd1

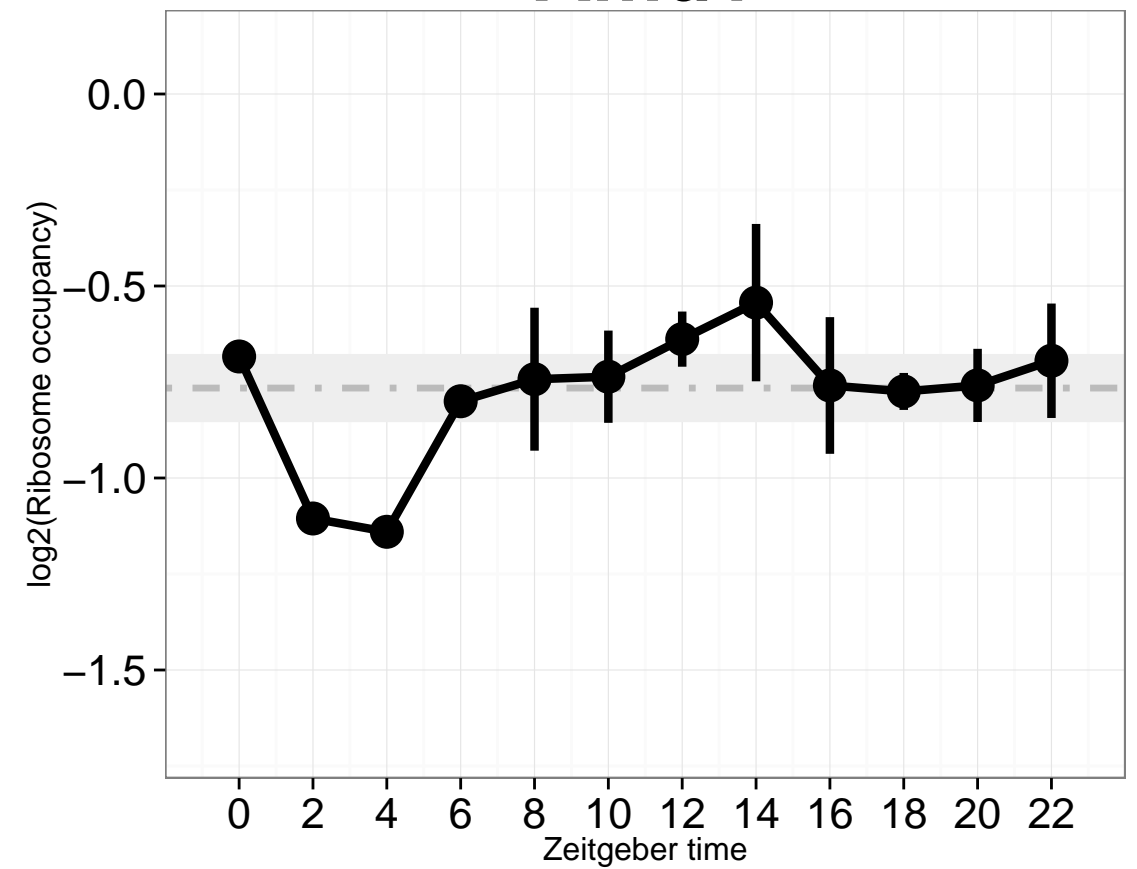

Supplement: Supplementary file 7 — Expression plots for kidney and liver for the 178 common rhythmic genes of Fig. 3c. (ZIP 3338.28 kb) [file 13059_2017_1222_MOESM7_ESM.zip › set_D_shared(178)/Amd1_kidney_set_D.pdf]

# Amd1

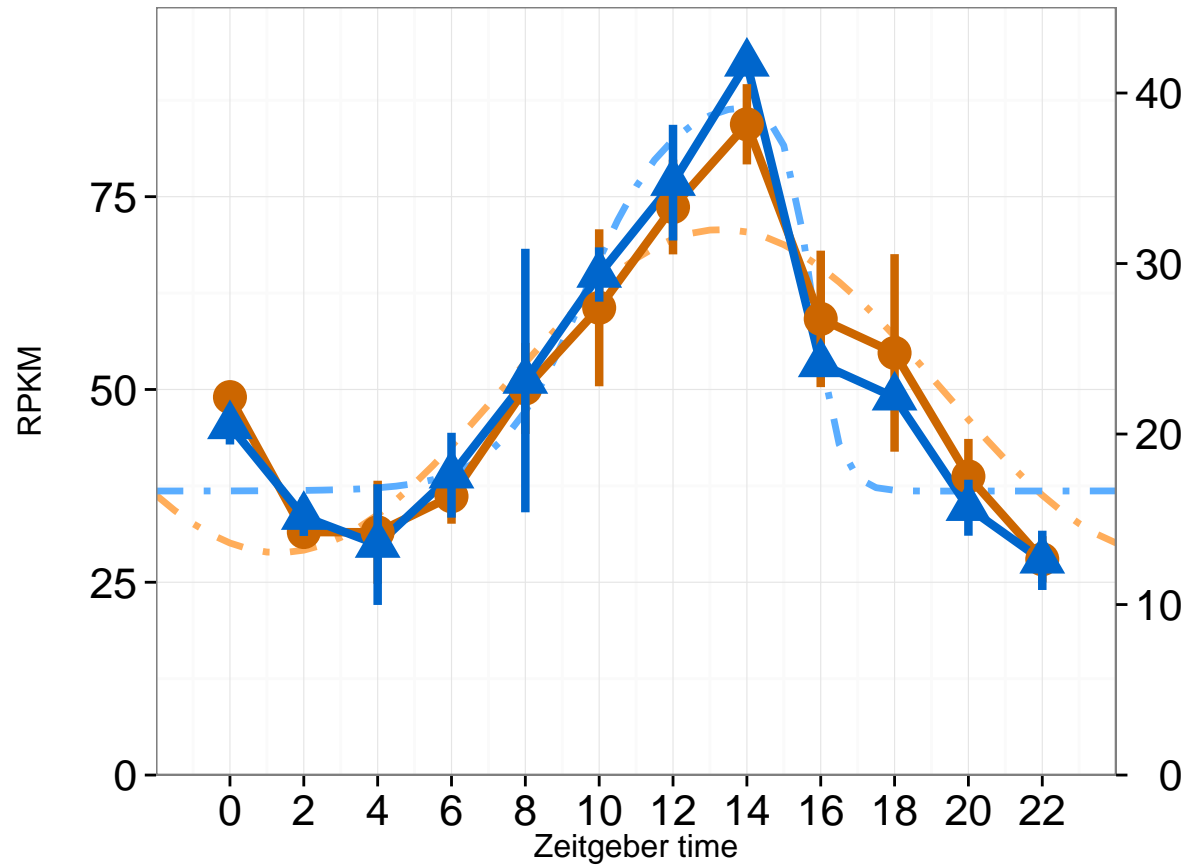

# Amd1

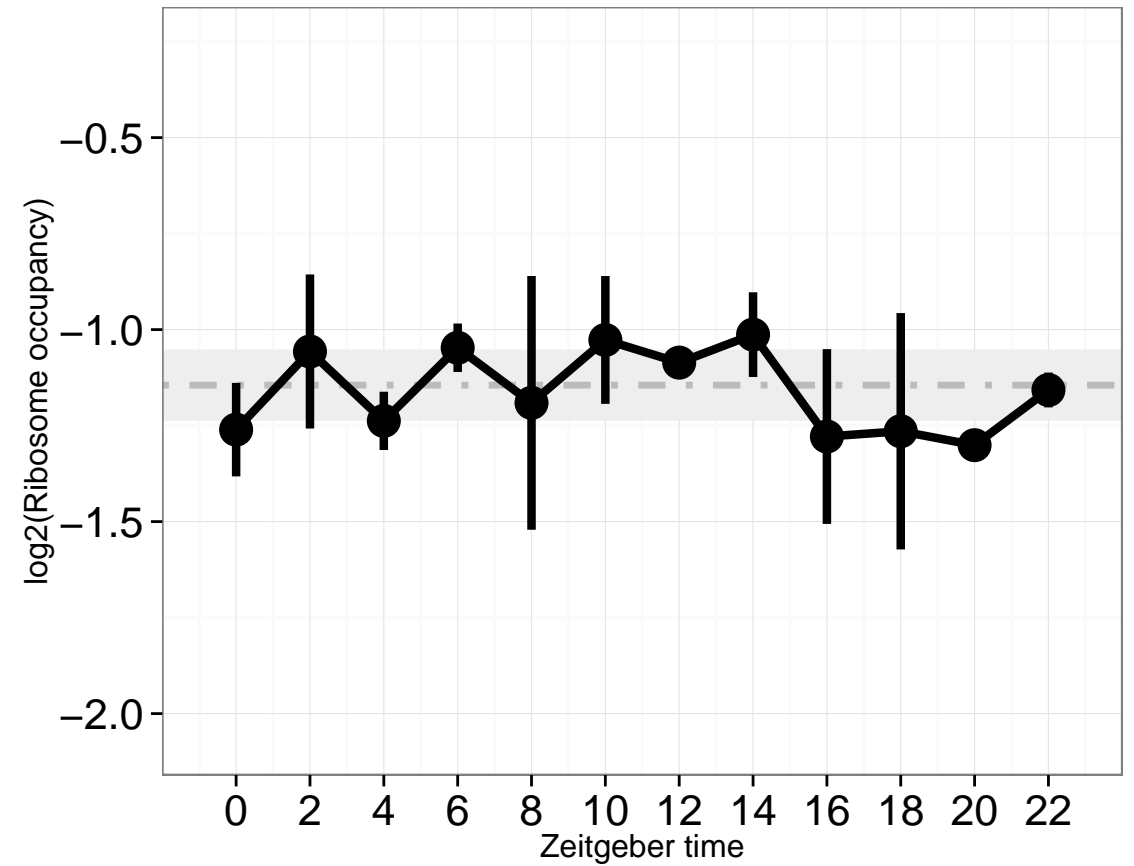

Supplement: Supplementary file 7 — Expression plots for kidney and liver for the 178 common rhythmic genes of Fig. 3c. (ZIP 3338.28 kb) [file 13059_2017_1222_MOESM7_ESM.zip › set_D_shared(178)/Amd1_liver_set_D.pdf]

## Amdhd1

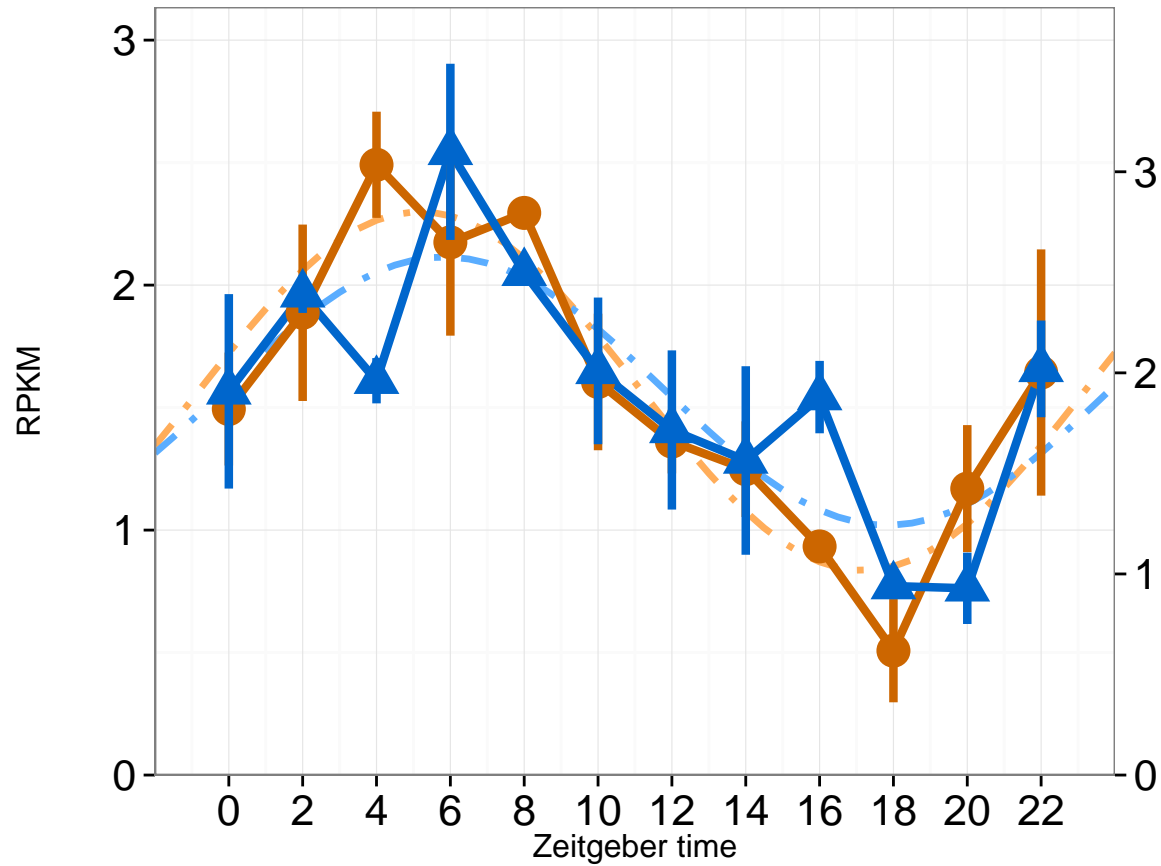

## Amdhd1

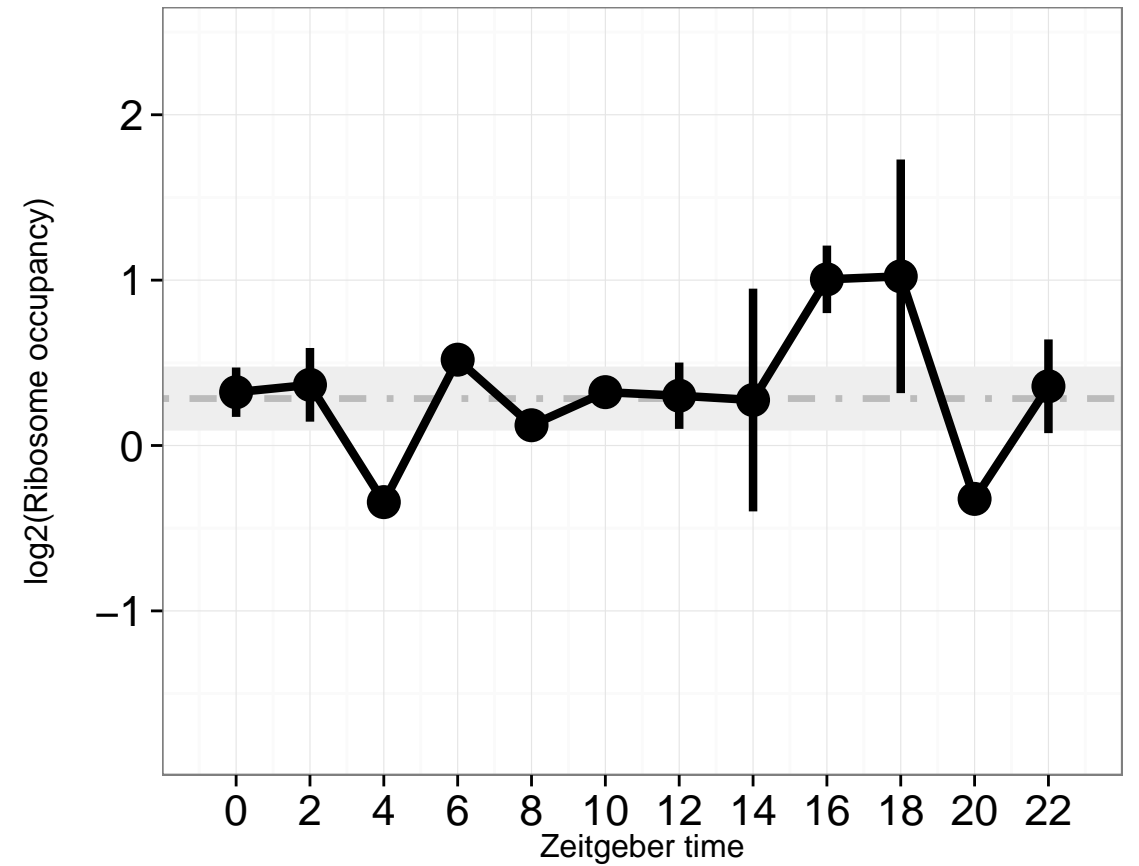

Supplement: Supplementary file 7 — Expression plots for kidney and liver for the 178 common rhythmic genes of Fig. 3c. (ZIP 3338.28 kb) [file 13059_2017_1222_MOESM7_ESM.zip › set_D_shared(178)/Amdhd1_kidney_set_D.pdf]

# Amdhd1

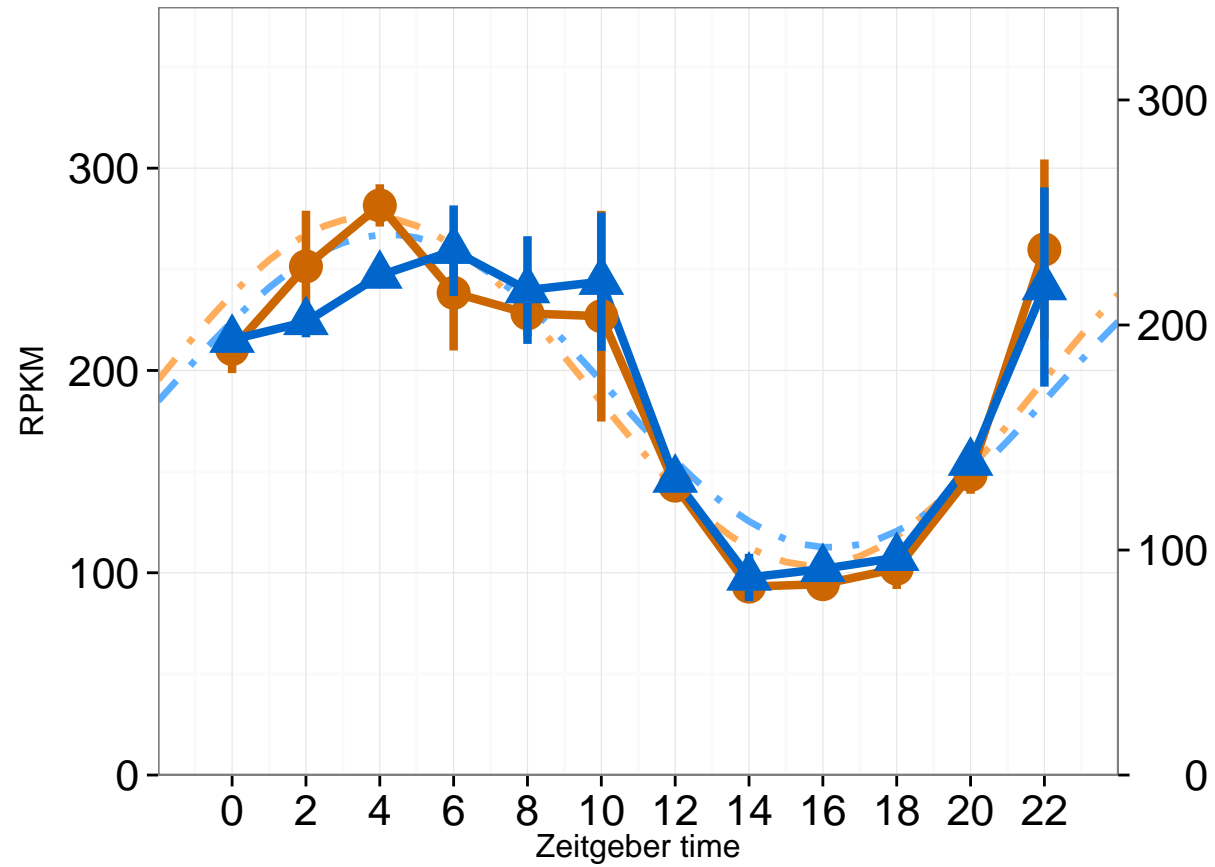

# Amdhd1

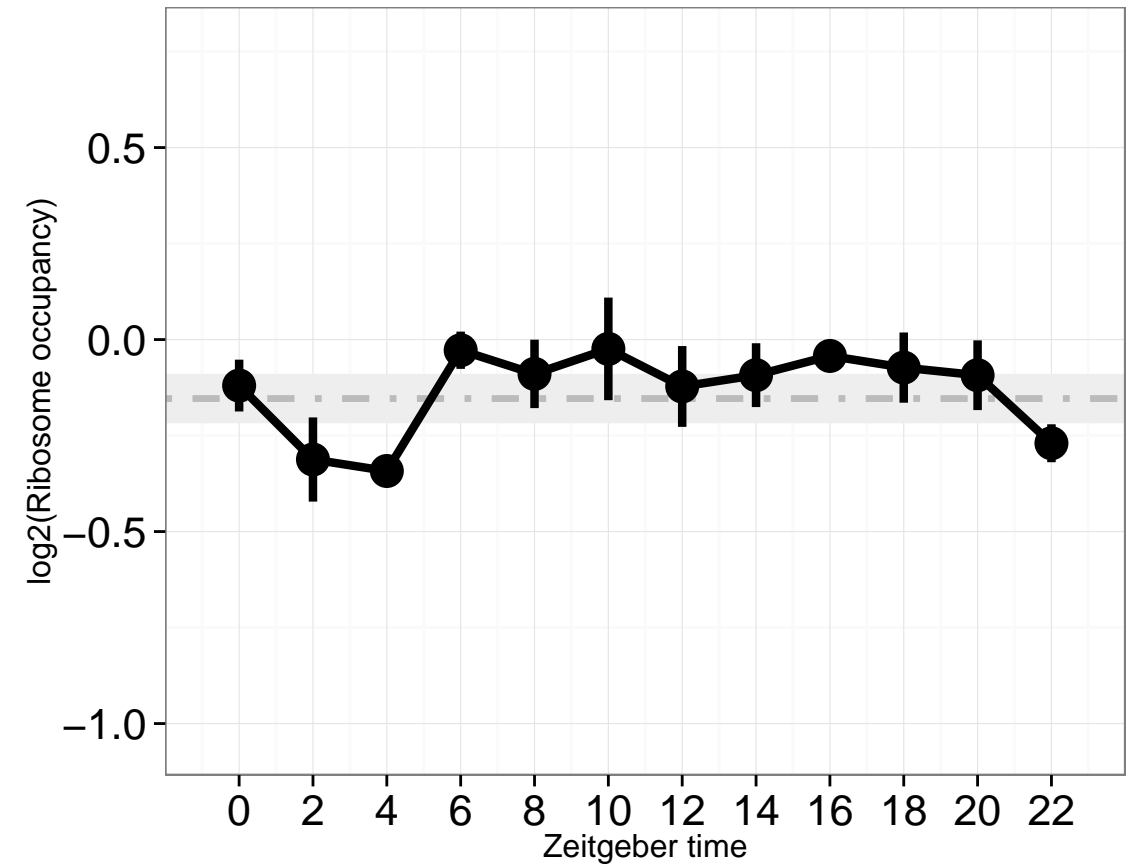

Supplement: Supplementary file 7 — Expression plots for kidney and liver for the 178 common rhythmic genes of Fig. 3c. (ZIP 3338.28 kb) [file 13059_2017_1222_MOESM7_ESM.zip › set_D_shared(178)/Amdhd1_liver_set_D.pdf]

# Aqp8

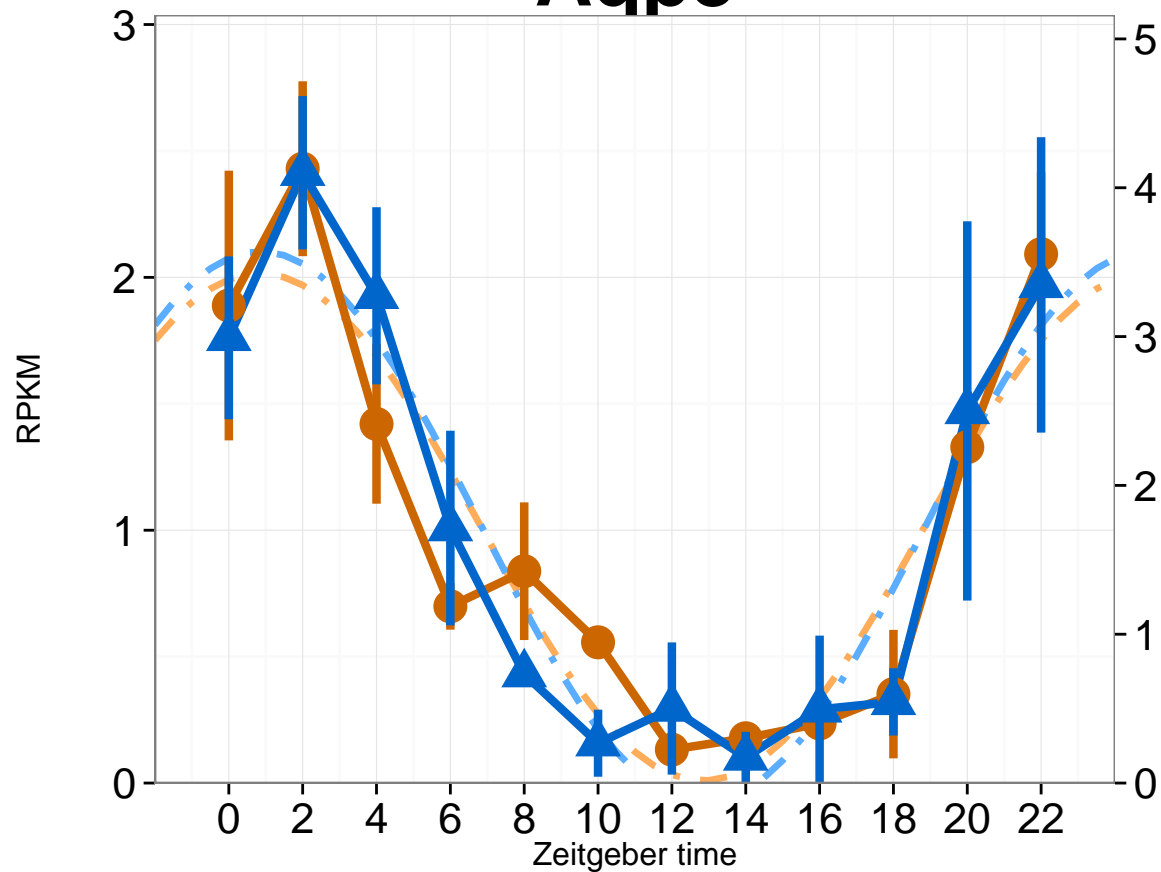

# Aqp8

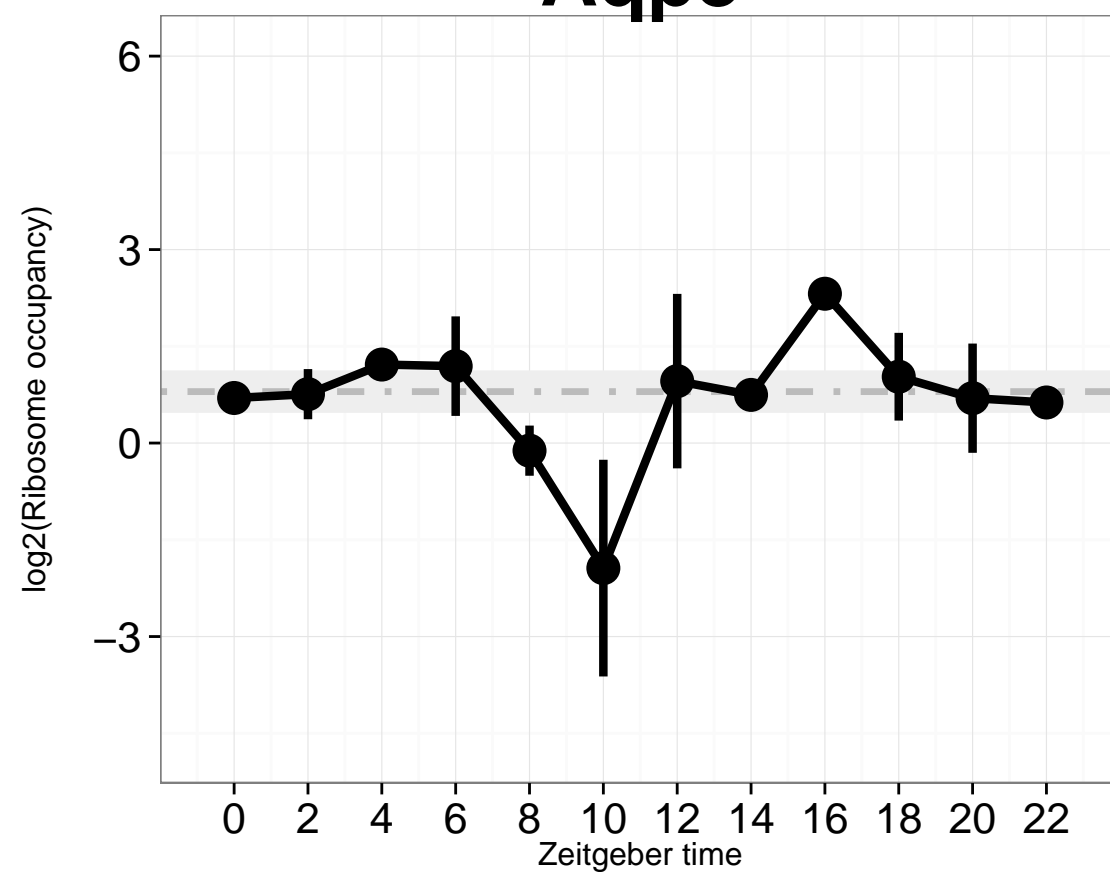

Supplement: Supplementary file 7 — Expression plots for kidney and liver for the 178 common rhythmic genes of Fig. 3c. (ZIP 3338.28 kb) [file 13059_2017_1222_MOESM7_ESM.zip › set_D_shared(178)/Aqp8_kidney_set_D.pdf]

# Aqp8

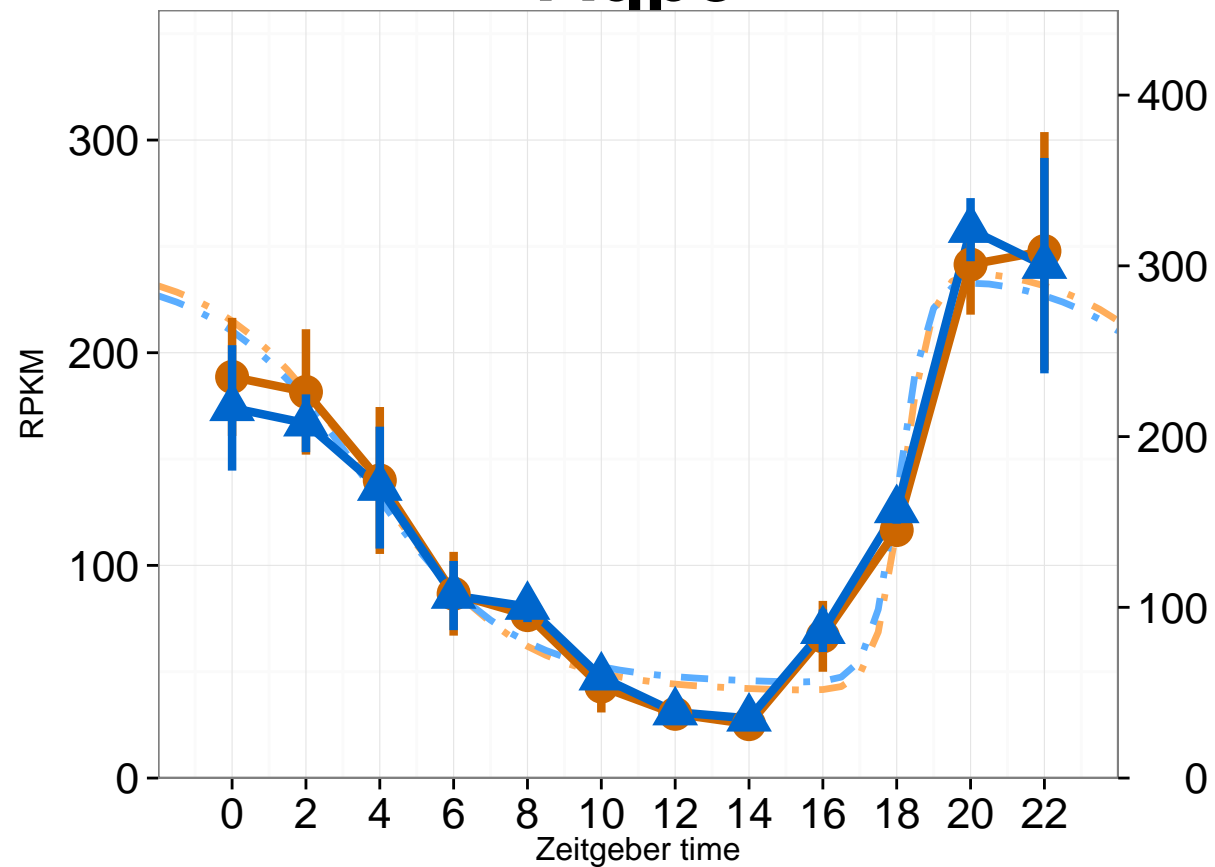

# Aqp8

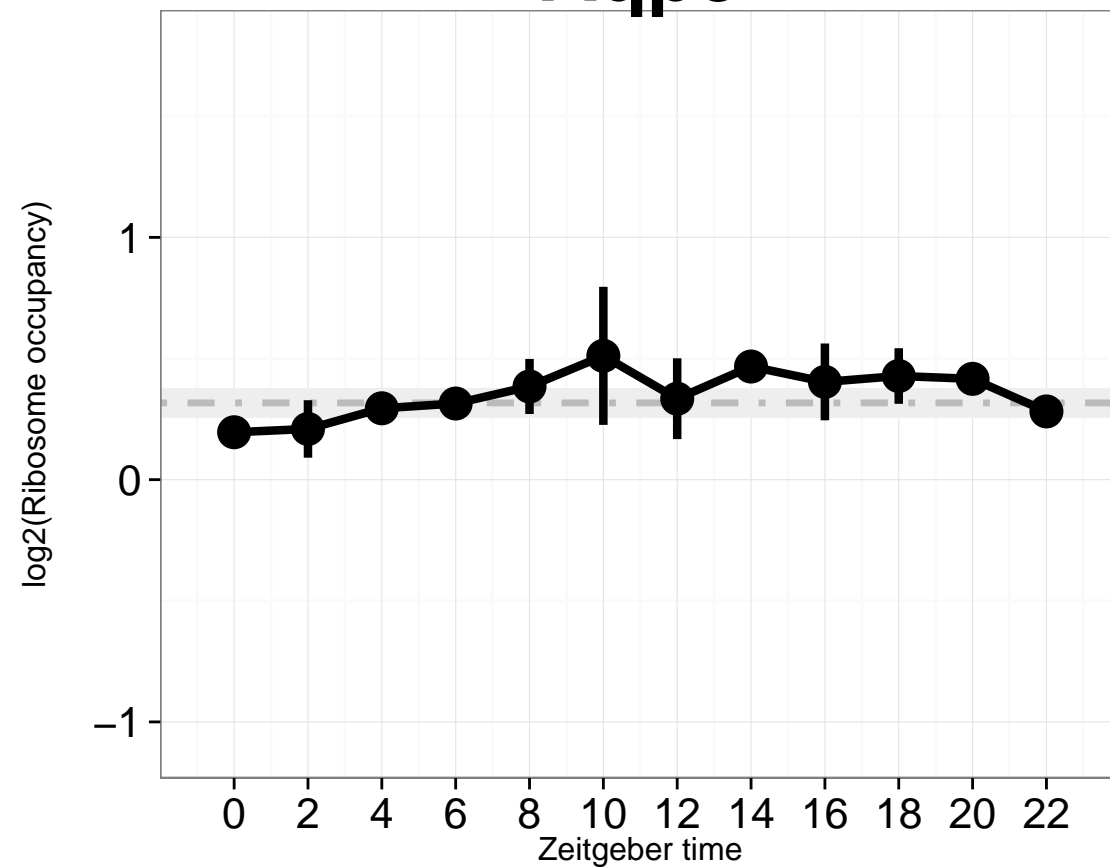

Supplement: Supplementary file 7 — Expression plots for kidney and liver for the 178 common rhythmic genes of Fig. 3c. (ZIP 3338.28 kb) [file 13059_2017_1222_MOESM7_ESM.zip › set_D_shared(178)/Aqp8_liver_set_D.pdf]

## Arhgef10l

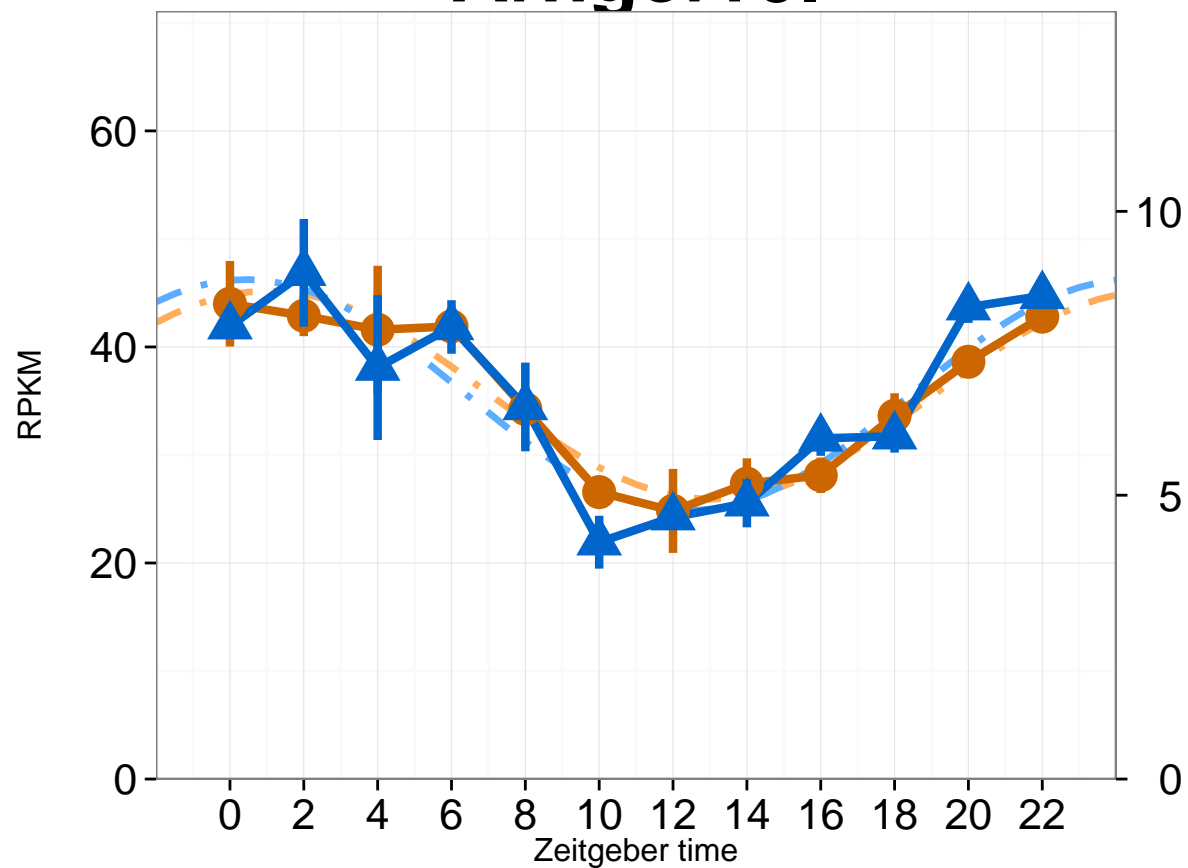

## Arhgef10l

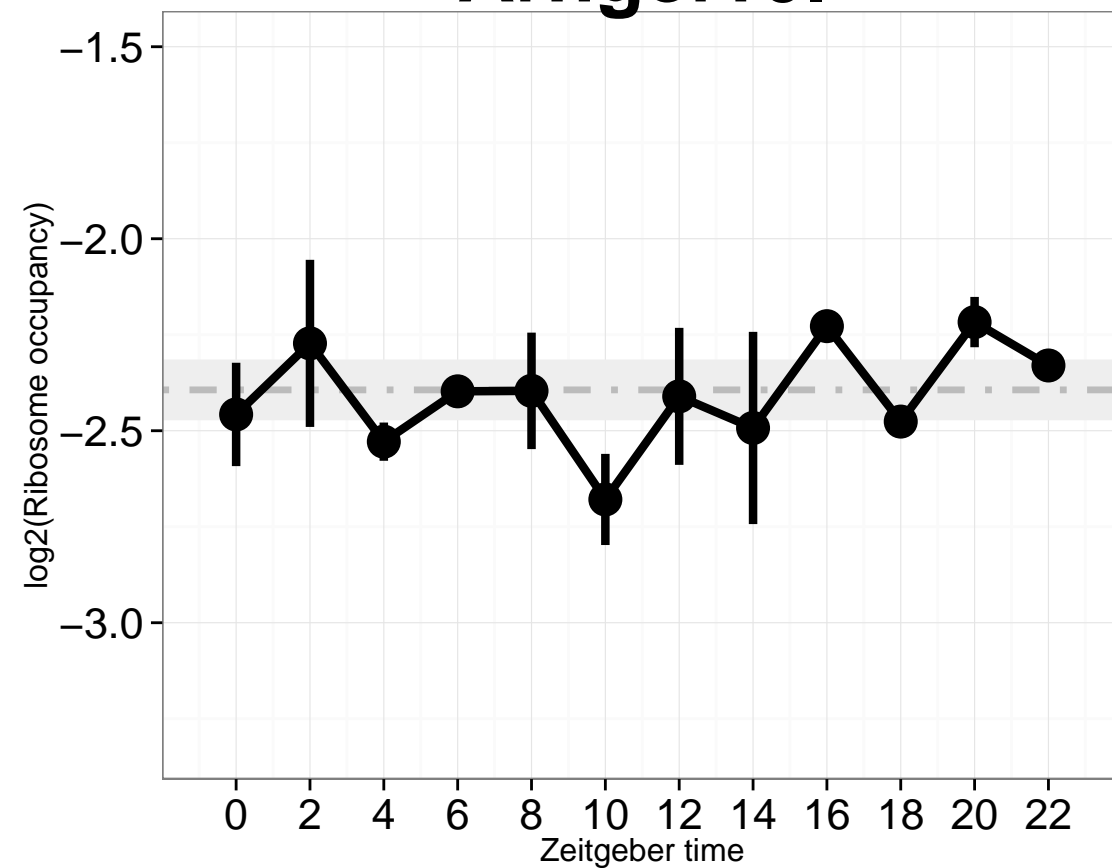

Supplement: Supplementary file 7 — Expression plots for kidney and liver for the 178 common rhythmic genes of Fig. 3c. (ZIP 3338.28 kb) [file 13059_2017_1222_MOESM7_ESM.zip › set_D_shared(178)/Arhgef10l_kidney_set_D.pdf]

# Arhgef10l

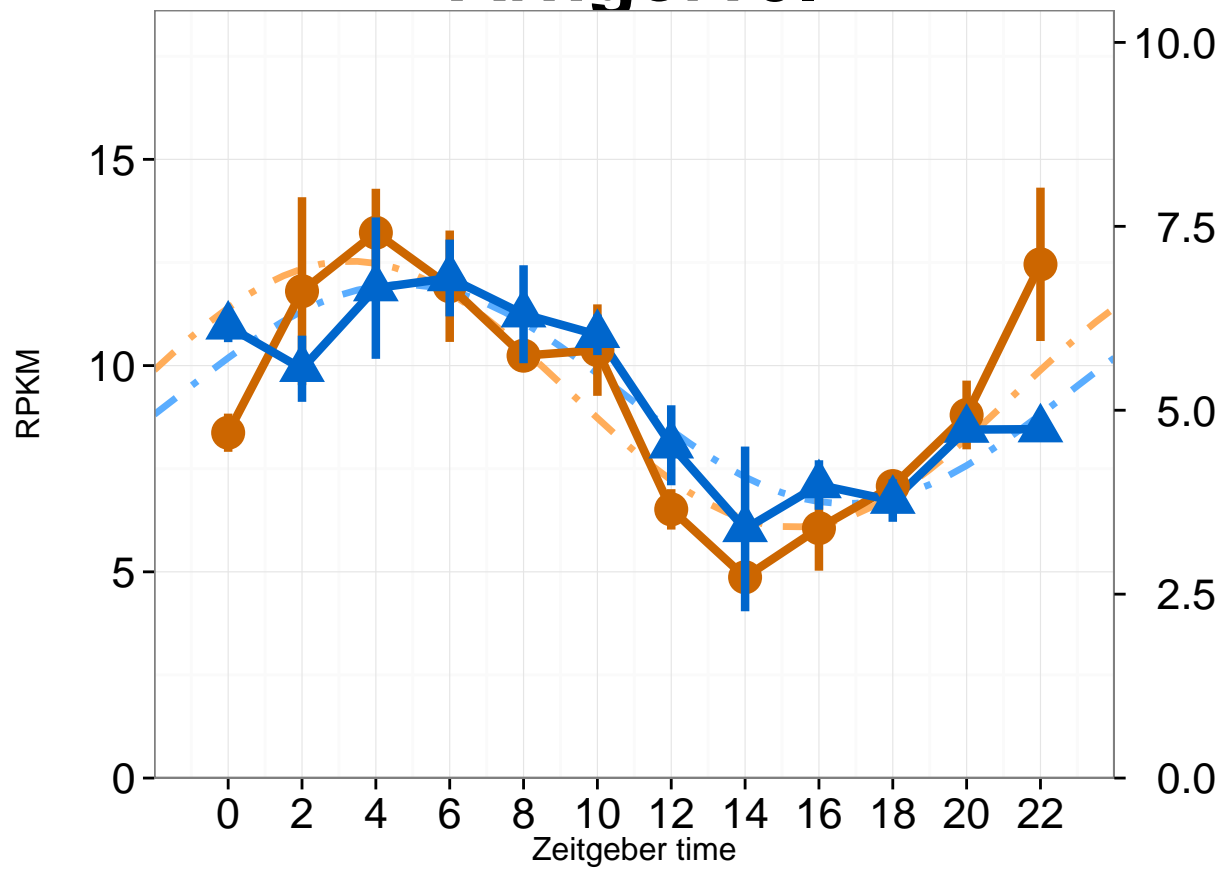

# Arhgef10l

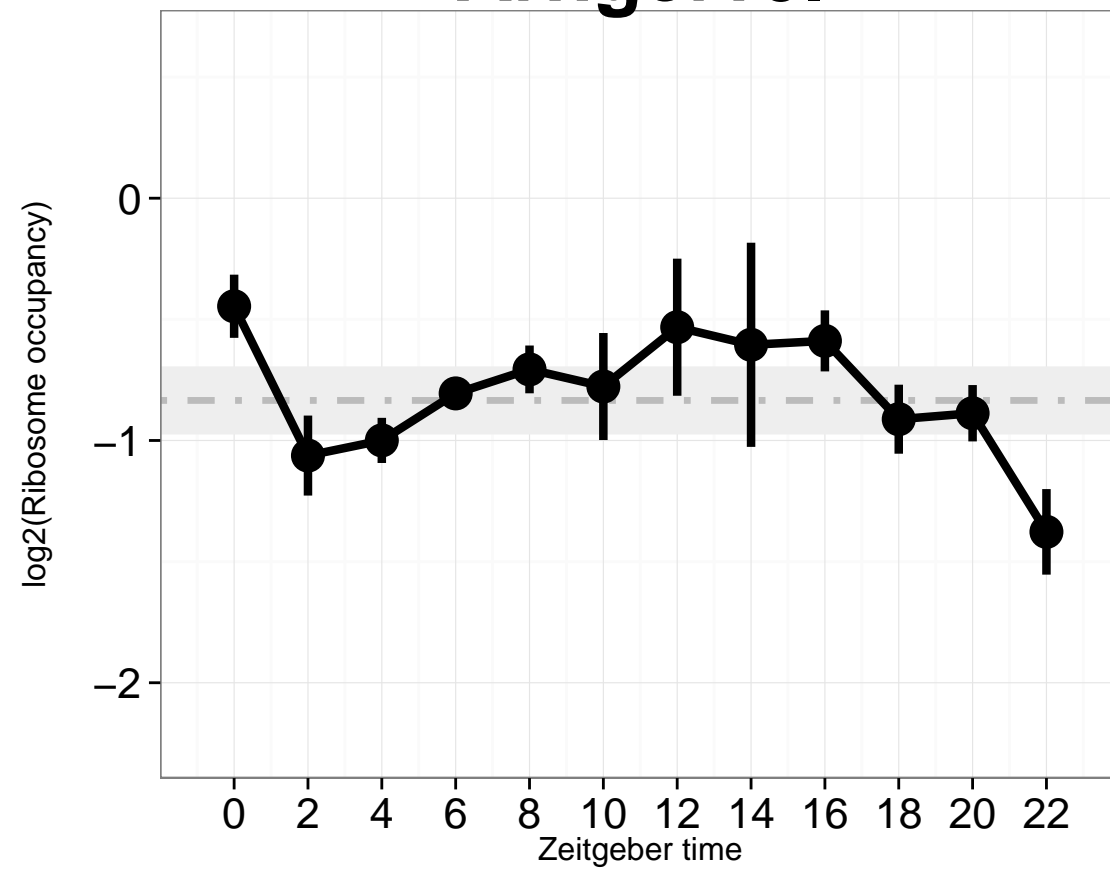

Supplement: Supplementary file 7 — Expression plots for kidney and liver for the 178 common rhythmic genes of Fig. 3c. (ZIP 3338.28 kb) [file 13059_2017_1222_MOESM7_ESM.zip › set_D_shared(178)/Arhgef10l_liver_set_D.pdf]

## Arhgef19

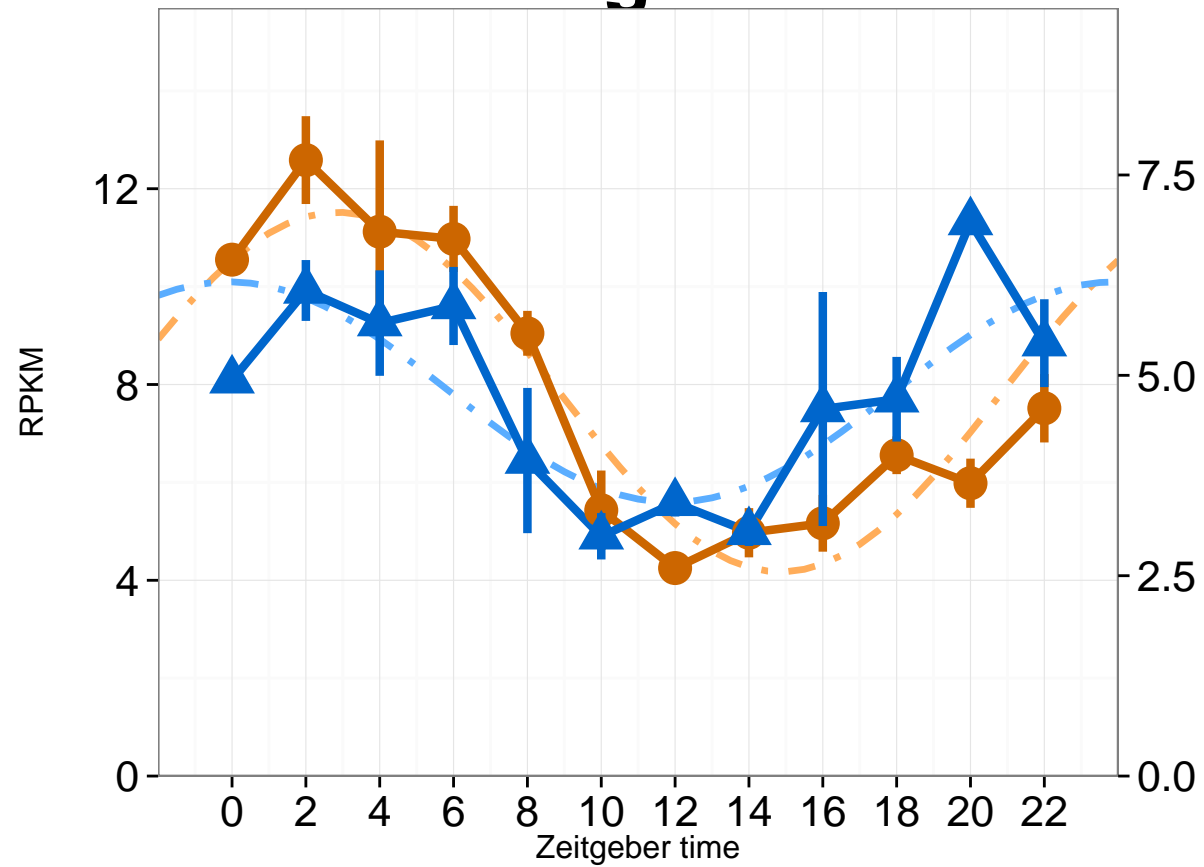

## Arhgef19

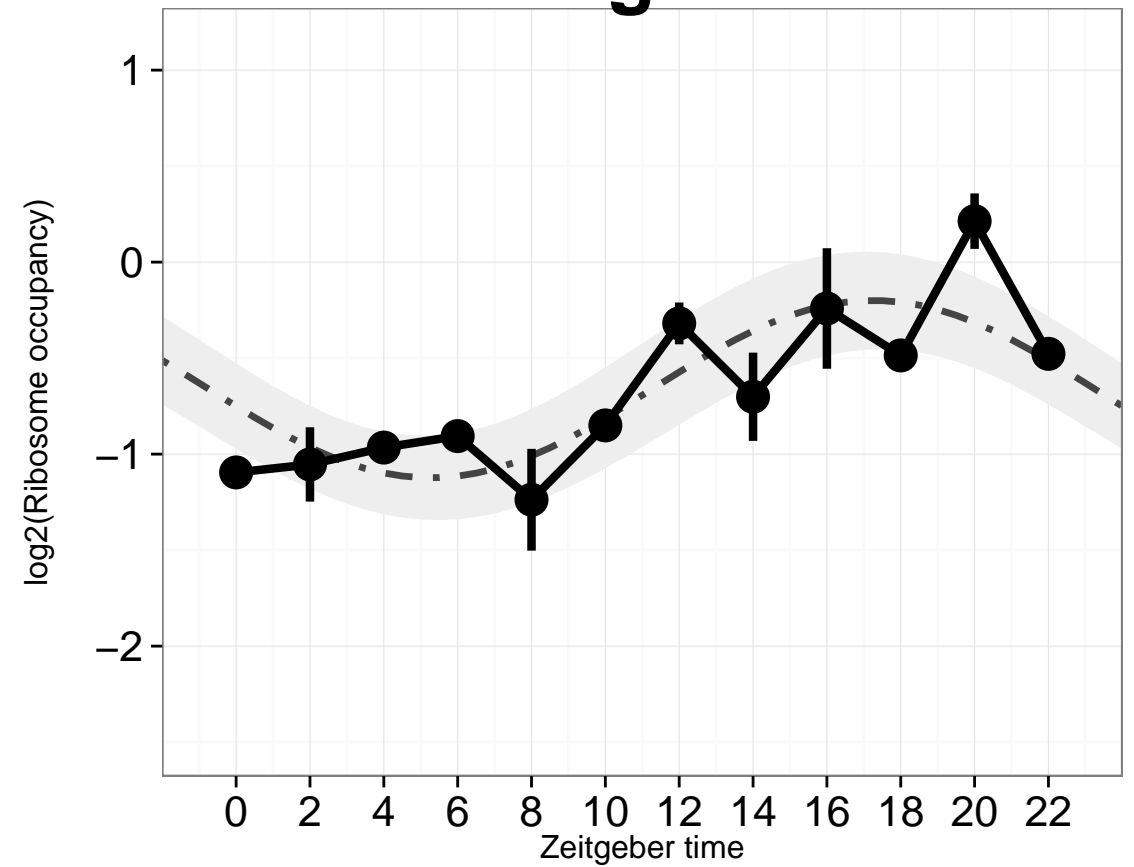

Supplement: Supplementary file 7 — Expression plots for kidney and liver for the 178 common rhythmic genes of Fig. 3c. (ZIP 3338.28 kb) [file 13059_2017_1222_MOESM7_ESM.zip › set_D_shared(178)/Arhgef19_kidney_set_D.pdf]

# Arhgef19

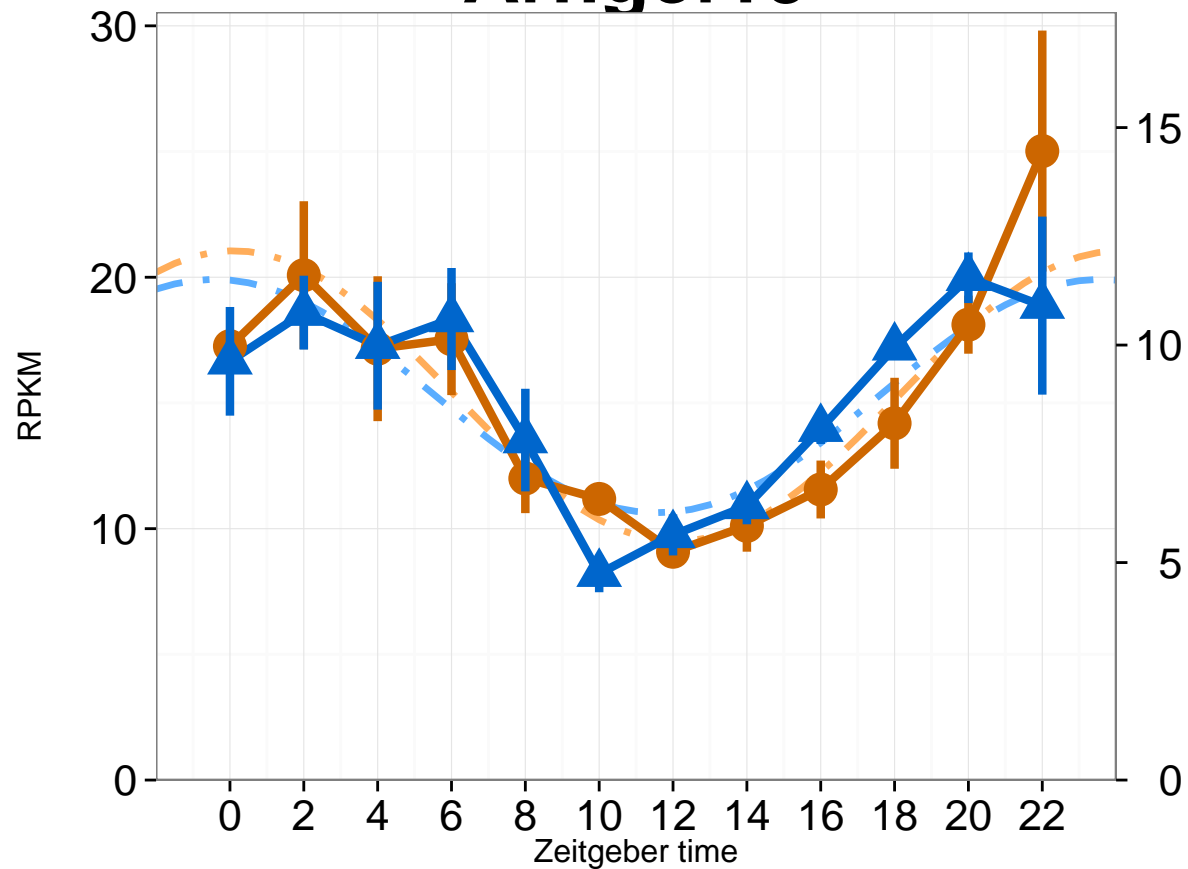

# Arhgef19

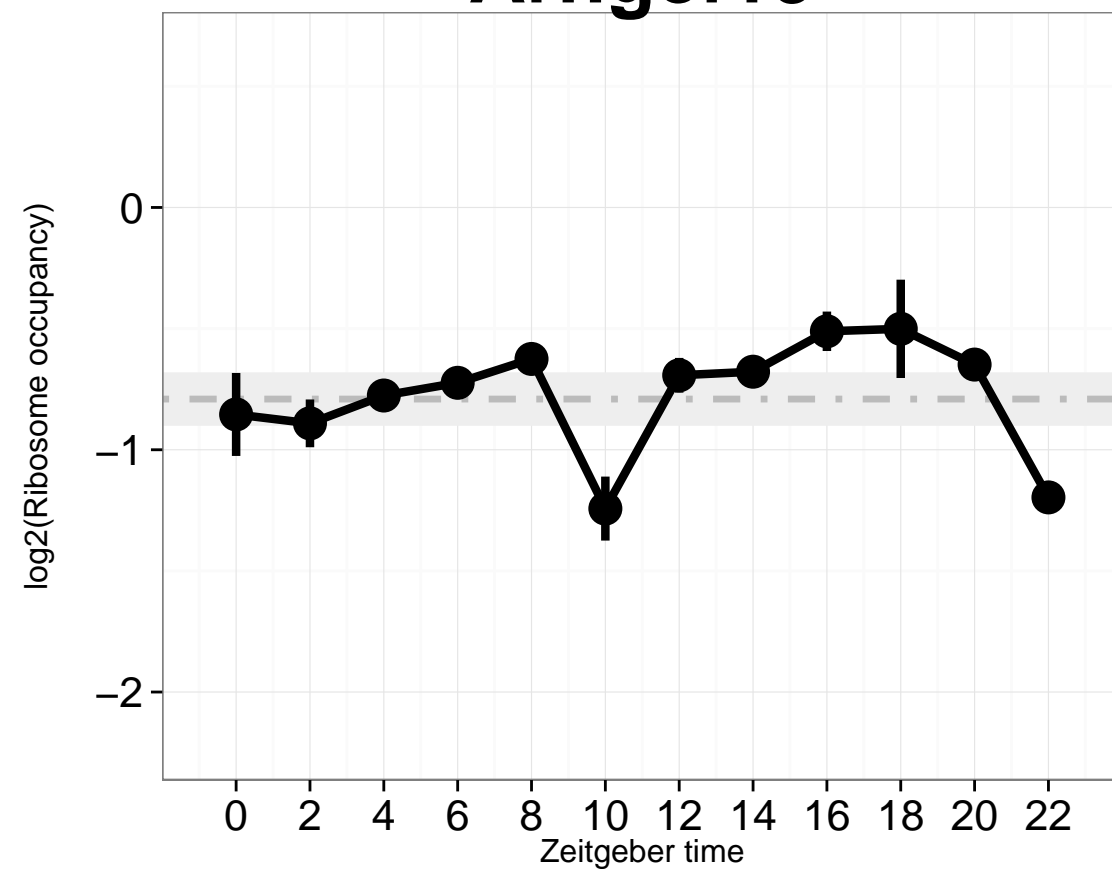

Supplement: Supplementary file 7 — Expression plots for kidney and liver for the 178 common rhythmic genes of Fig. 3c. (ZIP 3338.28 kb) [file 13059_2017_1222_MOESM7_ESM.zip › set_D_shared(178)/Arhgef19_liver_set_D.pdf]

## Arntl

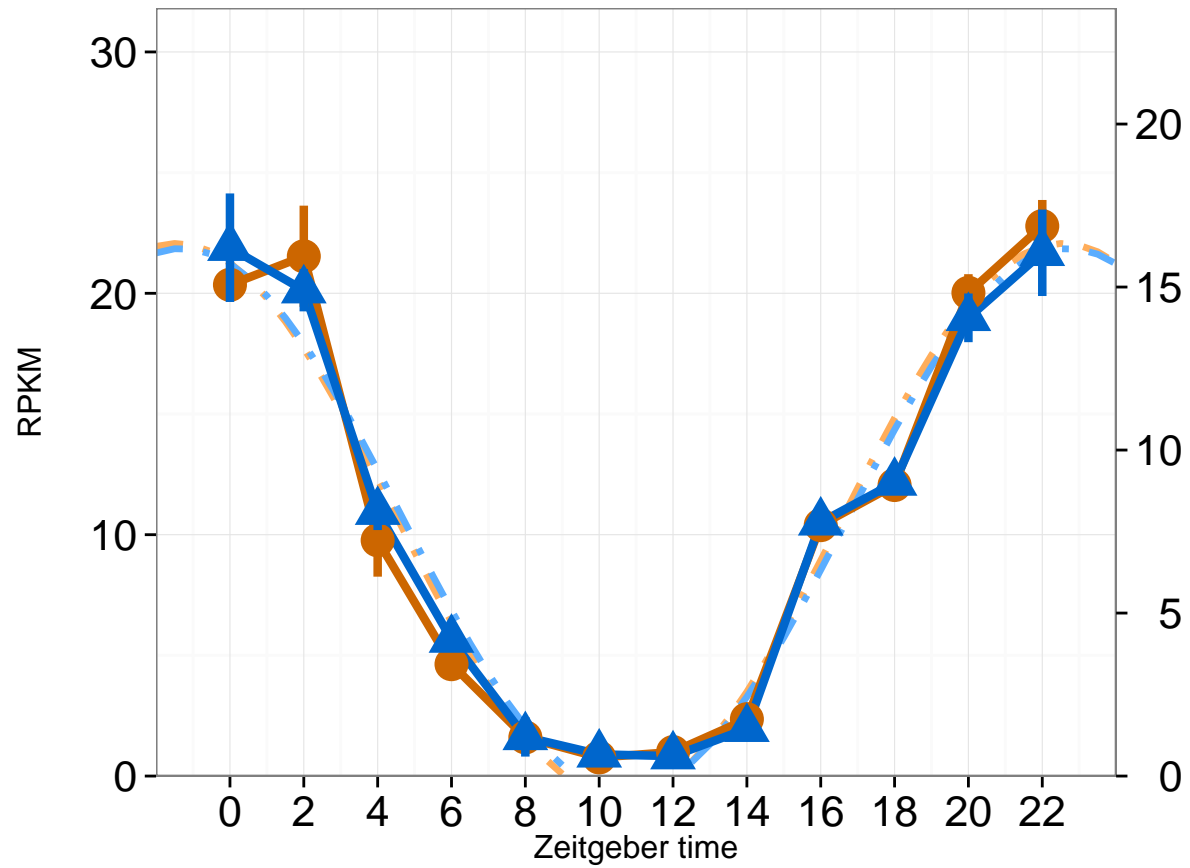

## Arntl

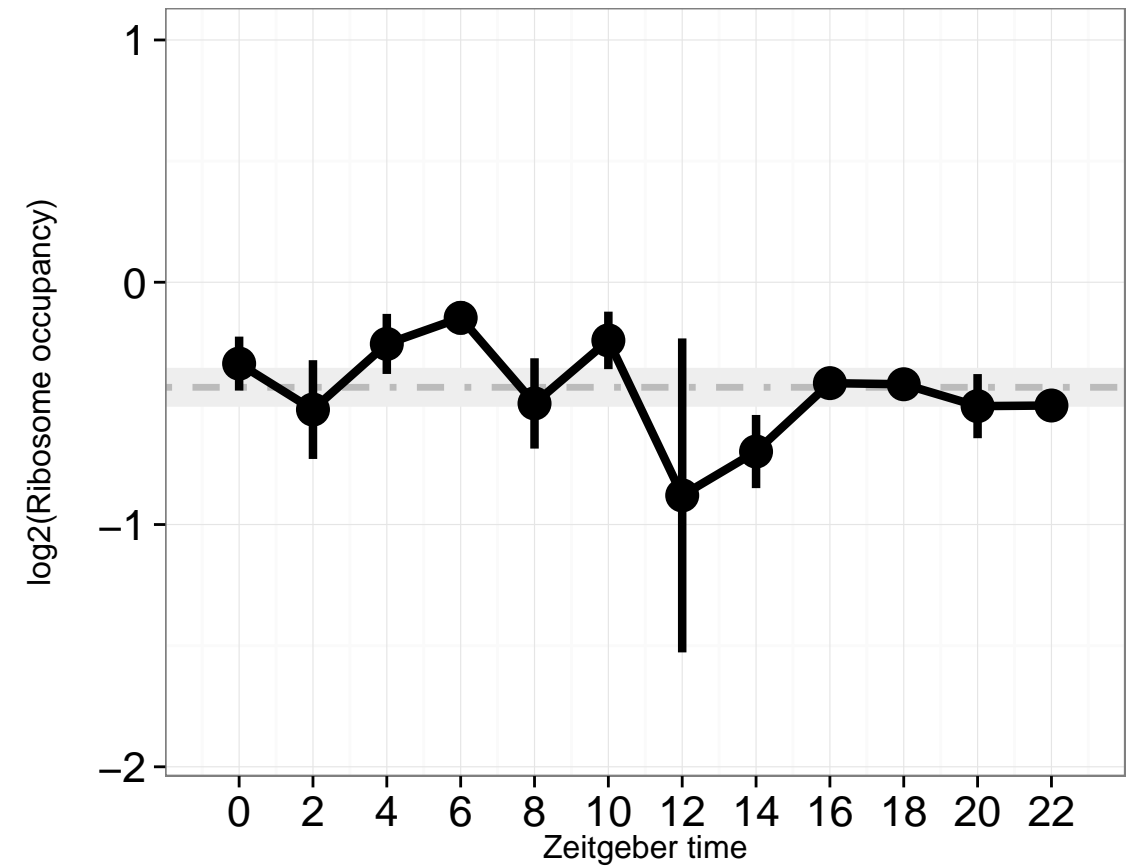

Supplement: Supplementary file 7 — Expression plots for kidney and liver for the 178 common rhythmic genes of Fig. 3c. (ZIP 3338.28 kb) [file 13059_2017_1222_MOESM7_ESM.zip › set_D_shared(178)/Arntl_kidney_set_D.pdf]

## Arntl

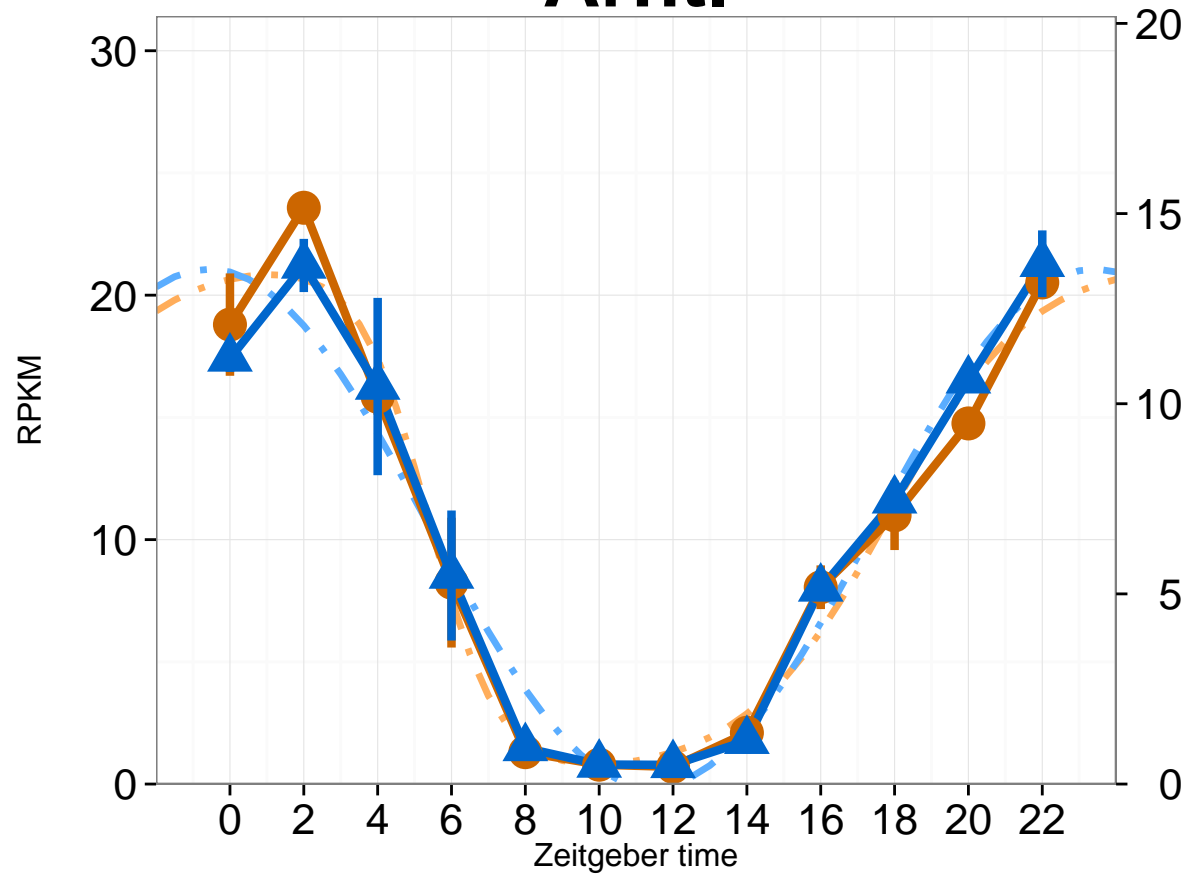

## Arntl

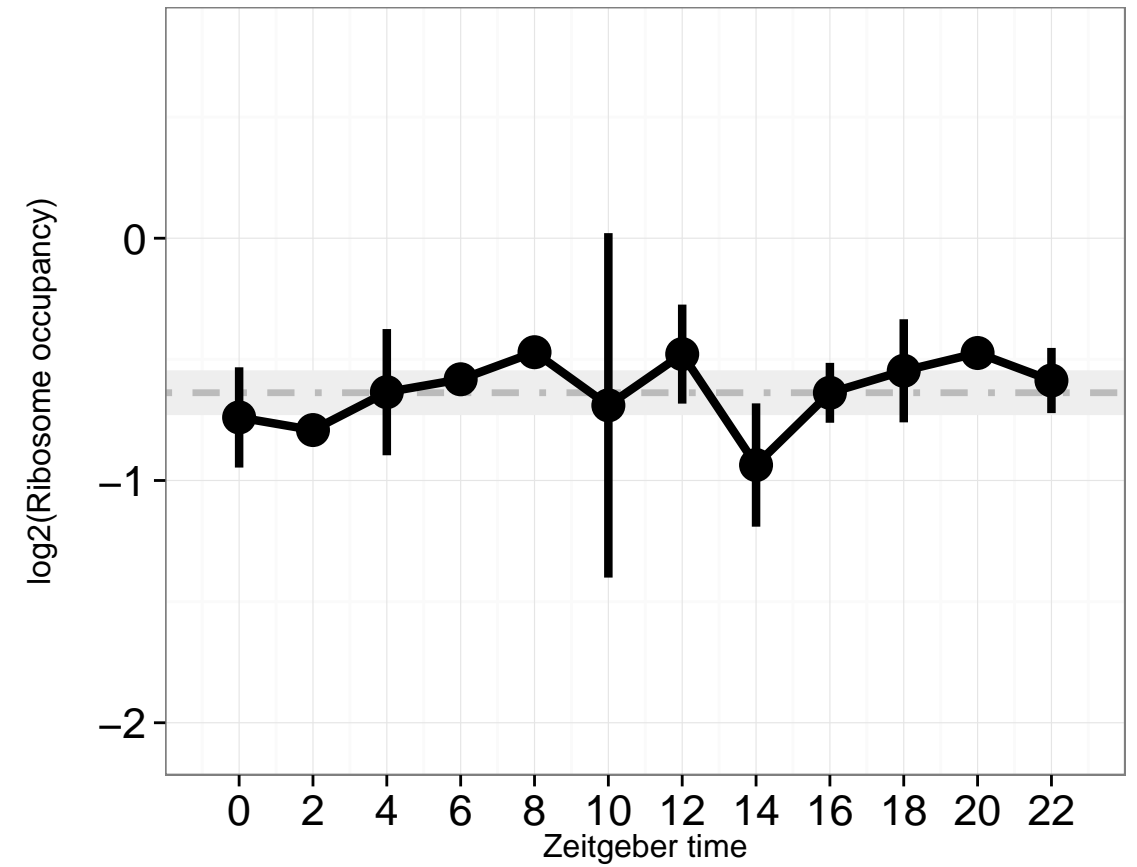

Supplement: Supplementary file 7 — Expression plots for kidney and liver for the 178 common rhythmic genes of Fig. 3c. (ZIP 3338.28 kb) [file 13059_2017_1222_MOESM7_ESM.zip › set_D_shared(178)/Arntl_liver_set_D.pdf]

# Arrdc3

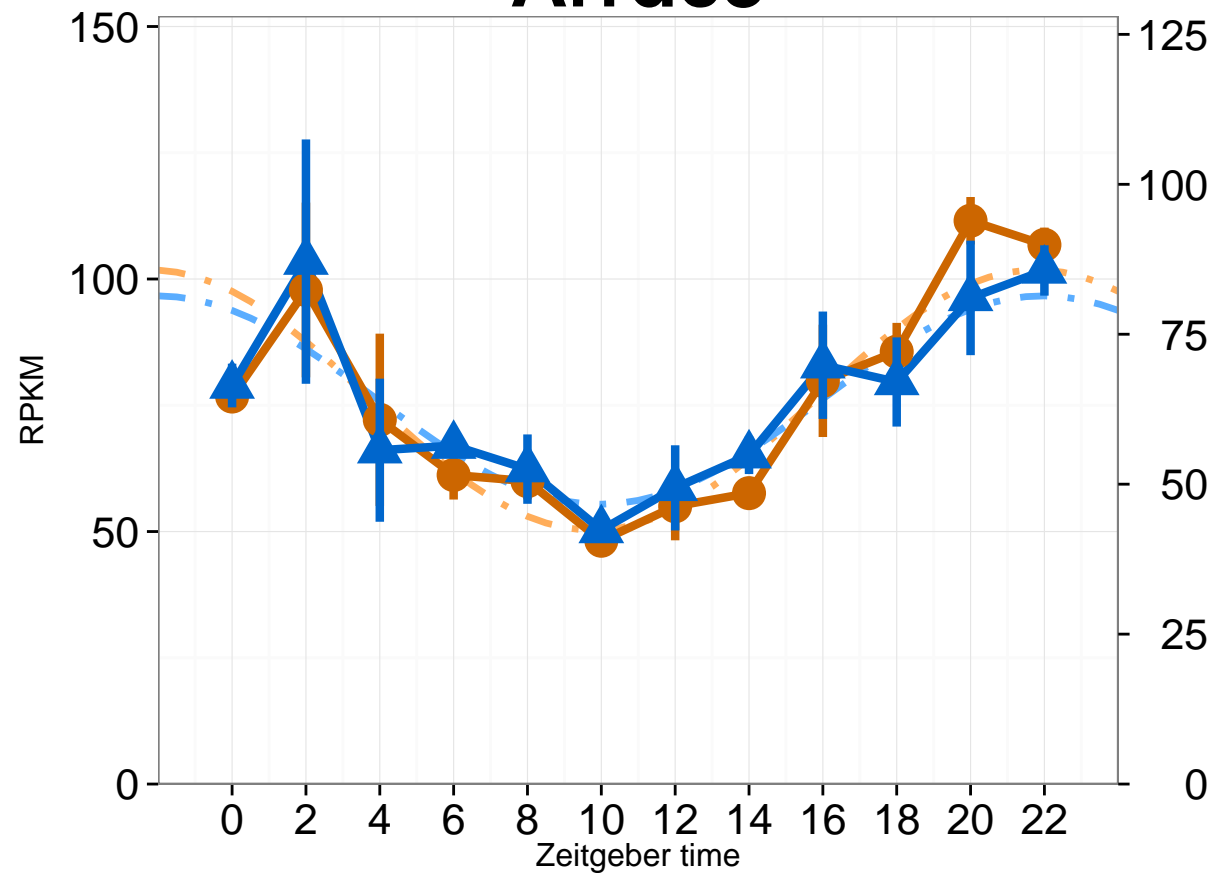

# Arrdc3

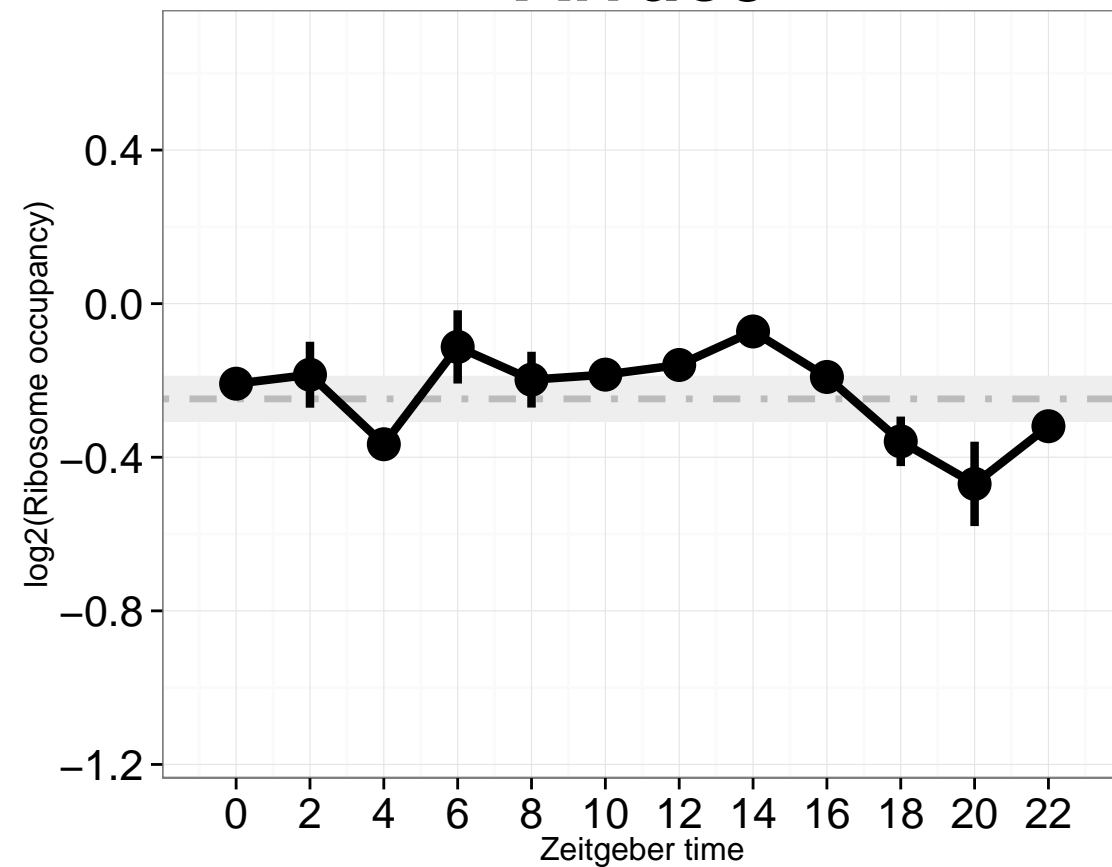

Supplement: Supplementary file 7 — Expression plots for kidney and liver for the 178 common rhythmic genes of Fig. 3c. (ZIP 3338.28 kb) [file 13059_2017_1222_MOESM7_ESM.zip › set_D_shared(178)/Arrdc3_kidney_set_D.pdf]

# Arrdc3

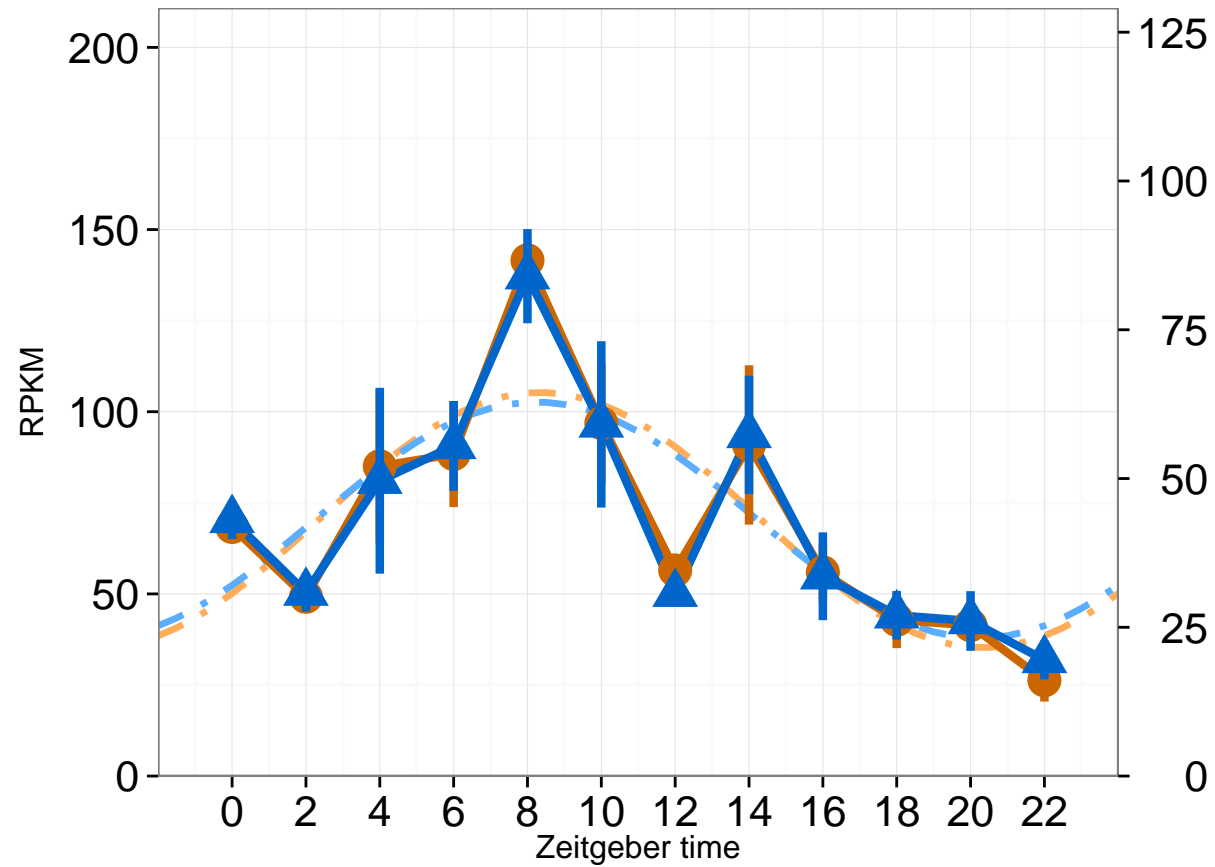

# Arrdc3

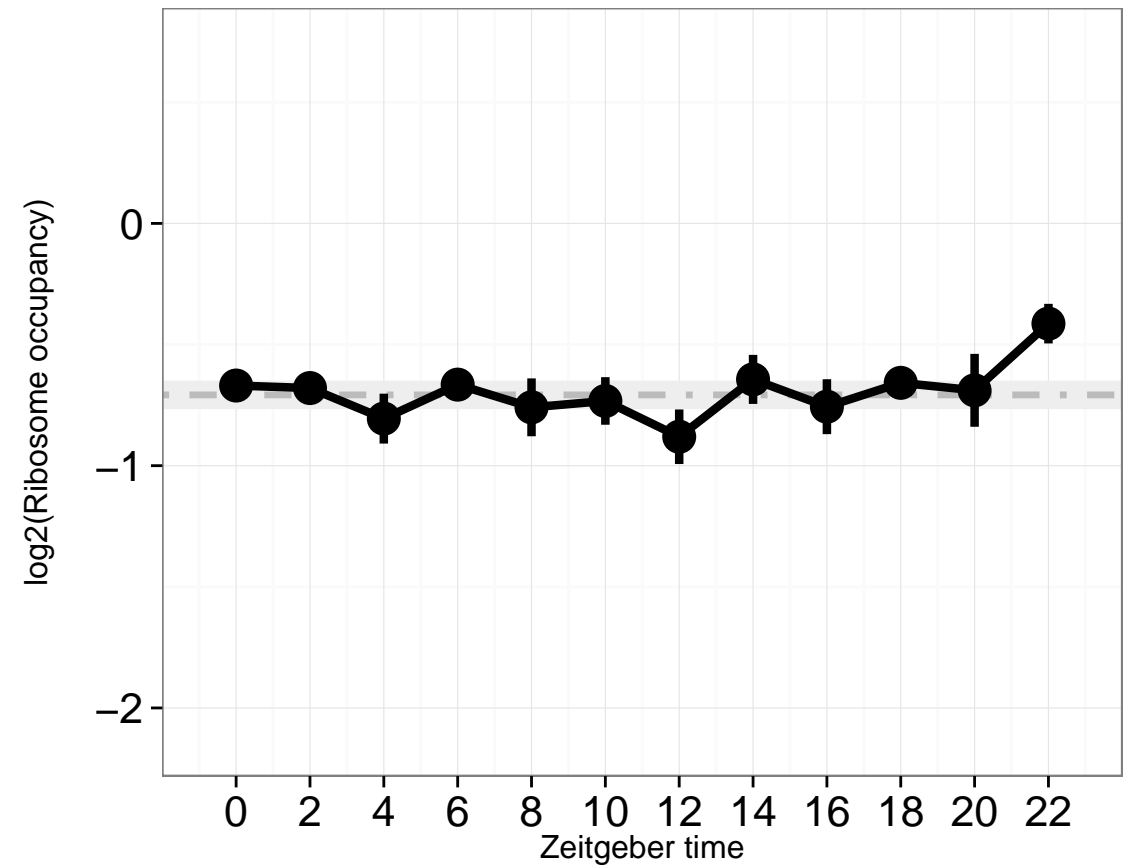

Supplement: Supplementary file 7 — Expression plots for kidney and liver for the 178 common rhythmic genes of Fig. 3c. (ZIP 3338.28 kb) [file 13059_2017_1222_MOESM7_ESM.zip › set_D_shared(178)/Arrdc3_liver_set_D.pdf]

## Arsq

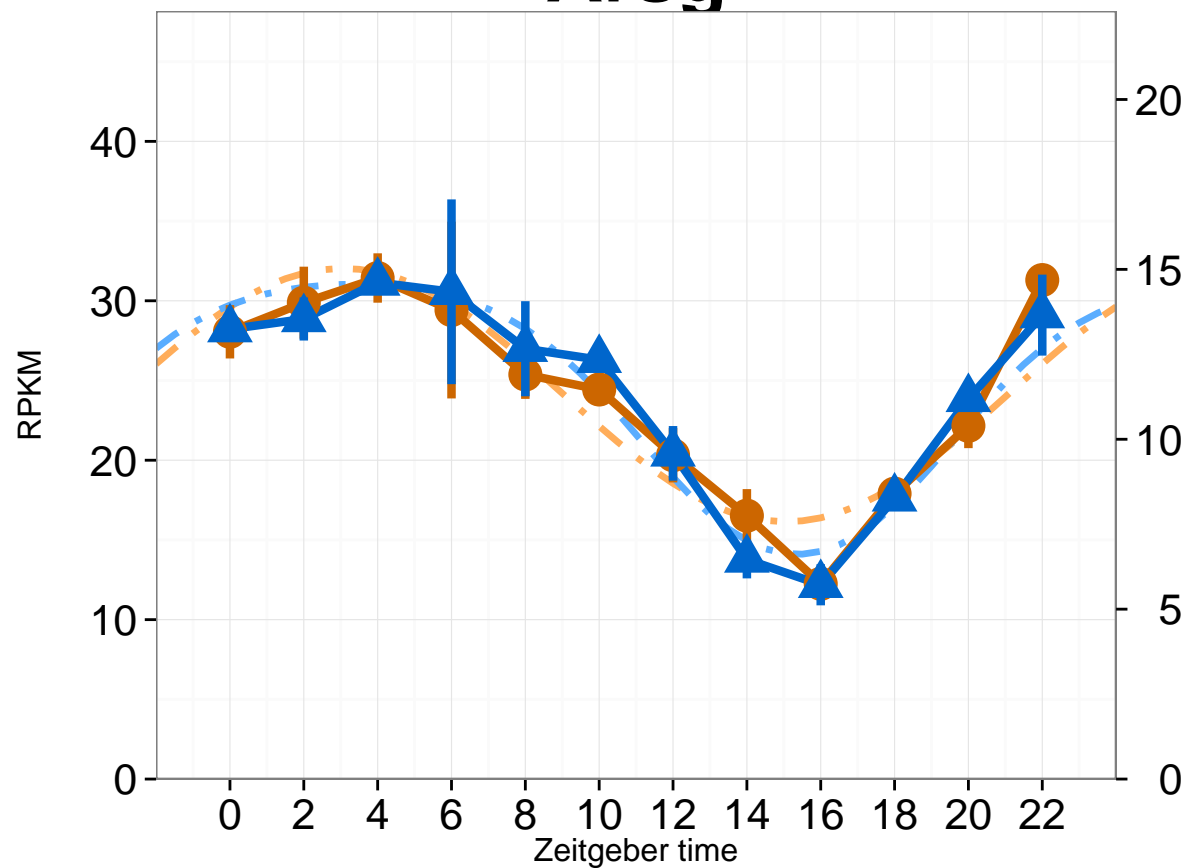

## Arsq

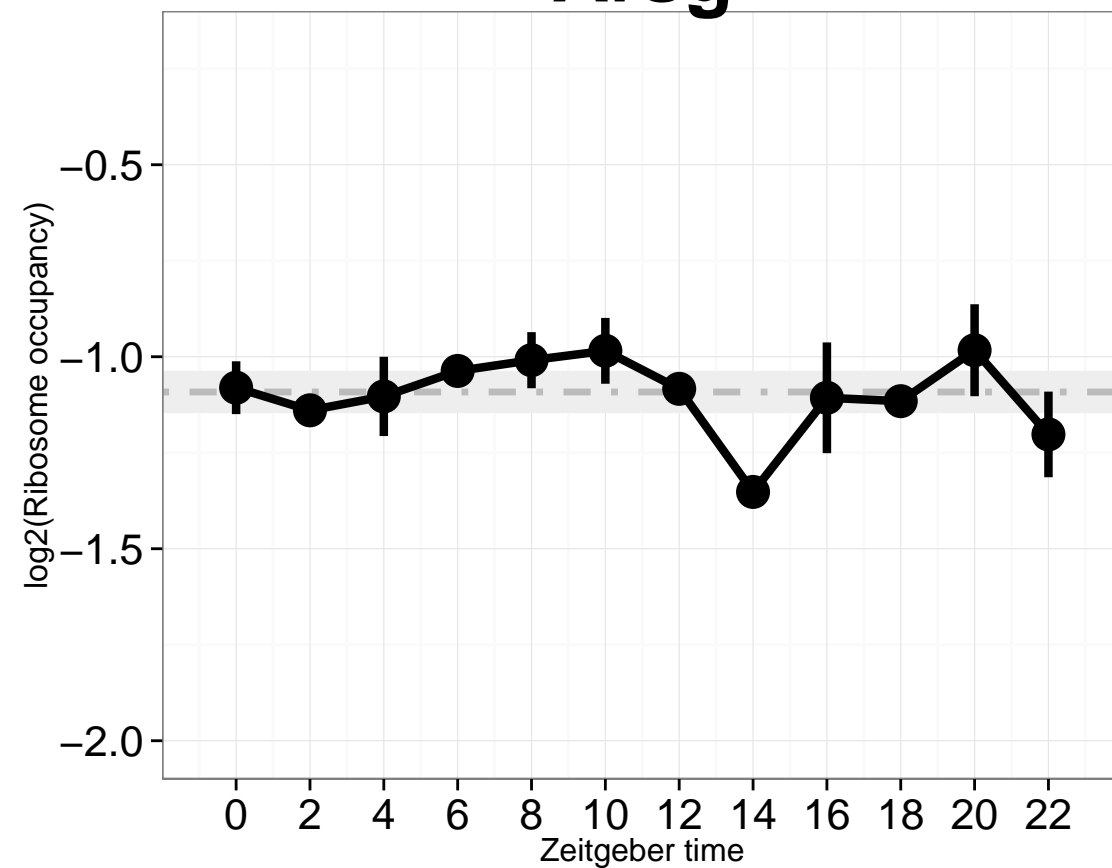

Supplement: Supplementary file 7 — Expression plots for kidney and liver for the 178 common rhythmic genes of Fig. 3c. (ZIP 3338.28 kb) [file 13059_2017_1222_MOESM7_ESM.zip › set_D_shared(178)/Arsg_kidney_set_D.pdf]

## Arsg

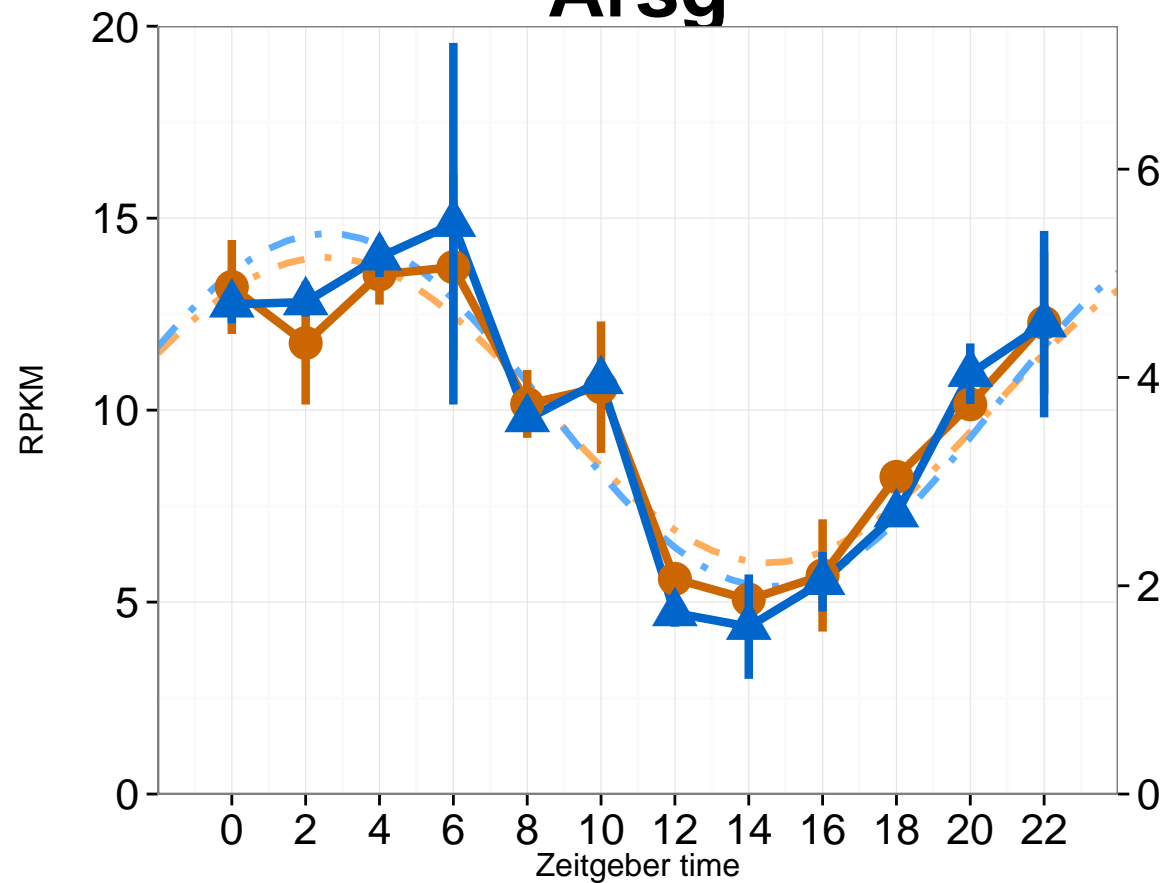

## Arsg

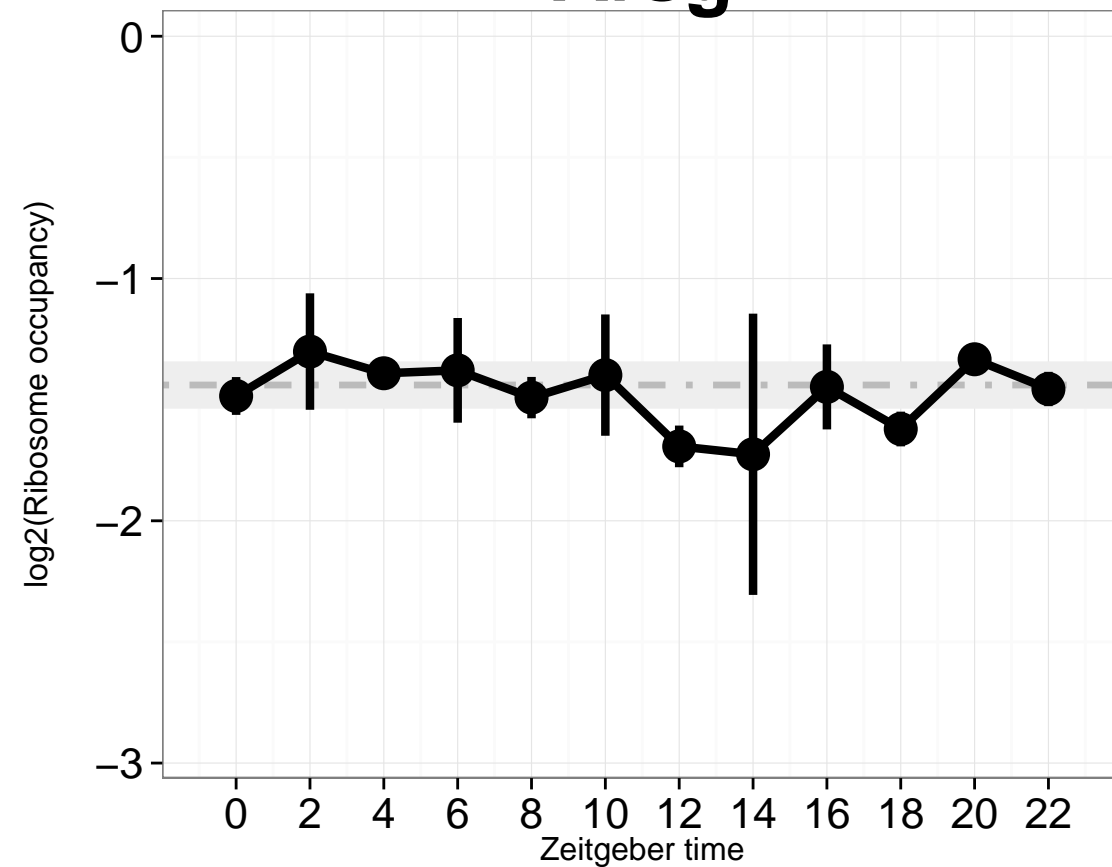

Supplement: Supplementary file 7 — Expression plots for kidney and liver for the 178 common rhythmic genes of Fig. 3c. (ZIP 3338.28 kb) [file 13059_2017_1222_MOESM7_ESM.zip › set_D_shared(178)/Arsg_liver_set_D.pdf]

# Avpr1a

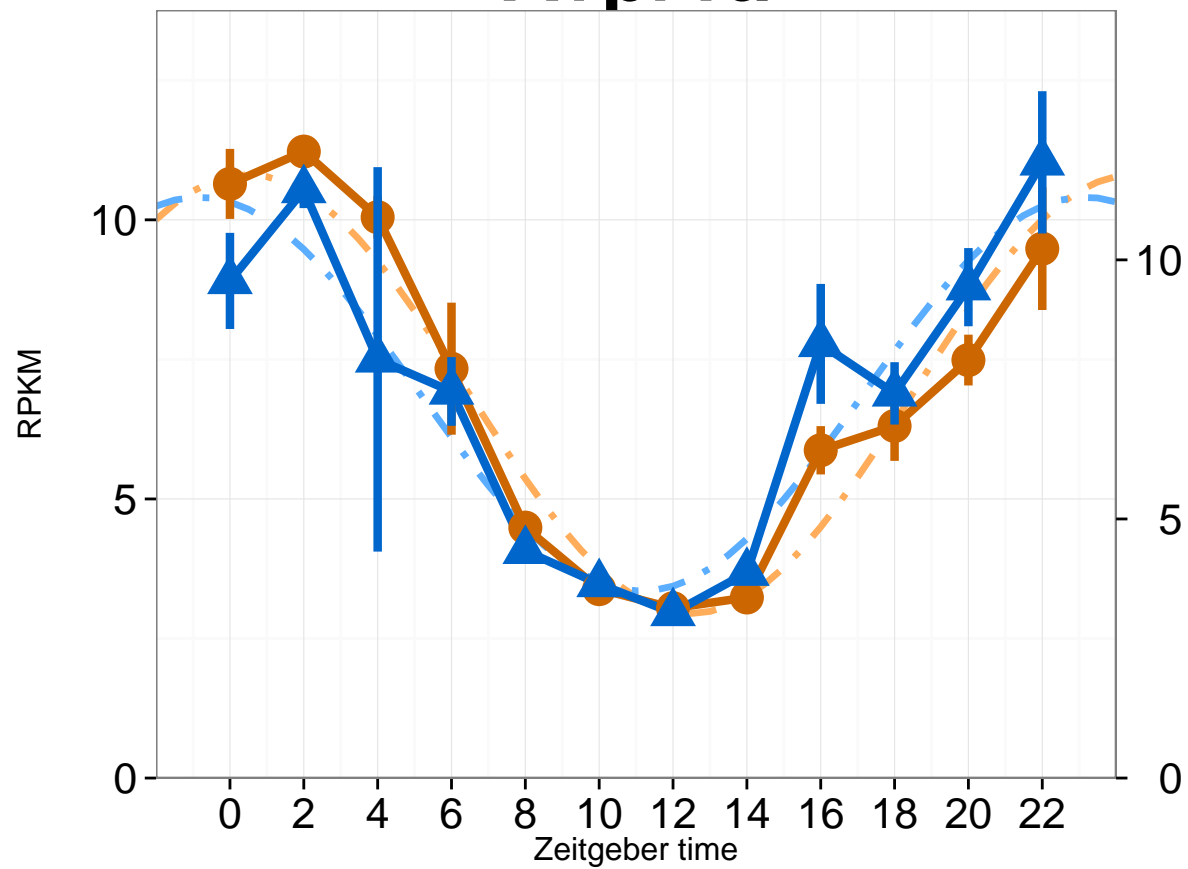

# Avpr1a

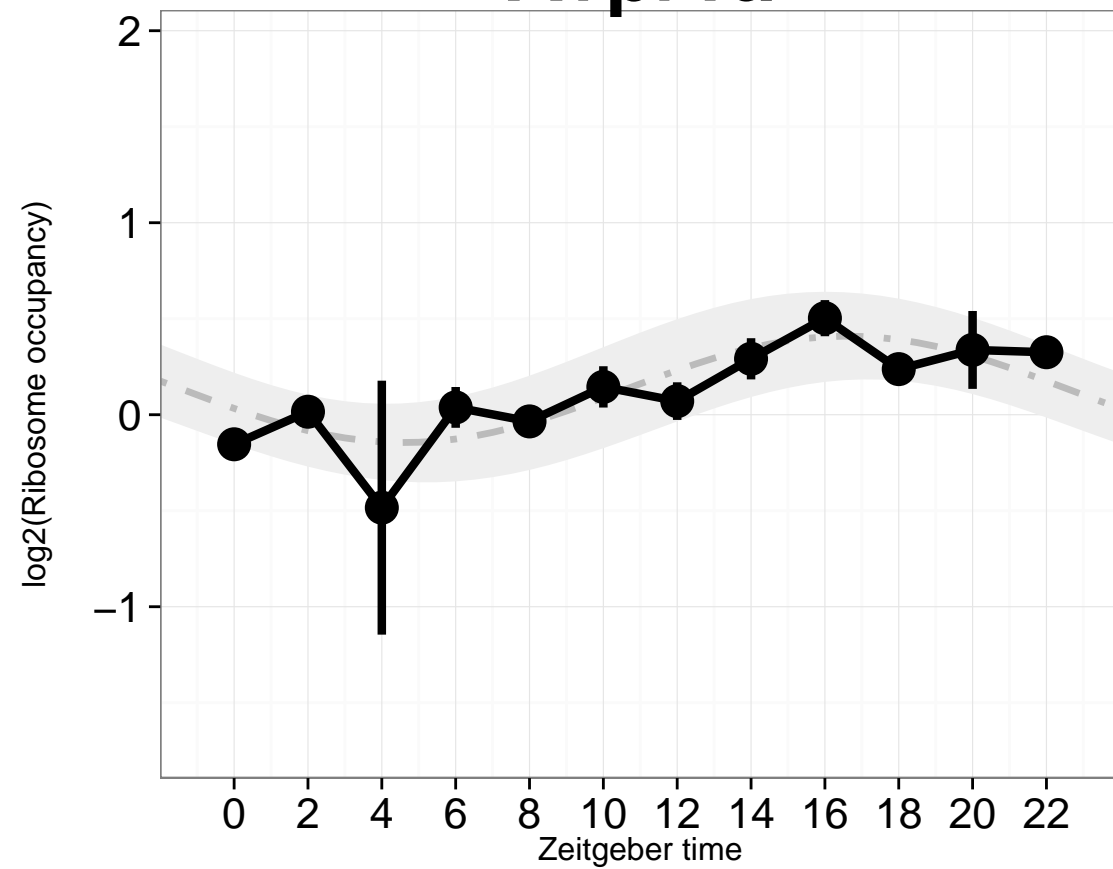

Supplement: Supplementary file 7 — Expression plots for kidney and liver for the 178 common rhythmic genes of Fig. 3c. (ZIP 3338.28 kb) [file 13059_2017_1222_MOESM7_ESM.zip › set_D_shared(178)/Avpr1a_kidney_set_D.pdf]

# Avpr1a

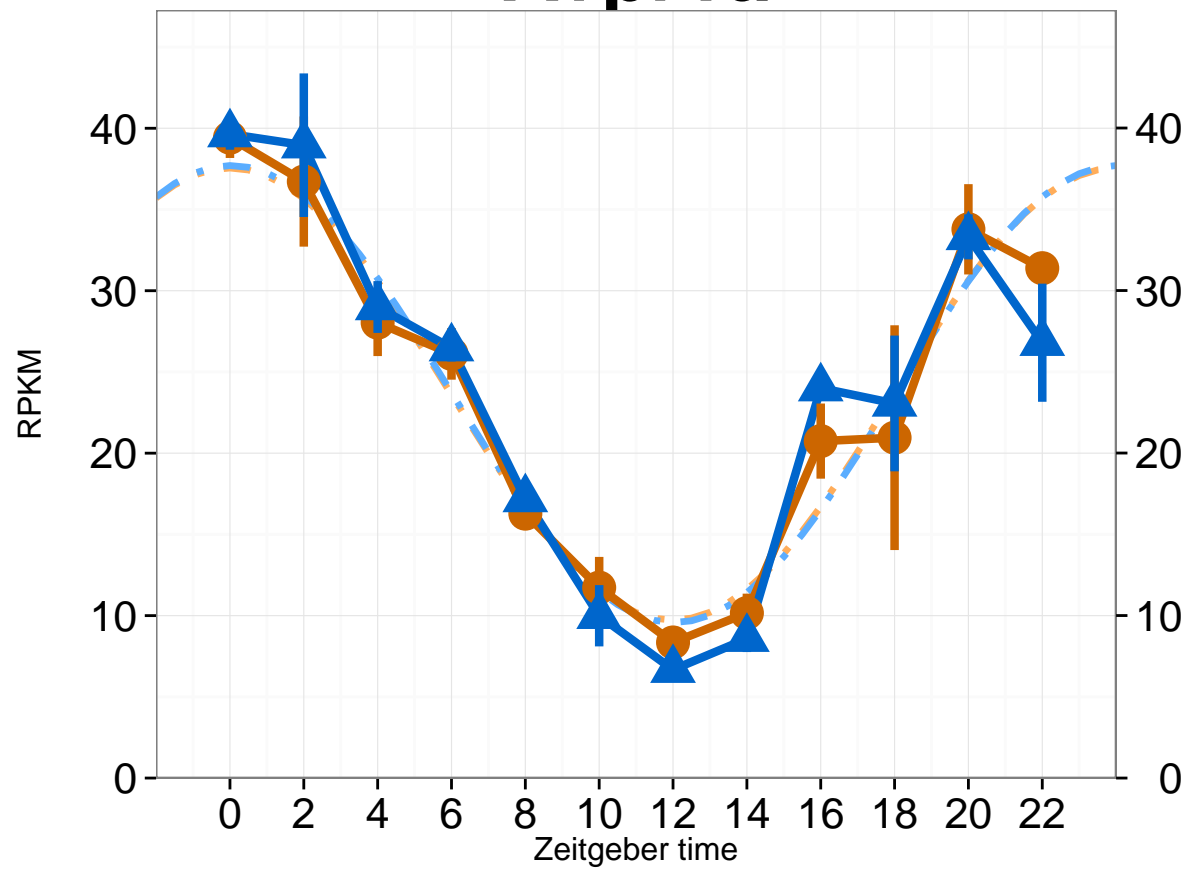

# Avpr1a

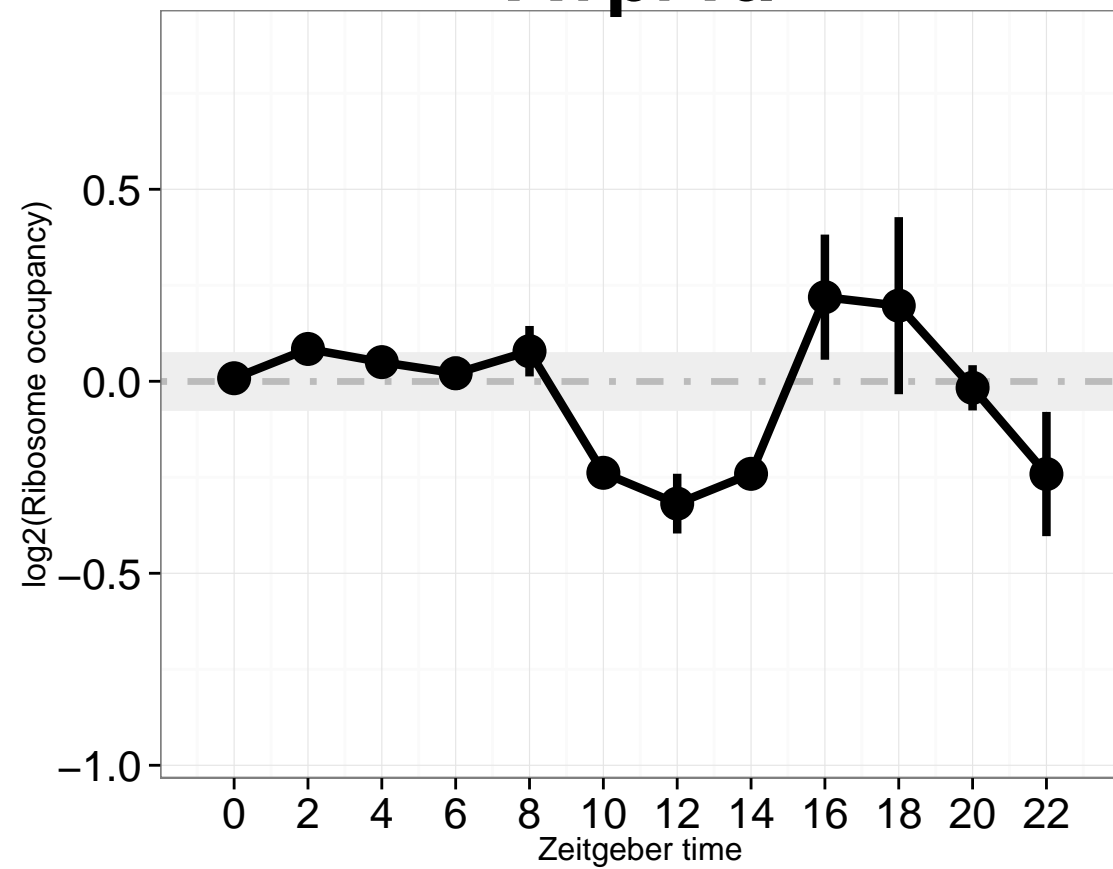

Supplement: Supplementary file 7 — Expression plots for kidney and liver for the 178 common rhythmic genes of Fig. 3c. (ZIP 3338.28 kb) [file 13059_2017_1222_MOESM7_ESM.zip › set_D_shared(178)/Avpr1a_liver_set_D.pdf]

## Baiap2

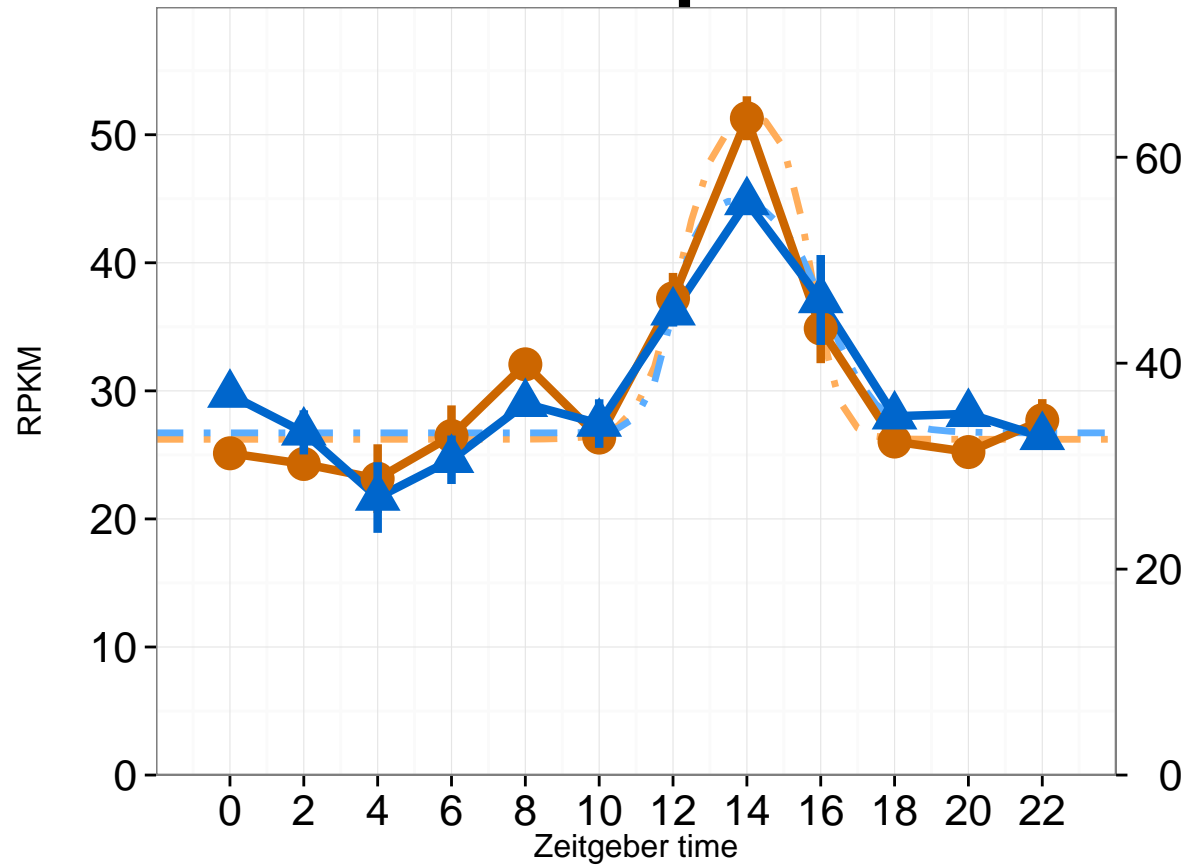

## Baiap2

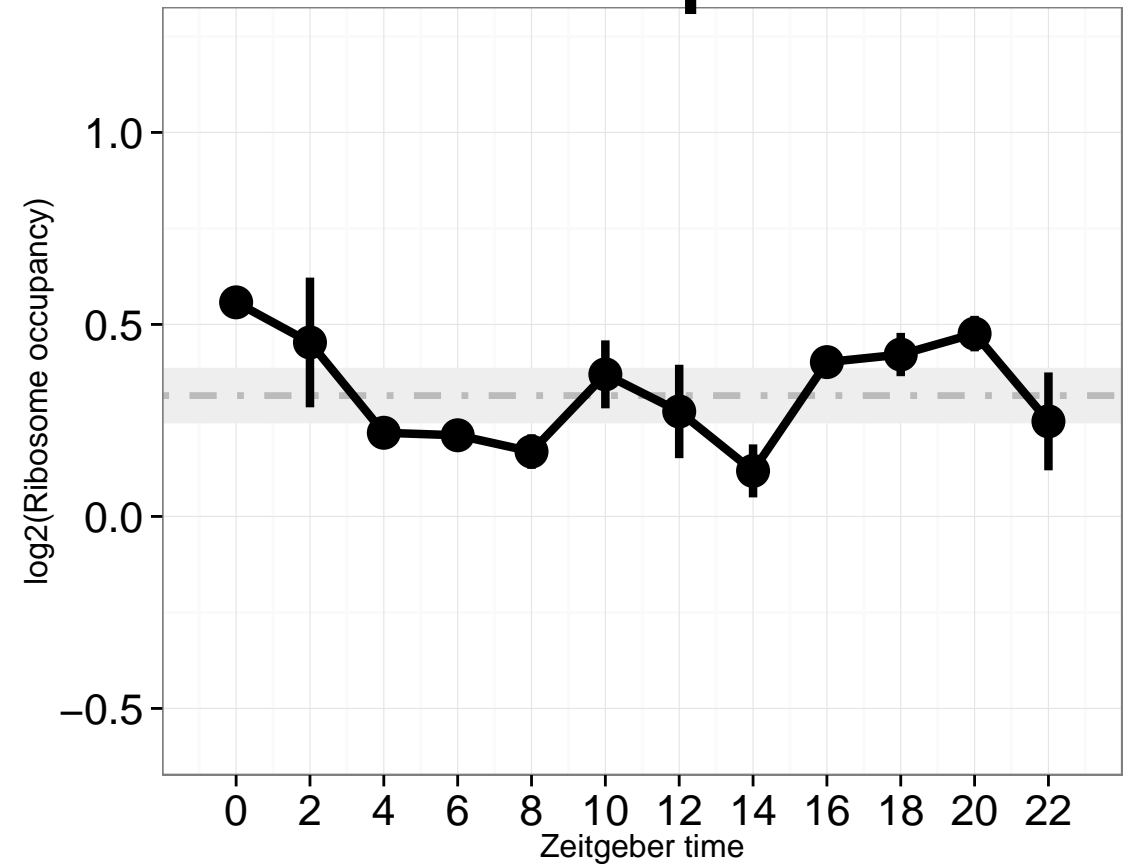

Supplement: Supplementary file 7 — Expression plots for kidney and liver for the 178 common rhythmic genes of Fig. 3c. (ZIP 3338.28 kb) [file 13059_2017_1222_MOESM7_ESM.zip › set_D_shared(178)/Baiap2_kidney_set_D.pdf]

## Baiap2

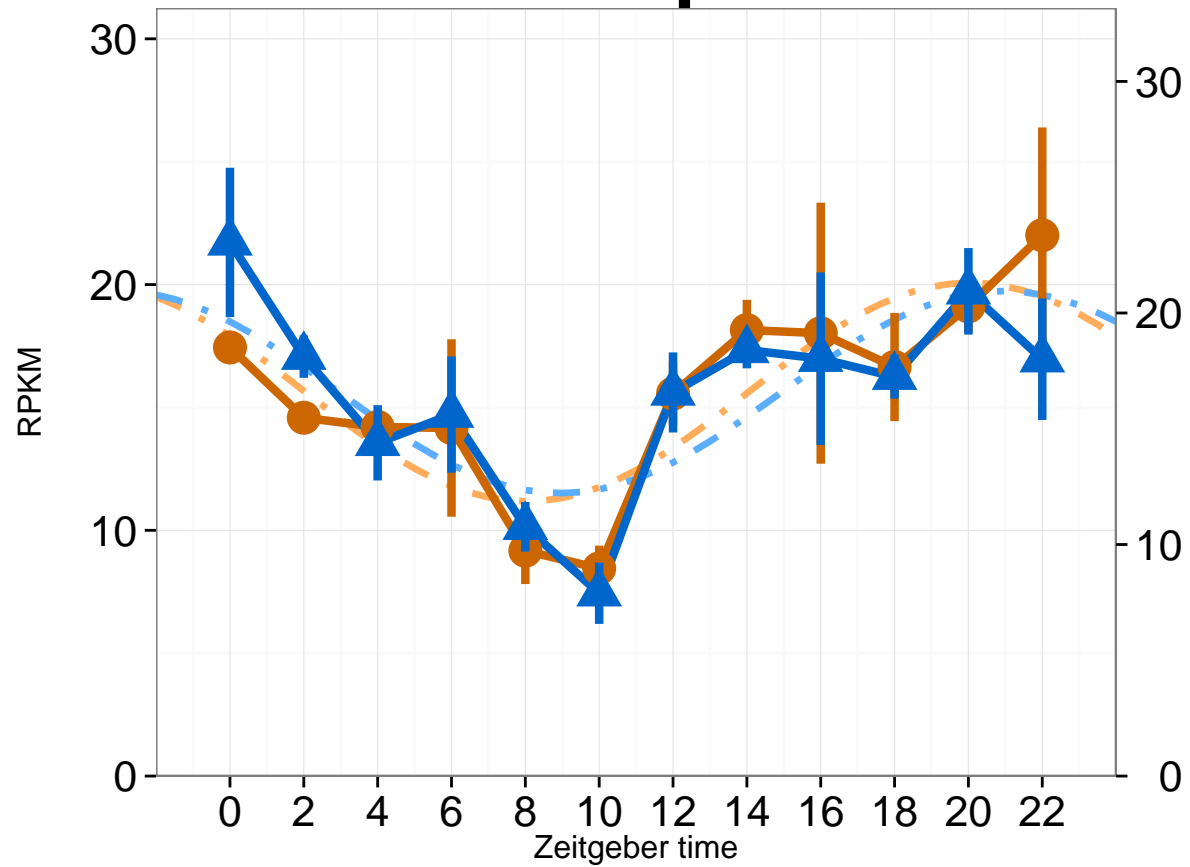

## Baiap2

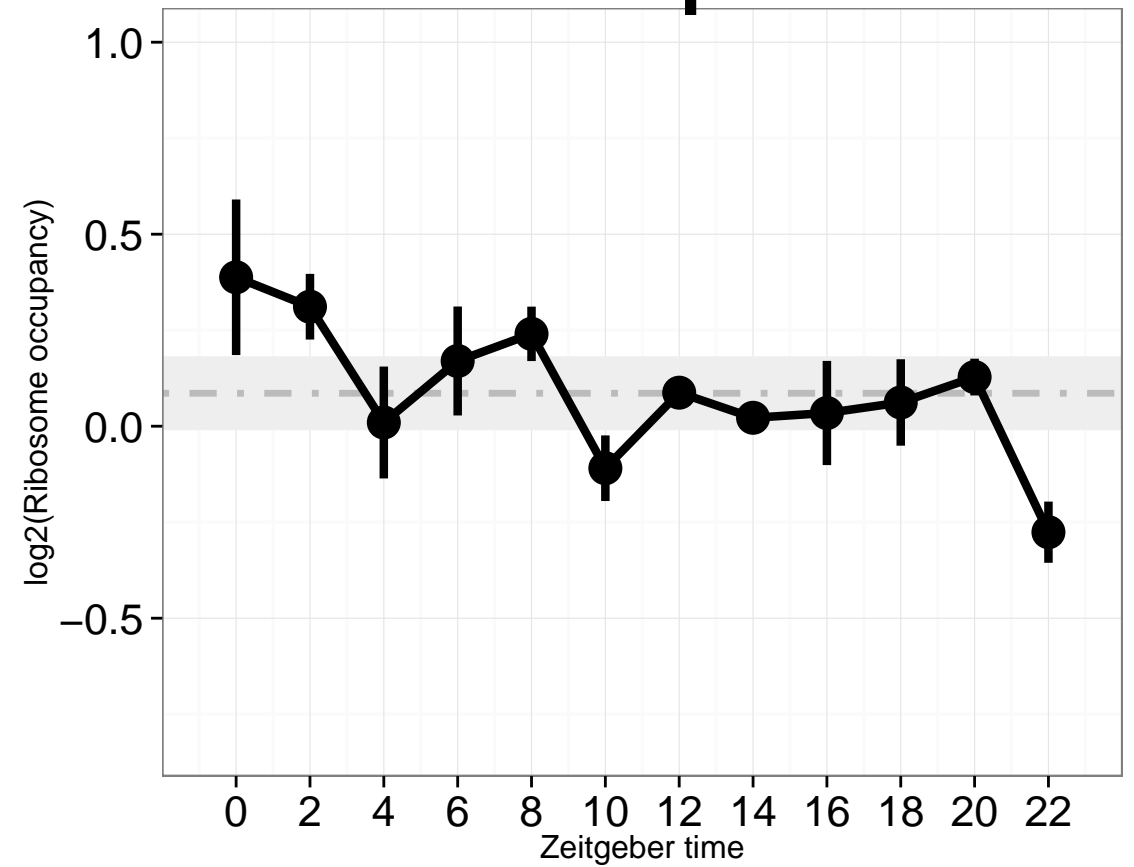

Supplement: Supplementary file 7 — Expression plots for kidney and liver for the 178 common rhythmic genes of Fig. 3c. (ZIP 3338.28 kb) [file 13059_2017_1222_MOESM7_ESM.zip › set_D_shared(178)/Baiap2_liver_set_D.pdf]

## Bhlhe40

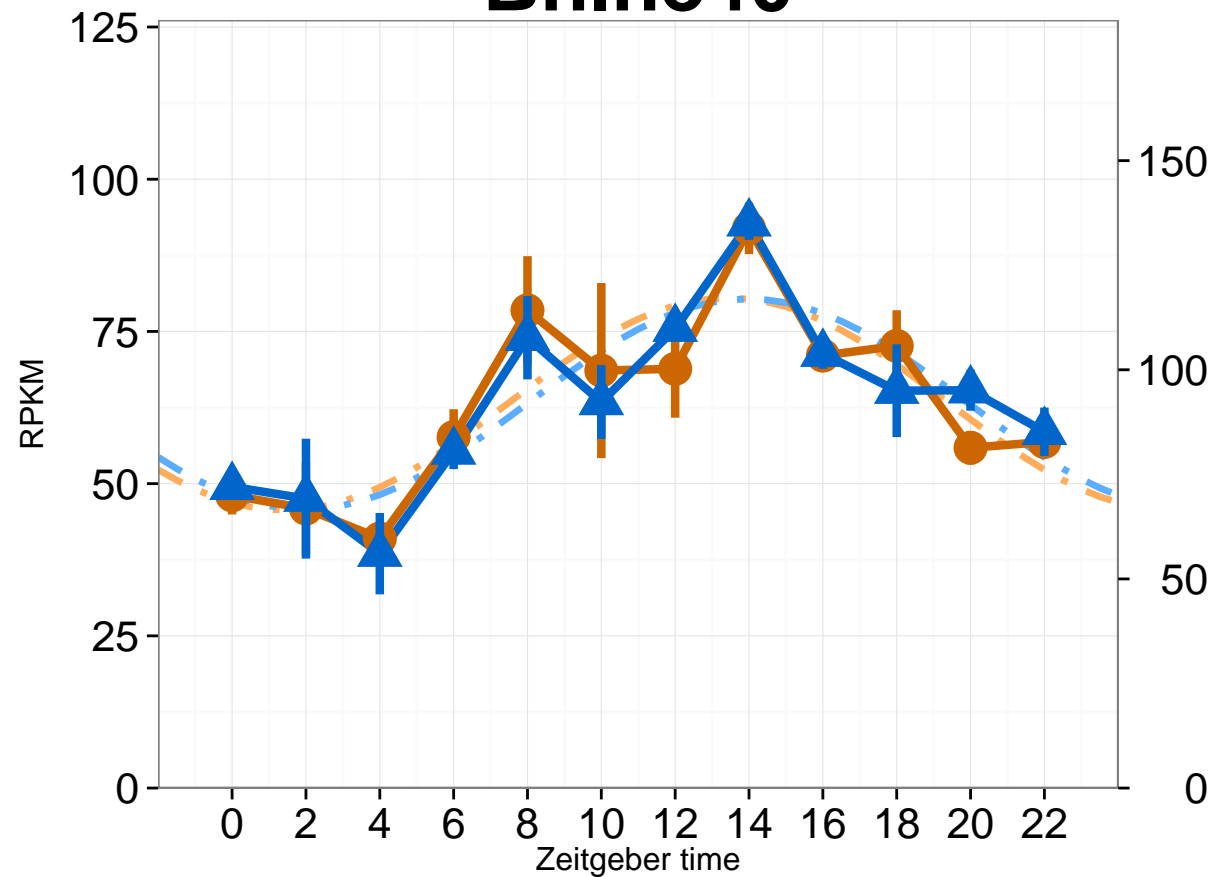

## Bhlhe40

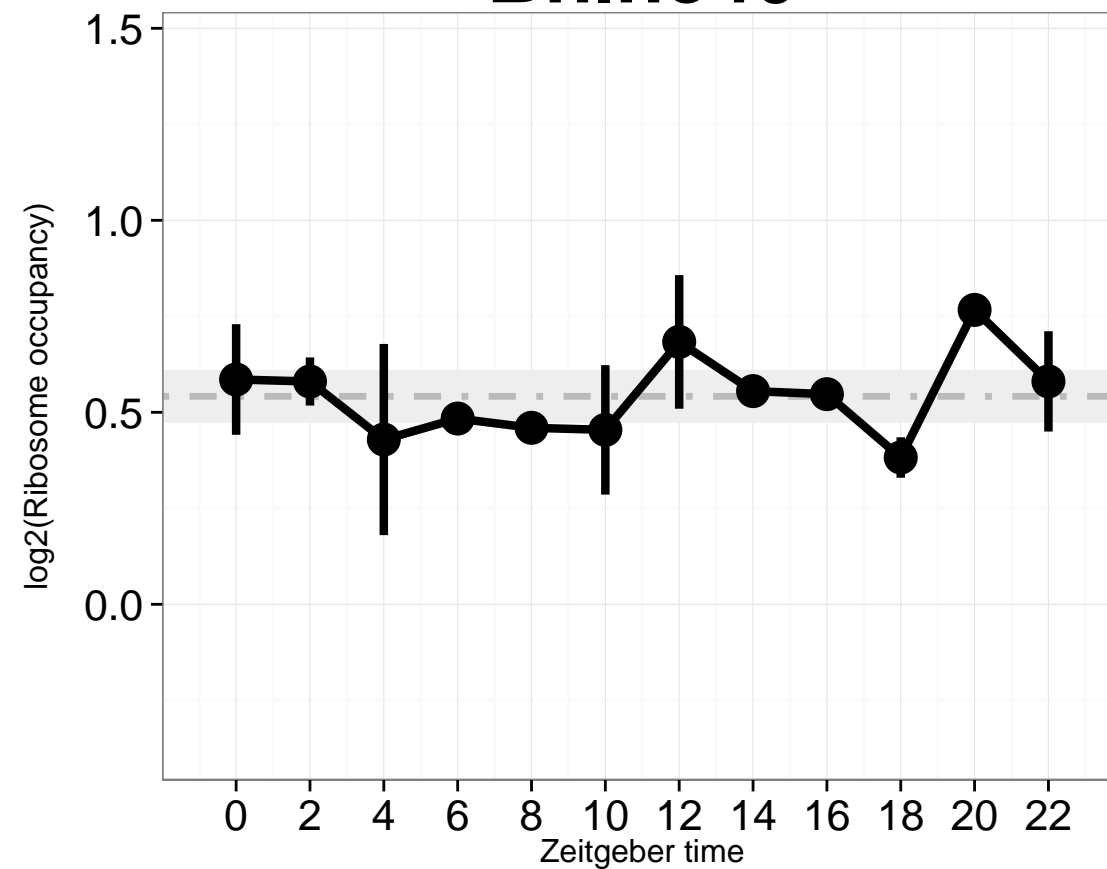

Supplement: Supplementary file 7 — Expression plots for kidney and liver for the 178 common rhythmic genes of Fig. 3c. (ZIP 3338.28 kb) [file 13059_2017_1222_MOESM7_ESM.zip › set_D_shared(178)/Bhlhe40_kidney_set_D.pdf]

# Bhlhe40

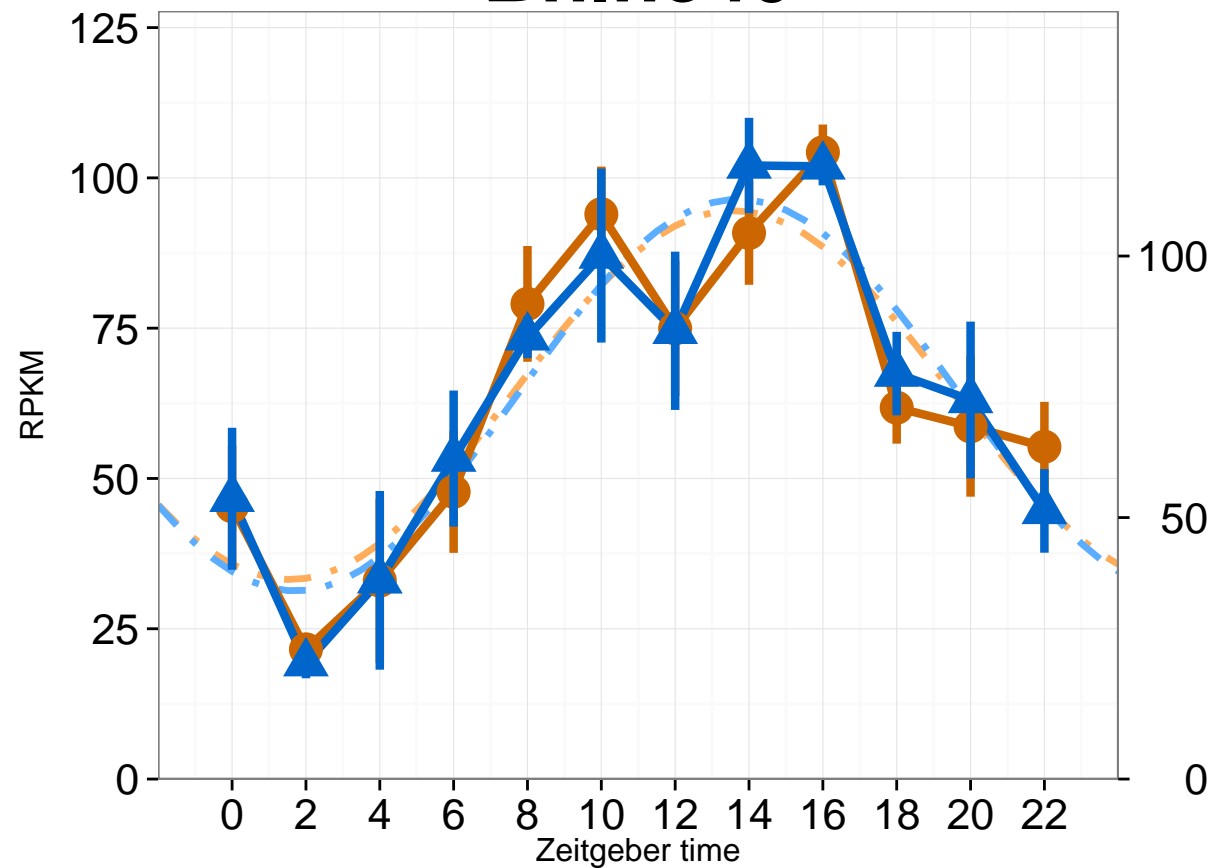

# Bhlhe40

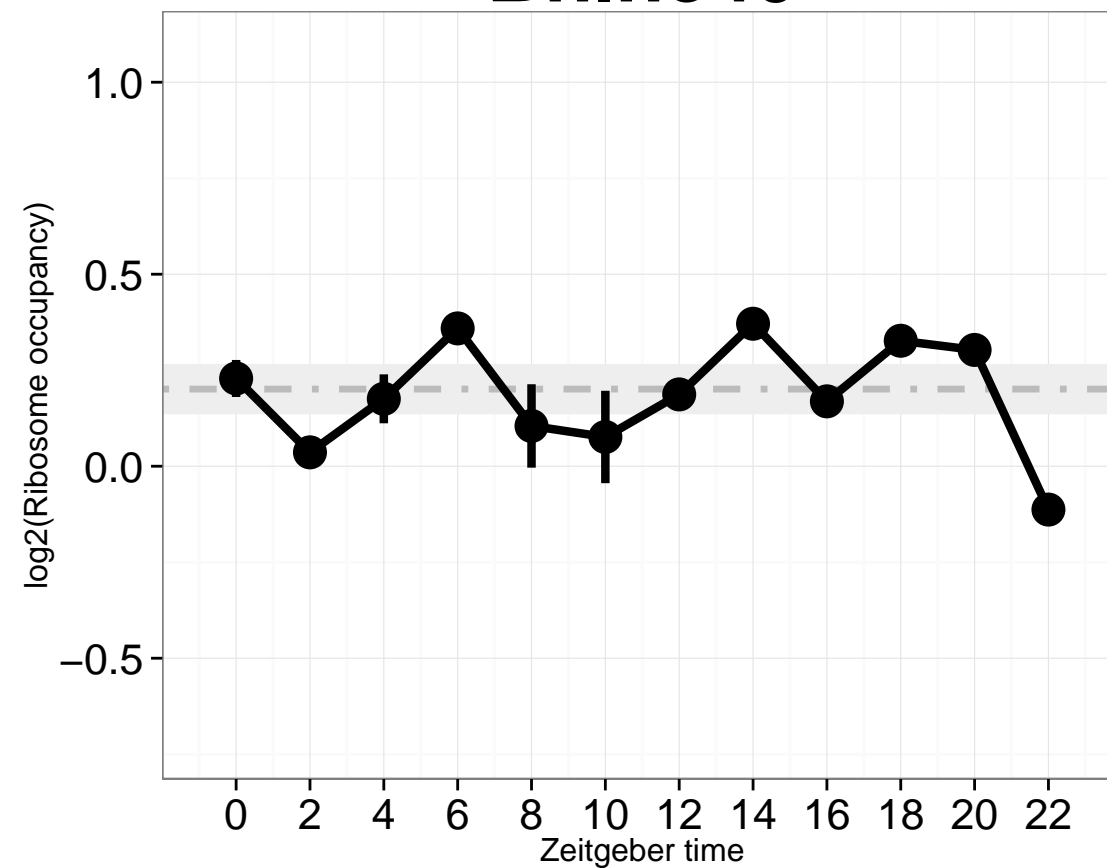

Supplement: Supplementary file 7 — Expression plots for kidney and liver for the 178 common rhythmic genes of Fig. 3c. (ZIP 3338.28 kb) [file 13059_2017_1222_MOESM7_ESM.zip › set_D_shared(178)/Bhlhe40_liver_set_D.pdf]

# Bhlhe41

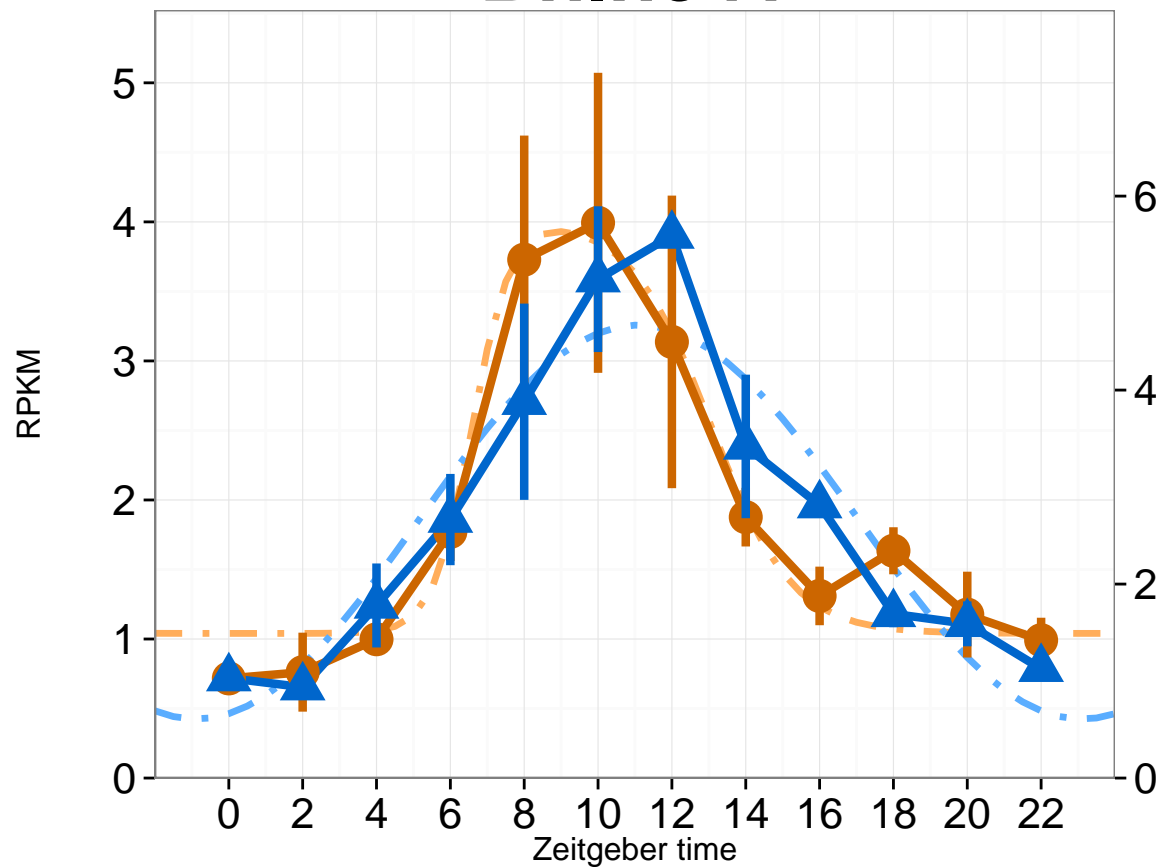

# Bhlhe41

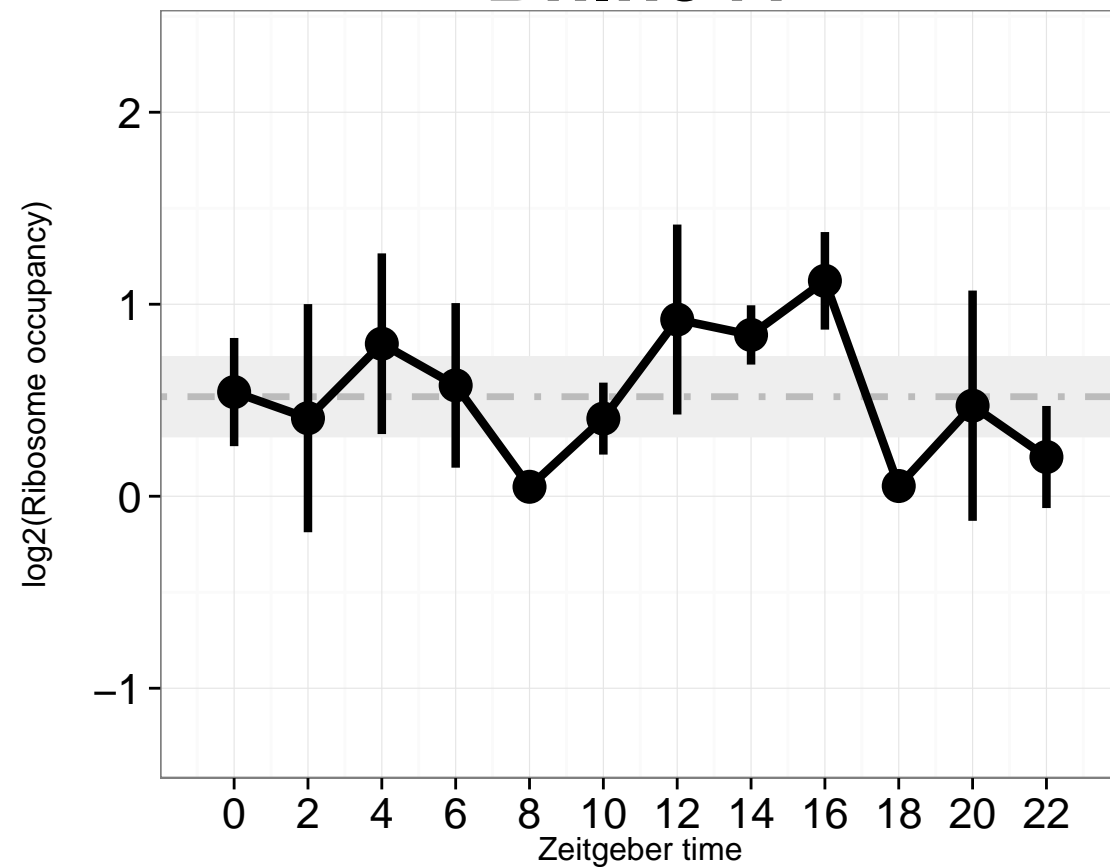

Supplement: Supplementary file 7 — Expression plots for kidney and liver for the 178 common rhythmic genes of Fig. 3c. (ZIP 3338.28 kb) [file 13059_2017_1222_MOESM7_ESM.zip › set_D_shared(178)/Bhlhe41_kidney_set_D.pdf]

# Bhlhe41

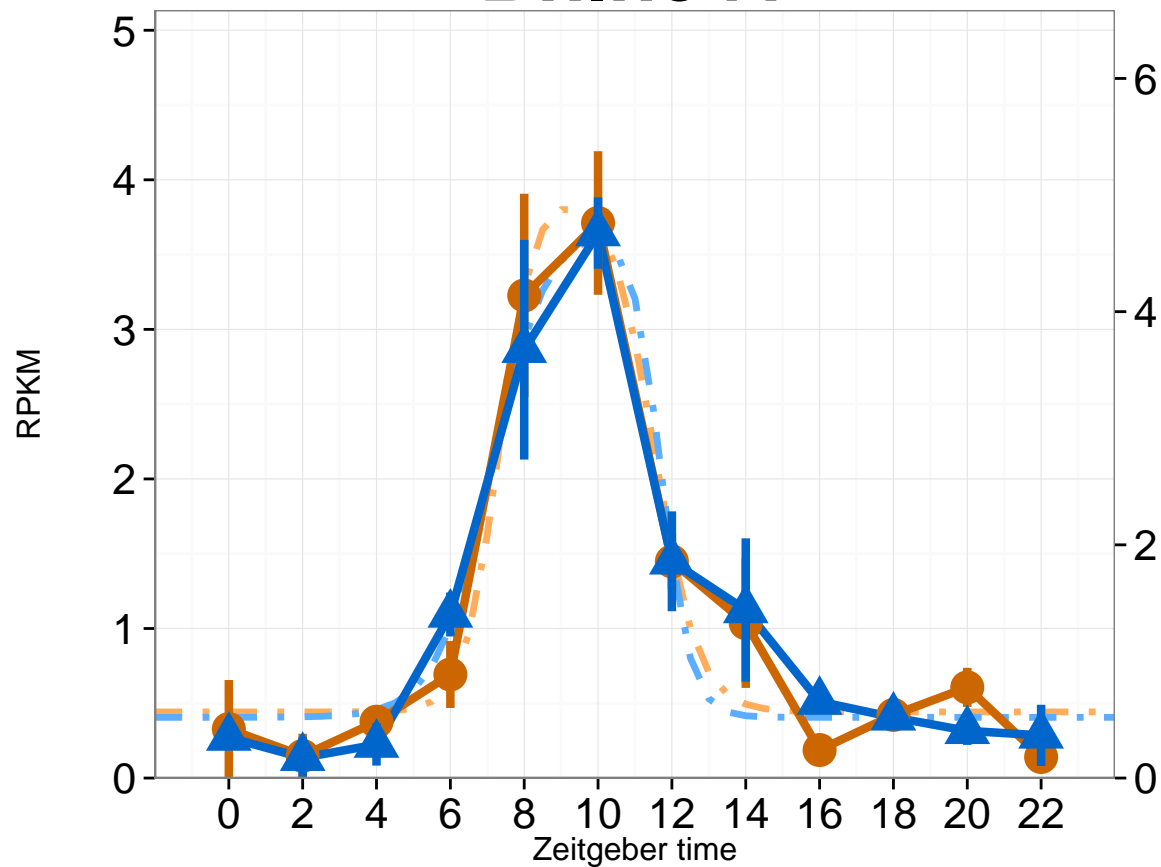

# Bhlhe41

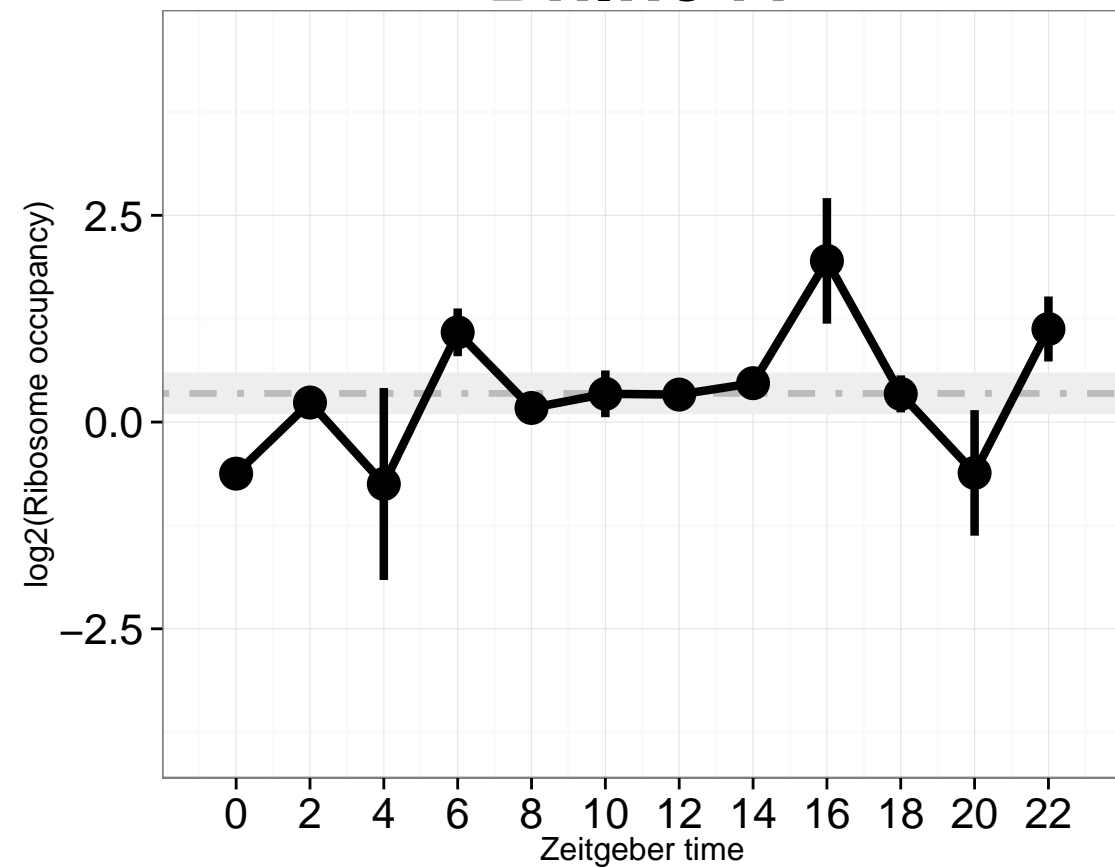

Supplement: Supplementary file 7 — Expression plots for kidney and liver for the 178 common rhythmic genes of Fig. 3c. (ZIP 3338.28 kb) [file 13059_2017_1222_MOESM7_ESM.zip › set_D_shared(178)/Bhlhe41_liver_set_D.pdf]

## Bmf

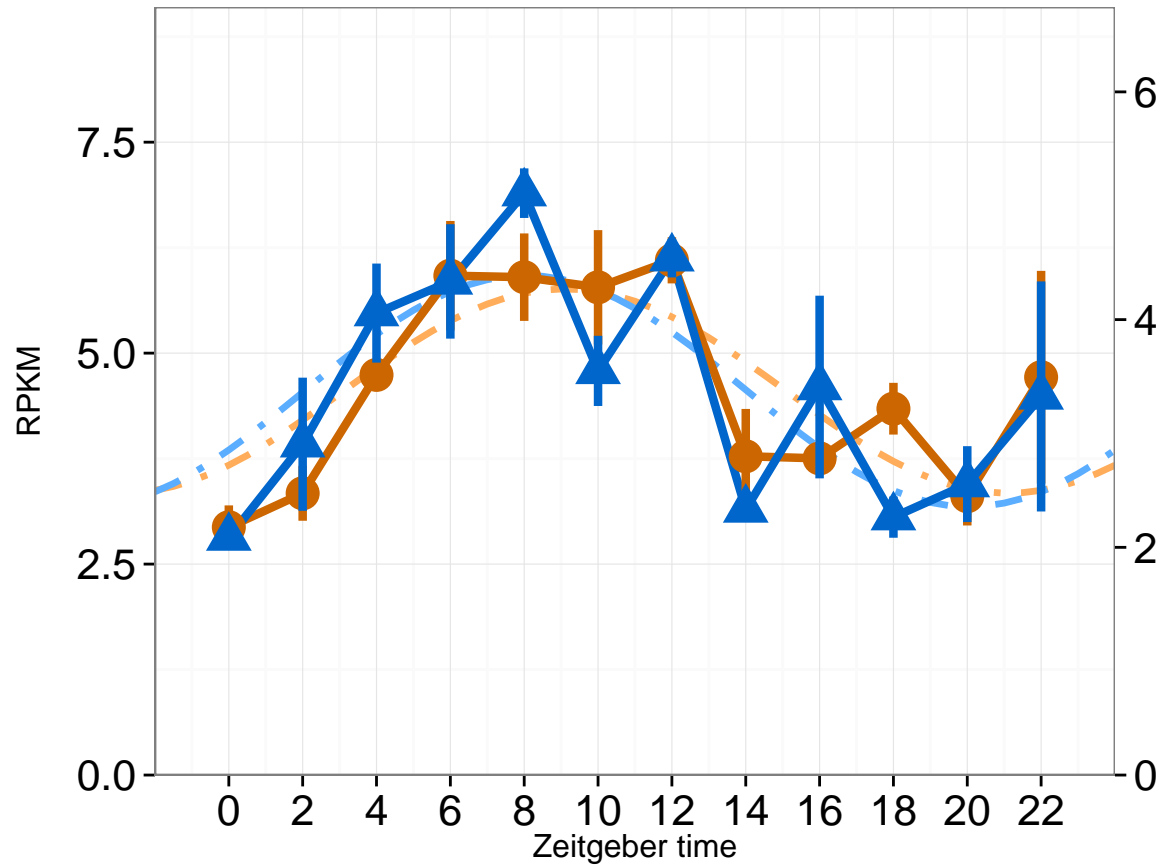

## Bmf

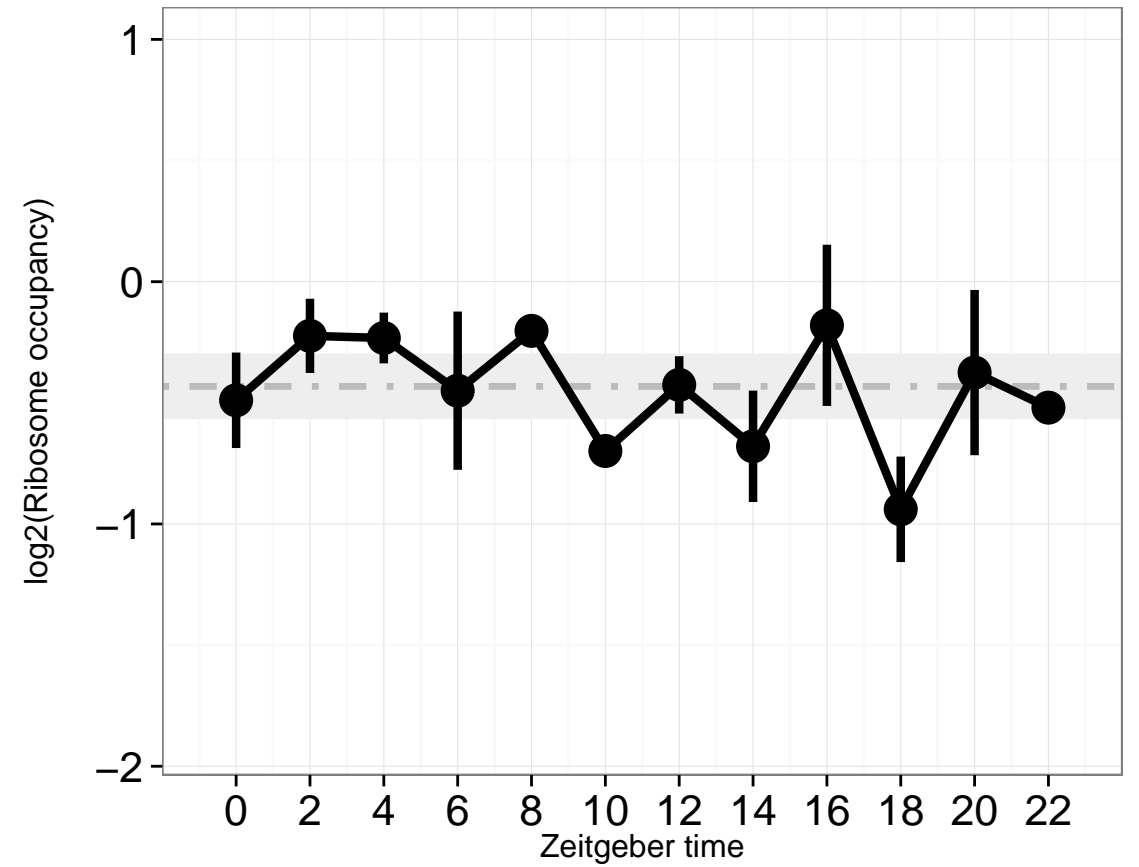

Supplement: Supplementary file 7 — Expression plots for kidney and liver for the 178 common rhythmic genes of Fig. 3c. (ZIP 3338.28 kb) [file 13059_2017_1222_MOESM7_ESM.zip › set_D_shared(178)/Bmf_kidney_set_D.pdf]

# Bmf

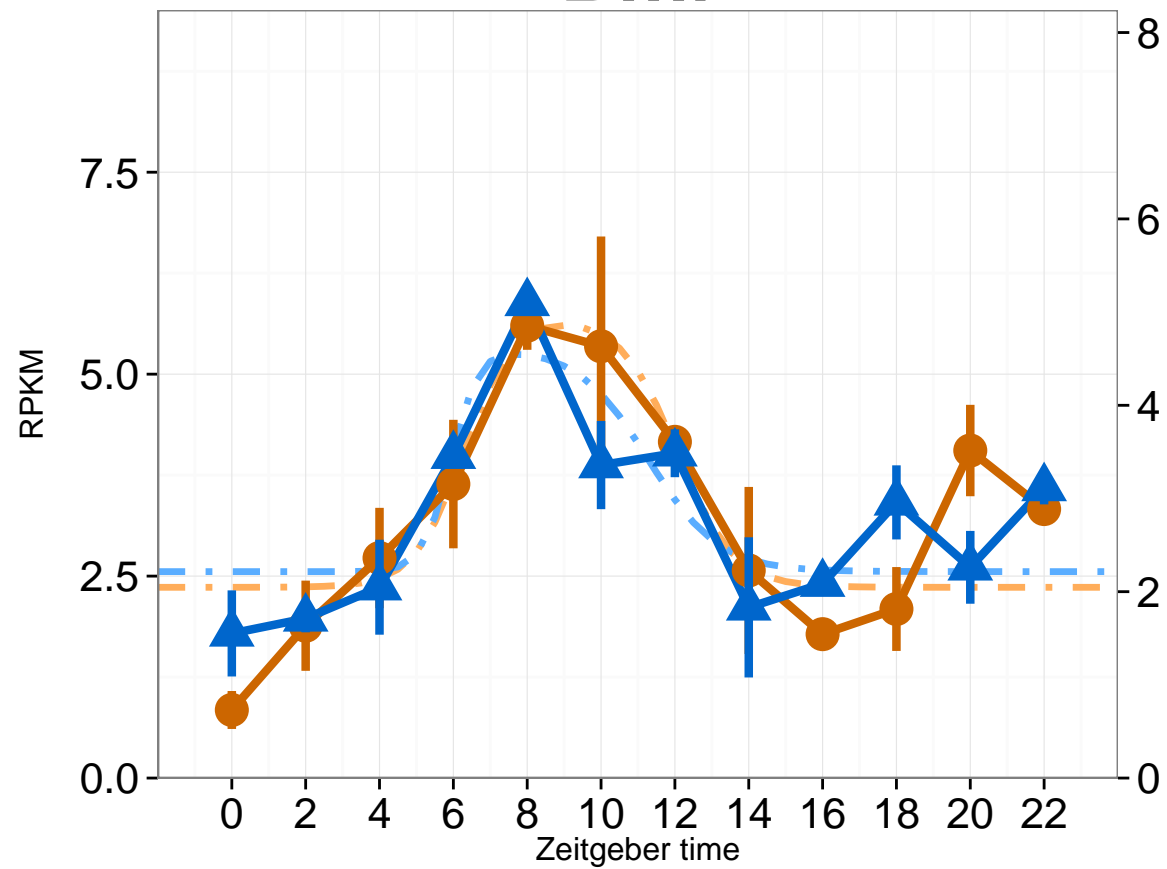

# Bmf

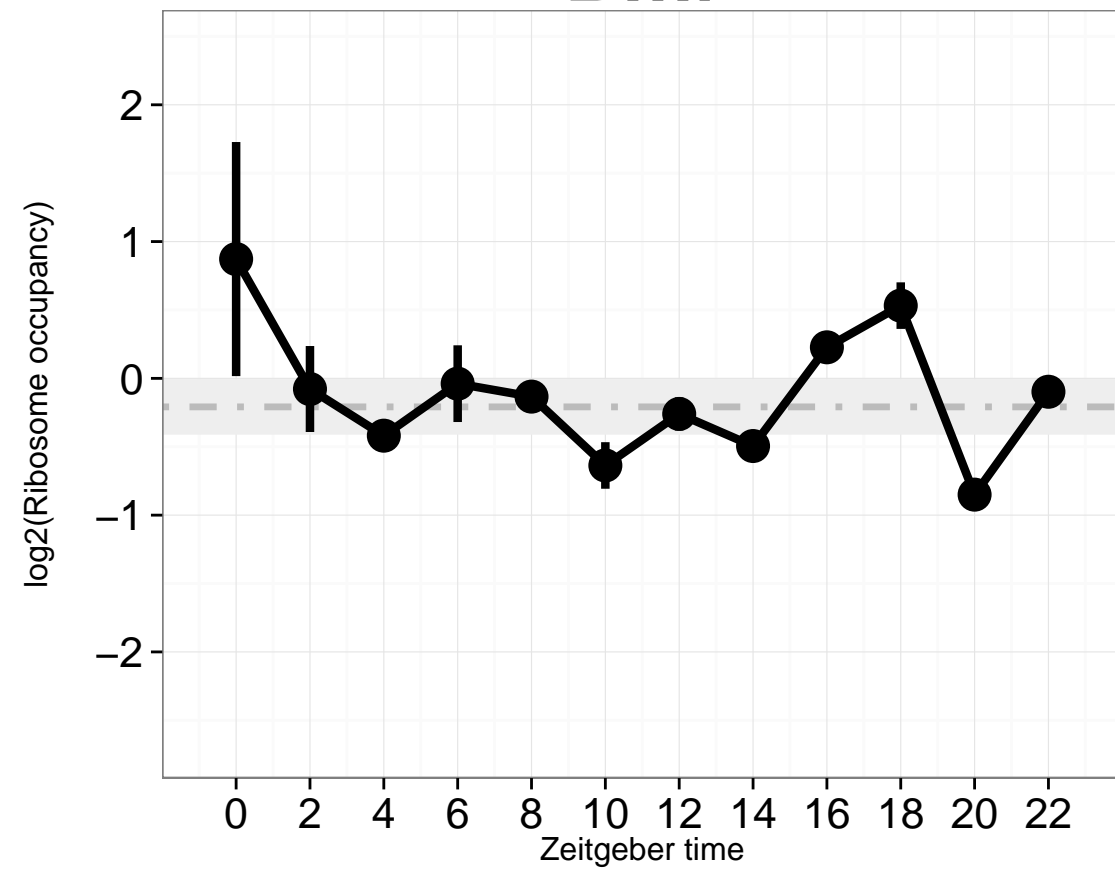

Supplement: Supplementary file 7 — Expression plots for kidney and liver for the 178 common rhythmic genes of Fig. 3c. (ZIP 3338.28 kb) [file 13059_2017_1222_MOESM7_ESM.zip › set_D_shared(178)/Bmf_liver_set_D.pdf]

# Brca1

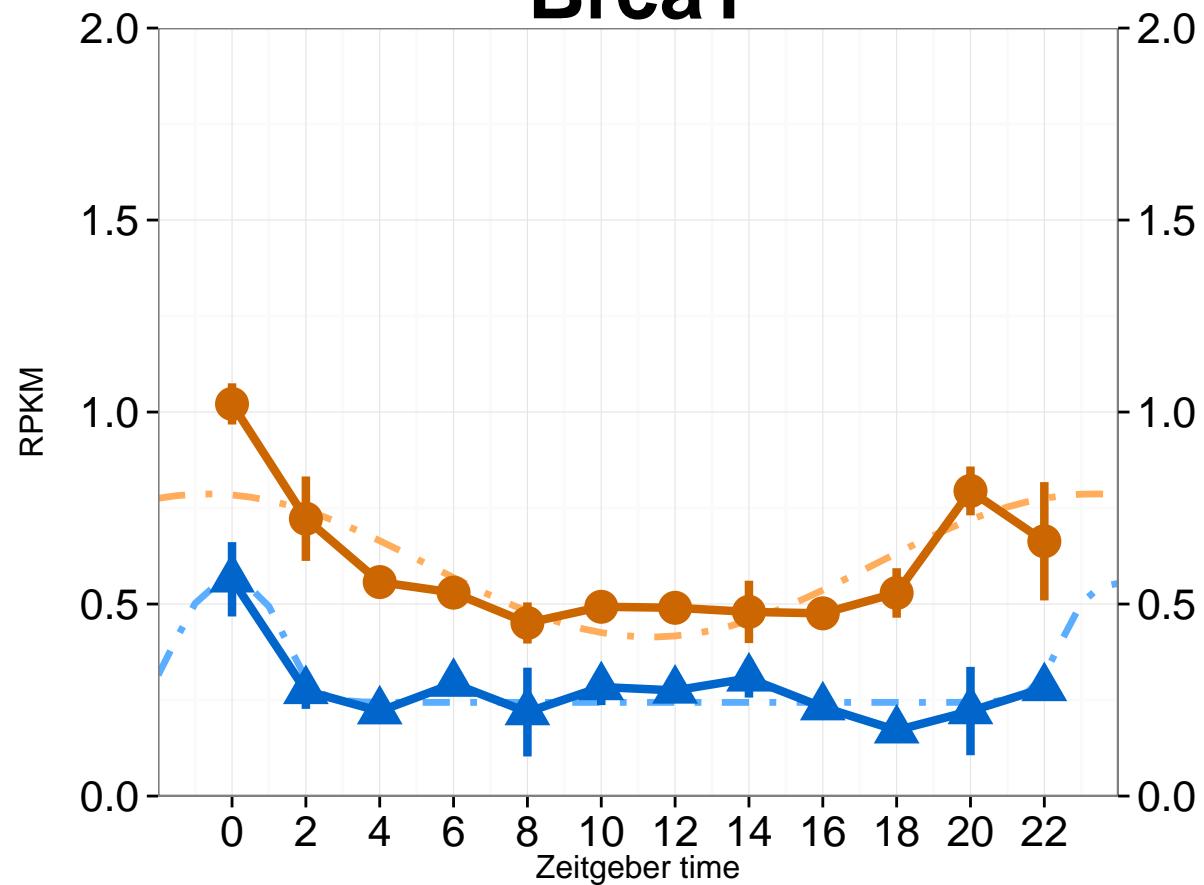

# Brca1

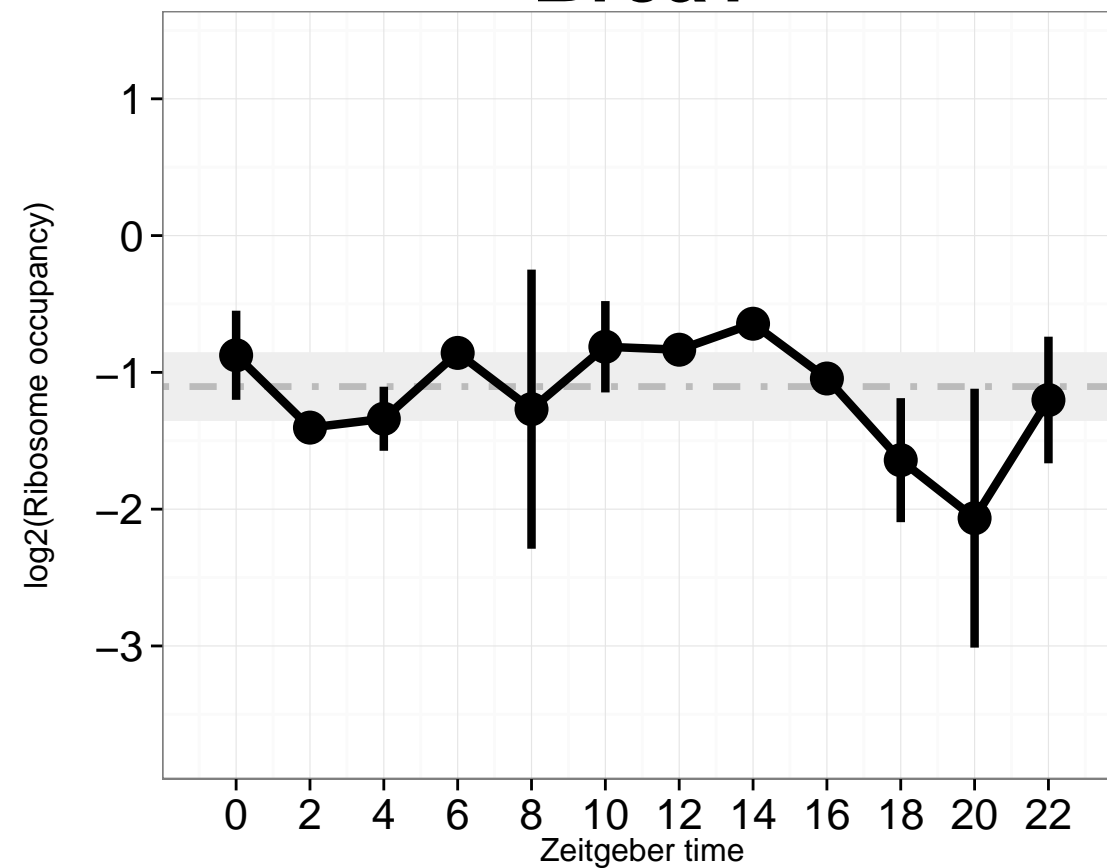

Supplement: Supplementary file 7 — Expression plots for kidney and liver for the 178 common rhythmic genes of Fig. 3c. (ZIP 3338.28 kb) [file 13059_2017_1222_MOESM7_ESM.zip › set_D_shared(178)/Brca1_kidney_set_D.pdf]

# Brca1

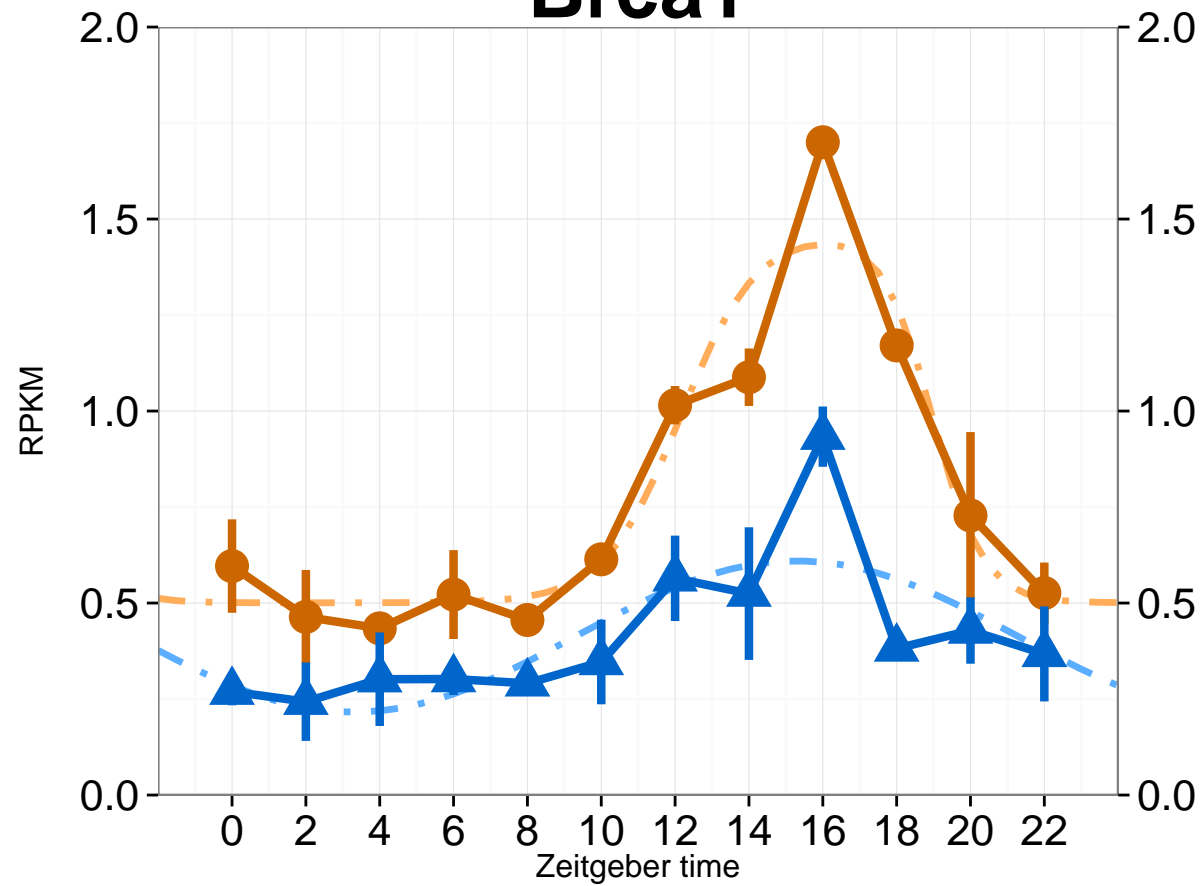

# Brca1

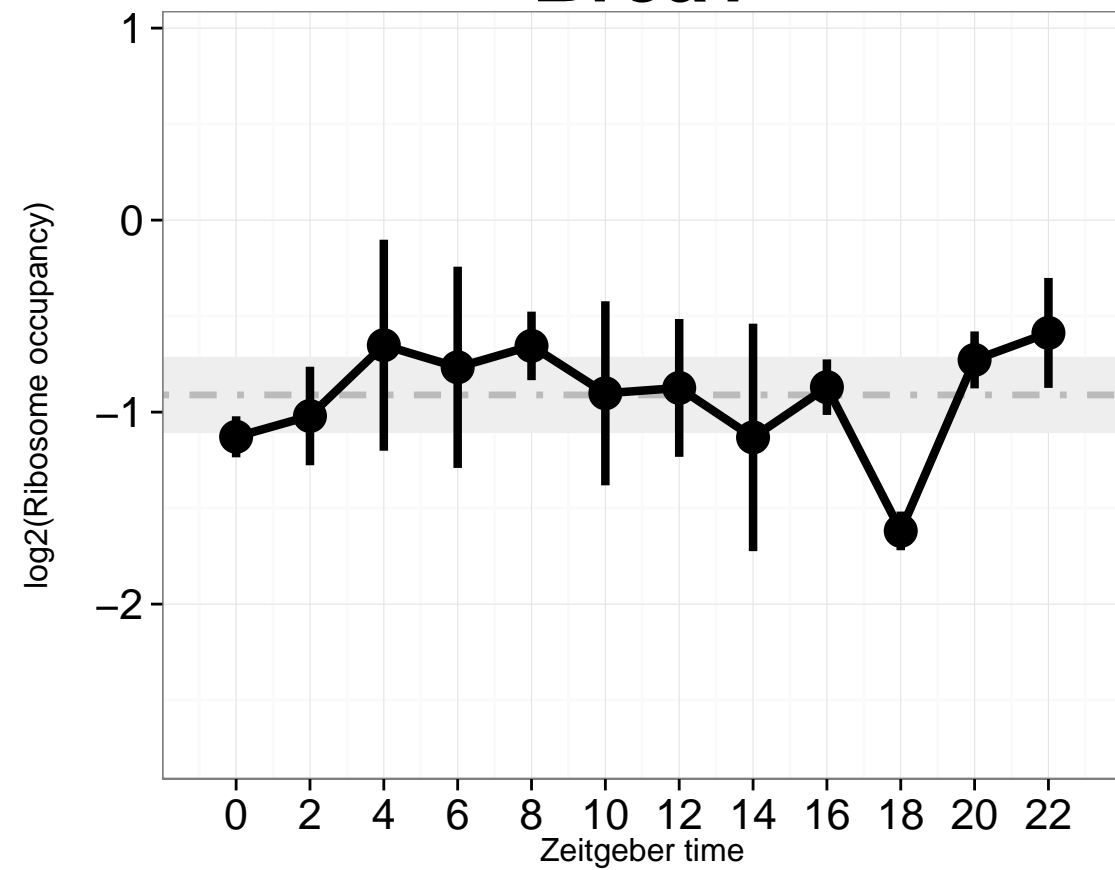

Supplement: Supplementary file 7 — Expression plots for kidney and liver for the 178 common rhythmic genes of Fig. 3c. (ZIP 3338.28 kb) [file 13059_2017_1222_MOESM7_ESM.zip › set_D_shared(178)/Brca1_liver_set_D.pdf]

# Car14

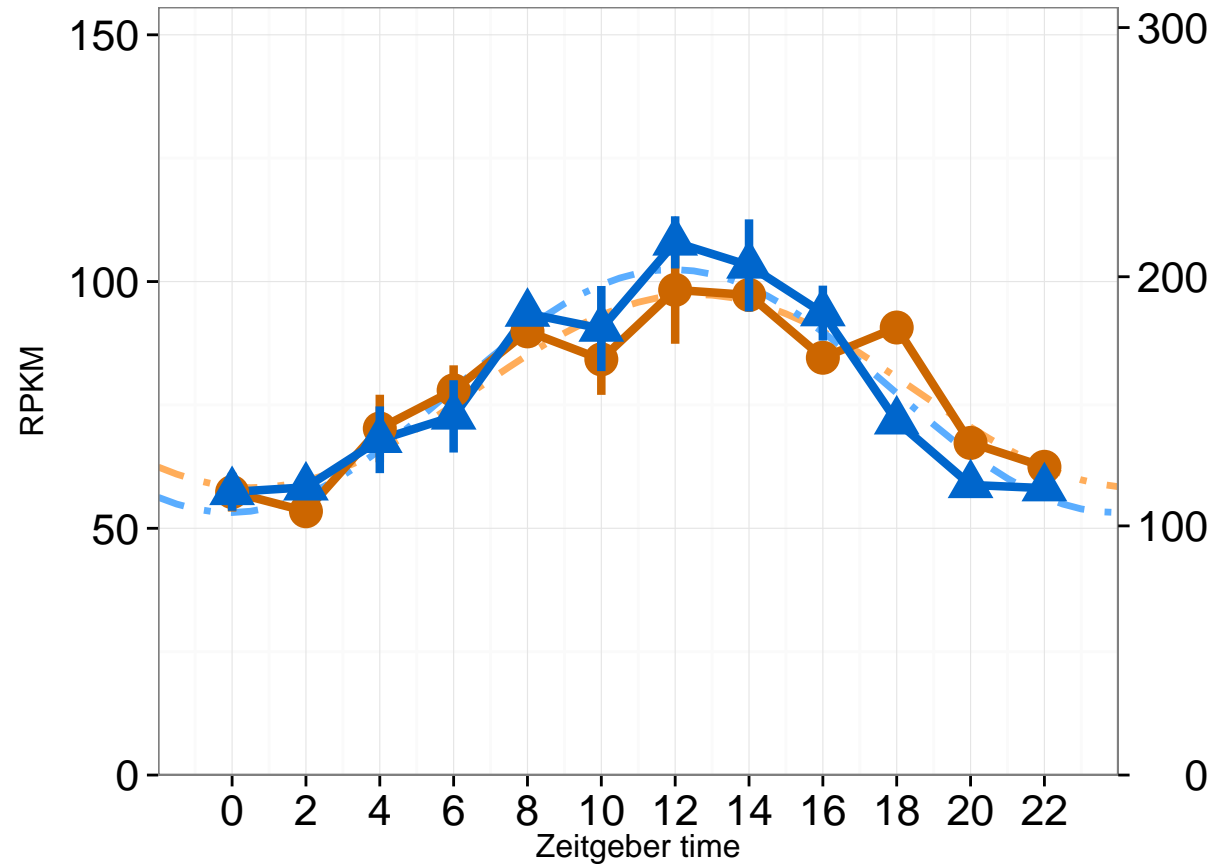

# Car14

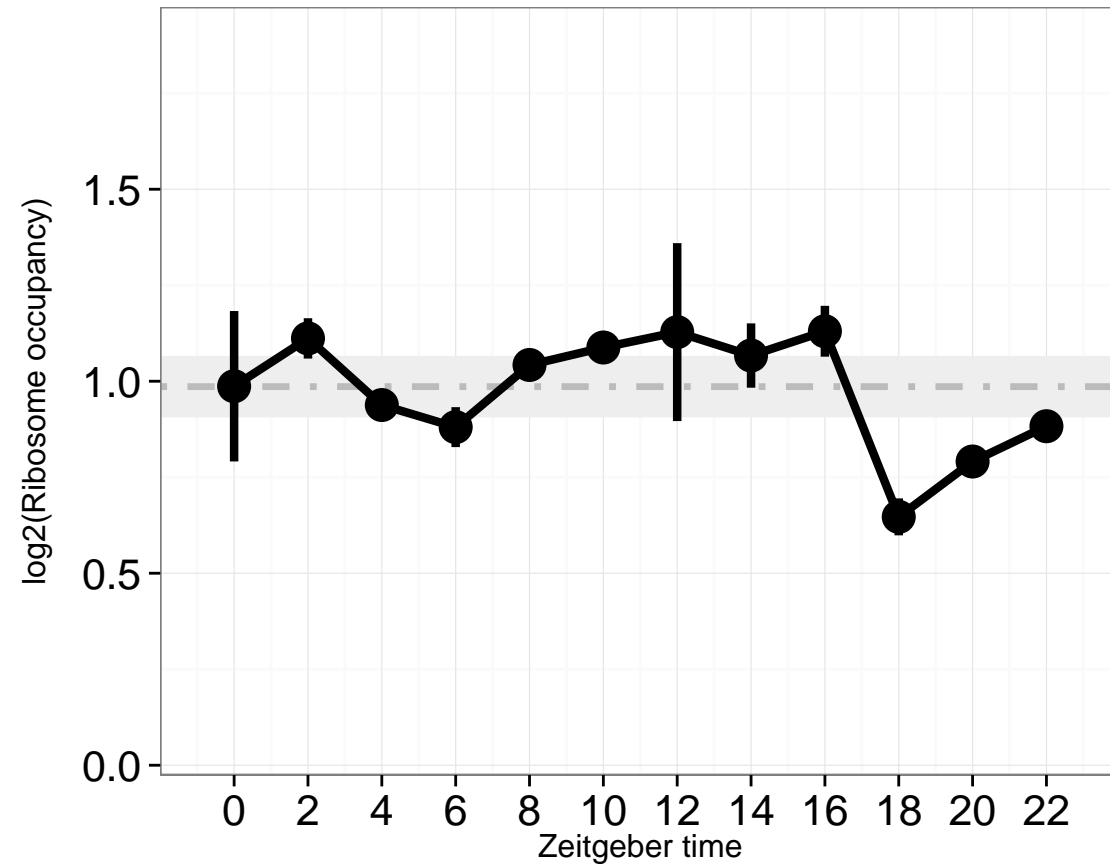

Supplement: Supplementary file 7 — Expression plots for kidney and liver for the 178 common rhythmic genes of Fig. 3c. (ZIP 3338.28 kb) [file 13059_2017_1222_MOESM7_ESM.zip › set_D_shared(178)/Car14_kidney_set_D.pdf]

## Car14

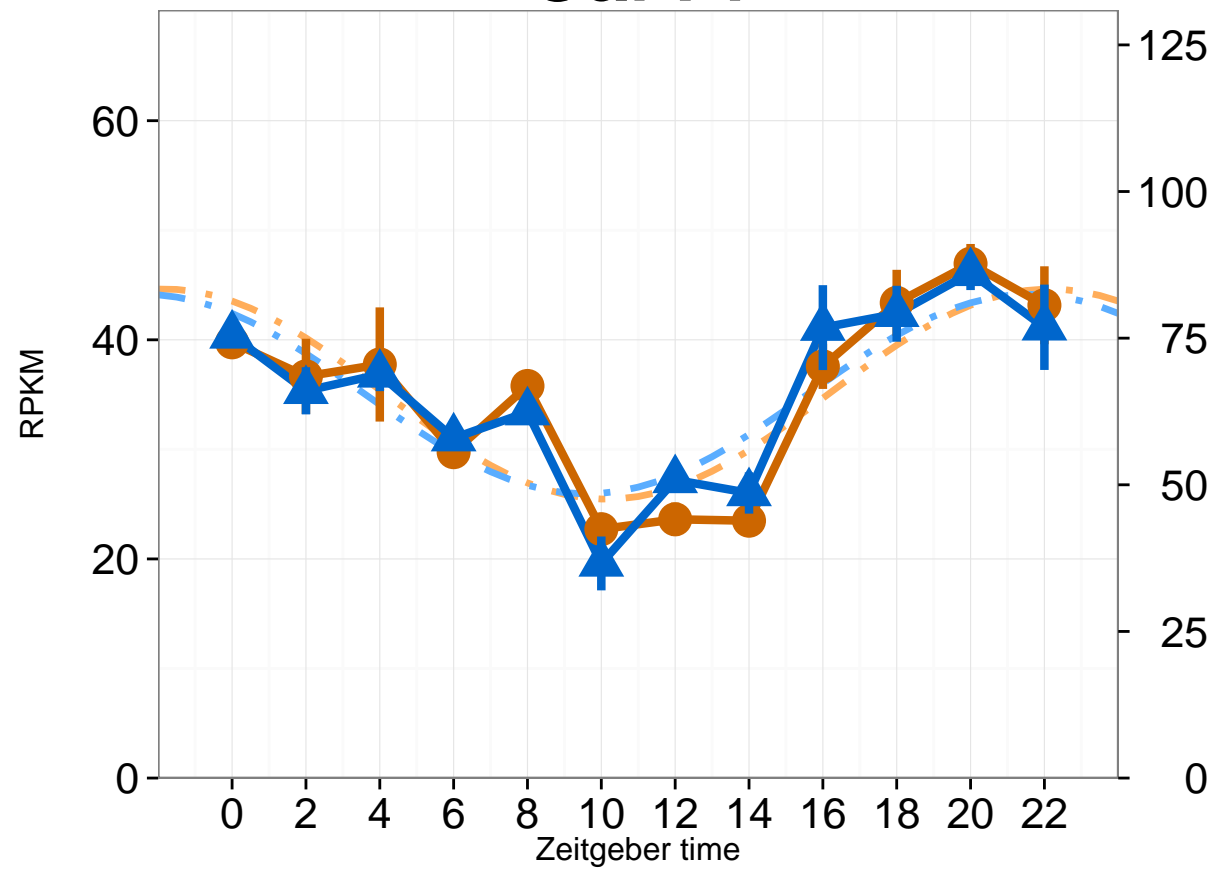

## Car14

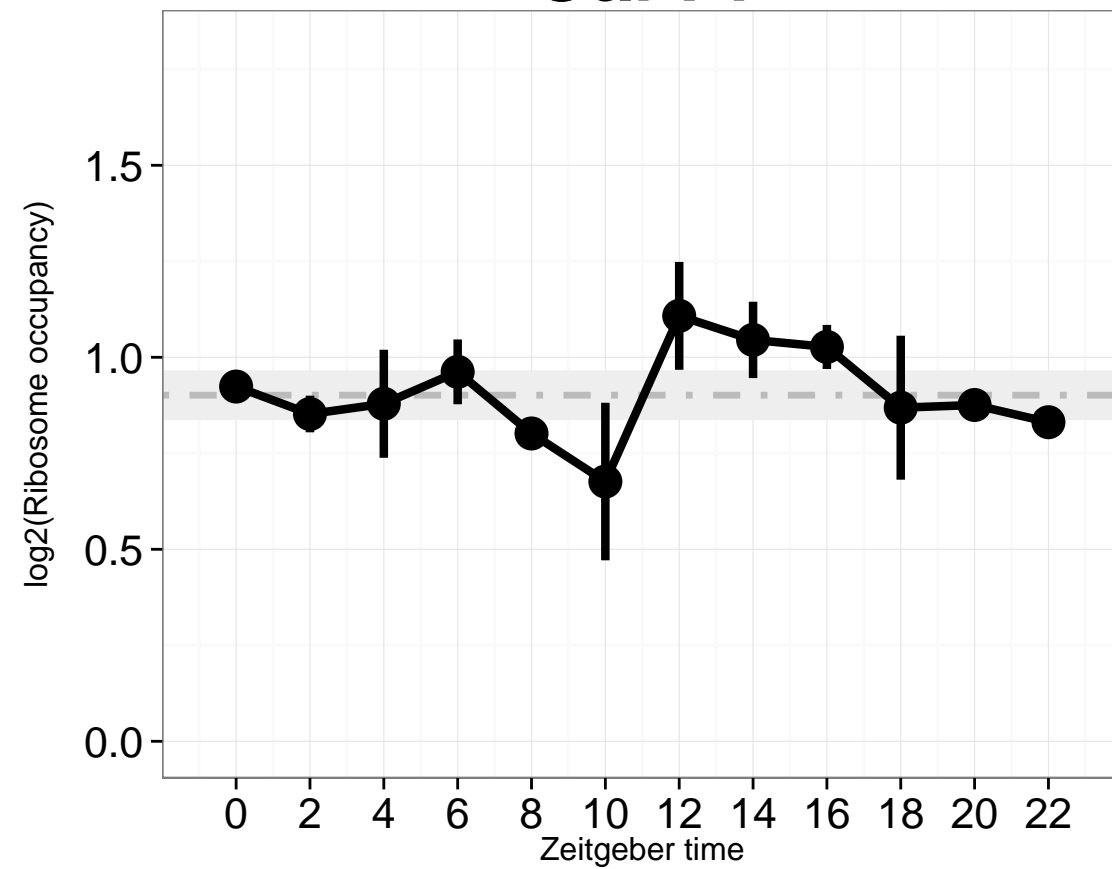

Supplement: Supplementary file 7 — Expression plots for kidney and liver for the 178 common rhythmic genes of Fig. 3c. (ZIP 3338.28 kb) [file 13059_2017_1222_MOESM7_ESM.zip › set_D_shared(178)/Car14_liver_set_D.pdf]

# Ccdc151

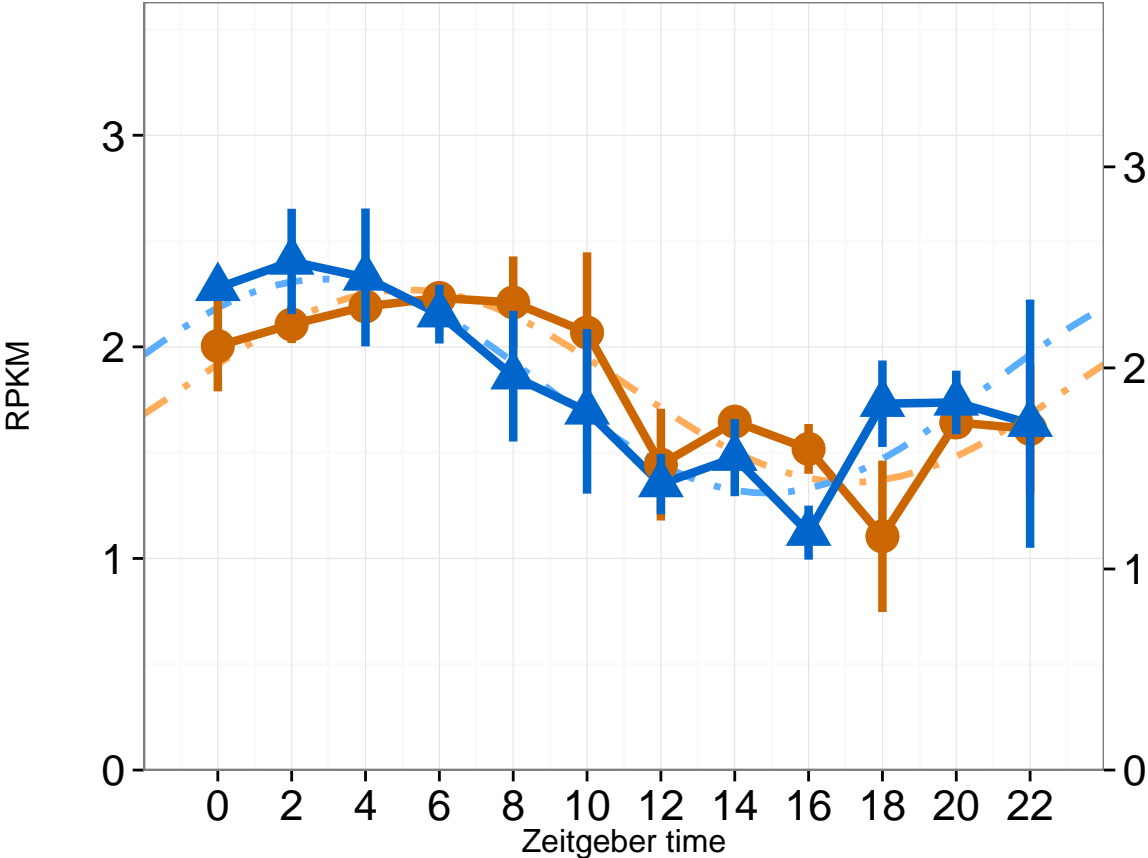

# Ccdc151

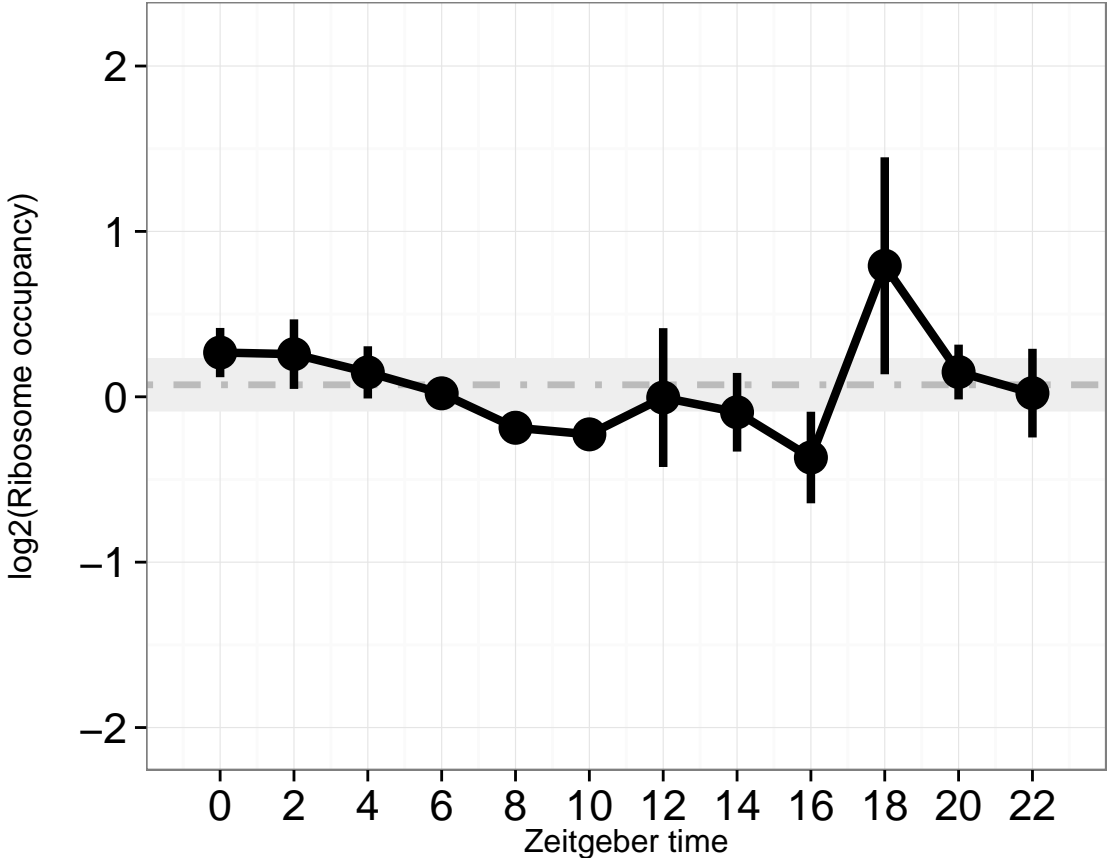

Supplement: Supplementary file 7 — Expression plots for kidney and liver for the 178 common rhythmic genes of Fig. 3c. (ZIP 3338.28 kb) [file 13059_2017_1222_MOESM7_ESM.zip › set_D_shared(178)/Ccdc151_kidney_set_D.pdf]

# Ccdc151

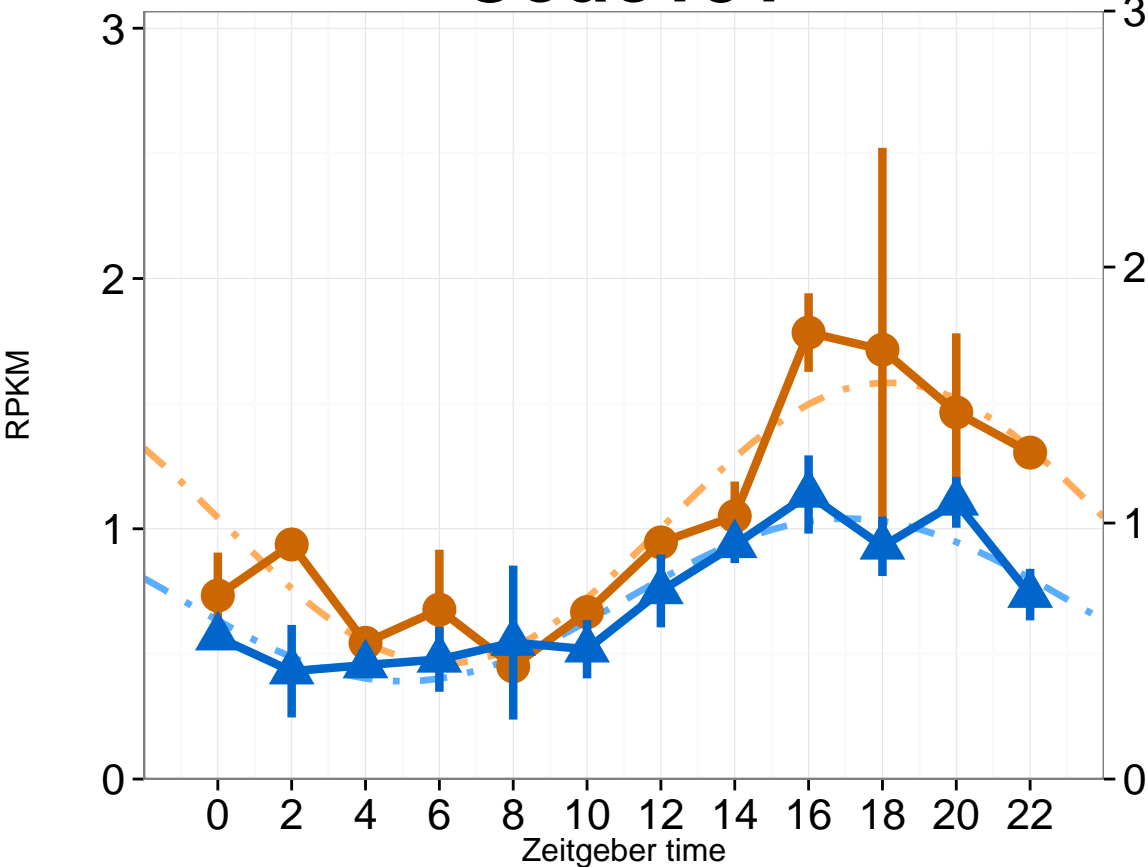

# Ccdc151

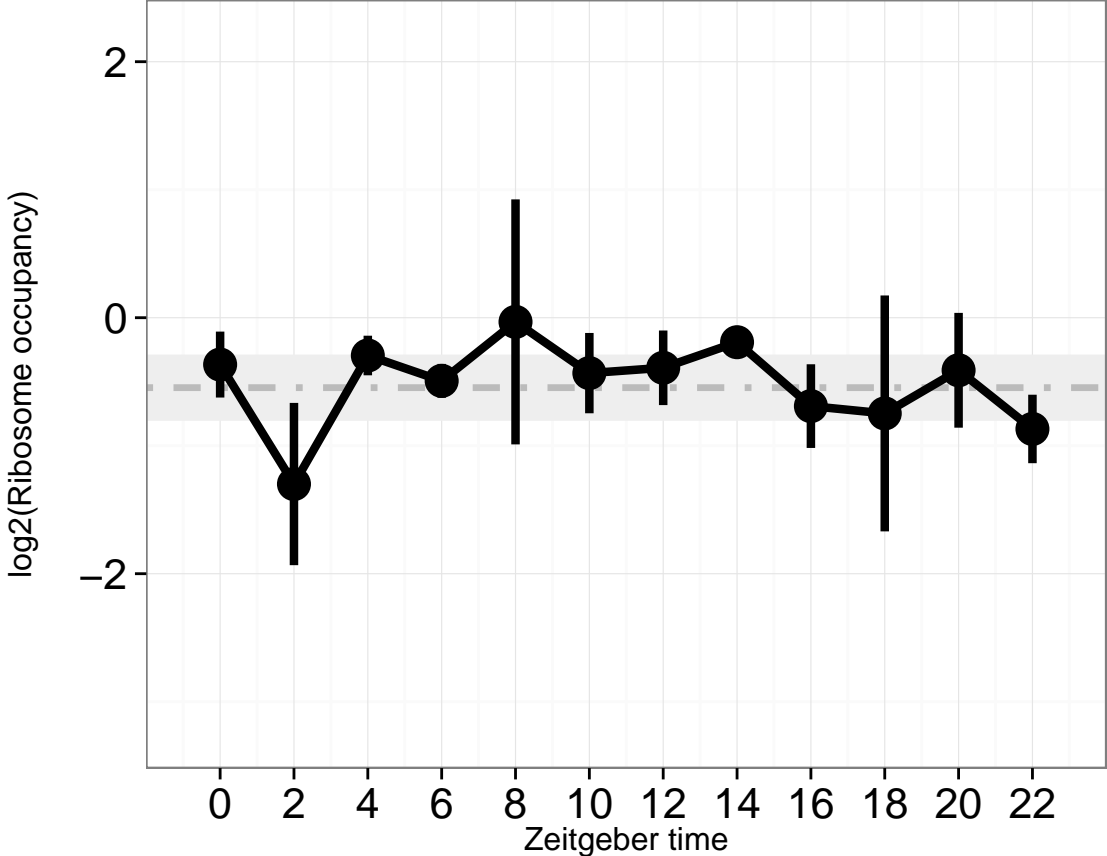

Supplement: Supplementary file 7 — Expression plots for kidney and liver for the 178 common rhythmic genes of Fig. 3c. (ZIP 3338.28 kb) [file 13059_2017_1222_MOESM7_ESM.zip › set_D_shared(178)/Ccdc151_liver_set_D.pdf]

## Cdk20

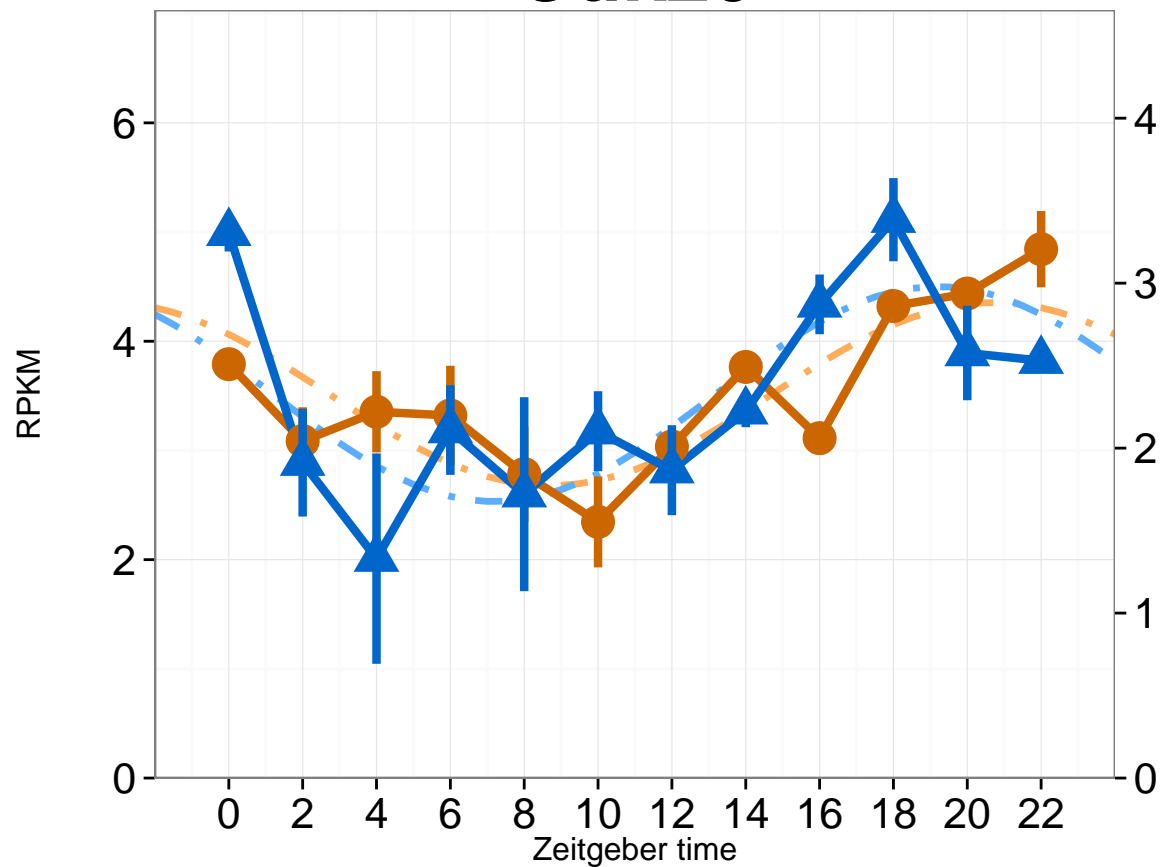

## Cdk20

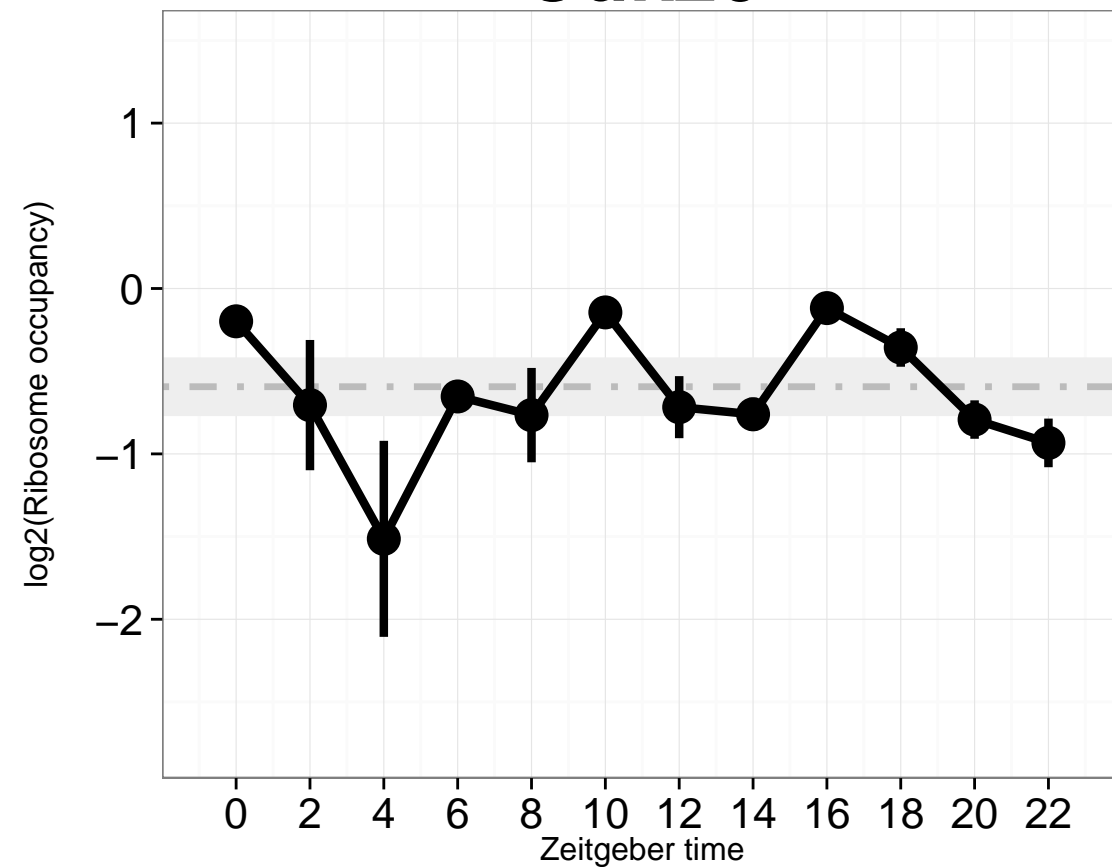

Supplement: Supplementary file 7 — Expression plots for kidney and liver for the 178 common rhythmic genes of Fig. 3c. (ZIP 3338.28 kb) [file 13059_2017_1222_MOESM7_ESM.zip › set_D_shared(178)/Cdk20_kidney_set_D.pdf]

## Cdk20

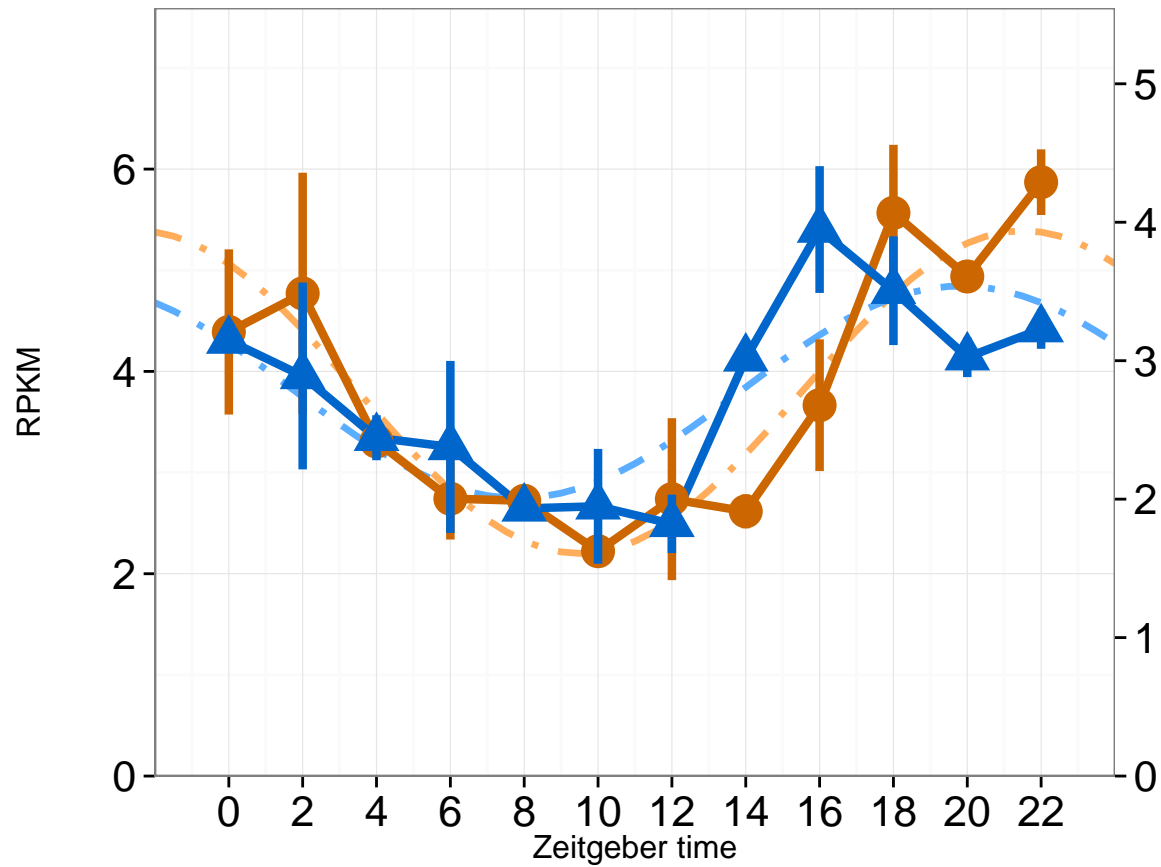

## Cdk20

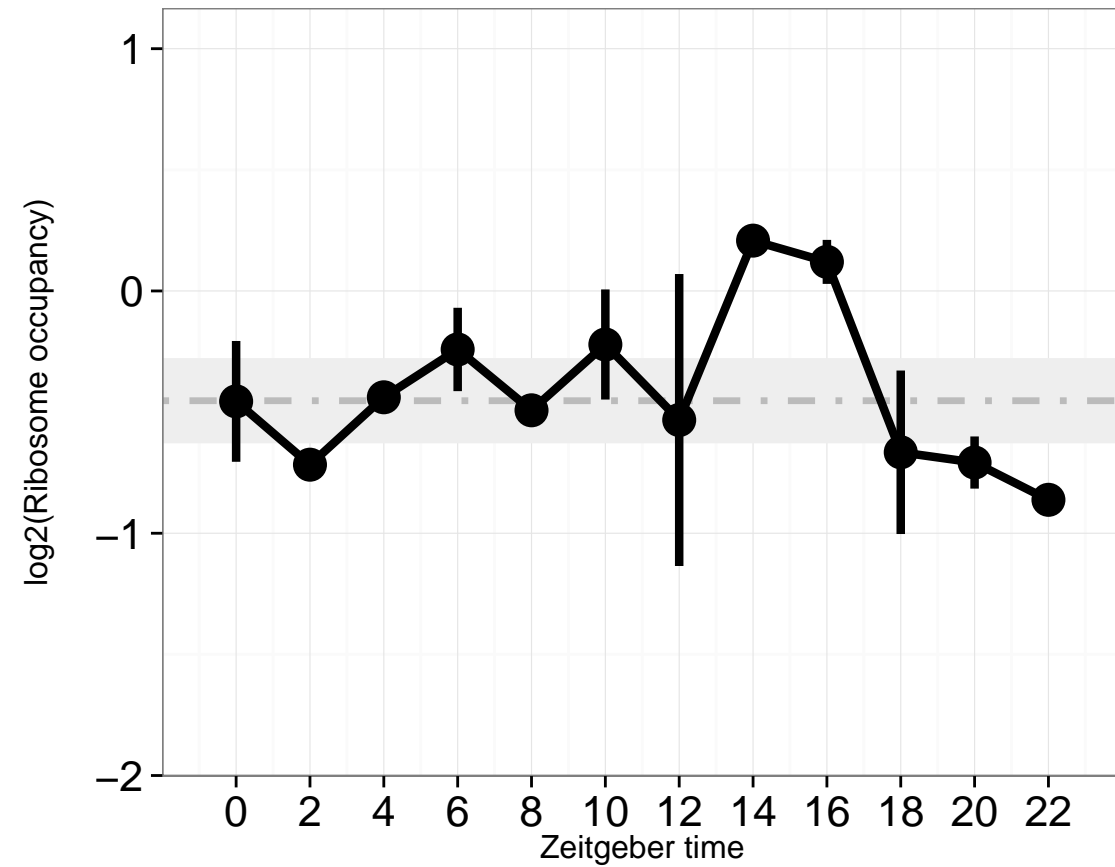

Supplement: Supplementary file 7 — Expression plots for kidney and liver for the 178 common rhythmic genes of Fig. 3c. (ZIP 3338.28 kb) [file 13059_2017_1222_MOESM7_ESM.zip › set_D_shared(178)/Cdk20_liver_set_D.pdf]

# Cdkn1a

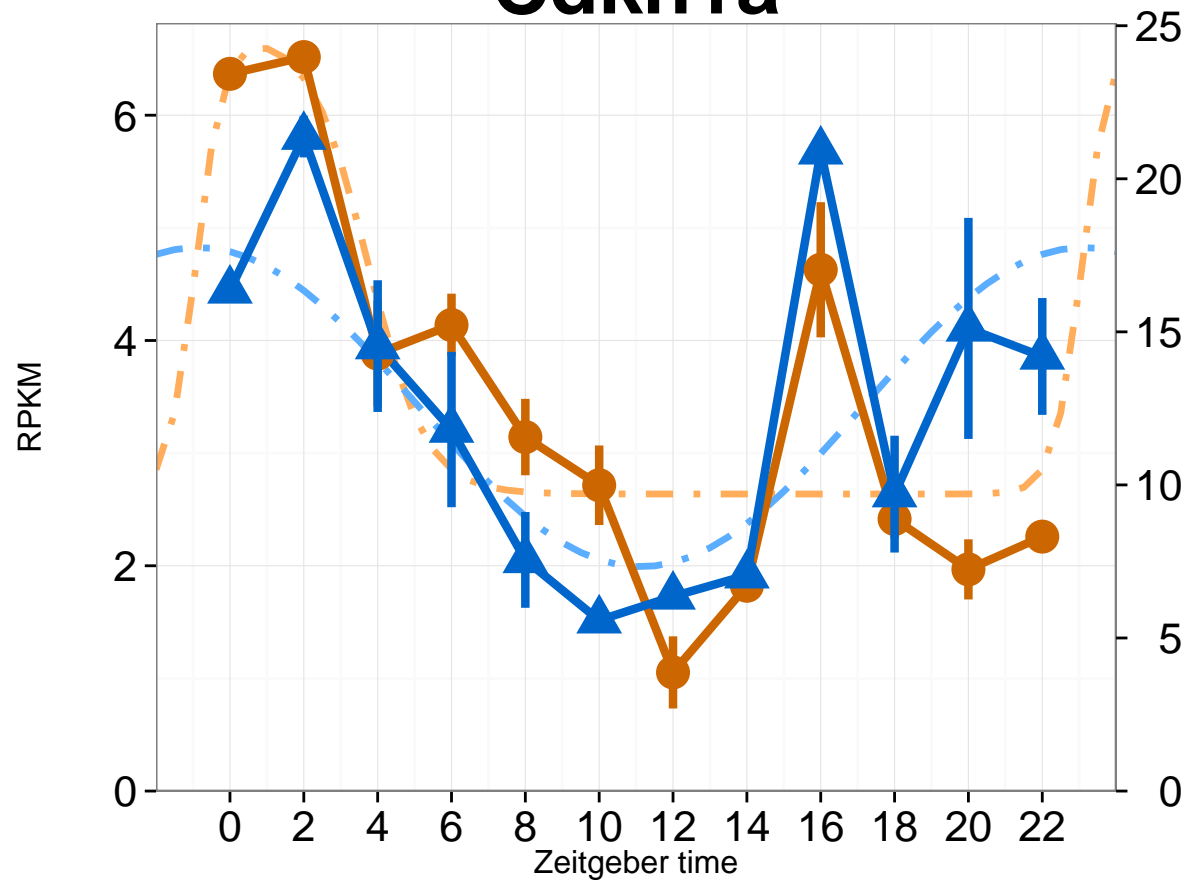

# Cdkn1a

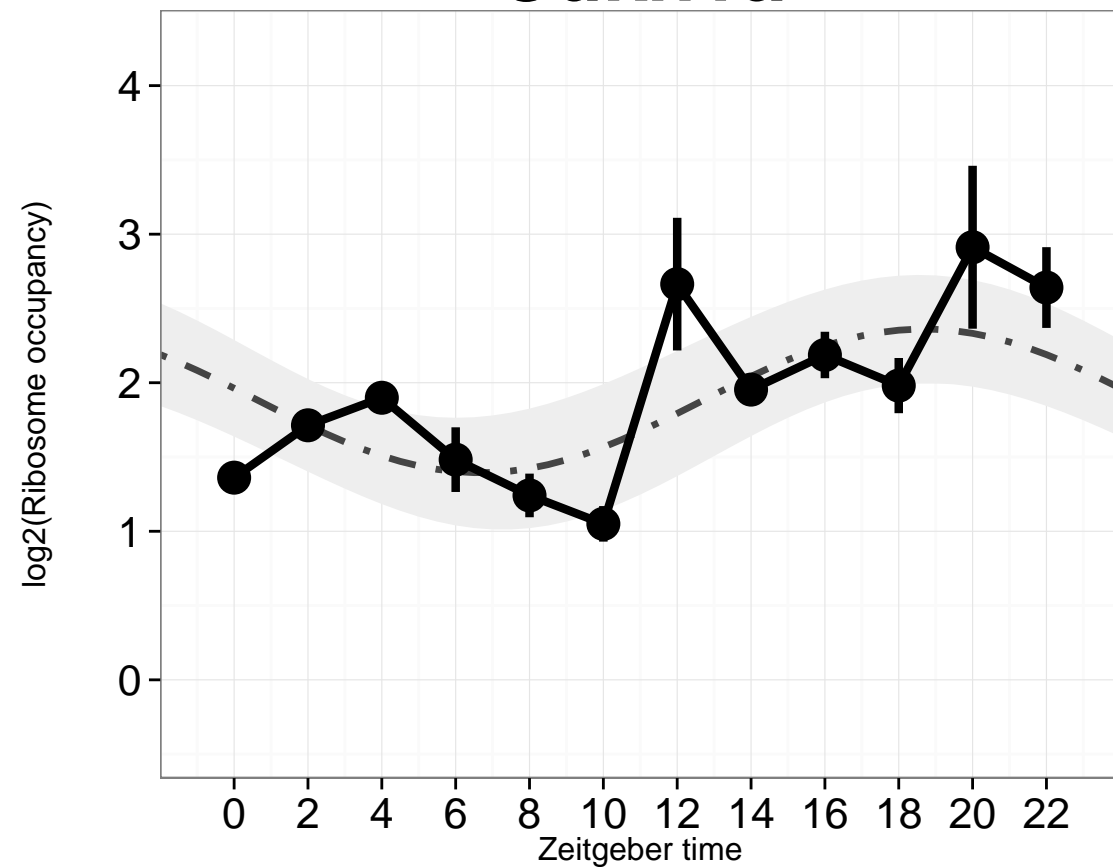

Supplement: Supplementary file 7 — Expression plots for kidney and liver for the 178 common rhythmic genes of Fig. 3c. (ZIP 3338.28 kb) [file 13059_2017_1222_MOESM7_ESM.zip › set_D_shared(178)/Cdkn1a_kidney_set_D.pdf]

# Cdkn1a

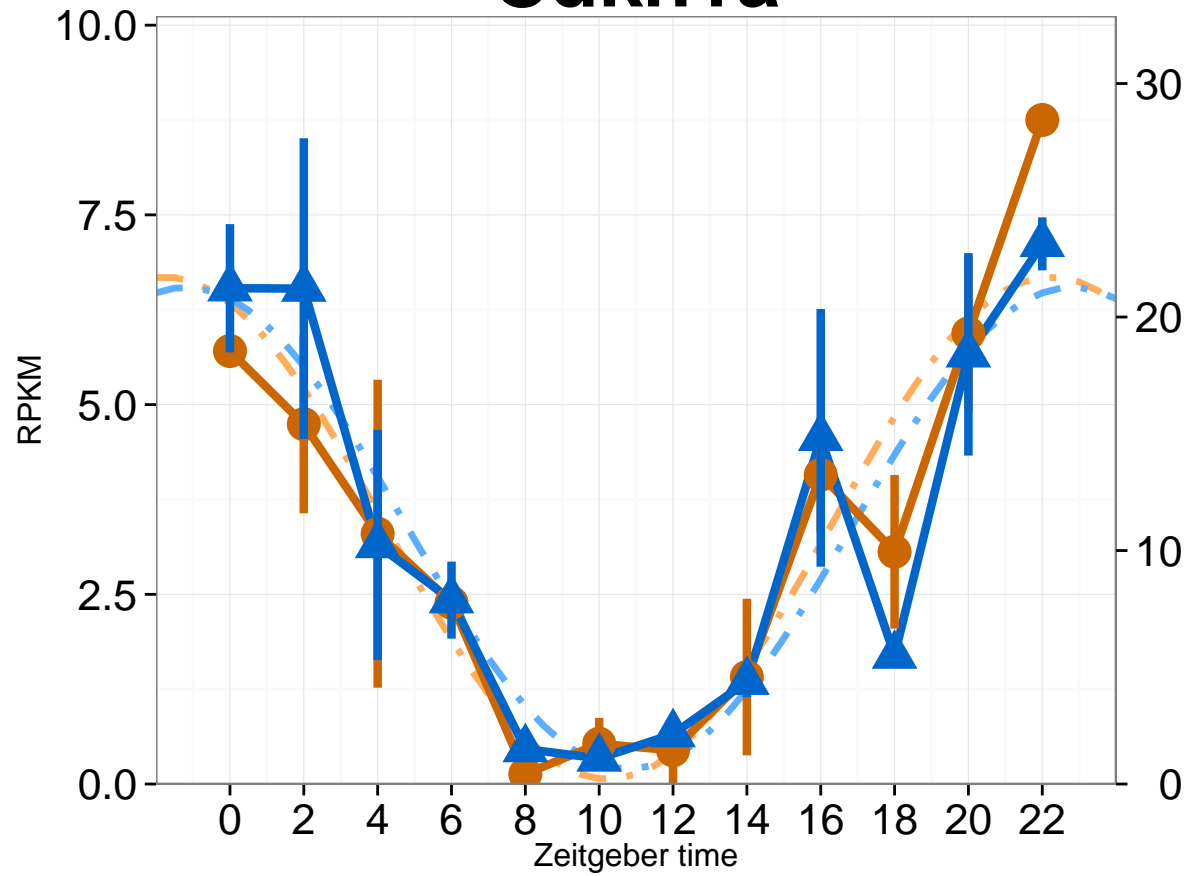

# Cdkn1a

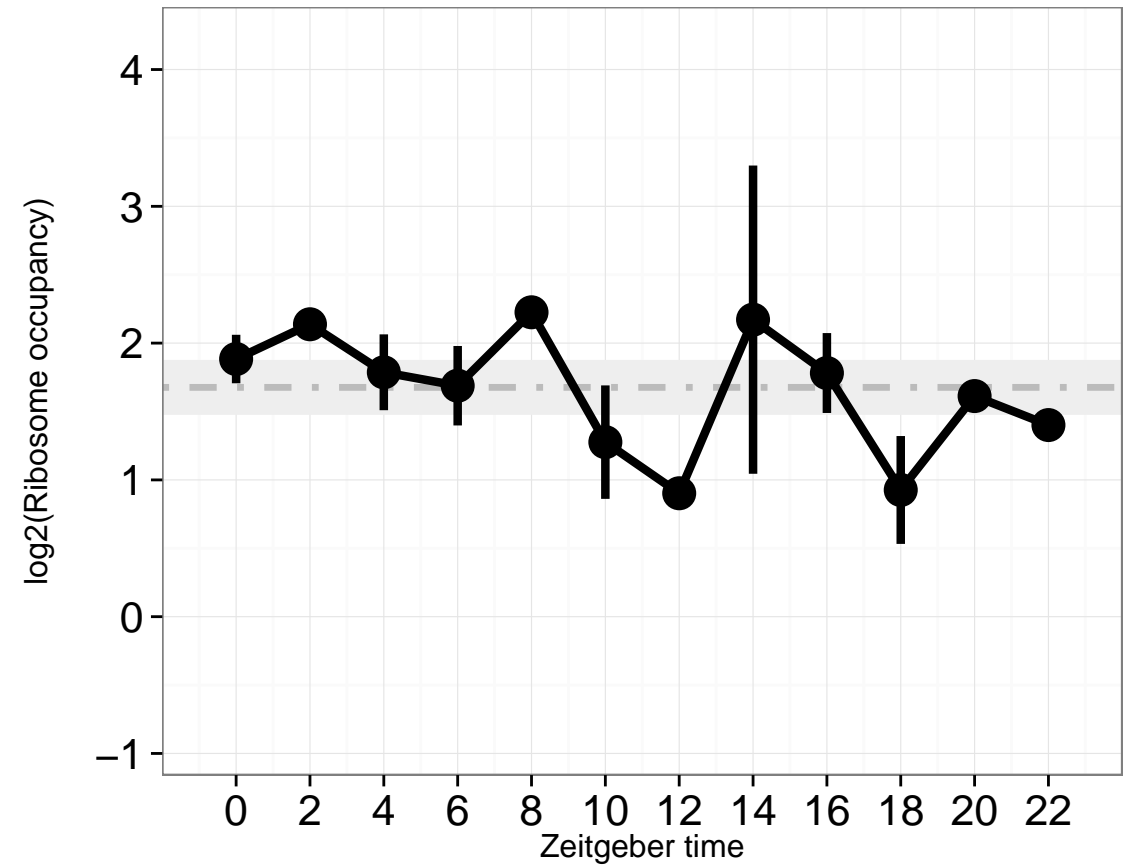

Supplement: Supplementary file 7 — Expression plots for kidney and liver for the 178 common rhythmic genes of Fig. 3c. (ZIP 3338.28 kb) [file 13059_2017_1222_MOESM7_ESM.zip › set_D_shared(178)/Cdkn1a_liver_set_D.pdf]

# Ces1d

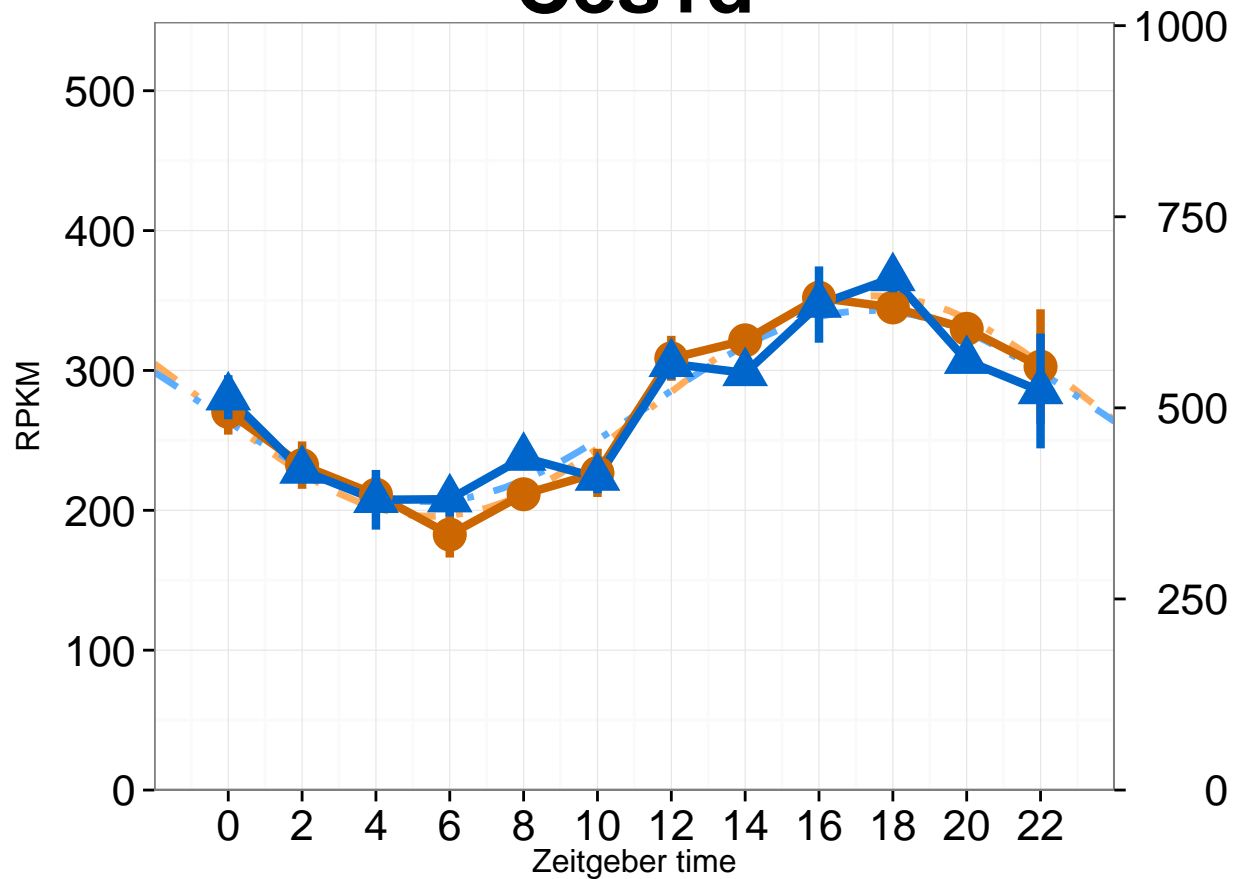

# Ces1d

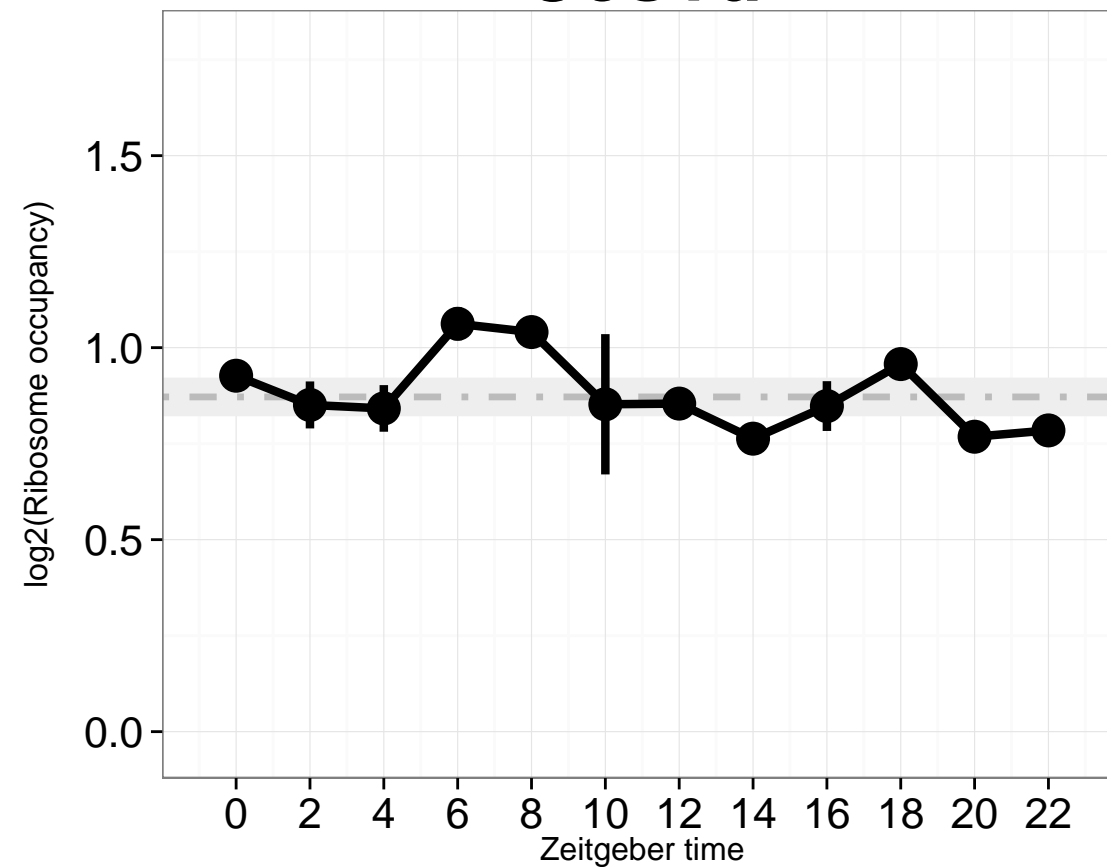

Supplement: Supplementary file 7 — Expression plots for kidney and liver for the 178 common rhythmic genes of Fig. 3c. (ZIP 3338.28 kb) [file 13059_2017_1222_MOESM7_ESM.zip › set_D_shared(178)/Ces1d_kidney_set_D.pdf]

## Ces1d

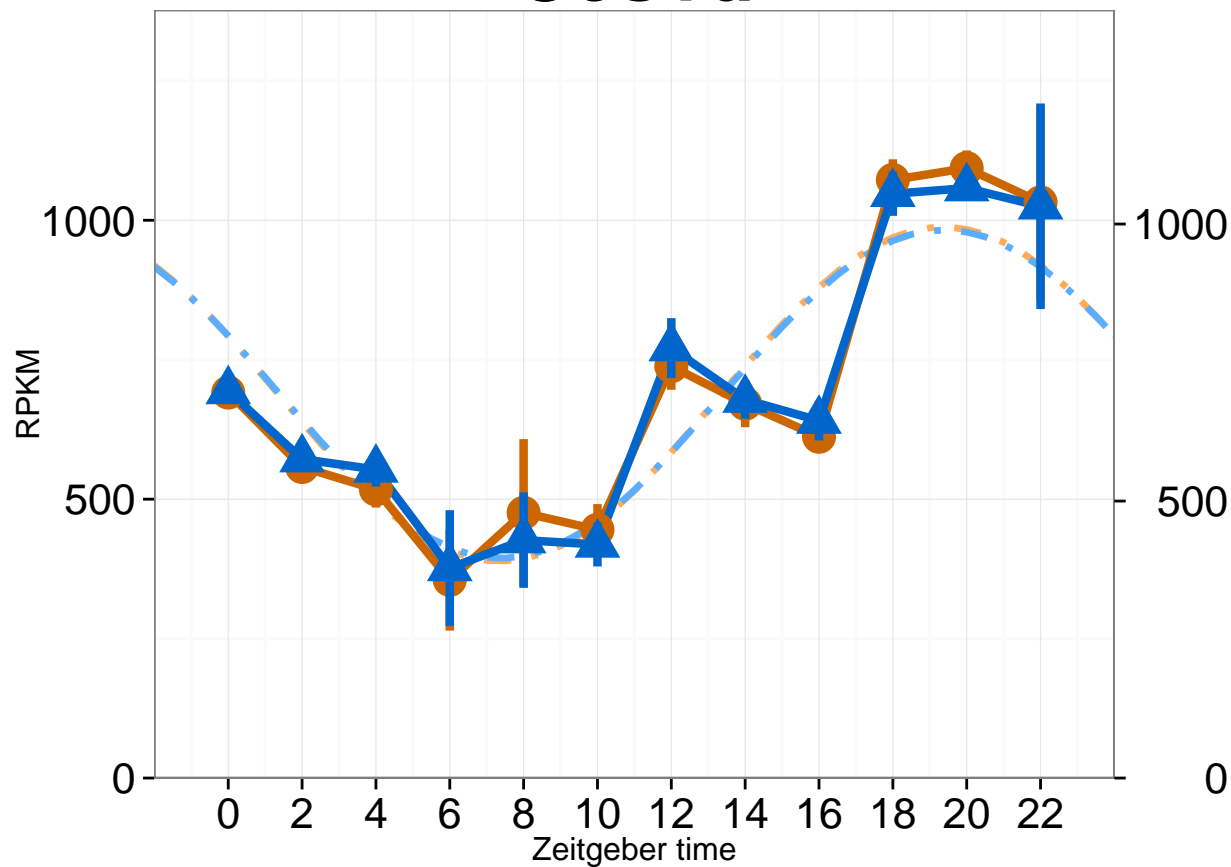

## Ces1d

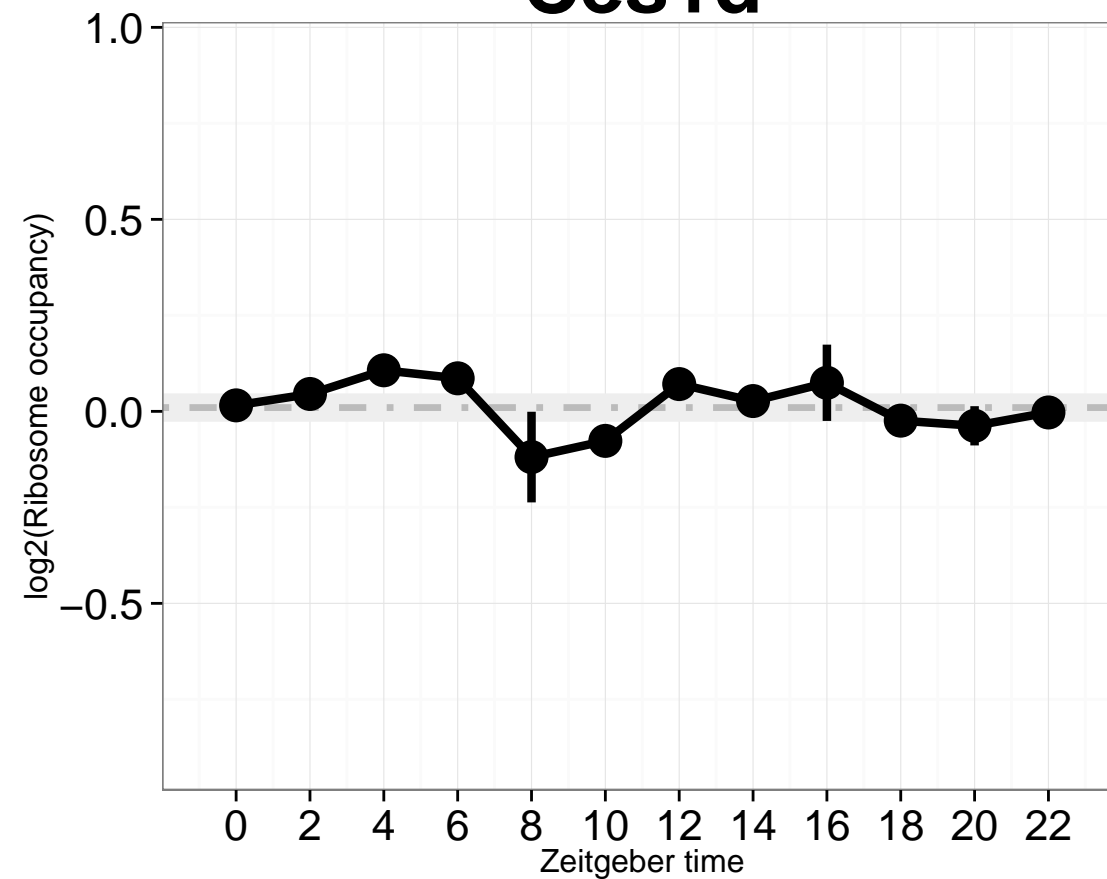

Supplement: Supplementary file 7 — Expression plots for kidney and liver for the 178 common rhythmic genes of Fig. 3c. (ZIP 3338.28 kb) [file 13059_2017_1222_MOESM7_ESM.zip › set_D_shared(178)/Ces1d_liver_set_D.pdf]

## Cgn

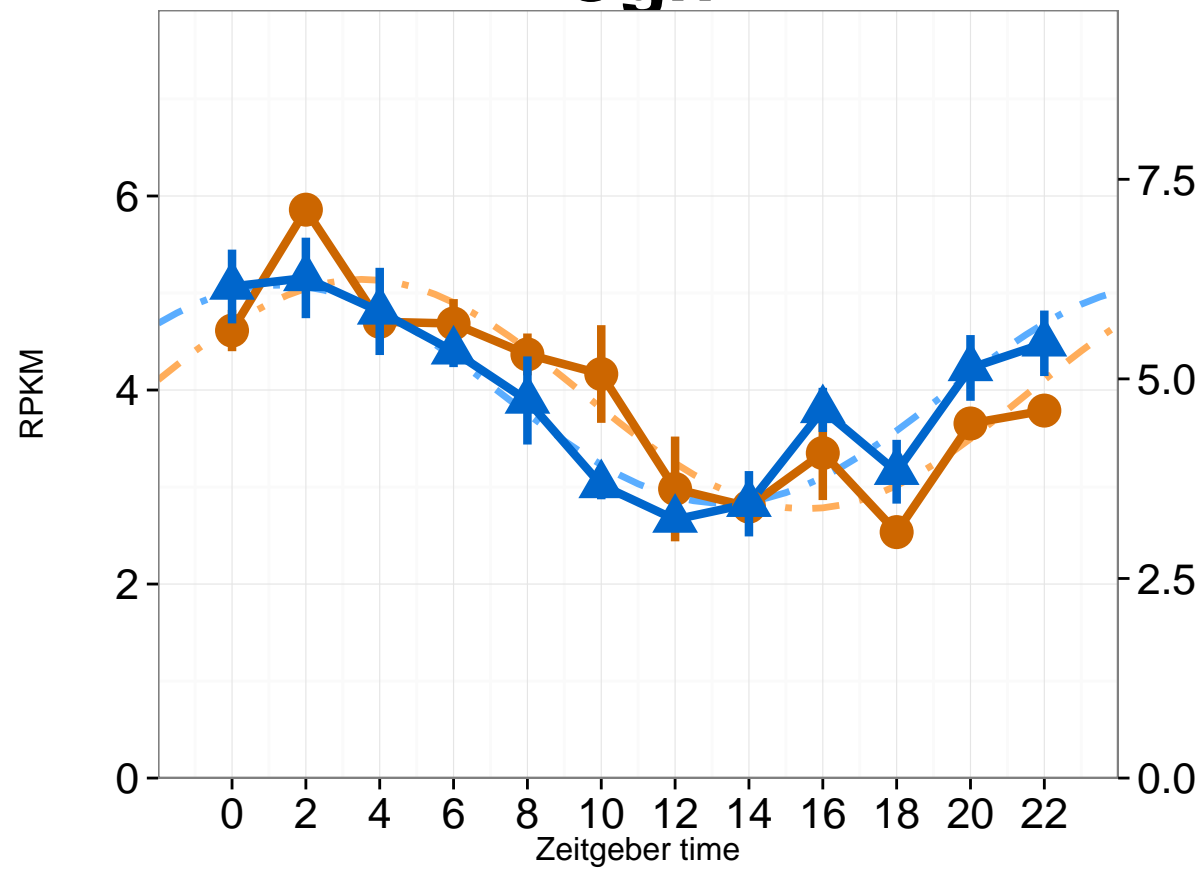

## Cgn

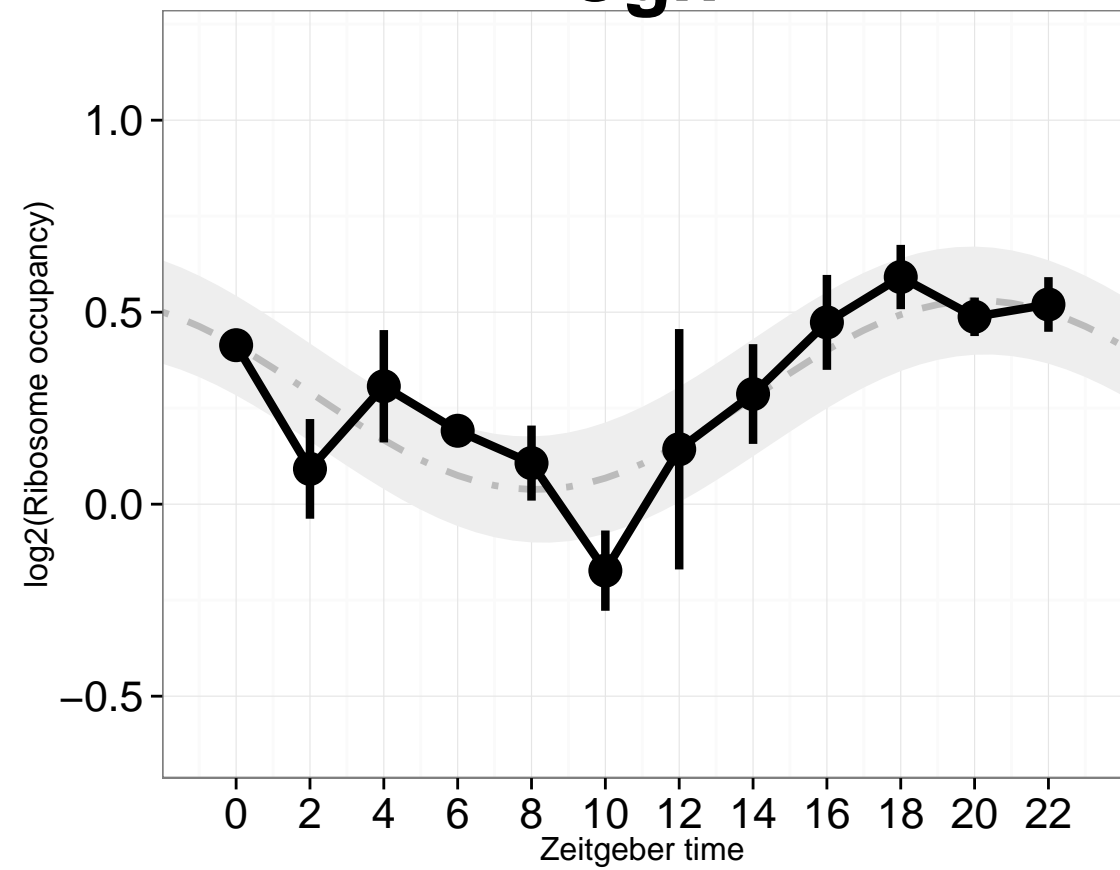

Supplement: Supplementary file 7 — Expression plots for kidney and liver for the 178 common rhythmic genes of Fig. 3c. (ZIP 3338.28 kb) [file 13059_2017_1222_MOESM7_ESM.zip › set_D_shared(178)/Cgn_kidney_set_D.pdf]

## Cgn

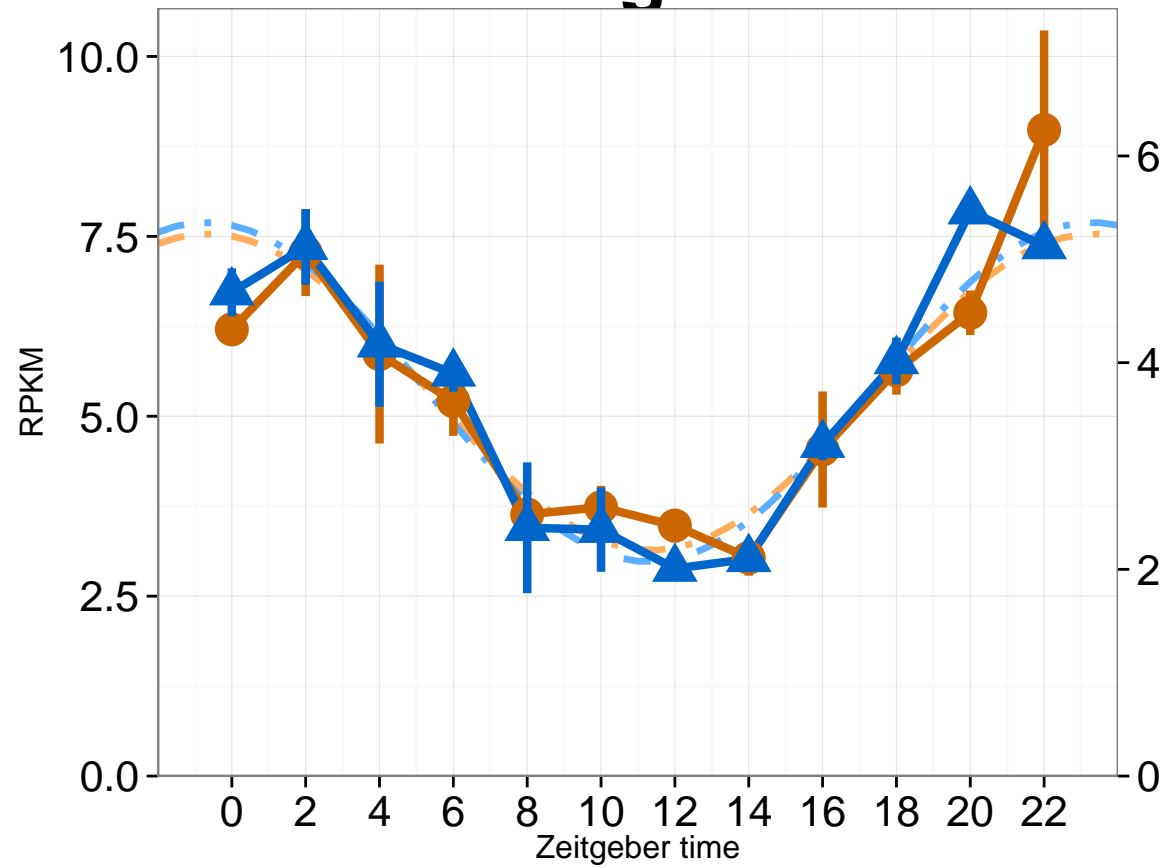

## Cgn

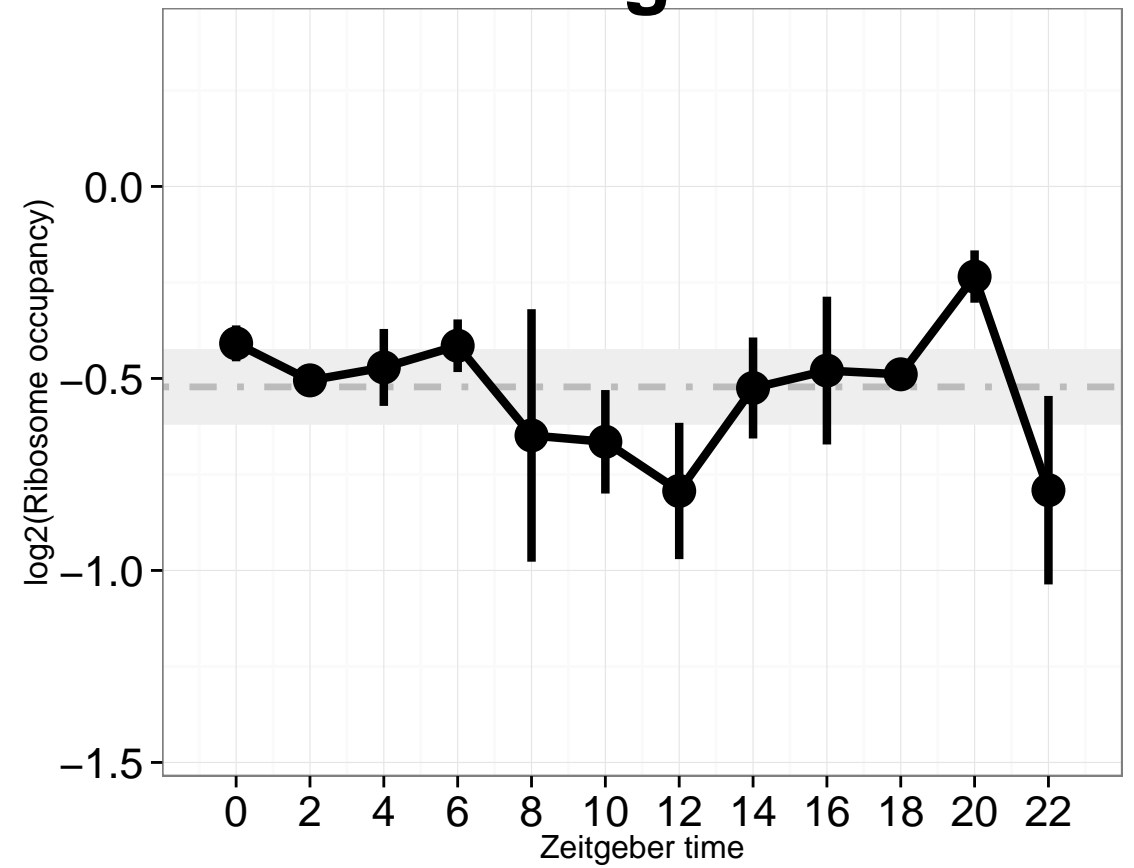

Supplement: Supplementary file 7 — Expression plots for kidney and liver for the 178 common rhythmic genes of Fig. 3c. (ZIP 3338.28 kb) [file 13059_2017_1222_MOESM7_ESM.zip › set_D_shared(178)/Cgn_liver_set_D.pdf]

# Chic1

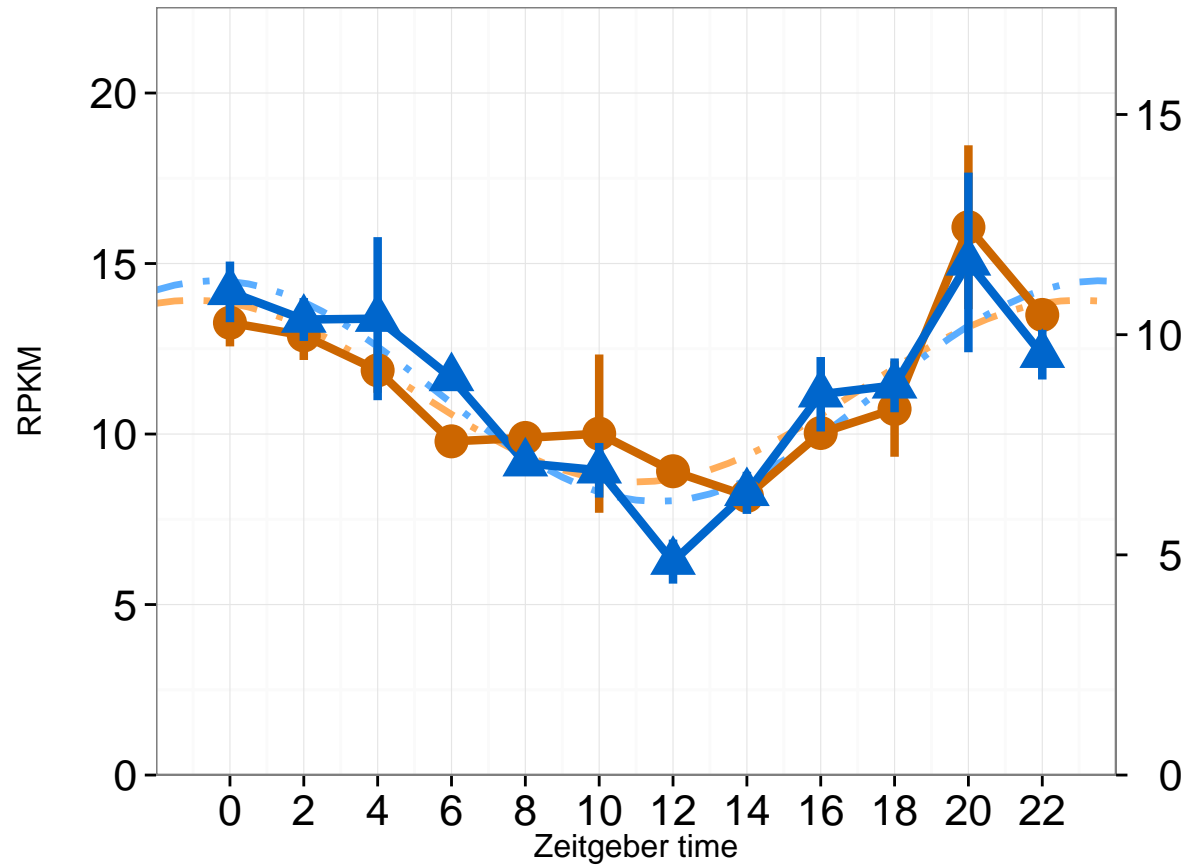

# Chic1

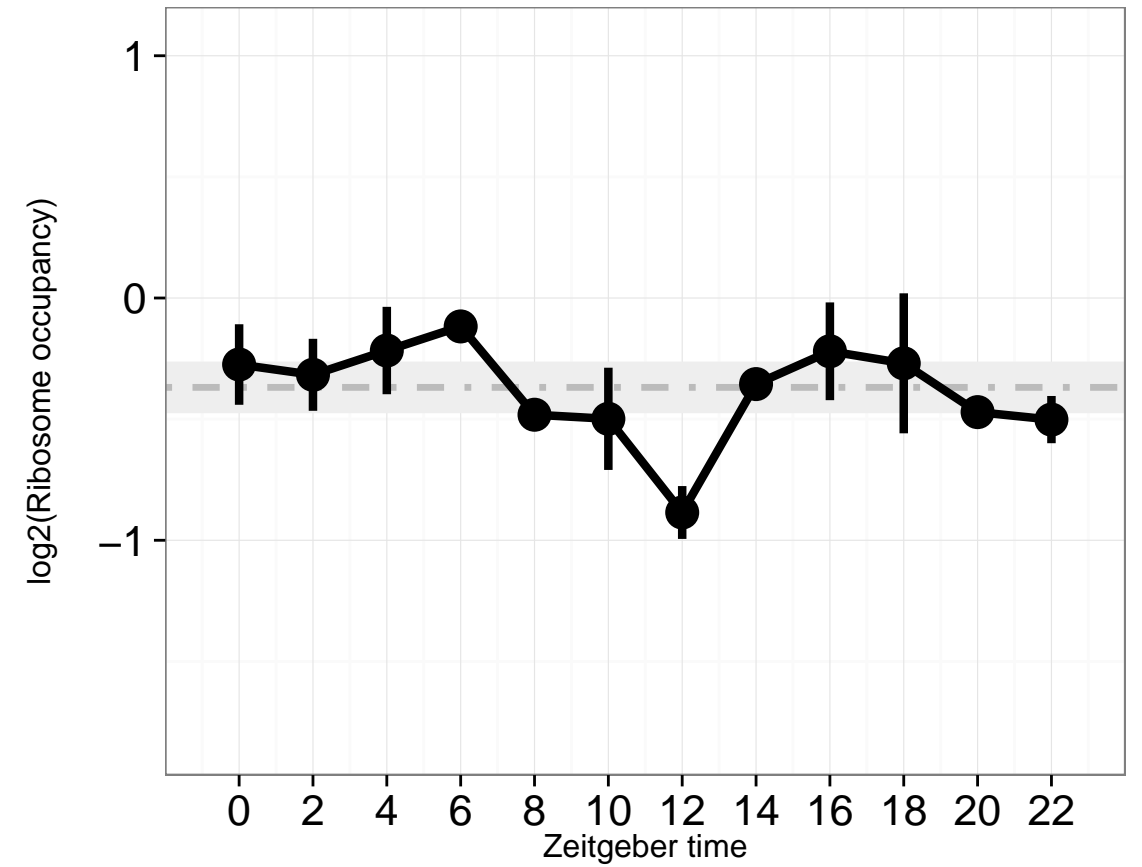

Supplement: Supplementary file 7 — Expression plots for kidney and liver for the 178 common rhythmic genes of Fig. 3c. (ZIP 3338.28 kb) [file 13059_2017_1222_MOESM7_ESM.zip › set_D_shared(178)/Chic1_kidney_set_D.pdf]

## Chic1

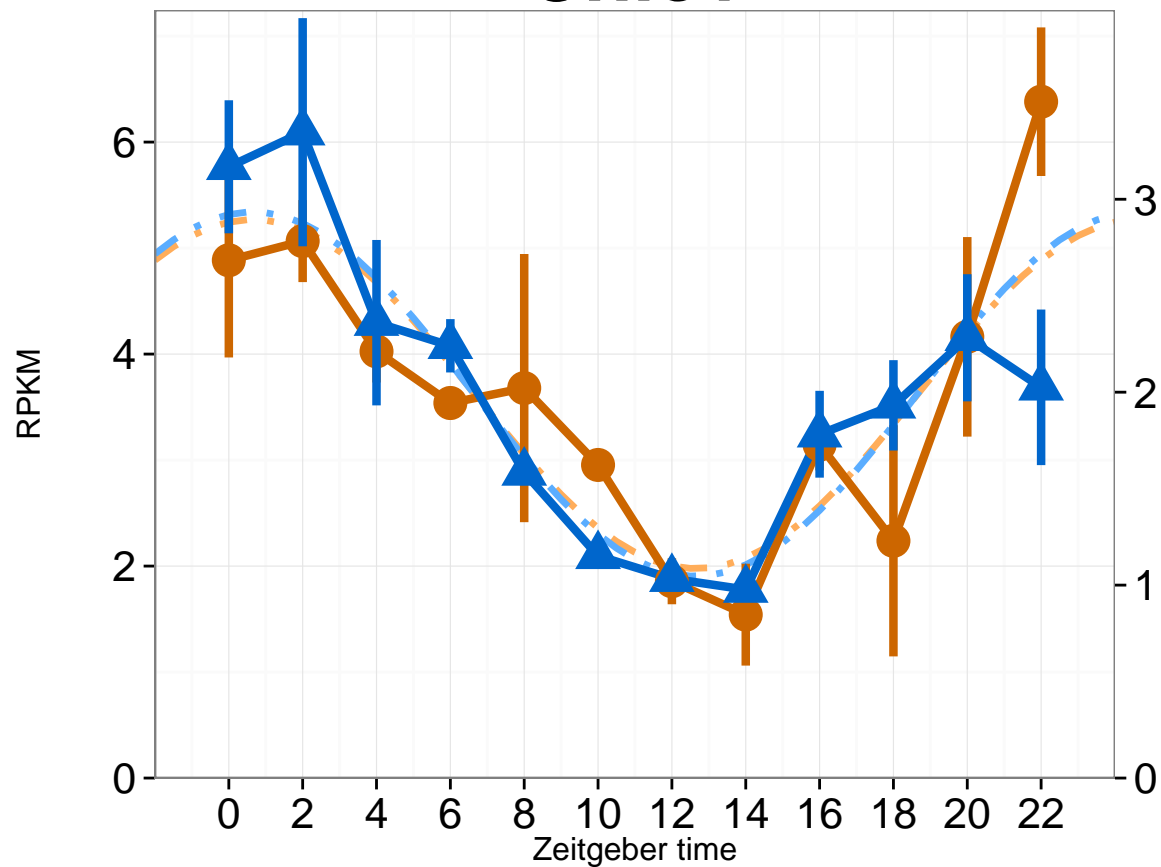

## Chic1

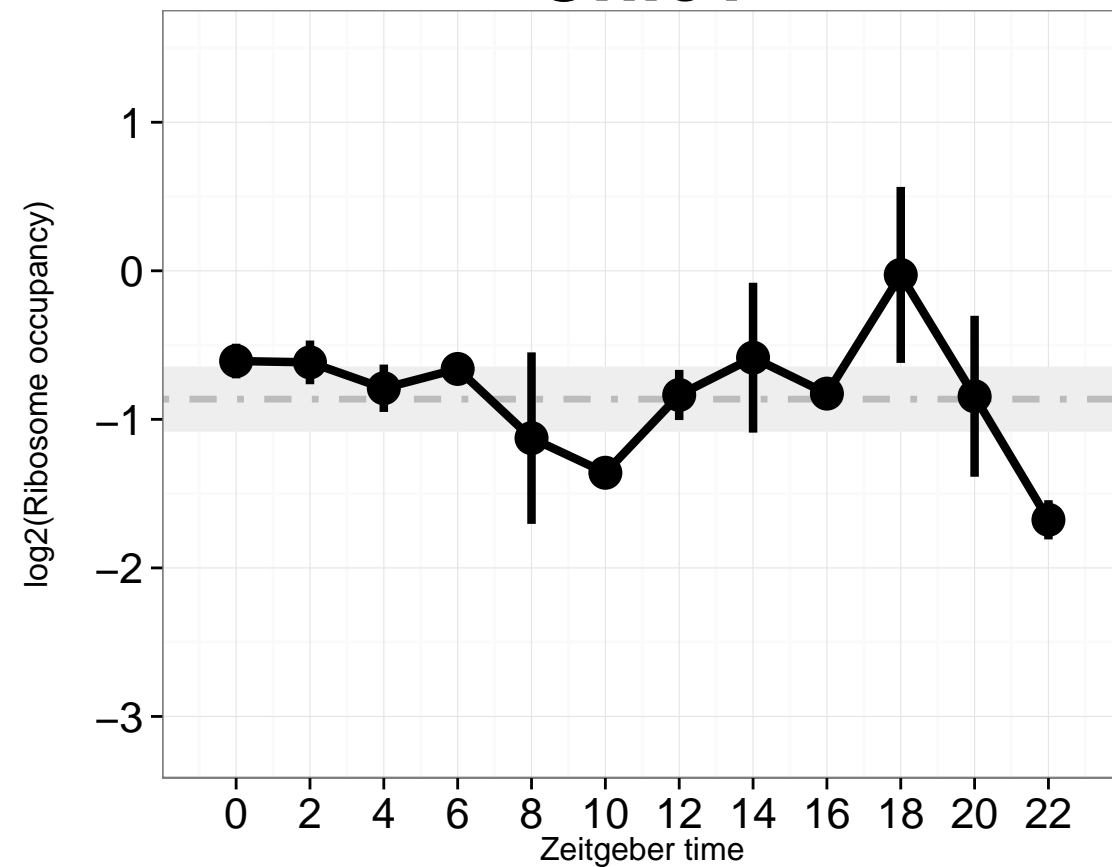

Supplement: Supplementary file 7 — Expression plots for kidney and liver for the 178 common rhythmic genes of Fig. 3c. (ZIP 3338.28 kb) [file 13059_2017_1222_MOESM7_ESM.zip › set_D_shared(178)/Chic1_liver_set_D.pdf]

# Chordc1

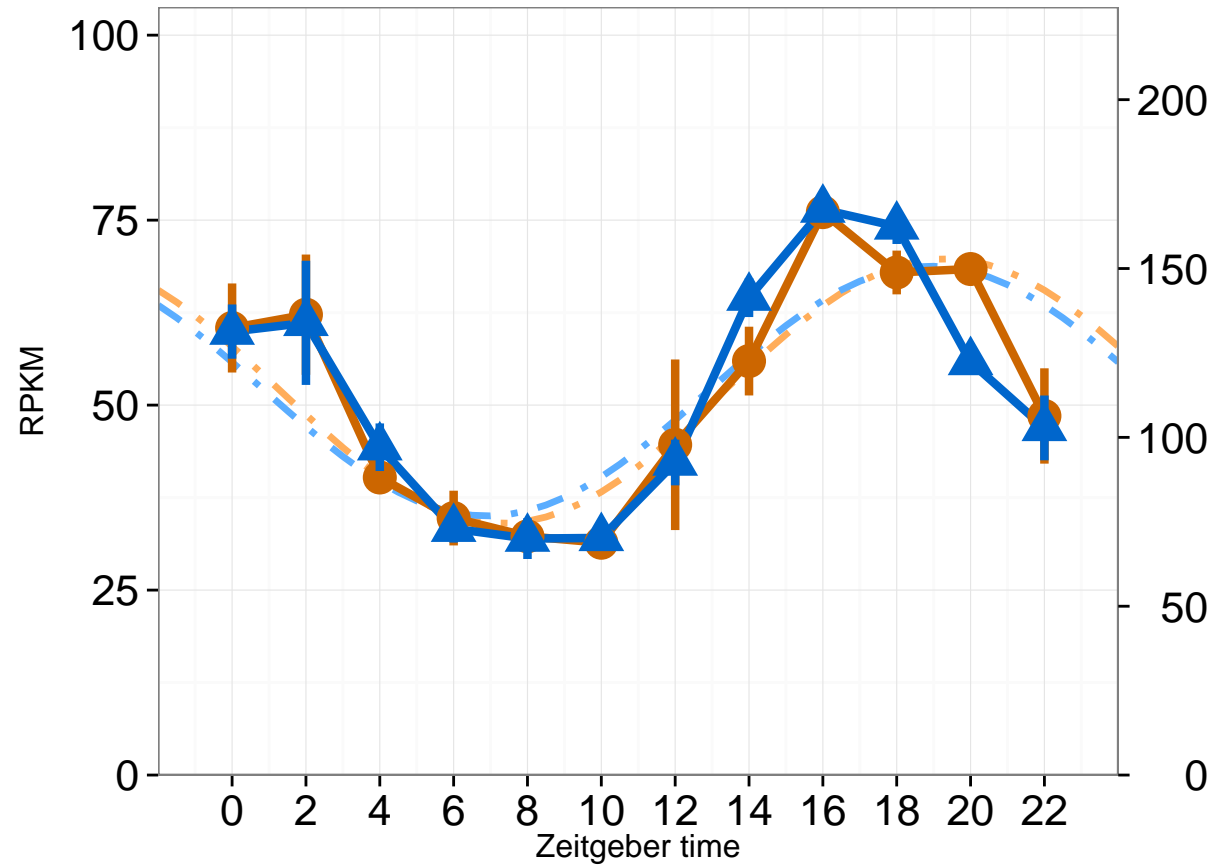

# Chordc1

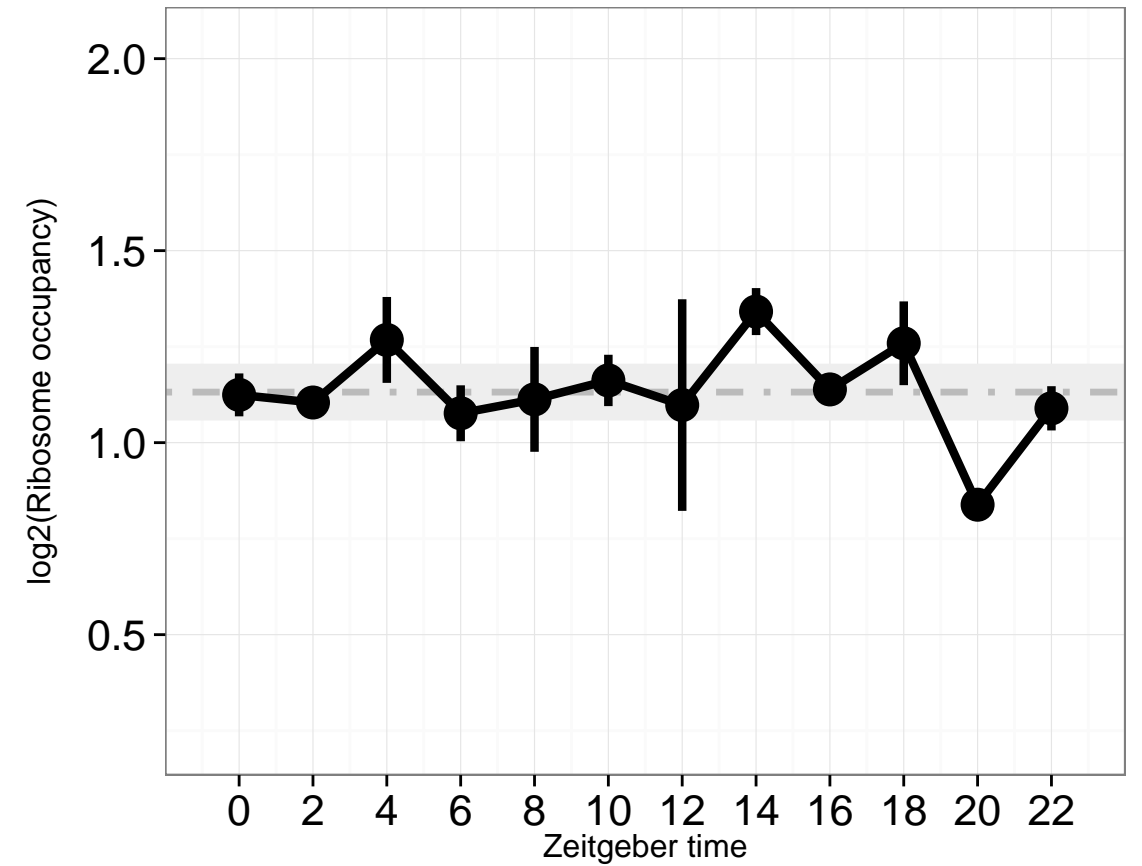

Supplement: Supplementary file 7 — Expression plots for kidney and liver for the 178 common rhythmic genes of Fig. 3c. (ZIP 3338.28 kb) [file 13059_2017_1222_MOESM7_ESM.zip › set_D_shared(178)/Chordc1_kidney_set_D.pdf]

## Chordc1

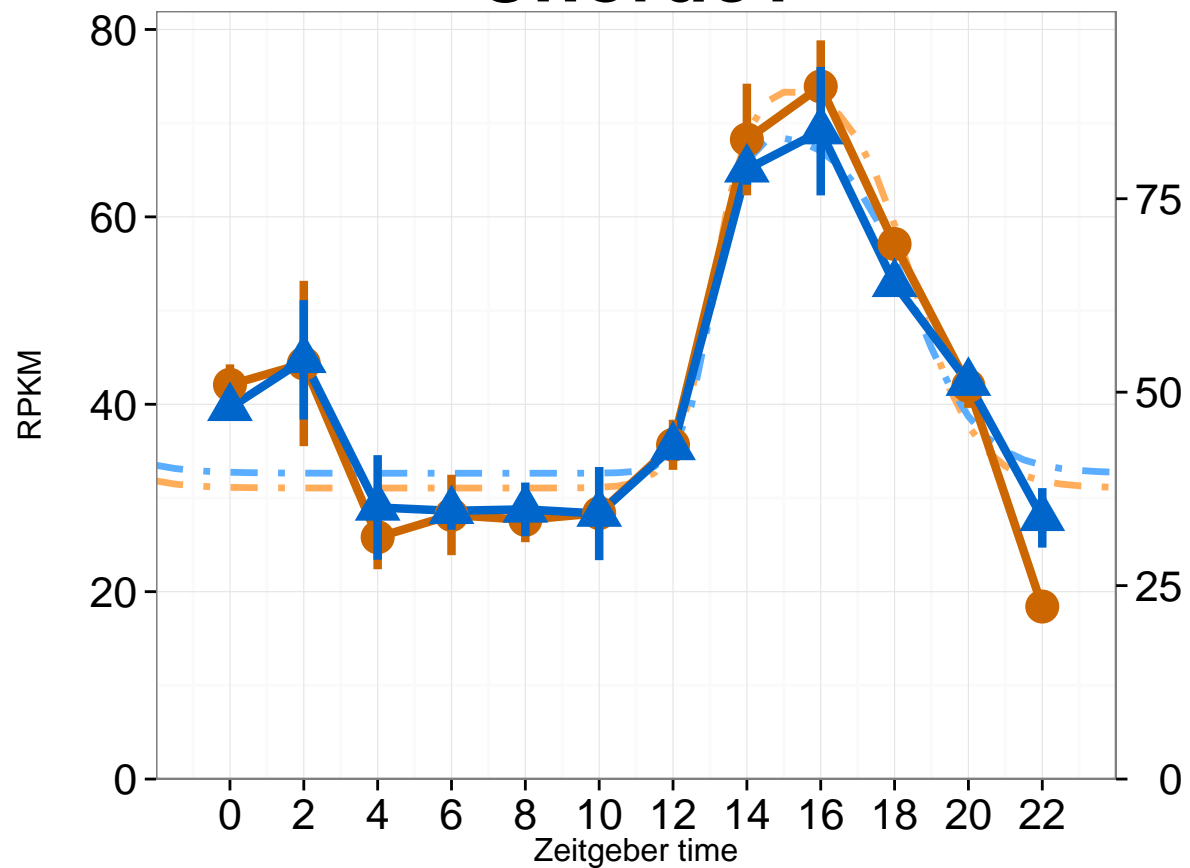

## Chordc1

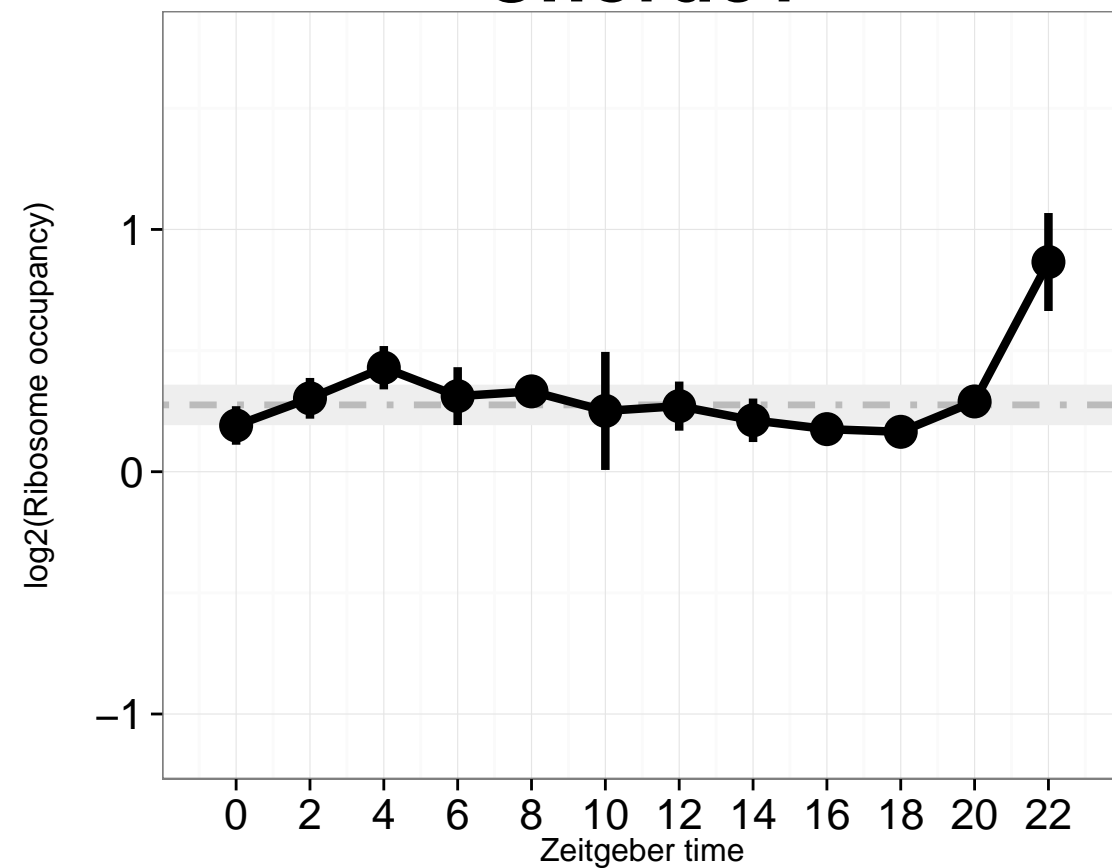

Supplement: Supplementary file 7 — Expression plots for kidney and liver for the 178 common rhythmic genes of Fig. 3c. (ZIP 3338.28 kb) [file 13059_2017_1222_MOESM7_ESM.zip › set_D_shared(178)/Chordc1_liver_set_D.pdf]

## Cirbp

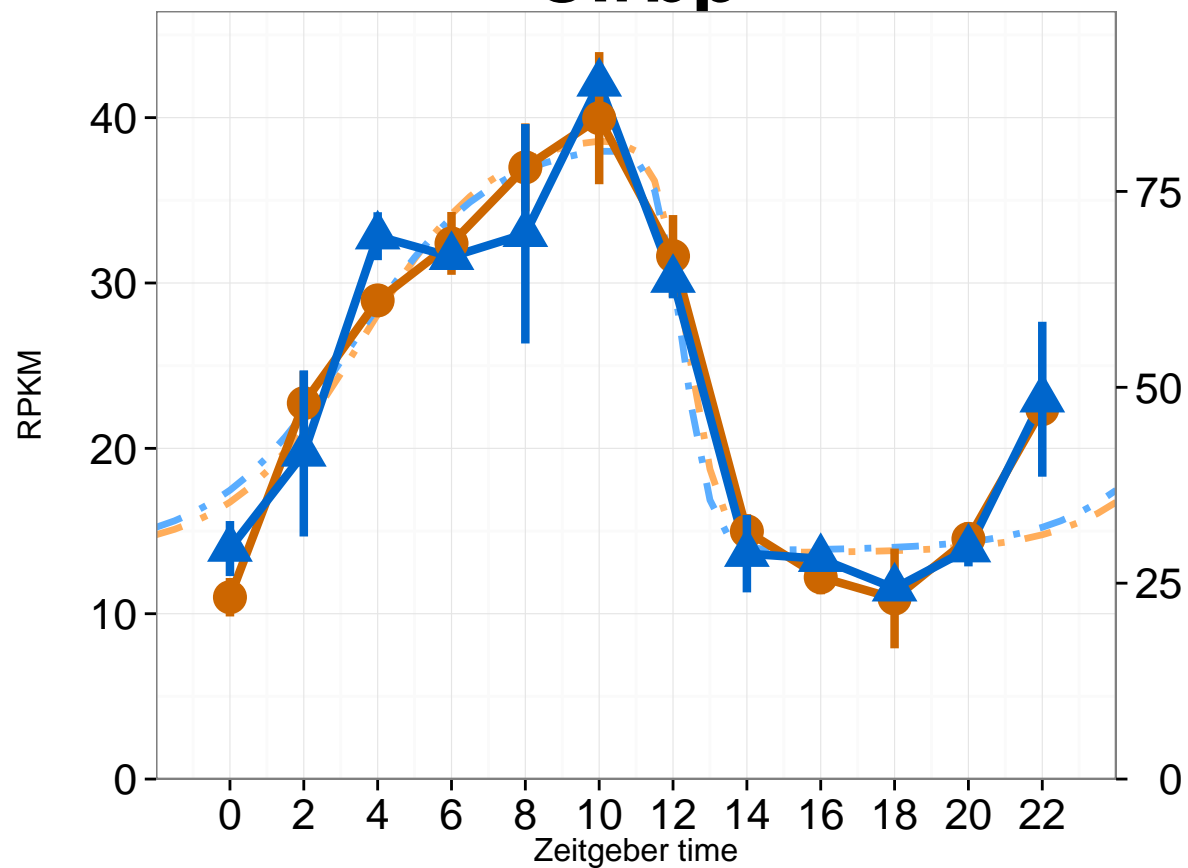

## Cirbp

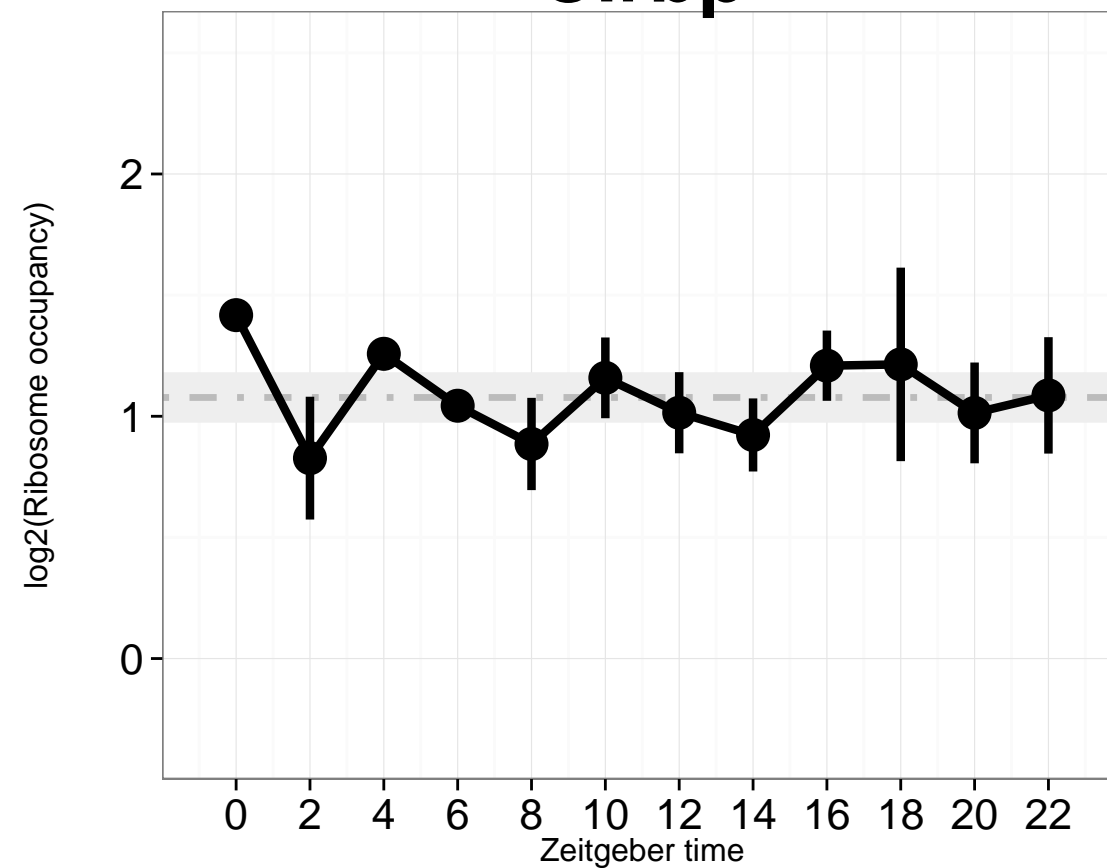

Supplement: Supplementary file 7 — Expression plots for kidney and liver for the 178 common rhythmic genes of Fig. 3c. (ZIP 3338.28 kb) [file 13059_2017_1222_MOESM7_ESM.zip › set_D_shared(178)/Cirbp_kidney_set_D.pdf]

## Cirbp

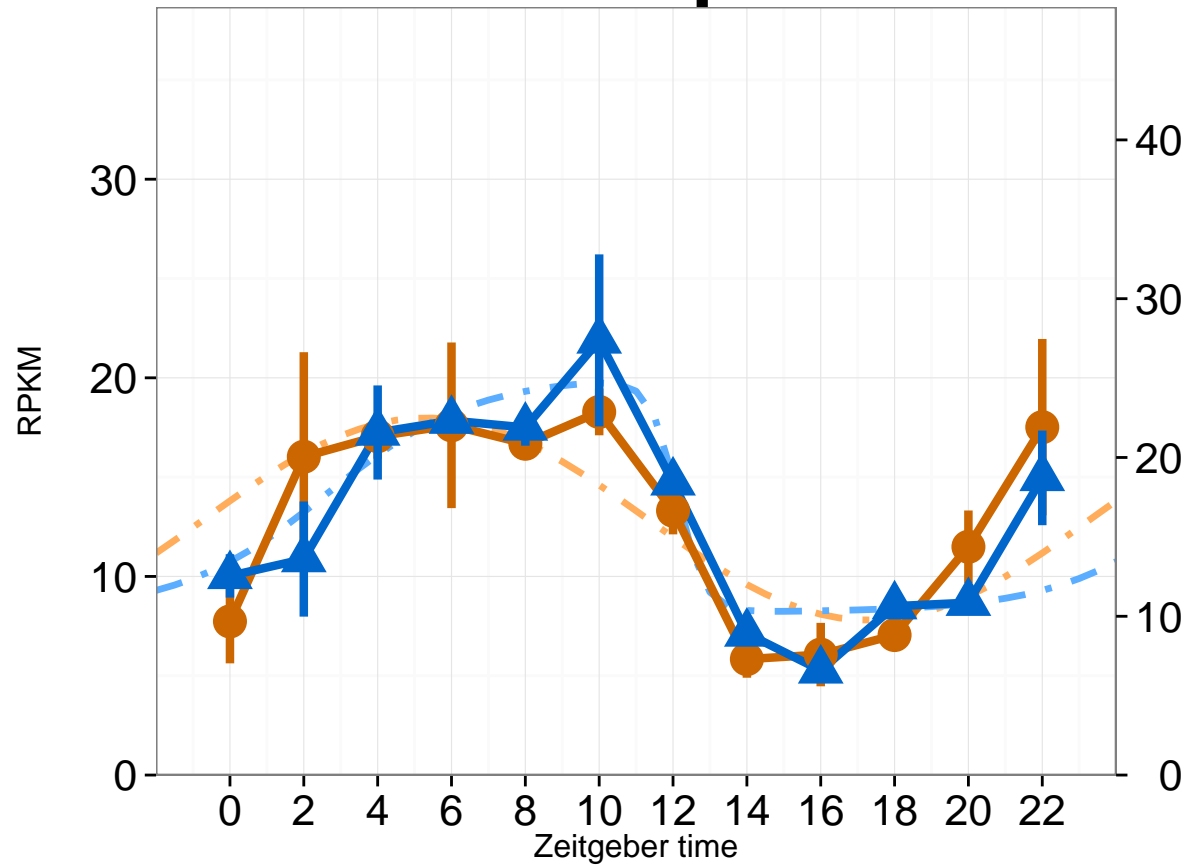

## Cirbp

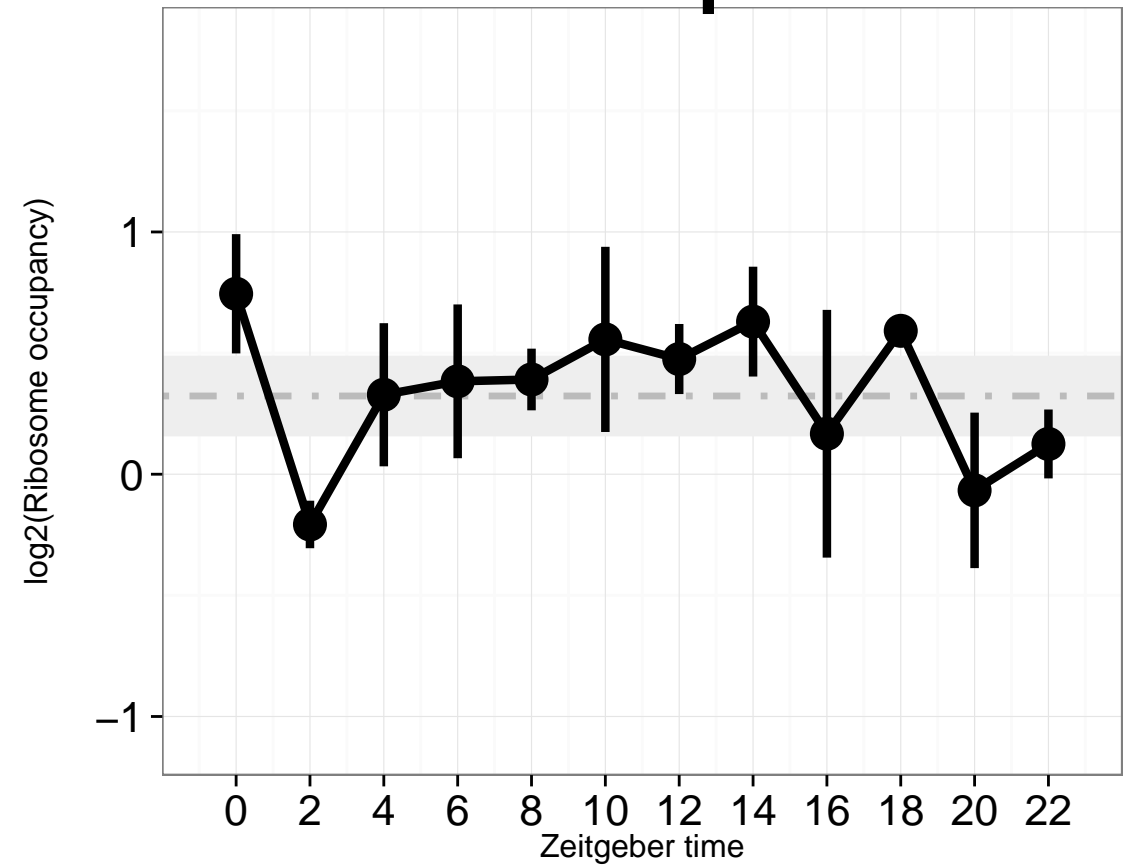

Supplement: Supplementary file 7 — Expression plots for kidney and liver for the 178 common rhythmic genes of Fig. 3c. (ZIP 3338.28 kb) [file 13059_2017_1222_MOESM7_ESM.zip › set_D_shared(178)/Cirbp_liver_set_D.pdf]

## Ckb

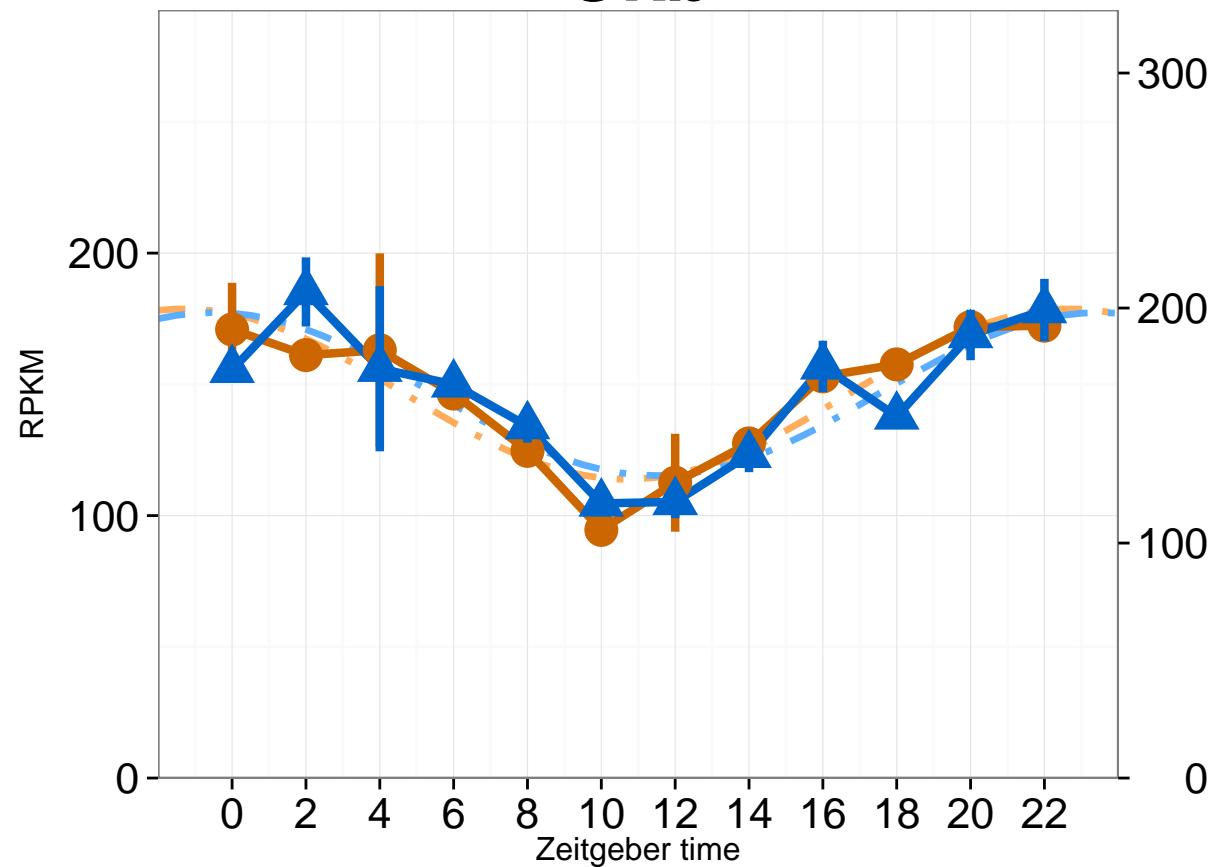

## Ckb

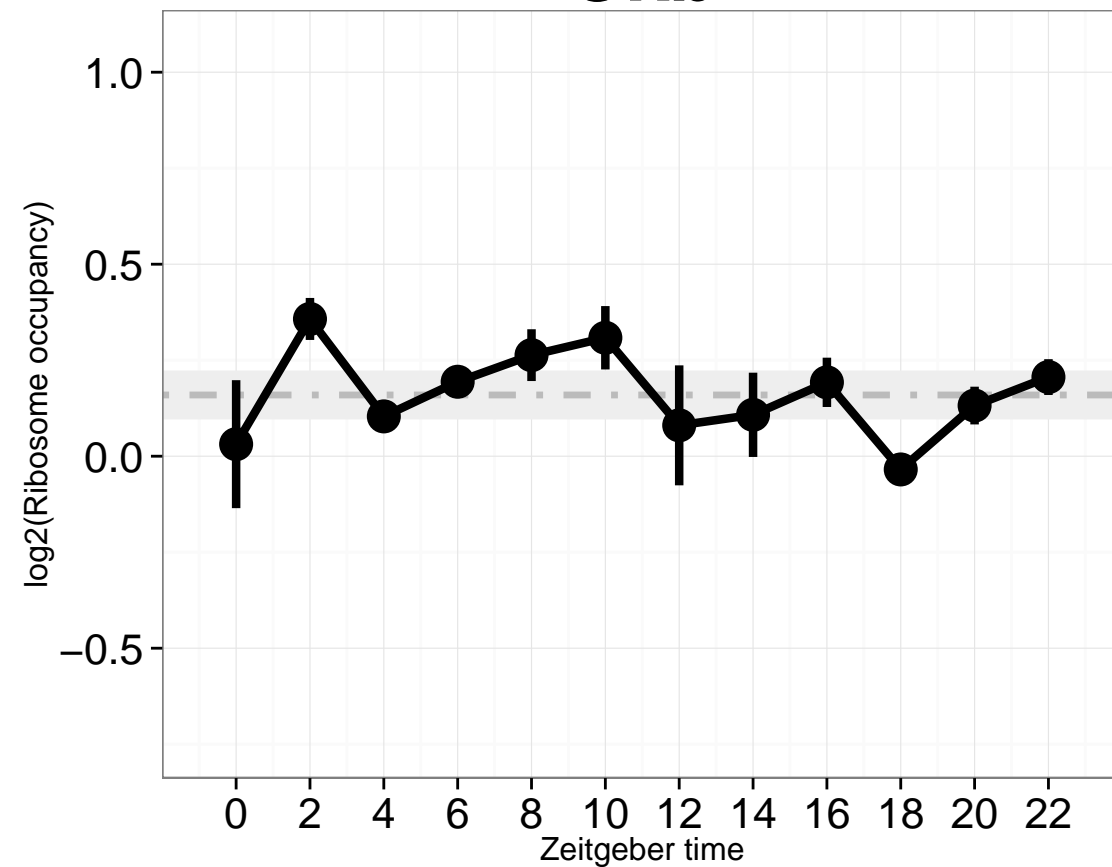

Supplement: Supplementary file 7 — Expression plots for kidney and liver for the 178 common rhythmic genes of Fig. 3c. (ZIP 3338.28 kb) [file 13059_2017_1222_MOESM7_ESM.zip › set_D_shared(178)/Ckb_kidney_set_D.pdf]

## Ckb

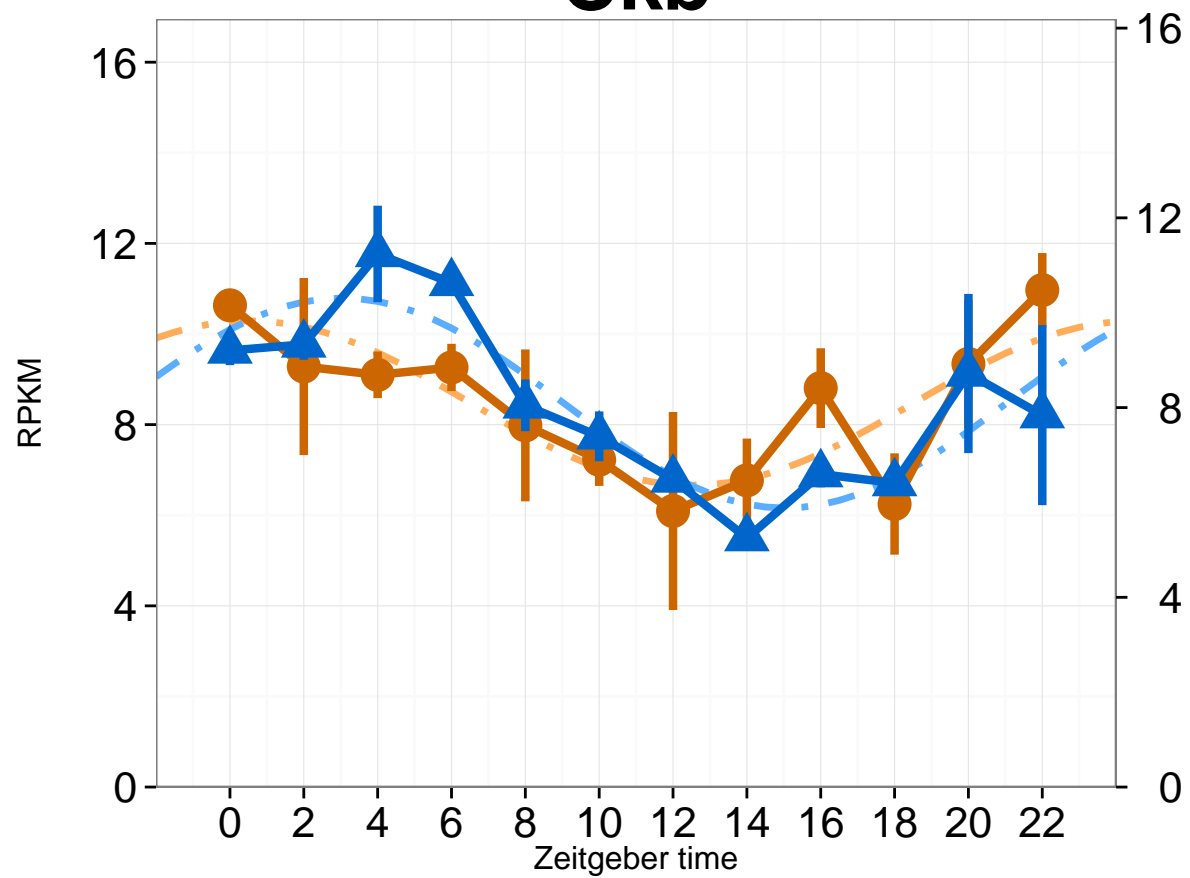

## Ckb

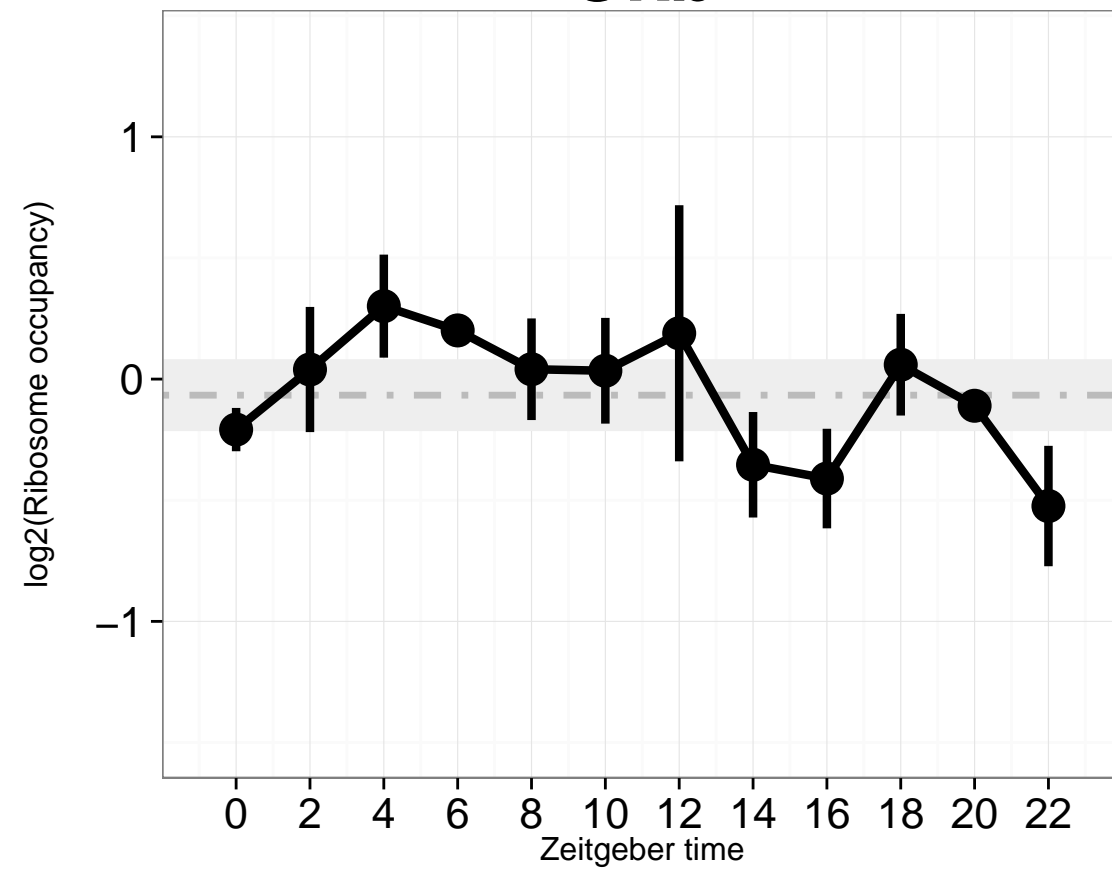

Supplement: Supplementary file 7 — Expression plots for kidney and liver for the 178 common rhythmic genes of Fig. 3c. (ZIP 3338.28 kb) [file 13059_2017_1222_MOESM7_ESM.zip › set_D_shared(178)/Ckb_liver_set_D.pdf]

# Clca1

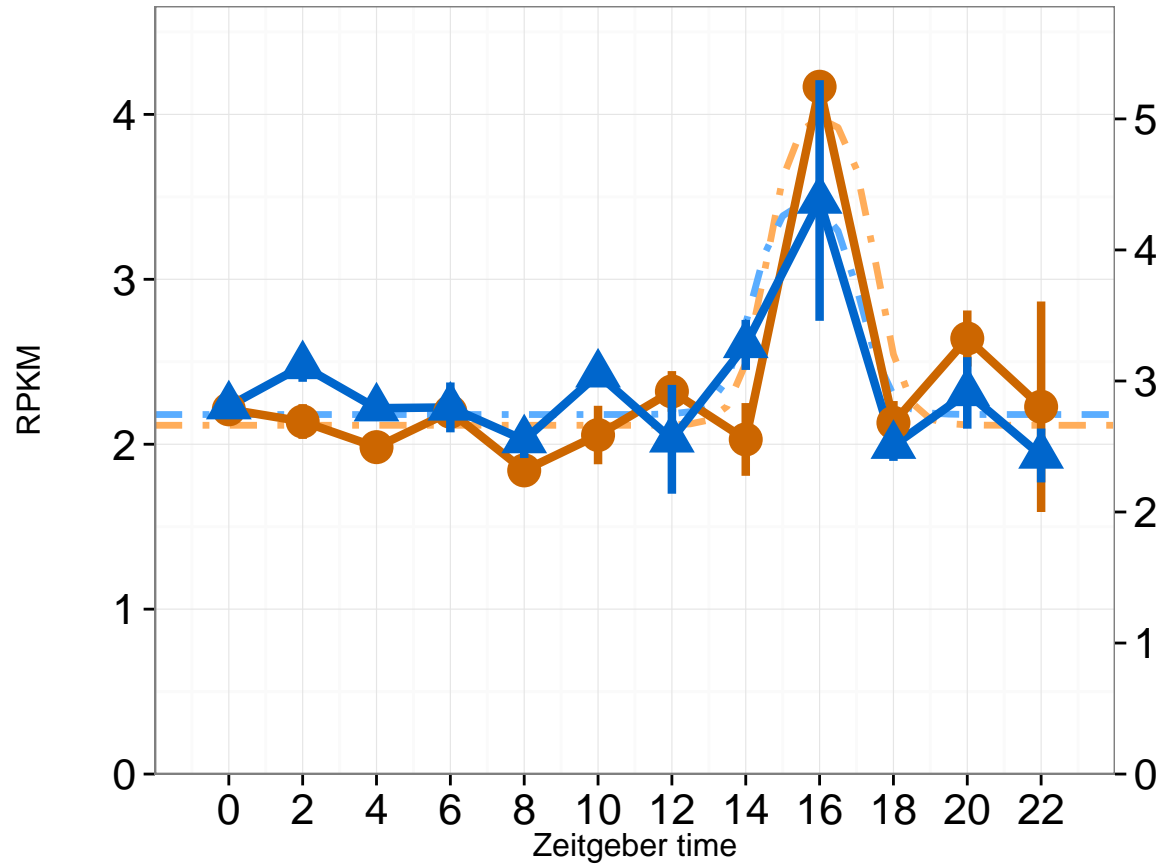

# Clca1

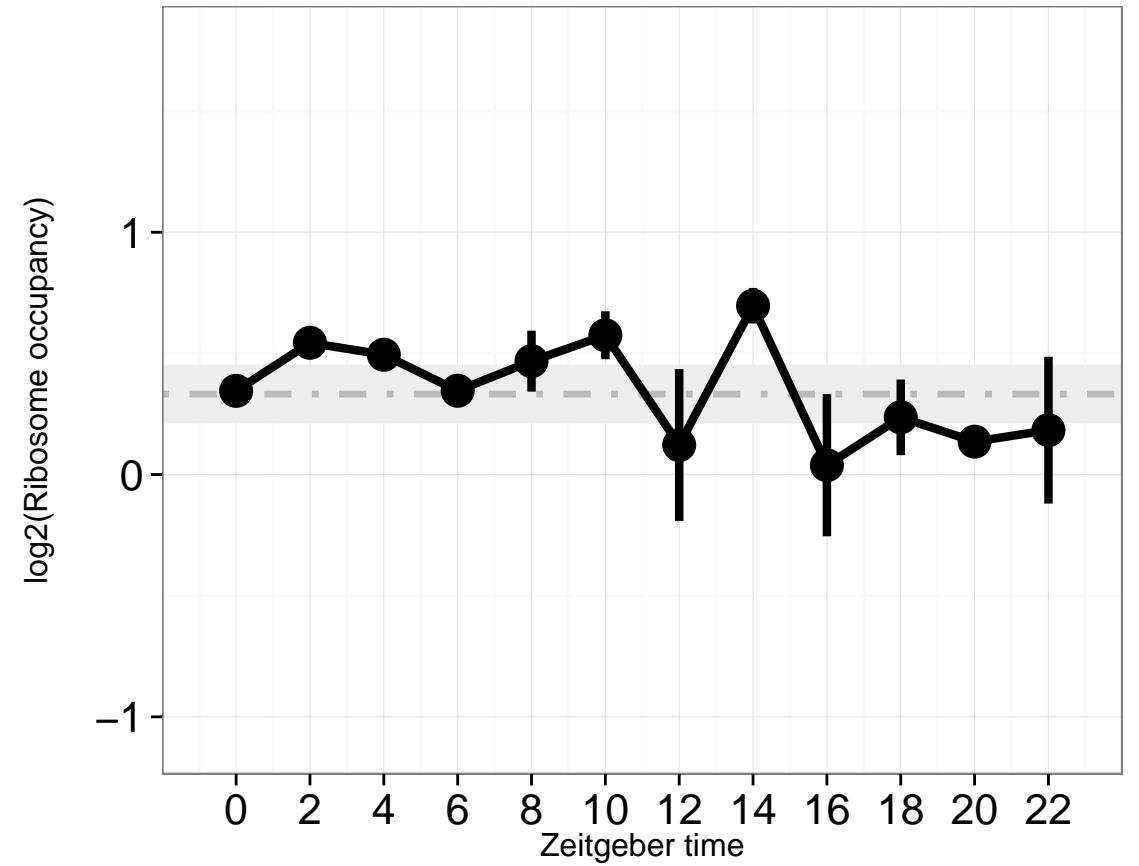

Supplement: Supplementary file 7 — Expression plots for kidney and liver for the 178 common rhythmic genes of Fig. 3c. (ZIP 3338.28 kb) [file 13059_2017_1222_MOESM7_ESM.zip › set_D_shared(178)/Clca1_kidney_set_D.pdf]

# Clca1

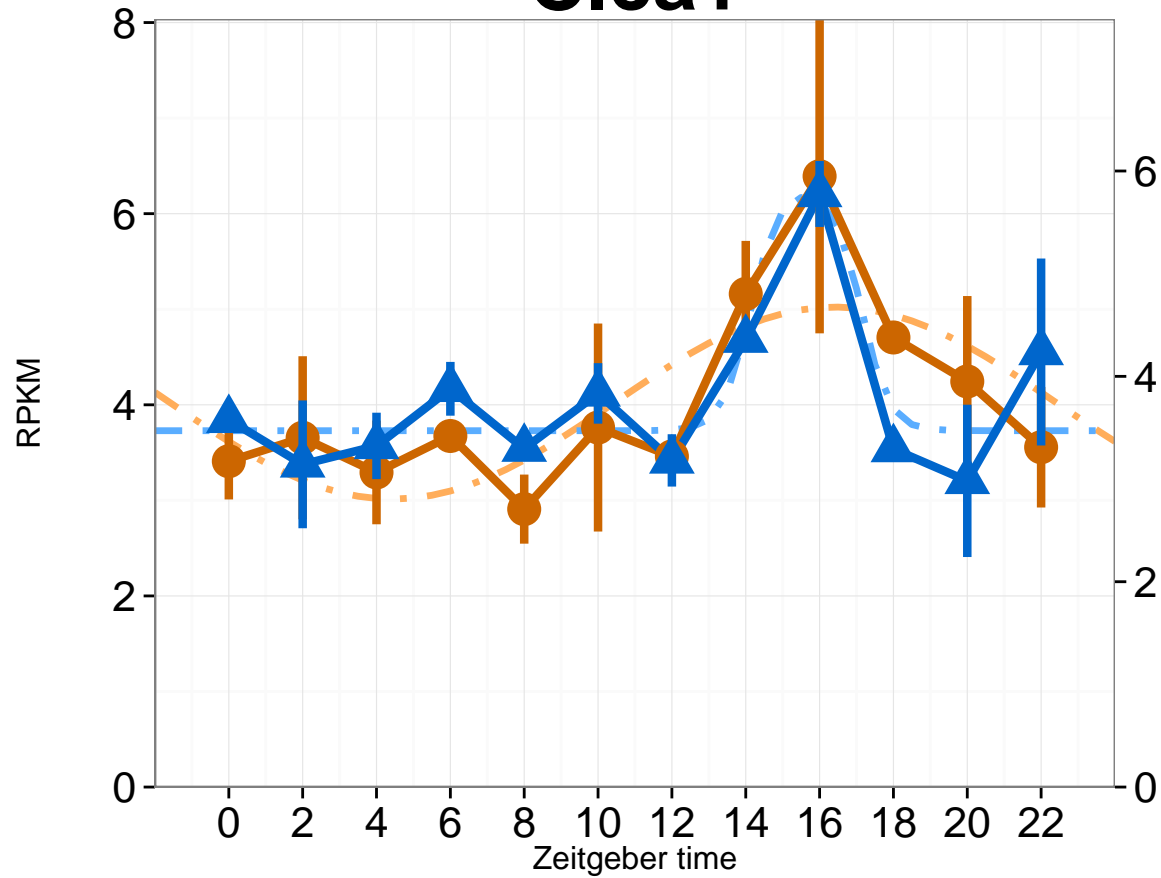

# Clca1

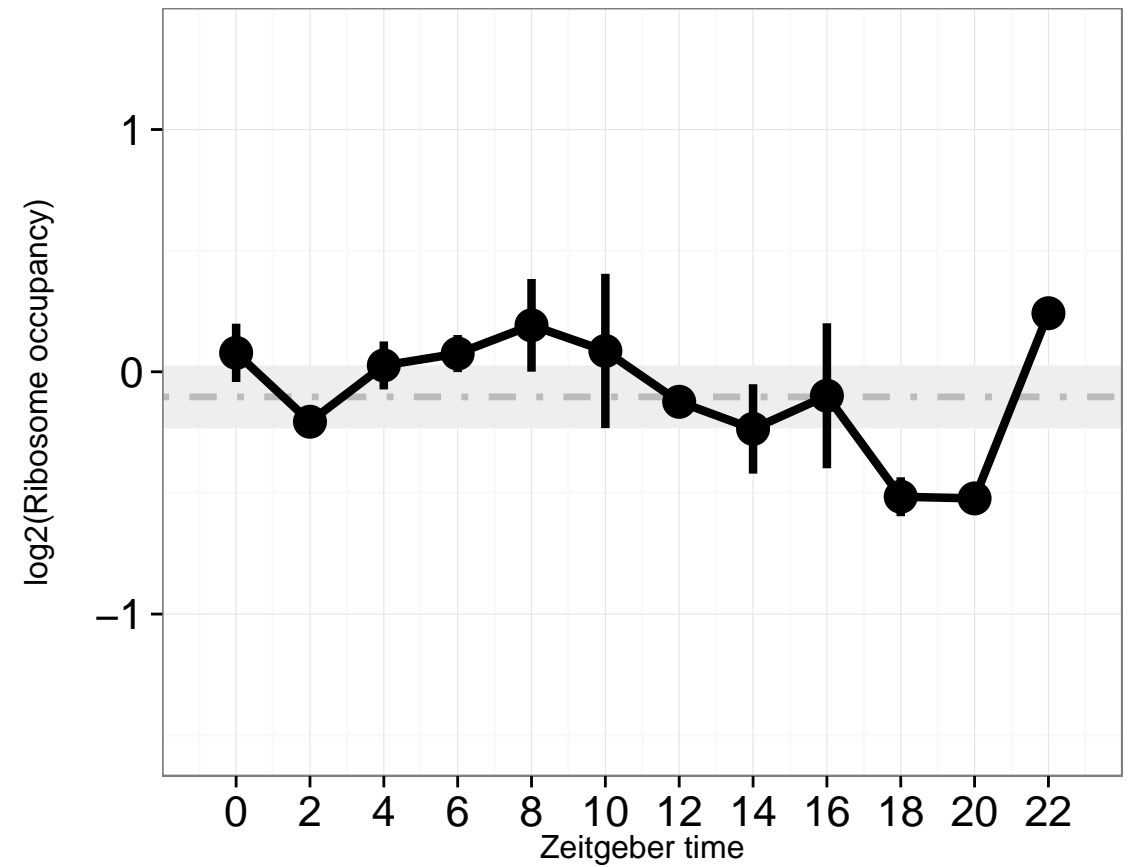

Supplement: Supplementary file 7 — Expression plots for kidney and liver for the 178 common rhythmic genes of Fig. 3c. (ZIP 3338.28 kb) [file 13059_2017_1222_MOESM7_ESM.zip › set_D_shared(178)/Clca1_liver_set_D.pdf]

## Cldn1

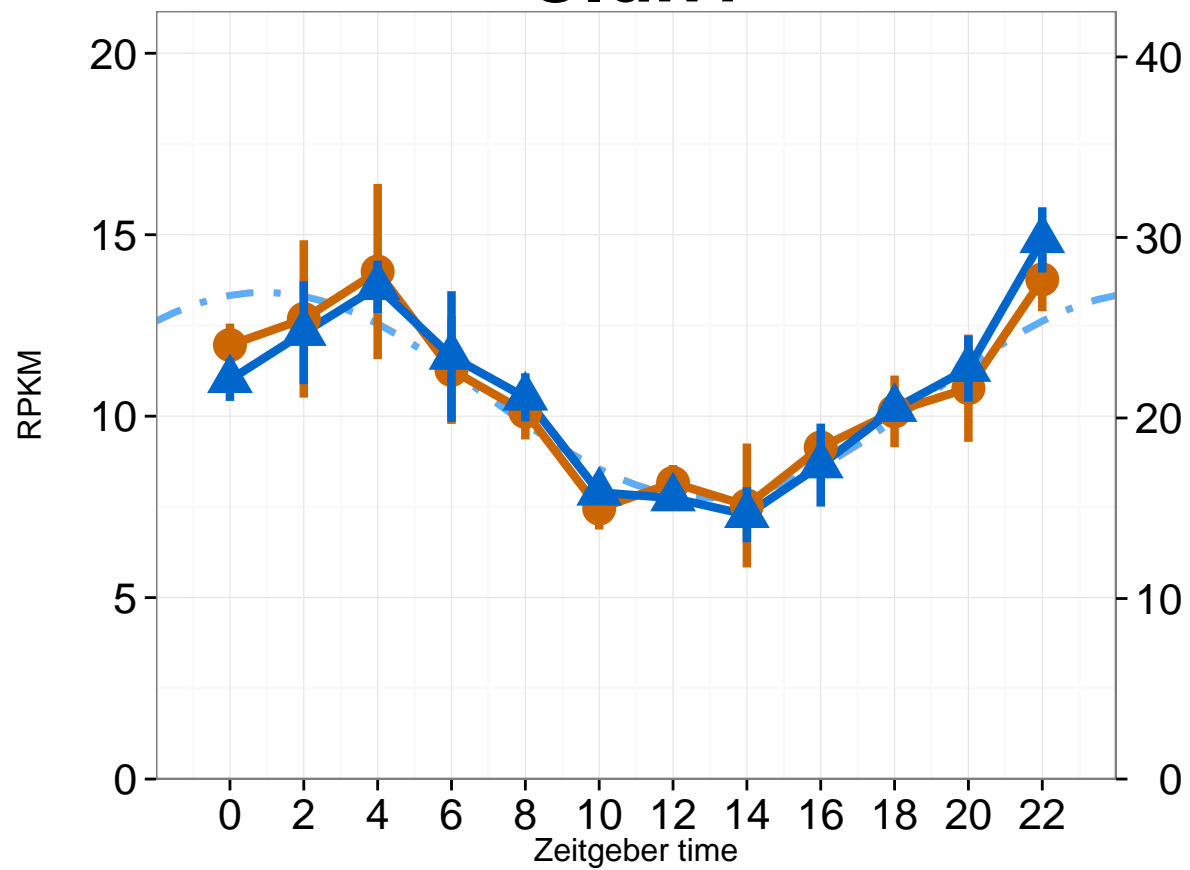

## Cldn1

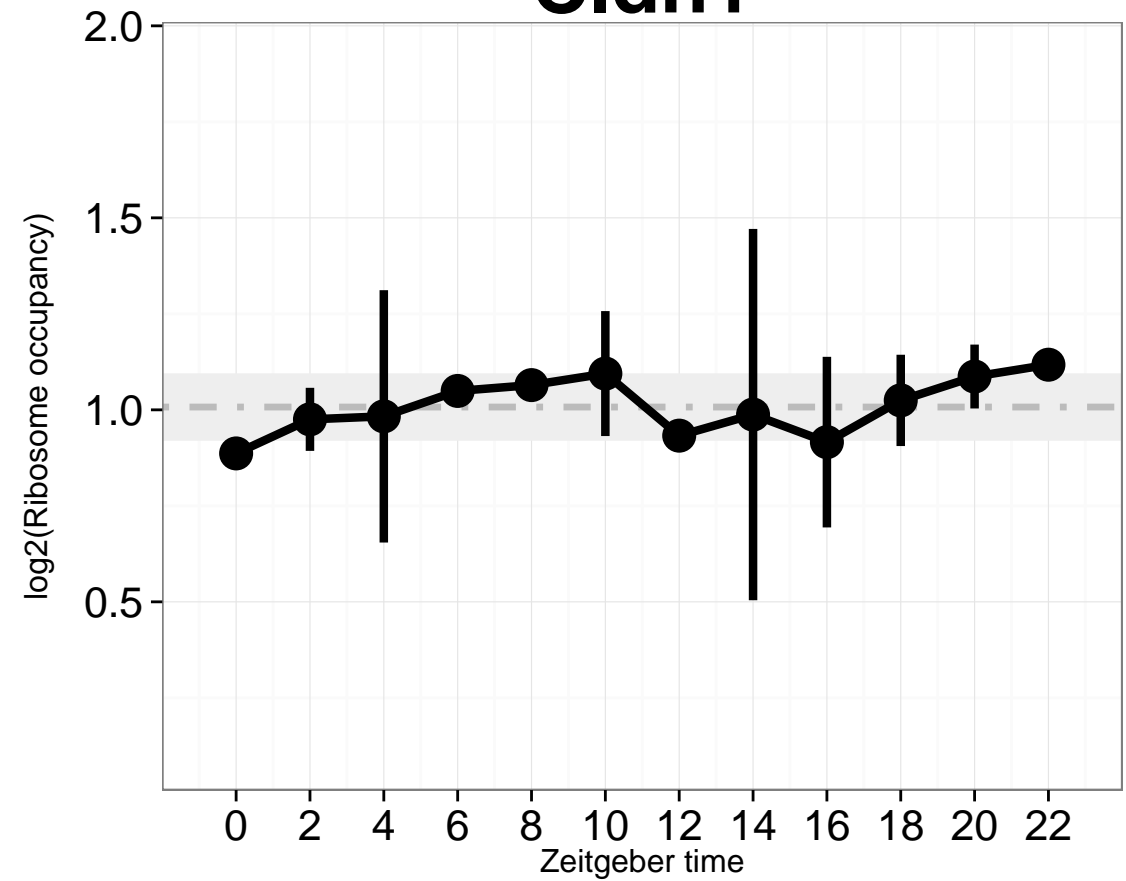

Supplement: Supplementary file 7 — Expression plots for kidney and liver for the 178 common rhythmic genes of Fig. 3c. (ZIP 3338.28 kb) [file 13059_2017_1222_MOESM7_ESM.zip › set_D_shared(178)/Cldn1_kidney_set_D.pdf]

## Cldn1

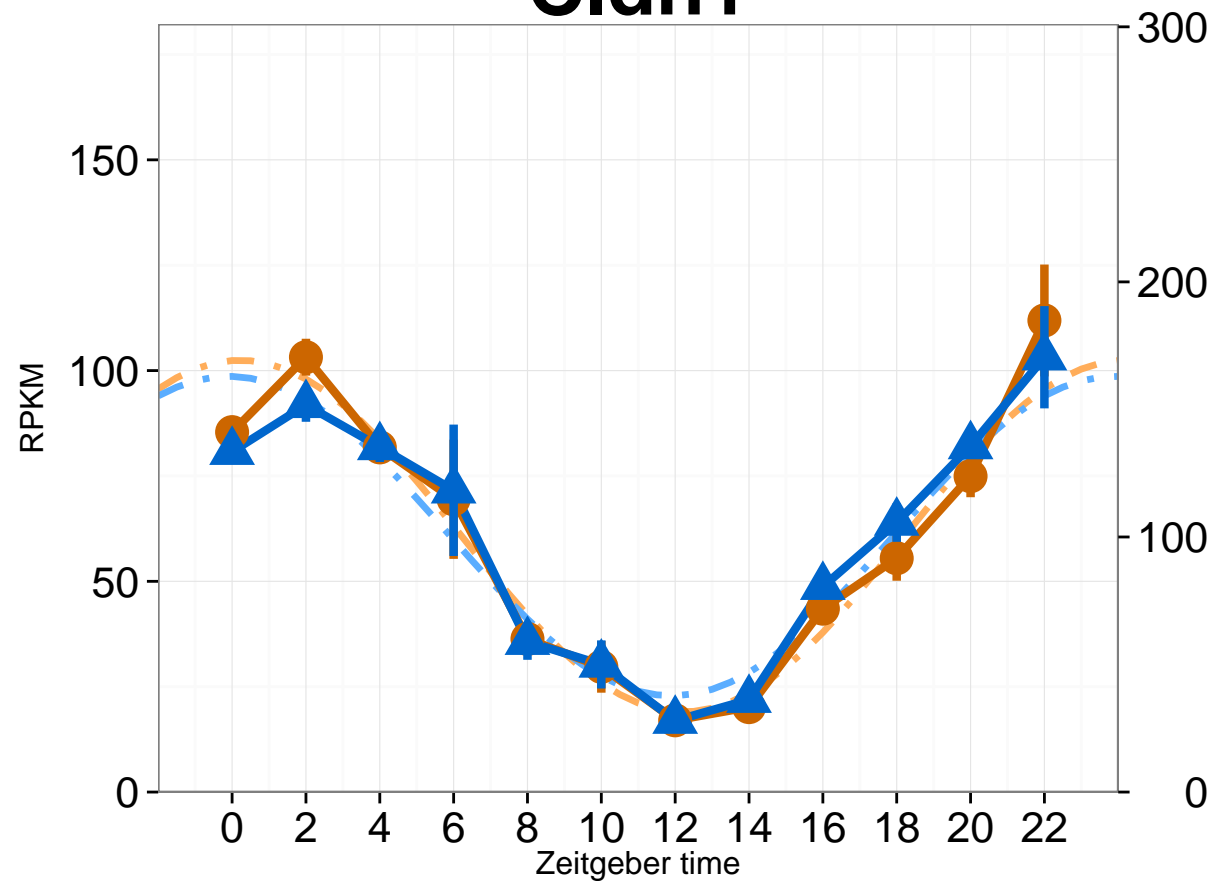

## Cldn1

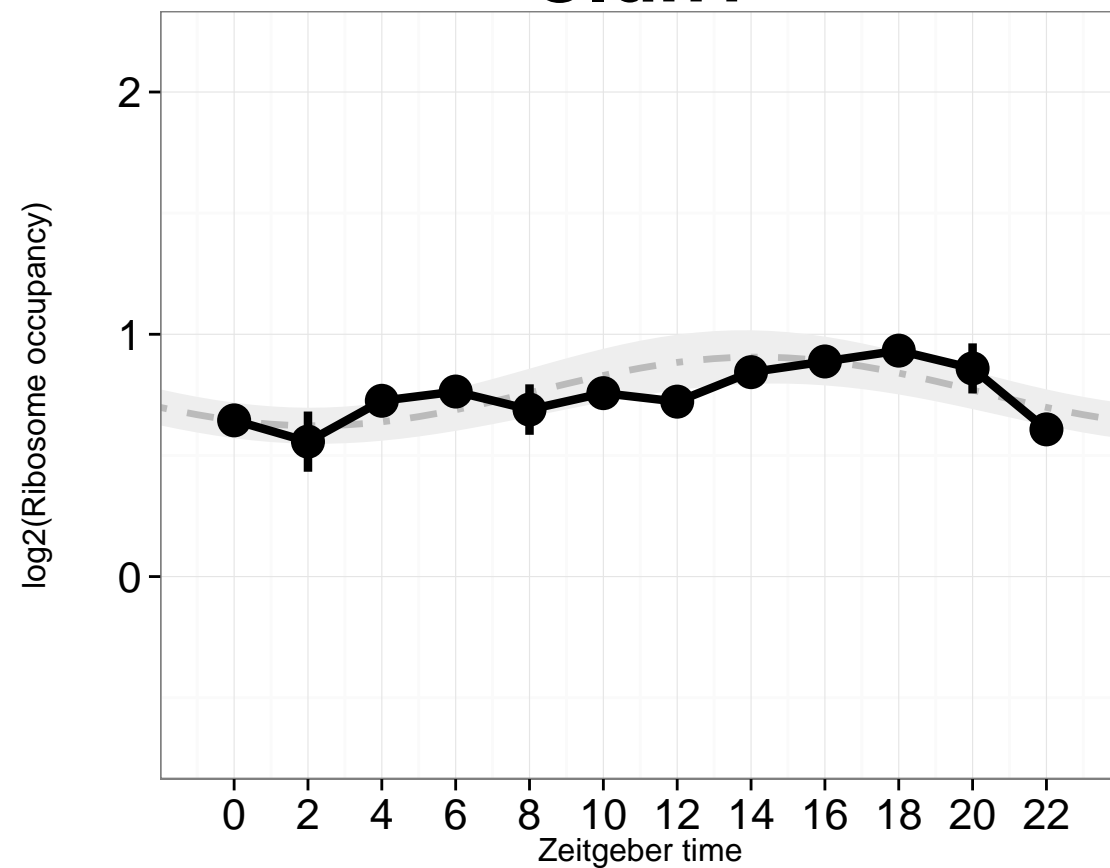

Supplement: Supplementary file 7 — Expression plots for kidney and liver for the 178 common rhythmic genes of Fig. 3c. (ZIP 3338.28 kb) [file 13059_2017_1222_MOESM7_ESM.zip › set_D_shared(178)/Cldn1_liver_set_D.pdf]

# Clip2

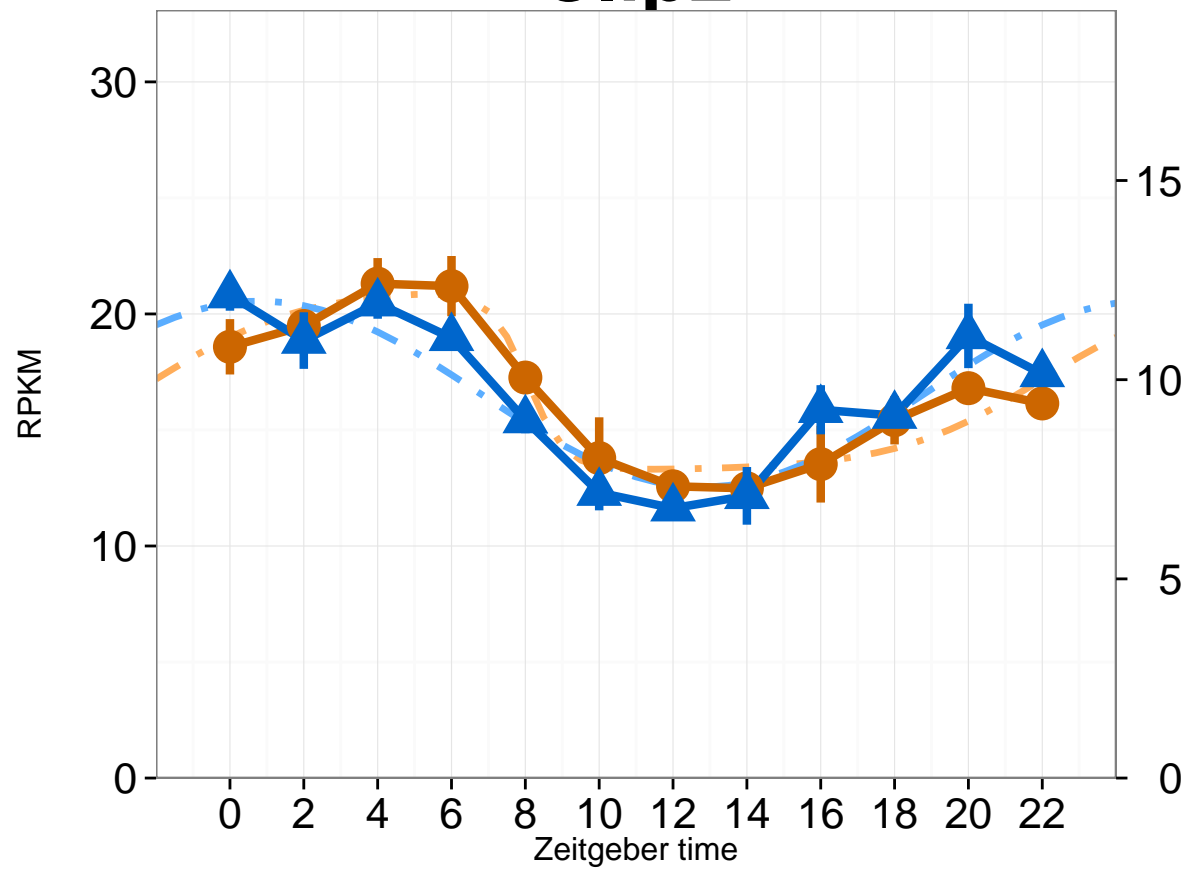

# Clip2

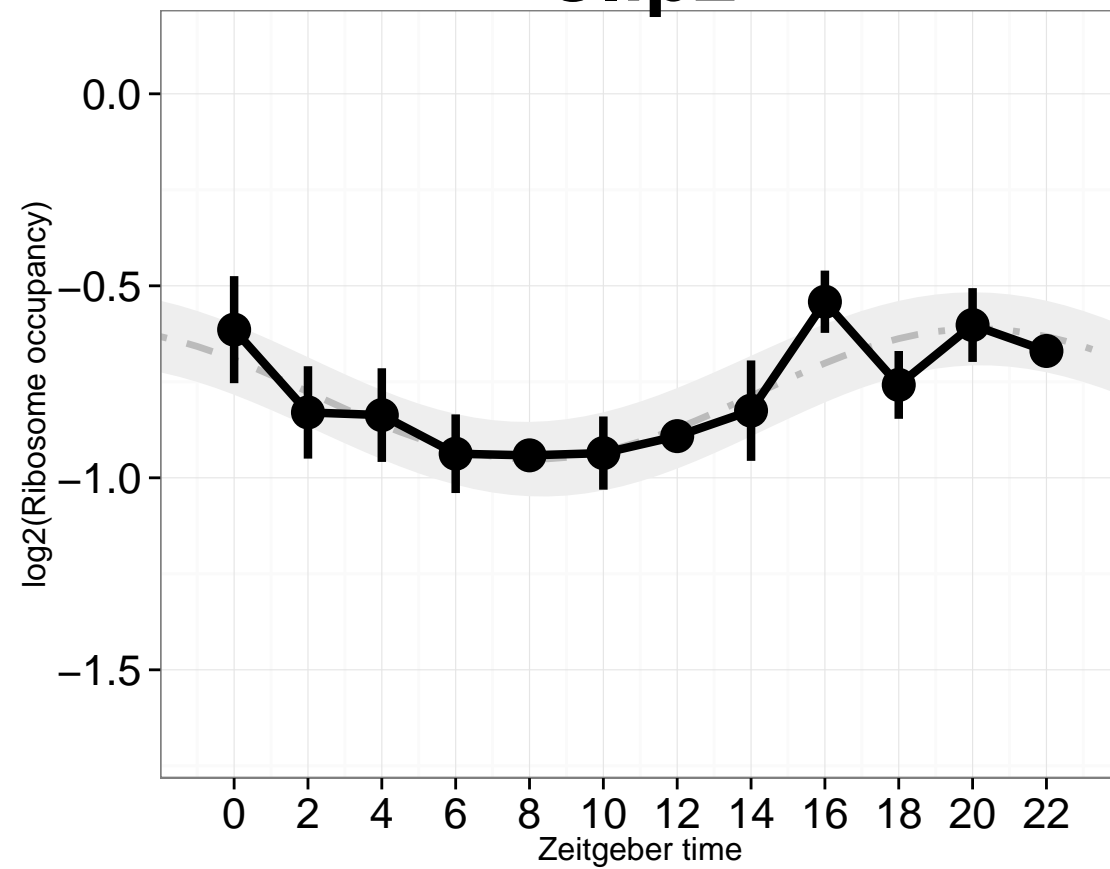

Supplement: Supplementary file 7 — Expression plots for kidney and liver for the 178 common rhythmic genes of Fig. 3c. (ZIP 3338.28 kb) [file 13059_2017_1222_MOESM7_ESM.zip › set_D_shared(178)/Clip2_kidney_set_D.pdf]

# Clip2

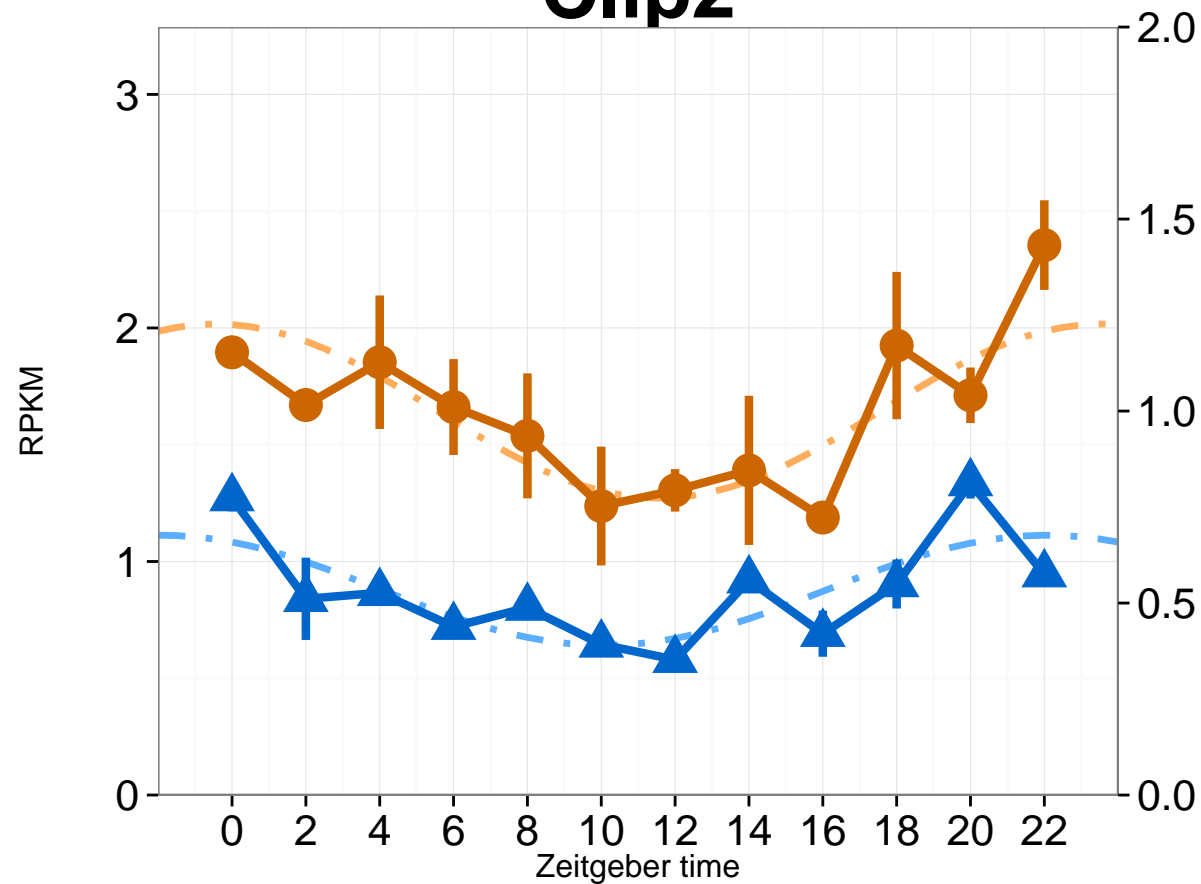

# Clip2

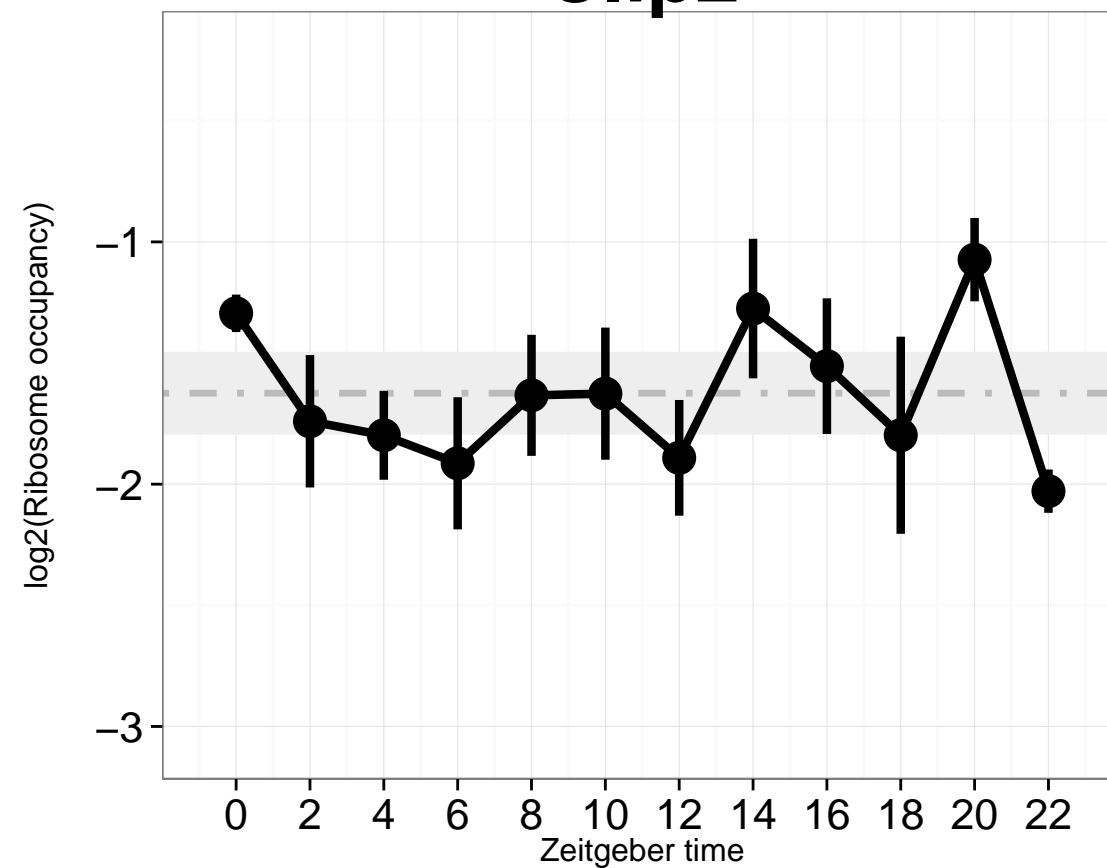

Supplement: Supplementary file 7 — Expression plots for kidney and liver for the 178 common rhythmic genes of Fig. 3c. (ZIP 3338.28 kb) [file 13059_2017_1222_MOESM7_ESM.zip › set_D_shared(178)/Clip2_liver_set_D.pdf]

## Clock

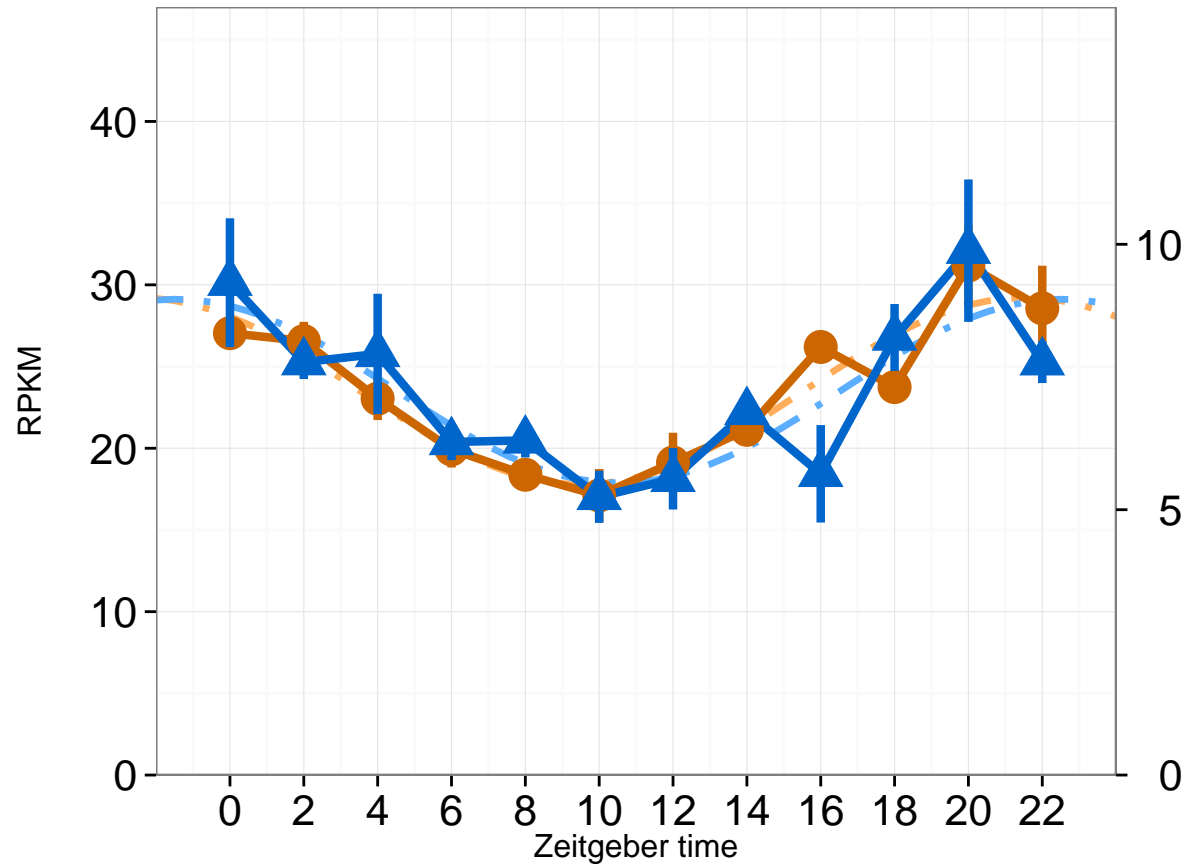

## Clock

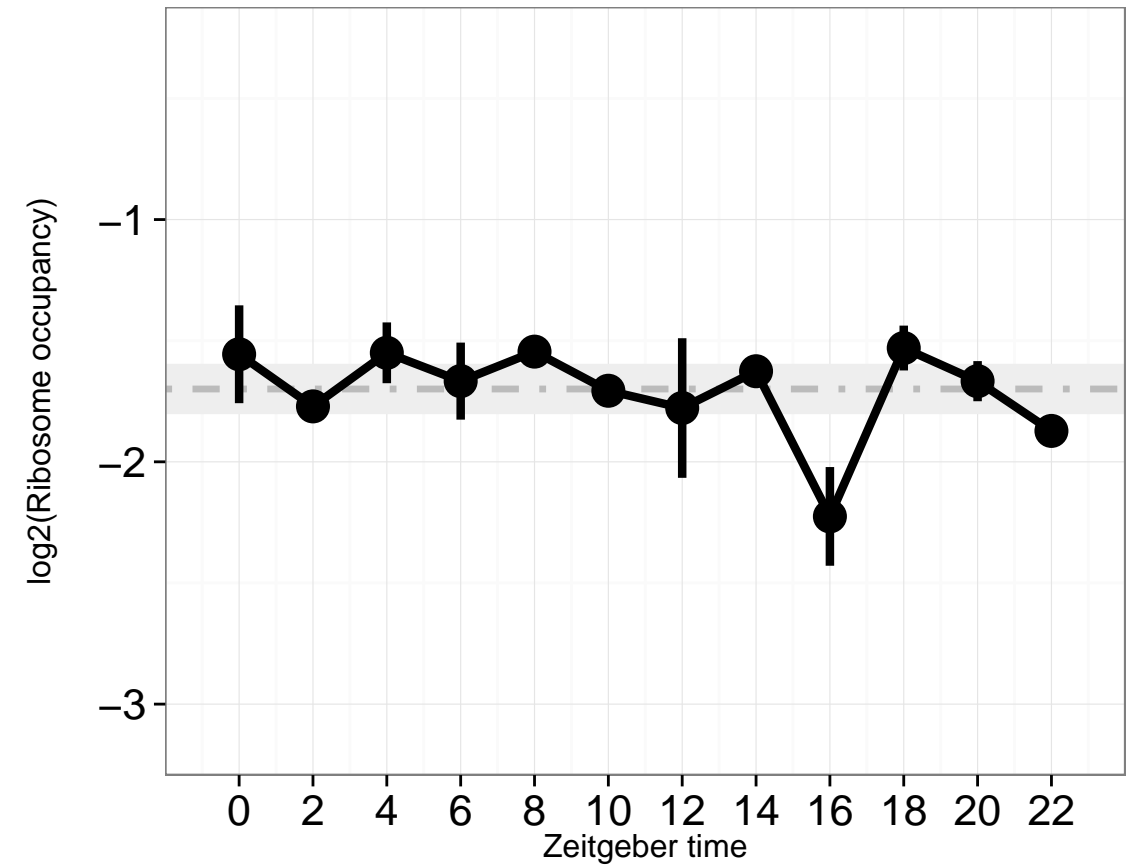

Supplement: Supplementary file 7 — Expression plots for kidney and liver for the 178 common rhythmic genes of Fig. 3c. (ZIP 3338.28 kb) [file 13059_2017_1222_MOESM7_ESM.zip › set_D_shared(178)/Clock_kidney_set_D.pdf]

## Clock

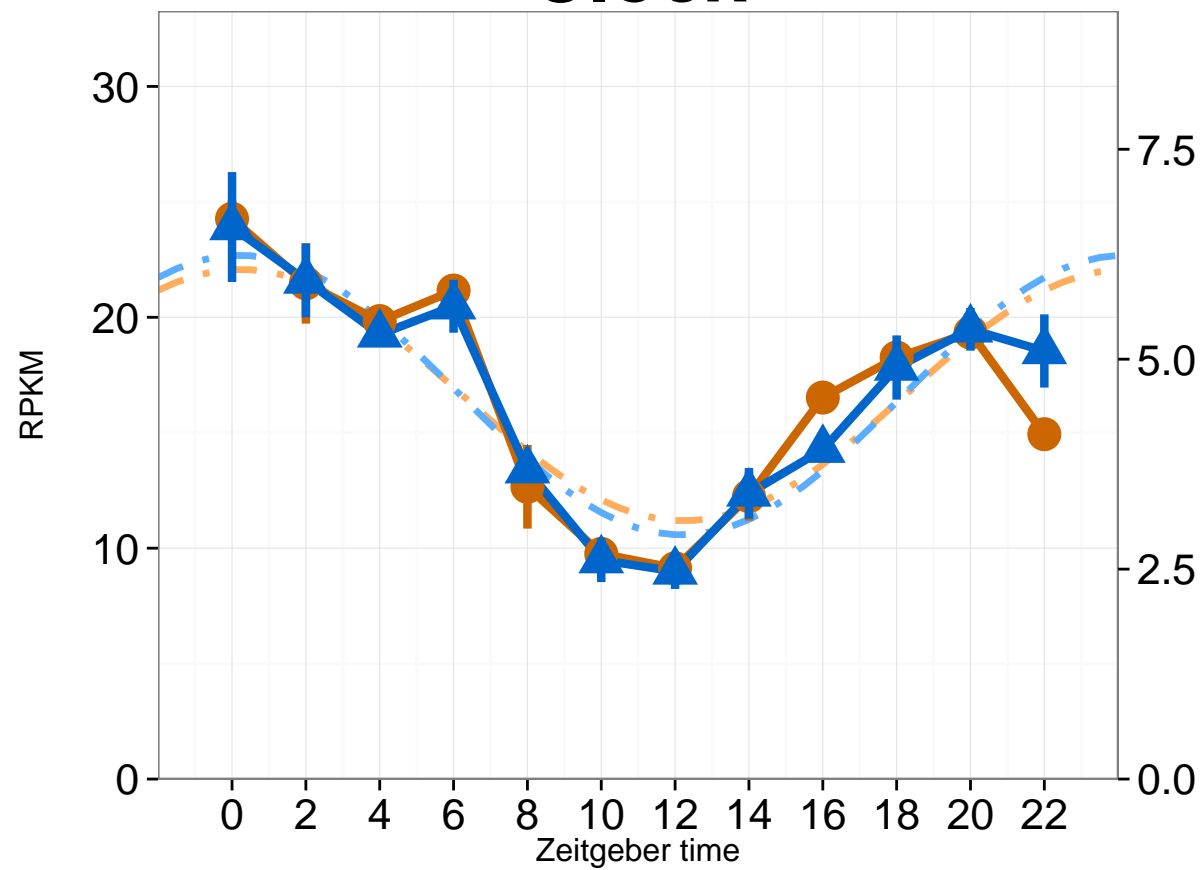

## Clock

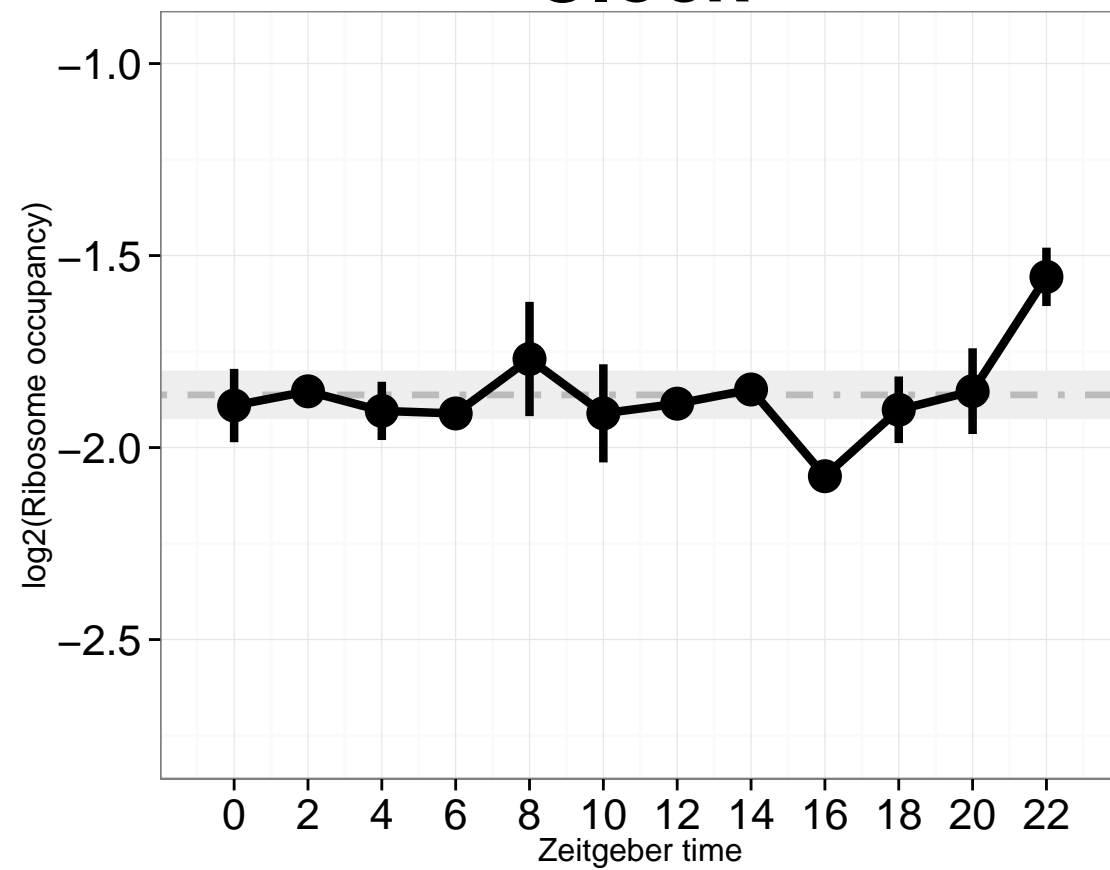

Supplement: Supplementary file 7 — Expression plots for kidney and liver for the 178 common rhythmic genes of Fig. 3c. (ZIP 3338.28 kb) [file 13059_2017_1222_MOESM7_ESM.zip › set_D_shared(178)/Clock_liver_set_D.pdf]

## Clpx

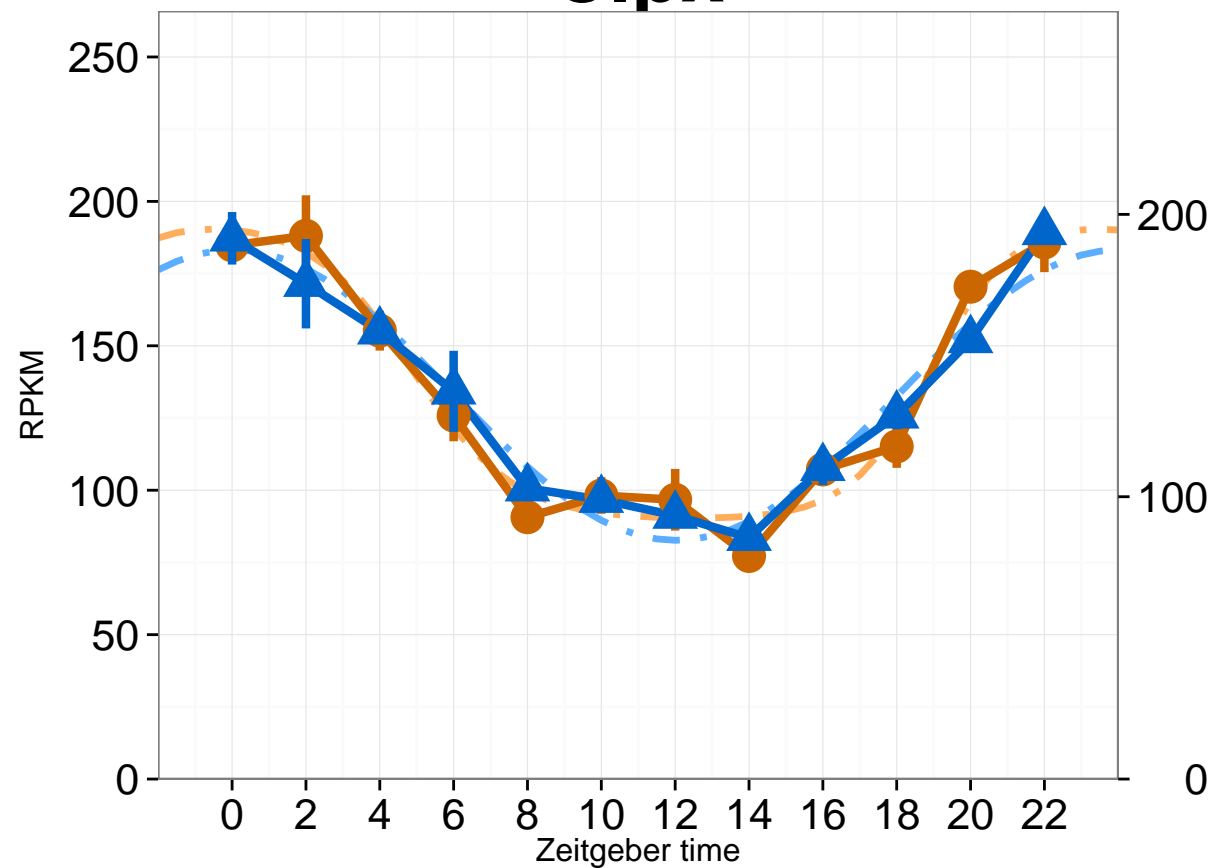

## Clpx

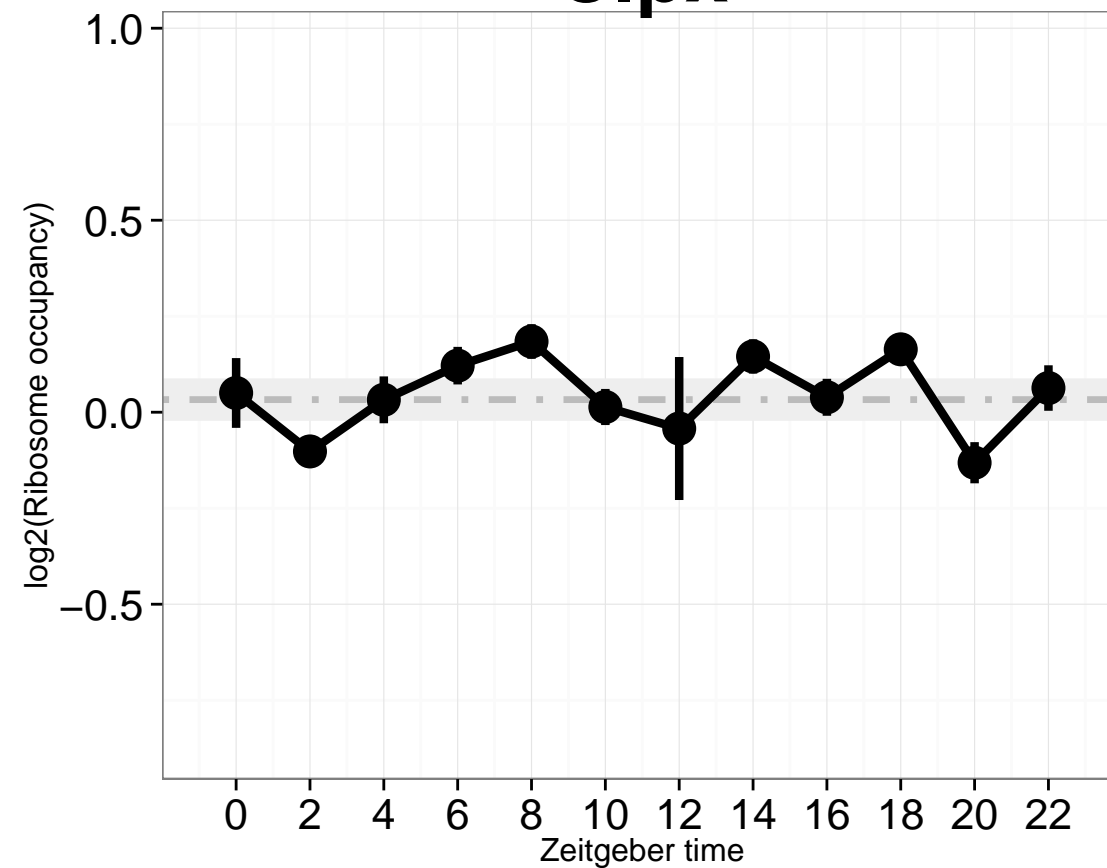

Supplement: Supplementary file 7 — Expression plots for kidney and liver for the 178 common rhythmic genes of Fig. 3c. (ZIP 3338.28 kb) [file 13059_2017_1222_MOESM7_ESM.zip › set_D_shared(178)/Clpx_kidney_set_D.pdf]

## Clpx

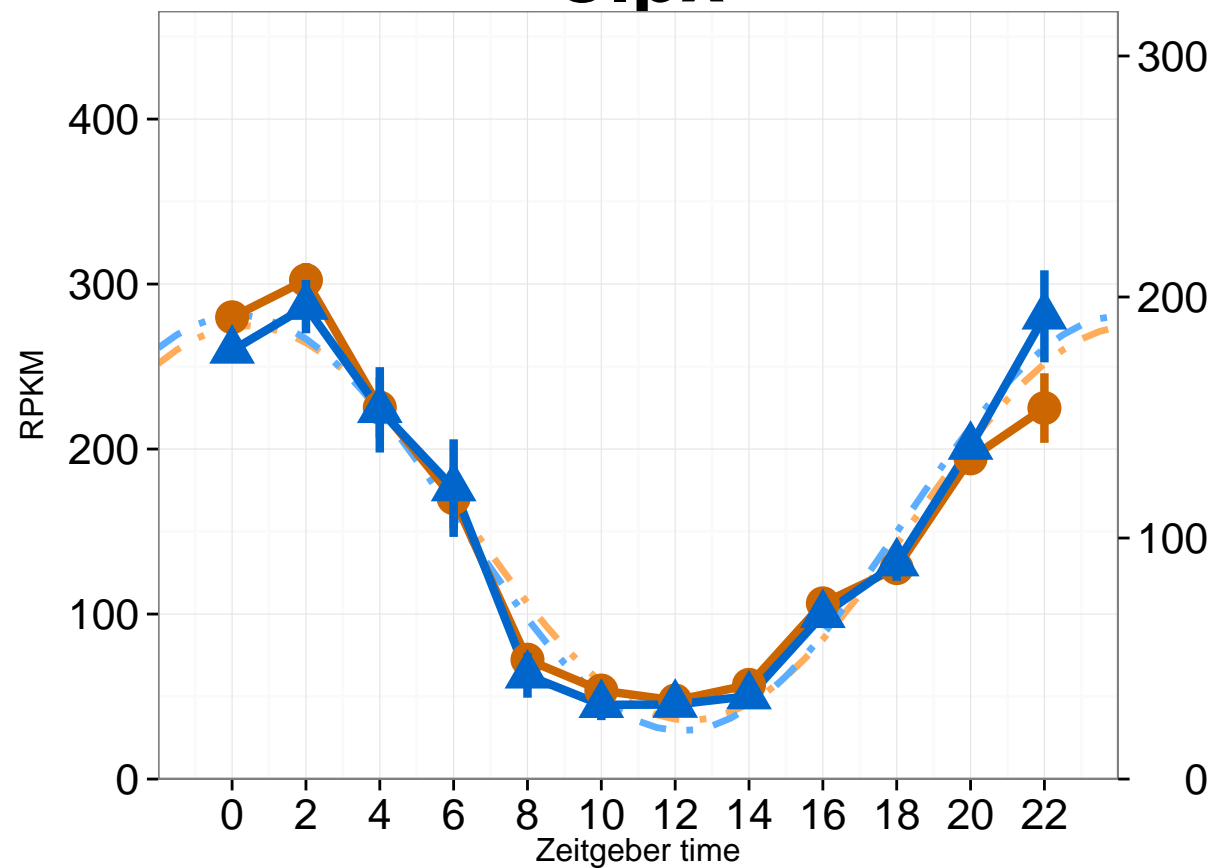

## Clpx

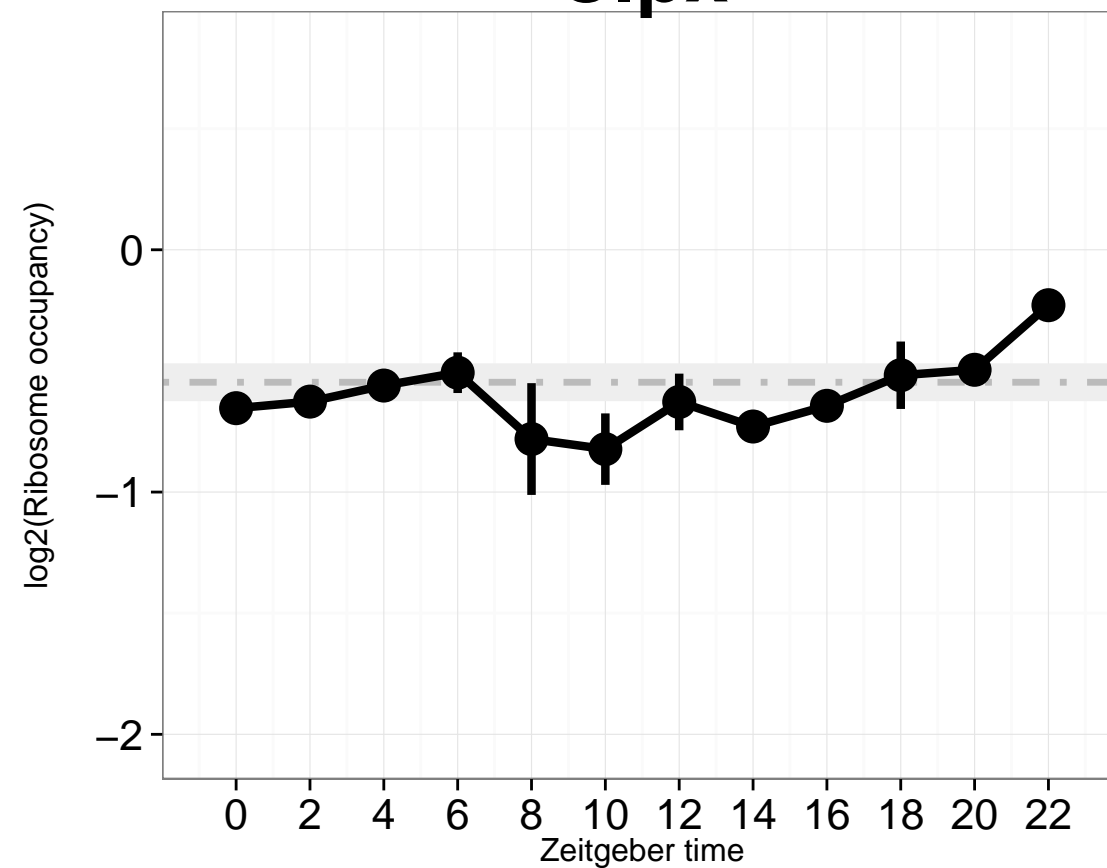

Supplement: Supplementary file 7 — Expression plots for kidney and liver for the 178 common rhythmic genes of Fig. 3c. (ZIP 3338.28 kb) [file 13059_2017_1222_MOESM7_ESM.zip › set_D_shared(178)/Clpx_liver_set_D.pdf]

## Cluh

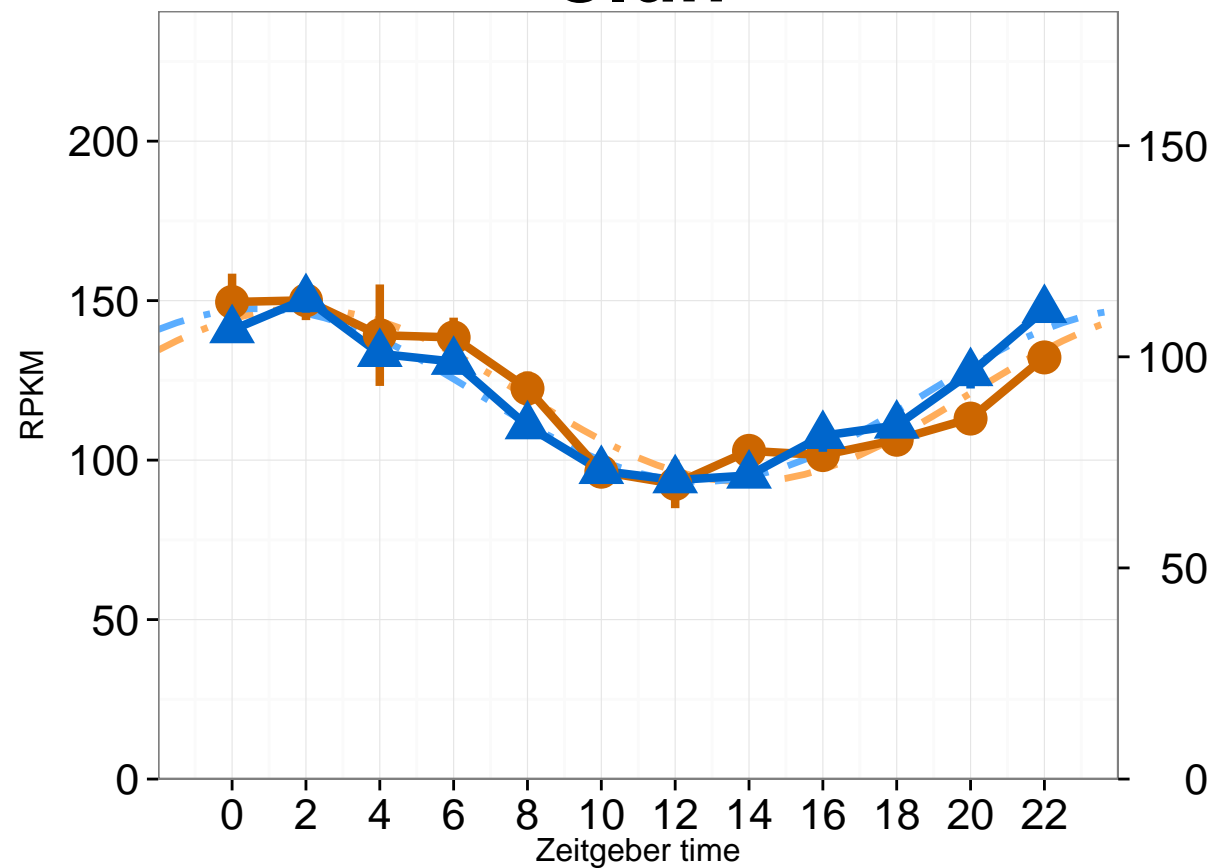

## Cluh

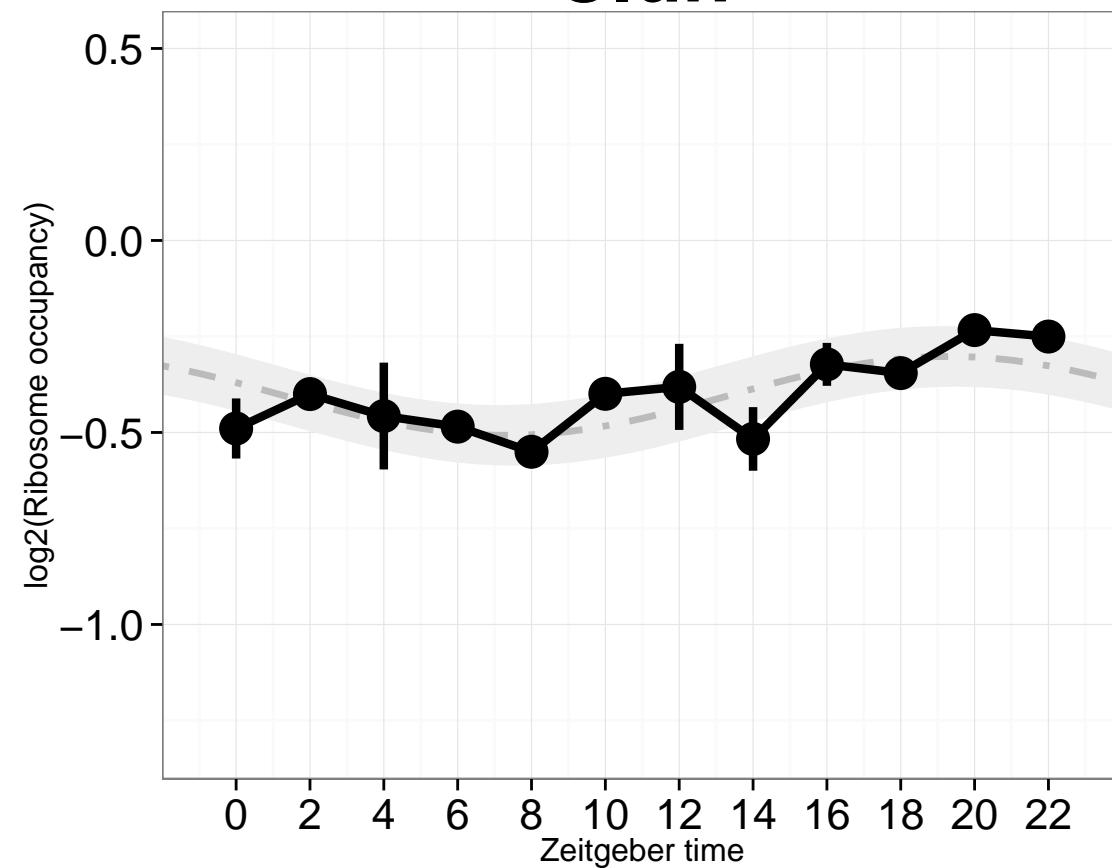

Supplement: Supplementary file 7 — Expression plots for kidney and liver for the 178 common rhythmic genes of Fig. 3c. (ZIP 3338.28 kb) [file 13059_2017_1222_MOESM7_ESM.zip › set_D_shared(178)/Cluh_kidney_set_D.pdf]

## Cluh

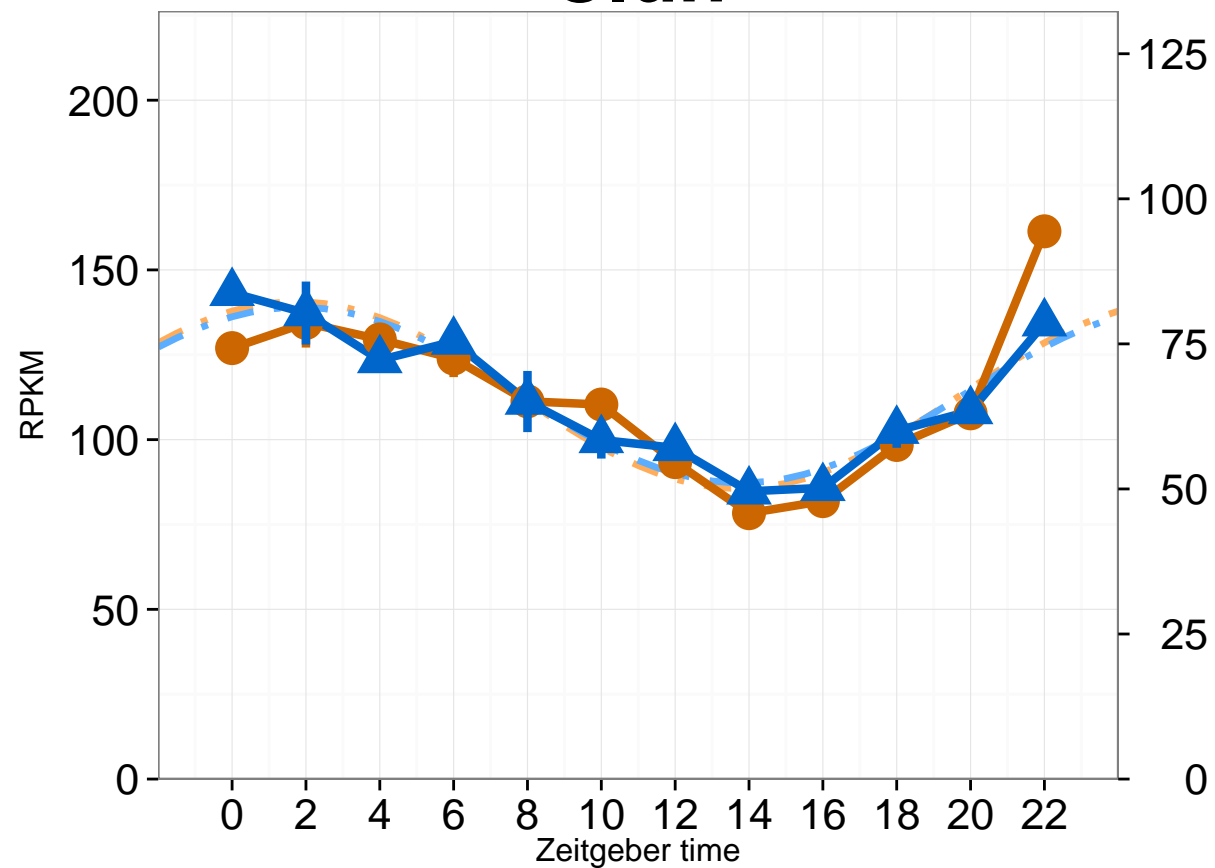

## Cluh

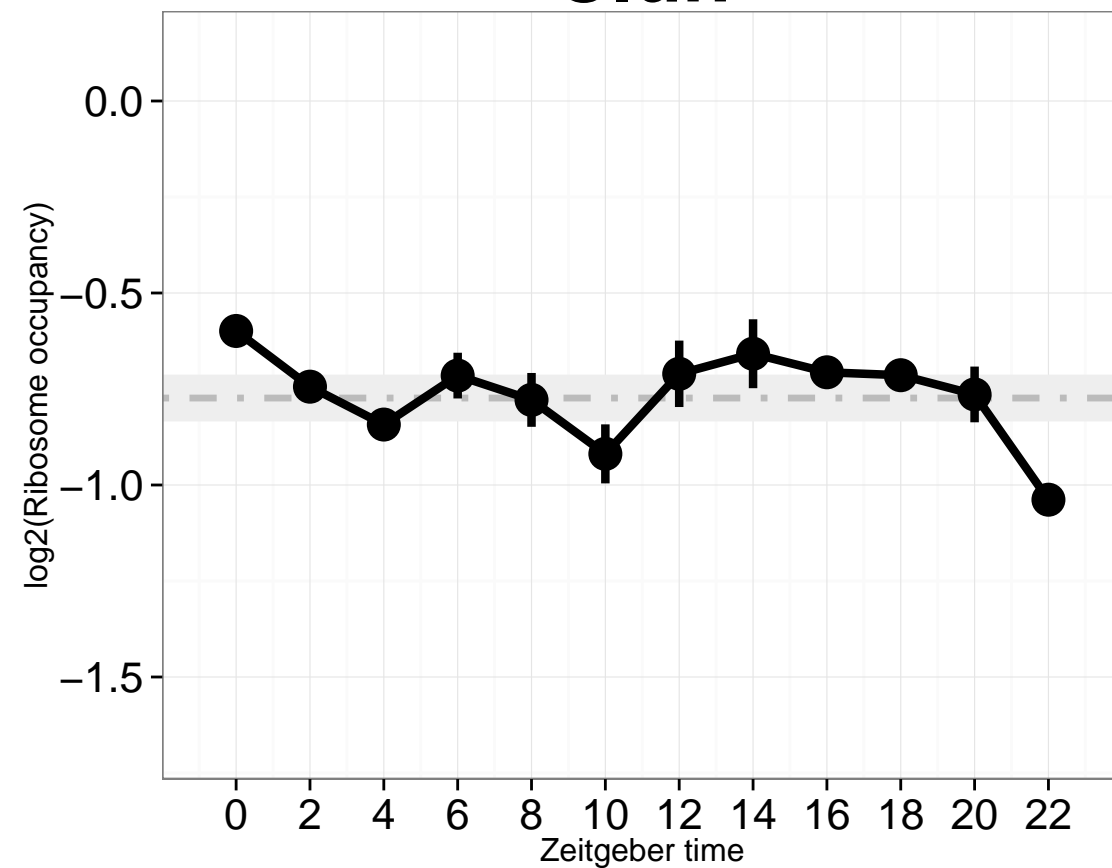

Supplement: Supplementary file 7 — Expression plots for kidney and liver for the 178 common rhythmic genes of Fig. 3c. (ZIP 3338.28 kb) [file 13059_2017_1222_MOESM7_ESM.zip › set_D_shared(178)/Cluh_liver_set_D.pdf]

## Cml5

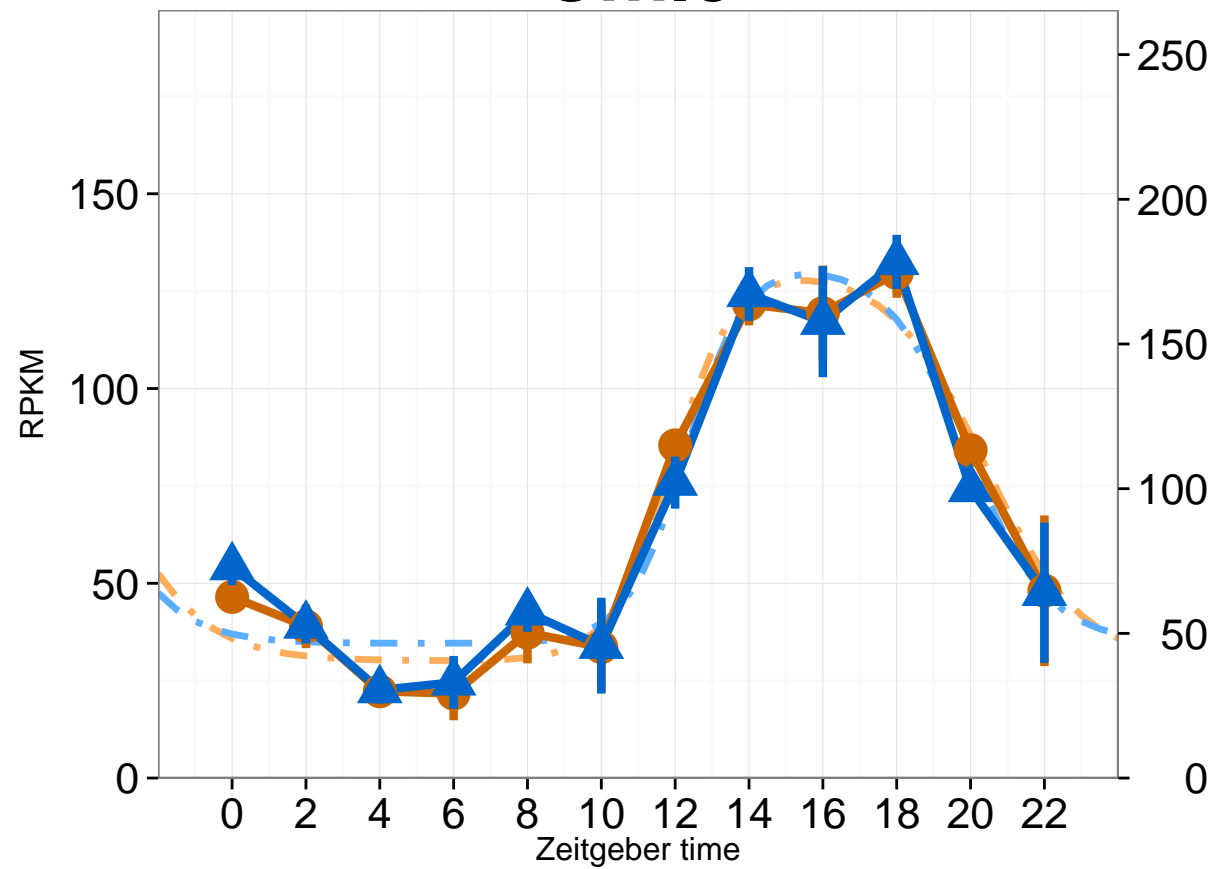

## Cml5

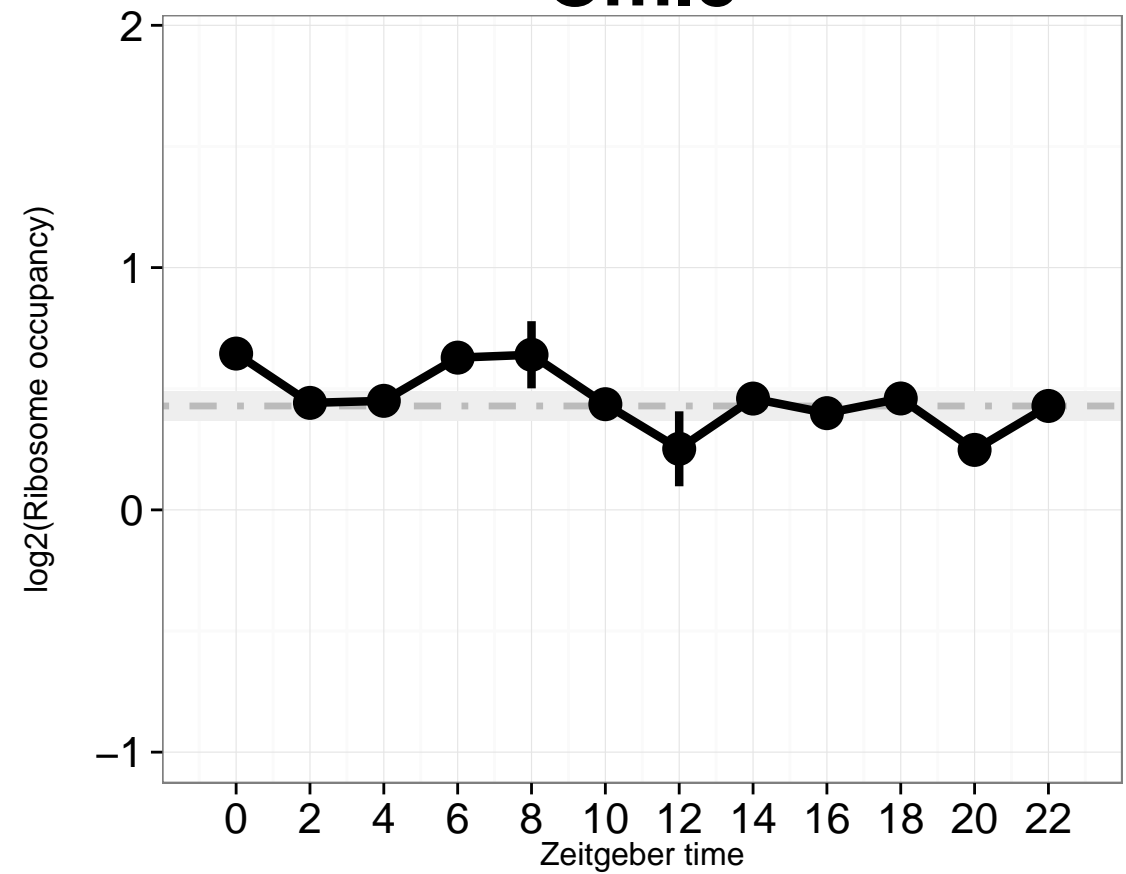

Supplement: Supplementary file 7 — Expression plots for kidney and liver for the 178 common rhythmic genes of Fig. 3c. (ZIP 3338.28 kb) [file 13059_2017_1222_MOESM7_ESM.zip › set_D_shared(178)/Cml5_kidney_set_D.pdf]

## Cml5

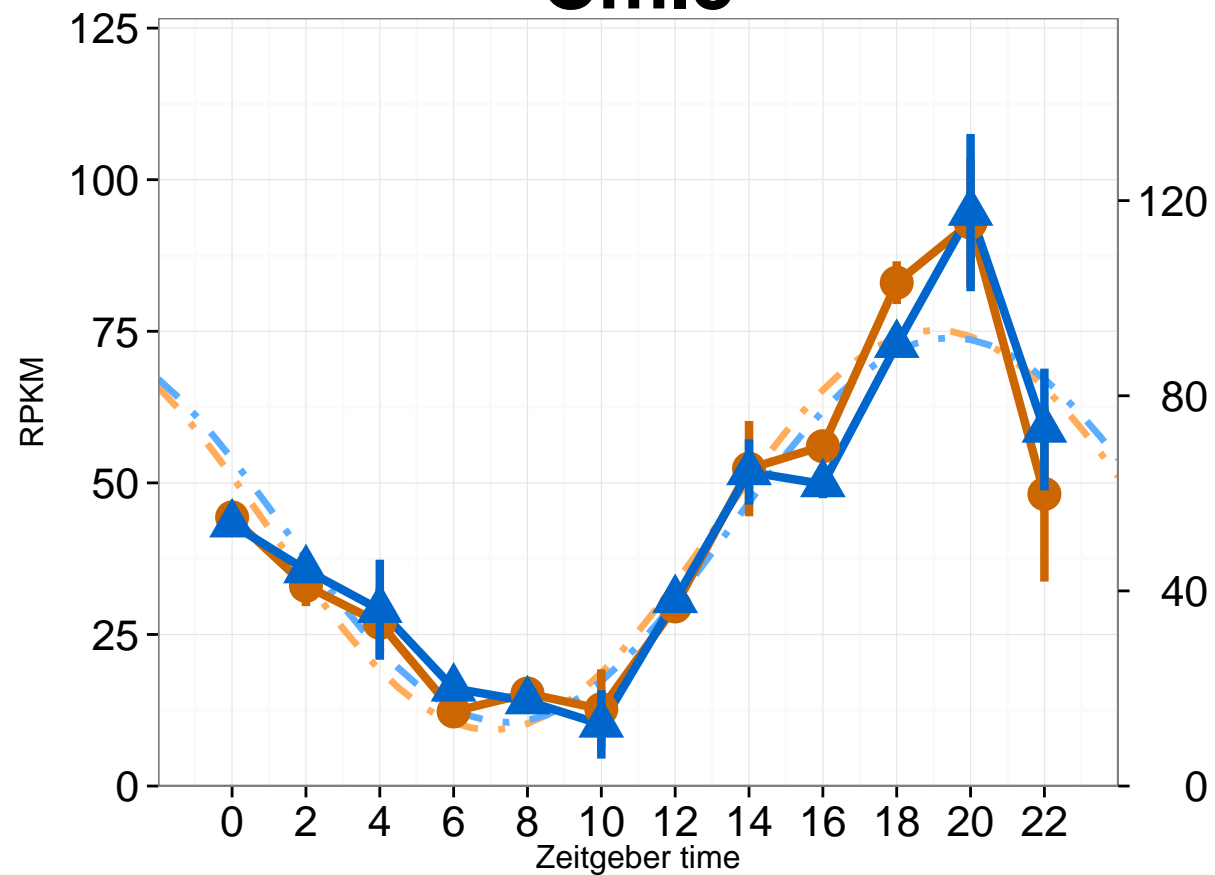

## Cml5

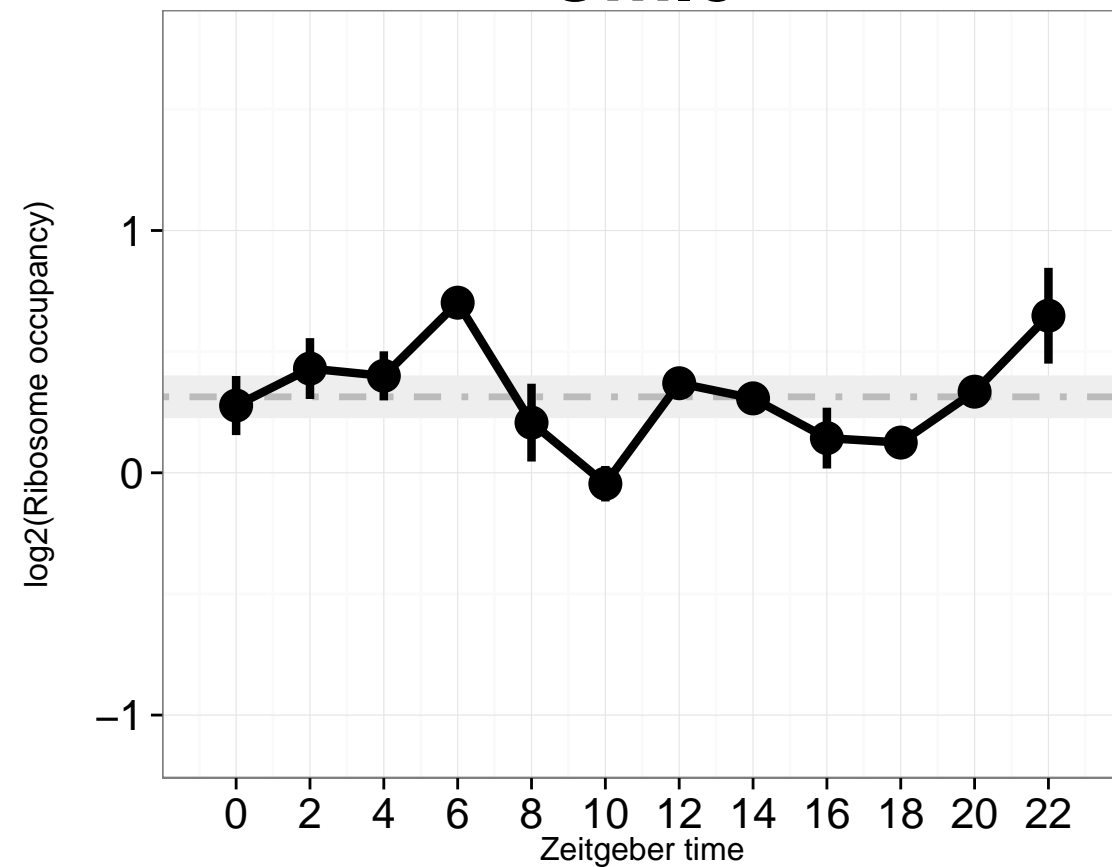

Supplement: Supplementary file 7 — Expression plots for kidney and liver for the 178 common rhythmic genes of Fig. 3c. (ZIP 3338.28 kb) [file 13059_2017_1222_MOESM7_ESM.zip › set_D_shared(178)/Cml5_liver_set_D.pdf]

# Col27a1

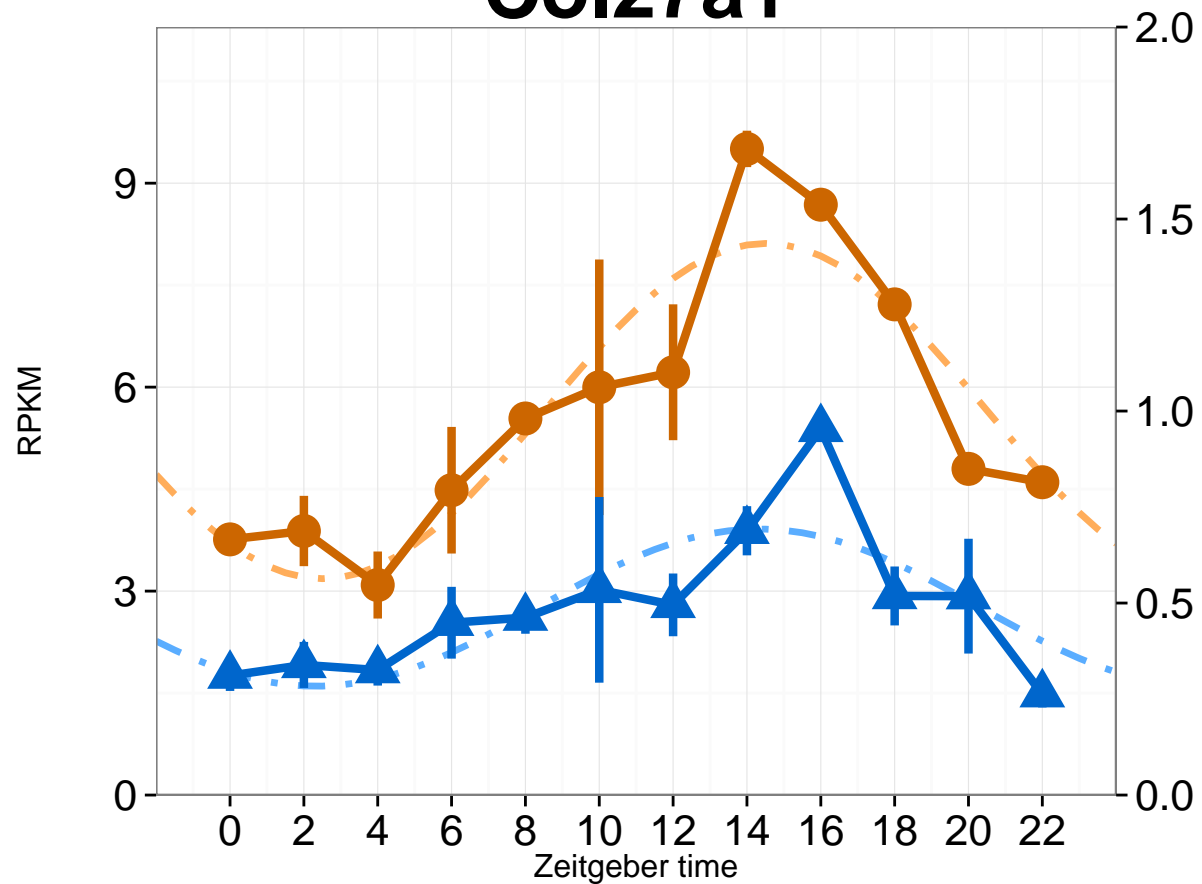

# Col27a1

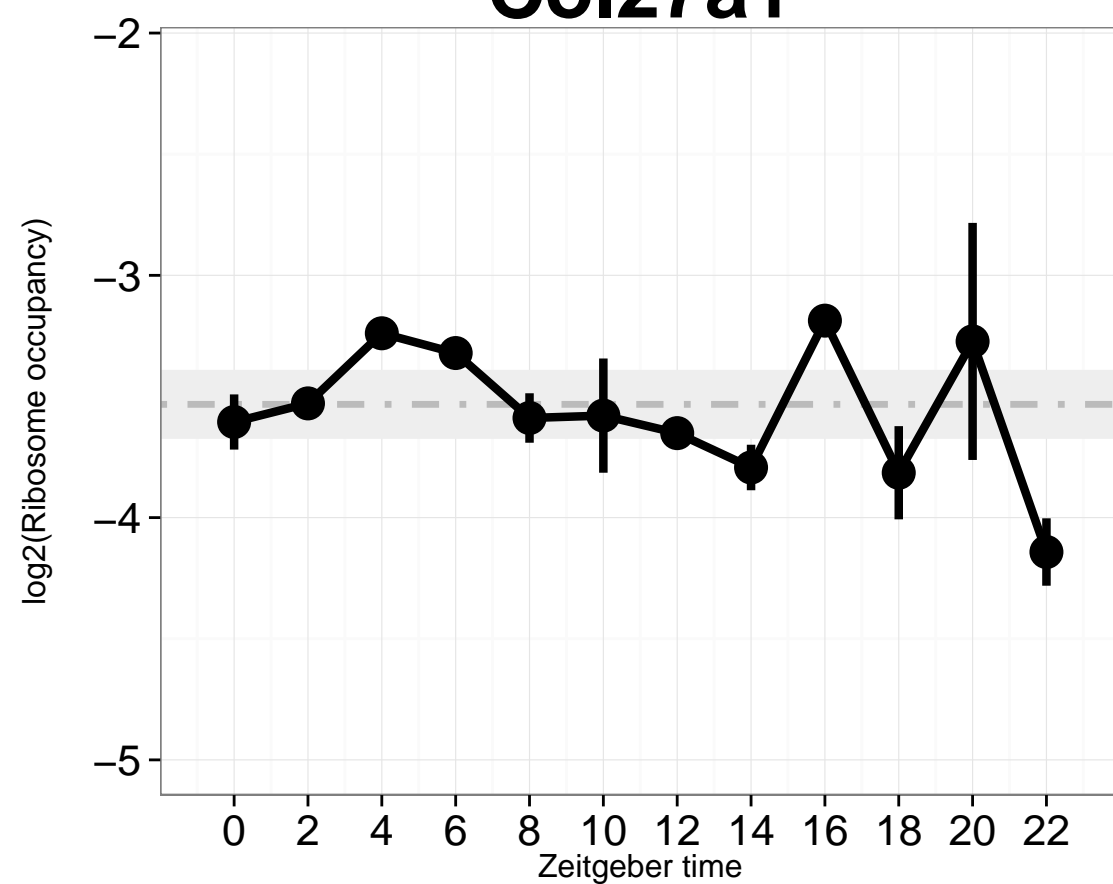

Supplement: Supplementary file 7 — Expression plots for kidney and liver for the 178 common rhythmic genes of Fig. 3c. (ZIP 3338.28 kb) [file 13059_2017_1222_MOESM7_ESM.zip › set_D_shared(178)/Col27a1_kidney_set_D.pdf]

# Col27a1

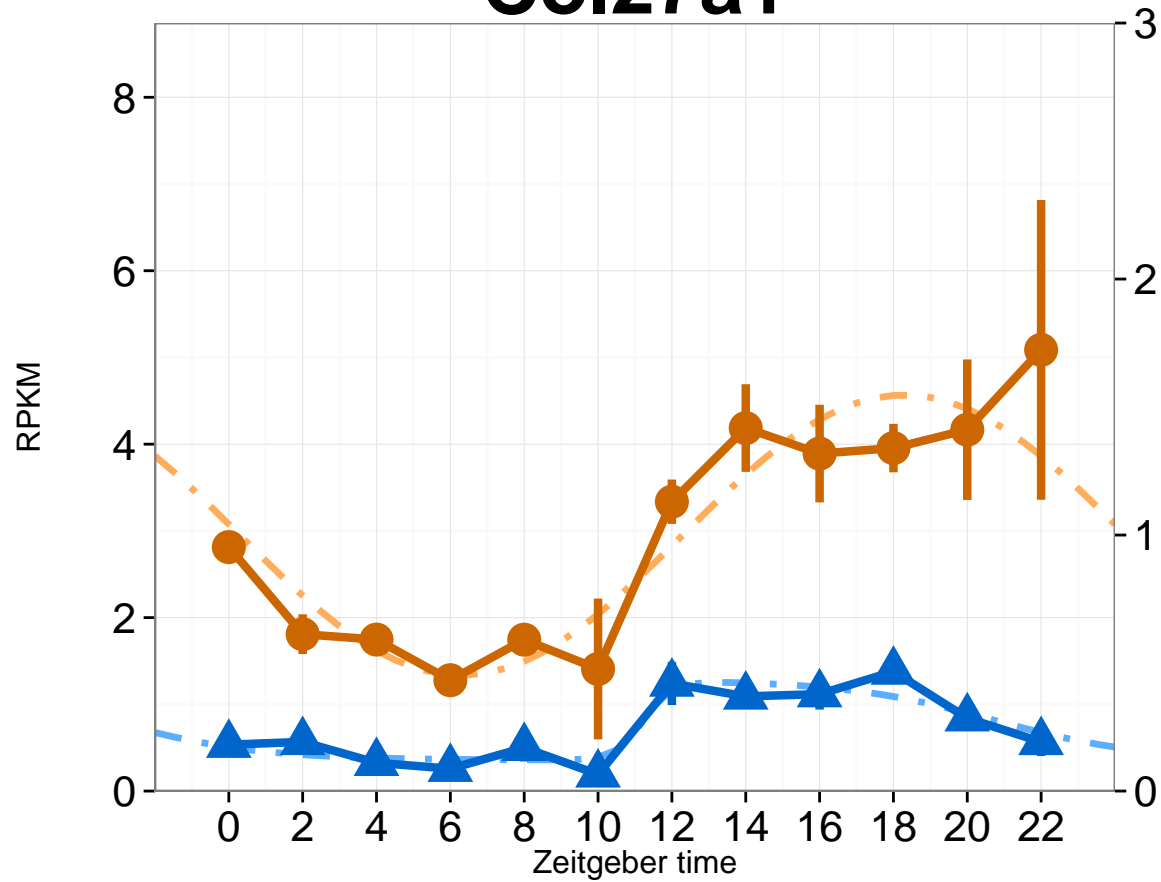

# Col27a1

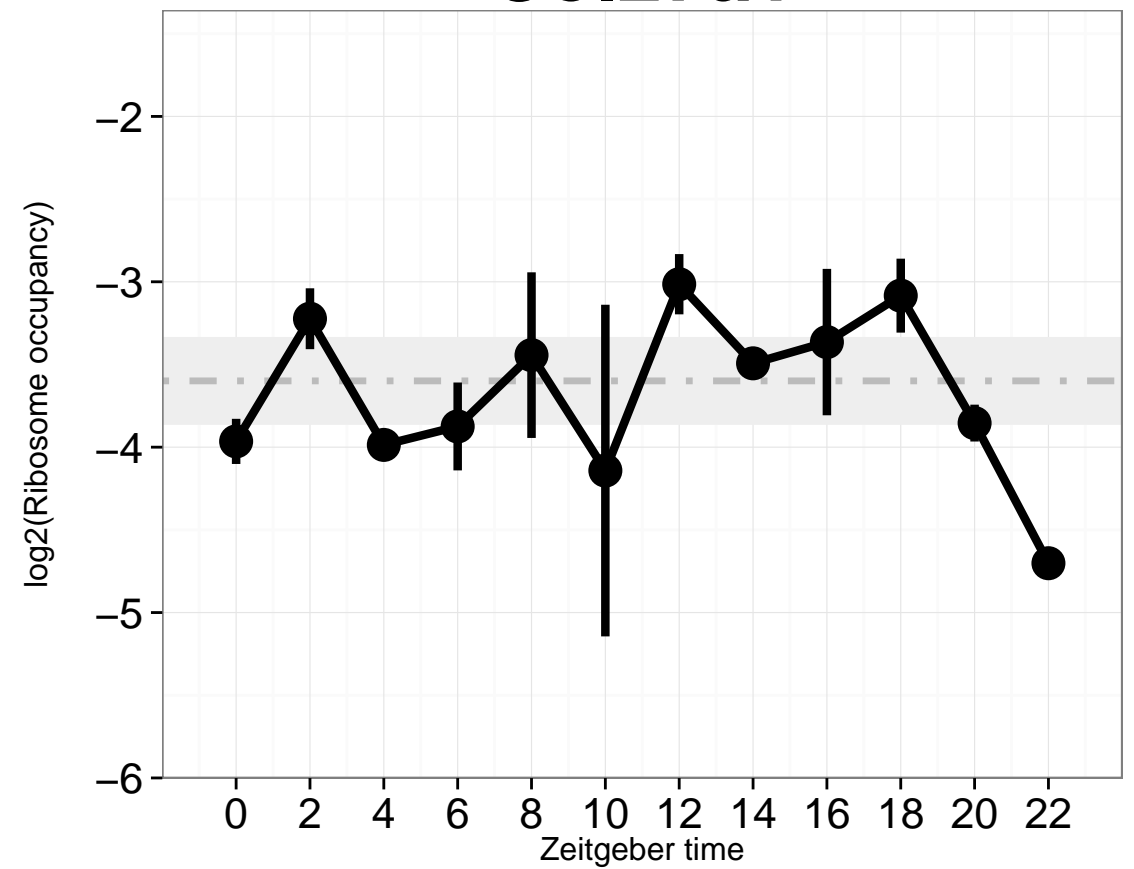

Supplement: Supplementary file 7 — Expression plots for kidney and liver for the 178 common rhythmic genes of Fig. 3c. (ZIP 3338.28 kb) [file 13059_2017_1222_MOESM7_ESM.zip › set_D_shared(178)/Col27a1_liver_set_D.pdf]

# Col5a3

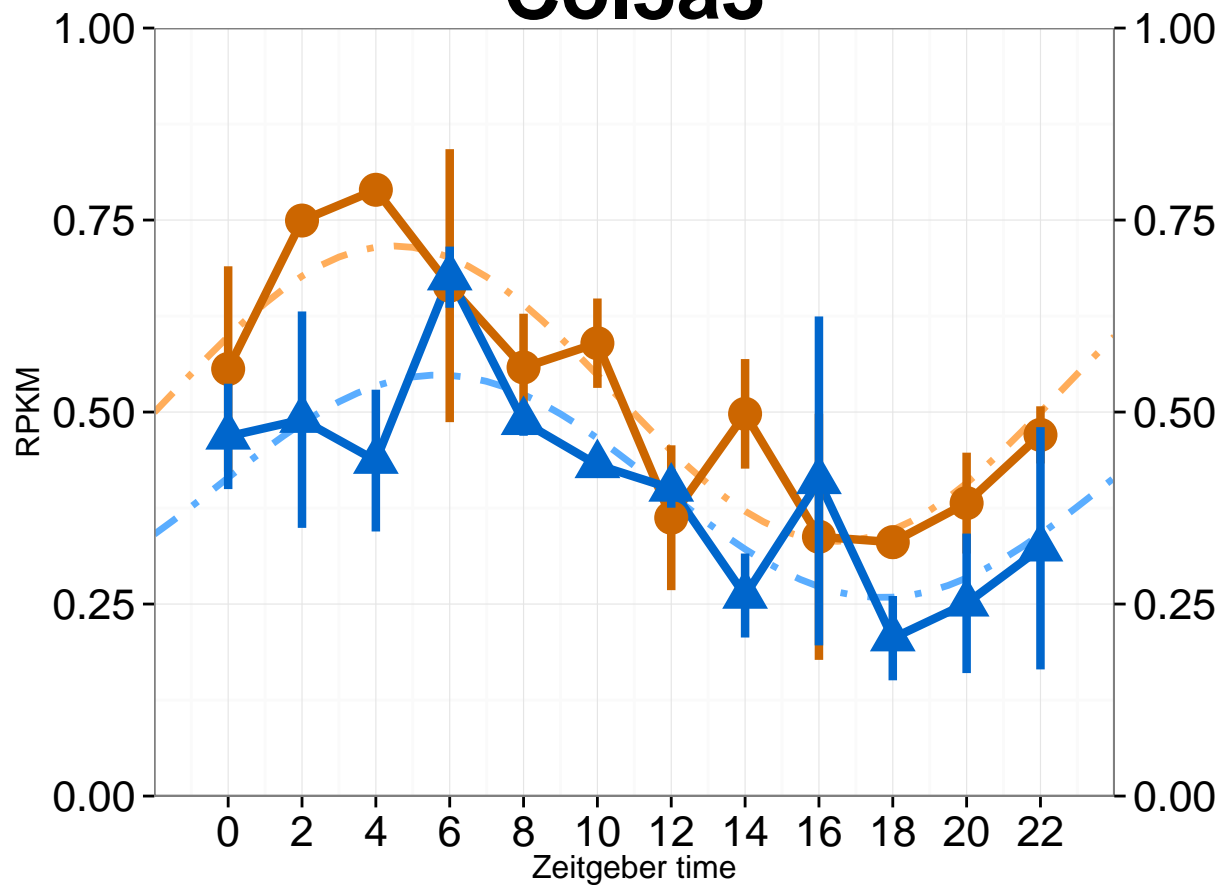

# Col5a3

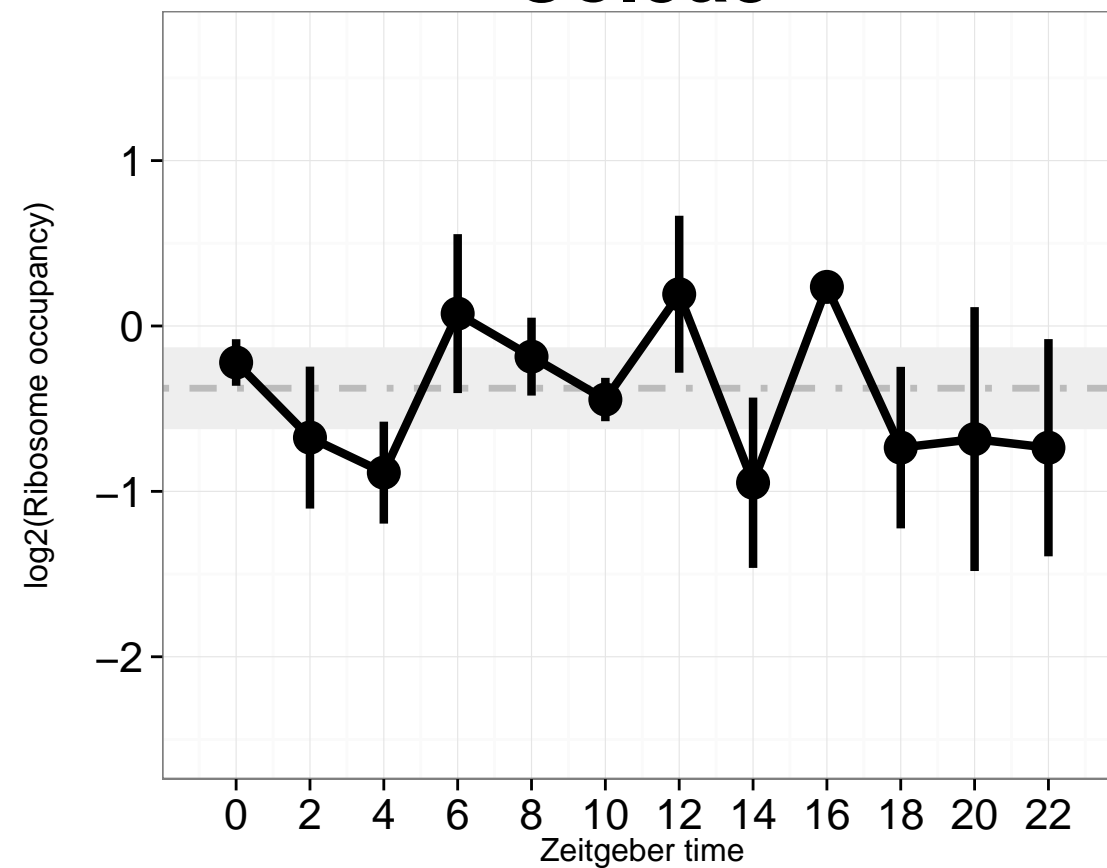

Supplement: Supplementary file 7 — Expression plots for kidney and liver for the 178 common rhythmic genes of Fig. 3c. (ZIP 3338.28 kb) [file 13059_2017_1222_MOESM7_ESM.zip › set_D_shared(178)/Col5a3_kidney_set_D.pdf]

# Col5a3

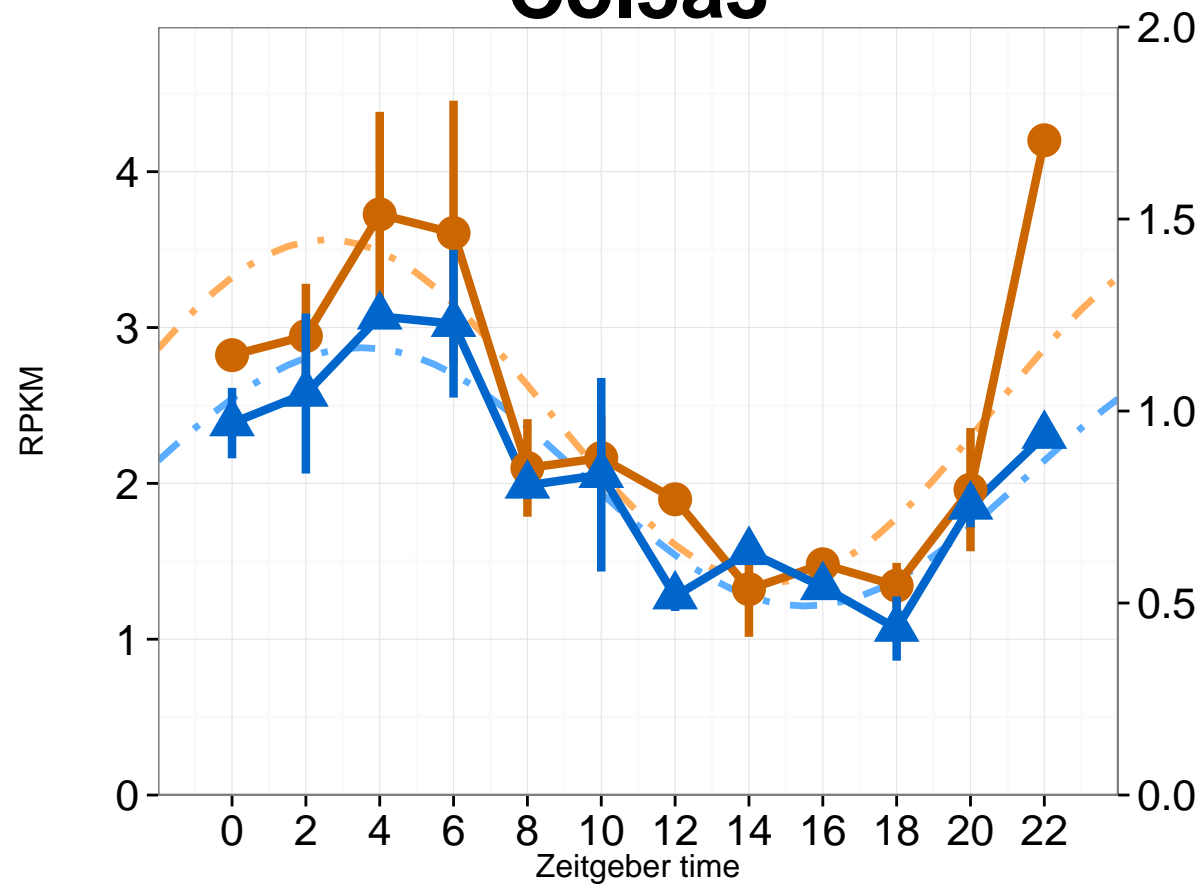

# Col5a3

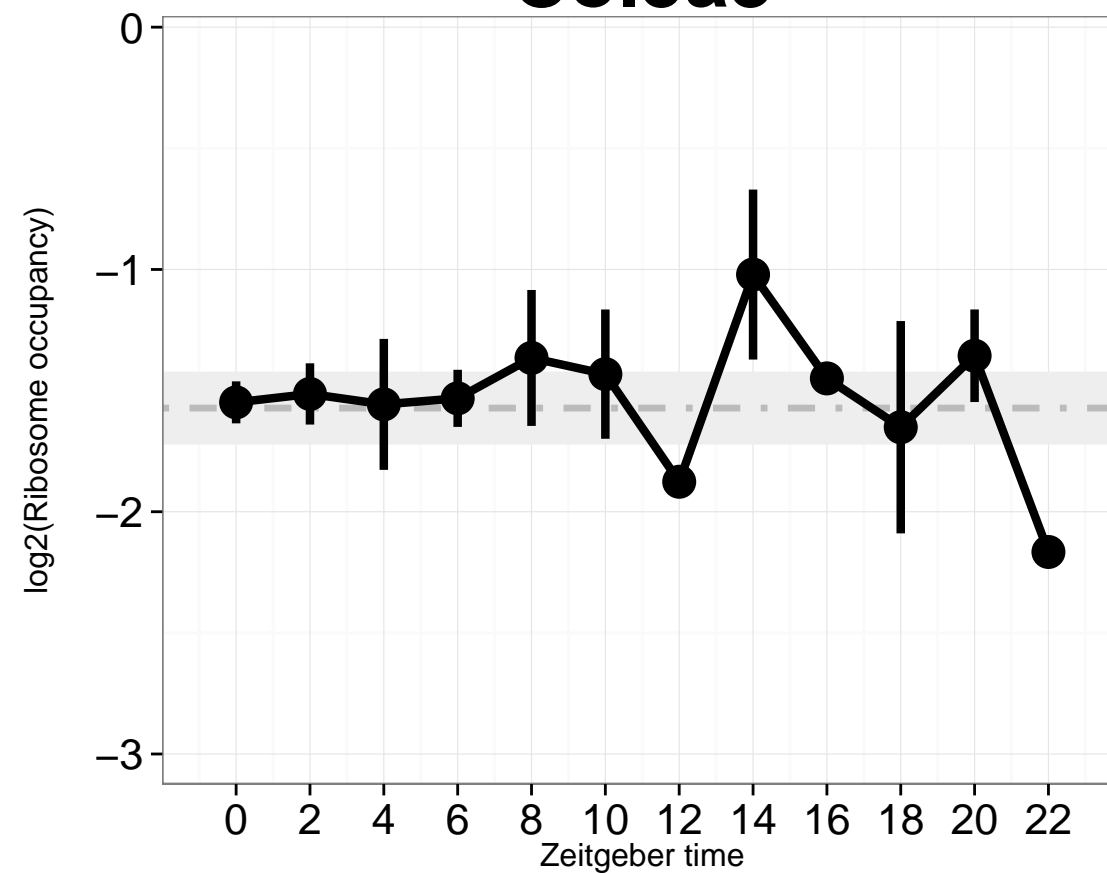

Supplement: Supplementary file 7 — Expression plots for kidney and liver for the 178 common rhythmic genes of Fig. 3c. (ZIP 3338.28 kb) [file 13059_2017_1222_MOESM7_ESM.zip › set_D_shared(178)/Col5a3_liver_set_D.pdf]

# Coq10b

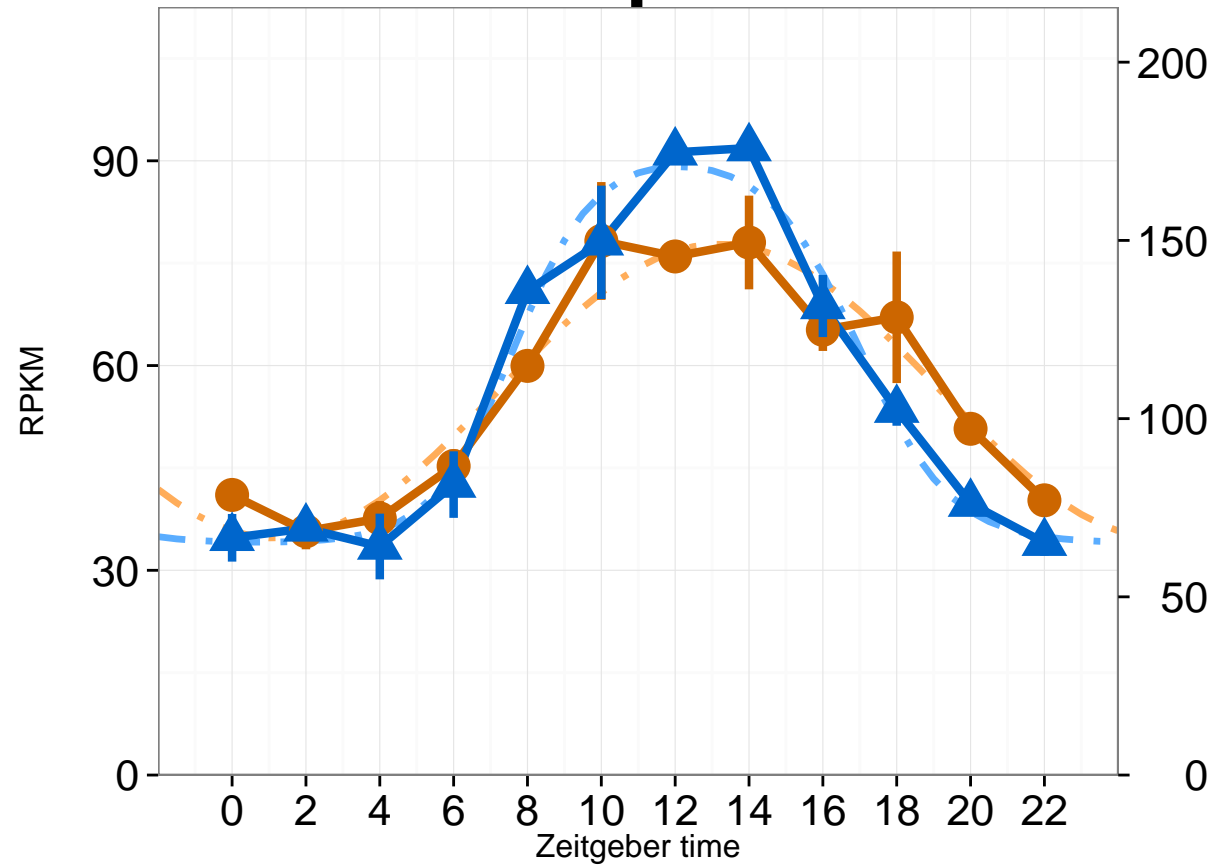

# Coq10b

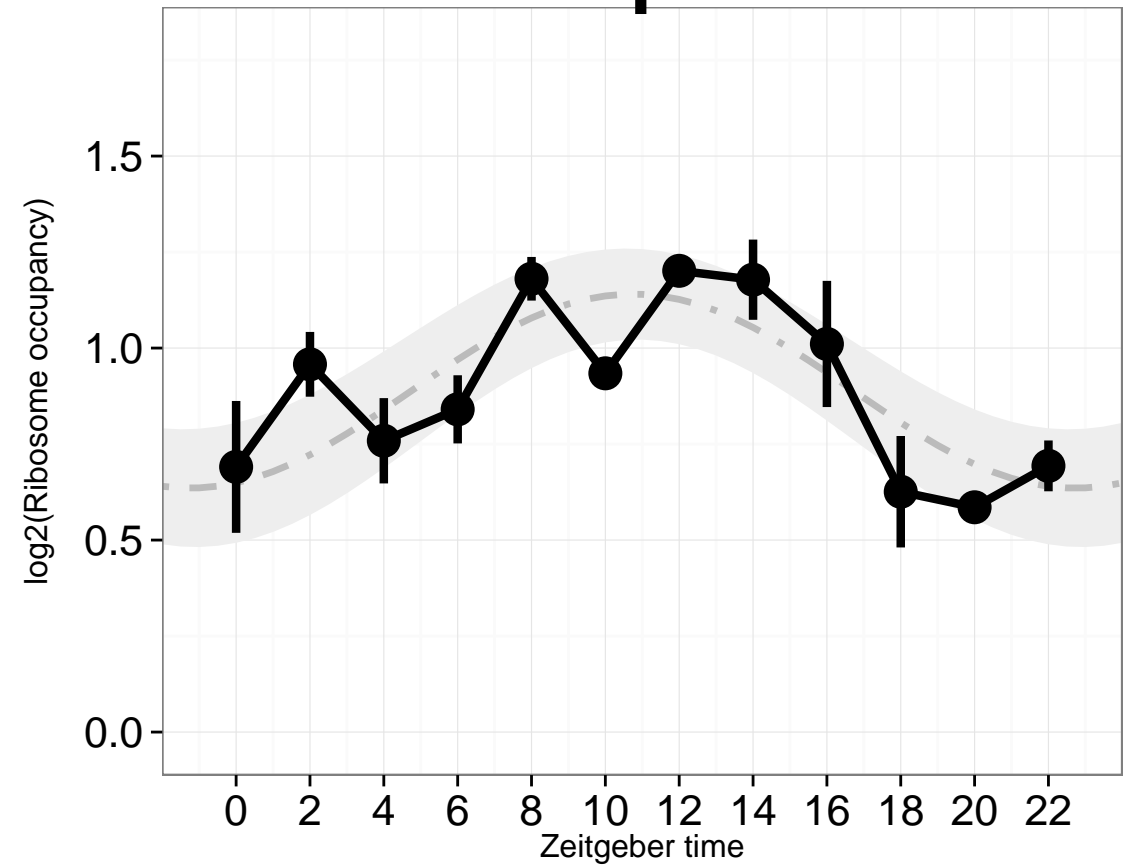

Supplement: Supplementary file 7 — Expression plots for kidney and liver for the 178 common rhythmic genes of Fig. 3c. (ZIP 3338.28 kb) [file 13059_2017_1222_MOESM7_ESM.zip › set_D_shared(178)/Coq10b_kidney_set_D.pdf]

# Coq10b

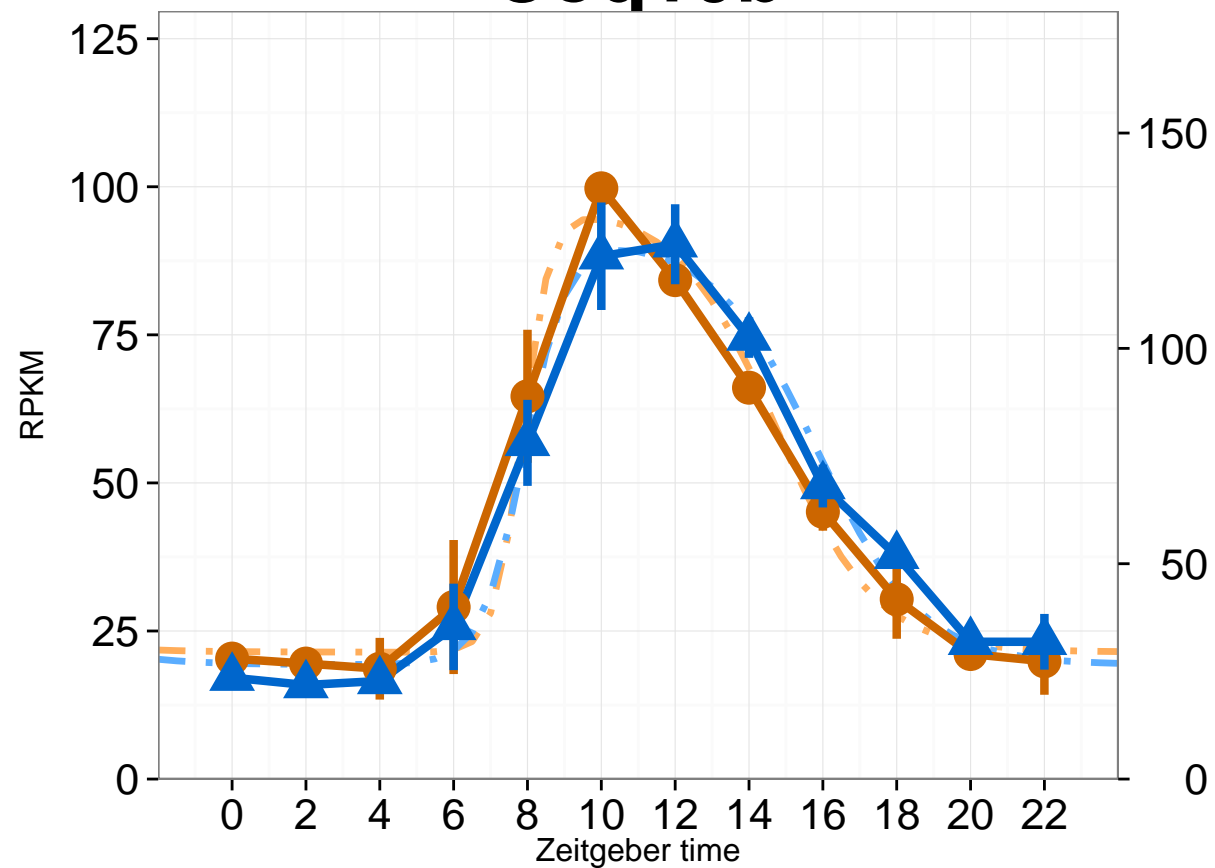

# Coq10b

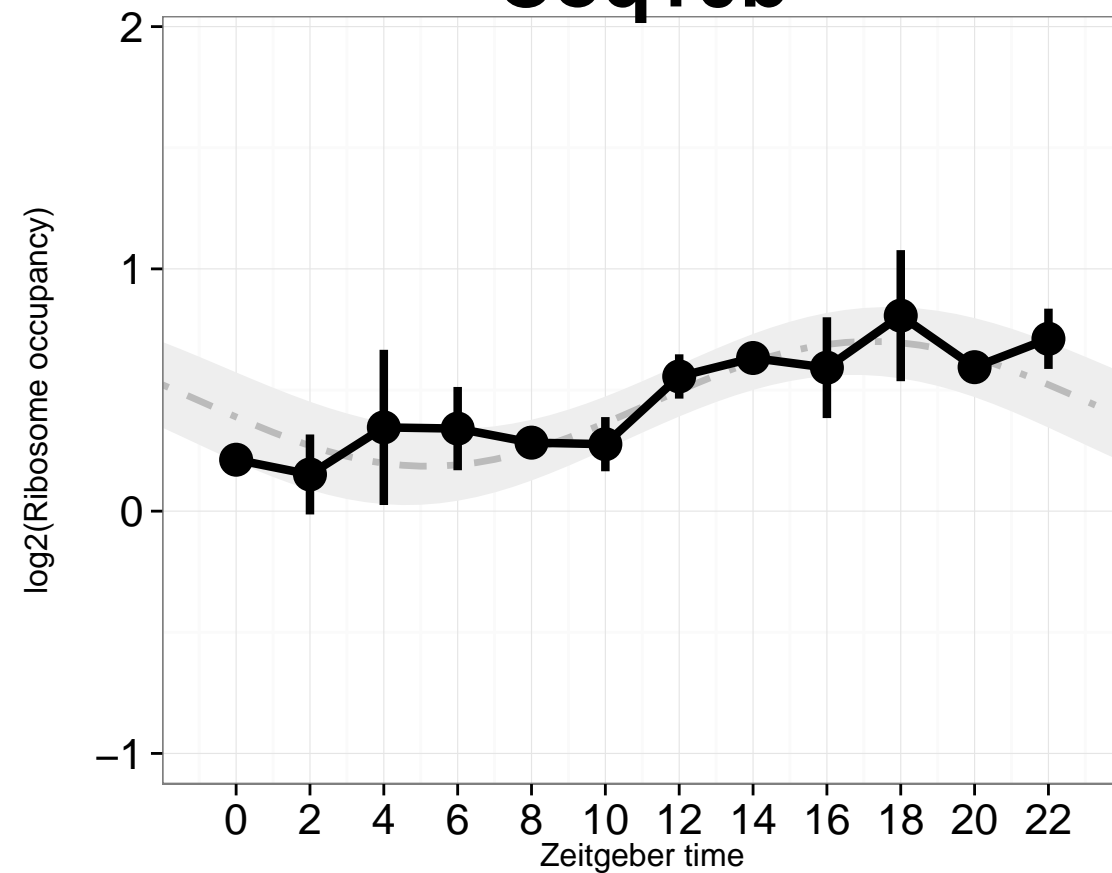

Supplement: Supplementary file 7 — Expression plots for kidney and liver for the 178 common rhythmic genes of Fig. 3c. (ZIP 3338.28 kb) [file 13059_2017_1222_MOESM7_ESM.zip › set_D_shared(178)/Coq10b_liver_set_D.pdf]

## Cry1

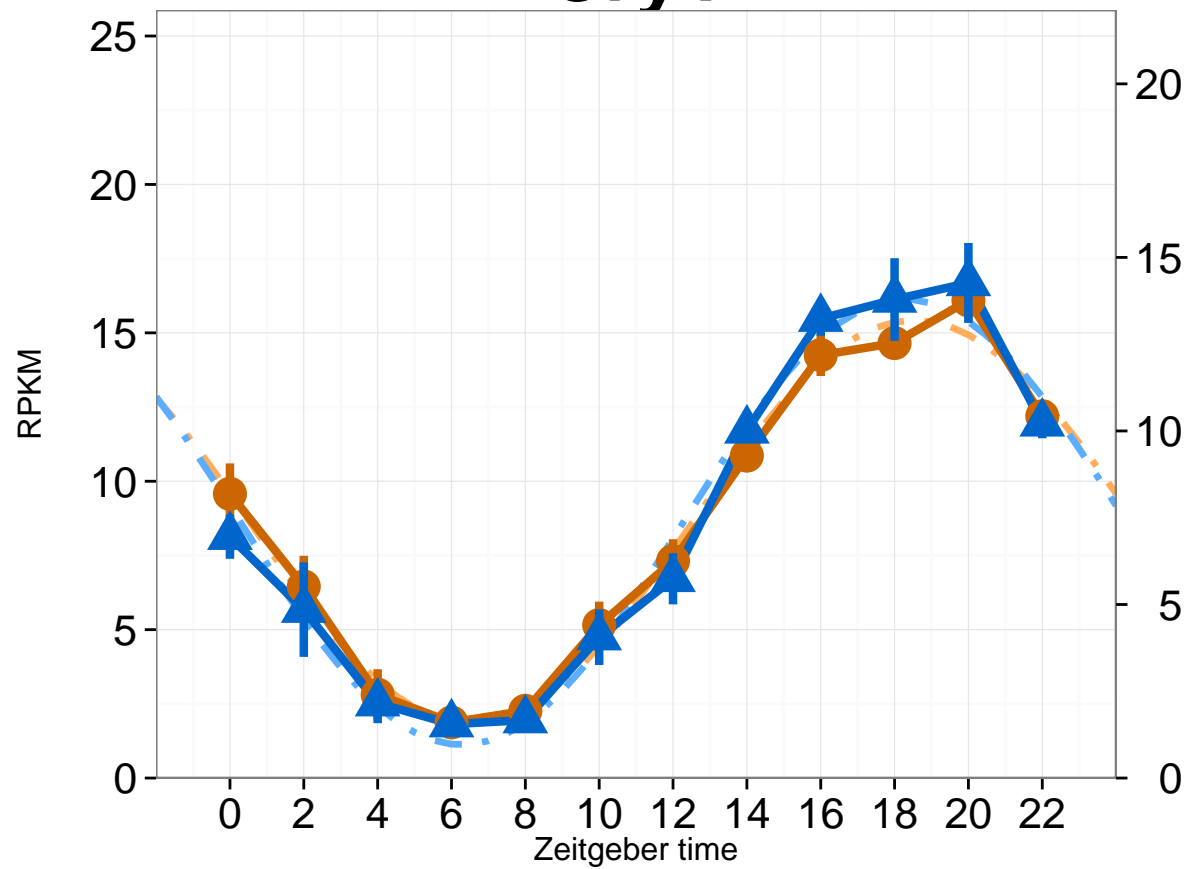

## Cry1

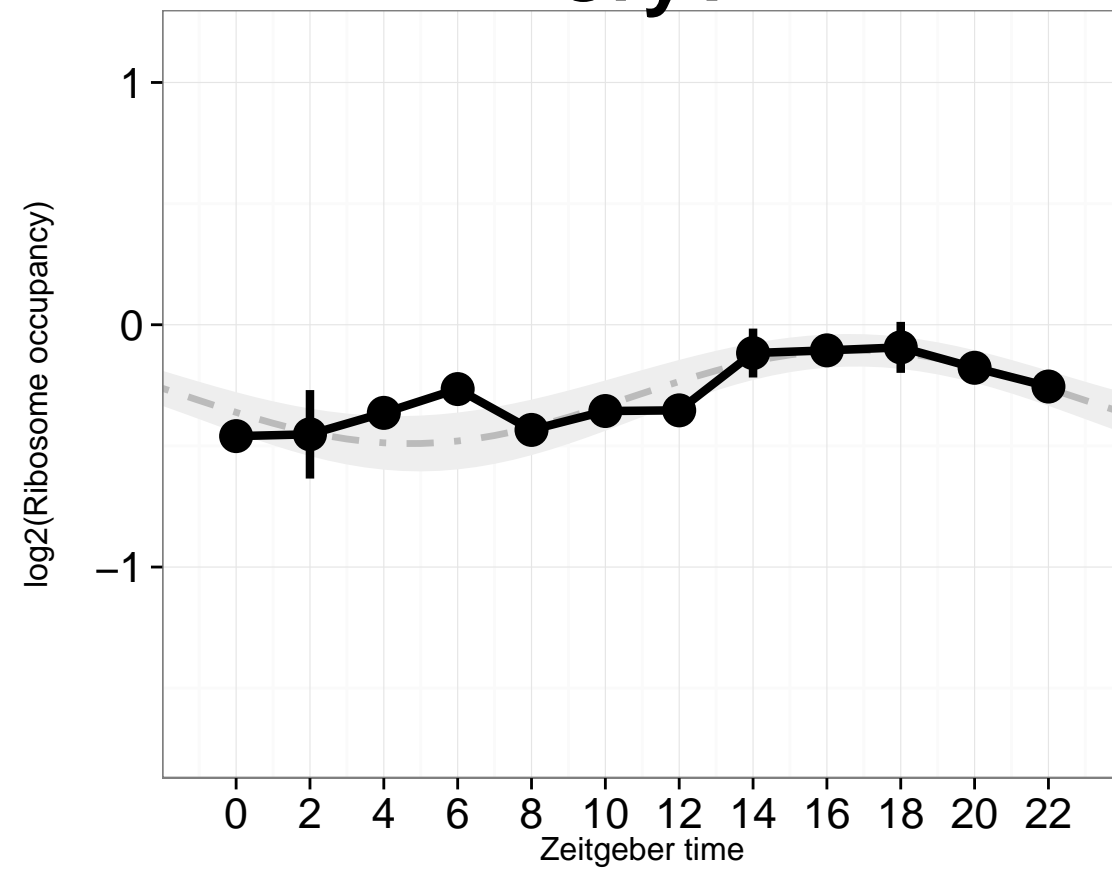

Supplement: Supplementary file 7 — Expression plots for kidney and liver for the 178 common rhythmic genes of Fig. 3c. (ZIP 3338.28 kb) [file 13059_2017_1222_MOESM7_ESM.zip › set_D_shared(178)/Cry1_kidney_set_D.pdf]

# Cry1

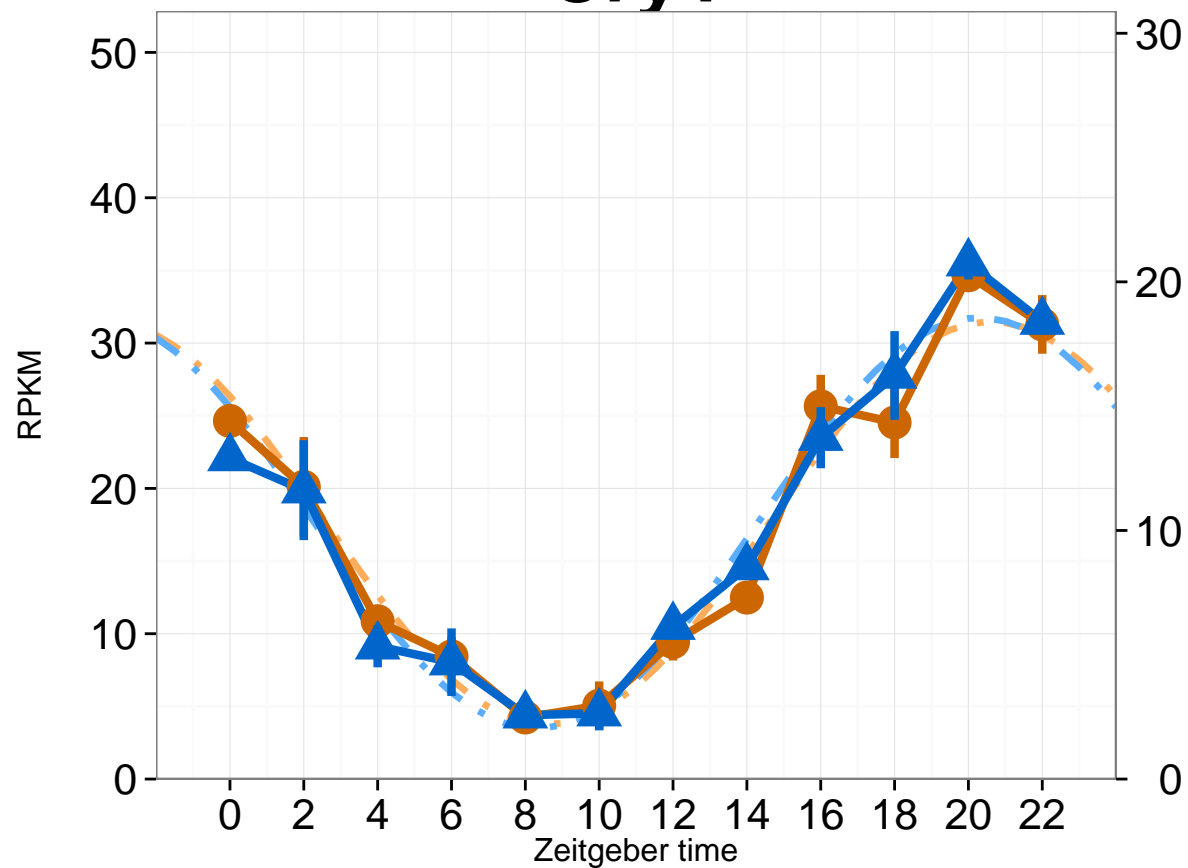

# Cry1

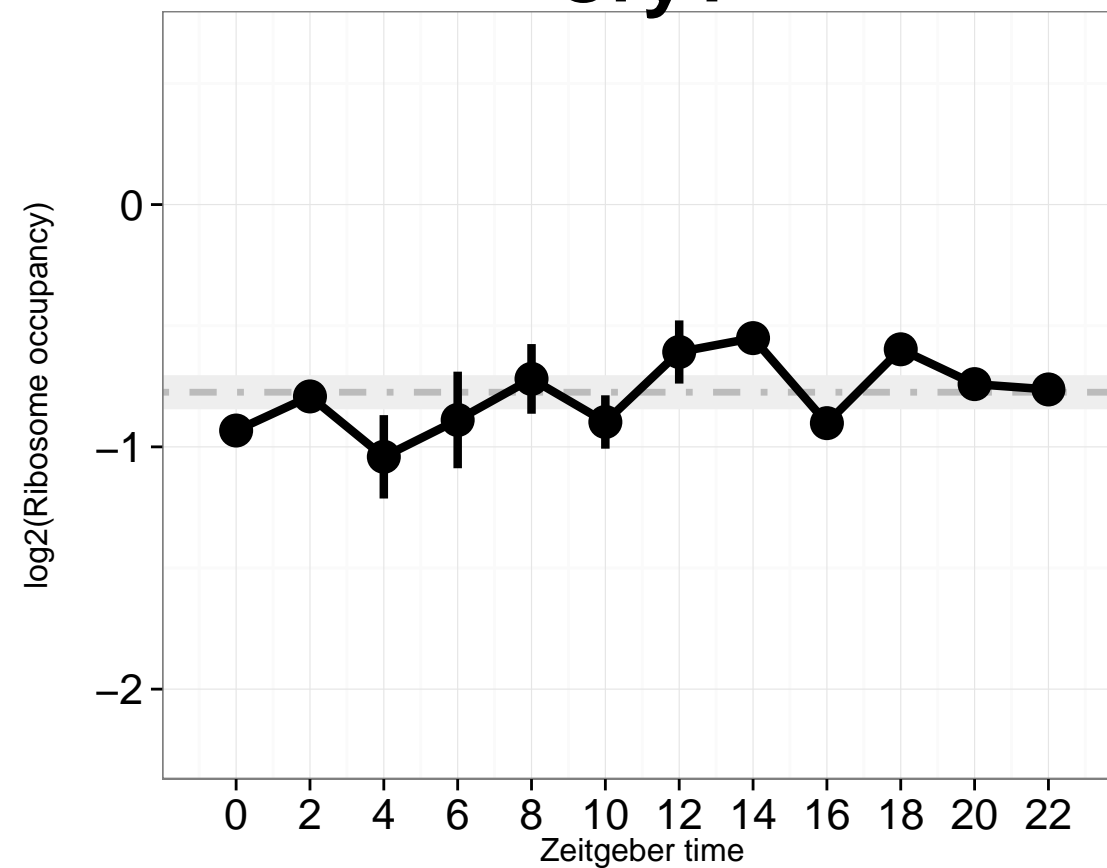

Supplement: Supplementary file 7 — Expression plots for kidney and liver for the 178 common rhythmic genes of Fig. 3c. (ZIP 3338.28 kb) [file 13059_2017_1222_MOESM7_ESM.zip › set_D_shared(178)/Cry1_liver_set_D.pdf]

## Cry2

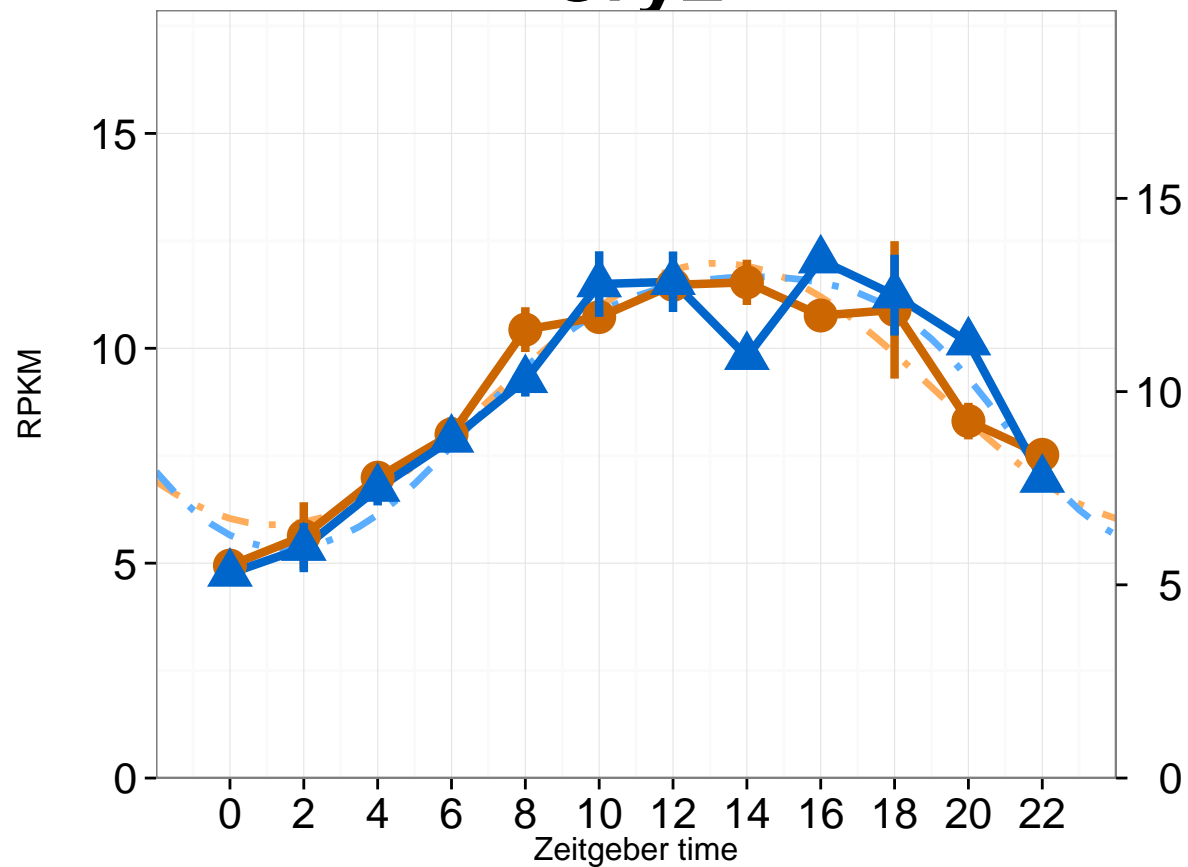

## Cry2

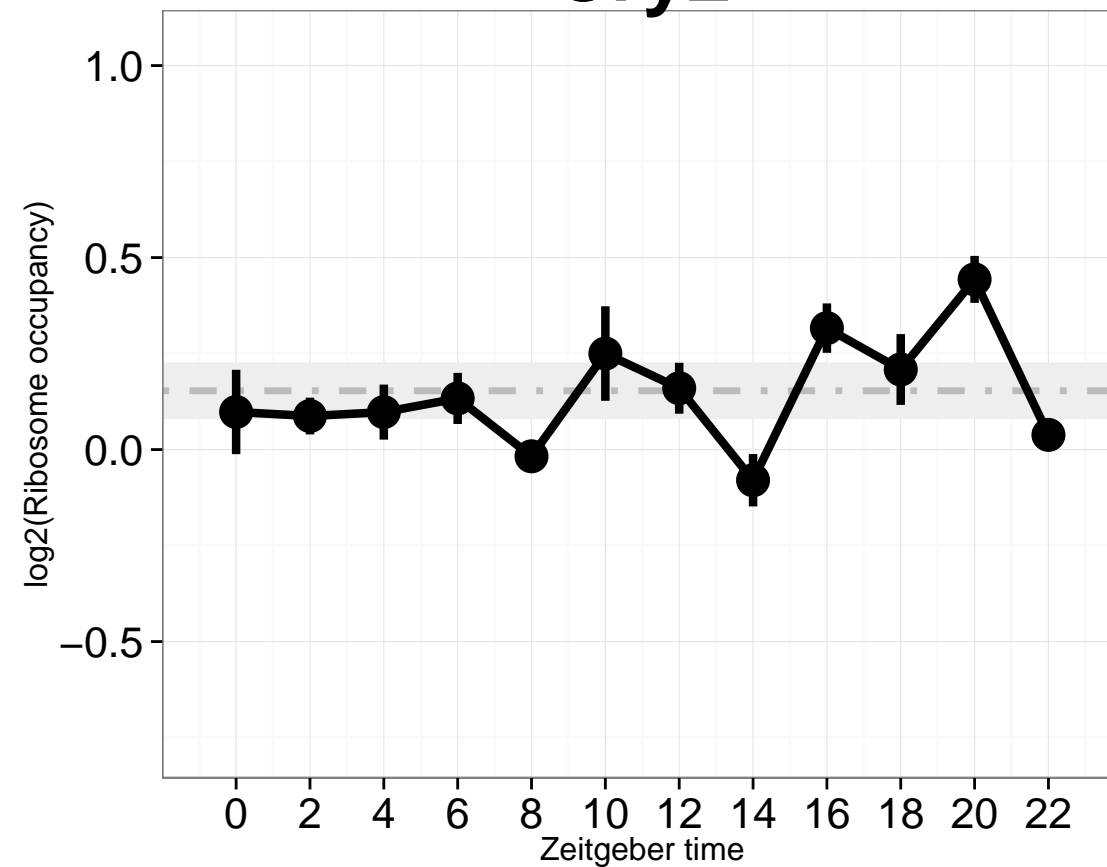

Supplement: Supplementary file 7 — Expression plots for kidney and liver for the 178 common rhythmic genes of Fig. 3c. (ZIP 3338.28 kb) [file 13059_2017_1222_MOESM7_ESM.zip › set_D_shared(178)/Cry2_kidney_set_D.pdf]

## Cry2

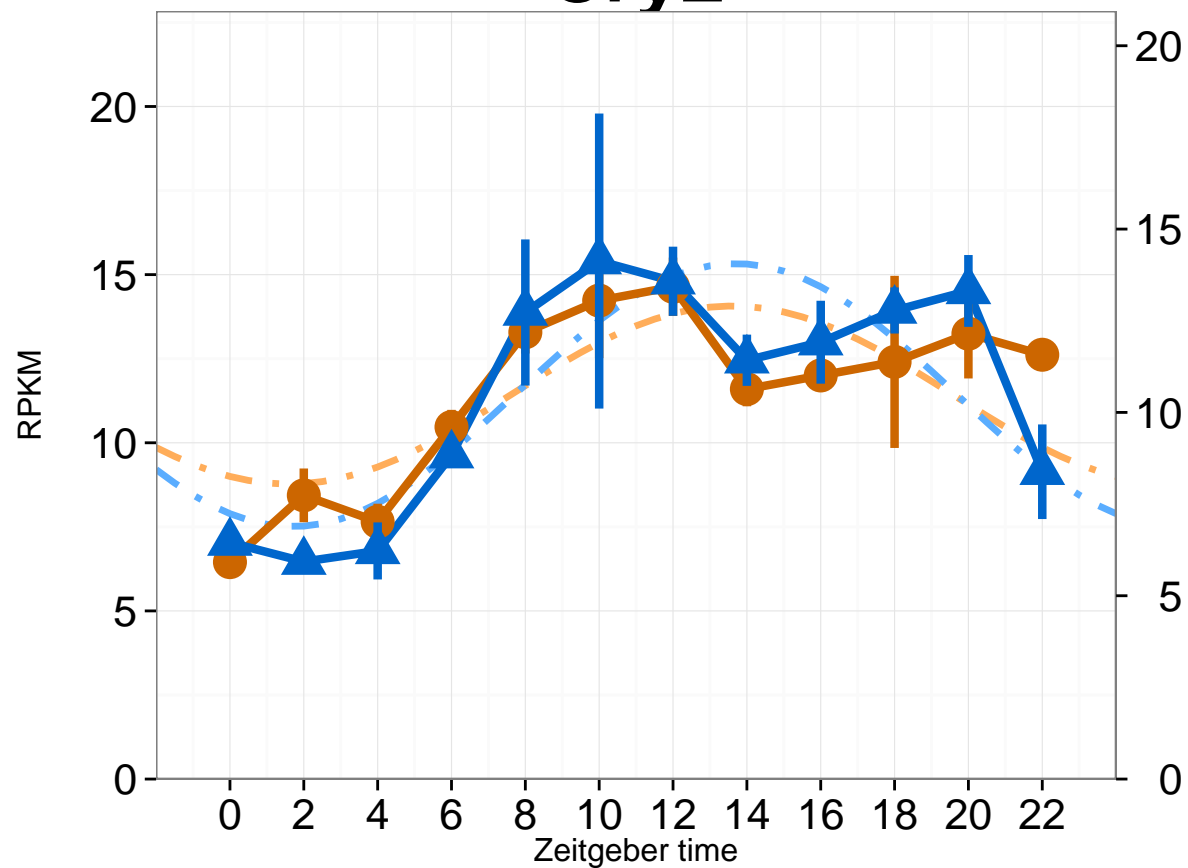

## Cry2

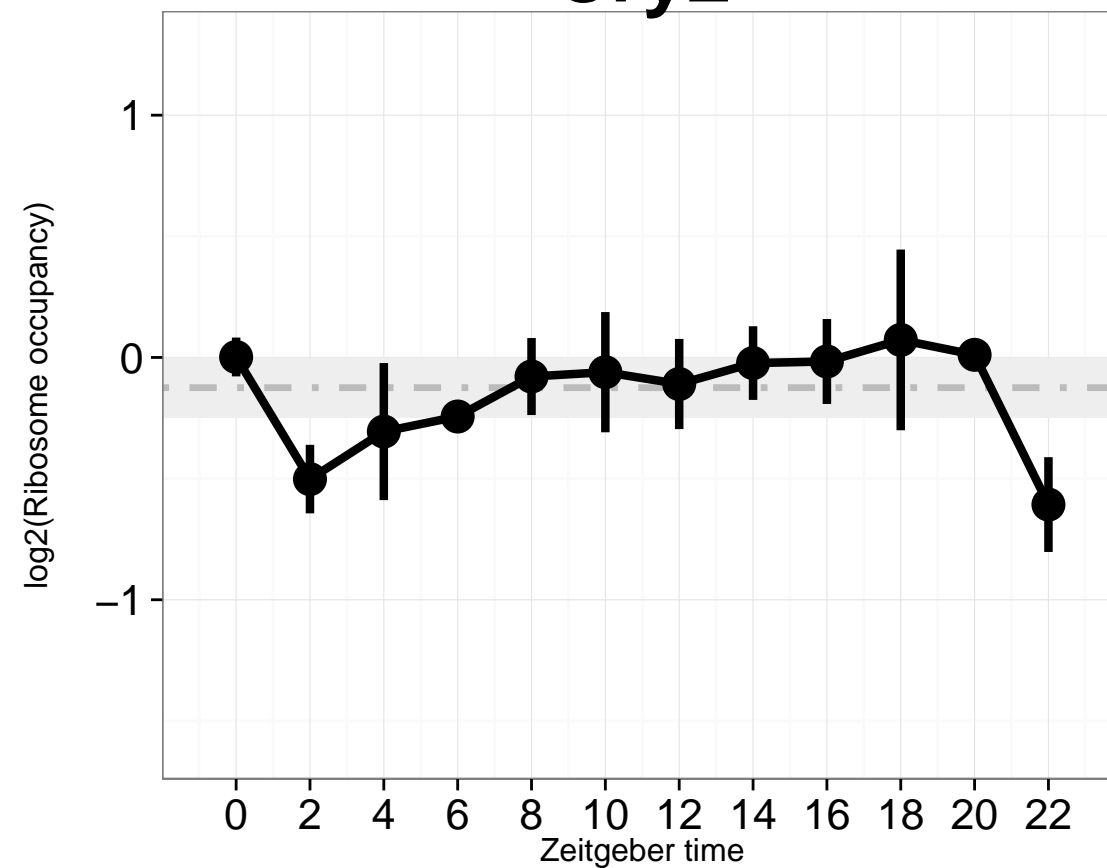

Supplement: Supplementary file 7 — Expression plots for kidney and liver for the 178 common rhythmic genes of Fig. 3c. (ZIP 3338.28 kb) [file 13059_2017_1222_MOESM7_ESM.zip › set_D_shared(178)/Cry2_liver_set_D.pdf]

# Csrnp1

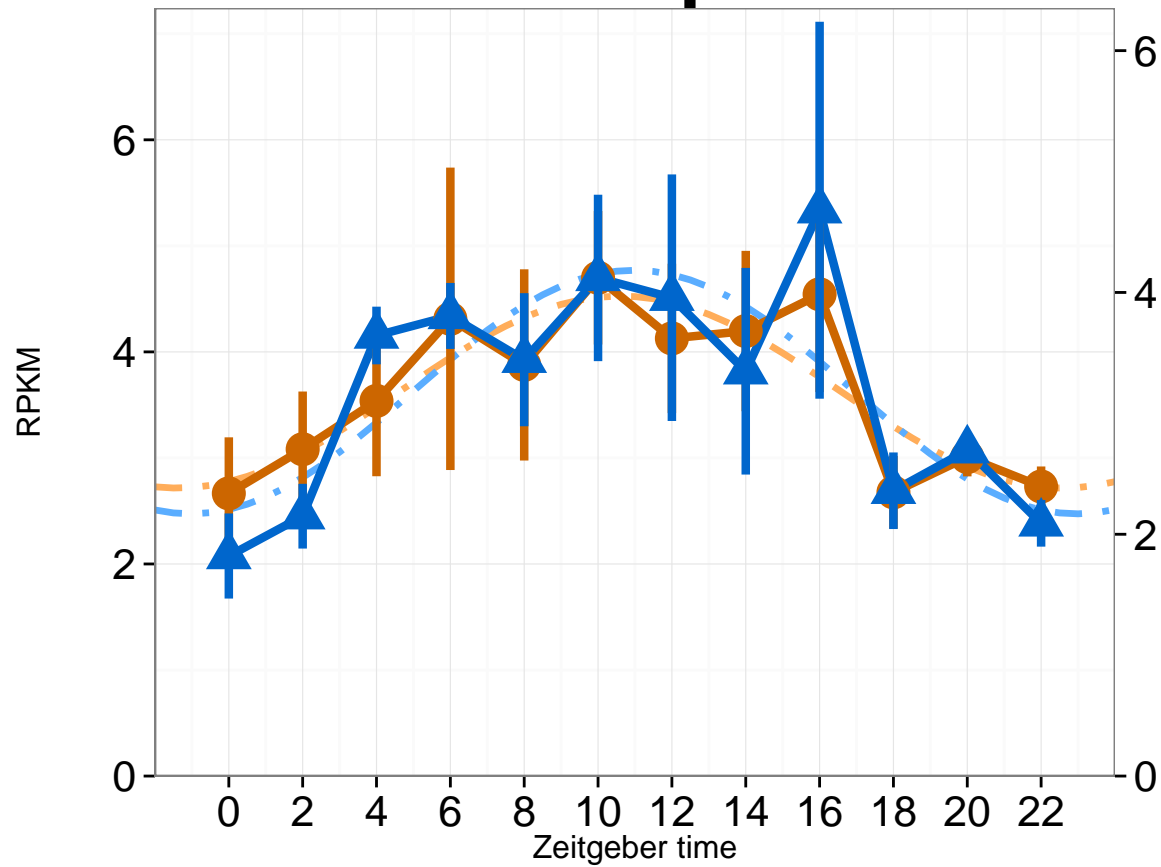

# Csrnp1

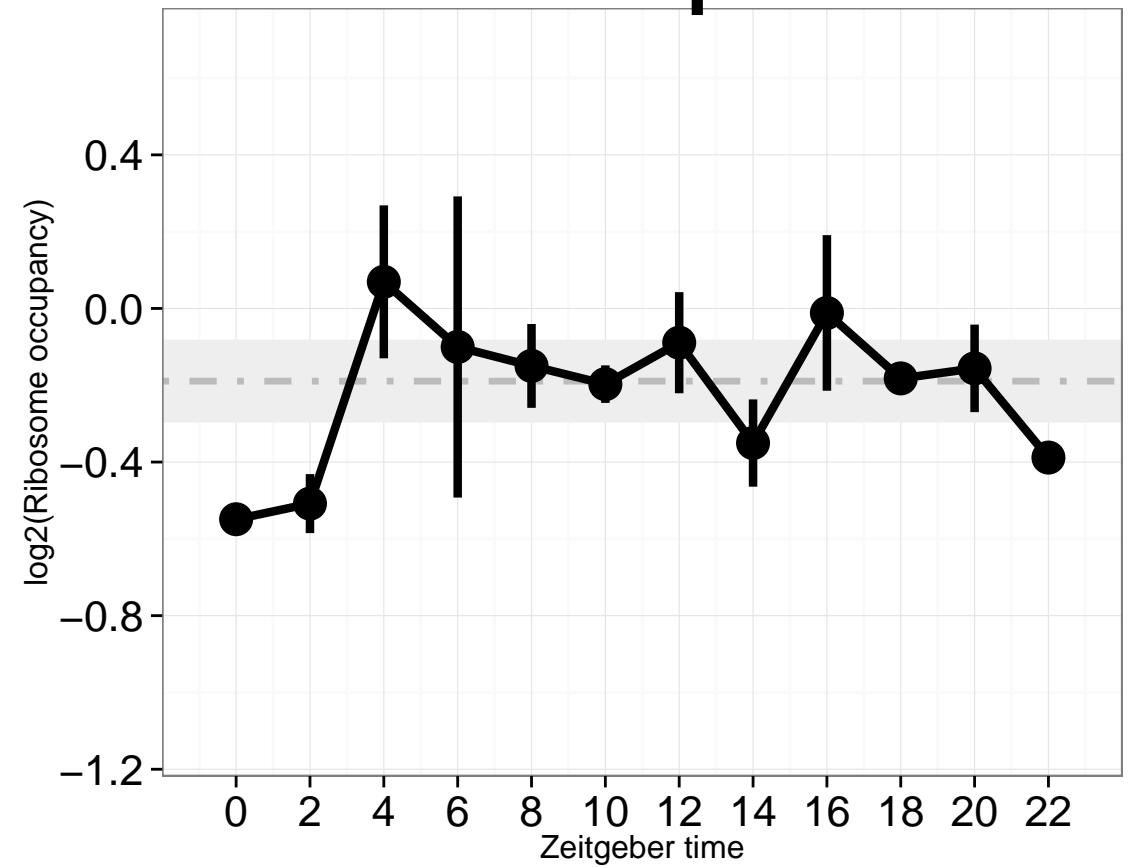

Supplement: Supplementary file 7 — Expression plots for kidney and liver for the 178 common rhythmic genes of Fig. 3c. (ZIP 3338.28 kb) [file 13059_2017_1222_MOESM7_ESM.zip › set_D_shared(178)/Csrnp1_kidney_set_D.pdf]

## Csrnp1

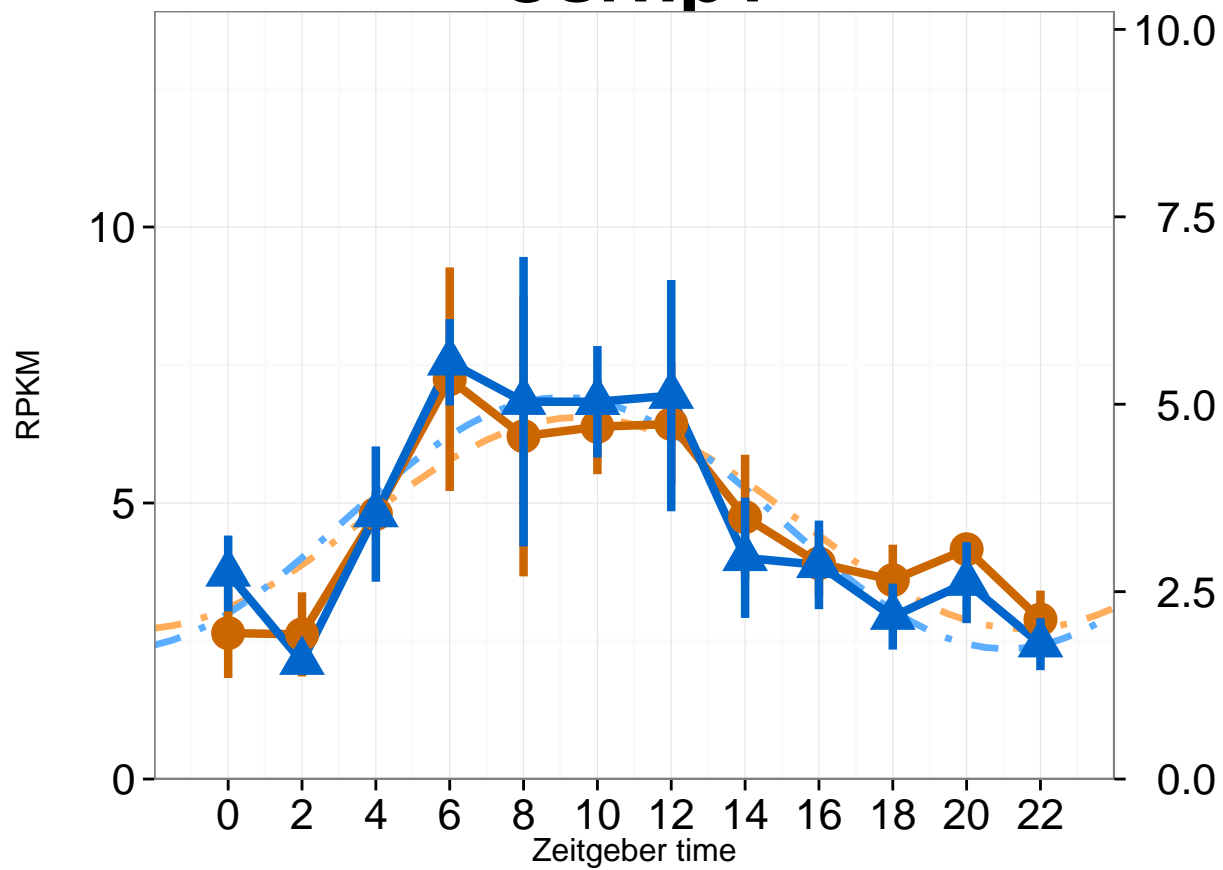

## Csrnp1

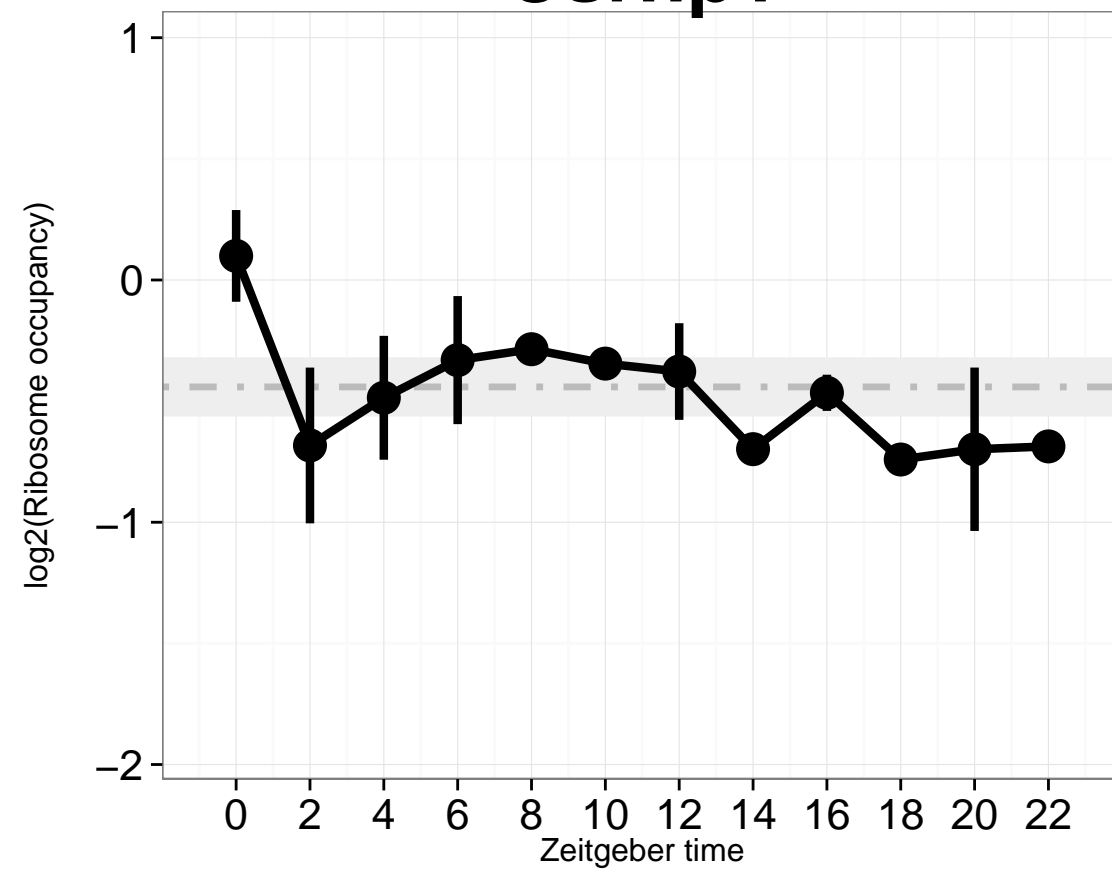

Supplement: Supplementary file 7 — Expression plots for kidney and liver for the 178 common rhythmic genes of Fig. 3c. (ZIP 3338.28 kb) [file 13059_2017_1222_MOESM7_ESM.zip › set_D_shared(178)/Csrnp1_liver_set_D.pdf]

# Cyp2a5

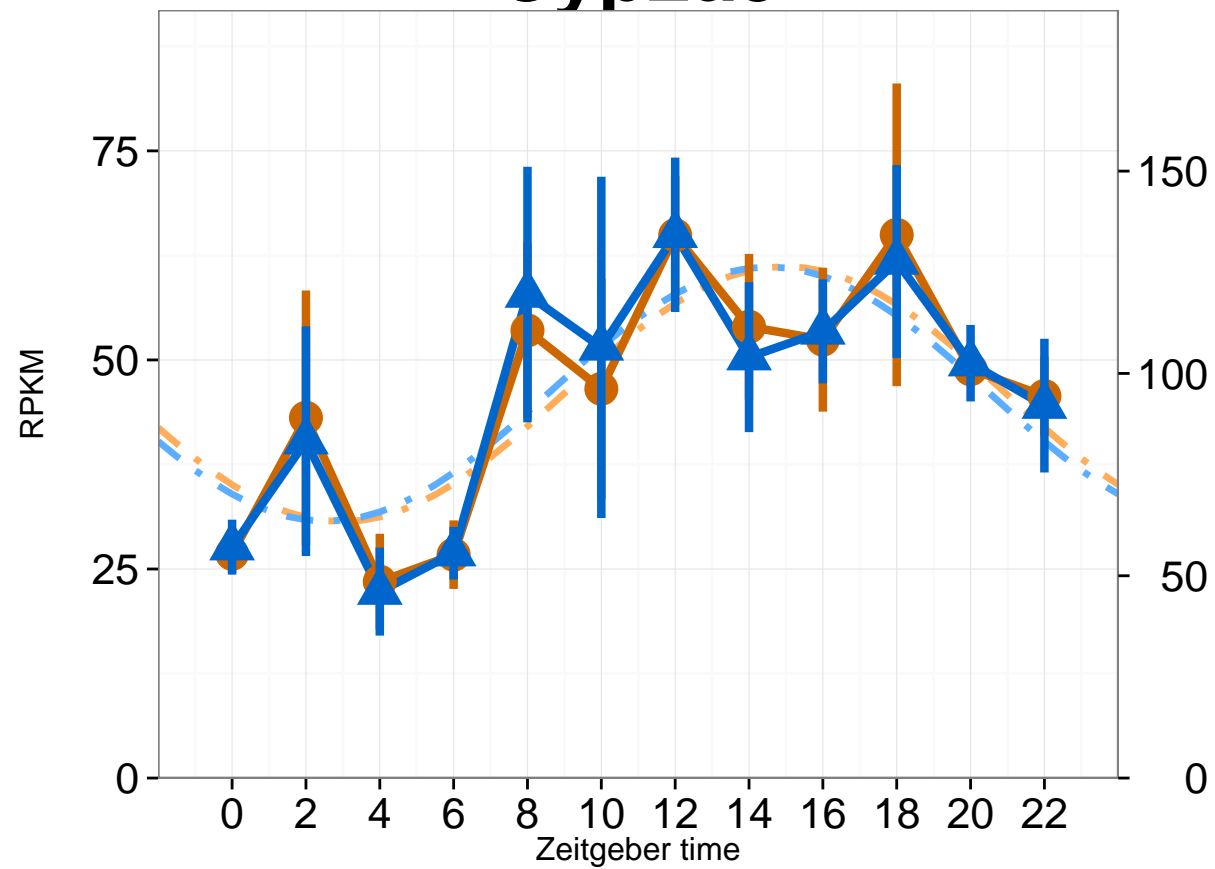

# Cyp2a5

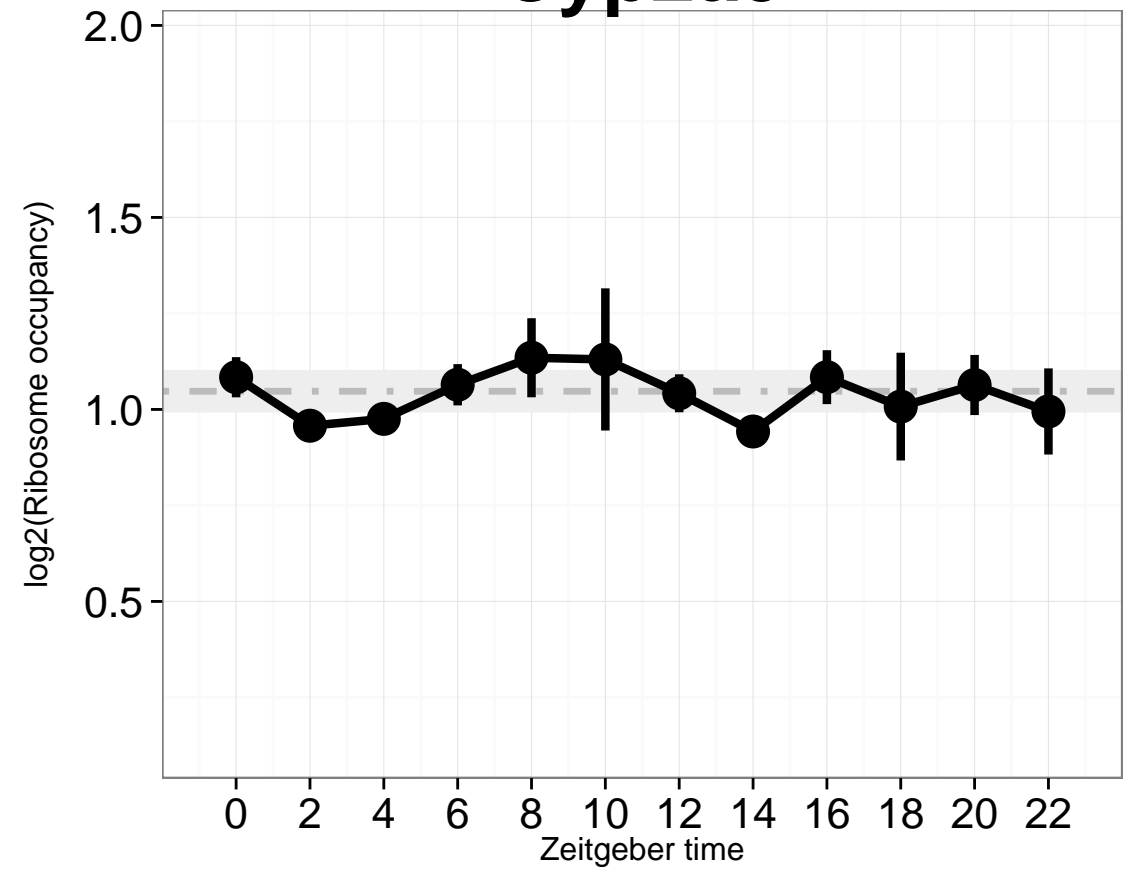

Supplement: Supplementary file 7 — Expression plots for kidney and liver for the 178 common rhythmic genes of Fig. 3c. (ZIP 3338.28 kb) [file 13059_2017_1222_MOESM7_ESM.zip › set_D_shared(178)/Cyp2a5_kidney_set_D.pdf]

# Cyp2a5

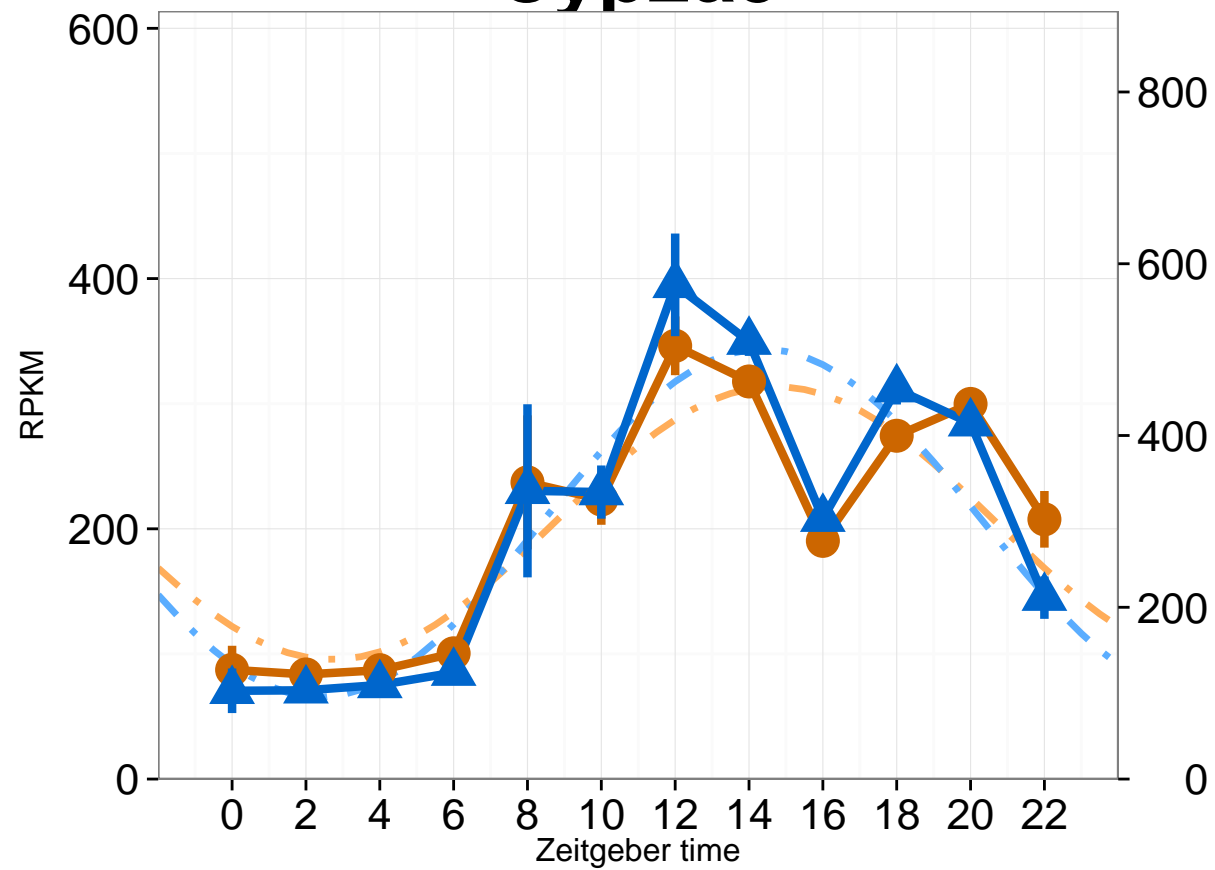

# Cyp2a5

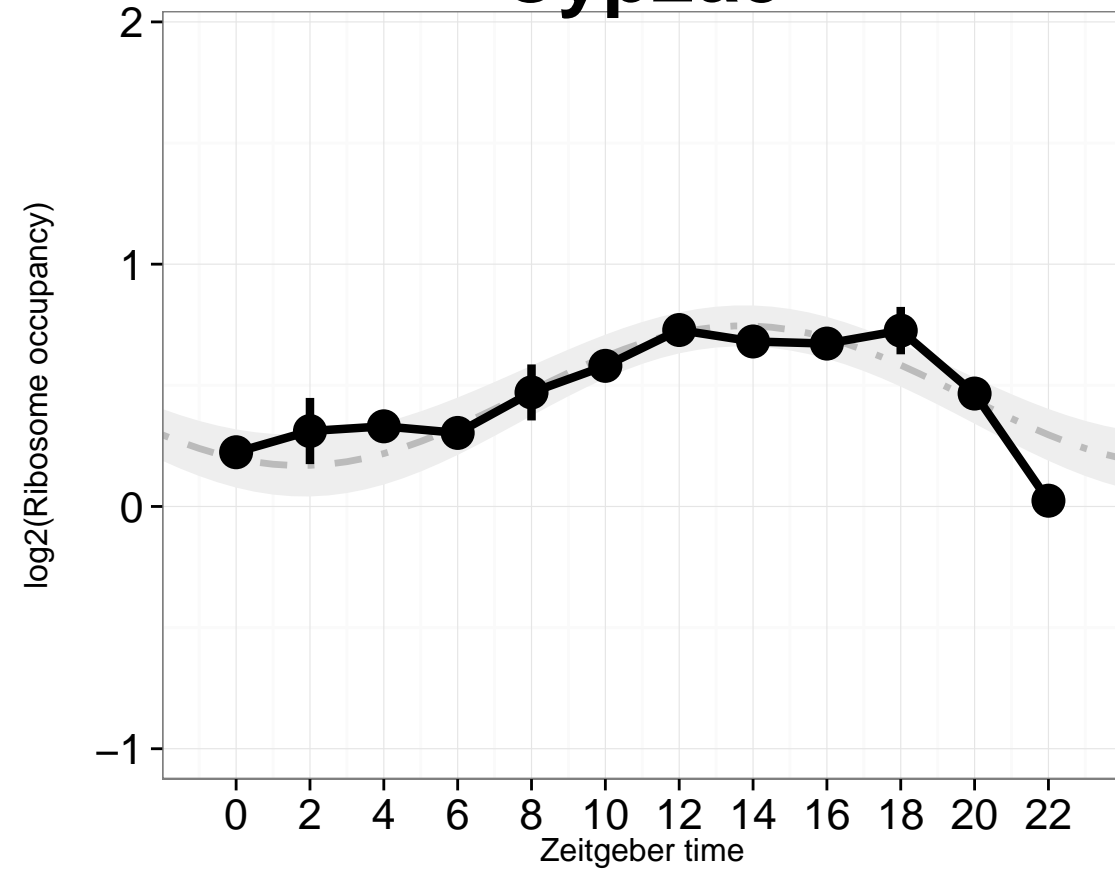

Supplement: Supplementary file 7 — Expression plots for kidney and liver for the 178 common rhythmic genes of Fig. 3c. (ZIP 3338.28 kb) [file 13059_2017_1222_MOESM7_ESM.zip › set_D_shared(178)/Cyp2a5_liver_set_D.pdf]

## Dapk1

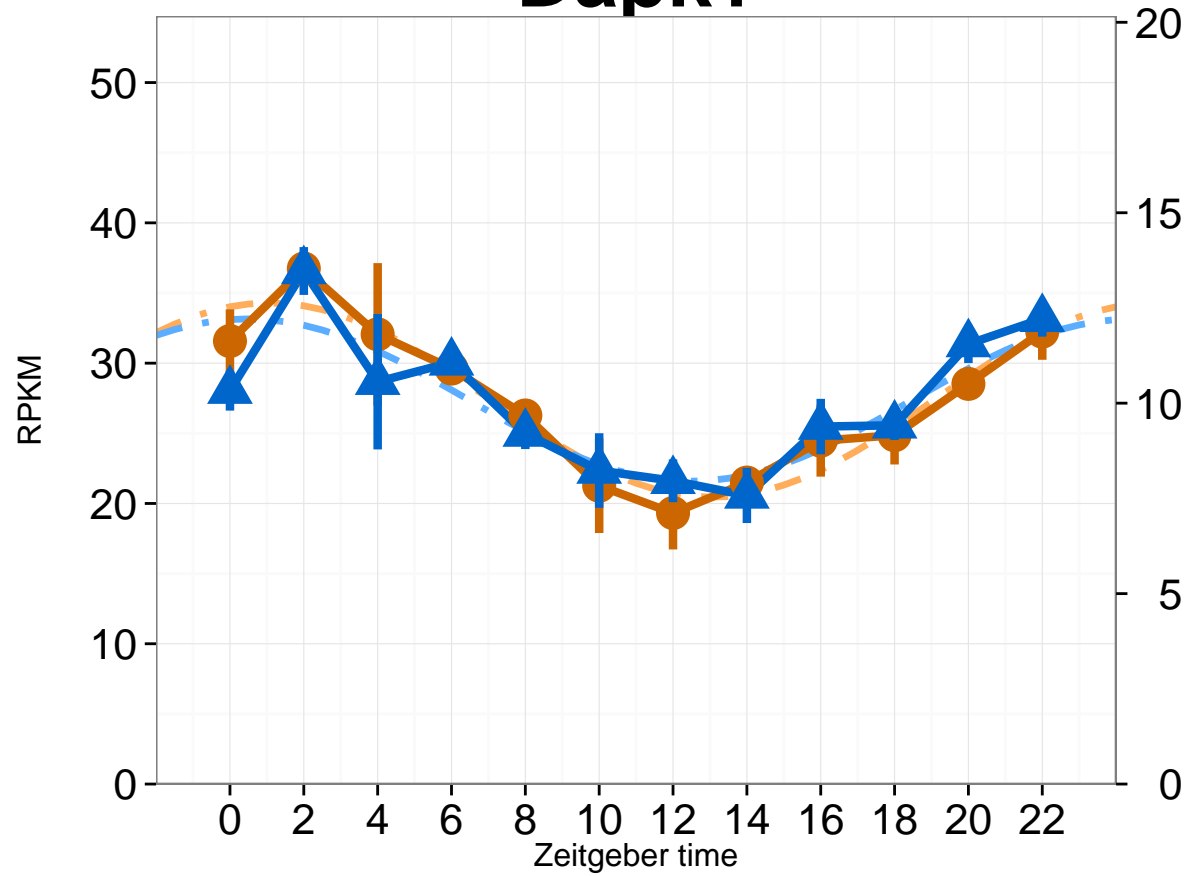

## Dapk1

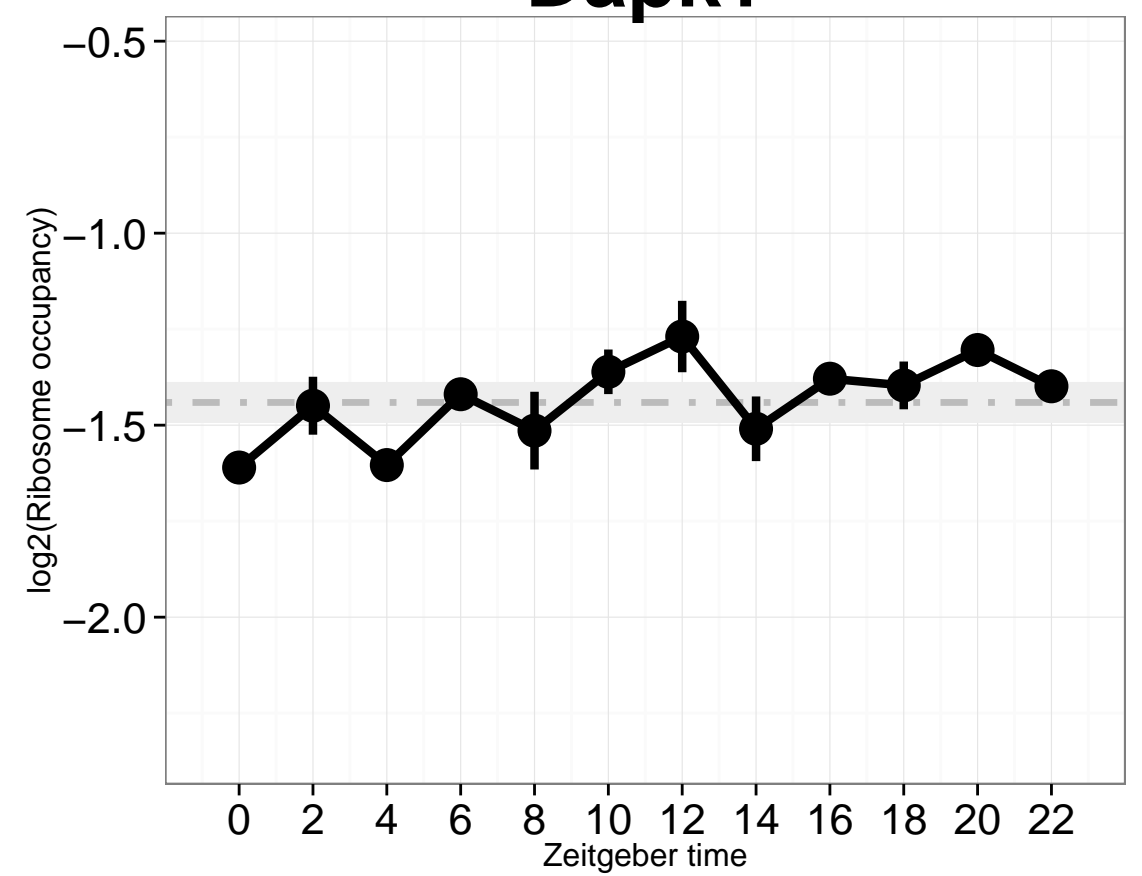

Supplement: Supplementary file 7 — Expression plots for kidney and liver for the 178 common rhythmic genes of Fig. 3c. (ZIP 3338.28 kb) [file 13059_2017_1222_MOESM7_ESM.zip › set_D_shared(178)/Dapk1_kidney_set_D.pdf]

## Dapk1

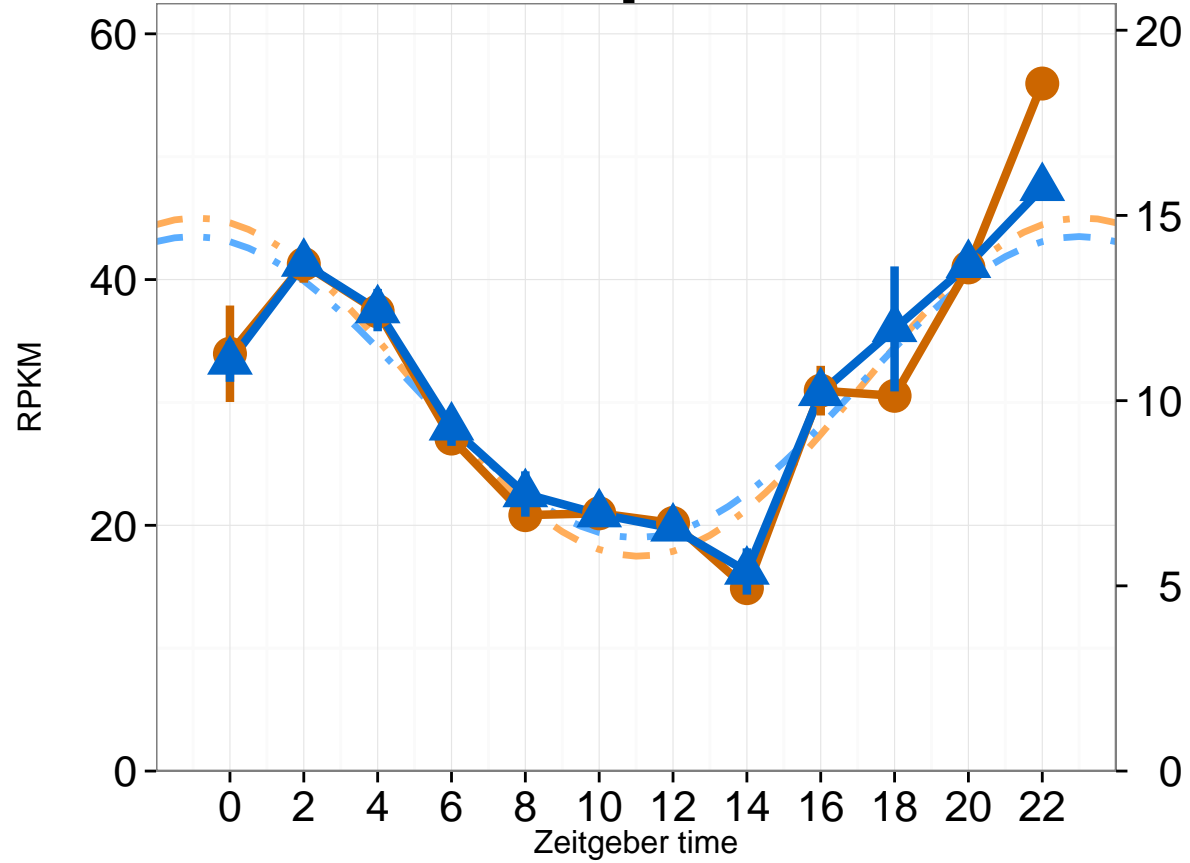

## Dapk1

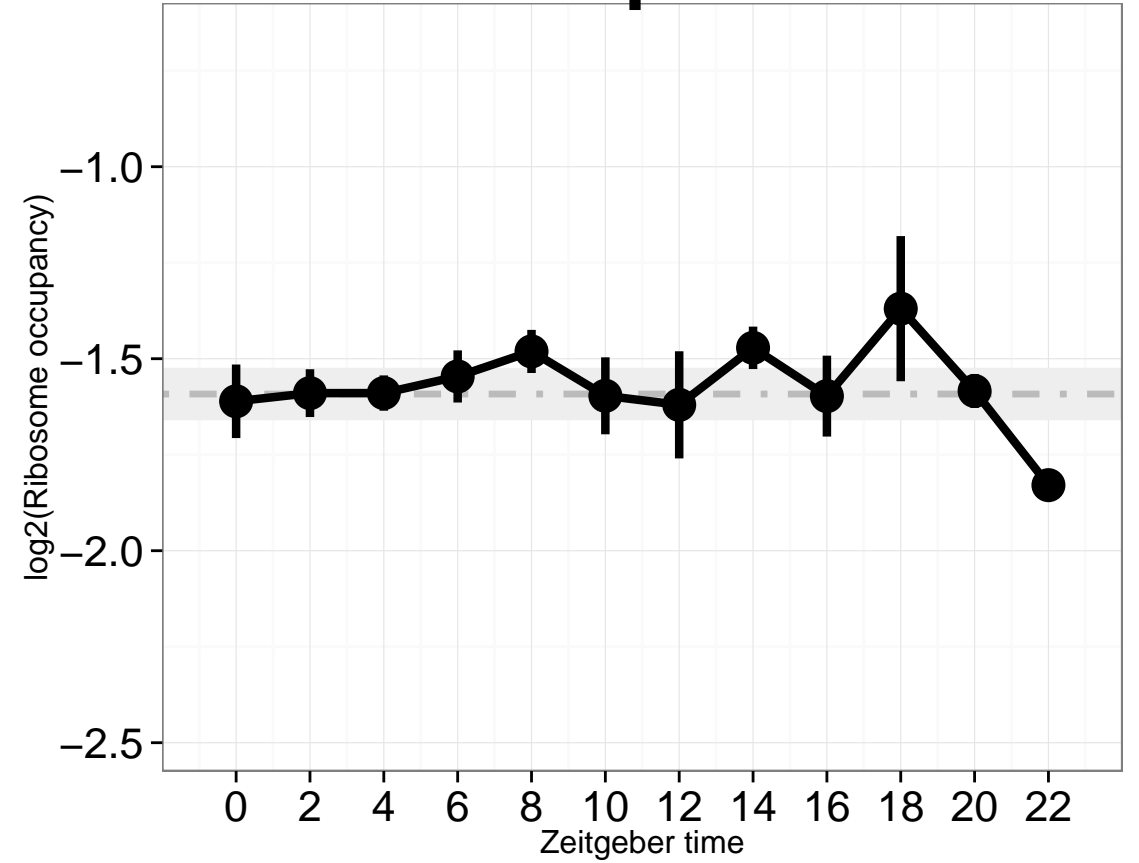

Supplement: Supplementary file 7 — Expression plots for kidney and liver for the 178 common rhythmic genes of Fig. 3c. (ZIP 3338.28 kb) [file 13059_2017_1222_MOESM7_ESM.zip › set_D_shared(178)/Dapk1_liver_set_D.pdf]
